# Chromatin conformation of human oral epithelium can identify orofacial cleft missing functional variants

**DOI:** 10.1038/s41368-022-00194-0

**Published:** 2022-08-25

**Authors:** Yao Xiao, Shengbo Jiao, Miao He, Da Lin, Huanyan Zuo, Jiahao Han, Yonghua Sun, Gang Cao, Zhi Chen, Huan Liu

**Affiliations:** 1 The State Key Laboratory Breeding Base of Basic Science of Stomatology & Key Laboratory for Oral Biomedicine of Ministry of Education, School and Hospital of Stomatology, Wuhan University, 237 Luoyu Road, Wuhan, China; 2 Department of Stomatology, Nanfang Hospital, Southern Medical University, Guangzhou, China; 3 State Key Laboratory of Freshwater Ecology and Biotechnology, Hubei Hongshan Laboratory, Institute of Hydrobiology, Innovation Academy for Seed Design, Chinese Academy of Sciences, Wuhan, China; 4 College of Advanced Agricultural Sciences, University of Chinese Academy of Sciences, Beijing, China; 5 State Key Laboratory of Agricultural Microbiology, Huazhong Agricultural University, Wuhan, China; 6 Taikang Center for Life and Medical Sciences, Wuhan University, Wuhan, China

**Keywords:** Functional genomics, Mechanisms of disease

## Abstract

Genome-wide association studies (GWASs) are the most widely used method to identify genetic risk loci associated with orofacial clefts (OFC). However, despite the increasing size of cohort, GWASs are still insufficient to detect all the heritability, suggesting there are more associations under the current stringent statistical threshold. In this study, we obtained an integrated epigenomic dataset based on the chromatin conformation of a human oral epithelial cell line (HIOEC) using RNA-seq, ATAC-seq, H3K27ac ChIP-seq, and DLO Hi-C. Presumably, this epigenomic dataset could reveal the missing functional variants located in the oral epithelial cell active enhancers/promoters along with their risk target genes, despite relatively less-stringent statistical association with OFC. Taken a non-syndromic cleft palate only (NSCPO) GWAS data of the Chinese Han population as an example, 3664 SNPs that cannot reach the strict significance threshold were subjected to this functional identification pipeline. In total, 254 potential risk SNPs residing in active cis-regulatory elements interacting with 1 718 promoters of oral epithelium-expressed genes were screened. Gapped k-mer machine learning based on enhancers interacting with epithelium-expressed genes along with in vivo and in vitro reporter assays were employed as functional validation. Among all the potential SNPs, we chose and confirmed that the risk alleles of rs560789 and rs174570 reduced the epithelial-specific enhancer activity by preventing the binding of transcription factors related to epithelial development. In summary, we established chromatin conformation datasets of human oral epithelial cells and provided a framework for testing and understanding how regulatory variants impart risk for clefts.

## Introduction

Non-syndromic orofacial clefts (NSOFC), notably cleft lip (CL) and cleft palate (CP), are the most common craniofacial birth defects in humans which affect ~1 in 700 individuals, and represent a substantial personal and societal burden.^1,2^ Accurate information on the risk of associated genetic anomalies and chromosomal defects is necessary to aid prenatal counseling.^3^

An agnostic approach to investigate the susceptible genes and genetic risk sites that are involved in the generation of NSOFC is the genome-wide association study (GWAS), from which over 40 different genes/loci have been identified to date.^1,4–9^ However, GWAS can explain only a small fraction of the heritability of complex traits,^10^ probably because single-nucleotide polymorphisms (SNPs) with modest effects are missed because they do not reach the stringent significance threshold,^11^ by which *P* < 5 × 10^−8^ is usually considered significant.^12^ In addition, most associated SNPs identified from the GWAS map to noncoding regions of the genome, and less than one-third of causal genes are the nearest gene to the GWAS hits.^13^ Therefore, biological annotation of disease-related SNPs is inherently challenging. For functional validation, integrative analysis of GWAS results with functional epigenetic features is useful for prioritizing candidate variants and for determining molecular mechanisms.^14^ However, an integrated tissue-specific epigenomic dataset for the palate has not yet been fully reported.

CP results from abnormal craniofacial developmental processes,^15^ involving complex and accurate interactions between the palatal epithelial and mesenchymal cells. Genes regulating the formation and dissolution of the epithelial seam in the fusion regions and the differentiation of periderm have been implicated in CP pathogenesis.^16,17^ Our previous identification of the conserved rules for DNA sequences in the active enhancers of zebrafish periderm facilitated the prioritization of human OFC-associated variants for downstream functional validations.^18^ Additionally, a previous study suggested that epigenomic features generated by oral epithelial keratinocytes can relatively better annotate common variants associated with orofacial clefts than other commonly used epigenome datasets.^19^ However, the target genes for active enhancers remain largely unknown, which could potentially be candidate genes for NSOFC and explain some of the missing inheritability. Thus, data on chromatin interactions in the oral epithelium, which facilitate the identification of target genes, are necessary to be complemented.

Herein, we hypothesized that there were more SNPs associated with NSOFC that functionally modulate the oral epithelium despite the inability of these SNPs to pass the stringent threshold significance for GWAS, which is in accordance with a previous functional pipeline for the factors affecting heart rhythm.^20^ For functional annotation of potential epithelium-related genes, we firstly generated an integrated map of gene expression, functional epigenetic features, and 3D interactions in human oral epithelial cells (a human immortalized oral epithelial cell (HIOEC) line.^21^) As an example of functional validation using this integrated dataset, we selected 3664 genetic variants with *P* < 10^−3^ as candidate SNPs from a non-syndromic cleft palate only (NSCPO) GWAS data of the Chinese Han population,^22^ and showed that NSCPO-related SNPs within enhancers of oral epithelial cells could physically interact with the promoters of genes expressed in oral epithelial cells, exerting a higher risk of NSCPO, which affects cell migration and proliferation.

## Results

### 3 664 NSCPO-related SNPs with sub-threshold were identified as candidate variants

**Fig. 1:**
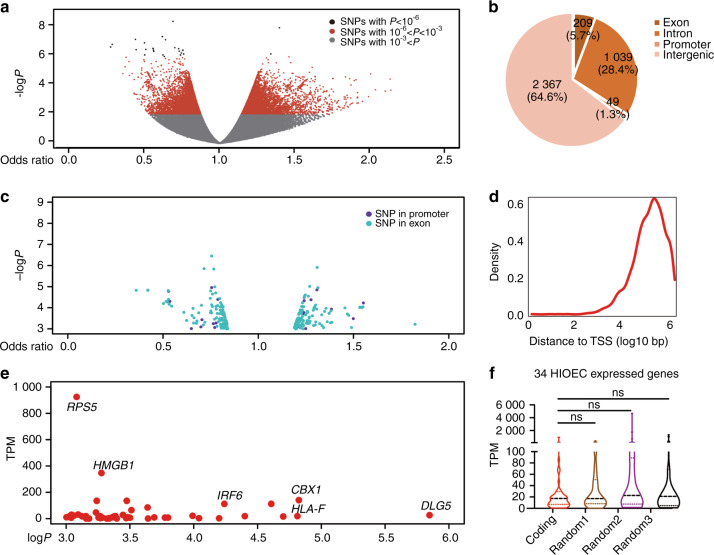
Identifying NSCPO-related candidate variants and those located within coding regions of the genome. **a** Scatter plot of the −log*P* value by Odds Ratio of all the SNPs identified from NSCPO GWAS, Gray dots represent SNPs with a *P* value >10^−3^, the red dots represent 3639 SNPs with a *P* value >10^−6^ and <10^−3^, the black dots represent 25 SNPs with a *P* value <10^−6^. **b** Pie chart showing the number and distribution of 3664 SNPs with *P* value <10^−3^ at the gene position. **c** Scatter plot of the −log*P* value by Odds Ratio of 209 SNPs located at exon regions (light blue dots) and 49 SNPs located at promoter regions annotated in SNPs with a *P* value <10^−3^. **d** Density plot showing the distance from transcription start site (TSS) (log10 bp) of SNPs with a *P* value <10^−3^. **e** Scatter plot of the value of transcripts per million (TPM) by −log*P* value in nsCPO GWAS of 55 SNPs that located in the exon region of genes which are detected in the HIOEC RNA-seq data. **f** Violin plot for TPMs of HIOEC genes with 34 random (random 1, random 2, and random 3) or the coding regions harboring SNPs, ns, non-significant, by Kolmogorov–Smirnov test

At a suggestive genome-wide significance threshold of 10^−6^ only 25 SNPs were identified in the NSCPO GWAS of the Chinese Han population^22^ (Supplementary Table 1), none of them has been verified to be associated with cleft palate. To avoid missing risk variants that did not reach the stringent significance threshold, we dropped the threshold of significance for GWAS data to a sub-threshold with *P* value <10^−3^, and 3664 SNPs were selected as candidate SNPs for further analysis (Fig. 1a).

### Most candidate SNPs were located within noncoding regions

To identify the genes affected by the candidate SNPs, we used gene annotation on the human reference genome hg19 from Gencode to identify the positions as well as the gene loci of all candidate SNPs. 94.3% SNPs associated with NSCPO were located within noncoding regions (Fig. 1b); Only 49 and 209 were in the promoters and exons, respectively (Fig. 1c, Supplementary Table 2). We integrated HIOEC RNA-seq and found that only 2 and 50 SNPs lay within the promoters and exons of genes expressed in the oral epithelium, respectively. Since more SNPs located within one gene, a total of 34 epithelial genes have been identified (Fig. 1e), in which *IRF6* has been reported as a contributor to cleft palate,^23–25^ while other genes have not been reported to be associated with CP. Also, these 34 genes showed no obvious characteristics, as their expression levels had no significant differences compared with that of 34 epithelial genes we randomly selected (Fig. 1f). Furthermore, we found that the candidate SNPs were mostly located 100 kb to 1 Mb away from the transcription start site (Fig. 1d), indicating that the candidate SNPs might lie at an arbitrary distance from the genes they regulate, skipping over intervening genes, or lying within the introns of other genes.

### SNPs located in the active enhancers/promoters of oral epithelial cells were potential functional variants

**Fig. 2:**
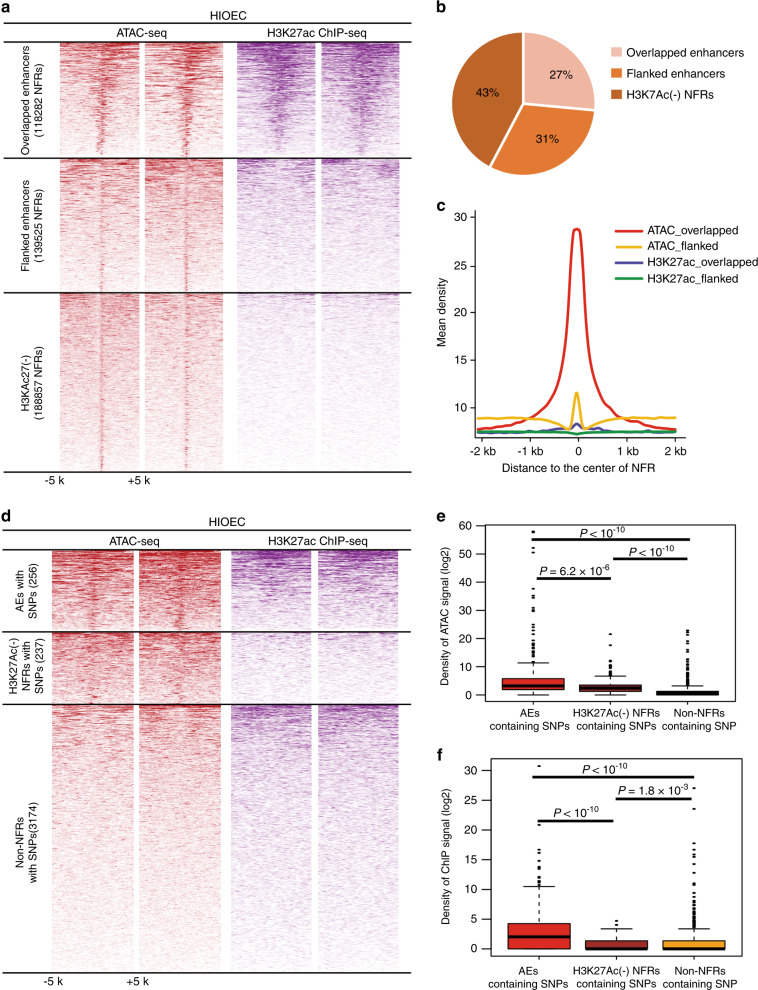
Identification of active enhancers and promoters (AEs) in oral epithelial cells containing candidate SNPs. **a** NFR summit-centered heatmap of ATAC-seq signal as well as H3K27ac ChIP-seq signal in the exact same regions in HIOEC for the peaks in overlapped enhancers, flanked enhancers, and H3K27ac(-) NFRs. Each set of signals has two biological repetitions. **b** Pie chart showing the distribution of NFRs in overlapped enhancers, flanked enhancers, and H3K27ac(-) NFRs. **c** Plots of average density of ATAC-seq and H3K27ac ChIP-seq signals in overlapped enhancers and flanked enhancers. **d** NFR summit-centered heatmap of ATAC-seq signal as well as H3K27ac ChIP-seq signal in the exact same regions in HIOEC for the regions containing SNPs. Each set of signals has two biological repetitions. **e**, **f** The Kolmogorov–Smirnov test showed that the density of ATAC signal (**e**) or H3K27ac ChIP-seq signal (**f**) of the AEs containing SNPs, the H3K27ac(-) NFRs containing SNPs, and the non-NFRs containing SNPs were significantly different

We reasoned that active enhancers and promoters are a subset of nucleosome-free regions (NFRs), which overlapped or flanked by nucleosomes with histone H3 acetylated on lysine 27 (H3K27ac), a marker of active chromatin.^26^ We re-analyzed the ATAC-seq and H3K27ac ChIP-seq data of HIOEC.^19^ For ATAC-seq data, the fragments shorter than 100 bp were identified as NFRs. In total, 118 282 overlapped enhancers/promoters (NFRs overlapped with H3K27ac ChIP-seq peaks) and 139 525 flanked enhancers/promoters (NFRs that were 1–20 kb apart flanked by H3K27ac ChIP-seq peaks) were defined as active enhancers or promoters of HIOEC (for convenience as AEs in the following description) (Fig. 2c, Supplementary Table 3), accounting for 58% of NFRs (Fig. 2b), whereas the remaining 188 857 NFRs were defined as H3K27ac(-) NFRs (Fig. 2a).

Genetic variations located in the AEs might alter the activity of the affected enhancers or promoters and consequently change the transcript abundance. There were 256 SNPs located in AEs, 237 SNPs located in H3K27ac(-) NFRs, and the remaining 3174 candidate SNPs were in non-NFRs (Fig. 2d). Both ATAC and H3K27ac ChIP signals of AEs containing SNPs were significantly higher than those of non-NFRs containing SNPs (*P* < 1 × 10^−10^, Kolmogorov–Smirnov test), and also differed significantly across AEs containing SNPs and H3K27ac(-) NFRs containing SNPs (Fig. 2e, f), confirming that the epigenetic features of the AEs containing SNPs were significantly different from those of the H3K27ac(-) NFR regions or non-NFR regions containing SNPs.

Using HOMER, we identified five enriched motifs, whose corresponding TFs, *SOX4,*^27^ *TP63,*^28,29^ *LEF1,*^30^ and *the HOXD* cluster^31^ have all been shown to be associated with CP. However, among the top five enriched motifs identified in H3K27ac(-) NFR containing SNPs, including *ZNF675, CTCF, FOXH1, OCT*, and *AMYB*, none have been reported to be related to CP (Supplementary Table 4). These results demonstrated that SNPs located in AEs of human oral epithelium are potential functional variants of NSCPO.

### HIOEC DLO Hi-C was reliable for analyzing chromatin interactions in the human oral epithelium

**Fig. 3:**
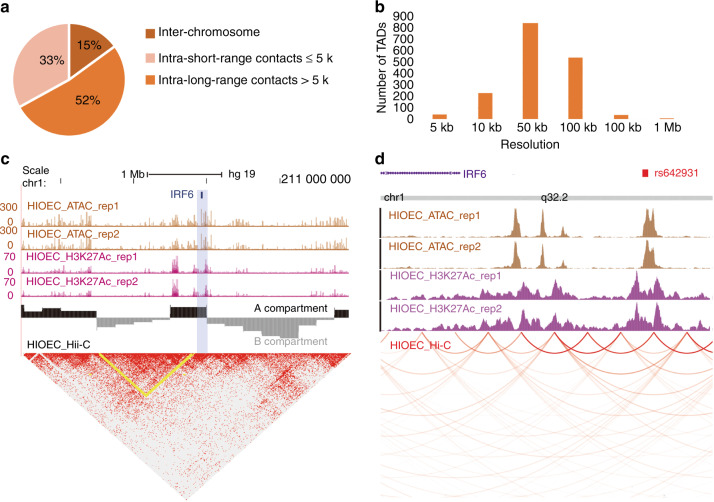
The chromosomal contacts identified by HIOEC DLO Hi-C. **a** Pie chart showing the distribution of interactions detected inter-chromosome, intra-chromosome (short-range contacts ≤ 5 kb), and intra-chromosome (long-range contacts >5 kb) from DLO Hi-C. **b** Bar chart showing the number of TADs at different resolutions (5 kb, 10 kb, 50 kb, 100 kb, 500 kb, 1 MB) from DLO Hi-C. **c** HIOEC DLO Hi-C, ATAC-seq, and H3K37ac ChIP-seq results of genomic region chr1:208.5 Mb–211.5 Mb. Upper: heatmap of DLO Hi-C interaction matrix with 25 kb resolution from Juicebox. TAD domains were indicated by the squares of contact frequency along with the diagonal (yellow); Middle: UCSC genome browser track of A compartments (black) and B compartments (gray); lower: UCSC genome browser track of ATAC-seq and H3K27ac ChIP-seq, each showed two repliacation. **d** WashU epigenome browser tracks of HIOEC ATAC-seq, H3K27ac ChIP-seq, and DLO Hi-C at genomic region near mcs9.7 (rs642961)

To verify the target genes to AEs harboring putative risk SNPs, we used an improved version of digestion-ligation-only Hi-C (DLO Hi-C), a low ligation noise, and low time-consuming chromosome conformation capture techniques,^32^ in the HIOEC cell line. After noise reduction and duplication removal, we obtained 73 766 502 read pairs in total (Supplementary Fig. 1a), 15% of which occurred inter-chromosome (trans-read pairs), whereas the remaining 85% were intra-chromosome interactions (cis-read pairs) (Fig. 3a). The high cis/trans ratios indicated the high library quality of our HIOEC DLO Hi-C data.^33^ We performed A/B compartment analysis and topologically associated domain (TAD) analysis using HIOEC DLO Hi-C data (Fig. 3b, TADs shown in Supplementary Table 5), and found that *IRF6*, a gene widely considered to be pathogenic for cleft palate, was located in the active compartment (compartment A), indicating that *IRF6* is in a transcriptionally active genomic region, and more importantly, it is close to a TAD boundary, where mutations might reconstruct the 3D structure of chromatin (Fig. 3c). Although TAD domains were quite stable across cell types^34^ (Supplementary Fig. 1d–f), looping interactions between promoters and regulatory elements are cell-type specific and occur mostly within TAD.^35^ As we found that despite the number of contacts in our DLO Hi-C was about 1/5 that of in human skin keratinocytes Hi-C (ENCODE project, ENCFF569RJM), the number of interactions between rs642961, a variant highly associated with IRF6, and other chromatin regions did not show any significant differences between the two Hi-C datasets (Fig. 3d, supplementary Fig. 1g). We also found that not only the *IRF6* locus (Fig. 3b), but other genetic loci reported to be associated with NSOFC, such as rs41268753 in *GRHL3*^36^ and rs2070875 in KRT8/18^18^ all had chromatin regions located in highly accessible and H3K27ac-modified regions and were associated with other chromatin segments interact extensively (Supplementary Fig. 1b, c). This explained why knockout of the enhancer containing rs2070875 resulted in reduced expression of both KRT8 and KRT18.^18^

### HIOEC DLO Hi-C data were able to identify NSCPO-related SNPs and AEs of oral epithelial cells

**Fig. 4:**
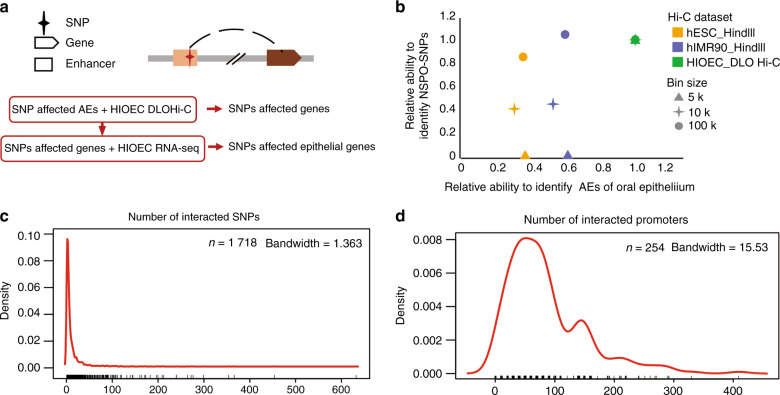
The chromosomal contacts identified by HIOEC DLO Hi-C were able to effectively identify NSCPO-related SNPs and AEs of oral epithelial cells. **a** Schematic diagram of identifying SNPs affected epithelial genes. **b** The dot plot showing the relative ability to identify NSCPO-SNPs and AEs of oral epithelial cells of the inter-chromosomal significant interactions of hESC_HindIII and hIMR90_HindIII Hi-C data compared to the HIOEC DLO Hi-C data under different bin size. The above three Hi-C data were filtered by Fit-Hi-C with a resolution of 10 Kb to identify the inter-chromosomal significant interactions with *P* < 0.05. **c** Density plot of the number of interacted SNPs per gene identified by HIOEC genome interactions from DLO Hi-C data. **d** Density plot of the number of interacted genes per SNP identified by HIOEC genome interactions from DLO Hi-C data

To improve the accuracy of identifying the physical interactions between promoter regions and risk AEs, Fit-Hi-C^37^ with a *P* value <0.05 was used to assign the significant intra-chromosomal contacts of HIOEC DLO Hi-C, and then integrated with HIOEC RNA-seq data to further confirm that the SNPs affected epithelial genes (Fig. 4a). We then obtained high-confidence contacts identified by Fit-Hi-C from Hi-C data of human embryonic stem cells (hESC_HindIII) and human fibroblasts (hIMR90_HindIII) published previously,^37^ expanded the centers of high-confidence contacts with different base pairs (bin size = 5 kb, 10 kb, 100 kb), detected the ability of NSCPO- candidate SNPs and AEs of oral epithelial cells per interaction, and compared the results with those of HIOEC DLO Hi-C. HIOEC DLO Hi-C had the strongest ability to identify candidate SNPs when bin size was small. However, regardless of the bin size, the ability of HIOEC DLO Hi-C to capture AEs per interaction was stronger than that of hESC_HindIII and hIMR90_HindIII (Fig. 4b). This suggests that HIOEC DLO Hi-C data with a bin size of 10 kb were able to identify NSCPO-related SNPs and AEs of oral epithelial cells compared with other Hi-C data.

We found 254 SNPs located in AEs that interacted with the promoters of 1718 genes expressed in HIOEC, which resulted in a total of 22815 functional interactions according to HIOEC DLO Hi-C data with a bin size of 10 kb (Fig. 4c, d).

### SNPs located within AEs affected epithelial-specific enhancer activity

We randomly selected and cloned eight AEs, mutated into the risk allele (Supplementary Fig. 2a), and then used the in vivo reporter assay of zebrafish to verify whether these eight AEs had epithelial activity (GFP-positive cells located in 11 hpf epithelial cells), and to verify whether single-base mutations change the activity of enhancers in the epithelium (Supplementary Fig. 2b). Each construct, in each biological replicate, was injected into more than 100 embryos and GFP expression was monitored 11 h after fertilization of the F0 generation. The results showed that four of the eight AEs had epithelial activity, and the risk allele constructs altered enhancer activity. Among them, mutations in the risk alleles at rs560789 and rs174570 decreased enhancer activity (Supplementary Fig. 2c).

### Risk allele of rs560789 and rs174570 reduced the activity of epithelial-specific enhancer and prevented the binding of transcription factors related to epithelial development

**Fig. 5:**
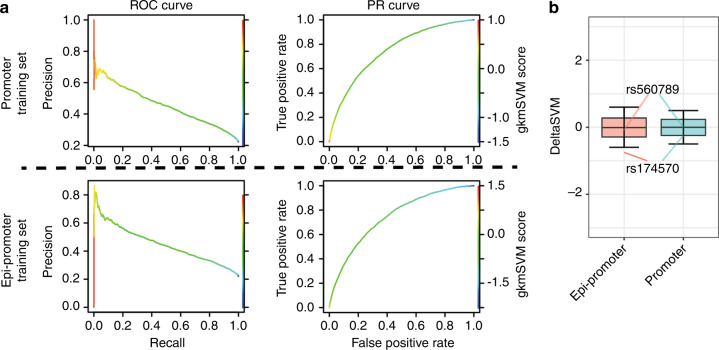
Epi-promoter training set confirmed the risk of rs174570 and rs56789 in NSCPO. **a** Receiver Operating Characteristic (ROC) and Precision-Recall (PR) curves using the gkmSVM trained on AEs interacting with all promoters in hg19 (promoter training set) and AEs interacting with all promoters for genes expressed (TPM > 0.5) in oral epithelial cells (Epi-promoter training set). **b** Box and whisker plots of deltaSVM scores for 1 000 random SNPs in genome and rs174570, rs56789, scored by classifiers by promoter training set and Epi-promoter training set

**Fig. 6:**
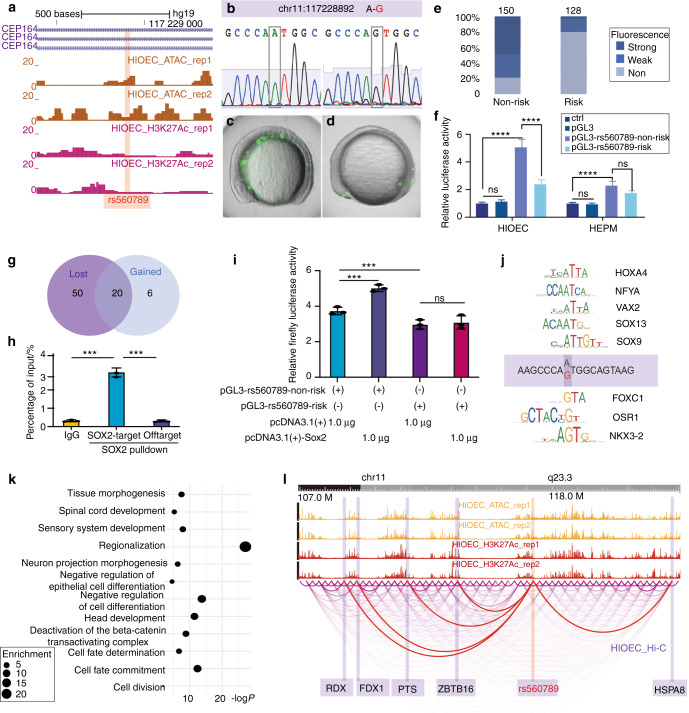
The risk allele of rs560789 reduced activity of epithelial-specific enhancer and prevented the binding of transcription factors related to epithelial development. **a** UCSC genome browser tracks of genomic fragments containing rs560789 that inserted into GFP reporter constructs and luciferase-based reporter vector. **b** Sanger-sequencing results of the products cloned into the GFP reporter plasmid showing the mutation of non-risk allele (A) to risk allele (G) in rs560789 (located at chr11:117228892). **c**, **d** Lateral views of wild-type zebrafish embryos at 11 hpf injected at the 1-cell stage with GFP reporter constructs inserted with enhancer containing non-risk allele (**c**) or risk allele (**d**). **e** 100% stacked column showing the relative percentage of the number of zebrafish embryos that expressed strong, weak, or non-fluorescent after GFP reporter injection. 156 and 120 zebrafish embryos were injected with plasmids containing fragments with non-risk allele and risk allele of rs560789, respectively. **f** Dual-luciferase assay for non-risk and risk allele of rs560789 in HIOEC and HEPM cells. ctrl, group without transfection; pGL3, group transfected with pGL3-promoter vector; ns, non-significance; ****P < 0.000 1. **g** Venn diagram showing 50 transcription factor motifs lost and six transcription factor motifs gained identified by Jaspar2022 after rs560789 mutated to risk allele. **h** qPCR of SOX2 ChIP at rs560789 (SOX2-target) or in *VIM* locus (SOX2-Offtarget, a region without SOX2 motif) using HIOEC cells. **i** Dual-luciferase assay for co-transfection of non-risk and risk alleles of rs560789 with pcDNA3.1(+) vector or SOX2 overexpression plasmid (pcDNA3.1(+)-Sox2) in HIOEC. **j** Examples of transcription factor that were lost or gained with rs560789 mutation of risk allele. **k** Dot plot showed the Gene Ontology (GO) enrichment assay for the 50 lost transcription factors. –log(*P* value) and false discovery rate (FDR) (enrichment value) for each term were generated by Metascape. **l** The new WashU epigenome browser tracks showing assay for ATAC-seq, H3K27ac ChIP-seq, and DLO Hi-C results of HIOEC at chr11:108 Mb–122 Mb. ATAC-seq and H3K27ac ChIP-seq results showed two replicates. Regions marked with light purple show examples of the promoters interacting with rs560789. Red arcs showed the interactions

Using a previously reported gapped k-mer machine learning approach,^18,38^ we generated scoring vector using two training sets, one based on all AEs interacted with all promoters (Promoter training set) and another based on all AEs interacted with promoters for genes expressed (TPM > 0.5) in oral epithelial cells (Epi-promoter training set) (Fig. 5a). Compared with 1000 random SNPs in hg19 genome, we found the scoring vector based on Epi-promoter training set can score rs174570 as an outlier, however rs56789 as neutral (Fig. 5b). Specifically, the single-nucleotide mutation on rs560789 or rs174570 (Fig. 6b, Supplementary Fig. 3b) resulted in concentrated GFP expression in the epidermis covering the head and trunk (Fig. 6c, d, Supplementary Fig. 3c, d), and a decreased proportion of embryos with GFP expression (Fig. 6e, Supplementary Fig. 3e). Dual-luciferase activity (DLA) assays in oral epithelium (HIOEC) or palate mesenchyme cells (human embryonic palatal mesenchyme, HEPM) showed that the non-risk allele-based chromatin regions significantly enhanced luciferase activity in oral epithelial cells, while the enhancement of luciferase activity in mesenchymal cells was weak, and the single-nucleotide mutations significantly reduced luciferase activity in oral epithelial cells (Fig. 6f). Reporter assays revealed that the non-risk elements had enhancer activity with epithelial tissue specificity and that the risk alleles significantly reduced enhancer activity.

To reveal motifs that changed in risk alleles, we used the online software JASPAR to analyze the motifs overlapping rs560789-non-risk alleles and -risk alleles (relative profile score threshold was 80%). The non-risk alleles contained 70 motifs, whereas the risk alleles contained only 26 motifs. Among them, 50 motifs were lost, and six motifs were gained caused by the risk allele (Fig. 6h). The “lost” motifs were mainly members of the *SOX* transcription factor family. According to previous reports, *SOX2* acts as a functional gene of cleft palate, and oral epithelium-specific deletion of *Sox2* resulted in significant orofacial cleft.^39,40^ Anti-SOX2 ChIP-qPCR validated that SOX2 bound to a genomic region overlapping rs560789 (Fig. 6h). In addition, DLA experiment confirmed that overexpression of *Sox2* (using pcDNA3.1(+)-Sox2) increased the activity of enhancer with the non-risk allele but the not non-risk allele of rs560789 (Fig. 6i). These results indicated that the risk allele of rs560789 could impair the function of SOX2, which would result in cleft palate. On the other hand, the “gained” motifs caused by the risk allele, including *OSR1* and *NKX3-2*, were not directly relevant to the pathogenesis of NSCPO (Fig. 6j).

Besides SOX2, a Gene Ontology (GO) analysis showed that the TFs corresponding to the 50 putative lost motifs were closely related to tissue differentiation processes, including the negative regulation of epithelial cell differentiation (GO:0050680, −log*P* = 2.047) (Fig. 6k). TFs included *HOXA2, ISL1, SOX17, HOXA5, SOX10*, and *EMX1*. A single-nucleotide mutation in rs174570 led to similar results. TFs corresponding to the 34 non-risk allele-based motifs (Supplementary Fig. 3g–i) were closely related to epithelial cell differentiation (GO:0030855, −log*P* = 5.10) (Supplementary Fig. 3j), whereas TFs corresponding to the 24 risk allele-based motifs (Supplementary Fig. 3g–i) were enriched in GO terms, including negative regulation of cell differentiation (GO:0045596, −log*P* = 6.08) and embryo development ending in birth or egg hatching (GO:0009792, −log*P* = 7.64) (Supplementary Fig. 3k).

### A potential target gene of rs560789 and rs174570 affected cell migration and proliferation

**Fig. 7:**
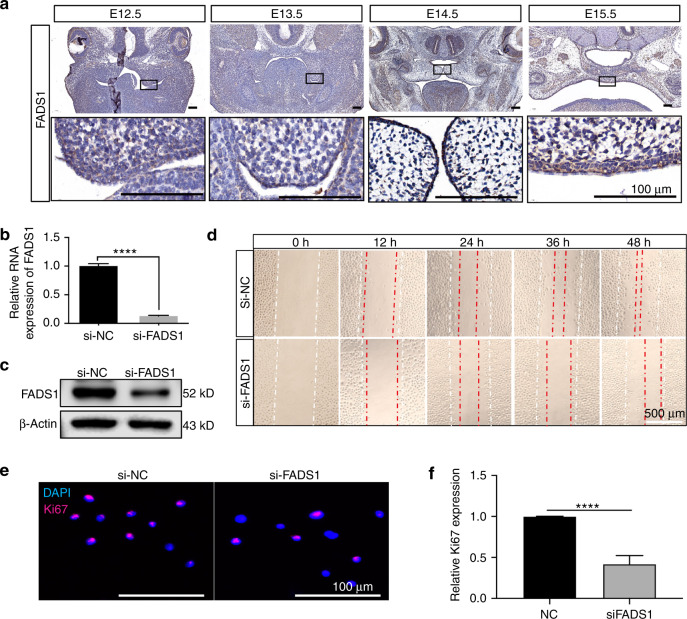
Knockdown of FADS1 reduced epithelial cell migration and proliferation. **a** IHC staining of FADS1 in mice palatal epithelium from E12.5 to E15.5. **b** The relative messenger RNA expression levels of FADS1 were decreased after si-FADS1 treatment. **c** Western blot of FADS1 and β-actin in HIOEC treated with siRNA of negative control (NC) or *FADS1*. **d** The migration of HIOEC with treatment of si-NC or si-FADS1. Images were taken 0, 12, 24, 36, 48 h after scratch. The white dashed line represents the initial boundary of the scratch, and the red dashed line represents the anterior boundary of the migrated cells. **e** Immunofluorescence staining of Ki67 in HIOEC after si-FADS1 or si-NC treatment. **f** Quantification analysis of the relative Ki67 expression. ****P < 0.000 1. Scale bar: 100 μm

Significant contacts of DLO Hi-C identified 16 and 30 target genes for rs560789 and rs174570, respectively (Fig. 6l). Among all these putative targets, rs174570 interacts with the promoter of *FADS1* (Supplementary Fig. 3l), which is expressed in the palatal epithelium during critical stages of palatal development (Fig. 7a). We successfully knocked down the expression of *FADS1* in HIOECs (Fig. 7b, c) and found that cell migration and proliferation were significantly reduced after *FADS1* knockdown (Fig. 7d–f).

## Discussion

GWASs have identified dozens of variants robustly associated with OFC. However, the causal variants, genes, and tissues/cell types at these loci remain largely unknown. Here, we dropped the usual threshold of significance for GWAS data from *P* < 5 × 10^−8^ to *P* < 10^−3^ as we postulated that too high a threshold would miss some critical SNPs. To reduce the false positives from sub-significant *P* value, we established a framework for using functional epigenetics data of oral epithelium to screen orofacial cleft missing functional variants, which provides new insights for prioritizing candidate variants and identifying disease-associated molecular mechanisms (Supplementary Fig. 4). Such pipeline was also raised by a previous functional study for identifying the potential factors affecting heart rhythm based on GWAS and epigenomic data.^20^ Specifically, our current work focus on epigenetic data of HIOEC, which we have previously shown to be more relevant to OFC.^19^

Enhancers are regions of the genome that are major gene-regulatory elements that control cell-type-specific gene expression programs, most often by looping through long distances to come in physical proximity to the promoters of their target genes.^41^ An important contribution of our study was mapping the active enhancer profiles of human oral epithelium by reanalyzing the open chromatin genome (ATAC-seq) and H3K27ac ChIP-seq data^42,43^ from HIOEC cells.^19^ AEs of HIOEC were shown to have the most significant enrichment of NSCPO variants, even stronger than specific enhancers identified in the high-resolution epigenomic atlas of human embryonic craniofacial tissues,^44^ which have been previously shown to have the most significant enrichment of NSOFC variants compared to hundreds of other cell types and tissues. However, as expected, we also noticed that the epigenomic data derived from this epithelial cell failed to annotate those variants solely functionally associated with other cell lineages, which we believe could be well compensated by the other integrated annotation for the whole craniofacial tissues^44^ or human neural crest cells.^45^

ATAC-seq combined with H3K27ac ChIP-seq is a highly efficient method for predicting AEs.^46–48^ However, in vivo reporter assays are essential for testing their activities. Based on our previous identification of the conserved rules for DNA sequences in the active enhancers of zebrafish periderm, the zebrafish transgenic reporter assay provides a convenient and rapid in vivo validation method for prioritizing human OFC-associated variants for downstream functional validation.^18^

Another highlight of this study is that we generated and characterized genome-wide chromatin contact matrices, and identified the active compartment, silent compartment, and TADs^49^ of the human oral epithelium, which helped to predict target genes as well as map oral epithelial gene-regulatory networks. In addition, we proved that HIOEC DLO Hi-C data were able to more effectively identify NSCPO-related SNPs than Hi-C data from other tissues. Hi-C datasets provide a map of the physical interactions between regulatory elements and oral epithelium-expressed genes. Using this map, we identified 254 functional NSCPO-SNPs within AEs of oral epithelial that physically interacted with 1 718 promoters, providing clues for revealing the risk genes of NSCPO (Supplementary Fig. 4). However, the interaction between AEs and promoters offers only the possibility of gene regulation, and the regulation between enhancers requires further validation.

In this work, we chose *FADS1* as a potential target gene of rs174570, which encode a fatty acid desaturase. Beiraghi et al. reported an acyl-CoA-desaturase coding gene, *SCD5* (initially named *ACOD4*), that localized at 4q21 and spanning a pericentric inversion breakpoint that cosegregates in two generations in a family with cleft palate.^50^ SCD5 activity is mechanistically connected to metabolic and signaling pathways that promote proliferation and survival, and inhibit differentiation in craniofacial tissues.^51^ Since FADS1 acts as a front-end fatty acyl-coenzyme A (CoA) desaturase and the knockdown of FADS1 resulted in a negative effect on oral epithelial cell proliferation and migration, FADS1 is a potential risk gene for cleft palate. However, despite of the possible different responses to the loss of FADS1 of mice and humans, it should be noted that targeted mutations of FADS1 have been created in mouse^52^ and reported no issues with orofacial clefting. We noted that a single SNP often interacts with multiple genes, in the case of rs174570, which interacts with the promoter of about 30 epithelial genes. This is also the case in our previous work about *KRT8*/*KRT18* locus.^18^ Thus, we could not determine whether there is a key gene that cause NSCPO, or they all have some minimal activity on phenotypic outcome that when combined as a module can contribute to NSCPO in a more complex pleiotropic fashion. These also suggested a further improvement on target gene prioritization for functional study pipeline is necessary.

The integrated functional epigenomic dataset of human oral epithelial cell could facilitate our understanding of the gene-regulatory network of oral epithelial cells and provides a valuable resource for researchers involved in oral epithelial biology. In the current study, for instance, our datasets provided a genetic basis for investigating palatal epithelium developmental processes and NSCPO pathogenesis. However, we did not focus on the functional epigenomics of mesenchymal cells, which are equally important in orofacial development as epithelial cells, and the use of cell lines also limited the distinction between epithelium and periderm and between different developmental time points.

Despite these limitations, we have demonstrated the framework of the functional genomics dataset of human oral epithelial cells in screening risk alleles of NSCPO. We have also proposed a systematic approach for investigating functional variants associated with oral epithelium-related disease, which should be applicable to the investigation of other complex diseases.

## Materials and methods

### Cell culture and differentiation

HIOEC were grown in Keratinocyte Serum Free Medium containing bovine pituitary extract (Cat No. 10744019, Lot No. 2120576; Thermo Fisher Scientific, USA) with antibiotics (100 U·mL^−1^ penicillin and 100 U·mL^−1^ streptomycin; Hyclone, USA). When cells reached ~90% confluency, the medium was supplemented with 1.2 mmol·L^−1^ CaCl_2_ (Sigma, MO, USA) to initiate the differentiation into a stratified layer.^53^ The HIOEC used to perform RNA-seq and DLO Hi-C procedures were treated with CaCl_2_ for 72 hours. All cells were incubated at 37 °C in 5% CO_2_ and the medium was refreshed every 2 days.

### Digestion-ligation-only Hi-C (DLO Hi-C) library preparation

The DLO Hi-C library was prepared using the DLO Hi-C 2.0 method.^54^ 2 × 10^6^ HIOEC cells were cross-linked with 1% formaldehyde (Sigma, USA), sequestered with 2 mol·L^−1^ glycine (BioFroxx, China), and subsequently lysed in 5 mL lysis buffer (10 mmol·L^−1^ Tris-HCl, 10 mmol·L^−1^ NaCl, 0.3% Igepal CA−630, and protein inhibitor cocktail, MedChemExpress). The nuclei suspensions were digested with 30 μL of MseI (NEB, 10 units per μL) and then ligated with annealed bio-MmeI-Linker (5′-TAGTCGGAGAACCAG/Bio dT/AG-3′) and T4 DNA ligase (NEB, 400 units per μL) for 1 h at room temperature with rotation at 15 r·min^−1^. The ligation products were reverse-cross-linked by incubating with T4 polynucleotide kinase (Takara) for 30 min at 37 °C and T4 DNA ligase (NEB, 400 units per μL) overnight at room temperature. After DNA purification, 10 μL of MmeI (NEB, 2 units per μL) was added to the purified DNA sample and incubated at 37 °C for 1 h to create 80-bp DLO Hi-C DNA fragments. Streptavidin beads (Thermo Fisher Scientific, USA) were used to purify the DLO Hi-C DNA fragments. For library indexing, 2 μL of PE-adaptor1 (500 ng·μL^−1^), 2 μL of PE-adaptor2 (500 ng·μL^−1^), 4 μL of 10× T4 DNA ligase buffer, and 3 μL of T4 DNA ligase (Thermo; 2 units per μL) were added to 29 μL of DLO Hi-C DNA fragments and incubated at room temperature for 20 min, and Phanta Super-Fidelity DNA Polymerase (Vazyme) with different customized TruSeq adapter pairs was added. The DNA fragments were purified using an equal volume of phenol:chloroform:isoamyl alcohol (25:24:1) to produce the final library, which was subjected to a HiSeq X Ten sequencer (Illumina, provided by Annoroad Gene Technology).

### RNA‐seq library generation and data analysis

Total RNA from CaCl_2_-treated HIOEC was isolated using the RNeasy Mini Kit (Qiagen), and residual genomic DNA was removed using DNase I (Promega). RNA-seq libraries were generated and indexed using the NEBNext Ultra RNA Library Prep Kit (New England Biolabs). Next, 150‐bp paired‐end sequencing was performed on a HiSeq X Ten sequencer (Illumina, provided by Annoroad Gene Technology). Sequencing reads were pseudo-aligned to the hg19 cDNA reference genome and quantified using Kallisto (v 0.44.0).^55^ Average TPM values for genes in three replicates were used for downstream analysis.

### Hi-C data analysis

The DNA library was pooled for high-throughput sequencing on a HiSeq X Ten PE150 (Illumina, provided by the Annoroad Genomics Company (China)), and obtained raw data with 158 226 229 reads. The human reference genome hg19 from the ENSEMBL website (ftp://ftp.ensembl.org/ pub/release-73/fasta/homo_sapiens/dna/) was used to analyze all data. Raw sequencing reads of the DLO Hi-C data were processed using the DLO Hi-C Tool (https://github.com/GangCaoLab/DLO-HiC-Tools).^56^ Briefly, raw data were filtered using linkers and sequences were mapped against the hg19 genome, PETs of self-ligation, re-ligation, and were removed. The iterative correction matrices were then generated. TADs were identified using the Arrowhead algorithm of Juicer.^57^ Loop calling was performed using Juicer HICCUPS with 5 kb and 10 kb bin sizes and default parameters. The A/B compartment configuration file was extracted from.hic file using the Juicer Tool according to a previously reported method.^58^ Chromatin interaction heat maps were produced using Juicebox. Fit-Hi-C^59^ (https://github.com/ay-lab/fithic) was used to assign statistical confidence estimates to the mid-range intra-chromosomal contacts of HIOEC DLO Hi-C at a resolution of 10Kb. Interactions with *p* values <0.05 were considered significant. Hi-C data used to compare TAD domains and interactions were obtained from ENCODE project, the file ID is ENCFF894RRQ and ENCFF569RJM.

### ATAC-seq data and H3K27ac ChIP-seq data analysis

Raw ATAC-seq fastq files and raw H3K27ac ChIP-seq data were obtained and analyzed as previously described.^19^ Briefly, raw data were trimmed with Trimmomatic (v 0.38),^60^ mapped to hg19 reference genome build using Bowtie 2,^61^ and sorting with SAMTools.^62^ Peaks were called using MACS2 (v2.1.1).^63^ For ATAC-seq data, the fragments shorter than 100 bp were identified as NFRs. Deeptools (v 2.0)^64^ were utilized to generate bigwig files which were inputs in the visualization tools UCSC genome browser and Juicebox.

### Annotation of ATAC-seq, H3K27ac ChIP-seq, DLO Hi-C, and GWAS datasets

Gene annotation on the human reference genome hg19 from the Gencode (ftp://ftp.ebi.ac.uk/pub/databases/gencode/Gencode_human/release_19/gencode.v19.annotation.gtf.gz) was used to annotate the gene names and the location of related gene loci. INTERSECT in Bedtools (v2.29.2)^65^ was utilized for the alignment of all the different chromatin regions using default parameters.

### Plasmid construction

The eight candidate AE domains centered with NSCPO-related SNPs and the full-length open-reading frame of *SOX2* were cloned with restriction endonuclease sites by PCR amplification using KOD-Plus-Mutagenesis Kit (Toyobo) and inserted each of them into vector plasmids. The single-nucleotide mutation of genetic variation site on the vector was carried out using the KOD-Plus- Mutagenesis Kit (Toyobo) following the manufacturer’s instructions (The primer sequence is shown in Supplementary Table 6). After confirmation by Sanger-Sequencing, candidate AEs were shuttled into pGL3-promoter plasmid for in vitro luciferase assay, and pSK-MSC-GFP-Tol2 plasmids for zebrafish in vivo enhancer assay. Open-reading frame of *SOX2* were inserted into pcDNA3.1(+) vector (purchased from Invitrogen) for in vitro luciferase assay.

### Zebrafish enhancer in vivo reporter assay

For each reporter construct, at least 100 embryos at one-cell stage were injected (20 pg reporter construct with 20 pg *tol2* mRNA) with three replicates performed on different days. The injected healthy embryos were examined and recorded by epifluorescence microscopy at 11 hpf to check for enhancer activity in the epidermis.^66^

### Machine learning

Similar to our previous protocol,^18^ all AEs that interacted with the promoters of epithelium-expressed genes (TPM > 0.05) were resized to 400 bp to maximize the ATAC-seq signal within each NFR. Additionally, AEs with more than 70% repeat sequences (hg19) were removed using the UCSC genome browser (http://genome.ucsc.edu). A total of 4 112 regions were subjected to a gapped k-mer super vector machine (gkmSVM)^38^ to generate a 10-fold larger negative training set of a random genomic 400-bp sequence in the hg19 genome. The positive and negative training sets were used to generate a scoring vector using the gkmSVM (*K* = 6, *L* = 10). This scoring vector was then used to score the risk and non-risk alleles of the SNP of interest.

### Dual-luciferase assay

HIOEC cells and HEPM cells were seeded in 12‐well plates, 20 ng of promoter-Renilla luciferase reporter plasmid (pRL-TK) and 1 μg of pGL3 reporter plasmid were co-transfected with electroporation using the previous protocols^67^ for each well. The cells were harvested 72 hours after transfection. Cell extracts were then isolated, and luciferase assays were performed using the Luciferase Assay System (Promega) according to the manufacturer’s instructions. Duplicate wells were analyzed. Cell lysates were normalized for protein and were analyzed for firefly luciferase activity and Renilla luciferase (pRL‐TK) activity as an internal standard for transfection efficiency.

### ChIP-qPCR

Chromatin immunoprecipitation was performed according to the protocols of chromatin immunoprecipitation (ChIP) Assay Kit (Cat No.16-157; EMD Millipore, NY, USA). Briefly, 1% formaldehyde cross‐linked 1.0 × 10^6^ HIOEC cells were collected and suspended in lysis buffer, and the cross‐linked DNA were sonicated into 200–1 000 bp fragments using Sonicator 3000 (Misonix, Farmingdale, NY, USA). Chromatin immunoprecipitation was performed using 60 μL of Protein A agarose and 4 μL of anti-SOX2 (Cat No. sc−365964, SantaCruz) or IgG (Abclonal). Immunoprecipitated DNA were then purified by treatment with RNase A, proteinase K, and multiple extractions with phenol/chloroform/isoamyl alcohol. Purified DNA was used as a template for qPCR analysis, The primer sequences for enhancer with rs560789 were: forward, 5′-CTGGCTTGCACTGGTTCTCT-3′ and reverse, 5′-GCTATGCGTGGAGTTGTCCT-3′; for Offtarget (chr10:17276056-17276160, located in *VIMENTIN* locus): forward, 5′-ATTGTGTTTGCCACCACAGC-3′ and reverse, 5′-CCTGGGCAGTAGAGCAAGAC-3′.

### Immunohistochemistry

The sagittal sections of mandibles from C57/BL mice at embryonic day 12.5 (E12.5) to E15.5. Antibody against FADS1 (Cat No. ab126706; Abcam, MA, USA) was diluted 100-fold with PBS and incubated at 4 °C overnight.

### Cell immunofluorescence

HIOEC were seeded on coverslips and were rinsed with PBS and fixed with 4% PFA at room temperature for 15 minutes. Next, the cells were permeabilized with 0.25% Triton X‐100 for 5 minutes, washed with PBS twice, and blocked with 2.5% bovine serum albumin in PBS for 1 hour. Antibody against KI67 (Cat No. ab15580; Abcam, MA, USA) was diluted 100-fold with PBS and incubated at 4 °C overnight.

### siRNA transfection

HIOEC were seeded in 12-well plates, grown until reaching 70%–80% confluency and the siRNAs (GenePharma, Shanghai, China) were transfected into cells at a final concentration of 50 nmol·L^−1^ with Lipofectamine 2000 (Invitrogen, Carlsbad CA, USA).

### RNA isolation and quantitative reverse transcriptase PCR (qPCR) analysis

HP Total RNA Kit (Omega bio-tech, Norcross, GA, USA) and RevertAid First Strand cDNA Synthesis Kit (Invitrogen) were used for total RNA extraction and cDNA synthesis. Then qPCR was performed with the CFX Connect real-time PCR system (BIO-RAD, USA) using the ChamQ SYBR qPCR Master Mix (Vazyme, Nanjing, China).

The primer sequences for GAPDH were: forward, 5′-ACTTTGGTATCGTGGAAGGACT-3′ and reverse, 5′-GCCTTGGCAGCGCCAGTAG -3′; for FADS1 were: forward, 5′-GGGTCTTTGGGACGTCCTTT-3′, reverse, 5′-TTGAGGTGCTGAAGACCGAC-3′ GAPDH was used as internal control and the expression of each gene was calculated using the 2^−ΔΔCT^ methods. The gene expression ratio was shown as the mean standard deviation from three independent experiments.

### Western blot analysis

Total proteins of mDPCs were isolated using lysis buffer (Feiyi Technology, China). 10% polyacrylamide gel, polyvinylidene difluoride western blotting membranes (Roche, Mannheim, Germany) and antibody of FADS1 (1:1 000, Cat No. ab126706; Abcam, MA, USA), and β-actin Antibody(5B7)-HRP conjugated (1:4 000, Cat No. Catalog: PMK058M, BioPM, China) were used.

### Cell migration assays

HIOEC were seeded in a six-well culture plate and grown to confluence. Then a straight line “scratch” was induced in the cell monolayer with a sterile p200 pipette tip. Cell debris on the edge of the scratch was removed by washing with a culture medium. The plate was then placed in a cell culture incubator for 48 h, and images were taken every 12 h.

## Supplementary information

Supplementary Figures and tables

## Data Availability

The data used and/or analyzed during the current study are contained within the manuscript. RNA-seq, ATAC-seq, and DLO-HiC sequencing data are available at the Genome Sequence Archive (http://bigd.big.ac.cn/gsaor, http://gsa.big.ac.cn) with BioProject accession: PRJCA008064. The most updated HIOEC Epigenome data can be accessed by “Add Hub” in the UCSC genome browser (https://data.cyverse.org/dav-anon/iplant/home/huan_liu/HIOEC_Epi_hub/hub.txt). Other data are available from the corresponding author on reasonable request.
