# Supplementary material for: Chromatin conformation of human oral epithelium can identify orofacial cleft missing functional variants
Source: Int J Oral Sci. 2022 Aug 25;14:43. doi: 10.1038/s41368-022-00194-0 (PMC9411193; doi:10.1038/s41368-022-00194-0)
Supplement: Supplementary file 1 — Supplementary Figures and tables [file 41368_2022_194_MOESM1_ESM.pdf]

Supplementary materials

Chromatin conformation of human oral epithelium can identify orofacial cleft missing functional variants

Yao Xiao[1,5], Shengbo Jiao[2,3], Miao He[1], Da Lin[4], Huanyan Zuo[1], Jiahao Han[1], Yonghua Sun[2,3], Gang Cao[4], Zhi Chen[1*], Huan Liu[1,6*]

[1]The State Key Laboratory Breeding Base of Basic Science of Stomatology & Key Laboratory for Oral Biomedicine of Ministry of Education, School and Hospital of Stomatology, Wuhan University, 237 Luoyu Road, Wuhan 430079, China

[2]State Key Laboratory of Freshwater Ecology and Biotechnology, Hubei Hongshan Laboratory, Institute of Hydrobiology, Innovation Academy for Seed Design, Chinese Academy of Sciences, Wuhan 430072, China

[3]College of Advanced Agricultural Sciences, University of Chinese Academy of Sciences, Beijing 100049, China

[4]State Key Laboratory of Agricultural Microbiology, Huazhong Agricultural University, Wuhan, China.

[5]Department of Stomatology, Nanfang Hospital, Southern Medical University, Guangzhou, P.R. China.

[6]Taikang Center for Life and Medical Sciences, Wuhan University, Wuhan 430071, China

These authors contributed equally: Yao Xiao, Shengbo Jiao

***Corresponding authors:**

***Zhi Chen**, DDS, PhD

Professor

The State Key Laboratory Breeding Base of Basic Science of Stomatology (Hubei-MOST)

Department of Endodontics

School and Hospital of Stomatology Wuhan University
237, Luoyu Road
Wuhan, China, 430079
Tel: +86-27-87686198; Fax: +86-27-87873849
Email: zhichen@whu.edu.cn
***Liu Huan**, DDS, PhD
Associate Professor
The State Key Laboratory Breeding Base of Basic Science of Stomatology (Hubei-MOST)
Associate Director for Department of Periodontology
School and Hospital of Stomatology Wuhan University
237, Luoyu Road
Wuhan, China, 430079
Tel.: 86-13476211742; Fax: +86-27-87873849
Email: liu.huan@whu.edu.cn

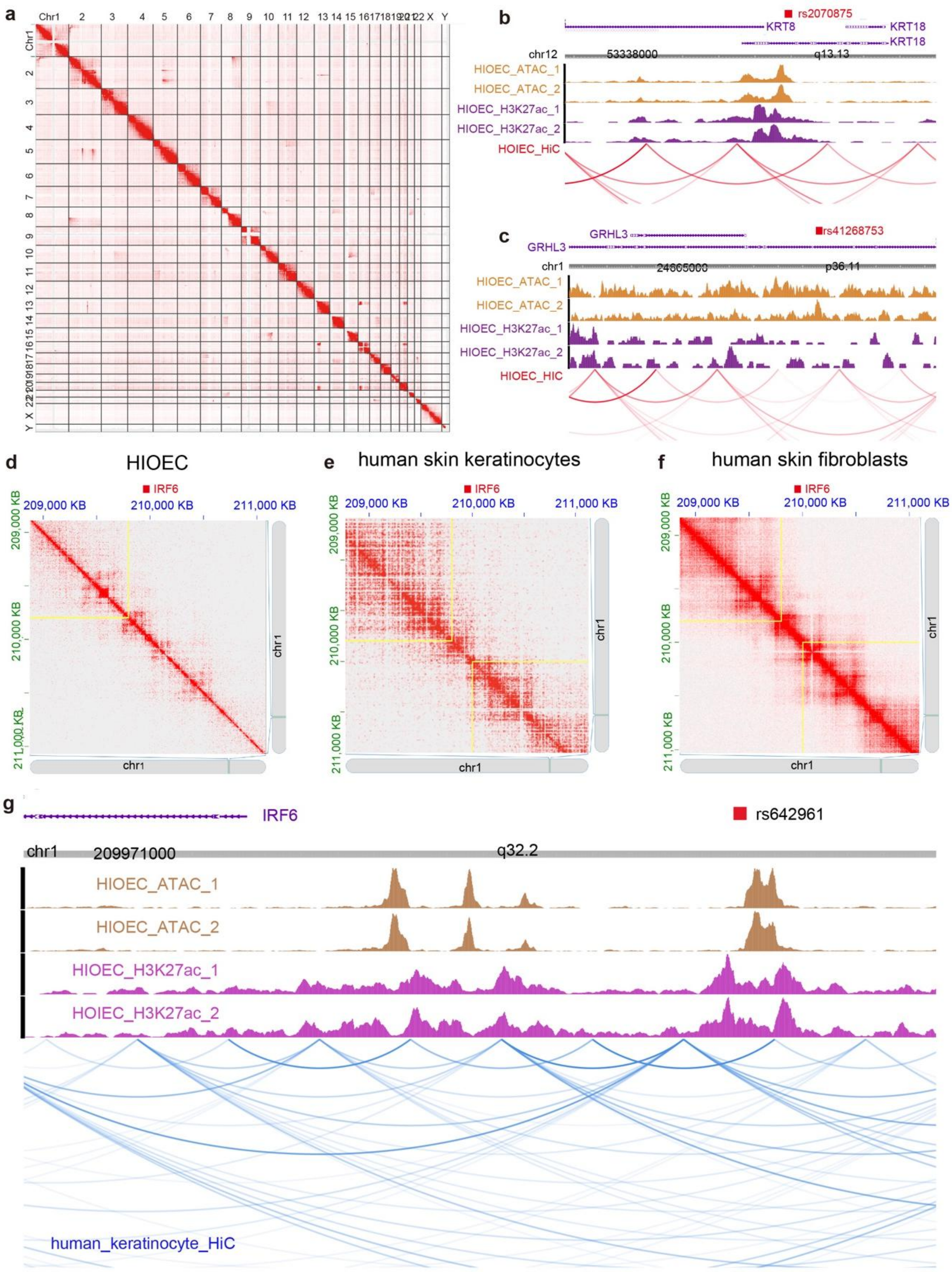

Supplementary Fig. 1 HIOEC DLO Hi-C analysis

a. Heatmap of HIOEC genome interactions from DLO Hi-C data at a resolution of 1Mb.

b. WashU epigenome browser tracks of HIOEC ATAC-seq, H3K27ac ChIP-seq and DLO Hi-C at genomic region near rs2070875. **c.** WashU epigenome browser tracks of HIOEC ATAC-seq, H3K27ac ChIP-seq and DLO Hi-C at genomic region near rs41268753. **d-f**. Heatmap of Hi-C data from HIOEC (**d**), human keratinocytes (**e**) and human skin fibroblasts (**f**). Yellow rectangles indicate TAD regions identified by each Hi-C. **g.** WashU epigenome browser tracks of HIOEC ATAC-seq, HIOEC H3K27ac ChIP-seq and arches of human keratinocytes Hi-C at genomic region near MCS9.7 (rs642961) which is also activate in skin keratinocytes.

a

GFP reporter plasmid information					
plasmis_name	insert_region	SNP		allele	risk allele
1	chr11: 62542688-62543434	rs11231210	chr11:62543180	G	A
1m				A	
2	chr11: 61597043-61597643	rs174570	chr11:61597212	G	A
2m				A	
3	chr11:71174114-71174715	rs2276362	chr11:71174452	G	A
3m				A	
4	chr11:117224765-117225422	rs499744	chr11:117225033	G	A
4m				A	
5	chr11:117228493-117229380	rs560789	chr11:117228892	A	G
5m				G	
10	chr17:39671379-39672046	rs2305556	chr17:39671724	A	G
10m				G	
11	chr11:118949022-118949735	rs1177562	chr11:118949331	G	A
11m				A	
12	chr11:126272909-126273647	rs12574844	chr11:126273386	G	A
12m				A	

b

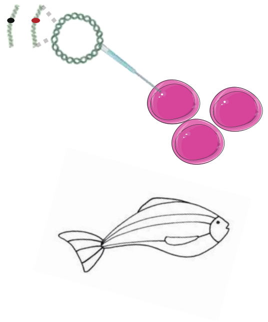

c

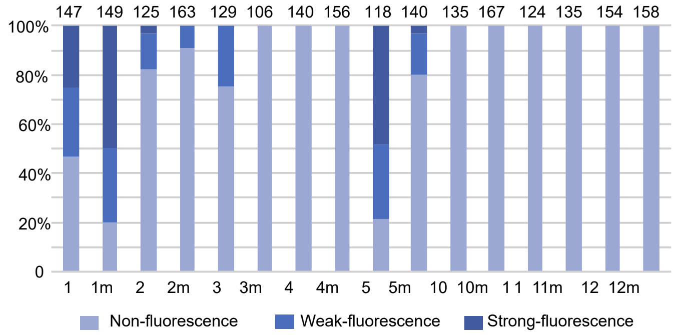

Supplementary Fig. 2 Effects of SNPs on enhancer activity in zebrafish reporter assays.

a. Table of information for the GFP reporter constructs. **b.** Schematic of zebrafish reporter assays. Reporter constructs were injected into 1-cell stage embryos and fluorescence intensity was checked at 11 hpf. **c.** 100% stacked column showing the relative percentage of the number of zebrafish embryos that expressed strong, weak, or non-fluorescent after GFP reporter injection. The numbers above each column represent the number of embryos injected for each reporter constructs.

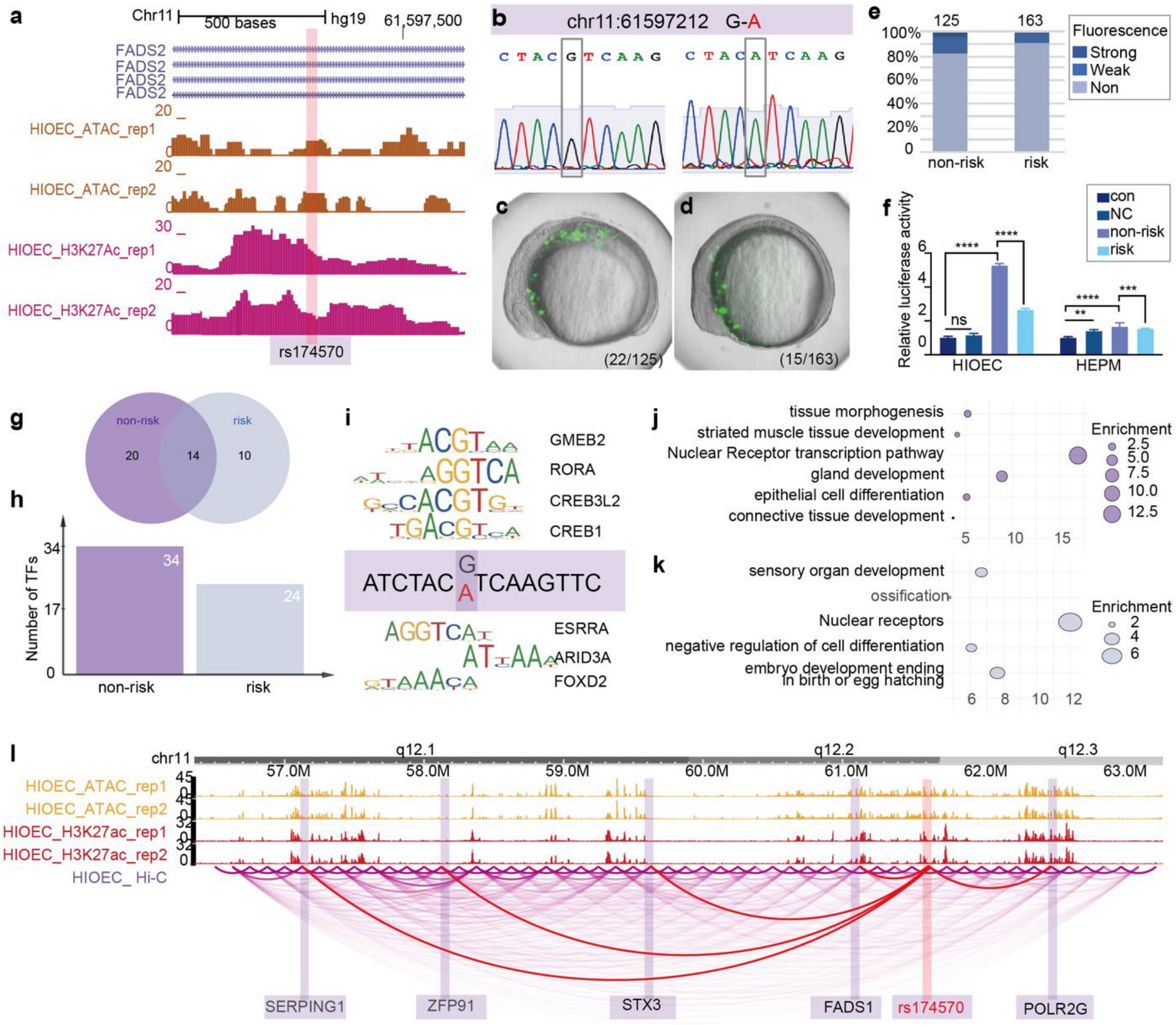

Supplemental Fig. 3. The risk allele of rs174570 reduced activity of epithelial-specific enhancer and prevented the binding of transcription factors related to epithelial development.

a. UCSC genome browser tracks of genomic region containing rs174570 that inserted into GFP reporter constructs and luciferase-based reporter vector. **b.** Sanger-sequencing results of the products cloned into the GFP reporter plasmid showing the mutation of non-risk allele (A) to risk allele (G) in rs174570 (located at chr11:61597212). **c-d.** Lateral views of wild-type zebrafish embryos at 11 hpf injected at the 1-cell stage with GFP reporter constructs inserted with enhancer containing non-risk allele (**c**) or risk allele (**d**). **e.** 100% stacked column showing the relative percentage of the number of zebrafish embryos that expressed strong, weak, or non-fluorescent after GFP injection. 125 and 163 zebrafish embryos were injected with plasmids containing fragments with non-risk allele and risk allele of rs174570, respectively. **f.** Dual luciferase assay for non-risk and risk allele of rs174570 in HIOEC and HEPM cells. con, group without transfection; NC, group transfected with pGL3-promoter vector; ns, non-significance; ****, p<0.0001. **g.** The number of TF motifs containing rs174570-risk or -non-risk alleles

identified by Jaspar2022. **h.** Venn diagram showing 20 transcription factor motifs lost and 10 transcription factor motifs gained after rs174570 mutated to risk allele. **i.** Examples of transcription factor that were lost or gained with rs174570 mutation of risk allele. **j.** Dot plot showed the Gene Ontology (GO) enrichment assay for the 34 transcription factors in non-risk allele. –log(P value) and false discovery rate (FDR) (enrichment value) for each term were generated by Metascape. **k.** Dot plot showed the Gene Ontology (GO) enrichment assay for the 24 transcription factors in risk allele. **l.** The new WashU epigenome browser tracks showing assay for ATAC-seq, H3K27ac ChIP-seq and DLO Hi-C results of HIOEC at chr11:56Mb-63.5Mb; ATAC-seq and H3K27ac ChIP-seq results showed 2 replicates. Regions marked with light purple show examples of the promoters interacted with rs174570. Red arcs showed the interactions.

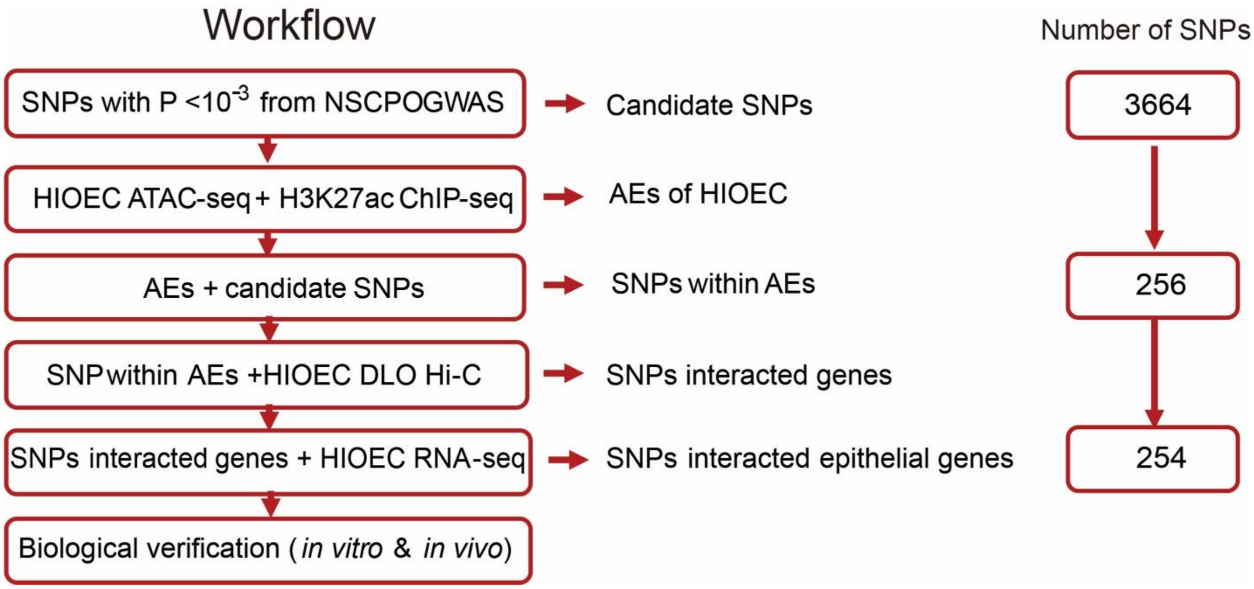

Supplementary Fig. 4 Workflow of screening an example NSCPO risk SNPs using oral epithelium functional genomics dataset.

3664 SNPs with P < 10^{-3} from NSCPO GWAS data were selected as candidate SNPs for further analysis**.** 256 SNPs within active enhancers/promoters (AEs) of HIOEC which identified from HIOEC ATAC-seq and H3K27ac ChIP-seq reanalysis were potential risk variants. HIOEC DLO Hi-C revealed genes that pysically interacted with risk variants, and HIOEC RNA-seq screened genes expressed in oral epithelial cells. Finally, 254 SNPs interacted with epithelial genes were obtained from our pipeline.

Supplementary Table 1. Information of SNPs with p < 10-6 from a non-syndromic cleft palate only (NSCPO) GWAS data of the Chinese Han population.

CHR	SNP	BP	A1	F_A	F_U	A2	CHISQ	P	OR
5	kgp4409118	132847845	G	0.2954	0.3663	C	30.2	3.64E-09	0.6897
11	rs11038167	44843134	A	0.5018	0.4336	C	24.65	1.02E-08	1.394
5	kgp4953068	73317553	G	0.1082	0.1602	A	27.37	4.08E-08	0.6196
12	rs10881108	47816463	A	0.03004	0.05889	G	23.95	6.42E-08	0.4422
11	rs11522752	71864717	C	0.1256	0.1765	A	25.2	6.72E-08	0.6407
10	kgp4603105	80956183	A	0.04167	0.07539	G	25.21	1.09E-07	0.5027
11	kgp10591163	30701518	G	0.01125	0.03167	A	22.69	1.38E-07	0.289
13	kgp16758336	93882459	G	0.01	0.03148	A	25.94	2.13E-07	0.278
19	rs10409042	3361995	A	0.2182	0.2716	G	20.46	2.40E-07	0.7029
13	kgp7664727	19761203	C	0.05752	0.09364	A	23.19	2.66E-07	0.5589
14	rs7156264	39673991	A	0.2879	0.3499	G	24.04	2.84E-07	0.7281
6	rs7745450	143308368	G	0.04075	0.0747	A	25.71	3.11E-07	0.5109
1	kgp1782951	15358288	A	0.0271	0.05419	G	23.09	3.25E-07	0.4472
15	rs3765115	76673716	A	0.3893	0.4602	G	30.27	3.52E-07	0.7557
9	rs10759848	119323187	A	0.05818	0.0952	C	23.12	3.52E-07	0.5611
15	rs2461870	76813920	A	0.3897	0.4599	C	29.7	3.71E-07	0.7555
10	rs1916527	57401749	C	0.3468	0.4128	A	24.22	4.23E-07	0.7318
11	rs11029442	26280017	A	0.05749	0.09077	G	19.35	4.36E-07	0.5644
10	rs2789698	81895970	A	0.3962	0.4634	G	24.09	5.53E-07	0.7454
8	rs11778385	134338367	A	0.4186	0.3519	G	25.51	6.54E-07	1.338
15	rs2469249	76772062	G	0.3925	0.4621	A	29.14	6.55E-07	0.7606
4	rs17023845	148727355	A	0.03622	0.0672	C	23.72	7.57E-07	0.5044
8	rs17678093	16187899	G	0.08097	0.1265	A	27.46	7.59E-07	0.624
21	rs2838625	46096731	A	0.2769	0.3381	G	24.03	7.72E-07	0.7396
11	rs11231210	62543180	G	0.08782	0.1267	A	20.55	9.93E-07	0.6335

Supplementary Table 2. Information of SNPs located within axons or promoter.

SNP	p value in GWAS	Gene	TPM	type
kgp11268193	0.000828	RPS5	924.438667	Exon
kgp19521263	6.32E-05	NDRG2	3.00269533	Exon
kgp2414760	0.000539	CSPG4	4.08915667	Exon
kgp9487626	0.00048	TPST2	70.0729	Promoter
rs1034405	0.000529	C3orf18	0.71762	Exon
rs1045411	0.000557	HMGB1	346.656333	Exon
rs10457	0.000417	WDR91	7.98332333	Exon
rs1048315	0.000316	SYNE2	10.8364467	Exon
rs1049868	0.000204	GSE1	7.16924333	Exon
rs1053328	1.42E-06	GINS2	9.70276	Exon
rs1058202	0.000806	DLG5	26.7311333	Exon
rs11204	0.000459	CALD1	31.771	Exon
rs1127648	0.000334	CSPG4	4.08915667	Exon
rs11865	3.98E-05	GSE1	7.16924333	Exon
rs1736911	1.54E-05	HLA-F	19.2622667	Exon
rs1736921	0.000335	HLA-F	19.2622667	Exon
rs174535	0.000576	TMEM258	135.59	Exon
rs174538	0.000584	TMEM258	135.59	Promoter
rs174546	0.000401	FADS1	47.0595667	Exon
rs17746075	5.75E-05	HLF	1.58839	Exon
rs2013162	0.000411	IRF6	112.448333	Exon
rs2076183	2.47E-05	HLA-F	19.2622667	Exon
rs2235371	0.000541	IRF6	112.448333	Exon
rs2272874	0.00023	HLA-F	19.2622667	Exon
rs2276180	0.000915	DCUN1D5	85.5320667	Exon
rs2293362	0.000228	TNS3	19.5644333	Exon
rs2305556	0.000459	KRT15	1.04142867	Exon
rs2899365	0.00052	PACSIN2	1.62567433	Exon
rs292557	0.000168	WDR91	7.98332333	Exon
rs292575	0.000159	WDR91	7.98332333	Exon
rs292592	0.000338	WDR91	7.98332333	Exon
rs3134428	1.99E-05	UBASH3B	3.97790667	Exon
rs3177979	0.000662	OAS1	16.4691	Exon
rs3734462	0.0007	AGPAT4	0.967637	Exon
rs3734813	0.000423	HLA-F	19.2622667	Exon
rs3741981	0.000941	OAS1	16.4691	Exon
rs3747134	1.50E-05	HPS4	18.1768667	Exon
rs3751978	0.000696	CBX1	140.867	Exon
rs3822853	0.000909	AGPAT4	0.967637	Exon

Supplementary Table 3. Active enhancer/promoters (AEs) of HIOEC.

chr1	9982	10371
chr1	13633	13832
chr1	20419	20618
chr1	28576	30483
chr1	30627	30752
chr1	31499	31712
chr1	33029	33228
chr1	34791	35071
chr1	242327	242536
chr1	242824	243264
chr1	335374	335518
chr1	653959	654033
chr1	666984	667158
chr1	676059	676337
chr1	679959	680200
chr1	713352	714158
chr1	714288	715434
chr1	724172	724516
chr1	726035	726080
chr1	726148	726313
chr1	726774	727072
chr1	762040	763598
chr1	787289	787525
chr1	833331	833462
chr1	839783	840686
chr1	842446	842645
chr1	875869	876272
chr1	877083	877408
chr1	878001	878387
chr1	893496	893618
chr1	893701	894922
chr1	895037	895830
chr1	895957	896756
chr1	901410	902853
chr1	911527	911867
chr1	912151	912574
chr1	933720	938346
chr1	941075	941301
chr1	942257	942461
chr1	942813	942902
chr1	944241	944558
chr1	944695	944864
chr1	945104	945168
chr1	946117	946316

chr1	947508	947673
chr1	948063	948704
chr1	948978	949206
chr1	949387	949601
chr1	949900	950214
chr1	951635	951697
chr1	952012	952257
chr1	954331	955351
chr1	955475	957363
chr1	957521	958275
chr1	959612	959880
chr1	960142	961605
chr1	961732	962234
chr1	993258	993499
chr1	993872	994952
chr1	995283	995763
chr1	997129	997388
chr1	998215	998558
chr1	998820	999083
chr1	999323	1000261
chr1	1003736	1004272
chr1	1004497	1004771
chr1	1009410	1009706
chr1	1013757	1016558
chr1	1050784	1051465
chr1	1051848	1052215
chr1	1079250	1079522
chr1	1080018	1080725
chr1	1093052	1093770
chr1	1098304	1100555
chr1	1136483	1137709
chr1	1145171	1145440
chr1	1153028	1153328
chr1	1165921	1166661
chr1	1166824	1167400
chr1	1167842	1168894
chr1	1169097	1169296
chr1	1176485	1176822
chr1	1181849	1182258
chr1	1186588	1186997
chr1	1197176	1197250
chr1	1199881	1201311
chr1	1202345	1202544
chr1	1207136	1208032
chr1	1208182	1209933
chr1	1217505	1217704

chr1	1238835	1241424
chr1	1242076	1242275
chr1	1242724	1243817
chr1	1243988	1245118
chr1	1245229	1245668
chr1	1252152	1252250
chr1	1253018	1253192
chr1	1253561	1253644
chr1	1253875	1254158
chr1	1258927	1261090
chr1	1279229	1280287
chr1	1280413	1281022
chr1	1281161	1281361
chr1	1283014	1284505
chr1	1284936	1285295
chr1	1286760	1286954
chr1	1289185	1289778
chr1	1290930	1291230
chr1	1298732	1298802
chr1	1299895	1300098
chr1	1309408	1310598
chr1	1311440	1311730
chr1	1310945	1311163
chr1	1317547	1317793
chr1	1325101	1325599
chr1	1334153	1335833
chr1	1338347	1338908
chr1	1340248	1340339
chr1	1340437	1340541
chr1	1341619	1341668
chr1	1341894	1343781
chr1	1344961	1345246
chr1	1355281	1355547
chr1	1355650	1357311
chr1	1357454	1358687
chr1	1359198	1359625
chr1	1360286	1360481
chr1	1362622	1363374
chr1	1368377	1368583
chr1	1368802	1369707
chr1	1371049	1371464
chr1	1392996	1393238
chr1	1396436	1396660
chr1	1396759	1398103
chr1	1399012	1399081
chr1	1399783	1400666

chr1	1405076	1405156
chr1	1405499	1405782
chr1	1405992	1406578
chr1	1406681	1407486
chr1	1407733	1408870
chr1	1409792	1410128
chr1	1411975	1412291
chr1	1440558	1440951
chr1	1441186	1441255
chr1	1441334	1441548
chr1	1446231	1446270
chr1	1446441	1447349
chr1	1447534	1448830
chr1	1449024	1449260
chr1	1450306	1450693
chr1	1509371	1511060
chr1	1511148	1511413
chr1	1522445	1522585
chr1	1522740	1522867
chr1	1530277	1530477
chr1	1530562	1530813
chr1	1534425	1534750
chr1	1534879	1535311
chr1	1535544	1535880
chr1	1544659	1544790
chr1	1547178	1547412
chr1	1548945	1549267
chr1	1549865	1550509
chr1	1550761	1552108
chr1	1562376	1562586
chr1	1562887	1564162
chr1	1564306	1568016
chr1	1568221	1568720
chr1	1568920	1569190
chr1	1580270	1580469
chr1	1590034	1591129
chr1	1591762	1592134
chr1	1592345	1592445
chr1	1593075	1593292
chr1	1593427	1593492
chr1	1594037	1594995
chr1	1604775	1605020
chr1	1616640	1616932
chr1	1617518	1618607
chr1	1618733	1618814
chr1	1619670	1620101

chr1	1620236	1620933
chr1	1621353	1621536
chr1	1621928	1622133
chr1	1623132	1625537
chr1	1626339	1626636
chr1	1627054	1627187
chr1	1631054	1632209
chr1	1655296	1655758
chr1	1655984	1656381
chr1	1656495	1656671
chr1	1657091	1657316
chr1	1659712	1660792
chr1	1667393	1667964
chr1	1671676	1672086
chr1	1672352	1673633
chr1	1673965	1674977
chr1	1676264	1677070
chr1	1677260	1677459
chr1	1677574	1677864
chr1	1678038	1678935
chr1	1689341	1690387
chr1	1694602	1694889
chr1	1695448	1695689
chr1	1695984	1696183
chr1	1699739	1699938
chr1	1704656	1704842
chr1	1707344	1707539
chr1	1709071	1711303
chr1	1712926	1713892
chr1	1714246	1714840
chr1	1715422	1715502
chr1	1736319	1736529
chr1	1760711	1760899
chr1	1766938	1767100
chr1	1767310	1768072
chr1	1788891	1789071
chr1	1790046	1790245
chr1	1790511	1791101
chr1	1791592	1791818
chr1	1792408	1792791
chr1	1794925	1795048
chr1	1805795	1805870
chr1	1805980	1806205
chr1	1806851	1807032
chr1	1815133	1815366
chr1	1821264	1824003

chr1	1824150	1824835
chr1	1825783	1826036
chr1	1828087	1828169
chr1	1828534	1829139
chr1	1836213	1836316
chr1	1837378	1840561
chr1	1840801	1841813
chr1	1850300	1850795
chr1	1851108	1851408
chr1	1874688	1874971
chr1	1875188	1875464
chr1	1889449	1890011
chr1	1890243	1890442
chr1	1890815	1891115
chr1	1891467	1892309
chr1	1975556	1975626
chr1	1975705	1976993
chr1	1977181	1977390
chr1	1977735	1977934
chr1	1980584	1980783
chr1	1981095	1981647
chr1	1981878	1982877
chr1	1990117	1990316
chr1	2064418	2064819
chr1	2065137	2065432
chr1	2071319	2071521
chr1	2071771	2072128
chr1	2072846	2073074
chr1	2118913	2119134
chr1	2120354	2120922
chr1	2121207	2121891
chr1	2122412	2122696
chr1	2124740	2126756
chr1	2131693	2131777
chr1	2130270	2131393
chr1	2135892	2136091
chr1	2136801	2137455
chr1	2137927	2138290
chr1	2138588	2140544
chr1	2141158	2141542
chr1	2141719	2142143
chr1	2142413	2142715
chr1	2143351	2143915
chr1	2144053	2145050
chr1	2145231	2145430
chr1	2157245	2157327

chr1	2158218	2159124
chr1	2160712	2162009
chr1	2162247	2162753
chr1	2162895	2163120
chr1	2166290	2167008
chr1	2176862	2176885
chr1	2177264	2177533
chr1	2186329	2186710
chr1	2186884	2188017
chr1	2190002	2190209
chr1	2219558	2219726
chr1	2219790	2219828
chr1	2221816	2222026
chr1	2222488	2223087
chr1	2245493	2246675
chr1	2253297	2253496
chr1	2253807	2254085
chr1	2266256	2266455
chr1	2321626	2322189
chr1	2322257	2322987
chr1	2323251	2324155
chr1	2324223	2324503
chr1	2342828	2345340
chr1	2347436	2347756
chr1	2347890	2348326
chr1	2358523	2361233
chr1	2362548	2362747
chr1	2362874	2363522
chr1	2367563	2367762
chr1	2378302	2378501
chr1	2380735	2380992
chr1	2382520	2382719
chr1	2382965	2383284
chr1	2383498	2384096
chr1	2384804	2385417
chr1	2437986	2438343
chr1	2454411	2454780
chr1	2455102	2455480
chr1	2457224	2457660
chr1	2457847	2458355
chr1	2460748	2462516
chr1	2464091	2464413
chr1	2464658	2466027
chr1	2466705	2467095
chr1	2477787	2479034
chr1	2479270	2479616

chr1	2479733	2480746
chr1	2487182	2488441
chr1	2516991	2517970
chr1	2518076	2519119
chr1	2573722	2574593
chr1	3369577	3369793
chr1	3369968	3370237
chr1	3370418	3371621
chr1	3377313	3377597
chr1	3381574	3382437
chr1	3383081	3385307
chr1	3387363	3389315
chr1	3389526	3390855
chr1	3391018	3391335
chr1	3398255	3398679
chr1	3398795	3399714
chr1	3399900	3400228
chr1	3447073	3447390
chr1	3447614	3448008
chr1	3456896	3457125
chr1	3457326	3457578
chr1	3539972	3540171
chr1	3540752	3540974
chr1	3541454	3542219
chr1	3565978	3566552
chr1	3569115	3569314
chr1	3591273	3591472
chr1	3594685	3594804
chr1	3594296	3594549
chr1	3595547	3595961
chr1	3596135	3596162
chr1	3600411	3601180
chr1	3601500	3601829
chr1	3605343	3605987
chr1	3606381	3607067
chr1	3612171	3612830
chr1	3614282	3614783
chr1	3614939	3615334
chr1	3615641	3616475
chr1	3617208	3617631
chr1	3619304	3619541
chr1	3619665	3620323
chr1	3623607	3624812
chr1	3625870	3626069
chr1	3627893	3629184
chr1	3629300	3629927

chr1	3632185	3632476
chr1	3632677	3634653
chr1	3636439	3636711
chr1	3660348	3660652
chr1	3663457	3663905
chr1	3664207	3664293
chr1	3688557	3689069
chr1	3689197	3689644
chr1	3710404	3711183
chr1	3712238	3713007
chr1	3713176	3713876
chr1	3773033	3773069
chr1	3773347	3773730
chr1	3774023	3774860
chr1	3816396	3816829
chr1	3817090	3818261
chr1	3819015	3819253
chr1	4714374	4714665
chr1	4714893	4715123
chr1	4715521	4715791
chr1	4728912	4729228
chr1	4729307	4729657
chr1	4898593	4898660
chr1	5961598	5961616
chr1	6052050	6052580
chr1	6052756	6053983
chr1	6054177	6054461
chr1	6075788	6075842
chr1	6075960	6076020
chr1	6085563	6086194
chr1	6086482	6086761
chr1	6087092	6087299
chr1	6216352	6216646
chr1	6217546	6217830
chr1	6218148	6218425
chr1	6252707	6252795
chr1	6253119	6253866
chr1	6257192	6257235
chr1	6258883	6259679
chr1	6259886	6260117
chr1	6264654	6265323
chr1	6265464	6266789
chr1	6279508	6280496
chr1	6280641	6280817
chr1	6282731	6282862
chr1	6289283	6289505

chr1	6294476	6295360
chr1	6295608	6296025
chr1	6296205	6296440
chr1	6309369	6309617
chr1	6310349	6310551
chr1	6320403	6320975
chr1	6321263	6321599
chr1	6328039	6328244
chr1	6355542	6355860
chr1	6359796	6360074
chr1	6360188	6361680
chr1	6362010	6362465
chr1	6377066	6377562
chr1	6385484	6385536
chr1	6385814	6386059
chr1	6403188	6403457
chr1	6404111	6404349
chr1	6404517	6404902
chr1	6409983	6410068
chr1	6410175	6410623
chr1	6411990	6412758
chr1	6412894	6413226
chr1	6413572	6414053
chr1	6414196	6414401
chr1	6414650	6414849
chr1	6417049	6417291
chr1	6417356	6417405
chr1	6417837	6418905
chr1	6419302	6419752
chr1	6425002	6425506
chr1	6425588	6425688
chr1	6426468	6426667
chr1	6427447	6427661
chr1	6429093	6429381
chr1	6433994	6434108
chr1	6434958	6435131
chr1	6442466	6442505
chr1	6443078	6443357
chr1	6444862	6445078
chr1	6445184	6445920
chr1	6446061	6446980
chr1	6448798	6449031
chr1	6449652	6449708
chr1	6450235	6450412
chr1	6451829	6452067
chr1	6452783	6453866

chr1	6454042	6454465
chr1	6456273	6456490
chr1	6462126	6462211
chr1	6464705	6464765
chr1	6466125	6466731
chr1	6470308	6470525
chr1	6470603	6470908
chr1	6472872	6473099
chr1	6473219	6476320
chr1	6477621	6477975
chr1	6478356	6478678
chr1	6478875	6480775
chr1	6482633	6483005
chr1	6483737	6484245
chr1	6484467	6484692
chr1	6484820	6485357
chr1	6485579	6485778
chr1	6493434	6493633
chr1	6497752	6497951
chr1	6498867	6499294
chr1	6499457	6499823
chr1	6499931	6500343
chr1	6500786	6501027
chr1	6507121	6507619
chr1	6508666	6509167
chr1	6514766	6515564
chr1	6531317	6532227
chr1	6532383	6532603
chr1	6532750	6532949
chr1	6536121	6536325
chr1	6545140	6546671
chr1	6547058	6547474
chr1	6547811	6548331
chr1	6548471	6552618
chr1	6556872	6558085
chr1	6574657	6574662
chr1	6580421	6580765
chr1	6581509	6581638
chr1	6581998	6582012
chr1	6605229	6605264
chr1	6605344	6605530
chr1	6612746	6613160
chr1	6613630	6613840
chr1	6614118	6614635
chr1	6614740	6614995
chr1	6619144	6619372

chr1	6639993	6641159
chr1	6647938	6648137
chr1	6659361	6659896
chr1	6660003	6663339
chr1	6666123	6666255
chr1	6668246	6668495
chr1	6668777	6669008
chr1	6669444	6670359
chr1	6671701	6671862
chr1	6672613	6672632
chr1	6672751	6673575
chr1	6673795	6675609
chr1	6685233	6686595
chr1	6719759	6719816
chr1	6746516	6746626
chr1	6761075	6761397
chr1	6761635	6762501
chr1	6803018	6803240
chr1	6803620	6804033
chr1	6804161	6804288
chr1	6813687	6814297
chr1	6815115	6815520
chr1	6821652	6822207
chr1	6822397	6822672
chr1	6828804	6828929
chr1	6830757	6830992
chr1	6844161	6845011
chr1	6845922	6846369
chr1	6863054	6863282
chr1	6919676	6919817
chr1	7177347	7177668
chr1	7177821	7178137
chr1	7439679	7439922
chr1	7462189	7462414
chr1	7531612	7531964
chr1	7609497	7609696
chr1	7609990	7610667
chr1	7610971	7611170
chr1	7708197	7708396
chr1	7740437	7740643
chr1	7805760	7806073
chr1	7813139	7813164
chr1	7830841	7831917
chr1	7843137	7843885
chr1	7844378	7844999
chr1	7870411	7870449

chr1	7962767	7962935
chr1	8002559	8002829
chr1	8013898	8014660
chr1	8014908	8015107
chr1	8021053	8021400
chr1	8021736	8022217
chr1	8035254	8035364
chr1	8042602	8042716
chr1	8059031	8059368
chr1	8060140	8060364
chr1	8060714	8060913
chr1	8063540	8063782
chr1	8064413	8064859
chr1	8065064	8065653
chr1	8066110	8066770
chr1	8067073	8067311
chr1	8069424	8069530
chr1	8075125	8075373
chr1	8076442	8077131
chr1	8077649	8078029
chr1	8080071	8080498
chr1	8081774	8082077
chr1	8083200	8083597
chr1	8085058	8087659
chr1	8087887	8088691
chr1	8100607	8100822
chr1	8100976	8101053
chr1	8107248	8107527
chr1	8107697	8108577
chr1	8116050	8116191
chr1	8116325	8116534
chr1	8119355	8119554
chr1	8120732	8120973
chr1	8121244	8121738
chr1	8129310	8129398
chr1	8131365	8131576
chr1	8135548	8136059
chr1	8136706	8137040
chr1	8137382	8137657
chr1	8138189	8138809
chr1	8149864	8150063
chr1	8150509	8150865
chr1	8154769	8154897
chr1	8156631	8156810
chr1	8156924	8156994
chr1	8157309	8157939

chr1	8181419	8181854
chr1	8193975	8194540
chr1	8197777	8198190
chr1	8201940	8202348
chr1	8206693	8207004
chr1	8214771	8214912
chr1	8215120	8215152
chr1	8215561	8216152
chr1	8227434	8227724
chr1	8229976	8230539
chr1	8231042	8231052
chr1	8233286	8233587
chr1	8234447	8234797
chr1	8234887	8235212
chr1	8242116	8242830
chr1	8257072	8257387
chr1	8257556	8258844
chr1	8259923	8260131
chr1	8261195	8261955
chr1	8262367	8263524
chr1	8263757	8264028
chr1	8264210	8265461
chr1	8265739	8266097
chr1	8270842	8271041
chr1	8271329	8272984
chr1	8273335	8273414
chr1	8273621	8273678
chr1	8276962	8277109
chr1	8277841	8278017
chr1	8278808	8279216
chr1	8282145	8282200
chr1	8283399	8284006
chr1	8284716	8285360
chr1	8285614	8285892
chr1	8290392	8290591
chr1	8291335	8291463
chr1	8294507	8294764
chr1	8298638	8298695
chr1	8298862	8299627
chr1	8299968	8300056
chr1	8300268	8300459
chr1	8302145	8302226
chr1	8317597	8317910
chr1	8318092	8318229
chr1	8318631	8320326
chr1	8374512	8374943

chr1	8378152	8378433
chr1	8403387	8403604
chr1	8403754	8404123
chr1	8455310	8455485
chr1	8482929	8483792
chr1	8483933	8484637
chr1	8543460	8543543
chr1	8554002	8554318
chr1	8557889	8557969
chr1	8558337	8558475
chr1	8585313	8586049
chr1	8586230	8586423
chr1	8600234	8600708
chr1	8720798	8720857
chr1	8721250	8721915
chr1	8742681	8742753
chr1	8761557	8761759
chr1	8762223	8762546
chr1	8763002	8763900
chr1	8772464	8773179
chr1	8786442	8786660
chr1	8793512	8793617
chr1	8793851	8794118
chr1	8854715	8855046
chr1	8856904	8857409
chr1	8857591	8857681
chr1	8877056	8878398
chr1	8878606	8878854
chr1	8882845	8882935
chr1	8883050	8883177
chr1	8911799	8912073
chr1	8914987	8915479
chr1	8919760	8919906
chr1	8926645	8926832
chr1	8931059	8931266
chr1	8931739	8932055
chr1	8932245	8932370
chr1	8933552	8933772
chr1	8933943	8934508
chr1	8936523	8939775
chr1	8940088	8940528
chr1	8941134	8941376
chr1	8942579	8942793
chr1	8945466	8945692
chr1	8948241	8948406
chr1	8949254	8949563

chr1	8949963	8950945
chr1	8956816	8956832
chr1	8957494	8957876
chr1	8959219	8959565
chr1	8959880	8960535
chr1	8960744	8960973
chr1	8962897	8963127
chr1	8963570	8963894
chr1	8964111	8964503
chr1	8965569	8966684
chr1	8967760	8967907
chr1	8970789	8971301
chr1	8971442	8971865
chr1	8978132	8978546
chr1	8984134	8984333
chr1	9001856	9001958
chr1	9015384	9015423
chr1	9033958	9034123
chr1	9046074	9046413
chr1	9046558	9046926
chr1	9047046	9047240
chr1	9050962	9051228
chr1	9087137	9087187
chr1	9097670	9097742
chr1	9097807	9098338
chr1	9098919	9099168
chr1	9099623	9099952
chr1	9127219	9127679
chr1	9129176	9129294
chr1	9129405	9129661
chr1	9130408	9130589
chr1	9140744	9141437
chr1	9167696	9167773
chr1	9169344	9169708
chr1	9169788	9169868
chr1	9170025	9170338
chr1	9170558	9170876
chr1	9173401	9173600
chr1	9173826	9174010
chr1	9174769	9175138
chr1	9176998	9177192
chr1	9187945	9188347
chr1	9188452	9189233
chr1	9189480	9190025
chr1	9218149	9218348
chr1	9224388	9224609

chr1	9241530	9243324
chr1	9249140	9249635
chr1	9254391	9254519
chr1	9256275	9256474
chr1	9256583	9257091
chr1	9261120	9261431
chr1	9261538	9261868
chr1	9262256	9262455
chr1	9288426	9288803
chr1	9289528	9289979
chr1	9291387	9291720
chr1	9291902	9292444
chr1	9292751	9292961
chr1	9293025	9294664
chr1	9294792	9295557
chr1	9297261	9297511
chr1	9301458	9301657
chr1	9302464	9302504
chr1	9302645	9302920
chr1	9303296	9303373
chr1	9326930	9327305
chr1	9330940	9331066
chr1	9335839	9336126
chr1	9336437	9336746
chr1	9337062	9337133
chr1	9341009	9341290
chr1	9349928	9351194
chr1	9351857	9352069
chr1	9352324	9354711
chr1	9355013	9355637
chr1	9359406	9359628
chr1	9359901	9360291
chr1	9360570	9361042
chr1	9361307	9361515
chr1	9362536	9362717
chr1	9362874	9363278
chr1	9363425	9363589
chr1	9363860	9364031
chr1	9376113	9376504
chr1	9377476	9379224
chr1	9381685	9381925
chr1	9391464	9391737
chr1	9399976	9400420
chr1	9410143	9410390
chr1	9449202	9449237
chr1	9470955	9471206

chr1	9471473	9471689
chr1	9473107	9474173
chr1	9488703	9488876
chr1	9489248	9489719
chr1	9498548	9499392
chr1	9503462	9503578
chr1	9503974	9504849
chr1	9506086	9506155
chr1	9536217	9536476
chr1	9536628	9537011
chr1	9590966	9590984
chr1	9591118	9591267
chr1	9591808	9591823
chr1	9599113	9599272
chr1	9599507	9600329
chr1	9601450	9601638
chr1	9615387	9615445
chr1	9615670	9615866
chr1	9621509	9621945
chr1	9647451	9647629
chr1	9648393	9648530
chr1	9648797	9649109
chr1	9649257	9649759
chr1	9653260	9653844
chr1	9686655	9687010
chr1	9692813	9693075
chr1	9704863	9705209
chr1	9705702	9705862
chr1	9711748	9712291
chr1	9718809	9719038
chr1	9719328	9719894
chr1	9721843	9722100
chr1	9731312	9731845
chr1	9734856	9735052
chr1	9741535	9741751
chr1	9745750	9746046
chr1	9747153	9747621
chr1	9748946	9749019
chr1	9747945	9748822
chr1	9749290	9749489
chr1	9749644	9750376
chr1	9753326	9753492
chr1	9759616	9760017
chr1	9760121	9760650
chr1	9760897	9761096
chr1	9761328	9761600

chr1	9765306	9765608
chr1	9779115	9779973
chr1	9785451	9785578
chr1	9813653	9813837
chr1	9814606	9814834
chr1	9818446	9819207
chr1	9823148	9823361
chr1	9825903	9826626
chr1	9826697	9826887
chr1	9827216	9827755
chr1	9829470	9829693
chr1	9835697	9836500
chr1	9842844	9843094
chr1	9858434	9858482
chr1	9864251	9864608
chr1	9864808	9865591
chr1	9869246	9869666
chr1	9870118	9870318
chr1	9872455	9872464
chr1	9872576	9872758
chr1	9882934	9884027
chr1	9884218	9885090
chr1	9885157	9885189
chr1	9885357	9885663
chr1	9885896	9886149
chr1	9894546	9895261
chr1	9897459	9897856
chr1	9895390	9897108
chr1	9900091	9900191
chr1	9900297	9900521
chr1	9900684	9900995
chr1	9902930	9903134
chr1	9911625	9911762
chr1	9912085	9912086
chr1	9912337	9912459
chr1	9912810	9913080
chr1	9916841	9917055
chr1	9924438	9924641
chr1	9925125	9925235
chr1	9925412	9925704
chr1	9926474	9926783
chr1	9929108	9929417
chr1	9929682	9929705
chr1	9930092	9930432
chr1	9934832	9935352
chr1	9936888	9937233

chr1	9937361	9937674
chr1	9937862	9938156
chr1	9940824	9940827
chr1	9941135	9941192
chr1	9941771	9941987
chr1	9943911	9944110
chr1	9948373	9948989
chr1	9949252	9950996
chr1	9951120	9951652
chr1	9952352	9953716
chr1	9954104	9955114
chr1	9955330	9955584
chr1	9957781	9957984
chr1	9958331	9958677
chr1	9958999	9959389
chr1	9969166	9970118
chr1	9970751	9971438
chr1	10001631	10001791
chr1	10002459	10002745
chr1	10002899	10003739
chr1	10010333	10010811
chr1	10018972	10019364
chr1	10056534	10058351
chr1	10078540	10078659
chr1	10078885	10078987
chr1	10091553	10091877
chr1	10092462	10092661
chr1	10094514	10094849
chr1	10093044	10093988
chr1	10101469	10101590
chr1	10101718	10101823
chr1	10103585	10104012
chr1	10115697	10116153
chr1	10117417	10117782
chr1	10118757	10118797
chr1	10123489	10125040
chr1	10126733	10127101
chr1	10133670	10133781
chr1	10143088	10143526
chr1	10168256	10168510
chr1	10168695	10168758
chr1	10251759	10251973
chr1	10269742	10269818
chr1	10270016	10270402
chr1	10270536	10271547
chr1	10271755	10272232

chr1	10289978	10290206
chr1	10290751	10291154
chr1	10295683	10295846
chr1	10310965	10311192
chr1	10311597	10311942
chr1	10312136	10312141
chr1	10312544	10312686
chr1	10317796	10318001
chr1	10325944	10326040
chr1	10401604	10401642
chr1	10437651	10438071
chr1	10447378	10448398
chr1	10448934	10449141
chr1	10458326	10458886
chr1	10459125	10460099
chr1	10460514	10460649
chr1	10460734	10461044
chr1	10463593	10463700
chr1	10475388	10475811
chr1	10487684	10487883
chr1	10488365	10488516
chr1	10490127	10490328
chr1	10490462	10491104
chr1	10491350	10491492
chr1	10497739	10497805
chr1	10504248	10504409
chr1	10505109	10505399
chr1	10515914	10516102
chr1	10516951	10517284
chr1	10517360	10518194
chr1	10518808	10519069
chr1	10519135	10519263
chr1	10519575	10519626
chr1	10520376	10520679
chr1	10521003	10521292
chr1	10525250	10525505
chr1	10532018	10532547
chr1	10532701	10532917
chr1	10533296	10533535
chr1	10534687	10535832
chr1	10536563	10536819
chr1	10539346	10539507
chr1	10542157	10542322
chr1	10554638	10554688
chr1	10555032	10555107
chr1	10555942	10556202

chr1	10556318	10556689
chr1	10570479	10570601
chr1	10570910	10571361
chr1	10588000	10589104
chr1	10621598	10621648
chr1	10635751	10635800
chr1	10658575	10658752
chr1	10659618	10659901
chr1	10679063	10679711
chr1	10680425	10680668
chr1	10693846	10693929
chr1	10694142	10696641
chr1	10697289	10697346
chr1	10698033	10698262
chr1	10703298	10703658
chr1	10753597	10754206
chr1	10755114	10755437
chr1	10783851	10784050
chr1	10791014	10791071
chr1	10791482	10791631
chr1	10800930	10801152
chr1	10855472	10855902
chr1	10856190	10856652
chr1	10864050	10865028
chr1	10865620	10865820
chr1	10877634	10877913
chr1	10878287	10878545
chr1	10878796	10879021
chr1	10881010	10881091
chr1	10880518	10880611
chr1	10882843	10883042
chr1	10883687	10884228
chr1	10884628	10885062
chr1	10885418	10885618
chr1	10885738	10886005
chr1	10890066	10890265
chr1	10891245	10891673
chr1	10892009	10892026
chr1	10927035	10927402
chr1	10927803	10928002
chr1	10951151	10951350
chr1	10951689	10951999
chr1	10957728	10957927
chr1	10958088	10958344
chr1	10959294	10960552
chr1	10960656	10962469

chr1	10963368	10963576
chr1	10979717	10979910
chr1	11025544	11026169
chr1	11069447	11069475
chr1	11071750	11073677
chr1	11073801	11074099
chr1	11081397	11081841
chr1	11086212	11086489
chr1	11115369	11115594
chr1	11115674	11115783
chr1	11118323	11118779
chr1	11118890	11119142
chr1	11119270	11119998
chr1	11120440	11120766
chr1	11121072	11121682
chr1	11121803	11121875
chr1	11159276	11160367
chr1	11160560	11160892
chr1	11199835	11200174
chr1	11208904	11209028
chr1	11216870	11217021
chr1	11218688	11218736
chr1	11231843	11232035
chr1	11322005	11322597
chr1	11322890	11323145
chr1	11323404	11323524
chr1	11333184	11333493
chr1	11334041	11334175
chr1	11334467	11334738
chr1	11375443	11375504
chr1	11376582	11377631
chr1	11538940	11539146
chr1	11539314	11539513
chr1	11704394	11704751
chr1	11705740	11705845
chr1	11713066	11713327
chr1	11714241	11714800
chr1	11714942	11715445
chr1	11723883	11725466
chr1	11740451	11741846
chr1	11779563	11779878
chr1	11790087	11790305
chr1	11790511	11790769
chr1	11791264	11793222
chr1	11795707	11797503
chr1	11799349	11799984

chr1	11803690	11803997
chr1	11841105	11841304
chr1	11844108	11844258
chr1	11846619	11846818
chr1	11846992	11847213
chr1	11847520	11847719
chr1	11847837	11848104
chr1	11849651	11849855
chr1	11850409	11851084
chr1	11851211	11851520
chr1	11851664	11852016
chr1	11852128	11852497
chr1	11856563	11856926
chr1	11857597	11857660
chr1	11860073	11860432
chr1	11862931	11863173
chr1	11863304	11864541
chr1	11864715	11866016
chr1	11866197	11866979
chr1	11867135	11867433
chr1	11869497	11869727
chr1	11881270	11881968
chr1	11899450	11899566
chr1	11923161	11923301
chr1	11951984	11952063
chr1	11956762	11956902
chr1	11961403	11961591
chr1	11961719	11962001
chr1	11963030	11963247
chr1	11967697	11968217
chr1	11968400	11970061
chr1	11970516	11970686
chr1	11974607	11974838
chr1	11975970	11976253
chr1	11984678	11984727
chr1	11985755	11986281
chr1	11986630	11986870
chr1	11988155	11988399
chr1	11988502	11988684
chr1	11989056	11990178
chr1	11990722	11990962
chr1	11991074	11991281
chr1	11991423	11991603
chr1	11994211	11995237
chr1	11999078	11999277
chr1	11999690	11999924

chr1	12026908	12027108
chr1	12027956	12028505
chr1	12028790	12029009
chr1	12033434	12033618
chr1	12039120	12039310
chr1	12039514	12039896
chr1	12040169	12040973
chr1	12041171	12042479
chr1	12044982	12045201
chr1	12045640	12045970
chr1	12046170	12046264
chr1	12046336	12046369
chr1	12069110	12069147
chr1	12073903	12074190
chr1	12074646	12074891
chr1	12075181	12075834
chr1	12078445	12078615
chr1	12079083	12080184
chr1	12104943	12104944
chr1	12111871	12112699
chr1	12116317	12116475
chr1	12123270	12124917
chr1	12191930	12192116
chr1	12192540	12192800
chr1	12193488	12193763
chr1	12204323	12204711
chr1	12208568	12208840
chr1	12226803	12227399
chr1	12227532	12227750
chr1	12263907	12264391
chr1	12289736	12291676
chr1	12292225	12292395
chr1	12292559	12292738
chr1	12332490	12332517
chr1	12446103	12446300
chr1	12446520	12446589
chr1	12495271	12495520
chr1	12496713	12497091
chr1	12497367	12497497
chr1	12538666	12538865
chr1	12548747	12548785
chr1	12655511	12655710
chr1	12676998	12678400
chr1	12678615	12679016
chr1	12679376	12679702
chr1	12938675	12938849

chr1	13654104	13654262
chr1	13910159	13911188
chr1	13913789	13914066
chr1	13914381	13914624
chr1	13920566	13920693
chr1	13926442	13926622
chr1	13946465	13946570
chr1	13990918	13991028
chr1	14026012	14026240
chr1	14026839	14027139
chr1	14075269	14075788
chr1	14076045	14076538
chr1	14083498	14083664
chr1	14085659	14086028
chr1	14093742	14093794
chr1	14125580	14125756
chr1	14127484	14127683
chr1	14137226	14137725
chr1	14463975	14463987
chr1	14464173	14464477
chr1	14464588	14464660
chr1	14516926	14517010
chr1	14993219	14993449
chr1	15058407	15059679
chr1	15059840	15060614
chr1	15068606	15068919
chr1	15069029	15069477
chr1	15069614	15069857
chr1	15202904	15203079
chr1	15203583	15203690
chr1	15234248	15234447
chr1	15237371	15237658
chr1	15238262	15239144
chr1	15239703	15239955
chr1	15240236	15240654
chr1	15250163	15250448
chr1	15251136	15251862
chr1	15266478	15266677
chr1	15271704	15272416
chr1	15272480	15272510
chr1	15272726	15272974
chr1	15273277	15273581
chr1	15273734	15273808
chr1	15274481	15274601
chr1	15296598	15296743
chr1	15323646	15323736

chr1	15323967	15324036
chr1	15359998	15360004
chr1	15479204	15481396
chr1	15481661	15482301
chr1	15482461	15482495
chr1	15484925	15485781
chr1	15494902	15495395
chr1	15498255	15498301
chr1	15500296	15501421
chr1	15498598	15500003
chr1	15503772	15503991
chr1	15504584	15504973
chr1	15505168	15505491
chr1	15505653	15506034
chr1	15516981	15517025
chr1	15521304	15521641
chr1	15522953	15523175
chr1	15526444	15526454
chr1	15526579	15527653
chr1	15573496	15574186
chr1	15588165	15588343
chr1	15664012	15664307
chr1	15667122	15667193
chr1	15668504	15668896
chr1	15671836	15672161
chr1	15677212	15677740
chr1	15678031	15678371
chr1	15678609	15679006
chr1	15679186	15679410
chr1	15681405	15681439
chr1	15682539	15682718
chr1	15683012	15683107
chr1	15683318	15683798
chr1	15691099	15691139
chr1	15722542	15722750
chr1	15727039	15727135
chr1	15727424	15727995
chr1	15735788	15738138
chr1	15742087	15744571
chr1	15745047	15745731
chr1	15747482	15747500
chr1	15756892	15757510
chr1	15759653	15759878
chr1	15761184	15761283
chr1	15786378	15786417
chr1	15792853	15792862

chr1	15792998	15793104
chr1	15850262	15850862
chr1	15851078	15851378
chr1	15851696	15852673
chr1	15853348	15853921
chr1	15859746	15859947
chr1	15884283	15884465
chr1	15885925	15886427
chr1	15895682	15895858
chr1	15910774	15911934
chr1	15921935	15922005
chr1	15928023	15928310
chr1	15929170	15929435
chr1	15929559	15930042
chr1	15930293	15930591
chr1	15942711	15943039
chr1	15943138	15944839
chr1	15945214	15945558
chr1	15946629	15947128
chr1	15957429	15957677
chr1	15981722	15982230
chr1	16005045	16005536
chr1	16009521	16009854
chr1	16006140	16009048
chr1	16010226	16010884
chr1	16010995	16011704
chr1	16019428	16019539
chr1	16028974	16028983
chr1	16050681	16050880
chr1	16056051	16056398
chr1	16056598	16056954
chr1	16062241	16062529
chr1	16068176	16068375
chr1	16071680	16071872
chr1	16074953	16075164
chr1	16075278	16075546
chr1	16075683	16076639
chr1	16077001	16077136
chr1	16084385	16084862
chr1	16085106	16087642
chr1	16087881	16087925
chr1	16088214	16088278
chr1	16088487	16089188
chr1	16090205	16091708
chr1	16091773	16093223
chr1	16094321	16094484

chr1	16094576	16094603
chr1	16103115	16103354
chr1	16104471	16104670
chr1	16106066	16106265
chr1	16106762	16107257
chr1	16107365	16107783
chr1	16109396	16109595
chr1	16110731	16110789
chr1	16111130	16112449
chr1	16112686	16115892
chr1	16116670	16117312
chr1	16117473	16117672
chr1	16118816	16118872
chr1	16119242	16119278
chr1	16119592	16120186
chr1	16120404	16120629
chr1	16120732	16120816
chr1	16122507	16122726
chr1	16122930	16124018
chr1	16124251	16124493
chr1	16121426	16122287
chr1	16124662	16124856
chr1	16125254	16127089
chr1	16127220	16128175
chr1	16129491	16130020
chr1	16132926	16133125
chr1	16139786	16140004
chr1	16142315	16142379
chr1	16142916	16143267
chr1	16143394	16143549
chr1	16145304	16145535
chr1	16146760	16147642
chr1	16147871	16148160
chr1	16148986	16149139
chr1	16149971	16150318
chr1	16155965	16156614
chr1	16157074	16157769
chr1	16158570	16160170
chr1	16160726	16162323
chr1	16162523	16163103
chr1	16164000	16164197
chr1	16166242	16166429
chr1	16171711	16172210
chr1	16173164	16175528
chr1	16175656	16175937
chr1	16176076	16176879

chr1	16179716	16179947
chr1	16185702	16185919
chr1	16186791	16187027
chr1	16207785	16207798
chr1	16209083	16209428
chr1	16209837	16210085
chr1	16212384	16212532
chr1	16212734	16213216
chr1	16224339	16224605
chr1	16225098	16225308
chr1	16229429	16229709
chr1	16242890	16243323
chr1	16244305	16244497
chr1	16245809	16246245
chr1	16249730	16250128
chr1	16274343	16277098
chr1	16278122	16280429
chr1	16277290	16278037
chr1	16280825	16281006
chr1	16283217	16283461
chr1	16283905	16283972
chr1	16290931	16291162
chr1	16301626	16302606
chr1	16302741	16302847
chr1	16319422	16319621
chr1	16333329	16333456
chr1	16333903	16334297
chr1	16335027	16335106
chr1	16338983	16339182
chr1	16339406	16340200
chr1	16375659	16376072
chr1	16399761	16400114
chr1	16400328	16400937
chr1	16401108	16402538
chr1	16402823	16403207
chr1	16405621	16405853
chr1	16407216	16407309
chr1	16409452	16409761
chr1	16410541	16410740
chr1	16412529	16412701
chr1	16414845	16416147
chr1	16437893	16437975
chr1	16438051	16438162
chr1	16446851	16447109
chr1	16447919	16448306
chr1	16448372	16448413

chr1	16459602	16459801
chr1	16463916	16464167
chr1	16465749	16466860
chr1	16467312	16467778
chr1	16467013	16467064
chr1	16467976	16469030
chr1	16469220	16469412
chr1	16469605	16469887
chr1	16470112	16472726
chr1	16473051	16474208
chr1	16474580	16474831
chr1	16476949	16477704
chr1	16478028	16478230
chr1	16483212	16484423
chr1	16484516	16485233
chr1	16485312	16485670
chr1	16478431	16483145
chr1	16485853	16486274
chr1	16486488	16486960
chr1	16487152	16487570
chr1	16487637	16487836
chr1	16489403	16489717
chr1	16489871	16490016
chr1	16490086	16490824
chr1	16491269	16491547
chr1	16491673	16492203
chr1	16492834	16493098
chr1	16493236	16494957
chr1	16495137	16495266
chr1	16496286	16496502
chr1	16497629	16497872
chr1	16498419	16498750
chr1	16499395	16500142
chr1	16498915	16499179
chr1	16500322	16501274
chr1	16501402	16501607
chr1	16501801	16503338
chr1	16504725	16505279
chr1	16505476	16505883
chr1	16506319	16506986
chr1	16507211	16509753
chr1	16509896	16511953
chr1	16512119	16512140
chr1	16512425	16512829
chr1	16513099	16513286
chr1	16513489	16513889

chr1	16515394	16515615
chr1	16514159	16515309
chr1	16515754	16515949
chr1	16532467	16532975
chr1	16533196	16534936
chr1	16538234	16539515
chr1	16541688	16541887
chr1	16543112	16544524
chr1	16561109	16561367
chr1	16562981	16563988
chr1	16564631	16564938
chr1	16566118	16566374
chr1	16575267	16575299
chr1	16627247	16627381
chr1	16647585	16648257
chr1	16648778	16649088
chr1	16651164	16651255
chr1	16670225	16670288
chr1	16675352	16675407
chr1	16678066	16679602
chr1	16689922	16690238
chr1	16691164	16691661
chr1	16693318	16693439
chr1	16693726	16694956
chr1	16697990	16698055
chr1	16698315	16698470
chr1	16698599	16698658
chr1	16700162	16700370
chr1	16701291	16701421
chr1	16701884	16702172
chr1	16723446	16723549
chr1	16764476	16764581
chr1	16766545	16768178
chr1	16768585	16768792
chr1	16819109	16819175
chr1	16819273	16819406
chr1	16825072	16826841
chr1	16830869	16830930
chr1	16832289	16832594
chr1	16834524	16834660
chr1	16836424	16836708
chr1	16837308	16837607
chr1	16839036	16841722
chr1	16841794	16842123
chr1	16857479	16857482
chr1	16857900	16858801

chr1	16859709	16859715
chr1	16859320	16859614
chr1	16860164	16862656
chr1	16874400	16874587
chr1	16886101	16886441
chr1	16887064	16887210
chr1	16887474	16887912
chr1	16891150	16891393
chr1	16891809	16892241
chr1	16893142	16893691
chr1	16906222	16906493
chr1	16912146	16912335
chr1	16912789	16913249
chr1	16917435	16917756
chr1	16918211	16918521
chr1	16918634	16918693
chr1	16918827	16919128
chr1	16919486	16919572
chr1	16920409	16920565
chr1	16921095	16921277
chr1	16922072	16922183
chr1	16924094	16924136
chr1	16928784	16929150
chr1	16935601	16935812
chr1	16936325	16936574
chr1	16937239	16937438
chr1	16937725	16937962
chr1	16938428	16938787
chr1	16938990	16940639
chr1	16940783	16940973
chr1	16941217	16941451
chr1	16941938	16942248
chr1	16943060	16943362
chr1	16944553	16944783
chr1	16947157	16947870
chr1	16948831	16949049
chr1	16949174	16950408
chr1	16950688	16951078
chr1	16951188	16951530
chr1	16953048	16953352
chr1	16959272	16959471
chr1	16961999	16962047
chr1	16962544	16963336
chr1	16963933	16964259
chr1	16964709	16964913
chr1	16967070	16967700

chr1	16968126	16968331
chr1	16968394	16968787
chr1	16969303	16969742
chr1	16969928	16972428
chr1	16973698	16974139
chr1	16974315	16974579
chr1	16978223	16978297
chr1	16981236	16981327
chr1	16984378	16984670
chr1	16985451	16985665
chr1	16991755	16994021
chr1	16994408	16994718
chr1	16998037	16998236
chr1	16998839	16999039
chr1	17000114	17000399
chr1	17016640	17016864
chr1	17019321	17020849
chr1	17021001	17021241
chr1	17027073	17027626
chr1	17032021	17032396
chr1	17033841	17034160
chr1	17034347	17035199
chr1	17035801	17036155
chr1	17036490	17036689
chr1	17042987	17043253
chr1	17052689	17052781
chr1	17054589	17054880
chr1	17056166	17056495
chr1	17062057	17062333
chr1	17065282	17065481
chr1	17066043	17067048
chr1	17067237	17068437
chr1	17070347	17070690
chr1	17071405	17071587
chr1	17075372	17075571
chr1	17076868	17077067
chr1	17079678	17079935
chr1	17080746	17080992
chr1	17091516	17091680
chr1	17106865	17107064
chr1	17107683	17108508
chr1	17185738	17185937
chr1	17186058	17186625
chr1	17198612	17200985
chr1	17201243	17201908
chr1	17202058	17202315

chr1	17202506	17202823
chr1	17203002	17203056
chr1	17204018	17204279
chr1	17215294	17216357
chr1	17216557	17216796
chr1	17221671	17223705
chr1	17225463	17225885
chr1	17226009	17226066
chr1	17229626	17229849
chr1	17229955	17229996
chr1	17230393	17231868
chr1	17232086	17232303
chr1	17236621	17236786
chr1	17239515	17239517
chr1	17239584	17240364
chr1	17240488	17241333
chr1	17248080	17248324
chr1	17251614	17252207
chr1	17252328	17252827
chr1	17255417	17256030
chr1	17256373	17256946
chr1	17258350	17258681
chr1	17259697	17260015
chr1	17266847	17267118
chr1	17268682	17268782
chr1	17274418	17275628
chr1	17275736	17275980
chr1	17276388	17276584
chr1	17278120	17278662
chr1	17278729	17279092
chr1	17287078	17287880
chr1	17287985	17288282
chr1	17288448	17288651
chr1	17298330	17298529
chr1	17306377	17306742
chr1	17306868	17307855
chr1	17308521	17308609
chr1	17308374	17308391
chr1	17309237	17309265
chr1	17310542	17311019
chr1	17311974	17312173
chr1	17329787	17330018
chr1	17330146	17330466
chr1	17337538	17338365
chr1	17342555	17342719
chr1	17367105	17368105

chr1	17372618	17372638
chr1	17375497	17375885
chr1	17380198	17380570
chr1	17380763	17381312
chr1	17381535	17381596
chr1	17387224	17387289
chr1	17390037	17390239
chr1	17393054	17393093
chr1	17424508	17424939
chr1	17425332	17425754
chr1	17426229	17426405
chr1	17445625	17445891
chr1	17452225	17452275
chr1	17452528	17452717
chr1	17454489	17454988
chr1	17455112	17455314
chr1	17456172	17456385
chr1	17465133	17465272
chr1	17494965	17495186
chr1	17516206	17516493
chr1	17516731	17517238
chr1	17517374	17517630
chr1	17517897	17518242
chr1	17518438	17518757
chr1	17518914	17518942
chr1	17519190	17519525
chr1	17520235	17520544
chr1	17533180	17533312
chr1	17533504	17533639
chr1	17534350	17535332
chr1	17535787	17536412
chr1	17538764	17539332
chr1	17539836	17540084
chr1	17541554	17541810
chr1	17542769	17543005
chr1	17543138	17543278
chr1	17551821	17551875
chr1	17552775	17553550
chr1	17558659	17559026
chr1	17559398	17559569
chr1	17559844	17560700
chr1	17562479	17562850
chr1	17562985	17563032
chr1	17563345	17563544
chr1	17563727	17564144
chr1	17565406	17565605

chr1	17571472	17571827
chr1	17577754	17578157
chr1	17583898	17584305
chr1	17584593	17585004
chr1	17585165	17585611
chr1	17585704	17586599
chr1	17591297	17591446
chr1	17591545	17591592
chr1	17591843	17592328
chr1	17602991	17603467
chr1	17603670	17603960
chr1	17608139	17608554
chr1	17614364	17614458
chr1	17615784	17616430
chr1	17616615	17616890
chr1	17617957	17618335
chr1	17630478	17630637
chr1	17636530	17636755
chr1	17675617	17675900
chr1	17691700	17691811
chr1	17692038	17692417
chr1	17695738	17696039
chr1	17729907	17730290
chr1	17741960	17742004
chr1	17742090	17742259
chr1	17745661	17745867
chr1	17753189	17753374
chr1	17757301	17757404
chr1	17763418	17763907
chr1	17764188	17765454
chr1	17765618	17765912
chr1	17766043	17766124
chr1	17766333	17768551
chr1	17769820	17770006
chr1	17770245	17770475
chr1	17782407	17782609
chr1	17785509	17785516
chr1	17786459	17787647
chr1	17802148	17802736
chr1	17803164	17803223
chr1	17803326	17803420
chr1	17803791	17803929
chr1	17804338	17804380
chr1	17806471	17806592
chr1	17813756	17814124
chr1	17814192	17815216

chr1	17815465	17815495
chr1	17818148	17818894
chr1	17847183	17847507
chr1	17865853	17866099
chr1	17866350	17866802
chr1	17878218	17878390
chr1	17878675	17879037
chr1	17879210	17879273
chr1	17880010	17880247
chr1	17880332	17880431
chr1	17881290	17881387
chr1	17887030	17887139
chr1	17907762	17907974
chr1	17909294	17909528
chr1	17927313	17927558
chr1	17943065	17943264
chr1	17944147	17944299
chr1	17944868	17945341
chr1	17951128	17951434
chr1	17959988	17960454
chr1	18192520	18193147
chr1	18193270	18193429
chr1	18199663	18199846
chr1	18199924	18199994
chr1	18200726	18201771
chr1	18202708	18202836
chr1	18994183	18994275
chr1	18994851	18995187
chr1	19212109	19212438
chr1	19212578	19212869
chr1	19213108	19213451
chr1	19213668	19214028
chr1	19214144	19214249
chr1	19228547	19229314
chr1	19249121	19249492
chr1	19249746	19250701
chr1	19252342	19252993
chr1	19253341	19254163
chr1	19265794	19265993
chr1	19266551	19266985
chr1	19267958	19268435
chr1	19275720	19275972
chr1	19276423	19276633
chr1	19276747	19277509
chr1	19281986	19282267
chr1	19282456	19282655

chr1	19282858	19283102
chr1	19283482	19284063
chr1	19289181	19289350
chr1	19291581	19292035
chr1	19292586	19292861
chr1	19298706	19298881
chr1	19324511	19324947
chr1	19325163	19325274
chr1	19337122	19337692
chr1	19338011	19338088
chr1	19356018	19356440
chr1	19356548	19357145
chr1	19359545	19359825
chr1	19359946	19360504
chr1	19360847	19361566
chr1	19379764	19379903
chr1	19392600	19393076
chr1	19393253	19393503
chr1	19399530	19399852
chr1	19400077	19400291
chr1	19400542	19401016
chr1	19401262	19401652
chr1	19401789	19402209
chr1	19407723	19408018
chr1	19535954	19536855
chr1	19536986	19537279
chr1	19543733	19543912
chr1	19577428	19578522
chr1	19578628	19579079
chr1	19580534	19580796
chr1	19615725	19615957
chr1	19637533	19638018
chr1	19638186	19639501
chr1	19641759	19641881
chr1	19662895	19663094
chr1	19677301	19677500
chr1	19686908	19687107
chr1	19687598	19687703
chr1	19687920	19688217
chr1	19688282	19688292
chr1	19688712	19688867
chr1	19762664	19762939
chr1	19766847	19766852
chr1	19767295	19767453
chr1	19767756	19768286
chr1	19795875	19795880

chr1	19810686	19812044
chr1	19812227	19812796
chr1	19916713	19917020
chr1	19922750	19924014
chr1	19925488	19925687
chr1	19938663	19938665
chr1	19938768	19939021
chr1	19962235	19962428
chr1	19966411	19966903
chr1	19969515	19970631
chr1	19970896	19972162
chr1	19972486	19972832
chr1	19973487	19973573
chr1	19973372	19973382
chr1	19974906	19974990
chr1	19975054	19975209
chr1	19978323	19978848
chr1	19978955	19979307
chr1	19985738	19986145
chr1	20085233	20085609
chr1	20125499	20127185
chr1	20127459	20127478
chr1	20190284	20190419
chr1	20190629	20190663
chr1	20208525	20208703
chr1	20208868	20209695
chr1	20538290	20538588
chr1	20810529	20810889
chr1	20811242	20811592
chr1	20812106	20813292
chr1	20820551	20821430
chr1	20821829	20821965
chr1	20822305	20822768
chr1	20823179	20823380
chr1	20823559	20823766
chr1	20833869	20834968
chr1	20879737	20880126
chr1	20928050	20928309
chr1	20929194	20929269
chr1	20930376	20930507
chr1	20930615	20931235
chr1	20938013	20938016
chr1	20938640	20938714
chr1	20939990	20940182
chr1	20939192	20939643
chr1	20940619	20940829

chr1	20959903	20960437
chr1	20960558	20960728
chr1	20960947	20961351
chr1	20981482	20981649
chr1	20987176	20987876
chr1	20988162	20988916
chr1	21022692	21024358
chr1	21024676	21025837
chr1	21032772	21032892
chr1	21033021	21033220
chr1	21044008	21044532
chr1	21044995	21045229
chr1	21051800	21052221
chr1	21052645	21053051
chr1	21054504	21054703
chr1	21055768	21056318
chr1	21056503	21057082
chr1	21057700	21059651
chr1	21059794	21060455
chr1	21060747	21060951
chr1	21061717	21062198
chr1	21062408	21062678
chr1	21065494	21065741
chr1	21066194	21066594
chr1	21112579	21114794
chr1	21115684	21115728
chr1	21210256	21210412
chr1	21210582	21210664
chr1	21223217	21223372
chr1	21223517	21223676
chr1	21277433	21277515
chr1	21348717	21348829
chr1	21349002	21349420
chr1	21399223	21399250
chr1	21403649	21403829
chr1	21406274	21406581
chr1	21417521	21417995
chr1	21451358	21451430
chr1	21454746	21454944
chr1	21457483	21457815
chr1	21475855	21475997
chr1	21476788	21476833
chr1	21477238	21477561
chr1	21478968	21478971
chr1	21479099	21479287
chr1	21480001	21480238

chr1	21501125	21501388
chr1	21502006	21502875
chr1	21503157	21504268
chr1	21517460	21517875
chr1	21521897	21522201
chr1	21527535	21527860
chr1	21528087	21528099
chr1	21530584	21530616
chr1	21530851	21530934
chr1	21558753	21558898
chr1	21583809	21584349
chr1	21584655	21584909
chr1	21585863	21586168
chr1	21587834	21589175
chr1	21590563	21590782
chr1	21616125	21617480
chr1	21617556	21617624
chr1	21629560	21629811
chr1	21645561	21645760
chr1	21649591	21649995
chr1	21650295	21652157
chr1	21655636	21655925
chr1	21656209	21656444
chr1	21660565	21661657
chr1	21660394	21660423
chr1	21661916	21662963
chr1	21671115	21672683
chr1	21728255	21728325
chr1	21728547	21728642
chr1	21763394	21763612
chr1	21766161	21766566
chr1	21766673	21767510
chr1	21835305	21835377
chr1	21835442	21835533
chr1	21927308	21927611
chr1	21933434	21933939
chr1	21939788	21939987
chr1	21941299	21941646
chr1	21941802	21942023
chr1	21943039	21943339
chr1	21948724	21948965
chr1	21949307	21949854
chr1	21953842	21954295
chr1	21961034	21961233
chr1	21963579	21963736
chr1	21965057	21965150

chr1	21965385	21965836
chr1	21967937	21968147
chr1	21977989	21978357
chr1	21982609	21982772
chr1	22030323	22030452
chr1	22056943	22057224
chr1	22087288	22087518
chr1	22091097	22091433
chr1	22091584	22091980
chr1	22093580	22093642
chr1	22107971	22108305
chr1	22108793	22110480
chr1	22112357	22112463
chr1	22114432	22115119
chr1	22183014	22183213
chr1	22188400	22188622
chr1	22188760	22189063
chr1	22194362	22194441
chr1	22196143	22196426
chr1	22196980	22197218
chr1	22197734	22198286
chr1	22254965	22255144
chr1	22258152	22258486
chr1	22258790	22259337
chr1	22259408	22260216
chr1	22260421	22261367
chr1	22261599	22261694
chr1	22261998	22263684
chr1	22263924	22264658
chr1	22266110	22266364
chr1	22269438	22269692
chr1	22281936	22283471
chr1	22283829	22284505
chr1	22286652	22286693
chr1	22286792	22286851
chr1	22287044	22287173
chr1	22292384	22292873
chr1	22295802	22296203
chr1	22298678	22299069
chr1	22300914	22300942
chr1	22301101	22301308
chr1	22301537	22301683
chr1	22303747	22304020
chr1	22326036	22326177
chr1	22326454	22326665
chr1	22335596	22335835

chr1	22345447	22345604
chr1	22349241	22349440
chr1	22349980	22350173
chr1	22351064	22352904
chr1	22353124	22353413
chr1	22365490	22365614
chr1	22366183	22366348
chr1	22366520	22366719
chr1	22368309	22368385
chr1	22369465	22370111
chr1	22371309	22371616
chr1	22373170	22373572
chr1	22374194	22374388
chr1	22374534	22374593
chr1	22374737	22374859
chr1	22376444	22376653
chr1	22376851	22376913
chr1	22377113	22377172
chr1	22378488	22378795
chr1	22378861	22380355
chr1	22380924	22381190
chr1	22384402	22384551
chr1	22396320	22396584
chr1	22396673	22397049
chr1	22418618	22418943
chr1	22419125	22419177
chr1	22429795	22429935
chr1	22462721	22462888
chr1	22463028	22463380
chr1	22463597	22464721
chr1	22465097	22465496
chr1	22468931	22469266
chr1	22469722	22470299
chr1	22475298	22475497
chr1	22492752	22492997
chr1	22556561	22556761
chr1	22563685	22563961
chr1	22565302	22565326
chr1	22565520	22565633
chr1	22580151	22580222
chr1	22601591	22601829
chr1	22602116	22602370
chr1	22739230	22739429
chr1	22739604	22739874
chr1	22755600	22755847
chr1	22778031	22778334

chr1	22778479	22779238
chr1	22866533	22866817
chr1	23037753	23038310
chr1	23065944	23067155
chr1	23076696	23077280
chr1	23077404	23077667
chr1	23078081	23078227
chr1	23117064	23117144
chr1	23117217	23117485
chr1	23134441	23134552
chr1	23143806	23144078
chr1	23152122	23152134
chr1	23152233	23152322
chr1	23152550	23152745
chr1	23153004	23153820
chr1	23154226	23154498
chr1	23154797	23155146
chr1	23155301	23155529
chr1	23155652	23156855
chr1	23157015	23157276
chr1	23157430	23159270
chr1	23161642	23161750
chr1	23187731	23187834
chr1	23188169	23188277
chr1	23188397	23188915
chr1	23279704	23279967
chr1	23302835	23303026
chr1	23303201	23303543
chr1	23304596	23304836
chr1	23306216	23306298
chr1	23337026	23337639
chr1	23345582	23345749
chr1	23345887	23346690
chr1	23388231	23388386
chr1	23425525	23425602
chr1	23426753	23427134
chr1	23428543	23428548
chr1	23429801	23430196
chr1	23428996	23429244
chr1	23439021	23439034
chr1	23441936	23442144
chr1	23449394	23449696
chr1	23453231	23453716
chr1	23489515	23489828
chr1	23493806	23495367
chr1	23495483	23495806

chr1	23496371	23496585
chr1	23503973	23504407
chr1	23505225	23505468
chr1	23510956	23511203
chr1	23511639	23511969
chr1	23513997	23514397
chr1	23515045	23516329
chr1	23516583	23516722
chr1	23516841	23516950
chr1	23519009	23519298
chr1	23521234	23521650
chr1	23522467	23522860
chr1	23523131	23523516
chr1	23523699	23523893
chr1	23524842	23525229
chr1	23528762	23528936
chr1	23530643	23530958
chr1	23536530	23536599
chr1	23536720	23536850
chr1	23542932	23542979
chr1	23543265	23544329
chr1	23551543	23551619
chr1	23562628	23562864
chr1	23585246	23585284
chr1	23589046	23589389
chr1	23599434	23599621
chr1	23601433	23601629
chr1	23604103	23604107
chr1	23655115	23655552
chr1	23655927	23655947
chr1	23656085	23656126
chr1	23669697	23670765
chr1	23670877	23671490
chr1	23674891	23675090
chr1	23693129	23693466
chr1	23694003	23694768
chr1	23694955	23696582
chr1	23697219	23697272
chr1	23697402	23697860
chr1	23703393	23704423
chr1	23704571	23704825
chr1	23710175	23710515
chr1	23710955	23711899
chr1	23712149	23712280
chr1	23713044	23713189
chr1	23726199	23726515

chr1	23728814	23729188
chr1	23729378	23729573
chr1	23729751	23729942
chr1	23730073	23731217
chr1	23750792	23751920
chr1	23778083	23778171
chr1	23778360	23778438
chr1	23779075	23779251
chr1	23779319	23779320
chr1	23787543	23787742
chr1	23803657	23803875
chr1	23804193	23804427
chr1	23809838	23811288
chr1	23823834	23824200
chr1	23854236	23854435
chr1	23857432	23857684
chr1	23858060	23858297
chr1	23861103	23861229
chr1	23865359	23865635
chr1	23866121	23866316
chr1	23866630	23866746
chr1	23868003	23868024
chr1	23868393	23868461
chr1	23870354	23870935
chr1	23871149	23871278
chr1	23880792	23881426
chr1	23881535	23883047
chr1	23883118	23883795
chr1	23884045	23884335
chr1	23884443	23885979
chr1	23886254	23886345
chr1	23888435	23888588
chr1	23888654	23888832
chr1	23889700	23889888
chr1	23890966	23891456
chr1	23892790	23892900
chr1	23893357	23893894
chr1	23894167	23895194
chr1	23897180	23897364
chr1	23903817	23904440
chr1	23907518	23907541
chr1	23909692	23909941
chr1	23911158	23911467
chr1	23911979	23912590
chr1	23913217	23913574
chr1	23913916	23914115

chr1	23915764	23915864
chr1	23916942	23917360
chr1	23917608	23917800
chr1	23917968	23918771
chr1	23921336	23921772
chr1	23918937	23920772
chr1	23927165	23927168
chr1	23930546	23930593
chr1	23945924	23946262
chr1	23957734	23957913
chr1	23961452	23961970
chr1	23962144	23962379
chr1	23962628	23962643
chr1	23963715	23964139
chr1	23967965	23968087
chr1	23988365	23988502
chr1	24017981	24018898
chr1	24026292	24026427
chr1	24027712	24027891
chr1	24029764	24029856
chr1	24055002	24055230
chr1	24069418	24070754
chr1	24070854	24071565
chr1	24085387	24085713
chr1	24090807	24090998
chr1	24098557	24098689
chr1	24101757	24101929
chr1	24104312	24104418
chr1	24104920	24105318
chr1	24106249	24106259
chr1	24106543	24106853
chr1	24116530	24117333
chr1	24117591	24119009
chr1	24124545	24125081
chr1	24125669	24126044
chr1	24126164	24127663
chr1	24151471	24152226
chr1	24160214	24160243
chr1	24160603	24161052
chr1	24191824	24191838
chr1	24193729	24193958
chr1	24194393	24194788
chr1	24195158	24195169
chr1	24224259	24224355
chr1	24231608	24231625
chr1	24243171	24243274

chr1	24243654	24243875
chr1	24257290	24257663
chr1	24257726	24257989
chr1	24277899	24278080
chr1	24278345	24278532
chr1	24279429	24279750
chr1	24281615	24281799
chr1	24281978	24282113
chr1	24283297	24284089
chr1	24285390	24286183
chr1	24286324	24286707
chr1	24298998	24299178
chr1	24306312	24307651
chr1	24307763	24308121
chr1	24310806	24311298
chr1	24312567	24312746
chr1	24315562	24315757
chr1	24327990	24328063
chr1	24345113	24345181
chr1	24345294	24345504
chr1	24345636	24345780
chr1	24349282	24349617
chr1	24372767	24372803
chr1	24374321	24374448
chr1	24378418	24378423
chr1	24379508	24379738
chr1	24394218	24394220
chr1	24394519	24394611
chr1	24435656	24435797
chr1	24441883	24442086
chr1	24444158	24444397
chr1	24444531	24444712
chr1	24446282	24447456
chr1	24468898	24469671
chr1	24469861	24470026
chr1	24472891	24473129
chr1	24475503	24475542
chr1	24475809	24475911
chr1	24476494	24477064
chr1	24478426	24478662
chr1	24477227	24478145
chr1	24481429	24481463
chr1	24513527	24513900
chr1	24514004	24514772
chr1	24516931	24517086
chr1	24518557	24518813

chr1	24521766	24522861
chr1	24528306	24530140
chr1	24530311	24530356
chr1	24532755	24533396
chr1	24542409	24542441
chr1	24542701	24543184
chr1	24543372	24543571
chr1	24560929	24561188
chr1	24562624	24562713
chr1	24563353	24563808
chr1	24609782	24609867
chr1	24610326	24610425
chr1	24610790	24611164
chr1	24611502	24611844
chr1	24611994	24612261
chr1	24612652	24613174
chr1	24616701	24617029
chr1	24617444	24617967
chr1	24620031	24621230
chr1	24621472	24621895
chr1	24626382	24626415
chr1	24626523	24626821
chr1	24627433	24627861
chr1	24643159	24643692
chr1	24643799	24644032
chr1	24644441	24645334
chr1	24645617	24646718
chr1	24647005	24647299
chr1	24647668	24647794
chr1	24647922	24649668
chr1	24649803	24650201
chr1	24656121	24656528
chr1	24656596	24657138
chr1	24657204	24657399
chr1	24661620	24661819
chr1	24683791	24684003
chr1	24689800	24690017
chr1	24691696	24692292
chr1	24695284	24695612
chr1	24696042	24696157
chr1	24698422	24698668
chr1	24700827	24702326
chr1	24703787	24703987
chr1	24706014	24706448
chr1	24706597	24706794
chr1	24708615	24709120

chr1	24710410	24711251
chr1	24711582	24711601
chr1	24718015	24718153
chr1	24718565	24718921
chr1	24719112	24719147
chr1	24720444	24720542
chr1	24736012	24736158
chr1	24736399	24736426
chr1	24738464	24738810
chr1	24739566	24740783
chr1	24741253	24742026
chr1	24742160	24742875
chr1	24743051	24743638
chr1	24743840	24743955
chr1	24744139	24744585
chr1	24745424	24746150
chr1	24828293	24829683
chr1	24829802	24830218
chr1	24842371	24842545
chr1	24869746	24869865
chr1	24882574	24882773
chr1	24913603	24913811
chr1	24917695	24917944
chr1	24925191	24925377
chr1	24968185	24969385
chr1	24969597	24970843
chr1	25001392	25001551
chr1	25004896	25005333
chr1	25005940	25006114
chr1	25009015	25009314
chr1	25009768	25010738
chr1	25011960	25012043
chr1	25012464	25012695
chr1	25014471	25014612
chr1	25028782	25029007
chr1	25030936	25031191
chr1	25031331	25031887
chr1	25032261	25032432
chr1	25034461	25034654
chr1	25043300	25043499
chr1	25045305	25046214
chr1	25046497	25047078
chr1	25048113	25048221
chr1	25048578	25048665
chr1	25049164	25049937
chr1	25050174	25050205

chr1	25051228	25051427
chr1	25053783	25054424
chr1	25055215	25055650
chr1	25056124	25056298
chr1	25058708	25058958
chr1	25069316	25069494
chr1	25070022	25070240
chr1	25070801	25070804
chr1	25071216	25071417
chr1	25071746	25072944
chr1	25073071	25073540
chr1	25083526	25083577
chr1	25088854	25088990
chr1	25095399	25095741
chr1	25096551	25096984
chr1	25101055	25101149
chr1	25132026	25132368
chr1	25174816	25175209
chr1	25256113	25256316
chr1	25275627	25275852
chr1	25292769	25292895
chr1	25293608	25293818
chr1	25299057	25299223
chr1	25307092	25307320
chr1	25307647	25308343
chr1	25333054	25333178
chr1	25333245	25333370
chr1	25333575	25334640
chr1	25342712	25342949
chr1	25343794	25344080
chr1	25344405	25345058
chr1	25346509	25346708
chr1	25348209	25349830
chr1	25350132	25350374
chr1	25347646	25348032
chr1	25351601	25351837
chr1	25352017	25352168
chr1	25352250	25352285
chr1	25357491	25358045
chr1	25358355	25358585
chr1	25358899	25359971
chr1	25448459	25448652
chr1	25465989	25466527
chr1	25497233	25497287
chr1	25520708	25521130
chr1	25521391	25521866

chr1	25527241	25527446
chr1	25548438	25548521
chr1	25559556	25559970
chr1	25558416	25559357
chr1	25565310	25565613
chr1	25566005	25566514
chr1	25566655	25567478
chr1	25567977	25568255
chr1	25572852	25573330
chr1	25573412	25573890
chr1	25574106	25574586
chr1	25586152	25586366
chr1	25593079	25594727
chr1	25612265	25612477
chr1	25614528	25615033
chr1	25656382	25656640
chr1	25663430	25663664
chr1	25663847	25665535
chr1	25670354	25670804
chr1	25677427	25677573
chr1	25678677	25679119
chr1	25697968	25698228
chr1	25756439	25756751
chr1	25757311	25758032
chr1	25761836	25761884
chr1	25830663	25830678
chr1	25870097	25870754
chr1	25871218	25871425
chr1	25882637	25883230
chr1	25883337	25883650
chr1	25883913	25884146
chr1	25887003	25887243
chr1	25887386	25887755
chr1	25888524	25888744
chr1	25890140	25890339
chr1	25892985	25893636
chr1	25893874	25894889
chr1	25895164	25896149
chr1	25899564	25900648
chr1	25942778	25942979
chr1	25943290	25943601
chr1	25944157	25944645
chr1	26035090	26035247
chr1	26126108	26126352
chr1	26126869	26127480
chr1	26128718	26128996

chr1	26145504	26147442
chr1	26148116	26148315
chr1	26164060	26164189
chr1	26183365	26183564
chr1	26184090	26184302
chr1	26185092	26185142
chr1	26185266	26185933
chr1	26186117	26186471
chr1	26186605	26186899
chr1	26188202	26188328
chr1	26197029	26197088
chr1	26197714	26198033
chr1	26198806	26198823
chr1	26199863	26199894
chr1	26200071	26200494
chr1	26200827	26202703
chr1	26205023	26205739
chr1	26213162	26213175
chr1	26214117	26214481
chr1	26216057	26216376
chr1	26216725	26217470
chr1	26218181	26218511
chr1	26218612	26219453
chr1	26219806	26220016
chr1	26220500	26220597
chr1	26221819	26222061
chr1	26222403	26222602
chr1	26231822	26231967
chr1	26232208	26233508
chr1	26238611	26239018
chr1	26248893	26249946
chr1	26280434	26280494
chr1	26323553	26323959
chr1	26324169	26324479
chr1	26324815	26325276
chr1	26341886	26342066
chr1	26345712	26345813
chr1	26361721	26362534
chr1	26362703	26363276
chr1	26372429	26372753
chr1	26437138	26437189
chr1	26437302	26438772
chr1	26439100	26439185
chr1	26450103	26450242
chr1	26452910	26453464
chr1	26453752	26453924

chr1	26487399	26487614
chr1	26487765	26488704
chr1	26490229	26491397
chr1	26495618	26495881
chr1	26496139	26497286
chr1	26503402	26504245
chr1	26504421	26504676
chr1	26528049	26528640
chr1	26552055	26552306
chr1	26559702	26559875
chr1	26560144	26561334
chr1	26561555	26561893
chr1	26601219	26602821
chr1	26603045	26603554
chr1	26603765	26603860
chr1	26604352	26604798
chr1	26605058	26607383
chr1	26610851	26611295
chr1	26611694	26612013
chr1	26624069	26625462
chr1	26626192	26626225
chr1	26625656	26626127
chr1	26632354	26633167
chr1	26633436	26633672
chr1	26645529	26645561
chr1	26654468	26654895
chr1	26655191	26655580
chr1	26656026	26656694
chr1	26657238	26657837
chr1	26657906	26658220
chr1	26658394	26658460
chr1	26659892	26660168
chr1	26662395	26663264
chr1	26663370	26664138
chr1	26665845	26666054
chr1	26666374	26666543
chr1	26666690	26666931
chr1	26667375	26667499
chr1	26667605	26667683
chr1	26668959	26669158
chr1	26674886	26675321
chr1	26675575	26675758
chr1	26675969	26676356
chr1	26677164	26679597
chr1	26679838	26680828
chr1	26681908	26683178

chr1	26683343	26683687
chr1	26683896	26684308
chr1	26685028	26685574
chr1	26685759	26687021
chr1	26693272	26693511
chr1	26698480	26698557
chr1	26698928	26699002
chr1	26701265	26701337
chr1	26701885	26702031
chr1	26704442	26704580
chr1	26704779	26705053
chr1	26712616	26712845
chr1	26713776	26713960
chr1	26723034	26723163
chr1	26731539	26731719
chr1	26731855	26731937
chr1	26757895	26758617
chr1	26758867	26759093
chr1	26762461	26762691
chr1	26772208	26772407
chr1	26796631	26796914
chr1	26798683	26799631
chr1	26799849	26799995
chr1	26807571	26807746
chr1	26813386	26813422
chr1	26814061	26814285
chr1	26821878	26822001
chr1	26822918	26823096
chr1	26826056	26828370
chr1	26850290	26850343
chr1	26850409	26850772
chr1	26852782	26852788
chr1	26855165	26855490
chr1	26855637	26856848
chr1	26856976	26857689
chr1	26857884	26858797
chr1	26860103	26860188
chr1	26864177	26864490
chr1	26866522	26866710
chr1	26866790	26867358
chr1	26867421	26868867
chr1	26869033	26870012
chr1	26870980	26871480
chr1	26885884	26885979
chr1	26929116	26929167
chr1	26934913	26935047

chr1	26946533	26946918
chr1	26947127	26948247
chr1	26960269	26960385
chr1	26960495	26960688
chr1	27003766	27004061
chr1	27014276	27014304
chr1	27014490	27014755
chr1	27018610	27020021
chr1	27020302	27020519
chr1	27021615	27024675
chr1	27024834	27024954
chr1	27031726	27031925
chr1	27049607	27050062
chr1	27050333	27050486
chr1	27079476	27079624
chr1	27080340	27080995
chr1	27099102	27099276
chr1	27099810	27099997
chr1	27099626	27099725
chr1	27102898	27103087
chr1	27113007	27115999
chr1	27128947	27129048
chr1	27132418	27132704
chr1	27136683	27137024
chr1	27138153	27138426
chr1	27138636	27139232
chr1	27139509	27139796
chr1	27151272	27151698
chr1	27152074	27153167
chr1	27153529	27154449
chr1	27154809	27154983
chr1	27164522	27164564
chr1	27170050	27170082
chr1	27170898	27170899
chr1	27171037	27171208
chr1	27171291	27171722
chr1	27174297	27175204
chr1	27175312	27175849
chr1	27176037	27177031
chr1	27177544	27177819
chr1	27178403	27178691
chr1	27179381	27179580
chr1	27182132	27182572
chr1	27179973	27181887
chr1	27182799	27185089
chr1	27185277	27186424

chr1	27187050	27187560
chr1	27187640	27191602
chr1	27191905	27192573
chr1	27193678	27194001
chr1	27194076	27194430
chr1	27194627	27195091
chr1	27195180	27195646
chr1	27195964	27196261
chr1	27198049	27198427
chr1	27198682	27199441
chr1	27201639	27201941
chr1	27203587	27203685
chr1	27204438	27204552
chr1	27204706	27204808
chr1	27214227	27214597
chr1	27216002	27217295
chr1	27218420	27218577
chr1	27220216	27221295
chr1	27226333	27227208
chr1	27227554	27227795
chr1	27230570	27231087
chr1	27234385	27234586
chr1	27247735	27248034
chr1	27248166	27248931
chr1	27256320	27256450
chr1	27260445	27260491
chr1	27263752	27263951
chr1	27265540	27265819
chr1	27266821	27267197
chr1	27267369	27267442
chr1	27268528	27268659
chr1	27273082	27273174
chr1	27285669	27287588
chr1	27287834	27288798
chr1	27289023	27289250
chr1	27289804	27290199
chr1	27293625	27293887
chr1	27296817	27297029
chr1	27302862	27303075
chr1	27306972	27307171
chr1	27317260	27317460
chr1	27319184	27319472
chr1	27319616	27319839
chr1	27320170	27322086
chr1	27322272	27323004
chr1	27323335	27327851

chr1	27327938	27328642
chr1	27328841	27330136
chr1	27330690	27331192
chr1	27331305	27331504
chr1	27331754	27332582
chr1	27333156	27333355
chr1	27333754	27333910
chr1	27334444	27335224
chr1	27335369	27337604
chr1	27337717	27339359
chr1	27339493	27340111
chr1	27340437	27340641
chr1	27343273	27343615
chr1	27343838	27343913
chr1	27344103	27344387
chr1	27344803	27344941
chr1	27355496	27355695
chr1	27356943	27357075
chr1	27357544	27358765
chr1	27358988	27359660
chr1	27359794	27360458
chr1	27370896	27370987
chr1	27371281	27371621
chr1	27372316	27372950
chr1	27388473	27388742
chr1	27402428	27402434
chr1	27416609	27416666
chr1	27417396	27417471
chr1	27417768	27417802
chr1	27419609	27419653
chr1	27424993	27426183
chr1	27431776	27431979
chr1	27437861	27437967
chr1	27438304	27438330
chr1	27439077	27439244
chr1	27439590	27439834
chr1	27442108	27442197
chr1	27442558	27442810
chr1	27443452	27443706
chr1	27445117	27445316
chr1	27446403	27446687
chr1	27448305	27448398
chr1	27451870	27451903
chr1	27453679	27453878
chr1	27460328	27460616
chr1	27461595	27461731

chr1	27462752	27462951
chr1	27466321	27466431
chr1	27471144	27471348
chr1	27471593	27471849
chr1	27472158	27472428
chr1	27472649	27472881
chr1	27472958	27473196
chr1	27474094	27474408
chr1	27478006	27478281
chr1	27479091	27479190
chr1	27479571	27481590
chr1	27481776	27482345
chr1	27488029	27488127
chr1	27488385	27488584
chr1	27518504	27518564
chr1	27518689	27519409
chr1	27524690	27525071
chr1	27560399	27560797
chr1	27561170	27562389
chr1	27568414	27568805
chr1	27573793	27574563
chr1	27611524	27611577
chr1	27622161	27622492
chr1	27625180	27625396
chr1	27625633	27625974
chr1	27626144	27627354
chr1	27648289	27648374
chr1	27648927	27649362
chr1	27666534	27666751
chr1	27666939	27668414
chr1	27668565	27670708
chr1	27670971	27671222
chr1	27672124	27672508
chr1	27673035	27673703
chr1	27691034	27691480
chr1	27691615	27693950
chr1	27694167	27694238
chr1	27709535	27709809
chr1	27710042	27710330
chr1	27712211	27712236
chr1	27717000	27717199
chr1	27718227	27718869
chr1	27719018	27719784
chr1	27721290	27721506
chr1	27721920	27722332
chr1	27744811	27744921

chr1	27748068	27748271
chr1	27763328	27763337
chr1	27781934	27782321
chr1	27782704	27782799
chr1	27790222	27790831
chr1	27793735	27793979
chr1	27803848	27804145
chr1	27809900	27810099
chr1	27815519	27815953
chr1	27816164	27816700
chr1	27816816	27817561
chr1	27823152	27823471
chr1	27828802	27828948
chr1	27829279	27829502
chr1	27829909	27830162
chr1	27830323	27830340
chr1	27830763	27830938
chr1	27831064	27831670
chr1	27842174	27842369
chr1	27842895	27843110
chr1	27843705	27844535
chr1	27844720	27844984
chr1	27849348	27850191
chr1	27850833	27851037
chr1	27851274	27851597
chr1	27852240	27853462
chr1	27854013	27855094
chr1	27855342	27855877
chr1	27856622	27856821
chr1	27857309	27857713
chr1	27864583	27864835
chr1	27865110	27865661
chr1	27866349	27867813
chr1	27868082	27868959
chr1	27869078	27870597
chr1	27882819	27883175
chr1	27883254	27883279
chr1	27883509	27883925
chr1	27884092	27884462
chr1	27884680	27885404
chr1	27885528	27885935
chr1	27895129	27895391
chr1	27895927	27896126
chr1	27901958	27902566
chr1	27915575	27915970
chr1	27916962	27917429

chr1	27921417	27921616
chr1	27925537	27925853
chr1	27928484	27930915
chr1	27935441	27935640
chr1	27937575	27937676
chr1	27952083	27952335
chr1	27952466	27952559
chr1	27960631	27961183
chr1	27962250	27962469
chr1	27968398	27968503
chr1	27975461	27975660
chr1	27976602	27977290
chr1	27983741	27983957
chr1	27985119	27987129
chr1	27989488	27990012
chr1	27990786	27991556
chr1	27994379	27994819
chr1	27997791	27998250
chr1	27998501	27998665
chr1	27998973	27999263
chr1	28051362	28052092
chr1	28052429	28053278
chr1	28053830	28053964
chr1	28099272	28099519
chr1	28098729	28098748
chr1	28099738	28100503
chr1	28101886	28102166
chr1	28107510	28107575
chr1	28109816	28110136
chr1	28112538	28112669
chr1	28156826	28157925
chr1	28187021	28187321
chr1	28189383	28189784
chr1	28190400	28190740
chr1	28190918	28191470
chr1	28194172	28194484
chr1	28198477	28198722
chr1	28199111	28200188
chr1	28200576	28200672
chr1	28201541	28202282
chr1	28202639	28202768
chr1	28203404	28203616
chr1	28203957	28203974
chr1	28204378	28204446
chr1	28204533	28204636
chr1	28217276	28217455

chr1	28230312	28230344
chr1	28240519	28241119
chr1	28241246	28241812
chr1	28245963	28246728
chr1	28260300	28260717
chr1	28261274	28261911
chr1	28270300	28270332
chr1	28277374	28277539
chr1	28285486	28286894
chr1	28287093	28287292
chr1	28295182	28295248
chr1	28295446	28295592
chr1	28306089	28306283
chr1	28364696	28364915
chr1	28389283	28389650
chr1	28390663	28390774
chr1	28392172	28392790
chr1	28394132	28394380
chr1	28414242	28415151
chr1	28415281	28415967
chr1	28449940	28450262
chr1	28450406	28450570
chr1	28455935	28456197
chr1	28461839	28462292
chr1	28462437	28462642
chr1	28479638	28480425
chr1	28496505	28496584
chr1	28496937	28496958
chr1	28497604	28497918
chr1	28498673	28498887
chr1	28499504	28499731
chr1	28501048	28501288
chr1	28517077	28517286
chr1	28519381	28520908
chr1	28521043	28521260
chr1	28521497	28521643
chr1	28521882	28521929
chr1	28522069	28522266
chr1	28522659	28522692
chr1	28523260	28524206
chr1	28558552	28558757
chr1	28558993	28559215
chr1	28559295	28560002
chr1	28561743	28562143
chr1	28562239	28563689
chr1	28565641	28565915

chr1	28568354	28568837
chr1	28570282	28570538
chr1	28572857	28572968
chr1	28574822	28574933
chr1	28573089	28574144
chr1	28575195	28575324
chr1	28575432	28575858
chr1	28575974	28576078
chr1	28577345	28577572
chr1	28577830	28578184
chr1	28585154	28585705
chr1	28586071	28587017
chr1	28587167	28587456
chr1	28588852	28589057
chr1	28589670	28589689
chr1	28592006	28592228
chr1	28602522	28602643
chr1	28610161	28610405
chr1	28632374	28632492
chr1	28640068	28640285
chr1	28653397	28653595
chr1	28653727	28653832
chr1	28656006	28656396
chr1	28655254	28655755
chr1	28668078	28668271
chr1	28670137	28670298
chr1	28672676	28673071
chr1	28675632	28675659
chr1	28678037	28679128
chr1	28679575	28681081
chr1	28684781	28685058
chr1	28686236	28686247
chr1	28686850	28687183
chr1	28692417	28692803
chr1	28693046	28693544
chr1	28694616	28695289
chr1	28695937	28697245
chr1	28699418	28700397
chr1	28700856	28701147
chr1	28704079	28704281
chr1	28708882	28708978
chr1	28718327	28718656
chr1	28718874	28718946
chr1	28727134	28727323
chr1	28736434	28736708
chr1	28743832	28744271

chr1	28744773	28745074
chr1	28763768	28764264
chr1	28764329	28764489
chr1	28770739	28770975
chr1	28776482	28776554
chr1	28776693	28776803
chr1	28796637	28797012
chr1	28797417	28797663
chr1	28797972	28798032
chr1	28798213	28798295
chr1	28826055	28826097
chr1	28827305	28827679
chr1	28832113	28832373
chr1	28832586	28833412
chr1	28833766	28834106
chr1	28834416	28834709
chr1	28836250	28836932
chr1	28837119	28837224
chr1	28835396	28836171
chr1	28837598	28837644
chr1	28837938	28838137
chr1	28838634	28838824
chr1	28839652	28839870
chr1	28840013	28840479
chr1	28840789	28841311
chr1	28841615	28841814
chr1	28841957	28842297
chr1	28842564	28842873
chr1	28843018	28843285
chr1	28843396	28843633
chr1	28844186	28844527
chr1	28844694	28846086
chr1	28847112	28847577
chr1	28852807	28853084
chr1	28853303	28853564
chr1	28853731	28854070
chr1	28854441	28854658
chr1	28868159	28868255
chr1	28878732	28880589
chr1	28895871	28896130
chr1	28897468	28897602
chr1	28899258	28899930
chr1	28902600	28902811
chr1	28901124	28901929
chr1	28903479	28903584
chr1	28906794	28907012

chr1	28907279	28909721
chr1	28916555	28916635
chr1	28919811	28920155
chr1	28918774	28919734
chr1	28920836	28921244
chr1	28921434	28922071
chr1	28924746	28924791
chr1	28924905	28925949
chr1	28926415	28926454
chr1	28928110	28928158
chr1	28928399	28928889
chr1	28929192	28929457
chr1	28929622	28929676
chr1	28952904	28953014
chr1	28953251	28953429
chr1	28967337	28967440
chr1	28968711	28968923
chr1	28969095	28969509
chr1	28969710	28970769
chr1	28971598	28971797
chr1	28974492	28975125
chr1	28975287	28975927
chr1	28994632	28995356
chr1	28995570	28996431
chr1	29001631	29001870
chr1	29002131	29002239
chr1	29002410	29002583
chr1	29048393	29048794
chr1	29062805	29064640
chr1	29064985	29065204
chr1	29066597	29066853
chr1	29072350	29072539
chr1	29073352	29073536
chr1	29077318	29077607
chr1	29089415	29089807
chr1	29106354	29106383
chr1	29111831	29112249
chr1	29155276	29155475
chr1	29171542	29171812
chr1	29203147	29203306
chr1	29208391	29208482
chr1	29208832	29209252
chr1	29211251	29211655
chr1	29211921	29212162
chr1	29212365	29212530
chr1	29213009	29214747

chr1	29217685	29217690
chr1	29219496	29219497
chr1	29240669	29241789
chr1	29241902	29242076
chr1	29431299	29431390
chr1	29448431	29451944
chr1	29459365	29459562
chr1	29472224	29472303
chr1	29472586	29472907
chr1	29473010	29473035
chr1	29473460	29473485
chr1	29503920	29504205
chr1	29504281	29504641
chr1	29507530	29508493
chr1	29508691	29508867
chr1	29511499	29511605
chr1	29511809	29512186
chr1	29512446	29512564
chr1	29513064	29513196
chr1	29517225	29517270
chr1	29517598	29518400
chr1	29518859	29519042
chr1	29519454	29519710
chr1	29522200	29522536
chr1	29527423	29527746
chr1	29528408	29528692
chr1	29528926	29529401
chr1	29545135	29545452
chr1	29557232	29557774
chr1	29557911	29558047
chr1	29562977	29564264
chr1	29564486	29565743
chr1	29566065	29566400
chr1	29585926	29586125
chr1	30179563	30180147
chr1	30180427	30180861
chr1	30181715	30182709
chr1	30181216	30181581
chr1	30182950	30183318
chr1	30184848	30185097
chr1	31124483	31124854
chr1	31128207	31128512
chr1	31190404	31191406
chr1	31191612	31192716
chr1	31214199	31214407
chr1	31216187	31216520

chr1	31218901	31219111
chr1	31220222	31220461
chr1	31220726	31221000
chr1	31221237	31221814
chr1	31224312	31224535
chr1	31229074	31229376
chr1	31229943	31230414
chr1	31255411	31255610
chr1	31259587	31259816
chr1	31269619	31269795
chr1	31270221	31271098
chr1	31275265	31275310
chr1	31296368	31296460
chr1	31297097	31297296
chr1	31324809	31324841
chr1	31325028	31325371
chr1	31350547	31350926
chr1	31360351	31360550
chr1	31374855	31375239
chr1	31381055	31381342
chr1	31382790	31383370
chr1	31385277	31385729
chr1	31386163	31386458
chr1	31406278	31406606
chr1	31406730	31407876
chr1	31410979	31411383
chr1	31480196	31480330
chr1	31480799	31480868
chr1	31483765	31483952
chr1	31486039	31486074
chr1	31498134	31498192
chr1	31498342	31498489
chr1	31505995	31506337
chr1	31506423	31506760
chr1	31512098	31512295
chr1	31513050	31513150
chr1	31520374	31520913
chr1	31521892	31522091
chr1	31522936	31523244
chr1	31525425	31525666
chr1	31531491	31531806
chr1	31538039	31539233
chr1	31543922	31543923
chr1	31562449	31562987
chr1	31568660	31568749
chr1	31574051	31574250

chr1	31575187	31575416
chr1	31575602	31575873
chr1	31579077	31579295
chr1	31579548	31579747
chr1	31584104	31584685
chr1	31592434	31592917
chr1	31623031	31623248
chr1	31625147	31625930
chr1	31627343	31627756
chr1	31627969	31628795
chr1	31631604	31631837
chr1	31634611	31635082
chr1	31635187	31635954
chr1	31637103	31637301
chr1	31637456	31637559
chr1	31639679	31640170
chr1	31643403	31643705
chr1	31643908	31644147
chr1	31654615	31655203
chr1	31712589	31712825
chr1	31769431	31769614
chr1	31769809	31770482
chr1	31770805	31770878
chr1	31776605	31776611
chr1	31779302	31779482
chr1	31800253	31800657
chr1	31807832	31807876
chr1	31845297	31845399
chr1	31845913	31846620
chr1	31846789	31847018
chr1	31847144	31847220
chr1	31848415	31848668
chr1	31863495	31863660
chr1	31866762	31866991
chr1	31867377	31867657
chr1	31867799	31868224
chr1	31869275	31869346
chr1	31869423	31869571
chr1	31870086	31870844
chr1	31871125	31871383
chr1	31871504	31871525
chr1	31873246	31873645
chr1	31885298	31885765
chr1	31886037	31888467
chr1	31888661	31889608
chr1	31890411	31890790

chr1	31891540	31891989
chr1	31892305	31892502
chr1	31894220	31895927
chr1	31896376	31896575
chr1	31898725	31898937
chr1	31899302	31899592
chr1	31904502	31905061
chr1	31907423	31908605
chr1	31909978	31910330
chr1	31910666	31910687
chr1	31911312	31911511
chr1	31944364	31944375
chr1	31944665	31944819
chr1	31955464	31956024
chr1	31956153	31956685
chr1	31989354	31989398
chr1	32013505	32013842
chr1	32014186	32014462
chr1	32014574	32015768
chr1	32028434	32028649
chr1	32028877	32029010
chr1	32029617	32029808
chr1	32038198	32038399
chr1	32038643	32040584
chr1	32040762	32041215
chr1	32041390	32041822
chr1	32042096	32043818
chr1	32044222	32044425
chr1	32046447	32046652
chr1	32047901	32048029
chr1	32053027	32053226
chr1	32053656	32053937
chr1	32054286	32054967
chr1	32059436	32059555
chr1	32083193	32083392
chr1	32083642	32083857
chr1	32100448	32100652
chr1	32103429	32103515
chr1	32103717	32103978
chr1	32104087	32104220
chr1	32104738	32104991
chr1	32105064	32105789
chr1	32106997	32107114
chr1	32109782	32110464
chr1	32110601	32111090
chr1	32115438	32115727

chr1	32130971	32132722
chr1	32133404	32134053
chr1	32134474	32136689
chr1	32141305	32142093
chr1	32152699	32152898
chr1	32154906	32155508
chr1	32155727	32156331
chr1	32165637	32168503
chr1	32168820	32170894
chr1	32171076	32171378
chr1	32173278	32173406
chr1	32173601	32173658
chr1	32173831	32174006
chr1	32177818	32177896
chr1	32178153	32178665
chr1	32179676	32179787
chr1	32180164	32181279
chr1	32181748	32181864
chr1	32184328	32184527
chr1	32226218	32226464
chr1	32226884	32227278
chr1	32228359	32228823
chr1	32229289	32229609
chr1	32253461	32254236
chr1	32254346	32254545
chr1	32254670	32255106
chr1	32263909	32264303
chr1	32275973	32276202
chr1	32276307	32276362
chr1	32293024	32293151
chr1	32321446	32321476
chr1	32369760	32370422
chr1	32370663	32370881
chr1	32376617	32376887
chr1	32388407	32388904
chr1	32397406	32397605
chr1	32398093	32398559
chr1	32400844	32401043
chr1	32402866	32403990
chr1	32404218	32404839
chr1	32405211	32405873
chr1	32408390	32408674
chr1	32409784	32410801
chr1	32418494	32418578
chr1	32420203	32420895
chr1	32421493	32422149

chr1	32423086	32423392
chr1	32429626	32429819
chr1	32442834	32443040
chr1	32443183	32443795
chr1	32478814	32480664
chr1	32527828	32527844
chr1	32528321	32528635
chr1	32528882	32528993
chr1	32530682	32530752
chr1	32531555	32532309
chr1	32533639	32533899
chr1	32535796	32536200
chr1	32537304	32537708
chr1	32538029	32538730
chr1	32538923	32539115
chr1	32572806	32574451
chr1	32574734	32575151
chr1	32581052	32582094
chr1	32597818	32598127
chr1	32598722	32599585
chr1	32599988	32600187
chr1	32600882	32601081
chr1	32601845	32602044
chr1	32628321	32628353
chr1	32644797	32646414
chr1	32647193	32647223
chr1	32647877	32648403
chr1	32650336	32650563
chr1	32665736	32666591
chr1	32666753	32667630
chr1	32670845	32671137
chr1	32671290	32671746
chr1	32684383	32684702
chr1	32685707	32685924
chr1	32686873	32687129
chr1	32687351	32688715
chr1	32689178	32689453
chr1	32691020	32691246
chr1	32698592	32698991
chr1	32699106	32699254
chr1	32701304	32702328
chr1	32706023	32707389
chr1	32707603	32708047
chr1	32708598	32708658
chr1	32711690	32711900
chr1	32715242	32715444

chr1	32730280	32730544
chr1	32731198	32731663
chr1	32739322	32739565
chr1	32754749	32755457
chr1	32756299	32757097
chr1	32757307	32758859
chr1	32763779	32764002
chr1	32774276	32774553
chr1	32800836	32801850
chr1	32808352	32808512
chr1	32816080	32816279
chr1	32816631	32817035
chr1	32818374	32818702
chr1	32817163	32817894
chr1	32827093	32827807
chr1	32859441	32860051
chr1	32860286	32860943
chr1	32913733	32914036
chr1	32930089	32930288
chr1	32930661	32931326
chr1	32957929	32958380
chr1	32963524	32963713
chr1	32965433	32966745
chr1	32972406	32972600
chr1	32986219	32986508
chr1	32986886	32986904
chr1	33004579	33004979
chr1	33005423	33005689
chr1	33055416	33055565
chr1	33077487	33077784
chr1	33077975	33078709
chr1	33102599	33103345
chr1	33115884	33116596
chr1	33116786	33117375
chr1	33119298	33119545
chr1	33144836	33144914
chr1	33163868	33163919
chr1	33164378	33164577
chr1	33166346	33166872
chr1	33167225	33167852
chr1	33168001	33169319
chr1	33176948	33176976
chr1	33177658	33177928
chr1	33181085	33181550
chr1	33181780	33182356
chr1	33182487	33182905

chr1	33190246	33190315
chr1	33190550	33190776
chr1	33190908	33191581
chr1	33195890	33196324
chr1	33206313	33208670
chr1	33208997	33209297
chr1	33210451	33210755
chr1	33211100	33211463
chr1	33213149	33213348
chr1	33219299	33221095
chr1	33221246	33221494
chr1	33221958	33222549
chr1	33222635	33223534
chr1	33223744	33225807
chr1	33225916	33227154
chr1	33228539	33229276
chr1	33229354	33229880
chr1	33230111	33230472
chr1	33230611	33230707
chr1	33227279	33227947
chr1	33234028	33234254
chr1	33234537	33234963
chr1	33240230	33240363
chr1	33241196	33241927
chr1	33246225	33246410
chr1	33246637	33246908
chr1	33247623	33247813
chr1	33254917	33255431
chr1	33257480	33257538
chr1	33264159	33264211
chr1	33279875	33280174
chr1	33282201	33284165
chr1	33338629	33338907
chr1	33358601	33358906
chr1	33359307	33359506
chr1	33366250	33367724
chr1	33367871	33367897
chr1	33371286	33371332
chr1	33399649	33399989
chr1	33400427	33400680
chr1	33405008	33405385
chr1	33405740	33405776
chr1	33407057	33407275
chr1	33407395	33407635
chr1	33419082	33419632
chr1	33419717	33420462

chr1	33428626	33429092
chr1	33429246	33430034
chr1	33430154	33430479
chr1	33430760	33430834
chr1	33438353	33439113
chr1	33444488	33444844
chr1	33446633	33446826
chr1	33447111	33447677
chr1	33449076	33449360
chr1	33453010	33453342
chr1	33453777	33454114
chr1	33454232	33454958
chr1	33455065	33455268
chr1	33456505	33457462
chr1	33457689	33458128
chr1	33458259	33458923
chr1	33459013	33459277
chr1	33461884	33461922
chr1	33470234	33470344
chr1	33470529	33471004
chr1	33471142	33471340
chr1	33472735	33472857
chr1	33473075	33473476
chr1	33474422	33474693
chr1	33501620	33502479
chr1	33502641	33502896
chr1	33508882	33509098
chr1	33513613	33514008
chr1	33514447	33514453
chr1	33515548	33515827
chr1	33515931	33516411
chr1	33516534	33516820
chr1	33522005	33522084
chr1	33546054	33546157
chr1	33546484	33547177
chr1	33560941	33560988
chr1	33564801	33564940
chr1	33565098	33565564
chr1	33565640	33566054
chr1	33580046	33580052
chr1	33587069	33587200
chr1	33592631	33593593
chr1	33593805	33594004
chr1	33617374	33617573
chr1	33646178	33646472
chr1	33646578	33647638

chr1	33647959	33648021
chr1	33686047	33687582
chr1	33684551	33685972
chr1	33688122	33688453
chr1	33694998	33695120
chr1	33699794	33699993
chr1	33721269	33721820
chr1	33722012	33722248
chr1	33722497	33722815
chr1	33723135	33723642
chr1	33724881	33725087
chr1	33771712	33773609
chr1	33774070	33774259
chr1	33774444	33774655
chr1	33791228	33791427
chr1	33793930	33794078
chr1	33801745	33801920
chr1	33804078	33804574
chr1	33804773	33804972
chr1	33806710	33806909
chr1	33807661	33807914
chr1	33808147	33808482
chr1	33808959	33809329
chr1	33812428	33815317
chr1	33815800	33816345
chr1	33832175	33832492
chr1	33853268	33853402
chr1	33853719	33853741
chr1	33895654	33895677
chr1	33895885	33896624
chr1	33897943	33898502
chr1	33899036	33899359
chr1	33899680	33899779
chr1	33905718	33906210
chr1	33921362	33921482
chr1	33937774	33937942
chr1	33938261	33938554
chr1	35208122	35208344
chr1	35220118	35222010
chr1	35225059	35225575
chr1	35228042	35229738
chr1	35231110	35231112
chr1	35243739	35243988
chr1	35244699	35244931
chr1	35245016	35249189
chr1	35249551	35249926

chr1	35250167	35250274
chr1	35324676	35325338
chr1	35325481	35325821
chr1	35331264	35332086
chr1	35449967	35451273
chr1	35496645	35497801
chr1	35513393	35513396
chr1	35542316	35542450
chr1	35544766	35545676
chr1	35551023	35551239
chr1	35551380	35551487
chr1	35654217	35654370
chr1	35657391	35658773
chr1	35659022	35659458
chr1	35660425	35660766
chr1	35685906	35686090
chr1	35734002	35734888
chr1	35782147	35782232
chr1	35811005	35811341
chr1	35913903	35914203
chr1	35914389	35914497
chr1	35914945	35915028
chr1	36021011	36021319
chr1	36021992	36023665
chr1	36023889	36024407
chr1	36038369	36038602
chr1	36038995	36039194
chr1	36039597	36039857
chr1	36090320	36090352
chr1	36106189	36107920
chr1	36108194	36108512
chr1	36165773	36165883
chr1	36173683	36173882
chr1	36183688	36183938
chr1	36184120	36185032
chr1	36234731	36235532
chr1	36239421	36239698
chr1	36272808	36274585
chr1	36300938	36301201
chr1	36348627	36349629
chr1	36395858	36397325
chr1	36438213	36438229
chr1	36474146	36474251
chr1	36474435	36474553
chr1	36500189	36500253
chr1	36500569	36500701

chr1	36534779	36534873
chr1	36545878	36546194
chr1	36546402	36546447
chr1	36546561	36546844
chr1	36547206	36547503
chr1	36547958	36547981
chr1	36549209	36549622
chr1	36549829	36550145
chr1	36553940	36555087
chr1	36556070	36556406
chr1	36556535	36556578
chr1	36555309	36555946
chr1	36564837	36565143
chr1	36565402	36565652
chr1	36571480	36571627
chr1	36590357	36590561
chr1	36590759	36591661
chr1	36599631	36599982
chr1	36600161	36600180
chr1	36600297	36600496
chr1	36600756	36600974
chr1	36601743	36602260
chr1	36602408	36602726
chr1	36605712	36605911
chr1	36613840	36614031
chr1	36614339	36615107
chr1	36615651	36617030
chr1	36617227	36617723
chr1	36620059	36620237
chr1	36620355	36620400
chr1	36620922	36624026
chr1	36624728	36625033
chr1	36625367	36627950
chr1	36628091	36628227
chr1	36628469	36628881
chr1	36630161	36630338
chr1	36630743	36630955
chr1	36631259	36631616
chr1	36631864	36632105
chr1	36632452	36633220
chr1	36633861	36634081
chr1	36634944	36635195
chr1	36634568	36634874
chr1	36635590	36637790
chr1	36638367	36638811
chr1	36638933	36639201

chr1	36639646	36639939
chr1	36640444	36640704
chr1	36642623	36642764
chr1	36647750	36648115
chr1	36649970	36650352
chr1	36651426	36651605
chr1	36653096	36654483
chr1	36666552	36666751
chr1	36682710	36682811
chr1	36683253	36683606
chr1	36683903	36684062
chr1	36688669	36688775
chr1	36689059	36689715
chr1	36690007	36690817
chr1	36703157	36703328
chr1	36709374	36709718
chr1	36710007	36710671
chr1	36713892	36714677
chr1	36716400	36716774
chr1	36722965	36723324
chr1	36732254	36732371
chr1	36742076	36742185
chr1	36742896	36743190
chr1	36751009	36751365
chr1	36764450	36764965
chr1	36765297	36765561
chr1	36765932	36766499
chr1	36768677	36768990
chr1	36770895	36771518
chr1	36771808	36772597
chr1	36772712	36773373
chr1	36784149	36784338
chr1	36788795	36789271
chr1	36808635	36808858
chr1	36815344	36815770
chr1	36816075	36816593
chr1	36816917	36817169
chr1	36817576	36817907
chr1	36825653	36825913
chr1	36834498	36834627
chr1	36838923	36840335
chr1	36840478	36840993
chr1	36847322	36847396
chr1	36848823	36848858
chr1	36850026	36850035
chr1	36850496	36850966

chr1	36851137	36852241
chr1	36852402	36852823
chr1	36852972	36853290
chr1	36861366	36861722
chr1	36862304	36862363
chr1	36862718	36863515
chr1	36863679	36863851
chr1	36882817	36882917
chr1	36891235	36891500
chr1	36891784	36892051
chr1	36902326	36902607
chr1	36902857	36902888
chr1	36911930	36911945
chr1	36914167	36914323
chr1	36915657	36915991
chr1	36916156	36916874
chr1	36917492	36917522
chr1	36929163	36930561
chr1	36931519	36931797
chr1	36948338	36948537
chr1	36952140	36952719
chr1	36952841	36953230
chr1	36962490	36963228
chr1	36977335	36977522
chr1	36977892	36978325
chr1	36978474	36978874
chr1	37914608	37914968
chr1	37915476	37916569
chr1	37917066	37918507
chr1	37918810	37918853
chr1	37919506	37919936
chr1	37920015	37920707
chr1	37920900	37921270
chr1	37921831	37922287
chr1	37923227	37923419
chr1	37937021	37937750
chr1	37938934	37948633
chr1	37949140	37949377
chr1	37953543	37953753
chr1	37949790	37953325
chr1	37959927	37960082
chr1	37964823	37964950
chr1	37969000	37969056
chr1	37979398	37980921
chr1	37999432	37999641
chr1	37999937	38000488

chr1	38003140	38003443
chr1	38016200	38016274
chr1	38019382	38019906
chr1	38020027	38020135
chr1	38027617	38027816
chr1	38029020	38029238
chr1	38029482	38029578
chr1	38042436	38042589
chr1	38061074	38061535
chr1	38152635	38152825
chr1	38155563	38156657
chr1	38157294	38157937
chr1	38158137	38158725
chr1	38198587	38198804
chr1	38205072	38205369
chr1	38259562	38259944
chr1	38272988	38274631
chr1	38324589	38325335
chr1	38325462	38326190
chr1	38326981	38327365
chr1	38327830	38328114
chr1	38328247	38328505
chr1	38328574	38329024
chr1	38341668	38341885
chr1	38341984	38342883
chr1	38369818	38369955
chr1	38396870	38397446
chr1	38397836	38398674
chr1	38398785	38398840
chr1	38431196	38431297
chr1	38454570	38455301
chr1	38455366	38456143
chr1	38465464	38466245
chr1	38466459	38466702
chr1	38467857	38468064
chr1	38468205	38468305
chr1	38468517	38468958
chr1	38469840	38471464
chr1	38475358	38475457
chr1	38477814	38478176
chr1	38478438	38479119
chr1	38490699	38490976
chr1	38491474	38492022
chr1	38492244	38492389
chr1	38492554	38492808
chr1	38493020	38493658

chr1	38493901	38494784
chr1	38495380	38496611
chr1	38512909	38513278
chr1	38582642	38582734
chr1	38583121	38583468
chr1	38589079	38589471
chr1	38591200	38591411
chr1	39269700	39269828
chr1	39314809	39315009
chr1	39324594	39325522
chr1	39325674	39325885
chr1	39326006	39326236
chr1	39336806	39336994
chr1	39338426	39339090
chr1	39339305	39339540
chr1	39407173	39407833
chr1	39415951	39415988
chr1	39416735	39417629
chr1	39418914	39419469
chr1	39424536	39425017
chr1	39425312	39425671
chr1	39427144	39427592
chr1	39427819	39428104
chr1	39428182	39428296
chr1	39451328	39451419
chr1	39451958	39451964
chr1	39452236	39452485
chr1	39452598	39453012
chr1	39456259	39456766
chr1	39456925	39458240
chr1	39460563	39460762
chr1	39491676	39492667
chr1	39502779	39502836
chr1	39514820	39515019
chr1	39519951	39520128
chr1	39546741	39547809
chr1	39567098	39567556
chr1	39568185	39568588
chr1	39568823	39569730
chr1	39569922	39572940
chr1	39573613	39573812
chr1	39574041	39574318
chr1	39575935	39576955
chr1	39578699	39579278
chr1	39587979	39588281
chr1	39589470	39589687

chr1	39592572	39592973
chr1	39593184	39593616
chr1	39594602	39595054
chr1	39595125	39595514
chr1	39596042	39596418
chr1	39596693	39596960
chr1	39597668	39597936
chr1	39598162	39599179
chr1	39601995	39602155
chr1	39606725	39606779
chr1	39606913	39607173
chr1	39608612	39608756
chr1	39620527	39620809
chr1	39624093	39624132
chr1	39630745	39631078
chr1	39632519	39632750
chr1	39633069	39633897
chr1	39638811	39639144
chr1	39642860	39643133
chr1	39644186	39644638
chr1	39650690	39651901
chr1	39652988	39653261
chr1	39649172	39650094
chr1	39657207	39658560
chr1	39659965	39660354
chr1	39667486	39667685
chr1	39668046	39668176
chr1	39670253	39671199
chr1	39679051	39679529
chr1	39680207	39680458
chr1	39701177	39701228
chr1	39724256	39724663
chr1	39733932	39734201
chr1	39734463	39734500
chr1	39734982	39735014
chr1	39735523	39735589
chr1	39735824	39736026
chr1	39740989	39741168
chr1	39741587	39742165
chr1	39796993	39797139
chr1	39799005	39799035
chr1	39844155	39844388
chr1	39853541	39853624
chr1	39857682	39858095
chr1	39860076	39860355
chr1	39863961	39864261

chr1	39864604	39864741
chr1	39872171	39872376
chr1	39873437	39873795
chr1	39873861	39874423
chr1	39874666	39875394
chr1	39919333	39919435
chr1	39919611	39919637
chr1	39933884	39934029
chr1	39956602	39957648
chr1	39980383	39981420
chr1	39982602	39982801
chr1	40040040	40040282
chr1	40040348	40041091
chr1	40041183	40042819
chr1	40067590	40067945
chr1	40072055	40072534
chr1	40098615	40098923
chr1	40105030	40105616
chr1	40119574	40119789
chr1	40137630	40137938
chr1	40140844	40140906
chr1	40167033	40167626
chr1	40188511	40188519
chr1	40204245	40205243
chr1	40231822	40231852
chr1	40234322	40234330
chr1	40236060	40236438
chr1	40236622	40237150
chr1	40254343	40255370
chr1	40264753	40264871
chr1	40265125	40265966
chr1	40266149	40266563
chr1	40268622	40268875
chr1	40277383	40277625
chr1	40282741	40283557
chr1	40316319	40316336
chr1	40316439	40316665
chr1	40317939	40318482
chr1	40332401	40332811
chr1	40340908	40341186
chr1	40348869	40349455
chr1	40349987	40350120
chr1	40358816	40358997
chr1	40359184	40359268
chr1	40360767	40361166
chr1	40366262	40367968

chr1	40370796	40371015
chr1	40385572	40385629
chr1	40385813	40385880
chr1	40386019	40386084
chr1	40389851	40390088
chr1	40391372	40391410
chr1	40391944	40392902
chr1	40393017	40393685
chr1	40394214	40394265
chr1	40399644	40400188
chr1	40400305	40401070
chr1	40402325	40402524
chr1	40403303	40403502
chr1	40409101	40410169
chr1	40412127	40412328
chr1	40412802	40412831
chr1	40417411	40417558
chr1	40418434	40418731
chr1	40420059	40420634
chr1	40420846	40422408
chr1	40422823	40423671
chr1	40424104	40424874
chr1	40425134	40425453
chr1	40425579	40426449
chr1	40426564	40426788
chr1	40426935	40427615
chr1	40430588	40430887
chr1	40431403	40431584
chr1	40431915	40432153
chr1	40432343	40432648
chr1	40436820	40437177
chr1	40437854	40438344
chr1	40434526	40436753
chr1	40438784	40439218
chr1	40439378	40439532
chr1	40439597	40439817
chr1	40442319	40442898
chr1	40442980	40443189
chr1	40447519	40447694
chr1	40448038	40448040
chr1	40454359	40454368
chr1	40455930	40456281
chr1	40476282	40476460
chr1	40478351	40478366
chr1	40478528	40478791
chr1	40502329	40502613

chr1	40505285	40506147
chr1	40506424	40507166
chr1	40517095	40517370
chr1	40525366	40525485
chr1	40525638	40525834
chr1	40532655	40532903
chr1	40542799	40542899
chr1	40544390	40544589
chr1	40562698	40562882
chr1	40563225	40563255
chr1	40573164	40573784
chr1	40597610	40597648
chr1	40618716	40618831
chr1	40626741	40626929
chr1	40627056	40627783
chr1	40707353	40707910
chr1	40710859	40711099
chr1	40712380	40712555
chr1	40723252	40723605
chr1	40723867	40724465
chr1	40725044	40725069
chr1	40729707	40730510
chr1	40768787	40771773
chr1	40772669	40772684
chr1	40780491	40781042
chr1	40781841	40782797
chr1	40804735	40804993
chr1	40810505	40810661
chr1	40838998	40840393
chr1	40847214	40847380
chr1	40857430	40857580
chr1	40857698	40857899
chr1	40858046	40858245
chr1	40870659	40870939
chr1	40876900	40877079
chr1	40877259	40877328
chr1	40915461	40915554
chr1	40915729	40916506
chr1	40916682	40916859
chr1	40942576	40943783
chr1	40974106	40975027
chr1	40996995	40997211
chr1	40997412	40997969
chr1	41049108	41049154
chr1	41057941	41058183
chr1	41101188	41101243

chr1	41131006	41131705
chr1	41156970	41157188
chr1	41157305	41158371
chr1	41164766	41165030
chr1	41168413	41168781
chr1	41169082	41169272
chr1	41174394	41175566
chr1	41177033	41177156
chr1	41177306	41177615
chr1	41178243	41178612
chr1	41188393	41188505
chr1	41189022	41189029
chr1	41192794	41192993
chr1	41193449	41193734
chr1	41247905	41248350
chr1	41249097	41249443
chr1	41249806	41250138
chr1	41315965	41316028
chr1	41318410	41318609
chr1	41318747	41319097
chr1	41325733	41325815
chr1	41325924	41325969
chr1	41326223	41327993
chr1	41328211	41328795
chr1	41329712	41329923
chr1	41330327	41330595
chr1	41333921	41334041
chr1	41334191	41334433
chr1	41335273	41335388
chr1	41337907	41338116
chr1	41339029	41339228
chr1	41341301	41341849
chr1	41343326	41344333
chr1	41351796	41352133
chr1	41352233	41352813
chr1	41353637	41353833
chr1	41355297	41356086
chr1	41356425	41356638
chr1	41356754	41356945
chr1	41363096	41363573
chr1	41364042	41364594
chr1	41364904	41365185
chr1	41365483	41365699
chr1	41367542	41367804
chr1	41368251	41368519
chr1	41402674	41402807

chr1	41414317	41414750
chr1	41433062	41433439
chr1	41433760	41434081
chr1	41444578	41444593
chr1	41444704	41446934
chr1	41449118	41449362
chr1	41449769	41450053
chr1	41469258	41469460
chr1	41469572	41469845
chr1	41472682	41473478
chr1	41509912	41510386
chr1	41511267	41511597
chr1	41574970	41576023
chr1	41589124	41589242
chr1	41618260	41618734
chr1	41621503	41621921
chr1	41705932	41706268
chr1	41706730	41707884
chr1	41708088	41708621
chr1	41823682	41824108
chr1	41824575	41824851
chr1	41826838	41827052
chr1	41827176	41827878
chr1	41831947	41832208
chr1	41837125	41837506
chr1	41846357	41846407
chr1	41847241	41847818
chr1	41847966	41848661
chr1	41868555	41868591
chr1	41884030	41884661
chr1	41884961	41885201
chr1	41887407	41887606
chr1	41940484	41940951
chr1	41948420	41949046
chr1	41949410	41949653
chr1	41950018	41950382
chr1	41950489	41950688
chr1	41950840	41951039
chr1	41951916	41952207
chr1	41957107	41957306
chr1	41957775	41957974
chr1	41958212	41959397
chr1	41959667	41959981
chr1	41960626	41962736
chr1	41962999	41963258
chr1	41963423	41963805

chr1	41964277	41964528
chr1	41965911	41966183
chr1	41966330	41966922
chr1	41982003	41982565
chr1	42079496	42079639
chr1	42079757	42079864
chr1	42084057	42084348
chr1	42084602	42084737
chr1	42092286	42092613
chr1	42127181	42127830
chr1	42128114	42129568
chr1	42130177	42131471
chr1	42132152	42132169
chr1	42132412	42133052
chr1	42133247	42133386
chr1	42135551	42135706
chr1	42136017	42136445
chr1	42137307	42137611
chr1	42137865	42138925
chr1	42139143	42139408
chr1	42140133	42140332
chr1	42144322	42145164
chr1	42145548	42145794
chr1	42146934	42148198
chr1	42151964	42152103
chr1	42205857	42206689
chr1	42204648	42205713
chr1	42207023	42207194
chr1	42207832	42208164
chr1	42208325	42208531
chr1	42267844	42268160
chr1	42268564	42268816
chr1	42268936	42269604
chr1	42277858	42278186
chr1	42313715	42313721
chr1	42315400	42315590
chr1	42315760	42315989
chr1	42317016	42317716
chr1	42321333	42321522
chr1	42354105	42354170
chr1	42356430	42356480
chr1	42357115	42357328
chr1	42361213	42361577
chr1	42363587	42363836
chr1	42364203	42364274
chr1	42366071	42366721

chr1	42367477	42367527
chr1	42367708	42367898
chr1	42383766	42384305
chr1	42421079	42421136
chr1	42501058	42501557
chr1	42612848	42612906
chr1	42631929	42632128
chr1	42638624	42639355
chr1	42639605	42640302
chr1	42641183	42641496
chr1	42747759	42747806
chr1	42747945	42747971
chr1	42799915	42800027
chr1	42800259	42801982
chr1	42921303	42922859
chr1	42928560	42928636
chr1	42950027	42950039
chr1	43123199	43124822
chr1	43145120	43145186
chr1	43146967	43147905
chr1	43148070	43148599
chr1	43148721	43149135
chr1	43149827	43150120
chr1	43155615	43155896
chr1	43189186	43189364
chr1	43202146	43202348
chr1	43208722	43208760
chr1	43208875	43209026
chr1	43211109	43211308
chr1	43231402	43231671
chr1	43231817	43232755
chr1	43232931	43233249
chr1	43233405	43233482
chr1	43250814	43251053
chr1	43282080	43282564
chr1	43282792	43283168
chr1	43311789	43313074
chr1	43388743	43389081
chr1	43389581	43391001
chr1	43391197	43391279
chr1	43391443	43391765
chr1	43397391	43397457
chr1	43400252	43400451
chr1	43401572	43404527
chr1	43405176	43405361
chr1	43405709	43405913

chr1	43406356	43408926
chr1	43409249	43410724
chr1	43410926	43411200
chr1	43411903	43412437
chr1	43414258	43414346
chr1	43414788	43415036
chr1	43416337	43416595
chr1	43417295	43417494
chr1	43418853	43419158
chr1	43420320	43420420
chr1	43422875	43423233
chr1	43423342	43424482
chr1	43424670	43425250
chr1	43425381	43425536
chr1	43427920	43430052
chr1	43432428	43432830
chr1	43432964	43433424
chr1	43445951	43446150
chr1	43446393	43446394
chr1	43446871	43446932
chr1	43473406	43473752
chr1	43487146	43487324
chr1	43487500	43487738
chr1	43487900	43488150
chr1	43488264	43488274
chr1	43524087	43524425
chr1	43524542	43524640
chr1	43533513	43534382
chr1	43539312	43539465
chr1	43585704	43586294
chr1	43618409	43618582
chr1	43618739	43618968
chr1	43637697	43637802
chr1	43672291	43672612
chr1	43672866	43673549
chr1	43691573	43691774
chr1	43736399	43736598
chr1	43750703	43751614
chr1	43813540	43813718
chr1	43814159	43815461
chr1	43823213	43823564
chr1	43823774	43824374
chr1	43824503	43825386
chr1	43826339	43826587
chr1	43827666	43827743
chr1	43828362	43828629

chr1	43831529	43832011
chr1	43832196	43833740
chr1	43833854	43834468
chr1	43834674	43834708
chr1	43836747	43836907
chr1	43838269	43838494
chr1	43838676	43839197
chr1	43854170	43854175
chr1	43855029	43855936
chr1	43888689	43889360
chr1	43889613	43890011
chr1	43911240	43911325
chr1	43918375	43918547
chr1	43918743	43919905
chr1	43920082	43920724
chr1	43938074	43938930
chr1	43941236	43942081
chr1	43943424	43943788
chr1	43945555	43945886
chr1	43969750	43969949
chr1	43970659	43970758
chr1	43971687	43971990
chr1	43972703	43972916
chr1	43975512	43975711
chr1	43975860	43975978
chr1	43988590	43988799
chr1	43989064	43989249
chr1	43990896	43992215
chr1	43992861	43993077
chr1	43995166	43995479
chr1	43996279	43997724
chr1	43997795	43998386
chr1	43998463	43998698
chr1	44001693	44001953
chr1	44010264	44010266
chr1	44010352	44010463
chr1	44011472	44011990
chr1	44012501	44012727
chr1	44013371	44017049
chr1	44018467	44018805
chr1	44020358	44020462
chr1	44022931	44023350
chr1	44027625	44027972
chr1	44029804	44030003
chr1	44033622	44033830
chr1	44045317	44045597

chr1	44047592	44047921
chr1	44048049	44048843
chr1	44051728	44051927
chr1	44052184	44052429
chr1	44052820	44053304
chr1	44053473	44053813
chr1	44061026	44061235
chr1	44097113	44097291
chr1	44101170	44101385
chr1	44102084	44102095
chr1	44105134	44105318
chr1	44105905	44106151
chr1	44109576	44111073
chr1	44113779	44114309
chr1	44115074	44115580
chr1	44115798	44116075
chr1	44116212	44116809
chr1	44116933	44117032
chr1	44117217	44117301
chr1	44117647	44117846
chr1	44118308	44118644
chr1	44119270	44119418
chr1	44129720	44129798
chr1	44129920	44129923
chr1	44131944	44132175
chr1	44132707	44132819
chr1	44134609	44134800
chr1	44137351	44137566
chr1	44137842	44138133
chr1	44138225	44138469
chr1	44139761	44139915
chr1	44151922	44152272
chr1	44171621	44171936
chr1	44172245	44173008
chr1	44173200	44173673
chr1	44173779	44174039
chr1	44176692	44178112
chr1	44180322	44180383
chr1	44229314	44229561
chr1	44231800	44233185
chr1	44240080	44240408
chr1	44249162	44249373
chr1	44249739	44249960
chr1	44266942	44267314
chr1	44287638	44288155
chr1	44302470	44302603

chr1	44327276	44327336
chr1	44348823	44348961
chr1	44350468	44351074
chr1	44351188	44351608
chr1	44369164	44369376
chr1	44370415	44370810
chr1	44371220	44371520
chr1	44374417	44374653
chr1	44378150	44379227
chr1	44379659	44379700
chr1	44379909	44381222
chr1	44382103	44382390
chr1	44386655	44386696
chr1	44387397	44388191
chr1	44388324	44388539
chr1	44389270	44389515
chr1	44393968	44394077
chr1	44395161	44395360
chr1	44395538	44395776
chr1	44396323	44397788
chr1	44398332	44398505
chr1	44398694	44403377
chr1	44403761	44404024
chr1	44403617	44403693
chr1	44405485	44405761
chr1	44411570	44413448
chr1	44435798	44436421
chr1	44439516	44441469
chr1	44443003	44443085
chr1	44444181	44444700
chr1	44445106	44446389
chr1	44446608	44447176
chr1	44454747	44454946
chr1	44456786	44458262
chr1	44458465	44458664
chr1	44458809	44459194
chr1	44459408	44459486
chr1	44494216	44494625
chr1	44495014	44495617
chr1	44495752	44497977
chr1	44509558	44509951
chr1	44513332	44514203
chr1	44515450	44515504
chr1	44620546	44620758
chr1	44620826	44621266
chr1	44678312	44678611

chr1	44678794	44678916
chr1	44679106	44679821
chr1	44692769	44693025
chr1	44705473	44705672
chr1	44792560	44793017
chr1	44793232	44793552
chr1	44794030	44794395
chr1	44820268	44820506
chr1	44820583	44821494
chr1	44870893	44871368
chr1	45018541	45018621
chr1	45019217	45019307
chr1	45020274	45020483
chr1	45020611	45020980
chr1	45021671	45021877
chr1	45082390	45082715
chr1	45082916	45083366
chr1	45097287	45098665
chr1	45139312	45139688
chr1	45139796	45140265
chr1	45140451	45140799
chr1	45141333	45141692
chr1	45142213	45142392
chr1	45156450	45156911
chr1	45173136	45173345
chr1	45178664	45178813
chr1	45187137	45188158
chr1	45188499	45188516
chr1	45193071	45193098
chr1	45196040	45197297
chr1	45198075	45198231
chr1	45205115	45205894
chr1	45206272	45206273
chr1	45230230	45230238
chr1	45239305	45239451
chr1	45240741	45241183
chr1	45241307	45241912
chr1	45242016	45242071
chr1	45242207	45242514
chr1	45242667	45243231
chr1	45246867	45246901
chr1	45252166	45252365
chr1	45259558	45259755
chr1	45260307	45260529
chr1	45262304	45262807
chr1	45264863	45265933

chr1	45266049	45266609
chr1	45266774	45266974
chr1	45267073	45268531
chr1	45269478	45269679
chr1	45272014	45274950
chr1	45279571	45280770
chr1	45280929	45281547
chr1	45283520	45283702
chr1	45283771	45283859
chr1	45284924	45286397
chr1	45308572	45309051
chr1	45309198	45309451
chr1	45321307	45321489
chr1	45321825	45321882
chr1	45390478	45390869
chr1	45409668	45409863
chr1	45423162	45423570
chr1	45451287	45451389
chr1	45451600	45451631
chr1	45451738	45452208
chr1	45452425	45452913
chr1	45467424	45467624
chr1	45475849	45476270
chr1	45476497	45476970
chr1	45477165	45477666
chr1	45477931	45479037
chr1	45479236	45479339
chr1	45671998	45672235
chr1	45768845	45769351
chr1	45769486	45770097
chr1	45770359	45770476
chr1	45794416	45794755
chr1	45791853	45794191
chr1	45804975	45806916
chr1	45813518	45813859
chr1	45813979	45814132
chr1	45814563	45814974
chr1	45815229	45815794
chr1	45821879	45821949
chr1	45825928	45825954
chr1	45826660	45826761
chr1	45846849	45846877
chr1	45847209	45847420
chr1	45847835	45847844
chr1	45859044	45859444
chr1	45859722	45860060

chr1	45868897	45869273
chr1	45954691	45954890
chr1	45955842	45956773
chr1	45957320	45957480
chr1	45959924	45959993
chr1	45962294	45962413
chr1	45965000	45965695
chr1	45966048	45966663
chr1	45966779	45966816
chr1	45966921	45967163
chr1	45978635	45978708
chr1	45979279	45979312
chr1	45979597	45979665
chr1	45982085	45982503
chr1	45986437	45987631
chr1	45987747	45988746
chr1	45993241	45993359
chr1	45998969	45999197
chr1	46015970	46017187
chr1	46017933	46018160
chr1	46022337	46022466
chr1	46022930	46023049
chr1	46022593	46022797
chr1	46023476	46023542
chr1	46048865	46048960
chr1	46049202	46050669
chr1	46078865	46079296
chr1	46120852	46120893
chr1	46142806	46142922
chr1	46151517	46152165
chr1	46152339	46152747
chr1	46152990	46153273
chr1	46153631	46154054
chr1	46154188	46154277
chr1	46162926	46163112
chr1	46183078	46183451
chr1	46196077	46196091
chr1	46215283	46215689
chr1	46215763	46216809
chr1	46268344	46270002
chr1	46306558	46306751
chr1	46357756	46358241
chr1	46358348	46358464
chr1	46417949	46418158
chr1	46420524	46420564
chr1	46505526	46505774

chr1	46531030	46531189
chr1	46531353	46531416
chr1	46543443	46543674
chr1	46550309	46550624
chr1	46594312	46594407
chr1	46597436	46597814
chr1	46598038	46599259
chr1	46600434	46600621
chr1	46600919	46601118
chr1	46601326	46601514
chr1	46636172	46636324
chr1	46636885	46637104
chr1	46637320	46637605
chr1	46637795	46638658
chr1	46639028	46639298
chr1	46639946	46640145
chr1	46645086	46645233
chr1	46645520	46645721
chr1	46646228	46646867
chr1	46647258	46647481
chr1	46647676	46647875
chr1	46648245	46648931
chr1	46649113	46650039
chr1	46650122	46650577
chr1	46650864	46651163
chr1	46655677	46656409
chr1	46657141	46657235
chr1	46663480	46665173
chr1	46668552	46668754
chr1	46668953	46669629
chr1	46683833	46684196
chr1	46684304	46684676
chr1	46688505	46688515
chr1	46688809	46689325
chr1	46689507	46689664
chr1	46694848	46694940
chr1	46701697	46701799
chr1	46710584	46710648
chr1	46712216	46712960
chr1	46713464	46714168
chr1	46754761	46754903
chr1	46764986	46765280
chr1	46767116	46769565
chr1	46799151	46799276
chr1	46804776	46805276
chr1	46805369	46807245

chr1	46807477	46807617
chr1	46813691	46813886
chr1	46867442	46867641
chr1	46868153	46868342
chr1	46868554	46868788
chr1	46892208	46892266
chr1	46932041	46932294
chr1	46994017	46994303
chr1	46998897	46999197
chr1	47019039	47019238
chr1	47037900	47038179
chr1	47069225	47069919
chr1	47070113	47070310
chr1	47077006	47078299
chr1	47081632	47082792
chr1	47082985	47083303
chr1	47133448	47134056
chr1	47184196	47184847
chr1	47185273	47185841
chr1	47218471	47218511
chr1	47222649	47222751
chr1	47222824	47222867
chr1	47223098	47223754
chr1	47323128	47323199
chr1	47323457	47323725
chr1	47573435	47573604
chr1	47644604	47644843
chr1	47645069	47645435
chr1	47645595	47645623
chr1	47646471	47646557
chr1	47647269	47647468
chr1	47655488	47656032
chr1	47656209	47656567
chr1	47691239	47691476
chr1	47719602	47719755
chr1	47719999	47720117
chr1	47731129	47731600
chr1	47749644	47749843
chr1	47778682	47779914
chr1	47780148	47780956
chr1	47791181	47791326
chr1	47798781	47799415
chr1	47799624	47800625
chr1	47817035	47817328
chr1	47902941	47903264
chr1	48035269	48035598

chr1	48848413	48848677
chr1	48938084	48938302
chr1	51069254	51069896
chr1	51071182	51071347
chr1	51076555	51076606
chr1	51086544	51086820
chr1	51086961	51087159
chr1	51157134	51157333
chr1	51171791	51171846
chr1	51215928	51216016
chr1	51339207	51339435
chr1	51339713	51339715
chr1	51424882	51425923
chr1	51426166	51426551
chr1	51433612	51435434
chr1	51435687	51436089
chr1	51436671	51436887
chr1	51442936	51444214
chr1	51529259	51529260
chr1	51636320	51636422
chr1	51700733	51700932
chr1	51701284	51702847
chr1	51726867	51727278
chr1	51733755	51733874
chr1	51734058	51734103
chr1	51762346	51762436
chr1	51762499	51762601
chr1	51762714	51763482
chr1	51775745	51775967
chr1	51778617	51778636
chr1	51778901	51779063
chr1	51779205	51780867
chr1	51781211	51781440
chr1	51795732	51795931
chr1	51796281	51796605
chr1	51983588	51984850
chr1	52024112	52024201
chr1	52024483	52024530
chr1	52029825	52029842
chr1	52030064	52030134
chr1	52034879	52035592
chr1	52035849	52035856
chr1	52082465	52082551
chr1	52082813	52083718
chr1	52088057	52088060
chr1	52088307	52088545

chr1	52102076	52102252
chr1	52102632	52102680
chr1	52102842	52103249
chr1	52104319	52104485
chr1	52110151	52110298
chr1	52114016	52114176
chr1	52154126	52154191
chr1	52156333	52156986
chr1	52159242	52159493
chr1	52194818	52195243
chr1	52195488	52196010
chr1	52238957	52239092
chr1	52294718	52295155
chr1	52304773	52305012
chr1	52343623	52345114
chr1	52362563	52362884
chr1	52363207	52364359
chr1	52402430	52402813
chr1	52421131	52421379
chr1	52455990	52456436
chr1	52465199	52465247
chr1	52498325	52499435
chr1	52499582	52499675
chr1	52507480	52507806
chr1	52508370	52509083
chr1	52520151	52522664
chr1	52525087	52525286
chr1	52537620	52537631
chr1	52539548	52539838
chr1	52593058	52593754
chr1	52603901	52604100
chr1	52604181	52604197
chr1	52605443	52606206
chr1	52607436	52608787
chr1	52624511	52624744
chr1	52624978	52625251
chr1	52677165	52677283
chr1	52721973	52722200
chr1	52789268	52789836
chr1	52830739	52832336
chr1	52833586	52833786
chr1	52834133	52834946
chr1	52869279	52869425
chr1	52869532	52870044
chr1	52870280	52870795
chr1	52925254	52925287

chr1	52948053	52948354
chr1	53017729	53017873
chr1	53017995	53019635
chr1	53020462	53020790
chr1	53022365	53022594
chr1	53022822	53023068
chr1	53040552	53040633
chr1	53052458	53052614
chr1	53067892	53068530
chr1	53097406	53097431
chr1	53097662	53097864
chr1	53098123	53098855
chr1	53098963	53099437
chr1	53102235	53102374
chr1	53102501	53102758
chr1	53124233	53124339
chr1	53124739	53124892
chr1	53125077	53125156
chr1	53125412	53125714
chr1	53128197	53129057
chr1	53129135	53129575
chr1	53131012	53131392
chr1	53131605	53131816
chr1	53131994	53132196
chr1	53134532	53134782
chr1	53135378	53135505
chr1	53138170	53138391
chr1	53138590	53138666
chr1	53138795	53138994
chr1	53163040	53164059
chr1	53164167	53164635
chr1	53166583	53166971
chr1	53168498	53169021
chr1	53169149	53169533
chr1	53191733	53191930
chr1	53192085	53192604
chr1	53195421	53195666
chr1	53222771	53222938
chr1	53223116	53223249
chr1	53280360	53280502
chr1	53299159	53299255
chr1	53308076	53309789
chr1	53309933	53309979
chr1	53321706	53321752
chr1	53321961	53322167
chr1	53326048	53326318

chr1	53326558	53326637
chr1	53386576	53387387
chr1	53387558	53387717
chr1	53392584	53393685
chr1	53394394	53394413
chr1	53405394	53405843
chr1	53407616	53407664
chr1	53410472	53410631
chr1	53410960	53411181
chr1	53415276	53415475
chr1	53480432	53480530
chr1	53480744	53480824
chr1	53497405	53497823
chr1	53527845	53528093
chr1	53611747	53611960
chr1	53612272	53612401
chr1	53619685	53619802
chr1	53640571	53640633
chr1	53662138	53662219
chr1	53662471	53663632
chr1	53664352	53664462
chr1	53685713	53686823
chr1	53703446	53703704
chr1	53704000	53704204
chr1	53704316	53704898
chr1	53739284	53739483
chr1	53791188	53791840
chr1	53792076	53793601
chr1	53793833	53794382
chr1	54007327	54008007
chr1	54204500	54204858
chr1	54205037	54205238
chr1	54302748	54303063
chr1	54303643	54304520
chr1	54355104	54356593
chr1	54373253	54373516
chr1	54375462	54375720
chr1	54375924	54376058
chr1	54410139	54410696
chr1	54410985	54411366
chr1	54411639	54413061
chr1	54418965	54419260
chr1	54422256	54422936
chr1	54423144	54423299
chr1	54447730	54447940
chr1	54517939	54519186

chr1	54519297	54519847
chr1	54587606	54587857
chr1	54619352	54619551
chr1	54619949	54620117
chr1	54627326	54627486
chr1	54633667	54634024
chr1	54665487	54666327
chr1	54666406	54666847
chr1	54668232	54668524
chr1	54718992	54719171
chr1	54788477	54788524
chr1	54788885	54788987
chr1	54801092	54801482
chr1	54801726	54801847
chr1	54870635	54870836
chr1	54871012	54871487
chr1	54872092	54873268
chr1	54873431	54873528
chr1	54873627	54873920
chr1	54875545	54875696
chr1	54940476	54940735
chr1	54951535	54952189
chr1	54953721	54954223
chr1	54954502	54955209
chr1	54965007	54965243
chr1	54965404	54965715
chr1	55007423	55009359
chr1	55029267	55030554
chr1	55031630	55031841
chr1	55032310	55032574
chr1	55032776	55032780
chr1	55033148	55033257
chr1	55034894	55035429
chr1	55036016	55036259
chr1	55036433	55038559
chr1	55033436	55034052
chr1	55038815	55038952
chr1	55039119	55039321
chr1	55039477	55039779
chr1	55044699	55044898
chr1	55047609	55047838
chr1	55181030	55181216
chr1	55181561	55181941
chr1	55221371	55221841
chr1	55227389	55227470
chr1	55229488	55230133

chr1	55230613	55230706
chr1	55247008	55247319
chr1	55260295	55260517
chr1	55265005	55265278
chr1	55266187	55266963
chr1	55267209	55267521
chr1	55270128	55270223
chr1	55315999	55316596
chr1	55320524	55320696
chr1	55321891	55322458
chr1	55322888	55323170
chr1	55323410	55323591
chr1	55323936	55324277
chr1	55324652	55324679
chr1	55324991	55325081
chr1	55326038	55326111
chr1	55330209	55330245
chr1	55341785	55342202
chr1	55344094	55344173
chr1	55348165	55348472
chr1	55349604	55349771
chr1	55351276	55354385
chr1	55370144	55370660
chr1	55372184	55372252
chr1	55388492	55389301
chr1	55392331	55392350
chr1	55393521	55393698
chr1	55393805	55394045
chr1	55400593	55400964
chr1	55402476	55402845
chr1	55410257	55410356
chr1	55410581	55410800
chr1	55411495	55411634
chr1	55412288	55412437
chr1	55413172	55413497
chr1	55416118	55416490
chr1	55421066	55421465
chr1	55436379	55436702
chr1	55437441	55437515
chr1	55446142	55446922
chr1	55504304	55504329
chr1	55504764	55504827
chr1	55505203	55506789
chr1	55507731	55507882
chr1	55508534	55508581
chr1	55519607	55519740

chr1	55525721	55525785
chr1	55533185	55533298
chr1	55546887	55547211
chr1	55547321	55547440
chr1	55590351	55590432
chr1	55606603	55606622
chr1	55661403	55661479
chr1	55679405	55680013
chr1	55680129	55681690
chr1	55701437	55701597
chr1	55750698	55750798
chr1	55819388	55819543
chr1	55894557	55895106
chr1	55895294	55895416
chr1	55939852	55940013
chr1	56982877	56982975
chr1	57044330	57044364
chr1	57044539	57044626
chr1	57111069	57111319
chr1	57297959	57298158
chr1	57745812	57745959
chr1	58056597	58056718
chr1	58056908	58057009
chr1	58057474	58057611
chr1	58057876	58057976
chr1	58297224	58297503
chr1	58310335	58310848
chr1	58360008	58360106
chr1	58715821	58716004
chr1	58981756	58981897
chr1	59011512	59012834
chr1	59038321	59038773
chr1	59039419	59040813
chr1	59041205	59043883
chr1	59044186	59044859
chr1	59045838	59046044
chr1	59049761	59050672
chr1	59051584	59052035
chr1	59057918	59058733
chr1	59082915	59083122
chr1	59083842	59084098
chr1	59084370	59084444
chr1	59085368	59085593
chr1	59118245	59118468
chr1	59119418	59119485
chr1	59165502	59166074

chr1	59176939	59177165
chr1	59192988	59193021
chr1	59193467	59193594
chr1	59204756	59204932
chr1	59205567	59205645
chr1	59218711	59219131
chr1	59219725	59219753
chr1	59219991	59220193
chr1	59220938	59221585
chr1	59228934	59229012
chr1	59245370	59246282
chr1	59246553	59246952
chr1	59247092	59247352
chr1	59247482	59248563
chr1	59248781	59249594
chr1	59249980	59251582
chr1	59251699	59251859
chr1	59255049	59255188
chr1	59275360	59275422
chr1	59276997	59277427
chr1	59278155	59278517
chr1	59279672	59280446
chr1	59280550	59281752
chr1	59281827	59282447
chr1	59290947	59291157
chr1	59314242	59314400
chr1	59314896	59315191
chr1	59348814	59349073
chr1	59349305	59349414
chr1	59360998	59361121
chr1	59368981	59370031
chr1	59374966	59375217
chr1	59387861	59388011
chr1	59388292	59388446
chr1	59393037	59393155
chr1	59395351	59395501
chr1	59395727	59395800
chr1	59397418	59397743
chr1	59398414	59399051
chr1	59437364	59437402
chr1	59441583	59441669
chr1	59442050	59442197
chr1	59481386	59482539
chr1	59485868	59486286
chr1	59550393	59550528
chr1	59573117	59573320

chr1	59573527	59573702
chr1	59602108	59602241
chr1	59604921	59605559
chr1	59608628	59608915
chr1	59609103	59609217
chr1	59609569	59609803
chr1	59612404	59613099
chr1	59616370	59616700
chr1	59616957	59617411
chr1	59758240	59758255
chr1	59758542	59758623
chr1	59758901	59759100
chr1	59761941	59763161
chr1	59795028	59795195
chr1	59795380	59795473
chr1	59825924	59826123
chr1	59840852	59841046
chr1	59841357	59841434
chr1	59893116	59893464
chr1	59910702	59910844
chr1	59911071	59911139
chr1	59949773	59950095
chr1	59953062	59953191
chr1	59953305	59953496
chr1	59964878	59965235
chr1	59965319	59965405
chr1	59965688	59965777
chr1	60062003	60063039
chr1	60138444	60139618
chr1	60139836	60140032
chr1	60140213	60140624
chr1	60171638	60171801
chr1	60173068	60173253
chr1	60195904	60196359
chr1	60196552	60196906
chr1	60216345	60216460
chr1	60216591	60216739
chr1	60224992	60225213
chr1	60227500	60227551
chr1	60231010	60231075
chr1	60232908	60233131
chr1	60279937	60280255
chr1	60280527	60281438
chr1	60299750	60299982
chr1	60358485	60358563
chr1	60392244	60392472

chr1	60392699	60392768
chr1	60747029	60747080
chr1	61038694	61038973
chr1	61039148	61039198
chr1	61039351	61039795
chr1	61343934	61344167
chr1	61344320	61344432
chr1	61470663	61470754
chr1	61508687	61509288
chr1	61515773	61516916
chr1	61519463	61519728
chr1	61522480	61522979
chr1	61523166	61524037
chr1	61524164	61524246
chr1	61542427	61543154
chr1	61543459	61543711
chr1	61543866	61544057
chr1	61545475	61545555
chr1	61545927	61545958
chr1	61547052	61549592
chr1	61563005	61563134
chr1	61586626	61586961
chr1	61604776	61605534
chr1	61607103	61607727
chr1	61608321	61609066
chr1	61614872	61615415
chr1	61635950	61636130
chr1	61719312	61719394
chr1	61744200	61744519
chr1	61751758	61752242
chr1	61759000	61759060
chr1	61784264	61784881
chr1	61787699	61788052
chr1	61788486	61788520
chr1	61788694	61788849
chr1	61789647	61789877
chr1	61802766	61803016
chr1	61803578	61804512
chr1	61918736	61918836
chr1	61919233	61919436
chr1	61947081	61947162
chr1	61962160	61962628
chr1	61970579	61971045
chr1	61984714	61984759
chr1	61984951	61985123
chr1	61985345	61985544

chr1	62051349	62051373
chr1	62054588	62054838
chr1	62071447	62071905
chr1	62072216	62072588
chr1	62084421	62084835
chr1	62107344	62107495
chr1	62158192	62158249
chr1	62189856	62190195
chr1	62190558	62191064
chr1	62191257	62191353
chr1	62206916	62209040
chr1	62209586	62209886
chr1	62220084	62220675
chr1	62223860	62224020
chr1	62303560	62303700
chr1	62305634	62305668
chr1	62317839	62317912
chr1	62458878	62459092
chr1	62548737	62548754
chr1	62587223	62587422
chr1	62587580	62588526
chr1	62601744	62601936
chr1	62611288	62611445
chr1	62650871	62651110
chr1	62660034	62661075
chr1	62691989	62692089
chr1	62694769	62694974
chr1	62695087	62695504
chr1	62696243	62696965
chr1	62698390	62699075
chr1	62702433	62702629
chr1	62702807	62702824
chr1	62773635	62773851
chr1	62784244	62784378
chr1	62784670	62785195
chr1	62789724	62790040
chr1	62820662	62820958
chr1	62821081	62821321
chr1	62864301	62864583
chr1	62900927	62901340
chr1	62901415	62901899
chr1	62902459	62903482
chr1	62903819	62904182
chr1	63088048	63088097
chr1	63088408	63088674
chr1	63153140	63154050

chr1	63155470	63155749
chr1	63156076	63156616
chr1	63178629	63178875
chr1	63181966	63182432
chr1	63212237	63212258
chr1	63243935	63244020
chr1	63249195	63250039
chr1	63720947	63721092
chr1	63782380	63783023
chr1	63787919	63788118
chr1	63832786	63833059
chr1	63833347	63834323
chr1	63871041	63871081
chr1	63928931	63929001
chr1	63929197	63929298
chr1	63941715	63941777
chr1	63988455	63988794
chr1	63988927	63989692
chr1	64011478	64011807
chr1	64013233	64013605
chr1	64058678	64060416
chr1	64095880	64096241
chr1	64096310	64096601
chr1	64127651	64129484
chr1	64197162	64197274
chr1	64239401	64239459
chr1	64239600	64239799
chr1	64505498	64505537
chr1	64649953	64650154
chr1	64728869	64729089
chr1	64729359	64729560
chr1	64774528	64774737
chr1	64789034	64789080
chr1	64808752	64808800
chr1	64809875	64810277
chr1	64814977	64815017
chr1	64935867	64936888
chr1	64937075	64937372
chr1	64941746	64942004
chr1	64942165	64942653
chr1	64970912	64971564
chr1	65063876	65064047
chr1	65070637	65070712
chr1	65120302	65120454
chr1	65120891	65121024
chr1	65209952	65210119

chr1	65210232	65210516
chr1	65210687	65211213
chr1	65211677	65211847
chr1	65289376	65289483
chr1	65289603	65289938
chr1	65363701	65363804
chr1	65364003	65364343
chr1	65383054	65383265
chr1	65414844	65415201
chr1	65418101	65418462
chr1	65431130	65432272
chr1	65455159	65455251
chr1	65455640	65455751
chr1	65467855	65469026
chr1	65502741	65502843
chr1	65532062	65533230
chr1	65533702	65533992
chr1	65534330	65534637
chr1	65545738	65545937
chr1	65606985	65607102
chr1	65612036	65612078
chr1	65612525	65612687
chr1	65612893	65615261
chr1	65628928	65628943
chr1	65629077	65629197
chr1	65630427	65630612
chr1	65639171	65639521
chr1	65640185	65640623
chr1	65643296	65643593
chr1	65643947	65644199
chr1	65644700	65644782
chr1	65650408	65650483
chr1	65681422	65681631
chr1	65713291	65713956
chr1	65719726	65720079
chr1	65720256	65721146
chr1	65731233	65731788
chr1	65731929	65732047
chr1	65808796	65809141
chr1	65810085	65810236
chr1	65859022	65859076
chr1	65883412	65883887
chr1	65885514	65887135
chr1	65889552	65890043
chr1	65891251	65891838
chr1	65895453	65895923

chr1	66719226	66719343
chr1	66727848	66728001
chr1	66728169	66728348
chr1	66736871	66737056
chr1	66737185	66737389
chr1	66737509	66738005
chr1	66760392	66760834
chr1	66766869	66767185
chr1	66781553	66781758
chr1	66797805	66798022
chr1	66799194	66799369
chr1	66882061	66882069
chr1	66902090	66902538
chr1	66912125	66912269
chr1	66987432	66987527
chr1	67029148	67029416
chr1	67029784	67030100
chr1	67030232	67030478
chr1	67041424	67041483
chr1	67041636	67041848
chr1	67064494	67064756
chr1	67389698	67390915
chr1	67391051	67391910
chr1	67394988	67395306
chr1	67395510	67396499
chr1	67396605	67396687
chr1	67399986	67400404
chr1	67410292	67410724
chr1	67417294	67417553
chr1	67479266	67479290
chr1	67518660	67520222
chr1	67550526	67550618
chr1	67614824	67615148
chr1	67628901	67629109
chr1	67772804	67772963
chr1	67773219	67773787
chr1	67773895	67774339
chr1	67790394	67790422
chr1	67793503	67793550
chr1	67799269	67799658
chr1	67802635	67802792
chr1	67805362	67805487
chr1	67808655	67808830
chr1	67813771	67814097
chr1	67814219	67814309
chr1	67821324	67821758

chr1	67832684	67833005
chr1	67837967	67837989
chr1	67865566	67866273
chr1	67870076	67870275
chr1	67881655	67881953
chr1	67894949	67895040
chr1	67895298	67895973
chr1	67896234	67897113
chr1	67937529	67937600
chr1	67966197	67966627
chr1	67996346	67996506
chr1	67996787	67996972
chr1	68001098	68001240
chr1	68001345	68001497
chr1	68024748	68025056
chr1	68025210	68025602
chr1	68027610	68027900
chr1	68035852	68036363
chr1	68036544	68036771
chr1	68037182	68037399
chr1	68038141	68038768
chr1	68041438	68042005
chr1	68042224	68042423
chr1	68046353	68046810
chr1	68084632	68084771
chr1	68096475	68096530
chr1	68100859	68101131
chr1	68148234	68148758
chr1	68149036	68149371
chr1	68149720	68150116
chr1	68150521	68150522
chr1	68150915	68152424
chr1	68152616	68152785
chr1	68152928	68153127
chr1	68153334	68153739
chr1	68154363	68154713
chr1	68176427	68176608
chr1	68179054	68179653
chr1	68182914	68182971
chr1	68183572	68183656
chr1	68183836	68183908
chr1	68189391	68189772
chr1	68191895	68192318
chr1	68201655	68202415
chr1	68204244	68204644
chr1	68214182	68214719

chr1	68215105	68216502
chr1	68217926	68218113
chr1	68218220	68218348
chr1	68220030	68220179
chr1	68225948	68225982
chr1	68226229	68226428
chr1	68231963	68232554
chr1	68257368	68258115
chr1	68269404	68269833
chr1	68280907	68282374
chr1	68285010	68285092
chr1	68295969	68296203
chr1	68297542	68298223
chr1	68298362	68299687
chr1	68302295	68302844
chr1	68304057	68304286
chr1	68304590	68304633
chr1	68313192	68313294
chr1	68360656	68360947
chr1	68663469	68663988
chr1	68664445	68664923
chr1	68684155	68684629
chr1	68686840	68687374
chr1	68687664	68687940
chr1	68691513	68691661
chr1	68692651	68692850
chr1	68694859	68694980
chr1	68695607	68695875
chr1	68696269	68697045
chr1	68697394	68698630
chr1	68961985	68962040
chr1	68962328	68962721
chr1	69520962	69521020
chr1	70034268	70034618
chr1	70159633	70159667
chr1	70168079	70168148
chr1	70500124	70500243
chr1	70509609	70509678
chr1	70509806	70509834
chr1	70556532	70556674
chr1	70556894	70556933
chr1	70598838	70599156
chr1	70670952	70671809
chr1	70686792	70686843
chr1	70687013	70687676
chr1	70693420	70693483

chr1	70750123	70750456
chr1	70785640	70785853
chr1	70793023	70793371
chr1	70793491	70793697
chr1	70794030	70794469
chr1	70797707	70798048
chr1	70819378	70820900
chr1	70832958	70833058
chr1	70876247	70876458
chr1	70877727	70878183
chr1	70876984	70877423
chr1	70976054	70976124
chr1	71513121	71513431
chr1	71546244	71546462
chr1	71546538	71546965
chr1	71547130	71547241
chr1	72747545	72747814
chr1	72748817	72749932
chr1	72750023	72750868
chr1	72832148	72832216
chr1	73089295	73089329
chr1	73357670	73357811
chr1	73357939	73358022
chr1	73358198	73358462
chr1	74039280	74039443
chr1	74059058	74059215
chr1	74059373	74059454
chr1	74140724	74140958
chr1	74626048	74626153
chr1	74663535	74663815
chr1	74664177	74664281
chr1	75198207	75199708
chr1	75199932	75200201
chr1	76188210	76188335
chr1	76189616	76190818
chr1	76191845	76191910
chr1	76210905	76211075
chr1	76231399	76231540
chr1	76231668	76231818
chr1	76233910	76233969
chr1	76241309	76241751
chr1	76251371	76251808
chr1	76252059	76252210
chr1	76256696	76257052
chr1	76320633	76320710
chr1	76321134	76321154

chr1	76429867	76430010
chr1	76430395	76430519
chr1	76540023	76540195
chr1	76620045	76620244
chr1	77367147	77367216
chr1	77460958	77461332
chr1	77463571	77463927
chr1	77464254	77464880
chr1	77496971	77497159
chr1	77505314	77505935
chr1	77685006	77685205
chr1	77747471	77747904
chr1	77977156	77977403
chr1	78002510	78002572
chr1	78005174	78005408
chr1	78006202	78006551
chr1	78104760	78104977
chr1	78105081	78105160
chr1	78147272	78147517
chr1	78147771	78148626
chr1	78148793	78149728
chr1	78150802	78150924
chr1	78152944	78153075
chr1	78153168	78153877
chr1	78210275	78210644
chr1	78224721	78225535
chr1	78245099	78245942
chr1	78286575	78286703
chr1	78289788	78290175
chr1	78290749	78291012
chr1	78291326	78291423
chr1	78353942	78355170
chr1	78366475	78366733
chr1	78441106	78441423
chr1	78442102	78442651
chr1	78442935	78443270
chr1	78443767	78444232
chr1	78444339	78445424
chr1	78445598	78445849
chr1	78469670	78469994
chr1	78470311	78470793
chr1	78484781	78484816
chr1	78499530	78499620
chr1	78511589	78512218
chr1	78578962	78579186
chr1	78611148	78611202

chr1	78695019	78695470
chr1	78806118	78806348
chr1	78957388	78957685
chr1	79052912	79052948
chr1	79083280	79083285
chr1	79083517	79083667
chr1	79085506	79086064
chr1	79115270	79115715
chr1	79116851	79117082
chr1	79117196	79117343
chr1	79117805	79118004
chr1	79338483	79338644
chr1	79554722	79554997
chr1	79571145	79571287
chr1	79594597	79594719
chr1	79659052	79659485
chr1	79769933	79769965
chr1	79788856	79789411
chr1	80520182	80520339
chr1	80841302	80841426
chr1	80841715	80841866
chr1	80858265	80858314
chr1	80883337	80883686
chr1	81608097	81608104
chr1	81639652	81639702
chr1	81639999	81640125
chr1	82147247	82147418
chr1	82164880	82164904
chr1	82165183	82165305
chr1	82168901	82168916
chr1	82625687	82625857
chr1	82691221	82691420
chr1	82692143	82692222
chr1	82783679	82783691
chr1	83600125	83600201
chr1	83647800	83647867
chr1	84326285	84326554
chr1	84366279	84366478
chr1	84367599	84367668
chr1	84394953	84395152
chr1	84464514	84464713
chr1	84464820	84465129
chr1	84477762	84477805
chr1	84543067	84544231
chr1	84569060	84569212
chr1	84639889	84640359

chr1	84713068	84713296
chr1	84764032	84765081
chr1	84785323	84785377
chr1	84899423	84899692
chr1	84926025	84926180
chr1	84944771	84944853
chr1	84945076	84945600
chr1	84969543	84969730
chr1	84970889	84972647
chr1	85038648	85039016
chr1	85039512	85040389
chr1	85069537	85069598
chr1	85086222	85086991
chr1	85155680	85156331
chr1	85156567	85156802
chr1	85163866	85163979
chr1	85164332	85164449
chr1	85193461	85193697
chr1	85215283	85216306
chr1	85300071	85300529
chr1	85300897	85301151
chr1	85301750	85302146
chr1	85308352	85308984
chr1	85312781	85313069
chr1	85313186	85313904
chr1	85316139	85316432
chr1	85317259	85317647
chr1	85317861	85317986
chr1	85319371	85319681
chr1	85319947	85320106
chr1	85320365	85320482
chr1	85321091	85321255
chr1	85349227	85349364
chr1	85352997	85353277
chr1	85353491	85353656
chr1	85354312	85355149
chr1	85355422	85355484
chr1	85358178	85359346
chr1	85363269	85363683
chr1	85373161	85373367
chr1	85406250	85406489
chr1	85410316	85411132
chr1	85411669	85411975
chr1	85463059	85463792
chr1	85513559	85514529
chr1	85527526	85527895

chr1	85528054	85528229
chr1	85663378	85663585
chr1	85665758	85665774
chr1	85666284	85667965
chr1	85690994	85691133
chr1	85706310	85706368
chr1	85724500	85724534
chr1	85724696	85725307
chr1	85725515	85725689
chr1	85740866	85741244
chr1	85741487	85742189
chr1	85742699	85742875
chr1	85744503	85744642
chr1	85744882	85745255
chr1	85755090	85755245
chr1	85755509	85755807
chr1	85760570	85760664
chr1	85762715	85762821
chr1	85762964	85763003
chr1	85763409	85763723
chr1	85763880	85764296
chr1	85779634	85779789
chr1	85785975	85786634
chr1	85794592	85794619
chr1	85794823	85795116
chr1	85930071	85931354
chr1	85939649	85939721
chr1	85960485	85960601
chr1	85960800	85960927
chr1	85961092	85961225
chr1	85961665	85961674
chr1	85990270	85990442
chr1	85992346	85992395
chr1	85992810	85993049
chr1	85993263	85993266
chr1	85996451	85996636
chr1	86021829	86022025
chr1	86022313	86022315
chr1	86036959	86036984
chr1	86037345	86037393
chr1	86037461	86037544
chr1	86037840	86038055
chr1	86038616	86038680
chr1	86041930	86043242
chr1	86043527	86044662
chr1	86045433	86045637

chr1	86045837	86048540
chr1	86049589	86050599
chr1	86050700	86050837
chr1	86048615	86049065
chr1	86072594	86072651
chr1	86072940	86073492
chr1	86077494	86078046
chr1	86078469	86078933
chr1	86095009	86095189
chr1	86173489	86174070
chr1	86174191	86174453
chr1	86315857	86315939
chr1	86316256	86316372
chr1	86620186	86620367
chr1	86620854	86621123
chr1	86846180	86846301
chr1	86860838	86861300
chr1	86861482	86862304
chr1	86885219	86885386
chr1	86889550	86889696
chr1	86889829	86889971
chr1	86895477	86895548
chr1	86895715	86896092
chr1	86967795	86967826
chr1	86974256	86974340
chr1	86982987	86983056
chr1	87012723	87012864
chr1	87033616	87033686
chr1	87102530	87102650
chr1	87102763	87102997
chr1	87113903	87113982
chr1	87114145	87114331
chr1	87169684	87169904
chr1	87170052	87171448
chr1	87179825	87179985
chr1	87180145	87180614
chr1	87239244	87240092
chr1	87240296	87240533
chr1	87245024	87245591
chr1	87246753	87247054
chr1	87311753	87312163
chr1	87312390	87312407
chr1	87332641	87332697
chr1	87379189	87379445
chr1	87379576	87380070
chr1	87380305	87381596

chr1	87437919	87438235
chr1	87597618	87597863
chr1	87597972	87598219
chr1	87685195	87685335
chr1	87685510	87685669
chr1	87690203	87690315
chr1	87691586	87692074
chr1	87692228	87692533
chr1	87752967	87753390
chr1	87791990	87791999
chr1	87794263	87795304
chr1	87796568	87796621
chr1	87797029	87797863
chr1	87800217	87800409
chr1	87800484	87800739
chr1	87800802	87801357
chr1	87865240	87865529
chr1	87889443	87889510
chr1	87889787	87889922
chr1	88232759	88232820
chr1	88271915	88271985
chr1	88300522	88300632
chr1	88335319	88335451
chr1	89076182	89076297
chr1	89113277	89113336
chr1	89149205	89149684
chr1	89149928	89150924
chr1	89229063	89229212
chr1	89229399	89229598
chr1	89239187	89239630
chr1	89239767	89240311
chr1	89267480	89267613
chr1	89267726	89267917
chr1	89268651	89268973
chr1	89321731	89321766
chr1	89343660	89343911
chr1	89356605	89357237
chr1	89357403	89357709
chr1	89444411	89444452
chr1	89449746	89450159
chr1	89450756	89450848
chr1	89451472	89451784
chr1	89458043	89458576
chr1	89458820	89459103
chr1	89459346	89459677
chr1	89487460	89487806

chr1	89488056	89488727
chr1	89530799	89531518
chr1	89591694	89592060
chr1	89737546	89737683
chr1	89740042	89740381
chr1	89740568	89740640
chr1	89873196	89873288
chr1	89920448	89920823
chr1	89989431	89991082
chr1	90013155	90013364
chr1	90013646	90013700
chr1	90018180	90018486
chr1	90058405	90058755
chr1	90098291	90099987
chr1	90100115	90100149
chr1	90101572	90101849
chr1	90102000	90102403
chr1	90175547	90175549
chr1	90185382	90185610
chr1	90186930	90186942
chr1	90228763	90229253
chr1	90272962	90273077
chr1	90273764	90274091
chr1	90285946	90286426
chr1	90286574	90287506
chr1	90287907	90287956
chr1	90288570	90288921
chr1	90292198	90292206
chr1	90292499	90292583
chr1	90308104	90309966
chr1	90331485	90331988
chr1	90350689	90351032
chr1	90352843	90353237
chr1	90355516	90355796
chr1	90356020	90356281
chr1	90372456	90372817
chr1	90373297	90373414
chr1	90373592	90373694
chr1	90374638	90374847
chr1	90374978	90375066
chr1	90376348	90376353
chr1	90376580	90376757
chr1	90408889	90409260
chr1	90411922	90411993
chr1	90418754	90419205
chr1	90419991	90420086

chr1	90460150	90461303
chr1	90468623	90468973
chr1	90477898	90478105
chr1	90667189	90667412
chr1	91316126	91317166
chr1	91317337	91317522
chr1	91425197	91425311
chr1	91481906	91482114
chr1	91486705	91488313
chr1	91852749	91853177
chr1	91869946	91870547
chr1	91965956	91966190
chr1	91966396	91967215
chr1	91995433	91995486
chr1	91995632	91995823
chr1	92002922	92003520
chr1	92018648	92019046
chr1	92022556	92022900
chr1	92033073	92033265
chr1	92034995	92035495
chr1	92048107	92048138
chr1	92048447	92048543
chr1	92073461	92073636
chr1	92075212	92075537
chr1	92076923	92077469
chr1	92079178	92079327
chr1	92079531	92079540
chr1	92116406	92116605
chr1	92196332	92196379
chr1	92196552	92196751
chr1	92197144	92197185
chr1	92198383	92198579
chr1	92202454	92203020
chr1	92269722	92270379
chr1	92272491	92272519
chr1	92284851	92284937
chr1	92295569	92295630
chr1	92298130	92298267
chr1	92310391	92311069
chr1	92316808	92316903
chr1	92317055	92317270
chr1	92317412	92317658
chr1	92329689	92330410
chr1	92344229	92344320
chr1	92347746	92347866
chr1	92350326	92351699

chr1	92351823	92352115
chr1	92355297	92355754
chr1	92355887	92356024
chr1	92359715	92360324
chr1	92356410	92357309
chr1	92366132	92366541
chr1	92392661	92392926
chr1	92393111	92393184
chr1	92495661	92496119
chr1	92545687	92546535
chr1	92650530	92650718
chr1	92742707	92742775
chr1	92764050	92765044
chr1	92952283	92952535
chr1	93033005	93033041
chr1	93249583	93249724
chr1	93249860	93251316
chr1	93270213	93270386
chr1	93270489	93270761
chr1	93297203	93297402
chr1	93297586	93298106
chr1	93324502	93324556
chr1	93324790	93324829
chr1	93373530	93373929
chr1	93374796	93374904
chr1	93399023	93399129
chr1	93399278	93399324
chr1	93416096	93416389
chr1	93424734	93424835
chr1	93425804	93425999
chr1	93426252	93427910
chr1	93428395	93428416
chr1	93527771	93527910
chr1	93544407	93544646
chr1	93544876	93545089
chr1	93545264	93545647
chr1	93545980	93546322
chr1	93621064	93621312
chr1	93645047	93646779
chr1	93687433	93687601
chr1	93687802	93687810
chr1	93688902	93689162
chr1	93770250	93770294
chr1	93787924	93788084
chr1	93788278	93788291
chr1	93790426	93790792

chr1	93791004	93791480
chr1	93803909	93804397
chr1	93810856	93812570
chr1	93907849	93908243
chr1	93912320	93912791
chr1	93913260	93913924
chr1	93914055	93915330
chr1	93913086	93913160
chr1	93985230	93985461
chr1	94046397	94047121
chr1	94050561	94050596
chr1	94050993	94051480
chr1	94057259	94058076
chr1	94058519	94058545
chr1	94058745	94059550
chr1	94060821	94061177
chr1	94062549	94062810
chr1	94063205	94063384
chr1	94063685	94063960
chr1	94064081	94064405
chr1	94071805	94072004
chr1	94077743	94077911
chr1	94080093	94081842
chr1	94082205	94083457
chr1	94083643	94084029
chr1	94085941	94086288
chr1	94087563	94087837
chr1	94089982	94090069
chr1	94091498	94091776
chr1	94095029	94095194
chr1	94095412	94095458
chr1	94095909	94096164
chr1	94096319	94096443
chr1	94108448	94108576
chr1	94127693	94128010
chr1	94128862	94129061
chr1	94129797	94131317
chr1	94132255	94132363
chr1	94134302	94134516
chr1	94138106	94138166
chr1	94138668	94139040
chr1	94140080	94140273
chr1	94143134	94143356
chr1	94144982	94145285
chr1	94145709	94147899
chr1	94156828	94156932

chr1	94157121	94157274
chr1	94160306	94160808
chr1	94160875	94160998
chr1	94157750	94159988
chr1	94161174	94161231
chr1	94161512	94161810
chr1	94162242	94162441
chr1	94163310	94163434
chr1	94163539	94163619
chr1	94164163	94164562
chr1	94165194	94166100
chr1	94166499	94167332
chr1	94167456	94168754
chr1	94169227	94169858
chr1	94171621	94172081
chr1	94201357	94201601
chr1	94203605	94204382
chr1	94205195	94205431
chr1	94219302	94219569
chr1	94220175	94220442
chr1	94220743	94221175
chr1	94223926	94224206
chr1	94224838	94224911
chr1	94230125	94230280
chr1	94232296	94232305
chr1	94233486	94233504
chr1	94239081	94239339
chr1	94242475	94242698
chr1	94244662	94244886
chr1	94244978	94245239
chr1	94240674	94241204
chr1	94245372	94245882
chr1	94269964	94270321
chr1	94270827	94270847
chr1	94271046	94271107
chr1	94278547	94278965
chr1	94279510	94279644
chr1	94279791	94279977
chr1	94280204	94280435
chr1	94281335	94281544
chr1	94282443	94282855
chr1	94283853	94284155
chr1	94284656	94284961
chr1	94293350	94294337
chr1	94309765	94310581
chr1	94311259	94313075

chr1	94313225	94313832
chr1	94343644	94345051
chr1	94374176	94375246
chr1	94426430	94426475
chr1	94426652	94426728
chr1	94454991	94455079
chr1	94470522	94470702
chr1	94505027	94505034
chr1	94511554	94511807
chr1	94516372	94516806
chr1	94526937	94527034
chr1	94527287	94527407
chr1	94547203	94547566
chr1	94547740	94547886
chr1	94663816	94664015
chr1	94675354	94675378
chr1	94701890	94702424
chr1	94702607	94703564
chr1	94712102	94713622
chr1	94736168	94736622
chr1	94764538	94764672
chr1	94765500	94766134
chr1	94766966	94767221
chr1	94782993	94783230
chr1	94788300	94788684
chr1	94789522	94789910
chr1	94790788	94791821
chr1	94801401	94801803
chr1	94882899	94883260
chr1	94883350	94883869
chr1	94884043	94884888
chr1	94887959	94888058
chr1	94888162	94888310
chr1	94946935	94946952
chr1	94992876	94993152
chr1	94997748	94998020
chr1	95003840	95004190
chr1	95005050	95005154
chr1	95005270	95005592
chr1	95006513	95007462
chr1	95007746	95008240
chr1	95008634	95008964
chr1	95010737	95011017
chr1	95015582	95015731
chr1	95017901	95017999
chr1	95018865	95019125

chr1	95020916	95021143
chr1	95045609	95045868
chr1	95063261	95063310
chr1	95063659	95063713
chr1	95064467	95064863
chr1	95064996	95065380
chr1	95071511	95071674
chr1	95072167	95072315
chr1	95072991	95073335
chr1	95082952	95083327
chr1	95085871	95087452
chr1	95087579	95087778
chr1	95088088	95089637
chr1	95089874	95089966
chr1	95102201	95102522
chr1	95111663	95112201
chr1	95112406	95113041
chr1	95113680	95114481
chr1	95119522	95119614
chr1	95119728	95120015
chr1	95120798	95120997
chr1	95123302	95123566
chr1	95123873	95124135
chr1	95164407	95164716
chr1	95173916	95174352
chr1	95175398	95175659
chr1	95179153	95179722
chr1	95189347	95189510
chr1	95191147	95191254
chr1	95192739	95193102
chr1	95193345	95193687
chr1	95193867	95194233
chr1	95195021	95195181
chr1	95195962	95196270
chr1	95200585	95201499
chr1	95205360	95205624
chr1	95239068	95239301
chr1	95239364	95239467
chr1	95239676	95239741
chr1	95249601	95249873
chr1	95257993	95258117
chr1	95258263	95258402
chr1	95267484	95268133
chr1	95285342	95285356
chr1	95285442	95286351
chr1	95298225	95298320

chr1	95302205	95302560
chr1	95319889	95319950
chr1	95320152	95321050
chr1	95328117	95329068
chr1	95329461	95330005
chr1	95341235	95341463
chr1	95341787	95342313
chr1	95391031	95393369
chr1	95437105	95437272
chr1	95505313	95506000
chr1	95507124	95507209
chr1	95509195	95509325
chr1	95538000	95538800
chr1	95539153	95539200
chr1	95582786	95583553
chr1	95632966	95633047
chr1	95635151	95635262
chr1	95699046	95699534
chr1	95699692	95700233
chr1	95700456	95700555
chr1	96196861	96196872
chr1	96208575	96208770
chr1	96541554	96541604
chr1	96757023	96757369
chr1	96775335	96775494
chr1	96828047	96828070
chr1	96828413	96828534
chr1	96836343	96836475
chr1	96836601	96836837
chr1	96881564	96882076
chr1	97025543	97026191
chr1	97186815	97188097
chr1	97432326	97432451
chr1	97547972	97548087
chr1	97559971	97560170
chr1	97743394	97743932
chr1	97955431	97955544
chr1	97955680	97955749
chr1	98047017	98047426
chr1	98315535	98315615
chr1	98366754	98366822
chr1	98370104	98370503
chr1	98385950	98386489
chr1	98403452	98403858
chr1	98510984	98511411
chr1	98518978	98519818

chr1	98520031	98520131
chr1	98552255	98552287
chr1	98742878	98743003
chr1	98788896	98789289
chr1	98789381	98789979
chr1	98791557	98791712
chr1	98791837	98792097
chr1	98817046	98817075
chr1	98817216	98817439
chr1	99126920	99128221
chr1	99142800	99142938
chr1	99143176	99143349
chr1	99205301	99205746
chr1	100045867	100045956
chr1	100046268	100046522
chr1	100086796	100087455
chr1	100111275	100111478
chr1	100125492	100125716
chr1	100128361	100128481
chr1	100229542	100229875
chr1	100231869	100232438
chr1	100274232	100274324
chr1	100280310	100280399
chr1	100315174	100316087
chr1	100408963	100408986
chr1	100428574	100428928
chr1	100434783	100435408
chr1	100435524	100436268
chr1	100437787	100438334
chr1	100502346	100502916
chr1	100503362	100504558
chr1	100512894	100513244
chr1	100596292	100596573
chr1	100598009	100598965
chr1	100714915	100715815
chr1	100731481	100731677
chr1	100731789	100732520
chr1	100816744	100817478
chr1	100817614	100817806
chr1	100817942	100819044
chr1	100967647	100968001
chr1	101360146	101361541
chr1	101361690	101362568
chr1	101470004	101470015
chr1	101490747	101491096
chr1	101491162	101492007

chr1	101616251	101616310
chr1	101653247	101653404
chr1	101666346	101666472
chr1	101701678	101701967
chr1	101702511	101703059
chr1	101704199	101704455
chr1	101725366	101725597
chr1	101775032	101775344
chr1	102223540	102223835
chr1	103093925	103094040
chr1	103094144	103094262
chr1	103345173	103345194
chr1	103572712	103572853
chr1	104067779	104067991
chr1	104068254	104069107
chr1	104186554	104186885
chr1	104250612	104250641
chr1	104607139	104607235
chr1	105630536	105630615
chr1	106797313	106797360
chr1	107158186	107158292
chr1	107158433	107158439
chr1	107541029	107541068
chr1	107599073	107599211
chr1	107599363	107600575
chr1	107682282	107682345
chr1	107682565	107682764
chr1	107682880	107683108
chr1	107683266	107683359
chr1	107683435	107684005
chr1	107684406	107684715
chr1	107805674	107805781
chr1	107973326	107973466
chr1	108072176	108072304
chr1	108072424	108072556
chr1	108073190	108073564
chr1	108073639	108074117
chr1	108074930	108074944
chr1	108075114	108075189
chr1	108078892	108079243
chr1	108087033	108087157
chr1	108090125	108090734
chr1	108113402	108113601
chr1	108115236	108115298
chr1	108115493	108115924
chr1	108136325	108136406

chr1	108220318	108220743
chr1	108225118	108225340
chr1	108225697	108226081
chr1	108228421	108228499
chr1	108228946	108229152
chr1	108230928	108231182
chr1	108231313	108231430
chr1	108231818	108232206
chr1	108291234	108291438
chr1	108293077	108293339
chr1	108419785	108419886
chr1	108479086	108479093
chr1	108479752	108479870
chr1	108507002	108507231
chr1	108507740	108508339
chr1	108550155	108550329
chr1	108559050	108559225
chr1	108583857	108583974
chr1	108588658	108588734
chr1	108600218	108600232
chr1	108600490	108600569
chr1	108650307	108650375
chr1	108725242	108725426
chr1	108741874	108742174
chr1	108742321	108743561
chr1	108760485	108760736
chr1	108815068	108815258
chr1	108815382	108815867
chr1	108963587	108964535
chr1	109203119	109203386
chr1	109203539	109204271
chr1	109234448	109234769
chr1	109234959	109236336
chr1	109246839	109246911
chr1	109247127	109247285
chr1	109262771	109262970
chr1	109263777	109264314
chr1	109288904	109289094
chr1	109289328	109290263
chr1	109358916	109359209
chr1	109370767	109371191
chr1	109371451	109371520
chr1	109373066	109373317
chr1	109388581	109389030
chr1	109419279	109419467
chr1	109419620	109420008

chr1	109422149	109422379
chr1	109423586	109423750
chr1	109430882	109431070
chr1	109438994	109439095
chr1	109462694	109462941
chr1	109504247	109504397
chr1	109505262	109506104
chr1	109506222	109506355
chr1	109570754	109571457
chr1	109576286	109576511
chr1	109579088	109579192
chr1	109583545	109584560
chr1	109584951	109585192
chr1	109593015	109593594
chr1	109617191	109617507
chr1	109618401	109618570
chr1	109618828	109618958
chr1	109627441	109627640
chr1	109629427	109629473
chr1	109632065	109632260
chr1	109632654	109634078
chr1	109635786	109635871
chr1	109642384	109642690
chr1	109642821	109644017
chr1	109645479	109645692
chr1	109655748	109656251
chr1	109656388	109656670
chr1	109706238	109706303
chr1	109739467	109741735
chr1	109744346	109744436
chr1	109751657	109752081
chr1	109752469	109752861
chr1	109753001	109753250
chr1	109756079	109756385
chr1	109757450	109757827
chr1	109759352	109759681
chr1	109756539	109757222
chr1	109768170	109768373
chr1	109768664	109769261
chr1	109781662	109782678
chr1	109782898	109783959
chr1	109784434	109784437
chr1	109785424	109785597
chr1	109792299	109792940
chr1	109799928	109800264
chr1	109804980	109805179

chr1	109805587	109806550
chr1	109806786	109806985
chr1	109809390	109809867
chr1	109809029	109809113
chr1	109815697	109815896
chr1	109816024	109816277
chr1	109816438	109818209
chr1	109819182	109819527
chr1	109819777	109820173
chr1	109824448	109826535
chr1	109839214	109839342
chr1	109940071	109940315
chr1	109940677	109941122
chr1	109967097	109967751
chr1	109968483	109969023
chr1	109969234	109969659
chr1	110009190	110009411
chr1	110026587	110027817
chr1	110036669	110037603
chr1	110051883	110052346
chr1	110052937	110053200
chr1	110075115	110075547
chr1	110088995	110089312
chr1	110089581	110089924
chr1	110090530	110090657
chr1	110090864	110091796
chr1	110105852	110106123
chr1	110110916	110111037
chr1	110113361	110113546
chr1	110138171	110138247
chr1	110138355	110138520
chr1	110153805	110154045
chr1	110161384	110161537
chr1	110161692	110161781
chr1	110162044	110163939
chr1	110185875	110186087
chr1	110198186	110198907
chr1	110282260	110282277
chr1	110282574	110282946
chr1	110283156	110283471
chr1	110316047	110316436
chr1	110319860	110319897
chr1	110320094	110320192
chr1	110334002	110334201
chr1	110334535	110335316
chr1	110363657	110363785

chr1	110364359	110364599
chr1	110414976	110415266
chr1	110419791	110420002
chr1	110420261	110420551
chr1	110422043	110422174
chr1	110435083	110435756
chr1	110435921	110436354
chr1	110450202	110450286
chr1	110452696	110454725
chr1	110469172	110469508
chr1	110470227	110470685
chr1	110471109	110471932
chr1	110472162	110472472
chr1	110518285	110518468
chr1	110520988	110521187
chr1	110522480	110522739
chr1	110523045	110523349
chr1	110527300	110528006
chr1	110546349	110546717
chr1	110546872	110547154
chr1	110572494	110572557
chr1	110576907	110577009
chr1	110577216	110578123
chr1	110751754	110752225
chr1	110752517	110754772
chr1	110771282	110771803
chr1	110772397	110773117
chr1	110778670	110779033
chr1	110786060	110786536
chr1	110802401	110802493
chr1	110802768	110802831
chr1	110880263	110882460
chr1	110882855	110883114
chr1	110949825	110950754
chr1	110951271	110951314
chr1	111019146	111019149
chr1	111459506	111459652
chr1	111505637	111506373
chr1	111506826	111507087
chr1	111682116	111683562
chr1	111707102	111707141
chr1	111745165	111746079
chr1	111746146	111747576
chr1	111757379	111757464
chr1	111765400	111765656
chr1	111989843	111990048

chr1	111991007	111991104
chr1	111991296	111991824
chr1	111992070	111992500
chr1	112050060	112050949
chr1	112051191	112051487
chr1	112084363	112085214
chr1	112135406	112135452
chr1	112161902	112161940
chr1	112162065	112162115
chr1	112162420	112163608
chr1	112247363	112247377
chr1	112247667	112247829
chr1	112250513	112250905
chr1	112257747	112257786
chr1	112281036	112282081
chr1	112282274	112282695
chr1	112283426	112283535
chr1	112297589	112297945
chr1	112298053	112299073
chr1	112862262	112862654
chr1	112901665	112902011
chr1	112902763	112902967
chr1	112933373	112933418
chr1	112933639	112934305
chr1	112936352	112937557
chr1	112938500	112939203
chr1	112939288	112939590
chr1	112946980	112947209
chr1	113003937	113004159
chr1	113004710	113004916
chr1	113006964	113007457
chr1	113007665	113007921
chr1	113008280	113008372
chr1	113008945	113009207
chr1	113030343	113030370
chr1	113044580	113045185
chr1	113050678	113052133
chr1	113086602	113087001
chr1	113118266	113118431
chr1	113148309	113148896
chr1	113157323	113157429
chr1	113160788	113161064
chr1	113161132	113161979
chr1	113162083	113162767
chr1	113170179	113170688
chr1	113179941	113180281

chr1	113180897	113181384
chr1	113188656	113189288
chr1	113211187	113211592
chr1	113216203	113218399
chr1	113229176	113229407
chr1	113229927	113230985
chr1	113231510	113231674
chr1	113232640	113232818
chr1	113232934	113233307
chr1	113235294	113235473
chr1	113239905	113240240
chr1	113241381	113241580
chr1	113242246	113243525
chr1	113243752	113244364
chr1	113246701	113247780
chr1	113247955	113248391
chr1	113248472	113250569
chr1	113250858	113251057
chr1	113251208	113251331
chr1	113256809	113257927
chr1	113258342	113258541
chr1	113258719	113259019
chr1	113261416	113261762
chr1	113275510	113275640
chr1	113278321	113278480
chr1	113278728	113278837
chr1	113278907	113279111
chr1	113286502	113287034
chr1	113305059	113306253
chr1	113349432	113349502
chr1	113349722	113350108
chr1	113351727	113352315
chr1	113378406	113378417
chr1	113378610	113378844
chr1	113380322	113380521
chr1	113380866	113381347
chr1	113383241	113383446
chr1	113383735	113383934
chr1	113384926	113385120
chr1	113387861	113388225
chr1	113389465	113389694
chr1	113391352	113391526
chr1	113394319	113395070
chr1	113391917	113394120
chr1	113395189	113395363
chr1	113397062	113397129

chr1	113397260	113397714
chr1	113405931	113405975
chr1	113408690	113410214
chr1	113413626	113413825
chr1	113416763	113417520
chr1	113417965	113418165
chr1	113423738	113423994
chr1	113424246	113424367
chr1	113430568	113430819
chr1	113446388	113447157
chr1	113447563	113447967
chr1	113448133	113448476
chr1	113448652	113448692
chr1	113472621	113472798
chr1	113477954	113478591
chr1	113487503	113487583
chr1	113487752	113487898
chr1	113497811	113499949
chr1	113500069	113500589
chr1	113500843	113501314
chr1	113520679	113521132
chr1	113523555	113523747
chr1	113531025	113531380
chr1	113532895	113533343
chr1	113534373	113534566
chr1	113534670	113534797
chr1	113536581	113536946
chr1	113537141	113537356
chr1	113538583	113538784
chr1	113540459	113540627
chr1	113564036	113564777
chr1	113576657	113576978
chr1	113577638	113577822
chr1	113577930	113578122
chr1	113585066	113585350
chr1	113585677	113585888
chr1	113615168	113616507
chr1	113625610	113625702
chr1	113643836	113643963
chr1	113850384	113851381
chr1	113866470	113866800
chr1	113867595	113868000
chr1	113869789	113869910
chr1	113931439	113931751
chr1	113931975	113933767
chr1	113934128	113934327

chr1	113934726	113934744
chr1	113939184	113939243
chr1	113999543	113999654
chr1	113999921	113999968
chr1	114084711	114084778
chr1	114085148	114085149
chr1	114195384	114195502
chr1	114195654	114195820
chr1	114285485	114285534
chr1	114290033	114290065
chr1	114300621	114300966
chr1	114301420	114302500
chr1	114347405	114347977
chr1	114354129	114354328
chr1	114354529	114354913
chr1	114355269	114355414
chr1	114403593	114403792
chr1	114446950	114447343
chr1	114447992	114448477
chr1	114448930	114449020
chr1	114449230	114449355
chr1	114471579	114471957
chr1	114472217	114472948
chr1	114473523	114473619
chr1	114521634	114521975
chr1	114879072	114879278
chr1	114888783	114889177
chr1	115016010	115016376
chr1	115052582	115053794
chr1	115054479	115054922
chr1	115112229	115112377
chr1	115124025	115124270
chr1	115124467	115124578
chr1	115152738	115152808
chr1	115153083	115153880
chr1	115161259	115161571
chr1	115211704	115211796
chr1	115212318	115212722
chr1	115212891	115213111
chr1	115214340	115214923
chr1	115213491	115213581
chr1	115253853	115254129
chr1	115258853	115259408
chr1	115259633	115259713
chr1	115261764	115261867
chr1	115299480	115300974

chr1	115322944	115323623
chr1	115345295	115345818
chr1	115347265	115347302
chr1	115347551	115347601
chr1	115351437	115351981
chr1	115397299	115397551
chr1	115594301	115594341
chr1	115594783	115594939
chr1	115595015	115595363
chr1	115605620	115605766
chr1	115631611	115631724
chr1	115631886	115632077
chr1	115632253	115632464
chr1	115632736	115632826
chr1	115660756	115660918
chr1	115677152	115677316
chr1	115681754	115681994
chr1	115700678	115701206
chr1	115710584	115711044
chr1	115711207	115711407
chr1	115711665	115711848
chr1	115727434	115727470
chr1	115732558	115732629
chr1	115732863	115733201
chr1	115752865	115753015
chr1	115765120	115765726
chr1	115765811	115766092
chr1	115767277	115767638
chr1	115794449	115794794
chr1	115836765	115836923
chr1	115842792	115843213
chr1	115843391	115843748
chr1	115844845	115845022
chr1	115845142	115845641
chr1	115851621	115851705
chr1	115851894	115851971
chr1	115857394	115857810
chr1	115863263	115863525
chr1	115865974	115866200
chr1	115873063	115873675
chr1	115875413	115876308
chr1	115879858	115881095
chr1	115882467	115882667
chr1	115954165	115954199
chr1	115973367	115973430
chr1	115975140	115975191

chr1	116012279	116012315
chr1	116012613	116013143
chr1	116035842	116035896
chr1	116039754	116039890
chr1	116088055	116088455
chr1	116089048	116089187
chr1	116106842	116106891
chr1	116184207	116185848
chr1	116197047	116197236
chr1	116197546	116198221
chr1	116211783	116212035
chr1	116314320	116314529
chr1	116324010	116324028
chr1	116324201	116324389
chr1	116325048	116326257
chr1	116350552	116350725
chr1	116372253	116372355
chr1	116379924	116382574
chr1	116417490	116417892
chr1	116420017	116420116
chr1	116518432	116519837
chr1	116520092	116520634
chr1	116523765	116523775
chr1	116544382	116544522
chr1	116544691	116545044
chr1	116576092	116576193
chr1	116652175	116653028
chr1	116653136	116653265
chr1	116674461	116674892
chr1	116675514	116675826
chr1	116681566	116681726
chr1	116694509	116694708
chr1	116694882	116695178
chr1	116710531	116710870
chr1	116710934	116711398
chr1	116810828	116810861
chr1	116851702	116851845
chr1	116852025	116852093
chr1	116854495	116855243
chr1	116883134	116883882
chr1	116901954	116901986
chr1	116915255	116917020
chr1	116917279	116917909
chr1	116918812	116919060
chr1	116919382	116919496
chr1	116920202	116920863

chr1	116952824	116952901
chr1	116960578	116960675
chr1	116960976	116961564
chr1	117038637	117038756
chr1	117038954	117039075
chr1	117039325	117039524
chr1	117047295	117047462
chr1	117052143	117052342
chr1	117057830	117057863
chr1	117079907	117080045
chr1	117080269	117080569
chr1	117095956	117096714
chr1	117098381	117098730
chr1	117099188	117099407
chr1	117099520	117099669
chr1	117112130	117112384
chr1	117112521	117114415
chr1	117133307	117133345
chr1	117161861	117162033
chr1	117163322	117163522
chr1	117164669	117164753
chr1	117165267	117165672
chr1	117168479	117168599
chr1	117184883	117185238
chr1	117185305	117185547
chr1	117189189	117189572
chr1	117209332	117210591
chr1	117223324	117223426
chr1	117223676	117223889
chr1	117230080	117230229
chr1	117230333	117230427
chr1	117230541	117230542
chr1	117236936	117237291
chr1	117238908	117239898
chr1	117262773	117262918
chr1	117262996	117263051
chr1	117265087	117265229
chr1	117265466	117265541
chr1	117281656	117282008
chr1	117282127	117282128
chr1	117283932	117284006
chr1	117308895	117308969
chr1	117313345	117313546
chr1	117315283	117315416
chr1	117315633	117315706
chr1	117342137	117342321

chr1	117351110	117351803
chr1	117369837	117370288
chr1	117390858	117390867
chr1	117396254	117396821
chr1	117416557	117416736
chr1	117420729	117421833
chr1	117422027	117422339
chr1	117424548	117425528
chr1	117424036	117424394
chr1	117429072	117429237
chr1	117429388	117429581
chr1	117449381	117449930
chr1	117450002	117450333
chr1	117452016	117453482
chr1	117453780	117455030
chr1	117456239	117456393
chr1	117457738	117457833
chr1	117458559	117458946
chr1	117459096	117459871
chr1	117461630	117462086
chr1	117465529	117465825
chr1	117470150	117470403
chr1	117501449	117501598
chr1	117515471	117515689
chr1	117549903	117550217
chr1	117594022	117594095
chr1	117601937	117602573
chr1	117603519	117603692
chr1	117610654	117610838
chr1	117635649	117636264
chr1	117636492	117636926
chr1	117653374	117653432
chr1	117663954	117665198
chr1	117670414	117670431
chr1	117689285	117689384
chr1	117900984	117901085
chr1	117909071	117909991
chr1	117910099	117910612
chr1	118147944	118148392
chr1	118296301	118296892
chr1	118471413	118471436
chr1	118471617	118472882
chr1	118475491	118475558
chr1	118510269	118510470
chr1	118664862	118664885
chr1	118690151	118690627

chr1	118727987	118728253
chr1	118752165	118752227
chr1	118752548	118752748
chr1	119028576	119028801
chr1	119544403	119544780
chr1	119550817	119551125
chr1	119682679	119684072
chr1	119796872	119797148
chr1	119807792	119807844
chr1	119818274	119818366
chr1	119822239	119822437
chr1	119901689	119901848
chr1	120156339	120156501
chr1	120188523	120188922
chr1	120189345	120191254
chr1	120206071	120206155
chr1	120216633	120217091
chr1	120217404	120217527
chr1	120217704	120217855
chr1	120217934	120218622
chr1	120219169	120219245
chr1	120254238	120255625
chr1	120255861	120256138
chr1	120460104	120460307
chr1	120472600	120472781
chr1	120516624	120516682
chr1	120530059	120530121
chr1	120540219	120540394
chr1	120558439	120558570
chr1	120564737	120565123
chr1	120567527	120567737
chr1	120569787	120569931
chr1	120574554	120574720
chr1	120579422	120579843
chr1	120587387	120588130
chr1	120589813	120590030
chr1	120593435	120593853
chr1	120594910	120595296
chr1	120596873	120597337
chr1	120601399	120601931
chr1	120602178	120602482
chr1	120604084	120604174
chr1	120604291	120605315
chr1	120606430	120606727
chr1	120607008	120607403
chr1	120608958	120609176

chr1	120610747	120612298
chr1	120612634	120613003
chr1	120617536	120617973
chr1	120623361	120623487
chr1	120648900	120649105
chr1	120669386	120669556
chr1	120687161	120687186
chr1	120687396	120687591
chr1	120688391	120688720
chr1	120753698	120754001
chr1	120754329	120754360
chr1	120759092	120759099
chr1	120759375	120759453
chr1	120759542	120759761
chr1	120760742	120761111
chr1	120761614	120761640
chr1	120762862	120763140
chr1	120765801	120766088
chr1	120796579	120796696
chr1	120835654	120837785
chr1	120837961	120838232
chr1	120838925	120840096
chr1	120866171	120866410
chr1	120925676	120926208
chr1	121117168	121117306
chr1	121118478	121118548
chr1	121131789	121132261
chr1	121137732	121138969
chr1	121143542	121144035
chr1	121156779	121157130
chr1	121159126	121159538
chr1	121160109	121160560
chr1	121260667	121261735
chr1	121285738	121285883
chr1	121286022	121286139
chr1	121315666	121315701
chr1	121351766	121352909
chr1	121353029	121353388
chr1	121353508	121355368
chr1	121355586	121358216
chr1	121358386	121358646
chr1	121358782	121359242
chr1	121359788	121360486
chr1	121361286	121361410
chr1	121362642	121363018
chr1	121364178	121364426

chr1	121365795	121366015
chr1	121366673	121366958
chr1	121367422	121367555
chr1	121377628	121377862
chr1	121386296	121386512
chr1	121386583	121386613
chr1	121394714	121394752
chr1	121395368	121395591
chr1	121398807	121399041
chr1	121403929	121404024
chr1	121405212	121405438
chr1	121426723	121426984
chr1	121430342	121430449
chr1	121467992	121468238
chr1	121471621	121471834
chr1	121478324	121479511
chr1	121479861	121480360
chr1	121480774	121482479
chr1	121482615	121485592
chr1	142535556	142536238
chr1	142536519	142536916
chr1	142537168	142539651
chr1	142539983	142540234
chr1	142540479	142541109
chr1	142542212	142542503
chr1	142601178	142601422
chr1	142784303	142784345
chr1	142786760	142787054
chr1	142787624	142787707
chr1	142788951	142789401
chr1	142794140	142794359
chr1	142808576	142808792
chr1	142816880	142817112
chr1	142817617	142817787
chr1	142894244	142894497
chr1	142967024	142967134
chr1	143228607	143228815
chr1	143249047	143249115
chr1	143278631	143278922
chr1	143280327	143280549
chr1	143281663	143281983
chr1	143283170	143283377
chr1	143451605	143451839
chr1	143467081	143467592
chr1	143467894	143468007
chr1	143645763	143646121

chr1	143646699	143646898
chr1	143647256	143647455
chr1	143672635	143672879
chr1	143673239	143673839
chr1	143686866	143687468
chr1	143708761	143709300
chr1	143730547	143730823
chr1	143742789	143745095
chr1	143745303	143745781
chr1	143879699	143879817
chr1	143879971	143880301
chr1	143884786	143885108
chr1	143885857	143886715
chr1	143911982	143913135
chr1	143913592	143915774
chr1	143916000	143916212
chr1	143916423	143916476
chr1	143917320	143917511
chr1	143989020	143989278
chr1	143990924	143991354
chr1	143992232	143993107
chr1	143997528	143997592
chr1	143997861	143998058
chr1	144001018	144001224
chr1	144001988	144002085
chr1	144005821	144006136
chr1	144006524	144006680
chr1	144007400	144007693
chr1	144008958	144008961
chr1	144009689	144009888
chr1	144010093	144011434
chr1	144011679	144011890
chr1	144012023	144013199
chr1	144013713	144014542
chr1	144014971	144015126
chr1	144015594	144015823
chr1	144016116	144016208
chr1	144018884	144019210
chr1	144028441	144028592
chr1	144028827	144028950
chr1	144029748	144030121
chr1	144030821	144031037
chr1	144031141	144031340
chr1	144044692	144044895
chr1	144068966	144069055
chr1	144069563	144069835

chr1	144070189	144070412
chr1	144072310	144072517
chr1	144072849	144072941
chr1	144311334	144311621
chr1	144312789	144313038
chr1	144339256	144340122
chr1	144340277	144340762
chr1	144340940	144341462
chr1	144341669	144341843
chr1	144491629	144492100
chr1	144519627	144520649
chr1	144520804	144521709
chr1	144521776	144521917
chr1	144527883	144528082
chr1	144529212	144531239
chr1	144531651	144531819
chr1	144532335	144532384
chr1	144532687	144534883
chr1	144535130	144535633
chr1	144535990	144537372
chr1	144537822	144538096
chr1	144538332	144538549
chr1	144539006	144539667
chr1	144545345	144545668
chr1	144549107	144549330
chr1	144551029	144551228
chr1	144565956	144566212
chr1	144567480	144568717
chr1	144572298	144572702
chr1	144572858	144573151
chr1	144573429	144573751
chr1	144587616	144587881
chr1	144589972	144590032
chr1	144591036	144591267
chr1	144593331	144594627
chr1	144594973	144595390
chr1	144595876	144596098
chr1	144596179	144596554
chr1	144598050	144598235
chr1	144681391	144681590
chr1	144686274	144686536
chr1	144689766	144690486
chr1	144691464	144691606
chr1	144692848	144692897
chr1	144694681	144694877
chr1	144706611	144707221

chr1	144707439	144707750
chr1	144707862	144709101
chr1	144832660	144832724
chr1	144858280	144858479
chr1	144864144	144864412
chr1	144884010	144884209
chr1	144884944	144885092
chr1	144885790	144885817
chr1	144891601	144892461
chr1	144892867	144892885
chr1	144893017	144893066
chr1	144908639	144909147
chr1	144914998	144915404
chr1	144918104	144918377
chr1	144930440	144933398
chr1	144941156	144941683
chr1	144965708	144965931
chr1	144968947	144969295
chr1	144969833	144970176
chr1	144982165	144984513
chr1	144986774	144987090
chr1	144987769	144988040
chr1	144989358	144989916
chr1	144991114	144992236
chr1	144992716	144993284
chr1	144993418	144994166
chr1	144995155	144995504
chr1	144995849	144995922
chr1	144996028	144996357
chr1	144996887	144997189
chr1	144999904	145000104
chr1	145004351	145004513
chr1	145005383	145005395
chr1	145005562	145005761
chr1	145007500	145007811
chr1	145007938	145007991
chr1	145009436	145009915
chr1	145012593	145012842
chr1	145014448	145014609
chr1	145015018	145015860
chr1	145016120	145016403
chr1	145016807	145016824
chr1	145017444	145017877
chr1	145020190	145020547
chr1	145022738	145022785
chr1	145022915	145022937

chr1	145024088	145024397
chr1	145026478	145026514
chr1	145029286	145029303
chr1	145032191	145032803
chr1	145033281	145033534
chr1	145033710	145034123
chr1	145034279	145034522
chr1	145034724	145034902
chr1	145037447	145037698
chr1	145037904	145038286
chr1	145038958	145040492
chr1	145041108	145041502
chr1	145042313	145043616
chr1	145043749	145044120
chr1	145045067	145045206
chr1	145045859	145045979
chr1	145048889	145049075
chr1	145056224	145056438
chr1	145057150	145057200
chr1	145057328	145057962
chr1	145058079	145058092
chr1	145058625	145058844
chr1	145059218	145059587
chr1	145060256	145060455
chr1	145061358	145061557
chr1	145061766	145062472
chr1	145062977	145063185
chr1	145063992	145064142
chr1	145064972	145065002
chr1	145067230	145067432
chr1	145068490	145068536
chr1	145068820	145069091
chr1	145072274	145072496
chr1	145073088	145073766
chr1	145075091	145076251
chr1	145078566	145078709
chr1	145081280	145081375
chr1	145083384	145084252
chr1	145090067	145090639
chr1	145091479	145091750
chr1	145091838	145092911
chr1	145096239	145096686
chr1	145096956	145097343
chr1	145097616	145097848
chr1	145100721	145100834
chr1	145110767	145110777

chr1	145110894	145110966
chr1	145113557	145113783
chr1	145114019	145114882
chr1	145117637	145117836
chr1	145127933	145128345
chr1	145130000	145130129
chr1	145133668	145133859
chr1	145138817	145138912
chr1	145139120	145140080
chr1	145148439	145148566
chr1	145149674	145149675
chr1	145149964	145150025
chr1	145170625	145170805
chr1	145172817	145173057
chr1	145173256	145173518
chr1	145189118	145189147
chr1	145196136	145196307
chr1	145204530	145204566
chr1	145208435	145210509
chr1	145212035	145212437
chr1	145213185	145213500
chr1	145214074	145214314
chr1	145214730	145215184
chr1	145215784	145216261
chr1	145216476	145217328
chr1	145218992	145220045
chr1	145224098	145224801
chr1	145226122	145226536
chr1	145227574	145227991
chr1	145233500	145234064
chr1	145241503	145241823
chr1	145243494	145244042
chr1	145246217	145246614
chr1	145247082	145247224
chr1	145255403	145255667
chr1	145259003	145259217
chr1	145271651	145271771
chr1	145273316	145273548
chr1	145278194	145278435
chr1	145286668	145286816
chr1	145288384	145288544
chr1	145289659	145290340
chr1	145291198	145291318
chr1	145292335	145292367
chr1	145292448	145292880
chr1	145293271	145294014

chr1	145294142	145294350
chr1	145299679	145299962
chr1	145300673	145300750
chr1	145301078	145301125
chr1	145313968	145314264
chr1	145342361	145342602
chr1	145350013	145350016
chr1	145352000	145352026
chr1	145352238	145352510
chr1	145361625	145361743
chr1	145374398	145374580
chr1	145381378	145383164
chr1	145385997	145386202
chr1	145395291	145395513
chr1	145395726	145396137
chr1	145396262	145396807
chr1	145396959	145397856
chr1	145397972	145398078
chr1	145398290	145399273
chr1	145399382	145399927
chr1	145421710	145421894
chr1	145427662	145427848
chr1	145433480	145433818
chr1	145437585	145438466
chr1	145442222	145442469
chr1	145443716	145443989
chr1	145448159	145448358
chr1	145448584	145449912
chr1	145454656	145455409
chr1	145455584	145456337
chr1	145457239	145457635
chr1	145460798	145460902
chr1	145470063	145470951
chr1	145471046	145472359
chr1	145472498	145473444
chr1	145476696	145476921
chr1	145477096	145477812
chr1	145505619	145505680
chr1	145506555	145506762
chr1	145507335	145508076
chr1	145515278	145516904
chr1	145524270	145524487
chr1	145524857	145525156
chr1	145542546	145543105
chr1	145543252	145543477
chr1	145543569	145543861

chr1	145574906	145575078
chr1	145575253	145576821
chr1	145589011	145589407
chr1	145589522	145589748
chr1	145610499	145611623
chr1	145612131	145612323
chr1	145616890	145616981
chr1	145621759	145621781
chr1	145622086	145622088
chr1	145652211	145652390
chr1	145652524	145652716
chr1	145682914	145683138
chr1	145697946	145698050
chr1	145713796	145714142
chr1	145728233	145728433
chr1	145728682	145728926
chr1	145746258	145746439
chr1	145750230	145750261
chr1	145758517	145758859
chr1	145826331	145826347
chr1	145826476	145827023
chr1	145827366	145827419
chr1	145945193	145945414
chr1	145955633	145955883
chr1	145963014	145963099
chr1	145963290	145963958
chr1	145968318	145969263
chr1	145969406	145970235
chr1	145979079	145980116
chr1	145991012	145991777
chr1	146008444	146008701
chr1	146008860	146009107
chr1	146009642	146009895
chr1	146081885	146082528
chr1	146082969	146082981
chr1	146373348	146374479
chr1	146374893	146374997
chr1	146518557	146518661
chr1	146518800	146518922
chr1	146555612	146555971
chr1	146556034	146557107
chr1	146633879	146634062
chr1	146638916	146639085
chr1	146639204	146639428
chr1	146640338	146640661
chr1	146642719	146643072

chr1	146643460	146644776
chr1	146649742	146649780
chr1	146697043	146697387
chr1	146713976	146714217
chr1	146714324	146714747
chr1	146714860	146715059
chr1	146736272	146736435
chr1	146744655	146744932
chr1	146745065	146745391
chr1	147070802	147071859
chr1	147072038	147072454
chr1	147141485	147141614
chr1	147141771	147143021
chr1	147193745	147193974
chr1	147194237	147194348
chr1	147229325	147229467
chr1	147229644	147230282
chr1	147232328	147232750
chr1	147232875	147233052
chr1	147233229	147233411
chr1	147362339	147362538
chr1	147400213	147400291
chr1	147400633	147401043
chr1	147401204	147401279
chr1	147497545	147497632
chr1	147504795	147504856
chr1	147505008	147506023
chr1	147510033	147511900
chr1	147520896	147521757
chr1	147532646	147533506
chr1	147533980	147534144
chr1	147551521	147551823
chr1	147623355	147623426
chr1	147623767	147624380
chr1	147624570	147624804
chr1	147727503	147727555
chr1	147728699	147728993
chr1	147729961	147730821
chr1	147731907	147731951
chr1	147735214	147735789
chr1	147735916	147736684
chr1	147736915	147737461
chr1	147749951	147750598
chr1	147751170	147751578
chr1	147752284	147752662
chr1	147752871	147753685

chr1	147774703	147775833
chr1	147776104	147776303
chr1	147777119	147777260
chr1	147777363	147777425
chr1	147792213	147792356
chr1	147805986	147807446
chr1	147824950	147825018
chr1	147833481	147833760
chr1	147833889	147834286
chr1	147834452	147834495
chr1	147850336	147850535
chr1	147860081	147861056
chr1	147874344	147874904
chr1	147896342	147896484
chr1	147930423	147930932
chr1	147931960	147932642
chr1	147932841	147932980
chr1	147931083	147931766
chr1	147993572	147994717
chr1	147999196	147999760
chr1	147999832	148001185
chr1	148006871	148007140
chr1	148007385	148007392
chr1	148008682	148008871
chr1	148009549	148009870
chr1	148010898	148010986
chr1	148013546	148013806
chr1	148177028	148177509
chr1	148176147	148176936
chr1	148193097	148193313
chr1	148231726	148231789
chr1	148240852	148241978
chr1	148242050	148242421
chr1	148247104	148248025
chr1	148248212	148248423
chr1	148248501	148248700
chr1	148301584	148301716
chr1	148522356	148522494
chr1	148526896	148527030
chr1	148527310	148527565
chr1	148527832	148528036
chr1	148528633	148528790
chr1	148530204	148530608
chr1	148531270	148532839
chr1	148534352	148534610
chr1	148537545	148537964

chr1	148541559	148541686
chr1	148548592	148548808
chr1	148554062	148554345
chr1	148555566	148557016
chr1	148557256	148557668
chr1	148595443	148595541
chr1	148597798	148599547
chr1	148599831	148600030
chr1	148604246	148605002
chr1	148605503	148605610
chr1	148683247	148683402
chr1	148760148	148760341
chr1	148760920	148761714
chr1	148766462	148766671
chr1	148767134	148767355
chr1	148767545	148767680
chr1	148852756	148852955
chr1	148853870	148854783
chr1	148855109	148855308
chr1	148877983	148878375
chr1	148881098	148881297
chr1	148893252	148893396
chr1	148899313	148899494
chr1	148900138	148900398
chr1	148912589	148913383
chr1	148913632	148913643
chr1	148915451	148915490
chr1	148915570	148915837
chr1	148927553	148932252
chr1	148932412	148932941
chr1	148933339	148933441
chr1	148935473	148935679
chr1	148939620	148940001
chr1	148940169	148940631
chr1	148941245	148941444
chr1	148942262	148942461
chr1	148942532	148942987
chr1	148943121	148944059
chr1	148945402	148945588
chr1	148948727	148948926
chr1	149034799	149035567
chr1	149222138	149222343
chr1	149222406	149223995
chr1	149224221	149225088
chr1	149286975	149287298
chr1	149290561	149290760

chr1	149293742	149294072
chr1	149294260	149294567
chr1	149297965	149298102
chr1	149298254	149298302
chr1	149298692	149298891
chr1	149299088	149299350
chr1	149304533	149304743
chr1	149336037	149336201
chr1	149379254	149379938
chr1	149439279	149439958
chr1	149513777	149513919
chr1	149514685	149514818
chr1	149576008	149576221
chr1	149576355	149577268
chr1	149577469	149577953
chr1	149603045	149603196
chr1	149605318	149605707
chr1	149605809	149606105
chr1	149658793	149658857
chr1	149679588	149679943
chr1	149684155	149684487
chr1	149684602	149684809
chr1	149743026	149743054
chr1	149764173	149764339
chr1	149764428	149765160
chr1	149765323	149765522
chr1	149782904	149783792
chr1	149783985	149784286
chr1	149784507	149784752
chr1	149785295	149785429
chr1	149802442	149802817
chr1	149802963	149804112
chr1	149804349	149804856
chr1	149804972	149805579
chr1	149806155	149806206
chr1	149809284	149809750
chr1	149810261	149810497
chr1	149811105	149811356
chr1	149811603	149812153
chr1	149812473	149812756
chr1	149812954	149814275
chr1	149814504	149815229
chr1	149815745	149816054
chr1	149819038	149819385
chr1	149819989	149820137
chr1	149821757	149822519

chr1	149822636	149823854
chr1	149824145	149825361
chr1	149825510	149825868
chr1	149826590	149827090
chr1	149827204	149827659
chr1	149830677	149830941
chr1	149831230	149831253
chr1	149832194	149832639
chr1	149831385	149832117
chr1	149832837	149833660
chr1	149834155	149834515
chr1	149856799	149857106
chr1	149857250	149858149
chr1	149858491	149860517
chr1	149860824	149861075
chr1	149871125	149872537
chr1	149872981	149873356
chr1	149889205	149889473
chr1	149899057	149899343
chr1	149899457	149900446
chr1	149903372	149903385
chr1	149904736	149904931
chr1	149907634	149907654
chr1	149908263	149909388
chr1	149909583	149910079
chr1	149910414	149910632
chr1	149910885	149911773
chr1	149934291	149934421
chr1	149935602	149935606
chr1	149967845	149968373
chr1	149981786	149981897
chr1	149982037	149983032
chr1	149993959	149994383
chr1	150022572	150022917
chr1	150038891	150040033
chr1	150121773	150121986
chr1	150122214	150122413
chr1	150122806	150123078
chr1	150155856	150156341
chr1	150156452	150156556
chr1	150158289	150159385
chr1	150185762	150185886
chr1	150186064	150186388
chr1	150206788	150207199
chr1	150207365	150207754
chr1	150208098	150208483

chr1	150209307	150209674
chr1	150208662	150209128
chr1	150228000	150228251
chr1	150231670	150231886
chr1	150232090	150232289
chr1	150232436	150233248
chr1	150234809	150235073
chr1	150235236	150235835
chr1	150240593	150241241
chr1	150241600	150241943
chr1	150242861	150243060
chr1	150253673	150254529
chr1	150260206	150260326
chr1	150266003	150266487
chr1	150266590	150266803
chr1	150286229	150286329
chr1	150286701	150287019
chr1	150293643	150294435
chr1	150294753	150294859
chr1	150335984	150337631
chr1	150337718	150338079
chr1	150445367	150445633
chr1	150450103	150450319
chr1	150450439	150450652
chr1	150451500	150451633
chr1	150455270	150455303
chr1	150459547	150460077
chr1	150478948	150479156
chr1	150480719	150480943
chr1	150487500	150488045
chr1	150488333	150488609
chr1	150488693	150489020
chr1	150489132	150489250
chr1	150489451	150489645
chr1	150509616	150511168
chr1	150511408	150511614
chr1	150511950	150511967
chr1	150520210	150520313
chr1	150521139	150522217
chr1	150524164	150524787
chr1	150524927	150525356
chr1	150533001	150536571
chr1	150539051	150539148
chr1	150540166	150542707
chr1	150542786	150543161
chr1	150544283	150544579

chr1	150544673	150545267
chr1	150545492	150545747
chr1	150547206	150547405
chr1	150550562	150552086
chr1	150552299	150552722
chr1	150564967	150565397
chr1	150574517	150574733
chr1	150577172	150577632
chr1	150577725	150578295
chr1	150580575	150580580
chr1	150583766	150584598
chr1	150585475	150585911
chr1	150586060	150587087
chr1	150588102	150588236
chr1	150592311	150592490
chr1	150596529	150596563
chr1	150596854	150597152
chr1	150598721	150599271
chr1	150601459	150601566
chr1	150601682	150603504
chr1	150669402	150669669
chr1	150669929	150670120
chr1	150721641	150721834
chr1	150737014	150737422
chr1	150737555	150737589
chr1	150738369	150738568
chr1	150782104	150782218
chr1	150785591	150785898
chr1	150842530	150842543
chr1	150848305	150849125
chr1	150849388	150849608
chr1	150851739	150851938
chr1	150854480	150854523
chr1	150868043	150868157
chr1	150895165	150895421
chr1	150898482	150898598
chr1	150898779	150899304
chr1	150937431	150937630
chr1	150943121	150943233
chr1	150943401	150943622
chr1	150945106	150945396
chr1	150945772	150947775
chr1	150949331	150949639
chr1	150949705	150949810
chr1	150949959	150950252
chr1	150952405	150952648

chr1	150954371	150954570
chr1	150973967	150974092
chr1	150976618	150976681
chr1	150978647	150978894
chr1	150979116	150979448
chr1	150981037	150981558
chr1	151020007	151021023
chr1	151021152	151021473
chr1	151022067	151022188
chr1	151025454	151025736
chr1	151025909	151026266
chr1	151028665	151029006
chr1	151029086	151029285
chr1	151027462	151028367
chr1	151030244	151030580
chr1	151030970	151031260
chr1	151031384	151031538
chr1	151031737	151033008
chr1	151033582	151033810
chr1	151034902	151035392
chr1	151035514	151035672
chr1	151042495	151043876
chr1	151070010	151070309
chr1	151080063	151080215
chr1	151103478	151103677
chr1	151104307	151104886
chr1	151105206	151105475
chr1	151118643	151120002
chr1	151136080	151136307
chr1	151137468	151138406
chr1	151138614	151139192
chr1	151144537	151144580
chr1	151145151	151145207
chr1	151162020	151162986
chr1	151163809	151164020
chr1	151164920	151165160
chr1	151167353	151169200
chr1	151170251	151170832
chr1	151171203	151171623
chr1	151172170	151172451
chr1	151172531	151173140
chr1	151174076	151174427
chr1	151178165	151178257
chr1	151183411	151183450
chr1	151183671	151183840
chr1	151201603	151201680

chr1	151210942	151211141
chr1	151224101	151224263
chr1	151226493	151226582
chr1	151226862	151227699
chr1	151227826	151228025
chr1	151253212	151253704
chr1	151253950	151254406
chr1	151254599	151255449
chr1	151255616	151255950
chr1	151256724	151256977
chr1	151257994	151258371
chr1	151284359	151284492
chr1	151299229	151300931
chr1	151313350	151313472
chr1	151319305	151320240
chr1	151371418	151371876
chr1	151372051	151372724
chr1	151373780	151373906
chr1	151416695	151416744
chr1	151429987	151431020
chr1	151431520	151431836
chr1	151432032	151432146
chr1	151445847	151446215
chr1	151467045	151467244
chr1	151483282	151483783
chr1	151483952	151484677
chr1	151484995	151486692
chr1	151488409	151488829
chr1	151488943	151489265
chr1	151509181	151509433
chr1	151509504	151509598
chr1	151509842	151509989
chr1	151511445	151511951
chr1	151512330	151513953
chr1	151515094	151515206
chr1	151518084	151518304
chr1	151519495	151519670
chr1	151519963	151521050
chr1	151521154	151521583
chr1	151525332	151525344
chr1	151525513	151525794
chr1	151529228	151529437
chr1	151531168	151531432
chr1	151542388	151542627
chr1	151542736	151542828
chr1	151555517	151555758

chr1	151557279	151557342
chr1	151558057	151558130
chr1	151558425	151558511
chr1	151559104	151559539
chr1	151566578	151566702
chr1	151566920	151567042
chr1	151578850	151578997
chr1	151579367	151579386
chr1	151580718	151580889
chr1	151581209	151581221
chr1	151583637	151584123
chr1	151584191	151585541
chr1	151652545	151653651
chr1	151712710	151712853
chr1	151735464	151736260
chr1	151740783	151741422
chr1	151742558	151742658
chr1	151746194	151746261
chr1	151746442	151746620
chr1	151762380	151763063
chr1	151763296	151764150
chr1	151801301	151801527
chr1	151804792	151804998
chr1	151805397	151805543
chr1	151807978	151808177
chr1	151808690	151808968
chr1	151809084	151809453
chr1	151809671	151813352
chr1	151829587	151829600
chr1	151840202	151840790
chr1	151859462	151860023
chr1	151881637	151882666
chr1	151913779	151913942
chr1	151918136	151918967
chr1	151919129	151919296
chr1	151940876	151940892
chr1	151943094	151943279
chr1	151943447	151943545
chr1	151944994	151945567
chr1	151945710	151945819
chr1	151949298	151949408
chr1	151953812	151953868
chr1	151960767	151963038
chr1	151964240	151967198
chr1	151967403	151967563
chr1	151968605	151968662

chr1	151971393	151971874
chr1	151973634	151974253
chr1	151974395	151974617
chr1	151986369	151986478
chr1	151996616	151996697
chr1	152001648	152002138
chr1	152006925	152007939
chr1	152008126	152008372
chr1	152008671	152009760
chr1	152010046	152010245
chr1	152019600	152021342
chr1	152021490	152021836
chr1	152022229	152022532
chr1	152023561	152023913
chr1	152024133	152024186
chr1	152024991	152025170
chr1	152080405	152080604
chr1	152081803	152082323
chr1	152082609	152082643
chr1	152082735	152082880
chr1	152083139	152083478
chr1	152083722	152084366
chr1	152084570	152084887
chr1	152085045	152085092
chr1	152085202	152085397
chr1	152114668	152114814
chr1	152117154	152117397
chr1	152135234	152135669
chr1	152137311	152137576
chr1	152140221	152141005
chr1	152155319	152156015
chr1	152158062	152158218
chr1	152159537	152159645
chr1	152161025	152162318
chr1	152162546	152162622
chr1	152162940	152163858
chr1	152164016	152164407
chr1	152359595	152359794
chr1	152365310	152365348
chr1	152413238	152413285
chr1	152432713	152432911
chr1	152460355	152461024
chr1	152464089	152464153
chr1	152474149	152474163
chr1	152474473	152474638
chr1	152474993	152475388

chr1	152475542	152475706
chr1	152477401	152477452
chr1	152477950	152478083
chr1	152479437	152479755
chr1	152489748	152490147
chr1	152552849	152553089
chr1	152553246	152553339
chr1	152690692	152691167
chr1	152717822	152717987
chr1	152732563	152732836
chr1	152843763	152844045
chr1	152852842	152852967
chr1	152877756	152878087
chr1	152880721	152880743
chr1	152901166	152901229
chr1	152901443	152901458
chr1	152926586	152926684
chr1	152926782	152926960
chr1	152945267	152945394
chr1	153005330	153005514
chr1	153005780	153005932
chr1	153018938	153019061
chr1	153019222	153019327
chr1	153072681	153073220
chr1	153095260	153095996
chr1	153147106	153147263
chr1	153147429	153147451
chr1	153195758	153195957
chr1	153329352	153329525
chr1	153329868	153330525
chr1	153332125	153332368
chr1	153336241	153336475
chr1	153336605	153336735
chr1	153336915	153337057
chr1	153337623	153337793
chr1	153338393	153338608
chr1	153338055	153338278
chr1	153347856	153347929
chr1	153348230	153348413
chr1	153359022	153359106
chr1	153461593	153461668
chr1	153462643	153462747
chr1	153463039	153463334
chr1	153463536	153463995
chr1	153465809	153465824
chr1	153481547	153481690

chr1	153483346	153483816
chr1	153485668	153485751
chr1	153487851	153488513
chr1	153488837	153488842
chr1	153489087	153489322
chr1	153490616	153490815
chr1	153497856	153499070
chr1	153499400	153499659
chr1	153502712	153502993
chr1	153505249	153507155
chr1	153507609	153508489
chr1	153508709	153509284
chr1	153510442	153510660
chr1	153511275	153511385
chr1	153514419	153514634
chr1	153517040	153517286
chr1	153517380	153517896
chr1	153518105	153518718
chr1	153521140	153521809
chr1	153522029	153522629
chr1	153523427	153523442
chr1	153530692	153531007
chr1	153532772	153533178
chr1	153533723	153533874
chr1	153535131	153535582
chr1	153535844	153541721
chr1	153534472	153534770
chr1	153541828	153541854
chr1	153544786	153544985
chr1	153545238	153545486
chr1	153549518	153549556
chr1	153549786	153550003
chr1	153550191	153550390
chr1	153580572	153581181
chr1	153581254	153583611
chr1	153583764	153584031
chr1	153584165	153585540
chr1	153585896	153586216
chr1	153586964	153589749
chr1	153586800	153586850
chr1	153589927	153590294
chr1	153598234	153599121
chr1	153599278	153600506
chr1	153605345	153605474
chr1	153606552	153607144
chr1	153607378	153607591

chr1	153625587	153626046
chr1	153630877	153630940
chr1	153631053	153631817
chr1	153631997	153632249
chr1	153632878	153632967
chr1	153633082	153633142
chr1	153638698	153638889
chr1	153643259	153643400
chr1	153643746	153644806
chr1	153650670	153651271
chr1	153651510	153652150
chr1	153677958	153678172
chr1	153678308	153678365
chr1	153680502	153680767
chr1	153680995	153682026
chr1	153699765	153700130
chr1	153700542	153701295
chr1	153701453	153701517
chr1	153703576	153703729
chr1	153703844	153704043
chr1	153704467	153705366
chr1	153747254	153748678
chr1	153755621	153756189
chr1	153756388	153756517
chr1	153760778	153761079
chr1	153763435	153763778
chr1	153764637	153764898
chr1	153769982	153770292
chr1	153868997	153869350
chr1	153874475	153874571
chr1	153875087	153875314
chr1	153877046	153877250
chr1	153878235	153878324
chr1	153881481	153881649
chr1	153893116	153893479
chr1	153894059	153895520
chr1	153895700	153896737
chr1	153917923	153919210
chr1	153929712	153929922
chr1	153930410	153931520
chr1	153933506	153933599
chr1	153934255	153937333
chr1	153938203	153938761
chr1	153938840	153939220
chr1	153939546	153939783
chr1	153939890	153941377

chr1	153943694	153943726
chr1	153943804	153944084
chr1	153948130	153948162
chr1	153948315	153948392
chr1	153949183	153949382
chr1	153949456	153951127
chr1	153956961	153957239
chr1	153957643	153959310
chr1	153959495	153960069
chr1	153962906	153963665
chr1	153965955	153966058
chr1	153987336	153987351
chr1	154126116	154126263
chr1	154148938	154149251
chr1	154152087	154152257
chr1	154154583	154156174
chr1	154157534	154157733
chr1	154168460	154168530
chr1	154169137	154169524
chr1	154169665	154169836
chr1	154185793	154185914
chr1	154187446	154187582
chr1	154189933	154190256
chr1	154192570	154193164
chr1	154193377	154194317
chr1	154244106	154244369
chr1	154244565	154244665
chr1	154244786	154245683
chr1	154247068	154247156
chr1	154293229	154293249
chr1	154297311	154298954
chr1	154306248	154306361
chr1	154319605	154319709
chr1	154363537	154363931
chr1	154377065	154377447
chr1	154377769	154379185
chr1	154379550	154380490
chr1	154383867	154384215
chr1	154384322	154384405
chr1	154389863	154390066
chr1	154390265	154393280
chr1	154393368	154393513
chr1	154393773	154394238
chr1	154394451	154395196
chr1	154395312	154395529
chr1	154398456	154398657

chr1	154399552	154399763
chr1	154402620	154404196
chr1	154404347	154405754
chr1	154406058	154406281
chr1	154409507	154409878
chr1	154426339	154426432
chr1	154447215	154447392
chr1	154448923	154449038
chr1	154452322	154452397
chr1	154529715	154531054
chr1	154531447	154531800
chr1	154531902	154532094
chr1	154576376	154576434
chr1	154579609	154579904
chr1	154580311	154580979
chr1	154581104	154581460
chr1	154581757	154582186
chr1	154594971	154595348
chr1	154599507	154600626
chr1	154686630	154686845
chr1	154701489	154701639
chr1	154702969	154703152
chr1	154880614	154880627
chr1	154904417	154905079
chr1	154905175	154905436
chr1	154908504	154910418
chr1	154911404	154911495
chr1	154910525	154911007
chr1	154927888	154928090
chr1	154928205	154930113
chr1	154932979	154935036
chr1	154939361	154940119
chr1	154940376	154940711
chr1	154940953	154942368
chr1	154946022	154946984
chr1	154942519	154945775
chr1	154947124	154947745
chr1	154948009	154948451
chr1	154952408	154952654
chr1	154954909	154955213
chr1	154955321	154955502
chr1	154955813	154957117
chr1	154959540	154959791
chr1	154960123	154960426
chr1	154963995	154964297
chr1	154966793	154966858

chr1	154967590	154968596
chr1	154968962	154969161
chr1	154969320	154969360
chr1	154971145	154973077
chr1	154973226	154975003
chr1	154975184	154977696
chr1	154977846	154979791
chr1	154980644	154981130
chr1	154983796	154984325
chr1	154986481	154986680
chr1	154989367	154990701
chr1	154993695	154993768
chr1	154994391	154994644
chr1	154994836	154995302
chr1	154995553	154995758
chr1	154997683	154998220
chr1	155011209	155011441
chr1	155017595	155017907
chr1	155020705	155021075
chr1	155021321	155021549
chr1	155022292	155025059
chr1	155025285	155026033
chr1	155036319	155037011
chr1	155043171	155044130
chr1	155045914	155046113
chr1	155051266	155051588
chr1	155051780	155053843
chr1	155054080	155054753
chr1	155054985	155055292
chr1	155057091	155058686
chr1	155085096	155085330
chr1	155085671	155085684
chr1	155086533	155086550
chr1	155096541	155097045
chr1	155097437	155097965
chr1	155098149	155100867
chr1	155101122	155102820
chr1	155103634	155104108
chr1	155104306	155104783
chr1	155107751	155109882
chr1	155111016	155111266
chr1	155111641	155112314
chr1	155112441	155113289
chr1	155140721	155141096
chr1	155144937	155147125
chr1	155158364	155158575

chr1	155161290	155161632
chr1	155162105	155162304
chr1	155162836	155164669
chr1	155165352	155165576
chr1	155176438	155176781
chr1	155177756	155179414
chr1	155190272	155190774
chr1	155191933	155192160
chr1	155194459	155194631
chr1	155195072	155195195
chr1	155195619	155196002
chr1	155196292	155197072
chr1	155197462	155197979
chr1	155202033	155202461
chr1	155204825	155205024
chr1	155206632	155206885
chr1	155210663	155211021
chr1	155211171	155211265
chr1	155212768	155212817
chr1	155212983	155215415
chr1	155223233	155225746
chr1	155231387	155232123
chr1	155232312	155232553
chr1	155242555	155243675
chr1	155247222	155247608
chr1	155255986	155256065
chr1	155256149	155256185
chr1	155265128	155265879
chr1	155276027	155276134
chr1	155277310	155277398
chr1	155277888	155278477
chr1	155278598	155280072
chr1	155282558	155282830
chr1	155284334	155284441
chr1	155292549	155295403
chr1	155386416	155386545
chr1	155404656	155404881
chr1	155481291	155481544
chr1	155489676	155489845
chr1	155497622	155497788
chr1	155516227	155516479
chr1	155517522	155517620
chr1	155518068	155518082
chr1	155529071	155529273
chr1	155533842	155534155
chr1	155534729	155535008

chr1	155531522	155533368
chr1	155536469	155536665
chr1	155578685	155578891
chr1	155579245	155579816
chr1	155580063	155580806
chr1	155607287	155607622
chr1	155618009	155618381
chr1	155618630	155618757
chr1	155620951	155621460
chr1	155621594	155621654
chr1	155657509	155659150
chr1	155674016	155674168
chr1	155675902	155675966
chr1	155676152	155676239
chr1	155706168	155706330
chr1	155715126	155715423
chr1	155715614	155716231
chr1	155730521	155731233
chr1	155730073	155730448
chr1	155733081	155733288
chr1	155733401	155734198
chr1	155746412	155746638
chr1	155826655	155827374
chr1	155828618	155829083
chr1	155833650	155833666
chr1	155833897	155834011
chr1	155843753	155843763
chr1	155862341	155863086
chr1	155863327	155863904
chr1	155866016	155866236
chr1	155879782	155879880
chr1	155880918	155882476
chr1	155889793	155889982
chr1	155893880	155894090
chr1	155901135	155901528
chr1	155901862	155901922
chr1	155902184	155902332
chr1	155902500	155902699
chr1	155903922	155904575
chr1	155908470	155908941
chr1	155910221	155910605
chr1	155911168	155911367
chr1	155931203	155931467
chr1	155931558	155931691
chr1	155932803	155932832
chr1	155932996	155933415

chr1	155934486	155934575
chr1	155940291	155940718
chr1	155942658	155942849
chr1	155945156	155945845
chr1	155946010	155946691
chr1	155947088	155949351
chr1	155951671	155951870
chr1	155952980	155953751
chr1	155954450	155954541
chr1	155954864	155954943
chr1	155958850	155959000
chr1	155968517	155968667
chr1	155973041	155973253
chr1	155973368	155973685
chr1	155979333	155979439
chr1	155990125	155990361
chr1	155990986	155991019
chr1	156022787	156025378
chr1	156030621	156031239
chr1	156031840	156032329
chr1	156032444	156032715
chr1	156049174	156049373
chr1	156051683	156052951
chr1	156060877	156060967
chr1	156064493	156064667
chr1	156065617	156065687
chr1	156065809	156065980
chr1	156066099	156067594
chr1	156069610	156069630
chr1	156069975	156070620
chr1	156071113	156071359
chr1	156072172	156072517
chr1	156072790	156073993
chr1	156074134	156074512
chr1	156074667	156075033
chr1	156075146	156075582
chr1	156075657	156076846
chr1	156078092	156078291
chr1	156082885	156084420
chr1	156084551	156088162
chr1	156088267	156088590
chr1	156088673	156089466
chr1	156092234	156094152
chr1	156094412	156097385
chr1	156097553	156097935
chr1	156098022	156098800

chr1	156089599	156092167
chr1	156098969	156101443
chr1	156102585	156102916
chr1	156115902	156116942
chr1	156122710	156123769
chr1	156130649	156130848
chr1	156131110	156131395
chr1	156132156	156132355
chr1	156135649	156135947
chr1	156163272	156163765
chr1	156163873	156164181
chr1	156176346	156176545
chr1	156182488	156183538
chr1	156183659	156183992
chr1	156184075	156184611
chr1	156186063	156186112
chr1	156186241	156186645
chr1	156187369	156188077
chr1	156188413	156188551
chr1	156191015	156191148
chr1	156195614	156195626
chr1	156203521	156203865
chr1	156228652	156228887
chr1	156231117	156231184
chr1	156231755	156231976
chr1	156232166	156232284
chr1	156245798	156245853
chr1	156246786	156246952
chr1	156247057	156247294
chr1	156251538	156252631
chr1	156252790	156253192
chr1	156254192	156254687
chr1	156255029	156255182
chr1	156254819	156254938
chr1	156264849	156265419
chr1	156265588	156265624
chr1	156271543	156271957
chr1	156279475	156279541
chr1	156280770	156280797
chr1	156281160	156281202
chr1	156290287	156290491
chr1	156306906	156307272
chr1	156307790	156308051
chr1	156308360	156308820
chr1	156329733	156329913
chr1	156367170	156367272

chr1	156367859	156368321
chr1	156368660	156368684
chr1	156368935	156368968
chr1	156373507	156373533
chr1	156426074	156426361
chr1	156426518	156427778
chr1	156428011	156428028
chr1	156431659	156433644
chr1	156433873	156434165
chr1	156434345	156434577
chr1	156435097	156435360
chr1	156436153	156436383
chr1	156439431	156439543
chr1	156459720	156459908
chr1	156460128	156460356
chr1	156467995	156468177
chr1	156468726	156469457
chr1	156469845	156471716
chr1	156471912	156472269
chr1	156472391	156473557
chr1	156473746	156473945
chr1	156474034	156474306
chr1	156474564	156475821
chr1	156487468	156488014
chr1	156541034	156541083
chr1	156541283	156541453
chr1	156542126	156542651
chr1	156546887	156546956
chr1	156548885	156548944
chr1	156549008	156549084
chr1	156557672	156558066
chr1	156560967	156562403
chr1	156569810	156569945
chr1	156571396	156572496
chr1	156572616	156572777
chr1	156579149	156579233
chr1	156594192	156594502
chr1	156629728	156629929
chr1	156630284	156630483
chr1	156630713	156631197
chr1	156631768	156631999
chr1	156654433	156654632
chr1	156654893	156655021
chr1	156655752	156656542
chr1	156659649	156660115
chr1	156661667	156661866

chr1	156674581	156677061
chr1	156683065	156683432
chr1	156695263	156695728
chr1	156695926	156696678
chr1	156696791	156697116
chr1	156697554	156698550
chr1	156697240	156697247
chr1	156698832	156699180
chr1	156701071	156701239
chr1	156710209	156710900
chr1	156711260	156711633
chr1	156711758	156711806
chr1	156712284	156712386
chr1	156714831	156715064
chr1	156716162	156720511
chr1	156720672	156722030
chr1	156715238	156716005
chr1	156725812	156725817
chr1	156732406	156732502
chr1	156736067	156736942
chr1	156737303	156738329
chr1	156782514	156782611
chr1	156783108	156786548
chr1	156786767	156786899
chr1	156861185	156861195
chr1	156861449	156862090
chr1	156862312	156862935
chr1	156865719	156866527
chr1	156866738	156867267
chr1	156867378	156868534
chr1	156877068	156877267
chr1	156889239	156889306
chr1	157014895	157016438
chr1	157102370	157102513
chr1	157106890	157106933
chr1	157107265	157107281
chr1	157107492	157109037
chr1	157125675	157125883
chr1	157126426	157126453
chr1	157126592	157126808
chr1	157133655	157133856
chr1	157933441	157933531
chr1	157938487	157938865
chr1	157939041	157939177
chr1	157939491	157939549
chr1	157962157	157962418

chr1	157962657	157965395
chr1	157970699	157970763
chr1	157970974	157971055
chr1	157971455	157971541
chr1	157978344	157978543
chr1	157979396	157979903
chr1	157980079	157981097
chr1	157981401	157981729
chr1	157986415	157987158
chr1	157988874	157989704
chr1	157989806	157990147
chr1	157991518	157991932
chr1	157992286	157992644
chr1	157998681	157998992
chr1	158005602	158005861
chr1	158006664	158007636
chr1	158008256	158008455
chr1	158007788	158008092
chr1	158083066	158083590
chr1	158095108	158095192
chr1	158098973	158099079
chr1	158099233	158099380
chr1	158115756	158116652
chr1	158116767	158117008
chr1	158119396	158119499
chr1	158147491	158147708
chr1	158150193	158150392
chr1	158150461	158150502
chr1	158150638	158151148
chr1	158968339	158968342
chr1	158969407	158969651
chr1	158969764	158970119
chr1	158970436	158970585
chr1	158979522	158979741
chr1	158979853	158980071
chr1	158982292	158982365
chr1	158982574	158982665
chr1	158985224	158985698
chr1	159061069	159061281
chr1	159061519	159062556
chr1	159231547	159231667
chr1	159231786	159231920
chr1	159567968	159568011
chr1	159568401	159568469
chr1	159721800	159721877
chr1	159749376	159749467

chr1	159750212	159750334
chr1	159750749	159751587
chr1	159756721	159756817
chr1	159787038	159787488
chr1	159787903	159788058
chr1	159818618	159819118
chr1	159824076	159825192
chr1	159849685	159850150
chr1	159850243	159850442
chr1	159851572	159852127
chr1	159852229	159852584
chr1	159859521	159860021
chr1	159861249	159861448
chr1	159869881	159870315
chr1	159870571	159870898
chr1	159880063	159880105
chr1	159882636	159882686
chr1	159885386	159885585
chr1	159885718	159886540
chr1	159886671	159886956
chr1	159887397	159887543
chr1	159892432	159895617
chr1	159895887	159896539
chr1	159902537	159903017
chr1	159903097	159903218
chr1	159906948	159907147
chr1	159913619	159913785
chr1	159913899	159914235
chr1	159914462	159914796
chr1	159915069	159915071
chr1	159915201	159916090
chr1	159930349	159930818
chr1	160000692	160001714
chr1	160001886	160002422
chr1	160012662	160012727
chr1	160013035	160013123
chr1	160053678	160054069
chr1	160067525	160068413
chr1	160070718	160070998
chr1	160094408	160094603
chr1	160094960	160095159
chr1	160174041	160174544
chr1	160174960	160176452
chr1	160187271	160187513
chr1	160231352	160232583
chr1	160232706	160232909

chr1	160253992	160254348
chr1	160254592	160254924
chr1	160255064	160255174
chr1	160312817	160313073
chr1	160313198	160313630
chr1	160368710	160368840
chr1	160370356	160371348
chr1	160377081	160378861
chr1	160381344	160381543
chr1	160384002	160384059
chr1	160384565	160384687
chr1	160384910	160385484
chr1	160644918	160645046
chr1	160922212	160922519
chr1	160960091	160960201
chr1	160963794	160964015
chr1	160965256	160965437
chr1	160966162	160966449
chr1	160967589	160967833
chr1	160973128	160973312
chr1	160973486	160973973
chr1	160976590	160976786
chr1	160983223	160983650
chr1	160983889	160984425
chr1	160984910	160985151
chr1	160985990	160986756
chr1	160986893	160987092
chr1	160987774	160988056
chr1	160989774	160991020
chr1	160989005	160989353
chr1	160991288	160992709
chr1	160995120	160995220
chr1	161003476	161003891
chr1	161007695	161008782
chr1	161014664	161016216
chr1	161043132	161045808
chr1	161050479	161051479
chr1	161051675	161051693
chr1	161052180	161052200
chr1	161052354	161054221
chr1	161054329	161054568
chr1	161055450	161055539
chr1	161056044	161056243
chr1	161058654	161059620
chr1	161059708	161059868
chr1	161064822	161064965

chr1	161084884	161085169
chr1	161085333	161085604
chr1	161089437	161089502
chr1	161087169	161088282
chr1	161101472	161103317
chr1	161123517	161124390
chr1	161128649	161129176
chr1	161129287	161129759
chr1	161130014	161130056
chr1	161130392	161130513
chr1	161135351	161135679
chr1	161135863	161136063
chr1	161136638	161137157
chr1	161146808	161147842
chr1	161151776	161152060
chr1	161168531	161168791
chr1	161171094	161171971
chr1	161172170	161172868
chr1	161179991	161180015
chr1	161195189	161195298
chr1	161195971	161196586
chr1	161196864	161197115
chr1	161197694	161197929
chr1	161231139	161231338
chr1	161275459	161276161
chr1	161283876	161284920
chr1	161351945	161352325
chr1	161352447	161352809
chr1	161353508	161353736
chr1	161359321	161359836
chr1	161360015	161360386
chr1	161360489	161360574
chr1	161368907	161368952
chr1	161369136	161369467
chr1	161369612	161369993
chr1	161391908	161392325
chr1	161409698	161410137
chr1	161410274	161410625
chr1	161410787	161411194
chr1	161411495	161412125
chr1	161412302	161412927
chr1	161417089	161417431
chr1	161417563	161419964
chr1	161420624	161420899
chr1	161423231	161423430
chr1	161424045	161426228

chr1	161426352	161426995
chr1	161431881	161435083
chr1	161438904	161439577
chr1	161439729	161441970
chr1	161442220	161442427
chr1	161450862	161451542
chr1	161493860	161494280
chr1	161499905	161500153
chr1	161500312	161500609
chr1	161500940	161501176
chr1	161509587	161509985
chr1	161582401	161582826
chr1	161591840	161592039
chr1	161617375	161617459
chr1	161695477	161696339
chr1	161696538	161697443
chr1	161699770	161699983
chr1	161703390	161703741
chr1	161706387	161706701
chr1	161708552	161708594
chr1	161719258	161719374
chr1	161719555	161720020
chr1	161734989	161735095
chr1	161735227	161735388
chr1	161735808	161735936
chr1	161736083	161736439
chr1	161762115	161762424
chr1	161762516	161762567
chr1	161762780	161762920
chr1	161875127	161875147
chr1	161932354	161932433
chr1	161932612	161932679
chr1	161993412	161993916
chr1	161994351	161994407
chr1	162006753	162006928
chr1	162029856	162030079
chr1	162039244	162040020
chr1	162111483	162111542
chr1	162319525	162319697
chr1	162320006	162320330
chr1	162341847	162341884
chr1	162342032	162342046
chr1	162467381	162468252
chr1	162469050	162469250
chr1	162508554	162509338
chr1	162511387	162511644

chr1	162511907	162512057
chr1	162514856	162515491
chr1	162530705	162531061
chr1	162531288	162532490
chr1	162532616	162532684
chr1	162575218	162575419
chr1	162575534	162575640
chr1	162666548	162666601
chr1	162760034	162761091
chr1	162792107	162792307
chr1	162883165	162883598
chr1	163291332	163291415
chr1	163291793	163292169
chr1	164756734	164756986
chr1	164757416	164757532
chr1	165345290	165345597
chr1	165567076	165567853
chr1	165571054	165571274
chr1	165599722	165599824
chr1	165600062	165601727
chr1	165613838	165613967
chr1	165665140	165665173
chr1	165665514	165665538
chr1	165665853	165666126
chr1	165666997	165668192
chr1	165737790	165738085
chr1	165738332	165738802
chr1	165742739	165742999
chr1	165796523	165796603
chr1	165796812	165797902
chr1	165798025	165798558
chr1	165811844	165811906
chr1	165820180	165820297
chr1	165820591	165820656
chr1	165823391	165823590
chr1	165826429	165826662
chr1	165852260	165852373
chr1	165865260	165865550
chr1	165865874	165865941
chr1	165866739	165867016
chr1	165867904	165868547
chr1	165868876	165869172
chr1	165869622	165870274
chr1	165872742	165873020
chr1	166135952	166136224
chr1	166808184	166808813

chr1	166808955	166809540
chr1	166844955	166845499
chr1	167028418	167028524
chr1	167130445	167130644
chr1	167188983	167189779
chr1	167190214	167191378
chr1	167191685	167191898
chr1	167202164	167202416
chr1	167236988	167237220
chr1	167424072	167425537
chr1	167514250	167514410
chr1	167522265	167522459
chr1	167522576	167522992
chr1	167533861	167533936
chr1	167542274	167542473
chr1	167599443	167599733
chr1	167632123	167632151
chr1	167632985	167633030
chr1	167633738	167633750
chr1	167635033	167635149
chr1	167635562	167635679
chr1	167635896	167636153
chr1	167682870	167683269
chr1	167683338	167685059
chr1	167685464	167686191
chr1	167690754	167692009
chr1	167690215	167690624
chr1	167694428	167694624
chr1	167710130	167710423
chr1	167710597	167710921
chr1	167721608	167721758
chr1	167722222	167722385
chr1	167722569	167722659
chr1	167743913	167744303
chr1	167759283	167759859
chr1	167765059	167765801
chr1	167766146	167766808
chr1	167769018	167769376
chr1	167772515	167772932
chr1	167789232	167790230
chr1	167891868	167892024
chr1	167904297	167905249
chr1	167905469	167907016
chr1	168029866	168029987
chr1	168048886	168049162
chr1	168051010	168051237

chr1	168051427	168051611
chr1	168081921	168082286
chr1	168105277	168106049
chr1	168106321	168106863
chr1	168147743	168148778
chr1	168148993	168149465
chr1	168194302	168194387
chr1	168194621	168195814
chr1	168195962	168195999
chr1	168230764	168230843
chr1	168270821	168271370
chr1	168348327	168348471
chr1	168408964	168409046
chr1	168440404	168440516
chr1	168445820	168445933
chr1	168452691	168452809
chr1	168597904	168597962
chr1	168605603	168605757
chr1	168605859	168606017
chr1	168606256	168606408
chr1	168889672	168890043
chr1	169074851	169075514
chr1	169075656	169076760
chr1	169077497	169077577
chr1	169077819	169077865
chr1	169078029	169078230
chr1	169078957	169079354
chr1	169082713	169083427
chr1	169085485	169085691
chr1	169102001	169102099
chr1	169332379	169332982
chr1	169334532	169334597
chr1	169336687	169336977
chr1	169337135	169337693
chr1	169453391	169453426
chr1	169453576	169453790
chr1	169454362	169455078
chr1	169455221	169455774
chr1	169455886	169456160
chr1	169480908	169481272
chr1	169496715	169496716
chr1	169498522	169498944
chr1	169508580	169508705
chr1	169750140	169750967
chr1	169763645	169764066
chr1	169764256	169764701

chr1	169862030	169862861
chr1	169863068	169863470
chr1	169863596	169863781
chr1	170032324	170032940
chr1	170043295	170043822
chr1	170044144	170044196
chr1	170500322	170500597
chr1	170500745	170501904
chr1	170532466	170532504
chr1	170594821	170594970
chr1	170928517	170928747
chr1	171282935	171283099
chr1	171283420	171283490
chr1	171389567	171389766
chr1	171403830	171403994
chr1	171404220	171404735
chr1	171407868	171408039
chr1	171410677	171410961
chr1	171437501	171437572
chr1	171437732	171438018
chr1	171454241	171454533
chr1	171454652	171455700
chr1	171486170	171486395
chr1	171554905	171554936
chr1	171555159	171555241
chr1	171710587	171712025
chr1	171712536	171712909
chr1	171750225	171750390
chr1	171750728	171751200
chr1	171763200	171763222
chr1	171778885	171778952
chr1	171786705	171786731
chr1	171810036	171811070
chr1	171811417	171811616
chr1	171829939	171830215
chr1	171867998	171868124
chr1	171868239	171868531
chr1	171870462	171870766
chr1	171871757	171871810
chr1	171871916	171872036
chr1	172009605	172009715
chr1	172108097	172108184
chr1	172123942	172124003
chr1	172224045	172224051
chr1	172238791	172238894
chr1	172327877	172328335

chr1	172384317	172384787
chr1	172386608	172386719
chr1	172392517	172392753
chr1	172400897	172401377
chr1	172412751	172413599
chr1	172416738	172416789
chr1	172419283	172419476
chr1	172445257	172445799
chr1	172468701	172468754
chr1	172501126	172501936
chr1	172502241	172503124
chr1	172557868	172558224
chr1	172606745	172606851
chr1	172607443	172607781
chr1	172607917	172608058
chr1	172608289	172608662
chr1	172688209	172688617
chr1	172688831	172688854
chr1	172689006	172689139
chr1	172715409	172715482
chr1	172715732	172715892
chr1	172795427	172795543
chr1	172807259	172807284
chr1	172807744	172807898
chr1	172828276	172828431
chr1	172828595	172828688
chr1	172878810	172879257
chr1	172879395	172879443
chr1	172886943	172887205
chr1	172887342	172887498
chr1	172902948	172903144
chr1	172903356	172903374
chr1	172903446	172903714
chr1	172917577	172917681
chr1	172920851	172920927
chr1	172921189	172921254
chr1	172924604	172924819
chr1	172940342	172940349
chr1	172940679	172940808
chr1	173018338	173018408
chr1	173021854	173022425
chr1	173025434	173025556
chr1	173025756	173025821
chr1	173031543	173031687
chr1	173091971	173092114
chr1	173092462	173092884

chr1	173098207	173098264
chr1	173118984	173119088
chr1	173119197	173119388
chr1	173131266	173131341
chr1	173159437	173159850
chr1	173160049	173160163
chr1	173171517	173171844
chr1	173238867	173238959
chr1	173362260	173362278
chr1	173389398	173389453
chr1	173393866	173393878
chr1	173394175	173394360
chr1	173398591	173398931
chr1	173399004	173399168
chr1	173399337	173399501
chr1	173400360	173400423
chr1	173442880	173443102
chr1	173444822	173445464
chr1	173445669	173446110
chr1	173446516	173447080
chr1	173447201	173447599
chr1	173496225	173496345
chr1	173523703	173523905
chr1	173606437	173606595
chr1	173611634	173611894
chr1	173612022	173612063
chr1	173629477	173629582
chr1	173683817	173683869
chr1	173684080	173684427
chr1	173684747	173684785
chr1	173728036	173728062
chr1	173767476	173767587
chr1	173793225	173794414
chr1	173794517	173794935
chr1	173830185	173830281
chr1	173830491	173830987
chr1	173831557	173832167
chr1	173836520	173837057
chr1	173834104	173834303
chr1	173837420	173838496
chr1	173915569	173915790
chr1	173915968	173916100
chr1	173919465	173919685
chr1	173990040	173990280
chr1	173990650	173991539
chr1	173991673	173991887

chr1	173993787	173993842
chr1	174122703	174123097
chr1	174128021	174128251
chr1	174128581	174129164
chr1	174129360	174129685
chr1	174187026	174187289
chr1	174208309	174208539
chr1	174375835	174375908
chr1	174768303	174768616
chr1	174768899	174769129
chr1	174784136	174784185
chr1	174787980	174788224
chr1	174788501	174788577
chr1	174818385	174818433
chr1	174945807	174945985
chr1	174967633	174969827
chr1	174992409	174992613
chr1	174992805	174992975
chr1	174998797	174999002
chr1	175160827	175161227
chr1	175161336	175162509
chr1	175190786	175190943
chr1	175191101	175191399
chr1	175191639	175191900
chr1	175238035	175238206
chr1	176088777	176088794
chr1	176175366	176177057
chr1	176210870	176210923
chr1	177884788	177885016
chr1	178001317	178001354
chr1	178006683	178006879
chr1	178006982	178007410
chr1	178024983	178025469
chr1	178027318	178027328
chr1	178029884	178030515
chr1	178061381	178061662
chr1	178062179	178062416
chr1	178062539	178064023
chr1	178064528	178064581
chr1	178064704	178064833
chr1	178071431	178071522
chr1	178095809	178096343
chr1	178142107	178142464
chr1	178161875	178162202
chr1	178189163	178189384
chr1	178189497	178189635

chr1	178207796	178208531
chr1	178236696	178237027
chr1	178237132	178237176
chr1	178237802	178238061
chr1	178242923	178243230
chr1	178247322	178247488
chr1	178252148	178252478
chr1	178252607	178252930
chr1	178268773	178269400
chr1	178277591	178277831
chr1	178279835	178280034
chr1	178289526	178289976
chr1	178292270	178292571
chr1	178293397	178293558
chr1	178299528	178299665
chr1	178310275	178310863
chr1	178313545	178314273
chr1	178366786	178366959
chr1	178368790	178368846
chr1	178386945	178387054
chr1	178434211	178434430
chr1	178439729	178439941
chr1	178456010	178456237
chr1	178499740	178499938
chr1	178506700	178507036
chr1	178511067	178512353
chr1	178531509	178531650
chr1	178531855	178531972
chr1	178577566	178577968
chr1	178578055	178578283
chr1	178578423	178578456
chr1	178579756	178579816
chr1	178580042	178580134
chr1	178581691	178581744
chr1	178602079	178602627
chr1	178624787	178625060
chr1	178625165	178625299
chr1	178653848	178653962
chr1	178654117	178654153
chr1	178654479	178654713
chr1	178694052	178695760
chr1	178994473	178994827
chr1	178994978	178996070
chr1	179050233	179050512
chr1	179050778	179052541
chr1	179098292	179098922

chr1	179099046	179099314
chr1	179111004	179111342
chr1	179111968	179112917
chr1	179138945	179139056
chr1	179144674	179145064
chr1	179148403	179148443
chr1	179162936	179163115
chr1	179197890	179198247
chr1	179198460	179198829
chr1	179199044	179199333
chr1	179199402	179199577
chr1	179199865	179199868
chr1	179201341	179201385
chr1	179202666	179203067
chr1	179203653	179203749
chr1	179261950	179261984
chr1	179262301	179262323
chr1	179262476	179263419
chr1	179263593	179264311
chr1	179276994	179277193
chr1	179279522	179279541
chr1	179279693	179279768
chr1	179279905	179280259
chr1	179334690	179335504
chr1	179784172	179784925
chr1	179846670	179847060
chr1	179850930	179851729
chr1	179851909	179852580
chr1	179852886	179853027
chr1	179853131	179853268
chr1	179895206	179895292
chr1	179895501	179895700
chr1	179921295	179921375
chr1	179922816	179923745
chr1	179923873	179924419
chr1	179924558	179924720
chr1	180012351	180012761
chr1	180067536	180067923
chr1	180092485	180092504
chr1	180095549	180095599
chr1	180095862	180096125
chr1	180120293	180120751
chr1	180120894	180121132
chr1	180123109	180123472
chr1	180123590	180124874
chr1	180137035	180137723

chr1	180137828	180137905
chr1	180198527	180198799
chr1	180278530	180278729
chr1	180279153	180279438
chr1	180468982	180469354
chr1	180471063	180472775
chr1	180474854	180475392
chr1	180484908	180485581
chr1	180490631	180491079
chr1	180491148	180491357
chr1	180491675	180491749
chr1	180494872	180495094
chr1	180499560	180499667
chr1	180499856	180500073
chr1	180549364	180549436
chr1	180549716	180549837
chr1	180554604	180555071
chr1	180555786	180555814
chr1	180555953	180556385
chr1	180600799	180600927
chr1	180601162	180601917
chr1	180603129	180603232
chr1	180698066	180698206
chr1	180742588	180742807
chr1	180761268	180761295
chr1	180796326	180796470
chr1	180880894	180882854
chr1	180893053	180893323
chr1	180917606	180918123
chr1	180918206	180918275
chr1	180921839	180923875
chr1	180985906	180986189
chr1	180990992	180991230
chr1	180991362	180992550
chr1	181002261	181002791
chr1	181002855	181003240
chr1	181007941	181008015
chr1	181012050	181012879
chr1	181013525	181014246
chr1	181014392	181015030
chr1	181047079	181047259
chr1	181047916	181048134
chr1	181053646	181053792
chr1	181056017	181056894
chr1	181057311	181057414
chr1	181057535	181057767

chr1	181059896	181060228
chr1	181057965	181059698
chr1	181063839	181064037
chr1	181064401	181064565
chr1	181064992	181065085
chr1	181065329	181065756
chr1	181066520	181066764
chr1	181068012	181068688
chr1	181068838	181069227
chr1	181069553	181070060
chr1	181070591	181071472
chr1	181071949	181072380
chr1	181073579	181074404
chr1	181074786	181075342
chr1	181080166	181080439
chr1	181080894	181081023
chr1	181081472	181081694
chr1	181081822	181082920
chr1	181085137	181085508
chr1	181085582	181085946
chr1	181086511	181086904
chr1	181087178	181087245
chr1	181087751	181087972
chr1	181088360	181088546
chr1	181088658	181088943
chr1	181089687	181090132
chr1	181096672	181097433
chr1	181101747	181101843
chr1	181103091	181103516
chr1	181103622	181104979
chr1	181105055	181105306
chr1	181105474	181105726
chr1	181105810	181106140
chr1	181106576	181106725
chr1	181108478	181109020
chr1	181109167	181109723
chr1	181110403	181111238
chr1	181111302	181111548
chr1	181109871	181109952
chr1	181111933	181112055
chr1	181128435	181128648
chr1	181128830	181129375
chr1	181129679	181129917
chr1	181737293	181737461
chr1	181737777	181737874
chr1	181998476	181999453

chr1	182054350	182054493
chr1	182057030	182057229
chr1	182057432	182057843
chr1	182070429	182070586
chr1	182070815	182070956
chr1	182112263	182112717
chr1	182113050	182113813
chr1	182143644	182143706
chr1	182144374	182144822
chr1	182150338	182150775
chr1	182155450	182155955
chr1	182187101	182187112
chr1	182187368	182187520
chr1	182259115	182259196
chr1	182269167	182269297
chr1	182348891	182349265
chr1	182358799	182359909
chr1	182360207	182360390
chr1	182360536	182361718
chr1	182366128	182366187
chr1	182366514	182366985
chr1	182367181	182367341
chr1	182557761	182558502
chr1	182572982	182573593
chr1	182573779	182573978
chr1	182583441	182583641
chr1	182583852	182584433
chr1	182584756	182584978
chr1	182753779	182753824
chr1	182758209	182758732
chr1	182758844	182759199
chr1	182773299	182773370
chr1	182807931	182807948
chr1	182808441	182809393
chr1	182931673	182931724
chr1	182931977	182932110
chr1	182991359	182993243
chr1	182994893	182994988
chr1	182993351	182993477
chr1	182995097	182995224
chr1	182998669	182999211
chr1	183024265	183024394
chr1	183024631	183024680
chr1	183029177	183029803
chr1	183063034	183063086
chr1	183063527	183063564

chr1	183117837	183117941
chr1	183118511	183119425
chr1	183128989	183129238
chr1	183129371	183129610
chr1	183132176	183132851
chr1	183133424	183133649
chr1	183133786	183133918
chr1	183134108	183134461
chr1	183134689	183134952
chr1	183149455	183149748
chr1	183149912	183150309
chr1	183154762	183155326
chr1	183155477	183155704
chr1	183160889	183161321
chr1	183164121	183164753
chr1	183166960	183167623
chr1	183167917	183168081
chr1	183178612	183178775
chr1	183179057	183179072
chr1	183181980	183182367
chr1	183183078	183183290
chr1	183188272	183188475
chr1	183199504	183199575
chr1	183199906	183199972
chr1	183203209	183203458
chr1	183203957	183204256
chr1	183237178	183237213
chr1	183237509	183237600
chr1	183241122	183241349
chr1	183241592	183241802
chr1	183241947	183241958
chr1	183242035	183242389
chr1	183246368	183246679
chr1	183247249	183247559
chr1	183248198	183248333
chr1	183248584	183249143
chr1	183249377	183249476
chr1	183250144	183250746
chr1	183250932	183251317
chr1	183288241	183288368
chr1	183287905	183288011
chr1	183430606	183430834
chr1	183431079	183431433
chr1	183436831	183436981
chr1	183437245	183437281
chr1	183439187	183439652

chr1	183440071	183440796
chr1	183440964	183442671
chr1	183450313	183450598
chr1	183462605	183463268
chr1	183484065	183484545
chr1	183537219	183537471
chr1	183538331	183538639
chr1	183577417	183577926
chr1	183602875	183603143
chr1	183604163	183605665
chr1	183774027	183774629
chr1	183851644	183851812
chr1	183884841	183885040
chr1	184006297	184006655
chr1	184020502	184020642
chr1	184021210	184021367
chr1	184026304	184026581
chr1	184355584	184357098
chr1	184357312	184357331
chr1	184357461	184357612
chr1	184360294	184360694
chr1	184360915	184361084
chr1	184385696	184385953
chr1	184393281	184393430
chr1	184393583	184393719
chr1	184460650	184460811
chr1	184483152	184483328
chr1	184515024	184515199
chr1	184581173	184581256
chr1	184581633	184581769
chr1	184723183	184723966
chr1	184724391	184724946
chr1	184922619	184922852
chr1	184923071	184923076
chr1	184923594	184923605
chr1	184923782	184923808
chr1	184940164	184940361
chr1	184942782	184942870
chr1	184943007	184943501
chr1	185014330	185015226
chr1	185015409	185015487
chr1	185060877	185060916
chr1	185124283	185124601
chr1	185125079	185125182
chr1	185125405	185126931
chr1	185284820	185284870

chr1	185285096	185286462
chr1	185286747	185287905
chr1	185306497	185306745
chr1	185336032	185336086
chr1	185344716	185345143
chr1	185370412	185370571
chr1	185381596	185381866
chr1	185382174	185382311
chr1	185489718	185489929
chr1	185497446	185497454
chr1	185534297	185534397
chr1	185534809	185534899
chr1	185543580	185543692
chr1	185650434	185650521
chr1	185703241	185703735
chr1	186028513	186028585
chr1	186051319	186051444
chr1	186051678	186051746
chr1	186063317	186063359
chr1	186144694	186144766
chr1	186144977	186145108
chr1	186152319	186152346
chr1	186156977	186157032
chr1	186181849	186182117
chr1	186334232	186334431
chr1	186344059	186344395
chr1	186344561	186345371
chr1	186505110	186505224
chr1	186528593	186529300
chr1	186546292	186546390
chr1	186546560	186546811
chr1	186587655	186587876
chr1	186612755	186612771
chr1	186628111	186628466
chr1	186631879	186632002
chr1	186649129	186649303
chr1	186649547	186650099
chr1	186704647	186704705
chr1	186706472	186706782
chr1	186719361	186719591
chr1	186719789	186719793
chr1	186761978	186762266
chr1	186797878	186798397
chr1	186798517	186798686
chr1	186828257	186828456
chr1	186896951	186897096

chr1	186927591	186927648
chr1	186943900	186943981
chr1	186955290	186955386
chr1	186970546	186971129
chr1	186976029	186976205
chr1	186976376	186976430
chr1	187811699	187811878
chr1	187812027	187812367
chr1	187831660	187831694
chr1	187831920	187832033
chr1	187897454	187897576
chr1	187997072	187997183
chr1	188319590	188319631
chr1	188675803	188675991
chr1	188751701	188751722
chr1	189626844	189626853
chr1	189627231	189627248
chr1	189627515	189627611
chr1	189627768	189627877
chr1	189629072	189629219
chr1	190648170	190648201
chr1	190648357	190648530
chr1	191313158	191313267
chr1	191577366	191577432
chr1	191578668	191578873
chr1	191871062	191871179
chr1	192074309	192074436
chr1	192194869	192194934
chr1	192195097	192195121
chr1	192500191	192500221
chr1	192670218	192670253
chr1	192775274	192775394
chr1	192777039	192778019
chr1	192778211	192778369
chr1	192778468	192778818
chr1	192803143	192803628
chr1	192862157	192862345
chr1	192870886	192871010
chr1	193018158	193018368
chr1	193027664	193028467
chr1	193028742	193029030
chr1	193029205	193029430
chr1	193073581	193075006
chr1	193080738	193081138
chr1	193084716	193084892
chr1	193085084	193085212

chr1	193090641	193090906
chr1	193091042	193091765
chr1	193101911	193102369
chr1	193180576	193180993
chr1	193291409	193291455
chr1	193291656	193291796
chr1	193407211	193407274
chr1	193456009	193456127
chr1	195561435	195561783
chr1	195633019	195633118
chr1	195633322	195633367
chr1	195733203	195733322
chr1	195821001	195821067
chr1	195821218	195821280
chr1	195877371	195877586
chr1	195927193	195927683
chr1	195972247	195972508
chr1	196527602	196527694
chr1	196527812	196527881
chr1	196577518	196578011
chr1	196620926	196621191
chr1	196712562	196712784
chr1	197004051	197004280
chr1	197062920	197062961
chr1	197106495	197106502
chr1	197106692	197106853
chr1	197115376	197116181
chr1	197126049	197126165
chr1	197149577	197149756
chr1	197169811	197170900
chr1	197652989	197653063
chr1	197705723	197705978
chr1	197743787	197744740
chr1	197751375	197751574
chr1	197752956	197753155
chr1	197808047	197808084
chr1	197871461	197872014
chr1	197872189	197872324
chr1	197882039	197882238
chr1	197945416	197945524
chr1	197945800	197945815
chr1	198125511	198125615
chr1	198125812	198127078
chr1	198127494	198127696
chr1	198127768	198128182
chr1	198128297	198128529

chr1	198136685	198136764
chr1	198138820	198138949
chr1	198302961	198303083
chr1	198412446	198412569
chr1	198412730	198412915
chr1	198418387	198418794
chr1	198431485	198431598
chr1	198431731	198431820
chr1	198626962	198627058
chr1	198806627	198806769
chr1	198850378	198850716
chr1	198901899	198902236
chr1	198904144	198904718
chr1	198904912	198905100
chr1	198906375	198906851
chr1	199241857	199242258
chr1	199660653	199660662
chr1	199660964	199661060
chr1	199711691	199711811
chr1	199712033	199712096
chr1	199728170	199728960
chr1	199753439	199753604
chr1	199824606	199824739
chr1	199825121	199825123
chr1	199826218	199826596
chr1	199828567	199828702
chr1	199918065	199918132
chr1	199918342	199918469
chr1	199998216	199998420
chr1	200153260	200153416
chr1	200153691	200153816
chr1	200180299	200180639
chr1	200201075	200201364
chr1	200249141	200249409
chr1	200270796	200270919
chr1	200271343	200271894
chr1	200289370	200289384
chr1	200303802	200303950
chr1	200304074	200304197
chr1	200305801	200305916
chr1	200321307	200321350
chr1	200328931	200329070
chr1	200341213	200341328
chr1	200341560	200341669
chr1	200348008	200348207
chr1	200348293	200348926

chr1	200350376	200351089
chr1	200351297	200351641
chr1	200352290	200352529
chr1	200358443	200358963
chr1	200378331	200380067
chr1	200382796	200383259
chr1	200384784	200385482
chr1	200385697	200386110
chr1	200386192	200386500
chr1	200407800	200407925
chr1	200452499	200452623
chr1	200452906	200453036
chr1	200458054	200458138
chr1	200519950	200519955
chr1	200589511	200589891
chr1	200590042	200590269
chr1	200590388	200590811
chr1	200591445	200591768
chr1	200609111	200609503
chr1	200610048	200610599
chr1	200623366	200624251
chr1	200626799	200626998
chr1	200628294	200629063
chr1	200635995	200636102
chr1	200638358	200638968
chr1	200643454	200644402
chr1	200639260	200639851
chr1	200652977	200653605
chr1	200707409	200707483
chr1	200707592	200707837
chr1	200708072	200709341
chr1	200719862	200720168
chr1	200724581	200724670
chr1	200725280	200725299
chr1	200740851	200741164
chr1	200741303	200741455
chr1	200811766	200812212
chr1	200842376	200842575
chr1	200860081	200861691
chr1	200862528	200862731
chr1	200862925	200863635
chr1	200863732	200865518
chr1	200865657	200865914
chr1	200867288	200867387
chr1	200867991	200868077
chr1	200882155	200882795

chr1	200988161	200988496
chr1	200988701	200989127
chr1	200989229	200989334
chr1	200989513	200989858
chr1	200991781	200992936
chr1	201058498	201058782
chr1	201082853	201083027
chr1	201083106	201084427
chr1	201122709	201124149
chr1	201223864	201224151
chr1	201224251	201224332
chr1	201227596	201227849
chr1	201228515	201229888
chr1	201230033	201230232
chr1	201230670	201230675
chr1	201235220	201235249
chr1	201235825	201236315
chr1	201236628	201236917
chr1	201237129	201237356
chr1	201237791	201238056
chr1	201238497	201238746
chr1	201243788	201243987
chr1	201244702	201245362
chr1	201246001	201246275
chr1	201246408	201246784
chr1	201252048	201254169
chr1	201254436	201254653
chr1	201254878	201255197
chr1	201255353	201255775
chr1	201255975	201256243
chr1	201256456	201256559
chr1	201256640	201256780
chr1	201257132	201257275
chr1	201257632	201258122
chr1	201258245	201258695
chr1	201259109	201259306
chr1	201264602	201264849
chr1	201264919	201265895
chr1	201267890	201268123
chr1	201268247	201268505
chr1	201268891	201270610
chr1	201271215	201272190
chr1	201273404	201273419
chr1	201273721	201274054
chr1	201274564	201274698
chr1	201275477	201275610

chr1	201275681	201277111
chr1	201277920	201278248
chr1	201277239	201277704
chr1	201278375	201278486
chr1	201278585	201278778
chr1	201279019	201279161
chr1	201279655	201280823
chr1	201280957	201282057
chr1	201288282	201288478
chr1	201303851	201303855
chr1	201315704	201315929
chr1	201316039	201316139
chr1	201317191	201317291
chr1	201330442	201330679
chr1	201353067	201353388
chr1	201353928	201353935
chr1	201354143	201354417
chr1	201357281	201357420
chr1	201362578	201362799
chr1	201364525	201364818
chr1	201365091	201365437
chr1	201365693	201369126
chr1	201377043	201377293
chr1	201404596	201405266
chr1	201417156	201417397
chr1	201417545	201417638
chr1	201425562	201425801
chr1	201424859	201425246
chr1	201426289	201426511
chr1	201428471	201428501
chr1	201429494	201429582
chr1	201430208	201431683
chr1	201431905	201433242
chr1	201433928	201433971
chr1	201434171	201434245
chr1	201435422	201435624
chr1	201435858	201438593
chr1	201441687	201441913
chr1	201441131	201441451
chr1	201444231	201444375
chr1	201444592	201444647
chr1	201445057	201446214
chr1	201449342	201449610
chr1	201450044	201450611
chr1	201450801	201451467
chr1	201457959	201458510

chr1	201458674	201459063
chr1	201472435	201473118
chr1	201474333	201475010
chr1	201475441	201475507
chr1	201475675	201476593
chr1	201481970	201482216
chr1	201486738	201487168
chr1	201507627	201508234
chr1	201508341	201508776
chr1	201617233	201617590
chr1	201617699	201618114
chr1	201618957	201619187
chr1	201619418	201619671
chr1	201682347	201682631
chr1	201682953	201683025
chr1	201683130	201683816
chr1	201684141	201684395
chr1	201708473	201709327
chr1	201709434	201709642
chr1	201709715	201709725
chr1	201709875	201710134
chr1	201725013	201725136
chr1	201755012	201755211
chr1	201798203	201798907
chr1	201814422	201814493
chr1	201814690	201814889
chr1	201852231	201852369
chr1	201852466	201852484
chr1	201857791	201858343
chr1	201873564	201873777
chr1	201907360	201907391
chr1	201924099	201924993
chr1	201951417	201951616
chr1	201951728	201952438
chr1	201952559	201952699
chr1	201955862	201956169
chr1	201956352	201956492
chr1	201965711	201965900
chr1	201975768	201975933
chr1	201976758	201976802
chr1	201977011	201977344
chr1	201978529	201979466
chr1	201979622	201980029
chr1	201980232	201980236
chr1	201980601	201980880
chr1	201986749	201987147

chr1	201987514	201988286
chr1	201988476	201988637
chr1	201991460	201991809
chr1	201997853	201998052
chr1	202002365	202002499
chr1	202003755	202003954
chr1	202012482	202012681
chr1	202013063	202013347
chr1	202014435	202014552
chr1	202015180	202015243
chr1	202015655	202016040
chr1	202016482	202016681
chr1	202018022	202018087
chr1	202025063	202025861
chr1	202026450	202026678
chr1	202027257	202027904
chr1	202033928	202034180
chr1	202046349	202046387
chr1	202046624	202046666
chr1	202060362	202060482
chr1	202061199	202061315
chr1	202061871	202061894
chr1	202062320	202062387
chr1	202069362	202069561
chr1	202081555	202081715
chr1	202087456	202087980
chr1	202088392	202088591
chr1	202100913	202101187
chr1	202110951	202110966
chr1	202113110	202113947
chr1	202114887	202114949
chr1	202123809	202124175
chr1	202174460	202174745
chr1	202191882	202192095
chr1	202205115	202205439
chr1	202250063	202250188
chr1	202250411	202251113
chr1	202251439	202251965
chr1	202252209	202252499
chr1	202252882	202253163
chr1	202253420	202253552
chr1	202264831	202265105
chr1	202310630	202311037
chr1	202311328	202311472
chr1	202317946	202318582
chr1	202334520	202334681

chr1	202393383	202393727
chr1	202425627	202425980
chr1	202452451	202452563
chr1	202512627	202513127
chr1	202538990	202539307
chr1	202545476	202545758
chr1	202545981	202546156
chr1	202546320	202546330
chr1	202555487	202555624
chr1	202557935	202558382
chr1	202558655	202558970
chr1	202559335	202559461
chr1	202559737	202560427
chr1	202560741	202561022
chr1	202561312	202561558
chr1	202562309	202563490
chr1	202568895	202569412
chr1	202570430	202570564
chr1	202772058	202772413
chr1	202775912	202775970
chr1	202776168	202777555
chr1	202779449	202779615
chr1	202780107	202781671
chr1	202781888	202782043
chr1	202784468	202784550
chr1	202806510	202806536
chr1	202816573	202816779
chr1	202829786	202829984
chr1	202857394	202858243
chr1	202858414	202858548
chr1	202861622	202862074
chr1	202895825	202896888
chr1	202921846	202921883
chr1	202925123	202925324
chr1	202926689	202928206
chr1	202928299	202928338
chr1	202928492	202928773
chr1	202933806	202935163
chr1	202935291	202935430
chr1	202935493	202936331
chr1	202936575	202937040
chr1	202975647	202976056
chr1	202976160	202977087
chr1	202995213	202995492
chr1	202995861	202996109
chr1	203236605	203236912

chr1	203249624	203249819
chr1	203273845	203273867
chr1	203274131	203274274
chr1	203274587	203275533
chr1	203275817	203276149
chr1	203290731	203290759
chr1	203296278	203296520
chr1	203296678	203297327
chr1	203307571	203307706
chr1	203431935	203432288
chr1	203433182	203433409
chr1	203450176	203450434
chr1	203457250	203457612
chr1	203481770	203481983
chr1	203482235	203482472
chr1	203483284	203483483
chr1	203495954	203496243
chr1	203503651	203503713
chr1	203504134	203504154
chr1	203505995	203505999
chr1	203506301	203506504
chr1	203527147	203527439
chr1	203561484	203561720
chr1	203576226	203576534
chr1	203577077	203577352
chr1	203594664	203594700
chr1	203595121	203595783
chr1	203596002	203596388
chr1	203596868	203596983
chr1	203597138	203599050
chr1	203602045	203602357
chr1	203605386	203605627
chr1	203618648	203618978
chr1	203619214	203620055
chr1	203632338	203632885
chr1	203642829	203642938
chr1	203643177	203643327
chr1	203643612	203643731
chr1	203644045	203644216
chr1	203646893	203646957
chr1	203647645	203647844
chr1	203648038	203648104
chr1	203648316	203648517
chr1	203661485	203662391
chr1	203662539	203662709
chr1	203671291	203671905

chr1	203720176	203720695
chr1	203721778	203722106
chr1	203722762	203723124
chr1	203723520	203724052
chr1	203725551	203725980
chr1	203726333	203726560
chr1	203738548	203738758
chr1	203738982	203739161
chr1	203741758	203741865
chr1	203745817	203745928
chr1	203761834	203762108
chr1	203764400	203764570
chr1	203764686	203765416
chr1	203813392	203813596
chr1	203827045	203827494
chr1	203829382	203831080
chr1	203858034	203858427
chr1	203858674	203859368
chr1	203866199	203866476
chr1	203876312	203876487
chr1	203895132	203895327
chr1	203895616	203896008
chr1	204020400	204020461
chr1	204026729	204026999
chr1	204027684	204027696
chr1	204042229	204043461
chr1	204043743	204044073
chr1	204047327	204047673
chr1	204047830	204048244
chr1	204048358	204048565
chr1	204048636	204049222
chr1	204049349	204049681
chr1	204049874	204050102
chr1	204098373	204098669
chr1	204099830	204100167
chr1	204120572	204121255
chr1	204121415	204121991
chr1	204122699	204122741
chr1	204123062	204123350
chr1	204131819	204132018
chr1	204140726	204140912
chr1	204159303	204159793
chr1	204165772	204165971
chr1	204167642	204167841
chr1	204167957	204168438
chr1	204182753	204183205

chr1	204222710	204223079
chr1	204223564	204223750
chr1	204257012	204257450
chr1	204263340	204263580
chr1	204263725	204263874
chr1	204270271	204270364
chr1	204270615	204270749
chr1	204288145	204289178
chr1	204289396	204290057
chr1	204332273	204332825
chr1	204346710	204346747
chr1	204346821	204347031
chr1	204349931	204349979
chr1	204367580	204367665
chr1	204377208	204377478
chr1	204379305	204379555
chr1	204380238	204380956
chr1	204381240	204381602
chr1	204386175	204387137
chr1	204387430	204387754
chr1	204391866	204392145
chr1	204400719	204401210
chr1	204404633	204405035
chr1	204405416	204405615
chr1	204407582	204407748
chr1	204412205	204413158
chr1	204413491	204413690
chr1	204462556	204462881
chr1	204463178	204464107
chr1	204475303	204475916
chr1	204476027	204476384
chr1	204476650	204477233
chr1	204478427	204478603
chr1	204478797	204478838
chr1	204485208	204486040
chr1	204486579	204486799
chr1	204493226	204493364
chr1	204494057	204494094
chr1	204494335	204494373
chr1	204514713	204514717
chr1	204547935	204548032
chr1	204677271	204677318
chr1	204677538	204677704
chr1	204884945	204884973
chr1	204885488	204885584
chr1	205090844	205091702

chr1	205110683	205110728
chr1	205141828	205141849
chr1	205142082	205142229
chr1	205144488	205145003
chr1	205145081	205145548
chr1	205179730	205181091
chr1	205195384	205195817
chr1	205195887	205195917
chr1	205196744	205196884
chr1	205197342	205197618
chr1	205198197	205198645
chr1	205227488	205228054
chr1	205228295	205229338
chr1	205230424	205230949
chr1	205231113	205231349
chr1	205232784	205232881
chr1	205234586	205234645
chr1	205235158	205235610
chr1	205237677	205237876
chr1	205244140	205244238
chr1	205243651	205243863
chr1	205244642	205244980
chr1	205245132	205246572
chr1	205248083	205248222
chr1	205248435	205248520
chr1	205248970	205249255
chr1	205251419	205251674
chr1	205252166	205252468
chr1	205253173	205254612
chr1	205254895	205255132
chr1	205257205	205257368
chr1	205257486	205257688
chr1	205262601	205263949
chr1	205264152	205264476
chr1	205264738	205265225
chr1	205265406	205265712
chr1	205265895	205266924
chr1	205267294	205267684
chr1	205267835	205269033
chr1	205269285	205269489
chr1	205269875	205271263
chr1	205271388	205271815
chr1	205271904	205272364
chr1	205272805	205273149
chr1	205274832	205275280
chr1	205280745	205280959

chr1	205282774	205282946
chr1	205284351	205284562
chr1	205286144	205289072
chr1	205290255	205291263
chr1	205293699	205294622
chr1	205310718	205310887
chr1	205399717	205399941
chr1	205409890	205410134
chr1	205419410	205419609
chr1	205446520	205446830
chr1	205447570	205447725
chr1	205449688	205449895
chr1	205462245	205462444
chr1	205473764	205474845
chr1	205476555	205476800
chr1	205560812	205561042
chr1	205561375	205561578
chr1	205569541	205569642
chr1	205576191	205576371
chr1	205600075	205600992
chr1	205601274	205601644
chr1	205602607	205602806
chr1	205604813	205604913
chr1	205648272	205648521
chr1	205648651	205649918
chr1	205703666	205703888
chr1	205709275	205709356
chr1	205717623	205719703
chr1	205735331	205735753
chr1	205743905	205744555
chr1	205744757	205745166
chr1	205781367	205782218
chr1	205782456	205783310
chr1	205876350	205876483
chr1	205877015	205877214
chr1	205894780	205894835
chr1	205902966	205903165
chr1	206078482	206078886
chr1	206080134	206080326
chr1	206090012	206090168
chr1	206133815	206133889
chr1	206135157	206135224
chr1	206135429	206137793
chr1	206138465	206139668
chr1	206164763	206165846
chr1	206173572	206173656

chr1	206223747	206224038
chr1	206265124	206265311
chr1	206265546	206265563
chr1	206290478	206290594
chr1	206311778	206311874
chr1	206319114	206319296
chr1	206319501	206319743
chr1	206490100	206490102
chr1	206491772	206491796
chr1	206493279	206493527
chr1	206493795	206494044
chr1	206531327	206531361
chr1	206532355	206532560
chr1	206535252	206535851
chr1	206548299	206548498
chr1	206553348	206553664
chr1	206567184	206567546
chr1	206577281	206577526
chr1	206581614	206581678
chr1	206581884	206581946
chr1	206583005	206583204
chr1	206603294	206603488
chr1	206642633	206642904
chr1	206643047	206644831
chr1	206644942	206645213
chr1	206654316	206654698
chr1	206655272	206655872
chr1	206656278	206656500
chr1	206674225	206674515
chr1	206674632	206674872
chr1	206679819	206679868
chr1	206680070	206680171
chr1	206680416	206680731
chr1	206680915	206681403
chr1	206683862	206684051
chr1	206685923	206686783
chr1	206687041	206687850
chr1	206698752	206698894
chr1	206730490	206730939
chr1	206756776	206756975
chr1	206762492	206762544
chr1	206763667	206763873
chr1	206785438	206785782
chr1	206785983	206786235
chr1	206806975	206807097
chr1	206807348	206807566

chr1	206807652	206807986
chr1	206808409	206808710
chr1	206808860	206809345
chr1	206809424	206809660
chr1	206811825	206812164
chr1	206848019	206848671
chr1	206857770	206858138
chr1	206858317	206858964
chr1	206859190	206859819
chr1	206883103	206883277
chr1	206883409	206883764
chr1	206997922	206997995
chr1	206999957	207000248
chr1	207020919	207020982
chr1	207021471	207021621
chr1	207032349	207032688
chr1	207036662	207036974
chr1	207038286	207038722
chr1	207046890	207047559
chr1	207058105	207058358
chr1	207058826	207059649
chr1	207061098	207061341
chr1	207063306	207064535
chr1	207065964	207066360
chr1	207068444	207069965
chr1	207071595	207072416
chr1	207078328	207078643
chr1	207078948	207079130
chr1	207081031	207081267
chr1	207081523	207081776
chr1	207082143	207083627
chr1	207084380	207084570
chr1	207084674	207084841
chr1	207085876	207085880
chr1	207097472	207097899
chr1	207175389	207175566
chr1	207176024	207176138
chr1	207176410	207176698
chr1	207178627	207178742
chr1	207180213	207181151
chr1	207191456	207191916
chr1	207193297	207195391
chr1	207196348	207196565
chr1	207200441	207200755
chr1	207200871	207201244
chr1	207202432	207202601

chr1	207203380	207204185
chr1	207204436	207204756
chr1	207204939	207205605
chr1	207205671	207206003
chr1	207206106	207206524
chr1	207208251	207208318
chr1	207208555	207208706
chr1	207223265	207223586
chr1	207223727	207224313
chr1	207225622	207225860
chr1	207225924	207226395
chr1	207224816	207225239
chr1	207226513	207226906
chr1	207227010	207227235
chr1	207251096	207251384
chr1	207378308	207378498
chr1	207389993	207390014
chr1	207390132	207390318
chr1	207406346	207406464
chr1	207413969	207414030
chr1	207414255	207414405
chr1	207482986	207483466
chr1	207489711	207489812
chr1	207489940	207489999
chr1	207490190	207490468
chr1	207494209	207494632
chr1	207494849	207495782
chr1	207563432	207563671
chr1	207566905	207566997
chr1	207567255	207567513
chr1	207627656	207627833
chr1	207672860	207672936
chr1	207818467	207818801
chr1	207880594	207881110
chr1	207881282	207881362
chr1	207913512	207913839
chr1	207925013	207925248
chr1	207925498	207926404
chr1	207970696	207971146
chr1	207978747	207979844
chr1	207980939	207981846
chr1	207989872	207990071
chr1	207996903	207997700
chr1	208002286	208002321
chr1	208020416	208020525
chr1	208020743	208020830

chr1	208021499	208021601
chr1	208022734	208022840
chr1	208041669	208043161
chr1	208063822	208064178
chr1	208132607	208132855
chr1	208136381	208136961
chr1	208137107	208138112
chr1	208220515	208220950
chr1	208258731	208259042
chr1	208259393	208259516
chr1	208259817	208260475
chr1	208280961	208282209
chr1	208282284	208282523
chr1	208301031	208301064
chr1	208308733	208308823
chr1	208309099	208309197
chr1	208329162	208329359
chr1	208330498	208330643
chr1	208336403	208336637
chr1	208340254	208340488
chr1	208345027	208345226
chr1	208357339	208357735
chr1	208358087	208358579
chr1	208374267	208374387
chr1	208380654	208381264
chr1	208381386	208381792
chr1	208382371	208383247
chr1	208383669	208383947
chr1	208387083	208387156
chr1	208387418	208387467
chr1	208392428	208392749
chr1	208392986	208393599
chr1	208394098	208394715
chr1	208405183	208405852
chr1	208404184	208405005
chr1	208406975	208407179
chr1	208408839	208408894
chr1	208409039	208410270
chr1	208410505	208410767
chr1	208411057	208411247
chr1	208412630	208413028
chr1	208415835	208415878
chr1	208416155	208417983
chr1	208980769	208981104
chr1	209151447	209151540
chr1	209154001	209154350

chr1	209162059	209162211
chr1	209162389	209162659
chr1	209212173	209212383
chr1	209212617	209212716
chr1	209213101	209213340
chr1	209222319	209222397
chr1	209381021	209381466
chr1	209402721	209402980
chr1	209403085	209403220
chr1	209422243	209422355
chr1	209508664	209508858
chr1	209527586	209527752
chr1	209527914	209528099
chr1	209528516	209529243
chr1	209530211	209530842
chr1	209536365	209536764
chr1	209537890	209538555
chr1	209540322	209541129
chr1	209542388	209542984
chr1	209543632	209544432
chr1	209549080	209549514
chr1	209556729	209558010
chr1	209560581	209561098
chr1	209561226	209561739
chr1	209562432	209563113
chr1	209571680	209572446
chr1	209575897	209575906
chr1	209576162	209576291
chr1	209576519	209576733
chr1	209577234	209577734
chr1	209577997	209578335
chr1	209578566	209578897
chr1	209583134	209583634
chr1	209584741	209585390
chr1	209585462	209585925
chr1	209589023	209589926
chr1	209594134	209594405
chr1	209594551	209594860
chr1	209600572	209600979
chr1	209601050	209602993
chr1	209603244	209603894
chr1	209604072	209604728
chr1	209604791	209607845
chr1	209608011	209608333
chr1	209608480	209609403
chr1	209658452	209658527

chr1	209678707	209678848
chr1	209775718	209775822
chr1	209780606	209780873
chr1	209798642	209798841
chr1	209806935	209807188
chr1	209814928	209815147
chr1	209815878	209816096
chr1	209816716	209817561
chr1	209818337	209818419
chr1	209819927	209820213
chr1	209820807	209821160
chr1	209821819	209822025
chr1	209822338	209823576
chr1	209823969	209825295
chr1	209825444	209826486
chr1	209826709	209826897
chr1	209827241	209827421
chr1	209827658	209827809
chr1	209827896	209828419
chr1	209828839	209829661
chr1	209830325	209831128
chr1	209831421	209831771
chr1	209846595	209846685
chr1	209846931	209847005
chr1	209847356	209847445
chr1	209847588	209847922
chr1	209848062	209849598
chr1	209866235	209866474
chr1	209920904	209921161
chr1	209921389	209921620
chr1	209942523	209942788
chr1	209957420	209958571
chr1	209965461	209965838
chr1	209971713	209971873
chr1	209977704	209978249
chr1	209978513	209980078
chr1	209980276	209980494
chr1	209981358	209981501
chr1	209981724	209981883
chr1	209982592	209983465
chr1	209989062	209990225
chr1	209994296	209994752
chr1	209996111	209996771
chr1	209996893	209997369
chr1	209999200	209999366
chr1	209999518	209999732

chr1	210000976	210001789
chr1	210013135	210013381
chr1	210013515	210013742
chr1	210017731	210017935
chr1	210019179	210019449
chr1	210046426	210047181
chr1	210057868	210058434
chr1	210074458	210074461
chr1	210111238	210111549
chr1	210405388	210405569
chr1	210406071	210407238
chr1	210424962	210425665
chr1	210430837	210430947
chr1	210439542	210439951
chr1	210465546	210466213
chr1	210484465	210484573
chr1	210501900	210503425
chr1	210547194	210547611
chr1	210547715	210547875
chr1	211431499	211433122
chr1	211433456	211434165
chr1	211434350	211434530
chr1	211498406	211498410
chr1	211499880	211501065
chr1	211555856	211555909
chr1	211556163	211556790
chr1	211589568	211590598
chr1	211688560	211689283
chr1	211689585	211689597
chr1	211690412	211690495
chr1	211691043	211691045
chr1	211724301	211724490
chr1	211724869	211725504
chr1	211750751	211752515
chr1	211752709	211752908
chr1	211753210	211753261
chr1	211753648	211753726
chr1	211763641	211763881
chr1	211777110	211777199
chr1	211781124	211781284
chr1	211821580	211822067
chr1	211822317	211822405
chr1	211823477	211823689
chr1	211826632	211827377
chr1	211848380	211848927
chr1	211849265	211849674

chr1	211854165	211854483
chr1	211872197	211872396
chr1	211886876	211887059
chr1	212002932	212003036
chr1	212003396	212004449
chr1	212101508	212101976
chr1	212102236	212102439
chr1	212102674	212103015
chr1	212118072	212118531
chr1	212125188	212125616
chr1	212201055	212201509
chr1	212203756	212204683
chr1	212208034	212208374
chr1	212208612	212208845
chr1	212209093	212209367
chr1	212214174	212214276
chr1	212261674	212261865
chr1	212262141	212262297
chr1	212352881	212353110
chr1	212455205	212455298
chr1	212455871	212455980
chr1	212457809	212457931
chr1	212458090	212458407
chr1	212458788	212459731
chr1	212459922	212460105
chr1	212464810	212464999
chr1	212575598	212575924
chr1	212587193	212588544
chr1	212605945	212606104
chr1	212606226	212606946
chr1	212629158	212629190
chr1	212642876	212643079
chr1	212643759	212643895
chr1	212643995	212644013
chr1	212644151	212644202
chr1	212650744	212650943
chr1	212651078	212651448
chr1	212658070	212658268
chr1	212658578	212658763
chr1	212661088	212661351
chr1	212662173	212662685
chr1	212675919	212676187
chr1	212676344	212676747
chr1	212687380	212687579
chr1	212719260	212719309
chr1	212719481	212719621

chr1	212731501	212732706
chr1	212740363	212740668
chr1	212753264	212753623
chr1	212768688	212770113
chr1	212779066	212779322
chr1	212779976	212781696
chr1	212782107	212783458
chr1	212872747	212873305
chr1	212923248	212924066
chr1	212964699	212965058
chr1	212965283	212966060
chr1	213030924	213031188
chr1	213031592	213032639
chr1	213046226	213046778
chr1	213123674	213124726
chr1	213124914	213125222
chr1	213188359	213189396
chr1	213224150	213224468
chr1	213224637	213225248
chr1	213228658	213228728
chr1	213243700	213243852
chr1	214386348	214386526
chr1	214453846	214455549
chr1	214509711	214509935
chr1	214559851	214560166
chr1	214560559	214560583
chr1	214560668	214560882
chr1	214567893	214568316
chr1	214577824	214578803
chr1	214588499	214588861
chr1	214589820	214590054
chr1	214593721	214594057
chr1	214595516	214596129
chr1	214601118	214601277
chr1	214601463	214601680
chr1	214605289	214605488
chr1	214612710	214612902
chr1	214613080	214613506
chr1	214614444	214614959
chr1	214613962	214614338
chr1	214619108	214619372
chr1	214620133	214620379
chr1	214620587	214620996
chr1	214621901	214622242
chr1	214627981	214628825
chr1	214640796	214640916

chr1	214648667	214649253
chr1	214649490	214650489
chr1	214646754	214647143
chr1	214650634	214651235
chr1	214668037	214668039
chr1	214668381	214668502
chr1	214696956	214697183
chr1	214700209	214700546
chr1	214722336	214722446
chr1	214722567	214722724
chr1	214723173	214725122
chr1	214725319	214725783
chr1	214725893	214726047
chr1	214737271	214737375
chr1	214740421	214740461
chr1	214743882	214743995
chr1	214746714	214747087
chr1	214747174	214747712
chr1	214747982	214748567
chr1	214755401	214755676
chr1	214758444	214758654
chr1	214766035	214766266
chr1	214776073	214776258
chr1	214776367	214777600
chr1	214778123	214778186
chr1	215038271	215038286
chr1	215038447	215038587
chr1	215038710	215039068
chr1	215075278	215075534
chr1	215076048	215076558
chr1	215094866	215094949
chr1	215130635	215131098
chr1	215163943	215163965
chr1	215175328	215175419
chr1	215196698	215196897
chr1	215740337	215741140
chr1	215741402	215741735
chr1	215756635	215756683
chr1	215756939	215757131
chr1	215805310	215805315
chr1	216488079	216488120
chr1	216638024	216638184
chr1	216704676	216704891
chr1	216774013	216774137
chr1	216774269	216774611
chr1	217075864	217077273

chr1	217202536	217202624
chr1	217202924	217203092
chr1	217203310	217203503
chr1	217263006	217263233
chr1	217308745	217309131
chr1	217366536	217366593
chr1	217543367	217543741
chr1	217735585	217736254
chr1	217803981	217804744
chr1	217833624	217833678
chr1	218382619	218382685
chr1	218457907	218458509
chr1	218458631	218458898
chr1	218467874	218467894
chr1	218469552	218469885
chr1	218472757	218472810
chr1	218518651	218518870
chr1	218519061	218519301
chr1	218550266	218550465
chr1	218550560	218550767
chr1	218551037	218551267
chr1	218553997	218554290
chr1	218633539	218633633
chr1	218633903	218633932
chr1	218636535	218636715
chr1	218649710	218649727
chr1	218650077	218650111
chr1	218653406	218653421
chr1	218654063	218654245
chr1	218669859	218670015
chr1	218670348	218670429
chr1	218671332	218671374
chr1	218879148	218879206
chr1	218879331	218879530
chr1	218879835	218880051
chr1	218885555	218885787
chr1	219047591	219047640
chr1	219047765	219047964
chr1	219048228	219048264
chr1	219347003	219347093
chr1	219347309	219347703
chr1	219347917	219348451
chr1	219352916	219352992
chr1	219972163	219972335
chr1	220101459	220101658
chr1	220218879	220219078

chr1	220219405	220219710
chr1	220220084	220220207
chr1	220262968	220263221
chr1	220263522	220264028
chr1	220267495	220267876
chr1	220268098	220268104
chr1	220392262	220392339
chr1	220444975	220445769
chr1	220446023	220446558
chr1	220454414	220454453
chr1	220473237	220473451
chr1	220474080	220474147
chr1	220509931	220510145
chr1	220510667	220511115
chr1	220516965	220517062
chr1	220543390	220543629
chr1	220544639	220544641
chr1	220670733	220670928
chr1	220671858	220671999
chr1	220672756	220672925
chr1	220684498	220684900
chr1	220685113	220685522
chr1	220689310	220689831
chr1	220690017	220690306
chr1	220700018	220700041
chr1	220700462	220700536
chr1	220700689	220701010
chr1	220701283	220702838
chr1	220863664	220864190
chr1	220906548	220906767
chr1	220921777	220922151
chr1	220960171	220960649
chr1	220960890	220961110
chr1	220961588	220961980
chr1	221052893	221053131
chr1	221054865	221055041
chr1	221590051	221590325
chr1	221593067	221593121
chr1	221612661	221612728
chr1	221613694	221613700
chr1	221786167	221786197
chr1	221840817	221840991
chr1	221914611	221915422
chr1	221915869	221916146
chr1	221916307	221916822
chr1	221931046	221931165

chr1	221983793	221983901
chr1	222005952	222006123
chr1	222012881	222013128
chr1	222013472	222013621
chr1	222060889	222060988
chr1	222417203	222417265
chr1	222417531	222417730
chr1	222638359	222638714
chr1	222638869	222639420
chr1	222663364	222663416
chr1	222695257	222695487
chr1	222755449	222755858
chr1	222762918	222763683
chr1	222790784	222791127
chr1	222791432	222792580
chr1	222817059	222817527
chr1	222817749	222818220
chr1	222884579	222885388
chr1	222885606	222886316
chr1	222886379	222886575
chr1	222951092	222951124
chr1	222951296	222951525
chr1	222988145	222988972
chr1	223053878	223054277
chr1	223062862	223063074
chr1	223168556	223168720
chr1	223296831	223297100
chr1	223307235	223307814
chr1	223311072	223311139
chr1	223406812	223407187
chr1	223595275	223595394
chr1	223673821	223674189
chr1	223743326	223744184
chr1	223744788	223745007
chr1	223886191	223886311
chr1	223887882	223888054
chr1	223888368	223888570
chr1	223888819	223889075
chr1	223889184	223889481
chr1	223890725	223890957
chr1	223894033	223894206
chr1	223894826	223896562
chr1	223899811	223899901
chr1	223900102	223902252
chr1	223902622	223903098
chr1	223903317	223903454

chr1	223904026	223904192
chr1	223905763	223906004
chr1	223906513	223906569
chr1	223906855	223906950
chr1	223907710	223908296
chr1	223908462	223908833
chr1	223909092	223909461
chr1	223914044	223914526
chr1	223914633	223914973
chr1	223926136	223926511
chr1	223926641	223928485
chr1	223929454	223929654
chr1	223929790	223930049
chr1	223931112	223931157
chr1	223964037	223964129
chr1	224003519	224003742
chr1	224003846	224003965
chr1	224032755	224032824
chr1	224032937	224033678
chr1	224033842	224034665
chr1	224074614	224074701
chr1	224075644	224075779
chr1	224076169	224076246
chr1	224100378	224100663
chr1	224177795	224178015
chr1	224179690	224180306
chr1	224180500	224181026
chr1	224181146	224181736
chr1	224186587	224186704
chr1	224200494	224201047
chr1	224201173	224202121
chr1	224202286	224202485
chr1	224253069	224253083
chr1	224253508	224253574
chr1	224267387	224267637
chr1	224267768	224267837
chr1	224294344	224294574
chr1	224301527	224301695
chr1	224301819	224302430
chr1	224303951	224304139
chr1	224320575	224320652
chr1	224320922	224321071
chr1	224321533	224321595
chr1	224321720	224321789
chr1	224361251	224361269
chr1	224369979	224370268

chr1	224370388	224371879
chr1	224392440	224392561
chr1	224400671	224400833
chr1	224407272	224407304
chr1	224407420	224407511
chr1	224447888	224448034
chr1	224454657	224454706
chr1	224454940	224454984
chr1	224488067	224488173
chr1	224517533	224518439
chr1	224521066	224521137
chr1	224528629	224528871
chr1	224544340	224544409
chr1	224544568	224545733
chr1	224620960	224622086
chr1	224622521	224623466
chr1	224623732	224624110
chr1	224638670	224638687
chr1	224642702	224642802
chr1	224644924	224645024
chr1	224645178	224645353
chr1	224670739	224671077
chr1	224671257	224671374
chr1	224685413	224686693
chr1	224686806	224686855
chr1	224690549	224691120
chr1	224690286	224690419
chr1	224691250	224691452
chr1	224691560	224691971
chr1	225117130	225117906
chr1	225531636	225531766
chr1	225566567	225566737
chr1	225567011	225567041
chr1	225612567	225612620
chr1	225613174	225613448
chr1	225614267	225614466
chr1	225614652	225614886
chr1	225615070	225616655
chr1	225632553	225632652
chr1	225632935	225633074
chr1	225633326	225633726
chr1	225636872	225637069
chr1	225637459	225637515
chr1	225637605	225637809
chr1	225651781	225651856
chr1	225662316	225662515

chr1	225696188	225696241
chr1	225791168	225791252
chr1	225813255	225813493
chr1	225839379	225839993
chr1	225840216	225841051
chr1	225841224	225841936
chr1	225842687	225843204
chr1	225844294	225844657
chr1	225845252	225845675
chr1	225887752	225888433
chr1	225892395	225892569
chr1	225896761	225897031
chr1	225897229	225897273
chr1	225898514	225898615
chr1	225901248	225901395
chr1	225922913	225924557
chr1	225924782	225925287
chr1	225925626	225925825
chr1	225934305	225934311
chr1	225934837	225934982
chr1	225954359	225954409
chr1	225959937	225960074
chr1	225964720	225965209
chr1	225965535	225966214
chr1	225997421	225997667
chr1	225997834	225998295
chr1	226001415	226001741
chr1	226004919	226005730
chr1	226008430	226008457
chr1	226063439	226063750
chr1	226063822	226063845
chr1	226069235	226071246
chr1	226099250	226099462
chr1	226103860	226104020
chr1	226105298	226105615
chr1	226106399	226106598
chr1	226107111	226107306
chr1	226110377	226110759
chr1	226110831	226111052
chr1	226111143	226111978
chr1	226112277	226112727
chr1	226123776	226123951
chr1	226124064	226124198
chr1	226131403	226131440
chr1	226151474	226151646
chr1	226151758	226151947

chr1	226155787	226155958
chr1	226163581	226163651
chr1	226187252	226187568
chr1	226193653	226193881
chr1	226249211	226250931
chr1	226251247	226251381
chr1	226251971	226252046
chr1	226252233	226252258
chr1	226252511	226252856
chr1	226270551	226271976
chr1	226276904	226277075
chr1	226283640	226283917
chr1	226284121	226284414
chr1	226285556	226285658
chr1	226285851	226286186
chr1	226286464	226286671
chr1	226297787	226298861
chr1	226303199	226303747
chr1	226308914	226310812
chr1	226310901	226311301
chr1	226312650	226313011
chr1	226311584	226312377
chr1	226313796	226313991
chr1	226314560	226317026
chr1	226319052	226319238
chr1	226337533	226337554
chr1	226369655	226370555
chr1	226371423	226371711
chr1	226373700	226374861
chr1	226384673	226384677
chr1	226459848	226460367
chr1	226496013	226497393
chr1	226497542	226497672
chr1	226577739	226577938
chr1	226594193	226594438
chr1	226594615	226594844
chr1	226595169	226595175
chr1	226595315	226595852
chr1	226596021	226596447
chr1	226736353	226736724
chr1	226790827	226791014
chr1	226812620	226812730
chr1	226845789	226845894
chr1	226851194	226851310
chr1	226889825	226889846
chr1	226890594	226891540

chr1	226925188	226925602
chr1	226925827	226927214
chr1	226950931	226951130
chr1	226951405	226951668
chr1	226951234	226951303
chr1	227002355	227002864
chr1	227015696	227015889
chr1	227016089	227016190
chr1	227019864	227020069
chr1	227027578	227027641
chr1	227058036	227058755
chr1	227126957	227127628
chr1	227127953	227128578
chr1	227161953	227162353
chr1	227231508	227231528
chr1	227231698	227231750
chr1	227318957	227319228
chr1	227413464	227413530
chr1	227487448	227487840
chr1	227500144	227500489
chr1	227503749	227503838
chr1	227505426	227506911
chr1	227531713	227531726
chr1	227532091	227532157
chr1	227544280	227544320
chr1	227544660	227544785
chr1	227544864	227545237
chr1	227545481	227545738
chr1	227625431	227625707
chr1	227729637	227730814
chr1	227751210	227751862
chr1	227915490	227915736
chr1	227915920	227916637
chr1	227922130	227922932
chr1	227921736	227921937
chr1	227923150	227923917
chr1	227952286	227952348
chr1	227952485	227952614
chr1	228074037	228074392
chr1	228097736	228098248
chr1	228122820	228123070
chr1	228135214	228135600
chr1	228153385	228153595
chr1	228168970	228169149
chr1	228194290	228194489
chr1	228194787	228195353

chr1	228200649	228200765
chr1	228201176	228201738
chr1	228202161	228202730
chr1	228202902	228203175
chr1	228225633	228225963
chr1	228248299	228248885
chr1	228268983	228271677
chr1	228271877	228272484
chr1	228272691	228272889
chr1	228273614	228273990
chr1	228274432	228274606
chr1	228274773	228274972
chr1	228275374	228275941
chr1	228290168	228291638
chr1	228296530	228296967
chr1	228297177	228298088
chr1	228318556	228318778
chr1	228319196	228319301
chr1	228326955	228327281
chr1	228327387	228327601
chr1	228327992	228330478
chr1	228330713	228330967
chr1	228340977	228341207
chr1	228345571	228346057
chr1	228346494	228346879
chr1	228349293	228351456
chr1	228352607	228353188
chr1	228353339	228354264
chr1	228354706	228354727
chr1	228400763	228401210
chr1	228401423	228401900
chr1	228415885	228416611
chr1	228417038	228417059
chr1	228463127	228463725
chr1	228565931	228567119
chr1	228592122	228592253
chr1	228592911	228593361
chr1	228593711	228594747
chr1	228595082	228595504
chr1	228604027	228605115
chr1	228605340	228605554
chr1	228633501	228634019
chr1	228644917	228645544
chr1	228645945	228647282
chr1	228674011	228674945
chr1	228675072	228676196

chr1	228676647	228676717
chr1	228688994	228689239
chr1	228744354	228746880
chr1	228747284	228747504
chr1	228747662	228750097
chr1	228750236	228751523
chr1	228751749	228752770
chr1	228753334	228753707
chr1	228754626	228755927
chr1	228756850	228757150
chr1	228757470	228758461
chr1	228758735	228759454
chr1	228759690	228760581
chr1	228761010	228762736
chr1	228762939	228765020
chr1	228765417	228767872
chr1	228768021	228768764
chr1	228769008	228769467
chr1	228769590	228771049
chr1	228771187	228771667
chr1	228772216	228773288
chr1	228773494	228775469
chr1	228775732	228778370
chr1	228778628	228779437
chr1	228779998	228782174
chr1	228782460	228782940
chr1	228800397	228800522
chr1	228974506	228974646
chr1	229163154	229163373
chr1	229252854	229252920
chr1	229253430	229253480
chr1	229253982	229254132
chr1	229254226	229254529
chr1	229327757	229327778
chr1	229361542	229361766
chr1	229365379	229365565
chr1	229366899	229367190
chr1	229367656	229367855
chr1	229374444	229374460
chr1	229404173	229404208
chr1	229404425	229404606
chr1	229405591	229405837
chr1	229406636	229407619
chr1	229408019	229408404
chr1	229432023	229432216
chr1	229432594	229432758

chr1	229477054	229477259
chr1	229477606	229479024
chr1	229479333	229479841
chr1	229643623	229644084
chr1	229644191	229644532
chr1	229644596	229644795
chr1	229693702	229694735
chr1	229694954	229695397
chr1	229715138	229715199
chr1	229760751	229761266
chr1	229761596	229761912
chr1	229762116	229762583
chr1	230202499	230202858
chr1	230202980	230203488
chr1	230203657	230204263
chr1	230204608	230204655
chr1	230204800	230204968
chr1	230219553	230220166
chr1	230220304	230221404
chr1	230222657	230223189
chr1	230226084	230226278
chr1	230226444	230226464
chr1	230227388	230227711
chr1	230239442	230239484
chr1	230239631	230239762
chr1	230240213	230240409
chr1	230240763	230241171
chr1	230241468	230241716
chr1	230241896	230242032
chr1	230258032	230258072
chr1	230258329	230258536
chr1	230268125	230268381
chr1	230269741	230269743
chr1	230269888	230270096
chr1	230276391	230276513
chr1	230276644	230276706
chr1	230463077	230463501
chr1	230463765	230464038
chr1	230464149	230464243
chr1	230464404	230464899
chr1	230496100	230496379
chr1	230504083	230504454
chr1	230561152	230561560
chr1	230594249	230594349
chr1	230594559	230594605
chr1	230595219	230595239

chr1	230595546	230595634
chr1	230777707	230777923
chr1	230778241	230778465
chr1	230778728	230778758
chr1	230817714	230817832
chr1	230819879	230819892
chr1	230833797	230833933
chr1	230905269	230905400
chr1	230908863	230909204
chr1	230960458	230961409
chr1	231003631	231004125
chr1	231004681	231005024
chr1	231113950	231114618
chr1	231115014	231115377
chr1	231152025	231152055
chr1	231152951	231153296
chr1	231156300	231156584
chr1	231174841	231174875
chr1	231175185	231176964
chr1	231307384	231307532
chr1	231307729	231307837
chr1	231308141	231308243
chr1	231376497	231377759
chr1	231471616	231471757
chr1	231472763	231472858
chr1	231473711	231474322
chr1	231475027	231475567
chr1	231475822	231476540
chr1	231474538	231474598
chr1	231494017	231494100
chr1	231531112	231531166
chr1	231531467	231531795
chr1	231543626	231543917
chr1	231550477	231550624
chr1	231550788	231550984
chr1	231556075	231556090
chr1	231556369	231556423
chr1	231556518	231558644
chr1	231559439	231559538
chr1	231663888	231664692
chr1	231719884	231720001
chr1	231739144	231739388
chr1	231739591	231739851
chr1	231762442	231763239
chr1	231764895	231765036
chr1	231909089	231909189

chr1	232357473	232357516
chr1	232396568	232396597
chr1	232396998	232397177
chr1	232454716	232454805
chr1	232470149	232470376
chr1	232558634	232558717
chr1	232644625	232644807
chr1	232710783	232711049
chr1	232763359	232763713
chr1	232765256	232765561
chr1	232766262	232766627
chr1	232781560	232781736
chr1	232781945	232781957
chr1	232806425	232806494
chr1	232840731	232840892
chr1	232841029	232841593
chr1	232842900	232843074
chr1	232881675	232881801
chr1	232903070	232903285
chr1	232903433	232903840
chr1	232940911	232941699
chr1	233004969	233005193
chr1	233085861	233086254
chr1	233086379	233086499
chr1	233086913	233087199
chr1	233221311	233221718
chr1	233243386	233243738
chr1	233248415	233248625
chr1	233248886	233249397
chr1	233249505	233249708
chr1	233250021	233250295
chr1	233315356	233315476
chr1	233336145	233336549
chr1	233427847	233428302
chr1	233430603	233431714
chr1	233463408	233464919
chr1	233503317	233503473
chr1	233560588	233560687
chr1	233560906	233560943
chr1	233608847	233609023
chr1	233749212	233749691
chr1	233749819	233751390
chr1	233804452	233804951
chr1	233824816	233824936
chr1	233843695	233843816
chr1	233844349	233844568

chr1	234040151	234040781
chr1	234040996	234041304
chr1	234044800	234044962
chr1	234045235	234045380
chr1	234270644	234270746
chr1	234275473	234275687
chr1	234275961	234276090
chr1	234342666	234342865
chr1	234396595	234396742
chr1	234492507	234492649
chr1	234492842	234492994
chr1	234505457	234505898
chr1	234508213	234508412
chr1	234508696	234509792
chr1	234534674	234534949
chr1	234539176	234539245
chr1	234613779	234613851
chr1	234614055	234614805
chr1	234615013	234615188
chr1	234622884	234623059
chr1	234625042	234625267
chr1	234625384	234625445
chr1	234630876	234631151
chr1	234631315	234631663
chr1	234636771	234636938
chr1	234634703	234635727
chr1	234637060	234637251
chr1	234641271	234641900
chr1	234644870	234644961
chr1	234645182	234645501
chr1	234645579	234646062
chr1	234651117	234651541
chr1	234651626	234651973
chr1	234659262	234659369
chr1	234668737	234668881
chr1	234669080	234669338
chr1	234669707	234669866
chr1	234670007	234670038
chr1	234672791	234672854
chr1	234674015	234674253
chr1	234674445	234675373
chr1	234679333	234679640
chr1	234679782	234680479
chr1	234680660	234680795
chr1	234700042	234700867
chr1	234724980	234725264

chr1	234725397	234725796
chr1	234734809	234734940
chr1	234735098	234735183
chr1	234735363	234736353
chr1	234737669	234737733
chr1	234738463	234738918
chr1	234741630	234742088
chr1	234742852	234745432
chr1	234745735	234747922
chr1	234748159	234748479
chr1	234748596	234748907
chr1	234749149	234749473
chr1	234752448	234752647
chr1	234754160	234755095
chr1	234783511	234783733
chr1	234783856	234783939
chr1	234792117	234792941
chr1	234793165	234793625
chr1	234793808	234793967
chr1	234794080	234794515
chr1	234795223	234795240
chr1	234819918	234820009
chr1	234820294	234820335
chr1	234858768	234859071
chr1	234859256	234860001
chr1	234860215	234860936
chr1	234861158	234861232
chr1	234861524	234861731
chr1	234861993	234862192
chr1	234862317	234862655
chr1	234862960	234863370
chr1	234863956	234864008
chr1	234908021	234908051
chr1	234908619	234908760
chr1	234912707	234912956
chr1	234913092	234913295
chr1	234913414	234913671
chr1	234918323	234918594
chr1	234936689	234936980
chr1	235001636	235002253
chr1	235002627	235002637
chr1	235002797	235002865
chr1	235021711	235021926
chr1	235061809	235062046
chr1	235086538	235086816
chr1	235105573	235105958

chr1	235106435	235106781
chr1	235107009	235107208
chr1	235114576	235114984
chr1	235116205	235116518
chr1	235116642	235117217
chr1	235132795	235133056
chr1	235133351	235133645
chr1	235133736	235134626
chr1	235147281	235147305
chr1	235147419	235147618
chr1	235147908	235148653
chr1	235153822	235153946
chr1	235242349	235242914
chr1	235243247	235243379
chr1	235246436	235246635
chr1	235246940	235247523
chr1	235248447	235248538
chr1	235257490	235257506
chr1	235267769	235267968
chr1	235268807	235268921
chr1	235291103	235292077
chr1	235292364	235292576
chr1	235298741	235298815
chr1	235322343	235322506
chr1	235324071	235325358
chr1	235416502	235416771
chr1	235476604	235476605
chr1	235476889	235476991
chr1	235480550	235480595
chr1	235489986	235491496
chr1	235491857	235492880
chr1	235493562	235493908
chr1	235511493	235511798
chr1	235530368	235530516
chr1	235530841	235531373
chr1	235555227	235555579
chr1	235561088	235561534
chr1	235566966	235567321
chr1	235567420	235567996
chr1	235575052	235575837
chr1	235580024	235580253
chr1	235580709	235580720
chr1	235606116	235606223
chr1	235606380	235606502
chr1	235632279	235632672
chr1	235633104	235633467

chr1	235664531	235664864
chr1	235666626	235666870
chr1	235666958	235667759
chr1	235668011	235668955
chr1	235695954	235696030
chr1	235752207	235752462
chr1	235813021	235813419
chr1	235813553	235813568
chr1	235894976	235895069
chr1	235902766	235902830
chr1	235904639	235904770
chr1	236029341	236031048
chr1	236071925	236072529
chr1	236073591	236074351
chr1	236076614	236076839
chr1	236076971	236076993
chr1	236080560	236081287
chr1	236081462	236081702
chr1	236083175	236083805
chr1	236086633	236086852
chr1	236095017	236095354
chr1	236095708	236095886
chr1	236096198	236096716
chr1	236096779	236096992
chr1	236107874	236108073
chr1	236134968	236135569
chr1	236162685	236162881
chr1	236163140	236163319
chr1	236173261	236173375
chr1	236173617	236173738
chr1	236180538	236180602
chr1	236184364	236184484
chr1	236227743	236228376
chr1	236252754	236252826
chr1	236260272	236260851
chr1	236269248	236269652
chr1	236270834	236270875
chr1	236272377	236272776
chr1	236304634	236304874
chr1	236305291	236305610
chr1	236305810	236306721
chr1	236444440	236445267
chr1	236445517	236445894
chr1	236466291	236466292
chr1	236558174	236559423
chr1	236571883	236572121

chr1	236584266	236584382
chr1	236618477	236618624
chr1	236618875	236619191
chr1	236628633	236628901
chr1	236629293	236629358
chr1	236629615	236629757
chr1	236665849	236665855
chr1	236666141	236666182
chr1	236666361	236666832
chr1	236680559	236680677
chr1	236680787	236681075
chr1	236686777	236687770
chr1	236687873	236688056
chr1	236694936	236695017
chr1	236695418	236695542
chr1	236704134	236704785
chr1	236767588	236768389
chr1	236849403	236850327
chr1	236897498	236897721
chr1	236958632	236959152
chr1	236959241	236959324
chr1	236959525	236959551
chr1	236963537	236963875
chr1	238216064	238216183
chr1	238225547	238225672
chr1	241473800	241473813
chr1	241520332	241520823
chr1	241587235	241587476
chr1	241587597	241587796
chr1	241628971	241628999
chr1	241629284	241629373
chr1	241646047	241646164
chr1	241681798	241682081
chr1	241682289	241683009
chr1	241683367	241683462
chr1	241694866	241694934
chr1	241754615	241754720
chr1	241755502	241755624
chr1	241771783	241772065
chr1	241775887	241776040
chr1	241776273	241776293
chr1	241802913	241804053
chr1	241805082	241805402
chr1	241821710	241822064
chr1	241852076	241852660
chr1	241865743	241865880

chr1	241866106	241866120
chr1	241866309	241866329
chr1	241870648	241870756
chr1	241912176	241912208
chr1	241966172	241966238
chr1	242001456	242001667
chr1	242010970	242011498
chr1	242011616	242011879
chr1	242087204	242087505
chr1	242148844	242148895
chr1	242149062	242149072
chr1	242149152	242149261
chr1	243264420	243264437
chr1	243264716	243265023
chr1	243268540	243268672
chr1	243418069	243419154
chr1	243419505	243419813
chr1	243428016	243428030
chr1	243430988	243431269
chr1	243451001	243451338
chr1	243458218	243458417
chr1	243645388	243645422
chr1	243761696	243761769
chr1	243822196	243822319
chr1	243882579	243882585
chr1	243893732	243893863
chr1	243894022	243894198
chr1	243897503	243897576
chr1	243897764	243898099
chr1	244012305	244012352
chr1	244012799	244014735
chr1	244014821	244015020
chr1	244045682	244045711
chr1	244079351	244079807
chr1	244079946	244081013
chr1	244081146	244081286
chr1	244081359	244081479
chr1	244081589	244081624
chr1	244087718	244087981
chr1	244090144	244090661
chr1	244108093	244108185
chr1	244210490	244211455
chr1	244212648	244213006
chr1	244262707	244262899
chr1	244326817	244327114
chr1	244327655	244327766

chr1	244361056	244361093
chr1	244361521	244361681
chr1	244394416	244395353
chr1	244402411	244402926
chr1	244436617	244436655
chr1	244436975	244437190
chr1	244437337	244437674
chr1	244437949	244438033
chr1	244462707	244463415
chr1	244466181	244466380
chr1	244485973	244486748
chr1	244504919	244505626
chr1	244516993	244517204
chr1	244532191	244532567
chr1	244532828	244532850
chr1	244533175	244533358
chr1	244534487	244535173
chr1	244535435	244535512
chr1	244545297	244545472
chr1	244556618	244556734
chr1	244599130	244599437
chr1	244614219	244615423
chr1	244615601	244615752
chr1	244623852	244623874
chr1	244624395	244625107
chr1	244763414	244763675
chr1	244815778	244815994
chr1	244816370	244816885
chr1	244817247	244817520
chr1	244893333	244893496
chr1	244893603	244894278
chr1	244898675	244898824
chr1	244903622	244903732
chr1	244912419	244912613
chr1	244997111	244997469
chr1	244997617	244999731
chr1	245026158	245026233
chr1	245026469	245027833
chr1	245028037	245028228
chr1	245028291	245028620
chr1	245028842	245028903
chr1	245029027	245029115
chr1	245081962	245082288
chr1	245132725	245134852
chr1	245135720	245135828
chr1	245248751	245248762

chr1	245249026	245249216
chr1	245249768	245250104
chr1	245254265	245254475
chr1	245254721	245255887
chr1	245270202	245270240
chr1	245317246	245317652
chr1	245317855	245318271
chr1	245318526	245318946
chr1	245319578	245319816
chr1	245319940	245320386
chr1	245830485	245830616
chr1	245951460	245951692
chr1	245986136	245986416
chr1	246302954	246303098
chr1	246303347	246303429
chr1	246324932	246325082
chr1	246329500	246329699
chr1	246329965	246330163
chr1	246459285	246459349
chr1	246487826	246488066
chr1	246660686	246660912
chr1	246666967	246667112
chr1	246669858	246670057
chr1	246670262	246670500
chr1	246670654	246671118
chr1	246729087	246729539
chr1	246729772	246730482
chr1	246730712	246730992
chr1	246732963	246733206
chr1	246751010	246751296
chr1	246751646	246751996
chr1	246772552	246772727
chr1	246796177	246796699
chr1	246853061	246853262
chr1	246862258	246862638
chr1	246887173	246887355
chr1	246887708	246888042
chr1	246932265	246932394
chr1	246932859	246933130
chr1	247000971	247001023
chr1	247072607	247072758
chr1	247093475	247093740
chr1	247094243	247094368
chr1	247094491	247095884
chr1	247170794	247171331
chr1	247171600	247171713

chr1	247241632	247241875
chr1	247267103	247267681
chr1	247273852	247274080
chr1	247274198	247275756
chr1	247289694	247290356
chr1	247334809	247335309
chr1	247373365	247373402
chr1	247373776	247374206
chr1	247494057	247494491
chr1	247494738	247495093
chr1	247495253	247495959
chr1	247496219	247496787
chr1	247497111	247497373
chr1	247506442	247506648
chr1	247536273	247536527
chr1	247611380	247611582
chr1	249119406	249121124
chr1	249123001	249123043
chr1	249123451	249123501
chr1	249131733	249131848
chr1	249132003	249133904
chr1	249136910	249137648
chr1	249139111	249139372
chr1	249151779	249153267
chr1	249153412	249153632
chr1	249153955	249154138
chr1	249157147	249157346
chr1	249157629	249157956
chr1	249166732	249168013
chr1	249199895	249200301
chr1	249200523	249200753
chr1	249200931	249201352
chr1	249240029	249240427
chr10	122449	122761
chr10	179464	179583
chr10	180513	182280
chr10	369757	369903
chr10	383428	383637
chr10	384096	384519
chr10	384674	384881
chr10	524736	525479
chr10	530473	531060
chr10	831085	831284
chr10	976890	977659
chr10	978024	978357
chr10	1032590	1032772

chr10	1033133	1033281
chr10	1033535	1033883
chr10	1034042	1034287
chr10	1034459	1034642
chr10	1081808	1082007
chr10	1082114	1082350
chr10	1094414	1094860
chr10	1095511	1095878
chr10	1096503	1096573
chr10	1100792	1101087
chr10	1101733	1102952
chr10	1105688	1106049
chr10	1106247	1106319
chr10	1996895	1996905
chr10	2154054	2154115
chr10	2154605	2154649
chr10	2159840	2160032
chr10	2160172	2160228
chr10	2161788	2161973
chr10	3108908	3109017
chr10	3109439	3109645
chr10	3109754	3110986
chr10	3111090	3111473
chr10	3122483	3122502
chr10	3122925	3123013
chr10	3132669	3132947
chr10	3133120	3133457
chr10	3133600	3134122
chr10	3136216	3136415
chr10	3137633	3138231
chr10	3138345	3138710
chr10	3139604	3139692
chr10	3141403	3141813
chr10	3148808	3149670
chr10	3149786	3150255
chr10	3173623	3173774
chr10	3215300	3215545
chr10	3215674	3215883
chr10	3268042	3268100
chr10	3269183	3269230
chr10	3269951	3270020
chr10	3270162	3270301
chr10	3291383	3291422
chr10	3466468	3466817
chr10	3494519	3494763
chr10	3495121	3495260

chr10	3495433	3495545
chr10	3497527	3497813
chr10	3509523	3510223
chr10	3511185	3511595
chr10	3514872	3515196
chr10	3542964	3542985
chr10	3598436	3598803
chr10	3666580	3666587
chr10	3666834	3666937
chr10	3677937	3678277
chr10	3678474	3678931
chr10	3679227	3679305
chr10	3710538	3710903
chr10	3711036	3711177
chr10	3763095	3763181
chr10	3782287	3782390
chr10	3782551	3782754
chr10	3782970	3783158
chr10	3791693	3791807
chr10	3792139	3792504
chr10	3796958	3797358
chr10	3799548	3800593
chr10	3805119	3805318
chr10	3806081	3806416
chr10	3817493	3817835
chr10	3818140	3818603
chr10	3818999	3819729
chr10	3821323	3821761
chr10	3822156	3823097
chr10	3823515	3823793
chr10	3823949	3824337
chr10	3824524	3825879
chr10	3825996	3826341
chr10	3826931	3827420
chr10	3827718	3828415
chr10	3828631	3828677
chr10	3828800	3829147
chr10	3847455	3847761
chr10	3848174	3848430
chr10	3851810	3852790
chr10	3853497	3853569
chr10	3853686	3853950
chr10	3867369	3867389
chr10	3867742	3867847
chr10	3893733	3894143
chr10	3894455	3895049

chr10	3928920	3929209
chr10	3930011	3930597
chr10	3940957	3941409
chr10	3966654	3966853
chr10	4111773	4111972
chr10	4285881	4285963
chr10	4292205	4292375
chr10	4292683	4292742
chr10	4386584	4386630
chr10	4555123	4555398
chr10	4575816	4575927
chr10	4576044	4576209
chr10	4583899	4583965
chr10	4705261	4705426
chr10	4705602	4705946
chr10	4713808	4713981
chr10	4719821	4720456
chr10	4734026	4734165
chr10	4734282	4734342
chr10	4768269	4768363
chr10	4768568	4768607
chr10	4812934	4812999
chr10	4813306	4813340
chr10	4850378	4850521
chr10	4850653	4850750
chr10	4855402	4855521
chr10	4868410	4869285
chr10	4985400	4985488
chr10	4989785	4990036
chr10	4990163	4990280
chr10	5005343	5005468
chr10	5046208	5046292
chr10	5061049	5061160
chr10	5113408	5113596
chr10	5113956	5114230
chr10	5294475	5294664
chr10	5326331	5326768
chr10	5333623	5333766
chr10	5333983	5334300
chr10	5334410	5334617
chr10	5335517	5335976
chr10	5360019	5360182
chr10	5454268	5454322
chr10	5454526	5455069
chr10	5488257	5488394
chr10	5488539	5488853

chr10	5491850	5491871
chr10	5508282	5508522
chr10	5508794	5508944
chr10	5529897	5530283
chr10	5566585	5566990
chr10	5567434	5568027
chr10	5580414	5580565
chr10	5580853	5581205
chr10	5587891	5587920
chr10	5589151	5589427
chr10	5590027	5590424
chr10	5591170	5591370
chr10	5592821	5593246
chr10	5593409	5593622
chr10	5608510	5608644
chr10	5638412	5638584
chr10	5638816	5638870
chr10	5651805	5651898
chr10	5708146	5708541
chr10	5726828	5727796
chr10	5733739	5734131
chr10	5734403	5735182
chr10	5762197	5762235
chr10	5762440	5762461
chr10	5842867	5843267
chr10	5854593	5854631
chr10	5854764	5855348
chr10	5855631	5855929
chr10	5930547	5930762
chr10	5931109	5931765
chr10	5932107	5932803
chr10	5986668	5986798
chr10	5987668	5987836
chr10	5987971	5988179
chr10	5992699	5993652
chr10	6013241	6013465
chr10	6017872	6017946
chr10	6018175	6019643
chr10	6019756	6020057
chr10	6092649	6092754
chr10	6092938	6093026
chr10	6130952	6132002
chr10	6183643	6183858
chr10	6186567	6187662
chr10	6199202	6199556
chr10	6205850	6206308

chr10	6243237	6243660
chr10	6244880	6246304
chr10	6260527	6261071
chr10	6281029	6281192
chr10	6317451	6317666
chr10	6341026	6341213
chr10	6438737	6438783
chr10	6438965	6439065
chr10	6443417	6443420
chr10	6443676	6443878
chr10	6534023	6534399
chr10	6544183	6544359
chr10	6621964	6622795
chr10	6763409	6763805
chr10	6764047	6764087
chr10	6764318	6764531
chr10	6771009	6771103
chr10	6778936	6779109
chr10	6779349	6779591
chr10	6780089	6780329
chr10	6781703	6782104
chr10	6820913	6820993
chr10	7829756	7829984
chr10	7830122	7830563
chr10	7860124	7860183
chr10	10437039	10437096
chr10	10801919	10802146
chr10	10827982	10828065
chr10	10828188	10828651
chr10	10834605	10834998
chr10	10839100	10839246
chr10	10839450	10839468
chr10	10839567	10839728
chr10	10908192	10908245
chr10	10953662	10953871
chr10	11078683	11078725
chr10	11594261	11594520
chr10	11653045	11653532
chr10	11653707	11653884
chr10	11692171	11692321
chr10	11727025	11727371
chr10	11784038	11784204
chr10	11784464	11785160
chr10	11799480	11799532
chr10	11800242	11800743
chr10	11824849	11825126

chr10	11825287	11825492
chr10	11834991	11835251
chr10	11835386	11835604
chr10	11835799	11835819
chr10	11836327	11836485
chr10	11837388	11837506
chr10	11852159	11852434
chr10	11859465	11859619
chr10	11861204	11861416
chr10	11862582	11862886
chr10	11862000	11862233
chr10	11863007	11863272
chr10	11865147	11865576
chr10	11865693	11865778
chr10	11865889	11866058
chr10	11866931	11867167
chr10	11867427	11867438
chr10	11880386	11880737
chr10	11916455	11916684
chr10	11917361	11917882
chr10	11918137	11918358
chr10	11920891	11921033
chr10	11922898	11923275
chr10	11923353	11924139
chr10	11924384	11924517
chr10	11926268	11927713
chr10	11936343	11936470
chr10	11940110	11940401
chr10	11940563	11940862
chr10	11943949	11944170
chr10	11957973	11958391
chr10	12083847	12084205
chr10	12084449	12084801
chr10	12085404	12085610
chr10	12095555	12095570
chr10	12095721	12095895
chr10	12109408	12109700
chr10	12110490	12110565
chr10	12110984	12111190
chr10	12171036	12171370
chr10	12171612	12172071
chr10	12223604	12223844
chr10	12228583	12228734
chr10	12235899	12236230
chr10	12236661	12237128
chr10	12237372	12237778

chr10	12237881	12238580
chr10	12262648	12262919
chr10	12264134	12264208
chr10	12284191	12284272
chr10	12312822	12312955
chr10	12313357	12313510
chr10	12323670	12323677
chr10	12371117	12371348
chr10	12390662	12390897
chr10	12391185	12391974
chr10	12392460	12392765
chr10	12745805	12745959
chr10	12747978	12748367
chr10	12749726	12749780
chr10	12751490	12751805
chr10	12761351	12761525
chr10	12761651	12761836
chr10	12762248	12762419
chr10	12762811	12762894
chr10	12767750	12767869
chr10	12875820	12875870
chr10	12970189	12970344
chr10	13141071	13141155
chr10	13141747	13142655
chr10	13142984	13143049
chr10	13190707	13190829
chr10	13203160	13203310
chr10	13341297	13342357
chr10	13343940	13344332
chr10	13344608	13344678
chr10	13389074	13390072
chr10	13390466	13390921
chr10	13425854	13425879
chr10	13628952	13629125
chr10	13629438	13629724
chr10	13732172	13732633
chr10	13749293	13749538
chr10	13750944	13751081
chr10	13812339	13812494
chr10	13815022	13815145
chr10	13819366	13819410
chr10	13819925	13820102
chr10	13829046	13829125
chr10	13829372	13829540
chr10	13829771	13829851
chr10	13908572	13908598

chr10	13915964	13916012
chr10	14227213	14227233
chr10	14428469	14428533
chr10	14645783	14645952
chr10	14646068	14646271
chr10	14824558	14824731
chr10	14851180	14851342
chr10	14851605	14851702
chr10	14879741	14880039
chr10	14880382	14880722
chr10	14880891	14881169
chr10	14919812	14919890
chr10	14920109	14920732
chr10	14920891	14921309
chr10	14994994	14995023
chr10	14995335	14996380
chr10	14996495	14996635
chr10	15001060	15001177
chr10	15001460	15002322
chr10	15138801	15139608
chr10	15139770	15139921
chr10	15165747	15165950
chr10	15166091	15166257
chr10	15209998	15210653
chr10	15242746	15242756
chr10	15242995	15243089
chr10	15243695	15243969
chr10	15244294	15244300
chr10	15244433	15244614
chr10	15901815	15902113
chr10	16479094	16479401
chr10	16563120	16563673
chr10	16858966	16859379
chr10	16859972	16860098
chr10	17104975	17105170
chr10	17117726	17117901
chr10	17242935	17243134
chr10	17243230	17243864
chr10	17258191	17258206
chr10	17270617	17270865
chr10	17271363	17271587
chr10	17272218	17272437
chr10	17390571	17390808
chr10	17391071	17391142
chr10	17496465	17496782
chr10	17554333	17554895

chr10	17569503	17569871
chr10	17655765	17655813
chr10	17658563	17659435
chr10	17659667	17660050
chr10	17685372	17686038
chr10	17686241	17686794
chr10	17687951	17688369
chr10	18899733	18899879
chr10	18916494	18916700
chr10	18940443	18940511
chr10	18940925	18940963
chr10	18947948	18948768
chr10	18949120	18949433
chr10	18955807	18955922
chr10	18956103	18956151
chr10	18971112	18971117
chr10	21106610	21106820
chr10	21128464	21128483
chr10	21318994	21319111
chr10	21367994	21368110
chr10	21462551	21463720
chr10	21473154	21473212
chr10	21581704	21582179
chr10	21584663	21584862
chr10	21593229	21593290
chr10	21601766	21601814
chr10	21603882	21605315
chr10	21605783	21605995
chr10	21618843	21619363
chr10	21622754	21623102
chr10	21623848	21624120
chr10	21624225	21624415
chr10	21625301	21626203
chr10	21631363	21631608
chr10	21635934	21636072
chr10	21644169	21645054
chr10	21651844	21652113
chr10	21652437	21652989
chr10	21653289	21653483
chr10	21653595	21653989
chr10	21654907	21655472
chr10	21657889	21658408
chr10	21658769	21658968
chr10	21661747	21662323
chr10	21687426	21687692
chr10	21687801	21687900

chr10	21688905	21689041
chr10	21698568	21698948
chr10	21707856	21708265
chr10	21716200	21716244
chr10	21716651	21716805
chr10	21717019	21717232
chr10	21783492	21786178
chr10	21805455	21805797
chr10	21806122	21806485
chr10	21806812	21806928
chr10	21807206	21808089
chr10	21808238	21808277
chr10	21808842	21809364
chr10	21811204	21811400
chr10	21813797	21813852
chr10	21813977	21815716
chr10	21815944	21816662
chr10	21822348	21823853
chr10	21824204	21824403
chr10	21844185	21844618
chr10	21870463	21870607
chr10	21890034	21890104
chr10	22013615	22013631
chr10	22099832	22100194
chr10	22100561	22100599
chr10	22215951	22216291
chr10	22218013	22218103
chr10	22218235	22218370
chr10	22221010	22221209
chr10	22231408	22231825
chr10	22242482	22242503
chr10	22291497	22291526
chr10	22291679	22292692
chr10	22292801	22293004
chr10	22517893	22518412
chr10	22518622	22518643
chr10	22535398	22535824
chr10	22540653	22541016
chr10	22541264	22541664
chr10	22605088	22605183
chr10	22605365	22605949
chr10	22610124	22610627
chr10	22623252	22623895
chr10	22629495	22630086
chr10	22716812	22717007
chr10	22717122	22717333

chr10	22725938	22726646
chr10	22726711	22727050
chr10	22875199	22875249
chr10	22894368	22894438
chr10	23002122	23002824
chr10	23003647	23003851
chr10	23105515	23105680
chr10	23105802	23105939
chr10	23106161	23106259
chr10	23114147	23114308
chr10	23384378	23385540
chr10	23614051	23614187
chr10	23620539	23621241
chr10	23627632	23627855
chr10	23628087	23628173
chr10	23632559	23633876
chr10	23634740	23634946
chr10	23635230	23635239
chr10	23640594	23641331
chr10	23654328	23654832
chr10	23662099	23662190
chr10	23662361	23662445
chr10	23673205	23673331
chr10	23673615	23673807
chr10	23706483	23706565
chr10	23708941	23709373
chr10	23721122	23721181
chr10	23727375	23727747
chr10	23727988	23729845
chr10	23767850	23767891
chr10	23801319	23801435
chr10	24498124	24498323
chr10	24527656	24527969
chr10	24528169	24528214
chr10	24536494	24536789
chr10	24538703	24538968
chr10	24539284	24539340
chr10	24543934	24544197
chr10	24543311	24543496
chr10	24544332	24544531
chr10	24544865	24545396
chr10	24548276	24548532
chr10	24581902	24582045
chr10	24582163	24582269
chr10	24582606	24582716
chr10	24588812	24589000

chr10	24608343	24608363
chr10	24613601	24613726
chr10	24613928	24613946
chr10	24620318	24621188
chr10	24633171	24633353
chr10	24633580	24633637
chr10	24649398	24649628
chr10	24652064	24652358
chr10	24671876	24673146
chr10	24673842	24673872
chr10	24717607	24717876
chr10	24721413	24721744
chr10	24724269	24724617
chr10	24745295	24745418
chr10	24746289	24746739
chr10	24746981	24747180
chr10	24752620	24753066
chr10	24753215	24754014
chr10	24754420	24755182
chr10	24755352	24755607
chr10	24772083	24772285
chr10	24772474	24772624
chr10	24779820	24779831
chr10	24780286	24780339
chr10	24788998	24789197
chr10	24819799	24819900
chr10	24842131	24842179
chr10	24892637	24892660
chr10	24909102	24909114
chr10	24919431	24919895
chr10	24932137	24932263
chr10	24932816	24933090
chr10	24975049	24975271
chr10	24988354	24988576
chr10	24995333	24995393
chr10	25004694	25004786
chr10	25007572	25007748
chr10	25009228	25009554
chr10	25009943	25011132
chr10	25011536	25012475
chr10	25012806	25013585
chr10	25013802	25013993
chr10	25020881	25020961
chr10	25021176	25021216
chr10	25045039	25045251
chr10	25045596	25045603

chr10	25097111	25097326
chr10	25103661	25104327
chr10	25109164	25109534
chr10	25109819	25110022
chr10	25138518	25138706
chr10	25147228	25147236
chr10	25162094	25162445
chr10	25178588	25178681
chr10	25178838	25178906
chr10	25210225	25210626
chr10	25233668	25233791
chr10	25240998	25243000
chr10	25244633	25244954
chr10	25246548	25247133
chr10	25247245	25247416
chr10	25251875	25251966
chr10	25252249	25252351
chr10	25304900	25305330
chr10	25305574	25305898
chr10	25306067	25306434
chr10	25315486	25315737
chr10	25347954	25348353
chr10	25348705	25348864
chr10	25349051	25349415
chr10	25350620	25351546
chr10	25351709	25351913
chr10	25352442	25352491
chr10	25353752	25354046
chr10	25356999	25357260
chr10	25357503	25357656
chr10	26776120	26776173
chr10	26776431	26776518
chr10	26776714	26776854
chr10	26777297	26777443
chr10	26895328	26895409
chr10	26910479	26911022
chr10	26911171	26911736
chr10	26985816	26985908
chr10	26986110	26987091
chr10	26987285	26987514
chr10	26987924	26988168
chr10	27003311	27003548
chr10	27069842	27070031
chr10	27070225	27070970
chr10	27073938	27074032
chr10	27074325	27074577

chr10	27147557	27147885
chr10	27149011	27149903
chr10	27150291	27150398
chr10	27154677	27154914
chr10	27158255	27158462
chr10	27158833	27158928
chr10	27171224	27171298
chr10	27234820	27235394
chr10	27388726	27390171
chr10	27417406	27417563
chr10	27443674	27445244
chr10	27442809	27443573
chr10	27529111	27529778
chr10	27529903	27531076
chr10	27531213	27531451
chr10	27531944	27532367
chr10	27541048	27541308
chr10	27541484	27542714
chr10	27608578	27608981
chr10	27609276	27609354
chr10	27735152	27735596
chr10	27763641	27763854
chr10	27771910	27772590
chr10	27789760	27789936
chr10	27792752	27792989
chr10	27793252	27793738
chr10	27818269	27818598
chr10	27831722	27831879
chr10	27865987	27866426
chr10	27935203	27935224
chr10	28031015	28031326
chr10	28031436	28031635
chr10	28031789	28032007
chr10	28032346	28032896
chr10	28034386	28034740
chr10	28287254	28288142
chr10	28343295	28344186
chr10	28376883	28376931
chr10	28378411	28378771
chr10	28377107	28377216
chr10	28580665	28580736
chr10	28581310	28581316
chr10	28591336	28592278
chr10	28592414	28592457
chr10	28648585	28648633
chr10	28657428	28657713

chr10	28664606	28664653
chr10	28664921	28665028
chr10	28670728	28670918
chr10	28810093	28810296
chr10	28820970	28822970
chr10	28870116	28870200
chr10	28881385	28881632
chr10	28881834	28881854
chr10	28882063	28882172
chr10	28957635	28957875
chr10	28966495	28966798
chr10	29273717	29274037
chr10	29280539	29280658
chr10	29353277	29353452
chr10	29698288	29698348
chr10	29698592	29699081
chr10	29760409	29760537
chr10	29767900	29768060
chr10	29768368	29768516
chr10	29791010	29791018
chr10	29865549	29866073
chr10	29878865	29879476
chr10	29885859	29886046
chr10	29887827	29887956
chr10	29888152	29888214
chr10	29907572	29907886
chr10	29910448	29910514
chr10	29912729	29912909
chr10	29913057	29913111
chr10	29916504	29916617
chr10	29916790	29916875
chr10	29922200	29922545
chr10	29923677	29924503
chr10	29925218	29925569
chr10	29935082	29935280
chr10	29935532	29935787
chr10	29947738	29947780
chr10	29948095	29948534
chr10	29950169	29950534
chr10	29957685	29957732
chr10	29977988	29978348
chr10	30002980	30003073
chr10	30003243	30003852
chr10	30023793	30025472
chr10	30025597	30025828
chr10	30032157	30032372

chr10	30073218	30073477
chr10	30073602	30073810
chr10	30092684	30092809
chr10	30092966	30093165
chr10	30096309	30096649
chr10	30096764	30096834
chr10	30100900	30101002
chr10	30127827	30127899
chr10	30128624	30128796
chr10	30129081	30129502
chr10	30129570	30129904
chr10	30147345	30147746
chr10	30151792	30151920
chr10	30152217	30152535
chr10	30235818	30236493
chr10	30236644	30236855
chr10	30237809	30238155
chr10	30238391	30238966
chr10	30239190	30239389
chr10	30240376	30240549
chr10	30240688	30241197
chr10	30316325	30316597
chr10	30509066	30509435
chr10	30637824	30638147
chr10	30638383	30638585
chr10	30648798	30649025
chr10	30676440	30676705
chr10	30707722	30707771
chr10	30711968	30712312
chr10	30712667	30712743
chr10	30720381	30720889
chr10	30721599	30723888
chr10	30724014	30724404
chr10	30725243	30725445
chr10	30735980	30736011
chr10	30737601	30737847
chr10	30736629	30736697
chr10	30762703	30762723
chr10	30823488	30823578
chr10	30843829	30844363
chr10	30844853	30844899
chr10	30845014	30845052
chr10	30845133	30845232
chr10	30845364	30845489
chr10	30846716	30847200
chr10	30872615	30872743

chr10	30971659	30971726
chr10	31073045	31073145
chr10	31073825	31074111
chr10	31274973	31275067
chr10	31293869	31293925
chr10	31320548	31321666
chr10	31422786	31422999
chr10	31423293	31423754
chr10	31607350	31608063
chr10	31608201	31608415
chr10	31642699	31642915
chr10	31891636	31892298
chr10	31892739	31893235
chr10	32034448	32034677
chr10	32047363	32047499
chr10	32047702	32047925
chr10	32048193	32048999
chr10	32049441	32049632
chr10	32057943	32058300
chr10	32058761	32058811
chr10	32151252	32151503
chr10	32155252	32155581
chr10	32216693	32218700
chr10	32225929	32226201
chr10	32229186	32229546
chr10	32229661	32229802
chr10	32230105	32230253
chr10	32230588	32230788
chr10	32233492	32233630
chr10	32233757	32233818
chr10	32253458	32253543
chr10	32258003	32258188
chr10	32258413	32258448
chr10	32338265	32338464
chr10	32344379	32344716
chr10	32344851	32345641
chr10	32359574	32359721
chr10	32367113	32367245
chr10	32428560	32428755
chr10	32432597	32432782
chr10	32433645	32433671
chr10	32438115	32438629
chr10	32438844	32438968
chr10	32452750	32453054
chr10	32500002	32500067
chr10	32587229	32587368

chr10	32616347	32616486
chr10	32634945	32635019
chr10	32635325	32636665
chr10	32641784	32641986
chr10	32666432	32666631
chr10	32667496	32667648
chr10	32667776	32667950
chr10	32668340	32668723
chr10	32672560	32672710
chr10	32734792	32735973
chr10	33226191	33226219
chr10	33226896	33227177
chr10	33227664	33227966
chr10	33228507	33228811
chr10	33229009	33229040
chr10	33229798	33230153
chr10	33246177	33247971
chr10	33249065	33249344
chr10	33250636	33251331
chr10	33252056	33252379
chr10	33269251	33269390
chr10	33269738	33270127
chr10	33270195	33270504
chr10	33270666	33270679
chr10	33270874	33271063
chr10	33271308	33271680
chr10	33272133	33272585
chr10	33287122	33287664
chr10	33294131	33294687
chr10	33294828	33295339
chr10	33297674	33297726
chr10	33297906	33298022
chr10	33299185	33299370
chr10	33299800	33299808
chr10	33415161	33415697
chr10	33415863	33416317
chr10	33422665	33422900
chr10	33423307	33423732
chr10	33482964	33482988
chr10	33483172	33483304
chr10	33505409	33506348
chr10	33506855	33506893
chr10	33526816	33526893
chr10	33527048	33527322
chr10	33527767	33527877
chr10	33537539	33537729

chr10	33541818	33542041
chr10	33547873	33547956
chr10	33548278	33548367
chr10	33552605	33552922
chr10	33553051	33553264
chr10	33553370	33553629
chr10	33562678	33562755
chr10	33565266	33565826
chr10	33566020	33566147
chr10	33580078	33580623
chr10	33580883	33581282
chr10	33622636	33622718
chr10	33622967	33626103
chr10	33654780	33654968
chr10	33665981	33666199
chr10	33860892	33860982
chr10	34165870	34165987
chr10	34169238	34169305
chr10	34186152	34186300
chr10	34186619	34186733
chr10	34189667	34189671
chr10	34216263	34216444
chr10	34241927	34242088
chr10	34569428	34570113
chr10	34575257	34575548
chr10	34594003	34594069
chr10	34594474	34594546
chr10	34608408	34608562
chr10	34716095	34716129
chr10	34760533	34760732
chr10	34762372	34762724
chr10	34762869	34763098
chr10	34763291	34763407
chr10	34779368	34779614
chr10	34780997	34781090
chr10	34781301	34781384
chr10	34782997	34783391
chr10	34793457	34793633
chr10	34809059	34809369
chr10	34815602	34815776
chr10	34815911	34816326
chr10	34845321	34845574
chr10	34869875	34869918
chr10	34871332	34871543
chr10	34910271	34910500
chr10	34953556	34953857

chr10	34954041	34954078
chr10	34955218	34956191
chr10	34988021	34988181
chr10	34990997	34991732
chr10	34999364	34999443
chr10	34999672	34999743
chr10	35000542	35000684
chr10	35008687	35009086
chr10	35010158	35010524
chr10	35020873	35021061
chr10	35035115	35035237
chr10	35093069	35093328
chr10	35094117	35094251
chr10	35103157	35104922
chr10	35173710	35173855
chr10	35173951	35174299
chr10	35242765	35243010
chr10	35243442	35243861
chr10	35245567	35245679
chr10	35263041	35263214
chr10	35263329	35263338
chr10	35266714	35267097
chr10	35267328	35267454
chr10	35277563	35277975
chr10	35280781	35281151
chr10	35283426	35283996
chr10	35285833	35285899
chr10	35294767	35294867
chr10	35295161	35295336
chr10	35329809	35330796
chr10	35347308	35347511
chr10	35368677	35368684
chr10	35369012	35369094
chr10	35378886	35379553
chr10	35379734	35379860
chr10	35415337	35415597
chr10	35415822	35417161
chr10	35484353	35485335
chr10	35556952	35557128
chr10	35595737	35595855
chr10	35596072	35596407
chr10	35596554	35596616
chr10	35620880	35621477
chr10	35624579	35625724
chr10	35625910	35626497
chr10	35629481	35629718

chr10	35642956	35643057
chr10	35661447	35661723
chr10	35709793	35709917
chr10	35710068	35710150
chr10	35710386	35710915
chr10	35711199	35711542
chr10	35736675	35737128
chr10	35737468	35737740
chr10	35742801	35742887
chr10	35743103	35743176
chr10	35781277	35781782
chr10	35928793	35929046
chr10	35929338	35929537
chr10	35930337	35931109
chr10	35965769	35965887
chr10	36054254	36054422
chr10	36176844	36177043
chr10	36184969	36184993
chr10	36201681	36201855
chr10	36201962	36202086
chr10	36202564	36202987
chr10	38145139	38145534
chr10	38146099	38147096
chr10	38264998	38265555
chr10	38265888	38265962
chr10	38296935	38297312
chr10	38299225	38300476
chr10	38316973	38317112
chr10	38317230	38317268
chr10	38343392	38343482
chr10	38343604	38343720
chr10	38358622	38358858
chr10	38382970	38383159
chr10	38383269	38383894
chr10	38644865	38645063
chr10	38645406	38645606
chr10	38645745	38645908
chr10	38690660	38691363
chr10	38691480	38691717
chr10	38692192	38692542
chr10	38694248	38694338
chr10	38774328	38774566
chr10	38775705	38776006
chr10	38776304	38776591
chr10	38777926	38778275
chr10	38781931	38782177

chr10	38782344	38782675
chr10	38784797	38784996
chr10	38785573	38785726
chr10	38794039	38794358
chr10	38798271	38798470
chr10	38806249	38806562
chr10	38808722	38809040
chr10	38814434	38814829
chr10	38815782	38818784
chr10	38870254	38870312
chr10	38871983	38872348
chr10	38874379	38874550
chr10	38881497	38882149
chr10	39076582	39077058
chr10	39078851	39079050
chr10	39079216	39079703
chr10	39081501	39081719
chr10	39082183	39082448
chr10	39085770	39086220
chr10	39086961	39087241
chr10	39087815	39088014
chr10	39088289	39088544
chr10	39093878	39094361
chr10	39101130	39101384
chr10	39105567	39105766
chr10	39107105	39107548
chr10	39107852	39108203
chr10	39111107	39111473
chr10	39111720	39112182
chr10	39121304	39121627
chr10	39124501	39124700
chr10	39125422	39125678
chr10	39128488	39129046
chr10	39136586	39136814
chr10	39138024	39138349
chr10	39138710	39138799
chr10	39139335	39139552
chr10	39144015	39144355
chr10	39146374	39146549
chr10	39148265	39148470
chr10	39151037	39151250
chr10	42354941	42363065
chr10	42363228	42369726
chr10	42369990	42373714
chr10	42373865	42376087
chr10	42376300	42397358

chr10	42397889	42400892
chr10	42401799	42402485
chr10	42404250	42404680
chr10	42406585	42406853
chr10	42408037	42408495
chr10	42409087	42409435
chr10	42527153	42528514
chr10	42528577	42528852
chr10	42529110	42531256
chr10	42538376	42540983
chr10	42531332	42538235
chr10	42541332	42542101
chr10	42542628	42544036
chr10	42544443	42545397
chr10	42545677	42546559
chr10	42596639	42600703
chr10	42661356	42661621
chr10	42686529	42686701
chr10	42797755	42797954
chr10	42798126	42798136
chr10	42799760	42800058
chr10	42800188	42800238
chr10	42800355	42800589
chr10	42801423	42801622
chr10	42802803	42802998
chr10	42805935	42806134
chr10	42808044	42808354
chr10	42810582	42810781
chr10	42814989	42815578
chr10	42816287	42816586
chr10	42817236	42817510
chr10	42817638	42818340
chr10	43047553	43048204
chr10	43048541	43048698
chr10	43089724	43089803
chr10	43090001	43090232
chr10	43133159	43133566
chr10	43133676	43134444
chr10	43136104	43136790
chr10	43142375	43142543
chr10	43187089	43187729
chr10	43249114	43250036
chr10	43250238	43250497
chr10	43277318	43278105
chr10	43278231	43278980
chr10	43332393	43332686

chr10	43332887	43333060
chr10	43346764	43346862
chr10	43522074	43522273
chr10	43600260	43601048
chr10	43605935	43606152
chr10	43632534	43633800
chr10	43633907	43634730
chr10	43666898	43667070
chr10	43697868	43698067
chr10	43698294	43699271
chr10	43773430	43773639
chr10	43856522	43856799
chr10	43857007	43858722
chr10	43876102	43876247
chr10	43876548	43876964
chr10	43879003	43879225
chr10	43884102	43884306
chr10	43886468	43886598
chr10	43887316	43887343
chr10	43888821	43889020
chr10	43893454	43893767
chr10	43893986	43894211
chr10	43894704	43895266
chr10	43891700	43893189
chr10	43898537	43898884
chr10	43899209	43900048
chr10	43900810	43901035
chr10	43901271	43901285
chr10	43901429	43901695
chr10	43901760	43902243
chr10	43902469	43906180
chr10	43906494	43907385
chr10	43912865	43913140
chr10	43913794	43914609
chr10	43915309	43916900
chr10	43917118	43917856
chr10	43923247	43923265
chr10	43931105	43933092
chr10	43947284	43948201
chr10	43948916	43949153
chr10	43949548	43949833
chr10	43950113	43951189
chr10	43951296	43952063
chr10	43956438	43957051
chr10	43957609	43957692
chr10	44068788	44068968

chr10	44069196	44070019
chr10	44070233	44070314
chr10	44101871	44102532
chr10	44143755	44144833
chr10	45454736	45454992
chr10	45469934	45470477
chr10	45470608	45470956
chr10	45471213	45471460
chr10	45473461	45473673
chr10	45475518	45476313
chr10	45476567	45476726
chr10	45477262	45477500
chr10	45496169	45497073
chr10	45730956	45731096
chr10	45914633	45915288
chr10	46089590	46090462
chr10	46167479	46168108
chr10	46186632	46186831
chr10	46222239	46222569
chr10	46222705	46223531
chr10	46286132	46286180
chr10	46564762	46564923
chr10	46914048	46914354
chr10	46914420	46914681
chr10	46947480	46947599
chr10	46948711	46948728
chr10	46948850	46949136
chr10	46951402	46953238
chr10	46953580	46953621
chr10	46953929	46954009
chr10	46956597	46956812
chr10	46958150	46958155
chr10	46958479	46958598
chr10	46959617	46959684
chr10	46959906	46960341
chr10	46960426	46961233
chr10	46962729	46963314
chr10	46970226	46971100
chr10	46983045	46983468
chr10	46989259	46989359
chr10	46989476	46989503
chr10	46992387	46993827
chr10	46994350	46994613
chr10	47009722	47009957
chr10	47018369	47018576
chr10	47022576	47023159

chr10	47029190	47029210
chr10	47033161	47033260
chr10	47044948	47045332
chr10	47046863	47046935
chr10	47049099	47049298
chr10	47055011	47055411
chr10	47055847	47056049
chr10	47056226	47059626
chr10	47073455	47073705
chr10	47074451	47074579
chr10	47074955	47075057
chr10	47100495	47100792
chr10	47107826	47108192
chr10	47108670	47108869
chr10	47112949	47113119
chr10	47123487	47123894
chr10	47131184	47131418
chr10	47132849	47132872
chr10	47133196	47133274
chr10	47135276	47135475
chr10	47137820	47138019
chr10	47150331	47150530
chr10	47151043	47151906
chr10	47152123	47152756
chr10	47161970	47162803
chr10	47162936	47163254
chr10	47168658	47168980
chr10	47169458	47169753
chr10	47171069	47172326
chr10	47172404	47174537
chr10	47170031	47170222
chr10	47176066	47176208
chr10	47242758	47244000
chr10	47377359	47377647
chr10	47386221	47386247
chr10	47386460	47386566
chr10	47389922	47390144
chr10	47391157	47391627
chr10	47395794	47396367
chr10	47653783	47654137
chr10	47655542	47656505
chr10	47686843	47687094
chr10	47740236	47740435
chr10	47743594	47743861
chr10	47745517	47745627
chr10	47746382	47749938

chr10	47757626	47757869
chr10	47758382	47758647
chr10	47767820	47770023
chr10	47770332	47770669
chr10	48179980	48180179
chr10	48185456	48185607
chr10	48185880	48186631
chr10	48248181	48248529
chr10	48250911	48251251
chr10	48254870	48256893
chr10	48257046	48257425
chr10	48257583	48258294
chr10	48258821	48258845
chr10	48259136	48259962
chr10	48267533	48267800
chr10	48267871	48268018
chr10	48265995	48267466
chr10	48275514	48275609
chr10	48275828	48275882
chr10	48276026	48276461
chr10	48276637	48278272
chr10	48278627	48278963
chr10	48330645	48330918
chr10	48331445	48332244
chr10	48332609	48333171
chr10	48333436	48333803
chr10	48334261	48335056
chr10	48335331	48336706
chr10	48342454	48343141
chr10	48354309	48354972
chr10	48355133	48355944
chr10	48555605	48556038
chr10	48571944	48572222
chr10	48572670	48572869
chr10	48585956	48586338
chr10	48827386	48827877
chr10	48951802	48952296
chr10	48952553	48952942
chr10	49094408	49094454
chr10	49195564	49196093
chr10	49196223	49197649
chr10	49197979	49197989
chr10	49198141	49200402
chr10	49200512	49200807
chr10	49204297	49204588
chr10	49204793	49204840

chr10	49204945	49205467
chr10	49233414	49233685
chr10	49461960	49462020
chr10	49462325	49462516
chr10	49514187	49515353
chr10	49515572	49515606
chr10	49517910	49518202
chr10	49527987	49528149
chr10	49540179	49540878
chr10	49541748	49542116
chr10	49571799	49571979
chr10	49573209	49573408
chr10	49622624	49623158
chr10	49624682	49625138
chr10	49625365	49625425
chr10	49673937	49675223
chr10	49675814	49675820
chr10	49679842	49679869
chr10	49679980	49680253
chr10	49797720	49797749
chr10	49798080	49798165
chr10	49812680	49813321
chr10	49813581	49813797
chr10	49814860	49815077
chr10	49863916	49864358
chr10	49864642	49864841
chr10	50626737	50627136
chr10	50627270	50627527
chr10	50742961	50743430
chr10	50746071	50748062
chr10	50757954	50758993
chr10	50760074	50760716
chr10	50777406	50777429
chr10	50809322	50809800
chr10	50970041	50970459
chr10	51039409	51039751
chr10	51052995	51053217
chr10	51053528	51053624
chr10	51060194	51060274
chr10	51060523	51060574
chr10	51120487	51120981
chr10	51189762	51189808
chr10	51369827	51370153
chr10	51370379	51370817
chr10	51370924	51371916
chr10	51372046	51372047

chr10	51488384	51488564
chr10	51488693	51489356
chr10	51554544	51554756
chr10	51561710	51562012
chr10	51564927	51565829
chr10	51566819	51566996
chr10	51571986	51572122
chr10	51572347	51572844
chr10	51573063	51573427
chr10	51574555	51574593
chr10	51591431	51591517
chr10	51591639	51591908
chr10	51612210	51612299
chr10	51622852	51623906
chr10	51624098	51624232
chr10	51624488	51624848
chr10	51738300	51738325
chr10	51827024	51828506
chr10	52072883	52073054
chr10	52077617	52077990
chr10	52081780	52081948
chr10	52082105	52082207
chr10	52085119	52086011
chr10	52086611	52087180
chr10	52153746	52153923
chr10	52155002	52155216
chr10	52155337	52155411
chr10	52177399	52178466
chr10	52257422	52257488
chr10	52257710	52257793
chr10	52261282	52261586
chr10	52263666	52263902
chr10	52264064	52264200
chr10	52346239	52346468
chr10	52364903	52365154
chr10	52381596	52381631
chr10	52382027	52385712
chr10	52499215	52500449
chr10	52609470	52609844
chr10	52687573	52687674
chr10	52750743	52751500
chr10	53459116	53459877
chr10	53638955	53639090
chr10	53993362	53993704
chr10	54016717	54017101
chr10	54017443	54017536

chr10	54067973	54068045
chr10	54068230	54068538
chr10	54072843	54073205
chr10	54073507	54074254
chr10	54074386	54074775
chr10	54075041	54075109
chr10	54078005	54078326
chr10	54078623	54078794
chr10	54079106	54079283
chr10	54094376	54094684
chr10	54147456	54147923
chr10	54195150	54195413
chr10	54203092	54203854
chr10	54211534	54211680
chr10	54211786	54211958
chr10	54212310	54212422
chr10	54212530	54212727
chr10	54239562	54239669
chr10	54246431	54247021
chr10	54299257	54299261
chr10	54624051	54624222
chr10	54624586	54624692
chr10	54642979	54643387
chr10	54643525	54644159
chr10	54644308	54644364
chr10	54790144	54790204
chr10	54790466	54790587
chr10	55074957	55074977
chr10	55361062	55361099
chr10	55379419	55379822
chr10	55664338	55664361
chr10	56868394	56868525
chr10	56903930	56904155
chr10	56905814	56905914
chr10	57023639	57023816
chr10	58120642	58121013
chr10	58121159	58121767
chr10	59383786	59384247
chr10	59416947	59417024
chr10	59452030	59452417
chr10	59456452	59456874
chr10	59457503	59457896
chr10	59463531	59463602
chr10	59463761	59463930
chr10	59465286	59465661
chr10	59551372	59551409

chr10	59551576	59551631
chr10	59559417	59559782
chr10	59752824	59752933
chr10	59783338	59783601
chr10	59847665	59848048
chr10	59938562	59938608
chr10	59938846	59938943
chr10	59940320	59940837
chr10	59948980	59949155
chr10	59997414	59997695
chr10	59999881	59999887
chr10	60024587	60024838
chr10	60026053	60026523
chr10	60026617	60027762
chr10	60028188	60028746
chr10	60028936	60029871
chr10	60042563	60042774
chr10	60094765	60095841
chr10	60110410	60110463
chr10	60110680	60110716
chr10	60120136	60120354
chr10	60144624	60145650
chr10	60145895	60145927
chr10	60174353	60174527
chr10	60174654	60174781
chr10	60195027	60195457
chr10	60195853	60196276
chr10	60198088	60198326
chr10	60271899	60273916
chr10	60291097	60291182
chr10	60349893	60349965
chr10	60400920	60401054
chr10	60401273	60401288
chr10	60422259	60422438
chr10	60461221	60461619
chr10	60515496	60515848
chr10	60535876	60536143
chr10	60557155	60557449
chr10	60580561	60580696
chr10	60580951	60581075
chr10	60616162	60616317
chr10	60616533	60616939
chr10	60839421	60839508
chr10	60841961	60842059
chr10	60933179	60933578
chr10	60933641	60933960

chr10	60935493	60936971
chr10	60941549	60941811
chr10	61050268	61050467
chr10	61097030	61097057
chr10	61097298	61097340
chr10	61387067	61387298
chr10	61469080	61470175
chr10	61569356	61569517
chr10	61625089	61625330
chr10	61637142	61637539
chr10	61654723	61655063
chr10	61660116	61660324
chr10	61662258	61662406
chr10	61664852	61664864
chr10	61665013	61667007
chr10	61667713	61668039
chr10	61668160	61668573
chr10	61725084	61725166
chr10	61725526	61725624
chr10	61745538	61745573
chr10	61745696	61745776
chr10	61882936	61883043
chr10	61883152	61883846
chr10	61907981	61908296
chr10	61940031	61940235
chr10	61963844	61963902
chr10	61997800	61997942
chr10	62073140	62073242
chr10	62144409	62144439
chr10	62144811	62145021
chr10	62173590	62173856
chr10	62186143	62186469
chr10	62188026	62188129
chr10	62194467	62194541
chr10	62206619	62206719
chr10	62225816	62225931
chr10	62233242	62233369
chr10	62233645	62233761
chr10	62239095	62239194
chr10	62239345	62239664
chr10	62240813	62240991
chr10	62241243	62241460
chr10	62244923	62245130
chr10	62247903	62247951
chr10	62248145	62248233
chr10	62302526	62302632

chr10	62366328	62366407
chr10	62366610	62366788
chr10	62424899	62425005
chr10	62425934	62426149
chr10	62492060	62492570
chr10	62492682	62493463
chr10	62493698	62493934
chr10	62494141	62494226
chr10	62520503	62520932
chr10	62528821	62528913
chr10	62537827	62538026
chr10	62538280	62538910
chr10	62576574	62576653
chr10	62703148	62704769
chr10	62723168	62723231
chr10	62723367	62723516
chr10	62723921	62724065
chr10	62724518	62724856
chr10	62761010	62761485
chr10	62950278	62950419
chr10	63247401	63247483
chr10	63388002	63388305
chr10	63388515	63389082
chr10	63391968	63392065
chr10	63392388	63392448
chr10	63403107	63403123
chr10	63403467	63403468
chr10	63422443	63422505
chr10	63422867	63423066
chr10	63493380	63493394
chr10	63546503	63546738
chr10	63595384	63595661
chr10	63597021	63597271
chr10	63656574	63656851
chr10	63656960	63657193
chr10	63657395	63657777
chr10	63658815	63659167
chr10	63659719	63660353
chr10	63660525	63660590
chr10	63661276	63662478
chr10	63662648	63663722
chr10	63664471	63664875
chr10	63697335	63697462
chr10	63697655	63697670
chr10	63699329	63699467
chr10	63699586	63699740

chr10	63702768	63702997
chr10	63710137	63710361
chr10	63735261	63735403
chr10	63751640	63752095
chr10	63752427	63752534
chr10	63752722	63752831
chr10	63779032	63779532
chr10	63785105	63785571
chr10	63786265	63786620
chr10	63787497	63788630
chr10	63795985	63795986
chr10	63796622	63796677
chr10	63796775	63797384
chr10	63798473	63798899
chr10	63802854	63803095
chr10	63803162	63803776
chr10	63806421	63806434
chr10	63806701	63806724
chr10	63807972	63808138
chr10	63808765	63808836
chr10	63809123	63809339
chr10	63810564	63810827
chr10	63811081	63811226
chr10	63836462	63836576
chr10	63842556	63842773
chr10	63843199	63843286
chr10	63843714	63843776
chr10	63843957	63844068
chr10	63993612	63993839
chr10	63994024	63994242
chr10	64003017	64003021
chr10	64003359	64003465
chr10	64004730	64005169
chr10	64010345	64010763
chr10	64010910	64011027
chr10	64024441	64024648
chr10	64028312	64028882
chr10	64133867	64134231
chr10	64563985	64565963
chr10	64578434	64578780
chr10	64852996	64853370
chr10	64860862	64860871
chr10	64861996	64862030
chr10	64865561	64866124
chr10	64866322	64866542
chr10	64867318	64867472

chr10	64870666	64870733
chr10	64870911	64871036
chr10	64873746	64874182
chr10	64875509	64876009
chr10	64876520	64876536
chr10	64876656	64876807
chr10	64892711	64892783
chr10	64893027	64893878
chr10	64899608	64899800
chr10	64900520	64900690
chr10	64917848	64917978
chr10	64922593	64922837
chr10	64924124	64924497
chr10	64976869	64977197
chr10	64996150	64996756
chr10	65008867	65009138
chr10	65018099	65018211
chr10	65024897	65025163
chr10	65027178	65027832
chr10	65028520	65029322
chr10	65030313	65030646
chr10	65043690	65044191
chr10	65047093	65047102
chr10	65051295	65051426
chr10	65060717	65061073
chr10	65088823	65088985
chr10	65134809	65135008
chr10	65135693	65135924
chr10	65136586	65137512
chr10	65141041	65141326
chr10	65169454	65169515
chr10	65190080	65190215
chr10	65190364	65190753
chr10	65192175	65192231
chr10	65216238	65216585
chr10	65224240	65224519
chr10	65224705	65226622
chr10	65251333	65251342
chr10	65251502	65251747
chr10	65263821	65263911
chr10	65264300	65264446
chr10	65264522	65264798
chr10	65274784	65275081
chr10	65277479	65277660
chr10	65280383	65281952
chr10	65303115	65303323

chr10	65312766	65313216
chr10	65313449	65314172
chr10	65314421	65314740
chr10	65324654	65324929
chr10	65346030	65346102
chr10	65348392	65348942
chr10	65370818	65371089
chr10	65388360	65390922
chr10	65393446	65393450
chr10	65393609	65393838
chr10	65432884	65432954
chr10	65433424	65433495
chr10	65442331	65442520
chr10	65442670	65442938
chr10	65459842	65459943
chr10	65460118	65460452
chr10	65466770	65467037
chr10	65467348	65467446
chr10	65471202	65471420
chr10	65471938	65471940
chr10	65472387	65472585
chr10	65479847	65479914
chr10	65801301	65801503
chr10	67330064	67330481
chr10	67331024	67331114
chr10	67331220	67331375
chr10	68264992	68265174
chr10	68386909	68386954
chr10	69523213	69523691
chr10	69523803	69524246
chr10	69524353	69524611
chr10	69524678	69525183
chr10	69550771	69550962
chr10	69551071	69551188
chr10	69577992	69578752
chr10	69579480	69579646
chr10	69597699	69597884
chr10	69603573	69603719
chr10	69608998	69610121
chr10	69615212	69615713
chr10	69643752	69645226
chr10	69645535	69646220
chr10	69725794	69726221
chr10	69812425	69812524
chr10	69834235	69835061
chr10	69835244	69835348

chr10	69847125	69847242
chr10	69847352	69847666
chr10	69853754	69854791
chr10	69854896	69855347
chr10	69856870	69857080
chr10	69857231	69857338
chr10	69865612	69865864
chr10	69895911	69896486
chr10	69913616	69913835
chr10	69968440	69968660
chr10	69972875	69972963
chr10	69984580	69984659
chr10	69990717	69991236
chr10	70057068	70057210
chr10	70085750	70086059
chr10	70088004	70088482
chr10	70091058	70092610
chr10	70090597	70090986
chr10	70092731	70092876
chr10	70108185	70108360
chr10	70162985	70162989
chr10	70163225	70163227
chr10	70165914	70167656
chr10	70167911	70168120
chr10	70194767	70194770
chr10	70209389	70209745
chr10	70231238	70231997
chr10	70285000	70285318
chr10	70286512	70287174
chr10	70287359	70288024
chr10	70308741	70309022
chr10	70309378	70309771
chr10	70310476	70310508
chr10	70310875	70310925
chr10	70320119	70320485
chr10	70321201	70321402
chr10	70352737	70352793
chr10	70354173	70354358
chr10	70354486	70354678
chr10	70359668	70360098
chr10	70360256	70360512
chr10	70377234	70377506
chr10	70407869	70408062
chr10	70480284	70481624
chr10	70483529	70483786
chr10	70487590	70487784

chr10	70492191	70492379
chr10	70494818	70495006
chr10	70565101	70565231
chr10	70565361	70565586
chr10	70576755	70576870
chr10	70586688	70587523
chr10	70599343	70599567
chr10	70648460	70648617
chr10	70657636	70657886
chr10	70660189	70660863
chr10	70661058	70661779
chr10	70714793	70714992
chr10	70715059	70716316
chr10	70716435	70717205
chr10	70725850	70726063
chr10	70746868	70747168
chr10	70748178	70748233
chr10	70748582	70749425
chr10	70752780	70753201
chr10	70798242	70798647
chr10	70804107	70804300
chr10	70805009	70805376
chr10	70805530	70806138
chr10	70808339	70808483
chr10	70810464	70810726
chr10	70818995	70818999
chr10	70820942	70822205
chr10	70825602	70826099
chr10	70853281	70853508
chr10	70883005	70883665
chr10	70883867	70884941
chr10	70886116	70886347
chr10	70920769	70921103
chr10	70939192	70940601
chr10	70976871	70977759
chr10	70980958	70981012
chr10	70981650	70982664
chr10	70982784	70982835
chr10	70984984	70985411
chr10	70989263	70989486
chr10	70991404	70991552
chr10	70992017	70992179
chr10	70992723	70992830
chr10	70994134	70994185
chr10	70994271	70994333
chr10	70998991	70999243

chr10	70999547	70999828
chr10	71001045	71001583
chr10	71003745	71003986
chr10	71016113	71016248
chr10	71028403	71028969
chr10	71029868	71030174
chr10	71075954	71076153
chr10	71076372	71076563
chr10	71077131	71079903
chr10	71081250	71081443
chr10	71081982	71082585
chr10	71082668	71083092
chr10	71083571	71083710
chr10	71083963	71084162
chr10	71084264	71084270
chr10	71084572	71084722
chr10	71084894	71085212
chr10	71085444	71086323
chr10	71087194	71087354
chr10	71090791	71091109
chr10	71092004	71093711
chr10	71093797	71094757
chr10	71094924	71095581
chr10	71096934	71099249
chr10	71100251	71101025
chr10	71105244	71105678
chr10	71108492	71109565
chr10	71109807	71110224
chr10	71112331	71112965
chr10	71117194	71117445
chr10	71118229	71118706
chr10	71118874	71119183
chr10	71119621	71119954
chr10	71120075	71120563
chr10	71120774	71120956
chr10	71121378	71121463
chr10	71122174	71122314
chr10	71122433	71123449
chr10	71125349	71125468
chr10	71134993	71135200
chr10	71140767	71141014
chr10	71142737	71142997
chr10	71144354	71144692
chr10	71145040	71146040
chr10	71147257	71147634
chr10	71149329	71149530

chr10	71149665	71149809
chr10	71151108	71151275
chr10	71152451	71152863
chr10	71155775	71155830
chr10	71166220	71166724
chr10	71167221	71167420
chr10	71167879	71167964
chr10	71168170	71168373
chr10	71168777	71169244
chr10	71185796	71186175
chr10	71186630	71187215
chr10	71198106	71198493
chr10	71198739	71199075
chr10	71199268	71200230
chr10	71200366	71200572
chr10	71203813	71203935
chr10	71205832	71206819
chr10	71208660	71208701
chr10	71208920	71209126
chr10	71209302	71209609
chr10	71209929	71210202
chr10	71210531	71210782
chr10	71211229	71211431
chr10	71214952	71215029
chr10	71228348	71228614
chr10	71267023	71267569
chr10	71267835	71267992
chr10	71389695	71390659
chr10	71399463	71400233
chr10	71400536	71401054
chr10	71463415	71463988
chr10	71464128	71464203
chr10	71488405	71488694
chr10	71811927	71812346
chr10	71812590	71813977
chr10	71877690	71877889
chr10	71890803	71890879
chr10	71891222	71891514
chr10	71891667	71893495
chr10	71900771	71900916
chr10	71902693	71902758
chr10	71903276	71903535
chr10	71904448	71904863
chr10	71904995	71906548
chr10	71906925	71907044
chr10	71907997	71908813

chr10	71909416	71909525
chr10	71922066	71922423
chr10	71929515	71930703
chr10	71930925	71931132
chr10	71944825	71944983
chr10	71951571	71952192
chr10	71952294	71952992
chr10	71953316	71953563
chr10	71954211	71954753
chr10	71956894	71957119
chr10	71981344	71981562
chr10	71982130	71982660
chr10	71991820	71993824
chr10	71997476	71997715
chr10	72015360	72015648
chr10	72015767	72016077
chr10	72024829	72025276
chr10	72027015	72027258
chr10	72027647	72027687
chr10	72032263	72032359
chr10	72032854	72033032
chr10	72049760	72049993
chr10	72050317	72050925
chr10	72050989	72051036
chr10	72055569	72055594
chr10	72055820	72056405
chr10	72056559	72056824
chr10	72062017	72062455
chr10	72091630	72092282
chr10	72101596	72102184
chr10	72108674	72108872
chr10	72112615	72113012
chr10	72123235	72124302
chr10	72124460	72124695
chr10	72124841	72125107
chr10	72126824	72126947
chr10	72134921	72135173
chr10	72136887	72137142
chr10	72137447	72138351
chr10	72138796	72138886
chr10	72141285	72142861
chr10	72143228	72143301
chr10	72146800	72147764
chr10	72159978	72160177
chr10	72163347	72163644
chr10	72163828	72164957

chr10	72165639	72166017
chr10	72221410	72221937
chr10	72237867	72238217
chr10	72238331	72238813
chr10	72394643	72395035
chr10	72575004	72575076
chr10	72575326	72575343
chr10	72575600	72576823
chr10	72576895	72577033
chr10	72590050	72590130
chr10	72627029	72627207
chr10	72647875	72648595
chr10	72661993	72662130
chr10	72972302	72973315
chr10	72973569	72973768
chr10	72974035	72974311
chr10	72977100	72977306
chr10	72977705	72977912
chr10	72998404	72998603
chr10	73004719	73004918
chr10	73005343	73005637
chr10	73006079	73006681
chr10	73006793	73007715
chr10	73016126	73016483
chr10	73016774	73016973
chr10	73019866	73020065
chr10	73020251	73020573
chr10	73025628	73025923
chr10	73026525	73026759
chr10	73028642	73028841
chr10	73029519	73030056
chr10	73078932	73079734
chr10	73136711	73136965
chr10	73487256	73487652
chr10	73488121	73489127
chr10	73489465	73489576
chr10	73499483	73499700
chr10	73500280	73500536
chr10	73500859	73501059
chr10	73518734	73519524
chr10	73526278	73526604
chr10	73526990	73527383
chr10	73527524	73528530
chr10	73528941	73528969
chr10	73533059	73533290
chr10	73533475	73533774

chr10	73573428	73573654
chr10	73598292	73598656
chr10	73598919	73599007
chr10	73606344	73606572
chr10	73606680	73606895
chr10	73610428	73611346
chr10	73611533	73611951
chr10	73619450	73619511
chr10	73619921	73620268
chr10	73620454	73620754
chr10	73622032	73622240
chr10	73622307	73622317
chr10	73626995	73627276
chr10	73627756	73627913
chr10	73628140	73628541
chr10	73628985	73629037
chr10	73630317	73630526
chr10	73630907	73631528
chr10	73631636	73632023
chr10	73632229	73632793
chr10	73633247	73633366
chr10	73641431	73641468
chr10	73648214	73649706
chr10	73650263	73650355
chr10	73656989	73657245
chr10	73679968	73680097
chr10	73685221	73685279
chr10	73716129	73716779
chr10	73723583	73726460
chr10	73726838	73727037
chr10	73727327	73727543
chr10	73727628	73727906
chr10	73728249	73728849
chr10	73730280	73730479
chr10	73733218	73733562
chr10	73734055	73735123
chr10	73735239	73735347
chr10	73735519	73736319
chr10	73737157	73737199
chr10	73737690	73737703
chr10	73737837	73738046
chr10	73748699	73749005
chr10	73757842	73757933
chr10	73758215	73759643
chr10	73760597	73760796
chr10	73761213	73761490

chr10	73763202	73763536
chr10	73764918	73765066
chr10	73767759	73770312
chr10	73770604	73770881
chr10	73770964	73771101
chr10	73786840	73786931
chr10	73787145	73787360
chr10	73787789	73788457
chr10	73795983	73796041
chr10	73801800	73801920
chr10	73801984	73802076
chr10	73804891	73805113
chr10	73808209	73808251
chr10	73818520	73818530
chr10	73829092	73829187
chr10	73829288	73829475
chr10	73840710	73841001
chr10	73846710	73846910
chr10	73866416	73866740
chr10	73866902	73866999
chr10	73867122	73867649
chr10	73867936	73868177
chr10	73898360	73898518
chr10	73922750	73922941
chr10	73923054	73923235
chr10	73930106	73930305
chr10	73930839	73931310
chr10	73934861	73935036
chr10	73951951	73952043
chr10	73958138	73958513
chr10	73959092	73959291
chr10	73960176	73960388
chr10	73971160	73971516
chr10	73975240	73976359
chr10	73976481	73976705
chr10	73979213	73979511
chr10	73986169	73986459
chr10	73992513	73992912
chr10	73998609	73999069
chr10	73999802	74000084
chr10	74000915	74001555
chr10	74002351	74002940
chr10	74003285	74005272
chr10	74007341	74009004
chr10	74010413	74010587
chr10	74006433	74006769

chr10	74010697	74011110
chr10	74012177	74012284
chr10	74013783	74013982
chr10	74014339	74015356
chr10	74015930	74016114
chr10	74016306	74016339
chr10	74016895	74017053
chr10	74017178	74017547
chr10	74018795	74019205
chr10	74019618	74021856
chr10	74021983	74022192
chr10	74025415	74025540
chr10	74022573	74023850
chr10	74029597	74029893
chr10	74030103	74030985
chr10	74031717	74033493
chr10	74033751	74036760
chr10	74037052	74037258
chr10	74037485	74037815
chr10	74038829	74039028
chr10	74041281	74041539
chr10	74044570	74044769
chr10	74045040	74045117
chr10	74046894	74047385
chr10	74048489	74048586
chr10	74051517	74052775
chr10	74052953	74053584
chr10	74053715	74054131
chr10	74054527	74054648
chr10	74055049	74055210
chr10	74056725	74060097
chr10	74055985	74056156
chr10	74060876	74061076
chr10	74061984	74062388
chr10	74063753	74064222
chr10	74067527	74067776
chr10	74068044	74068049
chr10	74068192	74068826
chr10	74068905	74069261
chr10	74069566	74070497
chr10	74071488	74071530
chr10	74074165	74074450
chr10	74074749	74076097
chr10	74076160	74077097
chr10	74077492	74077805
chr10	74078022	74083805

chr10	74083967	74084202
chr10	74084513	74084553
chr10	74084673	74085447
chr10	74085564	74085874
chr10	74087192	74087794
chr10	74088266	74089459
chr10	74089591	74089790
chr10	74089893	74091875
chr10	74093828	74094799
chr10	74091981	74093749
chr10	74094918	74096415
chr10	74097437	74097612
chr10	74098392	74098553
chr10	74098895	74098900
chr10	74099691	74099900
chr10	74100125	74100789
chr10	74110283	74110294
chr10	74113386	74114769
chr10	74115015	74115218
chr10	74115306	74115589
chr10	74122188	74122231
chr10	74123411	74124530
chr10	74125323	74125371
chr10	74144907	74144970
chr10	74213888	74213972
chr10	74299758	74299942
chr10	74312703	74313055
chr10	74347276	74347352
chr10	74372273	74372472
chr10	74385242	74385908
chr10	74386076	74386260
chr10	74414933	74415132
chr10	74425680	74426184
chr10	74435837	74436356
chr10	74436500	74436717
chr10	74446168	74446465
chr10	74447866	74448295
chr10	74451534	74451693
chr10	74451859	74452872
chr10	74454291	74454575
chr10	74477031	74477406
chr10	74479858	74480184
chr10	74500409	74500649
chr10	74562590	74562854
chr10	74572594	74572987
chr10	74591795	74592064

chr10	74605748	74606124
chr10	74635004	74635305
chr10	74647045	74647233
chr10	74649015	74649649
chr10	74801000	74801700
chr10	74804162	74804581
chr10	74820882	74820897
chr10	74821152	74821171
chr10	74855646	74857111
chr10	74870180	74870409
chr10	74870592	74871073
chr10	74877699	74877710
chr10	74877878	74878251
chr10	74915381	74915614
chr10	74918882	74919152
chr10	74927375	74928521
chr10	74969526	74969798
chr10	75006334	75007002
chr10	75007178	75007359
chr10	75007568	75007817
chr10	75011951	75013284
chr10	75015046	75015146
chr10	75021867	75022225
chr10	75038404	75038769
chr10	75118023	75118057
chr10	75124148	75124239
chr10	75136484	75136909
chr10	75139752	75139935
chr10	75166568	75166672
chr10	75171719	75171927
chr10	75172771	75173854
chr10	75174362	75174729
chr10	75179207	75179223
chr10	75239942	75240312
chr10	75253610	75253941
chr10	75254839	75256109
chr10	75258106	75258261
chr10	75319437	75319641
chr10	75350962	75351330
chr10	75361130	75361350
chr10	75375858	75376057
chr10	75384657	75386302
chr10	75396334	75396470
chr10	75396824	75396850
chr10	75401541	75401780
chr10	75403597	75403686

chr10	75403922	75404009
chr10	75421366	75421426
chr10	75488990	75489363
chr10	75489485	75490738
chr10	75498497	75498627
chr10	75500044	75500174
chr10	75500929	75502087
chr10	75503632	75504723
chr10	75516407	75516760
chr10	75522901	75523024
chr10	75531633	75533598
chr10	75534139	75534198
chr10	75534316	75534447
chr10	75539690	75539747
chr10	75540888	75541150
chr10	75541364	75542206
chr10	75544788	75546399
chr10	75546755	75547140
chr10	75569846	75570081
chr10	75570443	75572201
chr10	75575793	75576356
chr10	75579010	75579292
chr10	75579498	75579739
chr10	75585814	75586092
chr10	75586698	75587180
chr10	75587556	75587776
chr10	75604063	75604427
chr10	75633541	75634336
chr10	75634547	75634962
chr10	75635252	75635372
chr10	75635694	75635756
chr10	75636259	75636503
chr10	75637140	75637397
chr10	75638114	75638420
chr10	75638833	75639064
chr10	75639356	75639567
chr10	75642260	75642567
chr10	75643292	75643451
chr10	75643587	75643647
chr10	75644084	75644495
chr10	75645894	75650062
chr10	75651140	75651374
chr10	75652327	75652842
chr10	75652927	75653078
chr10	75653533	75653547
chr10	75654013	75654296

chr10	75654409	75654739
chr10	75655855	75656471
chr10	75658375	75658635
chr10	75658975	75659061
chr10	75659321	75659575
chr10	75663476	75663675
chr10	75665046	75665400
chr10	75665840	75666031
chr10	75666104	75667761
chr10	75667897	75668015
chr10	75668353	75668865
chr10	75669108	75671726
chr10	75671969	75672765
chr10	75674809	75675155
chr10	75677156	75677656
chr10	75678477	75678725
chr10	75678997	75679230
chr10	75679486	75680352
chr10	75680916	75681348
chr10	75687043	75687059
chr10	75688809	75688852
chr10	75691380	75692683
chr10	75693545	75693838
chr10	75695185	75695281
chr10	75698105	75698354
chr10	75698491	75698828
chr10	75700167	75700555
chr10	75700720	75701126
chr10	75703741	75703916
chr10	75704941	75705140
chr10	75709564	75709735
chr10	75719893	75720207
chr10	75725047	75726473
chr10	75727064	75727291
chr10	75729236	75729383
chr10	75742944	75743083
chr10	75752170	75752222
chr10	75756154	75756302
chr10	75757100	75757621
chr10	75757793	75759510
chr10	75760129	75760440
chr10	75759601	75759931
chr10	75763417	75763718
chr10	75764399	75764598
chr10	75769788	75769979
chr10	75781340	75781442

chr10	75806009	75806253
chr10	75807144	75807343
chr10	75807534	75808175
chr10	75811990	75812093
chr10	75816272	75816559
chr10	75821942	75822025
chr10	75840993	75841004
chr10	75841287	75841333
chr10	75859476	75859924
chr10	75877292	75877345
chr10	75878294	75878662
chr10	75909912	75911845
chr10	75912228	75912644
chr10	75912916	75913315
chr10	75935991	75936088
chr10	75936216	75937215
chr10	75940320	75940554
chr10	75946236	75946477
chr10	75949813	75950621
chr10	75973578	75973666
chr10	75983525	75984358
chr10	75987390	75987840
chr10	75991116	75991540
chr10	76001381	76001566
chr10	76025096	76025366
chr10	76026010	76026196
chr10	76051865	76052070
chr10	76052667	76053021
chr10	76053483	76054188
chr10	76229681	76229774
chr10	76233781	76233812
chr10	76234024	76234202
chr10	76262905	76263314
chr10	76301752	76302190
chr10	76353552	76353574
chr10	76466977	76467103
chr10	76551795	76551837
chr10	76559536	76559735
chr10	76584632	76585849
chr10	76586354	76586703
chr10	76586815	76587212
chr10	76587347	76587875
chr10	76638244	76638279
chr10	76657879	76658203
chr10	76677135	76677375
chr10	76682544	76682678

chr10	76683985	76684419
chr10	76817620	76818292
chr10	76824216	76824350
chr10	76827672	76827862
chr10	76862078	76862176
chr10	76862469	76862668
chr10	76863335	76863780
chr10	76864600	76865132
chr10	76866934	76867145
chr10	76868073	76868562
chr10	76869510	76869540
chr10	76869649	76869805
chr10	76870476	76870483
chr10	76870834	76872227
chr10	76876714	76877598
chr10	76886275	76886344
chr10	76897804	76897852
chr10	76901743	76902172
chr10	76903851	76904365
chr10	76906563	76906893
chr10	76931115	76931191
chr10	76943593	76943619
chr10	76946064	76946338
chr10	76946717	76946977
chr10	76947485	76947831
chr10	76948122	76948414
chr10	76948964	76949024
chr10	76951617	76952431
chr10	76959168	76959305
chr10	76961506	76961718
chr10	76962734	76963152
chr10	76963812	76963887
chr10	76963973	76965244
chr10	76965612	76965793
chr10	76967407	76968642
chr10	76969236	76971514
chr10	76974389	76975628
chr10	76979275	76979520
chr10	76983444	76983639
chr10	76983796	76984022
chr10	76985268	76985737
chr10	76987941	76988268
chr10	76988743	76988942
chr10	76990279	76990762
chr10	76992722	76993045
chr10	76993448	76994138

chr10	76994258	76995791
chr10	77009229	77009302
chr10	77010836	77011418
chr10	77023068	77023282
chr10	77054779	77055534
chr10	77055614	77055878
chr10	77056513	77056581
chr10	77110283	77110611
chr10	77117035	77117065
chr10	77118452	77118814
chr10	77154826	77157402
chr10	77157566	77157611
chr10	77158288	77159813
chr10	77160065	77160666
chr10	77160795	77161614
chr10	77161774	77162078
chr10	77162250	77165540
chr10	77167545	77168321
chr10	77168533	77169958
chr10	77171864	77172042
chr10	77179837	77179966
chr10	77182483	77182641
chr10	77184772	77184790
chr10	77184933	77185047
chr10	77185160	77185445
chr10	77186903	77187800
chr10	77188095	77189073
chr10	77189771	77190041
chr10	77190202	77191749
chr10	77226659	77226732
chr10	77228832	77228952
chr10	77229215	77229547
chr10	77230132	77230286
chr10	78044700	78044796
chr10	78045122	78045196
chr10	78089701	78089735
chr10	78112012	78112491
chr10	78343316	78343725
chr10	78343838	78343970
chr10	78344349	78344552
chr10	78908806	78909058
chr10	78920344	78920382
chr10	78920502	78920944
chr10	78922618	78922870
chr10	78923654	78924201
chr10	78924329	78924510

chr10	78925359	78925641
chr10	79005774	79005864
chr10	79014054	79014284
chr10	79015078	79015407
chr10	79050474	79050900
chr10	79054493	79054526
chr10	79115577	79115661
chr10	79136738	79136794
chr10	79150499	79150712
chr10	79151582	79151744
chr10	79151851	79152638
chr10	79152938	79153465
chr10	79165913	79166127
chr10	79220725	79220950
chr10	79221028	79221282
chr10	79221566	79221806
chr10	79263984	79264310
chr10	79264494	79264791
chr10	79265350	79265549
chr10	79266055	79266158
chr10	79273068	79273232
chr10	79273418	79273784
chr10	79288103	79288258
chr10	79291002	79291315
chr10	79291470	79291775
chr10	79292051	79292311
chr10	79293798	79293997
chr10	79342795	79343082
chr10	79346754	79346795
chr10	79347305	79347575
chr10	79389168	79389669
chr10	79396758	79397623
chr10	79398244	79398652
chr10	79401455	79401654
chr10	79419083	79419122
chr10	79423286	79423417
chr10	79471046	79471307
chr10	79478462	79478522
chr10	79479313	79479373
chr10	79479512	79479772
chr10	79482457	79482732
chr10	79482967	79483211
chr10	79497321	79497897
chr10	79503971	79504438
chr10	79516161	79516317
chr10	79516513	79517017

chr10	79557808	79558024
chr10	79563711	79563910
chr10	79568146	79568939
chr10	79578861	79579339
chr10	79579538	79580185
chr10	79580294	79580493
chr10	79585196	79585551
chr10	79586213	79586277
chr10	79587354	79587389
chr10	79588132	79588191
chr10	79604764	79604974
chr10	79605144	79605292
chr10	79605897	79606263
chr10	79606381	79606956
chr10	79625323	79625557
chr10	79626014	79626242
chr10	79628013	79628140
chr10	79629500	79629563
chr10	79632075	79632209
chr10	79632912	79633961
chr10	79634087	79634206
chr10	79634383	79634734
chr10	79634844	79634872
chr10	79635088	79635303
chr10	79635914	79635921
chr10	79637379	79637474
chr10	79638061	79638404
chr10	79647506	79647705
chr10	79648732	79649400
chr10	79649594	79650038
chr10	79654870	79655015
chr10	79655402	79656539
chr10	79657682	79659070
chr10	79659899	79660298
chr10	79660924	79661124
chr10	79659192	79659692
chr10	79664806	79665150
chr10	79665470	79665669
chr10	79665917	79666186
chr10	79666421	79666688
chr10	79666921	79667294
chr10	79667593	79668033
chr10	79668168	79668379
chr10	79669751	79670080
chr10	79671290	79671468
chr10	79671644	79671954

chr10	79683215	79683458
chr10	79685105	79685430
chr10	79685587	79687005
chr10	79687139	79687335
chr10	79700462	79700961
chr10	79702748	79703247
chr10	79703420	79703615
chr10	79704154	79704335
chr10	79704909	79705180
chr10	79708583	79709614
chr10	79725173	79725516
chr10	79730985	79731194
chr10	79788414	79789273
chr10	79789418	79789939
chr10	79793240	79793449
chr10	79793657	79794281
chr10	79794615	79795046
chr10	79797874	79798263
chr10	79798491	79798801
chr10	79804796	79804981
chr10	79805687	79805785
chr10	79806193	79806484
chr10	79806826	79807188
chr10	79848445	79848663
chr10	79908636	79908925
chr10	80016194	80016329
chr10	80062573	80063324
chr10	80273598	80273617
chr10	80516903	80517412
chr10	80533994	80534137
chr10	80534257	80534374
chr10	80685473	80685485
chr10	80697804	80698013
chr10	80706652	80706851
chr10	80708150	80708208
chr10	80707927	80708060
chr10	80708949	80709281
chr10	80709862	80710035
chr10	80711145	80711553
chr10	80712267	80712677
chr10	80716531	80716858
chr10	80724996	80725195
chr10	80730664	80730792
chr10	80732530	80735485
chr10	80735893	80736228
chr10	80736906	80737532

chr10	80737852	80738278
chr10	80744328	80744331
chr10	80744404	80744620
chr10	80757800	80758214
chr10	80761758	80761808
chr10	80762065	80762114
chr10	80777192	80777356
chr10	80778052	80778419
chr10	80778651	80779071
chr10	80780687	80780691
chr10	80809103	80809337
chr10	80809745	80809757
chr10	80811375	80811640
chr10	80814950	80814986
chr10	80826898	80827560
chr10	80828042	80831323
chr10	80831830	80831887
chr10	80843354	80843810
chr10	80908120	80908352
chr10	80908825	80909609
chr10	80909760	80912820
chr10	80914722	80914994
chr10	80916528	80916779
chr10	80916897	80918315
chr10	80918419	80918744
chr10	80922941	80923297
chr10	80943757	80943956
chr10	80961110	80961345
chr10	80988895	80989114
chr10	80989394	80989643
chr10	80993728	80994311
chr10	80994445	80994781
chr10	80997181	80997476
chr10	80997763	80998760
chr10	81001529	81001901
chr10	81002005	81003231
chr10	81003358	81003662
chr10	81003772	81004049
chr10	81024155	81024629
chr10	81024894	81025136
chr10	81026133	81026422
chr10	81026545	81026925
chr10	81027973	81028173
chr10	81058331	81058577
chr10	81058697	81059133
chr10	81059778	81060006

chr10	81076414	81076613
chr10	81076860	81078337
chr10	81078470	81078573
chr10	81079402	81079695
chr10	81079880	81079973
chr10	81080069	81082345
chr10	81082487	81082674
chr10	81082898	81083171
chr10	81085314	81085670
chr10	81086581	81086774
chr10	81085047	81085238
chr10	81087546	81087887
chr10	81088104	81088469
chr10	81088632	81089331
chr10	81089448	81089841
chr10	81090930	81091972
chr10	81093005	81093723
chr10	81094416	81094663
chr10	81095583	81095832
chr10	81097669	81097915
chr10	81104117	81104192
chr10	81105140	81105188
chr10	81105262	81105444
chr10	81107147	81108415
chr10	81108698	81108971
chr10	81109084	81109330
chr10	81109534	81109843
chr10	81203772	81205358
chr10	81205861	81206317
chr10	81214033	81214165
chr10	81214455	81214513
chr10	81247725	81248125
chr10	81261880	81262310
chr10	81288931	81289084
chr10	81518380	81518459
chr10	81585121	81587133
chr10	81664248	81664980
chr10	81741111	81741191
chr10	81741496	81742537
chr10	81744344	81744390
chr10	81744513	81744543
chr10	81744975	81745116
chr10	81818552	81818674
chr10	81818860	81818938
chr10	81835444	81835974
chr10	81838142	81838908

chr10	81839060	81839261
chr10	81840643	81840892
chr10	81841300	81842558
chr10	81860600	81860660
chr10	81905394	81905593
chr10	81905656	81905822
chr10	81914448	81915810
chr10	81918136	81918170
chr10	81919207	81919456
chr10	81925329	81925535
chr10	81925705	81926065
chr10	81950740	81950961
chr10	81951222	81951296
chr10	81951454	81951835
chr10	81953499	81953686
chr10	81963763	81963766
chr10	81963840	81966057
chr10	81966360	81967073
chr10	81967238	81967654
chr10	81990748	81990855
chr10	82116596	82116817
chr10	82117170	82117480
chr10	82142999	82143144
chr10	82151864	82152777
chr10	82167285	82168975
chr10	82169218	82169250
chr10	82172485	82172767
chr10	82173041	82173411
chr10	82173685	82174148
chr10	82186649	82186885
chr10	82189022	82189936
chr10	82212227	82213889
chr10	82217337	82217519
chr10	82214005	82216704
chr10	82217833	82218529
chr10	82218661	82219827
chr10	82220172	82220483
chr10	82220700	82221889
chr10	82222059	82222297
chr10	82223192	82223458
chr10	82224203	82224656
chr10	82227944	82228614
chr10	82233022	82233307
chr10	82233443	82234604
chr10	82230518	82232937
chr10	82237688	82237962

chr10	82242889	82243110
chr10	82243536	82243930
chr10	82254526	82255483
chr10	82257413	82257535
chr10	82258064	82258643
chr10	82258958	82259682
chr10	82259792	82259941
chr10	82260207	82260997
chr10	82261069	82262538
chr10	82262668	82262979
chr10	82263108	82263209
chr10	82263450	82263612
chr10	82264037	82264358
chr10	82264822	82267125
chr10	82268102	82268142
chr10	82269340	82269578
chr10	82283716	82283915
chr10	82295433	82295655
chr10	82295865	82296325
chr10	82350473	82350891
chr10	82459519	82459787
chr10	83359902	83360301
chr10	83360375	83360574
chr10	85836066	85836244
chr10	85898902	85899061
chr10	85899266	85899942
chr10	85937577	85937765
chr10	85939188	85939655
chr10	85939755	85940068
chr10	85954272	85954695
chr10	86017113	86017312
chr10	86087901	86088113
chr10	86088344	86089420
chr10	86108121	86108456
chr10	86111944	86112017
chr10	86184680	86184815
chr10	86189697	86189892
chr10	86237920	86237967
chr10	86238232	86238255
chr10	86254033	86254476
chr10	86287548	86287744
chr10	86288695	86288756
chr10	86294374	86294396
chr10	86297046	86297264
chr10	86392353	86392612
chr10	86394385	86394497

chr10	87401151	87401304
chr10	87401542	87401801
chr10	87401918	87403106
chr10	87403959	87404005
chr10	88106960	88107202
chr10	88125891	88126363
chr10	88126606	88126869
chr10	88159485	88159655
chr10	88159864	88160077
chr10	88160185	88160598
chr10	88161361	88161710
chr10	88235879	88236102
chr10	88260766	88260783
chr10	88280594	88281521
chr10	88281709	88282095
chr10	88295134	88295987
chr10	88296180	88296280
chr10	88304530	88304549
chr10	88402349	88402406
chr10	88470090	88470289
chr10	88470533	88472220
chr10	88515342	88516929
chr10	88517183	88517273
chr10	88537166	88537256
chr10	88555472	88555531
chr10	88699003	88699817
chr10	88717792	88718064
chr10	88718441	88718805
chr10	88719104	88719665
chr10	88727878	88728177
chr10	88728403	88730983
chr10	88731249	88732443
chr10	88732571	88733078
chr10	88734309	88734665
chr10	88738148	88738450
chr10	88785518	88785810
chr10	88842048	88842395
chr10	88853001	88853054
chr10	88853221	88853527
chr10	88853759	88854619
chr10	88854975	88856234
chr10	88902212	88902418
chr10	88960652	88961128
chr10	88962977	88965153
chr10	88966280	88966412
chr10	89101074	89103206

chr10	89142926	89143018
chr10	89196204	89196352
chr10	89197202	89197401
chr10	89264239	89265465
chr10	89346271	89346308
chr10	89395472	89395854
chr10	89416256	89416421
chr10	89419252	89419928
chr10	89461384	89461477
chr10	89500137	89500370
chr10	89512405	89512436
chr10	89512544	89512656
chr10	89521542	89521880
chr10	89521984	89522051
chr10	89577364	89577377
chr10	89577557	89577890
chr10	89578121	89578869
chr10	89602002	89602051
chr10	89602190	89602499
chr10	89602864	89602945
chr10	89605879	89606060
chr10	89621229	89621417
chr10	89621564	89622439
chr10	89622652	89624590
chr10	89720235	89720265
chr10	89802245	89802442
chr10	89802809	89802850
chr10	89851303	89851551
chr10	89851700	89851847
chr10	89855409	89855432
chr10	89870075	89870168
chr10	89891329	89891625
chr10	89912814	89913001
chr10	89917647	89917694
chr10	89917993	89918001
chr10	90041594	90041962
chr10	90342758	90343253
chr10	90581066	90581088
chr10	90608225	90608381
chr10	90608600	90608662
chr10	90611726	90611958
chr10	90618512	90618624
chr10	90639588	90640901
chr10	90642361	90642717
chr10	90749780	90749953
chr10	90753762	90754068

chr10	90750368	90751787
chr10	90782803	90782871
chr10	90791844	90792114
chr10	90792381	90792681
chr10	90889731	90889994
chr10	90966908	90967111
chr10	91002144	91002386
chr10	91010888	91011731
chr10	91011903	91012062
chr10	91020423	91020551
chr10	91020761	91020937
chr10	91035074	91035093
chr10	91035274	91035525
chr10	91037914	91038392
chr10	91039140	91039495
chr10	91055491	91055843
chr10	91061656	91062089
chr10	91062492	91062597
chr10	91062716	91062748
chr10	91087664	91088280
chr10	91093671	91093940
chr10	91097230	91097678
chr10	91100066	91100308
chr10	91102614	91102768
chr10	91106237	91106596
chr10	91111654	91112478
chr10	91111217	91111483
chr10	91151633	91151970
chr10	91152117	91152259
chr10	91152447	91152515
chr10	91152860	91153201
chr10	91153678	91153943
chr10	91174037	91175171
chr10	91175295	91175334
chr10	91191724	91191972
chr10	91226289	91226617
chr10	91241403	91241754
chr10	91275289	91275618
chr10	91275819	91276277
chr10	91282327	91282343
chr10	91282498	91282538
chr10	91292331	91292436
chr10	91294861	91295746
chr10	91308500	91308587
chr10	91313124	91313708
chr10	91315468	91315475

chr10	91393628	91393642
chr10	91403486	91403507
chr10	91403942	91405098
chr10	91405227	91405549
chr10	91425346	91425452
chr10	91460832	91461095
chr10	91461351	91462235
chr10	91596793	91597429
chr10	91597605	91597817
chr10	91605750	91605987
chr10	91681462	91681661
chr10	91683765	91683805
chr10	91811671	91811723
chr10	91939382	91939909
chr10	91982397	91982498
chr10	91982778	91982803
chr10	92576996	92577050
chr10	92577184	92577229
chr10	92578697	92578821
chr10	92616922	92617757
chr10	92618970	92619165
chr10	92631704	92632016
chr10	92671650	92671732
chr10	92671988	92671990
chr10	92681434	92681523
chr10	92922178	92922575
chr10	92922731	92923744
chr10	92980516	92980992
chr10	93100612	93100750
chr10	93101040	93101156
chr10	93109083	93109525
chr10	93110439	93110638
chr10	93169125	93170447
chr10	93170617	93170821
chr10	93315058	93315282
chr10	93358756	93358785
chr10	93376934	93377227
chr10	93377367	93377404
chr10	93392600	93393390
chr10	93402507	93402583
chr10	93403756	93403857
chr10	93411994	93412216
chr10	93412469	93412649
chr10	93412977	93413628
chr10	93449676	93449944
chr10	93451058	93451402

chr10	93451729	93451899
chr10	93504503	93505026
chr10	93505203	93505411
chr10	93556020	93556049
chr10	93556160	93556344
chr10	93557838	93557982
chr10	93558152	93558948
chr10	93647597	93647779
chr10	93661560	93661668
chr10	93661944	93661964
chr10	93667782	93668679
chr10	93668891	93669025
chr10	93669154	93669369
chr10	93683057	93683413
chr10	93683562	93684047
chr10	93714995	93715006
chr10	93715217	93715269
chr10	93771457	93771746
chr10	93847871	93847919
chr10	93861387	93861685
chr10	93882980	93883086
chr10	93953779	93954289
chr10	93960249	93960456
chr10	93960643	93960817
chr10	93968418	93968678
chr10	93982654	93982802
chr10	93982923	93983094
chr10	93998452	93998665
chr10	93998812	93998941
chr10	93999171	94000784
chr10	94002886	94002940
chr10	94003236	94003302
chr10	94049985	94051474
chr10	94087567	94087850
chr10	94088821	94088915
chr10	94089055	94089099
chr10	94089667	94090059
chr10	94252834	94252846
chr10	94330689	94330892
chr10	94332111	94332301
chr10	94332739	94334247
chr10	94334774	94335026
chr10	94335680	94335801
chr10	94336013	94336116
chr10	94341388	94341496
chr10	94342064	94342149

chr10	94342384	94342439
chr10	94351197	94351380
chr10	94351659	94351911
chr10	94352550	94352747
chr10	94352877	94353385
chr10	94404271	94404419
chr10	94410868	94410926
chr10	94448986	94450203
chr10	94450324	94450590
chr10	94455578	94455833
chr10	94581899	94582098
chr10	94608312	94608937
chr10	94731769	94732012
chr10	94790213	94790566
chr10	94822634	94823110
chr10	94897810	94897854
chr10	94898171	94898595
chr10	94898824	94898954
chr10	94899094	94899125
chr10	95048616	95048636
chr10	95095171	95095257
chr10	95095328	95095420
chr10	95099609	95099779
chr10	95136237	95136474
chr10	95136629	95137065
chr10	95139451	95139641
chr10	95139789	95139874
chr10	95143359	95144329
chr10	95163420	95163957
chr10	95166181	95167303
chr10	95172980	95173046
chr10	95173324	95173401
chr10	95183519	95183744
chr10	95184327	95184427
chr10	95184831	95184923
chr10	95186408	95186619
chr10	95194894	95195153
chr10	95196248	95197783
chr10	95198426	95198673
chr10	95198789	95199180
chr10	95201835	95202389
chr10	95205216	95205246
chr10	95205619	95206863
chr10	95218219	95219130
chr10	95221545	95222114
chr10	95222678	95223400

chr10	95224611	95224962
chr10	95225383	95225648
chr10	95223800	95224424
chr10	95225800	95226589
chr10	95228203	95229306
chr10	95230804	95232059
chr10	95235503	95236125
chr10	95241875	95242442
chr10	95242763	95243034
chr10	95255613	95256014
chr10	95256302	95256821
chr10	95360828	95361057
chr10	95461638	95462185
chr10	95462487	95462871
chr10	95653426	95654185
chr10	95943291	95943406
chr10	96122235	96122640
chr10	96122879	96123027
chr10	96141062	96141098
chr10	96161890	96162082
chr10	96162359	96163885
chr10	96204093	96204113
chr10	96304209	96304414
chr10	96304610	96305423
chr10	96305573	96306201
chr10	96312285	96312613
chr10	96943115	96943893
chr10	96974611	96974677
chr10	96980887	96981291
chr10	96989176	96989884
chr10	96995692	96995729
chr10	96995975	96996089
chr10	97006297	97006359
chr10	97006890	97007723
chr10	97007940	97008039
chr10	97008441	97008457
chr10	97008566	97008781
chr10	97020318	97020379
chr10	97020557	97020764
chr10	97020921	97021036
chr10	97021174	97021376
chr10	97024289	97024512
chr10	97026414	97026914
chr10	97033162	97033749
chr10	97035407	97035751
chr10	97035881	97036212

chr10	97037000	97037326
chr10	97039229	97039459
chr10	97045857	97046095
chr10	97046821	97046842
chr10	97049402	97049789
chr10	97050086	97051083
chr10	97054738	97055423
chr10	97068561	97068864
chr10	97068998	97069027
chr10	97094616	97094678
chr10	97190095	97190444
chr10	97312719	97312841
chr10	97415357	97416497
chr10	97453304	97453660
chr10	97454142	97454178
chr10	97666706	97667933
chr10	97802941	97804215
chr10	97804373	97804583
chr10	97849316	97850108
chr10	97884247	97884913
chr10	97889382	97890633
chr10	98040540	98040856
chr10	98262103	98262576
chr10	98264519	98265306
chr10	98272506	98272739
chr10	98273185	98273484
chr10	98338635	98338815
chr10	98345935	98347228
chr10	98480078	98480528
chr10	98561330	98561596
chr10	98590391	98590393
chr10	98591427	98591917
chr10	98592058	98593576
chr10	98600484	98601087
chr10	98602751	98603119
chr10	98610766	98610949
chr10	98616180	98616537
chr10	98624041	98624190
chr10	99039167	99039567
chr10	99051699	99052422
chr10	99078465	99079889
chr10	99080028	99080249
chr10	99092390	99093595
chr10	99093758	99095152
chr10	99096642	99097055
chr10	99159785	99160519

chr10	99160588	99161428
chr10	99166221	99166780
chr10	99166897	99167305
chr10	99167609	99167615
chr10	99168054	99168333
chr10	99168974	99169000
chr10	99169136	99169234
chr10	99171231	99171548
chr10	99172426	99173259
chr10	99173694	99173852
chr10	99182510	99182586
chr10	99182838	99182968
chr10	99184403	99184629
chr10	99184707	99185079
chr10	99185345	99185599
chr10	99185943	99186602
chr10	99186713	99187097
chr10	99187234	99187363
chr10	99187528	99187836
chr10	99200320	99200359
chr10	99200492	99200519
chr10	99205174	99205743
chr10	99205863	99206450
chr10	99206733	99206859
chr10	99217609	99217772
chr10	99223015	99223360
chr10	99223795	99224118
chr10	99225990	99227033
chr10	99227847	99228388
chr10	99239162	99239527
chr10	99240579	99240741
chr10	99240958	99241301
chr10	99257382	99258173
chr10	99258767	99259542
chr10	99298709	99298830
chr10	99299258	99299297
chr10	99300033	99300243
chr10	99300894	99301093
chr10	99301609	99302011
chr10	99302338	99302704
chr10	99303069	99303358
chr10	99309285	99309916
chr10	99310105	99310368
chr10	99312871	99313250
chr10	99313500	99313696
chr10	99330257	99330408

chr10	99330536	99331412
chr10	99331643	99332165
chr10	99332275	99332469
chr10	99332809	99332978
chr10	99333143	99333194
chr10	99333591	99334614
chr10	99334722	99335165
chr10	99336960	99337018
chr10	99343738	99344061
chr10	99355810	99356433
chr10	99356565	99356748
chr10	99369097	99369431
chr10	99371365	99371625
chr10	99372136	99372324
chr10	99392709	99394095
chr10	99399968	99400302
chr10	99400447	99400991
chr10	99401137	99401679
chr10	99402824	99403023
chr10	99405028	99405313
chr10	99407266	99407727
chr10	99408666	99408936
chr10	99409070	99409287
chr10	99409960	99410173
chr10	99411393	99411940
chr10	99436429	99436472
chr10	99438744	99439030
chr10	99439150	99439440
chr10	99443339	99443545
chr10	99443619	99444386
chr10	99445005	99445847
chr10	99446067	99446973
chr10	99447370	99448222
chr10	99449166	99450543
chr10	99461105	99461766
chr10	99466321	99466529
chr10	99470934	99470945
chr10	99472813	99473467
chr10	99473658	99475856
chr10	99477655	99479775
chr10	99480071	99480394
chr10	99480485	99480688
chr10	99481510	99481537
chr10	99481720	99481748
chr10	99484036	99484683
chr10	99486590	99486902

chr10	99488579	99488624
chr10	99490055	99490221
chr10	99491489	99491808
chr10	99493492	99493693
chr10	99496312	99496657
chr10	99496917	99497819
chr10	99534487	99534617
chr10	99545037	99545372
chr10	99545474	99545582
chr10	99545874	99546408
chr10	99546611	99546785
chr10	99581707	99582018
chr10	99609595	99609794
chr10	99629050	99629253
chr10	99648913	99650035
chr10	99894067	99895095
chr10	99902174	99902501
chr10	99971997	99972088
chr10	100164343	100164536
chr10	100174514	100174991
chr10	100206017	100206938
chr10	100227793	100227854
chr10	101088846	101089296
chr10	101089708	101089907
chr10	101152445	101152651
chr10	101154825	101155066
chr10	101156125	101156277
chr10	101190172	101190439
chr10	101190671	101191460
chr10	101379059	101379284
chr10	101379443	101381606
chr10	101414162	101414632
chr10	101414810	101414949
chr10	101416318	101416444
chr10	101416672	101416829
chr10	101418226	101418991
chr10	101419212	101420017
chr10	101421091	101421252
chr10	101421424	101421547
chr10	101441289	101441293
chr10	101441601	101441703
chr10	101442534	101442829
chr10	101445417	101445663
chr10	101449225	101449537
chr10	101491500	101491878
chr10	101491993	101492744

chr10	101519870	101520112
chr10	101520630	101520987
chr10	101535702	101536045
chr10	101539185	101539384
chr10	101539527	101540088
chr10	101545445	101545691
chr10	101594091	101594282
chr10	101600247	101600470
chr10	101666126	101666149
chr10	101666590	101666727
chr10	101677867	101678093
chr10	101680623	101680755
chr10	101680926	101681063
chr10	101683385	101683390
chr10	101690864	101691192
chr10	101692700	101692722
chr10	101722931	101723237
chr10	101736743	101737125
chr10	101743394	101743639
chr10	101743997	101744358
chr10	101754062	101754131
chr10	101754774	101754780
chr10	101765229	101765432
chr10	101765904	101766744
chr10	101767767	101767982
chr10	101768852	101769230
chr10	101769405	101770405
chr10	101770533	101770621
chr10	101803026	101803228
chr10	101803340	101803926
chr10	101804183	101804465
chr10	101806061	101806159
chr10	101939104	101939457
chr10	101943264	101943324
chr10	101943669	101943671
chr10	101945074	101946422
chr10	101946489	101946979
chr10	101988564	101989056
chr10	101989120	101989629
chr10	102027292	102027676
chr10	102045681	102046529
chr10	102046826	102046933
chr10	102105670	102106767
chr10	102105009	102105478
chr10	102106899	102107424
chr10	102107521	102107921

chr10	102108132	102108606
chr10	102108891	102109090
chr10	102109446	102109988
chr10	102110404	102110663
chr10	102116967	102116989
chr10	102118425	102118639
chr10	102118725	102118780
chr10	102122458	102122693
chr10	102123342	102123701
chr10	102124310	102124550
chr10	102124913	102125089
chr10	102125166	102125936
chr10	102130272	102131424
chr10	102132661	102133145
chr10	102133257	102134729
chr10	102241652	102241877
chr10	102280888	102281360
chr10	102289242	102290086
chr10	102295321	102295393
chr10	102295767	102295989
chr10	102322182	102322759
chr10	102413862	102414093
chr10	102414799	102415027
chr10	102415859	102416102
chr10	102672701	102673382
chr10	102728646	102729097
chr10	102729325	102729855
chr10	102746593	102748074
chr10	102756312	102760229
chr10	102773090	102773397
chr10	102791058	102791561
chr10	102791723	102792192
chr10	102801251	102801319
chr10	102802790	102803469
chr10	102812330	102812482
chr10	102819964	102820800
chr10	102820974	102823067
chr10	102825626	102825965
chr10	102826291	102826918
chr10	102827133	102827788
chr10	102880813	102881247
chr10	102981711	102981910
chr10	103113327	103113419
chr10	103113875	103114575
chr10	103124536	103124735
chr10	103126876	103127439

chr10	103127649	103128194
chr10	103140321	103140399
chr10	103329445	103330019
chr10	103330197	103330786
chr10	103347426	103347845
chr10	103348566	103348950
chr10	103453528	103453562
chr10	103453731	103455241
chr10	103542150	103542457
chr10	103542565	103543827
chr10	103574980	103575160
chr10	103575799	103576034
chr10	103577135	103577976
chr10	103578139	103578460
chr10	103578659	103579420
chr10	103590466	103591259
chr10	103596218	103596613
chr10	103655781	103655905
chr10	103668933	103669078
chr10	103698336	103698463
chr10	103698949	103699148
chr10	103711411	103711438
chr10	103815254	103816049
chr10	103816172	103816270
chr10	103824536	103826183
chr10	103872557	103872954
chr10	103873146	103875141
chr10	103876045	103876390
chr10	103875939	103875981
chr10	103877162	103877664
chr10	103877799	103877998
chr10	103878146	103878399
chr10	103878714	103879071
chr10	103879181	103879415
chr10	103879607	103880952
chr10	103881065	103881449
chr10	103887654	103888512
chr10	103891459	103891848
chr10	103892065	103892258
chr10	103892389	103892614
chr10	103892758	103894022
chr10	103894206	103894234
chr10	103895765	103895964
chr10	103910972	103911801
chr10	103912122	103912649
chr10	103914542	103914742

chr10	103929332	103929845
chr10	103957833	103958221
chr10	103986056	103986367
chr10	103989944	103990019
chr10	103990096	103990172
chr10	103990306	103990846
chr10	103991117	103991486
chr10	104001193	104001404
chr10	104001543	104001784
chr10	104003598	104003819
chr10	104004741	104004970
chr10	104005353	104005709
chr10	104012281	104012546
chr10	104031921	104031958
chr10	104084087	104084108
chr10	104102213	104102580
chr10	104102698	104102834
chr10	104128121	104128233
chr10	104138604	104138734
chr10	104152203	104152345
chr10	104153571	104153599
chr10	104153812	104154100
chr10	104154358	104154577
chr10	104154894	104156222
chr10	104158698	104159306
chr10	104159502	104159701
chr10	104161045	104161510
chr10	104161798	104162066
chr10	104179694	104180267
chr10	104180372	104180422
chr10	104180645	104181193
chr10	104181311	104182600
chr10	104190001	104190173
chr10	104191469	104192042
chr10	104192169	104192371
chr10	104192567	104192748
chr10	104193073	104193150
chr10	104193792	104194048
chr10	104194271	104194815
chr10	104209301	104210014
chr10	104210142	104211223
chr10	104211485	104211716
chr10	104213124	104213345
chr10	104213854	104213858
chr10	104215855	104216050
chr10	104221162	104221589

chr10	104222312	104223382
chr10	104238444	104238705
chr10	104247279	104247478
chr10	104249282	104249613
chr10	104251945	104252151
chr10	104261564	104261705
chr10	104262032	104262511
chr10	104262821	104262969
chr10	104263763	104264112
chr10	104264280	104264633
chr10	104278607	104278619
chr10	104302298	104302654
chr10	104315951	104316214
chr10	104344027	104344036
chr10	104363751	104364581
chr10	104367514	104367584
chr10	104387126	104389537
chr10	104390294	104390355
chr10	104390422	104390516
chr10	104392460	104393131
chr10	104393485	104393804
chr10	104394104	104394570
chr10	104396592	104396858
chr10	104401409	104401453
chr10	104401734	104404434
chr10	104404884	104405727
chr10	104405895	104406588
chr10	104422146	104422403
chr10	104423206	104423403
chr10	104456444	104456521
chr10	104456834	104456931
chr10	104459891	104460097
chr10	104460255	104460256
chr10	104473174	104474044
chr10	104474386	104475263
chr10	104475672	104475870
chr10	104502989	104503231
chr10	104503668	104504166
chr10	104515492	104515623
chr10	104523639	104523838
chr10	104528993	104529976
chr10	104531653	104532642
chr10	104535751	104535753
chr10	104536019	104536085
chr10	104536502	104537227
chr10	104539323	104539485

chr10	104539759	104539986
chr10	104578277	104578390
chr10	104579293	104579493
chr10	104588942	104589001
chr10	104613697	104614536
chr10	104615399	104616176
chr10	104629274	104629532
chr10	104677424	104677623
chr10	104678048	104679110
chr10	104874653	104875123
chr10	104913740	104914008
chr10	104951851	104952076
chr10	104952250	104953011
chr10	104986911	104987178
chr10	104989814	104990019
chr10	104990658	104991087
chr10	104991331	104991483
chr10	104992332	104993202
chr10	104999781	104999912
chr10	105001049	105001187
chr10	105001304	105002069
chr10	105036912	105037221
chr10	105037443	105037669
chr10	105044128	105044441
chr10	105110202	105110841
chr10	105111030	105111153
chr10	105116224	105116346
chr10	105120446	105120660
chr10	105120841	105121020
chr10	105127459	105127635
chr10	105127773	105128231
chr10	105128633	105128737
chr10	105155520	105155870
chr10	105156042	105156282
chr10	105156501	105156671
chr10	105206389	105206588
chr10	105211684	105212423
chr10	105245448	105245537
chr10	105250055	105250139
chr10	105250262	105250412
chr10	105253421	105254518
chr10	105263934	105264082
chr10	105287173	105288280
chr10	105314862	105315298
chr10	105325547	105325693
chr10	105327070	105327115

chr10	105343742	105343946
chr10	105344147	105344498
chr10	105345210	105345250
chr10	105345467	105345538
chr10	105351464	105351663
chr10	105435592	105435904
chr10	105436084	105437416
chr10	105437615	105437948
chr10	105439684	105439826
chr10	105446564	105446620
chr10	105448091	105448221
chr10	105455218	105455363
chr10	105456111	105456316
chr10	105469653	105469930
chr10	105470491	105470655
chr10	105474418	105474562
chr10	105475784	105476237
chr10	105476377	105476665
chr10	105476953	105478250
chr10	105495237	105495494
chr10	105495574	105495780
chr10	105496103	105496307
chr10	105496499	105496997
chr10	105498813	105498890
chr10	105499373	105499765
chr10	105504158	105504247
chr10	105505047	105505577
chr10	105505725	105506066
chr10	105506159	105506482
chr10	105506925	105507873
chr10	105508018	105508744
chr10	105516501	105516904
chr10	105517099	105517496
chr10	105517728	105519707
chr10	105519928	105520270
chr10	105521982	105522223
chr10	105522981	105523180
chr10	105524983	105525274
chr10	105529991	105530119
chr10	105531376	105531540
chr10	105532268	105533042
chr10	105539433	105539687
chr10	105540661	105541596
chr10	105542283	105542567
chr10	105544541	105544872
chr10	105545759	105546007

chr10	105546188	105546235
chr10	105546506	105546620
chr10	105549115	105549604
chr10	105549953	105550169
chr10	105552468	105552489
chr10	105556708	105556847
chr10	105560867	105561076
chr10	105576503	105576566
chr10	105609939	105610045
chr10	105610149	105610534
chr10	105611407	105611432
chr10	105612038	105612204
chr10	105613627	105614212
chr10	105614574	105616159
chr10	105616639	105616757
chr10	105619752	105619803
chr10	105629046	105629082
chr10	105629232	105629356
chr10	105630133	105631150
chr10	105631440	105632156
chr10	105632370	105632609
chr10	105632916	105633409
chr10	105639161	105639544
chr10	105641180	105642636
chr10	105644370	105644552
chr10	105644691	105644787
chr10	105647766	105647789
chr10	105647941	105648140
chr10	105648254	105648420
chr10	105649877	105650451
chr10	105660053	105660087
chr10	105660326	105660473
chr10	105667945	105668030
chr10	105668373	105668515
chr10	105670555	105670753
chr10	105677178	105677368
chr10	105676699	105677049
chr10	105677526	105677891
chr10	105678312	105678527
chr10	105683219	105683395
chr10	105701584	105701733
chr10	105718870	105718879
chr10	105726390	105727539
chr10	105728977	105729528
chr10	105737222	105737334
chr10	105794662	105794770

chr10	105814992	105815262
chr10	105841788	105842153
chr10	105842519	105842718
chr10	105844337	105844605
chr10	105845499	105846296
chr10	105850468	105850723
chr10	105853480	105854177
chr10	105869557	105870125
chr10	105877470	105878157
chr10	105880577	105881077
chr10	105881279	105881495
chr10	105882016	105882392
chr10	105961659	105961690
chr10	105991355	105991591
chr10	105991678	105992070
chr10	105992291	105992643
chr10	106014381	106015786
chr10	106016991	106017395
chr10	106019322	106019528
chr10	106020423	106020785
chr10	106028296	106028748
chr10	106029066	106029212
chr10	106034408	106034566
chr10	106034674	106035510
chr10	106052736	106053039
chr10	106053383	106053571
chr10	106060169	106061129
chr10	106079676	106079797
chr10	106080174	106080573
chr10	106080684	106080909
chr10	106081349	106081598
chr10	106081688	106082259
chr10	106082460	106082793
chr10	106083609	106083720
chr10	106083926	106084165
chr10	106087152	106087431
chr10	106087671	106088478
chr10	106088551	106089024
chr10	106089329	106090344
chr10	106090457	106090675
chr10	106090863	106091031
chr10	106091436	106091768
chr10	106095678	106095776
chr10	106096262	106096765
chr10	106097293	106099236
chr10	106099327	106099678

chr10	106109246	106110100
chr10	106184879	106184955
chr10	106205053	106205125
chr10	106211437	106211639
chr10	106569700	106569777
chr10	107557044	107557188
chr10	107557406	107557433
chr10	107711872	107711920
chr10	107712282	107712313
chr10	107729846	107729952
chr10	107838594	107838604
chr10	107838786	107838998
chr10	108120546	108120674
chr10	108158061	108158157
chr10	108158505	108158517
chr10	108161163	108161263
chr10	108183733	108184336
chr10	108184732	108185185
chr10	108195980	108196536
chr10	108199149	108199409
chr10	108200271	108201005
chr10	108266297	108267026
chr10	108331292	108331669
chr10	108332818	108332920
chr10	108333056	108333714
chr10	108336998	108337164
chr10	108337363	108337562
chr10	108412076	108412145
chr10	108590324	108590662
chr10	109306389	109306424
chr10	109413757	109414098
chr10	110075684	110075891
chr10	110118563	110118698
chr10	111652645	111652741
chr10	111654003	111654412
chr10	111655229	111655231
chr10	111672611	111672747
chr10	111672957	111673116
chr10	111682437	111683218
chr10	111683366	111683815
chr10	111721326	111721357
chr10	111767240	111767585
chr10	111767743	111768052
chr10	111768159	111768824
chr10	111895748	111895977
chr10	111896902	111897221

chr10	111900921	111900947
chr10	111967702	111968661
chr10	111968889	111969188
chr10	111969346	111969773
chr10	111969876	111970976
chr10	111971179	111971495
chr10	111984892	111984913
chr10	111985833	111986139
chr10	111986263	111986512
chr10	111986962	111987530
chr10	112033211	112033269
chr10	112033532	112033634
chr10	112039143	112039239
chr10	112064059	112064724
chr10	112064864	112065296
chr10	112065873	112065968
chr10	112066220	112066272
chr10	112071380	112071465
chr10	112086554	112086622
chr10	112095811	112096043
chr10	112096317	112096455
chr10	112112862	112113677
chr10	112113993	112114744
chr10	112116589	112116639
chr10	112116937	112117539
chr10	112117695	112117878
chr10	112119393	112119680
chr10	112123740	112123818
chr10	112124030	112124466
chr10	112124651	112124850
chr10	112128911	112129110
chr10	112152391	112152808
chr10	112153065	112153369
chr10	112155631	112155837
chr10	112156007	112156206
chr10	112157865	112158447
chr10	112158618	112159279
chr10	112159458	112159602
chr10	112161241	112161574
chr10	112165802	112165867
chr10	112167508	112168530
chr10	112168623	112169551
chr10	112169960	112172261
chr10	112172434	112172907
chr10	112174081	112174914
chr10	112176662	112176974

chr10	112177106	112177693
chr10	112180051	112180429
chr10	112180683	112180820
chr10	112188062	112188104
chr10	112188336	112188394
chr10	112205879	112206058
chr10	112206691	112207474
chr10	112214108	112214173
chr10	112216096	112216347
chr10	112216517	112216716
chr10	112216859	112217143
chr10	112241871	112242002
chr10	112247137	112248094
chr10	112250302	112250320
chr10	112253552	112253878
chr10	112254629	112255165
chr10	112255398	112256758
chr10	112256948	112257816
chr10	112257936	112258350
chr10	112258455	112258691
chr10	112258803	112259663
chr10	112259901	112259958
chr10	112260081	112260140
chr10	112260283	112261154
chr10	112261311	112261451
chr10	112262599	112263039
chr10	112272331	112272559
chr10	112272780	112272979
chr10	112277893	112278141
chr10	112285325	112285406
chr10	112285484	112285840
chr10	112286259	112286483
chr10	112287355	112287368
chr10	112287501	112287829
chr10	112288164	112288480
chr10	112288818	112289233
chr10	112289474	112290549
chr10	112290897	112291261
chr10	112307237	112307284
chr10	112326763	112326973
chr10	112327166	112328448
chr10	112349646	112349859
chr10	112368657	112368686
chr10	112368940	112368994
chr10	112369452	112369695
chr10	112392800	112393034

chr10	112403881	112404528
chr10	112424571	112424877
chr10	112425114	112425179
chr10	112431988	112432407
chr10	112501945	112502135
chr10	112505896	112506188
chr10	112518669	112518822
chr10	112518960	112519022
chr10	112611918	112612110
chr10	112612308	112612530
chr10	112612606	112612611
chr10	112612740	112613087
chr10	112620782	112620876
chr10	112620995	112621021
chr10	112621946	112622141
chr10	112622377	112622580
chr10	112625201	112625655
chr10	112631229	112631425
chr10	112631539	112632768
chr10	112653333	112653445
chr10	112661936	112662118
chr10	112662238	112662466
chr10	112678145	112678929
chr10	112679330	112679737
chr10	112679853	112680281
chr10	113943291	113943429
chr10	113943569	113943967
chr10	114121667	114121838
chr10	114122593	114122817
chr10	114205989	114206193
chr10	114206466	114206502
chr10	114206699	114207500
chr10	114277319	114277529
chr10	114325556	114325564
chr10	114344815	114344951
chr10	114345105	114345173
chr10	114350558	114350614
chr10	114350826	114350992
chr10	114391286	114391398
chr10	114476218	114476267
chr10	114484524	114484528
chr10	114501715	114501838
chr10	114502066	114502132
chr10	114502995	114503121
chr10	114526433	114526513
chr10	114526724	114526777

chr10	114571322	114571586
chr10	114709265	114709685
chr10	114710263	114710314
chr10	114710395	114710917
chr10	114711201	114712712
chr10	114712837	114713426
chr10	114713631	114713930
chr10	114748635	114748655
chr10	114750200	114750487
chr10	114751071	114751306
chr10	114751487	114751707
chr10	114751850	114752275
chr10	114752408	114752494
chr10	114816738	114816797
chr10	114818684	114818826
chr10	114826944	114826992
chr10	114827314	114827636
chr10	114829141	114829411
chr10	114829612	114829842
chr10	114833218	114834008
chr10	114846820	114847112
chr10	114853180	114853379
chr10	114853483	114853807
chr10	114858696	114858947
chr10	114859137	114859546
chr10	114886573	114886843
chr10	114891330	114891541
chr10	114891788	114891870
chr10	115100931	115101011
chr10	115438664	115438840
chr10	115438990	115439152
chr10	115439489	115439916
chr10	115440138	115440541
chr10	115440872	115440962
chr10	115548299	115548351
chr10	115548765	115548910
chr10	115613571	115614097
chr10	115614195	115616175
chr10	115655217	115655236
chr10	115784479	115784821
chr10	115786177	115786910
chr10	115789921	115790401
chr10	115802568	115804647
chr10	115804970	115805523
chr10	115820901	115821430
chr10	115827869	115828245

chr10	115828391	115828502
chr10	115860765	115861191
chr10	115911210	115911217
chr10	115911662	115911664
chr10	115933601	115934231
chr10	115999244	115999674
chr10	116004570	116005703
chr10	116005860	116006059
chr10	116006643	116007206
chr10	116031401	116031600
chr10	116066196	116066497
chr10	116066723	116067002
chr10	116079869	116080429
chr10	116080753	116080994
chr10	116163017	116163225
chr10	116163448	116165190
chr10	116259388	116259441
chr10	116259879	116260076
chr10	116260187	116260531
chr10	116264975	116265029
chr10	116265247	116265357
chr10	116278370	116278630
chr10	116278882	116279042
chr10	116279244	116279468
chr10	116280081	116280273
chr10	116280435	116280657
chr10	116285076	116285643
chr10	116286099	116286560
chr10	116286718	116287087
chr10	116384774	116384857
chr10	116391415	116391461
chr10	116391531	116391893
chr10	116392851	116393088
chr10	116444104	116444302
chr10	116444692	116444891
chr10	116539503	116539509
chr10	116539879	116540187
chr10	116581193	116582935
chr10	116616339	116616755
chr10	116636728	116636943
chr10	116698024	116698359
chr10	116852628	116853290
chr10	117614310	117614634
chr10	117621382	117621520
chr10	118188836	118189138
chr10	118189799	118189856

chr10	118190075	118190227
chr10	118199981	118200514
chr10	118501560	118502688
chr10	118507532	118507563
chr10	118507797	118508039
chr10	118513219	118513394
chr10	118530394	118530955
chr10	118531267	118531502
chr10	118533974	118534266
chr10	118535044	118535416
chr10	118537387	118537530
chr10	118608419	118608950
chr10	118609289	118609763
chr10	118648648	118648719
chr10	118706980	118707069
chr10	118707353	118707709
chr10	118763802	118764003
chr10	118764193	118764648
chr10	118896631	118896842
chr10	118896952	118897269
chr10	118976582	118976948
chr10	119133272	119133419
chr10	119133606	119134453
chr10	119134616	119135620
chr10	119164240	119164447
chr10	119215897	119215999
chr10	119301891	119302090
chr10	119762229	119762573
chr10	119805398	119806733
chr10	120101614	120101941
chr10	120102643	120102769
chr10	120102928	120102983
chr10	120183617	120183720
chr10	120219071	120219270
chr10	120260629	120261285
chr10	120261507	120261857
chr10	120263965	120264231
chr10	120264381	120264456
chr10	120282131	120282408
chr10	120282591	120283034
chr10	120283283	120283486
chr10	120283607	120283723
chr10	120513491	120514501
chr10	120515118	120515605
chr10	120552123	120552213
chr10	120553457	120553526

chr10	120591611	120591887
chr10	120599373	120599753
chr10	120629904	120630027
chr10	120630264	120630890
chr10	120718941	120719037
chr10	120719550	120719965
chr10	120759997	120760122
chr10	120760680	120760985
chr10	120763798	120763836
chr10	120766715	120766842
chr10	120788938	120789729
chr10	120790039	120790573
chr10	120791119	120791378
chr10	120835336	120835465
chr10	120837021	120837265
chr10	120839234	120841070
chr10	120856363	120856613
chr10	120857629	120857851
chr10	120859965	120860780
chr10	120861008	120862106
chr10	120862384	120862534
chr10	120862679	120863025
chr10	120863188	120863552
chr10	120863685	120864501
chr10	120873231	120873430
chr10	120879018	120879211
chr10	120922082	120922219
chr10	120924465	120924515
chr10	120924646	120925977
chr10	120937441	120938353
chr10	120938552	120938699
chr10	120949810	120950388
chr10	120951389	120951567
chr10	120951681	120951871
chr10	120957210	120957490
chr10	120958363	120958431
chr10	120961039	120961167
chr10	120965777	120967096
chr10	120967222	120967908
chr10	120968082	120968189
chr10	120968557	120968895
chr10	121004703	121005202
chr10	121010004	121010214
chr10	121013628	121013812
chr10	121014320	121014487
chr10	121030799	121031004

chr10	121040354	121040573
chr10	121063749	121064033
chr10	121064224	121064377
chr10	121065112	121065221
chr10	121065373	121065692
chr10	121066125	121066324
chr10	121074929	121075226
chr10	121075463	121075686
chr10	121075793	121075877
chr10	121076176	121076375
chr10	121076598	121077001
chr10	121275603	121275802
chr10	121277109	121277222
chr10	121280825	121280880
chr10	121281355	121281508
chr10	121282501	121282683
chr10	121282911	121283013
chr10	121288675	121288754
chr10	121301560	121303111
chr10	121307932	121308017
chr10	121308781	121309146
chr10	121309431	121309507
chr10	121355372	121356540
chr10	121356950	121357091
chr10	121410431	121412312
chr10	121412763	121412989
chr10	121414310	121414794
chr10	121414978	121415511
chr10	121416549	121416665
chr10	121417829	121418263
chr10	121418724	121418812
chr10	121425434	121425829
chr10	121426453	121426580
chr10	121426882	121427344
chr10	121427561	121427777
chr10	121427904	121427911
chr10	121430042	121430082
chr10	121430229	121430321
chr10	121430457	121430681
chr10	121431181	121431214
chr10	121434524	121434851
chr10	121435492	121435767
chr10	121438093	121438398
chr10	121441438	121442396
chr10	121438538	121441022
chr10	121445874	121446163

chr10	121446372	121446583
chr10	121446841	121447163
chr10	121484703	121486109
chr10	121494480	121494784
chr10	121497333	121497408
chr10	121497605	121497740
chr10	121510089	121510518
chr10	121520624	121520677
chr10	121527784	121527925
chr10	121528301	121528384
chr10	121529405	121529498
chr10	121529705	121529728
chr10	121577417	121577490
chr10	121577673	121578594
chr10	121631412	121633052
chr10	121651685	121653268
chr10	122183178	122183209
chr10	122216394	122216857
chr10	122264103	122264211
chr10	122271009	122271042
chr10	122610556	122610712
chr10	122611027	122611236
chr10	122677123	122677497
chr10	122677643	122677774
chr10	122708749	122708978
chr10	122709675	122709878
chr10	122725530	122725693
chr10	122829026	122829099
chr10	122836768	122836842
chr10	122837102	122838284
chr10	122838660	122838808
chr10	122851752	122851770
chr10	122851883	122852256
chr10	122872578	122872593
chr10	122872812	122872917
chr10	123020169	123020189
chr10	123072244	123072440
chr10	123072606	123072699
chr10	123072858	123073050
chr10	123084286	123084358
chr10	123086446	123086459
chr10	123092333	123092521
chr10	123334124	123334365
chr10	123336324	123336403
chr10	123336860	123337150
chr10	123338610	123338655

chr10	123356411	123357061
chr10	123357584	123357881
chr10	123358030	123358525
chr10	123367624	123367823
chr10	123367951	123368720
chr10	123370537	123370744
chr10	123686908	123688416
chr10	123690017	123690340
chr10	123690829	123690975
chr10	123716001	123716200
chr10	123734222	123735067
chr10	123754510	123755355
chr10	123779245	123779508
chr10	123816452	123816656
chr10	123822367	123822802
chr10	123823093	123823186
chr10	123827086	123827285
chr10	123829292	123829403
chr10	123841262	123841556
chr10	123844292	123844928
chr10	123845164	123845644
chr10	123845866	123846128
chr10	123846420	123846446
chr10	123848883	123849152
chr10	123849299	123849574
chr10	123856502	123856929
chr10	123860071	123860277
chr10	123861269	123861463
chr10	123862117	123862122
chr10	123871795	123872209
chr10	123872328	123873224
chr10	123873376	123873800
chr10	123876120	123876272
chr10	123880556	123880616
chr10	123899945	123900199
chr10	123900354	123900806
chr10	123901052	123901299
chr10	123901594	123901658
chr10	123906377	123906472
chr10	123906694	123906933
chr10	123916253	123916274
chr10	124023485	124023654
chr10	124039156	124039313
chr10	124041705	124041879
chr10	124050590	124050789
chr10	124052091	124052169

chr10	124053353	124053652
chr10	124053906	124054101
chr10	124054368	124054607
chr10	124054898	124054941
chr10	124058613	124058815
chr10	124067102	124067969
chr10	124068229	124068756
chr10	124069613	124069718
chr10	124084176	124084243
chr10	124107953	124108059
chr10	124133124	124133912
chr10	124134105	124135219
chr10	124148993	124149311
chr10	124180579	124180651
chr10	124180824	124180932
chr10	124201462	124201846
chr10	124220504	124220922
chr10	124221052	124222666
chr10	124222860	124223085
chr10	124228246	124228612
chr10	124228991	124229317
chr10	124234658	124234930
chr10	124234996	124235112
chr10	124235341	124235695
chr10	124236022	124236203
chr10	124236408	124236612
chr10	124250709	124251369
chr10	124251515	124251899
chr10	124253170	124253313
chr10	124253411	124253893
chr10	124254133	124255045
chr10	124268732	124268917
chr10	124269018	124269237
chr10	124269527	124269852
chr10	124270156	124270320
chr10	124325289	124325891
chr10	124611742	124611862
chr10	124614452	124614598
chr10	124621130	124621801
chr10	124638384	124639094
chr10	124639285	124639894
chr10	124713168	124714149
chr10	124714472	124714672
chr10	124717426	124717427
chr10	124739841	124740223
chr10	124740461	124740524

chr10	124767317	124768320
chr10	124768533	124768754
chr10	124907991	124908220
chr10	124909271	124909853
chr10	124913869	124914372
chr10	124914509	124914786
chr10	124915828	124916027
chr10	125751224	125751531
chr10	125754403	125755835
chr10	125756863	125757024
chr10	125757353	125758052
chr10	125760051	125760084
chr10	125760189	125760312
chr10	125770433	125770640
chr10	125770887	125770921
chr10	125813541	125813590
chr10	125813830	125813890
chr10	125828397	125828412
chr10	125829180	125829388
chr10	125850994	125853877
chr10	125864546	125864837
chr10	125865121	125865766
chr10	125866159	125866736
chr10	125978782	125978790
chr10	125978861	125978981
chr10	125984584	125984806
chr10	126028731	126029095
chr10	126029422	126029604
chr10	126030088	126030563
chr10	126043396	126043802
chr10	126045290	126046008
chr10	126077533	126078099
chr10	126082751	126083047
chr10	126083181	126083245
chr10	126083529	126083780
chr10	126106354	126107480
chr10	126107665	126107970
chr10	126124691	126124935
chr10	126125177	126125728
chr10	126128372	126128782
chr10	126129011	126129505
chr10	126137358	126137569
chr10	126138493	126138809
chr10	126149841	126151025
chr10	126160617	126160921
chr10	126166620	126166951

chr10	126195805	126196004
chr10	126199114	126199468
chr10	126218486	126218830
chr10	126228063	126228262
chr10	126307589	126307805
chr10	126315425	126315700
chr10	126320869	126321253
chr10	126321576	126321612
chr10	126336123	126338133
chr10	126342722	126342924
chr10	126343120	126343363
chr10	126343628	126344192
chr10	126429238	126429458
chr10	126431221	126431819
chr10	126431928	126432214
chr10	126432319	126432864
chr10	126433560	126433622
chr10	126434707	126434719
chr10	126479689	126480660
chr10	126480775	126481377
chr10	126481653	126481671
chr10	126490017	126490846
chr10	126547910	126548231
chr10	126554017	126554072
chr10	126596266	126596379
chr10	126604861	126604919
chr10	126605175	126606316
chr10	126606670	126607058
chr10	126609857	126610017
chr10	126723382	126723669
chr10	126725679	126725867
chr10	126726008	126726280
chr10	126726621	126726716
chr10	126728684	126728860
chr10	126731987	126732147
chr10	126732976	126733129
chr10	126734207	126734441
chr10	126734646	126734871
chr10	126737088	126737195
chr10	126739893	126740071
chr10	126748435	126748696
chr10	126752940	126753389
chr10	126753621	126755126
chr10	126772671	126772878
chr10	126774631	126774681
chr10	126775755	126776028

chr10	126776471	126776766
chr10	126813543	126813787
chr10	126838145	126838398
chr10	126847331	126847898
chr10	126848008	126849173
chr10	126849352	126851444
chr10	126865849	126866248
chr10	126906533	126906675
chr10	126906865	126907087
chr10	126919596	126921054
chr10	126921442	126921785
chr10	126923118	126923317
chr10	126951803	126952227
chr10	126952545	126953059
chr10	126953394	126953517
chr10	126953633	126953971
chr10	126954137	126954394
chr10	126955061	126955118
chr10	126958338	126958383
chr10	127407364	127408133
chr10	127408252	127408687
chr10	127511242	127512159
chr10	127512270	127512325
chr10	127567968	127568163
chr10	127575341	127575606
chr10	127575995	127576130
chr10	127580072	127580274
chr10	127580648	127581389
chr10	127581473	127581754
chr10	127582867	127583111
chr10	127583474	127584268
chr10	127582347	127582465
chr10	127584445	127585095
chr10	127585750	127585965
chr10	127589475	127589633
chr10	127590055	127590352
chr10	127661785	127661929
chr10	128570481	128570527
chr10	128591104	128591235
chr10	128593144	128594225
chr10	128594344	128595055
chr10	128599152	128599155
chr10	128610496	128610578
chr10	128610963	128611126
chr10	128612893	128613399
chr10	128613600	128613887

chr10	128614237	128614246
chr10	128811237	128811435
chr10	128839208	128839353
chr10	128839740	128839979
chr10	128946600	128946638
chr10	128946918	128946998
chr10	128947104	128947155
chr10	128994530	128994741
chr10	129042087	129042143
chr10	129537332	129537531
chr10	129705396	129705928
chr10	129721009	129721268
chr10	129721636	129721751
chr10	129735844	129736060
chr10	129745479	129745600
chr10	129845004	129845072
chr10	129845192	129845627
chr10	129845971	129846118
chr10	129846305	129847091
chr10	129861946	129862037
chr10	129862268	129863711
chr10	129914965	129915044
chr10	129923548	129923983
chr10	129924180	129924633
chr10	129924902	129925629
chr10	129928348	129928547
chr10	129945895	129946000
chr10	129948144	129948373
chr10	129948552	129948797
chr10	130008582	130008781
chr10	130262669	130262894
chr10	131264822	131264917
chr10	131264996	131265220
chr10	131265449	131266102
chr10	131323524	131323789
chr10	131343496	131343657
chr10	131770936	131771140
chr10	131908133	131909498
chr10	131909838	131910114
chr10	131933481	131933631
chr10	131934299	131935565
chr10	131936432	131936736
chr10	131936981	131937215
chr10	131988648	131988906
chr10	133758953	133759263
chr10	133760163	133760601

chr10	133761193	133761717
chr10	133794806	133795551
chr10	133795702	133796414
chr10	133811046	133811151
chr10	133814604	133814678
chr10	133879107	133879365
chr10	134122084	134122460
chr10	134133571	134133680
chr10	134134165	134134373
chr10	134135982	134136051
chr10	134136287	134136486
chr10	134136853	134137052
chr10	134139457	134139475
chr10	134143272	134143469
chr10	134143656	134143694
chr10	134144762	134145362
chr10	134145826	134146338
chr10	134209453	134210493
chr10	134210736	134211636
chr10	134220549	134222228
chr10	134226641	134226730
chr10	134226889	134226967
chr10	134228687	134228987
chr10	134231554	134232392
chr10	134234010	134234344
chr10	134234742	134235054
chr10	134235384	134235653
chr10	134237091	134237315
chr10	134237544	134237746
chr10	134238122	134238160
chr10	134257254	134257577
chr10	134259870	134262177
chr10	134266952	134267280
chr10	134350310	134350865
chr10	134351546	134352203
chr10	134479772	134479870
chr10	135070942	135071748
chr10	135071876	135072624
chr10	135073209	135073408
chr10	135074505	135075504
chr10	135079362	135079482
chr10	135088222	135088422
chr10	135088700	135089451
chr10	135089695	135090737
chr10	135091159	135093016
chr10	135106364	135106563

chr10	135107035	135107295
chr10	135121168	135121582
chr10	135121745	135123008
chr10	135161998	135162293
chr10	135170236	135170698
chr10	135170845	135171451
chr10	135186132	135187448
chr10	135191633	135192482
chr10	135192748	135193037
chr10	135194421	135194512
chr10	135207345	135208492
chr10	135461150	135461446
chr10	135481266	135481583
chr10	135491245	135491444
chr10	135499209	135499503
chr10	135499970	135501290
chr10	135502422	135502679
chr11	71497	71578
chr11	138785	139085
chr11	191437	191691
chr11	192668	193056
chr11	206605	209479
chr11	209592	210303
chr11	210440	211021
chr11	214991	215054
chr11	215786	215985
chr11	225761	226021
chr11	234927	235073
chr11	235513	236340
chr11	236453	237473
chr11	267979	268640
chr11	269874	270139
chr11	284055	284321
chr11	288098	289566
chr11	304733	305072
chr11	305308	305377
chr11	305707	305906
chr11	307495	307902
chr11	308107	309709
chr11	309889	311639
chr11	311812	312464
chr11	312590	312727
chr11	313092	315042
chr11	315249	321346
chr11	321449	322338
chr11	322435	322643

chr11	323061	323197
chr11	326118	326320
chr11	326490	326835
chr11	327017	327078
chr11	327452	327734
chr11	348316	348722
chr11	349368	349790
chr11	350413	351578
chr11	353393	353605
chr11	354215	354584
chr11	354918	354970
chr11	355143	356398
chr11	356761	356960
chr11	359546	359801
chr11	363856	364496
chr11	368873	369527
chr11	391652	396240
chr11	406779	407123
chr11	414641	414913
chr11	415933	417880
chr11	435408	435457
chr11	435748	435794
chr11	441473	442181
chr11	444024	444124
chr11	447338	447358
chr11	447696	449377
chr11	449571	451242
chr11	451378	451593
chr11	463637	463671
chr11	465408	465452
chr11	465833	465999
chr11	469475	469764
chr11	475005	475204
chr11	480908	481022
chr11	504025	504905
chr11	505092	505333
chr11	505604	506287
chr11	506403	508584
chr11	513901	514433
chr11	514627	514727
chr11	514881	515804
chr11	516942	517170
chr11	517780	519265
chr11	521650	521876
chr11	523268	523559
chr11	534194	535547

chr11	535703	537437
chr11	555217	555418
chr11	559754	560720
chr11	560920	562212
chr11	567873	570021
chr11	570589	570743
chr11	574663	575847
chr11	576170	578341
chr11	578648	579451
chr11	583641	584002
chr11	585249	585642
chr11	588215	588533
chr11	600805	600991
chr11	601129	601190
chr11	613745	616623
chr11	635272	635566
chr11	639122	639361
chr11	639654	640282
chr11	642727	642937
chr11	655076	655197
chr11	666741	666916
chr11	692554	692602
chr11	692694	692913
chr11	693103	693139
chr11	693269	698618
chr11	699404	699518
chr11	699715	699784
chr11	704819	707848
chr11	709312	709562
chr11	710566	710765
chr11	716976	717175
chr11	725370	725569
chr11	725948	726296
chr11	726431	727004
chr11	727641	728119
chr11	728309	728561
chr11	728800	728999
chr11	729199	729873
chr11	730216	730305
chr11	730440	730698
chr11	737784	737983
chr11	738161	738375
chr11	738944	739040
chr11	739466	739688
chr11	744163	744478
chr11	744707	745254

chr11	745717	746531
chr11	746817	748341
chr11	748434	748745
chr11	748940	749393
chr11	749976	750182
chr11	750330	750366
chr11	751612	751763
chr11	753012	753178
chr11	756474	756755
chr11	757289	757411
chr11	761077	761545
chr11	761739	763257
chr11	763428	764295
chr11	765496	765931
chr11	774479	774755
chr11	775191	775507
chr11	775754	778915
chr11	781332	781845
chr11	780854	781110
chr11	782037	782264
chr11	783073	783136
chr11	783387	783712
chr11	795026	796226
chr11	796426	797054
chr11	797197	798631
chr11	806103	806225
chr11	803784	805939
chr11	806710	807041
chr11	807328	807872
chr11	808095	810716
chr11	810915	811422
chr11	814239	814378
chr11	817821	820673
chr11	820992	821320
chr11	827074	827817
chr11	829237	830268
chr11	830637	831163
chr11	832288	835772
chr11	837551	837821
chr11	839133	839341
chr11	839618	839851
chr11	840769	846838
chr11	847044	847259
chr11	906278	906377
chr11	909634	910825
chr11	910937	912283

chr11	916226	916305
chr11	919457	919707
chr11	925293	926650
chr11	926921	927207
chr11	938965	939164
chr11	943548	943606
chr11	945635	945745
chr11	955706	956159
chr11	962522	962622
chr11	1050875	1051367
chr11	1060153	1060405
chr11	1073015	1074595
chr11	1146564	1146846
chr11	1283372	1284633
chr11	1285605	1285738
chr11	1320293	1320435
chr11	1324835	1325485
chr11	1327858	1328979
chr11	1330007	1331583
chr11	1331755	1331943
chr11	1356760	1357807
chr11	1404090	1404562
chr11	1410101	1410899
chr11	1568104	1568979
chr11	1569564	1569958
chr11	1579058	1579488
chr11	1579910	1581176
chr11	1581647	1581846
chr11	1592642	1593027
chr11	1593480	1594279
chr11	1598362	1598438
chr11	1616611	1616680
chr11	1671884	1672178
chr11	1676008	1676368
chr11	1676978	1677331
chr11	1691722	1691980
chr11	1714071	1714377
chr11	1714634	1715382
chr11	1715590	1715890
chr11	1726613	1726696
chr11	1770105	1770461
chr11	1783274	1783781
chr11	1783912	1784426
chr11	1784597	1785831
chr11	1790892	1791122
chr11	1791538	1791601

chr11	1791683	1791843
chr11	1792896	1794294
chr11	1794602	1794801
chr11	1817540	1817594
chr11	1841827	1842015
chr11	1847460	1848818
chr11	1849801	1850326
chr11	1850436	1851966
chr11	1852105	1853684
chr11	1853879	1854142
chr11	1854800	1856948
chr11	1858817	1859584
chr11	1864326	1864894
chr11	1865203	1865671
chr11	1865777	1866748
chr11	1879055	1879254
chr11	1882811	1882938
chr11	1883728	1886273
chr11	1891853	1892203
chr11	1892520	1893022
chr11	1894144	1895690
chr11	1895810	1899017
chr11	1899198	1900061
chr11	1900292	1900640
chr11	1901044	1901362
chr11	1901900	1902359
chr11	1903848	1904047
chr11	1940187	1940386
chr11	1944124	1944501
chr11	1945193	1945546
chr11	1945768	1946566
chr11	1947379	1949825
chr11	1952878	1953151
chr11	1961912	1961987
chr11	1962100	1962112
chr11	1964857	1965572
chr11	1965919	1966346
chr11	1967965	1968408
chr11	1968543	1970335
chr11	1970464	1970749
chr11	1971065	1971269
chr11	1985985	1986192
chr11	1989550	1989775
chr11	1990064	1990330
chr11	1999749	2000250
chr11	2007186	2007456

chr11	2008693	2009088
chr11	2010156	2010462
chr11	2011503	2011708
chr11	2016663	2016958
chr11	2017400	2017634
chr11	2017768	2018622
chr11	2019269	2020216
chr11	2020346	2020545
chr11	2021269	2021482
chr11	2022960	2023168
chr11	2113959	2114212
chr11	2120698	2120906
chr11	2159085	2159284
chr11	2173179	2173564
chr11	2187263	2188410
chr11	2196239	2197396
chr11	2198517	2200101
chr11	2202630	2203159
chr11	2200381	2202552
chr11	2204289	2204503
chr11	2208321	2208855
chr11	2208966	2209417
chr11	2214709	2215057
chr11	2216006	2216205
chr11	2217859	2218073
chr11	2221946	2222165
chr11	2222269	2222819
chr11	2223064	2223450
chr11	2224097	2224296
chr11	2224852	2225146
chr11	2228308	2228320
chr11	2229863	2230414
chr11	2277931	2279040
chr11	2286637	2286836
chr11	2290012	2292517
chr11	2325955	2326154
chr11	2327702	2328034
chr11	2398803	2400783
chr11	2420575	2421522
chr11	2421906	2423000
chr11	2467646	2467845
chr11	2550004	2550257
chr11	2553906	2554127
chr11	2554240	2556013
chr11	2575136	2575296
chr11	2585628	2585745

chr11	2589271	2589456
chr11	2589545	2589678
chr11	2590178	2590401
chr11	2595588	2595857
chr11	2596237	2596650
chr11	2720467	2720666
chr11	2720793	2721141
chr11	2721495	2722042
chr11	2763170	2763451
chr11	2790654	2791039
chr11	2791954	2792181
chr11	2794185	2794388
chr11	2799716	2800082
chr11	2800192	2800404
chr11	2824203	2824402
chr11	2846695	2847311
chr11	2847439	2848194
chr11	2858642	2858849
chr11	2883882	2884105
chr11	2898354	2898355
chr11	2898427	2898553
chr11	2905122	2906092
chr11	2906233	2907084
chr11	2907353	2907852
chr11	2909922	2910270
chr11	2912162	2914387
chr11	2914817	2915049
chr11	2917948	2918226
chr11	2919415	2919614
chr11	2919824	2921385
chr11	2922075	2922274
chr11	2922651	2924268
chr11	2924387	2924691
chr11	2932207	2933767
chr11	2934015	2934809
chr11	2939996	2940202
chr11	2948934	2952149
chr11	2952269	2952504
chr11	2954277	2954667
chr11	2954883	2955352
chr11	2955476	2955675
chr11	2960632	2961046
chr11	2961890	2962439
chr11	2962714	2962992
chr11	2972507	2972517
chr11	2984355	2984435

chr11	2984947	2985264
chr11	2997629	2997877
chr11	3001456	3001677
chr11	3002822	3003097
chr11	3003538	3003814
chr11	3011876	3012046
chr11	3012154	3013622
chr11	3013750	3014541
chr11	3021431	3022317
chr11	3024660	3025293
chr11	3025547	3025746
chr11	3026070	3026273
chr11	3026575	3026654
chr11	3026800	3026999
chr11	3027756	3027821
chr11	3037316	3037619
chr11	3043552	3044123
chr11	3044259	3044445
chr11	3044587	3044794
chr11	3070612	3071191
chr11	3071456	3071655
chr11	3074683	3074781
chr11	3077552	3079264
chr11	3079498	3079748
chr11	3080129	3080815
chr11	3087732	3088119
chr11	3088459	3088700
chr11	3115802	3116024
chr11	3116363	3116439
chr11	3116840	3117824
chr11	3120956	3121155
chr11	3122144	3122425
chr11	3131314	3131513
chr11	3144103	3144444
chr11	3144649	3145700
chr11	3145889	3146557
chr11	3146913	3147884
chr11	3148088	3148611
chr11	3151597	3151848
chr11	3185412	3186999
chr11	3189623	3189822
chr11	3399741	3400404
chr11	3400630	3400752
chr11	3437217	3437227
chr11	3443112	3443650
chr11	3443898	3445209

chr11	3494945	3494958
chr11	3497710	3498264
chr11	3602079	3602481
chr11	3637014	3637213
chr11	3664457	3664694
chr11	3695228	3695856
chr11	3710805	3710989
chr11	3718777	3718974
chr11	3723773	3723879
chr11	3744196	3744385
chr11	3744586	3744747
chr11	3766405	3766797
chr11	3768883	3769355
chr11	3769533	3769832
chr11	3773129	3773395
chr11	3797783	3797982
chr11	3808444	3808644
chr11	3809580	3809610
chr11	3812055	3812636
chr11	3813568	3813677
chr11	3816444	3816656
chr11	3818193	3818982
chr11	3819219	3819516
chr11	3819768	3820106
chr11	3820687	3820789
chr11	3825095	3825812
chr11	3828770	3828834
chr11	3828961	3830225
chr11	3854205	3854599
chr11	3860886	3861099
chr11	3861260	3863015
chr11	3863139	3863234
chr11	3875865	3876209
chr11	3876472	3877188
chr11	3877302	3877888
chr11	3887089	3887260
chr11	3905517	3905518
chr11	3925382	3925491
chr11	3926184	3926905
chr11	3927022	3927150
chr11	3927243	3927731
chr11	3928860	3929064
chr11	3937894	3938192
chr11	3938402	3938693
chr11	3940380	3940743
chr11	3940859	3941103

chr11	3941297	3941649
chr11	3945370	3945413
chr11	4066615	4066659
chr11	4066772	4066849
chr11	4066968	4066976
chr11	4079434	4079640
chr11	4080349	4080475
chr11	4093882	4094048
chr11	4115375	4117041
chr11	4117118	4117532
chr11	4125084	4125284
chr11	4146320	4146548
chr11	4146699	4146864
chr11	4208337	4208875
chr11	4414297	4414967
chr11	4475273	4475300
chr11	4485421	4485497
chr11	4564819	4565023
chr11	4628957	4629443
chr11	4629760	4629776
chr11	5063559	5063595
chr11	5307317	5307374
chr11	5617385	5617620
chr11	5618061	5618486
chr11	5621828	5621925
chr11	5641712	5642503
chr11	5645577	5646432
chr11	5646612	5646670
chr11	5646824	5646888
chr11	5647620	5647746
chr11	5705780	5706600
chr11	5710823	5711319
chr11	5829916	5830095
chr11	6240400	6240652
chr11	6240991	6241218
chr11	6255218	6255928
chr11	6256277	6257031
chr11	6319369	6319410
chr11	6343143	6343518
chr11	6340097	6342725
chr11	6409019	6409686
chr11	6410976	6411187
chr11	6411292	6411881
chr11	6412014	6412290
chr11	6439888	6440983
chr11	6494665	6495188

chr11	6495339	6496006
chr11	6502090	6502586
chr11	6502765	6503506
chr11	6524619	6524804
chr11	6525635	6525853
chr11	6526108	6526209
chr11	6544564	6544610
chr11	6552781	6552833
chr11	6624254	6626087
chr11	6627298	6627514
chr11	6628098	6628603
chr11	6632464	6634540
chr11	6639937	6640656
chr11	6640778	6641039
chr11	6703345	6703596
chr11	6703835	6704587
chr11	6704763	6705075
chr11	6712089	6712238
chr11	6715504	6715640
chr11	6846606	6846826
chr11	6947342	6947550
chr11	6947159	6947199
chr11	6947716	6948483
chr11	7025646	7025666
chr11	7041101	7041356
chr11	7534509	7535630
chr11	7572869	7572892
chr11	7593268	7593423
chr11	7613889	7613970
chr11	7621448	7621710
chr11	7621847	7622198
chr11	7694558	7695838
chr11	7879546	7880005
chr11	7892596	7893048
chr11	7894489	7894803
chr11	7895635	7895772
chr11	7895875	7895902
chr11	7914259	7914738
chr11	7926741	7927008
chr11	7927150	7927164
chr11	7927392	7928435
chr11	7931549	7931811
chr11	7998792	7999341
chr11	8008452	8008608
chr11	8008858	8009293
chr11	8040650	8040945

chr11	8041208	8041538
chr11	8041705	8041904
chr11	8160098	8160227
chr11	8160375	8160556
chr11	8250544	8250855
chr11	8255805	8256004
chr11	8256436	8256704
chr11	8407314	8407369
chr11	8555010	8555269
chr11	8588347	8588624
chr11	8615632	8615832
chr11	8625538	8626365
chr11	8672103	8672381
chr11	8692699	8693497
chr11	8703377	8706037
chr11	8706727	8707017
chr11	8707944	8708031
chr11	8708626	8708943
chr11	8709382	8710036
chr11	8710174	8710664
chr11	8710842	8710935
chr11	8733528	8733735
chr11	8738456	8738757
chr11	8738962	8739533
chr11	8739797	8741543
chr11	8741900	8743438
chr11	8756218	8756329
chr11	8765899	8766016
chr11	8788440	8788864
chr11	8789075	8789490
chr11	8789608	8790703
chr11	8790847	8790945
chr11	8791012	8791047
chr11	8793281	8793371
chr11	8794925	8795085
chr11	8795596	8796038
chr11	8796155	8796404
chr11	8827703	8827842
chr11	8828834	8830158
chr11	8830519	8832588
chr11	8834011	8834362
chr11	8832724	8833390
chr11	8835126	8835627
chr11	8835719	8836369
chr11	8837019	8837218
chr11	8848309	8848352

chr11	8850644	8850709
chr11	8853085	8853138
chr11	8853676	8853739
chr11	8853957	8854845
chr11	8861622	8861770
chr11	8883944	8884081
chr11	8889769	8889881
chr11	8891941	8892036
chr11	8892174	8892960
chr11	8927138	8927435
chr11	8928220	8928548
chr11	8931694	8933531
chr11	8934620	8934795
chr11	8939003	8939292
chr11	8945805	8946235
chr11	8956807	8957006
chr11	8981129	8981298
chr11	8984828	8986918
chr11	9024965	9026132
chr11	9060631	9060646
chr11	9113412	9113634
chr11	9159798	9159949
chr11	9160012	9160110
chr11	9166385	9166454
chr11	9180922	9181006
chr11	9181900	9182115
chr11	9187689	9187880
chr11	9201649	9201924
chr11	9232125	9232749
chr11	9236804	9237177
chr11	9241388	9241542
chr11	9251680	9251698
chr11	9258682	9258881
chr11	9269654	9269779
chr11	9284019	9284127
chr11	9284727	9285062
chr11	9285735	9286049
chr11	9286152	9287567
chr11	9295233	9295437
chr11	9295945	9296262
chr11	9298653	9298843
chr11	9313468	9313477
chr11	9322659	9322879
chr11	9329518	9329571
chr11	9334208	9334407
chr11	9335519	9336181

chr11	9336503	9336788
chr11	9348847	9349167
chr11	9352403	9352675
chr11	9353087	9353286
chr11	9360728	9360840
chr11	9371984	9372304
chr11	9373753	9374076
chr11	9374195	9374268
chr11	9375441	9375640
chr11	9377121	9377453
chr11	9378778	9378982
chr11	9379210	9379795
chr11	9383256	9383587
chr11	9384212	9384436
chr11	9384829	9386399
chr11	9391065	9391101
chr11	9391317	9391333
chr11	9397148	9397367
chr11	9397997	9398503
chr11	9405912	9407181
chr11	9426901	9427316
chr11	9470838	9471147
chr11	9474759	9474866
chr11	9481788	9482203
chr11	9482636	9483267
chr11	9483645	9484201
chr11	9484712	9485082
chr11	9493172	9493232
chr11	9496485	9497248
chr11	9513870	9514069
chr11	9525288	9525649
chr11	9527166	9527384
chr11	9528403	9528652
chr11	9553170	9553190
chr11	9556858	9557263
chr11	9559845	9559981
chr11	9586807	9587067
chr11	9587374	9587596
chr11	9587733	9588081
chr11	9589758	9590069
chr11	9590230	9590476
chr11	9590631	9591695
chr11	9594015	9595053
chr11	9595177	9597508
chr11	9600277	9600525
chr11	9602475	9602515

chr11	9602622	9602747
chr11	9617827	9618148
chr11	9622720	9623077
chr11	9630908	9631215
chr11	9631472	9632183
chr11	9633095	9633431
chr11	9634603	9636284
chr11	9636467	9636481
chr11	9663384	9663583
chr11	9684935	9686486
chr11	9694013	9694228
chr11	9736107	9736306
chr11	9738085	9738684
chr11	9738995	9739228
chr11	9740580	9740617
chr11	9748530	9748562
chr11	9774550	9775122
chr11	9775222	9775718
chr11	9775982	9776137
chr11	9776836	9776968
chr11	9777890	9778832
chr11	9782417	9782669
chr11	9779469	9782178
chr11	9783495	9783531
chr11	9785967	9786236
chr11	9792115	9792116
chr11	9804920	9805038
chr11	9821458	9821875
chr11	9823887	9824551
chr11	9852565	9852835
chr11	9860376	9860746
chr11	9862995	9863254
chr11	9872898	9873097
chr11	9916151	9916395
chr11	9921737	9922289
chr11	9927426	9927531
chr11	9927836	9928413
chr11	9965268	9965760
chr11	10004023	10005110
chr11	10144196	10144815
chr11	10161171	10161256
chr11	10161564	10161596
chr11	10204052	10204139
chr11	10229628	10229734
chr11	10229886	10230089
chr11	10255340	10255456

chr11	10306913	10307382
chr11	10314930	10316682
chr11	10319609	10319722
chr11	10323293	10328546
chr11	10328683	10330457
chr11	10330678	10330958
chr11	10336514	10336600
chr11	10343725	10344118
chr11	10344455	10344715
chr11	10372297	10372359
chr11	10380521	10380577
chr11	10387924	10388683
chr11	10427702	10427875
chr11	10428107	10428118
chr11	10428484	10429036
chr11	10436768	10436818
chr11	10471529	10472982
chr11	10473235	10473539
chr11	10474888	10474990
chr11	10475835	10475867
chr11	10476296	10476475
chr11	10476608	10478824
chr11	10479193	10479520
chr11	10507082	10507281
chr11	10509494	10511361
chr11	10511480	10512526
chr11	10513496	10513705
chr11	10520360	10520989
chr11	10526173	10526351
chr11	10527339	10527922
chr11	10531319	10531518
chr11	10531729	10531928
chr11	10539819	10540036
chr11	10541250	10541511
chr11	10548754	10549212
chr11	10561745	10562091
chr11	10562250	10562676
chr11	10562878	10563088
chr11	10608078	10608299
chr11	10610458	10610628
chr11	10613849	10614032
chr11	10614153	10614317
chr11	10615660	10616079
chr11	10616188	10616484
chr11	10625563	10626251
chr11	10654828	10654988

chr11	10669372	10669531
chr11	10680808	10681087
chr11	10756914	10757766
chr11	10757896	10758349
chr11	10772272	10773755
chr11	10809166	10809365
chr11	10817340	10817586
chr11	10824290	10824501
chr11	10829251	10831308
chr11	10864358	10864555
chr11	10864700	10865210
chr11	10878768	10880779
chr11	10901499	10901758
chr11	10919854	10920112
chr11	10920726	10922611
chr11	10925690	10925889
chr11	10928018	10928428
chr11	10951955	10952162
chr11	10952320	10954258
chr11	10955435	10955826
chr11	10956070	10956330
chr11	11334643	11335012
chr11	11335327	11335453
chr11	11413757	11413794
chr11	11438387	11438505
chr11	11443985	11444265
chr11	11542079	11542305
chr11	11606230	11606412
chr11	11642088	11642314
chr11	11642452	11643405
chr11	11643981	11644190
chr11	11644426	11644625
chr11	11807529	11807664
chr11	11834782	11834836
chr11	11835166	11835205
chr11	11862773	11864477
chr11	11864768	11865222
chr11	11866586	11866775
chr11	11866930	11866961
chr11	11870760	11870861
chr11	11870993	11871209
chr11	11938468	11938774
chr11	11952978	11953334
chr11	11966057	11966124
chr11	11966267	11966331
chr11	11997337	11997635

chr11	11998079	11999343
chr11	11999689	11999918
chr11	12000311	12000594
chr11	12001781	12002017
chr11	12009577	12009876
chr11	12015322	12015418
chr11	12019855	12020094
chr11	12020223	12020354
chr11	12027006	12027356
chr11	12028394	12031174
chr11	12063791	12064502
chr11	12065473	12066893
chr11	12068000	12068712
chr11	12068783	12068867
chr11	12069148	12069347
chr11	12071393	12071638
chr11	12087426	12088982
chr11	12089145	12089490
chr11	12090451	12090558
chr11	12097959	12098059
chr11	12098357	12098631
chr11	12101537	12102190
chr11	12111074	12111503
chr11	12111572	12112907
chr11	12106844	12110539
chr11	12116442	12116482
chr11	12120327	12120543
chr11	12124036	12124094
chr11	12131063	12131562
chr11	12131694	12132646
chr11	12132825	12133332
chr11	12134839	12135045
chr11	12139440	12139642
chr11	12144081	12144186
chr11	12144781	12144839
chr11	12146349	12146426
chr11	12146554	12146809
chr11	12146936	12147739
chr11	12147908	12148619
chr11	12149982	12150069
chr11	12175209	12175408
chr11	12180998	12181294
chr11	12181418	12181778
chr11	12181844	12181958
chr11	12184693	12184763
chr11	12185133	12185332

chr11	12185905	12186538
chr11	12186761	12186763
chr11	12188018	12188306
chr11	12188405	12189404
chr11	12192529	12192857
chr11	12194201	12194482
chr11	12196132	12196760
chr11	12197091	12198018
chr11	12200569	12200740
chr11	12200861	12201256
chr11	12202390	12202775
chr11	12203943	12205182
chr11	12205413	12205623
chr11	12206210	12206527
chr11	12206932	12207372
chr11	12208308	12208680
chr11	12208949	12209472
chr11	12211009	12211148
chr11	12211287	12211859
chr11	12212478	12213109
chr11	12221514	12222986
chr11	12236358	12236725
chr11	12245664	12246459
chr11	12253414	12253515
chr11	12254007	12254765
chr11	12257920	12258290
chr11	12267302	12267501
chr11	12269688	12269823
chr11	12289103	12289159
chr11	12294969	12295250
chr11	12303602	12303883
chr11	12306281	12306907
chr11	12307129	12307605
chr11	12308298	12310089
chr11	12311235	12311443
chr11	12398833	12400111
chr11	12410654	12410918
chr11	12411392	12411752
chr11	12412371	12412549
chr11	12414191	12414193
chr11	12414484	12414621
chr11	12431869	12431952
chr11	12433245	12433992
chr11	12434123	12434232
chr11	12452280	12452588
chr11	12500227	12500290

chr11	12500649	12500751
chr11	12561001	12561214
chr11	12567141	12567330
chr11	12691362	12691633
chr11	12695239	12695922
chr11	12696025	12697008
chr11	12697642	12698121
chr11	12698721	12698838
chr11	12698981	12699050
chr11	12705693	12705723
chr11	12709589	12709664
chr11	12714092	12714560
chr11	12714679	12716043
chr11	12718204	12718628
chr11	12724218	12724443
chr11	12725894	12726032
chr11	12728294	12728498
chr11	12742576	12742666
chr11	12742913	12743055
chr11	12743970	12744126
chr11	12745246	12746050
chr11	12746263	12746699
chr11	12747673	12748212
chr11	12768059	12768478
chr11	12768616	12768716
chr11	12772878	12773077
chr11	12778168	12778254
chr11	12782634	12782744
chr11	12789969	12790168
chr11	12790232	12790686
chr11	12791146	12792002
chr11	12792442	12793012
chr11	12799956	12800141
chr11	12809394	12809802
chr11	12820140	12820193
chr11	12820421	12820546
chr11	12821555	12821739
chr11	12821967	12822324
chr11	12823545	12823764
chr11	12824329	12825282
chr11	12827574	12827669
chr11	12827951	12828086
chr11	12831476	12831767
chr11	12836210	12836511
chr11	12840021	12840633
chr11	12849370	12850175

chr11	12859666	12860063
chr11	12860323	12861071
chr11	12862358	12863064
chr11	12863757	12863942
chr11	12870264	12870330
chr11	12870472	12870603
chr11	12873282	12873502
chr11	12877222	12877421
chr11	12909686	12909945
chr11	12910257	12910321
chr11	12910661	12911078
chr11	12911329	12911535
chr11	12911201	12911236
chr11	12911907	12912127
chr11	12912262	12912462
chr11	12914151	12914279
chr11	12915041	12915624
chr11	12921962	12922079
chr11	12922424	12922529
chr11	12985944	12986027
chr11	13029409	13029693
chr11	13029852	13030158
chr11	13030274	13032397
chr11	13032863	13033039
chr11	13094060	13094259
chr11	13094537	13095165
chr11	13095438	13095615
chr11	13098604	13098909
chr11	13112931	13113463
chr11	13113538	13113849
chr11	13119507	13119792
chr11	13120068	13120073
chr11	13141813	13142643
chr11	13159212	13159548
chr11	13181837	13182767
chr11	13190265	13190525
chr11	13191346	13191465
chr11	13204589	13204938
chr11	13228943	13230068
chr11	13237516	13237683
chr11	13277860	13277943
chr11	13281933	13282871
chr11	13283016	13283625
chr11	13284489	13284629
chr11	13298327	13298960
chr11	13299328	13300739

chr11	13354153	13354194
chr11	13354438	13354640
chr11	13355061	13355232
chr11	13356229	13356428
chr11	13356882	13357187
chr11	13358245	13358310
chr11	13359489	13360573
chr11	13360842	13361365
chr11	13458169	13458650
chr11	13461604	13462412
chr11	13483814	13484830
chr11	13484952	13485554
chr11	13487539	13487560
chr11	13487715	13487842
chr11	13685632	13685695
chr11	13688894	13689987
chr11	13690220	13691396
chr11	13692247	13692342
chr11	13742525	13742797
chr11	13950315	13950340
chr11	14211024	14211090
chr11	14298007	14298441
chr11	14309246	14309445
chr11	14309815	14309821
chr11	14310026	14310161
chr11	14321703	14322309
chr11	14322522	14322746
chr11	14328308	14328437
chr11	14330198	14330568
chr11	14332340	14332563
chr11	14334118	14334461
chr11	14335092	14335333
chr11	14339265	14340204
chr11	14340443	14340672
chr11	14351248	14351586
chr11	14358476	14358766
chr11	14358911	14359159
chr11	14373167	14373277
chr11	14376139	14376603
chr11	14378192	14378422
chr11	14379201	14381693
chr11	14382040	14383176
chr11	14402340	14402989
chr11	14403169	14403207
chr11	14416370	14416516
chr11	14420022	14420223

chr11	14427669	14427920
chr11	14431835	14432190
chr11	14432686	14433263
chr11	14437081	14437176
chr11	14443819	14443941
chr11	14444137	14444416
chr11	14444690	14444913
chr11	14445673	14445887
chr11	14468523	14468820
chr11	14469978	14470224
chr11	14477168	14477463
chr11	14520571	14521641
chr11	14521940	14522314
chr11	14541291	14542475
chr11	14665376	14665745
chr11	14665876	14666219
chr11	14666693	14666774
chr11	14694198	14694273
chr11	14912485	14913248
chr11	14913362	14913783
chr11	14914103	14914288
chr11	14916505	14916516
chr11	14926292	14927034
chr11	14929846	14930046
chr11	14930371	14930450
chr11	15671868	15672199
chr11	15987247	15987526
chr11	15989624	15989824
chr11	15990889	15991298
chr11	15991454	15991843
chr11	16023353	16023810
chr11	16117886	16117984
chr11	16119016	16119076
chr11	16119513	16119539
chr11	16175561	16175867
chr11	16176201	16176304
chr11	16176473	16176598
chr11	16327938	16328250
chr11	16594661	16594835
chr11	16625387	16625779
chr11	16626073	16626272
chr11	16626452	16627282
chr11	16628074	16629661
chr11	16634504	16634709
chr11	16635421	16635788
chr11	16759351	16759824

chr11	16760035	16760528
chr11	16809967	16810293
chr11	16812753	16813135
chr11	16813361	16813646
chr11	16896173	16896272
chr11	16897580	16897595
chr11	16923690	16923700
chr11	16923993	16924157
chr11	16940343	16940524
chr11	16945559	16945937
chr11	16946556	16946860
chr11	16946964	16947436
chr11	16957664	16957757
chr11	17035097	17035721
chr11	17036140	17036405
chr11	17037015	17037424
chr11	17086702	17086883
chr11	17098816	17099221
chr11	17099640	17100432
chr11	17103865	17104118
chr11	17104255	17104418
chr11	17119954	17119979
chr11	17134231	17134598
chr11	17206835	17206955
chr11	17228899	17231126
chr11	17244623	17244956
chr11	17251781	17251961
chr11	17277886	17278032
chr11	17278245	17279104
chr11	17285037	17285284
chr11	17289455	17289528
chr11	17297196	17299125
chr11	17335650	17335982
chr11	17373167	17374733
chr11	17374934	17375336
chr11	17375467	17375541
chr11	17375722	17375921
chr11	17376056	17376302
chr11	17411014	17411213
chr11	17808993	17809099
chr11	17820295	17820341
chr11	17820601	17820694
chr11	18033956	18035383
chr11	18127230	18127910
chr11	18128002	18128243
chr11	18153829	18153901

chr11	18154236	18154317
chr11	18165762	18165951
chr11	18166199	18166217
chr11	18190280	18190408
chr11	18265552	18265716
chr11	18266735	18266751
chr11	18267422	18267515
chr11	18269513	18269712
chr11	18269956	18270265
chr11	18268147	18268834
chr11	18270522	18270779
chr11	18277897	18278096
chr11	18282835	18282974
chr11	18286125	18286500
chr11	18286576	18287045
chr11	18287297	18287683
chr11	18287791	18287996
chr11	18288481	18289308
chr11	18289398	18290068
chr11	18297893	18298092
chr11	18299824	18299913
chr11	18329337	18329700
chr11	18329801	18330160
chr11	18334500	18335076
chr11	18335231	18336465
chr11	18343255	18344147
chr11	18344261	18344290
chr11	18344465	18344664
chr11	18350658	18351006
chr11	18361590	18361747
chr11	18388958	18389419
chr11	18399253	18399425
chr11	18404118	18404456
chr11	18404832	18405331
chr11	18405484	18406558
chr11	18408024	18408035
chr11	18409424	18409509
chr11	18410687	18410774
chr11	18410897	18410939
chr11	18412839	18413038
chr11	18413743	18414374
chr11	18414972	18415172
chr11	18415406	18417549
chr11	18417814	18418049
chr11	18428811	18429130
chr11	18429252	18429375

chr11	18430542	18430895
chr11	18433557	18433634
chr11	18433853	18434224
chr11	18452603	18452973
chr11	18517349	18517408
chr11	18544655	18544731
chr11	18547833	18549352
chr11	18550296	18550643
chr11	18552141	18552731
chr11	18592865	18592953
chr11	18593069	18593279
chr11	18609274	18610207
chr11	18610318	18611162
chr11	18612620	18612678
chr11	18615608	18616075
chr11	18616630	18617147
chr11	18617258	18617447
chr11	18644273	18644431
chr11	18651850	18652046
chr11	18655571	18655869
chr11	18656056	18656631
chr11	18678748	18678816
chr11	18690351	18690570
chr11	18690864	18690930
chr11	18698302	18699010
chr11	18699155	18699256
chr11	18720029	18721076
chr11	18727306	18728221
chr11	18728409	18728410
chr11	18742759	18742972
chr11	18743245	18743701
chr11	18771532	18771731
chr11	19092921	19093230
chr11	19098541	19098740
chr11	19098860	19098884
chr11	19098958	19099320
chr11	19101046	19101448
chr11	19101582	19101826
chr11	19102163	19102186
chr11	19102312	19102496
chr11	19109210	19109372
chr11	19109512	19109664
chr11	19111009	19111069
chr11	19111365	19111416
chr11	19115845	19115976
chr11	19124088	19124223

chr11	19138333	19139467
chr11	19238319	19238967
chr11	19254414	19254584
chr11	19261855	19262498
chr11	19262702	19263504
chr11	19263707	19264219
chr11	19265024	19265055
chr11	19265631	19265710
chr11	19265879	19266331
chr11	19266491	19266747
chr11	19281494	19281633
chr11	19297361	19297482
chr11	19298202	19298503
chr11	19298738	19299480
chr11	19392149	19392256
chr11	19396203	19396483
chr11	19402306	19402483
chr11	19459444	19459811
chr11	19463520	19463872
chr11	19585755	19585962
chr11	19686708	19686841
chr11	19689798	19690505
chr11	19690600	19690619
chr11	19690742	19690971
chr11	19694031	19694324
chr11	19694633	19694765
chr11	19695550	19695640
chr11	19696193	19696446
chr11	19701286	19701825
chr11	19702188	19702245
chr11	19733562	19733946
chr11	19734426	19734649
chr11	19849924	19850298
chr11	19889873	19889943
chr11	19973091	19973165
chr11	19973271	19973449
chr11	19975490	19975642
chr11	20000205	20001129
chr11	20016596	20016767
chr11	20016988	20017173
chr11	20024871	20025213
chr11	20027224	20027864
chr11	20032368	20033295
chr11	20035347	20035640
chr11	20037188	20037619
chr11	20044138	20044946

chr11	20048622	20049434
chr11	20052948	20053375
chr11	20054424	20054429
chr11	20057141	20057192
chr11	20057578	20057799
chr11	20068581	20068979
chr11	20070965	20071164
chr11	20071664	20071914
chr11	20072048	20072920
chr11	20073019	20073218
chr11	20075155	20075491
chr11	20076905	20078320
chr11	20079074	20079451
chr11	20133550	20133813
chr11	20133962	20134669
chr11	20134841	20134961
chr11	20150528	20150687
chr11	20153555	20153954
chr11	20382388	20382621
chr11	20385007	20386278
chr11	20389132	20389509
chr11	20390019	20390376
chr11	20408536	20410224
chr11	20419040	20419161
chr11	20473056	20473126
chr11	20473434	20473448
chr11	20495969	20496114
chr11	20496265	20496549
chr11	20498896	20499159
chr11	20499530	20499651
chr11	20505488	20505533
chr11	20505813	20505896
chr11	20520065	20520267
chr11	20588069	20588326
chr11	20588525	20588569
chr11	20631515	20631714
chr11	20632085	20632126
chr11	22480535	22481020
chr11	22481790	22482140
chr11	22478533	22478985
chr11	22488264	22488411
chr11	22488588	22488846
chr11	22498945	22499232
chr11	22531554	22531925
chr11	22532259	22532828
chr11	22540130	22540215

chr11	22542730	22542779
chr11	22646266	22647837
chr11	22677686	22678090
chr11	22688196	22688641
chr11	22724569	22724930
chr11	22732885	22732958
chr11	22809181	22809252
chr11	22850757	22850893
chr11	22851074	22851273
chr11	22941655	22941784
chr11	22942712	22942890
chr11	22997980	22998069
chr11	23258724	23258832
chr11	23259006	23259064
chr11	23340134	23340707
chr11	23561537	23561621
chr11	23745710	23745828
chr11	23747661	23747682
chr11	23749737	23749751
chr11	23878266	23878351
chr11	23919010	23919338
chr11	23924606	23924770
chr11	23924981	23925030
chr11	24317053	24317145
chr11	24412966	24413036
chr11	24422034	24422198
chr11	24769203	24769467
chr11	24970994	24971331
chr11	25087108	25087315
chr11	25087506	25087657
chr11	25089975	25090069
chr11	25090285	25090466
chr11	25445487	25445601
chr11	25756395	25756468
chr11	25767220	25767778
chr11	26003861	26003889
chr11	26066903	26066957
chr11	26156564	26156758
chr11	26209199	26209436
chr11	26209571	26209627
chr11	26210164	26210473
chr11	26248807	26248869
chr11	26249146	26249346
chr11	26256008	26256199
chr11	26306190	26306560
chr11	26308064	26308118

chr11	26308635	26308834
chr11	26600716	26600813
chr11	26601008	26601209
chr11	26604995	26605148
chr11	26605347	26605464
chr11	26642615	26643162
chr11	26671352	26671445
chr11	26842199	26842370
chr11	26842550	26842616
chr11	27013848	27013906
chr11	27015496	27015938
chr11	27061489	27061547
chr11	27062096	27062301
chr11	27106669	27107006
chr11	27109926	27109975
chr11	27110124	27110125
chr11	27146448	27146773
chr11	27180285	27180443
chr11	27180562	27180714
chr11	27195905	27196082
chr11	27206311	27206473
chr11	27231001	27231177
chr11	27231502	27231632
chr11	27234879	27234976
chr11	27249776	27249880
chr11	27339520	27339657
chr11	27372564	27372574
chr11	27384450	27384802
chr11	27385061	27385207
chr11	27406906	27406983
chr11	27444810	27445099
chr11	27445808	27445905
chr11	27449353	27449479
chr11	27450719	27451845
chr11	27469778	27470124
chr11	27477798	27477845
chr11	27477961	27478120
chr11	27485958	27486163
chr11	27486272	27486357
chr11	27488000	27488293
chr11	27489666	27491276
chr11	27492542	27493434
chr11	27493565	27495146
chr11	27528061	27528800
chr11	27545645	27546023
chr11	27657573	27657640

chr11	27720327	27720450
chr11	27720725	27722033
chr11	27722163	27722558
chr11	27956193	27956392
chr11	27956619	27956782
chr11	27998928	27998964
chr11	28108068	28108261
chr11	28128889	28130178
chr11	28131597	28132053
chr11	28162686	28162972
chr11	28167891	28168032
chr11	28312480	28312917
chr11	28364867	28365195
chr11	28557285	28557361
chr11	28557479	28557571
chr11	28641406	28641468
chr11	28641580	28642046
chr11	28679538	28679579
chr11	28694543	28694609
chr11	28694888	28694898
chr11	28695211	28695686
chr11	28723733	28724153
chr11	28760086	28760090
chr11	28760332	28760399
chr11	28780991	28781007
chr11	28781176	28781203
chr11	28830318	28830704
chr11	28836618	28837146
chr11	28841046	28841420
chr11	28841548	28841732
chr11	28844253	28844467
chr11	28855215	28855623
chr11	28856445	28856818
chr11	28857327	28858568
chr11	28882433	28882766
chr11	28885062	28885271
chr11	28899624	28899964
chr11	28940699	28941160
chr11	28984738	28985303
chr11	28988505	28988733
chr11	29003005	29003032
chr11	29040161	29040607
chr11	29282777	29283011
chr11	29302023	29302435
chr11	29303676	29303787
chr11	29304123	29304152

chr11	29315364	29315735
chr11	29331718	29331851
chr11	29332237	29332681
chr11	29334299	29334368
chr11	29334675	29334870
chr11	29350920	29351081
chr11	29548838	29549232
chr11	29549465	29549544
chr11	29795237	29795306
chr11	29830565	29830776
chr11	29831004	29831133
chr11	29936418	29936453
chr11	30129477	30129543
chr11	30219311	30219356
chr11	30219623	30219908
chr11	30344305	30344517
chr11	30344655	30345123
chr11	30606775	30607016
chr11	30607606	30607855
chr11	31276305	31276405
chr11	31346670	31346848
chr11	31391455	31391750
chr11	31530951	31531244
chr11	31531370	31531774
chr11	31649992	31650018
chr11	31667249	31667322
chr11	31667566	31667655
chr11	31667786	31668126
chr11	31677033	31677427
chr11	31677632	31677829
chr11	31677943	31677995
chr11	31819995	31820302
chr11	31826572	31826834
chr11	31827563	31827940
chr11	31831384	31831912
chr11	31832063	31832334
chr11	31832497	31833223
chr11	31833722	31833956
chr11	31834420	31834821
chr11	31835136	31835774
chr11	31836488	31836755
chr11	31837495	31837758
chr11	31890036	31890291
chr11	32112216	32113784
chr11	32140549	32140554
chr11	32158516	32158917

chr11	32159162	32159271
chr11	32319456	32319482
chr11	32324646	32324829
chr11	32325041	32325485
chr11	32334124	32334495
chr11	32344684	32344810
chr11	32354718	32354832
chr11	32355050	32355279
chr11	32456365	32456890
chr11	32571980	32572097
chr11	32602476	32602503
chr11	32602736	32602969
chr11	32603102	32603516
chr11	32604989	32605266
chr11	32605394	32606329
chr11	32611982	32612802
chr11	32665088	32665474
chr11	32728547	32729279
chr11	32806955	32807244
chr11	32813776	32813792
chr11	32814447	32814760
chr11	32830732	32830783
chr11	32843069	32843213
chr11	32843415	32843483
chr11	32845365	32845749
chr11	32850979	32851875
chr11	32852021	32852311
chr11	32856425	32856625
chr11	32859742	32860733
chr11	32898550	32898684
chr11	32902655	32902984
chr11	32913874	32916148
chr11	32923354	32923677
chr11	32985452	32985607
chr11	33003026	33003208
chr11	33021727	33021841
chr11	33032860	33033920
chr11	33035499	33035783
chr11	33037071	33037371
chr11	33037478	33038232
chr11	33060533	33062596
chr11	33063765	33063921
chr11	33065680	33065943
chr11	33066098	33066188
chr11	33067244	33067333
chr11	33067738	33067860

chr11	33070591	33070696
chr11	33081180	33081193
chr11	33081869	33082138
chr11	33082516	33083616
chr11	33083683	33084026
chr11	33086789	33087297
chr11	33092772	33092803
chr11	33138092	33138111
chr11	33152075	33152145
chr11	33165080	33165433
chr11	33167130	33167329
chr11	33167505	33167703
chr11	33168752	33168820
chr11	33170027	33170226
chr11	33172142	33172520
chr11	33181180	33181385
chr11	33181967	33182110
chr11	33182643	33183513
chr11	33186803	33186953
chr11	33194908	33194911
chr11	33262569	33263269
chr11	33277443	33277973
chr11	33278136	33280685
chr11	33284965	33285216
chr11	33288062	33288377
chr11	33298403	33298556
chr11	33332829	33332967
chr11	33366391	33366498
chr11	33393201	33393476
chr11	33394340	33394482
chr11	33397643	33398681
chr11	33578820	33579357
chr11	33654303	33654502
chr11	33654629	33655509
chr11	33655690	33656148
chr11	33656299	33656485
chr11	33656769	33656995
chr11	33657101	33657173
chr11	33657380	33658214
chr11	33664044	33664388
chr11	33679081	33679257
chr11	33709384	33709571
chr11	33711134	33711602
chr11	33711729	33713116
chr11	33713421	33714237
chr11	33714587	33715104

chr11	33720574	33720608
chr11	33721770	33722454
chr11	33722574	33723967
chr11	33724966	33725845
chr11	33728418	33728593
chr11	33739786	33739993
chr11	33740955	33741150
chr11	33743124	33743349
chr11	33744020	33744876
chr11	33744991	33745337
chr11	33745581	33746469
chr11	33755656	33756181
chr11	33756374	33756453
chr11	33756763	33758005
chr11	33758175	33759026
chr11	33794075	33794228
chr11	33795441	33796083
chr11	33796227	33796590
chr11	33827848	33827906
chr11	33850524	33851237
chr11	33857250	33857329
chr11	33890716	33891602
chr11	33891706	33892115
chr11	33917312	33917637
chr11	33934562	33934761
chr11	33951885	33952196
chr11	33952639	33952811
chr11	33958714	33959126
chr11	33962277	33962476
chr11	34019189	34019203
chr11	34019594	34019902
chr11	34051965	34051967
chr11	34052267	34052349
chr11	34052560	34052635
chr11	34055883	34056173
chr11	34066368	34067002
chr11	34072656	34073772
chr11	34073917	34074524
chr11	34074794	34075280
chr11	34088047	34088327
chr11	34089939	34089994
chr11	34094432	34094631
chr11	34126421	34127940
chr11	34156388	34156501
chr11	34195418	34196521
chr11	34197829	34197984

chr11	34202784	34202870
chr11	34211487	34211713
chr11	34230223	34230501
chr11	34230771	34230772
chr11	34247506	34247652
chr11	34255635	34256023
chr11	34259945	34260079
chr11	34260220	34260814
chr11	34261088	34261152
chr11	34264948	34265368
chr11	34265578	34265666
chr11	34270338	34270570
chr11	34272119	34272704
chr11	34272967	34273348
chr11	34273766	34273967
chr11	34285288	34285415
chr11	34287156	34287168
chr11	34288014	34288196
chr11	34293052	34293231
chr11	34293340	34293481
chr11	34295226	34295480
chr11	34295801	34295807
chr11	34295910	34296273
chr11	34296633	34296978
chr11	34297099	34297353
chr11	34300302	34300538
chr11	34329455	34329515
chr11	34330166	34330316
chr11	34365011	34365026
chr11	34366104	34367678
chr11	34368123	34368322
chr11	34377934	34379512
chr11	34379852	34381178
chr11	34392970	34393125
chr11	34393277	34393826
chr11	34416392	34416765
chr11	34417616	34417885
chr11	34418018	34418170
chr11	34418449	34418705
chr11	34426864	34427024
chr11	34449220	34449254
chr11	34459947	34460892
chr11	34461051	34461623
chr11	34522815	34523187
chr11	34523388	34523475
chr11	34523926	34524342

chr11	34534353	34534708
chr11	34585946	34586049
chr11	34586197	34586504
chr11	34607720	34607974
chr11	34621045	34621081
chr11	34621373	34621565
chr11	34625677	34626022
chr11	34627817	34628032
chr11	34642090	34642250
chr11	34642392	34643321
chr11	34643475	34643753
chr11	34646873	34646943
chr11	34648693	34648870
chr11	34649087	34649097
chr11	34655178	34656208
chr11	34657240	34657539
chr11	34658282	34658547
chr11	34658613	34658944
chr11	34661081	34661397
chr11	34661596	34661743
chr11	34662056	34662279
chr11	34662421	34662872
chr11	34666484	34666510
chr11	34666761	34666940
chr11	34675229	34675525
chr11	34675749	34675995
chr11	34676301	34676531
chr11	34676649	34676918
chr11	34689688	34689710
chr11	34703050	34703214
chr11	34704410	34704756
chr11	34706709	34706871
chr11	34707167	34707340
chr11	34771814	34772198
chr11	34831169	34831193
chr11	34831411	34831573
chr11	34937080	34939286
chr11	34998605	34998910
chr11	34999125	34999447
chr11	35030579	35031372
chr11	35031619	35031687
chr11	35031980	35032041
chr11	35033313	35033434
chr11	35035891	35036015
chr11	35051515	35051529
chr11	35051732	35051840

chr11	35052115	35052433
chr11	35052573	35052635
chr11	35053539	35053642
chr11	35057605	35057806
chr11	35061680	35062597
chr11	35078846	35079301
chr11	35078035	35078544
chr11	35099196	35099688
chr11	35103727	35103826
chr11	35104049	35104678
chr11	35109992	35110316
chr11	35132618	35133560
chr11	35134294	35134381
chr11	35136716	35137631
chr11	35162580	35163438
chr11	35159983	35162193
chr11	35163873	35164365
chr11	35164500	35165845
chr11	35166003	35166734
chr11	35166800	35167679
chr11	35167805	35168010
chr11	35179616	35180886
chr11	35184349	35185147
chr11	35186989	35187593
chr11	35187898	35188729
chr11	35189188	35189416
chr11	35194661	35195013
chr11	35195370	35195459
chr11	35195661	35195873
chr11	35199465	35199601
chr11	35199776	35200081
chr11	35200187	35200886
chr11	35212677	35212754
chr11	35212892	35212898
chr11	35238932	35239131
chr11	35247769	35247890
chr11	35257561	35257654
chr11	35259500	35259768
chr11	35297857	35297955
chr11	35298207	35298400
chr11	35327298	35327327
chr11	35358624	35358682
chr11	35385920	35386137
chr11	35413723	35413836
chr11	35440735	35441207
chr11	35536737	35536936

chr11	35537909	35537980
chr11	35547122	35547568
chr11	35547679	35547851
chr11	35547985	35548009
chr11	35571273	35571274
chr11	35589136	35589384
chr11	35589562	35590096
chr11	35601339	35601595
chr11	35606128	35606265
chr11	35620084	35620201
chr11	35637910	35637983
chr11	35638169	35638606
chr11	35639175	35642274
chr11	35642497	35642793
chr11	35643600	35644085
chr11	35645124	35645818
chr11	35651091	35651558
chr11	35651766	35652007
chr11	35661724	35661784
chr11	35685109	35685420
chr11	35683524	35684976
chr11	35705516	35705775
chr11	35747718	35747776
chr11	35770205	35770295
chr11	35772850	35773371
chr11	35773506	35773956
chr11	35818016	35818316
chr11	35818572	35818628
chr11	35819920	35820319
chr11	35820518	35820913
chr11	35824749	35825083
chr11	35843192	35843490
chr11	35843635	35844095
chr11	35915411	35915741
chr11	35916017	35916221
chr11	35929593	35929757
chr11	35929918	35930042
chr11	35949180	35949373
chr11	35949574	35949752
chr11	35965123	35966701
chr11	36029169	36029211
chr11	36030943	36031117
chr11	36031320	36031376
chr11	36031833	36032290
chr11	36033965	36034409
chr11	36034584	36034834

chr11	36040906	36041143
chr11	36058834	36059002
chr11	36059135	36059164
chr11	36128876	36128988
chr11	36163911	36164093
chr11	36166170	36166704
chr11	36167168	36167670
chr11	36167937	36168619
chr11	36169109	36169736
chr11	36170158	36170519
chr11	36170906	36171348
chr11	36171496	36171995
chr11	36177628	36177663
chr11	36177831	36178572
chr11	36232048	36232144
chr11	36233571	36233753
chr11	36276306	36276484
chr11	36276637	36276662
chr11	36279153	36279310
chr11	36310753	36311461
chr11	36318947	36319198
chr11	36331497	36331704
chr11	36331995	36332629
chr11	36334105	36334491
chr11	36355078	36355191
chr11	36371708	36371785
chr11	36376119	36376329
chr11	36376434	36376777
chr11	36383258	36384049
chr11	36384979	36385467
chr11	36386067	36386714
chr11	36393769	36394017
chr11	36397318	36399390
chr11	36399549	36399768
chr11	36407222	36407223
chr11	36411359	36411442
chr11	36411674	36411777
chr11	36415634	36415744
chr11	36418635	36418797
chr11	36419560	36419701
chr11	36442326	36442577
chr11	36468332	36468870
chr11	36469475	36469506
chr11	36469836	36470114
chr11	36473861	36474089
chr11	36475528	36475617

chr11	36476612	36476835
chr11	36477240	36477448
chr11	36488701	36489134
chr11	36493465	36493536
chr11	36500294	36500375
chr11	36500535	36500633
chr11	36514329	36514372
chr11	36522041	36522394
chr11	36530356	36530361
chr11	36530483	36530595
chr11	36530672	36531175
chr11	36531290	36532643
chr11	36532742	36532992
chr11	36616079	36616613
chr11	36796087	36796278
chr11	36796489	36796557
chr11	37525862	37526132
chr11	37526451	37527077
chr11	37528187	37528472
chr11	37532885	37532997
chr11	37533192	37533485
chr11	37809796	37809834
chr11	37809954	37810435
chr11	37903361	37903382
chr11	37927787	37927997
chr11	37997422	37997575
chr11	38111276	38111318
chr11	38836526	38836571
chr11	39012616	39012738
chr11	39033420	39033483
chr11	40351120	40351171
chr11	43333160	43333313
chr11	43334005	43334615
chr11	43349581	43349589
chr11	43373451	43373652
chr11	43380167	43381434
chr11	43398024	43398619
chr11	43425983	43426144
chr11	43426282	43426338
chr11	43529820	43530121
chr11	43568727	43570181
chr11	43611355	43611469
chr11	43645590	43645742
chr11	43665508	43666161
chr11	43666261	43666445
chr11	43701808	43702075

chr11	43702371	43703050
chr11	43703844	43704045
chr11	43708851	43709373
chr11	43760497	43760633
chr11	43811657	43811754
chr11	43811906	43812343
chr11	43839878	43840224
chr11	43841638	43841819
chr11	43846807	43847108
chr11	43852333	43852542
chr11	43852841	43852992
chr11	43873912	43873937
chr11	43890482	43890636
chr11	43902030	43902072
chr11	43902407	43902866
chr11	43926415	43926421
chr11	43926939	43927067
chr11	43942054	43942229
chr11	43942658	43943002
chr11	43963754	43964154
chr11	44087302	44088408
chr11	44117194	44118622
chr11	44118779	44118784
chr11	44119425	44119902
chr11	44134329	44134412
chr11	44212404	44212436
chr11	44337819	44338018
chr11	44466835	44467034
chr11	44578142	44578872
chr11	44581704	44581972
chr11	44582327	44582680
chr11	44582829	44583761
chr11	44583881	44584120
chr11	44584208	44584666
chr11	44585041	44587018
chr11	44587154	44587962
chr11	44588253	44588547
chr11	44589357	44589543
chr11	44590232	44590836
chr11	44591023	44591176
chr11	44591635	44592030
chr11	44593487	44593579
chr11	44594951	44595261
chr11	44595678	44595785
chr11	44596193	44597000
chr11	44598156	44598358

chr11	44609835	44610089
chr11	44610494	44610993
chr11	44613440	44613496
chr11	44613578	44613645
chr11	44614616	44614742
chr11	44613846	44614412
chr11	44634815	44635197
chr11	44655873	44656072
chr11	44659568	44659617
chr11	44659789	44659961
chr11	44749014	44749470
chr11	44832289	44832331
chr11	44883245	44883481
chr11	44883549	44883852
chr11	44884375	44884665
chr11	44971785	44972762
chr11	45124871	45124984
chr11	45129784	45129915
chr11	45167977	45168367
chr11	45168628	45169327
chr11	45169427	45169498
chr11	45179260	45179700
chr11	45179884	45180652
chr11	45200750	45203149
chr11	45227854	45228148
chr11	45392507	45392759
chr11	45686521	45687295
chr11	45753912	45754224
chr11	45754802	45755001
chr11	45757773	45758076
chr11	45758201	45759066
chr11	45759730	45760052
chr11	45760660	45760745
chr11	45761519	45761732
chr11	45792178	45792377
chr11	45825677	45828029
chr11	45868400	45869362
chr11	45869778	45869889
chr11	45921327	45922248
chr11	45938180	45938201
chr11	45938310	45938694
chr11	45938794	45940943
chr11	45941291	45941490
chr11	45945631	45945958
chr11	45943566	45945377
chr11	45946778	45946849

chr11	45947263	45947421
chr11	45949494	45952663
chr11	45952921	45953640
chr11	45953732	45953994
chr11	45986383	45986521
chr11	46004498	46004694
chr11	46071599	46071815
chr11	46087311	46087618
chr11	46087847	46088372
chr11	46096787	46096857
chr11	46097074	46097209
chr11	46134563	46134628
chr11	46140893	46141409
chr11	46141677	46141800
chr11	46141911	46142882
chr11	46143405	46144072
chr11	46144781	46144894
chr11	46148631	46149169
chr11	46165493	46165502
chr11	46165630	46165692
chr11	46181121	46181204
chr11	46181572	46181862
chr11	46231971	46232143
chr11	46237832	46237968
chr11	46257892	46258262
chr11	46258562	46261583
chr11	46262396	46262589
chr11	46264526	46265524
chr11	46267006	46267178
chr11	46269096	46269525
chr11	46270148	46270190
chr11	46270409	46270848
chr11	46285402	46285507
chr11	46316638	46316860
chr11	46317249	46317742
chr11	46326380	46326676
chr11	46327469	46327528
chr11	46335200	46335432
chr11	46337020	46337220
chr11	46342840	46343032
chr11	46345196	46345612
chr11	46349671	46349968
chr11	46354509	46354905
chr11	46357872	46358495
chr11	46358674	46359389
chr11	46360107	46360151

chr11	46360316	46360582
chr11	46363321	46363565
chr11	46364211	46364376
chr11	46365137	46365621
chr11	46366334	46368087
chr11	46369094	46369788
chr11	46369994	46370193
chr11	46375704	46375981
chr11	46377354	46377553
chr11	46378730	46379329
chr11	46383724	46383971
chr11	46384237	46384531
chr11	46385033	46385260
chr11	46390396	46390523
chr11	46391270	46391469
chr11	46401891	46404269
chr11	46412703	46413107
chr11	46413370	46413603
chr11	46432381	46432696
chr11	46433110	46433124
chr11	46475952	46475972
chr11	46479200	46479389
chr11	46492092	46492232
chr11	46492485	46493189
chr11	46524224	46524370
chr11	46535074	46535330
chr11	46535887	46536024
chr11	46542455	46542485
chr11	46568417	46568664
chr11	46575680	46576102
chr11	46579172	46579577
chr11	46582343	46583275
chr11	46586007	46586075
chr11	46586339	46586485
chr11	46613971	46614393
chr11	46615099	46615571
chr11	46615738	46615893
chr11	46619063	46619555
chr11	46631619	46631650
chr11	46636671	46637134
chr11	46638036	46639699
chr11	46641401	46641643
chr11	46669046	46669144
chr11	46670798	46670954
chr11	46671168	46671255
chr11	46677187	46677193c

chr11	46677301	46677573
chr11	46679046	46679768
chr11	46679834	46680186
chr11	46692842	46692980
chr11	46693168	46693182
chr11	46696017	46696356
chr11	46696486	46697604
chr11	46697979	46698267
chr11	46697786	46697818
chr11	46702885	46703327
chr11	46703967	46704198
chr11	46704452	46705792
chr11	46707742	46707941
chr11	46708187	46710295
chr11	46710807	46711315
chr11	46714619	46715301
chr11	46719883	46720095
chr11	46721708	46723448
chr11	46735945	46736357
chr11	46736541	46736890
chr11	46738234	46738460
chr11	46738704	46738758
chr11	46739253	46739673
chr11	46743165	46743541
chr11	46757538	46758125
chr11	46760230	46760286
chr11	46830385	46830495
chr11	46847755	46847822
chr11	46848960	46849261
chr11	46849559	46849570
chr11	46867128	46868456
chr11	46869896	46870195
chr11	46922042	46922522
chr11	46934020	46934064
chr11	46937673	46938464
chr11	46938685	46938884
chr11	46938993	46940132
chr11	46940383	46940594
chr11	46941767	46941842
chr11	46951630	46952077
chr11	46957989	46958103
chr11	46958252	46958689
chr11	46999734	46999920
chr11	47165151	47165370
chr11	47197299	47198512
chr11	47198653	47199304

chr11	47206436	47208984
chr11	47209939	47210020
chr11	47210245	47210416
chr11	47210491	47210927
chr11	47211195	47211563
chr11	47213226	47213652
chr11	47215308	47215771
chr11	47215944	47216260
chr11	47222162	47222540
chr11	47222752	47222777
chr11	47225429	47225570
chr11	47226444	47226669
chr11	47231438	47231687
chr11	47235466	47237448
chr11	47237708	47238637
chr11	47239390	47239598
chr11	47257247	47257254
chr11	47258195	47258394
chr11	47262611	47263005
chr11	47263687	47263886
chr11	47264969	47265306
chr11	47267796	47268306
chr11	47269865	47271261
chr11	47278610	47278934
chr11	47279215	47279414
chr11	47279522	47279909
chr11	47286768	47286884
chr11	47287094	47287160
chr11	47290020	47292514
chr11	47340717	47340943
chr11	47351860	47352059
chr11	47376252	47377669
chr11	47381347	47381626
chr11	47389686	47389760
chr11	47393760	47393856
chr11	47393964	47394534
chr11	47394932	47395244
chr11	47395431	47395630
chr11	47396291	47396561
chr11	47398084	47398327
chr11	47399358	47399740
chr11	47408565	47408763
chr11	47410004	47410203
chr11	47410894	47411159
chr11	47413166	47413438
chr11	47415794	47416457

chr11	47416607	47417163
chr11	47420400	47420548
chr11	47420683	47421217
chr11	47421425	47422243
chr11	47422503	47422702
chr11	47425799	47426089
chr11	47427220	47427389
chr11	47428308	47429956
chr11	47430141	47431671
chr11	47434653	47434786
chr11	47436017	47436216
chr11	47437179	47437774
chr11	47438039	47439039
chr11	47444872	47445151
chr11	47446656	47448902
chr11	47466517	47466732
chr11	47472442	47472708
chr11	47473642	47473955
chr11	47476106	47476408
chr11	47477916	47478193
chr11	47481836	47482035
chr11	47484232	47484543
chr11	47485364	47485491
chr11	47506643	47507211
chr11	47516850	47517357
chr11	47530330	47530569
chr11	47531735	47532160
chr11	47532704	47532921
chr11	47534903	47535345
chr11	47536449	47537302
chr11	47542501	47542772
chr11	47544968	47545611
chr11	47545841	47545847
chr11	47545967	47546499
chr11	47547353	47547547
chr11	47548216	47549015
chr11	47549473	47549589
chr11	47550357	47550867
chr11	47551848	47551989
chr11	47561359	47562712
chr11	47562969	47563168
chr11	47567229	47567556
chr11	47569397	47569916
chr11	47572335	47572565
chr11	47572764	47572969
chr11	47573755	47575232

chr11	47579251	47579444
chr11	47581473	47581872
chr11	47581959	47582755
chr11	47583668	47583943
chr11	47584538	47585180
chr11	47585921	47586334
chr11	47586605	47588165
chr11	47592663	47592946
chr11	47593467	47593470
chr11	47599924	47601070
chr11	47604181	47604216
chr11	47604513	47604778
chr11	47606228	47606427
chr11	47607214	47607437
chr11	47608396	47608595
chr11	47610128	47610544
chr11	47612820	47613310
chr11	47610660	47612593
chr11	47615573	47615939
chr11	47643047	47643276
chr11	47654339	47654495
chr11	47662106	47662305
chr11	47662563	47662902
chr11	47663431	47664514
chr11	47677905	47678104
chr11	47684708	47684885
chr11	47685043	47685263
chr11	47685666	47686065
chr11	47686343	47686592
chr11	47686696	47686993
chr11	47687544	47687743
chr11	47689457	47689725
chr11	47693764	47694497
chr11	47694904	47695113
chr11	47695232	47695235
chr11	47708056	47708400
chr11	47725098	47725285
chr11	47736264	47737222
chr11	47745618	47746010
chr11	47748870	47749083
chr11	47750406	47750526
chr11	47784264	47784440
chr11	47787275	47788939
chr11	47789139	47790620
chr11	47790710	47790924
chr11	47793544	47793570

chr11	47808283	47808613
chr11	47826947	47826965
chr11	47853119	47853321
chr11	47867397	47867642
chr11	47869293	47870192
chr11	47870434	47870641
chr11	47875382	47875451
chr11	47875556	47875695
chr11	47934301	47934545
chr11	47944830	47945079
chr11	47945759	47945817
chr11	47960774	47960942
chr11	47992852	47992999
chr11	48001874	48003285
chr11	48008294	48008403
chr11	48012337	48012518
chr11	48071446	48071543
chr11	48075706	48075754
chr11	48082462	48082661
chr11	48131698	48132006
chr11	48804976	48805339
chr11	48817820	48818092
chr11	48854656	48855053
chr11	48856879	48857078
chr11	48859307	48859607
chr11	48861763	48862040
chr11	48863695	48863912
chr11	48883267	48883599
chr11	48894981	48895170
chr11	48898891	48899254
chr11	48932365	48932709
chr11	48946400	48946766
chr11	48948353	48948552
chr11	48953270	48953629
chr11	48955293	48955541
chr11	48962831	48963200
chr11	50745636	50745765
chr11	50758229	50758408
chr11	50758895	50759118
chr11	50763988	50764286
chr11	50783311	50783510
chr11	51568249	51568288
chr11	51568775	51569154
chr11	51569516	51570354
chr11	51571962	51572338
chr11	51572536	51572914

chr11	51572983	51573135
chr11	51573259	51573627
chr11	51573815	51573948
chr11	51574226	51574425
chr11	51574530	51574893
chr11	51575526	51575880
chr11	51576586	51577120
chr11	51578597	51578973
chr11	51579187	51579822
chr11	51579979	51581231
chr11	51581831	51583969
chr11	51584035	51585182
chr11	51585630	51586046
chr11	51586112	51587474
chr11	51588167	51589526
chr11	51590219	51591517
chr11	51591652	51592114
chr11	51592349	51593481
chr11	55014323	55014775
chr11	55015060	55015259
chr11	55024840	55025188
chr11	55026183	55026449
chr11	56608987	56609037
chr11	56674011	56674679
chr11	56677481	56677602
chr11	56678239	56678596
chr11	56678800	56678849
chr11	56700207	56700364
chr11	56700583	56700813
chr11	56837462	56837540
chr11	56839976	56840609
chr11	56894174	56894339
chr11	57011492	57011583
chr11	57016920	57017260
chr11	57019051	57019096
chr11	57042558	57042898
chr11	57044818	57046402
chr11	57046578	57048152
chr11	57050434	57050786
chr11	57055337	57055593
chr11	57056328	57056527
chr11	57057802	57057938
chr11	57059526	57059813
chr11	57060175	57061699
chr11	57061956	57062510
chr11	57063051	57063418

chr11	57063866	57064133
chr11	57065376	57065946
chr11	57066309	57066491
chr11	57068979	57069178
chr11	57071246	57073694
chr11	57074629	57075081
chr11	57075304	57076678
chr11	57077040	57079905
chr11	57084743	57084942
chr11	57085704	57086097
chr11	57086206	57086729
chr11	57086852	57087237
chr11	57088172	57088354
chr11	57089044	57094067
chr11	57094991	57095394
chr11	57101518	57101924
chr11	57102479	57104082
chr11	57104309	57104613
chr11	57112848	57113479
chr11	57116895	57117676
chr11	57135694	57136210
chr11	57147486	57147549
chr11	57169492	57169611
chr11	57182007	57182025
chr11	57185557	57185756
chr11	57186226	57186339
chr11	57187554	57187655
chr11	57191162	57191512
chr11	57191899	57195243
chr11	57202049	57202224
chr11	57203550	57203722
chr11	57211938	57212113
chr11	57214305	57214504
chr11	57226080	57226462
chr11	57226713	57227028
chr11	57227342	57227679
chr11	57227989	57228734
chr11	57243356	57244636
chr11	57245964	57246219
chr11	57249739	57250765
chr11	57250900	57251345
chr11	57260103	57260440
chr11	57267310	57267685
chr11	57270127	57270164
chr11	57281887	57282744
chr11	57282896	57283124

chr11	57283346	57283806
chr11	57286064	57286125
chr11	57294480	57294656
chr11	57297684	57297717
chr11	57297839	57298188
chr11	57298465	57299049
chr11	57299748	57299976
chr11	57301357	57301424
chr11	57323028	57323165
chr11	57323748	57323893
chr11	57326924	57327020
chr11	57327950	57328098
chr11	57328166	57328374
chr11	57333608	57334087
chr11	57334162	57334361
chr11	57334469	57336209
chr11	57336593	57336869
chr11	57337131	57337503
chr11	57357048	57357217
chr11	57365115	57365565
chr11	57370729	57371031
chr11	57371454	57371500
chr11	57404752	57407680
chr11	57410532	57410740
chr11	57411841	57412106
chr11	57412170	57412237
chr11	57412543	57412849
chr11	57412930	57413425
chr11	57413997	57414447
chr11	57417021	57417376
chr11	57417487	57417733
chr11	57424534	57425099
chr11	57425209	57425782
chr11	57430588	57430834
chr11	57432492	57432675
chr11	57434064	57436359
chr11	57444936	57445135
chr11	57446814	57447002
chr11	57447225	57447234
chr11	57447592	57448006
chr11	57448283	57448391
chr11	57453321	57453542
chr11	57465205	57465375
chr11	57478868	57479816
chr11	57480032	57480475
chr11	57483711	57483983

chr11	57489616	57489853
chr11	57492382	57492522
chr11	57492759	57493165
chr11	57493628	57493939
chr11	57494709	57494993
chr11	57498654	57498866
chr11	57500330	57500532
chr11	57500732	57501195
chr11	57508349	57508755
chr11	57507890	57508085
chr11	57508961	57509906
chr11	57510149	57510352
chr11	57512195	57512493
chr11	57517704	57517923
chr11	57518877	57519130
chr11	57524289	57524582
chr11	57528889	57529508
chr11	57529952	57530278
chr11	57531065	57531489
chr11	57532144	57532657
chr11	57535354	57535629
chr11	57537531	57537765
chr11	57538361	57538473
chr11	57539335	57539702
chr11	57539943	57540417
chr11	57540701	57541064
chr11	57542234	57542451
chr11	57543519	57544356
chr11	57544784	57545232
chr11	57545474	57546693
chr11	57546767	57547270
chr11	57548469	57548693
chr11	57548792	57549536
chr11	57551590	57551946
chr11	57553790	57554137
chr11	57554667	57555979
chr11	57556176	57556313
chr11	57558528	57559286
chr11	57559512	57559920
chr11	57560485	57560699
chr11	57562532	57562533
chr11	57565606	57565805
chr11	57566333	57566428
chr11	57566736	57567175
chr11	57567873	57568277
chr11	57568505	57568521

chr11	57636658	57636865
chr11	57637017	57637214
chr11	57640558	57641411
chr11	57642424	57642766
chr11	57644437	57644578
chr11	57644811	57644830
chr11	57657139	57657332
chr11	57667762	57667789
chr11	57668885	57669294
chr11	57669508	57670157
chr11	57673034	57673236
chr11	57670900	57672267
chr11	57713906	57714144
chr11	57826472	57826801
chr11	57827244	57827644
chr11	58102189	58102350
chr11	58102469	58102556
chr11	58161698	58161729
chr11	58161973	58162049
chr11	58233273	58233326
chr11	58282122	58282321
chr11	58291833	58292366
chr11	58335376	58335465
chr11	58335786	58336186
chr11	58337382	58337713
chr11	58340914	58341690
chr11	58345009	58347625
chr11	58348867	58349112
chr11	58343027	58343450
chr11	58393653	58393890
chr11	58395081	58395863
chr11	58421693	58421892
chr11	58426641	58426655
chr11	58538393	58538846
chr11	58691231	58691370
chr11	58731318	58731537
chr11	58825477	58825827
chr11	58869574	58869777
chr11	58873908	58874573
chr11	58874736	58875506
chr11	58909482	58910570
chr11	58910732	58910991
chr11	58911992	58912750
chr11	58912832	58913347
chr11	58937122	58937220
chr11	58939156	58942252

chr11	58942495	58942647
chr11	58942740	58942818
chr11	58947208	58947414
chr11	58952645	58952844
chr11	58953565	58953769
chr11	58969281	58969289
chr11	58984671	58984741
chr11	58989167	58989406
chr11	59031857	59032595
chr11	59033386	59033982
chr11	59034181	59035423
chr11	59036643	59036709
chr11	59036084	59036347
chr11	59292232	59292431
chr11	59304259	59304513
chr11	59304706	59305518
chr11	59312454	59313554
chr11	59316521	59318960
chr11	59319033	59320150
chr11	59321394	59321465
chr11	59322576	59324184
chr11	59324296	59324951
chr11	59325074	59325333
chr11	59327648	59328403
chr11	59328527	59329180
chr11	59329589	59330283
chr11	59331085	59331354
chr11	59332196	59332469
chr11	59333129	59334063
chr11	59338422	59338759
chr11	59340424	59341275
chr11	59348233	59348473
chr11	59371943	59372237
chr11	59382184	59382397
chr11	59382518	59383333
chr11	59383627	59384414
chr11	59386583	59386811
chr11	59425670	59425949
chr11	59435581	59436691
chr11	59436841	59437881
chr11	59448353	59448419
chr11	59466685	59467035
chr11	59467195	59467479
chr11	59472823	59473175
chr11	59474270	59474778
chr11	59475988	59476212

chr11	59521195	59521578
chr11	59521706	59523276
chr11	59523631	59524283
chr11	59524816	59524872
chr11	59532970	59533169
chr11	59544337	59544382
chr11	59544528	59544536
chr11	59544736	59544941
chr11	59553008	59553057
chr11	59554303	59554409
chr11	59554693	59554974
chr11	59569199	59569452
chr11	59577543	59578567
chr11	59590784	59590808
chr11	59668760	59668821
chr11	59937071	59937512
chr11	60138525	60138724
chr11	60466108	60466191
chr11	60608719	60609317
chr11	60609571	60610444
chr11	60610609	60611245
chr11	60611408	60611903
chr11	60612011	60612327
chr11	60666782	60667122
chr11	60671031	60671251
chr11	60672517	60672852
chr11	60672952	60674123
chr11	60674272	60674834
chr11	60680932	60683544
chr11	60683974	60684162
chr11	60684789	60685164
chr11	60686162	60686180
chr11	60691456	60692505
chr11	60692726	60692947
chr11	60693126	60693569
chr11	60709979	60710227
chr11	60710365	60710450
chr11	60710516	60710855
chr11	60711388	60711389
chr11	60711520	60711792
chr11	60717701	60720471
chr11	60753514	60753691
chr11	60774906	60775527
chr11	60828765	60828884
chr11	60843638	60843802
chr11	60859650	60859955

chr11	60881157	60881500
chr11	60885020	60885219
chr11	60897449	60897728
chr11	60908677	60908897
chr11	60917516	60917736
chr11	60920701	60920904
chr11	60921102	60921400
chr11	60926215	60926443
chr11	60927243	60927452
chr11	60927773	60928079
chr11	60928246	60929461
chr11	60929671	60930100
chr11	61027484	61027593
chr11	61028358	61028676
chr11	61029384	61029482
chr11	61038370	61038582
chr11	61040333	61040550
chr11	61047207	61047292
chr11	61048785	61048996
chr11	61049327	61049526
chr11	61049665	61049948
chr11	61059500	61059682
chr11	61060763	61062711
chr11	61064243	61064561
chr11	61064805	61065287
chr11	61065661	61065819
chr11	61072125	61072324
chr11	61086448	61086594
chr11	61093510	61093647
chr11	61093755	61093914
chr11	61094591	61094601
chr11	61095055	61095317
chr11	61098197	61098400
chr11	61099675	61099866
chr11	61100069	61101148
chr11	61101372	61102190
chr11	61103416	61103801
chr11	61104295	61104533
chr11	61104603	61104919
chr11	61114753	61115279
chr11	61115921	61116120
chr11	61120822	61121036
chr11	61122558	61122838
chr11	61125564	61125685
chr11	61127163	61127848
chr11	61131469	61131709

chr11	61131927	61132304
chr11	61133328	61133877
chr11	61128634	61131172
chr11	61135323	61135627
chr11	61135765	61136440
chr11	61136664	61137794
chr11	61139387	61139515
chr11	61143394	61143685
chr11	61145379	61147068
chr11	61144932	61145316
chr11	61149128	61149562
chr11	61150248	61151014
chr11	61151738	61151828
chr11	61152710	61152712
chr11	61153026	61153269
chr11	61153418	61153459
chr11	61154525	61154625
chr11	61158004	61160587
chr11	61160814	61161112
chr11	61161779	61162102
chr11	61162509	61162815
chr11	61167196	61167534
chr11	61168269	61168468
chr11	61177564	61177806
chr11	61180854	61181046
chr11	61184697	61184791
chr11	61185092	61185399
chr11	61187800	61187912
chr11	61190252	61190290
chr11	61196655	61198043
chr11	61198559	61198789
chr11	61200690	61200782
chr11	61203189	61203406
chr11	61206316	61206542
chr11	61207631	61207763
chr11	61210304	61210452
chr11	61211168	61211378
chr11	61213942	61214120
chr11	61217139	61217234
chr11	61223710	61223878
chr11	61229560	61229726
chr11	61235183	61235484
chr11	61239204	61239570
chr11	61244485	61244684
chr11	61273411	61273610
chr11	61276877	61277321

chr11	61297098	61297404
chr11	61299867	61300066
chr11	61347807	61348308
chr11	61348741	61348956
chr11	61355061	61355764
chr11	61371194	61371454
chr11	61372037	61372236
chr11	61375276	61375470
chr11	61375590	61375849
chr11	61384346	61384549
chr11	61385518	61385589
chr11	61390588	61391124
chr11	61391710	61392129
chr11	61392665	61392822
chr11	61393190	61393501
chr11	61394006	61395317
chr11	61438659	61438893
chr11	61439033	61439101
chr11	61447784	61447999
chr11	61456048	61456247
chr11	61457139	61457288
chr11	61463012	61463458
chr11	61463749	61464612
chr11	61464822	61465387
chr11	61467244	61467578
chr11	61468375	61468644
chr11	61469314	61469540
chr11	61469824	61469879
chr11	61485811	61486416
chr11	61497316	61497635
chr11	61514926	61515185
chr11	61520108	61520307
chr11	61526886	61527114
chr11	61540411	61540700
chr11	61545293	61545580
chr11	61559367	61561050
chr11	61561374	61561508
chr11	61561625	61561734
chr11	61564839	61565099
chr11	61566739	61566993
chr11	61578917	61579537
chr11	61581471	61581901
chr11	61582080	61585661
chr11	61587395	61588756
chr11	61590988	61591262
chr11	61592769	61593170

chr11	61594810	61597576
chr11	61598274	61598381
chr11	61601897	61602130
chr11	61602749	61603201
chr11	61603340	61603387
chr11	61603467	61604167
chr11	61608637	61608847
chr11	61612987	61613186
chr11	61614354	61614372
chr11	61616398	61616622
chr11	61634291	61634580
chr11	61642675	61642874
chr11	61648031	61648782
chr11	61649171	61650605
chr11	61652733	61653087
chr11	61654609	61655612
chr11	61655858	61660377
chr11	61660748	61660947
chr11	61661353	61661969
chr11	61662332	61663042
chr11	61663675	61664055
chr11	61664293	61664639
chr11	61665151	61665711
chr11	61669513	61669743
chr11	61684507	61685505
chr11	61712261	61712460
chr11	61715456	61715564
chr11	61716019	61716129
chr11	61717088	61717156
chr11	61719688	61719887
chr11	61722065	61723770
chr11	61725095	61725295
chr11	61731246	61733774
chr11	61737491	61737942
chr11	61738138	61739438
chr11	61734047	61737249
chr11	61739549	61741809
chr11	61742014	61742348
chr11	61742484	61743384
chr11	61743994	61744391
chr11	61745431	61745713
chr11	61745846	61746881
chr11	61747194	61747335
chr11	61747958	61749139
chr11	61751625	61751903
chr11	61766608	61766619

chr11	61766830	61766947
chr11	61779939	61780080
chr11	61780307	61780324
chr11	61780563	61781486
chr11	61783175	61783510
chr11	61784954	61785286
chr11	61792808	61792960
chr11	61793190	61793721
chr11	61796587	61796778
chr11	61798406	61798580
chr11	61798778	61799034
chr11	61803114	61803324
chr11	61808744	61808784
chr11	61811732	61811762
chr11	61812080	61812315
chr11	61812439	61812664
chr11	61813114	61813426
chr11	61813596	61813811
chr11	61814088	61814340
chr11	61814501	61814568
chr11	61815238	61815449
chr11	61815784	61816015
chr11	61827083	61827137
chr11	61827652	61827893
chr11	61867082	61867292
chr11	61875146	61875417
chr11	61877863	61877935
chr11	61879884	61880053
chr11	61880189	61880287
chr11	61887797	61887985
chr11	61889865	61889934
chr11	61891015	61891157
chr11	61891567	61892647
chr11	61892892	61893153
chr11	61899600	61899613
chr11	61899250	61899391
chr11	61903823	61903902
chr11	61904532	61904810
chr11	61911456	61911655
chr11	62078879	62079065
chr11	62104522	62104828
chr11	62105004	62105859
chr11	62162230	62162964
chr11	62163109	62163133
chr11	62163508	62163532
chr11	62164637	62164973

chr11	62177139	62177338
chr11	62192029	62192309
chr11	62192460	62192659
chr11	62194785	62194915
chr11	62195190	62195251
chr11	62195509	62195951
chr11	62223410	62223503
chr11	62227404	62227474
chr11	62233567	62233898
chr11	62247830	62248029
chr11	62262104	62262231
chr11	62262824	62263088
chr11	62264505	62264673
chr11	62264864	62265347
chr11	62266524	62266756
chr11	62266887	62268482
chr11	62268622	62269134
chr11	62269876	62270175
chr11	62271336	62271498
chr11	62277516	62277715
chr11	62282674	62282948
chr11	62303968	62304197
chr11	62304421	62305214
chr11	62305355	62305679
chr11	62306385	62306772
chr11	62307036	62307738
chr11	62307995	62308194
chr11	62308684	62309160
chr11	62309666	62317642
chr11	62318000	62318294
chr11	62318644	62318766
chr11	62319208	62320150
chr11	62320301	62321373
chr11	62321514	62323146
chr11	62323368	62325928
chr11	62326144	62327017
chr11	62327232	62328956
chr11	62329210	62329301
chr11	62330460	62330828
chr11	62331353	62331422
chr11	62331985	62332481
chr11	62332606	62332889
chr11	62335079	62336151
chr11	62336518	62336784
chr11	62337593	62337825
chr11	62340358	62341400

chr11	62341703	62342458
chr11	62342633	62342998
chr11	62345330	62345569
chr11	62345709	62346032
chr11	62347059	62347424
chr11	62347681	62347835
chr11	62349485	62349635
chr11	62353084	62353418
chr11	62355649	62356266
chr11	62356797	62357156
chr11	62357686	62358609
chr11	62358751	62359070
chr11	62359308	62360106
chr11	62362996	62363400
chr11	62364623	62364830
chr11	62366000	62366009
chr11	62366202	62366235
chr11	62366529	62371340
chr11	62371477	62371676
chr11	62371883	62372184
chr11	62374209	62374408
chr11	62376488	62376782
chr11	62378492	62380741
chr11	62380883	62381750
chr11	62382005	62382824
chr11	62385422	62385650
chr11	62386916	62387442
chr11	62391140	62392017
chr11	62392244	62392494
chr11	62387831	62391055
chr11	62393064	62393264
chr11	62394848	62395446
chr11	62398924	62399440
chr11	62399558	62399829
chr11	62400904	62401293
chr11	62401500	62401823
chr11	62404002	62404139
chr11	62405616	62405946
chr11	62407408	62407607
chr11	62408130	62410129
chr11	62410732	62411264
chr11	62411347	62411864
chr11	62412804	62412868
chr11	62413104	62414222
chr11	62417497	62417576
chr11	62417667	62418164

chr11	62418586	62418607
chr11	62419812	62419900
chr11	62420039	62421341
chr11	62421668	62422169
chr11	62427998	62428274
chr11	62428417	62428601
chr11	62430867	62431023
chr11	62431277	62431654
chr11	62431792	62432636
chr11	62432796	62434132
chr11	62434607	62434753
chr11	62434846	62435156
chr11	62435375	62435492
chr11	62435795	62436125
chr11	62437023	62437222
chr11	62441361	62441522
chr11	62438578	62440997
chr11	62441799	62441838
chr11	62444068	62444117
chr11	62444733	62444756
chr11	62445901	62447675
chr11	62448147	62448406
chr11	62448539	62449095
chr11	62454203	62454544
chr11	62461348	62461578
chr11	62464095	62464310
chr11	62467340	62467627
chr11	62469600	62469788
chr11	62471444	62471790
chr11	62472081	62472364
chr11	62473082	62473812
chr11	62473971	62474170
chr11	62475422	62475621
chr11	62486353	62486571
chr11	62493169	62493480
chr11	62493670	62497411
chr11	62497481	62497955
chr11	62498265	62498615
chr11	62499509	62499821
chr11	62500315	62500439
chr11	62500545	62500631
chr11	62502574	62502600
chr11	62510573	62510844
chr11	62514814	62515169
chr11	62516997	62517582
chr11	62519187	62519436

chr11	62520762	62521030
chr11	62521196	62522630
chr11	62522998	62523197
chr11	62527892	62528019
chr11	62528394	62528864
chr11	62529062	62529601
chr11	62531386	62531667
chr11	62533215	62533359
chr11	62536757	62536799
chr11	62537014	62537156
chr11	62537510	62537667
chr11	62538086	62538620
chr11	62538797	62539473
chr11	62539540	62540195
chr11	62541133	62541277
chr11	62542607	62542682
chr11	62544518	62544971
chr11	62546225	62546778
chr11	62547623	62547996
chr11	62553055	62553296
chr11	62553566	62554080
chr11	62556241	62556318
chr11	62554204	62556134
chr11	62557839	62558042
chr11	62558169	62560077
chr11	62560191	62560396
chr11	62560987	62561235
chr11	62561416	62561574
chr11	62564901	62565142
chr11	62570026	62570182
chr11	62570834	62571031
chr11	62571821	62572950
chr11	62573216	62574117
chr11	62574237	62574602
chr11	62574834	62575116
chr11	62575749	62575871
chr11	62585680	62585841
chr11	62587006	62587241
chr11	62591033	62591486
chr11	62595910	62596011
chr11	62596275	62597801
chr11	62598228	62600358
chr11	62603323	62603538
chr11	62604003	62604395
chr11	62605579	62605641
chr11	62605999	62606198

chr11	62606396	62610212
chr11	62610349	62610799
chr11	62616828	62617110
chr11	62617676	62617786
chr11	62617868	62619754
chr11	62619957	62620056
chr11	62621157	62621419
chr11	62622373	62623361
chr11	62623520	62625440
chr11	62625791	62626125
chr11	62627457	62627745
chr11	62627990	62628355
chr11	62628507	62628819
chr11	62629317	62629754
chr11	62629981	62630944
chr11	62633662	62634044
chr11	62634639	62634911
chr11	62635403	62635660
chr11	62640601	62640916
chr11	62643298	62644032
chr11	62644610	62644921
chr11	62645118	62645122
chr11	62645238	62645695
chr11	62645817	62645902
chr11	62646294	62646862
chr11	62646972	62648215
chr11	62648365	62649745
chr11	62649874	62650235
chr11	62650362	62650744
chr11	62651318	62651517
chr11	62651617	62652217
chr11	62652307	62653030
chr11	62653390	62653554
chr11	62653798	62654693
chr11	62654909	62655416
chr11	62657971	62658250
chr11	62660769	62661085
chr11	62661228	62661629
chr11	62667186	62667349
chr11	62676680	62677180
chr11	62688968	62689167
chr11	62767732	62767931
chr11	62802669	62802913
chr11	63246535	63246757
chr11	63289226	63289317
chr11	63303788	63303865

chr11	63304092	63304234
chr11	63330801	63330973
chr11	63331991	63332514
chr11	63332690	63333110
chr11	63333186	63333387
chr11	63333816	63334184
chr11	63334462	63335216
chr11	63363485	63363692
chr11	63369377	63369670
chr11	63380613	63381325
chr11	63381810	63382320
chr11	63393392	63393426
chr11	63393656	63393801
chr11	63409263	63409379
chr11	63425414	63425620
chr11	63426730	63426815
chr11	63430762	63430859
chr11	63431292	63431683
chr11	63432675	63432940
chr11	63433259	63433705
chr11	63434378	63434833
chr11	63435317	63436006
chr11	63436624	63437117
chr11	63437258	63439621
chr11	63443025	63443334
chr11	63445762	63445813
chr11	63448014	63448394
chr11	63448500	63449637
chr11	63454025	63454275
chr11	63455315	63455391
chr11	63456743	63456989
chr11	63463175	63463188
chr11	63504556	63504638
chr11	63506180	63506349
chr11	63513308	63513365
chr11	63514460	63514709
chr11	63514852	63514943
chr11	63535097	63535326
chr11	63535547	63536123
chr11	63536273	63537205
chr11	63558844	63558993
chr11	63565120	63565121
chr11	63567872	63568050
chr11	63571700	63572186
chr11	63572861	63573300
chr11	63573454	63574217

chr11	63569573	63571267
chr11	63575340	63575624
chr11	63576194	63576316
chr11	63576592	63577090
chr11	63577514	63578427
chr11	63579054	63579507
chr11	63580005	63580787
chr11	63581024	63582122
chr11	63583176	63583524
chr11	63583778	63584304
chr11	63584588	63584718
chr11	63588893	63589749
chr11	63595425	63595698
chr11	63598089	63598288
chr11	63598759	63599458
chr11	63600997	63601228
chr11	63601495	63601736
chr11	63603331	63603740
chr11	63603183	63603192
chr11	63604861	63606070
chr11	63606224	63608440
chr11	63608901	63609336
chr11	63611190	63611359
chr11	63612780	63613581
chr11	63614768	63615223
chr11	63615364	63615546
chr11	63615710	63615843
chr11	63616418	63616490
chr11	63617096	63617220
chr11	63621670	63621997
chr11	63626046	63626465
chr11	63628040	63628127
chr11	63632219	63632234
chr11	63632376	63632689
chr11	63638786	63639996
chr11	63641066	63641372
chr11	63649184	63649383
chr11	63649845	63650048
chr11	63651014	63651213
chr11	63652488	63652616
chr11	63653145	63653465
chr11	63653680	63653715
chr11	63654069	63654523
chr11	63654698	63655218
chr11	63655344	63655439
chr11	63655515	63657656

chr11	63660060	63660226
chr11	63683751	63684418
chr11	63684683	63688045
chr11	63689683	63689995
chr11	63701673	63701963
chr11	63703329	63703527
chr11	63705070	63707867
chr11	63711126	63711227
chr11	63713204	63713485
chr11	63713673	63713732
chr11	63714508	63714615
chr11	63715189	63715503
chr11	63716419	63716631
chr11	63717310	63717327
chr11	63717422	63717957
chr11	63720446	63720680
chr11	63726718	63726917
chr11	63734787	63735003
chr11	63735405	63735722
chr11	63741247	63741893
chr11	63742001	63743413
chr11	63744135	63744555
chr11	63744656	63745047
chr11	63745610	63745799
chr11	63749317	63749724
chr11	63751067	63751266
chr11	63751766	63752020
chr11	63752399	63752540
chr11	63752798	63753802
chr11	63753923	63754681
chr11	63754858	63755097
chr11	63755455	63755654
chr11	63756408	63756616
chr11	63756697	63756960
chr11	63759504	63759703
chr11	63760110	63760309
chr11	63762574	63762862
chr11	63763306	63763569
chr11	63765787	63766522
chr11	63766705	63767567
chr11	63767760	63768698
chr11	63774089	63774569
chr11	63775093	63775292
chr11	63788575	63789146
chr11	63789598	63789797
chr11	63790374	63790726

chr11	63800944	63801142
chr11	63803566	63803790
chr11	63803988	63804354
chr11	63811349	63811548
chr11	63857272	63857471
chr11	63858247	63858771
chr11	63858894	63859290
chr11	63861139	63862035
chr11	63862791	63863071
chr11	63887720	63890191
chr11	63898777	63899109
chr11	63914588	63914670
chr11	63926640	63927203
chr11	63927793	63928177
chr11	63929196	63929219
chr11	63932498	63934395
chr11	63934516	63935018
chr11	63948800	63950788
chr11	63951764	63953176
chr11	63953490	63954674
chr11	63955128	63955821
chr11	63955908	63956324
chr11	63956526	63956801
chr11	63957412	63957942
chr11	63958547	63958808
chr11	63959515	63959558
chr11	63961027	63961328
chr11	63965611	63966072
chr11	63967846	63968096
chr11	63968345	63968888
chr11	63970107	63970310
chr11	63971483	63971737
chr11	63972321	63973208
chr11	63974256	63974801
chr11	63974934	63975375
chr11	63981772	63981817
chr11	63982462	63982666
chr11	63983356	63983806
chr11	63989439	63989666
chr11	63989954	63990073
chr11	63991191	63991618
chr11	63992278	63992482
chr11	63992913	63994826
chr11	63994977	63995870
chr11	63996099	63996547
chr11	63997527	63997888

chr11	63998029	63999187
chr11	64000644	64001048
chr11	64001277	64001915
chr11	64002252	64003244
chr11	64004278	64004501
chr11	64006864	64010756
chr11	64012431	64014870
chr11	64015141	64015589
chr11	64018098	64020235
chr11	64020467	64020768
chr11	64024494	64025110
chr11	64028440	64028799
chr11	64030278	64030541
chr11	64031206	64031639
chr11	64034202	64034516
chr11	64035101	64039939
chr11	64040056	64040457
chr11	64040573	64040867
chr11	64041270	64041291
chr11	64041034	64041154
chr11	64041681	64041969
chr11	64042200	64042483
chr11	64043461	64043752
chr11	64043860	64044277
chr11	64044621	64044978
chr11	64045215	64045233
chr11	64045404	64045438
chr11	64045622	64045637
chr11	64046922	64047280
chr11	64048170	64048699
chr11	64050060	64050341
chr11	64051227	64052133
chr11	64052303	64054004
chr11	64054866	64055078
chr11	64059039	64060173
chr11	64061146	64061425
chr11	64066030	64066103
chr11	64066202	64066453
chr11	64067321	64067782
chr11	64068011	64069511
chr11	64069915	64070191
chr11	64070391	64070769
chr11	64070874	64071117
chr11	64071381	64076074
chr11	64076222	64076421
chr11	64079607	64080199

chr11	64077041	64079285
chr11	64080715	64080957
chr11	64081549	64081844
chr11	64084228	64087800
chr11	64088084	64090021
chr11	64090287	64090904
chr11	64091016	64093631
chr11	64093725	64094139
chr11	64094441	64094636
chr11	64095160	64095304
chr11	64095629	64096088
chr11	64096171	64096557
chr11	64097177	64097658
chr11	64097800	64097817
chr11	64098020	64098330
chr11	64098586	64101120
chr11	64101507	64102623
chr11	64102838	64103303
chr11	64104261	64104725
chr11	64105005	64105666
chr11	64106013	64106480
chr11	64106670	64107246
chr11	64109459	64110817
chr11	64111894	64112140
chr11	64112755	64115654
chr11	64115872	64116343
chr11	64117802	64118439
chr11	64121929	64124154
chr11	64124532	64127162
chr11	64132429	64132585
chr11	64134565	64134809
chr11	64140383	64141081
chr11	64141784	64141793
chr11	64146059	64146698
chr11	64152587	64152827
chr11	64154389	64154596
chr11	64158915	64158922
chr11	64159711	64159959
chr11	64167922	64168196
chr11	64176092	64176130
chr11	64193558	64193566
chr11	64204322	64204424
chr11	64204674	64204843
chr11	64205362	64205407
chr11	64206032	64206128
chr11	64206635	64206854

chr11	64215418	64215617
chr11	64216129	64217239
chr11	64257051	64257309
chr11	64257487	64257966
chr11	64259513	64259778
chr11	64287326	64287531
chr11	64291970	64292229
chr11	64295146	64295163
chr11	64303790	64304069
chr11	64304342	64304364
chr11	64304487	64304787
chr11	64304959	64305258
chr11	64305495	64306048
chr11	64307602	64307913
chr11	64306476	64307366
chr11	64308149	64309071
chr11	64309204	64311135
chr11	64311639	64311866
chr11	64312111	64314339
chr11	64314549	64315116
chr11	64315733	64315935
chr11	64316464	64317170
chr11	64317384	64317829
chr11	64318058	64318491
chr11	64319107	64319280
chr11	64320517	64320733
chr11	64321128	64321671
chr11	64322785	64322997
chr11	64323421	64323673
chr11	64325493	64325543
chr11	64333310	64335027
chr11	64336495	64336854
chr11	64337936	64338178
chr11	64338518	64340270
chr11	64353125	64353341
chr11	64397719	64397918
chr11	64407254	64407453
chr11	64409388	64409587
chr11	64410347	64412279
chr11	64412476	64413132
chr11	64417802	64418001
chr11	64420835	64421164
chr11	64422536	64423484
chr11	64423596	64424909
chr11	64425157	64428607
chr11	64428714	64429086

chr11	64429308	64429512
chr11	64429938	64430179
chr11	64430552	64430688
chr11	64431071	64431221
chr11	64431636	64431847
chr11	64443274	64443545
chr11	64446071	64446077
chr11	64450874	64451137
chr11	64452009	64452841
chr11	64453195	64453565
chr11	64455753	64455830
chr11	64455448	64455594
chr11	64470330	64470524
chr11	64477277	64477494
chr11	64490761	64491521
chr11	64494937	64495136
chr11	64497022	64497221
chr11	64506427	64506626
chr11	64510034	64511002
chr11	64511430	64513411
chr11	64513525	64514439
chr11	64515157	64517057
chr11	64517173	64517621
chr11	64518195	64518725
chr11	64523937	64524136
chr11	64524584	64524885
chr11	64529425	64529810
chr11	64530952	64531418
chr11	64531564	64531906
chr11	64535009	64535404
chr11	64538031	64538124
chr11	64542862	64543089
chr11	64544835	64545246
chr11	64545357	64546846
chr11	64546985	64547276
chr11	64550392	64550705
chr11	64551293	64551409
chr11	64552032	64552188
chr11	64552687	64552937
chr11	64555352	64555573
chr11	64562833	64563032
chr11	64566473	64566713
chr11	64569890	64570644
chr11	64570807	64571759
chr11	64572136	64572335
chr11	64575325	64575627

chr11	64576032	64576312
chr11	64576978	64578051
chr11	64578239	64578816
chr11	64578979	64579334
chr11	64580849	64580894
chr11	64580992	64581202
chr11	64582490	64582724
chr11	64582859	64583350
chr11	64583660	64584006
chr11	64586376	64586387
chr11	64598527	64598726
chr11	64602425	64602763
chr11	64602930	64603171
chr11	64608386	64613907
chr11	64613980	64614328
chr11	64614436	64614635
chr11	64614792	64615773
chr11	64616160	64616444
chr11	64616566	64616879
chr11	64618100	64618679
chr11	64618785	64619190
chr11	64621152	64621351
chr11	64622103	64622532
chr11	64622885	64625162
chr11	64625587	64625957
chr11	64626807	64627103
chr11	64628441	64628640
chr11	64631273	64631523
chr11	64632144	64632485
chr11	64633562	64633799
chr11	64633911	64634081
chr11	64635097	64635616
chr11	64634377	64635033
chr11	64635799	64636602
chr11	64636906	64637504
chr11	64637792	64638065
chr11	64638298	64638517
chr11	64642684	64643085
chr11	64644133	64646076
chr11	64646386	64647365
chr11	64648682	64648960
chr11	64652701	64652900
chr11	64653071	64653986
chr11	64654516	64654763
chr11	64655258	64655776
chr11	64657390	64657684

chr11	64659268	64659629
chr11	64667258	64668126
chr11	64668836	64669035
chr11	64680051	64680291
chr11	64682968	64683560
chr11	64683686	64684868
chr11	64685106	64686384
chr11	64686662	64686872
chr11	64689940	64690107
chr11	64691177	64693235
chr11	64725829	64726070
chr11	64734326	64734683
chr11	64735791	64736052
chr11	64736528	64737489
chr11	64737906	64740096
chr11	64750599	64750815
chr11	64751813	64752021
chr11	64755005	64755174
chr11	64762483	64762682
chr11	64762810	64763009
chr11	64763240	64763293
chr11	64763711	64764958
chr11	64765375	64765419
chr11	64768785	64769034
chr11	64772001	64772389
chr11	64772861	64773773
chr11	64773876	64775837
chr11	64776195	64776343
chr11	64777897	64778234
chr11	64779527	64779824
chr11	64780466	64780485
chr11	64780605	64782929
chr11	64783855	64784082
chr11	64788133	64788381
chr11	64792326	64792439
chr11	64793967	64794320
chr11	64794433	64795954
chr11	64797131	64797285
chr11	64804607	64804702
chr11	64804770	64805048
chr11	64808048	64809905
chr11	64810191	64810559
chr11	64810831	64811196
chr11	64814825	64815376
chr11	64816601	64816848
chr11	64835897	64836965

chr11	64837090	64837299
chr11	64838006	64838203
chr11	64838796	64839042
chr11	64843589	64843709
chr11	64847433	64847490
chr11	64847579	64848357
chr11	64848611	64848940
chr11	64849143	64849503
chr11	64850149	64850447
chr11	64850683	64852881
chr11	64849880	64849921
chr11	64862317	64862528
chr11	64862902	64863101
chr11	64863297	64864446
chr11	64864858	64865294
chr11	64865625	64865842
chr11	64864696	64864753
chr11	64866973	64867176
chr11	64870338	64870529
chr11	64879372	64881502
chr11	64884154	64885103
chr11	64885431	64886062
chr11	64887433	64887921
chr11	64888019	64888240
chr11	64888414	64888639
chr11	64888736	64890470
chr11	64890864	64891339
chr11	64892029	64892324
chr11	64893698	64893897
chr11	64894133	64894332
chr11	64903515	64903810
chr11	64900478	64903149
chr11	64906382	64906786
chr11	64917192	64917429
chr11	64917869	64918974
chr11	64919695	64919704
chr11	64919948	64920015
chr11	64936023	64936162
chr11	64943458	64943741
chr11	64946097	64946296
chr11	64947039	64947188
chr11	64947543	64947836
chr11	64947979	64952116
chr11	64952324	64952523
chr11	64953665	64953934
chr11	64967390	64967763

chr11	64970831	64971093
chr11	64980333	64980775
chr11	64989439	64989638
chr11	64992082	64993683
chr11	64993770	64993969
chr11	64999829	64999993
chr11	65021113	65021251
chr11	65021710	65022412
chr11	65025485	65026104
chr11	65027472	65027714
chr11	65028880	65029219
chr11	65029363	65030615
chr11	65030942	65031226
chr11	65035261	65035522
chr11	65042424	65042578
chr11	65042882	65042922
chr11	65047129	65047313
chr11	65047438	65047772
chr11	65050273	65050395
chr11	65052697	65052932
chr11	65055558	65055694
chr11	65055925	65055947
chr11	65056021	65056275
chr11	65058075	65058337
chr11	65058527	65058787
chr11	65058975	65059805
chr11	65059927	65060264
chr11	65060952	65061096
chr11	65060508	65060874
chr11	65063372	65063571
chr11	65070225	65070424
chr11	65070568	65070855
chr11	65073824	65074268
chr11	65076097	65077145
chr11	65077318	65077490
chr11	65078089	65078406
chr11	65078559	65078776
chr11	65079258	65079943
chr11	65080645	65080956
chr11	65081453	65083257
chr11	65083521	65086937
chr11	65090898	65091126
chr11	65091220	65091336
chr11	65092339	65092528
chr11	65093027	65093226
chr11	65097392	65097516

chr11	65097636	65098602
chr11	65100073	65100295
chr11	65100599	65102195
chr11	65103319	65103486
chr11	65103742	65104040
chr11	65104693	65104892
chr11	65105233	65105362
chr11	65109628	65109828
chr11	65115232	65115404
chr11	65116976	65117077
chr11	65117152	65117214
chr11	65121756	65122084
chr11	65122216	65123440
chr11	65125505	65125704
chr11	65127972	65128194
chr11	65128594	65128799
chr11	65130382	65130561
chr11	65133186	65134319
chr11	65134798	65135042
chr11	65135469	65135668
chr11	65135871	65136132
chr11	65136321	65136366
chr11	65137918	65138643
chr11	65138933	65139091
chr11	65139476	65139892
chr11	65140449	65140648
chr11	65141044	65141328
chr11	65142187	65142481
chr11	65142845	65143044
chr11	65143332	65143606
chr11	65148574	65149717
chr11	65149954	65151975
chr11	65152096	65152893
chr11	65154901	65155226
chr11	65153049	65154745
chr11	65155329	65155653
chr11	65157982	65158181
chr11	65158959	65159362
chr11	65176548	65177304
chr11	65181997	65182365
chr11	65183333	65183922
chr11	65184045	65187958
chr11	65193374	65196295
chr11	65196525	65197015
chr11	65197246	65197918
chr11	65188276	65193023

chr11	65200393	65200793
chr11	65201281	65201784
chr11	65202868	65203401
chr11	65205033	65205183
chr11	65206785	65207159
chr11	65207308	65207516
chr11	65207774	65208459
chr11	65208703	65208909
chr11	65209501	65210516
chr11	65211033	65211287
chr11	65211982	65212254
chr11	65214920	65215654
chr11	65216981	65217332
chr11	65219952	65222662
chr11	65222827	65223139
chr11	65224487	65224666
chr11	65224787	65225193
chr11	65225670	65225797
chr11	65228456	65228783
chr11	65234183	65234364
chr11	65234775	65234850
chr11	65235025	65235234
chr11	65235480	65236017
chr11	65236246	65236314
chr11	65236571	65236935
chr11	65237351	65240068
chr11	65241710	65242059
chr11	65242307	65243030
chr11	65243110	65247706
chr11	65248663	65249459
chr11	65249772	65250133
chr11	65240853	65241478
chr11	65251652	65251962
chr11	65252700	65253248
chr11	65253332	65253745
chr11	65254659	65255139
chr11	65255300	65256235
chr11	65256594	65256793
chr11	65256912	65257213
chr11	65258450	65260902
chr11	65260965	65261784
chr11	65263427	65265531
chr11	65265618	65266161
chr11	65267777	65268267
chr11	65271507	65272014
chr11	65257333	65258350

chr11	65272547	65273458
chr11	65276795	65277536
chr11	65277928	65279438
chr11	65279507	65279554
chr11	65273604	65276702
chr11	65279957	65280389
chr11	65283178	65283432
chr11	65284583	65284868
chr11	65285924	65286037
chr11	65287861	65288004
chr11	65291723	65292284
chr11	65292494	65293940
chr11	65295632	65296091
chr11	65306473	65310411
chr11	65314555	65314808
chr11	65318320	65318676
chr11	65322803	65323109
chr11	65323548	65323883
chr11	65324951	65326642
chr11	65327182	65328019
chr11	65328686	65328919
chr11	65330494	65330693
chr11	65331293	65331640
chr11	65332661	65332750
chr11	65333902	65334126
chr11	65334432	65334632
chr11	65335270	65335525
chr11	65335693	65336212
chr11	65336326	65339118
chr11	65339540	65340751
chr11	65342049	65344638
chr11	65345170	65345791
chr11	65356495	65356782
chr11	65358310	65358847
chr11	65359616	65360903
chr11	65361232	65361431
chr11	65361603	65361802
chr11	65364480	65364679
chr11	65374152	65374570
chr11	65374781	65375420
chr11	65380679	65382812
chr11	65383238	65384253
chr11	65384358	65384705
chr11	65405493	65406807
chr11	65414287	65415690
chr11	65416038	65416227

chr11	65416572	65416600
chr11	65417796	65418065
chr11	65418406	65418643
chr11	65418884	65419086
chr11	65419211	65421087
chr11	65422309	65422508
chr11	65428712	65428810
chr11	65429267	65431555
chr11	65431873	65432133
chr11	65448485	65448734
chr11	65448882	65448979
chr11	65456933	65457141
chr11	65474724	65474997
chr11	65476849	65477116
chr11	65477662	65480696
chr11	65481766	65482015
chr11	65483284	65483510
chr11	65487172	65488201
chr11	65488394	65488529
chr11	65497373	65497396
chr11	65534858	65535055
chr11	65535183	65535742
chr11	65537294	65537308
chr11	65537879	65538167
chr11	65538398	65538499
chr11	65540372	65540468
chr11	65541754	65541970
chr11	65542033	65542236
chr11	65543085	65543321
chr11	65543668	65543755
chr11	65544277	65544784
chr11	65547389	65548490
chr11	65548765	65549330
chr11	65552530	65552560
chr11	65552361	65552390
chr11	65554593	65555676
chr11	65559348	65560124
chr11	65560244	65560516
chr11	65561036	65561236
chr11	65567528	65567787
chr11	65585260	65585746
chr11	65586141	65586199
chr11	65586450	65586578
chr11	65587223	65587548
chr11	65589070	65589238
chr11	65600419	65601310

chr11	65602579	65602660
chr11	65601462	65601861
chr11	65612940	65613156
chr11	65624354	65625840
chr11	65625943	65629423
chr11	65639868	65641020
chr11	65641198	65641911
chr11	65642833	65643038
chr11	65654893	65659296
chr11	65659430	65660012
chr11	65660933	65661368
chr11	65662130	65662658
chr11	65663191	65663669
chr11	65663976	65667928
chr11	65668813	65669477
chr11	65668222	65668467
chr11	65669743	65670711
chr11	65670869	65671360
chr11	65671440	65672024
chr11	65674722	65675110
chr11	65678267	65678631
chr11	65678869	65679348
chr11	65679468	65684615
chr11	65685047	65686655
chr11	65686813	65688214
chr11	65690712	65690735
chr11	65690472	65690595
chr11	65694845	65694927
chr11	65695846	65695922
chr11	65696935	65696979
chr11	65700160	65700699
chr11	65703529	65703559
chr11	65708435	65708650
chr11	65716299	65716333
chr11	65719708	65719738
chr11	65722138	65722423
chr11	65722530	65722687
chr11	65722959	65722963
chr11	65727819	65729940
chr11	65730093	65730454
chr11	65730816	65731558
chr11	65735747	65735777
chr11	65736964	65737294
chr11	65737361	65737374
chr11	65738170	65739008
chr11	65741558	65742037

chr11	65752919	65753196
chr11	65756720	65757103
chr11	65758359	65758669
chr11	65767298	65767970
chr11	65768414	65770672
chr11	65770767	65770854
chr11	65772279	65772927
chr11	65775715	65777503
chr11	65778055	65778433
chr11	65778612	65779725
chr11	65782004	65782532
chr11	65782707	65783051
chr11	65789504	65789913
chr11	65794569	65795190
chr11	65795530	65795677
chr11	65797967	65798001
chr11	65798625	65798767
chr11	65798074	65798505
chr11	65799380	65799383
chr11	65801111	65801141
chr11	65817814	65818062
chr11	65818165	65819569
chr11	65819801	65820406
chr11	65826597	65826956
chr11	65827626	65827656
chr11	65828075	65828245
chr11	65829255	65829342
chr11	65832555	65832781
chr11	65833374	65833612
chr11	65836029	65836281
chr11	65836775	65837689
chr11	65837793	65838864
chr11	65839085	65839407
chr11	65841315	65841601
chr11	65844746	65845000
chr11	65847827	65848028
chr11	65848146	65848766
chr11	65848844	65849387
chr11	65862948	65863196
chr11	65867090	65867299
chr11	65867536	65867603
chr11	65869880	65870063
chr11	65871125	65871315
chr11	65879357	65879879
chr11	65880236	65880993
chr11	65883217	65883238

chr11	65885988	65886206
chr11	65886646	65887269
chr11	65887453	65887565
chr11	65893646	65893860
chr11	65894158	65895055
chr11	65900323	65900392
chr11	65901103	65901168
chr11	65901746	65901945
chr11	65904332	65904556
chr11	65904929	65904990
chr11	65905226	65905249
chr11	65909365	65909772
chr11	65909860	65910613
chr11	65944121	65944357
chr11	65977042	65977290
chr11	65982272	65982496
chr11	65989236	65989481
chr11	65989815	65990233
chr11	65990439	65990620
chr11	65991346	65991424
chr11	66015950	66015978
chr11	66023718	66026517
chr11	66034280	66037151
chr11	66037307	66037684
chr11	66038462	66038661
chr11	66038775	66038916
chr11	66045301	66045728
chr11	66046079	66046383
chr11	66049081	66049374
chr11	66055666	66057455
chr11	66075703	66075902
chr11	66078347	66078775
chr11	66078952	66080249
chr11	66080423	66081146
chr11	66085643	66086731
chr11	66091952	66093035
chr11	66093358	66093906
chr11	66094027	66096920
chr11	66097077	66097390
chr11	66097660	66097942
chr11	66098860	66099059
chr11	66102819	66107348
chr11	66111312	66112538
chr11	66112727	66113121
chr11	66113919	66113945
chr11	66114164	66115008

chr11	66115250	66116065
chr11	66116377	66116476
chr11	66117199	66117388
chr11	66126763	66126863
chr11	66135843	66136094
chr11	66137420	66140503
chr11	66141012	66141191
chr11	66141517	66141852
chr11	66147842	66148223
chr11	66149128	66149388
chr11	66153200	66153390
chr11	66153645	66154509
chr11	66159613	66159662
chr11	66175815	66175869
chr11	66175556	66175651
chr11	66200735	66200942
chr11	66201295	66201494
chr11	66205127	66206952
chr11	66211502	66211578
chr11	66213528	66213710
chr11	66216222	66216308
chr11	66222136	66222454
chr11	66233780	66235361
chr11	66246765	66247684
chr11	66247804	66248431
chr11	66248717	66249257
chr11	66250583	66250774
chr11	66253240	66253547
chr11	66277674	66277884
chr11	66278094	66278684
chr11	66283621	66283826
chr11	66286337	66286621
chr11	66291479	66291543
chr11	66312926	66314331
chr11	66326152	66326463
chr11	66335366	66336570
chr11	66349573	66349772
chr11	66359985	66361640
chr11	66361896	66361912
chr11	66365258	66365355
chr11	66369243	66369332
chr11	66374156	66374158
chr11	66374501	66374925
chr11	66375121	66375800
chr11	66376918	66376980
chr11	66378487	66378686

chr11	66381365	66381582
chr11	66382969	66383171
chr11	66383416	66383893
chr11	66384093	66385162
chr11	66385744	66385914
chr11	66386515	66387079
chr11	66388152	66388193
chr11	66388330	66388808
chr11	66390004	66390250
chr11	66399416	66399527
chr11	66400963	66401109
chr11	66403731	66404167
chr11	66405067	66405842
chr11	66404418	66404561
chr11	66406184	66406947
chr11	66415994	66416261
chr11	66416574	66416777
chr11	66418975	66419146
chr11	66420769	66421036
chr11	66421883	66421999
chr11	66423702	66423901
chr11	66425037	66425063
chr11	66425293	66425328
chr11	66425489	66425726
chr11	66431940	66431961
chr11	66435219	66435448
chr11	66439300	66439509
chr11	66442815	66442957
chr11	66444403	66444599
chr11	66444740	66446393
chr11	66451794	66452391
chr11	66452614	66455239
chr11	66458289	66458592
chr11	66462553	66462911
chr11	66475673	66475991
chr11	66476278	66476505
chr11	66477172	66477900
chr11	66479555	66479931
chr11	66480898	66481165
chr11	66487437	66487935
chr11	66486694	66487374
chr11	66488190	66488858
chr11	66490113	66490248
chr11	66494415	66495626
chr11	66495839	66496679
chr11	66510570	66510684

chr11	66510966	66511631
chr11	66511697	66513385
chr11	66514491	66514771
chr11	66515190	66515420
chr11	66520228	66520231
chr11	66527748	66527888
chr11	66536292	66536705
chr11	66535159	66535824
chr11	66539067	66539222
chr11	66541280	66541467
chr11	66583121	66583448
chr11	66599248	66599346
chr11	66599767	66599858
chr11	66600666	66600736
chr11	66601149	66601480
chr11	66601607	66601641
chr11	66603446	66603845
chr11	66605503	66605759
chr11	66608723	66609220
chr11	66609405	66609648
chr11	66609762	66611587
chr11	66622794	66625305
chr11	66630622	66630838
chr11	66644813	66645093
chr11	66645485	66646570
chr11	66647116	66647827
chr11	66648100	66648978
chr11	66651605	66651841
chr11	66651915	66651981
chr11	66650691	66651499
chr11	66652823	66653121
chr11	66656345	66656602
chr11	66656842	66656975
chr11	66659395	66660121
chr11	66660399	66660574
chr11	66664388	66664701
chr11	66665948	66666038
chr11	66671219	66671593
chr11	66671769	66671956
chr11	66672371	66672470
chr11	66673250	66673990
chr11	66674175	66674382
chr11	66674841	66674917
chr11	66690493	66690638
chr11	66696824	66697088
chr11	66702103	66702181

chr11	66702724	66702862
chr11	66703250	66703502
chr11	66703805	66703955
chr11	66704263	66704344
chr11	66705068	66705070
chr11	66715775	66715793
chr11	66716126	66716279
chr11	66717474	66718098
chr11	66718454	66718653
chr11	66720476	66720761
chr11	66723222	66723369
chr11	66723767	66723966
chr11	66724319	66724734
chr11	66725061	66725741
chr11	66726313	66727287
chr11	66727668	66728137
chr11	66729793	66729906
chr11	66733334	66733384
chr11	66732798	66733206
chr11	66734306	66734783
chr11	66735052	66735998
chr11	66736296	66736936
chr11	66737070	66737560
chr11	66737752	66739399
chr11	66740048	66740696
chr11	66741465	66742516
chr11	66742858	66743132
chr11	66743376	66743575
chr11	66747487	66747634
chr11	66749471	66750130
chr11	66749165	66749334
chr11	66751616	66752345
chr11	66760589	66760767
chr11	66761320	66761519
chr11	66775119	66775318
chr11	66776573	66776582
chr11	66788776	66789042
chr11	66789341	66789796
chr11	66789962	66790654
chr11	66790791	66791691
chr11	66797291	66797535
chr11	66799434	66799633
chr11	66799897	66800186
chr11	66800252	66800639
chr11	66800760	66801340
chr11	66802515	66802694

chr11	66803416	66803615
chr11	66805691	66805951
chr11	66806893	66807093
chr11	66808347	66808454
chr11	66813766	66814092
chr11	66814669	66814893
chr11	66818452	66818745
chr11	66820467	66822607
chr11	66822822	66826201
chr11	66826318	66827514
chr11	66827732	66828130
chr11	66828406	66829123
chr11	66829286	66830053
chr11	66831438	66832122
chr11	66830274	66831243
chr11	66832368	66833208
chr11	66835205	66835702
chr11	66836360	66836567
chr11	66837757	66838014
chr11	66840782	66841273
chr11	66841613	66842211
chr11	66842318	66843470
chr11	66847455	66847681
chr11	66843763	66847210
chr11	66847885	66848396
chr11	66848627	66850591
chr11	66851102	66851273
chr11	66851913	66852152
chr11	66852464	66852786
chr11	66853734	66853859
chr11	66854377	66854576
chr11	66856463	66856844
chr11	66859578	66859585
chr11	66860533	66860646
chr11	66860737	66860813
chr11	66861768	66861967
chr11	66866386	66866600
chr11	66867173	66867372
chr11	66867992	66868304
chr11	66869348	66869547
chr11	66882614	66882866
chr11	66884921	66886236
chr11	66886755	66887408
chr11	66887548	66888739
chr11	66890245	66890567
chr11	66892553	66892790

chr11	66893037	66893698
chr11	66895398	66895747
chr11	66897167	66897617
chr11	66897749	66897921
chr11	66911484	66911704
chr11	66912538	66913334
chr11	66918012	66918360
chr11	66934345	66934696
chr11	66935655	66935974
chr11	66937941	66938221
chr11	66941508	66941717
chr11	66941834	66942085
chr11	66957089	66957130
chr11	66957641	66958107
chr11	66969004	66969230
chr11	66970441	66970515
chr11	66971889	66972219
chr11	66973807	66974034
chr11	66981538	66981646
chr11	66997918	66998181
chr11	67002654	67003284
chr11	67006203	67007305
chr11	67007468	67008988
chr11	67012503	67012640
chr11	67013557	67013756
chr11	67015656	67016110
chr11	67016361	67016514
chr11	67017322	67017376
chr11	67022109	67022308
chr11	67025728	67025783
chr11	67025848	67025974
chr11	67026700	67026899
chr11	67028321	67028378
chr11	67028490	67030902
chr11	67031208	67031423
chr11	67031529	67031728
chr11	67033247	67033780
chr11	67034162	67035713
chr11	67044584	67045020
chr11	67045235	67045532
chr11	67045656	67045921
chr11	67055324	67057851
chr11	67058245	67058962
chr11	67060555	67060591
chr11	67059940	67060056
chr11	67060765	67061024

chr11	67065050	67065066
chr11	67069930	67072032
chr11	67072233	67072432
chr11	67073614	67074089
chr11	67077664	67086279
chr11	67098808	67099073
chr11	67110747	67110946
chr11	67113175	67113386
chr11	67113757	67114699
chr11	67115521	67115700
chr11	67118194	67118626
chr11	67118920	67119138
chr11	67119582	67119790
chr11	67119994	67122910
chr11	67124049	67124488
chr11	67131070	67131202
chr11	67134097	67134342
chr11	67134780	67135319
chr11	67136759	67136958
chr11	67139056	67139255
chr11	67139688	67142566
chr11	67144388	67144587
chr11	67144963	67145162
chr11	67145910	67146109
chr11	67146785	67147084
chr11	67147297	67147666
chr11	67148172	67148346
chr11	67149408	67149612
chr11	67152336	67152821
chr11	67153141	67153340
chr11	67156170	67156369
chr11	67157481	67157514
chr11	67157683	67157765
chr11	67157984	67159265
chr11	67159434	67160363
chr11	67167534	67167943
chr11	67168225	67170186
chr11	67182621	67182820
chr11	67183086	67183419
chr11	67188062	67188806
chr11	67189068	67190068
chr11	67190267	67190517
chr11	67194142	67194306
chr11	67194750	67196986
chr11	67197235	67197942
chr11	67203987	67204196

chr11	67206664	67206889
chr11	67210075	67210296
chr11	67210475	67212233
chr11	67212988	67213262
chr11	67214345	67214911
chr11	67220761	67221261
chr11	67229515	67229659
chr11	67230233	67230432
chr11	67230676	67233155
chr11	67233258	67233990
chr11	67234436	67234635
chr11	67235085	67237458
chr11	67237638	67237856
chr11	67249496	67250947
chr11	67251065	67251780
chr11	67252955	67253235
chr11	67254534	67254941
chr11	67271432	67273557
chr11	67273879	67274549
chr11	67274907	67276894
chr11	67280024	67280240
chr11	67297187	67297394
chr11	67326956	67327203
chr11	67327941	67328178
chr11	67331096	67331355
chr11	67341969	67342094
chr11	67347341	67347541
chr11	67347733	67348082
chr11	67348348	67348387
chr11	67348841	67349314
chr11	67350055	67350580
chr11	67350745	67353029
chr11	67353352	67353529
chr11	67354278	67354498
chr11	67356104	67356369
chr11	67357893	67358274
chr11	67373696	67374220
chr11	67374427	67375784
chr11	67381314	67382248
chr11	67383397	67383622
chr11	67396799	67397671
chr11	67397867	67398592
chr11	67437654	67437888
chr11	67441784	67442105
chr11	67442369	67442518
chr11	67442590	67442742

chr11	67450066	67450306
chr11	67463299	67463361
chr11	67463550	67463715
chr11	67463799	67464119
chr11	67468682	67468699
chr11	67469080	67469280
chr11	67469647	67469853
chr11	67487865	67487869
chr11	67571677	67574238
chr11	67706690	67706841
chr11	67722781	67723402
chr11	67745806	67746462
chr11	67768386	67768617
chr11	67769302	67769486
chr11	67769613	67769837
chr11	67770107	67770306
chr11	67770633	67772729
chr11	67772920	67773119
chr11	67774481	67774694
chr11	67775876	67778753
chr11	67779004	67779271
chr11	67779813	67782824
chr11	67783633	67784024
chr11	67784183	67784945
chr11	67794909	67797916
chr11	67798105	67799676
chr11	67805817	67806836
chr11	67806954	67808037
chr11	67808221	67808457
chr11	67809001	67809262
chr11	67825427	67825525
chr11	67880953	67881233
chr11	67887625	67888764
chr11	67888896	67890172
chr11	67890330	67890554
chr11	67894136	67894804
chr11	67902815	67903014
chr11	67913319	67913545
chr11	67914107	67914306
chr11	67916475	67916613
chr11	67917289	67917523
chr11	67932031	67932230
chr11	67951012	67951192
chr11	67959145	67959175
chr11	67965584	67966159
chr11	67969417	67969590

chr11	67976647	67977116
chr11	67977221	67977599
chr11	67979111	67979342
chr11	67979829	67980402
chr11	67980549	67982212
chr11	67983493	67983992
chr11	68035969	68036155
chr11	68037323	68037522
chr11	68037792	68038279
chr11	68038565	68040212
chr11	68041893	68042200
chr11	68043137	68044541
chr11	68046193	68046447
chr11	68046847	68047543
chr11	68047828	68048178
chr11	68049526	68049973
chr11	68050185	68050276
chr11	68058464	68058530
chr11	68063220	68063445
chr11	68064966	68065281
chr11	68067299	68067335
chr11	68067424	68067678
chr11	68077454	68077745
chr11	68080079	68083313
chr11	68083666	68083748
chr11	68087329	68087635
chr11	68089219	68089722
chr11	68090628	68090855
chr11	68090921	68090938
chr11	68103598	68103798
chr11	68104507	68104806
chr11	68105021	68105757
chr11	68106136	68106511
chr11	68107444	68107757
chr11	68107902	68107931
chr11	68109761	68111141
chr11	68111472	68111786
chr11	68111889	68112506
chr11	68112811	68113141
chr11	68121116	68122972
chr11	68123250	68123844
chr11	68124198	68124757
chr11	68126821	68127020
chr11	68127351	68127624
chr11	68128149	68128266
chr11	68136269	68136468

chr11	68159592	68159804
chr11	68203230	68203429
chr11	68205079	68205357
chr11	68217943	68218002
chr11	68226287	68226397
chr11	68227039	68227604
chr11	68227710	68229036
chr11	68230011	68230417
chr11	68232544	68232613
chr11	68232750	68232763
chr11	68234925	68235124
chr11	68236694	68236951
chr11	68240602	68240792
chr11	68241627	68241808
chr11	68248807	68248910
chr11	68256268	68256334
chr11	68256564	68256765
chr11	68277262	68277441
chr11	68282754	68282797
chr11	68293396	68293754
chr11	68309675	68310290
chr11	68314147	68314364
chr11	68320764	68320961
chr11	68340068	68340213
chr11	68341944	68342087
chr11	68350367	68350737
chr11	68352635	68352764
chr11	68418974	68419660
chr11	68419875	68420088
chr11	68425479	68425905
chr11	68451987	68452534
chr11	68515074	68515273
chr11	68518359	68519554
chr11	68523330	68523604
chr11	68557663	68557836
chr11	68559918	68560138
chr11	68560972	68561171
chr11	68564968	68565158
chr11	68574129	68574405
chr11	68584534	68585446
chr11	68585938	68586616
chr11	68587917	68588227
chr11	68587574	68587649
chr11	68589712	68589993
chr11	68591256	68591313
chr11	68599179	68599374

chr11	68601532	68601786
chr11	68603816	68604069
chr11	68605244	68605582
chr11	68605722	68606751
chr11	68606940	68607865
chr11	68608059	68609926
chr11	68610187	68611505
chr11	68611627	68611923
chr11	68612054	68612698
chr11	68622204	68622846
chr11	68625357	68625602
chr11	68637737	68638195
chr11	68638414	68638513
chr11	68654206	68654418
chr11	68663007	68663324
chr11	68670920	68672675
chr11	68761128	68761327
chr11	68775604	68775803
chr11	68776478	68777325
chr11	68777470	68777874
chr11	68777991	68778989
chr11	68815857	68815866
chr11	68816006	68817321
chr11	68818968	68819167
chr11	68847478	68848397
chr11	68866550	68866566
chr11	68885602	68885679
chr11	68888790	68888989
chr11	68889810	68890419
chr11	68890543	68891461
chr11	68897413	68897554
chr11	68899283	68899482
chr11	68903843	68903990
chr11	68904814	68905013
chr11	68908164	68908228
chr11	68908375	68908483
chr11	68924598	68924811
chr11	68933257	68933476
chr11	68933826	68934078
chr11	68934277	68934752
chr11	68935290	68935598
chr11	68935737	68935975
chr11	68936201	68936544
chr11	68936609	68936619
chr11	68937018	68937815
chr11	68938366	68938578

chr11	68940948	68941051
chr11	68941266	68942509
chr11	68983513	68983712
chr11	69020001	69020105
chr11	69020177	69020200
chr11	69038034	69038428
chr11	69041600	69042415
chr11	69042561	69042586
chr11	69046006	69046419
chr11	69048156	69048586
chr11	69048751	69049502
chr11	69063961	69066689
chr11	69067909	69068223
chr11	69068647	69068966
chr11	69069288	69070583
chr11	69072455	69072654
chr11	69072833	69073094
chr11	69151241	69151435
chr11	69152699	69153023
chr11	69167400	69167529
chr11	69167885	69168005
chr11	69191444	69192520
chr11	69191345	69191377
chr11	69192690	69193098
chr11	69193431	69193630
chr11	69193950	69194348
chr11	69233252	69233533
chr11	69234915	69236250
chr11	69236955	69237158
chr11	69251412	69251857
chr11	69251991	69252584
chr11	69256122	69257031
chr11	69257324	69259322
chr11	69259445	69259958
chr11	69260570	69260715
chr11	69264763	69264962
chr11	69338990	69339340
chr11	69366689	69367181
chr11	69367717	69367981
chr11	69370208	69370249
chr11	69402009	69402213
chr11	69402433	69402758
chr11	69412571	69412789
chr11	69413144	69413285
chr11	69413672	69414818
chr11	69415415	69415666

chr11	69435560	69435882
chr11	69453626	69453788
chr11	69450829	69453548
chr11	69453920	69459623
chr11	69485308	69485422
chr11	69489167	69490725
chr11	69492966	69493103
chr11	69496847	69497207
chr11	69500947	69501118
chr11	69502723	69502825
chr11	69502992	69503629
chr11	69506038	69506303
chr11	69506465	69506468
chr11	69516900	69517333
chr11	69518891	69519090
chr11	69521864	69522139
chr11	69523789	69524196
chr11	69559144	69559275
chr11	69582777	69582965
chr11	69794418	69795494
chr11	69819008	69819050
chr11	69831838	69832138
chr11	69863389	69863406
chr11	69863632	69863874
chr11	69908980	69909498
chr11	69909891	69910121
chr11	69917252	69917378
chr11	69924859	69925176
chr11	69984872	69985098
chr11	69985844	69986779
chr11	69998152	69998368
chr11	70003184	70003206
chr11	70003286	70003460
chr11	70006479	70007062
chr11	70018704	70018925
chr11	70042153	70042352
chr11	70045904	70046095
chr11	70046525	70046849
chr11	70048357	70048780
chr11	70049013	70049224
chr11	70049392	70050855
chr11	70050972	70051390
chr11	70054322	70054521
chr11	70055332	70055531
chr11	70066576	70066703
chr11	70067093	70067148

chr11	70078448	70078865
chr11	70079310	70079625
chr11	70091368	70091460
chr11	70098833	70099032
chr11	70102745	70102813
chr11	70115914	70116506
chr11	70116803	70117939
chr11	70136980	70137339
chr11	70157901	70158055
chr11	70169968	70170113
chr11	70170524	70170580
chr11	70194695	70194884
chr11	70204344	70204412
chr11	70226493	70226760
chr11	70238991	70239227
chr11	70242451	70242797
chr11	70243628	70246852
chr11	70247059	70247136
chr11	70247453	70247844
chr11	70248818	70248859
chr11	70248966	70249141
chr11	70264788	70264992
chr11	70272539	70273032
chr11	70951491	70951560
chr11	70951726	70952345
chr11	70952583	70952759
chr11	70962450	70962939
chr11	70963322	70963699
chr11	70998526	70998602
chr11	71010070	71010395
chr11	71010761	71011210
chr11	71158046	71160490
chr11	71160611	71161115
chr11	71163640	71164131
chr11	71164256	71165503
chr11	71198781	71199053
chr11	71199368	71199930
chr11	71200780	71201082
chr11	71291337	71291499
chr11	71350376	71350927
chr11	71456968	71457217
chr11	71497178	71497980
chr11	71498129	71499307
chr11	71499497	71499757
chr11	71505140	71505413
chr11	71507980	71508314

chr11	71524469	71525170
chr11	71631412	71631502
chr11	71638257	71638623
chr11	71638855	71640593
chr11	71656104	71656658
chr11	71657334	71657682
chr11	71659396	71659582
chr11	71684545	71684563
chr11	71734535	71734693
chr11	71735909	71737039
chr11	71737111	71737174
chr11	71741954	71742263
chr11	71745611	71745819
chr11	71749199	71749531
chr11	71750577	71750729
chr11	71751427	71754166
chr11	71755741	71755940
chr11	71766949	71767270
chr11	71769693	71770562
chr11	71771266	71771618
chr11	71771793	71772139
chr11	71772276	71772345
chr11	71772642	71772684
chr11	71772840	71773085
chr11	71780892	71781386
chr11	71783247	71784207
chr11	71785569	71785991
chr11	71790217	71791703
chr11	71791901	71792347
chr11	71793243	71793309
chr11	71793825	71793871
chr11	71795110	71795511
chr11	71795608	71796032
chr11	71799499	71799571
chr11	71799688	71799769
chr11	71802595	71802713
chr11	71804180	71804203
chr11	71804312	71804420
chr11	71804488	71804725
chr11	71805894	71806009
chr11	71806608	71806682
chr11	71807161	71807191
chr11	71807328	71807527
chr11	71810102	71810444
chr11	71811443	71811540
chr11	71810760	71811270

chr11	71812085	71812463
chr11	71813213	71814402
chr11	71814613	71815418
chr11	71817900	71817951
chr11	71822086	71822514
chr11	71822651	71824661
chr11	71825246	71825503
chr11	71825961	71826014
chr11	71829288	71829522
chr11	71830815	71831014
chr11	71845699	71845936
chr11	71848997	71849114
chr11	71849308	71849896
chr11	71850743	71850833
chr11	71850989	71851611
chr11	71852114	71852632
chr11	71852892	71853870
chr11	71853978	71854277
chr11	71854963	71855633
chr11	71855935	71856799
chr11	71856959	71857484
chr11	71862337	71862536
chr11	71863383	71863864
chr11	71869357	71869556
chr11	71880146	71880392
chr11	71880539	71880684
chr11	71880771	71881164
chr11	71881519	71882003
chr11	71882654	71882683
chr11	71882982	71883425
chr11	71884596	71884634
chr11	71884710	71886216
chr11	71887590	71888231
chr11	71888437	71888480
chr11	71888996	71890097
chr11	71890483	71891185
chr11	71893468	71893745
chr11	71894140	71894680
chr11	71891432	71893178
chr11	71895729	71896054
chr11	71897585	71897847
chr11	71899807	71899924
chr11	71903892	71903988
chr11	71906256	71906527
chr11	71909327	71909594
chr11	71910329	71911265

chr11	71914407	71914601
chr11	71933182	71933415
chr11	71933488	71936394
chr11	71936514	71937062
chr11	71937385	71937658
chr11	71950442	71951531
chr11	71951742	71952280
chr11	71980864	71981135
chr11	71984008	71984212
chr11	71984347	71984424
chr11	72099387	72099556
chr11	72125067	72125266
chr11	72132129	72132319
chr11	72133035	72133116
chr11	72144789	72145658
chr11	72145784	72146128
chr11	72146224	72146466
chr11	72150822	72150845
chr11	72151020	72151577
chr11	72159082	72159164
chr11	72182688	72182796
chr11	72193466	72193750
chr11	72194494	72195228
chr11	72246167	72246366
chr11	72260406	72260996
chr11	72263914	72264158
chr11	72278465	72278724
chr11	72279861	72280416
chr11	72281003	72281720
chr11	72283031	72283136
chr11	72284353	72284474
chr11	72295297	72296750
chr11	72352587	72352788
chr11	72353019	72355325
chr11	72356100	72356131
chr11	72355636	72355809
chr11	72372749	72372948
chr11	72410615	72410855
chr11	72411386	72412206
chr11	72426619	72426870
chr11	72430118	72430322
chr11	72434287	72435247
chr11	72431497	72434222
chr11	72436877	72437419
chr11	72437547	72437751
chr11	72437856	72438277

chr11	72438666	72438865
chr11	72440409	72440614
chr11	72442101	72442256
chr11	72443369	72443725
chr11	72443843	72444183
chr11	72445032	72445289
chr11	72446123	72446503
chr11	72446620	72449166
chr11	72449897	72450225
chr11	72450692	72450939
chr11	72451633	72454273
chr11	72451410	72451563
chr11	72455757	72456141
chr11	72460155	72460806
chr11	72462032	72463412
chr11	72463600	72464240
chr11	72469128	72469355
chr11	72472489	72472576
chr11	72484581	72485033
chr11	72491246	72491445
chr11	72491655	72493370
chr11	72496223	72496699
chr11	72497731	72497932
chr11	72499646	72499863
chr11	72503802	72504212
chr11	72504436	72504701
chr11	72504846	72506487
chr11	72508528	72508727
chr11	72516727	72516939
chr11	72518229	72519085
chr11	72521636	72521733
chr11	72522761	72523312
chr11	72523657	72526645
chr11	72526776	72527308
chr11	72527421	72527620
chr11	72531735	72531934
chr11	72532328	72533335
chr11	72538432	72539186
chr11	72542288	72542609
chr11	72542039	72542225
chr11	72542830	72543480
chr11	72547044	72547054
chr11	72549638	72549844
chr11	72549995	72550319
chr11	72551793	72552247
chr11	72662269	72662360

chr11	72662605	72662668
chr11	72784290	72784332
chr11	72798672	72798825
chr11	72798978	72799236
chr11	72824416	72825037
chr11	72825259	72826296
chr11	72843594	72843834
chr11	72851395	72854667
chr11	72855435	72855467
chr11	72863169	72863479
chr11	72864015	72864214
chr11	72864359	72864589
chr11	72883083	72883247
chr11	72883494	72883529
chr11	72886103	72886363
chr11	72888509	72888902
chr11	72896116	72897875
chr11	72897992	72898662
chr11	72898762	72899047
chr11	72899362	72900035
chr11	72901604	72901650
chr11	72902790	72902794
chr11	72902860	72903434
chr11	72903599	72904828
chr11	72905287	72907134
chr11	72907404	72907405
chr11	72907626	72907780
chr11	72908985	72909274
chr11	72908620	72908895
chr11	72909854	72910162
chr11	72918522	72918560
chr11	72928839	72930143
chr11	72938172	72938197
chr11	72938516	72939035
chr11	72939939	72940180
chr11	72947906	72948735
chr11	72949094	72949293
chr11	72949796	72950078
chr11	72951169	72951564
chr11	72953362	72953802
chr11	72953966	72954355
chr11	72967007	72967247
chr11	72968255	72969387
chr11	72973815	72973991
chr11	72974144	72974298
chr11	73000112	73000669

chr11	73000922	73001241
chr11	73005951	73006117
chr11	73016709	73016739
chr11	73018037	73018396
chr11	73018556	73021146
chr11	73021257	73021456
chr11	73024499	73024574
chr11	73024367	73024418
chr11	73025824	73026023
chr11	73036780	73037015
chr11	73045326	73045982
chr11	73046228	73046548
chr11	73048396	73048598
chr11	73053886	73054171
chr11	73062588	73062840
chr11	73087392	73089149
chr11	73089891	73090143
chr11	73093996	73094242
chr11	73094460	73094728
chr11	73096768	73097082
chr11	73097825	73099061
chr11	73099256	73099602
chr11	73099755	73100721
chr11	73104103	73104526
chr11	73111051	73111379
chr11	73189829	73189880
chr11	73281353	73281905
chr11	73282518	73282580
chr11	73308296	73309974
chr11	73336889	73337292
chr11	73372200	73372803
chr11	73407868	73408017
chr11	73425742	73425944
chr11	73454079	73454268
chr11	73460935	73460997
chr11	73461902	73462248
chr11	73464239	73464560
chr11	73468650	73469079
chr11	73470771	73472198
chr11	73472353	73472815
chr11	73481985	73482178
chr11	73488950	73489306
chr11	73490122	73491093
chr11	73495237	73495583
chr11	73495719	73495790
chr11	73498462	73499952

chr11	73518126	73518251
chr11	73525514	73525675
chr11	73526693	73526869
chr11	73558794	73559061
chr11	73560072	73560220
chr11	73577578	73577988
chr11	73578916	73579134
chr11	73582409	73582608
chr11	73586908	73587303
chr11	73587450	73588580
chr11	73589873	73590072
chr11	73591709	73591875
chr11	73600401	73600496
chr11	73610196	73610329
chr11	73613615	73613677
chr11	73667450	73667814
chr11	73675251	73675450
chr11	73675697	73675941
chr11	73684745	73685087
chr11	73691717	73691916
chr11	73692816	73695025
chr11	73736535	73736812
chr11	73737804	73738032
chr11	73738192	73738475
chr11	73748609	73748750
chr11	73768737	73769013
chr11	73843183	73843441
chr11	73846068	73846849
chr11	73849515	73850068
chr11	73858196	73858405
chr11	73865656	73865711
chr11	73865936	73866113
chr11	73881037	73881272
chr11	73881690	73882180
chr11	73882409	73882611
chr11	73882918	73883491
chr11	73891940	73892191
chr11	73893886	73894085
chr11	73989649	73989789
chr11	73999173	73999586
chr11	74022084	74022889
chr11	74030189	74030643
chr11	74041515	74041717
chr11	74100103	74100356
chr11	74108416	74108621
chr11	74108954	74109432

chr11	74109580	74110332
chr11	74124725	74124806
chr11	74129766	74129899
chr11	74164174	74164288
chr11	74203404	74203631
chr11	74203698	74205458
chr11	74215735	74216868
chr11	74218944	74219205
chr11	74243829	74243974
chr11	74264735	74265170
chr11	74280904	74280959
chr11	74281513	74281575
chr11	74294102	74294157
chr11	74302135	74302209
chr11	74303299	74304058
chr11	74304185	74304193
chr11	74304379	74304578
chr11	74323964	74324076
chr11	74342563	74343087
chr11	74345478	74345809
chr11	74347657	74348068
chr11	74381746	74382045
chr11	74382391	74382701
chr11	74383065	74383162
chr11	74383444	74385310
chr11	74394343	74394748
chr11	74402829	74403029
chr11	74406011	74406210
chr11	74406912	74406939
chr11	74407093	74407305
chr11	74407495	74409040
chr11	74412309	74412517
chr11	74413197	74413555
chr11	74422808	74423007
chr11	74436374	74436494
chr11	74459239	74459522
chr11	74459597	74460922
chr11	74461536	74462242
chr11	74465474	74466399
chr11	74494427	74494606
chr11	74494805	74494862
chr11	74532118	74532195
chr11	74644132	74644285
chr11	74650901	74650944
chr11	74652649	74652763
chr11	74653510	74654077

chr11	74654327	74654976
chr11	74659550	74660758
chr11	74660863	74661207
chr11	74663926	74664043
chr11	74677701	74678110
chr11	74699621	74700908
chr11	74742921	74743140
chr11	74865108	74865552
chr11	74938170	74938363
chr11	74952085	74953068
chr11	74959552	74959751
chr11	74959999	74960239
chr11	74985688	74985933
chr11	75002723	75002922
chr11	75024208	75024606
chr11	75037613	75038017
chr11	75039992	75040383
chr11	75048187	75048426
chr11	75051552	75052479
chr11	75062377	75063968
chr11	75087081	75087375
chr11	75097929	75098022
chr11	75108703	75108762
chr11	75110183	75110289
chr11	75110513	75111179
chr11	75111618	75112475
chr11	75113052	75113413
chr11	75113995	75114525
chr11	75117274	75117480
chr11	75118207	75118633
chr11	75119232	75119435
chr11	75119671	75119852
chr11	75127585	75127793
chr11	75127990	75128059
chr11	75128303	75128471
chr11	75129931	75130365
chr11	75131494	75131853
chr11	75134059	75134247
chr11	75136916	75137115
chr11	75137473	75137712
chr11	75138726	75139319
chr11	75139556	75142602
chr11	75164289	75164611
chr11	75201318	75201819
chr11	75202957	75203770
chr11	75211508	75211828

chr11	75212505	75212704
chr11	75236043	75237084
chr11	75237232	75238148
chr11	75247692	75248378
chr11	75261084	75261283
chr11	75271283	75271702
chr11	75272262	75272582
chr11	75272688	75275843
chr11	75275945	75276716
chr11	75278469	75278668
chr11	75279085	75279284
chr11	75287887	75288091
chr11	75288196	75288226
chr11	75291899	75292094
chr11	75293276	75293475
chr11	75293740	75295015
chr11	75311494	75311686
chr11	75314083	75314230
chr11	75314731	75315811
chr11	75316771	75317088
chr11	75364765	75364958
chr11	75367374	75367482
chr11	75378324	75378982
chr11	75384884	75385143
chr11	75416233	75416402
chr11	75417489	75417755
chr11	75434659	75434858
chr11	75470174	75470178
chr11	75472000	75472248
chr11	75473076	75473303
chr11	75473884	75474023
chr11	75474185	75474556
chr11	75478054	75478416
chr11	75478745	75480491
chr11	75482395	75482598
chr11	75482966	75483343
chr11	75506072	75506347
chr11	75506602	75507140
chr11	75510566	75510764
chr11	75523385	75523404
chr11	75523797	75524056
chr11	75524468	75524553
chr11	75524799	75525849
chr11	75526075	75527926
chr11	75530973	75531182
chr11	75531346	75532110

chr11	75565041	75565230
chr11	75677518	75677703
chr11	75718766	75719243
chr11	75719564	75719573
chr11	75719695	75719889
chr11	75862997	75863211
chr11	75863458	75863822
chr11	75872537	75872736
chr11	75917252	75918690
chr11	75919098	75922031
chr11	75945511	75946911
chr11	75947028	75947656
chr11	75981307	75981553
chr11	75997827	75998036
chr11	76082134	76082620
chr11	76084147	76084322
chr11	76086478	76086551
chr11	76091227	76092208
chr11	76092428	76092832
chr11	76155325	76155708
chr11	76155811	76156872
chr11	76157140	76157178
chr11	76288820	76289051
chr11	76340199	76341015
chr11	76341089	76341372
chr11	76354903	76355283
chr11	76381114	76381352
chr11	76391842	76392063
chr11	76401975	76402509
chr11	76404044	76404342
chr11	76405640	76405656
chr11	76405955	76406154
chr11	76411882	76412116
chr11	76417419	76417712
chr11	76474433	76474666
chr11	76474932	76475352
chr11	76475604	76475642
chr11	76475834	76475995
chr11	76478865	76479658
chr11	76479828	76480515
chr11	76480677	76480876
chr11	76481093	76482703
chr11	76483070	76483374
chr11	76484167	76484323
chr11	76484547	76485014
chr11	76485127	76485373

chr11	76492559	76495612
chr11	76495966	76496559
chr11	76498660	76499285
chr11	76499390	76499877
chr11	76500010	76500456
chr11	76500851	76501045
chr11	76503243	76503953
chr11	76504867	76505320
chr11	76510660	76510759
chr11	76510833	76511079
chr11	76515714	76515884
chr11	76515981	76516314
chr11	76518625	76518989
chr11	76519053	76519125
chr11	76521519	76521623
chr11	76534417	76536784
chr11	76541571	76541679
chr11	76547082	76547298
chr11	76571259	76571260
chr11	76571474	76571685
chr11	76571858	76572860
chr11	76573051	76573069
chr11	76595876	76596075
chr11	76596347	76596387
chr11	76615180	76615387
chr11	76630079	76630164
chr11	76630399	76630469
chr11	76645447	76645659
chr11	76731941	76732083
chr11	76750682	76751118
chr11	76757246	76757531
chr11	76758211	76758624
chr11	76776756	76777297
chr11	76777426	76778361
chr11	76784150	76784368
chr11	76802096	76802295
chr11	76858650	76858849
chr11	76860587	76860786
chr11	76879579	76879817
chr11	76902035	76902477
chr11	76902605	76903253
chr11	76907124	76907239
chr11	76955350	76955901
chr11	76960544	76960564
chr11	76960772	76960838
chr11	76961906	76962045

chr11	76990411	76990502
chr11	76999042	76999181
chr11	76999269	76999308
chr11	77010475	77011412
chr11	77025067	77025117
chr11	77061628	77061939
chr11	77062287	77062722
chr11	77072457	77072579
chr11	77118642	77118701
chr11	77118907	77119230
chr11	77122602	77122968
chr11	77123148	77123742
chr11	77182947	77183174
chr11	77183364	77183600
chr11	77184026	77185432
chr11	77185555	77185687
chr11	77187124	77187348
chr11	77259401	77259528
chr11	77259645	77259702
chr11	77269639	77269853
chr11	77270814	77272423
chr11	77297447	77297696
chr11	77299531	77299596
chr11	77300321	77301002
chr11	77303920	77304165
chr11	77313384	77313812
chr11	77337290	77337710
chr11	77344864	77345861
chr11	77349331	77349599
chr11	77348054	77349135
chr11	77353603	77353618
chr11	77355447	77355893
chr11	77378848	77379135
chr11	77416566	77416998
chr11	77498731	77499187
chr11	77499308	77499311
chr11	77499834	77500068
chr11	77510896	77510993
chr11	77511207	77511263
chr11	77511596	77511905
chr11	77512683	77512882
chr11	77514099	77515034
chr11	77525126	77525307
chr11	77530313	77530461
chr11	77530614	77533032
chr11	77568967	77569175

chr11	77585651	77585850
chr11	77589177	77589366
chr11	77589546	77589710
chr11	77704848	77704882
chr11	77704987	77705283
chr11	77705524	77705633
chr11	77706077	77706349
chr11	77722960	77723405
chr11	77733630	77734570
chr11	77738219	77738456
chr11	77739375	77740372
chr11	77741646	77741926
chr11	77743787	77743959
chr11	77744554	77744793
chr11	77748602	77748825
chr11	77749633	77749970
chr11	77750318	77751835
chr11	77751958	77752182
chr11	77756854	77757340
chr11	77758825	77758863
chr11	77760251	77760450
chr11	77762334	77763185
chr11	77763546	77763703
chr11	77763869	77763926
chr11	77770761	77771691
chr11	77772642	77773114
chr11	77773518	77773762
chr11	77786708	77786907
chr11	77789822	77790221
chr11	77790310	77790842
chr11	77791022	77791199
chr11	77812406	77812458
chr11	77815908	77816023
chr11	77849482	77850639
chr11	77850749	77851369
chr11	77863403	77863506
chr11	77875817	77876017
chr11	77889180	77889401
chr11	77890305	77890577
chr11	77892017	77892810
chr11	77896518	77897498
chr11	77898203	77900788
chr11	77901063	77901308
chr11	77902521	77902720
chr11	77946083	77946229
chr11	77989053	77989221

chr11	78117491	78117857
chr11	78127743	78127993
chr11	78128137	78129825
chr11	78130576	78131223
chr11	78143479	78143650
chr11	78156550	78156584
chr11	78171723	78171818
chr11	78186291	78186318
chr11	78231402	78231601
chr11	78231860	78232091
chr11	78236970	78237102
chr11	78273388	78273703
chr11	78275507	78275584
chr11	78278188	78278337
chr11	78284348	78284659
chr11	78284947	78285204
chr11	78285279	78286578
chr11	78307515	78308557
chr11	78409988	78410030
chr11	78507975	78508123
chr11	78509874	78510124
chr11	78512675	78512907
chr11	78549552	78549879
chr11	78551982	78552074
chr11	78598125	78598491
chr11	78671658	78672276
chr11	78797369	78797546
chr11	78803801	78804107
chr11	78804480	78804585
chr11	78866953	78867170
chr11	78868552	78868781
chr11	78908446	78908452
chr11	78954823	78955092
chr11	78955350	78955617
chr11	78956045	78956358
chr11	78961048	78961117
chr11	79086786	79087648
chr11	79115404	79115425
chr11	79115609	79115691
chr11	79125957	79126561
chr11	79126693	79126723
chr11	79128661	79128891
chr11	79129982	79130193
chr11	79130408	79130603
chr11	79147999	79152410
chr11	79172208	79172504

chr11	79218709	79218823
chr11	79613643	79613843
chr11	79652247	79652379
chr11	79735204	79735260
chr11	79794426	79795298
chr11	79814442	79814533
chr11	79814917	79814932
chr11	79866801	79866860
chr11	80026620	80026723
chr11	80126036	80126246
chr11	80153209	80153224
chr11	80304935	80304986
chr11	80410831	80411050
chr11	80411239	80411313
chr11	80669511	80669611
chr11	80691240	80691445
chr11	80716649	80717089
chr11	82393489	82393610
chr11	82429282	82429693
chr11	82442040	82442192
chr11	82442815	82443112
chr11	82443386	82445327
chr11	82448995	82449095
chr11	82458377	82458998
chr11	82482598	82483278
chr11	82487016	82487101
chr11	82554408	82554873
chr11	82569386	82570254
chr11	82575198	82575722
chr11	82578568	82578873
chr11	82580670	82580676
chr11	82580782	82581071
chr11	82582583	82582588
chr11	82610851	82612691
chr11	82612823	82613600
chr11	82638527	82638677
chr11	82689487	82689767
chr11	82691284	82691787
chr11	82721078	82721441
chr11	82724305	82724409
chr11	82745236	82745865
chr11	82754045	82754069
chr11	82755954	82756528
chr11	82762687	82763784
chr11	82764919	82765052
chr11	82765994	82766026

chr11	82773961	82774377
chr11	82781513	82783572
chr11	82783685	82784135
chr11	82803751	82803893
chr11	82862542	82862708
chr11	82862917	82863051
chr11	82866784	82866983
chr11	82867279	82867543
chr11	82868110	82869598
chr11	82871108	82871930
chr11	82882011	82882241
chr11	82884910	82885213
chr11	82885322	82885372
chr11	82886973	82887007
chr11	82887135	82887187
chr11	82904284	82904689
chr11	82904928	82905986
chr11	82918113	82918276
chr11	82961059	82961409
chr11	82996489	82997426
chr11	82997530	82997747
chr11	83284189	83284260
chr11	83285574	83285874
chr11	83434937	83434984
chr11	83435105	83435373
chr11	83539228	83539323
chr11	85338462	85339373
chr11	85339717	85339822
chr11	85358145	85359330
chr11	85360046	85360245
chr11	85364747	85364861
chr11	85366490	85366739
chr11	85374775	85376050
chr11	85376220	85376392
chr11	85453962	85454407
chr11	85518179	85518367
chr11	85522328	85522601
chr11	85522804	85522986
chr11	85523623	85523822
chr11	85555361	85555578
chr11	85555736	85556463
chr11	85565567	85565984
chr11	85566153	85566410
chr11	85586531	85586772
chr11	85595937	85596078
chr11	85658462	85658591

chr11	85658944	85659081
chr11	85659331	85659422
chr11	85659826	85660353
chr11	85664542	85664600
chr11	85672897	85673096
chr11	85673320	85673452
chr11	85673797	85674113
chr11	85681808	85681841
chr11	85719606	85719680
chr11	85719793	85719851
chr11	85747904	85748182
chr11	85752952	85753345
chr11	85754009	85754235
chr11	85757630	85757898
chr11	85758297	85758351
chr11	85758616	85758680
chr11	85759141	85759391
chr11	85775265	85776249
chr11	85778320	85778666
chr11	85778960	85781185
chr11	85810289	85810488
chr11	85827015	85827410
chr11	85827580	85827777
chr11	85828069	85828344
chr11	85862153	85863003
chr11	85863362	85863733
chr11	85866188	85866723
chr11	85885007	85885371
chr11	85885989	85886293
chr11	85887386	85887489
chr11	85887943	85888027
chr11	85894008	85894335
chr11	85894775	85894809
chr11	85904540	85904601
chr11	85904978	85904982
chr11	85913555	85914020
chr11	85922724	85922823
chr11	85955112	85956679
chr11	85960831	85961040
chr11	86000535	86000734
chr11	86003203	86003259
chr11	86012945	86013014
chr11	86013221	86013844
chr11	86038396	86038580
chr11	86046428	86046443
chr11	86046578	86046807

chr11	86052472	86052776
chr11	86071734	86072215
chr11	86082198	86082682
chr11	86083698	86083796
chr11	86083929	86083970
chr11	86125092	86125211
chr11	86125349	86125524
chr11	86127857	86128378
chr11	86129975	86130377
chr11	86167103	86167267
chr11	86167587	86167624
chr11	86171477	86171763
chr11	86195448	86195810
chr11	86208399	86208441
chr11	86208598	86208886
chr11	86227266	86227276
chr11	86232875	86233224
chr11	86235023	86235373
chr11	86235536	86236076
chr11	86259729	86259741
chr11	86262626	86262812
chr11	86263353	86263461
chr11	86269175	86269374
chr11	86270344	86270765
chr11	86278362	86278458
chr11	86303214	86303408
chr11	86303487	86303686
chr11	86303766	86303808
chr11	86319362	86319574
chr11	86334224	86334380
chr11	86334824	86335123
chr11	86343636	86344282
chr11	86382523	86382584
chr11	86382733	86383552
chr11	86403323	86403400
chr11	86403511	86403711
chr11	86407018	86407809
chr11	86409758	86410001
chr11	86410105	86410449
chr11	86430790	86431442
chr11	86443797	86443836
chr11	86446605	86447631
chr11	86448996	86449262
chr11	86451320	86452534
chr11	86452852	86453231
chr11	86454175	86455169

chr11	86455588	86455844
chr11	86495782	86495829
chr11	86507002	86507178
chr11	86507282	86507424
chr11	86510021	86510419
chr11	86511006	86512891
chr11	86513469	86514210
chr11	86514828	86515027
chr11	86517082	86517317
chr11	86529762	86529798
chr11	86581309	86581413
chr11	86626878	86627132
chr11	86627358	86627537
chr11	86665375	86665614
chr11	86665922	86667877
chr11	86694588	86694862
chr11	86702604	86702888
chr11	86715188	86715723
chr11	86740478	86740617
chr11	86740763	86740872
chr11	86748066	86749614
chr11	86808869	86808925
chr11	86884601	86884862
chr11	86887247	86887391
chr11	86977864	86977883
chr11	86992519	86992574
chr11	86992792	86992864
chr11	87063999	87064262
chr11	87149244	87149301
chr11	87294787	87294930
chr11	87295114	87295229
chr11	87315483	87315579
chr11	87562126	87562391
chr11	87562551	87562669
chr11	87637124	87637491
chr11	87637878	87637990
chr11	87783966	87784034
chr11	87784259	87784286
chr11	87784416	87784458
chr11	87784818	87785408
chr11	87835697	87836245
chr11	87836816	87836884
chr11	87850470	87850699
chr11	87902613	87903462
chr11	87905185	87905520
chr11	87907184	87907550

chr11	87907719	87908649
chr11	87908763	87909319
chr11	87911717	87911970
chr11	87912250	87912791
chr11	87913140	87913712
chr11	87914925	87915270
chr11	87916469	87916629
chr11	88049115	88049119
chr11	88049300	88049517
chr11	88053424	88053501
chr11	88059401	88059479
chr11	88068462	88068879
chr11	88069734	88070151
chr11	88070441	88071916
chr11	88086556	88086647
chr11	88086802	88086903
chr11	88096717	88097189
chr11	88131531	88131935
chr11	88138148	88138611
chr11	88143738	88144140
chr11	88151772	88152136
chr11	88152989	88154036
chr11	88154717	88155073
chr11	88164992	88165177
chr11	88167306	88167394
chr11	88167487	88168130
chr11	88213549	88213637
chr11	88241816	88242895
chr11	88248410	88248549
chr11	88253599	88253664
chr11	88269045	88269511
chr11	88271285	88271612
chr11	88277441	88277600
chr11	88288057	88288347
chr11	88288497	88288719
chr11	88663726	88663883
chr11	88664054	88664178
chr11	88682174	88682254
chr11	88715927	88716296
chr11	89113459	89113760
chr11	89114179	89114295
chr11	89114404	89114823
chr11	89224507	89224721
chr11	89350164	89350244
chr11	89409469	89409612
chr11	89868386	89868439

chr11	89953554	89953922
chr11	89955492	89956777
chr11	89984134	89984312
chr11	90020958	90021051
chr11	90077735	90077934
chr11	90202082	90202185
chr11	90202362	90202459
chr11	90209856	90209968
chr11	90455768	90456100
chr11	90480392	90480627
chr11	90536590	90536968
chr11	90538408	90538708
chr11	90542713	90543126
chr11	90573274	90573596
chr11	90578327	90578609
chr11	90667683	90667895
chr11	90814925	90815148
chr11	92733644	92734119
chr11	92738666	92738889
chr11	92753265	92754235
chr11	92787993	92788514
chr11	92903146	92903174
chr11	92930446	92931354
chr11	93063614	93063843
chr11	93103747	93103805
chr11	93275896	93276348
chr11	93276778	93277116
chr11	93378601	93378731
chr11	93394450	93395426
chr11	93419058	93419348
chr11	93453364	93454036
chr11	93462230	93462568
chr11	93467884	93468194
chr11	93471423	93471710
chr11	93471880	93472106
chr11	93473150	93473732
chr11	93473890	93474690
chr11	93474835	93475430
chr11	93516937	93518524
chr11	93518918	93519245
chr11	93571644	93571792
chr11	93583214	93583738
chr11	93840664	93840863
chr11	93845738	93845844
chr11	93850395	93850594
chr11	93851200	93851279

chr11	93860802	93860991
chr11	93865239	93865821
chr11	93861314	93864921
chr11	93866918	93866998
chr11	93867828	93868766
chr11	93869133	93869889
chr11	93870063	93870288
chr11	93872701	93873000
chr11	93874500	93874747
chr11	93874989	93875072
chr11	93883679	93883881
chr11	93885610	93885843
chr11	93971765	93971976
chr11	94075630	94075829
chr11	94182520	94182702
chr11	94183028	94183099
chr11	94188978	94189256
chr11	94226423	94227001
chr11	94227164	94227686
chr11	94237924	94237936
chr11	94276741	94278796
chr11	94279321	94279446
chr11	94335917	94335997
chr11	94353574	94353597
chr11	94356565	94356729
chr11	94372114	94372309
chr11	94411402	94411777
chr11	94411903	94412207
chr11	94439088	94440497
chr11	94461719	94461798
chr11	94462006	94462041
chr11	94464956	94465394
chr11	94465588	94466199
chr11	94467697	94468184
chr11	94469279	94469548
chr11	94470015	94470061
chr11	94470415	94470919
chr11	94471142	94471610
chr11	94472385	94472653
chr11	94473047	94474928
chr11	94475935	94476201
chr11	94477540	94477750
chr11	94479633	94481410
chr11	94482385	94482599
chr11	94482722	94483086
chr11	94483739	94483944

chr11	94484351	94484727
chr11	94495345	94495476
chr11	94499752	94501423
chr11	94503221	94503601
chr11	94501562	94503075
chr11	94507604	94507818
chr11	94509326	94510199
chr11	94513249	94513271
chr11	94513394	94513567
chr11	94514860	94515127
chr11	94518739	94518965
chr11	94521143	94521343
chr11	94522546	94522672
chr11	94526161	94526276
chr11	94527955	94528292
chr11	94564639	94564924
chr11	94565760	94565845
chr11	94578010	94578212
chr11	94578658	94578806
chr11	94579118	94579511
chr11	94581466	94581704
chr11	94585093	94585539
chr11	94585732	94585799
chr11	94587360	94587432
chr11	94601807	94602006
chr11	94606859	94607048
chr11	94609642	94610416
chr11	94630469	94630509
chr11	94631689	94631888
chr11	94651332	94651422
chr11	94660338	94660389
chr11	94706279	94707163
chr11	94768827	94768880
chr11	94798488	94798687
chr11	94798856	94798913
chr11	94799865	94801410
chr11	94801646	94801873
chr11	94802052	94802166
chr11	94802818	94802951
chr11	94803140	94803202
chr11	94809625	94809841
chr11	94822482	94823874
chr11	94833422	94833552
chr11	94837186	94837814
chr11	94840267	94841758
chr11	94867544	94867712

chr11	94867905	94868178
chr11	94869419	94869618
chr11	94882774	94884720
chr11	94884846	94885146
chr11	94885417	94885570
chr11	94886027	94886123
chr11	94886340	94886355
chr11	94961423	94961760
chr11	94963164	94963401
chr11	94963669	94964379
chr11	94964585	94965008
chr11	94965321	94965538
chr11	94965709	94965818
chr11	94978160	94978265
chr11	94978624	94978720
chr11	95013287	95013362
chr11	95070293	95070945
chr11	95078655	95078797
chr11	95092475	95092606
chr11	95093030	95093229
chr11	95096638	95096661
chr11	95097798	95098687
chr11	95177455	95178127
chr11	95218808	95218944
chr11	95232328	95232437
chr11	95232661	95233084
chr11	95245305	95246256
chr11	95247532	95247601
chr11	95247710	95247945
chr11	95249894	95250372
chr11	95254067	95254446
chr11	95254766	95254807
chr11	95255937	95256156
chr11	95256467	95256524
chr11	95438670	95438798
chr11	95500676	95500875
chr11	95500997	95501321
chr11	95521990	95523022
chr11	95523207	95524535
chr11	95524713	95524946
chr11	95560858	95560947
chr11	95564494	95564808
chr11	95569142	95569345
chr11	95617729	95617891
chr11	95656818	95657684
chr11	95658154	95658556

chr11	95766223	95766310
chr11	95770716	95771002
chr11	95772222	95772421
chr11	95788138	95788358
chr11	95795220	95795550
chr11	95829016	95829247
chr11	95831346	95831674
chr11	95836388	95836442
chr11	95842691	95842843
chr11	95855302	95856441
chr11	95857346	95857513
chr11	95882195	95882343
chr11	95888745	95889858
chr11	95895975	95896351
chr11	95901819	95902218
chr11	95916237	95916394
chr11	95916521	95916840
chr11	95920893	95921300
chr11	95921597	95921713
chr11	95940789	95941248
chr11	95953403	95953565
chr11	95953714	95953803
chr11	95960393	95960814
chr11	95961489	95961834
chr11	95961950	95962141
chr11	95970884	95971098
chr11	95976270	95977103
chr11	95979989	95980269
chr11	95987918	95988122
chr11	95992569	95992851
chr11	95996451	95996979
chr11	95999387	95999507
chr11	96005736	96006076
chr11	96006158	96006385
chr11	96012630	96012907
chr11	96013010	96013144
chr11	96036206	96036229
chr11	96036437	96036585
chr11	96039994	96041328
chr11	96047454	96047628
chr11	96047746	96047849
chr11	96048759	96048855
chr11	96058070	96058424
chr11	96074680	96075456
chr11	96075741	96076393
chr11	96076676	96076799

chr11	96122384	96123711
chr11	96126978	96127075
chr11	96197269	96197422
chr11	96619683	96619853
chr11	96619965	96620008
chr11	97614184	97614654
chr11	97627895	97627999
chr11	97628088	97628384
chr11	98432081	98432280
chr11	99196505	99196635
chr11	99564772	99565133
chr11	100417863	100417986
chr11	100558045	100558748
chr11	101317292	101317430
chr11	101349289	101349367
chr11	101454021	101454272
chr11	101488958	101489066
chr11	101489460	101489503
chr11	101491033	101491118
chr11	101589178	101589266
chr11	101591949	101592029
chr11	101592224	101592423
chr11	101632027	101632442
chr11	101741105	101741302
chr11	101741572	101741707
chr11	101785094	101786337
chr11	101786450	101787071
chr11	101877549	101877912
chr11	101878213	101878888
chr11	101881494	101881901
chr11	101882205	101882295
chr11	101885045	101885248
chr11	101917904	101918749
chr11	101934969	101935112
chr11	101935344	101935529
chr11	101980108	101980374
chr11	101980514	101983108
chr11	101989707	101989735
chr11	101989844	101989932
chr11	101990688	101991027
chr11	101991248	101991478
chr11	102005577	102005741
chr11	102008822	102009243
chr11	102009855	102010042
chr11	102027932	102028331
chr11	102028951	102029318

chr11	102039210	102039653
chr11	102045939	102045963
chr11	102047522	102048285
chr11	102048679	102049432
chr11	102071213	102071423
chr11	102082501	102082766
chr11	102090402	102090617
chr11	102091572	102091949
chr11	102094325	102095040
chr11	102096664	102097095
chr11	102097441	102097870
chr11	102100371	102100691
chr11	102109344	102109574
chr11	102110813	102110949
chr11	102113113	102113438
chr11	102113529	102113533
chr11	102114975	102115210
chr11	102115354	102116244
chr11	102121179	102121685
chr11	102124130	102124460
chr11	102126859	102127669
chr11	102132776	102132961
chr11	102138305	102139378
chr11	102139622	102140270
chr11	102140541	102141235
chr11	102150471	102150770
chr11	102158642	102158817
chr11	102158943	102159121
chr11	102164659	102165076
chr11	102165519	102166080
chr11	102176599	102177485
chr11	102178360	102178569
chr11	102187708	102188771
chr11	102189215	102189607
chr11	102194660	102195035
chr11	102210423	102210905
chr11	102212508	102212690
chr11	102214640	102214879
chr11	102216608	102216922
chr11	102217286	102219161
chr11	102290200	102290284
chr11	102291501	102291597
chr11	102317654	102317717
chr11	102322351	102324475
chr11	102325598	102325642
chr11	102327087	102327159

chr11	102327348	102327754
chr11	102329146	102329607
chr11	102342252	102342521
chr11	102347026	102347247
chr11	102347532	102347980
chr11	102364939	102365364
chr11	102366391	102366787
chr11	102401524	102401657
chr11	102434814	102435241
chr11	102469745	102471134
chr11	102483597	102483889
chr11	102508796	102508807
chr11	102601541	102602089
chr11	102604250	102604370
chr11	102629229	102629345
chr11	102631026	102631217
chr11	102631415	102631925
chr11	102651129	102651369
chr11	102653906	102654122
chr11	102654255	102654526
chr11	102654984	102655043
chr11	102684397	102684600
chr11	102695317	102695422
chr11	102700495	102700697
chr11	102717278	102717362
chr11	102720000	102720474
chr11	102722119	102722318
chr11	102724694	102725115
chr11	102739365	102739539
chr11	102740335	102740489
chr11	102771083	102771477
chr11	102777309	102777507
chr11	102782416	102782772
chr11	102783008	102783036
chr11	102783165	102783459
chr11	102785404	102785578
chr11	102866961	102867097
chr11	102867224	102867386
chr11	102906906	102907123
chr11	102938149	102938271
chr11	102949492	102949780
chr11	102950594	102950892
chr11	102961131	102961472
chr11	102961796	102961853
chr11	102962036	102963419
chr11	102979811	102980023

chr11	102980262	102981030
chr11	102981188	102981475
chr11	103041601	103041657
chr11	103041813	103041919
chr11	103150542	103150910
chr11	103266281	103266762
chr11	103304283	103304549
chr11	103395777	103396129
chr11	103399868	103399875
chr11	103400158	103400401
chr11	103403179	103403212
chr11	103408754	103408990
chr11	103411786	103411962
chr11	103412144	103412537
chr11	103480011	103480273
chr11	103480459	103480547
chr11	103495942	103496186
chr11	103496311	103496466
chr11	103497428	103497799
chr11	103650177	103650246
chr11	103743708	103743904
chr11	103798622	103798623
chr11	103811331	103811762
chr11	103875821	103875955
chr11	103903826	103903972
chr11	103904166	103904288
chr11	103947201	103947214
chr11	103947438	103947617
chr11	103991474	103991807
chr11	104146738	104146766
chr11	104322754	104323343
chr11	104323552	104323634
chr11	104418748	104418822
chr11	104494186	104494323
chr11	104576663	104576732
chr11	104642055	104642165
chr11	104642588	104642847
chr11	104643631	104643768
chr11	104643987	104644084
chr11	104656160	104656164
chr11	104700839	104700857
chr11	104827245	104827633
chr11	104828025	104828164
chr11	104828272	104828372
chr11	104838173	104838789
chr11	104839084	104839622

chr11	104868027	104868315
chr11	104905661	104906146
chr11	104915896	104916285
chr11	104917644	104918042
chr11	104918215	104918361
chr11	104942159	104942404
chr11	104948037	104948382
chr11	104948561	104948943
chr11	104951071	104951466
chr11	104970857	104971583
chr11	104972011	104972356
chr11	104981070	104981443
chr11	105001188	105001407
chr11	105003432	105003785
chr11	105003861	105004258
chr11	105015656	105015745
chr11	105015948	105015993
chr11	105022482	105023287
chr11	105052301	105052397
chr11	105052571	105052630
chr11	105060348	105060617
chr11	105115127	105115575
chr11	105186033	105186091
chr11	105337319	105337374
chr11	105337706	105337800
chr11	105339049	105339192
chr11	105458714	105458854
chr11	105544073	105544168
chr11	105780946	105781138
chr11	105808115	105808215
chr11	105891882	105892254
chr11	105892353	105892991
chr11	105893305	105893550
chr11	105901982	105902432
chr11	105902571	105902592
chr11	105947259	105948134
chr11	105948502	105949216
chr11	105958600	105958681
chr11	105962201	105962215
chr11	105962440	105962623
chr11	106022663	106022830
chr11	106022972	106023174
chr11	106023577	106023665
chr11	106044696	106045081
chr11	106049393	106049472
chr11	106063820	106064209

chr11	106152143	106152859
chr11	106239310	106239520
chr11	106239796	106239920
chr11	106252671	106252739
chr11	106262514	106262540
chr11	106262803	106262906
chr11	106269659	106269846
chr11	106366146	106366172
chr11	106567795	106568075
chr11	106660454	106660515
chr11	106775677	106775722
chr11	106820592	106820752
chr11	106832277	106832329
chr11	106832521	106832632
chr11	107256290	107256365
chr11	107267993	107268448
chr11	107328277	107329060
chr11	107363863	107363933
chr11	107368883	107369074
chr11	107415926	107416401
chr11	107433473	107433534
chr11	107433860	107433982
chr11	107434461	107434563
chr11	107434693	107434713
chr11	107435686	107436722
chr11	107436823	107436973
chr11	107461391	107462329
chr11	107560799	107560838
chr11	107684384	107684612
chr11	107697882	107698232
chr11	107701589	107702246
chr11	107703371	107703541
chr11	107710768	107712232
chr11	107712746	107713622
chr11	107714662	107714914
chr11	107715022	107715152
chr11	107719158	107719289
chr11	107720146	107720219
chr11	107720469	107720590
chr11	107722740	107723146
chr11	107726866	107730729
chr11	107763595	107763885
chr11	107777684	107778088
chr11	107778247	107778487
chr11	107791683	107791763
chr11	107798676	107798820

chr11	107806039	107806302
chr11	107870882	107871081
chr11	107874678	107874897
chr11	107875267	107875689
chr11	107879277	107879518
chr11	107879692	107880623
chr11	107887227	107887616
chr11	107888301	107888500
chr11	107890586	107890622
chr11	107906201	107906286
chr11	107906395	107906463
chr11	107909852	107910083
chr11	107940753	107940968
chr11	107988197	107988259
chr11	107988729	107988964
chr11	107991495	107992812
chr11	108011576	108011584
chr11	108070178	108070363
chr11	108070594	108071392
chr11	108071629	108071710
chr11	108092460	108093386
chr11	108093757	108095048
chr11	108113876	108114006
chr11	108190189	108190561
chr11	108209641	108209805
chr11	108211617	108211808
chr11	108212026	108212201
chr11	108288291	108288411
chr11	108337716	108338780
chr11	108356494	108356653
chr11	108360638	108360737
chr11	108367956	108369739
chr11	108370975	108371006
chr11	108371160	108371275
chr11	108371449	108371623
chr11	108416641	108416868
chr11	108441993	108442192
chr11	108442762	108442883
chr11	108462911	108464377
chr11	108464601	108465006
chr11	108466406	108466714
chr11	108468512	108468816
chr11	108473942	108474008
chr11	108480370	108480844
chr11	108487068	108487389
chr11	108487526	108487795

chr11	108518914	108519126
chr11	108519298	108519467
chr11	108526035	108526111
chr11	108535431	108535623
chr11	108535790	108536380
chr11	108537168	108537495
chr11	108564614	108564972
chr11	108565286	108565700
chr11	108565829	108565885
chr11	108568613	108568735
chr11	108568845	108568983
chr11	108569404	108570101
chr11	108604199	108604379
chr11	108604619	108604640
chr11	108646245	108646413
chr11	108659303	108659353
chr11	108724589	108724693
chr11	108741054	108741242
chr11	108741393	108741463
chr11	108741548	108742094
chr11	108742284	108742324
chr11	108757440	108757693
chr11	108766564	108766963
chr11	108776977	108777077
chr11	108777312	108777496
chr11	108800036	108800465
chr11	108818562	108818960
chr11	108834527	108834791
chr11	108844269	108844568
chr11	108886003	108886351
chr11	109567322	109567493
chr11	109723932	109724049
chr11	109785088	109785184
chr11	109811427	109811547
chr11	109816937	109817452
chr11	109870704	109870842
chr11	109893256	109893702
chr11	109940465	109940610
chr11	109947068	109947475
chr11	109947599	109947694
chr11	109951997	109952652
chr11	109957450	109957763
chr11	109958014	109958128
chr11	109962622	109965023
chr11	109966300	109966534
chr11	109966665	109966705

chr11	109967424	109967659
chr11	109969132	109969423
chr11	109969527	109969724
chr11	110001527	110001808
chr11	110002150	110002491
chr11	110019477	110019872
chr11	110045639	110045769
chr11	110046707	110047121
chr11	110047633	110047911
chr11	110051782	110051859
chr11	110054893	110056264
chr11	110064900	110065435
chr11	110065746	110066276
chr11	110066547	110068038
chr11	110071107	110071356
chr11	110072098	110072103
chr11	110072340	110072495
chr11	110079676	110080422
chr11	110122467	110122615
chr11	110122856	110122979
chr11	110150919	110151721
chr11	110161073	110161311
chr11	110166384	110170103
chr11	110176256	110176401
chr11	110176585	110176822
chr11	110185134	110185226
chr11	110188373	110188480
chr11	110208231	110208736
chr11	110208925	110208939
chr11	110216305	110216370
chr11	110228225	110228819
chr11	110234030	110234251
chr11	110236854	110237434
chr11	110243815	110244510
chr11	110245619	110245669
chr11	110275125	110275212
chr11	110300503	110301503
chr11	110301716	110302008
chr11	110303830	110304024
chr11	110343243	110343461
chr11	110343582	110343620
chr11	111101356	111102116
chr11	111153912	111154056
chr11	111297418	111297804
chr11	111307258	111307332
chr11	111325749	111325948

chr11	111336352	111336506
chr11	111381059	111381451
chr11	111382722	111382826
chr11	111383079	111383749
chr11	111395040	111395130
chr11	111397217	111397737
chr11	111402349	111402683
chr11	111410904	111411043
chr11	111411186	111412927
chr11	111425621	111425649
chr11	111426196	111426332
chr11	111428476	111428562
chr11	111428759	111428914
chr11	111433125	111433137
chr11	111435367	111435641
chr11	111435778	111436007
chr11	111472403	111473050
chr11	111473168	111474454
chr11	111475140	111475481
chr11	111502295	111502740
chr11	111522012	111522339
chr11	111526711	111526867
chr11	111547575	111547662
chr11	111547791	111547941
chr11	111577708	111577862
chr11	111578007	111578198
chr11	111589120	111589274
chr11	111603195	111603433
chr11	111605683	111606049
chr11	111606155	111606541
chr11	111606704	111607383
chr11	111616051	111616408
chr11	111618223	111618345
chr11	111636307	111637780
chr11	111637860	111638115
chr11	111655497	111655534
chr11	111660806	111661176
chr11	111663765	111664114
chr11	111664477	111664683
chr11	111664950	111664983
chr11	111671144	111671541
chr11	111672077	111672166
chr11	111712429	111712540
chr11	111715972	111716877
chr11	111717015	111717167
chr11	111717454	111717831

chr11	111722394	111722484
chr11	111722624	111722715
chr11	111741413	111742542
chr11	111748187	111750712
chr11	111781451	111781536
chr11	111782088	111782599
chr11	111782710	111783254
chr11	111783819	111784115
chr11	111785019	111785328
chr11	111786509	111786739
chr11	111789123	111790170
chr11	111793559	111793738
chr11	111797221	111797474
chr11	111797881	111798637
chr11	111802423	111802505
chr11	111802710	111803101
chr11	111805069	111805071
chr11	111807399	111808354
chr11	111812273	111812489
chr11	111813155	111813214
chr11	111813531	111813665
chr11	111813830	111813986
chr11	111814968	111815287
chr11	111815409	111815742
chr11	111831522	111831689
chr11	111831925	111832106
chr11	111843252	111843614
chr11	111847540	111848643
chr11	111848937	111849770
chr11	111852987	111853147
chr11	111857306	111857407
chr11	111857575	111857709
chr11	111859432	111860261
chr11	111867311	111867841
chr11	111867986	111868336
chr11	111883482	111883938
chr11	111884364	111884746
chr11	111894993	111896891
chr11	111919257	111919956
chr11	111944782	111945634
chr11	111950567	111950668
chr11	111956734	111958060
chr11	111976668	111976916
chr11	111982472	111982631
chr11	111984323	111984492
chr11	111988222	111988359

chr11	111990031	111991960
chr11	111993975	111994078
chr11	111996374	111996868
chr11	111999033	111999769
chr11	112002130	112002878
chr11	112027871	112028574
chr11	112034570	112035081
chr11	112037171	112037743
chr11	112033105	112034195
chr11	112043903	112044244
chr11	112045828	112045948
chr11	112046212	112046434
chr11	112065698	112066413
chr11	112066577	112067109
chr11	112084557	112084685
chr11	112084891	112084995
chr11	112090459	112090890
chr11	112091881	112091901
chr11	112092186	112092353
chr11	112093948	112094104
chr11	112096536	112098135
chr11	112120602	112120650
chr11	112125433	112125511
chr11	112125617	112126236
chr11	112126484	112126512
chr11	112148680	112149052
chr11	112151566	112151637
chr11	112160196	112160456
chr11	112160520	112161573
chr11	112163449	112164201
chr11	112168705	112168951
chr11	112174801	112174924
chr11	112181085	112181265
chr11	112181468	112181630
chr11	112185238	112185578
chr11	112188714	112189662
chr11	112191182	112191792
chr11	112192011	112193056
chr11	112193242	112193441
chr11	112204929	112205772
chr11	112225594	112225639
chr11	112235434	112236111
chr11	112236215	112236354
chr11	112295945	112296167
chr11	112319549	112319901
chr11	112324786	112325000

chr11	112341354	112341458
chr11	112341795	112341983
chr11	112459140	112459339
chr11	112481657	112481715
chr11	112483638	112484406
chr11	112484605	112484773
chr11	112485324	112485784
chr11	112491597	112491944
chr11	112524052	112524396
chr11	112527703	112527884
chr11	112796259	112796471
chr11	113176391	113176450
chr11	113179162	113179399
chr11	113179528	113179715
chr11	113184531	113184739
chr11	113184897	113184973
chr11	113185097	113186233
chr11	113187095	113187375
chr11	113189068	113189181
chr11	113212674	113212854
chr11	113215010	113215360
chr11	113216124	113216323
chr11	113216541	113217441
chr11	113231770	113231895
chr11	113233655	113233988
chr11	113234431	113234569
chr11	113243041	113243375
chr11	113243753	113244142
chr11	113244343	113244530
chr11	113247681	113247917
chr11	113248227	113249123
chr11	113255180	113255390
chr11	113255599	113256245
chr11	113256560	113256713
chr11	113257370	113259125
chr11	113272239	113272533
chr11	113316929	113317128
chr11	113317416	113317791
chr11	113317932	113319271
chr11	113432866	113432972
chr11	113511396	113511508
chr11	113560892	113562011
chr11	113592390	113592891
chr11	113632998	113633232
chr11	113643781	113644932
chr11	113648618	113649083

chr11	113649354	113649460
chr11	113667667	113667805
chr11	113676861	113677051
chr11	113689178	113689590
chr11	113716350	113716559
chr11	113730305	113730374
chr11	113730500	113730901
chr11	113733170	113733180
chr11	113734092	113734339
chr11	113745185	113746354
chr11	113746499	113747274
chr11	113748017	113748211
chr11	113754975	113755170
chr11	113755362	113755436
chr11	113755948	113756152
chr11	113761631	113761853
chr11	113906708	113907781
chr11	113911502	113911662
chr11	113928648	113928661
chr11	113928949	113933500
chr11	113934218	113934680
chr11	113934863	113935499
chr11	113936700	113936767
chr11	113935721	113936011
chr11	113937833	113937869
chr11	113938117	113938136
chr11	113938319	113938602
chr11	113943121	113943242
chr11	113948370	113948434
chr11	113950204	113950720
chr11	113950962	113951322
chr11	113952496	113952651
chr11	113955387	113955405
chr11	113958912	113959290
chr11	113963833	113963939
chr11	113965815	113965862
chr11	113977131	113977315
chr11	114001653	114001711
chr11	114003969	114004261
chr11	114004637	114004738
chr11	114005520	114005639
chr11	114005874	114006859
chr11	114007998	114008152
chr11	114008572	114008817
chr11	114009485	114010011
chr11	114013933	114014134

chr11	114018804	114019087
chr11	114020718	114021117
chr11	114021457	114021797
chr11	114022404	114022981
chr11	114023173	114023429
chr11	114024819	114027064
chr11	114028413	114029184
chr11	114029491	114031246
chr11	114032900	114034512
chr11	114034662	114035241
chr11	114035773	114035972
chr11	114036313	114036567
chr11	114036649	114037212
chr11	114037584	114038057
chr11	114039528	114039572
chr11	114031484	114032814
chr11	114040494	114041201
chr11	114042585	114042738
chr11	114043116	114043507
chr11	114044995	114044999
chr11	114046584	114046871
chr11	114047938	114048186
chr11	114048373	114048572
chr11	114049416	114049539
chr11	114049770	114050164
chr11	114050492	114050780
chr11	114051200	114051821
chr11	114052657	114052920
chr11	114064030	114064379
chr11	114064784	114065242
chr11	114065890	114066217
chr11	114066867	114067175
chr11	114067883	114068414
chr11	114068489	114068505
chr11	114071210	114071591
chr11	114075000	114075108
chr11	114076711	114076864
chr11	114079269	114079419
chr11	114115229	114115473
chr11	114128273	114129857
chr11	114130071	114131036
chr11	114131162	114131578
chr11	114151613	114151813
chr11	114155120	114155197
chr11	114163107	114163306
chr11	114165603	114166267

chr11	114166802	114167685
chr11	114178261	114178400
chr11	114178559	114178639
chr11	114188443	114188829
chr11	114211243	114211602
chr11	114219366	114219565
chr11	114237550	114237753
chr11	114240372	114240579
chr11	114270264	114271234
chr11	114271348	114271813
chr11	114283294	114283970
chr11	114309842	114311327
chr11	116577657	116577883
chr11	116578409	116578637
chr11	116603363	116603562
chr11	116613503	116613641
chr11	116641503	116641564
chr11	116642295	116642616
chr11	116643050	116644833
chr11	116657963	116659474
chr11	116663657	116664000
chr11	116679551	116679692
chr11	116679894	116679992
chr11	116680107	116680576
chr11	116706060	116706837
chr11	116750559	116750703
chr11	116750826	116750959
chr11	116758043	116758241
chr11	116819066	116819447
chr11	116829245	116829345
chr11	116831625	116831701
chr11	116841430	116841597
chr11	116855320	116855644
chr11	116857135	116857851
chr11	116860921	116861061
chr11	116866091	116866330
chr11	116879860	116880089
chr11	116881028	116881227
chr11	116881529	116882602
chr11	116898052	116898150
chr11	116898223	116898422
chr11	116899702	116899990
chr11	116908370	116908423
chr11	116935072	116935123
chr11	116935265	116935339
chr11	116943220	116943349

chr11	116948876	116949000
chr11	116949176	116949423
chr11	116955727	116956182
chr11	116959359	116959558
chr11	116959801	116960086
chr11	116960358	116960772
chr11	116961052	116961197
chr11	116967111	116967146
chr11	116967589	116967783
chr11	116967862	116968061
chr11	116968126	116969128
chr11	116969355	116969989
chr11	116970247	116970604
chr11	116972232	116972315
chr11	116976443	116976804
chr11	117007799	117008145
chr11	117016846	117017484
chr11	117014166	117016542
chr11	117020484	117021173
chr11	117023477	117023697
chr11	117026684	117026977
chr11	117027364	117027662
chr11	117038432	117038474
chr11	117042711	117042882
chr11	117043314	117044315
chr11	117044908	117045764
chr11	117048144	117049320
chr11	117047521	117047528
chr11	117049497	117050692
chr11	117055680	117055698
chr11	117062033	117062232
chr11	117069519	117070291
chr11	117077171	117077385
chr11	117077582	117077804
chr11	117090842	117090940
chr11	117092417	117092618
chr11	117093310	117093648
chr11	117098170	117098198
chr11	117102226	117104169
chr11	117110445	117110496
chr11	117119559	117119645
chr11	117122610	117122809
chr11	117150243	117150374
chr11	117151699	117152075
chr11	117152621	117152661
chr11	117186165	117187625

chr11	117189232	117189356
chr11	117198244	117198433
chr11	117198578	117199711
chr11	117199809	117199967
chr11	117200197	117200407
chr11	117201652	117201676
chr11	117277699	117277898
chr11	117588825	117589024
chr11	117675588	117675589
chr11	117679565	117679691
chr11	117688128	117688623
chr11	117688727	117689366
chr11	117694754	117695115
chr11	117695325	117695524
chr11	117697990	117698197
chr11	117700716	117700876
chr11	117777062	117777109
chr11	117778218	117778890
chr11	117799832	117800124
chr11	117800772	117801082
chr11	117942958	117943183
chr11	117943252	117943276
chr11	117943782	117943873
chr11	117948653	117948891
chr11	117945045	117948464
chr11	117949995	117950194
chr11	117951476	117951917
chr11	117952259	117952381
chr11	117954497	117954752
chr11	117954943	117955102
chr11	117955289	117955601
chr11	117956069	117956268
chr11	117961171	117961314
chr11	117961459	117961623
chr11	117977468	117977503
chr11	117977780	117977912
chr11	117979315	117979435
chr11	117986674	117986915
chr11	118012639	118012753
chr11	118012936	118013293
chr11	118013392	118013475
chr11	118013619	118014007
chr11	118016330	118017260
chr11	118023252	118024281
chr11	118036071	118036106
chr11	118036376	118037847

chr11	118038196	118038742
chr11	118040502	118040746
chr11	118042173	118042485
chr11	118043067	118043165
chr11	118044005	118044341
chr11	118044561	118044636
chr11	118045011	118045026
chr11	118045372	118045689
chr11	118046036	118046629
chr11	118046927	118047100
chr11	118047367	118047444
chr11	118048156	118048486
chr11	118049153	118049161
chr11	118050670	118051092
chr11	118053721	118053894
chr11	118069273	118069401
chr11	118082121	118082366
chr11	118082481	118082485
chr11	118103309	118103340
chr11	118112176	118112302
chr11	118112455	118112710
chr11	118115992	118116055
chr11	118118277	118118309
chr11	118121768	118122138
chr11	118122596	118123293
chr11	118123565	118123791
chr11	118134323	118135714
chr11	118136825	118137076
chr11	118146221	118146403
chr11	118147844	118148816
chr11	118151979	118152003
chr11	118152112	118152320
chr11	118161601	118161912
chr11	118162028	118162593
chr11	118166352	118166610
chr11	118187218	118187422
chr11	118230047	118230539
chr11	118234475	118234620
chr11	118251202	118251320
chr11	118251596	118251685
chr11	118254080	118254316
chr11	118269492	118269572
chr11	118269745	118269902
chr11	118271211	118273282
chr11	118273960	118274483
chr11	118292694	118292887

chr11	118295246	118295622
chr11	118296109	118296231
chr11	118305552	118305751
chr11	118306478	118308596
chr11	118308788	118308982
chr11	118309232	118309772
chr11	118332703	118332747
chr11	118334056	118334255
chr11	118334684	118335149
chr11	118359216	118359525
chr11	118377801	118377943
chr11	118399997	118400139
chr11	118400377	118400576
chr11	118400910	118402582
chr11	118408374	118408749
chr11	118409322	118409625
chr11	118412393	118412740
chr11	118429389	118429545
chr11	118436247	118436783
chr11	118437063	118437230
chr11	118442553	118444259
chr11	118445550	118445749
chr11	118447602	118448033
chr11	118462647	118462774
chr11	118464884	118465088
chr11	118465698	118465877
chr11	118475960	118476590
chr11	118478337	118480086
chr11	118481305	118481981
chr11	118482171	118482471
chr11	118483672	118483680
chr11	118485583	118485903
chr11	118487073	118488010
chr11	118488166	118488365
chr11	118488647	118489103
chr11	118489251	118489681
chr11	118489844	118490200
chr11	118490342	118490611
chr11	118491243	118495742
chr11	118490766	118491037
chr11	118536740	118536778
chr11	118559913	118561555
chr11	118561855	118562253
chr11	118562380	118562756
chr11	118562889	118563811
chr11	118569254	118569600

chr11	118570974	118571010
chr11	118577680	118577844
chr11	118586517	118586793
chr11	118589665	118589720
chr11	118590039	118590238
chr11	118600258	118600465
chr11	118605888	118606018
chr11	118607531	118607547
chr11	118636864	118637134
chr11	118644959	118645162
chr11	118648237	118648474
chr11	118649661	118649837
chr11	118650648	118650753
chr11	118653398	118653602
chr11	118654483	118654710
chr11	118660729	118663274
chr11	118672266	118672402
chr11	118689861	118690079
chr11	118691475	118691501
chr11	118692069	118692193
chr11	118713254	118713477
chr11	118713591	118713892
chr11	118716028	118716521
chr11	118716752	118716995
chr11	118753914	118754018
chr11	118758274	118758751
chr11	118770653	118770983
chr11	118774709	118775181
chr11	118776331	118777024
chr11	118777345	118777733
chr11	118777888	118778383
chr11	118778762	118779247
chr11	118779444	118783146
chr11	118783266	118796748
chr11	118796851	118798228
chr11	118798372	118801800
chr11	118802439	118802712
chr11	118804753	118805083
chr11	118805717	118806086
chr11	118808967	118809343
chr11	118810582	118811399
chr11	118819422	118819757
chr11	118822924	118823141
chr11	118832848	118833008
chr11	118839536	118839700
chr11	118847069	118847268

chr11	118854370	118854595
chr11	118858417	118859983
chr11	118860959	118861099
chr11	118863101	118863300
chr11	118863803	118864229
chr11	118864734	118865051
chr11	118866314	118866538
chr11	118867366	118867493
chr11	118867651	118869474
chr11	118869605	118870039
chr11	118871214	118871558
chr11	118875192	118875595
chr11	118881093	118881232
chr11	118885682	118885771
chr11	118885879	118886172
chr11	118886666	118886902
chr11	118887008	118887104
chr11	118887351	118887557
chr11	118888619	118890489
chr11	118890718	118890834
chr11	118891121	118891451
chr11	118891658	118891890
chr11	118892660	118892772
chr11	118892983	118893010
chr11	118893606	118893918
chr11	118900286	118901977
chr11	118903796	118904291
chr11	118913300	118913715
chr11	118920935	118921155
chr11	118925627	118925826
chr11	118926165	118926364
chr11	118926728	118927842
chr11	118928027	118929288
chr11	118931897	118931993
chr11	118932657	118932890
chr11	118933325	118933710
chr11	118933918	118934513
chr11	118936115	118936193
chr11	118937753	118938332
chr11	118938555	118938881
chr11	118951411	118951430
chr11	118951796	118952089
chr11	118954763	118955881
chr11	118955995	118956779
chr11	118957084	118957169
chr11	118957427	118957647

chr11	118957730	118958054
chr11	118958340	118958393
chr11	118962126	118962363
chr11	118964339	118966154
chr11	118966525	118966964
chr11	118969725	118969948
chr11	118970930	118974381
chr11	118976105	118976313
chr11	118977071	118978788
chr11	118991216	118992121
chr11	118992267	118992743
chr11	118993853	118993970
chr11	118995809	118995836
chr11	118995983	118996034
chr11	118996122	118996501
chr11	118996648	118996886
chr11	118997418	118997457
chr11	119012396	119012595
chr11	119019394	119020241
chr11	119025590	119025809
chr11	119036649	119040898
chr11	119041583	119041842
chr11	119051208	119051224
chr11	119051316	119051411
chr11	119057848	119058047
chr11	119062185	119062416
chr11	119068187	119068407
chr11	119068556	119068938
chr11	119069670	119069869
chr11	119076219	119078082
chr11	119080703	119081202
chr11	119086804	119087194
chr11	119087868	119088095
chr11	119088648	119089362
chr11	119089468	119089640
chr11	119095906	119096105
chr11	119099364	119099622
chr11	119100389	119100790
chr11	119102262	119102461
chr11	119115384	119115413
chr11	119155313	119155437
chr11	119176819	119176835
chr11	119184058	119184410
chr11	119186025	119187860
chr11	119188092	119190624
chr11	119191299	119193018

chr11	119193188	119193513
chr11	119193618	119193911
chr11	119194434	119194633
chr11	119194935	119195051
chr11	119197468	119197474
chr11	119199562	119199676
chr11	119203539	119203617
chr11	119204700	119205991
chr11	119208501	119208928
chr11	119210669	119211581
chr11	119223068	119223239
chr11	119225637	119225665
chr11	119225824	119225851
chr11	119227264	119227650
chr11	119229054	119229253
chr11	119230910	119230995
chr11	119231351	119231722
chr11	119231844	119232160
chr11	119232318	119232867
chr11	119234401	119235466
chr11	119238709	119238964
chr11	119239246	119239639
chr11	119246332	119247463
chr11	119247970	119248410
chr11	119248592	119248806
chr11	119249151	119249388
chr11	119251896	119252206
chr11	119252480	119252688
chr11	119253030	119253246
chr11	119260296	119260489
chr11	119286952	119287161
chr11	119287865	119288315
chr11	119288721	119289039
chr11	119292749	119293409
chr11	119298686	119298746
chr11	119318517	119318789
chr11	119341359	119341927
chr11	119345639	119345840
chr11	119486612	119486924
chr11	119536950	119537562
chr11	119538360	119538559
chr11	119539771	119540026
chr11	119540583	119540951
chr11	119546394	119546625
chr11	119546695	119546811
chr11	119547119	119547426

chr11	119547674	119548217
chr11	119551032	119551419
chr11	119552215	119553144
chr11	119553790	119554026
chr11	119554167	119557024
chr11	119558472	119558671
chr11	119559133	119559689
chr11	119560025	119571798
chr11	119571932	119573201
chr11	119573353	119573382
chr11	119573849	119574266
chr11	119578791	119578831
chr11	119578946	119579002
chr11	119580042	119580384
chr11	119590576	119590898
chr11	119591856	119592358
chr11	119593318	119593517
chr11	119593678	119593697
chr11	119594138	119594408
chr11	119596264	119600761
chr11	119601586	119601785
chr11	119716815	119717014
chr11	119736941	119737221
chr11	119926204	119927598
chr11	119927991	119928451
chr11	119931462	119931594
chr11	119966011	119966117
chr11	119991474	119994632
chr11	119995282	119995759
chr11	119997152	119997305
chr11	119998965	120000277
chr11	120000507	120000902
chr11	120001057	120001699
chr11	120001847	120002321
chr11	120002686	120004383
chr11	120004608	120005007
chr11	120005282	120005856
chr11	120005969	120008925
chr11	120009095	120009532
chr11	120013400	120013412
chr11	120017667	120019234
chr11	120019776	120019949
chr11	120020157	120020309
chr11	120023939	120024218
chr11	120039470	120040293
chr11	120050550	120050984

chr11	120076426	120076737
chr11	120078657	120078664
chr11	120078769	120078971
chr11	120079154	120079176
chr11	120079428	120079661
chr11	120080135	120080332
chr11	120080669	120083209
chr11	120107222	120107421
chr11	120110664	120111078
chr11	120173953	120174249
chr11	120174690	120174939
chr11	120179000	120179148
chr11	120186034	120186106
chr11	120187125	120187534
chr11	120187783	120188020
chr11	120195909	120196600
chr11	120198949	120199071
chr11	120199239	120199579
chr11	120201866	120202454
chr11	120202620	120202761
chr11	120205770	120206120
chr11	120206292	120206871
chr11	120206984	120208288
chr11	120208515	120208871
chr11	120210173	120210678
chr11	120210889	120211088
chr11	120216986	120217191
chr11	120254749	120254859
chr11	120271363	120271963
chr11	120294921	120295236
chr11	120302354	120303218
chr11	120381990	120382226
chr11	120382453	120382798
chr11	120435343	120435572
chr11	120436040	120436251
chr11	120436448	120436644
chr11	120437430	120437551
chr11	120617947	120618151
chr11	120856561	120856925
chr11	120857179	120857269
chr11	120894691	120895662
chr11	121162711	121163222
chr11	121163479	121164319
chr11	121227212	121227361
chr11	121228988	121229194
chr11	121286254	121286554

chr11	121287892	121288106
chr11	121297836	121298232
chr11	121318462	121318578
chr11	121324081	121324275
chr11	121322392	121323769
chr11	121331520	121331719
chr11	121332227	121332431
chr11	121335200	121335582
chr11	121335718	121335761
chr11	121336568	121337412
chr11	121337519	121338042
chr11	121338813	121339155
chr11	121347605	121347719
chr11	121353329	121353528
chr11	121390916	121391140
chr11	121392553	121392560
chr11	121411377	121411646
chr11	121419297	121419578
chr11	121420960	121421719
chr11	121422206	121422539
chr11	121424236	121424782
chr11	121424987	121425127
chr11	121462480	121462553
chr11	121466322	121466697
chr11	121466848	121467645
chr11	121468388	121468715
chr11	121486304	121486741
chr11	121526120	121527101
chr11	121593044	121593259
chr11	121593450	121593783
chr11	121762729	121762859
chr11	121763097	121763999
chr11	121765335	121765640
chr11	121766891	121767023
chr11	121775545	121775792
chr11	121807634	121807912
chr11	121834534	121834851
chr11	121835162	121835395
chr11	121835745	121835809
chr11	121835990	121836288
chr11	121894402	121894711
chr11	121909584	121909619
chr11	121914842	121915041
chr11	121915797	121916180
chr11	121916348	121916422
chr11	121941027	121941090

chr11	121955931	121956046
chr11	121956190	121956296
chr11	121968159	121968770
chr11	121969967	121970406
chr11	121970976	121971480
chr11	121971707	121971710
chr11	121972364	121972454
chr11	121975108	121975166
chr11	121977901	121978027
chr11	121994382	121994814
chr11	122007760	122008199
chr11	122011161	122011267
chr11	122011486	122011699
chr11	122028841	122029090
chr11	122029200	122029497
chr11	122029761	122030475
chr11	122032832	122033212
chr11	122027754	122028111
chr11	122037757	122038291
chr11	122038920	122039182
chr11	122049502	122049861
chr11	122051108	122051646
chr11	122057154	122057633
chr11	122059891	122060347
chr11	122062868	122063067
chr11	122063363	122063830
chr11	122064799	122065117
chr11	122067650	122068260
chr11	122072810	122074344
chr11	122106597	122106745
chr11	122119606	122119741
chr11	122121680	122121879
chr11	122159999	122160123
chr11	122233079	122233162
chr11	122238501	122238939
chr11	122292352	122294048
chr11	122289796	122290257
chr11	122310739	122312179
chr11	122362162	122362267
chr11	122394592	122394791
chr11	122426652	122426762
chr11	122427173	122427842
chr11	122450855	122451245
chr11	122451398	122452131
chr11	122452295	122452412
chr11	122472592	122472644

chr11	122477836	122478196
chr11	122499965	122500056
chr11	122500268	122500545
chr11	122500923	122501276
chr11	122503943	122504353
chr11	122505279	122505290
chr11	122502630	122503729
chr11	122505464	122506015
chr11	122508092	122508103
chr11	122511488	122511757
chr11	122513417	122513818
chr11	122525754	122527911
chr11	122532521	122533024
chr11	122533142	122533414
chr11	122546875	122547499
chr11	122597135	122597184
chr11	122601526	122601601
chr11	122635235	122635437
chr11	122648553	122648665
chr11	122648950	122649289
chr11	122656910	122657201
chr11	122657641	122657657
chr11	122658226	122658231
chr11	122673740	122673939
chr11	122674207	122675157
chr11	122675305	122676037
chr11	122687874	122688271
chr11	122724609	122724751
chr11	122724889	122725016
chr11	122727156	122727591
chr11	122727794	122727942
chr11	122732344	122732469
chr11	122734438	122734535
chr11	122753215	122754405
chr11	122767518	122767715
chr11	122768626	122768670
chr11	122781517	122781566
chr11	122781670	122781846
chr11	122794360	122794365
chr11	122854837	122855274
chr11	122906896	122907047
chr11	122915704	122915834
chr11	122924602	122925017
chr11	122925647	122925839
chr11	122927158	122927315
chr11	122929305	122929663

chr11	122931039	122931141
chr11	122931420	122931550
chr11	122931992	122934434
chr11	122935506	122935830
chr11	122936312	122936792
chr11	122937384	122937632
chr11	122943054	122943253
chr11	122944967	122945166
chr11	122954291	122954445
chr11	122957611	122957810
chr11	122963908	122964028
chr11	122966097	122966264
chr11	122969282	122969406
chr11	122982117	122982462
chr11	122982784	122983293
chr11	122989112	122990005
chr11	122992937	122993593
chr11	123001200	123001238
chr11	123002942	123003160
chr11	123003407	123003695
chr11	123015839	123015975
chr11	123016113	123016997
chr11	123021593	123021765
chr11	123028937	123029086
chr11	123029338	123029899
chr11	123030363	123030803
chr11	123031399	123031654
chr11	123043911	123044239
chr11	123044455	123044613
chr11	123045402	123045432
chr11	123045637	123046148
chr11	123046295	123046495
chr11	123051132	123051142
chr11	123054008	123054315
chr11	123054564	123054743
chr11	123057237	123057554
chr11	123064719	123067406
chr11	123068346	123068861
chr11	123131949	123132176
chr11	123143259	123143546
chr11	123172909	123173164
chr11	123173383	123173572
chr11	123182400	123182554
chr11	123277004	123277203
chr11	123300969	123301618
chr11	123324727	123324922

chr11	123327249	123327509
chr11	123344741	123345050
chr11	123349025	123349075
chr11	123471556	123471954
chr11	123545396	123545459
chr11	123548936	123548995
chr11	123549265	123549360
chr11	123550484	123550773
chr11	123565326	123565531
chr11	123588792	123588876
chr11	123611183	123612704
chr11	123612779	123613059
chr11	123844489	123844589
chr11	123844807	123844968
chr11	123847944	123848261
chr11	123848625	123848788
chr11	123849046	123849078
chr11	123942322	123942489
chr11	123951243	123951450
chr11	123951685	123951728
chr11	123985902	123985970
chr11	123986259	123986412
chr11	124065354	124065435
chr11	124065661	124065963
chr11	124066185	124066245
chr11	124093239	124093248
chr11	124492336	124493432
chr11	124497445	124497560
chr11	124503277	124503303
chr11	124514700	124514960
chr11	124542738	124543544
chr11	124543851	124544227
chr11	124546329	124546648
chr11	124587129	124587392
chr11	124609423	124609702
chr11	124609873	124610435
chr11	124615488	124616912
chr11	124627609	124627822
chr11	124628379	124628653
chr11	124632633	124633053
chr11	124640143	124640487
chr11	124669187	124670937
chr11	124671152	124671379
chr11	124706631	124707024
chr11	124745547	124747930
chr11	124767416	124769149

chr11	124769699	124770545
chr11	124770690	124770866
chr11	124771586	124771771
chr11	124790522	124791626
chr11	124791889	124792128
chr11	124823598	124823894
chr11	124824110	124824486
chr11	124824644	124824862
chr11	124840788	124840863
chr11	124932469	124934107
chr11	124939713	124941969
chr11	124945131	124946537
chr11	124951670	124954093
chr11	124954490	124954738
chr11	124961301	124962121
chr11	124968718	124968776
chr11	124980834	124981607
chr11	125034675	125034955
chr11	125170442	125170623
chr11	125293783	125293902
chr11	125332564	125332711
chr11	125353377	125353779
chr11	125364545	125366601
chr11	125379705	125379800
chr11	125383146	125383515
chr11	125384114	125384930
chr11	125387113	125387369
chr11	125387586	125387686
chr11	125390297	125390437
chr11	125438786	125440643
chr11	125455846	125456017
chr11	125458038	125458250
chr11	125458511	125458627
chr11	125458775	125458828
chr11	125459704	125459820
chr11	125461190	125461443
chr11	125461507	125462295
chr11	125462697	125463628
chr11	125464219	125464445
chr11	125491851	125492267
chr11	125493842	125494123
chr11	125495101	125496854
chr11	125500276	125500486
chr11	125527848	125527854
chr11	125754222	125754484
chr11	125756559	125758633

chr11	125771990	125773083
chr11	125773990	125774483
chr11	125821814	125821981
chr11	125822844	125823316
chr11	125823474	125823597
chr11	125823671	125823673
chr11	125838524	125838594
chr11	125846051	125846432
chr11	125931581	125931868
chr11	125932079	125933566
chr11	125990249	125990287
chr11	126000638	126000894
chr11	126002019	126002234
chr11	126006234	126006489
chr11	126010451	126010580
chr11	126032609	126032808
chr11	126033286	126033504
chr11	126064018	126064022
chr11	126080851	126081504
chr11	126081697	126082126
chr11	126091891	126092029
chr11	126093175	126093363
chr11	126103231	126103462
chr11	126137933	126137956
chr11	126138247	126138805
chr11	126139074	126139841
chr11	126152396	126154267
chr11	126154516	126155080
chr11	126173445	126174588
chr11	126174728	126175126
chr11	126177191	126177422
chr11	126177529	126177604
chr11	126187703	126189383
chr11	126203335	126203581
chr11	126210691	126210736
chr11	126210305	126210582
chr11	126210852	126211333
chr11	126211536	126212544
chr11	126212896	126212913
chr11	126215711	126216617
chr11	126216975	126217268
chr11	126217363	126217401
chr11	126220315	126220574
chr11	126223152	126226889
chr11	126222199	126222666
chr11	126227713	126228136

chr11	126229526	126229976
chr11	126230094	126230320
chr11	126230443	126230684
chr11	126231807	126231865
chr11	126232006	126232178
chr11	126232824	126233023
chr11	126241586	126241902
chr11	126242541	126242895
chr11	126243827	126244132
chr11	126244284	126244338
chr11	126245671	126245875
chr11	126251615	126251943
chr11	126252431	126252879
chr11	126261926	126262791
chr11	126263452	126263561
chr11	126263798	126265422
chr11	126265491	126265573
chr11	126272168	126273933
chr11	126274147	126275876
chr11	126276042	126276519
chr11	126286043	126287005
chr11	126301443	126301719
chr11	126301905	126302580
chr11	126310929	126311128
chr11	126350419	126350730
chr11	126354783	126354887
chr11	126354442	126354576
chr11	126420263	126420428
chr11	127760926	127761145
chr11	127894695	127894747
chr11	127896037	127896971
chr11	127897537	127897877
chr11	127900671	127900870
chr11	127901457	127901897
chr11	127909678	127909888
chr11	127922789	127923380
chr11	127924600	127924799
chr11	127925427	127925585
chr11	127999603	127999802
chr11	128016869	128017395
chr11	128027591	128028037
chr11	128041153	128041413
chr11	128041529	128041816
chr11	128042222	128042595
chr11	128044537	128044887
chr11	128050270	128050490

chr11	128050637	128050765
chr11	128076394	128076784
chr11	128077335	128079100
chr11	128080054	128080886
chr11	128116315	128116421
chr11	128121040	128121489
chr11	128142112	128142202
chr11	128142463	128142537
chr11	128159922	128160014
chr11	128269397	128269528
chr11	128279359	128279576
chr11	128279682	128279788
chr11	128318321	128318975
chr11	128323110	128323909
chr11	128325324	128325411
chr11	128326146	128326350
chr11	128326724	128327198
chr11	128328126	128328486
chr11	128337847	128338016
chr11	128341219	128341446
chr11	128344868	128345309
chr11	128345547	128345764
chr11	128345912	128346250
chr11	128354362	128354486
chr11	128358396	128358563
chr11	128358771	128358953
chr11	128359181	128359405
chr11	128362171	128362810
chr11	128363114	128363485
chr11	128364747	128364946
chr11	128366506	128367180
chr11	128367366	128367685
chr11	128372546	128372801
chr11	128372959	128373046
chr11	128373359	128373592
chr11	128373734	128374090
chr11	128376396	128377064
chr11	128377202	128377848
chr11	128379078	128379344
chr11	128379987	128380253
chr11	128387466	128387812
chr11	128388463	128388840
chr11	128389455	128389673
chr11	128390488	128390786
chr11	128391396	128392984
chr11	128418596	128419090

chr11	128457284	128457476
chr11	128500277	128500679
chr11	128502566	128502688
chr11	128556790	128557076
chr11	128557238	128557437
chr11	128562497	128566036
chr11	128579266	128579822
chr11	128584730	128584996
chr11	128585173	128585230
chr11	128592967	128593063
chr11	128701002	128701146
chr11	128701579	128701778
chr11	128702083	128702091
chr11	128733133	128733519
chr11	128754528	128754731
chr11	128757782	128757914
chr11	128758221	128758396
chr11	128759673	128759872
chr11	128760450	128761089
chr11	128762931	128763246
chr11	128768473	128769056
chr11	128774794	128775572
chr11	128775750	128776273
chr11	128779017	128779383
chr11	128779516	128779762
chr11	128792678	128792743
chr11	128809758	128810139
chr11	128810438	128811184
chr11	128811367	128811575
chr11	128826309	128826508
chr11	128893872	128894343
chr11	128918817	128918992
chr11	128931317	128931473
chr11	128931582	128931739
chr11	129013144	129013340
chr11	129013624	129013725
chr11	129016115	129016259
chr11	129016373	129016475
chr11	129060009	129060352
chr11	129061556	129061881
chr11	129062017	129062348
chr11	129118674	129118883
chr11	129119256	129119592
chr11	129148479	129148910
chr11	129149196	129149452
chr11	129149652	129150045

chr11	129150506	129150680
chr11	129234401	129234504
chr11	129243610	129243718
chr11	129243816	129244448
chr11	129244641	129245392
chr11	129246024	129246516
chr11	129309481	129309544
chr11	129328720	129328887
chr11	129455066	129455426
chr11	129490419	129490689
chr11	129495546	129496323
chr11	129503706	129503952
chr11	129506664	129506750
chr11	129506864	129507005
chr11	129507355	129507793
chr11	129508062	129508421
chr11	129509151	129509374
chr11	129509496	129509673
chr11	129510530	129510549
chr11	129510667	129510835
chr11	129511855	129512228
chr11	129512905	129513602
chr11	129513709	129513800
chr11	129565395	129565720
chr11	129571572	129571627
chr11	129573362	129573561
chr11	129574160	129574233
chr11	129574551	129575309
chr11	129594487	129594568
chr11	129594829	129595242
chr11	129601012	129601076
chr11	129601174	129601366
chr11	129602212	129602315
chr11	129608371	129608467
chr11	129620251	129620873
chr11	129636995	129637235
chr11	129655597	129655644
chr11	129657831	129657844
chr11	129659602	129659838
chr11	129686005	129686612
chr11	129687816	129687987
chr11	129693163	129693562
chr11	129695239	129695738
chr11	129696309	129696522
chr11	129696992	129697240
chr11	129697886	129699063

chr11	129699337	129699793
chr11	129701147	129701391
chr11	129707184	129707273
chr11	129709401	129709697
chr11	129710162	129710186
chr11	129710298	129710718
chr11	129716323	129716883
chr11	129718075	129718324
chr11	129718668	129718924
chr11	129722026	129722251
chr11	129723434	129723915
chr11	129724054	129724793
chr11	129724952	129725024
chr11	129726140	129726340
chr11	129731071	129731729
chr11	129731984	129732373
chr11	129732987	129733150
chr11	129733267	129733407
chr11	129764825	129766021
chr11	129781198	129781553
chr11	129793159	129793568
chr11	129802544	129802733
chr11	129802842	129803311
chr11	129850831	129851006
chr11	129851916	129852204
chr11	129856266	129856367
chr11	129861558	129861757
chr11	129871300	129874188
chr11	129890969	129891328
chr11	129892088	129892314
chr11	129892654	129893130
chr11	129893361	129893529
chr11	129896751	129896901
chr11	129899226	129899401
chr11	129901868	129902430
chr11	129906600	129907411
chr11	129907818	129908062
chr11	129909764	129910130
chr11	129911025	129911216
chr11	129911814	129912043
chr11	129912197	129912775
chr11	129913759	129913982
chr11	129917213	129917269
chr11	129921141	129921396
chr11	129938504	129939590
chr11	129939726	129941214

chr11	129941979	129942307
chr11	129941278	129941645
chr11	129946692	129947027
chr11	129986421	129986628
chr11	129994288	129994469
chr11	130001186	130001385
chr11	130001723	130001883
chr11	130002542	130002590
chr11	130003092	130003296
chr11	130003564	130003830
chr11	130009218	130009295
chr11	130015527	130015971
chr11	130017250	130017285
chr11	130017573	130017622
chr11	130018329	130018414
chr11	130029448	130030728
chr11	130033069	130033102
chr11	130033353	130033589
chr11	130034347	130034482
chr11	130035859	130036514
chr11	130036636	130036811
chr11	130045397	130045799
chr11	130047869	130048271
chr11	130052222	130052477
chr11	130067384	130067693
chr11	130084688	130084887
chr11	130145657	130145943
chr11	130179941	130180122
chr11	130183757	130185587
chr11	130197540	130197786
chr11	130276996	130277163
chr11	130284103	130284590
chr11	130298030	130298236
chr11	130304653	130305144
chr11	130317952	130318151
chr11	130500943	130501021
chr11	130504844	130504897
chr11	130505271	130505518
chr11	130717276	130717623
chr11	130785647	130786752
chr11	130817596	130818214
chr11	130870060	130870239
chr11	130870345	130870697
chr11	130871893	130872132
chr11	130872282	130872483
chr11	130902906	130903125

chr11	130903231	130903716
chr11	130911207	130911286
chr11	130911593	130911648
chr11	131758144	131758209
chr11	131777557	131777621
chr11	131780283	131780839
chr11	131847080	131847598
chr11	131847785	131847975
chr11	131894034	131894259
chr11	131894546	131895069
chr11	131895172	131895229
chr11	131934799	131934874
chr11	131935222	131935325
chr11	131941922	131942702
chr11	131958683	131958788
chr11	134053076	134053097
chr11	134093174	134093350
chr11	134093600	134095481
chr11	134095795	134095895
chr11	134096126	134096413
chr11	134122695	134124603
chr11	134136398	134136880
chr11	134137006	134137283
chr11	134201308	134201343
chr11	134201827	134202420
chr11	134202557	134202756
chr12	72065	72398
chr12	73505	75153
chr12	76470	76625
chr12	92377	92680
chr12	95314	95755
chr12	466130	466174
chr12	498503	498810
chr12	498946	499081
chr12	510414	510441
chr12	510763	511189
chr12	515851	516456
chr12	569147	570057
chr12	577030	577107
chr12	612041	612229
chr12	618360	618559
chr12	619865	620166
chr12	635641	635685
chr12	642426	642525
chr12	664060	664326
chr12	678655	681773

chr12	681897	682022
chr12	682627	682657
chr12	684904	684924
chr12	685165	685306
chr12	685428	685727
chr12	707688	707847
chr12	716932	717090
chr12	718706	719386
chr12	719690	719792
chr12	724128	724231
chr12	726588	726840
chr12	733118	733255
chr12	741215	741533
chr12	741640	742001
chr12	747055	747102
chr12	751647	752053
chr12	752204	752763
chr12	752839	753075
chr12	778044	778142
chr12	799324	799523
chr12	799708	800041
chr12	821578	821698
chr12	860206	860455
chr12	860637	861465
chr12	861693	862819
chr12	863234	863686
chr12	863961	864445
chr12	888624	888823
chr12	898566	898721
chr12	925613	925947
chr12	926658	927070
chr12	956872	957255
chr12	965355	965557
chr12	966722	967125
chr12	983192	983458
chr12	1007403	1007516
chr12	1007643	1007754
chr12	1023191	1023545
chr12	1023860	1023941
chr12	1054871	1055162
chr12	1057774	1059467
chr12	1078950	1078974
chr12	1079233	1079416
chr12	1098955	1100288
chr12	1100400	1100850
chr12	1101104	1101457

chr12	1102973	1103312
chr12	1115333	1115714
chr12	1122599	1123665
chr12	1224632	1225456
chr12	1298307	1298394
chr12	1326313	1326349
chr12	1402264	1402603
chr12	1554385	1554429
chr12	1554588	1554657
chr12	1582563	1582978
chr12	1583183	1584289
chr12	1584898	1585598
chr12	1597982	1598181
chr12	1608930	1609008
chr12	1609274	1609412
chr12	1627903	1628488
chr12	1639162	1639863
chr12	1640207	1640429
chr12	1650423	1650749
chr12	1692653	1692759
chr12	1700490	1700594
chr12	1701676	1703537
chr12	1703727	1704025
chr12	1704189	1704373
chr12	1713969	1714017
chr12	1714309	1715320
chr12	1715559	1715763
chr12	1734799	1734920
chr12	1739277	1739557
chr12	1762008	1762333
chr12	1770520	1770939
chr12	1769332	1770402
chr12	1771243	1771913
chr12	1799803	1799894
chr12	1800027	1800997
chr12	1869834	1869933
chr12	1870091	1870228
chr12	1885659	1886153
chr12	1886963	1887325
chr12	1904944	1905290
chr12	1905680	1906624
chr12	1913645	1914412
chr12	2102640	2102929
chr12	2103360	2103554
chr12	2113151	2113797
chr12	2162278	2162564

chr12	2245152	2245547
chr12	2364992	2365220
chr12	2445450	2445478
chr12	2445638	2445947
chr12	2448415	2448571
chr12	2903451	2905879
chr12	2915929	2915961
chr12	2921483	2922481
chr12	2922715	2922956
chr12	2985606	2986622
chr12	2986725	2987232
chr12	2999293	3000495
chr12	3000755	3000891
chr12	3002250	3002553
chr12	3042132	3042441
chr12	3054113	3054127
chr12	3066848	3067081
chr12	3067189	3067467
chr12	3067624	3068395
chr12	3068707	3069832
chr12	3100436	3100556
chr12	3100745	3100785
chr12	3185286	3185620
chr12	3185715	3187167
chr12	3229314	3229700
chr12	3277758	3277784
chr12	3278070	3278269
chr12	3309545	3310036
chr12	3325810	3326627
chr12	3326757	3327770
chr12	3475109	3475417
chr12	3563064	3563799
chr12	3563943	3564602
chr12	3565952	3566692
chr12	3566935	3567224
chr12	3568377	3568390
chr12	3636355	3636392
chr12	3636710	3636719
chr12	3807299	3807438
chr12	3807661	3807829
chr12	3861962	3862002
chr12	3862385	3862600
chr12	3879835	3880090
chr12	3982081	3982576
chr12	3982705	3982864
chr12	4104993	4105149

chr12	4108435	4108598
chr12	4108780	4109142
chr12	4120090	4120420
chr12	4120697	4120965
chr12	4140049	4140649
chr12	4140778	4141220
chr12	4162193	4162393
chr12	4222140	4222526
chr12	4224869	4225068
chr12	4225228	4225504
chr12	4226273	4226341
chr12	4238236	4238470
chr12	4251881	4252535
chr12	4266249	4266275
chr12	4266466	4266677
chr12	4273564	4274551
chr12	4377823	4377929
chr12	4378153	4379174
chr12	4379343	4381112
chr12	4381387	4382339
chr12	4382626	4384223
chr12	4384439	4385069
chr12	4385132	4385704
chr12	4385924	4386534
chr12	4429590	4429867
chr12	4430330	4430733
chr12	4593545	4593726
chr12	4593950	4594027
chr12	4627622	4627698
chr12	4647424	4648260
chr12	4713973	4714422
chr12	4757576	4758461
chr12	6156811	6156838
chr12	6156915	6157134
chr12	6157313	6157358
chr12	6172546	6172747
chr12	6181521	6181564
chr12	6181922	6182080
chr12	6187773	6187972
chr12	6190445	6190491
chr12	6193417	6194033
chr12	6194144	6194774
chr12	6195636	6195928
chr12	6197943	6198276
chr12	6202167	6202282
chr12	6217108	6217318

chr12	6225540	6225895
chr12	6275614	6275969
chr12	6276272	6277860
chr12	6286674	6286795
chr12	6289338	6290346
chr12	6290698	6291450
chr12	6291580	6292854
chr12	6292995	6293574
chr12	6303147	6303348
chr12	6304755	6304985
chr12	6305125	6305705
chr12	6309096	6309395
chr12	6309540	6311232
chr12	6311586	6311664
chr12	6315528	6315762
chr12	6317314	6317417
chr12	6317594	6317660
chr12	6318032	6318619
chr12	6319001	6319266
chr12	6323441	6323456
chr12	6323654	6325344
chr12	6326310	6326605
chr12	6325462	6325847
chr12	6327451	6327590
chr12	6328092	6328460
chr12	6332507	6332786
chr12	6333851	6334181
chr12	6335129	6336234
chr12	6338340	6338808
chr12	6360462	6360470
chr12	6376850	6376997
chr12	6378536	6378870
chr12	6380706	6382992
chr12	6383076	6383465
chr12	6385577	6385598
chr12	6386561	6386772
chr12	6387144	6388495
chr12	6388635	6388666
chr12	6388736	6388920
chr12	6389157	6389969
chr12	6393650	6393871
chr12	6394187	6394293
chr12	6394459	6394597
chr12	6394840	6395410
chr12	6395702	6396627
chr12	6397186	6397437

chr12	6397568	6397799
chr12	6401958	6402084
chr12	6404092	6404418
chr12	6406122	6406171
chr12	6406374	6406387
chr12	6419667	6420546
chr12	6420870	6422070
chr12	6422424	6422901
chr12	6423360	6423559
chr12	6423024	6423288
chr12	6424188	6424481
chr12	6430728	6430816
chr12	6432283	6432482
chr12	6432732	6433207
chr12	6434620	6434844
chr12	6439756	6439779
chr12	6441329	6441719
chr12	6441976	6442728
chr12	6442834	6445431
chr12	6445570	6446924
chr12	6447059	6447662
chr12	6448361	6449134
chr12	6449385	6451477
chr12	6451640	6451724
chr12	6452375	6452478
chr12	6453820	6454740
chr12	6460835	6461020
chr12	6470535	6470993
chr12	6472351	6473136
chr12	6471223	6472116
chr12	6473403	6473709
chr12	6476841	6476901
chr12	6478117	6478781
chr12	6479194	6480760
chr12	6480996	6481209
chr12	6481387	6482635
chr12	6482962	6486395
chr12	6486654	6487250
chr12	6488801	6488959
chr12	6489473	6490032
chr12	6492488	6494896
chr12	6495124	6495241
chr12	6495452	6495534
chr12	6498793	6498947
chr12	6499919	6500122
chr12	6531339	6531417

chr12	6531603	6531719
chr12	6532548	6532731
chr12	6539584	6539755
chr12	6539888	6540174
chr12	6540240	6540584
chr12	6540785	6541008
chr12	6559766	6561202
chr12	6561319	6562175
chr12	6579378	6580591
chr12	6597600	6597799
chr12	6602227	6602425
chr12	6603175	6603629
chr12	6605642	6605891
chr12	6606870	6607303
chr12	6616347	6617156
chr12	6637609	6637936
chr12	6638434	6638943
chr12	6640221	6640422
chr12	6640595	6640749
chr12	6641037	6641267
chr12	6641585	6645040
chr12	6645104	6645432
chr12	6645585	6645828
chr12	6647393	6647669
chr12	6648219	6648742
chr12	6649362	6650277
chr12	6656480	6656902
chr12	6657052	6657676
chr12	6660885	6661138
chr12	6661266	6661465
chr12	6667057	6667564
chr12	6676819	6678059
chr12	6695611	6696205
chr12	6710359	6710567
chr12	6714577	6716644
chr12	6716797	6717103
chr12	6717267	6717562
chr12	6717844	6718061
chr12	6721827	6722075
chr12	6722337	6722891
chr12	6723220	6723769
chr12	6724217	6724516
chr12	6727101	6727334
chr12	6742098	6742591
chr12	6744486	6745845
chr12	6771833	6772306

chr12	6772504	6772730
chr12	6797469	6798594
chr12	6798839	6799151
chr12	6809040	6810011
chr12	6819920	6819925
chr12	6821209	6821433
chr12	6821717	6821994
chr12	6832753	6833319
chr12	6833483	6834018
chr12	6835112	6835261
chr12	6844777	6844967
chr12	6845160	6845442
chr12	6851850	6851938
chr12	6854766	6854878
chr12	6855262	6855782
chr12	6861428	6862775
chr12	6863072	6863413
chr12	6864070	6864311
chr12	6864853	6865110
chr12	6867143	6867375
chr12	6872938	6874022
chr12	6874230	6877214
chr12	6877923	6878162
chr12	6881141	6881340
chr12	6887323	6888173
chr12	6930724	6931405
chr12	6933732	6933931
chr12	6934596	6934795
chr12	6936335	6936686
chr12	6936813	6939109
chr12	6945061	6945311
chr12	6959579	6959682
chr12	6959939	6960185
chr12	6960719	6961047
chr12	6961255	6962064
chr12	6962213	6962622
chr12	6976365	6978696
chr12	6980354	6980675
chr12	6980804	6983062
chr12	6983244	6983360
chr12	6999776	7000233
chr12	7000425	7001238
chr12	7013723	7013888
chr12	7014138	7014403
chr12	7023449	7024030
chr12	7024267	7024546

chr12	7033339	7034688
chr12	7034799	7036306
chr12	7036487	7037189
chr12	7037306	7038690
chr12	7039398	7039520
chr12	7044146	7044283
chr12	7046596	7047983
chr12	7049666	7049888
chr12	7052010	7052927
chr12	7053013	7053835
chr12	7053970	7054173
chr12	7054422	7054684
chr12	7055108	7055464
chr12	7055931	7056467
chr12	7056680	7056913
chr12	7058799	7058808
chr12	7070048	7074321
chr12	7074593	7074749
chr12	7078440	7079853
chr12	7079974	7081013
chr12	7125035	7126524
chr12	7144418	7144781
chr12	7147056	7147099
chr12	7147165	7147609
chr12	7167008	7167309
chr12	7167546	7167763
chr12	7167990	7168238
chr12	7185475	7185711
chr12	7186392	7187416
chr12	7243041	7243433
chr12	7244262	7245265
chr12	7260240	7261843
chr12	7262027	7262164
chr12	7262288	7262359
chr12	7263126	7263325
chr12	7265959	7265962
chr12	7282095	7283783
chr12	7300418	7300519
chr12	7307908	7308282
chr12	7308585	7309038
chr12	7341346	7343181
chr12	7585861	7586023
chr12	7586169	7586231
chr12	7613450	7613469
chr12	7921156	7921437
chr12	7978255	7978439

chr12	7978743	7978864
chr12	8012787	8012986
chr12	8013408	8013623
chr12	8030849	8031105
chr12	8031304	8031465
chr12	8043756	8044200
chr12	8080576	8080799
chr12	8080914	8081022
chr12	8081332	8081481
chr12	8085776	8085902
chr12	8086128	8086180
chr12	8088635	8088823
chr12	8089045	8089245
chr12	8090640	8090908
chr12	8111400	8111592
chr12	8112171	8112406
chr12	8112645	8112767
chr12	8113057	8113683
chr12	8119151	8119672
chr12	8120943	8121158
chr12	8122778	8123009
chr12	8123137	8123258
chr12	8123578	8123890
chr12	8147351	8147499
chr12	8164261	8164356
chr12	8179045	8180268
chr12	8184270	8184492
chr12	8184739	8186103
chr12	8233835	8235415
chr12	8261674	8261756
chr12	8309534	8309809
chr12	8332776	8333307
chr12	8394447	8394716
chr12	8394980	8396912
chr12	8542787	8543279
chr12	8771700	8771801
chr12	8810115	8810413
chr12	8811060	8811148
chr12	8815237	8815691
chr12	8829231	8829311
chr12	8830989	8831302
chr12	8831617	8831828
chr12	8833626	8834806
chr12	8834932	8835215
chr12	8840375	8840534
chr12	8849754	8850340

chr12	8850574	8851398
chr12	8851631	8851830
chr12	8980123	8980255
chr12	8980564	8980585
chr12	8981138	8981398
chr12	9043423	9043719
chr12	9065811	9065948
chr12	9066582	9067586
chr12	9067817	9067993
chr12	9101418	9101631
chr12	9101898	9101966
chr12	9102115	9103150
chr12	9216868	9217679
chr12	9374170	9374200
chr12	9436179	9436213
chr12	9436672	9437524
chr12	9441891	9442168
chr12	9499829	9499991
chr12	9500238	9500333
chr12	9599535	9599750
chr12	9599814	9600363
chr12	9600557	9601413
chr12	9626791	9626939
chr12	9627061	9627146
chr12	9791457	9791555
chr12	9800450	9800786
chr12	9801268	9801517
chr12	9801946	9802388
chr12	9803277	9803695
chr12	9825967	9826418
chr12	9874036	9874328
chr12	9892301	9892500
chr12	9966491	9966546
chr12	9966672	9966927
chr12	10022175	10022429
chr12	10025234	10025349
chr12	10029560	10029693
chr12	10035893	10036092
chr12	10049519	10049648
chr12	10162502	10162546
chr12	10162745	10162840
chr12	10268587	10268730
chr12	10284726	10284973
chr12	10306074	10306457
chr12	10320329	10320437
chr12	10323138	10323223

chr12	10328605	10328815
chr12	10328881	10329194
chr12	10330899	10330968
chr12	10335310	10335360
chr12	10341661	10341993
chr12	10344397	10344468
chr12	10344810	10344906
chr12	10364446	10364918
chr12	10365036	10366402
chr12	10374462	10374610
chr12	10492061	10492390
chr12	10514259	10514590
chr12	10515577	10515776
chr12	10516373	10518441
chr12	10537929	10538280
chr12	10544933	10545410
chr12	10545558	10545620
chr12	10548021	10548397
chr12	10548532	10548739
chr12	10572976	10573211
chr12	10574707	10575201
chr12	10588587	10588843
chr12	10590461	10590591
chr12	10592247	10592542
chr12	10605758	10605907
chr12	10765332	10765726
chr12	10765898	10766458
chr12	10794357	10794595
chr12	10821034	10821226
chr12	10826370	10826858
chr12	10826961	10827223
chr12	10869249	10869409
chr12	10869574	10869754
chr12	10870003	10870125
chr12	10870305	10870513
chr12	10870874	10871487
chr12	10872188	10872387
chr12	10874271	10875978
chr12	10876164	10876424
chr12	10902546	10902794
chr12	10903065	10903134
chr12	11092945	11093070
chr12	11094835	11094851
chr12	11095174	11095317
chr12	11323591	11324171
chr12	11324340	11324485

chr12	11560939	11561164
chr12	11561731	11561772
chr12	11613352	11613377
chr12	11729460	11729526
chr12	11733870	11734059
chr12	11788272	11788681
chr12	11801615	11803353
chr12	11803596	11804147
chr12	11809536	11809871
chr12	11827629	11827828
chr12	11857931	11858381
chr12	11872616	11873074
chr12	11881915	11881975
chr12	11883943	11884209
chr12	11884401	11884744
chr12	11887332	11887388
chr12	11891429	11891606
chr12	11894364	11894404
chr12	11894549	11894736
chr12	11896741	11897005
chr12	11898832	11899007
chr12	11899131	11899534
chr12	11938616	11939015
chr12	11948390	11949528
chr12	11954786	11954904
chr12	11967332	11967343
chr12	11983184	11983265
chr12	11983477	11983770
chr12	12028473	12028729
chr12	12031342	12031586
chr12	12038335	12038807
chr12	12186010	12186321
chr12	12199992	12200135
chr12	12200360	12200559
chr12	12234567	12234586
chr12	12418696	12418895
chr12	12419200	12420548
chr12	12488517	12488664
chr12	12491448	12491784
chr12	12502634	12503336
chr12	12509702	12509874
chr12	12510076	12510681
chr12	12551074	12551534
chr12	12551658	12551671
chr12	12584089	12584149
chr12	12585326	12585575

chr12	12592484	12592651
chr12	12592846	12593009
chr12	12598616	12599208
chr12	12602478	12602739
chr12	12602895	12602958
chr12	12618254	12618330
chr12	12622239	12622429
chr12	12622539	12622754
chr12	12686182	12686354
chr12	12686530	12686669
chr12	12714409	12715442
chr12	12713701	12714317
chr12	12715572	12716145
chr12	12760164	12760400
chr12	12761817	12761869
chr12	12764339	12765715
chr12	12808797	12808873
chr12	12848907	12849103
chr12	12849231	12849341
chr12	12849496	12849905
chr12	12857490	12857650
chr12	12869162	12870040
chr12	12870255	12870968
chr12	12872986	12873275
chr12	12876488	12877533
chr12	12877753	12879175
chr12	12890373	12890420
chr12	12939493	12940579
chr12	12941966	12942084
chr12	12953343	12953738
chr12	12954551	12954810
chr12	12956050	12956124
chr12	12956450	12956632
chr12	12956926	12957508
chr12	12957672	12957919
chr12	12959284	12959382
chr12	12965953	12966035
chr12	12966275	12966535
chr12	12972998	12973284
chr12	12976453	12976627
chr12	12983603	12983632
chr12	12988115	12988981
chr12	12989980	12990423
chr12	12991128	12991689
chr12	12992677	12993068
chr12	12993215	12993335

chr12	12994411	12994829
chr12	13001776	13001971
chr12	13023370	13023394
chr12	13023669	13023781
chr12	13024998	13026287
chr12	13031937	13032136
chr12	13032309	13032377
chr12	13033241	13033805
chr12	13034381	13034546
chr12	13034733	13034897
chr12	13035661	13036955
chr12	13038132	13038369
chr12	13038673	13038756
chr12	13039708	13039718
chr12	13043340	13044289
chr12	13044600	13044800
chr12	13045300	13046170
chr12	13049292	13049399
chr12	13051232	13051755
chr12	13052127	13052449
chr12	13052892	13053217
chr12	13053943	13054793
chr12	13054998	13055335
chr12	13055596	13055876
chr12	13056995	13057216
chr12	13145361	13145755
chr12	13152779	13154658
chr12	13197415	13197729
chr12	13224230	13224427
chr12	13224546	13224711
chr12	13246449	13246479
chr12	13247439	13247645
chr12	13250987	13251863
chr12	13252318	13253500
chr12	13253596	13254587
chr12	13288577	13289118
chr12	13316985	13317205
chr12	13318326	13318870
chr12	13320050	13320526
chr12	13324752	13325122
chr12	13325271	13325380
chr12	13326437	13326851
chr12	13328321	13328541
chr12	13328771	13328871
chr12	13332451	13332813
chr12	13340786	13340837

chr12	13348834	13349372
chr12	13349643	13350346
chr12	13350447	13350728
chr12	13359236	13359769
chr12	13365055	13365066
chr12	13375242	13375497
chr12	13376007	13376113
chr12	13376464	13376612
chr12	13376676	13376933
chr12	13408913	13409098
chr12	14339172	14339275
chr12	14409488	14409830
chr12	14410418	14410502
chr12	14410977	14411467
chr12	14412912	14414044
chr12	14417780	14417834
chr12	14418045	14418110
chr12	14426304	14427361
chr12	14460312	14460780
chr12	14470213	14470722
chr12	14472861	14473069
chr12	14475552	14475675
chr12	14479458	14479648
chr12	14518207	14518309
chr12	14518471	14519039
chr12	14520284	14520483
chr12	14537911	14537932
chr12	14547335	14547673
chr12	14553086	14553170
chr12	14635356	14635578
chr12	14635866	14635949
chr12	14667046	14667257
chr12	14667412	14667498
chr12	14667908	14667964
chr12	14668207	14668286
chr12	14720203	14720743
chr12	14922287	14923534
chr12	14924171	14924223
chr12	14927246	14928497
chr12	14928609	14928842
chr12	14929411	14929746
chr12	14955885	14956490
chr12	14956720	14956862
chr12	14964062	14964164
chr12	14989591	14989826
chr12	14994726	14994849

chr12	14995044	14995144
chr12	15082130	15082317
chr12	15092541	15092543
chr12	15103925	15104044
chr12	15104274	15104433
chr12	15105147	15105661
chr12	15113702	15114552
chr12	15114803	15114946
chr12	15427587	15427711
chr12	15431772	15432119
chr12	15743517	15743648
chr12	15815489	15815619
chr12	15815808	15815917
chr12	15911114	15911214
chr12	15941117	15941499
chr12	15941651	15941668
chr12	15941844	15943060
chr12	16005604	16005654
chr12	16034681	16036066
chr12	16063921	16064038
chr12	16064150	16064702
chr12	16109306	16109388
chr12	16115680	16115752
chr12	16116049	16116101
chr12	16399159	16399252
chr12	16500285	16501624
chr12	16501814	16502398
chr12	16503497	16504038
chr12	16505552	16505800
chr12	16708584	16708730
chr12	16721895	16721942
chr12	16722077	16722185
chr12	16736796	16736907
chr12	16759319	16759581
chr12	16765627	16765702
chr12	16765889	16765969
chr12	18081927	18082338
chr12	18632136	18632173
chr12	18632638	18632659
chr12	18836516	18837007
chr12	18843522	18843564
chr12	18843978	18844048
chr12	18876631	18877146
chr12	19014298	19014514
chr12	19152715	19152948
chr12	19249947	19250057

chr12	19250288	19250457
chr12	19282641	19284120
chr12	19284303	19284417
chr12	19351427	19351613
chr12	19353272	19353304
chr12	19353487	19353521
chr12	19402466	19402494
chr12	19427178	19427232
chr12	19452118	19452245
chr12	19452408	19452518
chr12	19452713	19452856
chr12	19453027	19453126
chr12	19458232	19458491
chr12	19462705	19462733
chr12	19534929	19535012
chr12	19535227	19535481
chr12	19549330	19549990
chr12	19550251	19550550
chr12	19550721	19550953
chr12	19553801	19554232
chr12	19555661	19555818
chr12	19584741	19585207
chr12	19592089	19592272
chr12	19592486	19593546
chr12	19593749	19593795
chr12	19635550	19636312
chr12	19636746	19636804
chr12	19636961	19637102
chr12	19638225	19638584
chr12	19658723	19658869
chr12	19683598	19683650
chr12	19683878	19683918
chr12	19705825	19705918
chr12	19707399	19707591
chr12	19727504	19727513
chr12	19773121	19773256
chr12	19796843	19796988
chr12	19836206	19836244
chr12	19842730	19842774
chr12	19843061	19843136
chr12	21590366	21590432
chr12	21590559	21591353
chr12	21654749	21655103
chr12	21655264	21655380
chr12	21680520	21680782
chr12	21712748	21712758

chr12	21713051	21713118
chr12	21796096	21796108
chr12	21809780	21811356
chr12	22195961	22195985
chr12	22199107	22200116
chr12	22254598	22254646
chr12	22255283	22255354
chr12	22283968	22284077
chr12	22407907	22408354
chr12	22410286	22410395
chr12	22423899	22423998
chr12	22424215	22424279
chr12	22428562	22428855
chr12	22488139	22488420
chr12	22563344	22563514
chr12	22563877	22563898
chr12	22670996	22671010
chr12	22682240	22682581
chr12	22696354	22697485
chr12	22697637	22697879
chr12	22777569	22779003
chr12	23128077	23128189
chr12	24379854	24380034
chr12	24397693	24397946
chr12	24714694	24714773
chr12	24716107	24716395
chr12	24716927	24717072
chr12	24907805	24907888
chr12	25006539	25006892
chr12	25051540	25051832
chr12	25053877	25053893
chr12	25054108	25054287
chr12	25055091	25055182
chr12	25055288	25055763
chr12	25055929	25056128
chr12	25075123	25075448
chr12	25085249	25085329
chr12	25099807	25100255
chr12	25101294	25101654
chr12	25101996	25102323
chr12	25144432	25144521
chr12	25207049	25207127
chr12	25207362	25207432
chr12	25207733	25207841
chr12	25288300	25288777
chr12	25304388	25304498

chr12	25348172	25348572
chr12	25403085	25404407
chr12	25429043	25429150
chr12	25429256	25429461
chr12	25437144	25437376
chr12	25465783	25465929
chr12	25486413	25486819
chr12	25487014	25487227
chr12	25525906	25525998
chr12	25526118	25526355
chr12	25538331	25539238
chr12	25539518	25540756
chr12	25611115	25611637
chr12	25738129	25738186
chr12	25738449	25738535
chr12	25864212	25864243
chr12	25864475	25864562
chr12	25923978	25924064
chr12	25924327	25924395
chr12	26044084	26044379
chr12	26077875	26078056
chr12	26111107	26111464
chr12	26111599	26111908
chr12	26112099	26112480
chr12	26112553	26112736
chr12	26112862	26112958
chr12	26170377	26170524
chr12	26250304	26250340
chr12	26266442	26266502
chr12	26266810	26267735
chr12	26275159	26275234
chr12	26275343	26275852
chr12	26276262	26276461
chr12	26277635	26278414
chr12	26278505	26278956
chr12	26279113	26279656
chr12	26279832	26279844
chr12	26306238	26306339
chr12	26306763	26306866
chr12	26348590	26349320
chr12	26379404	26379696
chr12	26417416	26417668
chr12	26424813	26425036
chr12	26425168	26425337
chr12	26427487	26427654
chr12	26427832	26428142

chr12	26451905	26452437
chr12	26470903	26471967
chr12	26472336	26472564
chr12	26472903	26473054
chr12	26527056	26527469
chr12	26646550	26646658
chr12	26646945	26647030
chr12	26649043	26649113
chr12	26649486	26649555
chr12	26676864	26677099
chr12	26781130	26781242
chr12	26902792	26902841
chr12	26984878	26986286
chr12	26986414	26987099
chr12	26998212	26998408
chr12	27006589	27006814
chr12	27007002	27007111
chr12	27024391	27024418
chr12	27090666	27091309
chr12	27091418	27092077
chr12	27166549	27167916
chr12	27174827	27175816
chr12	27202032	27202084
chr12	27202337	27202383
chr12	27230141	27230228
chr12	27233082	27233233
chr12	27233455	27233540
chr12	27234273	27234621
chr12	27234923	27235108
chr12	27241005	27241272
chr12	27265421	27265554
chr12	27295056	27295176
chr12	27295323	27295399
chr12	27330620	27330998
chr12	27332717	27332789
chr12	27333052	27333145
chr12	27373627	27373699
chr12	27373819	27373950
chr12	27378740	27379509
chr12	27396760	27396928
chr12	27397160	27397476
chr12	27397656	27397830
chr12	27472384	27472459
chr12	27482384	27482622
chr12	27484091	27484253
chr12	27484390	27484749

chr12	27484887	27486774
chr12	27488217	27488564
chr12	27489043	27489810
chr12	27495651	27495850
chr12	27496471	27496978
chr12	27500591	27500655
chr12	27501031	27501161
chr12	27536604	27537087
chr12	27676474	27677338
chr12	27677513	27677615
chr12	27678357	27678607
chr12	27694375	27694697
chr12	27695590	27696001
chr12	27702096	27702544
chr12	27702632	27703100
chr12	27717724	27717863
chr12	27733755	27734131
chr12	27742200	27742745
chr12	27744563	27744693
chr12	27744806	27745087
chr12	27745455	27746425
chr12	27746544	27746683
chr12	27763212	27763399
chr12	27763569	27763682
chr12	27772239	27772402
chr12	27779620	27779772
chr12	27780156	27780497
chr12	27795604	27795650
chr12	27863091	27863151
chr12	27863301	27863398
chr12	27863479	27864590
chr12	27900907	27901089
chr12	27901207	27901370
chr12	27904379	27905196
chr12	27932623	27934130
chr12	27934413	27934419
chr12	27938382	27939482
chr12	27974135	27974534
chr12	27974896	27975699
chr12	28052451	28052678
chr12	28075494	28075498
chr12	28119141	28119218
chr12	28119883	28120228
chr12	28121759	28122016
chr12	28122413	28122601
chr12	28122816	28123160

chr12	28123302	28124427
chr12	28125672	28125749
chr12	28126499	28127125
chr12	28127653	28129535
chr12	28152344	28152954
chr12	28174637	28175024
chr12	28176335	28176879
chr12	28177220	28177430
chr12	28179706	28179944
chr12	28185640	28186405
chr12	28186604	28186861
chr12	28241654	28241866
chr12	28283563	28283780
chr12	28283883	28284064
chr12	28325716	28325784
chr12	28343096	28344274
chr12	28344587	28344724
chr12	28650956	28651152
chr12	28689065	28689126
chr12	28731655	28731864
chr12	29090491	29090523
chr12	29301807	29302372
chr12	29533452	29534101
chr12	29534320	29534529
chr12	29543630	29543694
chr12	29769952	29770039
chr12	29794038	29794355
chr12	29894384	29894880
chr12	29896702	29896947
chr12	29897099	29897252
chr12	29909802	29909848
chr12	29910090	29910451
chr12	29935124	29935301
chr12	29935723	29937372
chr12	30569567	30569615
chr12	30569784	30569857
chr12	30570472	30570501
chr12	30570870	30571109
chr12	30844038	30844181
chr12	30847832	30847838
chr12	30848156	30849152
chr12	30879077	30879160
chr12	30906868	30908318
chr12	30947006	30947238
chr12	30947436	30947878
chr12	30948012	30950435

chr12	30960466	30960736
chr12	30973394	30973486
chr12	30975210	30976227
chr12	31004369	31004723
chr12	31225943	31226091
chr12	31226199	31226654
chr12	31226797	31227105
chr12	31227271	31228072
chr12	31231455	31232114
chr12	31232442	31232701
chr12	31233223	31233524
chr12	31233673	31233968
chr12	31256316	31257094
chr12	31270341	31270746
chr12	31399005	31399174
chr12	31405051	31405240
chr12	31475932	31476143
chr12	31476546	31479035
chr12	31479471	31479473
chr12	31556299	31556491
chr12	31589384	31589928
chr12	31644704	31645015
chr12	31645267	31645607
chr12	31660965	31661028
chr12	31671612	31671770
chr12	31672047	31672176
chr12	31677827	31678026
chr12	31727224	31727649
chr12	31735084	31735250
chr12	31738505	31738537
chr12	31742656	31744292
chr12	31800399	31800551
chr12	31806106	31806244
chr12	31806483	31806547
chr12	31811854	31812005
chr12	31812439	31812741
chr12	31812828	31813176
chr12	31881286	31881851
chr12	31882186	31882620
chr12	31885785	31887051
chr12	31888722	31888757
chr12	31888938	31889185
chr12	31889331	31889714
chr12	31897358	31897722
chr12	31901932	31903016
chr12	31904439	31904735

chr12	31909866	31910383
chr12	31911240	31911269
chr12	31916027	31916307
chr12	31916429	31916449
chr12	31972289	31972430
chr12	32111960	32112967
chr12	32142861	32143017
chr12	32143204	32143403
chr12	32204358	32204485
chr12	32213454	32213596
chr12	32260421	32260648
chr12	32260732	32261199
chr12	32259307	32260341
chr12	32268524	32268651
chr12	32268945	32269110
chr12	32269382	32269581
chr12	32292545	32292749
chr12	32292813	32292983
chr12	32543652	32544235
chr12	32551747	32552850
chr12	32553053	32553534
chr12	32589824	32590524
chr12	32592957	32593100
chr12	32593315	32594083
chr12	32596436	32596588
chr12	32597845	32597890
chr12	32598046	32598161
chr12	32601566	32601847
chr12	32606961	32607054
chr12	32607210	32607319
chr12	32655913	32655953
chr12	32687281	32687436
chr12	32688156	32688265
chr12	32688522	32688568
chr12	32719882	32719990
chr12	32721120	32721580
chr12	32738656	32738931
chr12	32803806	32803856
chr12	32810492	32810590
chr12	32829184	32829417
chr12	32830724	32831212
chr12	32831848	32831995
chr12	32832264	32833060
chr12	32869262	32869496
chr12	32871752	32872355
chr12	32907777	32909412

chr12	32937067	32937180
chr12	32974596	32974620
chr12	33041860	33042218
chr12	33042456	33042879
chr12	33044828	33044863
chr12	33045082	33045668
chr12	33048589	33049098
chr12	33049251	33050451
chr12	33054319	33054772
chr12	33071995	33072680
chr12	33073288	33073960
chr12	34120687	34120823
chr12	34142172	34142255
chr12	34148238	34148680
chr12	34175112	34175163
chr12	34175450	34175841
chr12	34831608	34831955
chr12	34833332	34833665
chr12	34837477	34837844
chr12	34838490	34838856
chr12	34841298	34842308
chr12	34846167	34846547
chr12	34846778	34847121
chr12	34847771	34847860
chr12	34850758	34851100
chr12	34852534	34852875
chr12	34853966	34854077
chr12	38016274	38016537
chr12	38027299	38027557
chr12	38028919	38029293
chr12	38030864	38030912
chr12	38033608	38033895
chr12	38532348	38532455
chr12	38538651	38538882
chr12	38677117	38677133
chr12	38683235	38683304
chr12	38710232	38710260
chr12	38710553	38710962
chr12	38778617	38779218
chr12	38780071	38780441
chr12	38786282	38786444
chr12	38786722	38786809
chr12	38941375	38941772
chr12	39298549	39298754
chr12	39298892	39300428
chr12	39300840	39300906

chr12	39461860	39461970
chr12	39496288	39496298
chr12	39496729	39496744
chr12	39723050	39723133
chr12	39820455	39820676
chr12	39836096	39837579
chr12	39838757	39839012
chr12	39897583	39897635
chr12	39928809	39929016
chr12	39999849	39999964
chr12	40013510	40013636
chr12	40013831	40014055
chr12	40405238	40405321
chr12	40499332	40499782
chr12	40552600	40553079
chr12	40618300	40618853
chr12	41084151	41084925
chr12	41085764	41086143
chr12	41086427	41087218
chr12	41087640	41087734
chr12	41091465	41092275
chr12	41223422	41223781
chr12	41286361	41286612
chr12	41300813	41301442
chr12	41352826	41353187
chr12	41879037	41879334
chr12	41916002	41916107
chr12	42488020	42488431
chr12	42524503	42524659
chr12	42524767	42524863
chr12	42529349	42529470
chr12	42537256	42537535
chr12	42537847	42538716
chr12	42538984	42539037
chr12	42631398	42632742
chr12	42719476	42719975
chr12	42720194	42720253
chr12	42720507	42720865
chr12	42820529	42820711
chr12	42820863	42820927
chr12	42876533	42876595
chr12	42877599	42877668
chr12	42876886	42877470
chr12	42877934	42878166
chr12	42983182	42983530
chr12	43355095	43355289

chr12	43355464	43355614
chr12	43699671	43699865
chr12	44152162	44152525
chr12	44152862	44153583
chr12	44199479	44200417
chr12	44229440	44230292
chr12	45609079	45610846
chr12	45613331	45613528
chr12	45614134	45614246
chr12	45626979	45627141
chr12	45628236	45629011
chr12	45629128	45629887
chr12	45632019	45632163
chr12	45662459	45662586
chr12	45683938	45684299
chr12	45692879	45692886
chr12	45693050	45693240
chr12	45730812	45730940
chr12	45773375	45773493
chr12	46006084	46006162
chr12	46038330	46038626
chr12	46069525	46069540
chr12	46121106	46121939
chr12	46122377	46124096
chr12	46124413	46124691
chr12	46126025	46126352
chr12	46156257	46156368
chr12	46253670	46253875
chr12	46254068	46254101
chr12	46382228	46382342
chr12	46383445	46384540
chr12	46384749	46386065
chr12	46519200	46519307
chr12	46553853	46553979
chr12	46554234	46554782
chr12	46557994	46558393
chr12	46603991	46604244
chr12	46654863	46655033
chr12	46655191	46655302
chr12	46658028	46658311
chr12	46660218	46660545
chr12	46661061	46661530
chr12	46662245	46663870
chr12	46661661	46662134
chr12	46664062	46664142
chr12	46750386	46751331

chr12	46752144	46752151
chr12	46761457	46761803
chr12	46764874	46766190
chr12	46766319	46766882
chr12	46767089	46767572
chr12	46776731	46778246
chr12	46778814	46779089
chr12	46779256	46779551
chr12	46780304	46780524
chr12	46786378	46786520
chr12	46786689	46786831
chr12	46824125	46824540
chr12	46825412	46825879
chr12	46832738	46832812
chr12	46835987	46836327
chr12	46840491	46840614
chr12	46844335	46844524
chr12	46845992	46846393
chr12	46847226	46847620
chr12	46849553	46849975
chr12	46862002	46862144
chr12	46862301	46862397
chr12	46867333	46867522
chr12	46867656	46867825
chr12	46886325	46886743
chr12	46886844	46887226
chr12	46917230	46917263
chr12	46917883	46918190
chr12	46941462	46942143
chr12	46942630	46942990
chr12	46943450	46944161
chr12	46947157	46947572
chr12	46949277	46949584
chr12	46949874	46950267
chr12	46950618	46951017
chr12	46951345	46951570
chr12	46953180	46953520
chr12	46956033	46956271
chr12	46956628	46957020
chr12	47051829	47052030
chr12	47054125	47054455
chr12	47054648	47055300
chr12	47055449	47056186
chr12	47056953	47057126
chr12	47058245	47058869
chr12	47059056	47059490

chr12	47059955	47060265
chr12	47068645	47068739
chr12	47068966	47069090
chr12	47070619	47070984
chr12	47071548	47071889
chr12	47072221	47072617
chr12	47096368	47096795
chr12	47097001	47097213
chr12	47224750	47224949
chr12	47302184	47302490
chr12	47302700	47303386
chr12	47336733	47336796
chr12	47357701	47357777
chr12	47358071	47358153
chr12	47358288	47358684
chr12	47411646	47411732
chr12	47416150	47416191
chr12	47415425	47415448
chr12	47416400	47416464
chr12	47417740	47417873
chr12	47426804	47426863
chr12	47427135	47427177
chr12	47472513	47472593
chr12	47472777	47472917
chr12	47473115	47474896
chr12	47476537	47477352
chr12	47478593	47479308
chr12	47479557	47479987
chr12	47481813	47482698
chr12	47488582	47488981
chr12	47489730	47489828
chr12	47489991	47490014
chr12	47578871	47578976
chr12	47605914	47606183
chr12	47606510	47606595
chr12	47614519	47614566
chr12	47614865	47614866
chr12	47630107	47630499
chr12	47642868	47643957
chr12	47661297	47661460
chr12	47661593	47661863
chr12	47664998	47665438
chr12	47670498	47671049
chr12	47671839	47672228
chr12	47673568	47673777
chr12	47697897	47698329

chr12	47701513	47701632
chr12	47786812	47787218
chr12	48045451	48045540
chr12	48080480	48080542
chr12	48099219	48099818
chr12	48099980	48100079
chr12	48110006	48110205
chr12	48142609	48143011
chr12	48144930	48145894
chr12	48146100	48148152
chr12	48148610	48148862
chr12	48148984	48149085
chr12	48151732	48153582
chr12	48155919	48156120
chr12	48156960	48157548
chr12	48157672	48157959
chr12	48158225	48158820
chr12	48159047	48159450
chr12	48161175	48161279
chr12	48161485	48161745
chr12	48162041	48162298
chr12	48162450	48162706
chr12	48163847	48164205
chr12	48166176	48166561
chr12	48166702	48166797
chr12	48167017	48167855
chr12	48169430	48169692
chr12	48170064	48170263
chr12	48174392	48174647
chr12	48175175	48175371
chr12	48175457	48175741
chr12	48176046	48176711
chr12	48200763	48200963
chr12	48203363	48203733
chr12	48203941	48204140
chr12	48202422	48203174
chr12	48204297	48204324
chr12	48204556	48204962
chr12	48205880	48206030
chr12	48206220	48208193
chr12	48208917	48209252
chr12	48209707	48209865
chr12	48210531	48210730
chr12	48212388	48215216
chr12	48217497	48217916
chr12	48218828	48219015

chr12	48218041	48218306
chr12	48219142	48219391
chr12	48219503	48219587
chr12	48220092	48220236
chr12	48221354	48221472
chr12	48222176	48223544
chr12	48224787	48224886
chr12	48229051	48229630
chr12	48230354	48230547
chr12	48257005	48257332
chr12	48257546	48258168
chr12	48258430	48258567
chr12	48270146	48270931
chr12	48277229	48277380
chr12	48290838	48290958
chr12	48291270	48291591
chr12	48296074	48296300
chr12	48297031	48298769
chr12	48298893	48299664
chr12	48339975	48340922
chr12	48342901	48343873
chr12	48344092	48344395
chr12	48370844	48371115
chr12	48395106	48395395
chr12	48396642	48397073
chr12	48472680	48473082
chr12	48498446	48498618
chr12	48499311	48500313
chr12	48512619	48513702
chr12	48550499	48550681
chr12	48550875	48551296
chr12	48551455	48551780
chr12	48583616	48583711
chr12	48591235	48591964
chr12	48592149	48592414
chr12	48592529	48592804
chr12	48690264	48690378
chr12	48690583	48690772
chr12	48743949	48745312
chr12	48880055	48880881
chr12	48882726	48883650
chr12	48974937	48975013
chr12	49065217	49065699
chr12	49075168	49076040
chr12	49076268	49076532
chr12	49110066	49111066

chr12	49137972	49138087
chr12	49147682	49147860
chr12	49182856	49183452
chr12	49188225	49188499
chr12	49188982	49189127
chr12	49207757	49207949
chr12	49208153	49208705
chr12	49208809	49209873
chr12	49211849	49213037
chr12	49219630	49219679
chr12	49245665	49245945
chr12	49246205	49246441
chr12	49246933	49247175
chr12	49259905	49260025
chr12	49261138	49261193
chr12	49300119	49300318
chr12	49318310	49319267
chr12	49350045	49350316
chr12	49350465	49351695
chr12	49352047	49352258
chr12	49364859	49365166
chr12	49392052	49392480
chr12	49392591	49392937
chr12	49394217	49394247
chr12	49411684	49411878
chr12	49412002	49412596
chr12	49412754	49413109
chr12	49413265	49413572
chr12	49443716	49444104
chr12	49451864	49452668
chr12	49452881	49453330
chr12	49453433	49455222
chr12	49462932	49464139
chr12	49479151	49479156
chr12	49483817	49484016
chr12	49499607	49499866
chr12	49503884	49504537
chr12	49504863	49505079
chr12	49505144	49505347
chr12	49516690	49516854
chr12	49517094	49517234
chr12	49523348	49523547
chr12	49523912	49524590
chr12	49524721	49525176
chr12	49525578	49526018
chr12	49581725	49581972

chr12	49595222	49595391
chr12	49595608	49595735
chr12	49616427	49616623
chr12	49616852	49617053
chr12	49619090	49619289
chr12	49627826	49627916
chr12	49628122	49628871
chr12	49628949	49629021
chr12	49629136	49629395
chr12	49634692	49634963
chr12	49640203	49640462
chr12	49641234	49641430
chr12	49649234	49649782
chr12	49653927	49654115
chr12	49657897	49659680
chr12	49659919	49660125
chr12	49661232	49661596
chr12	49661900	49662275
chr12	49662357	49662529
chr12	49662673	49662883
chr12	49662951	49663426
chr12	49683295	49683503
chr12	49687697	49687946
chr12	49691414	49691902
chr12	49709773	49710046
chr12	49715958	49716396
chr12	49716525	49716775
chr12	49717043	49717561
chr12	49736559	49736815
chr12	49738861	49739278
chr12	49739978	49740450
chr12	49741124	49741323
chr12	49741541	49741779
chr12	49753560	49753608
chr12	49758729	49759221
chr12	49760130	49761845
chr12	49763686	49763986
chr12	49797077	49797157
chr12	49797804	49798028
chr12	49802255	49802329
chr12	49806802	49806828
chr12	49822963	49822987
chr12	49876134	49876350
chr12	49876563	49876906
chr12	49881475	49881717
chr12	49884784	49885495

chr12	49885606	49886063
chr12	49896126	49896532
chr12	49897196	49897924
chr12	49899473	49899830
chr12	49900900	49901371
chr12	49903833	49904034
chr12	49907800	49908210
chr12	49909235	49909419
chr12	49928176	49928213
chr12	49928338	49928436
chr12	49931701	49932009
chr12	49932800	49933216
chr12	49946924	49947166
chr12	49947269	49947276
chr12	49948865	49949285
chr12	49960848	49961163
chr12	49961296	49962758
chr12	50016019	50016918
chr12	50017114	50018163
chr12	50018311	50018339
chr12	50018604	50018824
chr12	50018979	50019204
chr12	50035571	50035970
chr12	50036090	50036340
chr12	50037642	50037757
chr12	50038202	50038437
chr12	50056736	50057442
chr12	50100292	50101365
chr12	50132672	50132840
chr12	50134843	50134999
chr12	50135315	50136216
chr12	50144138	50144280
chr12	50170144	50171016
chr12	50171176	50171241
chr12	50172269	50172313
chr12	50178448	50178655
chr12	50182133	50182328
chr12	50221048	50221305
chr12	50221409	50221737
chr12	50222011	50222164
chr12	50222307	50222514
chr12	50222653	50222954
chr12	50226861	50226960
chr12	50235780	50236120
chr12	50236355	50236679
chr12	50236903	50237213

chr12	50265365	50265368
chr12	50265530	50265724
chr12	50265973	50266272
chr12	50331412	50331598
chr12	50353385	50353634
chr12	50360990	50361285
chr12	50361570	50361952
chr12	50387299	50387382
chr12	50418635	50418711
chr12	50418840	50419219
chr12	50419486	50419525
chr12	50432634	50433123
chr12	50433337	50433540
chr12	50433720	50433772
chr12	50449467	50450177
chr12	50450651	50451188
chr12	50451320	50451907
chr12	50469210	50469409
chr12	50478063	50478537
chr12	50478607	50479703
chr12	50482402	50482809
chr12	50504101	50504382
chr12	50505170	50505369
chr12	50505942	50506638
chr12	50507315	50507653
chr12	50555345	50555544
chr12	50559850	50560106
chr12	50560216	50561168
chr12	50561464	50561661
chr12	50564117	50564201
chr12	50564486	50564528
chr12	50579963	50580154
chr12	50580466	50580703
chr12	50582168	50582602
chr12	50595721	50596173
chr12	50596311	50596451
chr12	50597201	50597449
chr12	50602779	50603229
chr12	50604239	50604440
chr12	50606562	50606850
chr12	50608327	50608557
chr12	50608876	50609363
chr12	50611013	50611368
chr12	50612120	50612654
chr12	50612886	50613232
chr12	50613943	50614549

chr12	50614976	50615447
chr12	50616012	50616639
chr12	50617743	50617988
chr12	50618080	50618239
chr12	50618692	50618791
chr12	50628099	50628636
chr12	50640596	50640980
chr12	50641313	50641448
chr12	50648234	50648744
chr12	50651341	50651400
chr12	50651521	50651720
chr12	50652000	50652329
chr12	50655391	50655594
chr12	50659508	50659649
chr12	50659818	50659977
chr12	50676920	50677412
chr12	50677518	50677720
chr12	50697927	50698038
chr12	50706297	50706638
chr12	50707190	50707447
chr12	50707650	50707849
chr12	50742887	50743171
chr12	50774217	50774272
chr12	50774417	50774845
chr12	50783912	50784160
chr12	50784490	50784526
chr12	50787655	50787856
chr12	50788220	50788419
chr12	50789469	50789804
chr12	50790332	50790350
chr12	50790539	50790779
chr12	50794304	50794480
chr12	50794702	50795575
chr12	50798829	50799003
chr12	50804872	50805198
chr12	50812193	50812194
chr12	50812302	50812665
chr12	50813307	50813635
chr12	50817633	50817932
chr12	50819555	50819700
chr12	50898115	50899481
chr12	50902623	50902832
chr12	50903021	50903119
chr12	50926189	50927025
chr12	50930893	50931348
chr12	50931456	50931739

chr12	50935759	50935980
chr12	50936114	50936134
chr12	50936371	50936536
chr12	50944497	50944652
chr12	50950545	50950892
chr12	50966114	50966215
chr12	50991517	50991688
chr12	50992303	50992509
chr12	50992864	50993167
chr12	51008035	51008289
chr12	51009612	51010108
chr12	51012938	51013463
chr12	51013786	51013985
chr12	51014630	51014789
chr12	51035295	51035701
chr12	51036502	51036700
chr12	51035823	51036202
chr12	51036907	51036936
chr12	51126353	51126955
chr12	51152719	51152788
chr12	51156957	51157717
chr12	51157872	51158704
chr12	51158919	51159353
chr12	51162626	51162891
chr12	51177825	51178142
chr12	51178813	51179017
chr12	51179468	51179672
chr12	51180410	51181079
chr12	51218767	51218977
chr12	51318314	51318656
chr12	51329226	51329426
chr12	51405421	51405496
chr12	51418026	51418672
chr12	51418797	51419043
chr12	51419405	51420567
chr12	51421776	51421978
chr12	51422163	51422747
chr12	51434142	51434223
chr12	51441464	51442312
chr12	51442667	51442762
chr12	51449965	51450454
chr12	51452446	51452581
chr12	51463707	51464367
chr12	51471770	51471777
chr12	51476543	51477409
chr12	51477539	51477704

chr12	51512457	51512548
chr12	51512871	51512932
chr12	51520986	51521496
chr12	51527151	51527316
chr12	51527421	51527584
chr12	51598573	51598929
chr12	51601914	51602075
chr12	51611056	51613040
chr12	51631861	51632194
chr12	51632318	51632413
chr12	51632583	51633293
chr12	51633564	51633627
chr12	51656843	51656847
chr12	51659888	51659939
chr12	51660206	51660264
chr12	51662620	51662856
chr12	51663054	51665141
chr12	51675190	51675489
chr12	51694747	51694879
chr12	51701981	51702046
chr12	51720581	51721415
chr12	51724987	51725043
chr12	51765033	51765369
chr12	51765538	51765767
chr12	51765892	51766285
chr12	51767368	51767573
chr12	51781429	51782147
chr12	51782861	51782864
chr12	51783510	51783709
chr12	51784032	51784277
chr12	51784535	51786801
chr12	51787819	51788076
chr12	51788228	51788411
chr12	51984463	51984679
chr12	51985193	51985452
chr12	52030629	52030699
chr12	52207724	52208819
chr12	52213712	52214125
chr12	52214269	52215778
chr12	52262945	52263150
chr12	52279676	52279801
chr12	52286379	52286578
chr12	52334832	52335140
chr12	52335393	52335614
chr12	52335819	52335870
chr12	52336089	52336178

chr12	52344812	52345149
chr12	52345611	52346465
chr12	52348584	52349139
chr12	52364839	52365215
chr12	52365369	52365392
chr12	52365968	52366376
chr12	52416175	52416385
chr12	52417000	52417561
chr12	52419118	52419230
chr12	52425614	52425820
chr12	52426149	52426521
chr12	52427099	52427453
chr12	52430339	52430680
chr12	52430995	52431666
chr12	52444174	52444909
chr12	52445171	52445517
chr12	52445822	52446383
chr12	52462977	52464529
chr12	52533199	52533286
chr12	52533725	52533747
chr12	52533817	52534202
chr12	52534910	52535011
chr12	52536143	52536395
chr12	52536846	52541171
chr12	52547850	52549657
chr12	52549748	52551550
chr12	52551717	52552190
chr12	52541296	52547743
chr12	52553672	52553960
chr12	52554709	52554921
chr12	52555341	52555693
chr12	52556374	52556573
chr12	52556793	52556907
chr12	52557619	52559225
chr12	52559444	52559718
chr12	52565934	52566373
chr12	52569157	52570662
chr12	52573031	52573230
chr12	52575234	52575494
chr12	52575712	52575997
chr12	52576304	52576427
chr12	52576677	52577449
chr12	52577628	52578139
chr12	52579549	52580095
chr12	52580476	52581021
chr12	52581221	52581349

chr12	52583690	52583889
chr12	52584629	52585011
chr12	52585464	52587116
chr12	52591991	52592686
chr12	52592975	52593636
chr12	52597182	52597887
chr12	52601541	52601736
chr12	52602288	52602861
chr12	52603196	52603483
chr12	52604084	52604427
chr12	52604633	52605155
chr12	52605448	52605647
chr12	52606699	52607310
chr12	52608088	52608287
chr12	52608579	52608706
chr12	52608927	52609217
chr12	52609687	52609915
chr12	52611351	52611499
chr12	52622823	52622985
chr12	52623707	52624549
chr12	52629691	52630347
chr12	52624708	52629583
chr12	52630981	52631101
chr12	52632361	52632871
chr12	52633289	52633650
chr12	52634123	52634362
chr12	52634600	52634627
chr12	52636316	52636404
chr12	52636579	52636773
chr12	52636907	52637348
chr12	52637545	52638014
chr12	52638121	52638824
chr12	52639674	52639780
chr12	52640329	52640741
chr12	52641032	52641310
chr12	52642870	52643350
chr12	52664215	52664348
chr12	52664626	52664792
chr12	52672478	52673678
chr12	52673933	52674715
chr12	52676139	52676451
chr12	52674826	52675347
chr12	52677801	52677853
chr12	52678102	52678301
chr12	52701842	52703044
chr12	52712857	52713100

chr12	52721008	52721063
chr12	52721155	52721469
chr12	52721658	52721779
chr12	52732749	52732859
chr12	52739230	52740048
chr12	52765751	52765776
chr12	52776024	52776124
chr12	52783207	52784139
chr12	52784345	52784456
chr12	52784724	52785549
chr12	52786067	52786537
chr12	52786733	52787040
chr12	52809345	52809478
chr12	52809564	52809672
chr12	52809802	52810156
chr12	52810531	52810751
chr12	52811246	52811409
chr12	52817090	52817625
chr12	52827917	52828752
chr12	52829284	52829364
chr12	52829767	52829902
chr12	52830265	52830641
chr12	52832234	52832478
chr12	52833037	52833075
chr12	52834096	52834175
chr12	52834597	52835786
chr12	52836212	52836672
chr12	52836894	52837009
chr12	52845311	52845487
chr12	52845557	52846375
chr12	52849603	52849763
chr12	52849893	52849992
chr12	52867205	52867673
chr12	52867828	52867970
chr12	52885099	52885635
chr12	52886152	52886490
chr12	52886631	52887467
chr12	52887708	52888803
chr12	52889139	52889565
chr12	52890044	52891107
chr12	52900934	52900998
chr12	52901187	52901241
chr12	52902324	52902408
chr12	52906408	52906754
chr12	52907320	52907718
chr12	52907998	52909502

chr12	52910009	52910986
chr12	52911254	52911924
chr12	52912452	52913567
chr12	52913642	52918315
chr12	52918543	52920094
chr12	52920249	52920499
chr12	52920649	52920655
chr12	52928621	52928925
chr12	52932561	52932921
chr12	52933079	52933612
chr12	52933724	52933755
chr12	52933983	52934246
chr12	52973370	52974240
chr12	52974812	52975836
chr12	52976028	52976362
chr12	52976529	52976916
chr12	53241856	53242093
chr12	53253681	53254363
chr12	53260513	53260741
chr12	53261048	53261053
chr12	53261281	53262300
chr12	53262756	53263472
chr12	53263686	53263849
chr12	53263987	53265217
chr12	53265371	53266122
chr12	53266419	53267070
chr12	53267329	53268834
chr12	53269793	53270931
chr12	53271054	53272176
chr12	53272248	53273827
chr12	53274045	53274249
chr12	53278315	53278376
chr12	53278592	53278675
chr12	53278902	53279003
chr12	53296275	53296494
chr12	53297162	53297625
chr12	53297793	53298803
chr12	53298925	53299568
chr12	53299805	53300069
chr12	53300545	53302573
chr12	53317892	53318339
chr12	53318579	53318898
chr12	53319028	53319265
chr12	53319516	53320038
chr12	53320823	53321055
chr12	53321571	53321966

chr12	53339535	53340015
chr12	53340583	53340782
chr12	53341835	53344183
chr12	53358458	53358747
chr12	53359054	53359606
chr12	53365294	53366193
chr12	53366404	53366670
chr12	53368531	53368928
chr12	53369203	53369407
chr12	53374560	53374681
chr12	53386960	53386977
chr12	53399216	53399597
chr12	53399703	53399895
chr12	53400120	53400440
chr12	53400507	53401008
chr12	53432705	53432855
chr12	53439496	53441044
chr12	53441241	53442829
chr12	53443037	53445008
chr12	53446757	53447534
chr12	53447668	53448950
chr12	53464037	53464236
chr12	53465144	53465579
chr12	53472308	53473909
chr12	53474580	53474779
chr12	53491005	53491210
chr12	53491364	53491820
chr12	53492005	53492481
chr12	53492857	53493292
chr12	53568759	53568958
chr12	53569911	53570141
chr12	53573584	53574183
chr12	53574413	53575169
chr12	53613057	53613256
chr12	53613703	53614161
chr12	53614847	53615827
chr12	53616954	53617849
chr12	53618012	53618781
chr12	53618854	53619370
chr12	53619493	53619847
chr12	53624713	53625969
chr12	53626255	53627074
chr12	53644859	53645753
chr12	53645882	53646244
chr12	53651034	53651233
chr12	53661480	53662500

chr12	53666927	53667099
chr12	53688431	53688543
chr12	53688819	53689163
chr12	53689331	53689917
chr12	53692922	53693315
chr12	53693510	53694404
chr12	53714764	53715641
chr12	53717845	53719335
chr12	53719527	53719736
chr12	53719895	53720121
chr12	53738712	53739170
chr12	53742255	53742813
chr12	53743041	53743240
chr12	53745940	53746139
chr12	53746778	53746806
chr12	53747018	53747045
chr12	53747975	53747976
chr12	53759221	53759335
chr12	53764194	53765053
chr12	53765228	53765858
chr12	53766271	53766788
chr12	53771877	53772091
chr12	53772646	53773265
chr12	53773952	53776174
chr12	53776285	53776646
chr12	53815032	53815441
chr12	53821315	53821453
chr12	53822456	53822676
chr12	53827237	53827620
chr12	53827729	53827971
chr12	53834050	53834265
chr12	53834774	53835336
chr12	53835628	53836106
chr12	53842576	53842775
chr12	53845407	53845554
chr12	53846074	53846722
chr12	53857707	53857728
chr12	53860361	53860510
chr12	53866745	53866837
chr12	53872021	53872182
chr12	53882901	53883038
chr12	53886524	53886875
chr12	53887035	53887509
chr12	53892617	53895243
chr12	53895441	53895747
chr12	53902284	53902427

chr12	53903730	53903846
chr12	53912364	53912505
chr12	53933813	53934012
chr12	53936014	53936277
chr12	53937579	53937625
chr12	53948105	53948192
chr12	53955447	53955766
chr12	53962544	53962868
chr12	53962983	53963073
chr12	54007617	54007694
chr12	54007917	54008031
chr12	54013368	54013577
chr12	54019548	54020504
chr12	54020618	54020685
chr12	54068946	54069879
chr12	54070271	54070299
chr12	54070515	54070549
chr12	54088805	54090535
chr12	54120112	54120156
chr12	54120611	54121251
chr12	54121649	54121847
chr12	54137190	54137549
chr12	54582327	54582660
chr12	54609192	54609233
chr12	54609479	54609506
chr12	54609882	54610206
chr12	54610537	54610826
chr12	54611059	54611119
chr12	54611299	54611578
chr12	54613556	54614373
chr12	54616101	54616436
chr12	54616796	54616906
chr12	54617396	54617418
chr12	54622882	54622996
chr12	54623322	54623496
chr12	54623651	54623784
chr12	54645487	54645658
chr12	54652882	54653026
chr12	54653307	54653610
chr12	54656149	54656647
chr12	54673141	54674419
chr12	54674545	54675222
chr12	54679658	54680118
chr12	54682054	54683132
chr12	54688790	54688866
chr12	54691930	54692039

chr12	54693746	54693796
chr12	54694068	54694267
chr12	54694587	54694911
chr12	54698389	54698572
chr12	54699089	54699363
chr12	54700049	54700303
chr12	54700743	54700886
chr12	54701286	54701362
chr12	54701825	54702034
chr12	54705626	54705736
chr12	54716795	54716851
chr12	54718321	54718578
chr12	54718951	54719235
chr12	54719573	54719886
chr12	54720470	54720730
chr12	54720916	54721347
chr12	54724618	54724653
chr12	54724909	54725037
chr12	54726882	54727081
chr12	54751841	54752300
chr12	54752364	54752943
chr12	54753153	54753670
chr12	54764924	54765160
chr12	54765995	54766228
chr12	54766464	54767684
chr12	54767980	54768257
chr12	54768917	54769038
chr12	54769172	54769273
chr12	54774594	54774777
chr12	54775623	54776157
chr12	54776686	54777007
chr12	54777139	54778021
chr12	54778148	54778427
chr12	54780158	54780222
chr12	54781543	54781958
chr12	54782126	54782409
chr12	54783239	54785126
chr12	54786978	54787233
chr12	54791756	54791836
chr12	54792683	54792891
chr12	54805009	54805145
chr12	54804485	54804657
chr12	54805474	54805688
chr12	54811481	54813459
chr12	54816485	54816730
chr12	54816815	54816893

chr12	54817325	54817543
chr12	54831620	54831731
chr12	55328768	55329061
chr12	55329262	55329457
chr12	56075093	56075292
chr12	56109375	56109499
chr12	56109599	56109735
chr12	56109891	56110813
chr12	56120225	56120478
chr12	56121523	56124051
chr12	56124197	56124325
chr12	56131670	56132632
chr12	56132901	56132921
chr12	56133831	56133845
chr12	56136517	56136877
chr12	56137728	56138078
chr12	56211098	56211613
chr12	56211764	56212438
chr12	56220798	56221070
chr12	56222558	56223347
chr12	56223587	56223871
chr12	56223997	56225428
chr12	56236402	56236669
chr12	56241350	56241697
chr12	56242144	56242484
chr12	56247606	56247656
chr12	56301150	56301243
chr12	56308674	56309086
chr12	56309909	56310230
chr12	56312874	56313184
chr12	56317632	56317828
chr12	56320119	56321270
chr12	56321367	56322056
chr12	56323873	56326824
chr12	56326903	56327696
chr12	56329599	56330017
chr12	56331676	56331776
chr12	56331932	56332265
chr12	56332594	56333128
chr12	56333596	56334454
chr12	56348608	56348807
chr12	56349516	56349952
chr12	56350161	56350281
chr12	56351472	56351739
chr12	56357046	56357174
chr12	56360108	56360885

chr12	56361015	56361383
chr12	56365693	56366303
chr12	56366671	56367307
chr12	56367777	56368521
chr12	56375605	56375805
chr12	56390259	56390458
chr12	56390635	56390919
chr12	56391136	56391753
chr12	56400635	56402268
chr12	56422447	56423284
chr12	56434951	56435397
chr12	56435680	56436460
chr12	56440910	56441031
chr12	56441229	56441309
chr12	56448496	56448714
chr12	56448805	56449320
chr12	56453753	56454128
chr12	56455866	56456378
chr12	56465260	56465797
chr12	56471402	56471446
chr12	56472891	56474900
chr12	56497552	56498204
chr12	56498331	56499375
chr12	56504274	56504427
chr12	56504534	56504800
chr12	56509107	56509522
chr12	56509905	56510872
chr12	56511618	56511716
chr12	56512114	56512770
chr12	56512959	56513670
chr12	56513945	56514107
chr12	56519157	56519293
chr12	56519835	56520063
chr12	56520325	56520401
chr12	56521512	56521614
chr12	56522065	56523456
chr12	56523629	56523828
chr12	56530162	56530302
chr12	56545357	56545433
chr12	56545585	56546503
chr12	56550562	56553047
chr12	56553222	56554027
chr12	56554247	56554252
chr12	56554455	56554688
chr12	56581754	56581832
chr12	56582576	56583309

chr12	56583593	56584513
chr12	56586942	56587214
chr12	56590947	56590959
chr12	56609406	56609464
chr12	56614246	56616461
chr12	56617533	56618554
chr12	56618869	56619068
chr12	56619631	56619830
chr12	56639892	56639964
chr12	56650647	56650874
chr12	56651013	56652701
chr12	56654321	56654534
chr12	56655510	56655781
chr12	56656448	56656688
chr12	56655909	56656234
chr12	56657995	56658109
chr12	56659009	56659152
chr12	56660040	56660380
chr12	56660612	56661396
chr12	56675418	56675831
chr12	56685164	56685332
chr12	56685443	56686265
chr12	56693516	56694112
chr12	56694376	56694498
chr12	56694841	56694965
chr12	56695244	56695566
chr12	56701300	56701744
chr12	56707724	56707895
chr12	56708595	56708862
chr12	56709028	56710117
chr12	56710242	56710429
chr12	56714904	56715134
chr12	56726797	56727030
chr12	56727114	56727760
chr12	56727870	56728378
chr12	56731776	56731830
chr12	56732290	56732328
chr12	56732659	56732936
chr12	56752586	56753008
chr12	56753657	56753924
chr12	56754256	56754642
chr12	56829227	56829477
chr12	56842108	56842390
chr12	56842544	56843453
chr12	56855080	56855420
chr12	56858043	56858137

chr12	56858346	56858497
chr12	56859044	56859126
chr12	56862101	56862165
chr12	56862319	56862565
chr12	56862803	56863011
chr12	56881531	56882193
chr12	56907825	56907864
chr12	56911667	56912079
chr12	56912464	56912710
chr12	56913897	56914454
chr12	56915064	56915879
chr12	56916176	56916716
chr12	56918162	56918632
chr12	56920413	56920753
chr12	56924346	56924557
chr12	56930836	56931339
chr12	56943552	56943754
chr12	56954158	56954351
chr12	56959819	56959847
chr12	56963755	56963978
chr12	56964176	56964297
chr12	56965811	56965965
chr12	56967172	56967291
chr12	56967454	56967513
chr12	56967966	56968293
chr12	56975576	56976109
chr12	56976286	56976790
chr12	56987566	56987850
chr12	56991034	56991233
chr12	57014316	57014454
chr12	57022606	57022768
chr12	57022927	57024698
chr12	57025920	57026596
chr12	57026781	57027259
chr12	57027421	57028848
chr12	57028989	57029816
chr12	57029975	57030794
chr12	57030991	57031263
chr12	57032408	57032608
chr12	57034588	57034799
chr12	57038914	57039311
chr12	57039412	57039730
chr12	57040091	57040326
chr12	57051749	57052045
chr12	57054071	57054210
chr12	57054381	57054523

chr12	57055829	57056015
chr12	57061435	57061465
chr12	57061681	57061757
chr12	57064845	57065004
chr12	57079596	57079858
chr12	57080157	57080508
chr12	57081102	57083787
chr12	57092579	57092678
chr12	57105517	57105698
chr12	57113860	57114059
chr12	57114961	57115200
chr12	57115951	57116516
chr12	57117644	57118006
chr12	57118692	57119038
chr12	57119262	57119542
chr12	57145109	57145383
chr12	57145472	57146444
chr12	57174061	57174417
chr12	57230837	57230940
chr12	57291791	57292228
chr12	57349314	57349334
chr12	57374096	57374424
chr12	57379016	57379286
chr12	57379972	57380121
chr12	57399742	57400318
chr12	57427065	57427363
chr12	57459357	57459553
chr12	57463418	57463617
chr12	57471751	57472449
chr12	57472888	57473032
chr12	57479185	57479349
chr12	57479662	57479811
chr12	57480003	57480957
chr12	57481154	57482569
chr12	57482897	57484477
chr12	57487303	57487698
chr12	57487977	57488486
chr12	57488835	57490065
chr12	57490198	57490527
chr12	57490867	57490999
chr12	57495398	57495449
chr12	57495521	57495737
chr12	57497301	57497505
chr12	57503673	57506173
chr12	57506328	57506533
chr12	57519934	57520109

chr12	57521852	57522223
chr12	57522635	57522842
chr12	57543338	57543648
chr12	57546035	57546273
chr12	57560066	57560492
chr12	57561209	57561762
chr12	57569551	57569991
chr12	57609587	57609786
chr12	57619027	57619268
chr12	57623068	57623267
chr12	57623387	57624624
chr12	57632150	57632361
chr12	57632476	57632700
chr12	57633211	57634643
chr12	57637362	57637561
chr12	57657766	57657994
chr12	57673921	57674399
chr12	57701489	57701663
chr12	57701814	57701956
chr12	57775501	57776075
chr12	57776209	57776562
chr12	57778724	57778927
chr12	57782836	57782890
chr12	57783082	57783267
chr12	57810312	57810532
chr12	57822507	57822520
chr12	57824013	57825177
chr12	57826633	57826884
chr12	57848512	57848711
chr12	57849642	57850459
chr12	57850945	57851663
chr12	57852452	57853515
chr12	57853628	57853845
chr12	57855611	57856018
chr12	57856397	57856602
chr12	57869689	57869889
chr12	57881309	57881620
chr12	57881849	57882315
chr12	57882869	57883085
chr12	57902671	57902675
chr12	57903803	57904184
chr12	57911536	57911848
chr12	57912727	57913082
chr12	57913349	57914176
chr12	57914599	57916003
chr12	57916164	57917320

chr12	57917431	57917918
chr12	57918091	57918335
chr12	57919029	57919200
chr12	57939854	57941095
chr12	57954903	57955213
chr12	57984545	57984881
chr12	57985028	57985251
chr12	57985337	57985792
chr12	57997580	57998094
chr12	57998158	57999243
chr12	58004598	58005869
chr12	58015324	58015675
chr12	58026629	58026980
chr12	58087359	58087473
chr12	58087964	58088130
chr12	58119690	58120962
chr12	58130438	58130637
chr12	58131255	58132044
chr12	58138153	58139584
chr12	58144287	58144533
chr12	58145154	58146126
chr12	58146487	58146719
chr12	58147737	58147953
chr12	58148186	58148565
chr12	58148722	58149792
chr12	58150282	58150517
chr12	58150853	58150958
chr12	58157839	58157951
chr12	58158103	58158278
chr12	58158596	58159682
chr12	58160117	58160801
chr12	58161121	58162096
chr12	58165317	58167118
chr12	58176572	58176979
chr12	58177293	58177492
chr12	58187890	58188142
chr12	58188809	58189260
chr12	58189436	58189564
chr12	58225075	58225158
chr12	58230195	58230560
chr12	58231077	58231491
chr12	58231721	58231948
chr12	58237766	58237886
chr12	58238666	58240897
chr12	58241065	58241246
chr12	58241488	58241562

chr12	58251748	58251783
chr12	58258737	58259533
chr12	58281755	58282315
chr12	58282438	58283153
chr12	58283317	58283893
chr12	58284337	58284869
chr12	58287104	58287125
chr12	58287632	58287918
chr12	58289987	58290628
chr12	58299063	58299160
chr12	58329671	58329849
chr12	58330064	58330207
chr12	58334500	58336002
chr12	58461628	58461976
chr12	58463484	58463683
chr12	58656622	58656627
chr12	58704315	58704660
chr12	58704923	58705079
chr12	58819208	58819967
chr12	59270894	59271080
chr12	59271564	59271592
chr12	59300698	59301079
chr12	59309490	59309691
chr12	59313242	59313613
chr12	59313724	59314396
chr12	59314542	59314621
chr12	59433878	59434077
chr12	59434949	59435377
chr12	59435568	59435846
chr12	59440757	59441250
chr12	59449227	59449684
chr12	59450746	59450761
chr12	59450971	59451064
chr12	59471281	59471627
chr12	59472844	59473191
chr12	59751591	59751763
chr12	59797386	59797422
chr12	59853957	59854116
chr12	59963885	59963973
chr12	59989365	59989622
chr12	59989796	59991413
chr12	59991625	59991716
chr12	60089184	60089532
chr12	60092394	60092737
chr12	60174560	60174660
chr12	60322363	60322579

chr12	60322854	60322925
chr12	60323236	60323287
chr12	60542919	60543071
chr12	60546511	60546695
chr12	60547014	60547031
chr12	60660484	60660683
chr12	61314957	61315193
chr12	62586671	62586808
chr12	62605325	62605379
chr12	62653155	62653419
chr12	62653499	62654114
chr12	62654242	62654603
chr12	62662762	62663092
chr12	62859871	62860222
chr12	62860285	62860291
chr12	62860439	62861261
chr12	62996105	62996560
chr12	62996674	62997596
chr12	63054526	63054555
chr12	63118610	63118733
chr12	63118949	63119201
chr12	63126168	63126171
chr12	63126255	63126474
chr12	63127071	63127135
chr12	63166565	63166748
chr12	63180151	63180367
chr12	63192534	63192589
chr12	63193109	63193273
chr12	63256693	63256909
chr12	63328111	63328958
chr12	63812888	63813028
chr12	63813216	63813256
chr12	64061860	64063332
chr12	64173379	64174376
chr12	64215588	64216046
chr12	64216199	64216599
chr12	64233518	64233549
chr12	64237325	64239131
chr12	64239539	64239895
chr12	64240020	64240493
chr12	64241240	64241663
chr12	64243443	64243487
chr12	64263473	64263781
chr12	64291764	64291963
chr12	64294800	64295390
chr12	64298469	64298819

chr12	64299269	64299469
chr12	64309238	64309604
chr12	64310170	64310424
chr12	64310871	64311319
chr12	64311605	64311908
chr12	64312099	64313080
chr12	64316241	64316372
chr12	64316496	64316662
chr12	64322591	64322644
chr12	64322783	64323060
chr12	64349924	64349938
chr12	64361014	64361322
chr12	64361562	64361876
chr12	64372248	64372671
chr12	64374104	64374381
chr12	64390998	64391298
chr12	64411785	64412289
chr12	64474301	64474778
chr12	64482130	64482208
chr12	64482487	64482799
chr12	64498355	64498613
chr12	64498861	64499118
chr12	64514501	64514700
chr12	64554386	64554619
chr12	64563715	64563908
chr12	64564051	64564190
chr12	64566171	64566209
chr12	64574726	64575075
chr12	64575138	64575293
chr12	64575536	64575890
chr12	64580684	64580813
chr12	64581225	64581442
chr12	64615369	64615468
chr12	64615680	64616544
chr12	64726722	64726810
chr12	64727060	64727134
chr12	64729126	64729270
chr12	64734403	64734773
chr12	64779374	64779676
chr12	64781068	64781528
chr12	64781894	64782100
chr12	64782684	64782704
chr12	64783100	64783348
chr12	64783721	64784589
chr12	64784696	64784766
chr12	64785047	64785279

chr12	64797736	64797966
chr12	64799810	64800171
chr12	64798236	64799094
chr12	64824680	64824968
chr12	64843937	64844250
chr12	64845151	64845222
chr12	64845401	64846467
chr12	64846590	64846995
chr12	64855292	64855328
chr12	64936839	64937102
chr12	64943619	64943717
chr12	65002582	65002737
chr12	65002935	65003873
chr12	65004239	65005154
chr12	65005266	65005564
chr12	65012884	65013004
chr12	65013251	65013356
chr12	65015420	65015673
chr12	65019139	65019855
chr12	65027672	65027869
chr12	65047824	65048202
chr12	65063117	65063179
chr12	65064356	65064395
chr12	65064796	65065241
chr12	65065397	65065422
chr12	65066630	65066655
chr12	65066896	65066942
chr12	65073172	65073193
chr12	65073448	65073836
chr12	65074010	65074134
chr12	65074336	65074390
chr12	65118032	65118190
chr12	65118352	65118549
chr12	65128010	65128066
chr12	65131321	65131406
chr12	65131637	65131693
chr12	65135150	65135289
chr12	65138897	65139132
chr12	65152459	65152503
chr12	65152649	65152853
chr12	65153039	65153194
chr12	65153428	65153653
chr12	65174051	65175814
chr12	65189974	65190007
chr12	65204573	65204777
chr12	65211999	65212098

chr12	65212304	65212498
chr12	65217944	65218774
chr12	65218882	65219332
chr12	65294732	65294749
chr12	65363121	65363205
chr12	65377562	65377654
chr12	65398021	65398194
chr12	65562905	65563182
chr12	65563355	65563513
chr12	65563696	65564600
chr12	65672052	65673376
chr12	65694592	65694911
chr12	65700621	65700949
chr12	65804374	65804458
chr12	65931087	65931167
chr12	65950400	65950479
chr12	65950762	65950921
chr12	65990092	65990277
chr12	65990465	65990937
chr12	65991605	65991608
chr12	65996285	65996417
chr12	65996590	65997038
chr12	65997233	65997315
chr12	65997458	65997667
chr12	66002328	66002769
chr12	66002996	66003085
chr12	66005222	66005564
chr12	66025284	66026031
chr12	66028034	66028230
chr12	66028862	66028970
chr12	66042800	66042928
chr12	66043096	66043238
chr12	66050002	66050253
chr12	66050572	66050921
chr12	66051034	66051132
chr12	66063072	66063142
chr12	66064455	66065068
chr12	66113892	66114290
chr12	66122677	66122759
chr12	66122983	66123367
chr12	66135063	66135200
chr12	66135317	66136860
chr12	66159007	66159531
chr12	66216414	66220170
chr12	66239978	66240267
chr12	66246713	66246876

chr12	66262029	66262471
chr12	66272358	66272402
chr12	66280934	66281293
chr12	66283296	66283403
chr12	66285566	66286311
chr12	66286739	66286851
chr12	66296548	66296594
chr12	66335249	66335455
chr12	66342369	66342918
chr12	66343944	66344031
chr12	66346547	66346901
chr12	66349357	66349569
chr12	66352214	66352253
chr12	66352489	66352527
chr12	66481221	66481275
chr12	66523869	66524526
chr12	66524677	66525019
chr12	66526142	66526248
chr12	66526521	66526594
chr12	66526757	66527114
chr12	66538819	66538971
chr12	66539166	66539266
chr12	66562928	66563139
chr12	66563320	66564017
chr12	66581260	66581514
chr12	66582705	66583508
chr12	66584380	66584723
chr12	66629155	66629446
chr12	66696348	66696845
chr12	66696973	66696980
chr12	66908380	66908401
chr12	67463233	67463680
chr12	67662109	67662416
chr12	67662551	67663029
chr12	67663138	67664158
chr12	67669374	67669670
chr12	67934790	67934989
chr12	67986797	67987083
chr12	68039481	68039558
chr12	68041952	68042359
chr12	68042583	68044015
chr12	68221693	68221723
chr12	68304077	68304294
chr12	68304768	68304850
chr12	68435874	68435936
chr12	68436411	68436601

chr12	68575967	68576047
chr12	68617216	68617345
chr12	68630694	68630711
chr12	68725431	68725575
chr12	68725846	68726059
chr12	68726446	68726562
chr12	68753004	68753038
chr12	68753739	68753764
chr12	68758904	68759246
chr12	68833906	68834086
chr12	68915252	68915388
chr12	68951046	68951273
chr12	68952976	68953476
chr12	68954965	68955506
chr12	68978269	68978842
chr12	69003853	69004014
chr12	69005104	69005547
chr12	69027575	69027841
chr12	69030392	69030492
chr12	69069869	69070433
chr12	69080309	69080501
chr12	69080746	69081306
chr12	69131128	69131327
chr12	69139691	69140849
chr12	69174494	69174527
chr12	69175195	69175486
chr12	69180872	69181709
chr12	69188635	69189056
chr12	69193956	69194217
chr12	69195363	69195732
chr12	69201125	69202976
chr12	69213366	69213544
chr12	69215718	69215917
chr12	69238671	69238794
chr12	69257696	69258115
chr12	69326649	69327499
chr12	69364761	69365573
chr12	69374249	69375102
chr12	69400897	69401010
chr12	69401346	69401888
chr12	69402213	69402812
chr12	69418159	69418203
chr12	69418486	69418626
chr12	69446742	69446773
chr12	69447135	69447206
chr12	69450523	69450570

chr12	69453242	69453363
chr12	69453511	69453739
chr12	69458902	69459114
chr12	69459554	69459630
chr12	69462188	69462247
chr12	69462583	69462652
chr12	69464072	69464233
chr12	69633024	69633170
chr12	69633384	69634235
chr12	69677892	69677908
chr12	69751264	69751364
chr12	69751608	69751730
chr12	69753304	69753375
chr12	69753488	69753892
chr12	69754215	69754363
chr12	69776932	69777001
chr12	69821578	69821700
chr12	69863836	69864944
chr12	69921631	69922004
chr12	69928190	69928476
chr12	69947943	69947958
chr12	69948704	69949112
chr12	69949329	69949621
chr12	69963590	69963716
chr12	69963930	69964238
chr12	69978944	69979004
chr12	69979255	69979859
chr12	70033544	70033552
chr12	70042181	70042260
chr12	70042664	70042673
chr12	70047737	70047805
chr12	70049730	70050087
chr12	70052003	70052348
chr12	70132038	70133593
chr12	70133778	70133959
chr12	70266751	70266890
chr12	70267223	70267279
chr12	70267448	70267592
chr12	70272972	70273269
chr12	70360216	70360292
chr12	70360768	70360998
chr12	70381546	70381975
chr12	70406799	70407126
chr12	70443755	70444082
chr12	70446720	70446755
chr12	70447036	70447177

chr12	70636402	70638150
chr12	70759519	70759871
chr12	70759986	70760569
chr12	70828394	70828704
chr12	70843270	70843577
chr12	70914706	70914881
chr12	70937619	70937710
chr12	70943077	70943609
chr12	70943837	70944020
chr12	71003662	71004061
chr12	71039971	71040031
chr12	72056598	72058517
chr12	72079270	72079542
chr12	72079649	72080417
chr12	72104314	72104552
chr12	72113867	72114203
chr12	72148298	72148425
chr12	72148628	72149261
chr12	72233149	72233932
chr12	72234460	72234649
chr12	72259038	72259333
chr12	72666016	72666268
chr12	74257658	74257683
chr12	74436988	74437087
chr12	74437402	74437623
chr12	74931328	74931473
chr12	74931675	74932236
chr12	74932415	74932585
chr12	74933026	74933036
chr12	74933175	74933225
chr12	75284277	75284378
chr12	75524506	75524772
chr12	75552791	75552993
chr12	75571737	75571763
chr12	75723843	75724042
chr12	75728405	75728782
chr12	75735603	75735831
chr12	75783295	75783306
chr12	75784349	75784758
chr12	75784941	75785371
chr12	75874271	75874804
chr12	75883479	75883642
chr12	75904986	75905257
chr12	75905587	75905845
chr12	76017282	76018005
chr12	76034784	76035209

chr12	76045298	76045505
chr12	76050365	76050537
chr12	76050696	76050862
chr12	76051114	76051561
chr12	76052608	76052772
chr12	76052898	76052963
chr12	76053917	76054330
chr12	76054581	76054866
chr12	76078596	76078849
chr12	76084318	76084325
chr12	76084697	76084705
chr12	76106055	76106102
chr12	76106253	76106358
chr12	76114548	76114628
chr12	76114869	76115019
chr12	76116429	76116832
chr12	76141105	76141249
chr12	76175416	76175665
chr12	76180905	76181216
chr12	76214490	76214494
chr12	76214638	76214881
chr12	76256336	76256373
chr12	76256566	76256990
chr12	76322341	76322728
chr12	76326949	76327078
chr12	76327198	76327270
chr12	76337629	76338615
chr12	76371472	76371685
chr12	76371980	76372216
chr12	76372977	76373022
chr12	76413880	76414227
chr12	76414411	76414961
chr12	76418746	76419022
chr12	76420514	76420522
chr12	76423745	76425441
chr12	76425578	76426234
chr12	76426396	76426857
chr12	76426948	76426990
chr12	76477561	76478445
chr12	76478645	76479013
chr12	76499302	76499372
chr12	76499715	76499730
chr12	76518259	76518581
chr12	76577653	76577670
chr12	76630597	76631449
chr12	76653414	76653556

chr12	76658964	76659109
chr12	76660637	76660668
chr12	76705600	76705652
chr12	76713382	76713794
chr12	76722917	76723329
chr12	76741715	76742657
chr12	76785486	76785692
chr12	76816383	76816585
chr12	76816895	76817300
chr12	76843433	76843672
chr12	76844536	76844894
chr12	76849487	76849609
chr12	76849729	76849880
chr12	76860597	76860839
chr12	76864680	76864751
chr12	76952467	76952708
chr12	76952826	76953545
chr12	76953721	76954083
chr12	76977883	76977999
chr12	76982570	76982686
chr12	76982934	76983092
chr12	77005339	77005498
chr12	77005691	77005821
chr12	77015288	77015406
chr12	77030205	77030522
chr12	77031895	77031918
chr12	77157429	77158601
chr12	77193159	77193272
chr12	77193461	77193507
chr12	77214728	77214947
chr12	77271982	77273587
chr12	77405055	77405103
chr12	77425771	77425977
chr12	77453420	77453551
chr12	77456688	77456823
chr12	77458865	77458924
chr12	77459048	77459366
chr12	77459539	77460109
chr12	77516252	77516424
chr12	78078917	78078965
chr12	78333736	78334493
chr12	78337285	78337631
chr12	78687196	78687262
chr12	79097926	79097976
chr12	79118300	79118806
chr12	79257188	79257661

chr12	79257788	79258384
chr12	79258766	79258979
chr12	79280831	79280933
chr12	79322453	79322679
chr12	79322790	79323227
chr12	79760903	79761046
chr12	79768394	79768727
chr12	79768948	79769169
chr12	79802077	79802141
chr12	79803364	79803722
chr12	79809750	79809760
chr12	79814239	79814375
chr12	79940848	79941027
chr12	79941388	79942058
chr12	79960175	79960581
chr12	80012798	80012887
chr12	80013085	80013207
chr12	80028858	80029364
chr12	80029495	80029793
chr12	80068557	80068767
chr12	80075232	80075359
chr12	80079168	80079234
chr12	80083366	80084771
chr12	80085653	80086056
chr12	80086149	80086517
chr12	80086725	80087393
chr12	80085036	80085561
chr12	80119790	80120154
chr12	80193403	80193739
chr12	80242237	80242350
chr12	80306022	80306411
chr12	80327623	80327992
chr12	80328111	80329800
chr12	80439382	80439472
chr12	80580456	80580506
chr12	80663155	80663335
chr12	80974054	80974119
chr12	80991955	80992162
chr12	81171780	81172253
chr12	82751670	82751869
chr12	82752063	82752106
chr12	82752281	82752851
chr12	82752939	82753054
chr12	82868876	82868877
chr12	83044342	83044446
chr12	83079879	83080178

chr12	83080732	83081843
chr12	83081983	83082189
chr12	84896079	84896350
chr12	84896481	84896633
chr12	84902491	84902779
chr12	84902988	84903179
chr12	85268621	85268636
chr12	85305019	85305991
chr12	85306177	85306542
chr12	85306652	85307346
chr12	85410580	85410612
chr12	85470712	85470911
chr12	85673924	85674123
chr12	85859022	85859067
chr12	86020193	86020274
chr12	86229958	86230073
chr12	86531612	86531907
chr12	86532126	86532199
chr12	86541873	86542248
chr12	87106103	87106261
chr12	87106430	87106485
chr12	87549428	87549543
chr12	87551512	87551629
chr12	87551882	87552020
chr12	87668656	87668693
chr12	87821622	87821884
chr12	87833790	87833946
chr12	87834214	87834314
chr12	87903214	87903381
chr12	87903509	87903585
chr12	87960708	87961025
chr12	88214669	88215017
chr12	88389351	88389464
chr12	88429065	88429161
chr12	88429278	88429776
chr12	88430312	88430365
chr12	88430558	88430719
chr12	88535395	88535963
chr12	88536087	88536343
chr12	88536454	88536899
chr12	88635904	88636023
chr12	88692433	88692549
chr12	88692696	88692784
chr12	88693235	88693406
chr12	88693558	88693915
chr12	88699379	88699510

chr12	88703915	88704114
chr12	88718838	88719009
chr12	88719158	88719367
chr12	88728023	88728329
chr12	88814314	88814513
chr12	88973626	88974132
chr12	88974339	88974538
chr12	89247960	89248126
chr12	89248345	89248532
chr12	89259572	89259732
chr12	89319762	89320044
chr12	89328130	89328277
chr12	89328472	89328555
chr12	89338091	89338154
chr12	89338369	89338424
chr12	89410793	89411216
chr12	89413711	89413762
chr12	89511258	89511515
chr12	89554494	89554611
chr12	89556351	89556622
chr12	89573728	89573849
chr12	89574064	89574194
chr12	89575587	89575659
chr12	89577802	89577914
chr12	89578288	89578314
chr12	89619149	89620048
chr12	89630826	89631049
chr12	89638485	89639270
chr12	89740159	89740391
chr12	89740796	89740927
chr12	89741168	89741242
chr12	89743069	89743439
chr12	89743626	89743769
chr12	89744377	89744786
chr12	89745113	89746343
chr12	89746617	89746976
chr12	89747077	89748794
chr12	89749106	89749202
chr12	89764291	89765202
chr12	89767543	89767859
chr12	89767944	89768326
chr12	89769272	89769463
chr12	89769729	89769907
chr12	89778020	89778227
chr12	89833911	89834098
chr12	89845611	89845981

chr12	89855842	89855893
chr12	89918180	89920004
chr12	89920197	89920267
chr12	90100926	90101194
chr12	90101345	90103336
chr12	90103612	90103894
chr12	90125430	90125475
chr12	90125731	90125738
chr12	90150428	90151183
chr12	90163378	90163485
chr12	90288399	90288407
chr12	90341763	90341825
chr12	90484694	90484793
chr12	90485442	90485510
chr12	90628197	90628414
chr12	90628662	90628711
chr12	91775349	91775554
chr12	91800983	91801182
chr12	91807250	91807414
chr12	91807679	91807703
chr12	91923975	91923997
chr12	92172951	92173142
chr12	92212632	92212831
chr12	92296592	92296791
chr12	92427962	92428168
chr12	92428641	92429065
chr12	92435550	92435719
chr12	92435894	92436004
chr12	92436785	92436906
chr12	92449897	92450056
chr12	92453747	92454110
chr12	92471969	92472318
chr12	92474164	92474387
chr12	92474540	92474712
chr12	92488139	92488275
chr12	92530938	92531288
chr12	92531898	92532228
chr12	92537813	92537983
chr12	92538374	92539500
chr12	92540037	92540529
chr12	92555179	92555270
chr12	92666127	92666144
chr12	92666376	92666684
chr12	92734871	92735161
chr12	92763428	92763727
chr12	92833351	92833515

chr12	92900271	92900599
chr12	92900672	92900883
chr12	92901012	92901069
chr12	92909989	92910012
chr12	92933148	92933335
chr12	92937182	92937191
chr12	92945765	92945900
chr12	92946076	92946104
chr12	92947077	92947559
chr12	92986030	92986197
chr12	92987548	92987567
chr12	92987782	92988176
chr12	92989491	92989830
chr12	92996321	92996359
chr12	92996499	92996643
chr12	93002599	93002600
chr12	93043435	93043751
chr12	93128972	93129354
chr12	93139953	93140125
chr12	93140287	93140377
chr12	93178890	93178997
chr12	93221698	93221949
chr12	93293073	93293158
chr12	93293362	93293725
chr12	93300022	93300259
chr12	93305874	93305907
chr12	93306017	93306397
chr12	93320902	93321162
chr12	93321540	93321899
chr12	93322138	93322545
chr12	93322774	93323787
chr12	93332314	93332534
chr12	93332650	93332813
chr12	93344766	93344789
chr12	93364777	93365303
chr12	93365688	93365922
chr12	93367021	93367907
chr12	93516176	93516463
chr12	93516644	93516838
chr12	93623198	93623606
chr12	93628299	93628309
chr12	93637836	93637925
chr12	93638086	93638298
chr12	93739672	93740386
chr12	93770841	93771471
chr12	93771677	93772667

chr12	93793183	93793367
chr12	93823482	93823530
chr12	93823688	93824000
chr12	93834914	93835659
chr12	93835838	93836625
chr12	93836752	93836927
chr12	93855594	93855700
chr12	93860983	93861133
chr12	93861318	93861739
chr12	93908545	93908732
chr12	93963737	93965460
chr12	93965644	93966369
chr12	93966583	93966862
chr12	93967128	93967724
chr12	93984299	93984546
chr12	93985140	93985579
chr12	94018369	94019545
chr12	94070879	94071019
chr12	94071160	94071924
chr12	94136311	94136727
chr12	94179004	94179454
chr12	94203986	94204280
chr12	94215671	94215790
chr12	94494484	94494524
chr12	94494602	94494922
chr12	94495087	94495258
chr12	94528260	94528430
chr12	94542756	94543004
chr12	94565634	94565762
chr12	94565854	94566199
chr12	94566671	94566834
chr12	94596132	94596183
chr12	94596507	94596671
chr12	94656117	94656292
chr12	94853278	94853701
chr12	94853846	94854483
chr12	94947199	94947342
chr12	94954450	94954595
chr12	94986152	94986308
chr12	95008990	95009151
chr12	95009300	95009533
chr12	95009598	95010202
chr12	95010341	95010556
chr12	95013052	95013432
chr12	95015026	95016017
chr12	95034279	95034303

chr12	95034637	95034769
chr12	95042984	95043004
chr12	95043180	95044486
chr12	95049992	95050130
chr12	95050361	95050373
chr12	95054306	95054395
chr12	95119596	95119631
chr12	95158019	95158102
chr12	95192703	95192876
chr12	95388060	95388106
chr12	95396921	95397430
chr12	95397618	95397758
chr12	95466561	95467676
chr12	95491364	95491651
chr12	95496955	95497439
chr12	95498109	95498308
chr12	95510505	95511474
chr12	95512210	95512242
chr12	95512365	95512922
chr12	95545771	95545970
chr12	95563143	95563497
chr12	95567551	95568001
chr12	95572821	95573195
chr12	95576649	95577164
chr12	95594468	95594714
chr12	95594859	95595476
chr12	95597791	95598682
chr12	95599343	95599525
chr12	95599655	95599853
chr12	95608318	95608464
chr12	95610699	95611328
chr12	95611553	95611984
chr12	95612228	95612272
chr12	95612581	95612876
chr12	95777972	95778066
chr12	95790634	95790788
chr12	95791438	95791496
chr12	95822516	95823140
chr12	95866913	95867549
chr12	95867993	95868137
chr12	95868310	95868514
chr12	95928190	95928550
chr12	95942382	95942823
chr12	96017655	96017848
chr12	96021758	96022318
chr12	96026968	96027770

chr12	96028529	96029124
chr12	96029369	96029542
chr12	96031455	96031662
chr12	96043199	96043581
chr12	96044453	96044652
chr12	96047009	96047356
chr12	96047626	96048105
chr12	96157724	96157854
chr12	96182395	96182532
chr12	96182931	96185209
chr12	96185439	96185524
chr12	96190144	96190420
chr12	96196651	96196907
chr12	96197044	96197142
chr12	96200024	96200523
chr12	96236080	96236593
chr12	96252716	96252963
chr12	96253291	96253543
chr12	96335909	96336721
chr12	96336859	96337238
chr12	96392157	96392293
chr12	96428343	96429768
chr12	96429887	96430025
chr12	96440956	96441155
chr12	96441258	96441305
chr12	96441507	96442046
chr12	96449184	96449252
chr12	96455846	96455992
chr12	96576912	96577169
chr12	96585562	96585774
chr12	96587298	96587343
chr12	96587433	96588067
chr12	96588293	96588485
chr12	96588614	96590234
chr12	96590812	96590867
chr12	96591732	96592202
chr12	96592649	96593037
chr12	96603137	96603263
chr12	96607561	96608273
chr12	96608974	96609041
chr12	96609309	96609419
chr12	96611648	96612012
chr12	96612990	96614297
chr12	96614662	96614798
chr12	96615105	96615202
chr12	96619066	96619068

chr12	96619959	96620008
chr12	96622387	96622689
chr12	96622950	96623442
chr12	96631320	96631860
chr12	96709688	96710083
chr12	96757347	96757943
chr12	96792827	96794353
chr12	96794474	96795291
chr12	96807963	96808197
chr12	96808477	96808849
chr12	96809825	96810559
chr12	96819163	96819600
chr12	96819678	96820159
chr12	96820558	96821257
chr12	96821436	96821586
chr12	96822291	96822591
chr12	96839487	96839556
chr12	96839781	96840234
chr12	96842069	96842114
chr12	96842323	96842507
chr12	96846131	96846393
chr12	96850961	96851394
chr12	96853327	96853438
chr12	96861016	96861148
chr12	96861364	96861548
chr12	96878908	96879183
chr12	96880685	96881178
chr12	96883359	96883786
chr12	96894626	96895192
chr12	96903820	96904019
chr12	96904138	96904228
chr12	97205233	97205269
chr12	97283510	97283731
chr12	97300494	97301390
chr12	97301540	97301971
chr12	98791393	98791648
chr12	98810864	98811063
chr12	98812231	98812591
chr12	98818422	98818523
chr12	98832901	98833107
chr12	98833735	98834268
chr12	98840438	98840852
chr12	98850544	98851137
chr12	98858182	98858257
chr12	98868835	98869232
chr12	98869433	98869477

chr12	98872420	98873071
chr12	98884413	98885092
chr12	98894608	98894846
chr12	98896535	98897227
chr12	98897370	98897594
chr12	98897754	98898263
chr12	98905415	98905491
chr12	98908757	98910452
chr12	98922297	98922346
chr12	98926564	98926745
chr12	98959709	98959811
chr12	98960774	98961289
chr12	98986198	98986559
chr12	98987004	98987210
chr12	98987500	98988261
chr12	98997268	98997323
chr12	99029733	99029811
chr12	99030122	99030180
chr12	99037754	99038869
chr12	99039137	99039674
chr12	99139551	99139836
chr12	100522517	100522585
chr12	100535374	100536691
chr12	100536853	100537022
chr12	100557563	100557680
chr12	100591227	100591355
chr12	100591696	100591895
chr12	100592692	100594149
chr12	100594408	100594781
chr12	100619642	100619784
chr12	100623258	100623429
chr12	100623639	100623768
chr12	100623890	100624164
chr12	100625466	100626097
chr12	100660351	100660844
chr12	100661084	100661698
chr12	100661838	100662072
chr12	100784206	100784418
chr12	100967176	100967375
chr12	100967509	100967947
chr12	100968519	100968701
chr12	100970775	100970869
chr12	101673255	101673507
chr12	101673630	101673818
chr12	101673980	101674086
chr12	101692188	101692392

chr12	101801164	101802240
chr12	101802328	101802589
chr12	102091088	102092310
chr12	102167532	102167731
chr12	102223811	102224677
chr12	102224901	102225078
chr12	102233797	102233996
chr12	102249052	102249215
chr12	102249357	102249412
chr12	102250827	102251159
chr12	102255684	102255880
chr12	102256337	102256392
chr12	102270112	102270120
chr12	102270251	102270318
chr12	102270627	102272119
chr12	102284538	102284866
chr12	102285228	102285743
chr12	102288420	102288704
chr12	102288815	102289004
chr12	102319411	102319610
chr12	102329748	102329828
chr12	102454967	102455685
chr12	102455925	102456101
chr12	102513448	102513881
chr12	102514083	102515111
chr12	103954248	103954395
chr12	103955138	103955321
chr12	103969288	103969495
chr12	104141805	104141809
chr12	104193888	104194013
chr12	104234529	104235452
chr12	104252693	104253404
chr12	104274531	104274825
chr12	104274982	104274985
chr12	104299770	104299836
chr12	104301357	104301421
chr12	104301668	104301868
chr12	104322881	104323494
chr12	104324347	104325254
chr12	104328679	104328880
chr12	104350121	104350144
chr12	104350354	104351744
chr12	104359271	104360068
chr12	104400726	104400783
chr12	104405014	104405161
chr12	104405381	104405503

chr12	104443151	104443446
chr12	104443627	104443881
chr12	104451760	104451818
chr12	104452545	104452630
chr12	104452880	104452967
chr12	104457740	104459284
chr12	104514888	104514915
chr12	104515105	104515182
chr12	104530968	104531942
chr12	104532222	104532439
chr12	104609287	104609910
chr12	104610772	104610857
chr12	104612494	104612847
chr12	104613053	104614256
chr12	104648641	104648893
chr12	104649157	104649513
chr12	104649843	104650300
chr12	104652385	104652593
chr12	104656865	104657121
chr12	104657329	104657593
chr12	104657773	104658056
chr12	104665953	104666372
chr12	104668613	104668637
chr12	104671074	104671811
chr12	104674410	104674885
chr12	104679707	104681683
chr12	104682394	104682748
chr12	104684231	104684455
chr12	104686014	104686213
chr12	104687184	104687384
chr12	104688234	104688307
chr12	104689015	104689468
chr12	104694364	104694761
chr12	104695052	104695419
chr12	104716525	104716587
chr12	104720300	104720512
chr12	104722301	104722586
chr12	104760149	104760167
chr12	104774721	104774815
chr12	104848672	104848749
chr12	104850744	104852032
chr12	104852145	104852885
chr12	104979769	104979826
chr12	104981997	104982021
chr12	104982740	104982985
chr12	104998269	104998301

chr12	105062197	105062565
chr12	105062636	105063639
chr12	105063799	105064242
chr12	105064663	105065364
chr12	105066118	105066536
chr12	105080045	105080074
chr12	105132433	105132903
chr12	105134172	105134371
chr12	105351465	105352108
chr12	105352302	105352576
chr12	105352708	105353147
chr12	105368188	105368514
chr12	105368633	105368859
chr12	105369368	105369779
chr12	105379361	105379950
chr12	105380145	105380547
chr12	105477316	105477397
chr12	105477678	105478403
chr12	105494347	105494511
chr12	105501097	105501411
chr12	105501551	105502051
chr12	105503557	105503791
chr12	105629071	105629960
chr12	105630169	105630269
chr12	105647519	105648136
chr12	105648344	105648385
chr12	105720704	105720997
chr12	105724369	105724966
chr12	105725233	105725442
chr12	105726152	105726452
chr12	105728718	105728792
chr12	105811894	105811968
chr12	105839371	105839627
chr12	105955654	105956424
chr12	106322488	106322708
chr12	106327694	106328054
chr12	106328331	106328476
chr12	106424417	106424788
chr12	106500598	106500697
chr12	106501130	106501172
chr12	106502215	106502496
chr12	106503582	106503606
chr12	106504187	106504923
chr12	106506566	106506786
chr12	106507223	106507842
chr12	106529729	106529827

chr12	106529992	106530041
chr12	106532401	106533586
chr12	106620800	106621025
chr12	106621187	106621486
chr12	106621604	106621810
chr12	106628298	106628388
chr12	106628692	106628725
chr12	106631170	106631596
chr12	106639131	106639760
chr12	106639964	106642701
chr12	106665536	106665988
chr12	106676088	106676125
chr12	106676362	106676523
chr12	106682160	106682651
chr12	106689751	106689822
chr12	106690294	106690466
chr12	106695397	106695514
chr12	106695754	106697311
chr12	106711450	106711531
chr12	106711838	106711860
chr12	106730394	106730573
chr12	106736777	106737050
chr12	106751238	106751314
chr12	106751488	106752327
chr12	106763281	106763797
chr12	107167727	107167929
chr12	107168055	107168795
chr12	107348254	107348288
chr12	107348986	107351091
chr12	107380579	107381291
chr12	107485724	107485842
chr12	107486143	107486380
chr12	107486933	107487370
chr12	107487512	107487711
chr12	107487820	107488086
chr12	107488280	107488360
chr12	107525848	107525970
chr12	107689442	107689630
chr12	107712214	107712786
chr12	107713087	107713420
chr12	107713688	107713966
chr12	107714216	107714420
chr12	107714559	107715066
chr12	107779311	107779853
chr12	107826146	107826345
chr12	107829973	107830172

chr12	107830735	107831028
chr12	107908810	107909769
chr12	108078917	108079078
chr12	108079376	108079967
chr12	108089765	108089884
chr12	108142326	108142630
chr12	108153233	108153602
chr12	108154212	108154555
chr12	108154765	108155032
chr12	108155253	108155683
chr12	108297821	108297893
chr12	108840853	108840979
chr12	108841572	108841578
chr12	108841764	108841886
chr12	108876413	108876696
chr12	108876984	108877479
chr12	108908022	108908235
chr12	108908650	108908849
chr12	108909059	108909713
chr12	108934776	108934909
chr12	108935137	108935139
chr12	108954682	108955401
chr12	108955878	108956002
chr12	108956239	108956806
chr12	108957063	108957087
chr12	108957167	108957393
chr12	108959725	108959836
chr12	109006291	109006484
chr12	109021530	109021943
chr12	109022039	109023553
chr12	109023822	109024068
chr12	109024182	109024418
chr12	109033920	109034372
chr12	109058288	109059563
chr12	109084819	109085434
chr12	109085637	109085922
chr12	109086062	109086467
chr12	109088646	109088986
chr12	109089737	109090722
chr12	109091705	109092010
chr12	109094646	109094907
chr12	109095027	109095224
chr12	109095569	109096517
chr12	109098085	109098555
chr12	109114925	109115045
chr12	109115203	109115376

chr12	109118136	109118141
chr12	109116816	109117464
chr12	109118344	109118467
chr12	109123597	109125336
chr12	109125446	109126438
chr12	109126904	109127169
chr12	109127863	109128608
chr12	109132905	109133210
chr12	109150880	109151065
chr12	109151514	109151718
chr12	109151851	109152037
chr12	109153070	109153168
chr12	109165393	109165497
chr12	109197248	109197258
chr12	109206233	109206344
chr12	109206708	109207101
chr12	109208210	109208763
chr12	109208955	109209836
chr12	109210133	109210538
chr12	109210779	109210796
chr12	109215517	109216571
chr12	109220992	109221300
chr12	109222449	109222551
chr12	109222710	109222972
chr12	109225067	109225266
chr12	109226562	109227167
chr12	109227360	109227644
chr12	109228700	109228908
chr12	109233036	109233832
chr12	109234472	109234553
chr12	109231930	109232645
chr12	109235263	109235280
chr12	109236154	109236852
chr12	109237005	109237447
chr12	109238206	109238264
chr12	109239483	109239537
chr12	109244556	109244765
chr12	109249986	109251358
chr12	109247526	109248771
chr12	109251492	109252135
chr12	109252217	109252855
chr12	109253138	109253574
chr12	109275856	109276124
chr12	109276252	109276381
chr12	109276453	109276481
chr12	109307487	109307784

chr12	109343601	109344124
chr12	109344228	109344442
chr12	109344699	109345057
chr12	109482723	109483081
chr12	109490066	109491221
chr12	109491482	109491817
chr12	109529414	109529713
chr12	109530233	109530649
chr12	109531048	109531281
chr12	109531437	109531857
chr12	109534921	109535230
chr12	109535366	109535565
chr12	109536033	109536280
chr12	109548933	109549493
chr12	109549667	109549810
chr12	109592263	109592923
chr12	109593080	109593215
chr12	109622406	109622763
chr12	109629194	109630208
chr12	109630448	109630697
chr12	109631231	109631296
chr12	109631526	109631870
chr12	109636516	109636783
chr12	109636890	109637302
chr12	109637538	109638434
chr12	109640684	109641005
chr12	109643366	109643889
chr12	109714890	109714971
chr12	109715220	109715392
chr12	109729814	109730030
chr12	109738487	109738760
chr12	109747874	109748080
chr12	109823203	109823531
chr12	109899786	109900265
chr12	109900536	109901093
chr12	109901297	109901683
chr12	109914243	109914539
chr12	109914906	109915800
chr12	109935446	109935766
chr12	109956513	109956689
chr12	110010803	110011288
chr12	110011534	110011865
chr12	110019440	110019797
chr12	110019961	110020416
chr12	110020544	110020748
chr12	110036029	110036128

chr12	110211901	110212052
chr12	110256930	110257489
chr12	110257896	110258307
chr12	110258489	110258877
chr12	110259052	110259317
chr12	110267701	110267913
chr12	110274583	110274779
chr12	110275479	110275552
chr12	110277111	110277146
chr12	110280191	110280228
chr12	110291168	110291264
chr12	110302028	110302864
chr12	110303060	110303109
chr12	110306100	110306229
chr12	110311145	110311572
chr12	110317147	110317727
chr12	110317880	110318309
chr12	110318783	110318989
chr12	110321670	110322202
chr12	110323368	110323850
chr12	110324590	110324804
chr12	110325899	110326124
chr12	110326346	110326709
chr12	110327009	110327430
chr12	110329121	110329292
chr12	110329433	110329456
chr12	110337790	110338344
chr12	110338558	110339168
chr12	110360411	110360453
chr12	110403788	110403991
chr12	110406603	110406978
chr12	110433240	110433354
chr12	110433592	110434100
chr12	110434270	110434388
chr12	110436520	110438271
chr12	110439468	110439831
chr12	110440482	110440585
chr12	110440760	110441326
chr12	110449948	110450090
chr12	110461411	110461498
chr12	110462781	110463073
chr12	110464529	110464689
chr12	110476580	110477675
chr12	110480482	110480774
chr12	110481204	110481301
chr12	110485827	110486273

chr12	110486497	110486791
chr12	110493199	110493365
chr12	110520271	110520582
chr12	110538070	110538745
chr12	110545657	110545979
chr12	110546833	110547566
chr12	110562157	110562599
chr12	110579396	110579520
chr12	110592070	110592443
chr12	110617125	110617463
chr12	110649995	110650130
chr12	110661500	110661698
chr12	110661942	110662003
chr12	110662146	110662792
chr12	110684784	110685698
chr12	110685843	110686344
chr12	110687169	110687400
chr12	110687548	110687684
chr12	110687787	110687887
chr12	110689742	110689783
chr12	110691658	110692133
chr12	110693639	110693796
chr12	110707364	110708690
chr12	110718119	110718888
chr12	110719046	110720101
chr12	110720227	110720283
chr12	110725765	110726148
chr12	110735006	110735221
chr12	110737457	110737759
chr12	110738908	110739418
chr12	110740926	110741265
chr12	110741614	110742298
chr12	110744654	110744885
chr12	110745130	110745472
chr12	110750362	110751209
chr12	110753570	110753782
chr12	110768646	110768730
chr12	110776319	110776595
chr12	110778484	110778677
chr12	110778911	110779025
chr12	110781864	110782206
chr12	110782278	110782718
chr12	110782900	110783028
chr12	110783274	110783477
chr12	110783633	110783870
chr12	110792222	110792452

chr12	110793195	110793628
chr12	110798449	110798648
chr12	110800761	110800857
chr12	110801089	110801126
chr12	110808299	110808624
chr12	110822022	110822436
chr12	110827943	110828142
chr12	110832375	110832528
chr12	110840596	110842487
chr12	110868905	110869193
chr12	110869341	110869820
chr12	110873273	110873546
chr12	110887447	110888144
chr12	110888267	110888477
chr12	110894434	110894457
chr12	110894591	110894756
chr12	110905799	110906085
chr12	110906514	110907561
chr12	110915382	110915397
chr12	110936945	110937012
chr12	110938902	110939957
chr12	110940082	110940528
chr12	110950420	110950581
chr12	110981303	110981385
chr12	110981680	110981868
chr12	110982984	110984023
chr12	110984257	110984355
chr12	110984990	110985182
chr12	110990352	110990459
chr12	111000295	111000537
chr12	111000710	111001037
chr12	111001767	111001859
chr12	111003690	111003871
chr12	111015889	111016873
chr12	111019007	111019178
chr12	111019410	111019437
chr12	111019954	111021244
chr12	111022857	111023308
chr12	111047014	111047351
chr12	111051346	111052269
chr12	111052382	111052530
chr12	111126608	111126892
chr12	111136617	111136820
chr12	111179371	111179728
chr12	111179873	111180756
chr12	111181014	111181155

chr12	111285040	111285112
chr12	111325541	111325777
chr12	111332946	111333227
chr12	111334247	111334351
chr12	111334614	111334870
chr12	111334983	111335182
chr12	111354387	111354600
chr12	111355218	111356041
chr12	111359699	111359974
chr12	111368477	111368709
chr12	111372169	111372217
chr12	111372605	111372723
chr12	111494851	111494856
chr12	111500321	111500933
chr12	111501096	111501538
chr12	111501665	111501979
chr12	111505561	111505716
chr12	111806295	111806942
chr12	111832239	111832518
chr12	111834349	111834670
chr12	111834996	111835927
chr12	111837329	111837541
chr12	111839939	111840197
chr12	111840531	111841200
chr12	111841362	111841815
chr12	111843425	111845361
chr12	111846330	111846559
chr12	111846824	111847368
chr12	111847498	111847971
chr12	111851205	111851483
chr12	111857122	111857321
chr12	111859132	111859219
chr12	111859865	111860339
chr12	111860844	111861151
chr12	111861227	111861240
chr12	111862911	111863142
chr12	111863293	111863640
chr12	111863769	111864371
chr12	111864905	111865240
chr12	111866257	111866805
chr12	111866952	111867107
chr12	111867421	111867869
chr12	111868455	111869103
chr12	111872417	111874063
chr12	111869493	111870748
chr12	111874323	111874611

chr12	111874869	111876831
chr12	111876908	111877507
chr12	111879926	111880417
chr12	111880628	111881936
chr12	111882606	111882948
chr12	112007740	112007850
chr12	112008093	112008243
chr12	112009463	112009493
chr12	112015938	112016083
chr12	112016196	112016475
chr12	112016683	112016862
chr12	112023849	112023989
chr12	112033593	112033896
chr12	112035117	112035346
chr12	112035549	112038040
chr12	112043831	112043961
chr12	112061349	112061523
chr12	112062280	112062495
chr12	112062633	112063294
chr12	112111032	112111237
chr12	112123127	112123776
chr12	112123919	112124830
chr12	112203867	112205550
chr12	112210694	112211071
chr12	112211590	112212167
chr12	112212323	112212448
chr12	112213040	112213459
chr12	112216821	112217350
chr12	112226004	112226163
chr12	112226525	112226695
chr12	112244312	112244483
chr12	112279517	112279688
chr12	112279795	112281028
chr12	112284767	112284828
chr12	112380424	112380707
chr12	112380816	112381123
chr12	112412877	112413246
chr12	112437129	112437342
chr12	112443698	112444023
chr12	112449403	112449638
chr12	112449792	112450972
chr12	112451264	112452289
chr12	112456522	112457069
chr12	112474315	112474541
chr12	112529430	112529650
chr12	112534808	112534855

chr12	112540886	112540984
chr12	112544610	112544903
chr12	112545821	112546770
chr12	112546902	112547439
chr12	112549155	112550947
chr12	112554413	112555601
chr12	112556527	112556675
chr12	112559506	112559586
chr12	112559826	112560112
chr12	112560753	112561004
chr12	112562935	112563006
chr12	112563239	112564014
chr12	112566403	112566620
chr12	112585778	112585800
chr12	112587728	112587856
chr12	112629581	112629730
chr12	112630044	112630078
chr12	112656020	112656294
chr12	112657116	112657247
chr12	112741277	112741317
chr12	112781295	112781445
chr12	112819142	112820674
chr12	112825152	112825814
chr12	112846851	112847381
chr12	112847545	112847728
chr12	112853362	112853504
chr12	112856380	112857629
chr12	113277436	113277673
chr12	113344481	113344690
chr12	113344799	113345071
chr12	113354522	113354578
chr12	113373984	113374513
chr12	113374689	113375430
chr12	113375850	113376733
chr12	113377592	113378028
chr12	113416013	113416835
chr12	113417091	113417495
chr12	113423485	113423870
chr12	113430892	113430949
chr12	113456639	113456794
chr12	113478248	113478514
chr12	113478818	113479216
chr12	113520543	113520810
chr12	113536449	113536823
chr12	113541480	113541483
chr12	113573070	113573525

chr12	113592714	113592918
chr12	113612209	113612220
chr12	113613180	113613379
chr12	113621718	113622146
chr12	113622309	113624332
chr12	113625910	113626168
chr12	113630438	113630681
chr12	113631298	113632144
chr12	113632361	113632608
chr12	113634977	113635226
chr12	113642467	113642734
chr12	113652593	113652738
chr12	113658437	113658587
chr12	113659247	113660095
chr12	113660223	113660637
chr12	113660812	113661329
chr12	113666430	113666515
chr12	113666790	113666810
chr12	113667286	113667655
chr12	113667819	113668048
chr12	113669171	113670051
chr12	113670211	113670277
chr12	113673945	113674303
chr12	113676281	113676426
chr12	113677330	113677652
chr12	113678847	113680428
chr12	113680559	113680951
chr12	113681699	113682087
chr12	113682333	113682445
chr12	113683396	113683631
chr12	113684029	113684749
chr12	113684881	113685422
chr12	113685590	113685694
chr12	113709612	113709835
chr12	113710234	113710283
chr12	113737720	113737923
chr12	113738674	113738888
chr12	113744961	113744977
chr12	113745756	113746097
chr12	113761786	113762134
chr12	113771399	113771467
chr12	113771901	113772892
chr12	113773024	113773483
chr12	113795854	113796722
chr12	113859555	113860047
chr12	113860153	113860812

chr12	113886062	113886158
chr12	114296686	114296723
chr12	114298710	114299419
chr12	114300903	114301129
chr12	114301924	114302128
chr12	114310608	114310614
chr12	114393291	114393350
chr12	114400170	114400257
chr12	114403529	114403742
chr12	114403962	114404068
chr12	114946327	114946423
chr12	115110913	115111008
chr12	115121051	115121104
chr12	115121778	115121858
chr12	115695532	115695760
chr12	115695956	115696101
chr12	115760088	115760121
chr12	115760394	115760626
chr12	115761280	115761436
chr12	115761626	115761782
chr12	115779192	115779297
chr12	115779662	115779699
chr12	115791784	115792292
chr12	115792480	115792812
chr12	115821275	115821504
chr12	115821865	115821964
chr12	115822124	115822154
chr12	115822355	115822598
chr12	116559219	116559308
chr12	116559589	116559687
chr12	116561595	116562128
chr12	116571038	116571298
chr12	116573007	116573105
chr12	116573617	116573747
chr12	116581142	116581253
chr12	116581965	116582079
chr12	116598060	116598133
chr12	116598331	116598454
chr12	116602980	116603358
chr12	116656752	116656768
chr12	116673975	116673991
chr12	116679176	116679242
chr12	116679395	116679523
chr12	116704061	116704129
chr12	116704260	116704460
chr12	116707054	116707068

chr12	116713636	116713679
chr12	116713896	116713926
chr12	116714673	116714928
chr12	116715104	116716176
chr12	116730252	116730352
chr12	116755715	116756082
chr12	116756219	116756880
chr12	116757606	116758412
chr12	116814944	116815371
chr12	116819590	116819789
chr12	116819959	116821144
chr12	116821418	116821512
chr12	116827556	116827792
chr12	116844236	116844372
chr12	116848398	116848434
chr12	116860865	116860975
chr12	116861171	116861233
chr12	116862395	116862713
chr12	116862965	116863856
chr12	116863943	116864327
chr12	116864582	116864678
chr12	116871832	116872973
chr12	116873061	116873656
chr12	116881510	116881680
chr12	116891662	116891732
chr12	116892352	116893691
chr12	116926884	116926989
chr12	116927214	116927285
chr12	116930979	116931245
chr12	116934371	116934666
chr12	116946450	116946564
chr12	116946650	116946665
chr12	116955675	116955874
chr12	116958900	116959028
chr12	116962826	116962902
chr12	116963187	116963791
chr12	116965791	116966268
chr12	116970897	116971724
chr12	116980348	116980452
chr12	116996911	116997833
chr12	117174927	117175244
chr12	117175456	117175907
chr12	117176292	117176425
chr12	117256711	117257409
chr12	117348564	117348591
chr12	117348776	117349324

chr12	117353512	117353949
chr12	117354215	117354333
chr12	117354505	117354726
chr12	117450079	117450133
chr12	117450436	117450563
chr12	117536973	117537346
chr12	117569634	117569813
chr12	117578494	117578507
chr12	117580797	117580893
chr12	117581213	117581334
chr12	117627243	117627514
chr12	117627784	117628319
chr12	117632830	117633244
chr12	117766059	117766173
chr12	117766247	117766275
chr12	117780857	117780932
chr12	117782891	117782958
chr12	117787909	117787941
chr12	117798614	117798813
chr12	118391454	118391457
chr12	118406490	118407264
chr12	118424238	118424540
chr12	118424684	118424903
chr12	118454200	118454285
chr12	118454538	118455111
chr12	118490541	118490866
chr12	118498071	118500231
chr12	118500427	118500676
chr12	118501406	118501586
chr12	118541041	118542079
chr12	118542197	118542418
chr12	118558688	118558728
chr12	118573175	118573691
chr12	118573929	118574709
chr12	118584537	118584730
chr12	118601572	118601778
chr12	118620026	118620144
chr12	118675716	118675875
chr12	118676004	118676068
chr12	118709571	118710230
chr12	118710548	118710880
chr12	118740179	118740286
chr12	118744842	118745201
chr12	118745441	118746059
chr12	118754174	118754339
chr12	118794566	118795089

chr12	118795331	118795643
chr12	118798631	118799115
chr12	118809108	118810104
chr12	118810263	118810764
chr12	118811149	118811553
chr12	118813991	118814873
chr12	118815382	118815497
chr12	119145315	119145493
chr12	120105307	120105477
chr12	120105656	120106438
chr12	120107311	120107516
chr12	120154228	120154356
chr12	120314749	120315098
chr12	120366665	120367009
chr12	120375869	120375917
chr12	120379028	120379170
chr12	120412064	120412074
chr12	120426707	120427322
chr12	120479995	120480552
chr12	120492422	120492630
chr12	120493022	120493317
chr12	120493492	120493839
chr12	120497320	120497519
chr12	120497750	120497972
chr12	120498625	120498839
chr12	120522553	120522650
chr12	120522987	120523220
chr12	120524611	120524814
chr12	120553489	120553543
chr12	120553668	120554045
chr12	120554381	120555614
chr12	120555720	120556959
chr12	120557252	120557755
chr12	120619685	120619871
chr12	120620406	120620730
chr12	120632156	120633660
chr12	120633797	120634524
chr12	120635973	120636059
chr12	120637745	120638109
chr12	120638507	120638885
chr12	120639081	120639844
chr12	120640269	120640551
chr12	120646316	120646340
chr12	120647402	120647743
chr12	120662981	120663248
chr12	120663614	120663848

chr12	120664272	120664668
chr12	120665038	120665283
chr12	120671452	120674420
chr12	120674668	120676431
chr12	120666098	120670966
chr12	120676870	120677199
chr12	120677627	120677855
chr12	120678297	120678662
chr12	120678774	120679609
chr12	120679744	120680061
chr12	120680241	120680599
chr12	120682753	120683254
chr12	120684053	120684252
chr12	120685304	120685948
chr12	120686315	120686901
chr12	120687308	120688078
chr12	120684477	120684931
chr12	120689077	120689457
chr12	120690500	120690706
chr12	120690899	120691518
chr12	120692139	120692452
chr12	120692702	120692901
chr12	120694032	120696387
chr12	120696619	120696980
chr12	120697338	120697631
chr12	120697948	120698097
chr12	120698570	120699072
chr12	120699609	120700157
chr12	120701885	120703532
chr12	120700266	120701702
chr12	120714725	120715052
chr12	120715370	120715396
chr12	120727370	120727477
chr12	120727544	120727634
chr12	120728458	120729571
chr12	120729702	120730801
chr12	120730974	120731207
chr12	120732021	120732194
chr12	120739870	120739937
chr12	120740105	120740562
chr12	120740833	120741123
chr12	120754889	120755345
chr12	120755722	120755793
chr12	120763195	120763362
chr12	120763568	120763637
chr12	120763735	120763957

chr12	120789847	120790044
chr12	120806148	120806706
chr12	120859939	120860078
chr12	120868535	120869140
chr12	120871944	120872309
chr12	120875425	120875688
chr12	120875881	120876267
chr12	120877816	120877929
chr12	120883849	120884792
chr12	120890201	120890765
chr12	120904338	120904693
chr12	120906123	120906152
chr12	120906426	120906919
chr12	120907237	120908144
chr12	120911133	120911332
chr12	120911841	120912237
chr12	120912340	120912539
chr12	120912642	120912865
chr12	120932952	120933267
chr12	120933961	120934564
chr12	120963592	120964091
chr12	120965688	120965984
chr12	120966133	120966493
chr12	120966576	120967978
chr12	120971996	120973222
chr12	120981543	120982038
chr12	120982128	120982380
chr12	120987138	120987530
chr12	120987850	120988647
chr12	120989891	120990140
chr12	121018823	121019222
chr12	121019520	121019714
chr12	121022110	121022993
chr12	121040029	121040404
chr12	121086704	121086736
chr12	121107824	121108033
chr12	121121971	121122413
chr12	121122526	121122691
chr12	121123943	121124190
chr12	121124308	121125800
chr12	121126451	121127115
chr12	121147833	121148063
chr12	121148253	121149068
chr12	121163326	121163417
chr12	121163572	121164175
chr12	121221002	121221075

chr12	121221211	121221388
chr12	121281345	121281624
chr12	121287601	121288026
chr12	121308272	121308326
chr12	121341011	121342046
chr12	121342412	121342707
chr12	121345812	121346119
chr12	121346250	121346318
chr12	121352906	121353033
chr12	121357601	121357625
chr12	121363277	121364454
chr12	121366667	121367479
chr12	121371363	121371472
chr12	121375196	121375318
chr12	121378517	121378769
chr12	121378938	121378983
chr12	121380724	121380921
chr12	121382541	121382699
chr12	121382773	121383119
chr12	121400310	121400318
chr12	121400866	121401111
chr12	121409803	121409977
chr12	121411879	121412045
chr12	121414453	121414516
chr12	121446919	121446959
chr12	121453805	121454238
chr12	121464393	121464655
chr12	121465241	121465316
chr12	121471964	121472155
chr12	121474494	121474710
chr12	121476552	121476939
chr12	121477056	121477319
chr12	121497824	121498219
chr12	121533720	121533808
chr12	121546803	121547002
chr12	121562099	121562282
chr12	121562543	121562846
chr12	121567189	121567369
chr12	121571210	121571435
chr12	121580433	121580837
chr12	121580916	121581275
chr12	121581431	121582818
chr12	121604425	121604663
chr12	121605021	121605082
chr12	121608541	121609082
chr12	121622069	121622199

chr12	121622480	121622676
chr12	121628202	121628322
chr12	121628638	121629083
chr12	121630804	121630836
chr12	121630969	121631150
chr12	121647342	121648686
chr12	121652250	121652904
chr12	121653037	121653198
chr12	121664642	121665108
chr12	121665904	121666038
chr12	121667421	121668624
chr12	121714356	121714853
chr12	121715141	121715246
chr12	121733692	121734522
chr12	121734706	121736589
chr12	121777409	121777677
chr12	121789334	121790035
chr12	121790303	121790836
chr12	121798342	121798725
chr12	121826131	121826338
chr12	121826691	121826929
chr12	121828612	121828714
chr12	121829030	121829139
chr12	121829283	121829430
chr12	121837262	121837716
chr12	121837976	121838984
chr12	121889998	121890043
chr12	121904252	121904585
chr12	121904797	121905126
chr12	121974786	121976277
chr12	121976855	121977054
chr12	121987835	121987853
chr12	121990486	121990568
chr12	121990804	121990947
chr12	121991074	121991128
chr12	122014276	122014480
chr12	122014583	122014864
chr12	122015265	122016592
chr12	122017056	122017777
chr12	122018102	122018552
chr12	122018929	122019128
chr12	122028005	122028256
chr12	122028523	122028693
chr12	122041730	122041961
chr12	122053834	122053989
chr12	122062816	122063089

chr12	122063931	122066185
chr12	122073529	122073863
chr12	122096922	122097125
chr12	122097256	122097455
chr12	122109414	122111215
chr12	122120417	122120521
chr12	122124608	122124702
chr12	122125125	122125356
chr12	122128793	122128992
chr12	122147277	122147498
chr12	122149608	122151612
chr12	122151787	122152105
chr12	122152345	122152578
chr12	122153164	122153356
chr12	122154751	122155059
chr12	122155268	122155411
chr12	122155487	122155811
chr12	122157784	122157945
chr12	122179213	122179521
chr12	122184496	122184535
chr12	122185068	122186120
chr12	122187329	122187558
chr12	122194175	122194395
chr12	122202938	122203234
chr12	122210441	122210574
chr12	122211035	122211199
chr12	122212760	122212973
chr12	122213090	122213403
chr12	122213638	122213837
chr12	122214907	122215311
chr12	122219594	122220933
chr12	122221442	122221504
chr12	122221615	122223562
chr12	122223673	122225626
chr12	122228776	122229125
chr12	122229866	122230172
chr12	122230459	122232773
chr12	122234614	122236026
chr12	122236432	122236653
chr12	122237049	122237510
chr12	122237645	122239558
chr12	122239674	122240101
chr12	122240619	122243427
chr12	122243625	122244139
chr12	122249615	122250018
chr12	122250124	122250643

chr12	122251080	122251515
chr12	122253484	122253969
chr12	122258595	122258809
chr12	122259209	122259519
chr12	122270763	122270914
chr12	122272414	122272706
chr12	122274390	122274688
chr12	122272862	122273872
chr12	122277185	122277555
chr12	122277666	122278185
chr12	122280256	122280327
chr12	122280430	122280532
chr12	122296353	122296447
chr12	122326155	122326380
chr12	122326524	122327238
chr12	122330363	122330575
chr12	122332010	122332274
chr12	122335147	122335494
chr12	122336081	122336138
chr12	122349879	122350296
chr12	122350558	122350864
chr12	122351796	122352041
chr12	122356486	122357040
chr12	122361446	122361811
chr12	122362490	122363157
chr12	122365399	122365601
chr12	122368758	122368981
chr12	122371677	122371844
chr12	122376978	122377002
chr12	122377319	122377521
chr12	122379998	122380624
chr12	122387665	122388392
chr12	122388834	122389222
chr12	122390203	122390418
chr12	122429824	122430756
chr12	122430922	122431082
chr12	122431586	122431964
chr12	122439205	122439378
chr12	122439587	122439791
chr12	122445986	122446159
chr12	122459395	122460172
chr12	122460539	122461637
chr12	122462201	122462338
chr12	122463866	122464454
chr12	122464660	122464859
chr12	122465369	122465579

chr12	122465717	122465969
chr12	122467554	122467782
chr12	122468059	122468487
chr12	122471443	122471543
chr12	122473252	122473474
chr12	122474173	122474363
chr12	122479680	122479756
chr12	122479870	122480063
chr12	122486450	122486682
chr12	122487987	122488172
chr12	122492228	122492340
chr12	122500093	122500316
chr12	122500538	122501934
chr12	122502250	122502873
chr12	122503007	122503545
chr12	122509039	122509042
chr12	122510185	122510263
chr12	122510425	122510692
chr12	122510819	122510859
chr12	122512011	122512209
chr12	122515869	122516493
chr12	122516663	122516892
chr12	122517026	122517870
chr12	122517934	122518757
chr12	122519112	122520019
chr12	122580904	122581258
chr12	122581371	122581770
chr12	122593163	122593645
chr12	122595426	122595766
chr12	122596751	122596996
chr12	122598136	122598757
chr12	122607034	122607266
chr12	122607394	122607815
chr12	122608030	122608484
chr12	122648292	122648738
chr12	122664366	122664593
chr12	122664662	122665323
chr12	122665498	122665697
chr12	122667482	122668078
chr12	122675256	122675513
chr12	122681050	122681434
chr12	122681576	122681918
chr12	122682435	122683179
chr12	122683572	122683658
chr12	122683779	122683816
chr12	122687506	122687632

chr12	122687942	122689765
chr12	122709928	122710596
chr12	122711038	122711281
chr12	122750368	122750966
chr12	122751094	122751790
chr12	122827999	122828073
chr12	122851341	122851819
chr12	122875835	122875961
chr12	122884044	122884842
chr12	122884919	122885134
chr12	122885237	122885735
chr12	122886049	122886378
chr12	122905916	122907185
chr12	122907295	122907776
chr12	122907889	122908519
chr12	122910480	122910698
chr12	122923114	122923772
chr12	122930947	122931212
chr12	122933837	122934074
chr12	122934209	122934437
chr12	122937936	122938465
chr12	122938604	122938614
chr12	122942727	122943360
chr12	122948731	122948942
chr12	122949082	122949902
chr12	122950471	122950704
chr12	122951458	122951988
chr12	122952551	122952713
chr12	122952818	122952945
chr12	122955095	122955295
chr12	122956222	122956538
chr12	122976043	122976236
chr12	122984648	122986132
chr12	122987172	122987369
chr12	123002570	123002832
chr12	123011056	123011562
chr12	123012183	123012455
chr12	123011785	123011980
chr12	123068069	123068191
chr12	123122682	123122750
chr12	123134574	123134596
chr12	123140207	123140383
chr12	123162095	123162269
chr12	123163494	123164478
chr12	123164984	123165030
chr12	123180151	123180320

chr12	123181096	123181232
chr12	123183897	123184193
chr12	123184527	123185469
chr12	123186029	123186313
chr12	123187671	123188353
chr12	123189007	123189268
chr12	123192554	123192678
chr12	123193726	123193914
chr12	123197386	123197790
chr12	123198022	123198801
chr12	123201072	123201536
chr12	123204551	123206026
chr12	123210588	123211110
chr12	123221204	123221359
chr12	123225966	123226171
chr12	123236837	123237073
chr12	123237330	123238061
chr12	123257989	123258152
chr12	123258873	123259564
chr12	123259695	123259968
chr12	123275185	123275295
chr12	123275621	123275636
chr12	123293249	123293637
chr12	123310601	123310647
chr12	123315275	123315498
chr12	123315671	123315980
chr12	123318807	123319473
chr12	123319685	123320185
chr12	123320318	123321030
chr12	123321179	123321186
chr12	123324957	123325600
chr12	123347594	123347976
chr12	123348158	123348594
chr12	123352263	123352579
chr12	123352914	123353195
chr12	123356202	123356239
chr12	123356506	123356749
chr12	123363540	123363620
chr12	123372696	123374121
chr12	123374284	123374632
chr12	123376268	123377120
chr12	123379173	123379689
chr12	123379915	123380724
chr12	123371483	123372382
chr12	123383234	123383486
chr12	123386864	123387215

chr12	123388995	123389675
chr12	123391712	123391966
chr12	123392072	123392250
chr12	123393491	123393634
chr12	123401131	123401348
chr12	123401524	123401735
chr12	123402016	123402198
chr12	123402527	123402853
chr12	123403210	123403847
chr12	123404536	123405037
chr12	123411777	123411929
chr12	123423193	123423367
chr12	123425524	123426273
chr12	123428107	123428547
chr12	123429713	123429821
chr12	123430145	123430312
chr12	123430962	123431213
chr12	123431618	123432904
chr12	123435502	123435520
chr12	123435783	123436249
chr12	123450201	123450654
chr12	123453235	123453465
chr12	123458294	123459064
chr12	123459290	123460753
chr12	123464060	123464568
chr12	123464674	123466122
chr12	123503436	123503741
chr12	123570740	123570891
chr12	123610906	123611459
chr12	123612436	123612490
chr12	123614406	123614518
chr12	123614650	123614669
chr12	123618808	123618889
chr12	123635692	123636042
chr12	123647774	123647780
chr12	123647921	123648093
chr12	123663889	123664049
chr12	123717139	123717286
chr12	123717498	123717702
chr12	123717998	123718884
chr12	123723485	123723934
chr12	123734984	123735443
chr12	123735617	123735920
chr12	123736409	123736608
chr12	123742683	123742868
chr12	123754626	123754839

chr12	123754974	123756215
chr12	123847976	123848067
chr12	123848209	123849362
chr12	123849506	123849774
chr12	123849969	123850641
chr12	123863873	123863962
chr12	123864212	123864343
chr12	123867753	123868267
chr12	123868720	123869418
chr12	123869588	123869807
chr12	123872595	123872776
chr12	123872881	123873259
chr12	123873975	123874895
chr12	123877514	123877661
chr12	123886260	123886517
chr12	123916611	123916873
chr12	123920210	123921264
chr12	123921834	123922138
chr12	123942486	123943669
chr12	123993332	123993690
chr12	123995149	123995893
chr12	123995998	123996442
chr12	123996887	123996947
chr12	123997427	123997878
chr12	124000329	124000549
chr12	124001587	124001645
chr12	124003522	124004242
chr12	124004390	124005181
chr12	124009525	124009647
chr12	124009719	124009991
chr12	124015279	124015531
chr12	124016748	124017299
chr12	124017523	124018094
chr12	124018322	124019178
chr12	124023617	124023720
chr12	124030799	124031208
chr12	124054891	124056127
chr12	124066477	124066816
chr12	124067993	124068898
chr12	124069303	124069972
chr12	124080304	124080501
chr12	124086160	124087071
chr12	124087305	124087469
chr12	124117956	124118126
chr12	124118361	124118613
chr12	124118708	124118964

chr12	124128426	124128678
chr12	124144905	124145252
chr12	124155340	124155419
chr12	124155812	124156180
chr12	124196005	124196703
chr12	124196874	124197560
chr12	124197754	124197843
chr12	124198054	124198294
chr12	124198417	124198598
chr12	124201123	124201264
chr12	124201643	124201662
chr12	124204683	124205132
chr12	124236080	124236279
chr12	124242056	124242154
chr12	124246531	124246848
chr12	124247829	124248352
chr12	124248622	124248705
chr12	124328696	124328995
chr12	124329176	124329487
chr12	124456188	124456504
chr12	124456660	124457562
chr12	124457735	124458770
chr12	124475013	124475204
chr12	124866251	124866450
chr12	124868941	124869810
chr12	124870141	124871001
chr12	124872666	124873278
chr12	124873459	124874039
chr12	124875445	124875644
chr12	124876209	124876672
chr12	124876822	124877291
chr12	124895953	124896169
chr12	124906866	124907190
chr12	124907408	124908291
chr12	124923479	124924179
chr12	124925021	124925260
chr12	124984683	124985134
chr12	124985410	124986005
chr12	124994144	124994380
chr12	124997455	124997942
chr12	125001683	125001882
chr12	125002029	125003696
chr12	125003885	125004287
chr12	125036492	125036853
chr12	125036921	125037008
chr12	125037266	125037511

chr12	125050762	125052067
chr12	125052289	125052924
chr12	125056566	125056575
chr12	125060688	125060901
chr12	125062963	125063162
chr12	125063341	125063529
chr12	125065824	125065917
chr12	125073486	125073910
chr12	125076260	125076355
chr12	125077597	125077686
chr12	125092202	125092401
chr12	125092808	125093014
chr12	125094039	125094422
chr12	125094610	125094920
chr12	125101397	125101610
chr12	125102916	125103535
chr12	125104836	125106555
chr12	125110670	125111474
chr12	125111580	125111722
chr12	125116950	125119136
chr12	125120671	125120906
chr12	125121014	125121277
chr12	125121732	125122296
chr12	125125171	125125609
chr12	125165138	125165289
chr12	125167702	125168383
chr12	125169181	125169485
chr12	125169637	125170095
chr12	125170173	125170393
chr12	125170578	125170958
chr12	125171830	125171879
chr12	125172007	125172325
chr12	125174058	125174068
chr12	125179231	125179489
chr12	125188506	125188535
chr12	125196410	125196662
chr12	125197404	125197473
chr12	125197913	125198174
chr12	125198309	125198622
chr12	125198882	125199075
chr12	125199727	125199832
chr12	125200316	125200512
chr12	125204862	125205173
chr12	125212198	125212382
chr12	125212841	125212945
chr12	125230851	125231316

chr12	125231697	125231917
chr12	125232293	125232455
chr12	125247980	125248151
chr12	125250230	125250253
chr12	125252817	125253016
chr12	125258123	125258408
chr12	125260056	125260263
chr12	125279143	125279389
chr12	125282816	125282947
chr12	125283033	125283034
chr12	125312348	125314324
chr12	125320495	125320738
chr12	125321797	125321950
chr12	125322527	125322911
chr12	125323124	125323372
chr12	125323573	125324166
chr12	125337063	125337211
chr12	125347791	125349610
chr12	125351587	125351711
chr12	125351917	125352013
chr12	125352121	125352226
chr12	125353100	125353126
chr12	125353775	125354237
chr12	125364771	125365298
chr12	125365573	125365749
chr12	125382954	125383011
chr12	125385242	125385430
chr12	125389331	125389979
chr12	125393538	125393752
chr12	125393897	125394639
chr12	125394786	125396112
chr12	125396177	125396523
chr12	125396746	125400254
chr12	125400832	125402164
chr12	125403505	125403976
chr12	125404117	125405019
chr12	125405689	125406473
chr12	125402267	125403222
chr12	125407829	125409333
chr12	125411307	125411543
chr12	125411737	125412044
chr12	125412244	125412359
chr12	125412568	125414356
chr12	125414706	125414768
chr12	125423040	125423448
chr12	125423547	125424550

chr12	125424769	125424870
chr12	125425076	125425306
chr12	125425583	125425674
chr12	125473454	125473946
chr12	125477628	125477823
chr12	125477925	125478097
chr12	125478279	125479476
chr12	125491553	125491691
chr12	125533851	125534134
chr12	125534398	125534685
chr12	125549867	125550632
chr12	125550784	125551292
chr12	125563665	125564102
chr12	125564414	125565185
chr12	125577213	125577510
chr12	125608621	125608672
chr12	125608847	125608859
chr12	125625343	125626150
chr12	125632885	125633436
chr12	126536052	126536273
chr12	126536607	126536772
chr12	127559524	127559749
chr12	127567865	127567881
chr12	127568097	127568212
chr12	127592568	127592684
chr12	127609835	127610623
chr12	127610790	127611169
chr12	127614200	127614441
chr12	127614581	127614609
chr12	127647788	127647951
chr12	127650308	127650496
chr12	127651053	127651152
chr12	127715340	127715493
chr12	127715786	127715931
chr12	127725522	127725605
chr12	127725949	127725992
chr12	127754640	127755259
chr12	127778024	127778118
chr12	129268448	129268453
chr12	129281694	129281976
chr12	129282182	129282549
chr12	129286794	129287161
chr12	129306573	129306762
chr12	129307669	129308346
chr12	129308681	129309432
chr12	131302760	131302980

chr12	131311165	131311244
chr12	131317328	131317387
chr12	131322872	131323297
chr12	131323382	131323810
chr12	131353693	131353887
chr12	131354931	131355130
chr12	131355753	131356400
chr12	131356578	131357359
chr12	131450281	131450397
chr12	132195074	132195629
chr12	132195847	132196003
chr12	132205410	132205711
chr12	132205840	132205898
chr12	132221559	132221845
chr12	132222059	132222262
chr12	132243255	132243454
chr12	132259649	132259896
chr12	132260006	132261065
chr12	132267964	132268287
chr12	132268517	132269050
chr12	132277704	132277971
chr12	132280165	132280456
chr12	132311352	132311551
chr12	132312536	132314513
chr12	132314797	132315442
chr12	132316299	132317117
chr12	132337913	132338337
chr12	132343544	132343605
chr12	132344180	132344260
chr12	132344476	132346216
chr12	132346733	132346993
chr12	132347943	132348142
chr12	132348892	132349024
chr12	132349274	132349746
chr12	132354347	132354547
chr12	132354723	132355489
chr12	132356502	132356830
chr12	132378675	132379361
chr12	132379512	132380255
chr12	132380873	132381417
chr12	132384515	132384831
chr12	132412829	132413627
chr12	132413787	132414865
chr12	132415109	132415331
chr12	132433642	132433788
chr12	132434038	132434235

chr12	132434376	132435405
chr12	132440327	132440412
chr12	132568767	132569214
chr12	132569302	132569773
chr12	132627897	132628181
chr12	132628309	132628762
chr12	132628955	132629603
chr12	132641912	132642111
chr12	132643113	132643320
chr12	132647081	132647349
chr12	132673863	132674108
chr12	132674339	132674862
chr12	132854670	132855033
chr12	132861294	132861493
chr12	132861685	132862207
chr12	132862620	132862931
chr12	132863068	132863535
chr12	132863850	132864049
chr12	132903485	132903932
chr12	132904748	132905069
chr12	133005735	133005943
chr12	133007427	133007543
chr12	133007226	133007351
chr12	133022185	133022550
chr12	133065933	133066435
chr12	133101516	133101890
chr12	133136056	133136439
chr12	133136589	133137448
chr12	133174990	133175189
chr12	133190036	133190186
chr12	133199274	133199546
chr12	133262403	133262602
chr12	133263002	133265067
chr12	133286524	133288304
chr12	133289749	133289796
chr12	133290155	133290595
chr12	133332752	133332814
chr12	133333212	133333402
chr12	133335221	133335331
chr12	133336621	133338411
chr12	133338756	133339273
chr12	133343383	133343508
chr12	133343572	133343729
chr12	133343938	133344151
chr12	133404250	133406069
chr12	133431914	133432058

chr12	133442019	133442225
chr12	133442737	133443323
chr12	133443726	133444000
chr12	133461294	133461484
chr12	133463586	133463929
chr12	133464448	133464931
chr12	133481253	133481409
chr12	133483993	133484164
chr12	133484501	133485008
chr12	133485402	133485958
chr12	133486045	133486140
chr12	133531314	133533902
chr12	133562607	133562755
chr12	133563098	133563526
chr12	133613466	133614683
chr12	133656296	133656681
chr12	133656914	133657849
chr12	133706417	133707434
chr12	133757792	133757857
chr12	133758042	133758486
chr12	133841487	133841909
chr13	20175860	20176717
chr13	20179615	20179647
chr13	20179934	20180224
chr13	20207253	20207311
chr13	20207839	20208309
chr13	20356194	20356817
chr13	20357124	20357268
chr13	20364970	20365291
chr13	20368267	20369163
chr13	20435008	20435530
chr13	20436465	20436499
chr13	20436894	20437124
chr13	20437256	20437547
chr13	20438043	20439089
chr13	20484457	20484471
chr13	20484583	20484932
chr13	20531598	20532621
chr13	20532939	20533477
chr13	20534169	20534172
chr13	20693053	20693330
chr13	20701612	20701863
chr13	20702056	20702559
chr13	20735400	20735652
chr13	20748884	20749020
chr13	20766673	20767111

chr13	20851081	20851398
chr13	20925574	20925679
chr13	20961715	20961862
chr13	20976136	20976267
chr13	20993847	20994129
chr13	21005401	21005600
chr13	21099613	21100043
chr13	21100257	21100529
chr13	21140501	21141863
chr13	21277570	21277777
chr13	21286644	21286723
chr13	21347362	21347959
chr13	21348210	21348369
chr13	21368613	21368876
chr13	21387974	21388040
chr13	21388225	21388285
chr13	21388588	21388673
chr13	21476159	21476200
chr13	21476708	21477492
chr13	21517980	21518098
chr13	21572196	21572456
chr13	21591396	21592156
chr13	21634300	21636041
chr13	21643609	21643707
chr13	21650351	21650383
chr13	21711788	21711960
chr13	21714783	21714831
chr13	21750220	21750689
chr13	21750852	21751243
chr13	21754865	21755167
chr13	21871446	21871522
chr13	21873153	21873350
chr13	21900498	21900776
chr13	22032529	22033320
chr13	22051197	22051322
chr13	22122779	22122888
chr13	22123012	22123147
chr13	22177579	22177741
chr13	22178043	22178318
chr13	22178498	22178603
chr13	22243896	22244297
chr13	23488778	23489174
chr13	23489291	23489892
chr13	23893794	23893867
chr13	23895843	23895967
chr13	23896098	23896118

chr13	23917587	23917770
chr13	23949070	23949708
chr13	23952219	23952324
chr13	23952547	23952659
chr13	23958567	23958613
chr13	23989207	23989252
chr13	24001520	24001690
chr13	24029917	24030122
chr13	24040653	24040875
chr13	24127932	24128137
chr13	24132934	24133619
chr13	24152991	24153305
chr13	24153505	24153851
chr13	24248965	24249067
chr13	24252894	24252916
chr13	24345049	24345535
chr13	24345638	24346199
chr13	24346424	24346530
chr13	24396774	24396788
chr13	24443080	24443130
chr13	24462586	24462711
chr13	24463052	24463479
chr13	24463758	24463867
chr13	24463980	24464386
chr13	24594794	24594808
chr13	24624349	24624413
chr13	24722781	24723037
chr13	24734235	24735724
chr13	24735851	24735938
chr13	24923636	24923683
chr13	25075242	25075432
chr13	25077335	25077670
chr13	25084994	25085727
chr13	25085869	25087160
chr13	25154892	25154939
chr13	25223247	25223464
chr13	25223536	25224010
chr13	25238805	25239300
chr13	25239630	25239681
chr13	25243005	25243046
chr13	25243269	25243562
chr13	25245115	25246593
chr13	25254464	25255102
chr13	25256210	25256521
chr13	25268556	25268781
chr13	25302013	25302276

chr13	25302438	25302549
chr13	25321890	25322422
chr13	25351354	25351383
chr13	25416749	25417164
chr13	25417359	25417395
chr13	25425218	25425605
chr13	25425690	25425794
chr13	25425916	25426424
chr13	25485421	25485632
chr13	25496382	25496987
chr13	25497170	25497774
chr13	25542206	25542587
chr13	25547141	25547679
chr13	25563205	25563374
chr13	25569508	25569728
chr13	25788213	25788274
chr13	25788839	25789047
chr13	25860659	25862162
chr13	25869140	25869190
chr13	25875017	25876366
chr13	25879166	25879573
chr13	25891869	25892356
chr13	25893003	25893454
chr13	25903814	25903906
chr13	25904023	25904276
chr13	25931594	25931723
chr13	25946013	25946342
chr13	26148860	26148984
chr13	26307673	26307764
chr13	26435194	26435348
chr13	26475710	26475919
chr13	26476350	26476468
chr13	26625703	26626042
chr13	26760264	26761305
chr13	26794728	26795101
chr13	26795544	26796822
chr13	26827883	26829060
chr13	26829170	26829264
chr13	27054592	27054774
chr13	27083325	27083426
chr13	27131218	27131518
chr13	27131837	27132689
chr13	27190586	27190656
chr13	27277884	27278047
chr13	27586760	27587000
chr13	27612816	27612841

chr13	27699414	27699507
chr13	27705189	27705815
chr13	27709594	27709820
chr13	27713663	27713796
chr13	27743316	27743571
chr13	27744523	27744549
chr13	27744952	27745566
chr13	27746066	27746976
chr13	27825203	27826390
chr13	27830893	27831172
chr13	27832784	27832805
chr13	27844336	27845564
chr13	27884265	27884464
chr13	27935750	27935882
chr13	27936562	27936887
chr13	27949104	27949227
chr13	27998178	27999245
chr13	27999416	28000701
chr13	28001232	28001332
chr13	28023996	28025603
chr13	28107493	28107692
chr13	28123656	28123766
chr13	28124060	28124431
chr13	28174292	28174452
chr13	28192149	28192443
chr13	28193544	28196632
chr13	28208264	28209233
chr13	28218430	28218755
chr13	28527951	28528152
chr13	28528675	28528911
chr13	28586839	28586998
chr13	28712039	28714353
chr13	28714579	28714941
chr13	28736611	28736757
chr13	28747902	28747996
chr13	28755419	28755661
chr13	28755829	28755966
chr13	29106497	29106696
chr13	29149135	29149211
chr13	29232936	29233791
chr13	29291130	29291172
chr13	29292338	29292587
chr13	29292697	29292896
chr13	29293335	29293458
chr13	29327617	29328066
chr13	30031389	30031428

chr13	30046756	30046858
chr13	30142154	30142390
chr13	30142853	30143028
chr13	30143302	30143536
chr13	30157971	30157980
chr13	30163599	30163910
chr13	30168307	30170746
chr13	30250207	30250537
chr13	30250764	30251805
chr13	30267379	30267655
chr13	30267785	30268204
chr13	30276917	30277026
chr13	30277221	30277638
chr13	30421632	30422180
chr13	30423240	30423439
chr13	30423721	30424877
chr13	30434381	30434465
chr13	30436263	30436403
chr13	30451746	30452264
chr13	30476677	30476807
chr13	30479940	30480075
chr13	30480304	30480374
chr13	30493298	30493551
chr13	30497781	30498253
chr13	30498572	30498903
chr13	30499146	30499194
chr13	30499522	30499707
chr13	30499851	30500114
chr13	30681922	30682365
chr13	30682799	30683093
chr13	30683302	30683554
chr13	30687427	30687451
chr13	30688770	30689343
chr13	30693207	30693470
chr13	30711914	30712406
chr13	30716446	30716664
chr13	30716827	30717606
chr13	30878357	30878558
chr13	30880796	30882047
chr13	30882772	30882819
chr13	30891599	30891889
chr13	30916216	30916327
chr13	30916629	30916762
chr13	30935553	30935776
chr13	30969282	30969700
chr13	30969878	30970040

chr13	30970539	30970992
chr13	30982286	30982477
chr13	30982625	30983690
chr13	30995284	30996974
chr13	31003222	31003391
chr13	31004592	31004845
chr13	31005368	31006105
chr13	31038194	31039352
chr13	31039615	31040169
chr13	31040279	31041054
chr13	31143967	31144129
chr13	31144265	31144485
chr13	31147467	31147491
chr13	31151352	31151406
chr13	31161380	31161733
chr13	31191005	31191727
chr13	31192013	31192805
chr13	31247763	31248026
chr13	31248193	31248866
chr13	31249239	31249352
chr13	31256147	31256337
chr13	31264398	31264656
chr13	31265028	31265180
chr13	31282922	31283013
chr13	31293550	31293680
chr13	31308519	31308680
chr13	31308886	31308970
chr13	31314467	31314779
chr13	31317410	31317765
chr13	31318101	31318246
chr13	31318355	31318444
chr13	31321301	31321507
chr13	31367125	31367210
chr13	31370226	31370455
chr13	31370569	31371030
chr13	31381301	31381320
chr13	31381697	31381758
chr13	31384329	31384459
chr13	31384731	31384890
chr13	31402696	31402897
chr13	31403150	31403391
chr13	31403741	31404027
chr13	31407608	31408052
chr13	31415694	31415893
chr13	31417752	31417951
chr13	31418869	31419205

chr13	31420299	31420498
chr13	31439266	31439493
chr13	31439773	31440198
chr13	31487439	31487656
chr13	31496608	31496712
chr13	31497041	31497122
chr13	31550692	31550695
chr13	31552244	31552336
chr13	31618886	31618910
chr13	31619275	31619864
chr13	31620009	31620589
chr13	31667827	31668045
chr13	31668356	31668896
chr13	31669123	31669174
chr13	31734952	31735170
chr13	31735603	31735960
chr13	31736642	31736948
chr13	31740951	31741142
chr13	31746073	31746332
chr13	31746450	31746522
chr13	31746594	31746760
chr13	31746954	31747158
chr13	31773813	31775134
chr13	31775281	31775459
chr13	31779575	31779685
chr13	32324407	32324486
chr13	32330841	32330855
chr13	32341222	32341342
chr13	32341593	32341741
chr13	32341813	32342012
chr13	32347623	32347957
chr13	32360176	32360221
chr13	32420789	32421141
chr13	32483121	32483493
chr13	32505089	32505434
chr13	32519286	32519787
chr13	32520094	32520182
chr13	32556624	32556761
chr13	32574863	32575135
chr13	32575277	32575343
chr13	32575632	32575766
chr13	32593723	32594065
chr13	32605113	32605167
chr13	32605535	32605903
chr13	32663659	32663786
chr13	32717325	32717355

chr13	32737649	32737726
chr13	32739876	32739957
chr13	32816720	32817085
chr13	32829155	32829218
chr13	32889031	32890356
chr13	32898089	32898408
chr13	32904527	32904676
chr13	32904882	32904946
chr13	33001275	33002312
chr13	33002540	33002748
chr13	33006908	33007030
chr13	33007208	33007466
chr13	33017254	33017308
chr13	33094574	33094846
chr13	33112288	33112899
chr13	33111935	33112214
chr13	33113134	33113701
chr13	33159933	33160967
chr13	33161235	33162274
chr13	33213691	33213731
chr13	33243407	33243478
chr13	33257261	33257419
chr13	33293907	33294245
chr13	33305774	33306101
chr13	33307833	33308202
chr13	33308809	33308978
chr13	33399016	33399285
chr13	33403833	33404063
chr13	33404396	33404588
chr13	33591043	33591242
chr13	33591416	33591753
chr13	33592032	33592142
chr13	33693970	33694616
chr13	33700679	33700762
chr13	33743403	33743505
chr13	33743668	33743837
chr13	33781648	33781743
chr13	33781965	33782126
chr13	33782358	33782519
chr13	33787869	33787974
chr13	33792089	33792318
chr13	33792421	33792931
chr13	33802724	33803278
chr13	33804328	33804398
chr13	33804511	33804944
chr13	33805313	33805574

chr13	33809235	33809369
chr13	33809905	33810104
chr13	33825202	33825383
chr13	33830615	33830622
chr13	33836131	33836285
chr13	33836851	33836999
chr13	33848418	33848491
chr13	33848717	33848897
chr13	33853930	33854138
chr13	33854355	33854858
chr13	33856430	33856788
chr13	33859058	33859921
chr13	33860086	33860283
chr13	33886463	33886679
chr13	33923887	33924945
chr13	33925489	33925496
chr13	33962409	33962608
chr13	33962806	33963037
chr13	34057694	34057901
chr13	34068974	34069018
chr13	34069297	34069386
chr13	34116268	34116549
chr13	34116671	34117348
chr13	34117744	34118060
chr13	34208136	34209258
chr13	34209904	34210238
chr13	34212121	34212482
chr13	34214002	34214372
chr13	34216410	34217900
chr13	34247835	34248029
chr13	34250180	34250261
chr13	34250398	34250545
chr13	34250747	34250902
chr13	34253397	34253590
chr13	34253795	34253861
chr13	34271156	34272006
chr13	34297918	34298007
chr13	34298160	34298388
chr13	34339074	34339238
chr13	34361459	34361682
chr13	34374356	34374754
chr13	34392048	34392140
chr13	34392276	34393004
chr13	34419831	34420345
chr13	34433746	34433823
chr13	34514982	34515471

chr13	34591652	34591664
chr13	34618267	34618397
chr13	34622754	34622884
chr13	34623076	34623304
chr13	34640068	34640168
chr13	34640445	34640498
chr13	34644140	34644262
chr13	34644436	34644450
chr13	34731057	34731135
chr13	34820878	34821020
chr13	35437509	35437750
chr13	35438209	35438221
chr13	35516529	35517119
chr13	36123928	36123963
chr13	36126678	36126967
chr13	36173620	36173834
chr13	36474473	36474600
chr13	36871437	36871546
chr13	36871675	36872512
chr13	36907158	36907262
chr13	36907368	36907433
chr13	36913916	36914276
chr13	36916218	36916235
chr13	36918387	36918707
chr13	36919215	36920960
chr13	36921148	36921224
chr13	36992178	36992186
chr13	37002022	37002113
chr13	37002544	37002664
chr13	37005666	37006298
chr13	37028273	37028442
chr13	37336044	37336168
chr13	37340573	37340856
chr13	37393070	37394171
chr13	37394289	37394318
chr13	37444199	37444333
chr13	37457615	37457617
chr13	37493456	37494626
chr13	37539871	37540048
chr13	37540266	37540353
chr13	37552214	37552603
chr13	37560744	37561123
chr13	37561303	37561502
chr13	37572427	37573788
chr13	37573915	37575363
chr13	37598066	37598336

chr13	37632981	37633876
chr13	37634042	37634159
chr13	37881166	37881239
chr13	37935778	37935928
chr13	37991228	37991389
chr13	38084463	38084682
chr13	38230379	38231332
chr13	38231459	38231626
chr13	38276456	38276538
chr13	38290007	38290028
chr13	38444287	38444515
chr13	38444729	38444828
chr13	38445024	38445331
chr13	38489244	38489280
chr13	38528313	38529149
chr13	38530677	38531013
chr13	38754156	38754639
chr13	38883107	38883444
chr13	38920943	38921234
chr13	38922945	38924619
chr13	38960507	38960574
chr13	38991804	38992100
chr13	38992296	38992307
chr13	39489848	39489920
chr13	39526972	39527109
chr13	39580666	39581048
chr13	39610421	39610898
chr13	39611571	39613087
chr13	39623642	39623665
chr13	39719434	39719472
chr13	39722969	39723171
chr13	39848439	39848461
chr13	39887861	39888055
chr13	39991921	39992083
chr13	40081166	40081313
chr13	40081497	40081628
chr13	40083839	40084221
chr13	40084341	40084751
chr13	40086838	40087269
chr13	40102102	40102182
chr13	40102346	40102544
chr13	40105781	40106104
chr13	40139688	40140165
chr13	40176623	40176688
chr13	40176879	40178136
chr13	40186141	40186310

chr13	40229625	40230491
chr13	40286101	40286300
chr13	40287075	40287164
chr13	40352056	40352168
chr13	40410184	40410281
chr13	40488111	40488201
chr13	40488352	40488470
chr13	40609134	40609365
chr13	40643616	40643693
chr13	40650877	40651365
chr13	40651447	40651633
chr13	40651857	40652056
chr13	40683295	40683896
chr13	40690191	40690202
chr13	40690432	40690577
chr13	40697339	40697411
chr13	40726324	40726393
chr13	40726507	40726742
chr13	40745485	40745520
chr13	40746248	40746314
chr13	40764875	40765302
chr13	40780898	40781127
chr13	40795466	40795618
chr13	40795764	40795841
chr13	40807714	40807792
chr13	40808083	40808222
chr13	40808489	40808688
chr13	40810399	40811167
chr13	40814384	40814739
chr13	40905047	40905465
chr13	40906616	40906897
chr13	40907361	40907826
chr13	40959984	40960204
chr13	40976177	40976280
chr13	40976348	40976556
chr13	40976884	40977156
chr13	41019585	41019816
chr13	41020065	41020085
chr13	41028000	41028140
chr13	41163762	41163882
chr13	41165922	41165992
chr13	41184776	41184838
chr13	41230657	41230934
chr13	41238214	41239920
chr13	41240042	41240734
chr13	41241106	41241843

chr13	41242747	41242781
chr13	41246503	41247487
chr13	41259068	41259084
chr13	41308897	41309406
chr13	41344901	41345754
chr13	41351115	41351498
chr13	41360775	41361013
chr13	41362845	41364737
chr13	41489394	41489568
chr13	41492399	41492409
chr13	41492750	41492840
chr13	41495331	41496233
chr13	41512118	41512168
chr13	41538024	41538463
chr13	41538539	41538944
chr13	41539587	41539821
chr13	41548986	41549392
chr13	41556908	41557254
chr13	41558901	41559224
chr13	41568064	41568735
chr13	41582469	41582803
chr13	41593267	41593724
chr13	41594171	41594449
chr13	41596802	41596849
chr13	41604895	41604908
chr13	41627435	41627939
chr13	41628419	41628921
chr13	41629298	41629523
chr13	41634130	41636468
chr13	41685547	41685807
chr13	41703842	41704066
chr13	41706115	41707267
chr13	41707994	41708084
chr13	41710736	41710962
chr13	41711318	41711575
chr13	41711682	41711912
chr13	41712013	41713189
chr13	41719312	41720109
chr13	41756447	41756689
chr13	41762746	41763097
chr13	41767492	41767910
chr13	41768256	41768657
chr13	41768782	41769002
chr13	41793149	41793462
chr13	41837322	41838094
chr13	41858834	41859146

chr13	41859522	41859524
chr13	41880502	41880582
chr13	41885012	41886228
chr13	41886420	41886619
chr13	41889346	41889669
chr13	41898856	41899362
chr13	41927012	41927995
chr13	41929712	41930447
chr13	42031190	42032504
chr13	42163157	42163440
chr13	42164786	42165127
chr13	42184410	42184753
chr13	42185024	42185371
chr13	42226985	42227027
chr13	42269048	42269095
chr13	42269324	42269403
chr13	42294905	42294972
chr13	42295226	42295377
chr13	42376931	42377076
chr13	42377381	42377498
chr13	42479987	42480101
chr13	42483766	42483947
chr13	42490799	42490990
chr13	42534410	42535642
chr13	42537198	42537397
chr13	42548494	42549042
chr13	42600251	42600910
chr13	42601022	42601188
chr13	42613920	42614927
chr13	42615075	42616311
chr13	42621711	42622415
chr13	42622564	42623189
chr13	42623319	42623783
chr13	42680060	42680194
chr13	42680492	42680724
chr13	42690233	42690462
chr13	42845365	42845900
chr13	42846207	42846539
chr13	42846656	42846886
chr13	43061262	43061413
chr13	43192501	43192758
chr13	43270186	43270271
chr13	43270748	43270757
chr13	43294441	43294557
chr13	43294843	43295010
chr13	43302673	43302980

chr13	43545173	43545254
chr13	43546039	43546114
chr13	43565747	43566657
chr13	43567531	43567730
chr13	43564996	43565238
chr13	43570717	43570727
chr13	43571063	43571084
chr13	43577084	43577519
chr13	43579589	43579908
chr13	43598983	43599973
chr13	43601361	43601614
chr13	43597160	43598156
chr13	43606030	43606321
chr13	43606849	43607021
chr13	43607882	43608107
chr13	43619645	43619704
chr13	43619826	43619847
chr13	43629298	43629766
chr13	43632328	43632436
chr13	43727999	43728220
chr13	43843096	43843440
chr13	44379154	44379316
chr13	44440044	44440124
chr13	44440363	44440374
chr13	44453001	44454357
chr13	44549717	44550421
chr13	44681996	44682323
chr13	44715775	44715972
chr13	44716258	44716810
chr13	44717356	44717417
chr13	44717541	44717819
chr13	44734084	44734158
chr13	44762383	44762785
chr13	44804292	44804424
chr13	44812224	44812580
chr13	44845170	44845637
chr13	44848536	44848780
chr13	44875075	44875779
chr13	44880001	44880662
chr13	44880842	44881181
chr13	44884816	44885220
chr13	44885488	44885667
chr13	44887011	44887735
chr13	44887914	44888258
chr13	44935326	44935394
chr13	44938897	44939439

chr13	45009189	45009419
chr13	45009701	45010059
chr13	45010768	45010961
chr13	45011167	45011431
chr13	45097526	45097857
chr13	45150468	45152242
chr13	45284676	45285269
chr13	45288116	45288651
chr13	45300378	45300406
chr13	45300643	45300701
chr13	45319239	45319534
chr13	45319661	45319805
chr13	45333073	45333415
chr13	45333479	45333679
chr13	45333749	45334091
chr13	45334291	45334321
chr13	45376362	45376522
chr13	45389807	45389889
chr13	45390147	45390295
chr13	45391450	45391741
chr13	45491686	45492014
chr13	45492166	45492912
chr13	45496646	45496984
chr13	45544111	45544191
chr13	45563121	45563442
chr13	45563691	45564449
chr13	45621509	45621625
chr13	45621743	45621895
chr13	45629440	45629674
chr13	45693816	45694098
chr13	45694323	45695044
chr13	45736937	45737302
chr13	45742245	45742285
chr13	45742597	45742819
chr13	45755059	45755182
chr13	45756222	45756456
chr13	45768677	45769030
chr13	45770824	45771020
chr13	45771151	45771169
chr13	45780367	45780537
chr13	45780659	45781110
chr13	45783366	45783668
chr13	45793984	45794439
chr13	45810174	45810576
chr13	45813651	45813850
chr13	45827652	45827755

chr13	45827871	45828067
chr13	45855118	45855124
chr13	45855469	45855602
chr13	45885370	45885553
chr13	45903803	45903834
chr13	45904031	45904222
chr13	45905172	45905198
chr13	45905982	45906376
chr13	45907851	45908344
chr13	45914456	45914589
chr13	45914719	45915323
chr13	45916149	45916599
chr13	45926073	45926313
chr13	45934784	45935096
chr13	45935252	45936442
chr13	45939879	45940040
chr13	45941491	45941609
chr13	45941728	45941988
chr13	45942780	45942919
chr13	45991633	45991677
chr13	45991871	45992876
chr13	46038603	46038933
chr13	46039077	46039817
chr13	46056676	46057064
chr13	46085747	46086142
chr13	46086256	46086685
chr13	46102283	46102317
chr13	46243662	46243728
chr13	46249521	46249707
chr13	46373836	46373845
chr13	46447557	46447820
chr13	46626444	46626812
chr13	46627044	46627216
chr13	46636586	46636770
chr13	46681020	46681086
chr13	46709153	46709424
chr13	46709571	46709735
chr13	46785897	46786133
chr13	46870187	46870212
chr13	46870411	46870596
chr13	46871073	46871159
chr13	46899512	46899638
chr13	46928834	46929028
chr13	46929381	46929582
chr13	46948449	46948505
chr13	46961448	46961717

chr13	46962026	46962092
chr13	47043420	47043535
chr13	47043846	47044258
chr13	47125869	47128718
chr13	47128911	47129055
chr13	47169348	47169812
chr13	47169981	47170127
chr13	47174771	47174802
chr13	47175338	47175469
chr13	47188739	47188819
chr13	47189129	47189132
chr13	47370599	47371698
chr13	48073619	48073728
chr13	48073868	48074008
chr13	48419154	48419602
chr13	48519830	48520002
chr13	48574671	48574887
chr13	48575040	48575410
chr13	48575607	48576506
chr13	48580635	48580651
chr13	48580818	48580862
chr13	48583351	48583798
chr13	48588506	48588667
chr13	48611355	48612442
chr13	48613027	48613220
chr13	48613698	48613848
chr13	48613912	48614278
chr13	48621650	48621849
chr13	48646433	48646524
chr13	48668692	48668883
chr13	48669067	48669184
chr13	48669425	48669782
chr13	48701080	48701152
chr13	48705891	48705955
chr13	48729248	48729627
chr13	48729953	48730384
chr13	48730609	48731007
chr13	48731918	48732148
chr13	48732508	48733158
chr13	48733897	48734391
chr13	48734945	48735368
chr13	48784241	48784788
chr13	48796466	48797003
chr13	48809627	48810007
chr13	48810725	48811048
chr13	48813547	48813891

chr13	48806903	48808188
chr13	48818344	48818566
chr13	48877069	48877785
chr13	48877901	48878417
chr13	48878777	48878909
chr13	48880259	48880495
chr13	48908162	48908200
chr13	48908409	48908523
chr13	48974317	48974686
chr13	48974938	48975448
chr13	48987108	48987522
chr13	49023089	49023288
chr13	49074775	49075364
chr13	49081138	49081557
chr13	49082147	49082587
chr13	49105981	49107487
chr13	49153103	49153551
chr13	49154376	49154407
chr13	49357577	49357813
chr13	49466386	49466447
chr13	49549666	49550663
chr13	49550839	49551177
chr13	49627396	49627518
chr13	49627274	49627280
chr13	49627673	49627717
chr13	49634886	49634911
chr13	49635113	49635159
chr13	49637655	49637795
chr13	49637948	49638060
chr13	49656709	49657256
chr13	49660927	49661083
chr13	49672332	49672743
chr13	49673432	49673489
chr13	49684449	49684555
chr13	49688055	49688389
chr13	49789491	49789587
chr13	49793580	49794073
chr13	49794208	49795159
chr13	49821551	49821981
chr13	49822085	49822466
chr13	49822794	49823056
chr13	49875496	49876177
chr13	49887935	49888654
chr13	49922492	49922691
chr13	50017482	50018185
chr13	50018444	50019495

chr13	50068835	50068918
chr13	50069285	50070554
chr13	50159266	50159489
chr13	50159774	50160526
chr13	50161343	50161755
chr13	50181104	50181318
chr13	50202231	50202514
chr13	50202735	50202846
chr13	50264593	50265687
chr13	50265940	50266357
chr13	50365499	50365754
chr13	50365822	50365853
chr13	50365976	50367129
chr13	50367281	50367841
chr13	50380550	50380798
chr13	50385367	50385578
chr13	50402668	50402833
chr13	50408826	50409059
chr13	50421888	50422524
chr13	50432254	50433067
chr13	50433637	50434140
chr13	50481722	50481799
chr13	50481936	50482250
chr13	50492286	50492642
chr13	50509479	50510467
chr13	50510976	50511557
chr13	50570262	50570472
chr13	50570664	50570918
chr13	50571188	50571838
chr13	50572188	50572407
chr13	50632394	50632593
chr13	50645537	50645568
chr13	50655142	50656105
chr13	50656427	50657161
chr13	50697714	50697890
chr13	50698046	50698572
chr13	50698775	50700705
chr13	50701082	50701407
chr13	50701602	50701901
chr13	50704079	50704483
chr13	50970620	50970872
chr13	51272488	51272593
chr13	51305488	51305883
chr13	51306828	51307078
chr13	51330487	51330844
chr13	51483896	51484981

chr13	51486172	51486182
chr13	51574637	51574731
chr13	51796559	51796845
chr13	51954435	51954778
chr13	52011695	52012206
chr13	52025694	52025980
chr13	52026086	52027111
chr13	52027483	52028300
chr13	52053373	52053572
chr13	52093850	52093932
chr13	52094345	52095009
chr13	52108632	52108842
chr13	52130845	52131302
chr13	52131617	52132520
chr13	52150907	52151354
chr13	52151851	52152207
chr13	52154151	52154193
chr13	52157737	52158073
chr13	52158516	52159579
chr13	52159826	52159975
chr13	52160199	52160215
chr13	52164982	52165509
chr13	52177835	52177914
chr13	52192783	52193009
chr13	52205131	52205631
chr13	52214826	52215225
chr13	52217168	52217233
chr13	52217502	52217809
chr13	52218037	52218474
chr13	52218768	52219203
chr13	52219308	52219853
chr13	52248402	52248827
chr13	52255301	52255630
chr13	52288549	52289439
chr13	52289794	52289798
chr13	52303075	52303429
chr13	52303622	52303633
chr13	52303962	52304653
chr13	52307401	52308229
chr13	52376034	52376142
chr13	52376217	52376269
chr13	52377539	52378531
chr13	52378658	52378919
chr13	52389062	52389310
chr13	52393863	52393983
chr13	52399449	52399547

chr13	52399782	52399843
chr13	52585277	52586357
chr13	52586518	52586709
chr13	52701858	52702288
chr13	52702570	52702704
chr13	52703034	52703288
chr13	52733341	52734193
chr13	52767719	52768006
chr13	52768169	52769104
chr13	52769449	52770113
chr13	52778038	52778076
chr13	52778331	52778530
chr13	52778594	52778872
chr13	52917724	52917774
chr13	52923143	52923532
chr13	52930471	52930580
chr13	52935325	52935842
chr13	52936657	52936741
chr13	52979494	52980693
chr13	52984937	52985230
chr13	52988928	52989432
chr13	52985633	52986183
chr13	53002715	53002834
chr13	53008381	53008795
chr13	53020464	53020762
chr13	53022191	53022397
chr13	53022921	53023629
chr13	53023905	53024888
chr13	53026934	53026976
chr13	53028484	53028635
chr13	53028786	53030279
chr13	53034110	53034231
chr13	53054858	53055219
chr13	53144346	53144545
chr13	53173276	53174763
chr13	53182372	53182472
chr13	53190776	53191941
chr13	53197987	53198101
chr13	53220352	53220531
chr13	53226024	53226834
chr13	53227014	53227483
chr13	53425223	53425462
chr13	53425799	53426013
chr13	53499331	53499333
chr13	53499759	53500693
chr13	53501113	53501230

chr13	53501430	53501441
chr13	53585228	53585255
chr13	54768596	54768598
chr13	54784756	54784862
chr13	55146221	55146729
chr13	55308132	55308166
chr13	55528272	55528334
chr13	56684903	56684997
chr13	56721108	56721148
chr13	56925543	56925635
chr13	56926555	56926594
chr13	56998310	56998409
chr13	56998723	56998913
chr13	57086477	57086785
chr13	57294951	57295073
chr13	57295193	57295358
chr13	57481761	57482093
chr13	57889620	57889640
chr13	58198716	58198761
chr13	58571262	58571302
chr13	58693481	58693534
chr13	59566198	59566325
chr13	60348513	60348615
chr13	60348884	60349151
chr13	60351718	60351809
chr13	60421041	60421118
chr13	60421273	60421355
chr13	60576296	60576438
chr13	60586630	60586930
chr13	60590321	60590333
chr13	60590649	60590689
chr13	60593814	60593904
chr13	60737547	60738038
chr13	60738142	60738810
chr13	60969929	60972261
chr13	60976238	60976490
chr13	61136228	61136367
chr13	61332773	61333118
chr13	61333743	61333839
chr13	61333958	61334004
chr13	61362292	61362335
chr13	61362640	61362684
chr13	61834730	61834929
chr13	62110808	62110874
chr13	62239838	62240165
chr13	62868310	62868458

chr13	62993100	62993357
chr13	63217223	63217422
chr13	63411995	63412079
chr13	65126276	65126335
chr13	65249682	65249881
chr13	65263536	65263722
chr13	65298294	65298335
chr13	67354984	67355065
chr13	67427771	67428053
chr13	67501204	67501707
chr13	67508095	67508436
chr13	67695602	67695664
chr13	67803625	67803904
chr13	67804693	67805437
chr13	67873675	67873732
chr13	67882306	67882549
chr13	67983248	67983262
chr13	68148914	68149153
chr13	68365504	68365555
chr13	68622030	68622215
chr13	68622334	68622387
chr13	68631023	68631369
chr13	68631474	68631707
chr13	68652633	68652724
chr13	68764938	68764984
chr13	69501497	69501586
chr13	70827699	70827826
chr13	70828023	70828064
chr13	70828209	70828359
chr13	70884885	70885119
chr13	70927221	70927383
chr13	71115613	71115726
chr13	71115854	71115971
chr13	71538371	71538595
chr13	72439793	72439924
chr13	72440621	72440691
chr13	72441125	72441324
chr13	72453247	72453431
chr13	72671727	72672059
chr13	72718694	72719007
chr13	72740457	72740613
chr13	72778615	72778705
chr13	72820701	72820917
chr13	72830057	72830135
chr13	72959083	72959277
chr13	72965317	72965486

chr13	73116066	73116176
chr13	73131153	73131317
chr13	73164265	73164581
chr13	73212258	73212981
chr13	73300397	73300609
chr13	73301269	73301856
chr13	73302064	73302601
chr13	73348241	73348494
chr13	73355457	73355963
chr13	73356418	73356605
chr13	73366198	73366245
chr13	73366402	73366539
chr13	73414092	73414352
chr13	73475680	73475885
chr13	73545195	73545428
chr13	73557162	73557979
chr13	73574191	73574465
chr13	73599613	73599772
chr13	73605703	73605888
chr13	73606040	73606143
chr13	73607524	73607728
chr13	73627651	73627675
chr13	73627810	73628073
chr13	73630169	73630684
chr13	73632507	73634041
chr13	73634535	73634735
chr13	73634991	73635392
chr13	73635974	73636312
chr13	73640161	73640523
chr13	73652306	73652399
chr13	73656659	73656863
chr13	73664531	73664539
chr13	73743365	73743562
chr13	73751296	73751345
chr13	73774301	73774552
chr13	73803034	73803039
chr13	73822790	73822948
chr13	73823129	73823241
chr13	73835836	73835908
chr13	73836157	73836442
chr13	73847864	73848298
chr13	73861049	73861514
chr13	73878265	73878390
chr13	73899805	73899884
chr13	73900100	73900247
chr13	73908621	73908962

chr13	73909330	73909712
chr13	73910066	73910431
chr13	73910957	73911371
chr13	73911766	73912029
chr13	73912510	73912938
chr13	73914263	73914427
chr13	73914563	73914612
chr13	73915819	73916306
chr13	73939403	73940225
chr13	73942644	73943636
chr13	73973733	73973740
chr13	73987045	73987427
chr13	73995324	73996089
chr13	74002315	74002693
chr13	74002878	74003232
chr13	74003825	74003936
chr13	74004112	74004231
chr13	74039559	74040196
chr13	74054945	74055080
chr13	74071479	74072041
chr13	74116629	74116644
chr13	74116826	74116977
chr13	74118277	74118411
chr13	74172187	74172262
chr13	74172758	74172907
chr13	74176729	74176846
chr13	74177798	74178144
chr13	74251080	74251875
chr13	74282546	74282660
chr13	74282810	74282883
chr13	74610997	74611012
chr13	74707834	74710271
chr13	74710412	74710827
chr13	75016935	75016981
chr13	75017194	75017263
chr13	75017751	75017875
chr13	75018007	75018478
chr13	75018675	75018732
chr13	75024648	75024721
chr13	75341173	75341384
chr13	75344659	75344672
chr13	75346380	75346466
chr13	75399748	75399852
chr13	75463696	75463842
chr13	75903925	75904126
chr13	75939930	75940124

chr13	75942654	75942666
chr13	75946545	75946671
chr13	75991849	75991936
chr13	75992151	75992159
chr13	76026869	76027217
chr13	76033057	76033131
chr13	76033360	76033446
chr13	76054727	76054734
chr13	76054982	76056771
chr13	76056883	76056982
chr13	76063502	76063975
chr13	76064125	76064485
chr13	76111507	76112519
chr13	76119021	76119097
chr13	76123664	76124762
chr13	76133869	76133926
chr13	76209776	76211326
chr13	76240774	76240895
chr13	76241068	76241202
chr13	76270157	76270385
chr13	76278989	76279850
chr13	76289769	76290110
chr13	76290954	76291457
chr13	76334600	76334916
chr13	76345913	76346034
chr13	76346232	76346322
chr13	76372145	76372280
chr13	76372398	76372506
chr13	76567877	76567976
chr13	76867500	76867830
chr13	76953486	76954227
chr13	76957886	76957999
chr13	76958129	76958148
chr13	77233164	77233643
chr13	77241910	77242056
chr13	77426808	77427497
chr13	77428742	77429427
chr13	77430134	77430787
chr13	77453346	77453656
chr13	77453847	77454010
chr13	77458345	77458739
chr13	77458829	77461030
chr13	77464205	77464637
chr13	77463401	77463808
chr13	77469171	77469236
chr13	77491823	77492252

chr13	77510902	77511238
chr13	77511561	77511736
chr13	77511941	77512028
chr13	77544529	77544582
chr13	77554190	77554648
chr13	77565812	77566098
chr13	77566264	77566774
chr13	77566880	77567228
chr13	77568434	77568649
chr13	77599597	77599940
chr13	77600879	77601280
chr13	77601501	77601836
chr13	77759993	77760242
chr13	77874289	77874633
chr13	77899595	77899858
chr13	77899930	77901694
chr13	77903078	77904146
chr13	78052222	78052809
chr13	78090695	78090857
chr13	78095821	78096553
chr13	78109578	78110071
chr13	78111484	78111536
chr13	78112026	78112135
chr13	78118567	78118592
chr13	78132722	78133069
chr13	78271849	78272145
chr13	78596283	78596287
chr13	78596579	78596682
chr13	79232421	79233267
chr13	79702607	79702720
chr13	79979273	79981027
chr13	79981504	79981741
chr13	80054935	80056243
chr13	80056444	80056749
chr13	80059040	80059703
chr13	80064948	80065148
chr13	80065630	80066202
chr13	80066429	80066921
chr13	80068664	80068902
chr13	80102600	80102860
chr13	80163692	80164383
chr13	80179019	80179369
chr13	80196033	80196237
chr13	80205387	80205496
chr13	80205717	80205874
chr13	80221641	80221807

chr13	80222024	80222195
chr13	80255545	80255570
chr13	80324303	80324420
chr13	80349943	80349974
chr13	80353960	80354052
chr13	80510597	80510677
chr13	80511075	80511270
chr13	80660399	80660529
chr13	80912987	80913738
chr13	80913964	80915049
chr13	80915235	80915967
chr13	80916609	80916968
chr13	80917281	80917490
chr13	81043866	81044355
chr13	81048145	81048247
chr13	81051836	81052051
chr13	81083434	81083675
chr13	81444316	81444662
chr13	81445632	81446258
chr13	81745437	81745492
chr13	83273164	83273206
chr13	84023753	84024191
chr13	84515755	84516148
chr13	84615074	84615274
chr13	84975092	84975242
chr13	85013307	85013382
chr13	85019883	85020017
chr13	85418706	85418749
chr13	85441444	85441770
chr13	85499685	85499886
chr13	85810587	85810968
chr13	86071670	86071847
chr13	86072048	86072091
chr13	86083555	86084101
chr13	86157004	86157294
chr13	86225492	86225606
chr13	86233943	86233998
chr13	86346281	86346316
chr13	86346668	86346673
chr13	86373309	86373511
chr13	86373718	86373846
chr13	86574726	86574925
chr13	87244433	87244470
chr13	87508910	87509007
chr13	87776197	87776323
chr13	87776770	87776792

chr13	87911694	87912049
chr13	87983345	87983472
chr13	87983685	87983860
chr13	88179301	88179456
chr13	88321709	88321736
chr13	88321915	88321975
chr13	88323164	88323519
chr13	88323659	88325683
chr13	88326542	88327015
chr13	88325763	88326473
chr13	88329638	88330387
chr13	88332567	88332783
chr13	88337403	88337462
chr13	88462542	88463722
chr13	88479716	88480108
chr13	88651419	88652007
chr13	88675131	88675156
chr13	89561351	89561662
chr13	89719112	89719213
chr13	89890812	89890905
chr13	89891027	89891087
chr13	89891630	89891639
chr13	89892060	89892153
chr13	89892368	89892489
chr13	90087119	90087257
chr13	90108012	90108166
chr13	90108316	90108400
chr13	90110149	90110370
chr13	90110498	90110648
chr13	90166869	90166894
chr13	90280330	90280387
chr13	90577369	90577493
chr13	91067464	91067489
chr13	91067680	91067817
chr13	91095666	91095992
chr13	91097350	91097510
chr13	91097643	91097835
chr13	91100027	91100324
chr13	91247006	91247307
chr13	91338984	91339061
chr13	91670050	91670255
chr13	91827215	91827682
chr13	91999100	91999165
chr13	91999485	92000493
chr13	92000667	92001246
chr13	92001522	92001595

chr13	92051046	92051245
chr13	92120600	92121025
chr13	95201083	95201937
chr13	95202110	95202696
chr13	95247836	95248911
chr13	95253670	95253816
chr13	95254093	95254861
chr13	95256631	95257091
chr13	95309246	95309644
chr13	95365216	95365415
chr13	95736063	95736437
chr13	95767709	95767875
chr13	95768005	95768192
chr13	95841607	95841707
chr13	95845066	95845123
chr13	95904708	95904800
chr13	95919655	95919956
chr13	95920196	95920395
chr13	95920532	95920691
chr13	95926954	95926991
chr13	95928360	95928639
chr13	95933037	95933193
chr13	95951462	95952000
chr13	95952757	95954034
chr13	95966627	95966758
chr13	95967183	95967399
chr13	95968288	95968345
chr13	95987605	95988315
chr13	96033203	96033304
chr13	96056216	96056584
chr13	96137171	96137236
chr13	96137799	96137846
chr13	96138137	96138277
chr13	96138443	96138553
chr13	96146654	96146676
chr13	96161953	96162334
chr13	96165181	96165561
chr13	96167875	96168074
chr13	96168357	96168359
chr13	96182434	96182495
chr13	96182885	96182921
chr13	96204945	96205300
chr13	96241004	96241276
chr13	96256103	96256382
chr13	96295724	96296109
chr13	96296278	96296878

chr13	96297146	96297487
chr13	96328271	96329138
chr13	96329447	96330094
chr13	96362716	96363017
chr13	96365942	96366143
chr13	96366383	96366672
chr13	96366884	96367096
chr13	96400753	96401188
chr13	96436646	96437213
chr13	96436161	96436416
chr13	96437340	96437633
chr13	96445589	96445638
chr13	96486379	96486568
chr13	96534113	96534447
chr13	96576874	96577275
chr13	96704871	96704916
chr13	96705021	96705560
chr13	96706056	96706333
chr13	96742955	96743165
chr13	97646294	97646304
chr13	97777830	97778037
chr13	97778104	97778229
chr13	97874549	97875009
chr13	97875826	97876576
chr13	97908506	97909084
chr13	97910065	97910728
chr13	97912288	97912311
chr13	97925314	97926173
chr13	97926971	97927696
chr13	97927809	97927838
chr13	97931018	97931138
chr13	97933384	97933562
chr13	97996007	97996017
chr13	97997119	97997140
chr13	98084990	98085163
chr13	98085792	98086188
chr13	98086720	98087167
chr13	98220086	98220198
chr13	98508021	98508121
chr13	98599493	98599598
chr13	98599712	98599984
chr13	98600228	98600566
chr13	98601192	98601240
chr13	98627558	98628722
chr13	98628864	98629691
chr13	98657276	98657483

chr13	98720807	98721155
chr13	98735687	98735904
chr13	98736532	98736664
chr13	98794625	98795241
chr13	98795462	98796281
chr13	98796462	98796647
chr13	98796810	98797046
chr13	98800431	98800579
chr13	98862754	98862877
chr13	98867250	98867380
chr13	98907534	98907735
chr13	98907876	98908525
chr13	99097808	99098030
chr13	99113006	99113171
chr13	99128644	99128855
chr13	99129476	99129851
chr13	99136284	99137117
chr13	99137516	99137990
chr13	99140373	99140557
chr13	99161719	99162218
chr13	99187324	99187557
chr13	99187787	99187933
chr13	99188294	99188699
chr13	99188955	99189257
chr13	99189376	99189880
chr13	99222099	99222167
chr13	99223096	99223428
chr13	99223554	99223772
chr13	99224183	99224518
chr13	99224707	99225030
chr13	99226314	99226513
chr13	99227725	99228070
chr13	99228156	99229350
chr13	99229607	99230104
chr13	99230351	99230566
chr13	99235105	99235131
chr13	99235448	99235607
chr13	99305567	99305608
chr13	99309314	99309430
chr13	99404639	99404896
chr13	99428745	99428913
chr13	99487119	99487297
chr13	99492339	99492344
chr13	99562839	99563129
chr13	99594723	99595122
chr13	99644611	99644715

chr13	99644841	99645040
chr13	99684014	99684156
chr13	99684380	99684600
chr13	99698110	99698562
chr13	99698722	99699106
chr13	99702198	99703060
chr13	99709939	99710211
chr13	99719327	99719922
chr13	99731909	99731987
chr13	99737942	99738602
chr13	99738733	99739931
chr13	99740046	99740481
chr13	99802693	99803243
chr13	99803364	99804013
chr13	99848787	99849746
chr13	99852387	99852884
chr13	99853075	99854192
chr13	99918151	99918231
chr13	99922135	99922146
chr13	99932342	99932653
chr13	99992120	99992461
chr13	100085435	100085729
chr13	100150538	100150630
chr13	100151000	100151306
chr13	100153070	100153239
chr13	100153734	100154601
chr13	100157500	100157827
chr13	100258446	100258705
chr13	100258907	100259398
chr13	100262335	100262399
chr13	100266466	100266610
chr13	100307373	100307637
chr13	100308089	100308527
chr13	100310678	100310944
chr13	100633019	100633281
chr13	100633409	100633778
chr13	100675037	100675759
chr13	100740596	100741166
chr13	100741298	100742233
chr13	100752755	100753153
chr13	100861973	100862643
chr13	100864138	100864252
chr13	100865552	100866189
chr13	100867174	100867280
chr13	100867384	100867573
chr13	100882503	100882597

chr13	100887899	100888165
chr13	100928016	100928123
chr13	100928248	100928465
chr13	100966937	100966953
chr13	101044934	101045030
chr13	101045149	101045607
chr13	101167091	101167092
chr13	101194491	101194559
chr13	101194694	101195354
chr13	101200043	101200153
chr13	101200432	101200548
chr13	101240478	101241371
chr13	101285897	101286264
chr13	101325681	101326085
chr13	101326342	101327245
chr13	101327397	101327963
chr13	101863666	101863700
chr13	102059195	102059269
chr13	102068408	102069253
chr13	102102717	102102800
chr13	102102950	102103232
chr13	102107095	102107460
chr13	102108139	102108230
chr13	102198248	102198297
chr13	102293053	102293687
chr13	102301790	102301900
chr13	102302195	102302242
chr13	102781089	102781091
chr13	102938499	102938754
chr13	102939042	102939201
chr13	103021662	103021839
chr13	103046325	103047428
chr13	103052419	103052766
chr13	103053316	103053614
chr13	103238940	103239101
chr13	103244638	103245187
chr13	103248400	103249090
chr13	103249370	103250118
chr13	103250593	103250788
chr13	103257320	103257357
chr13	103257652	103257667
chr13	103300450	103300834
chr13	103359730	103359901
chr13	103360063	103360352
chr13	103377213	103377447
chr13	103377585	103377839

chr13	103382002	103382438
chr13	103425120	103425265
chr13	103425415	103426577
chr13	103450947	103451320
chr13	103451472	103452242
chr13	103452407	103453248
chr13	103477698	103478322
chr13	103478502	103479089
chr13	103499005	103499360
chr13	103497696	103498640
chr13	103522813	103523012
chr13	103563618	103563833
chr13	103563969	103564157
chr13	103568670	103568782
chr13	103568872	103568941
chr13	103809648	103809847
chr13	103935221	103935282
chr13	104384537	104384698
chr13	104967933	104968033
chr13	104968268	104968421
chr13	104970401	104970501
chr13	105224309	105224358
chr13	105302702	105302928
chr13	105998453	105998541
chr13	105998787	105998893
chr13	106095216	106095363
chr13	106095537	106095720
chr13	106105320	106105359
chr13	106105473	106105672
chr13	106114505	106114871
chr13	106124220	106124424
chr13	106240356	106240651
chr13	106317531	106317612
chr13	106317923	106318024
chr13	106571781	106572182
chr13	106573032	106573099
chr13	106578138	106578310
chr13	106632252	106632326
chr13	106636633	106636674
chr13	106643177	106643266
chr13	106643798	106643851
chr13	106644493	106644496
chr13	106752611	106752710
chr13	106796493	106797111
chr13	106803564	106804215
chr13	106806289	106806472

chr13	106808674	106808836
chr13	106809282	106809326
chr13	106971294	106971376
chr13	107014906	107015321
chr13	107028490	107028574
chr13	107029112	107029289
chr13	107045964	107046094
chr13	107046547	107046636
chr13	107124206	107124352
chr13	107124750	107124984
chr13	107125137	107125315
chr13	107125562	107125564
chr13	107155678	107155695
chr13	107160044	107160180
chr13	107160334	107160673
chr13	107173607	107173906
chr13	107174445	107174471
chr13	107174754	107174851
chr13	107175536	107175653
chr13	107175946	107176011
chr13	107183435	107183647
chr13	107184393	107184699
chr13	107184914	107185279
chr13	107185360	107185997
chr13	107186147	107187431
chr13	107187591	107188042
chr13	107219402	107220813
chr13	107221951	107222040
chr13	107222418	107222608
chr13	107223202	107223287
chr13	107569724	107570610
chr13	107910990	107911144
chr13	108001621	108001639
chr13	108866836	108867642
chr13	108870076	108870230
chr13	108870455	108871529
chr13	108871993	108872194
chr13	109116108	109116113
chr13	109406389	109406491
chr13	109430594	109430793
chr13	109568042	109568647
chr13	109571667	109571925
chr13	109572080	109572237
chr13	109597165	109597502
chr13	109609994	109610479
chr13	109616626	109616698

chr13	109616810	109616978
chr13	109624161	109624344
chr13	109627130	109627334
chr13	109645976	109646055
chr13	109740422	109740621
chr13	109797233	109797473
chr13	109797633	109797675
chr13	109797921	109798000
chr13	109798128	109798281
chr13	109799481	109799977
chr13	109800550	109801047
chr13	109803416	109804027
chr13	109804177	109804376
chr13	109806494	109807380
chr13	109807459	109808190
chr13	109808502	109810189
chr13	109810717	109811111
chr13	109813113	109813389
chr13	109813505	109813923
chr13	109814493	109814511
chr13	109850946	109850963
chr13	109863919	109864109
chr13	109864247	109864361
chr13	109871850	109871973
chr13	109888670	109888824
chr13	109895571	109896381
chr13	109902465	109902718
chr13	109907035	109907455
chr13	109909209	109909879
chr13	109917139	109918521
chr13	109919237	109919531
chr13	109920861	109921208
chr13	109921392	109922126
chr13	109922436	109922637
chr13	109922945	109925618
chr13	109926497	109926789
chr13	109919827	109920353
chr13	109926911	109927214
chr13	109927341	109927563
chr13	110036302	110036450
chr13	110037045	110037391
chr13	110042319	110042376
chr13	110042492	110042713
chr13	110076238	110076338
chr13	110076630	110076847
chr13	110146838	110146948

chr13	110147542	110147678
chr13	110161687	110161797
chr13	110162215	110162223
chr13	110299422	110299781
chr13	110305151	110305314
chr13	110318430	110318546
chr13	110319231	110319822
chr13	110352469	110352540
chr13	110356773	110357191
chr13	110357322	110357731
chr13	110357930	110358175
chr13	110368544	110368635
chr13	110389978	110390731
chr13	110390887	110391225
chr13	110395986	110396065
chr13	110396477	110396588
chr13	110396753	110397349
chr13	110403371	110403549
chr13	110414073	110414090
chr13	110430545	110430576
chr13	110430731	110430996
chr13	110432342	110432710
chr13	110433221	110435023
chr13	110435950	110437170
chr13	110437327	110437530
chr13	110438423	110438706
chr13	110439245	110440311
chr13	110442522	110442650
chr13	110590938	110591028
chr13	110591220	110591337
chr13	110631533	110631650
chr13	110631776	110631945
chr13	110651127	110651640
chr13	110658994	110659027
chr13	110664511	110664948
chr13	110669652	110670428
chr13	110722835	110722860
chr13	110723134	110723334
chr13	110736618	110736735
chr13	110746778	110747112
chr13	110747280	110747633
chr13	110749659	110749863
chr13	110761138	110761360
chr13	110762520	110762618
chr13	110765229	110765458
chr13	110770112	110770197

chr13	110775253	110775278
chr13	110775639	110775873
chr13	110778440	110778811
chr13	110778916	110779155
chr13	110779300	110779397
chr13	110787089	110787253
chr13	110790035	110791234
chr13	110862181	110862327
chr13	110862674	110862713
chr13	110874457	110874669
chr13	110874817	110875059
chr13	110900069	110900397
chr13	110907947	110908369
chr13	110911303	110911692
chr13	110911814	110911878
chr13	110912096	110912099
chr13	110912232	110912438
chr13	110913802	110913962
chr13	110914448	110914664
chr13	110958423	110958622
chr13	110958841	110960999
chr13	110967909	110968108
chr13	110971988	110972249
chr13	110972515	110972714
chr13	110972890	110972968
chr13	110999493	110999697
chr13	111000315	111000654
chr13	111040092	111040291
chr13	111040793	111040888
chr13	111057233	111057426
chr13	111061995	111062233
chr13	111064390	111064457
chr13	111064808	111064958
chr13	111065174	111065432
chr13	111067906	111068020
chr13	111068776	111068919
chr13	111075331	111075622
chr13	111094210	111094236
chr13	111094466	111094937
chr13	111095690	111096333
chr13	111096727	111096932
chr13	111097162	111097238
chr13	111097355	111097420
chr13	111097495	111097602
chr13	111098271	111098777
chr13	111099060	111099179

chr13	111100151	111100637
chr13	111099636	111099955
chr13	111100920	111101010
chr13	111102031	111102067
chr13	111139487	111139746
chr13	111159431	111159482
chr13	111170090	111170289
chr13	111177128	111177500
chr13	111213244	111214092
chr13	111214230	111215157
chr13	111242875	111243013
chr13	111245069	111245213
chr13	111267426	111267831
chr13	111267941	111269041
chr13	111269123	111269327
chr13	111345157	111345235
chr13	111355657	111355728
chr13	111356783	111357524
chr13	111358018	111358775
chr13	111364346	111365294
chr13	111365616	111367728
chr13	111368168	111368662
chr13	111368820	111368888
chr13	111369636	111370131
chr13	111522052	111522251
chr13	111522355	111522581
chr13	111522942	111523329
chr13	111523813	111523949
chr13	111524253	111524542
chr13	111526743	111527388
chr13	111560837	111561021
chr13	111561185	111561355
chr13	111566378	111568381
chr13	111568706	111569200
chr13	111594635	111594834
chr13	111595512	111595792
chr13	111596473	111596916
chr13	111604394	111604617
chr13	111604914	111605083
chr13	111605190	111605472
chr13	111606584	111606714
chr13	111607027	111607491
chr13	111607750	111608726
chr13	111609802	111610107
chr13	111617370	111618230
chr13	111620100	111620214

chr13	111719312	111719731
chr13	111805464	111806855
chr13	111840549	111840665
chr13	111856928	111857211
chr13	111875992	111876104
chr13	113047499	113047648
chr13	113241639	113242762
chr13	113260579	113260716
chr13	113292468	113292606
chr13	113294009	113294187
chr13	113312291	113312298
chr13	113312047	113312142
chr13	113343915	113346109
chr13	113348161	113348280
chr13	113351938	113352106
chr13	113365713	113366032
chr13	113366228	113366607
chr13	113372826	113372998
chr13	113373144	113373547
chr13	113376833	113377348
chr13	113377654	113377935
chr13	113379654	113379853
chr13	113394785	113394854
chr13	113526834	113528011
chr13	113570270	113570526
chr13	113585218	113585273
chr13	113597536	113597900
chr13	113676947	113677167
chr13	113806922	113807632
chr13	113807759	113808064
chr13	113808692	113809012
chr13	113841500	113841793
chr13	113842039	113842054
chr13	113847247	113847310
chr13	113847517	113847676
chr13	113862354	113864660
chr13	113865562	113865953
chr13	113870543	113871101
chr13	113871242	113871370
chr13	113886090	113886409
chr13	113929575	113929607
chr13	113929856	113930083
chr13	113951119	113951413
chr13	113950603	113950798
chr13	113951667	113952335
chr13	113953652	113953694

chr13	113958915	113959208
chr13	113959850	113960116
chr13	113970346	113970470
chr13	113986010	113986651
chr13	114017035	114018463
chr13	114018749	114019072
chr13	114020880	114021079
chr13	114047859	114048389
chr13	114082186	114082596
chr13	114102852	114103045
chr13	114103241	114103502
chr13	114103722	114104521
chr13	114128431	114128587
chr13	114143879	114144078
chr13	114144350	114146640
chr13	114147612	114148346
chr13	114238704	114238925
chr13	114239202	114239987
chr13	114241828	114242140
chr13	114242412	114242484
chr13	114461790	114462731
chr13	114484470	114484686
chr13	114484929	114484932
chr13	114520853	114521052
chr13	114523662	114524229
chr13	114524369	114524805
chr13	114539567	114540270
chr13	114540441	114541580
chr13	114541759	114541983
chr13	114542297	114542545
chr13	114542768	114543033
chr13	114551655	114552886
chr13	114553216	114553353
chr13	114563175	114563462
chr13	114563615	114564036
chr13	114564287	114564951
chr13	114565091	114565634
chr13	114565803	114567332
chr13	114567577	114567919
chr13	114588610	114588674
chr13	114600070	114600133
chr13	114600482	114600641
chr13	114616501	114616700
chr13	114618947	114619185
chr13	114619298	114620286
chr13	114628357	114628553

chr13	114629420	114630472
chr13	114630629	114630851
chr13	114631013	114631547
chr13	114632020	114632319
chr13	114740956	114741155
chr13	114745464	114745541
chr13	114745915	114746755
chr13	114750183	114750493
chr13	114750792	114751290
chr13	114778475	114778747
chr13	114780732	114781926
chr13	114782418	114782670
chr13	114797527	114797800
chr13	114801938	114802012
chr13	114802114	114802205
chr13	114807345	114807739
chr13	114829029	114829328
chr13	114829483	114829872
chr13	114830069	114832008
chr13	114832678	114832929
chr13	114843791	114843990
chr13	114880419	114880868
chr13	114881118	114881341
chr13	114884079	114885300
chr13	114896573	114898382
chr13	114899459	114899559
chr13	114908624	114909099
chr13	115000114	115001037
chr13	115036450	115036663
chr13	115045962	115047360
chr13	115047466	115048232
chr13	115060251	115060524
chr13	115060597	115060649
chr13	115078620	115078946
chr13	115079521	115080409
chr14	19000548	19001085
chr14	19001179	19001655
chr14	19001823	19002192
chr14	19003995	19004462
chr14	19005099	19005373
chr14	19007770	19008234
chr14	19009171	19009671
chr14	19010364	19010736
chr14	19011373	19011729
chr14	19012405	19013068
chr14	19013255	19013472

chr14	19013633	19013976
chr14	19014935	19015156
chr14	19016746	19016960
chr14	19017690	19017954
chr14	19018847	19019297
chr14	19019610	19019781
chr14	19033949	19033983
chr14	19038998	19039346
chr14	19042040	19042321
chr14	19044659	19044790
chr14	20730819	20730925
chr14	20731309	20731382
chr14	20773454	20774058
chr14	20774237	20774413
chr14	20800793	20801726
chr14	20810783	20812084
chr14	20869086	20869374
chr14	20869561	20869860
chr14	20880879	20882299
chr14	20884167	20884296
chr14	20895477	20895701
chr14	20903868	20904125
chr14	20904321	20905268
chr14	20922569	20923911
chr14	20925215	20925366
chr14	20926565	20926583
chr14	20928679	20928820
chr14	20928943	20929668
chr14	20930107	20930373
chr14	20935838	20936063
chr14	20936956	20939016
chr14	20945594	20946024
chr14	20963211	20963756
chr14	20999096	20999260
chr14	21077459	21077599
chr14	21077876	21078562
chr14	21078976	21079007
chr14	21082110	21083053
chr14	21092777	21093501
chr14	21093943	21094138
chr14	21094333	21094472
chr14	21098820	21099059
chr14	21099804	21100058
chr14	21121704	21121903
chr14	21131609	21132036
chr14	21132410	21132656

chr14	21149141	21153357
chr14	21157859	21158213
chr14	21429333	21429820
chr14	21438831	21439644
chr14	21441362	21441576
chr14	21443169	21443435
chr14	21454895	21455073
chr14	21455894	21456138
chr14	21457664	21457875
chr14	21458100	21458784
chr14	21482762	21482873
chr14	21492358	21492647
chr14	21492710	21493790
chr14	21507468	21507515
chr14	21508143	21508868
chr14	21509258	21509457
chr14	21509738	21510866
chr14	21511085	21511287
chr14	21513095	21513178
chr14	21514481	21514681
chr14	21530988	21531085
chr14	21536877	21539638
chr14	21539912	21540537
chr14	21555602	21555840
chr14	21565252	21565845
chr14	21565975	21566323
chr14	21566616	21567352
chr14	21567571	21567838
chr14	21568976	21569785
chr14	21570112	21570218
chr14	21570472	21571059
chr14	21571124	21571512
chr14	21571600	21572738
chr14	21572947	21573100
chr14	21574349	21574446
chr14	21574597	21574951
chr14	21575016	21575088
chr14	21575199	21575431
chr14	21659666	21659772
chr14	21680776	21680964
chr14	21686191	21687222
chr14	21688263	21688339
chr14	21701832	21701922
chr14	21712731	21713176
chr14	21713322	21713368
chr14	21714149	21714634

chr14	21720572	21720729
chr14	21725737	21725749
chr14	21725990	21726053
chr14	21726401	21726600
chr14	21728896	21729031
chr14	21729147	21729252
chr14	21732294	21732655
chr14	21733596	21733943
chr14	21737007	21737291
chr14	21737397	21737622
chr14	21737881	21738535
chr14	21745544	21745715
chr14	21747029	21747221
chr14	21769017	21769446
chr14	21776529	21776861
chr14	21777022	21777451
chr14	21851564	21853017
chr14	21853252	21853350
chr14	21873303	21873356
chr14	21876316	21876338
chr14	21907046	21907228
chr14	21904855	21906505
chr14	21918870	21919151
chr14	21923068	21923294
chr14	21923659	21924410
chr14	21924619	21925172
chr14	21944373	21945905
chr14	21963560	21963713
chr14	21977153	21977472
chr14	21978799	21979850
chr14	21985014	21985153
chr14	21993640	21994699
chr14	21998611	21999150
chr14	21999255	21999466
chr14	22003341	22003506
chr14	22025568	22026530
chr14	22026794	22026881
chr14	22239858	22239915
chr14	22626909	22627070
chr14	22627273	22627400
chr14	22668859	22668968
chr14	22678006	22678053
chr14	22678849	22679058
chr14	22680991	22681016
chr14	22681306	22681435
chr14	22688621	22688642

chr14	22693920	22694064
chr14	22757471	22757925
chr14	22759376	22759656
chr14	22764793	22764969
chr14	22896687	22896880
chr14	22901490	22901627
chr14	22902140	22902162
chr14	22988298	22988826
chr14	22989230	22989434
chr14	23005792	23005980
chr14	23006122	23006302
chr14	23020129	23020242
chr14	23020514	23020745
chr14	23024817	23025255
chr14	23025432	23025804
chr14	23026034	23026397
chr14	23026912	23027280
chr14	23028727	23028865
chr14	23039298	23039419
chr14	23039721	23040160
chr14	23041716	23041946
chr14	23056697	23056919
chr14	23057794	23058138
chr14	23058333	23058992
chr14	23066716	23068157
chr14	23082880	23083852
chr14	23113100	23113254
chr14	23115621	23115982
chr14	23116569	23116891
chr14	23121650	23121710
chr14	23122196	23122218
chr14	23127095	23127243
chr14	23127751	23127889
chr14	23131885	23131905
chr14	23181491	23181579
chr14	23182303	23183060
chr14	23223832	23223862
chr14	23235629	23236691
chr14	23246853	23247064
chr14	23250874	23251026
chr14	23251369	23251411
chr14	23251885	23252117
chr14	23258386	23258839
chr14	23259211	23259511
chr14	23260995	23261013
chr14	23272324	23272585

chr14	23274427	23274560
chr14	23276026	23276216
chr14	23276732	23277137
chr14	23280029	23280106
chr14	23280722	23280741
chr14	23281431	23281795
chr14	23284838	23285037
chr14	23286953	23287080
chr14	23287606	23287772
chr14	23288029	23288380
chr14	23289043	23289358
chr14	23289894	23290383
chr14	23291015	23291825
chr14	23291985	23292106
chr14	23292307	23292684
chr14	23293005	23293211
chr14	23293983	23294205
chr14	23294325	23294534
chr14	23295850	23295925
chr14	23298857	23299623
chr14	23301860	23302069
chr14	23302949	23303388
chr14	23303807	23303885
chr14	23304641	23304953
chr14	23305391	23309178
chr14	23309697	23310483
chr14	23311863	23312274
chr14	23313082	23313559
chr14	23315566	23316047
chr14	23319460	23320659
chr14	23316912	23319355
chr14	23320878	23321195
chr14	23321418	23321734
chr14	23321964	23322815
chr14	23323826	23323845
chr14	23324496	23324626
chr14	23328842	23329221
chr14	23329518	23329806
chr14	23331903	23332055
chr14	23333313	23333660
chr14	23334285	23334431
chr14	23336916	23337194
chr14	23337441	23337669
chr14	23337782	23337852
chr14	23338722	23339263
chr14	23339490	23339901

chr14	23340128	23342807
chr14	23344218	23344476
chr14	23355380	23356623
chr14	23356860	23357578
chr14	23357707	23358141
chr14	23359188	23359223
chr14	23359475	23359781
chr14	23360073	23360287
chr14	23367293	23367478
chr14	23367770	23368088
chr14	23369560	23369857
chr14	23372942	23372995
chr14	23375722	23375752
chr14	23377551	23377552
chr14	23377717	23377752
chr14	23378017	23378216
chr14	23379138	23379185
chr14	23385306	23385735
chr14	23387018	23387050
chr14	23387182	23387239
chr14	23387345	23387709
chr14	23387961	23388503
chr14	23388654	23388927
chr14	23390790	23391687
chr14	23391865	23391911
chr14	23392802	23392907
chr14	23395503	23395662
chr14	23395769	23395968
chr14	23397786	23398587
chr14	23398901	23399158
chr14	23399339	23399677
chr14	23399794	23400307
chr14	23405473	23405617
chr14	23405680	23405885
chr14	23422550	23422698
chr14	23423990	23424189
chr14	23424803	23425264
chr14	23425605	23425744
chr14	23425871	23426345
chr14	23426473	23426802
chr14	23426933	23427242
chr14	23435131	23435608
chr14	23436403	23437074
chr14	23437311	23437350
chr14	23437539	23438188
chr14	23439128	23439393

chr14	23440050	23440249
chr14	23442207	23442367
chr14	23442664	23442925
chr14	23443147	23444473
chr14	23445881	23446440
chr14	23446618	23449969
chr14	23444916	23445717
chr14	23450075	23452645
chr14	23452787	23452915
chr14	23453062	23453476
chr14	23455980	23456213
chr14	23462001	23462130
chr14	23463420	23464080
chr14	23464425	23464544
chr14	23475844	23476338
chr14	23476490	23476686
chr14	23479164	23479771
chr14	23503567	23504706
chr14	23505208	23505446
chr14	23508423	23508730
chr14	23510413	23510699
chr14	23525766	23527399
chr14	23529176	23529538
chr14	23529700	23530193
chr14	23530265	23530352
chr14	23530683	23530961
chr14	23531160	23531408
chr14	23531654	23532268
chr14	23533760	23534157
chr14	23537823	23538158
chr14	23539532	23539754
chr14	23540551	23540706
chr14	23540840	23541654
chr14	23541958	23542238
chr14	23544822	23545473
chr14	23547807	23548003
chr14	23548334	23548370
chr14	23563573	23564343
chr14	23564729	23565231
chr14	23565492	23565860
chr14	23578726	23579668
chr14	23584110	23585437
chr14	23589994	23590639
chr14	23591766	23592038
chr14	23592325	23592672
chr14	23593879	23594325

chr14	23593144	23593611
chr14	23602232	23602283
chr14	23602400	23602613
chr14	23604230	23604447
chr14	23605584	23605786
chr14	23606905	23607749
chr14	23611243	23611368
chr14	23611618	23611674
chr14	23611905	23611973
chr14	23615555	23615765
chr14	23615856	23615904
chr14	23622537	23622653
chr14	23623084	23623493
chr14	23623790	23624084
chr14	23624241	23624886
chr14	23629437	23629723
chr14	23629999	23630865
chr14	23634135	23634370
chr14	23640492	23641426
chr14	23641565	23641806
chr14	23650662	23650823
chr14	23651822	23652198
chr14	23652287	23653146
chr14	23654317	23654699
chr14	23656327	23656706
chr14	23664748	23665085
chr14	23667137	23667381
chr14	23678922	23680081
chr14	23686518	23686629
chr14	23696813	23697242
chr14	23706821	23707466
chr14	23720783	23720989
chr14	23721523	23721967
chr14	23734353	23734598
chr14	23754456	23756082
chr14	23763983	23764260
chr14	23768056	23768442
chr14	23768833	23769067
chr14	23769240	23770260
chr14	23770336	23771131
chr14	23771234	23771739
chr14	23771891	23772342
chr14	23772642	23773044
chr14	23774875	23775218
chr14	23775475	23776757
chr14	23776861	23777265

chr14	23777334	23778077
chr14	23778353	23778446
chr14	23781379	23781638
chr14	23789672	23790205
chr14	23790688	23791746
chr14	23795815	23796014
chr14	23796281	23796510
chr14	23797213	23797260
chr14	23821011	23821765
chr14	23832770	23833161
chr14	23833371	23833875
chr14	23834039	23835994
chr14	23836101	23836326
chr14	23923515	23923617
chr14	23938166	23938497
chr14	23938983	23939154
chr14	24020030	24021217
chr14	24021571	24021861
chr14	24024189	24024572
chr14	24025069	24026459
chr14	24035959	24038105
chr14	24038283	24038374
chr14	24038685	24039177
chr14	24046131	24046855
chr14	24163578	24164062
chr14	24164208	24164261
chr14	24193078	24193570
chr14	24194464	24194516
chr14	24194807	24196104
chr14	24422398	24423575
chr14	24438571	24439458
chr14	24454076	24455000
chr14	24455085	24455288
chr14	24455919	24457141
chr14	24457251	24459244
chr14	24475931	24476278
chr14	24502556	24502723
chr14	24503173	24503294
chr14	24503953	24504225
chr14	24504378	24504390
chr14	24504535	24506352
chr14	24520671	24521450
chr14	24550228	24550787
chr14	24550930	24551524
chr14	24562544	24563229
chr14	24563528	24565108

chr14	24577933	24578189
chr14	24578372	24578436
chr14	24579245	24579752
chr14	24582588	24585216
chr14	24604815	24605196
chr14	24605320	24606588
chr14	24609461	24610842
chr14	24609069	24609122
chr14	24611120	24611995
chr14	24614357	24614504
chr14	24614857	24616513
chr14	24616657	24618055
chr14	24618125	24618872
chr14	24628310	24629349
chr14	24629602	24631667
chr14	24631990	24632464
chr14	24632572	24632717
chr14	24637110	24637313
chr14	24640460	24640789
chr14	24643587	24643818
chr14	24644960	24645208
chr14	24657280	24657988
chr14	24658356	24658408
chr14	24663900	24665726
chr14	24666245	24666431
chr14	24667406	24667808
chr14	24668707	24668708
chr14	24681623	24681870
chr14	24682144	24682983
chr14	24684230	24684429
chr14	24684620	24685360
chr14	24688542	24688593
chr14	24693649	24694341
chr14	24698475	24698754
chr14	24698844	24699382
chr14	24700928	24703147
chr14	24705640	24705650
chr14	24708810	24708844
chr14	24707656	24708338
chr14	24709034	24709094
chr14	24709341	24709652
chr14	24709997	24710607
chr14	24710832	24713149
chr14	24715813	24716235
chr14	24720961	24721080
chr14	24727327	24727458

chr14	24727588	24728049
chr14	24728176	24729141
chr14	24732109	24732645
chr14	24733303	24734478
chr14	24742785	24744077
chr14	24740002	24741233
chr14	24745352	24745785
chr14	24748464	24749013
chr14	24749162	24750108
chr14	24751074	24751350
chr14	24752379	24752574
chr14	24752839	24753270
chr14	24753646	24754644
chr14	24754857	24755482
chr14	24755588	24755616
chr14	24755839	24756077
chr14	24757268	24757465
chr14	24758428	24758596
chr14	24765932	24766437
chr14	24766788	24767233
chr14	24767954	24769636
chr14	24777556	24777755
chr14	24778102	24778283
chr14	24778525	24781736
chr14	24782176	24782270
chr14	24800857	24801893
chr14	24802125	24802737
chr14	24802941	24804038
chr14	24817658	24817908
chr14	24824139	24824402
chr14	24827384	24827463
chr14	24834767	24836345
chr14	24836498	24837048
chr14	24837285	24838208
chr14	24840972	24841175
chr14	24860557	24860741
chr14	24861465	24861892
chr14	24865740	24865895
chr14	24866087	24866097
chr14	24868039	24868691
chr14	24895140	24895268
chr14	24895929	24896314
chr14	24898384	24900627
chr14	24900896	24901424
chr14	24911040	24911987
chr14	24912129	24912374

chr14	24995748	24995829
chr14	25011487	25012145
chr14	25026024	25026220
chr14	25147224	25147558
chr14	25148076	25148252
chr14	25275651	25275774
chr14	25518970	25519080
chr14	25519154	25519278
chr14	26670254	26670570
chr14	26751857	26752224
chr14	27066830	27067120
chr14	27067268	27067553
chr14	27243505	27243771
chr14	27244956	27245145
chr14	27311017	27311178
chr14	27311474	27311803
chr14	27454022	27454111
chr14	27896988	27897085
chr14	27897267	27897391
chr14	28268303	28268637
chr14	28285092	28285652
chr14	28526647	28526974
chr14	28851995	28852167
chr14	28852311	28852380
chr14	29047090	29047441
chr14	29120404	29120851
chr14	29121886	29122168
chr14	29122337	29122433
chr14	29122674	29122787
chr14	29122915	29123124
chr14	29140686	29140897
chr14	29145709	29145788
chr14	29146023	29146133
chr14	29233672	29233782
chr14	29234935	29235399
chr14	29235652	29235981
chr14	29254851	29255109
chr14	29706379	29706391
chr14	30018366	30018378
chr14	30146830	30147091
chr14	30275096	30275207
chr14	30376031	30376148
chr14	30396061	30397268
chr14	30406651	30406699
chr14	30662934	30663322
chr14	30664102	30664301

chr14	30680154	30680192
chr14	30912641	30912730
chr14	31028035	31028752
chr14	31072618	31072631
chr14	31072893	31072973
chr14	31077899	31078283
chr14	31079909	31079974
chr14	31080524	31080544
chr14	31090548	31091921
chr14	31098675	31099062
chr14	31108162	31108499
chr14	31125475	31125715
chr14	31160027	31160081
chr14	31343308	31344702
chr14	31390572	31390824
chr14	31443879	31444092
chr14	31450228	31450468
chr14	31474214	31474405
chr14	31486112	31486296
chr14	31494408	31496831
chr14	31498777	31499108
chr14	31500743	31501381
chr14	31503010	31503361
chr14	31503465	31503511
chr14	31516771	31517086
chr14	31517531	31518124
chr14	31523782	31524198
chr14	31524438	31524783
chr14	31525587	31525973
chr14	31526137	31526600
chr14	31527963	31529130
chr14	31530002	31530394
chr14	31533691	31533806
chr14	31539579	31540053
chr14	31542858	31543056
chr14	31549312	31549341
chr14	31549472	31549896
chr14	31551214	31551323
chr14	31559928	31560104
chr14	31563476	31563864
chr14	31568659	31568849
chr14	31606415	31606862
chr14	31625387	31625408
chr14	31633285	31633574
chr14	31634921	31635677
chr14	31640384	31640654

chr14	31653487	31653707
chr14	31661235	31661434
chr14	31664397	31664739
chr14	31665097	31665689
chr14	31669186	31669258
chr14	31669497	31669801
chr14	31674898	31675594
chr14	31675936	31677780
chr14	31698777	31698934
chr14	31699198	31699409
chr14	31712141	31712351
chr14	31712988	31713634
chr14	31714368	31714949
chr14	31732588	31733248
chr14	31760613	31760708
chr14	31795342	31795382
chr14	31795551	31795683
chr14	31805831	31806000
chr14	31806130	31806352
chr14	31825465	31825646
chr14	31830468	31830799
chr14	31831988	31832165
chr14	31883970	31884184
chr14	31888943	31889740
chr14	31889956	31890365
chr14	31897913	31898033
chr14	31926064	31926587
chr14	31926832	31926991
chr14	31931516	31931778
chr14	31931923	31932050
chr14	32030374	32031055
chr14	32288052	32288155
chr14	32312832	32312914
chr14	32334555	32334813
chr14	32371129	32371243
chr14	32371491	32371622
chr14	32396323	32396686
chr14	32396817	32397074
chr14	32403866	32404242
chr14	32407220	32407390
chr14	32417173	32417485
chr14	32419128	32419294
chr14	32420283	32420433
chr14	32491837	32492057
chr14	32492263	32492388
chr14	32492593	32493009

chr14	32496519	32496539
chr14	32497840	32497993
chr14	32543085	32543142
chr14	32544983	32545932
chr14	32546176	32547622
chr14	32548098	32548460
chr14	32554398	32554624
chr14	32555238	32555543
chr14	32556865	32557004
chr14	32670148	32670409
chr14	32670726	32672025
chr14	32672418	32673039
chr14	32673239	32673556
chr14	32699188	32699693
chr14	32721119	32721318
chr14	32723800	32724240
chr14	32728865	32729282
chr14	32811233	32811528
chr14	32811700	32811764
chr14	32814509	32814766
chr14	32840135	32840316
chr14	32852148	32852298
chr14	32867397	32867424
chr14	32867569	32867869
chr14	32874109	32874381
chr14	32887352	32887556
chr14	32887680	32887751
chr14	32938108	32938505
chr14	32941858	32943190
chr14	32944220	32944434
chr14	32976784	32977308
chr14	33029405	33029418
chr14	33029796	33029840
chr14	33075548	33075862
chr14	33408520	33408748
chr14	33408972	33409199
chr14	33590260	33590556
chr14	33992505	33992775
chr14	34025597	34025636
chr14	34033959	34034429
chr14	34058461	34058556
chr14	34058812	34058941
chr14	34080766	34080958
chr14	34115944	34116696
chr14	34116991	34117123
chr14	34145258	34145648

chr14	34261902	34262048
chr14	34407585	34407916
chr14	34419700	34420211
chr14	34420443	34420624
chr14	34459459	34459487
chr14	34504572	34504577
chr14	34504704	34504975
chr14	34505182	34505404
chr14	34511836	34511892
chr14	34512215	34512262
chr14	34512824	34513347
chr14	34523039	34523085
chr14	34529030	34529961
chr14	34550407	34551405
chr14	34554224	34554542
chr14	34567948	34568035
chr14	34582227	34582819
chr14	34608320	34608681
chr14	34608797	34609500
chr14	34616647	34616694
chr14	34630624	34631262
chr14	34671674	34671732
chr14	34672691	34673006
chr14	34688845	34689349
chr14	34689629	34689828
chr14	34690985	34691470
chr14	34766823	34767758
chr14	34800876	34801725
chr14	34828981	34829093
chr14	34832994	34833490
chr14	34833793	34833926
chr14	34837787	34838352
chr14	34838903	34839330
chr14	34844184	34844528
chr14	34844613	34844983
chr14	34857407	34857738
chr14	34858025	34858150
chr14	34858299	34858408
chr14	34923327	34923374
chr14	34927468	34927581
chr14	34930157	34930226
chr14	34930734	34931492
chr14	34931639	34932225
chr14	34932725	34932811
chr14	34958112	34958471
chr14	34959585	34959888

chr14	34960451	34960526
chr14	34965841	34967068
chr14	34967905	34968157
chr14	34981353	34981479
chr14	34982104	34982330
chr14	34988361	34988888
chr14	34988976	34989424
chr14	34989500	34990128
chr14	35001663	35001872
chr14	35003420	35003621
chr14	35008438	35009186
chr14	35009455	35009661
chr14	35014942	35018224
chr14	35020328	35020543
chr14	35024027	35026710
chr14	35026925	35027064
chr14	35028822	35028946
chr14	35034813	35035046
chr14	35039507	35039752
chr14	35042109	35042111
chr14	35045534	35046360
chr14	35046547	35046746
chr14	35049298	35049478
chr14	35056777	35056892
chr14	35057039	35057635
chr14	35087571	35087888
chr14	35089435	35089673
chr14	35093790	35093878
chr14	35098061	35098348
chr14	35098636	35099820
chr14	35111453	35111523
chr14	35119849	35120544
chr14	35131822	35131963
chr14	35135302	35135377
chr14	35135641	35135652
chr14	35162289	35162384
chr14	35169097	35169125
chr14	35169259	35169692
chr14	35177546	35177958
chr14	35180348	35180444
chr14	35182814	35184325
chr14	35187552	35187562
chr14	35187696	35187867
chr14	35192864	35193064
chr14	35193939	35193990
chr14	35202491	35202622

chr14	35202833	35202838
chr14	35213203	35213211
chr14	35219962	35220227
chr14	35241983	35242274
chr14	35248117	35248296
chr14	35250377	35250904
chr14	35283809	35283985
chr14	35286532	35286670
chr14	35286875	35286926
chr14	35288074	35288268
chr14	35295891	35296015
chr14	35296207	35296272
chr14	35312186	35312909
chr14	35317187	35317278
chr14	35320606	35320711
chr14	35320872	35321270
chr14	35325525	35325724
chr14	35325789	35326630
chr14	35333519	35333692
chr14	35342051	35342114
chr14	35342241	35344859
chr14	35445077	35445092
chr14	35450021	35450827
chr14	35451017	35452651
chr14	35506464	35506619
chr14	35515097	35516486
chr14	35553341	35553361
chr14	35589569	35589709
chr14	35590350	35592298
chr14	35592437	35592703
chr14	35602744	35602850
chr14	35625176	35625393
chr14	35625535	35625622
chr14	35628993	35629610
chr14	35665860	35666147
chr14	35666232	35666586
chr14	35682414	35682759
chr14	35683058	35684034
chr14	35684303	35685065
chr14	35703266	35703598
chr14	35746338	35746599
chr14	35749788	35749887
chr14	35756909	35756921
chr14	35761186	35762363
chr14	35763399	35763874
chr14	35765091	35765290

chr14	35769118	35769317
chr14	35771034	35771347
chr14	35775414	35775732
chr14	35787335	35787663
chr14	35789768	35789795
chr14	35790011	35790119
chr14	35800681	35802958
chr14	35803084	35803954
chr14	35804513	35805367
chr14	35805475	35806897
chr14	35807289	35807629
chr14	35809475	35809845
chr14	35810087	35810149
chr14	35810268	35811390
chr14	35814182	35814409
chr14	35824426	35824690
chr14	35825183	35825369
chr14	35829771	35830001
chr14	35830178	35830397
chr14	35834660	35834924
chr14	35835483	35835848
chr14	35836716	35837052
chr14	35837287	35837309
chr14	35838442	35838956
chr14	35839064	35839922
chr14	35840551	35840624
chr14	35841674	35842248
chr14	35842803	35843122
chr14	35843278	35844124
chr14	35844492	35845380
chr14	35860091	35860309
chr14	35860506	35860664
chr14	35861026	35861273
chr14	35865909	35866270
chr14	35866526	35867772
chr14	35868088	35869120
chr14	35869372	35869719
chr14	35869903	35870746
chr14	35871420	35873020
chr14	35873142	35874946
chr14	35876137	35876857
chr14	35877890	35878182
chr14	35881656	35881868
chr14	35882406	35882592
chr14	35883564	35883715
chr14	35886491	35886595

chr14	35888499	35889621
chr14	35890576	35890731
chr14	35891629	35891652
chr14	35891780	35891960
chr14	35907658	35907950
chr14	35908098	35908187
chr14	36002834	36003088
chr14	36138959	36139097
chr14	36139396	36139488
chr14	36156566	36156577
chr14	36277678	36278506
chr14	36278692	36278730
chr14	36285025	36285183
chr14	36291662	36292167
chr14	36293733	36294064
chr14	36294284	36294487
chr14	36295146	36295319
chr14	36295573	36296409
chr14	36299534	36299733
chr14	36327929	36328311
chr14	36335892	36336091
chr14	36346781	36346892
chr14	36347695	36348013
chr14	36749100	36749451
chr14	36785492	36785679
chr14	36789276	36790145
chr14	37050197	37050422
chr14	37117003	37117285
chr14	37236828	37236929
chr14	37285644	37285831
chr14	37285958	37286093
chr14	37289576	37289729
chr14	37290204	37290471
chr14	37443324	37443643
chr14	37451250	37451409
chr14	37451585	37451749
chr14	37550735	37550828
chr14	37586243	37586532
chr14	37641220	37641799
chr14	37666587	37667633
chr14	37668790	37669003
chr14	37917651	37917687
chr14	38049226	38049285
chr14	38053028	38053349
chr14	38063328	38063401
chr14	38063710	38064312

chr14	38064537	38064681
chr14	38064903	38064984
chr14	38065144	38065343
chr14	38091649	38092034
chr14	39484678	39484925
chr14	39485694	39485713
chr14	39518773	39518947
chr14	39566676	39566703
chr14	39571678	39572989
chr14	39583227	39583376
chr14	39583535	39583898
chr14	39624276	39624318
chr14	39633772	39633971
chr14	39638677	39640135
chr14	39643771	39644133
chr14	39644461	39645126
chr14	39657449	39657622
chr14	39672916	39673811
chr14	39687334	39687577
chr14	39687729	39687940
chr14	39701987	39702350
chr14	39734466	39734658
chr14	39735397	39736925
chr14	39745239	39745272
chr14	39820898	39821064
chr14	39897079	39897230
chr14	39900328	39901689
chr14	39901851	39902644
chr14	39961503	39961706
chr14	40426534	40426571
chr14	40887150	40887159
chr14	40916533	40916578
chr14	41607001	41607074
chr14	41607232	41607303
chr14	42074029	42074755
chr14	42299826	42300083
chr14	42300254	42300301
chr14	42662265	42662484
chr14	42684369	42684565
chr14	43353450	43353693
chr14	44008438	44008633
chr14	44090547	44090857
chr14	44577787	44577943
chr14	44578193	44578241
chr14	44786064	44786125
chr14	45030634	45030807

chr14	45315487	45315620
chr14	45366118	45366186
chr14	45366482	45367269
chr14	45381241	45381589
chr14	45382848	45383236
chr14	45430312	45431171
chr14	45431433	45432531
chr14	45432900	45433297
chr14	45433492	45433642
chr14	45480585	45480784
chr14	45483226	45483486
chr14	45552985	45553094
chr14	45553279	45554000
chr14	45559016	45559380
chr14	45561004	45561206
chr14	45582678	45582987
chr14	45588921	45589129
chr14	45595205	45595567
chr14	45600496	45600538
chr14	45603012	45604086
chr14	45604795	45604905
chr14	45605222	45605691
chr14	45609859	45609922
chr14	45620876	45621090
chr14	45646290	45646627
chr14	45649673	45649891
chr14	45653646	45653845
chr14	45716653	45717019
chr14	45722127	45723596
chr14	45737707	45737734
chr14	45831003	45831320
chr14	45895935	45895941
chr14	46020410	46020479
chr14	46020695	46020947
chr14	46149335	46149447
chr14	46183130	46183189
chr14	46254471	46254570
chr14	46523709	46523733
chr14	46524049	46524119
chr14	46957569	46957726
chr14	46957912	46957998
chr14	46959357	46959474
chr14	46959658	46959858
chr14	47041538	47041557
chr14	48144921	48145184
chr14	49715761	49715923

chr14	49966334	49966582
chr14	50019078	50019410
chr14	50047432	50047557
chr14	50047775	50048001
chr14	50048119	50048160
chr14	50050752	50050978
chr14	50051709	50052028
chr14	50052528	50053029
chr14	50053263	50053774
chr14	50059867	50060171
chr14	50064289	50064652
chr14	50065050	50066142
chr14	50066253	50066323
chr14	50066536	50067115
chr14	50068570	50068931
chr14	50070817	50070953
chr14	50073265	50073513
chr14	50082490	50082901
chr14	50083331	50083592
chr14	50084103	50084619
chr14	50086768	50088533
chr14	50088707	50089044
chr14	50090542	50091250
chr14	50099685	50100019
chr14	50100139	50103051
chr14	50106049	50106230
chr14	50113061	50113275
chr14	50146824	50146925
chr14	50153768	50154156
chr14	50154306	50155395
chr14	50159234	50159253
chr14	50159600	50160353
chr14	50229337	50229414
chr14	50234039	50236585
chr14	50236911	50237192
chr14	50318969	50320025
chr14	50320276	50320945
chr14	50328325	50328882
chr14	50328985	50329533
chr14	50329848	50330261
chr14	50332101	50332467
chr14	50333271	50333478
chr14	50333790	50334155
chr14	50334406	50335003
chr14	50335272	50335404
chr14	50342411	50342864

chr14	50345193	50345323
chr14	50346323	50346457
chr14	50346690	50346867
chr14	50347132	50347469
chr14	50358977	50361006
chr14	50362243	50362488
chr14	50364739	50365019
chr14	50373897	50374285
chr14	50374496	50375101
chr14	50378277	50378725
chr14	50380767	50380966
chr14	50396545	50396866
chr14	50413301	50413864
chr14	50416900	50418346
chr14	50419459	50419816
chr14	50420844	50421182
chr14	50428224	50428644
chr14	50429253	50429770
chr14	50433485	50433574
chr14	50439030	50439174
chr14	50439298	50439986
chr14	50441793	50442308
chr14	50462227	50462426
chr14	50465825	50466265
chr14	50467003	50467969
chr14	50468131	50468902
chr14	50469417	50470852
chr14	50473736	50474261
chr14	50474507	50474743
chr14	50475203	50476182
chr14	50483837	50483988
chr14	50485372	50485391
chr14	50490406	50490731
chr14	50490799	50490994
chr14	50502194	50502544
chr14	50503835	50504034
chr14	50504977	50505930
chr14	50506378	50507736
chr14	50513869	50514068
chr14	50527230	50529774
chr14	50547796	50548093
chr14	50548204	50549289
chr14	50560523	50560556
chr14	50560800	50560807
chr14	50574643	50574714
chr14	50583036	50583639

chr14	50610204	50610340
chr14	50634081	50634495
chr14	50653760	50654006
chr14	50685588	50685906
chr14	50692252	50692494
chr14	50697191	50699202
chr14	50770442	50770891
chr14	50778264	50778341
chr14	50778474	50779810
chr14	50785904	50786630
chr14	50786994	50787502
chr14	50787738	50788393
chr14	50809389	50809602
chr14	50814382	50814527
chr14	50827813	50827982
chr14	50830394	50830497
chr14	50830715	50830943
chr14	50831576	50831881
chr14	50862276	50862477
chr14	50862771	50864225
chr14	50864330	50864631
chr14	50914274	50914339
chr14	50914619	50914652
chr14	50919938	50920018
chr14	50988078	50988199
chr14	50988464	50988585
chr14	50988997	50989139
chr14	50998512	51000079
chr14	51009587	51009660
chr14	51026586	51027000
chr14	51027252	51027862
chr14	51068137	51068521
chr14	51086606	51086699
chr14	51122282	51122552
chr14	51133570	51133844
chr14	51134012	51135625
chr14	51143904	51144711
chr14	51181431	51181591
chr14	51208299	51208526
chr14	51288603	51288827
chr14	51296854	51298245
chr14	51298532	51298610
chr14	51326079	51326383
chr14	51326537	51327098
chr14	51363073	51363506
chr14	51363705	51364137

chr14	51404019	51404400
chr14	51404513	51404743
chr14	51405670	51406001
chr14	51409810	51412198
chr14	51412500	51412815
chr14	51418962	51418986
chr14	51421551	51422822
chr14	51423544	51423991
chr14	51428448	51428657
chr14	51544154	51544385
chr14	51544723	51544762
chr14	51546307	51546425
chr14	51553978	51554150
chr14	51560405	51560645
chr14	51561581	51561883
chr14	51562055	51563049
chr14	51590117	51590282
chr14	51706593	51706739
chr14	51707001	51707541
chr14	51709284	51709492
chr14	51750088	51750182
chr14	51750346	51750435
chr14	51794930	51795028
chr14	51800502	51801203
chr14	51804156	51804267
chr14	51804381	51804562
chr14	51808955	51809098
chr14	51823348	51823463
chr14	51824166	51824413
chr14	51834277	51834613
chr14	51835341	51835391
chr14	51835673	51835708
chr14	51848654	51848972
chr14	51849177	51849354
chr14	51849470	51849783
chr14	51853769	51853982
chr14	51854113	51854236
chr14	51861510	51862660
chr14	51862843	51864195
chr14	51865482	51866114
chr14	51909239	51909966
chr14	51941682	51941927
chr14	51946672	51946871
chr14	51952117	51952316
chr14	51955233	51955995
chr14	51956868	51957958

chr14	51965246	51965503
chr14	51999426	52000397
chr14	52000614	52001337
chr14	52001412	52001789
chr14	52006161	52006810
chr14	52007178	52007385
chr14	52011657	52012457
chr14	52013877	52014114
chr14	52014253	52014458
chr14	52019465	52019565
chr14	52019031	52019282
chr14	52021257	52021445
chr14	52027210	52027582
chr14	52029481	52029660
chr14	52031665	52032343
chr14	52083692	52083779
chr14	52083961	52084114
chr14	52099999	52100411
chr14	52115199	52115437
chr14	52116387	52116617
chr14	52117332	52118369
chr14	52119882	52120261
chr14	52118495	52119681
chr14	52141474	52141629
chr14	52183373	52183641
chr14	52219348	52219574
chr14	52236112	52236386
chr14	52242747	52243240
chr14	52372541	52372891
chr14	52455833	52456960
chr14	52534486	52534694
chr14	52534901	52535817
chr14	52547129	52547501
chr14	52547672	52547919
chr14	52781271	52781553
chr14	52795156	52795348
chr14	52821833	52822019
chr14	52924568	52924654
chr14	52924872	52925222
chr14	53018308	53018359
chr14	53018465	53018757
chr14	53019611	53020222
chr14	53161449	53162957
chr14	53167267	53167751
chr14	53167983	53168244
chr14	53169929	53170092

chr14	53170310	53170430
chr14	53173488	53173801
chr14	53173920	53174478
chr14	53190956	53191159
chr14	53196400	53196530
chr14	53196777	53197658
chr14	53201048	53201087
chr14	53211925	53212228
chr14	53245325	53245859
chr14	53252594	53252744
chr14	53255794	53256011
chr14	53257638	53259181
chr14	53302341	53302581
chr14	53329703	53330053
chr14	53338476	53338676
chr14	53340404	53340575
chr14	53340683	53340778
chr14	53367413	53367483
chr14	53367675	53367924
chr14	53369839	53370192
chr14	53372244	53372513
chr14	53372886	53373111
chr14	53385289	53385653
chr14	53393918	53394109
chr14	53416391	53416788
chr14	53417033	53417911
chr14	53418057	53418523
chr14	53428143	53428196
chr14	53618307	53620563
chr14	53684125	53684840
chr14	53745744	53746209
chr14	53773169	53773226
chr14	53925361	53925564
chr14	53976090	53976449
chr14	54391503	54391564
chr14	54419752	54420010
chr14	54420221	54420888
chr14	54421099	54421624
chr14	54421736	54421935
chr14	54424201	54424213
chr14	54430147	54430454
chr14	54460957	54461229
chr14	54637048	54637117
chr14	54656435	54656539
chr14	54656719	54656844
chr14	54678667	54678678

chr14	54779290	54779508
chr14	54863239	54863580
chr14	54863702	54864926
chr14	54906313	54906761
chr14	54907496	54908094
chr14	54908233	54908669
chr14	54909423	54909571
chr14	54912979	54913446
chr14	54913871	54914307
chr14	54919945	54920025
chr14	54920378	54920387
chr14	54920742	54921091
chr14	54923966	54924324
chr14	54928861	54928894
chr14	54929008	54929388
chr14	54947663	54948191
chr14	54954475	54954834
chr14	54955026	54956215
chr14	54958569	54958714
chr14	54970573	54970615
chr14	54976582	54977624
chr14	55023257	55023669
chr14	55031465	55031603
chr14	55031822	55035297
chr14	55035834	55036386
chr14	55037379	55037416
chr14	55037584	55037751
chr14	55060522	55060883
chr14	55064418	55064980
chr14	55066647	55066698
chr14	55116994	55117079
chr14	55118846	55119366
chr14	55119699	55119959
chr14	55125464	55125826
chr14	55136608	55136763
chr14	55143990	55144137
chr14	55144363	55144435
chr14	55149505	55149779
chr14	55149966	55150076
chr14	55150805	55151532
chr14	55152346	55152350
chr14	55155838	55155964
chr14	55164064	55164138
chr14	55239030	55239311
chr14	55239817	55239964
chr14	55240274	55241076

chr14	55241282	55241534
chr14	55272315	55272534
chr14	55334876	55334918
chr14	55337034	55337565
chr14	55347996	55348518
chr14	55348672	55348853
chr14	55349002	55349017
chr14	55355252	55355499
chr14	55361786	55361930
chr14	55367449	55367534
chr14	55368189	55369760
chr14	55401868	55402023
chr14	55402698	55403181
chr14	55493484	55494401
chr14	55505477	55505745
chr14	55508215	55508313
chr14	55508580	55508594
chr14	55517876	55518095
chr14	55518358	55519004
chr14	55536774	55537381
chr14	55538504	55539348
chr14	55541295	55541442
chr14	55542373	55542744
chr14	55543132	55543354
chr14	55543837	55545258
chr14	55556208	55556467
chr14	55556683	55556866
chr14	55561266	55561314
chr14	55561536	55561921
chr14	55565566	55565847
chr14	55566429	55566724
chr14	55568817	55569352
chr14	55569700	55570041
chr14	55570147	55570427
chr14	55570957	55571269
chr14	55574480	55575438
chr14	55581850	55581859
chr14	55584532	55584731
chr14	55584977	55585104
chr14	55589211	55589407
chr14	55595630	55597296
chr14	55611040	55611113
chr14	55657703	55658962
chr14	55679805	55680202
chr14	55687886	55688107
chr14	55727491	55727634

chr14	55736463	55736561
chr14	55737678	55737898
chr14	55738046	55739031
chr14	55764678	55764819
chr14	55795010	55795023
chr14	55797289	55797413
chr14	55833021	55833343
chr14	55834187	55834397
chr14	55878086	55878432
chr14	55878567	55878868
chr14	55879326	55879742
chr14	55896725	55896881
chr14	55913652	55913909
chr14	55914657	55914833
chr14	55915075	55915284
chr14	55921267	55921806
chr14	55925480	55925572
chr14	55949887	55950242
chr14	56002578	56002915
chr14	56045892	56046977
chr14	56047190	56048220
chr14	56052607	56052915
chr14	56055952	56056300
chr14	56066077	56066600
chr14	56348447	56348475
chr14	56355715	56355879
chr14	56356086	56356198
chr14	56559435	56559550
chr14	56585078	56585462
chr14	56656438	56656446
chr14	56806571	56807170
chr14	57046014	57047054
chr14	57050794	57051135
chr14	57075342	57075797
chr14	57133225	57133841
chr14	57734948	57736166
chr14	57736458	57736766
chr14	57743041	57743393
chr14	57819593	57819726
chr14	57849349	57849408
chr14	57856468	57858237
chr14	57858482	57858555
chr14	58499255	58499529
chr14	58539345	58539354
chr14	58545195	58545307
chr14	58618349	58618908

chr14	58632892	58633202
chr14	58638212	58638335
chr14	58666109	58667512
chr14	58706458	58706818
chr14	58710535	58710808
chr14	58711050	58711449
chr14	58711622	58712148
chr14	58724935	58725207
chr14	58744459	58744513
chr14	58747916	58748415
chr14	58764154	58764252
chr14	58764564	58764866
chr14	58765213	58766283
chr14	58826512	58826711
chr14	58861108	58861307
chr14	58862163	58863354
chr14	58893480	58894314
chr14	58894522	58894864
chr14	58894971	58895307
chr14	58897024	58897258
chr14	58922311	58922494
chr14	58955250	58955477
chr14	59104449	59105247
chr14	59130987	59131030
chr14	59213388	59213453
chr14	59480351	59480418
chr14	59654737	59655173
chr14	59655471	59656451
chr14	59656892	59657049
chr14	59658599	59658959
chr14	59659548	59659719
chr14	59683231	59683530
chr14	59734758	59734943
chr14	59740492	59740790
chr14	59741627	59742083
chr14	59742550	59742825
chr14	59745184	59745290
chr14	59748476	59749523
chr14	59773445	59773662
chr14	59773773	59773946
chr14	59788056	59788518
chr14	59813875	59814605
chr14	59820780	59821057
chr14	59821183	59821362
chr14	59821650	59821808
chr14	59824735	59824869

chr14	59825174	59825264
chr14	59828012	59828509
chr14	59830233	59830932
chr14	59873982	59874149
chr14	59905047	59905202
chr14	59911234	59911341
chr14	59931162	59932136
chr14	59949439	59949881
chr14	59950054	59951659
chr14	59948668	59949136
chr14	60097247	60097538
chr14	60131862	60132003
chr14	60217421	60217669
chr14	60528135	60528150
chr14	60557926	60559295
chr14	60561075	60561437
chr14	60573193	60573392
chr14	60582850	60583149
chr14	60618394	60618540
chr14	60618744	60618932
chr14	60628027	60628133
chr14	60628458	60629009
chr14	60630540	60630784
chr14	60631311	60632415
chr14	60646163	60646676
chr14	60650336	60650444
chr14	60650589	60650749
chr14	60714115	60714412
chr14	60714591	60714735
chr14	60714896	60715757
chr14	60716088	60717026
chr14	60733130	60733139
chr14	60733311	60733474
chr14	60794267	60794902
chr14	60798013	60798545
chr14	60951998	60952749
chr14	60952997	60953046
chr14	61092216	61092714
chr14	61114186	61115463
chr14	61115682	61116171
chr14	61116309	61116508
chr14	61120060	61120259
chr14	61122670	61122869
chr14	61123245	61124529
chr14	61153719	61153911
chr14	61188057	61191039

chr14	61191261	61191464
chr14	61201484	61201859
chr14	61203222	61203390
chr14	61203499	61203606
chr14	61224380	61224672
chr14	61227334	61227449
chr14	61227171	61227270
chr14	61227611	61227738
chr14	61242027	61242327
chr14	61272722	61272873
chr14	61447401	61449120
chr14	61491973	61492297
chr14	61544628	61545096
chr14	61568566	61569346
chr14	61569474	61569820
chr14	61570125	61571223
chr14	61611706	61612565
chr14	61641735	61641895
chr14	61644342	61645823
chr14	61645985	61646297
chr14	61648568	61648743
chr14	61650186	61650335
chr14	61654970	61656287
chr14	61663745	61663866
chr14	61736539	61736744
chr14	61746165	61748522
chr14	61753130	61753349
chr14	61787896	61788578
chr14	61788700	61790141
chr14	61793789	61794066
chr14	61806262	61806327
chr14	61806534	61806639
chr14	61812971	61813335
chr14	61822503	61822656
chr14	61822830	61823096
chr14	61854927	61855380
chr14	61855525	61856341
chr14	61866137	61866250
chr14	61886554	61886647
chr14	61892795	61893116
chr14	61927937	61928456
chr14	61929418	61929871
chr14	61932830	61933386
chr14	61933802	61934162
chr14	61936901	61939342
chr14	61939878	61940071

chr14	61942078	61942147
chr14	61942259	61942373
chr14	61942985	61943184
chr14	61943410	61943974
chr14	61944849	61945346
chr14	61946781	61946985
chr14	61949330	61949498
chr14	61950483	61950605
chr14	61959408	61959439
chr14	61959938	61960318
chr14	61969112	61969822
chr14	61969890	61970359
chr14	61977395	61977416
chr14	61977607	61977730
chr14	61979274	61979665
chr14	61985508	61985799
chr14	61990495	61990856
chr14	61991243	61991591
chr14	61991705	61993034
chr14	61993109	61993978
chr14	61994227	61994905
chr14	61995029	61995916
chr14	61997696	61998034
chr14	61998229	61998864
chr14	62000317	62000516
chr14	62002222	62002357
chr14	62003091	62003418
chr14	62004078	62005097
chr14	62012475	62012526
chr14	62014079	62014542
chr14	62019624	62020199
chr14	62020424	62021070
chr14	62021795	62022242
chr14	62027049	62027445
chr14	62027743	62028743
chr14	62030768	62031842
chr14	62032648	62033142
chr14	62028855	62030330
chr14	62035161	62035566
chr14	62035728	62036574
chr14	62036769	62036954
chr14	62040800	62041465
chr14	62052429	62053000
chr14	62053250	62054119
chr14	62068232	62068431
chr14	62083098	62083356

chr14	62087291	62087490
chr14	62087618	62087630
chr14	62109058	62109081
chr14	62113369	62113468
chr14	62113583	62113748
chr14	62120249	62120617
chr14	62129170	62129625
chr14	62161641	62161926
chr14	62162271	62163289
chr14	62175698	62175954
chr14	62177529	62177779
chr14	62180699	62180798
chr14	62182577	62182783
chr14	62184919	62185125
chr14	62187634	62187932
chr14	62210976	62211175
chr14	62213112	62213405
chr14	62215998	62216345
chr14	62216528	62216917
chr14	62217494	62218228
chr14	62219406	62219805
chr14	62220261	62220485
chr14	62221856	62222374
chr14	62224261	62224459
chr14	62228518	62228938
chr14	62229090	62230098
chr14	62232033	62232276
chr14	62279548	62280027
chr14	62391713	62391912
chr14	62393698	62394704
chr14	62401036	62401213
chr14	62401445	62401559
chr14	62431662	62432333
chr14	62469081	62469314
chr14	62469455	62469654
chr14	62676006	62676023
chr14	62676315	62676445
chr14	62680198	62680458
chr14	62895406	62896037
chr14	63141566	63141834
chr14	63158408	63159346
chr14	63160886	63161127
chr14	63506774	63506951
chr14	63518273	63518452
chr14	63641690	63641748
chr14	63646012	63646131

chr14	63842613	63842901
chr14	63863791	63863793
chr14	63864009	63864145
chr14	63904646	63905291
chr14	63906421	63906493
chr14	63906625	63906925
chr14	63915544	63915977
chr14	63935017	63935040
chr14	63937971	63938337
chr14	63948976	63949276
chr14	63950695	63951220
chr14	63959147	63959783
chr14	63959862	63960074
chr14	63961406	63961696
chr14	64006624	64006846
chr14	64006918	64006968
chr14	64008800	64010122
chr14	64010380	64010804
chr14	64011391	64011542
chr14	64018523	64018573
chr14	64030687	64030802
chr14	64098496	64098695
chr14	64099693	64100001
chr14	64107973	64109367
chr14	64111432	64111559
chr14	64111669	64111861
chr14	64120392	64120584
chr14	64140866	64140963
chr14	64173978	64174027
chr14	64174220	64174320
chr14	64180528	64180836
chr14	64193846	64194757
chr14	64194902	64195101
chr14	64204831	64205065
chr14	64219321	64219575
chr14	64219800	64219984
chr14	64236100	64236280
chr14	64240214	64240625
chr14	64243740	64243985
chr14	64265805	64266080
chr14	64304393	64304565
chr14	64305216	64305518
chr14	64319484	64320812
chr14	64330629	64330859
chr14	64331036	64331439
chr14	64336584	64336865

chr14	64338913	64339159
chr14	64357006	64357266
chr14	64360313	64360429
chr14	64389634	64390308
chr14	64404855	64405282
chr14	64405644	64405683
chr14	64406102	64406203
chr14	64459277	64459605
chr14	64459833	64459989
chr14	64520421	64520826
chr14	64526813	64527887
chr14	64529490	64529772
chr14	64541647	64541813
chr14	64541960	64542082
chr14	64549545	64550000
chr14	64562462	64562641
chr14	64574741	64575175
chr14	64577620	64577955
chr14	64579512	64580453
chr14	64580750	64581337
chr14	64584377	64585491
chr14	64590817	64591033
chr14	64616784	64616959
chr14	64645612	64645811
chr14	64658769	64658893
chr14	64661169	64661368
chr14	64760225	64760390
chr14	64760737	64761533
chr14	64774020	64774094
chr14	64804447	64805426
chr14	64805667	64806141
chr14	64818024	64818206
chr14	64832504	64832747
chr14	64853826	64854619
chr14	64855009	64855579
chr14	64855857	64856205
chr14	64875241	64875491
chr14	64876856	64877312
chr14	64885649	64885989
chr14	64887873	64888037
chr14	64888156	64888342
chr14	64912227	64912465
chr14	64931790	64932853
chr14	64968237	64968371
chr14	64968510	64968966
chr14	64969255	64972577

chr14	64973282	64973677
chr14	64974099	64974476
chr14	64977562	64978195
chr14	64987478	64987805
chr14	65003246	65003568
chr14	65005783	65007030
chr14	65007521	65007880
chr14	65008147	65008346
chr14	65016006	65016062
chr14	65016273	65016624
chr14	65036071	65036681
chr14	65044784	65044964
chr14	65070893	65071118
chr14	65071820	65072152
chr14	65086053	65086466
chr14	65087860	65087889
chr14	65096176	65096342
chr14	65098083	65098160
chr14	65099896	65100019
chr14	65100263	65100418
chr14	65103751	65103831
chr14	65107005	65107290
chr14	65121257	65121328
chr14	65123649	65123734
chr14	65133111	65133643
chr14	65133796	65133803
chr14	65138371	65138623
chr14	65141329	65141458
chr14	65142957	65143068
chr14	65145199	65145330
chr14	65145689	65145777
chr14	65146374	65146377
chr14	65146503	65147804
chr14	65148365	65149228
chr14	65158898	65158968
chr14	65168548	65168747
chr14	65169416	65173069
chr14	65173408	65174158
chr14	65174335	65176133
chr14	65181220	65181369
chr14	65181508	65181549
chr14	65181900	65184701
chr14	65184956	65185042
chr14	65185175	65185656
chr14	65186444	65187399
chr14	65188144	65188562

chr14	65188784	65188882
chr14	65188971	65189713
chr14	65189887	65190898
chr14	65191818	65192206
chr14	65193286	65193512
chr14	65193624	65193945
chr14	65202077	65202192
chr14	65221838	65221953
chr14	65222173	65223245
chr14	65223732	65226146
chr14	65226447	65226659
chr14	65227869	65228137
chr14	65228578	65229628
chr14	65231102	65231635
chr14	65232504	65233453
chr14	65255804	65256051
chr14	65257874	65258028
chr14	65273066	65273079
chr14	65273547	65273709
chr14	65305534	65305801
chr14	65346355	65346889
chr14	65363121	65363338
chr14	65371864	65371982
chr14	65372335	65372382
chr14	65377606	65377724
chr14	65380812	65380907
chr14	65381193	65381711
chr14	65408013	65408122
chr14	65409165	65409548
chr14	65409727	65409853
chr14	65410165	65410325
chr14	65438516	65438855
chr14	65438423	65438452
chr14	65439007	65439594
chr14	65439880	65440079
chr14	65450295	65450598
chr14	65453559	65454377
chr14	65472413	65472646
chr14	65493348	65493547
chr14	65514351	65515023
chr14	65568346	65569203
chr14	65569565	65570202
chr14	65570415	65570467
chr14	65598365	65598506
chr14	65646466	65646604
chr14	65646709	65647612

chr14	65678072	65678322
chr14	65678446	65678621
chr14	65689083	65689097
chr14	65704758	65705157
chr14	65705460	65705656
chr14	65721563	65721737
chr14	65721863	65722108
chr14	65722206	65722445
chr14	65722619	65723046
chr14	65723783	65723955
chr14	65820144	65820327
chr14	65821302	65821501
chr14	65822538	65823133
chr14	65823526	65823684
chr14	65823830	65823902
chr14	65843213	65843275
chr14	65878264	65878349
chr14	65878586	65879241
chr14	65879377	65880619
chr14	65888386	65888483
chr14	65929156	65929555
chr14	65950402	65950712
chr14	66125138	66125181
chr14	66584356	66584442
chr14	66584572	66584669
chr14	66845773	66845843
chr14	66962471	66962600
chr14	66970256	66970601
chr14	66973990	66975789
chr14	66976103	66976327
chr14	66980911	66981304
chr14	67213036	67213112
chr14	67326266	67326356
chr14	67398787	67399442
chr14	67399677	67399687
chr14	67441359	67441683
chr14	67585179	67585289
chr14	67633368	67633447
chr14	67660750	67660768
chr14	67695089	67695288
chr14	67707036	67708864
chr14	67713423	67713695
chr14	67714382	67714754
chr14	67778605	67778607
chr14	67810472	67810962
chr14	67824770	67825473

chr14	67825964	67826491
chr14	67826666	67827637
chr14	67852148	67852162
chr14	67852369	67852513
chr14	67853895	67854269
chr14	67856980	67856982
chr14	67874517	67874689
chr14	67876179	67876180
chr14	67876368	67877681
chr14	67880009	67880593
chr14	67877924	67879929
chr14	67882842	67883053
chr14	67884340	67885002
chr14	67885223	67886509
chr14	67893266	67893555
chr14	67894082	67894338
chr14	67896167	67896489
chr14	67906810	67906823
chr14	67907126	67907474
chr14	67910962	67911163
chr14	67911343	67912057
chr14	67927173	67927382
chr14	67928318	67928523
chr14	67928974	67929203
chr14	67930683	67930882
chr14	67942989	67943179
chr14	67943641	67943754
chr14	67945155	67945167
chr14	67945969	67946112
chr14	67946336	67946447
chr14	67949941	67949952
chr14	67974345	67974662
chr14	67980938	67981290
chr14	67981425	67982552
chr14	67999533	68000712
chr14	68023641	68023997
chr14	68024817	68024890
chr14	68026849	68026864
chr14	68030232	68030854
chr14	68030968	68031083
chr14	68031237	68031698
chr14	68032990	68033439
chr14	68033517	68033810
chr14	68066350	68067452
chr14	68069789	68070521
chr14	68086037	68086053

chr14	68086817	68087293
chr14	68087419	68087584
chr14	68089368	68089774
chr14	68089903	68090403
chr14	68098372	68098392
chr14	68102180	68102540
chr14	68102970	68103024
chr14	68103233	68103451
chr14	68117367	68117489
chr14	68117732	68117839
chr14	68130277	68130807
chr14	68131422	68131661
chr14	68132639	68132757
chr14	68132864	68133030
chr14	68136589	68136918
chr14	68140757	68142221
chr14	68142603	68142798
chr14	68146872	68147223
chr14	68160421	68160824
chr14	68161361	68162055
chr14	68162248	68162775
chr14	68167207	68167737
chr14	68171529	68171565
chr14	68282827	68283198
chr14	68283457	68283684
chr14	68284303	68284699
chr14	68285846	68286045
chr14	68286146	68286657
chr14	68286761	68287667
chr14	68400915	68401124
chr14	68431132	68431665
chr14	68604319	68604549
chr14	68604847	68604942
chr14	68606842	68607471
chr14	68658796	68658812
chr14	68677711	68677832
chr14	68699221	68699315
chr14	68699540	68699735
chr14	68701264	68701423
chr14	68745288	68745378
chr14	68913649	68913801
chr14	68925549	68925672
chr14	68925860	68926122
chr14	68927077	68927351
chr14	68931424	68931921
chr14	68932015	68932231

chr14	68933152	68933441
chr14	68933677	68933780
chr14	68935351	68935806
chr14	68942945	68943748
chr14	68951130	68951217
chr14	68972609	68973195
chr14	68979331	68980190
chr14	68981222	68981389
chr14	68981531	68981616
chr14	68987305	68988251
chr14	68989088	68989498
chr14	68993749	68993792
chr14	69002096	69002207
chr14	69002398	69002621
chr14	69012973	69013172
chr14	69013539	69016282
chr14	69016665	69017535
chr14	69017794	69018110
chr14	69019369	69019699
chr14	69021521	69022081
chr14	69027541	69027697
chr14	69040697	69040708
chr14	69044338	69045016
chr14	69046034	69046233
chr14	69047373	69047626
chr14	69094876	69095345
chr14	69141732	69142065
chr14	69143053	69143778
chr14	69143956	69144415
chr14	69144611	69144802
chr14	69144915	69145137
chr14	69148370	69148422
chr14	69148531	69148681
chr14	69149270	69149705
chr14	69149880	69150103
chr14	69150262	69150367
chr14	69150773	69151022
chr14	69151362	69151451
chr14	69151862	69152456
chr14	69152731	69153053
chr14	69153194	69153254
chr14	69154201	69154469
chr14	69154601	69154944
chr14	69155783	69156073
chr14	69159525	69159724
chr14	69161459	69163048

chr14	69168646	69168715
chr14	69168953	69169152
chr14	69169479	69169902
chr14	69170109	69170233
chr14	69170548	69170862
chr14	69171252	69172045
chr14	69172301	69172768
chr14	69172994	69173188
chr14	69246446	69246695
chr14	69247427	69247684
chr14	69252089	69252418
chr14	69252584	69253875
chr14	69254141	69254468
chr14	69255369	69255541
chr14	69255820	69258874
chr14	69258979	69263286
chr14	69264245	69264384
chr14	69267062	69267120
chr14	69267277	69267355
chr14	69282537	69283606
chr14	69291516	69292020
chr14	69310636	69310777
chr14	69351085	69351328
chr14	69366223	69366440
chr14	69367664	69367836
chr14	69373774	69373802
chr14	69378509	69378794
chr14	69380496	69380535
chr14	69380611	69380753
chr14	69381204	69381428
chr14	69389442	69389641
chr14	69391431	69391684
chr14	69391860	69391868
chr14	69397901	69397978
chr14	69398046	69398239
chr14	69398345	69398605
chr14	69401819	69402167
chr14	69402603	69402849
chr14	69405381	69406379
chr14	69406526	69407744
chr14	69408361	69408639
chr14	69408869	69409482
chr14	69409595	69410476
chr14	69410638	69411756
chr14	69411968	69414925
chr14	69415330	69417528

chr14	69417607	69417842
chr14	69421130	69421500
chr14	69417981	69420401
chr14	69422685	69422705
chr14	69425373	69425694
chr14	69426813	69427026
chr14	69428496	69428843
chr14	69429405	69429767
chr14	69432672	69432883
chr14	69433001	69433302
chr14	69437484	69437787
chr14	69439934	69440233
chr14	69441203	69442036
chr14	69442233	69442454
chr14	69442716	69446070
chr14	69446204	69447609
chr14	69448047	69448561
chr14	69448700	69448805
chr14	69449249	69449448
chr14	69453680	69453709
chr14	69453920	69454033
chr14	69454694	69455527
chr14	69472053	69472196
chr14	69472755	69472954
chr14	69515554	69515790
chr14	69516678	69516806
chr14	69543980	69544179
chr14	69544601	69544944
chr14	69545839	69546519
chr14	69555279	69555762
chr14	69568529	69568596
chr14	69579767	69579890
chr14	69596072	69596577
chr14	69599836	69600287
chr14	69605600	69605799
chr14	69617369	69617737
chr14	69618027	69620554
chr14	69620628	69620708
chr14	69620883	69620994
chr14	69657949	69659101
chr14	69672787	69672833
chr14	69843268	69843530
chr14	69858159	69858415
chr14	69864405	69865951
chr14	69877727	69877926
chr14	69894325	69894580

chr14	69907603	69907625
chr14	69926811	69926962
chr14	70006975	70007211
chr14	70019931	70020027
chr14	70046666	70046768
chr14	70051716	70051815
chr14	70070601	70070704
chr14	70071040	70071129
chr14	70072605	70072886
chr14	70074243	70074479
chr14	70075839	70075893
chr14	70077071	70077253
chr14	70077444	70077891
chr14	70078429	70079855
chr14	70082258	70082415
chr14	70082545	70082621
chr14	70110447	70110712
chr14	70117351	70117624
chr14	70117749	70118117
chr14	70119221	70119250
chr14	70120793	70120946
chr14	70121092	70121253
chr14	70123633	70124005
chr14	70124513	70124699
chr14	70124947	70125202
chr14	70125371	70125814
chr14	70129635	70129781
chr14	70149352	70149585
chr14	70151176	70152865
chr14	70161300	70161499
chr14	70177203	70177428
chr14	70178925	70178999
chr14	70179178	70180628
chr14	70182488	70184135
chr14	70184690	70185033
chr14	70185354	70185729
chr14	70185797	70186808
chr14	70190172	70190404
chr14	70193268	70193804
chr14	70197926	70198002
chr14	70199344	70199481
chr14	70207706	70207833
chr14	70233437	70234845
chr14	70239135	70239554
chr14	70343480	70343485
chr14	70346075	70346805

chr14	70394775	70394974
chr14	70447046	70447334
chr14	70447647	70448103
chr14	70452252	70452628
chr14	70462912	70463311
chr14	70464482	70464670
chr14	70464835	70465203
chr14	70465336	70465548
chr14	70465790	70466537
chr14	70469605	70469804
chr14	70475126	70475398
chr14	70476542	70477241
chr14	70477481	70478065
chr14	70478169	70478449
chr14	70480292	70480355
chr14	70494478	70494595
chr14	70499089	70499091
chr14	70654974	70655442
chr14	70721228	70721456
chr14	70825839	70826372
chr14	70826503	70826788
chr14	70883233	70883794
chr14	71067202	71067451
chr14	71067559	71067984
chr14	71068334	71068354
chr14	71089048	71089196
chr14	71108416	71109283
chr14	71130989	71131226
chr14	71132110	71132260
chr14	71132365	71132775
chr14	71132892	71133255
chr14	71155427	71155503
chr14	71155780	71155812
chr14	71164649	71164712
chr14	71165009	71165132
chr14	71176733	71176856
chr14	71177150	71177161
chr14	71180740	71181056
chr14	71185131	71186047
chr14	71221550	71221731
chr14	71221925	71222189
chr14	71222364	71222756
chr14	71237105	71237330
chr14	71237536	71238157
chr14	71250757	71250776
chr14	71252680	71252720

chr14	71252973	71253258
chr14	71255982	71255988
chr14	71274677	71274718
chr14	71275058	71276401
chr14	71276506	71277922
chr14	71278961	71279025
chr14	71284322	71284441
chr14	71285251	71285481
chr14	71288552	71289671
chr14	71355270	71355608
chr14	71355736	71355947
chr14	71358551	71358888
chr14	71373629	71375495
chr14	71375680	71375789
chr14	71388411	71388490
chr14	71388704	71388748
chr14	71472111	71472193
chr14	71472608	71472629
chr14	71543418	71543543
chr14	71584855	71585206
chr14	71585836	71586158
chr14	71633396	71633606
chr14	71642182	71642254
chr14	71757617	71757916
chr14	71786601	71786838
chr14	71787083	71788264
chr14	71788360	71789133
chr14	71789391	71789751
chr14	71791699	71791781
chr14	71821480	71822882
chr14	71828073	71828386
chr14	71838065	71839414
chr14	71839523	71839769
chr14	71839898	71840202
chr14	71844241	71844474
chr14	71846949	71847355
chr14	71867592	71867927
chr14	71869117	71869502
chr14	71881388	71881659
chr14	71882070	71882172
chr14	71883111	71883489
chr14	71906680	71906875
chr14	71918120	71918476
chr14	71926755	71926805
chr14	71945395	71945621
chr14	72083853	72083917

chr14	72192508	72192678
chr14	72220286	72220492
chr14	72398923	72399490
chr14	73359241	73359806
chr14	73392285	73392876
chr14	73393558	73394136
chr14	73427487	73427600
chr14	73427696	73428338
chr14	73428617	73430393
chr14	73431748	73431752
chr14	73432332	73432368
chr14	73432466	73432744
chr14	73438315	73438414
chr14	73438846	73439081
chr14	73439338	73439731
chr14	73469077	73469592
chr14	73476973	73477172
chr14	73482792	73483123
chr14	73493064	73493800
chr14	73494197	73494407
chr14	73504072	73504188
chr14	73510600	73510620
chr14	73524781	73525088
chr14	73525192	73526297
chr14	73529931	73530113
chr14	73531804	73532003
chr14	73564739	73565111
chr14	73602687	73602969
chr14	73603139	73604099
chr14	73604288	73604594
chr14	73646163	73646456
chr14	73646730	73647391
chr14	73655088	73655121
chr14	73655292	73655612
chr14	73704210	73705037
chr14	73712442	73712641
chr14	73713346	73713425
chr14	73712864	73713222
chr14	73714705	73715922
chr14	73716072	73716088
chr14	73778057	73778415
chr14	73881501	73881763
chr14	73886001	73886193
chr14	73889944	73890219
chr14	73900559	73900812
chr14	73918761	73918888

chr14	73919796	73919919
chr14	73924068	73925303
chr14	73925443	73925747
chr14	73930010	73930266
chr14	73930548	73930662
chr14	73937473	73937672
chr14	73937796	73937901
chr14	73937997	73938189
chr14	73938765	73938964
chr14	73939334	73939395
chr14	73940401	73940564
chr14	73941445	73941705
chr14	73944367	73945190
chr14	73956757	73956780
chr14	73957212	73957540
chr14	73957662	73958361
chr14	73959452	73959483
chr14	73975206	73975444
chr14	74034202	74034529
chr14	74034778	74037261
chr14	74037596	74037695
chr14	74040488	74040879
chr14	74043095	74043566
chr14	74043904	74044003
chr14	74058314	74058359
chr14	74058617	74059442
chr14	74078967	74080122
chr14	74098027	74098142
chr14	74099927	74099998
chr14	74100338	74101349
chr14	74106684	74106738
chr14	74111132	74112335
chr14	74129095	74129248
chr14	74129357	74129605
chr14	74179128	74179838
chr14	74180062	74181209
chr14	74181490	74181649
chr14	74190141	74190340
chr14	74190592	74190663
chr14	74191271	74192101
chr14	74192398	74192597
chr14	74193014	74193249
chr14	74193585	74193868
chr14	74194359	74194501
chr14	74194736	74195962
chr14	74196597	74196916

chr14	74197374	74197391
chr14	74197461	74197700
chr14	74203652	74203896
chr14	74206302	74206661
chr14	74206849	74208356
chr14	74208523	74209797
chr14	74209880	74210487
chr14	74210602	74211589
chr14	74211941	74212294
chr14	74212527	74212735
chr14	74213140	74213395
chr14	74213664	74213970
chr14	74217913	74217942
chr14	74218640	74218813
chr14	74219397	74220009
chr14	74220457	74221046
chr14	74224183	74225133
chr14	74221170	74224083
chr14	74225254	74227782
chr14	74228228	74228525
chr14	74235432	74235454
chr14	74236213	74236427
chr14	74236089	74236121
chr14	74237022	74237080
chr14	74237273	74237365
chr14	74238076	74238125
chr14	74238480	74239077
chr14	74240838	74241321
chr14	74249166	74249268
chr14	74252494	74254849
chr14	74252147	74252387
chr14	74256505	74257258
chr14	74257607	74257784
chr14	74258556	74258620
chr14	74261530	74261737
chr14	74262157	74262423
chr14	74269151	74269986
chr14	74271471	74271670
chr14	74272873	74273645
chr14	74286874	74286920
chr14	74293920	74293973
chr14	74296836	74297157
chr14	74305202	74305431
chr14	74315537	74315698
chr14	74315843	74316065
chr14	74318237	74318413

chr14	74317634	74318150
chr14	74318549	74319768
chr14	74323337	74323628
chr14	74334856	74334955
chr14	74350116	74350178
chr14	74353038	74353263
chr14	74353417	74354192
chr14	74355084	74355451
chr14	74355726	74356103
chr14	74381705	74381821
chr14	74384106	74384305
chr14	74398488	74398560
chr14	74409680	74409771
chr14	74416373	74416759
chr14	74416945	74417526
chr14	74417642	74417905
chr14	74456637	74456674
chr14	74460882	74460918
chr14	74473843	74474179
chr14	74484985	74486469
chr14	74503631	74503774
chr14	74513493	74513821
chr14	74514078	74514392
chr14	74528509	74529241
chr14	74529548	74529594
chr14	74529919	74530001
chr14	74550557	74551162
chr14	74551412	74552364
chr14	74642621	74642665
chr14	74718197	74718451
chr14	74737064	74737322
chr14	74741678	74741996
chr14	74742497	74742787
chr14	74743067	74743114
chr14	74767954	74769658
chr14	74769861	74770194
chr14	74783103	74783272
chr14	74955623	74955687
chr14	74958551	74959225
chr14	74959456	74960045
chr14	74960500	74961084
chr14	74998163	74998575
chr14	75016373	75016727
chr14	75026540	75026554
chr14	75030353	75030464
chr14	75032802	75035347

chr14	75035580	75036265
chr14	75036344	75036543
chr14	75036616	75037814
chr14	75038272	75041200
chr14	75041312	75041792
chr14	75043300	75043528
chr14	75044025	75044337
chr14	75045317	75045611
chr14	75046027	75047489
chr14	75047610	75047925
chr14	75049676	75049904
chr14	75054872	75054916
chr14	75075585	75075587
chr14	75076349	75076571
chr14	75077693	75079393
chr14	75079540	75079752
chr14	75082174	75082365
chr14	75084234	75084453
chr14	75084595	75084751
chr14	75084902	75085431
chr14	75086619	75087325
chr14	75087990	75088278
chr14	75089357	75089750
chr14	75090004	75090048
chr14	75096179	75096491
chr14	75099353	75099356
chr14	75114388	75114876
chr14	75115203	75115602
chr14	75116013	75116297
chr14	75116722	75116844
chr14	75119712	75119757
chr14	75122222	75122310
chr14	75122500	75123067
chr14	75123170	75123402
chr14	75124196	75124419
chr14	75125530	75125731
chr14	75126313	75126512
chr14	75127000	75127131
chr14	75165339	75165772
chr14	75165933	75166221
chr14	75179410	75180058
chr14	75204678	75204938
chr14	75229658	75229791
chr14	75229993	75231310
chr14	75315534	75315579
chr14	75315797	75315974

chr14	75317288	75317360
chr14	75345052	75345098
chr14	75348092	75348381
chr14	75348585	75349133
chr14	75349462	75350106
chr14	75352856	75353312
chr14	75355023	75355350
chr14	75365301	75365427
chr14	75383118	75383305
chr14	75407037	75407236
chr14	75412758	75413678
chr14	75419150	75419761
chr14	75421340	75422685
chr14	75426581	75427045
chr14	75431774	75431890
chr14	75432196	75432395
chr14	75432927	75433033
chr14	75439930	75440905
chr14	75440970	75440991
chr14	75446301	75446500
chr14	75454772	75454856
chr14	75456269	75456487
chr14	75457531	75457727
chr14	75459204	75459258
chr14	75469027	75469155
chr14	75469681	75470060
chr14	75471991	75472140
chr14	75511030	75511123
chr14	75511591	75511718
chr14	75517671	75518382
chr14	75529996	75530931
chr14	75534564	75534878
chr14	75535747	75536526
chr14	75555497	75555674
chr14	75585780	75586063
chr14	75590673	75591141
chr14	75592530	75592693
chr14	75593081	75594612
chr14	75595126	75595350
chr14	75599430	75599469
chr14	75601424	75601765
chr14	75615766	75615945
chr14	75624315	75624521
chr14	75625959	75626268
chr14	75633748	75633990
chr14	75640725	75640982

chr14	75642596	75643944
chr14	75646245	75646594
chr14	75648022	75648241
chr14	75649553	75649772
chr14	75665469	75665929
chr14	75666048	75666291
chr14	75666647	75668092
chr14	75670916	75671185
chr14	75671904	75672016
chr14	75676298	75676574
chr14	75680282	75680306
chr14	75684381	75684569
chr14	75688428	75689484
chr14	75690537	75690566
chr14	75690693	75690808
chr14	75691956	75692245
chr14	75696784	75696879
chr14	75697039	75697637
chr14	75698185	75698384
chr14	75699748	75699924
chr14	75700029	75700079
chr14	75700887	75701704
chr14	75702724	75703265
chr14	75709068	75709875
chr14	75711957	75712119
chr14	75717173	75717413
chr14	75718356	75718553
chr14	75718756	75719357
chr14	75719593	75719661
chr14	75720917	75721802
chr14	75722609	75722816
chr14	75723162	75723491
chr14	75723876	75724182
chr14	75724466	75724578
chr14	75724828	75725168
chr14	75725271	75725781
chr14	75725943	75726554
chr14	75727733	75728043
chr14	75732180	75732431
chr14	75734896	75735082
chr14	75741529	75741613
chr14	75741828	75742187
chr14	75742518	75742624
chr14	75743462	75744595
chr14	75743045	75743330
chr14	75744712	75745053

chr14	75745222	75745802
chr14	75745999	75746198
chr14	75749343	75749552
chr14	75750418	75751255
chr14	75760481	75762931
chr14	75763151	75763914
chr14	75765228	75766229
chr14	75768444	75769024
chr14	75769310	75769612
chr14	75770158	75770309
chr14	75770602	75770647
chr14	75772037	75772348
chr14	75866838	75867037
chr14	75867472	75867536
chr14	75867663	75868066
chr14	75888960	75889312
chr14	75893911	75894655
chr14	75895018	75896095
chr14	75916844	75917041
chr14	75917203	75917763
chr14	75919059	75919861
chr14	75920155	75920547
chr14	75954128	75954516
chr14	75955356	75955545
chr14	75981338	75981766
chr14	76002165	76002922
chr14	76008091	76008297
chr14	76020699	76020843
chr14	76021229	76021313
chr14	76021986	76022050
chr14	76022163	76022267
chr14	76025689	76026362
chr14	76026562	76026717
chr14	76027165	76027360
chr14	76027601	76028027
chr14	76044234	76044556
chr14	76045000	76045276
chr14	76045434	76045751
chr14	76115172	76115205
chr14	76126236	76126711
chr14	76126996	76127250
chr14	76127519	76128130
chr14	76175831	76175921
chr14	76177661	76177724
chr14	76177968	76178225
chr14	76297715	76298156

chr14	76298629	76298781
chr14	76334194	76334285
chr14	76334608	76334613
chr14	76345336	76345778
chr14	76408051	76408344
chr14	76447649	76449279
chr14	76451886	76452757
chr14	76617938	76617961
chr14	76618096	76619127
chr14	76637835	76638047
chr14	76667384	76667583
chr14	76669965	76670413
chr14	76733622	76734571
chr14	77016644	77017444
chr14	77041877	77041953
chr14	77227715	77228342
chr14	77260788	77261017
chr14	77277190	77277409
chr14	77277528	77277734
chr14	77278490	77279289
chr14	77279437	77279565
chr14	77330890	77331097
chr14	77335673	77335754
chr14	77342574	77342911
chr14	77370962	77371161
chr14	77371619	77371723
chr14	77372799	77373226
chr14	77373417	77373567
chr14	77379391	77380113
chr14	77380761	77381730
chr14	77382306	77382651
chr14	77383061	77383904
chr14	77384038	77385123
chr14	77385324	77386828
chr14	77395573	77396283
chr14	77396608	77397077
chr14	77397444	77397652
chr14	77403194	77403431
chr14	77403609	77403987
chr14	77404110	77404698
chr14	77404911	77405187
chr14	77405292	77405377
chr14	77405890	77406117
chr14	77410224	77410679
chr14	77412840	77412972
chr14	77413157	77414272

chr14	77414561	77414743
chr14	77415134	77415243
chr14	77416099	77416424
chr14	77417192	77417573
chr14	77417731	77418443
chr14	77418758	77420499
chr14	77420565	77424257
chr14	77424490	77427175
chr14	77427299	77428717
chr14	77428873	77429305
chr14	77440642	77440756
chr14	77464184	77464309
chr14	77469976	77470039
chr14	77470859	77471900
chr14	77472015	77472586
chr14	77473960	77474329
chr14	77474441	77474861
chr14	77484584	77484754
chr14	77485475	77485616
chr14	77486036	77486367
chr14	77486869	77487338
chr14	77489301	77491064
chr14	77491590	77491849
chr14	77491936	77492928
chr14	77493055	77495114
chr14	77495264	77496361
chr14	77496655	77496910
chr14	77497161	77497501
chr14	77498017	77499627
chr14	77499739	77501053
chr14	77501685	77501815
chr14	77502193	77502380
chr14	77504212	77504425
chr14	77504604	77504927
chr14	77506184	77506709
chr14	77507012	77507803
chr14	77508524	77508932
chr14	77509594	77510751
chr14	77511072	77511140
chr14	77511362	77511528
chr14	77511815	77512131
chr14	77512252	77512593
chr14	77517626	77517786
chr14	77518634	77518889
chr14	77518997	77519322
chr14	77531989	77532071

chr14	77532372	77532611
chr14	77534501	77534701
chr14	77537316	77537438
chr14	77537841	77538200
chr14	77538550	77539176
chr14	77541395	77541453
chr14	77542933	77543104
chr14	77543537	77543784
chr14	77554181	77554380
chr14	77554578	77554642
chr14	77554896	77555051
chr14	77555923	77556065
chr14	77563262	77564300
chr14	77564569	77565643
chr14	77590839	77591501
chr14	77645814	77645843
chr14	77648165	77648379
chr14	77648482	77648793
chr14	77736126	77736329
chr14	77769591	77769691
chr14	77769837	77770148
chr14	77770293	77770442
chr14	77776334	77776546
chr14	77780275	77780472
chr14	77780717	77780937
chr14	77781947	77782380
chr14	77782606	77782688
chr14	77782846	77782868
chr14	77786485	77786624
chr14	77786793	77788594
chr14	77842805	77843963
chr14	77909868	77910050
chr14	77923316	77923913
chr14	77924071	77924761
chr14	77924987	77925288
chr14	77925491	77925823
chr14	77964694	77965678
chr14	77965821	77966041
chr14	77974456	77974652
chr14	77974859	77975029
chr14	78022051	78022119
chr14	78057151	78057706
chr14	78057798	78058166
chr14	78059695	78060229
chr14	78066331	78066623
chr14	78081909	78083150

chr14	78108268	78108811
chr14	78169512	78169572
chr14	78173593	78174031
chr14	78174132	78174218
chr14	78174441	78174698
chr14	78226787	78227744
chr14	78227994	78228360
chr14	78254037	78254249
chr14	78257476	78257692
chr14	78259842	78260017
chr14	78262387	78262843
chr14	78264324	78264343
chr14	78265785	78266066
chr14	78266171	78267229
chr14	78268066	78268185
chr14	78268263	78268265
chr14	79478031	79478617
chr14	79511522	79511682
chr14	79745887	79746098
chr14	80048364	80049042
chr14	80050883	80051021
chr14	80051136	80051294
chr14	80892368	80892422
chr14	81034659	81034912
chr14	81036554	81036759
chr14	81053634	81053722
chr14	81087992	81088221
chr14	81088375	81088524
chr14	81104832	81105202
chr14	81397182	81397317
chr14	81397604	81397867
chr14	81407724	81408006
chr14	81408290	81408706
chr14	81421635	81421834
chr14	81635972	81637049
chr14	81637413	81637457
chr14	81638532	81638628
chr14	81678544	81678566
chr14	81685458	81685550
chr14	81685657	81686091
chr14	81686363	81686544
chr14	81686683	81687201
chr14	81687317	81688387
chr14	81734251	81734880
chr14	81735752	81735854
chr14	81769534	81769592

chr14	81769970	81770375
chr14	81770827	81771017
chr14	81778214	81778746
chr14	81779553	81779981
chr14	81780131	81780344
chr14	81780494	81780589
chr14	81780885	81781159
chr14	81786941	81787171
chr14	81789029	81789511
chr14	81790276	81790884
chr14	81793079	81793202
chr14	81795831	81796743
chr14	81796828	81798354
chr14	81800921	81800977
chr14	81830099	81830446
chr14	81833454	81833538
chr14	81838139	81838519
chr14	81851206	81851604
chr14	81856530	81856878
chr14	81857532	81857588
chr14	81859522	81860456
chr14	81862473	81862546
chr14	81862711	81862853
chr14	81866347	81867642
chr14	81867728	81868811
chr14	81873262	81873461
chr14	81875010	81875038
chr14	81875255	81875310
chr14	81880697	81880886
chr14	81898356	81898557
chr14	81898669	81899231
chr14	81899823	81899972
chr14	81901628	81903366
chr14	81919521	81919655
chr14	81926703	81926902
chr14	81930854	81931399
chr14	81934951	81935439
chr14	81999686	81999800
chr14	81999941	82000324
chr14	82000477	82000706
chr14	85291935	85292134
chr14	85324123	85324271
chr14	85442570	85442816
chr14	85542754	85542823
chr14	85544093	85544337
chr14	85554986	85554990

chr14	85699955	85700864
chr14	85700995	85701372
chr14	85798749	85798802
chr14	85798984	85799140
chr14	85880574	85880906
chr14	85881186	85881767
chr14	85881893	85882137
chr14	85883114	85883545
chr14	85887596	85888032
chr14	85888178	85888258
chr14	85890222	85890640
chr14	85903457	85903936
chr14	85906298	85906787
chr14	85935143	85935604
chr14	85938108	85938512
chr14	85983098	85983709
chr14	85994375	85994725
chr14	85994864	85999394
chr14	86001348	86001715
chr14	86001800	86002045
chr14	86003171	86003426
chr14	85999523	86001278
chr14	86022626	86023035
chr14	86032894	86033212
chr14	86032264	86032758
chr14	86033315	86033359
chr14	86039185	86039381
chr14	86039593	86039597
chr14	86057031	86057203
chr14	86073856	86074379
chr14	86401051	86401163
chr14	87599474	87599876
chr14	87600343	87600392
chr14	87699910	87699960
chr14	87801571	87801759
chr14	87896977	87897171
chr14	87916621	87916783
chr14	87958192	87958269
chr14	88336210	88336411
chr14	88340230	88340410
chr14	88341008	88341070
chr14	88341298	88341397
chr14	88344020	88344191
chr14	88344501	88344733
chr14	88458931	88459985
chr14	88851349	88851400

chr14	88851601	88852483
chr14	88934861	88935076
chr14	89017475	89018388
chr14	89018673	89018780
chr14	89020315	89020370
chr14	89020541	89021566
chr14	89028843	89029225
chr14	89029341	89030062
chr14	89079479	89079678
chr14	89079974	89079993
chr14	89082995	89083075
chr14	89083309	89083389
chr14	89183899	89184068
chr14	89258694	89259358
chr14	89290483	89291451
chr14	89345349	89345520
chr14	89345634	89345778
chr14	89454212	89454411
chr14	89466856	89467525
chr14	89493486	89493799
chr14	89506643	89507009
chr14	89608261	89608470
chr14	89715430	89715617
chr14	89715853	89715983
chr14	89769789	89769886
chr14	89770531	89770707
chr14	89774832	89775009
chr14	89806460	89806644
chr14	89834771	89834884
chr14	89839526	89839724
chr14	89839887	89839895
chr14	89869787	89869853
chr14	89874340	89876048
chr14	89876345	89876565
chr14	89879992	89880107
chr14	89880356	89880484
chr14	89880851	89881099
chr14	89882188	89882525
chr14	89882644	89883343
chr14	89883454	89884659
chr14	89891545	89891978
chr14	89892307	89892828
chr14	89894819	89895210
chr14	89895429	89895864
chr14	89895945	89896255
chr14	89910967	89911292

chr14	89934591	89934740
chr14	89946457	89946603
chr14	89973604	89973719
chr14	89998265	89998464
chr14	90006610	90007172
chr14	90007284	90007434
chr14	90013518	90013559
chr14	90085270	90085840
chr14	90168152	90168698
chr14	90203562	90203574
chr14	90267289	90268079
chr14	90284497	90284507
chr14	90284692	90284853
chr14	90322414	90322519
chr14	90333086	90333238
chr14	90375634	90375800
chr14	90378926	90379136
chr14	90420634	90421201
chr14	90421326	90422082
chr14	90422275	90423341
chr14	90527022	90527247
chr14	90575962	90576026
chr14	90722651	90723109
chr14	90747369	90747706
chr14	90748481	90748691
chr14	90774167	90774249
chr14	90797878	90798789
chr14	90811850	90812153
chr14	90822702	90822901
chr14	90848543	90848657
chr14	90848927	90849764
chr14	90849875	90850500
chr14	90862807	90863294
chr14	90863415	90863906
chr14	90864045	90865026
chr14	90865225	90865278
chr14	90865754	90866298
chr14	90875112	90875311
chr14	90885080	90885175
chr14	90885385	90885485
chr14	90885951	90886213
chr14	90898711	90898895
chr14	90907657	90907917
chr14	90909598	90909608
chr14	90909769	90910498
chr14	90910906	90911529

chr14	90920537	90920790
chr14	90921096	90921295
chr14	90924930	90925339
chr14	90928234	90928276
chr14	90975819	90976195
chr14	90982393	90982671
chr14	90982968	90983048
chr14	90992032	90992043
chr14	91087626	91087825
chr14	91090507	91090590
chr14	91090859	91091274
chr14	91091396	91092299
chr14	91095749	91095849
chr14	91099446	91099607
chr14	91111533	91111662
chr14	91225001	91225200
chr14	91225274	91225412
chr14	91261975	91262119
chr14	91263752	91263912
chr14	91264760	91264970
chr14	91267091	91267290
chr14	91272943	91273142
chr14	91281976	91282441
chr14	91282930	91283445
chr14	91293758	91293764
chr14	91294077	91294306
chr14	91294726	91294848
chr14	91305774	91305903
chr14	91307397	91307596
chr14	91315587	91316064
chr14	91316865	91317715
chr14	91337289	91337685
chr14	91337904	91338548
chr14	91350362	91350430
chr14	91390971	91391507
chr14	91475521	91475823
chr14	91487516	91487852
chr14	91488871	91489363
chr14	91525863	91526079
chr14	91526197	91526521
chr14	91526691	91527019
chr14	91527622	91527662
chr14	91528141	91528203
chr14	91553757	91553827
chr14	91554037	91554363
chr14	91579922	91582398

chr14	91585960	91586206
chr14	91586905	91587147
chr14	91590656	91591237
chr14	91591395	91591490
chr14	91592048	91592375
chr14	91593604	91593971
chr14	91594323	91594509
chr14	91601531	91601617
chr14	91604813	91604892
chr14	91606902	91607029
chr14	91615732	91615782
chr14	91625573	91625836
chr14	91643426	91643485
chr14	91664734	91664939
chr14	91669899	91670103
chr14	91709376	91709905
chr14	91714138	91714359
chr14	91714600	91714935
chr14	91718837	91719122
chr14	91719286	91720309
chr14	91722773	91722871
chr14	91723851	91724050
chr14	91724363	91725070
chr14	91728782	91728981
chr14	91730581	91731526
chr14	91733754	91734045
chr14	91750310	91750325
chr14	91750398	91750536
chr14	91783681	91784258
chr14	91785147	91785243
chr14	91812680	91813125
chr14	91816562	91816914
chr14	91826721	91828534
chr14	91829684	91829748
chr14	91849588	91849910
chr14	91857815	91858033
chr14	91858993	91859885
chr14	91861908	91862141
chr14	91862322	91864026
chr14	91882085	91882453
chr14	91882577	91885307
chr14	91891712	91891774
chr14	91959618	91959961
chr14	91960989	91961289
chr14	91964412	91964673
chr14	91975361	91977453

chr14	91982867	91983097
chr14	92001358	92001522
chr14	92001657	92002048
chr14	92040405	92040604
chr14	92080667	92080949
chr14	92112463	92112616
chr14	92116282	92116424
chr14	92121038	92121254
chr14	92121694	92121714
chr14	92203934	92204066
chr14	92221879	92222351
chr14	92302199	92303508
chr14	92336581	92336872
chr14	92337838	92338142
chr14	92339699	92340186
chr14	92376592	92376614
chr14	92391258	92391765
chr14	92412976	92414351
chr14	92415605	92415615
chr14	92446649	92446908
chr14	92463303	92463862
chr14	92505595	92505708
chr14	92505911	92507449
chr14	92569766	92570021
chr14	92571888	92572942
chr14	92573166	92573320
chr14	92587469	92589564
chr14	92594431	92594567
chr14	92601781	92601912
chr14	92716971	92717002
chr14	92789544	92789969
chr14	92938582	92938724
chr14	92939793	92940212
chr14	92961486	92961730
chr14	92961955	92962138
chr14	92969620	92970170
chr14	92970332	92970744
chr14	92977338	92978531
chr14	92979690	92980878
chr14	93002797	93002899
chr14	93007380	93007462
chr14	93013990	93014004
chr14	93029812	93030011
chr14	93031504	93031758
chr14	93031862	93032232
chr14	93063150	93063287

chr14	93063785	93063828
chr14	93077591	93077785
chr14	93109967	93110286
chr14	93110886	93111391
chr14	93112228	93112531
chr14	93112801	93113093
chr14	93114526	93114548
chr14	93116696	93117215
chr14	93120363	93120438
chr14	93125928	93126126
chr14	93140813	93141012
chr14	93153468	93153766
chr14	93174897	93175371
chr14	93189969	93190172
chr14	93191664	93191764
chr14	93192124	93193242
chr14	93195396	93195758
chr14	93196339	93196702
chr14	93213917	93214049
chr14	93214296	93215313
chr14	93250111	93250128
chr14	93252045	93252850
chr14	93259341	93259545
chr14	93259871	93260306
chr14	93260623	93261686
chr14	93346685	93346767
chr14	93391794	93391993
chr14	93392670	93392869
chr14	93393074	93394640
chr14	93396201	93396412
chr14	93397506	93397898
chr14	93412789	93413141
chr14	93413650	93413903
chr14	93414743	93414942
chr14	93422493	93423000
chr14	93430000	93430267
chr14	93438805	93439095
chr14	93439444	93440473
chr14	93441028	93441280
chr14	93452440	93452703
chr14	93468385	93468607
chr14	93492809	93492931
chr14	93498432	93498738
chr14	93499772	93502229
chr14	93504388	93504709
chr14	93505007	93505273

chr14	93502606	93504256
chr14	93505393	93505968
chr14	93506288	93508527
chr14	93509287	93512375
chr14	93512860	93514313
chr14	93514964	93515321
chr14	93515723	93516338
chr14	93517680	93517980
chr14	93519991	93520214
chr14	93521261	93521506
chr14	93523603	93523854
chr14	93524153	93524433
chr14	93524695	93525000
chr14	93525645	93525904
chr14	93526028	93527043
chr14	93527410	93527457
chr14	93527732	93528028
chr14	93528346	93528361
chr14	93528524	93528768
chr14	93539029	93539224
chr14	93539538	93539746
chr14	93540259	93541718
chr14	93550868	93550955
chr14	93553562	93553846
chr14	93553911	93554110
chr14	93551655	93553382
chr14	93554571	93555108
chr14	93557432	93557841
chr14	93559807	93560006
chr14	93560358	93560625
chr14	93561886	93562046
chr14	93564371	93564380
chr14	93566480	93566964
chr14	93567666	93568245
chr14	93568730	93568777
chr14	93569680	93570074
chr14	93570190	93572863
chr14	93573051	93573540
chr14	93573669	93573829
chr14	93574890	93575336
chr14	93577936	93578115
chr14	93578794	93579322
chr14	93579583	93580327
chr14	93580486	93581658
chr14	93581790	93583367
chr14	93585280	93585496

chr14	93596600	93597008
chr14	93597753	93598147
chr14	93598507	93598856
chr14	93599047	93600345
chr14	93600794	93601354
chr14	93603510	93603734
chr14	93603845	93603978
chr14	93604188	93604280
chr14	93605428	93605718
chr14	93605845	93606107
chr14	93616071	93616147
chr14	93616605	93617568
chr14	93619501	93619574
chr14	93617866	93618443
chr14	93619813	93619850
chr14	93626265	93626470
chr14	93626556	93626808
chr14	93642590	93642658
chr14	93644650	93644927
chr14	93650353	93651978
chr14	93652705	93653117
chr14	93666864	93666985
chr14	93667260	93667318
chr14	93672552	93674657
chr14	93677493	93677692
chr14	93684514	93684721
chr14	93685487	93685677
chr14	93701699	93701896
chr14	93706833	93707135
chr14	93725266	93725892
chr14	93729014	93729157
chr14	93767126	93767165
chr14	93768078	93768701
chr14	93771391	93771449
chr14	93773686	93773942
chr14	93784128	93784592
chr14	93785227	93785496
chr14	93792581	93792903
chr14	93796857	93797310
chr14	93797440	93797701
chr14	93797929	93799461
chr14	93799630	93799977
chr14	93963282	93963291
chr14	94254925	94255205
chr14	94404416	94404552
chr14	94404625	94404859

chr14	94408800	94408999
chr14	94410410	94410609
chr14	94426030	94426302
chr14	94472808	94473007
chr14	94478830	94479051
chr14	94489211	94489465
chr14	94492067	94492527
chr14	94492717	94493096
chr14	94528632	94528875
chr14	94528995	94529263
chr14	94531299	94531498
chr14	94532372	94532552
chr14	94546801	94546843
chr14	94547022	94547506
chr14	94547738	94548210
chr14	94576835	94577728
chr14	94577847	94578241
chr14	94578493	94578501
chr14	94579072	94579271
chr14	94595323	94596299
chr14	94596487	94596767
chr14	94611087	94611315
chr14	94612614	94612659
chr14	94637511	94637570
chr14	94639737	94640237
chr14	94640617	94641937
chr14	94643174	94643508
chr14	94679318	94679843
chr14	94778720	94778821
chr14	94778926	94779203
chr14	94779476	94779972
chr14	94786888	94787062
chr14	94801181	94801183
chr14	94801567	94801575
chr14	94837511	94837710
chr14	94839264	94839610
chr14	94839763	94840144
chr14	94840420	94840632
chr14	94840776	94840971
chr14	94842663	94842850
chr14	94856644	94857492
chr14	94857919	94858311
chr14	94859104	94859369
chr14	94867703	94867827
chr14	95016178	95016358
chr14	95064817	95064890

chr14	95064985	95065184
chr14	95065391	95065475
chr14	95065839	95066363
chr14	95066630	95067467
chr14	95067613	95067970
chr14	95068200	95068334
chr14	95068488	95068811
chr14	95069645	95069680
chr14	95076070	95076623
chr14	95076905	95077427
chr14	95077598	95077865
chr14	95076782	95076791
chr14	95573541	95573631
chr14	95606702	95607045
chr14	95622551	95625103
chr14	95664394	95664593
chr14	95715848	95715971
chr14	95719849	95720013
chr14	95721243	95721267
chr14	95721591	95721606
chr14	95721802	95722025
chr14	95722186	95723150
chr14	95725369	95725475
chr14	95729984	95730035
chr14	95731564	95731741
chr14	95731990	95732444
chr14	95757100	95757516
chr14	95757770	95757969
chr14	95784942	95785216
chr14	95785627	95786201
chr14	95786394	95787103
chr14	95787191	95787476
chr14	95787983	95788342
chr14	95788864	95789115
chr14	95789312	95789726
chr14	95790244	95790389
chr14	95793945	95793997
chr14	95795168	95795330
chr14	95796932	95797192
chr14	95833705	95833836
chr14	95946520	95946719
chr14	95949749	95949954
chr14	95973355	95973545
chr14	95974093	95974318
chr14	95982099	95982172
chr14	95982277	95982403

chr14	95982607	95983366
chr14	95985546	95986021
chr14	95986253	95986694
chr14	95986925	95987063
chr14	95989674	95989762
chr14	95989884	95990027
chr14	95990131	95991005
chr14	95991126	95991654
chr14	95993688	95993867
chr14	95994088	95994173
chr14	95996804	95996893
chr14	95998061	95998211
chr14	95999620	96000457
chr14	96000549	96000979
chr14	96001476	96002106
chr14	96002373	96002611
chr14	96013763	96014030
chr14	96025448	96025591
chr14	96025785	96026040
chr14	96505753	96506158
chr14	96552122	96552174
chr14	96552378	96552423
chr14	96564041	96564369
chr14	96566744	96567014
chr14	96567172	96567520
chr14	96570698	96571006
chr14	96571281	96571686
chr14	96670349	96670407
chr14	96670673	96672148
chr14	96672455	96672840
chr14	96679566	96680035
chr14	96682058	96682356
chr14	96682553	96682741
chr14	96683020	96683190
chr14	96684917	96685055
chr14	96685586	96685631
chr14	96689811	96690044
chr14	96690442	96690774
chr14	96690927	96691656
chr14	96691840	96692430
chr14	96692555	96692733
chr14	96712457	96713237
chr14	96713615	96713834
chr14	96714708	96714764
chr14	96715274	96715554
chr14	96715770	96715826

chr14	96719169	96719616
chr14	96719726	96720067
chr14	96721220	96721363
chr14	96722051	96722373
chr14	96722756	96723213
chr14	96727090	96727167
chr14	96731166	96731317
chr14	96731430	96732137
chr14	96732689	96733768
chr14	96733873	96736079
chr14	96736748	96736915
chr14	96737443	96737707
chr14	96738021	96738115
chr14	96763696	96763784
chr14	96828685	96828714
chr14	96828971	96829723
chr14	96829864	96830783
chr14	96856861	96857060
chr14	96857633	96858234
chr14	96858492	96858773
chr14	96890515	96890881
chr14	96890997	96891028
chr14	96963489	96963527
chr14	96967925	96969669
chr14	97019595	97019948
chr14	97228960	97229130
chr14	97244225	97244424
chr14	97249597	97249788
chr14	97263732	97264634
chr14	97287792	97287817
chr14	97288126	97288276
chr14	97290625	97290796
chr14	97290940	97291010
chr14	97573712	97573883
chr14	98238015	98238292
chr14	98444050	98444096
chr14	98444612	98445148
chr14	98475333	98475470
chr14	98536044	98536178
chr14	98536609	98536697
chr14	98543540	98543593
chr14	98543853	98543938
chr14	98652646	98652718
chr14	98652834	98652864
chr14	98960883	98961050
chr14	98961316	98961352

chr14	98963954	98964248
chr14	98964370	98964611
chr14	98967420	98967897
chr14	99370085	99370101
chr14	99370313	99370721
chr14	99371739	99371817
chr14	99372480	99372658
chr14	99373180	99373631
chr14	99374137	99374158
chr14	99374981	99375582
chr14	99375946	99376359
chr14	99416178	99416338
chr14	99419180	99419342
chr14	99419590	99419693
chr14	99419936	99421044
chr14	99423418	99423607
chr14	99423809	99424127
chr14	99424238	99424550
chr14	99502007	99502424
chr14	99502579	99503070
chr14	99503161	99503287
chr14	99505030	99505310
chr14	99530527	99530772
chr14	99531094	99531389
chr14	99532017	99532032
chr14	99533201	99533224
chr14	99566081	99566165
chr14	99599915	99600105
chr14	99621846	99621922
chr14	99684540	99684739
chr14	99685010	99685323
chr14	99685551	99685693
chr14	99690785	99691088
chr14	99706423	99706515
chr14	99710522	99710538
chr14	99710778	99711374
chr14	99711440	99712971
chr14	99716242	99716350
chr14	99724093	99724275
chr14	99732812	99732922
chr14	99735534	99735558
chr14	99735900	99735936
chr14	99736266	99737738
chr14	99738028	99740362
chr14	99740558	99740909
chr14	99799381	99799567

chr14	99845604	99845834
chr14	99882040	99882070
chr14	99887116	99887320
chr14	99946038	99947436
chr14	99947729	99948281
chr14	99950505	99950731
chr14	99970472	99970489
chr14	99985154	99985353
chr14	99990151	99990350
chr14	99995920	99996091
chr14	99995511	99995854
chr14	100003189	100003505
chr14	100003781	100003980
chr14	100004275	100004486
chr14	100004651	100004907
chr14	100031882	100032193
chr14	100039007	100039364
chr14	100040412	100041024
chr14	100041143	100041863
chr14	100044385	100047352
chr14	100042243	100044315
chr14	100048621	100048929
chr14	100049732	100050094
chr14	100058300	100058529
chr14	100061455	100061774
chr14	100064598	100064748
chr14	100067215	100067254
chr14	100067608	100067689
chr14	100068276	100070346
chr14	100070570	100072239
chr14	100077974	100078902
chr14	100079052	100079329
chr14	100079467	100079946
chr14	100080454	100080564
chr14	100081640	100081877
chr14	100086396	100086968
chr14	100087497	100087719
chr14	100087990	100088337
chr14	100089230	100089467
chr14	100089868	100090152
chr14	100094740	100094767
chr14	100095083	100095121
chr14	100111078	100112381
chr14	100120517	100120669
chr14	100131627	100131863
chr14	100149124	100150220

chr14	100150650	100151110
chr14	100196494	100196896
chr14	100210942	100211141
chr14	100211383	100211629
chr14	100218794	100219188
chr14	100257281	100257465
chr14	100414399	100414507
chr14	100415050	100415501
chr14	100416517	100416730
chr14	100420387	100420486
chr14	100420549	100420586
chr14	100423701	100424245
chr14	100437297	100438169
chr14	100438366	100438927
chr14	100439612	100439682
chr14	100447365	100447701
chr14	100448837	100448958
chr14	100462171	100462460
chr14	100463029	100464177
chr14	100485988	100486145
chr14	100491480	100491941
chr14	100494079	100494492
chr14	100495699	100496030
chr14	100496334	100498973
chr14	100519212	100519276
chr14	100525681	100525729
chr14	100526184	100526192
chr14	100534793	100535008
chr14	100538645	100538844
chr14	100539105	100539456
chr14	100540803	100541133
chr14	100542122	100542370
chr14	100542480	100543436
chr14	100550048	100550580
chr14	100559175	100559292
chr14	100562953	100563160
chr14	100565741	100566141
chr14	100572838	100573045
chr14	100573174	100574080
chr14	100574319	100575177
chr14	100575544	100575854
chr14	100616217	100617682
chr14	100632845	100633641
chr14	100633853	100634067
chr14	100646319	100646518
chr14	100657438	100657637

chr14	100658047	100658260
chr14	100658453	100660028
chr14	100660534	100660733
chr14	100661414	100661633
chr14	100662263	100662526
chr14	100664086	100664291
chr14	100664703	100664982
chr14	100665266	100665695
chr14	100669840	100670039
chr14	100670265	100670485
chr14	100670644	100671639
chr14	100672150	100672366
chr14	100675414	100675722
chr14	100679141	100679340
chr14	100679628	100679665
chr14	100679817	100680852
chr14	100681247	100681587
chr14	100687673	100688727
chr14	100692429	100692587
chr14	100704063	100704987
chr14	100705112	100706383
chr14	100706495	100707209
chr14	100710574	100711028
chr14	100711991	100712190
chr14	100712741	100712835
chr14	100714520	100714719
chr14	100725649	100725837
chr14	100727413	100727416
chr14	100738409	100738608
chr14	100751070	100752580
chr14	100756836	100757039
chr14	100771893	100773824
chr14	100776775	100777018
chr14	100784303	100784516
chr14	100785602	100785726
chr14	100786353	100787592
chr14	100807148	100807157
chr14	100807577	100808277
chr14	100808526	100808619
chr14	100817737	100817779
chr14	100841720	100843897
chr14	100844445	100844863
chr14	100864160	100864545
chr14	100864769	100864781
chr14	100873253	100873434
chr14	100873526	100873557

chr14	100876391	100876440
chr14	100885771	100885857
chr14	100905258	100905564
chr14	100905866	100906030
chr14	100906294	100906578
chr14	100906763	100906935
chr14	100907290	100907383
chr14	101012985	101013384
chr14	101033970	101034169
chr14	101052674	101053507
chr14	101112500	101112719
chr14	101121368	101121567
chr14	101137023	101137301
chr14	101155817	101156053
chr14	101157047	101157246
chr14	101158139	101160502
chr14	101213255	101213357
chr14	101213645	101214030
chr14	101228475	101228687
chr14	101942732	101943100
chr14	101947610	101947788
chr14	102153098	102153220
chr14	102171419	102171562
chr14	102172696	102172895
chr14	102173726	102174119
chr14	102188788	102188910
chr14	102191926	102192117
chr14	102197037	102197535
chr14	102197739	102198464
chr14	102199290	102199471
chr14	102201558	102201596
chr14	102226289	102226633
chr14	102227319	102227722
chr14	102228202	102228765
chr14	102228896	102228936
chr14	102248033	102248566
chr14	102258635	102258889
chr14	102275946	102276061
chr14	102276175	102276898
chr14	102347009	102347100
chr14	102409570	102409769
chr14	102413887	102414271
chr14	102414704	102415558
chr14	102416327	102416502
chr14	102425795	102426009
chr14	102426079	102426099

chr14	102428331	102428684
chr14	102429217	102429311
chr14	102430908	102432084
chr14	102432473	102432537
chr14	102437786	102438186
chr14	102438612	102439197
chr14	102439722	102440071
chr14	102444093	102444312
chr14	102446425	102447606
chr14	102456941	102457182
chr14	102492844	102492854
chr14	102495084	102495236
chr14	102511343	102511417
chr14	102511480	102511542
chr14	102521629	102521943
chr14	102537396	102537482
chr14	102538245	102538385
chr14	102545353	102545842
chr14	102550327	102550638
chr14	102551826	102552004
chr14	102552633	102555605
chr14	102556648	102556847
chr14	102562386	102562485
chr14	102562773	102562835
chr14	102563324	102563325
chr14	102591840	102592021
chr14	102594018	102594200
chr14	102605201	102606019
chr14	102606203	102607388
chr14	102623427	102623658
chr14	102653352	102653766
chr14	102654085	102654289
chr14	102656442	102656571
chr14	102656995	102657438
chr14	102666754	102666862
chr14	102686790	102687059
chr14	102687149	102687496
chr14	102701962	102702077
chr14	102702435	102702587
chr14	102703184	102703413
chr14	102704263	102704515
chr14	102721798	102722017
chr14	102722174	102722543
chr14	102729140	102729888
chr14	102738110	102738355
chr14	102743724	102743923

chr14	102744287	102744486
chr14	102745882	102747764
chr14	102749003	102749521
chr14	102750138	102750521
chr14	102750956	102751357
chr14	102757941	102757969
chr14	102760389	102760537
chr14	102770833	102772215
chr14	102773377	102773581
chr14	102775077	102775186
chr14	102776416	102776431
chr14	102776556	102776727
chr14	102778282	102778402
chr14	102778584	102778754
chr14	102778826	102779348
chr14	102780443	102781324
chr14	102781432	102781480
chr14	102781983	102782640
chr14	102783097	102783289
chr14	102783461	102784174
chr14	102784268	102784796
chr14	102784991	102785019
chr14	102785217	102786916
chr14	102789761	102789810
chr14	102791818	102792126
chr14	102801336	102801373
chr14	102802067	102802429
chr14	102802597	102802622
chr14	102805912	102806111
chr14	102806820	102807011
chr14	102828511	102830374
chr14	102838294	102838677
chr14	102838990	102839072
chr14	102880076	102880382
chr14	102897146	102897255
chr14	102902874	102903065
chr14	102904272	102904377
chr14	102904647	102904822
chr14	102908109	102908786
chr14	102926012	102926267
chr14	102943339	102943538
chr14	102945971	102946283
chr14	102948306	102948495
chr14	102948639	102948689
chr14	102955313	102955632
chr14	102956098	102956142

chr14	102956545	102956622
chr14	102957445	102958635
chr14	102958737	102958752
chr14	102960563	102961634
chr14	102962788	102963125
chr14	102963532	102963928
chr14	102965131	102965589
chr14	102965731	102967680
chr14	102968011	102968724
chr14	102969745	102970488
chr14	102971015	102971530
chr14	102971876	102972214
chr14	102972481	102976034
chr14	102976408	102977732
chr14	102982166	102982234
chr14	102982663	102983967
chr14	102984131	102984330
chr14	102985859	102985923
chr14	102989191	102989636
chr14	102990151	102992078
chr14	102992974	102993361
chr14	102993483	102995076
chr14	102995300	102995960
chr14	102996710	102996995
chr14	102997129	102997546
chr14	102998357	102998789
chr14	102999035	102999143
chr14	102999614	103000087
chr14	103001196	103001255
chr14	103001323	103002467
chr14	103006099	103008125
chr14	103008367	103008724
chr14	103008831	103009916
chr14	103010026	103010188
chr14	103010402	103012378
chr14	103016458	103016700
chr14	103017763	103019853
chr14	103020438	103020810
chr14	103021040	103022270
chr14	103022374	103022487
chr14	103023021	103023222
chr14	103023749	103024321
chr14	103024478	103025342
chr14	103025540	103025987
chr14	103030527	103030704
chr14	103034118	103034608

chr14	103034725	103035044
chr14	103035247	103035700
chr14	103038694	103038857
chr14	103045157	103047201
chr14	103047729	103047904
chr14	103049457	103049719
chr14	103049926	103050493
chr14	103050748	103051881
chr14	103051985	103052234
chr14	103052516	103052757
chr14	103053506	103053743
chr14	103055117	103055631
chr14	103055963	103056319
chr14	103055810	103055847
chr14	103056891	103057360
chr14	103057481	103058718
chr14	103058948	103060390
chr14	103060839	103061289
chr14	103061516	103061779
chr14	103068977	103069381
chr14	103070655	103070875
chr14	103072577	103072807
chr14	103071192	103071802
chr14	103075101	103075320
chr14	103121500	103121557
chr14	103120905	103121376
chr14	103121740	103121868
chr14	103126292	103126395
chr14	103141377	103141482
chr14	103144577	103144833
chr14	103156723	103156960
chr14	103159940	103160064
chr14	103160830	103160939
chr14	103178302	103178573
chr14	103202072	103202271
chr14	103210829	103210977
chr14	103240489	103240614
chr14	103240688	103240829
chr14	103241099	103241880
chr14	103243738	103244034
chr14	103244240	103244854
chr14	103245879	103246289
chr14	103253076	103253449
chr14	103261339	103261446
chr14	103261616	103262028
chr14	103262166	103262310

chr14	103270876	103271077
chr14	103273969	103274148
chr14	103274380	103274709
chr14	103278228	103278370
chr14	103285436	103285770
chr14	103284998	103285079
chr14	103340540	103340739
chr14	103393227	103393699
chr14	103393883	103396883
chr14	103403143	103403212
chr14	103405085	103405284
chr14	103431979	103432017
chr14	103432203	103432313
chr14	103438696	103438897
chr14	103439014	103439219
chr14	103453442	103453773
chr14	103453915	103454068
chr14	103459768	103460639
chr14	103460702	103460759
chr14	103475766	103476398
chr14	103476939	103477980
chr14	103478513	103478688
chr14	103480677	103480752
chr14	103481163	103481197
chr14	103481260	103481451
chr14	103482272	103482366
chr14	103481591	103482199
chr14	103485375	103485595
chr14	103488047	103488139
chr14	103488810	103489261
chr14	103490380	103490773
chr14	103492003	103492402
chr14	103492508	103492876
chr14	103516976	103517027
chr14	103521357	103521686
chr14	103522719	103523757
chr14	103524234	103524913
chr14	103525080	103525279
chr14	103531281	103531545
chr14	103532929	103533135
chr14	103533430	103534970
chr14	103535173	103535372
chr14	103541514	103543024
chr14	103557456	103558418
chr14	103566956	103567530
chr14	103571664	103571915

chr14	103575971	103576649
chr14	103576914	103577188
chr14	103580975	103581125
chr14	103584404	103584700
chr14	103585811	103585870
chr14	103585937	103586062
chr14	103588652	103589041
chr14	103589187	103590463
chr14	103593160	103593544
chr14	103593668	103593952
chr14	103594355	103595842
chr14	103595969	103596168
chr14	103602893	103603519
chr14	103603778	103604196
chr14	103606769	103606920
chr14	103609506	103610850
chr14	103621912	103622240
chr14	103651719	103651791
chr14	103651951	103652536
chr14	103653701	103654006
chr14	103654159	103654173
chr14	103654913	103655690
chr14	103710877	103710951
chr14	103710662	103710802
chr14	103720553	103720563
chr14	103726614	103727301
chr14	103744958	103745356
chr14	103745482	103746790
chr14	103751186	103751273
chr14	103759388	103759861
chr14	103762250	103762657
chr14	103780898	103781079
chr14	103781559	103783051
chr14	103799274	103800363
chr14	103800469	103801010
chr14	103801165	103801793
chr14	103802222	103802457
chr14	103805809	103805847
chr14	103811651	103812014
chr14	103814775	103814897
chr14	103819555	103819754
chr14	103824264	103824651
chr14	103828070	103828676
chr14	103829175	103829464
chr14	103837919	103838118
chr14	103838831	103838862

chr14	103839045	103839321
chr14	103847339	103847489
chr14	103848528	103848995
chr14	103849760	103850033
chr14	103851636	103852949
chr14	103857352	103857594
chr14	103861182	103861407
chr14	103861647	103861983
chr14	103864768	103865155
chr14	103884157	103884372
chr14	103884739	103885109
chr14	103894177	103894291
chr14	103902943	103902991
chr14	103903120	103903252
chr14	103933863	103934102
chr14	103944100	103944304
chr14	103950323	103950330
chr14	103962512	103962696
chr14	103963139	103963457
chr14	103966781	103966900
chr14	103967463	103967678
chr14	103978106	103978360
chr14	103978555	103979603
chr14	103979892	103980179
chr14	103980258	103980285
chr14	103982942	103983156
chr14	103985239	103985624
chr14	103987340	103989184
chr14	103989495	103989713
chr14	103993623	103994394
chr14	103994696	103996237
chr14	103998531	103998737
chr14	104005571	104005770
chr14	104005952	104008401
chr14	104018499	104018843
chr14	104019110	104019312
chr14	104024046	104024296
chr14	104024463	104024862
chr14	104027579	104030068
chr14	104030970	104031368
chr14	104036788	104037037
chr14	104038241	104038436
chr14	104039759	104039909
chr14	104043357	104043398
chr14	104043670	104043824
chr14	104044373	104044564

chr14	104046904	104047412
chr14	104047526	104047642
chr14	104047755	104047980
chr14	104048091	104048271
chr14	104048479	104049147
chr14	104052541	104052665
chr14	104053106	104053259
chr14	104058953	104059188
chr14	104063960	104063986
chr14	104090369	104091042
chr14	104091116	104092302
chr14	104093143	104093419
chr14	104093894	104096390
chr14	104097001	104097378
chr14	104100164	104100381
chr14	104102786	104102881
chr14	104104072	104104379
chr14	104110442	104110899
chr14	104115038	104115215
chr14	104129633	104129901
chr14	104130404	104130544
chr14	104131627	104131972
chr14	104145693	104145750
chr14	104153410	104153872
chr14	104157875	104158248
chr14	104160456	104160700
chr14	104176777	104177120
chr14	104177336	104179172
chr14	104179781	104180041
chr14	104180344	104181792
chr14	104182010	104183152
chr14	104187196	104187398
chr14	104187709	104188136
chr14	104223308	104223635
chr14	104249835	104250035
chr14	104268552	104268889
chr14	104267958	104268192
chr14	104269020	104269177
chr14	104269955	104270551
chr14	104277730	104277929
chr14	104282551	104282722
chr14	104310258	104310461
chr14	104312889	104313424
chr14	104313604	104314879
chr14	104316779	104316954
chr14	104318862	104318871

chr14	104321700	104322061
chr14	104326836	104327193
chr14	104331277	104331476
chr14	104331784	104331832
chr14	104333749	104333966
chr14	104334118	104336000
chr14	104337072	104337480
chr14	104337842	104338300
chr14	104338485	104339615
chr14	104346073	104346837
chr14	104350374	104350573
chr14	104355763	104356110
chr14	104376292	104376506
chr14	104377009	104377205
chr14	104381903	104382093
chr14	104385481	104385515
chr14	104386908	104387107
chr14	104387196	104387929
chr14	104388119	104388256
chr14	104398988	104399858
chr14	104399951	104400251
chr14	104447173	104447351
chr14	104461103	104461390
chr14	104566742	104567350
chr14	104571240	104571553
chr14	104582657	104584130
chr14	104638056	104638437
chr14	104742451	104742621
chr14	105116475	105116811
chr14	105131462	105131555
chr14	105131726	105131981
chr14	105137932	105138143
chr14	105142624	105145149
chr14	105146651	105149833
chr14	105153503	105156482
chr14	105156637	105156932
chr14	105189823	105191606
chr14	105199285	105200046
chr14	105202159	105202671
chr14	105202866	105203152
chr14	105213893	105214040
chr14	105215720	105216240
chr14	105216440	105216639
chr14	105218545	105218975
chr14	105219116	105220096
chr14	105220213	105220412

chr14	105235154	105235406
chr14	105260242	105261793
chr14	105262150	105262520
chr14	105265557	105265809
chr14	105266122	105266693
chr14	105267274	105268108
chr14	105281524	105283689
chr14	105286950	105287127
chr14	105291916	105292988
chr14	105293214	105294096
chr14	105330263	105331544
chr14	105331722	105333268
chr14	105399449	105399862
chr14	105433516	105438559
chr14	105438716	105439535
chr14	105439703	105441047
chr14	105441151	105441570
chr14	105443308	105443507
chr14	105443627	105444605
chr14	105444936	105445135
chr14	105446224	105446527
chr14	105451570	105452878
chr14	105469260	105469541
chr14	105469962	105470862
chr14	105471156	105471905
chr14	105481827	105481950
chr14	105482855	105483750
chr14	105484044	105485044
chr14	105485282	105486542
chr14	105486644	105487463
chr14	105487658	105488528
chr14	105488668	105488867
chr14	105489578	105489674
chr14	105490349	105491700
chr14	105492403	105492839
chr14	105493077	105493416
chr14	105494941	105495172
chr14	105498783	105499025
chr14	105510707	105510865
chr14	105534886	105534941
chr14	105546069	105546268
chr14	105552699	105553202
chr14	105553435	105554253
chr14	105554954	105555396
chr14	105556824	105557991
chr14	105558219	105560911

chr14	105561253	105561306
chr14	105563456	105563778
chr14	105564002	105564433
chr14	105581454	105581621
chr14	105581788	105581844
chr14	105584995	105585241
chr14	105627005	105627204
chr14	105627397	105627667
chr14	105631639	105634755
chr14	105640568	105641039
chr14	105646584	105646792
chr14	105647091	105648241
chr14	105659830	105660151
chr14	105710013	105710421
chr14	105712500	105712783
chr14	105714212	105714790
chr14	105714963	105715724
chr14	105758800	105758942
chr14	105765097	105765250
chr14	105766177	105768292
chr14	105769277	105769476
chr14	105774335	105774574
chr14	105780080	105780853
chr14	105781218	105782193
chr14	105805616	105805815
chr14	105820596	105820795
chr14	105864141	105865458
chr14	105877510	105877792
chr14	105880785	105881653
chr14	105881933	105882120
chr14	105885391	105886057
chr14	105886262	105887365
chr14	105890088	105890093
chr14	105897182	105897259
chr14	105897327	105897540
chr14	105940670	105940869
chr14	105941161	105942798
chr14	105942958	105943300
chr14	105947327	105947987
chr14	105948115	105948387
chr14	105949703	105949989
chr14	105952020	105952268
chr14	105953508	105954083
chr14	105955925	105957145
chr14	105957564	105958103
chr14	105992410	105992724

chr14	105992965	105993681
chr14	105994332	105996434
chr15	20002399	20002630
chr15	20005154	20005790
chr15	20013163	20013417
chr15	20332790	20333133
chr15	20710797	20710889
chr15	20711847	20712079
chr15	20764169	20764227
chr15	20885822	20885837
chr15	21892484	21892527
chr15	21892743	21892744
chr15	22514361	22514570
chr15	22692836	22693178
chr15	22698308	22698346
chr15	22832887	22833281
chr15	22833446	22833978
chr15	22834092	22834211
chr15	22891864	22892913
chr15	22893096	22893510
chr15	22977546	22977663
chr15	22984122	22984357
chr15	22984571	22984585
chr15	23032879	23033195
chr15	23034572	23035249
chr15	23085667	23086314
chr15	23086688	23087237
chr15	23158119	23158499
chr15	23207685	23207803
chr15	23208031	23208689
chr15	23216991	23217273
chr15	23377439	23377851
chr15	23482214	23482476
chr15	25683262	25683773
chr15	25683968	25684137
chr15	25684528	25684573
chr15	26327905	26328023
chr15	28566577	28566996
chr15	28567587	28567922
chr15	28639390	28639477
chr15	28641194	28641549
chr15	28699800	28699948
chr15	28753627	28753874
chr15	28758983	28759176
chr15	28760392	28760766
chr15	28762154	28762289

chr15	28762461	28762539
chr15	28834106	28834420
chr15	28834886	28835248
chr15	28982652	28983510
chr15	28983641	28983766
chr15	29064793	29064974
chr15	29130664	29131087
chr15	29131305	29131609
chr15	29561584	29562021
chr15	29963306	29963551
chr15	29966885	29967701
chr15	30049083	30049470
chr15	30067129	30067137
chr15	30104852	30104924
chr15	30113521	30113676
chr15	30114098	30114662
chr15	30114863	30115093
chr15	30130615	30131017
chr15	30135826	30135928
chr15	30136134	30136214
chr15	30136640	30136697
chr15	30136995	30137088
chr15	30213415	30213740
chr15	30216974	30216991
chr15	30226373	30226531
chr15	30226718	30226831
chr15	30230208	30230576
chr15	30238518	30238591
chr15	30744411	30744465
chr15	30763240	30764145
chr15	30793930	30793943
chr15	30794168	30794242
chr15	30917477	30918712
chr15	30918843	30918999
chr15	30916379	30916730
chr15	30919126	30919182
chr15	31058877	31059010
chr15	31195714	31196501
chr15	31283406	31284462
chr15	31284701	31284775
chr15	31435657	31435880
chr15	31442780	31442999
chr15	31443471	31443562
chr15	31456029	31456108
chr15	31456415	31456610
chr15	31506786	31507191

chr15	31507372	31508880
chr15	31555408	31555616
chr15	31555866	31556301
chr15	31556416	31556621
chr15	31556735	31556963
chr15	31617624	31618580
chr15	31618953	31619692
chr15	31619798	31620996
chr15	31621311	31621483
chr15	31644044	31644058
chr15	31656786	31657610
chr15	31657889	31658160
chr15	31658319	31658769
chr15	31664257	31664258
chr15	31664702	31664912
chr15	31683774	31683797
chr15	31684003	31684870
chr15	31688622	31688672
chr15	31689010	31689209
chr15	31689529	31689882
chr15	31690166	31690756
chr15	31690859	31691054
chr15	31691359	31692138
chr15	31692656	31692800
chr15	31726876	31727193
chr15	31727369	31727924
chr15	31733260	31733424
chr15	31733679	31734143
chr15	31734816	31735350
chr15	31749768	31749873
chr15	31757488	31758290
chr15	31775544	31775903
chr15	31931109	31931584
chr15	32796978	32797052
chr15	32797881	32797989
chr15	32798164	32798208
chr15	32828058	32828172
chr15	32828289	32828502
chr15	32828634	32829318
chr15	32905754	32906100
chr15	32907439	32907951
chr15	32908209	32908584
chr15	32949603	32949880
chr15	32951606	32951784
chr15	32952051	32952165
chr15	32954850	32954961

chr15	32975160	32975210
chr15	33117314	33117376
chr15	33136406	33136597
chr15	33141077	33141097
chr15	33141261	33141494
chr15	33142175	33143026
chr15	33143923	33144130
chr15	33148087	33148088
chr15	33159546	33159837
chr15	33306393	33306474
chr15	33314749	33315190
chr15	33378909	33379035
chr15	33396573	33396655
chr15	33437622	33437833
chr15	33437974	33438193
chr15	33487385	33487516
chr15	33571047	33571570
chr15	33571757	33571854
chr15	33571954	33572053
chr15	34330674	34330873
chr15	34331079	34331588
chr15	34393882	34394049
chr15	34394427	34394493
chr15	34447361	34447462
chr15	34501764	34502246
chr15	34516956	34517435
chr15	34523537	34524195
chr15	34531551	34531913
chr15	34610803	34610884
chr15	34629551	34629750
chr15	34629972	34630274
chr15	34635121	34635390
chr15	34658666	34659367
chr15	34659491	34659738
chr15	34727090	34727376
chr15	34728215	34728282
chr15	34728412	34729621
chr15	34730165	34730243
chr15	34875246	34875875
chr15	34974860	34974987
chr15	35261771	35261987
chr15	35262240	35262329
chr15	35280234	35280543
chr15	35280653	35280852
chr15	35280996	35281143
chr15	35283659	35283943

chr15	35292478	35292732
chr15	35292947	35293147
chr15	35311783	35311962
chr15	35331644	35332042
chr15	35548987	35549050
chr15	35837633	35838761
chr15	36663095	36663122
chr15	36684835	36685058
chr15	36720876	36720891
chr15	36871205	36871632
chr15	36872020	36872403
chr15	36878007	36878358
chr15	36904355	36904714
chr15	36905058	36905075
chr15	36936019	36936096
chr15	37391301	37391568
chr15	37391698	37391778
chr15	37392464	37392732
chr15	37392869	37393511
chr15	37393646	37393866
chr15	38330883	38330908
chr15	38331192	38331312
chr15	38342072	38342261
chr15	38361624	38361885
chr15	38364569	38365378
chr15	38421827	38422286
chr15	38500176	38500231
chr15	38510346	38510618
chr15	38510774	38510886
chr15	38519079	38519083
chr15	38543496	38545994
chr15	38546439	38546910
chr15	38547022	38547102
chr15	38558563	38558942
chr15	38746129	38746171
chr15	38746390	38746989
chr15	38856750	38857002
chr15	39371400	39371664
chr15	39420437	39420855
chr15	39464167	39464336
chr15	39464599	39464677
chr15	39469651	39470237
chr15	39481077	39481538
chr15	39484115	39484364
chr15	39488053	39488829
chr15	39490305	39490753

chr15	39494847	39495207
chr15	39505564	39506053
chr15	39524701	39524763
chr15	39579549	39579821
chr15	39580048	39580097
chr15	39592986	39593329
chr15	39603122	39603134
chr15	39683294	39683422
chr15	39683967	39684088
chr15	39695911	39696362
chr15	39696831	39697041
chr15	39697321	39697472
chr15	39712448	39713006
chr15	39713154	39713396
chr15	39848894	39849059
chr15	39867020	39867030
chr15	39871753	39874163
chr15	39875417	39875976
chr15	39908942	39909632
chr15	39910659	39911012
chr15	39916897	39917577
chr15	39921479	39922640
chr15	39923174	39923354
chr15	39923825	39923918
chr15	39933573	39933707
chr15	39933834	39934065
chr15	39934298	39934637
chr15	39950166	39950270
chr15	40074478	40074596
chr15	40074730	40075364
chr15	40096530	40096650
chr15	40169810	40170277
chr15	40177808	40177884
chr15	40180321	40180334
chr15	40188395	40188594
chr15	40200835	40200907
chr15	40210991	40211349
chr15	40211753	40213581
chr15	40225715	40227202
chr15	40228311	40228419
chr15	40228611	40228693
chr15	40258632	40258640
chr15	40262389	40262500
chr15	40294401	40294461
chr15	40330763	40331776
chr15	40337414	40337613

chr15	40339356	40339629
chr15	40339907	40340229
chr15	40368977	40369403
chr15	40373892	40374091
chr15	40390723	40391018
chr15	40391689	40391922
chr15	40395159	40395853
chr15	40395980	40396272
chr15	40397183	40397554
chr15	40398491	40398876
chr15	40398982	40399185
chr15	40400836	40401138
chr15	40442936	40443036
chr15	40447791	40448079
chr15	40452721	40453711
chr15	40483016	40483197
chr15	40524956	40525180
chr15	40527433	40527496
chr15	40530717	40533177
chr15	40533569	40533806
chr15	40535419	40535732
chr15	40537303	40537406
chr15	40537745	40538038
chr15	40544856	40545708
chr15	40559463	40559790
chr15	40567146	40567442
chr15	40573763	40574336
chr15	40583001	40583840
chr15	40615608	40615907
chr15	40616108	40617042
chr15	40617467	40617692
chr15	40620241	40620526
chr15	40626187	40626401
chr15	40631331	40633136
chr15	40634618	40635082
chr15	40635257	40636540
chr15	40659922	40660646
chr15	40672274	40672403
chr15	40675133	40675607
chr15	40681404	40681500
chr15	40697105	40697477
chr15	40697602	40697764
chr15	40697973	40698774
chr15	40713951	40714150
chr15	40728140	40729053
chr15	40731985	40732633

chr15	40732834	40733079
chr15	40733287	40733792
chr15	40733915	40734114
chr15	40734240	40734552
chr15	40735013	40735069
chr15	40747420	40747747
chr15	40748117	40749101
chr15	40757247	40757474
chr15	40762920	40763926
chr15	40799637	40799869
chr15	40803389	40804414
chr15	40850759	40851005
chr15	40856670	40858054
chr15	40861260	40861361
chr15	40861539	40862351
chr15	40875211	40875409
chr15	40882127	40882505
chr15	40885931	40885977
chr15	40886141	40887109
chr15	40890470	40890704
chr15	40971527	40971717
chr15	40986163	40987692
chr15	40987831	40988198
chr15	40988530	40988778
chr15	41014255	41014417
chr15	41039596	41039958
chr15	41046547	41046870
chr15	41047072	41047935
chr15	41051155	41051356
chr15	41055281	41056127
chr15	41056480	41057405
chr15	41057534	41058078
chr15	41058786	41059191
chr15	41060534	41060844
chr15	41060987	41062076
chr15	41062180	41062740
chr15	41068769	41068791
chr15	41068904	41068968
chr15	41079292	41079440
chr15	41079825	41080457
chr15	41080689	41081018
chr15	41083095	41083481
chr15	41085731	41085866
chr15	41086032	41086128
chr15	41089114	41089498
chr15	41089994	41090201

chr15	41090904	41090975
chr15	41099089	41099778
chr15	41099903	41100233
chr15	41100345	41100692
chr15	41117325	41117734
chr15	41127402	41127808
chr15	41135580	41138581
chr15	41139242	41139258
chr15	41139558	41139757
chr15	41141470	41141562
chr15	41144286	41144394
chr15	41145333	41145609
chr15	41149499	41149924
chr15	41151276	41151475
chr15	41152094	41152247
chr15	41153005	41153574
chr15	41154107	41154306
chr15	41154493	41154690
chr15	41157355	41157793
chr15	41157927	41158139
chr15	41158378	41158655
chr15	41163008	41163326
chr15	41163797	41164955
chr15	41165294	41166755
chr15	41167836	41169181
chr15	41169911	41170377
chr15	41170961	41171201
chr15	41171387	41171746
chr15	41180170	41180346
chr15	41183599	41183630
chr15	41183999	41184151
chr15	41184337	41184488
chr15	41184863	41186488
chr15	41186765	41187040
chr15	41187264	41187433
chr15	41194451	41194542
chr15	41194996	41195264
chr15	41196594	41196816
chr15	41198883	41199238
chr15	41206170	41206357
chr15	41207556	41207755
chr15	41218059	41218621
chr15	41220191	41220732
chr15	41221152	41221379
chr15	41221597	41222033
chr15	41233540	41235054

chr15	41244816	41245586
chr15	41245714	41245890
chr15	41247292	41247507
chr15	41264017	41264285
chr15	41291299	41291481
chr15	41291598	41291797
chr15	41407459	41408741
chr15	41408888	41409062
chr15	41431285	41431355
chr15	41431552	41431653
chr15	41477357	41477404
chr15	41499812	41500692
chr15	41522696	41524091
chr15	41541312	41542117
chr15	41542257	41542395
chr15	41552019	41552121
chr15	41556236	41556635
chr15	41573023	41573222
chr15	41573329	41573855
chr15	41575544	41575980
chr15	41576217	41576771
chr15	41579314	41579483
chr15	41604865	41605166
chr15	41607025	41607105
chr15	41623538	41623925
chr15	41624169	41624697
chr15	41625048	41625539
chr15	41629895	41630088
chr15	41675450	41675618
chr15	41687779	41688103
chr15	41694361	41694684
chr15	41694790	41694894
chr15	41698854	41698856
chr15	41699701	41699846
chr15	41702954	41703293
chr15	41703576	41703933
chr15	41708344	41709160
chr15	41709282	41709965
chr15	41728964	41729129
chr15	41769397	41769578
chr15	41770039	41770248
chr15	41782417	41782509
chr15	41782913	41782959
chr15	41783068	41783135
chr15	41784587	41784827
chr15	41785515	41786047

chr15	41786239	41786439
chr15	41786986	41787218
chr15	41794487	41794686
chr15	41836013	41836950
chr15	41846405	41846574
chr15	41848428	41848711
chr15	41850318	41850623
chr15	41851057	41852018
chr15	41877333	41877994
chr15	41878274	41878354
chr15	41912758	41913100
chr15	41913229	41914542
chr15	41921765	41921964
chr15	41924077	41924148
chr15	41952058	41952517
chr15	41952997	41953211
chr15	41953696	41954147
chr15	41954305	41954333
chr15	42065170	42065368
chr15	42066048	42066555
chr15	42066677	42067629
chr15	42067705	42068187
chr15	42069180	42070031
chr15	42071793	42072145
chr15	42080722	42080783
chr15	42083014	42083132
chr15	42083312	42084000
chr15	42086189	42086668
chr15	42090239	42090459
chr15	42090908	42090936
chr15	42093676	42093683
chr15	42094088	42094602
chr15	42095789	42097718
chr15	42098025	42098255
chr15	42098474	42098553
chr15	42098698	42099614
chr15	42100352	42100576
chr15	42100957	42101627
chr15	42111684	42111865
chr15	42112217	42112249
chr15	42118786	42120045
chr15	42120297	42121439
chr15	42121971	42122242
chr15	42124513	42125079
chr15	42125326	42125876
chr15	42126165	42126359

chr15	42131030	42131250
chr15	42162560	42162883
chr15	42173858	42174408
chr15	42177361	42177509
chr15	42186741	42187455
chr15	42187560	42187853
chr15	42190062	42190267
chr15	42209873	42210002
chr15	42210259	42210540
chr15	42222934	42223156
chr15	42223421	42223535
chr15	42223838	42224104
chr15	42225989	42226470
chr15	42226954	42227203
chr15	42227440	42227456
chr15	42232253	42232609
chr15	42233089	42233201
chr15	42236114	42236542
chr15	42236718	42237113
chr15	42237205	42237383
chr15	42239055	42239057
chr15	42239357	42239597
chr15	42240090	42240299
chr15	42260190	42260272
chr15	42260960	42261234
chr15	42263679	42263730
chr15	42263947	42265364
chr15	42288257	42288447
chr15	42301978	42302315
chr15	42302565	42302810
chr15	42304160	42304520
chr15	42303328	42303729
chr15	42323219	42323943
chr15	42324065	42324170
chr15	42324690	42325013
chr15	42325869	42326138
chr15	42327416	42327727
chr15	42327871	42328127
chr15	42328383	42328803
chr15	42329269	42329272
chr15	42330001	42330285
chr15	42341095	42341356
chr15	42346519	42346572
chr15	42349094	42350521
chr15	42376364	42376532
chr15	42377006	42377463

chr15	42386740	42386977
chr15	42450827	42450866
chr15	42486612	42486748
chr15	42499907	42500815
chr15	42567321	42567678
chr15	42565090	42566830
chr15	42624502	42624705
chr15	42763708	42763855
chr15	42782708	42783382
chr15	42783692	42784200
chr15	42787294	42788374
chr15	42791382	42791576
chr15	42791745	42791841
chr15	42794376	42794595
chr15	42801682	42801818
chr15	42803072	42803181
chr15	42803295	42803433
chr15	42840436	42841612
chr15	42842878	42843077
chr15	42867118	42867283
chr15	42867867	42868100
chr15	42873433	42874013
chr15	42971872	42972162
chr15	42972232	42972673
chr15	43013701	43013767
chr15	43028443	43029680
chr15	43030018	43030428
chr15	43118755	43119202
chr15	43124745	43125229
chr15	43137778	43137977
chr15	43138467	43138582
chr15	43151112	43151124
chr15	43182565	43182812
chr15	43182954	43183345
chr15	43183753	43184147
chr15	43203311	43203373
chr15	43203498	43203670
chr15	43212042	43213057
chr15	43213992	43214094
chr15	43214790	43214950
chr15	43225335	43225407
chr15	43227053	43227150
chr15	43227411	43227896
chr15	43377897	43377975
chr15	43393141	43393628
chr15	43396148	43396225

chr15	43396372	43396475
chr15	43396666	43396977
chr15	43397562	43398634
chr15	43415592	43415772
chr15	43425121	43426655
chr15	43442338	43442669
chr15	43442748	43443173
chr15	43477594	43478135
chr15	43478429	43478511
chr15	43501804	43501965
chr15	43511735	43512119
chr15	43621642	43623096
chr15	43662644	43663872
chr15	43664046	43664052
chr15	43682774	43683008
chr15	43683190	43683319
chr15	43733000	43733187
chr15	43733427	43733502
chr15	43757412	43757611
chr15	43785010	43785551
chr15	43785721	43785863
chr15	43802472	43803058
chr15	43859008	43859223
chr15	43881413	43881680
chr15	43881908	43882719
chr15	43884833	43886074
chr15	43886353	43886628
chr15	43893960	43894040
chr15	43894317	43894580
chr15	43958390	43958789
chr15	43959639	43959812
chr15	43978044	43978243
chr15	43981341	43981585
chr15	43981814	43982594
chr15	43984769	43985633
chr15	43985865	43986682
chr15	44037770	44038531
chr15	44038667	44039454
chr15	44041863	44042094
chr15	44063299	44063417
chr15	44064227	44064430
chr15	44066501	44066576
chr15	44067541	44067750
chr15	44068204	44069123
chr15	44069263	44070058
chr15	44083812	44084292

chr15	44084526	44085404
chr15	44085775	44085894
chr15	44089759	44089957
chr15	44090465	44090635
chr15	44090780	44091066
chr15	44092437	44093428
chr15	44116483	44117202
chr15	44118831	44119416
chr15	44121136	44121326
chr15	44292610	44292813
chr15	44301517	44301630
chr15	44358443	44358483
chr15	44380304	44380327
chr15	44394154	44394223
chr15	44394448	44394590
chr15	44450073	44450208
chr15	44512132	44512238
chr15	44579980	44580183
chr15	44580314	44580838
chr15	44581008	44581855
chr15	44588204	44588304
chr15	44588502	44588598
chr15	44588737	44589226
chr15	44589351	44589719
chr15	44632537	44632773
chr15	44718645	44719124
chr15	44719380	44720303
chr15	44720546	44720958
chr15	44736604	44736737
chr15	44760884	44761279
chr15	44827866	44829119
chr15	44829321	44830030
chr15	44838627	44838769
chr15	44838892	44838929
chr15	44862008	44862237
chr15	44955167	44955773
chr15	44956101	44956232
chr15	45003312	45004507
chr15	45004753	45005139
chr15	45012212	45012234
chr15	45012347	45012445
chr15	45018386	45019522
chr15	45020600	45021120
chr15	45021278	45021938
chr15	45076416	45077025
chr15	45314812	45315264

chr15	45315379	45315749
chr15	45316151	45316480
chr15	45350754	45350921
chr15	45353785	45354193
chr15	45421476	45422658
chr15	45454415	45455627
chr15	45458375	45459835
chr15	45479577	45480174
chr15	45480405	45480751
chr15	45480874	45481171
chr15	45489805	45490064
chr15	45490239	45490748
chr15	45491059	45491639
chr15	45491759	45492017
chr15	45492128	45492592
chr15	45492742	45493264
chr15	45571166	45571336
chr15	45571678	45571922
chr15	45599437	45599441
chr15	45669957	45670844
chr15	45691810	45691878
chr15	45694216	45694304
chr15	45694570	45696086
chr15	45722359	45722496
chr15	45730691	45730947
chr15	45732744	45733139
chr15	45737267	45737406
chr15	45738324	45738350
chr15	45746736	45747136
chr15	45747332	45747807
chr15	45748090	45748867
chr15	45749032	45750096
chr15	45750246	45750337
chr15	45751316	45751322
chr15	45814388	45815011
chr15	45815126	45815325
chr15	45878636	45879013
chr15	45879129	45880091
chr15	45906693	45906788
chr15	45922644	45922674
chr15	45923372	45924469
chr15	45926643	45927074
chr15	45927234	45928281
chr15	45929701	45930146
chr15	45932726	45933241
chr15	45933429	45933731

chr15	45933899	45934071
chr15	45934235	45934624
chr15	45936746	45936754
chr15	45937710	45938449
chr15	45940730	45941180
chr15	45943396	45943408
chr15	45996859	45997290
chr15	45997481	45997553
chr15	45997865	45997988
chr15	45998091	45998257
chr15	46148742	46148807
chr15	46211885	46212019
chr15	46212453	46212502
chr15	47388110	47388153
chr15	47676803	47676962
chr15	47753425	47753603
chr15	47758460	47759062
chr15	47913203	47913217
chr15	47958300	47958397
chr15	47980685	47981046
chr15	48009326	48009652
chr15	48009866	48010148
chr15	48010581	48011001
chr15	48049068	48049188
chr15	48094172	48094439
chr15	48095957	48096004
chr15	48104499	48104734
chr15	48104915	48105165
chr15	48112311	48112384
chr15	48112715	48112772
chr15	48112848	48113268
chr15	48325158	48325334
chr15	48442077	48442416
chr15	48469980	48470789
chr15	48622802	48624563
chr15	48765019	48765113
chr15	48858117	48858147
chr15	48869804	48870070
chr15	48919469	48919609
chr15	48933929	48933956
chr15	48936913	48937124
chr15	48937353	48937659
chr15	48938129	48938447
chr15	48996096	48996133
chr15	49052056	49052196
chr15	49052363	49052454

chr15	49102782	49103178
chr15	49160253	49160519
chr15	49160743	49160862
chr15	49161004	49161108
chr15	49169891	49170104
chr15	49170382	49170811
chr15	49181696	49181799
chr15	49267852	49268361
chr15	49338141	49339064
chr15	49447214	49447768
chr15	49447992	49448251
chr15	49462018	49462841
chr15	49528085	49528138
chr15	49785006	49785077
chr15	49785411	49785487
chr15	49912832	49912995
chr15	49913264	49913489
chr15	49919538	49919888
chr15	50474423	50474720
chr15	50474952	50475166
chr15	50485015	50485844
chr15	50502356	50502368
chr15	50502891	50503093
chr15	50572785	50572843
chr15	50608140	50608289
chr15	50645447	50645695
chr15	50646062	50646280
chr15	50646398	50648066
chr15	50715842	50716063
chr15	50716153	50716247
chr15	50716653	50716944
chr15	50834973	50835038
chr15	50835354	50835368
chr15	50928495	50928693
chr15	50945588	50945606
chr15	50945827	50946044
chr15	50969531	50969631
chr15	50970329	50970582
chr15	50977474	50977737
chr15	50978198	50979624
chr15	51039138	51039171
chr15	51054165	51054364
chr15	51057151	51058342
chr15	51090046	51090121
chr15	51090433	51090512
chr15	51090944	51090986

chr15	51108725	51108768
chr15	51146643	51146878
chr15	51169146	51169284
chr15	51173313	51173981
chr15	51176405	51176489
chr15	51176725	51177124
chr15	51177398	51178491
chr15	51199972	51200730
chr15	51200859	51201252
chr15	51202322	51202737
chr15	51201389	51201529
chr15	51202884	51203120
chr15	51288869	51289187
chr15	51316911	51317068
chr15	51317193	51317319
chr15	51317767	51317869
chr15	51372711	51372853
chr15	51386167	51386400
chr15	51633782	51634075
chr15	51913997	51915445
chr15	51935303	51935304
chr15	51935611	51935810
chr15	52023647	52024312
chr15	52029230	52030335
chr15	52031277	52031655
chr15	52043593	52044305
chr15	52044439	52044855
chr15	52069113	52069267
chr15	52093112	52093545
chr15	52093614	52093761
chr15	52120815	52120905
chr15	52121671	52122666
chr15	52134992	52135286
chr15	52138248	52138885
chr15	52153836	52154191
chr15	52156220	52156723
chr15	52156986	52157251
chr15	52158452	52158738
chr15	52160967	52161501
chr15	52164377	52164577
chr15	52166745	52167281
chr15	52167393	52168059
chr15	52168314	52168801
chr15	52174196	52174489
chr15	52180437	52180771
chr15	52224096	52224290

chr15	52263428	52264559
chr15	52273718	52274070
chr15	52274335	52274485
chr15	52285562	52285638
chr15	52287099	52287242
chr15	52287470	52287652
chr15	52303101	52303245
chr15	52303354	52303620
chr15	52310755	52311317
chr15	52311851	52312382
chr15	52313351	52313674
chr15	52323675	52323883
chr15	52323992	52324155
chr15	52342642	52342915
chr15	52359747	52360101
chr15	52360202	52360239
chr15	52360554	52360586
chr15	52363615	52363769
chr15	52363893	52364209
chr15	52368887	52369747
chr15	52369982	52371008
chr15	52371089	52371437
chr15	52372228	52372368
chr15	52372657	52372674
chr15	52385453	52386666
chr15	52387679	52387816
chr15	52388097	52388219
chr15	52392601	52393181
chr15	52393347	52393467
chr15	52394948	52395049
chr15	52395638	52395791
chr15	52395967	52396132
chr15	52404049	52404893
chr15	52405069	52405220
chr15	52417626	52417629
chr15	52436452	52436553
chr15	52436999	52437217
chr15	52471405	52472747
chr15	52512786	52512795
chr15	52521477	52521592
chr15	52543078	52543081
chr15	52544717	52545098
chr15	52545294	52545422
chr15	52587476	52587960
chr15	52611223	52611436
chr15	52611587	52611724

chr15	52623934	52624087
chr15	52626713	52627152
chr15	52627351	52627413
chr15	52629627	52629762
chr15	52730244	52730560
chr15	52732685	52732686
chr15	52753673	52753916
chr15	52789064	52789434
chr15	52805030	52805319
chr15	52820377	52821889
chr15	52850340	52850789
chr15	52851778	52852116
chr15	52860309	52860676
chr15	52860849	52861590
chr15	52944068	52944241
chr15	52944376	52944538
chr15	52945467	52945949
chr15	52970242	52970400
chr15	52970581	52971944
chr15	53021127	53021448
chr15	55488592	55489570
chr15	55509793	55509853
chr15	55513255	55513811
chr15	55542531	55542815
chr15	55545717	55545746
chr15	55545956	55546080
chr15	55554777	55555001
chr15	55572083	55572271
chr15	55581305	55583075
chr15	55610633	55611634
chr15	55636384	55636391
chr15	55636760	55636859
chr15	55681707	55681770
chr15	55699865	55700490
chr15	55700775	55701091
chr15	55790632	55790882
chr15	55830465	55830592
chr15	55879727	55880078
chr15	55880418	55880626
chr15	55880768	55881196
chr15	55919821	55920426
chr15	55934092	55934159
chr15	55941199	55941320
chr15	56034401	56035466
chr15	56189220	56190259
chr15	56196411	56196904

chr15	56199940	56199952
chr15	56200117	56200440
chr15	56201349	56201606
chr15	56209309	56209317
chr15	56209448	56209525
chr15	56210114	56210544
chr15	56285153	56286595
chr15	56286856	56287168
chr15	56290514	56291056
chr15	56291834	56292496
chr15	56293780	56294283
chr15	56298854	56298876
chr15	56310010	56310443
chr15	56315357	56315720
chr15	56311233	56312506
chr15	56320670	56320674
chr15	56320802	56320907
chr15	56330156	56330518
chr15	56332171	56332770
chr15	56332938	56333090
chr15	56334177	56334292
chr15	56334470	56334566
chr15	56343099	56343340
chr15	56343824	56344176
chr15	56344676	56344894
chr15	56345183	56345263
chr15	56352643	56352829
chr15	56460597	56460666
chr15	56535104	56535290
chr15	56535475	56537484
chr15	56538045	56538340
chr15	56633328	56633361
chr15	56657285	56657472
chr15	56657668	56657869
chr15	56661479	56661851
chr15	56757050	56757103
chr15	56757461	56757622
chr15	57025108	57026101
chr15	57098248	57098364
chr15	57179106	57179367
chr15	57179504	57180173
chr15	57199034	57199389
chr15	57209849	57210702
chr15	57210906	57211054
chr15	57211122	57212327
chr15	57256589	57256785

chr15	57261237	57261421
chr15	57271497	57271602
chr15	57308093	57308141
chr15	57359979	57360056
chr15	57389469	57389813
chr15	57390160	57390467
chr15	57598439	57598829
chr15	57599059	57599280
chr15	57615791	57616435
chr15	57625355	57625505
chr15	57668422	57668619
chr15	57668738	57669039
chr15	57844180	57844408
chr15	57846011	57846199
chr15	57846453	57846589
chr15	57853455	57854003
chr15	57863171	57863598
chr15	57883748	57884452
chr15	57889127	57889279
chr15	57903912	57904262
chr15	57940093	57940292
chr15	57941300	57941720
chr15	57998255	57998736
chr15	57998864	57999397
chr15	58311792	58312112
chr15	58526616	58527098
chr15	58551022	58551487
chr15	58553077	58553353
chr15	58559407	58559550
chr15	58623556	58623944
chr15	58624029	58624461
chr15	58624646	58624852
chr15	58681728	58681992
chr15	58688371	58688786
chr15	58734168	58734632
chr15	58750014	58750252
chr15	58761899	58762581
chr15	58763604	58763708
chr15	58763823	58763877
chr15	58764651	58764852
chr15	58764969	58765504
chr15	58765902	58766238
chr15	58767293	58767419
chr15	58767769	58767875
chr15	58791296	58791383
chr15	58813475	58813725

chr15	58844055	58844535
chr15	58970961	58971065
chr15	59023941	59023990
chr15	59030457	59030680
chr15	59040931	59041978
chr15	59042325	59042486
chr15	59062763	59063017
chr15	59063491	59064535
chr15	59085810	59086565
chr15	59104660	59105139
chr15	59131750	59131766
chr15	59131904	59132031
chr15	59157980	59158131
chr15	59158270	59158446
chr15	59158700	59158986
chr15	59167543	59167633
chr15	59167939	59167962
chr15	59216125	59216270
chr15	59225152	59225716
chr15	59225833	59226531
chr15	59243744	59244257
chr15	59249928	59249930
chr15	59255199	59255241
chr15	59279604	59280852
chr15	59331957	59332194
chr15	59397017	59397256
chr15	59397369	59397967
chr15	59398184	59398434
chr15	59437005	59437158
chr15	59444009	59444101
chr15	59457863	59458599
chr15	59460725	59461058
chr15	59462369	59463110
chr15	59483628	59483687
chr15	59483900	59484245
chr15	59484484	59484498
chr15	59569270	59569357
chr15	59569483	59569810
chr15	59572515	59572767
chr15	59576016	59576150
chr15	59576469	59576678
chr15	59576748	59577090
chr15	59577591	59577701
chr15	59587146	59587661
chr15	59587825	59588861
chr15	59590390	59590947

chr15	59592477	59592691
chr15	59592850	59592952
chr15	59597617	59598079
chr15	59605984	59606016
chr15	59631481	59631680
chr15	59631788	59632157
chr15	59642258	59642275
chr15	59642660	59642795
chr15	59656762	59656790
chr15	59657303	59657502
chr15	59659263	59660089
chr15	59661754	59662617
chr15	59662681	59662887
chr15	59663556	59665022
chr15	59665369	59665491
chr15	59668924	59669095
chr15	59680854	59681347
chr15	59730449	59730721
chr15	59748263	59748405
chr15	59748574	59748757
chr15	59749301	59749689
chr15	59775090	59775284
chr15	59846370	59846553
chr15	59847828	59848091
chr15	59848194	59848654
chr15	59859600	59859644
chr15	59859759	59859989
chr15	59862505	59862577
chr15	59862881	59862973
chr15	59886646	59887067
chr15	59949482	59949691
chr15	59950087	59950092
chr15	59980661	59982361
chr15	59992312	59992642
chr15	60016745	60016752
chr15	60017010	60017222
chr15	60095831	60095903
chr15	60121116	60121155
chr15	60121352	60121476
chr15	60351532	60351597
chr15	60362986	60362992
chr15	60383305	60383431
chr15	60419695	60419842
chr15	60591694	60591895
chr15	60609376	60609566
chr15	60621338	60621625

chr15	60641962	60642345
chr15	60657918	60658018
chr15	60663500	60664883
chr15	60665034	60665380
chr15	60665446	60665806
chr15	60666755	60666847
chr15	60667354	60667775
chr15	60668731	60669099
chr15	60670070	60670416
chr15	60671642	60672074
chr15	60673678	60674000
chr15	60682487	60682658
chr15	60683219	60683789
chr15	60684769	60685407
chr15	60685645	60686002
chr15	60687767	60688251
chr15	60688393	60689200
chr15	60686219	60687238
chr15	60689360	60691494
chr15	60699725	60699998
chr15	60700582	60701182
chr15	60701439	60701706
chr15	60706230	60706306
chr15	60706443	60706657
chr15	60715741	60715847
chr15	60715989	60716122
chr15	60719531	60719644
chr15	60757443	60757705
chr15	60762496	60762497
chr15	60770328	60771379
chr15	60771490	60771691
chr15	60802194	60802298
chr15	60812658	60812683
chr15	60883287	60883494
chr15	60883848	60884305
chr15	60884452	60884656
chr15	61519619	61520018
chr15	61520415	61520608
chr15	62041103	62041201
chr15	62041723	62041793
chr15	62351900	62352646
chr15	62356152	62356339
chr15	62356456	62356826
chr15	62358881	62358945
chr15	62360379	62360790
chr15	62363946	62364045

chr15	62368083	62368196
chr15	62411344	62411931
chr15	62682203	62682546
chr15	62682701	62682927
chr15	62683356	62684138
chr15	62695383	62695466
chr15	62695667	62695678
chr15	62895920	62895974
chr15	62896398	62896404
chr15	63034371	63034475
chr15	63034680	63034749
chr15	63045586	63046204
chr15	63046405	63046518
chr15	63050183	63050615
chr15	63050813	63050841
chr15	63062872	63063158
chr15	63061854	63062206
chr15	63120797	63120851
chr15	63127450	63127587
chr15	63127744	63128003
chr15	63143005	63143334
chr15	63188314	63188948
chr15	63189077	63189868
chr15	63192353	63193000
chr15	63194108	63194120
chr15	63212980	63213135
chr15	63220481	63220675
chr15	63232560	63232773
chr15	63233073	63233428
chr15	63298411	63298710
chr15	63298981	63299054
chr15	63311571	63311652
chr15	63312022	63312221
chr15	63315888	63315899
chr15	63329398	63329597
chr15	63330072	63330787
chr15	63332711	63333214
chr15	63333913	63333945
chr15	63334291	63336245
chr15	63340045	63340250
chr15	63340367	63341642
chr15	63389218	63389316
chr15	63413931	63414671
chr15	63428010	63428512
chr15	63449202	63450457
chr15	63478197	63478232

chr15	63481078	63481496
chr15	63481650	63482120
chr15	63482193	63482260
chr15	63482502	63482890
chr15	63482996	63483093
chr15	63485884	63486242
chr15	63486347	63486419
chr15	63521371	63521777
chr15	63525385	63525490
chr15	63539160	63539682
chr15	63568916	63569124
chr15	63569192	63569684
chr15	63569799	63570140
chr15	63581057	63581105
chr15	63640622	63640644
chr15	63640913	63640988
chr15	63664401	63664653
chr15	63673230	63673380
chr15	63673504	63674111
chr15	63741056	63741430
chr15	63741903	63741957
chr15	63775121	63775837
chr15	63779517	63779920
chr15	63784882	63784998
chr15	63793901	63793983
chr15	63794361	63795023
chr15	63795160	63796590
chr15	63796910	63797711
chr15	63797970	63798108
chr15	63806848	63807204
chr15	63809827	63810195
chr15	63814415	63814477
chr15	63814798	63814819
chr15	63891625	63891824
chr15	63892322	63893794
chr15	63893984	63894191
chr15	64010893	64010918
chr15	64011160	64011247
chr15	64011603	64011944
chr15	64014056	64014287
chr15	64125485	64125541
chr15	64125740	64126894
chr15	64201847	64202022
chr15	64225966	64226059
chr15	64274475	64274674
chr15	64296764	64296825

chr15	64297220	64297332
chr15	64299235	64299494
chr15	64338040	64338847
chr15	64350545	64350606
chr15	64385252	64386085
chr15	64386258	64386679
chr15	64387831	64387996
chr15	64388131	64388448
chr15	64388903	64389104
chr15	64443719	64444245
chr15	64444430	64444887
chr15	64453505	64453785
chr15	64454716	64454975
chr15	64455614	64456024
chr15	64479519	64479736
chr15	64480133	64480317
chr15	64538910	64539394
chr15	64540452	64540548
chr15	64542521	64542877
chr15	64619021	64619231
chr15	64647570	64648307
chr15	64648578	64648982
chr15	64653690	64653811
chr15	64670698	64671087
chr15	64673002	64673564
chr15	64673904	64674025
chr15	64679675	64679843
chr15	64679969	64680317
chr15	64703805	64704184
chr15	64704469	64704575
chr15	64704724	64704791
chr15	64736131	64736908
chr15	64752304	64753416
chr15	64753571	64754173
chr15	64759764	64759969
chr15	64794998	64795227
chr15	64813992	64814095
chr15	64828320	64828797
chr15	64842647	64842988
chr15	64863931	64864438
chr15	64866387	64866764
chr15	64891962	64892127
chr15	64907728	64907957
chr15	64911363	64911417
chr15	64928079	64928201
chr15	64944334	64944412

chr15	64985277	64985506
chr15	64992557	64992577
chr15	64995022	64995836
chr15	65059279	65059532
chr15	65060641	65060856
chr15	65067071	65067742
chr15	65067946	65068320
chr15	65115433	65116972
chr15	65117281	65117329
chr15	65117505	65117795
chr15	65117992	65118706
chr15	65121218	65121392
chr15	65121508	65121569
chr15	65127783	65127879
chr15	65133304	65133929
chr15	65134050	65135414
chr15	65135705	65135807
chr15	65136000	65136288
chr15	65138679	65138878
chr15	65139574	65139684
chr15	65139789	65139989
chr15	65140071	65140203
chr15	65142743	65142942
chr15	65143255	65143718
chr15	65144114	65144386
chr15	65144947	65145106
chr15	65148052	65148239
chr15	65148882	65149793
chr15	65149941	65150497
chr15	65165481	65166183
chr15	65174375	65175205
chr15	65175890	65176177
chr15	65176605	65176657
chr15	65184555	65184619
chr15	65185387	65185447
chr15	65185526	65186846
chr15	65186956	65187220
chr15	65187416	65187626
chr15	65187855	65188115
chr15	65189301	65189552
chr15	65203690	65204310
chr15	65274362	65274827
chr15	65281094	65282676
chr15	65321325	65322204
chr15	65325662	65325900
chr15	65325991	65326074

chr15	65336736	65337111
chr15	65424826	65426683
chr15	65470250	65470354
chr15	65470579	65470686
chr15	65471088	65471107
chr15	65476687	65478190
chr15	65482425	65482560
chr15	65539063	65539124
chr15	65579043	65579706
chr15	65584396	65584580
chr15	65587994	65589165
chr15	65590032	65590132
chr15	65590238	65590450
chr15	65595871	65596484
chr15	65596666	65597544
chr15	65597729	65597748
chr15	65669572	65670345
chr15	65677409	65677712
chr15	65713611	65713830
chr15	65714221	65714420
chr15	65792006	65792041
chr15	65809461	65810068
chr15	65822512	65823434
chr15	65825031	65825374
chr15	65894253	65894692
chr15	65902842	65903457
chr15	65903715	65904027
chr15	65904349	65904492
chr15	65940522	65940603
chr15	65947306	65947353
chr15	65958875	65958951
chr15	66025078	66025400
chr15	66079895	66080486
chr15	66083381	66083629
chr15	66083792	66084442
chr15	66084574	66085251
chr15	66088895	66088971
chr15	66089185	66089448
chr15	66116435	66116698
chr15	66117726	66117906
chr15	66133432	66133555
chr15	66133675	66133941
chr15	66148972	66149090
chr15	66149221	66149357
chr15	66161415	66161709
chr15	66161948	66162465

chr15	66162615	66162842
chr15	66163264	66163627
chr15	66545450	66545656
chr15	66545943	66546167
chr15	66585626	66586630
chr15	66586980	66587199
chr15	66648417	66649536
chr15	66651828	66652308
chr15	66652427	66652620
chr15	66654397	66654803
chr15	66656888	66657167
chr15	66662422	66662689
chr15	66665216	66665620
chr15	66665743	66665809
chr15	66678031	66678230
chr15	66678806	66678838
chr15	66678955	66680520
chr15	66752001	66753152
chr15	66753549	66753945
chr15	66754018	66754803
chr15	66771500	66771541
chr15	66772502	66772975
chr15	66773267	66773360
chr15	66789530	66790063
chr15	66790319	66790691
chr15	66796782	66798084
chr15	66844723	66844986
chr15	66870673	66870790
chr15	66939718	66939917
chr15	66947306	66947608
chr15	66963777	66963982
chr15	66992555	66992754
chr15	66994138	66994473
chr15	66994684	66994927
chr15	66995695	66996014
chr15	67094551	67095138
chr15	67095421	67095583
chr15	67125251	67125254
chr15	67134444	67134643
chr15	67175512	67175794
chr15	67176123	67176400
chr15	67223453	67223755
chr15	67224078	67224187
chr15	67224540	67224959
chr15	67239352	67239417
chr15	67248416	67248490

chr15	67248917	67249306
chr15	67316130	67316771
chr15	67317136	67317170
chr15	67317328	67317576
chr15	67317973	67318441
chr15	67318807	67319024
chr15	67319950	67320202
chr15	67320360	67320622
chr15	67331873	67332074
chr15	67332334	67332440
chr15	67333180	67333379
chr15	67333664	67334030
chr15	67334161	67334860
chr15	67336317	67336986
chr15	67337106	67337172
chr15	67339659	67339848
chr15	67340904	67341884
chr15	67341990	67342482
chr15	67342730	67342840
chr15	67357501	67359181
chr15	67359462	67360170
chr15	67366650	67367245
chr15	67368566	67368581
chr15	67368864	67368907
chr15	67383435	67383501
chr15	67383868	67383967
chr15	67386452	67386515
chr15	67386600	67386743
chr15	67387338	67387394
chr15	67397507	67398382
chr15	67398525	67399014
chr15	67399088	67400247
chr15	67409246	67409355
chr15	67411166	67411602
chr15	67411797	67411817
chr15	67412578	67413118
chr15	67413209	67413428
chr15	67413545	67413793
chr15	67414240	67414644
chr15	67415399	67415598
chr15	67416218	67416745
chr15	67417180	67418755
chr15	67418943	67420089
chr15	67420260	67420660
chr15	67435417	67435467
chr15	67438588	67440191

chr15	67440389	67440798
chr15	67441327	67441422
chr15	67442588	67443195
chr15	67455541	67455617
chr15	67466319	67468433
chr15	67517142	67518008
chr15	67532629	67532826
chr15	67532944	67532946
chr15	67535276	67535380
chr15	67545338	67545718
chr15	67546372	67546851
chr15	67547206	67547541
chr15	67757880	67757946
chr15	67812804	67813246
chr15	67813485	67814139
chr15	67831611	67832096
chr15	67834731	67835510
chr15	67835607	67835869
chr15	68038817	68039146
chr15	68127898	68128255
chr15	68131896	68132269
chr15	68132460	68132629
chr15	68132771	68133076
chr15	68133326	68133500
chr15	68156697	68156919
chr15	68176634	68176887
chr15	68177045	68177577
chr15	68234373	68234395
chr15	68290361	68290811
chr15	68291178	68291359
chr15	68291509	68291519
chr15	68346146	68346291
chr15	68346697	68347464
chr15	68347608	68348001
chr15	68418098	68418358
chr15	68418775	68419086
chr15	68431676	68431700
chr15	68501853	68502105
chr15	68520930	68521284
chr15	68521347	68522557
chr15	68545511	68545619
chr15	68548956	68549527
chr15	68549696	68549849
chr15	68551100	68551398
chr15	68553600	68553732
chr15	68560842	68561130

chr15	68563221	68563695
chr15	68564108	68565899
chr15	68567516	68567782
chr15	68567999	68568055
chr15	68569437	68569956
chr15	68570067	68571262
chr15	68572827	68573082
chr15	68573872	68574210
chr15	68577663	68577908
chr15	68591150	68591468
chr15	68598181	68598689
chr15	68635834	68636074
chr15	68644709	68644869
chr15	68645038	68645324
chr15	68850810	68851643
chr15	68851748	68852036
chr15	68852484	68852937
chr15	68869798	68869852
chr15	68870016	68870135
chr15	68870806	68871426
chr15	68878173	68878594
chr15	68931340	68931677
chr15	68932229	68932781
chr15	68933177	68935124
chr15	68935615	68936005
chr15	68966880	68967834
chr15	69109679	69110227
chr15	69110356	69110898
chr15	69111280	69112374
chr15	69112591	69113968
chr15	69114035	69114623
chr15	69168111	69168251
chr15	69177780	69177847
chr15	69222177	69222712
chr15	69222872	69223173
chr15	69284481	69284491
chr15	69324716	69324906
chr15	69336190	69336327
chr15	69337237	69337354
chr15	69337510	69337852
chr15	69366585	69366970
chr15	69452326	69452678
chr15	69452970	69453372
chr15	69453705	69453924
chr15	69529155	69529206
chr15	69591152	69591662

chr15	69683434	69683527
chr15	69683946	69684131
chr15	69686687	69686886
chr15	69706158	69706283
chr15	69706663	69707247
chr15	69707471	69707568
chr15	69744186	69745929
chr15	69754415	69755156
chr15	69755263	69755802
chr15	69814660	69814833
chr15	69815084	69815088
chr15	69815763	69815815
chr15	69823690	69823936
chr15	69849517	69849662
chr15	69850017	69850207
chr15	69857516	69858235
chr15	69858364	69858748
chr15	69860018	69860171
chr15	69860577	69860946
chr15	69861319	69861394
chr15	70315849	70316358
chr15	70316476	70316719
chr15	70387764	70390267
chr15	70390970	70391829
chr15	70392321	70392368
chr15	70392643	70392811
chr15	70392945	70393253
chr15	70404027	70404265
chr15	70487813	70488925
chr15	70489173	70489729
chr15	70489866	70489896
chr15	70490739	70491012
chr15	70538538	70538845
chr15	70560832	70561246
chr15	70564179	70564302
chr15	70616599	70616619
chr15	70616877	70617045
chr15	70642004	70642245
chr15	70668277	70668320
chr15	70669093	70669355
chr15	70719125	70719215
chr15	70722366	70722418
chr15	70722732	70722859
chr15	70730772	70730994
chr15	70767632	70767831
chr15	70785377	70785940

chr15	70786273	70786456
chr15	70786524	70786691
chr15	70787651	70788051
chr15	70796716	70796994
chr15	70798369	70798464
chr15	70799684	70799946
chr15	70800044	70800166
chr15	70801300	70801499
chr15	70819239	70819404
chr15	70862572	70862730
chr15	71006962	71007054
chr15	71033317	71033421
chr15	71033639	71033746
chr15	71034478	71034510
chr15	71054762	71055805
chr15	71056199	71056424
chr15	71071011	71071286
chr15	71073536	71073711
chr15	71073910	71074023
chr15	71087618	71087793
chr15	71088016	71088060
chr15	71104186	71104501
chr15	71118934	71119012
chr15	71119222	71119382
chr15	71126126	71127021
chr15	71131872	71131892
chr15	71144834	71145069
chr15	71145295	71145495
chr15	71145661	71146879
chr15	71147081	71147340
chr15	71184179	71184755
chr15	71184992	71185481
chr15	71194462	71194528
chr15	71194680	71195069
chr15	71304107	71304144
chr15	71335692	71335814
chr15	71336385	71336591
chr15	71335970	71336138
chr15	71343045	71343263
chr15	71385569	71386205
chr15	71406877	71407040
chr15	71407345	71407810
chr15	71408023	71409074
chr15	71424025	71424257
chr15	71491726	71492170
chr15	71529893	71529967

chr15	71530322	71530350
chr15	71567388	71567742
chr15	71568596	71568717
chr15	71568851	71568956
chr15	71571324	71571634
chr15	71571751	71572061
chr15	71586397	71586814
chr15	71587189	71589017
chr15	71622041	71622205
chr15	71629429	71630154
chr15	71647666	71648115
chr15	71656388	71656526
chr15	71656678	71656821
chr15	71737188	71737684
chr15	71737833	71738007
chr15	71739529	71740392
chr15	71740954	71741067
chr15	71741200	71741280
chr15	71763434	71764393
chr15	71825516	71825641
chr15	71830640	71830882
chr15	71902114	71902366
chr15	72039817	72039918
chr15	72072771	72073120
chr15	72334505	72334761
chr15	72409006	72409652
chr15	72409818	72410565
chr15	72410680	72411181
chr15	72447466	72447714
chr15	72447871	72448500
chr15	72448683	72448806
chr15	72450870	72451006
chr15	72459470	72460074
chr15	72460279	72462281
chr15	72462608	72462942
chr15	72464239	72464882
chr15	72467151	72467354
chr15	72470377	72470727
chr15	72470826	72471164
chr15	72471914	72472069
chr15	72472780	72473092
chr15	72474528	72474811
chr15	72474926	72475460
chr15	72476313	72476950
chr15	72477123	72477201
chr15	72479888	72480178

chr15	72485712	72485925
chr15	72487290	72487350
chr15	72487892	72487930
chr15	72488387	72488900
chr15	72489171	72490159
chr15	72490276	72490897
chr15	72494310	72494324
chr15	72494813	72494989
chr15	72495613	72495775
chr15	72495984	72496341
chr15	72496751	72497023
chr15	72514738	72514881
chr15	72515049	72515380
chr15	72518550	72520020
chr15	72520272	72522015
chr15	72522231	72523509
chr15	72523716	72524869
chr15	72529718	72530187
chr15	72530306	72530560
chr15	72564300	72564911
chr15	72565193	72565684
chr15	72604898	72605030
chr15	72632407	72632420
chr15	72667863	72668237
chr15	72668405	72668865
chr15	72754356	72754796
chr15	72765884	72766506
chr15	72766681	72767816
chr15	72778510	72778599
chr15	72781357	72781552
chr15	72847004	72847214
chr15	72929004	72929263
chr15	72929457	72929811
chr15	72978128	72979169
chr15	73075413	73076070
chr15	73076300	73077021
chr15	73161892	73162018
chr15	73284018	73284448
chr15	73290935	73290964
chr15	73291036	73291145
chr15	73344033	73344543
chr15	73344828	73345051
chr15	73345543	73345775
chr15	73660880	73661079
chr15	73892287	73892650
chr15	73893958	73894032

chr15	73894107	73894522
chr15	73894713	73894979
chr15	73921817	73921971
chr15	73924729	73925713
chr15	73925967	73926253
chr15	73926387	73926646
chr15	73926811	73927083
chr15	73929064	73929237
chr15	73939581	73939645
chr15	73943127	73943272
chr15	73976017	73976369
chr15	73976538	73977811
chr15	73989030	73989265
chr15	74044776	74045276
chr15	74068178	74068276
chr15	74070276	74070462
chr15	74219282	74220310
chr15	74265175	74265417
chr15	74274104	74275991
chr15	74276188	74276625
chr15	74280143	74280411
chr15	74280743	74281135
chr15	74281480	74281773
chr15	74283804	74284544
chr15	74284817	74284950
chr15	74287027	74287899
chr15	74288126	74288327
chr15	74289325	74289756
chr15	74309230	74309399
chr15	74392197	74393054
chr15	74658296	74658765
chr15	74666240	74666669
chr15	74667237	74667436
chr15	74670823	74671390
chr15	74672071	74672276
chr15	74674543	74675046
chr15	74676398	74676649
chr15	74677050	74677306
chr15	74677611	74678553
chr15	74679284	74679483
chr15	74680302	74680733
chr15	74682532	74682660
chr15	74687132	74687243
chr15	74688595	74689366
chr15	74689484	74690421
chr15	74690527	74690629

chr15	74691026	74691044
chr15	74691369	74691633
chr15	74692825	74693095
chr15	74693186	74693262
chr15	74693400	74693599
chr15	74695439	74695638
chr15	74699179	74699879
chr15	74709269	74709612
chr15	74712576	74713084
chr15	74713965	74714369
chr15	74714581	74715252
chr15	74715555	74716619
chr15	74724683	74724882
chr15	74725001	74726345
chr15	74748245	74748385
chr15	74753141	74753360
chr15	74753682	74754149
chr15	74774006	74774037
chr15	74775203	74775352
chr15	74790296	74790339
chr15	74818305	74818450
chr15	74818653	74818767
chr15	74822114	74822636
chr15	74827993	74828317
chr15	74828528	74828596
chr15	74832870	74833356
chr15	74833546	74833920
chr15	74834093	74834288
chr15	74834418	74834564
chr15	74834996	74835223
chr15	74850297	74850434
chr15	74880618	74880903
chr15	74881043	74881858
chr15	74890316	74890594
chr15	74890826	74891342
chr15	74906457	74909015
chr15	74909681	74910203
chr15	74966481	74966681
chr15	74972011	74972086
chr15	74972227	74972252
chr15	74987918	74989080
chr15	75009376	75009487
chr15	75017359	75017558
chr15	75018284	75019120
chr15	75019376	75019563
chr15	75024255	75024538

chr15	75024961	75025234
chr15	75027077	75027235
chr15	75053695	75053796
chr15	75055347	75055859
chr15	75056635	75057111
chr15	75056228	75056482
chr15	75061137	75061355
chr15	75063629	75064065
chr15	75073584	75074143
chr15	75074412	75076030
chr15	75076319	75076605
chr15	75078353	75078552
chr15	75087178	75087635
chr15	75088292	75088499
chr15	75088681	75088999
chr15	75089337	75089937
chr15	75091602	75092119
chr15	75092351	75093115
chr15	75099304	75099349
chr15	75099865	75100129
chr15	75100219	75101340
chr15	75101588	75102581
chr15	75102919	75103322
chr15	75110053	75110193
chr15	75110395	75110429
chr15	75110558	75111044
chr15	75134303	75136083
chr15	75142205	75142415
chr15	75151916	75152306
chr15	75155410	75156259
chr15	75157265	75157518
chr15	75157622	75157779
chr15	75157895	75157976
chr15	75158413	75159511
chr15	75160593	75160649
chr15	75162422	75163237
chr15	75163730	75163915
chr15	75164027	75164203
chr15	75165162	75165596
chr15	75165888	75166586
chr15	75182348	75183059
chr15	75184273	75184579
chr15	75193850	75193915
chr15	75198072	75199770
chr15	75229772	75231315
chr15	75239697	75239876

chr15	75241709	75241972
chr15	75242498	75242855
chr15	75244386	75244862
chr15	75245525	75245724
chr15	75246862	75246993
chr15	75247283	75247482
chr15	75247740	75250884
chr15	75287971	75288505
chr15	75315799	75316525
chr15	75321989	75322188
chr15	75322294	75322815
chr15	75323857	75324055
chr15	75328726	75329102
chr15	75333532	75333756
chr15	75334845	75334955
chr15	75339145	75339367
chr15	75339701	75340093
chr15	75382639	75383041
chr15	75396936	75397318
chr15	75399491	75400088
chr15	75400345	75400738
chr15	75401023	75401252
chr15	75404002	75404005
chr15	75454714	75454780
chr15	75467908	75468035
chr15	75468163	75468343
chr15	75493186	75493590
chr15	75493770	75496263
chr15	75496412	75496611
chr15	75496741	75497215
chr15	75591496	75591739
chr15	75628084	75628813
chr15	75629066	75629156
chr15	75639581	75641202
chr15	75659893	75660132
chr15	75660416	75661407
chr15	75661659	75661696
chr15	75664151	75664480
chr15	75729398	75729597
chr15	75735743	75736200
chr15	75740644	75740770
chr15	75742516	75742657
chr15	75742774	75742973
chr15	75743377	75744034
chr15	75744321	75746233
chr15	75749049	75749316

chr15	75746800	75748935
chr15	75749806	75750014
chr15	75808836	75809314
chr15	75821302	75821379
chr15	75825556	75825810
chr15	75865398	75865643
chr15	75867223	75867593
chr15	75870294	75872067
chr15	75912759	75913491
chr15	75917053	75918708
chr15	75919945	75920629
chr15	75930468	75930873
chr15	75930980	75931265
chr15	75931492	75931776
chr15	75932028	75932507
chr15	75932632	75933845
chr15	75933915	75934308
chr15	75934559	75934560
chr15	75939176	75941250
chr15	75942814	75943413
chr15	75944524	75945096
chr15	75943619	75944286
chr15	75948306	75948700
chr15	75953571	75954418
chr15	75956395	75957052
chr15	75957525	75960742
chr15	75961005	75962581
chr15	75962932	75963131
chr15	75965850	75966113
chr15	75971276	75972492
chr15	75987027	75987486
chr15	75988602	75988876
chr15	75989222	75989432
chr15	75995746	75995970
chr15	75997384	75997629
chr15	76004817	76005148
chr15	76010595	76010697
chr15	76016516	76016758
chr15	76024240	76024480
chr15	76030200	76031017
chr15	76050575	76051502
chr15	76120269	76120483
chr15	76129498	76129664
chr15	76134914	76137012
chr15	76195691	76197118
chr15	76197203	76197402

chr15	76214113	76214366
chr15	76214513	76215170
chr15	76239385	76239498
chr15	76239635	76239945
chr15	76349272	76349369
chr15	76349542	76349680
chr15	76351420	76352557
chr15	76365621	76366363
chr15	76430701	76431191
chr15	76442772	76443140
chr15	76443546	76444308
chr15	76448443	76448943
chr15	76452899	76453098
chr15	76453311	76453863
chr15	76455373	76455580
chr15	76467961	76468167
chr15	76470244	76470941
chr15	76471051	76471390
chr15	76475048	76475165
chr15	76481384	76481465
chr15	76481762	76481870
chr15	76483356	76483520
chr15	76483956	76484439
chr15	76484825	76485036
chr15	76485375	76485548
chr15	76488746	76489022
chr15	76489228	76489427
chr15	76489657	76489902
chr15	76602393	76602526
chr15	76603024	76604327
chr15	76604916	76605861
chr15	76627420	76627539
chr15	76628163	76628816
chr15	76629170	76629282
chr15	76629816	76630571
chr15	76630754	76631064
chr15	76632606	76633104
chr15	76633257	76633456
chr15	76633934	76634133
chr15	76634752	76635426
chr15	76638094	76638308
chr15	76638475	76638970
chr15	76639558	76639816
chr15	76693923	76694011
chr15	76725614	76725828
chr15	77122590	77122646

chr15	77196722	77196892
chr15	77197251	77198259
chr15	77223669	77223888
chr15	77224054	77224529
chr15	77285853	77285994
chr15	77287218	77287795
chr15	77290206	77290405
chr15	77353608	77353961
chr15	77356784	77356820
chr15	77362641	77362662
chr15	77362825	77363511
chr15	77711797	77712470
chr15	77712793	77712910
chr15	77713140	77713610
chr15	77778902	77778951
chr15	77779128	77779213
chr15	77789059	77789202
chr15	77835537	77835736
chr15	77835972	77836306
chr15	78180878	78181149
chr15	78236761	78236997
chr15	78286146	78286426
chr15	78286650	78286999
chr15	78287157	78287498
chr15	78300119	78301228
chr15	78304949	78305004
chr15	78312170	78312187
chr15	78339441	78339631
chr15	78348757	78349104
chr15	78354551	78354636
chr15	78357701	78357785
chr15	78367599	78368898
chr15	78369082	78369932
chr15	78378655	78378812
chr15	78423508	78424453
chr15	78426035	78426285
chr15	78426479	78426618
chr15	78427372	78427628
chr15	78439000	78439104
chr15	78439247	78439696
chr15	78440129	78440364
chr15	78441087	78441488
chr15	78441701	78442614
chr15	78469796	78469995
chr15	78512050	78512360
chr15	78533494	78533693

chr15	78556543	78557828
chr15	78591314	78591864
chr15	78592068	78592306
chr15	78615001	78615101
chr15	78667789	78668045
chr15	78729807	78730275
chr15	78730515	78731204
chr15	78731267	78731605
chr15	78799214	78799490
chr15	78799939	78800368
chr15	78832405	78833831
chr15	78857826	78858311
chr15	78858464	78858864
chr15	78932860	78933852
chr15	79007575	79007867
chr15	79048977	79049471
chr15	79050055	79050254
chr15	79102857	79103817
chr15	79103998	79104254
chr15	79152211	79152623
chr15	79153117	79153135
chr15	79164923	79166225
chr15	79197899	79198234
chr15	79198513	79198764
chr15	79198911	79198969
chr15	79209035	79209082
chr15	79222346	79222989
chr15	79236764	79237407
chr15	79237645	79237848
chr15	79243906	79244035
chr15	79280602	79281402
chr15	79285237	79285682
chr15	79294904	79295114
chr15	79295423	79296161
chr15	79296502	79296849
chr15	79297147	79297346
chr15	79297526	79297591
chr15	79297661	79297803
chr15	79603037	79603343
chr15	79603517	79604232
chr15	79604300	79604461
chr15	79607174	79607360
chr15	79608301	79608417
chr15	80185477	80185742
chr15	80188524	80189925
chr15	80191234	80191482

chr15	80215276	80216627
chr15	80303518	80303741
chr15	80351923	80352753
chr15	80352862	80353246
chr15	80363240	80363456
chr15	80364562	80365057
chr15	80404459	80404523
chr15	80445097	80445829
chr15	80452591	80453474
chr15	80458497	80458716
chr15	80471832	80472149
chr15	80495007	80495143
chr15	80495355	80495621
chr15	80504627	80504736
chr15	80777731	80777809
chr15	80778006	80778140
chr15	80781391	80781802
chr15	80786976	80786992
chr15	80842582	80842644
chr15	80860327	80860413
chr15	80860656	80861445
chr15	80872332	80872697
chr15	80872887	80873645
chr15	80874180	80874964
chr15	80875720	80875787
chr15	80877830	80878003
chr15	80878180	80878558
chr15	80879345	80879544
chr15	80880433	80880712
chr15	80881073	80881328
chr15	80896965	80897127
chr15	80897245	80897510
chr15	80900772	80901639
chr15	80901876	80902091
chr15	80908283	80908847
chr15	80954978	80955154
chr15	80955364	80955853
chr15	80971996	80972187
chr15	80983956	80984191
chr15	80986671	80986899
chr15	80987029	80987402
chr15	80987617	80989152
chr15	81001772	81001935
chr15	81002159	81002304
chr15	81006236	81006435
chr15	81069684	81069938

chr15	81070300	81070425
chr15	81070792	81071192
chr15	81071509	81071933
chr15	81247320	81247441
chr15	81247781	81248635
chr15	81273224	81273259
chr15	81277131	81277308
chr15	81281556	81282190
chr15	81282368	81282582
chr15	81291943	81291987
chr15	81292335	81294750
chr15	81294933	81295159
chr15	81384453	81384616
chr15	81384750	81385043
chr15	81398064	81398204
chr15	81399104	81399349
chr15	81403354	81404285
chr15	81406325	81406683
chr15	81408819	81409220
chr15	81447906	81448247
chr15	81449486	81449552
chr15	81449885	81450193
chr15	81450312	81450435
chr15	81481870	81482544
chr15	81589163	81589670
chr15	81589916	81590498
chr15	81596527	81596697
chr15	81605107	81605369
chr15	81605712	81606407
chr15	81607190	81607295
chr15	81609307	81609645
chr15	81614635	81614710
chr15	81615274	81615450
chr15	81615861	81616601
chr15	81616765	81617191
chr15	81628950	81629091
chr15	82337910	82338365
chr15	82339191	82339444
chr15	82397552	82397730
chr15	82397996	82398195
chr15	82415413	82415541
chr15	82554796	82554995
chr15	82560749	82560766
chr15	82561198	82561315
chr15	82646823	82647115
chr15	82647649	82648031

chr15	82738265	82738507
chr15	82797591	82797841
chr15	82797912	82798100
chr15	82824127	82824613
chr15	82825039	82825361
chr15	82825641	82825840
chr15	82945217	82945219
chr15	82945320	82945619
chr15	83007222	83007486
chr15	83023320	83023599
chr15	83024049	83024551
chr15	83042323	83042336
chr15	83182143	83182738
chr15	83208798	83208956
chr15	83316747	83316946
chr15	83348923	83349167
chr15	83349757	83349957
chr15	83377895	83378164
chr15	83378731	83379077
chr15	83410900	83411214
chr15	83418858	83419897
chr15	83477620	83477728
chr15	83477995	83478187
chr15	83478407	83479556
chr15	83479774	83480023
chr15	83496782	83496798
chr15	83501150	83501268
chr15	83501493	83502033
chr15	83620931	83621413
chr15	83621522	83621859
chr15	83654475	83655388
chr15	83679612	83679734
chr15	83679857	83680386
chr15	83680504	83681017
chr15	83734810	83735194
chr15	83735296	83736093
chr15	83736346	83736503
chr15	83775802	83776690
chr15	83865544	83865679
chr15	83875382	83876610
chr15	83876884	83877095
chr15	83944218	83944361
chr15	83946968	83947306
chr15	83952245	83953368
chr15	83951480	83952057
chr15	83953475	83954188

chr15	83954314	83955352
chr15	83956279	83956918
chr15	83956995	83957517
chr15	83957694	83958111
chr15	83973828	83974374
chr15	83974504	83974735
chr15	83975027	83975153
chr15	83979372	83979634
chr15	83979791	83980204
chr15	83980595	83980973
chr15	83989137	83989420
chr15	83990768	83991319
chr15	83992092	83992659
chr15	84026147	84026317
chr15	84034612	84034631
chr15	84046737	84046918
chr15	84047267	84048430
chr15	84048565	84048938
chr15	84049074	84049949
chr15	84115543	84116907
chr15	84415836	84416200
chr15	84513785	84513847
chr15	84517341	84517453
chr15	84737364	84737393
chr15	84737683	84737870
chr15	84841157	84841356
chr15	84841507	84842342
chr15	84842562	84842624
chr15	84863835	84864098
chr15	84898106	84898843
chr15	84899103	84899427
chr15	84975974	84976463
chr15	84976795	84977017
chr15	84981734	84981933
chr15	85042984	85044144
chr15	85044276	85044555
chr15	85113105	85114654
chr15	85122995	85123088
chr15	85144306	85144505
chr15	85173717	85174462
chr15	85174720	85175128
chr15	85176016	85176167
chr15	85176309	85176378
chr15	85176667	85177103
chr15	85177310	85177540
chr15	85194240	85194658

chr15	85197001	85197567
chr15	85197747	85198075
chr15	85200799	85202199
chr15	85228962	85229280
chr15	85236627	85237106
chr15	85258588	85260470
chr15	85266086	85266244
chr15	85268095	85268107
chr15	85268402	85268441
chr15	85278023	85278486
chr15	85278695	85279167
chr15	85290507	85292451
chr15	85386335	85386400
chr15	85402175	85402395
chr15	85468018	85468170
chr15	85523203	85523272
chr15	85523404	85524440
chr15	85524686	85526204
chr15	85720150	85720379
chr15	85720815	85720892
chr15	85777159	85777946
chr15	85796816	85797015
chr15	85820111	85820227
chr15	85840115	85840326
chr15	85886724	85887198
chr15	85887500	85887806
chr15	85888027	85888226
chr15	85888362	85888680
chr15	85889960	85890076
chr15	85892393	85892413
chr15	85892543	85892694
chr15	85900815	85901021
chr15	85901147	85901349
chr15	85901614	85901645
chr15	85922845	85923036
chr15	85923127	85923556
chr15	85923876	85924698
chr15	85951227	85951516
chr15	85956837	85957111
chr15	85977109	85977227
chr15	85987395	85987445
chr15	86000846	86000958
chr15	86002004	86002075
chr15	86002216	86002882
chr15	86131299	86131383
chr15	86162131	86162409

chr15	86337585	86338124
chr15	86338442	86338593
chr15	86353659	86353686
chr15	86879177	86879212
chr15	86879597	86880185
chr15	86890478	86890649
chr15	86890826	86890883
chr15	86916629	86916812
chr15	89009384	89009411
chr15	89010024	89010307
chr15	89010456	89011166
chr15	89013114	89013376
chr15	89089388	89089505
chr15	89090083	89090224
chr15	89157598	89157757
chr15	89164055	89164347
chr15	89164560	89164909
chr15	89165385	89165577
chr15	89166285	89166442
chr15	89166724	89166944
chr15	89167831	89168034
chr15	89173286	89173651
chr15	89173859	89174058
chr15	89179243	89180286
chr15	89181419	89182991
chr15	89184919	89185112
chr15	89248697	89248948
chr15	89438262	89438807
chr15	89444054	89444348
chr15	89454630	89455113
chr15	89455310	89455479
chr15	89455544	89456021
chr15	89456267	89457328
chr15	89459302	89459683
chr15	89461267	89461330
chr15	89461900	89461999
chr15	89462157	89462442
chr15	89463761	89464494
chr15	89559808	89560007
chr15	89630744	89631348
chr15	89631707	89632152
chr15	89633957	89633999
chr15	89634226	89634422
chr15	89644468	89645109
chr15	89645450	89645581
chr15	89647646	89647823

chr15	89648242	89648461
chr15	89648630	89648835
chr15	89662879	89663157
chr15	89663512	89663699
chr15	89664633	89664651
chr15	89664923	89665144
chr15	89666291	89666772
chr15	89710535	89711102
chr15	89786443	89786647
chr15	89786848	89788082
chr15	89876802	89877153
chr15	89877393	89878091
chr15	89878426	89878775
chr15	89902045	89902323
chr15	89902450	89902649
chr15	89910578	89910811
chr15	89910974	89911613
chr15	89911681	89911744
chr15	89912416	89912811
chr15	89913201	89913394
chr15	89913928	89914079
chr15	89914194	89914200
chr15	89921365	89923548
chr15	89923656	89923889
chr15	89938019	89939841
chr15	89942713	89943772
chr15	89945335	89945370
chr15	90039572	90040187
chr15	90041158	90041535
chr15	90042114	90042317
chr15	90056394	90056976
chr15	90057442	90057743
chr15	90057948	90058852
chr15	90118170	90118319
chr15	90118765	90119402
chr15	90120169	90120449
chr15	90197169	90197499
chr15	90197723	90199006
chr15	90206027	90206390
chr15	90206593	90206825
chr15	90208684	90209263
chr15	90233356	90233976
chr15	90283519	90283777
chr15	90293241	90294686
chr15	90316252	90316620
chr15	90316694	90316845

chr15	90317984	90318091
chr15	90319275	90320211
chr15	90323388	90323543
chr15	90323782	90323836
chr15	90324044	90324786
chr15	90326429	90326589
chr15	90324882	90325856
chr15	90327903	90328118
chr15	90328426	90328681
chr15	90355818	90355865
chr15	90356107	90356150
chr15	90356679	90356878
chr15	90357657	90358455
chr15	90364129	90364482
chr15	90364706	90364938
chr15	90365130	90365267
chr15	90375339	90375544
chr15	90377883	90377885
chr15	90379592	90380449
chr15	90380647	90380800
chr15	90387678	90387920
chr15	90388684	90388741
chr15	90388862	90389196
chr15	90391365	90392206
chr15	90418194	90418213
chr15	90436803	90437705
chr15	90455310	90456333
chr15	90458655	90459007
chr15	90460720	90461336
chr15	90466201	90466211
chr15	90501210	90501358
chr15	90501714	90501767
chr15	90542855	90544237
chr15	90544450	90544741
chr15	90544959	90546284
chr15	90547173	90547356
chr15	90547436	90547480
chr15	90547625	90548830
chr15	90558813	90559409
chr15	90559898	90560282
chr15	90563039	90563197
chr15	90574312	90574384
chr15	90576577	90577003
chr15	90577348	90578745
chr15	90578882	90579158
chr15	90581045	90581244

chr15	90581844	90581979
chr15	90582135	90582441
chr15	90584332	90584659
chr15	90585666	90585688
chr15	90590739	90590924
chr15	90602718	90603029
chr15	90603423	90603576
chr15	90605004	90606201
chr15	90606441	90606658
chr15	90613773	90613986
chr15	90614806	90614859
chr15	90618299	90618839
chr15	90619210	90619935
chr15	90630825	90631125
chr15	90645175	90645737
chr15	90646050	90646304
chr15	90648155	90648375
chr15	90649631	90649945
chr15	90650330	90650421
chr15	90701080	90701262
chr15	90701484	90701859
chr15	90704761	90705017
chr15	90705192	90705730
chr15	90708756	90708953
chr15	90709279	90709661
chr15	90711565	90711862
chr15	90720807	90720828
chr15	90722154	90722160
chr15	90727143	90728931
chr15	90729188	90729496
chr15	90730050	90730112
chr15	90731159	90731409
chr15	90731750	90732237
chr15	90732368	90732628
chr15	90733589	90734344
chr15	90734482	90735363
chr15	90736445	90736510
chr15	90735490	90735779
chr15	90744142	90745615
chr15	90745765	90745866
chr15	90752690	90752762
chr15	90753992	90755162
chr15	90755310	90756881
chr15	90757149	90757190
chr15	90757255	90757481
chr15	90757781	90758098

chr15	90758831	90758925
chr15	90763235	90763437
chr15	90776230	90777145
chr15	90777515	90777874
chr15	90792360	90792616
chr15	90792999	90793163
chr15	90804817	90805163
chr15	90805331	90805527
chr15	90807809	90809807
chr15	90836456	90836661
chr15	90858517	90858555
chr15	90891949	90892148
chr15	90895215	90895266
chr15	90895556	90895829
chr15	90896211	90896321
chr15	90905252	90905685
chr15	90907512	90907623
chr15	90907726	90907821
chr15	90930842	90931202
chr15	90932477	90932806
chr15	90931515	90932225
chr15	90943644	90944258
chr15	90944470	90944531
chr15	90945466	90945926
chr15	90955851	90956217
chr15	90956350	90956493
chr15	90963174	90963293
chr15	90985785	90986287
chr15	91046036	91046131
chr15	91062891	91063057
chr15	91072721	91073417
chr15	91073737	91074006
chr15	91094380	91094504
chr15	91135495	91135594
chr15	91190445	91190696
chr15	91191359	91191783
chr15	91208062	91209329
chr15	91236935	91236982
chr15	91260195	91260271
chr15	91260643	91260891
chr15	91261156	91261184
chr15	91265265	91265403
chr15	91318619	91318768
chr15	91382204	91382222
chr15	91382425	91382655
chr15	91382881	91383271

chr15	91383459	91383744
chr15	91388067	91388490
chr15	91407191	91407478
chr15	91408119	91408195
chr15	91411176	91411445
chr15	91413212	91415695
chr15	91415809	91416423
chr15	91445632	91445945
chr15	91446073	91446450
chr15	91446632	91446851
chr15	91473296	91473988
chr15	91475132	91475763
chr15	91475907	91476106
chr15	91477883	91478285
chr15	91478494	91479280
chr15	91479543	91479708
chr15	91497454	91497474
chr15	91497740	91497849
chr15	91498048	91499204
chr15	91499366	91501262
chr15	91501508	91501581
chr15	91510648	91510825
chr15	91515116	91515367
chr15	91536217	91536667
chr15	91537121	91537782
chr15	91538120	91538532
chr15	91564710	91566129
chr15	91593318	91593517
chr15	92395754	92396145
chr15	92396304	92398433
chr15	92399924	92400088
chr15	92409208	92410662
chr15	92410769	92410932
chr15	92411090	92411358
chr15	92416010	92416042
chr15	92433827	92433942
chr15	92438162	92438312
chr15	92484286	92484571
chr15	92485738	92486301
chr15	92486456	92486787
chr15	92491067	92492086
chr15	92493524	92493738
chr15	92494031	92494255
chr15	92496464	92496730
chr15	92496932	92497214
chr15	92497418	92497453

chr15	92499083	92500028
chr15	92508669	92509635
chr15	92516109	92516142
chr15	92516647	92516835
chr15	92517058	92517110
chr15	92517963	92517973
chr15	92602435	92602639
chr15	92613109	92613431
chr15	92613551	92613857
chr15	92617840	92617869
chr15	92673824	92673941
chr15	92701388	92701434
chr15	93340616	93341074
chr15	93344640	93345801
chr15	93346850	93347939
chr15	93350638	93350942
chr15	93351373	93353052
chr15	93353306	93353520
chr15	93353771	93353849
chr15	93354137	93354236
chr15	93354789	93355182
chr15	93363414	93363585
chr15	93363793	93363933
chr15	93365034	93365084
chr15	93374768	93375453
chr15	93375640	93376102
chr15	93383814	93383850
chr15	93425411	93425472
chr15	93426267	93426655
chr15	93443278	93443829
chr15	93447253	93447494
chr15	93447806	93447997
chr15	93449907	93450313
chr15	93451103	93451569
chr15	93452032	93452217
chr15	93456533	93456560
chr15	93456745	93456944
chr15	93457980	93458423
chr15	93459783	93460013
chr15	93460517	93461434
chr15	93464167	93464372
chr15	93464566	93465461
chr15	93466762	93466970
chr15	93493367	93493485
chr15	93524695	93525028
chr15	93571714	93572062

chr15	93572234	93572531
chr15	93573614	93573842
chr15	93591217	93591584
chr15	93615704	93615950
chr15	93616056	93616259
chr15	94373148	94373282
chr15	94773206	94773406
chr15	94773787	94775521
chr15	94775994	94776018
chr15	94782626	94783086
chr15	94808412	94808611
chr15	95013728	95013748
chr15	95014082	95014163
chr15	95036360	95036439
chr15	95036647	95037031
chr15	95037980	95038352
chr15	95217677	95217874
chr15	95220311	95221060
chr15	95334039	95334408
chr15	95388056	95388512
chr15	95770034	95770780
chr15	95790216	95790283
chr15	95790567	95790813
chr15	96100738	96100903
chr15	96101088	96101110
chr15	96103019	96103230
chr15	96103376	96103496
chr15	96178160	96178411
chr15	96596223	96596485
chr15	96596612	96596873
chr15	96694997	96695037
chr15	96695233	96695422
chr15	96715405	96715519
chr15	96769130	96769388
chr15	96773952	96774577
chr15	96812166	96812596
chr15	96813820	96813923
chr15	96814119	96814262
chr15	96816877	96816991
chr15	96817354	96817437
chr15	96819704	96819872
chr15	96865302	96865786
chr15	96868942	96869522
chr15	96869843	96870349
chr15	96870438	96870859
chr15	96873259	96873452

chr15	96874495	96874884
chr15	96874980	96875865
chr15	96876310	96876872
chr15	96877002	96877157
chr15	96879234	96879517
chr15	96883732	96884148
chr15	96884520	96884664
chr15	96897495	96897658
chr15	96900446	96900772
chr15	96901137	96901171
chr15	98045325	98045683
chr15	98047359	98047709
chr15	98418549	98419198
chr15	98447743	98447806
chr15	98502976	98502996
chr15	98503948	98505070
chr15	98520708	98520751
chr15	98520866	98521102
chr15	98524048	98524428
chr15	98538761	98538796
chr15	98546547	98546919
chr15	98547328	98547585
chr15	98578359	98578812
chr15	98666615	98667146
chr15	98667305	98667765
chr15	98667910	98668332
chr15	98698000	98698205
chr15	98698470	98698956
chr15	98707865	98708064
chr15	98708889	98709219
chr15	98709385	98709526
chr15	98865122	98865234
chr15	98865476	98865549
chr15	98865868	98866067
chr15	98965186	98965385
chr15	98971391	98971542
chr15	99091581	99092322
chr15	99105467	99105523
chr15	99154385	99154491
chr15	99157288	99157483
chr15	99157846	99158373
chr15	99190115	99190229
chr15	99190377	99191602
chr15	99191845	99192059
chr15	99192274	99193855
chr15	99194461	99194643

chr15	99194909	99195299
chr15	99200986	99201477
chr15	99212621	99213083
chr15	99223691	99223905
chr15	99225992	99226261
chr15	99226466	99226900
chr15	99270524	99270864
chr15	99271116	99271303
chr15	99289675	99289913
chr15	99297775	99297843
chr15	99298514	99298760
chr15	99304441	99304459
chr15	99317883	99319038
chr15	99322552	99322764
chr15	99342793	99343200
chr15	99343282	99343391
chr15	99343496	99343720
chr15	99343902	99344101
chr15	99344239	99344381
chr15	99346602	99347074
chr15	99393610	99393809
chr15	99394443	99396173
chr15	99396361	99398065
chr15	99398448	99398802
chr15	99399743	99399749
chr15	99401076	99401286
chr15	99408345	99408732
chr15	99408848	99408877
chr15	99409064	99409587
chr15	99409756	99410060
chr15	99412044	99412289
chr15	99416103	99416473
chr15	99416671	99417507
chr15	99418005	99418399
chr15	99424785	99424975
chr15	99425807	99426006
chr15	99428095	99428294
chr15	99432400	99432406
chr15	99433092	99433828
chr15	99439454	99440173
chr15	99440406	99440937
chr15	99446530	99447346
chr15	99447648	99448065
chr15	99449541	99450280
chr15	99457773	99457886
chr15	99557797	99557995

chr15	99558119	99559337
chr15	99559788	99559818
chr15	99601783	99602160
chr15	99602335	99602980
chr15	99640523	99640752
chr15	99645117	99645998
chr15	99646149	99646390
chr15	99662782	99662906
chr15	99663776	99664005
chr15	99664422	99664731
chr15	99717941	99718016
chr15	99718247	99718326
chr15	99786054	99786316
chr15	99789577	99789970
chr15	99790079	99790083
chr15	99791184	99791960
chr15	99804299	99804849
chr15	99846287	99846403
chr15	99846571	99846670
chr15	99861105	99861389
chr15	99864197	99864597
chr15	99866448	99866486
chr15	99949523	99949666
chr15	99965802	99966099
chr15	99970696	99970877
chr15	99975523	99975991
chr15	99982851	99983131
chr15	99990629	99991229
chr15	99994706	99994756
chr15	99994920	99995980
chr15	100016735	100017367
chr15	100026602	100026983
chr15	100027458	100027553
chr15	100104979	100106905
chr15	100145231	100145403
chr15	100145571	100145654
chr15	100145904	100146246
chr15	100159067	100159128
chr15	100222946	100222994
chr15	100243852	100244206
chr15	100248087	100248098
chr15	100248684	100248781
chr15	100249178	100249268
chr15	100273005	100273642
chr15	100273905	100274112
chr15	100346115	100347685

chr15	100465944	100468403
chr15	100469535	100469866
chr15	100468760	100469425
chr15	100503583	100503717
chr15	100503924	100504066
chr15	100504219	100504702
chr15	100506518	100506783
chr15	100507878	100508420
chr15	100516514	100517306
chr15	100517717	100517927
chr15	100518306	100518417
chr15	100519224	100519247
chr15	100519376	100519434
chr15	100538693	100538905
chr15	100557332	100557447
chr15	100560525	100560820
chr15	100582736	100583866
chr15	100600712	100601156
chr15	100614171	100614209
chr15	100650902	100651381
chr15	100651495	100652017
chr15	100652334	100652485
chr15	100848866	100849135
chr15	100881146	100881380
chr15	100881729	100882163
chr15	100882463	100882647
chr15	100882727	100882840
chr15	100889916	100891485
chr15	100913057	100913352
chr15	100913496	100913991
chr15	100914356	100914378
chr15	101105769	101105856
chr15	101141947	101142387
chr15	101142774	101143595
chr15	101167950	101168095
chr15	101168216	101168340
chr15	101227687	101227733
chr15	101250449	101250747
chr15	101252538	101252908
chr15	101253308	101253840
chr15	101254060	101254693
chr15	101256222	101256421
chr15	101258793	101259096
chr15	101260420	101260852
chr15	101261079	101261477
chr15	101261682	101262139

chr15	101262252	101262864
chr15	101263554	101264100
chr15	101266199	101266356
chr15	101266909	101267056
chr15	101267174	101267610
chr15	101267733	101268216
chr15	101389637	101389840
chr15	101389944	101390315
chr15	101413159	101413352
chr15	101419204	101421301
chr15	101422043	101422280
chr15	101457625	101458041
chr15	101458491	101459610
chr15	101459767	101460493
chr15	101469419	101469510
chr15	101469677	101469715
chr15	101547105	101547582
chr15	101547973	101548010
chr15	101548353	101548622
chr15	101608702	101609599
chr15	101609733	101610014
chr15	101610722	101611476
chr15	101629304	101629505
chr15	101652667	101653423
chr15	101665048	101665080
chr15	101707861	101708361
chr15	101716902	101717106
chr15	101728914	101729715
chr15	101739341	101739359
chr15	101739967	101740071
chr15	101747961	101748002
chr15	101753859	101754259
chr15	101790649	101790832
chr15	101791018	101791524
chr15	101791950	101792864
chr15	101816883	101818099
chr15	101834134	101834678
chr15	101834955	101835459
chr15	101835617	101836051
chr15	101836970	101837246
chr15	101837505	101837653
chr15	101889137	101889317
chr15	101889570	101889855
chr15	101897989	101898011
chr15	101898081	101898644
chr15	101898883	101899167

chr15	101899464	101899732
chr15	101900128	101900193
chr15	101902186	101902414
chr15	101985657	101985692
chr15	101986761	101986960
chr15	101999298	101999327
chr15	101999651	101999952
chr15	102018393	102018474
chr15	102029007	102029711
chr15	102030011	102030421
chr15	102041634	102041709
chr15	102041816	102041833
chr15	102115792	102116010
chr15	102119634	102119942
chr15	102178169	102178503
chr15	102181652	102181660
chr15	102182136	102182193
chr15	102188134	102188299
chr15	102191913	102192579
chr15	102192712	102193040
chr15	102193176	102193683
chr15	102233013	102233033
chr15	102233320	102233455
chr15	102264060	102264723
chr15	102285675	102286212
chr15	102286405	102287295
chr15	102494266	102494549
chr15	102495967	102496138
chr15	102499937	102500164
chr15	102500958	102502592
chr15	102503982	102504447
chr15	102504774	102505021
chr15	102505239	102505678
chr15	102509317	102509694
chr15	102521152	102521372
chr16	60128	60379
chr16	68859	69058
chr16	72610	72708
chr16	102840	103567
chr16	103918	104021
chr16	121251	121450
chr16	121680	122945
chr16	126556	126854
chr16	127241	128606
chr16	188047	189001
chr16	230301	230500

chr16	231111	231364
chr16	278994	279472
chr16	284156	285308
chr16	303723	303773
chr16	396495	396512
chr16	396863	396942
chr16	401659	401832
chr16	401952	402402
chr16	403278	403477
chr16	403978	404240
chr16	404547	404959
chr16	415566	415857
chr16	420170	420536
chr16	420821	421046
chr16	431100	431851
chr16	432128	432668
chr16	432923	433095
chr16	433256	433262
chr16	447203	447609
chr16	450958	451175
chr16	451374	452274
chr16	473252	473566
chr16	473908	474242
chr16	474649	475893
chr16	476582	476781
chr16	544808	545051
chr16	575252	575263
chr16	575974	576275
chr16	576747	577712
chr16	578080	578557
chr16	619061	619730
chr16	620653	620815
chr16	619927	620534
chr16	637536	637581
chr16	637724	639836
chr16	640194	640831
chr16	641215	641643
chr16	642909	643276
chr16	643373	643583
chr16	671894	672208
chr16	672487	672736
chr16	676689	677032
chr16	685535	686284
chr16	686589	686791
chr16	691842	692608
chr16	692790	693454

chr16	693650	693817
chr16	695356	695592
chr16	698407	699906
chr16	711191	711390
chr16	718120	718589
chr16	729976	730974
chr16	731138	731372
chr16	733783	734425
chr16	734703	734991
chr16	739649	740884
chr16	744892	745299
chr16	745452	745918
chr16	762643	762779
chr16	762970	763669
chr16	764236	764946
chr16	765256	766267
chr16	769501	769605
chr16	769688	769884
chr16	770034	770419
chr16	770572	771529
chr16	771719	772202
chr16	776485	776741
chr16	777107	777965
chr16	778207	778562
chr16	787973	788255
chr16	789044	789265
chr16	790482	791278
chr16	791429	791635
chr16	798904	799418
chr16	810072	812439
chr16	818806	819015
chr16	827498	827817
chr16	832851	833050
chr16	837789	838373
chr16	838656	839285
chr16	856877	857807
chr16	858062	858307
chr16	1020328	1020867
chr16	1132238	1132437
chr16	1133173	1133425
chr16	1345585	1345710
chr16	1347104	1347367
chr16	1347603	1347804
chr16	1352219	1352642
chr16	1353195	1353387
chr16	1358362	1359044

chr16	1359612	1359837
chr16	1360761	1361308
chr16	1401312	1401866
chr16	1402265	1402366
chr16	1429104	1429573
chr16	1429781	1430066
chr16	1457603	1457757
chr16	1458022	1458305
chr16	1464070	1464463
chr16	1466095	1466608
chr16	1470163	1470493
chr16	1470956	1471398
chr16	1524622	1525026
chr16	1525146	1525436
chr16	1541083	1541095
chr16	1542721	1542978
chr16	1543405	1543948
chr16	1580593	1580837
chr16	1593303	1594105
chr16	1595788	1595987
chr16	1660187	1661611
chr16	1661855	1662013
chr16	1662351	1662781
chr16	1663196	1663287
chr16	1664656	1664988
chr16	1727825	1728169
chr16	1728287	1729201
chr16	1734635	1734851
chr16	1738786	1739145
chr16	1739412	1739599
chr16	1739830	1740117
chr16	1741590	1741632
chr16	1751505	1751523
chr16	1751698	1751874
chr16	1754764	1755081
chr16	1755557	1756162
chr16	1756317	1756605
chr16	1757672	1757683
chr16	1769894	1770184
chr16	1772100	1772138
chr16	1794114	1794414
chr16	1795509	1795944
chr16	1796012	1796248
chr16	1796421	1797276
chr16	1804676	1804971
chr16	1820902	1821634

chr16	1822761	1822991
chr16	1823201	1823486
chr16	1830763	1831098
chr16	1831726	1832561
chr16	1832974	1833728
chr16	1834125	1834673
chr16	1834931	1835085
chr16	1876356	1876634
chr16	1876842	1877165
chr16	1877388	1877587
chr16	1878051	1878407
chr16	1920580	1920645
chr16	1920912	1920969
chr16	1922475	1922674
chr16	1962922	1963009
chr16	1978870	1979717
chr16	1979901	1980202
chr16	1985096	1985182
chr16	1985268	1985540
chr16	1988208	1988237
chr16	1992735	1992757
chr16	1993087	1993124
chr16	1993411	1994151
chr16	1999391	1999568
chr16	2000368	2000780
chr16	2002560	2002814
chr16	2009318	2010078
chr16	2010450	2010579
chr16	2013506	2013650
chr16	2014100	2014674
chr16	2015008	2015333
chr16	2015436	2015635
chr16	2021306	2022437
chr16	2029522	2030652
chr16	2034035	2034760
chr16	2040031	2040230
chr16	2042495	2042840
chr16	2044348	2044488
chr16	2046453	2046652
chr16	2047681	2047730
chr16	2057056	2057766
chr16	2057908	2058059
chr16	2058400	2059666
chr16	2059994	2060450
chr16	2062159	2062230
chr16	2065253	2065574

chr16	2065734	2066219
chr16	2069733	2070063
chr16	2072597	2073040
chr16	2076617	2076846
chr16	2077081	2077718
chr16	2083561	2083760
chr16	2091477	2091676
chr16	2097438	2097819
chr16	2097923	2098239
chr16	2115098	2115251
chr16	2177705	2177904
chr16	2178297	2179238
chr16	2179513	2179838
chr16	2184673	2185552
chr16	2186286	2186921
chr16	2190465	2190679
chr16	2198751	2199327
chr16	2203805	2203980
chr16	2204263	2204386
chr16	2204671	2204696
chr16	2204892	2205313
chr16	2205757	2206525
chr16	2211119	2211354
chr16	2211508	2211804
chr16	2212116	2212474
chr16	2213530	2213729
chr16	2229255	2229454
chr16	2246818	2247107
chr16	2255065	2255328
chr16	2255438	2256362
chr16	2256528	2256750
chr16	2264202	2264766
chr16	2265174	2265885
chr16	2273000	2273135
chr16	2273289	2274464
chr16	2300415	2300590
chr16	2300758	2301922
chr16	2317525	2318796
chr16	2319062	2319429
chr16	2389888	2389891
chr16	2389957	2390661
chr16	2391147	2391188
chr16	2478734	2479211
chr16	2479414	2479905
chr16	2480456	2480589
chr16	2498241	2498660

chr16	2509437	2509781
chr16	2510113	2510373
chr16	2517603	2518687
chr16	2521296	2522049
chr16	2523272	2523555
chr16	2523705	2523969
chr16	2524113	2525073
chr16	2525187	2525560
chr16	2563343	2564958
chr16	2570391	2571117
chr16	2571411	2571604
chr16	2581680	2581955
chr16	2582192	2583535
chr16	2586782	2587244
chr16	2587352	2587667
chr16	2588061	2588441
chr16	2653085	2653264
chr16	2653599	2653798
chr16	2654143	2654381
chr16	2656273	2656305
chr16	2656544	2656660
chr16	2657815	2657827
chr16	2713615	2713626
chr16	2713977	2714166
chr16	2714276	2714342
chr16	2722983	2723396
chr16	2732165	2732345
chr16	2732485	2733243
chr16	2734788	2734920
chr16	2770340	2771786
chr16	2773000	2773232
chr16	2801546	2803428
chr16	2818543	2818761
chr16	2818868	2820429
chr16	2826763	2827078
chr16	2827330	2828707
chr16	2835357	2835628
chr16	2885602	2885862
chr16	2886075	2886576
chr16	2896782	2896866
chr16	2906623	2906708
chr16	2907414	2908081
chr16	2908187	2908556
chr16	2908831	2909261
chr16	2917972	2918454
chr16	2918703	2918726

chr16	2932063	2932596
chr16	2932788	2932989
chr16	2933289	2933771
chr16	2954412	2955355
chr16	2956197	2956439
chr16	2956541	2956600
chr16	2956719	2956887
chr16	2956993	2957014
chr16	2960007	2960034
chr16	2960351	2960729
chr16	2961616	2961661
chr16	2961892	2962457
chr16	2962707	2962805
chr16	2965126	2965395
chr16	2965706	2966529
chr16	2968061	2968749
chr16	2974317	2974494
chr16	2974644	2974843
chr16	3009028	3009107
chr16	3012769	3015033
chr16	3019295	3019757
chr16	3020358	3020576
chr16	3029229	3029301
chr16	3029780	3031155
chr16	3032697	3032896
chr16	3033100	3033498
chr16	3033670	3034269
chr16	3039594	3040229
chr16	3040741	3042539
chr16	3042739	3044324
chr16	3044593	3044792
chr16	3045491	3045772
chr16	3045986	3046309
chr16	3049567	3049753
chr16	3055101	3055166
chr16	3059545	3059733
chr16	3060169	3060424
chr16	3060508	3061344
chr16	3062507	3062890
chr16	3063006	3063431
chr16	3069798	3070829
chr16	3071040	3072334
chr16	3072551	3073314
chr16	3073673	3074402
chr16	3086420	3086650
chr16	3095348	3095425

chr16	3096378	3097051
chr16	3108313	3109385
chr16	3109651	3110023
chr16	3114730	3115199
chr16	3115345	3115658
chr16	3150521	3150579
chr16	3156157	3156463
chr16	3156812	3157013
chr16	3162566	3162785
chr16	3174412	3175074
chr16	3177922	3178023
chr16	3179263	3179488
chr16	3184384	3184865
chr16	3185140	3185493
chr16	3199531	3200468
chr16	3200764	3201160
chr16	3201871	3202806
chr16	3207552	3207791
chr16	3208056	3208679
chr16	3209119	3209391
chr16	3209765	3209917
chr16	3218617	3219208
chr16	3221417	3221550
chr16	3224298	3224571
chr16	3224766	3225627
chr16	3225870	3225947
chr16	3233691	3233847
chr16	3234055	3234130
chr16	3234421	3234457
chr16	3238619	3239481
chr16	3239687	3239823
chr16	3284680	3284723
chr16	3284873	3285093
chr16	3285224	3285486
chr16	3292747	3292839
chr16	3293053	3293311
chr16	3306768	3307208
chr16	3307447	3307498
chr16	3308826	3309268
chr16	3309843	3310059
chr16	3313493	3313757
chr16	3314015	3314469
chr16	3314768	3314918
chr16	3332419	3333405
chr16	3333519	3333896
chr16	3333999	3334095

chr16	3355045	3355459
chr16	3355779	3356103
chr16	3414689	3414751
chr16	3415241	3415484
chr16	3415790	3415850
chr16	3450652	3450973
chr16	3451287	3451521
chr16	3493339	3493644
chr16	3493789	3494030
chr16	3494283	3494437
chr16	3507415	3507772
chr16	3507991	3508799
chr16	3550675	3550796
chr16	3551069	3551540
chr16	3661385	3661526
chr16	3661951	3662153
chr16	3668742	3668787
chr16	3669054	3669155
chr16	3766986	3767845
chr16	3788917	3788985
chr16	3889371	3889532
chr16	3929733	3930078
chr16	3930846	3931687
chr16	3931956	3931961
chr16	4000312	4000476
chr16	4009081	4009288
chr16	4103872	4104295
chr16	4165585	4165873
chr16	4166693	4167294
chr16	4186149	4186997
chr16	4190695	4191046
chr16	4226800	4226978
chr16	4229019	4229336
chr16	4233212	4233994
chr16	4234161	4234551
chr16	4263832	4264120
chr16	4264539	4264613
chr16	4264996	4265018
chr16	4273332	4273371
chr16	4303146	4303765
chr16	4321170	4321562
chr16	4321641	4322301
chr16	4322422	4322624
chr16	4323085	4323903
chr16	4324459	4324658
chr16	4329552	4329775

chr16	4329899	4330049
chr16	4338862	4338973
chr16	4340171	4340291
chr16	4340493	4340692
chr16	4340964	4340978
chr16	4348540	4348622
chr16	4349008	4349769
chr16	4352668	4352781
chr16	4356850	4357049
chr16	4357115	4357134
chr16	4357296	4357746
chr16	4358108	4358261
chr16	4364981	4365994
chr16	4366236	4366899
chr16	4370822	4371033
chr16	4373123	4373359
chr16	4373980	4374180
chr16	4377898	4378324
chr16	4394638	4394848
chr16	4400618	4401330
chr16	4402101	4402625
chr16	4421879	4423206
chr16	4423711	4424851
chr16	4425375	4425808
chr16	4442391	4442590
chr16	4442851	4443183
chr16	4443436	4443596
chr16	4444272	4444363
chr16	4465928	4466458
chr16	4475294	4475754
chr16	4514012	4514452
chr16	4514640	4514853
chr16	4524372	4524641
chr16	4524797	4524920
chr16	4525471	4525937
chr16	4526473	4526975
chr16	4527199	4527349
chr16	4527682	4528433
chr16	4538033	4538277
chr16	4545079	4545195
chr16	4545447	4545552
chr16	4545717	4546747
chr16	4548850	4548904
chr16	4549160	4549243
chr16	4550501	4550724
chr16	4587306	4587495

chr16	4587606	4589013
chr16	4635700	4635889
chr16	4647581	4647789
chr16	4661048	4662609
chr16	4662858	4663058
chr16	4663354	4665095
chr16	4665801	4666145
chr16	4666499	4667074
chr16	4668705	4668999
chr16	4669501	4669623
chr16	4674282	4675316
chr16	4683167	4683296
chr16	4715407	4715588
chr16	4725057	4725430
chr16	4730174	4730823
chr16	4730889	4730891
chr16	4731070	4731292
chr16	4743150	4744810
chr16	4783963	4784373
chr16	4817915	4818612
chr16	4816692	4817766
chr16	4824816	4825054
chr16	4850740	4850964
chr16	4851762	4852152
chr16	4852725	4853227
chr16	4896860	4898282
chr16	4898569	4898643
chr16	4912665	4912677
chr16	4919338	4919595
chr16	4933054	4933348
chr16	4946687	4946787
chr16	4946856	4946886
chr16	4947038	4947091
chr16	4961978	4961999
chr16	4962364	4962408
chr16	4963738	4963955
chr16	4966555	4966938
chr16	4968223	4968335
chr16	4968485	4968575
chr16	4968677	4968997
chr16	4969303	4969515
chr16	4969670	4969886
chr16	4972029	4972336
chr16	4972625	4973086
chr16	4973230	4973423
chr16	4974861	4975060

chr16	4975286	4975590
chr16	4985875	4986392
chr16	4986533	4987072
chr16	4987322	4987803
chr16	4987933	4988200
chr16	4990032	4990460
chr16	4990805	4991004
chr16	4992718	4992917
chr16	4993006	4993206
chr16	4993578	4993875
chr16	4994322	4994798
chr16	4995474	4995648
chr16	4997934	4998241
chr16	5006888	5006891
chr16	5007191	5007989
chr16	5037889	5038108
chr16	5121649	5121675
chr16	5121844	5122507
chr16	5147348	5147858
chr16	5148469	5148991
chr16	5289788	5289987
chr16	8688340	8688523
chr16	8715292	8715719
chr16	8771487	8771762
chr16	8772769	8772811
chr16	8773011	8773376
chr16	8810781	8810896
chr16	8846221	8846284
chr16	8891140	8891495
chr16	8891852	8892461
chr16	8922728	8922975
chr16	8942734	8943093
chr16	8943522	8943899
chr16	8943970	8945280
chr16	8953634	8953929
chr16	8954451	8954720
chr16	8955203	8955664
chr16	8961708	8962099
chr16	8962287	8962547
chr16	8964581	8964826
chr16	8965252	8965448
chr16	8971964	8972855
chr16	8973306	8973525
chr16	8974380	8974549
chr16	8978771	8979274
chr16	9056321	9056624

chr16	9056766	9057111
chr16	9086149	9086236
chr16	9162027	9162378
chr16	9162563	9162651
chr16	9166790	9167207
chr16	9170284	9170620
chr16	9171062	9171586
chr16	9172788	9173061
chr16	9184424	9184644
chr16	9184838	9185070
chr16	9185458	9186700
chr16	9186803	9186967
chr16	9193169	9193295
chr16	9196239	9196376
chr16	9204638	9204837
chr16	9209488	9209529
chr16	9221316	9221708
chr16	10275727	10275952
chr16	10351616	10351806
chr16	10375545	10375701
chr16	10395344	10395568
chr16	10395889	10395899
chr16	10400596	10400957
chr16	10402200	10402521
chr16	10402634	10403008
chr16	10422029	10422251
chr16	10422403	10422550
chr16	10428194	10428761
chr16	10435567	10435797
chr16	10479886	10480368
chr16	10522617	10522677
chr16	10522936	10523020
chr16	10523134	10523535
chr16	10603381	10603798
chr16	10609330	10609478
chr16	10615821	10615902
chr16	10627597	10627799
chr16	10630427	10630826
chr16	10636121	10636325
chr16	10641069	10641260
chr16	10662673	10662912
chr16	10671305	10671638
chr16	10673646	10675467
chr16	10688369	10688663
chr16	10688820	10689010
chr16	10689359	10689646

chr16	10691557	10691828
chr16	10691908	10692288
chr16	10692762	10692979
chr16	10694128	10694223
chr16	10694541	10694713
chr16	10694979	10695422
chr16	10695695	10696334
chr16	10703535	10703565
chr16	10706517	10706569
chr16	10708746	10709072
chr16	10710109	10710542
chr16	10710743	10711252
chr16	10719744	10720102
chr16	10720534	10720703
chr16	10722875	10722997
chr16	10723181	10723513
chr16	10744118	10744245
chr16	10744680	10745141
chr16	10745389	10745407
chr16	10745510	10747079
chr16	10747190	10747573
chr16	10748301	10748322
chr16	10747686	10748097
chr16	10750116	10750315
chr16	10779291	10779294
chr16	10823773	10823997
chr16	10831186	10831272
chr16	10832391	10832519
chr16	10832588	10833157
chr16	10837398	10837995
chr16	10838191	10838716
chr16	10842881	10842900
chr16	10922838	10923285
chr16	10965904	10966045
chr16	10972570	10972866
chr16	11012883	11013012
chr16	11033088	11033413
chr16	11034440	11035215
chr16	11035358	11036315
chr16	11036418	11036617
chr16	11036771	11037081
chr16	11038041	11038140
chr16	11038334	11039182
chr16	11039423	11039610
chr16	11039726	11040745
chr16	11044150	11044241

chr16	11047752	11048071
chr16	11067649	11067687
chr16	11067804	11068018
chr16	11085778	11086009
chr16	11086910	11087153
chr16	11102878	11103486
chr16	11103627	11103802
chr16	11108002	11108080
chr16	11108453	11108593
chr16	11110312	11110679
chr16	11133957	11133985
chr16	11143233	11143254
chr16	11171392	11171431
chr16	11171740	11171994
chr16	11185092	11185184
chr16	11187568	11187984
chr16	11198618	11198850
chr16	11199079	11199316
chr16	11203311	11203403
chr16	11207068	11207310
chr16	11227036	11227236
chr16	11283656	11284108
chr16	11293007	11293407
chr16	11293648	11294296
chr16	11296019	11296589
chr16	11296716	11297105
chr16	11297686	11297773
chr16	11298712	11298911
chr16	11299631	11300167
chr16	11300743	11301695
chr16	11302205	11303713
chr16	11306221	11306614
chr16	11307188	11307454
chr16	11331123	11331322
chr16	11334487	11334703
chr16	11335095	11335207
chr16	11348367	11351893
chr16	11372853	11373060
chr16	11391560	11391873
chr16	11397106	11397408
chr16	11399708	11400261
chr16	11400921	11401054
chr16	11405628	11406039
chr16	11406449	11407201
chr16	11407968	11408090
chr16	11408567	11409253

chr16	11422714	11422850
chr16	11434141	11434183
chr16	11434266	11434438
chr16	11435297	11435767
chr16	11436031	11436431
chr16	11437276	11437616
chr16	11436731	11436850
chr16	11438972	11439102
chr16	11439319	11440053
chr16	11440372	11440420
chr16	11442110	11442380
chr16	11443928	11444276
chr16	11450012	11450211
chr16	11451311	11451901
chr16	11452039	11452272
chr16	11452511	11452925
chr16	11453112	11453479
chr16	11453603	11454002
chr16	11461414	11461640
chr16	11461758	11462218
chr16	11500502	11500700
chr16	11501656	11501744
chr16	11502277	11502551
chr16	11502845	11503214
chr16	11503894	11504294
chr16	11505194	11505397
chr16	11538720	11538791
chr16	11539831	11540034
chr16	11552134	11552638
chr16	11568603	11568808
chr16	11575558	11575785
chr16	11577329	11577849
chr16	11578576	11578621
chr16	11587159	11587320
chr16	11588481	11588877
chr16	11588983	11590067
chr16	11590139	11590275
chr16	11590487	11592822
chr16	11600033	11600261
chr16	11601703	11601902
chr16	11603162	11603477
chr16	11625615	11625616
chr16	11625950	11626050
chr16	11626229	11626420
chr16	11627838	11628304
chr16	11628465	11628632

chr16	11628742	11628751
chr16	11630461	11630523
chr16	11633956	11634007
chr16	11634590	11634664
chr16	11640186	11640541
chr16	11647867	11647981
chr16	11651363	11651593
chr16	11654091	11655243
chr16	11655310	11655745
chr16	11655810	11656954
chr16	11657248	11657735
chr16	11659454	11659653
chr16	11660131	11660152
chr16	11660270	11660498
chr16	11660874	11660915
chr16	11661660	11661859
chr16	11662080	11662632
chr16	11663218	11663417
chr16	11664900	11664978
chr16	11665771	11665980
chr16	11667644	11668152
chr16	11668547	11668697
chr16	11671150	11671258
chr16	11671936	11672135
chr16	11674673	11675026
chr16	11679410	11680216
chr16	11680335	11681806
chr16	11681910	11682101
chr16	11686647	11686741
chr16	11687643	11687692
chr16	11687852	11687909
chr16	11688283	11688515
chr16	11689681	11689821
chr16	11690632	11690998
chr16	11691102	11691338
chr16	11691565	11692053
chr16	11692774	11693033
chr16	11693658	11694081
chr16	11695563	11696026
chr16	11697653	11698120
chr16	11700696	11700874
chr16	11701797	11702559
chr16	11703094	11703779
chr16	11704067	11704620
chr16	11705579	11706977
chr16	11707069	11708022

chr16	11708369	11709049
chr16	11709258	11709805
chr16	11710042	11710229
chr16	11710694	11710828
chr16	11711757	11713491
chr16	11715060	11715076
chr16	11716050	11716389
chr16	11718769	11719637
chr16	11720079	11720430
chr16	11720893	11722999
chr16	11723217	11724524
chr16	11724598	11725075
chr16	11725375	11725530
chr16	11727581	11727607
chr16	11727717	11727980
chr16	11728541	11728905
chr16	11729439	11729583
chr16	11730114	11730456
chr16	11731142	11731700
chr16	11730591	11731041
chr16	11733579	11735532
chr16	11735665	11736115
chr16	11736207	11736553
chr16	11737121	11737149
chr16	11738739	11739094
chr16	11745971	11746246
chr16	11750425	11750645
chr16	11752348	11753852
chr16	11754112	11754363
chr16	11756099	11756255
chr16	11756967	11757215
chr16	11757337	11757372
chr16	11761712	11762211
chr16	11762497	11763382
chr16	11766516	11766928
chr16	11768214	11768480
chr16	11768941	11769532
chr16	11769889	11770102
chr16	11770330	11771584
chr16	11772066	11772493
chr16	11774619	11775729
chr16	11776207	11776358
chr16	11776724	11776900
chr16	11789665	11789891
chr16	11804458	11804608
chr16	11808437	11808500

chr16	11808738	11808889
chr16	11835595	11836801
chr16	11836971	11837887
chr16	11856422	11856649
chr16	11875527	11876004
chr16	11876623	11877437
chr16	11877667	11877709
chr16	11877867	11878103
chr16	11880249	11880356
chr16	11883660	11883884
chr16	11887527	11887768
chr16	11890118	11890593
chr16	11890706	11891128
chr16	11891276	11893023
chr16	11893090	11893330
chr16	11893618	11894050
chr16	11903435	11903593
chr16	11906150	11906316
chr16	11928349	11928832
chr16	11929091	11929432
chr16	11930521	11930976
chr16	11932269	11932679
chr16	11936089	11936199
chr16	11942709	11943092
chr16	11944660	11945428
chr16	11943739	11943978
chr16	11945569	11946172
chr16	11946731	11947376
chr16	11947509	11947519
chr16	11950378	11950533
chr16	11950732	11950867
chr16	11960691	11961445
chr16	11973982	11974265
chr16	11995175	11995265
chr16	11995660	11996070
chr16	11997257	11997319
chr16	12008609	12009861
chr16	12010126	12010886
chr16	12013085	12013231
chr16	12018762	12018961
chr16	12019426	12019625
chr16	12028183	12028213
chr16	12029925	12030173
chr16	12042676	12042875
chr16	12046831	12047242
chr16	12049436	12049714

chr16	12049915	12050294
chr16	12050398	12050525
chr16	12051886	12051891
chr16	12052664	12052733
chr16	12053091	12053259
chr16	12054971	12054972
chr16	12056706	12057001
chr16	12057607	12057970
chr16	12066474	12066524
chr16	12068152	12068359
chr16	12069467	12071549
chr16	12109452	12109651
chr16	12121536	12121588
chr16	12123759	12124049
chr16	12130844	12130995
chr16	12131214	12131556
chr16	12135330	12135506
chr16	12135767	12136009
chr16	12142949	12142992
chr16	12156130	12156288
chr16	12156907	12157106
chr16	12213360	12213392
chr16	12220162	12220394
chr16	13401522	13401560
chr16	14013332	14013672
chr16	14013858	14014505
chr16	14015056	14015085
chr16	14030756	14031054
chr16	14033933	14034065
chr16	14051105	14052046
chr16	14053479	14053521
chr16	14053690	14053711
chr16	14087543	14087664
chr16	14092871	14093075
chr16	14096762	14097297
chr16	14099781	14099991
chr16	14101310	14101668
chr16	14104054	14104286
chr16	14104660	14104773
chr16	14109810	14110246
chr16	14106447	14108706
chr16	14118285	14118512
chr16	14143617	14143884
chr16	14144498	14144570
chr16	14144767	14144983
chr16	14163721	14163920

chr16	14164204	14165767
chr16	14182268	14182331
chr16	14189865	14190380
chr16	14190949	14191418
chr16	14193332	14193531
chr16	14238573	14238730
chr16	14238884	14238888
chr16	14242371	14242523
chr16	14242776	14242857
chr16	14249397	14250143
chr16	14266477	14266652
chr16	14275362	14275529
chr16	14343651	14343714
chr16	14378866	14380177
chr16	14380440	14380706
chr16	14394420	14394775
chr16	14395008	14395849
chr16	14396032	14397956
chr16	14398158	14398452
chr16	14400650	14400878
chr16	14401213	14402060
chr16	14402827	14403384
chr16	14402211	14402611
chr16	14403568	14403962
chr16	14418621	14418744
chr16	14419996	14420127
chr16	14448715	14448906
chr16	14449485	14449697
chr16	14451993	14452599
chr16	14456609	14456822
chr16	14457054	14457329
chr16	14457499	14459562
chr16	14459778	14459803
chr16	14471629	14472160
chr16	14472878	14473349
chr16	14473435	14473479
chr16	14475787	14476839
chr16	14489122	14489412
chr16	14490336	14490619
chr16	14491481	14491703
chr16	14492528	14494211
chr16	14494323	14494740
chr16	14497061	14497286
chr16	14499491	14499798
chr16	14503354	14503523
chr16	14524595	14524622

chr16	14543715	14543741
chr16	14544028	14544072
chr16	14569688	14569738
chr16	14580375	14580668
chr16	14580878	14581163
chr16	14581469	14581592
chr16	14583732	14583806
chr16	14591864	14592009
chr16	14592262	14592479
chr16	14595342	14595514
chr16	14595646	14595946
chr16	14597127	14597261
chr16	14597656	14597978
chr16	14622931	14623185
chr16	14642596	14642829
chr16	14657008	14657148
chr16	14657287	14657448
chr16	14658698	14659095
chr16	14675055	14675137
chr16	14699984	14700165
chr16	14705536	14705578
chr16	14723278	14724551
chr16	14726039	14726567
chr16	14726804	14727908
chr16	14728041	14728321
chr16	14733806	14733970
chr16	14744564	14744626
chr16	14745105	14745502
chr16	14751250	14751359
chr16	14751484	14751495
chr16	14788419	14788817
chr16	14795732	14797100
chr16	14797251	14797840
chr16	14800588	14800924
chr16	14801637	14802119
chr16	14802719	14803339
chr16	14808992	14809209
chr16	14810946	14811002
chr16	14826034	14826340
chr16	14828203	14828241
chr16	14831571	14831932
chr16	14834791	14836914
chr16	14839724	14839815
chr16	14840018	14840237
chr16	14840943	14841289
chr16	14842345	14842396

chr16	14849567	14849772
chr16	14861429	14861924
chr16	14917467	14917687
chr16	14918086	14919226
chr16	14924138	14924269
chr16	14924440	14924837
chr16	14925777	14925881
chr16	14927161	14927387
chr16	14927527	14928942
chr16	14930915	14931045
chr16	14931157	14931307
chr16	14933478	14933575
chr16	14934514	14934595
chr16	14940760	14940891
chr16	14946023	14947759
chr16	14948584	14948748
chr16	14983029	14983155
chr16	14985555	14986308
chr16	14994567	14994766
chr16	14995050	14995476
chr16	14995899	14996101
chr16	15000656	15000769
chr16	15003863	15004061
chr16	15004139	15004987
chr16	15005259	15006842
chr16	15006969	15007743
chr16	15008263	15008462
chr16	15008838	15008934
chr16	15009075	15009459
chr16	15021916	15022123
chr16	15027694	15027947
chr16	15029892	15030214
chr16	15034074	15034093
chr16	15035030	15035429
chr16	15036153	15036450
chr16	15036616	15036756
chr16	15037163	15037400
chr16	15040183	15040353
chr16	15046612	15046836
chr16	15056090	15056115
chr16	15058449	15058667
chr16	15060284	15060343
chr16	15064329	15064877
chr16	15065248	15065859
chr16	15067751	15070367
chr16	15072042	15072186

chr16	15077679	15077923
chr16	15079671	15079985
chr16	15081552	15081759
chr16	15090630	15090730
chr16	15095001	15095167
chr16	15098937	15099218
chr16	15107612	15107881
chr16	15109067	15109088
chr16	15111726	15111823
chr16	15114692	15115166
chr16	15118965	15119212
chr16	15119293	15119413
chr16	15132471	15132561
chr16	15135882	15135896
chr16	15137206	15137313
chr16	15139048	15139354
chr16	15145248	15145627
chr16	15146743	15147000
chr16	15148568	15148705
chr16	15148907	15149907
chr16	15150245	15150726
chr16	15156198	15156598
chr16	15156774	15156914
chr16	15161725	15161912
chr16	15166122	15166594
chr16	15172911	15172985
chr16	15185016	15185215
chr16	15187013	15187340
chr16	15187589	15188100
chr16	15188294	15188538
chr16	15190754	15190959
chr16	15193016	15193215
chr16	15195670	15195869
chr16	15196616	15196815
chr16	15206705	15206834
chr16	15207332	15207416
chr16	15207740	15207982
chr16	15240725	15240924
chr16	15241326	15242051
chr16	15242166	15242594
chr16	15242890	15243622
chr16	15244896	15245234
chr16	15247173	15248710
chr16	15249029	15249257
chr16	15249423	15249913
chr16	15450328	15450706

chr16	15451091	15451135
chr16	15454742	15454980
chr16	15466572	15466623
chr16	15466905	15467239
chr16	15468233	15468675
chr16	15470892	15470908
chr16	15472632	15473043
chr16	15474267	15474706
chr16	15474978	15475013
chr16	15475963	15476178
chr16	15476304	15477106
chr16	15482559	15482758
chr16	15488804	15489058
chr16	15489209	15490004
chr16	15490823	15491022
chr16	15492119	15492260
chr16	15492800	15492808
chr16	15497308	15497760
chr16	15498686	15498698
chr16	15502610	15502809
chr16	15509347	15509880
chr16	15510173	15510521
chr16	15514689	15514896
chr16	15515571	15515872
chr16	15521439	15521797
chr16	15524863	15525092
chr16	15526419	15526813
chr16	15528350	15528819
chr16	15529427	15529794
chr16	15531524	15531920
chr16	15545907	15546217
chr16	15554781	15554957
chr16	15570263	15570380
chr16	15574071	15574193
chr16	15575779	15576079
chr16	15576559	15576669
chr16	15579828	15579918
chr16	15595929	15596288
chr16	15599627	15599765
chr16	15612899	15613043
chr16	15633579	15633837
chr16	15635794	15636034
chr16	15647112	15647311
chr16	15651561	15651763
chr16	15669797	15670612
chr16	15675839	15676167

chr16	15682764	15683007
chr16	15683190	15683192
chr16	15683919	15684770
chr16	15684876	15685608
chr16	15686000	15686216
chr16	15686453	15686669
chr16	15686794	15687045
chr16	15702072	15702086
chr16	15707258	15708487
chr16	15712020	15712213
chr16	15712909	15713191
chr16	15712467	15712672
chr16	15713722	15713990
chr16	15717806	15718061
chr16	15732176	15732471
chr16	15735652	15736040
chr16	15736227	15737860
chr16	15737965	15738577
chr16	15738807	15739457
chr16	15740138	15740817
chr16	15741014	15741563
chr16	15741628	15741841
chr16	15741952	15742523
chr16	15742676	15742940
chr16	15745735	15746718
chr16	15747036	15747516
chr16	15747822	15748280
chr16	15743122	15745414
chr16	15750635	15750781
chr16	15751392	15751764
chr16	15752589	15752869
chr16	15753354	15753363
chr16	15753481	15753572
chr16	15754776	15754838
chr16	15755392	15755590
chr16	15755846	15756292
chr16	15758302	15758559
chr16	15758937	15759160
chr16	15759431	15759925
chr16	15760342	15761083
chr16	15761613	15761868
chr16	15762043	15762249
chr16	15762614	15762849
chr16	15763350	15763830
chr16	15764034	15767505
chr16	15767631	15767851

chr16	15768489	15768830
chr16	15768990	15769603
chr16	15769831	15770032
chr16	15770129	15770664
chr16	15771266	15771465
chr16	15772349	15772393
chr16	15773663	15773865
chr16	15775238	15775401
chr16	15775899	15776347
chr16	15776661	15777012
chr16	15782338	15782448
chr16	15783127	15783336
chr16	15784009	15784553
chr16	15788170	15788219
chr16	15788311	15788573
chr16	15791150	15791359
chr16	15792449	15792596
chr16	15794050	15794249
chr16	15795003	15795080
chr16	15796230	15796716
chr16	15800734	15800810
chr16	15804158	15804433
chr16	15805580	15805851
chr16	15820264	15820271
chr16	15820444	15820507
chr16	15821708	15821907
chr16	15827115	15827550
chr16	15828134	15828469
chr16	15837848	15838308
chr16	15849690	15849883
chr16	15850599	15850794
chr16	15850892	15851027
chr16	15874372	15874600
chr16	15900796	15900995
chr16	15915613	15915812
chr16	15924571	15924770
chr16	15952039	15952289
chr16	15953789	15953851
chr16	15966013	15966124
chr16	15968639	15969080
chr16	15969682	15970446
chr16	15973248	15973733
chr16	15974330	15974693
chr16	15976178	15976203
chr16	15976742	15977021
chr16	15978634	15979437

chr16	15980512	15980864
chr16	15981845	15983260
chr16	15983954	15984287
chr16	15985045	15985265
chr16	15986766	15986824
chr16	15988557	15989222
chr16	15989356	15989952
chr16	15990237	15990372
chr16	15991677	15992035
chr16	15992128	15992716
chr16	15991092	15991119
chr16	15992822	15993202
chr16	15993326	15994072
chr16	15994957	15995432
chr16	15995715	15996292
chr16	15996514	15997627
chr16	16006829	16007020
chr16	16009417	16009712
chr16	16011151	16011188
chr16	16013218	16013576
chr16	16013705	16014316
chr16	16015235	16015468
chr16	16017981	16018385
chr16	16018817	16019136
chr16	16021023	16021254
chr16	16021692	16022058
chr16	16022233	16022504
chr16	16023408	16023491
chr16	16024198	16024520
chr16	16028315	16028566
chr16	16030675	16030680
chr16	16030870	16031260
chr16	16032997	16033313
chr16	16033670	16033873
chr16	16035018	16035227
chr16	16035675	16036260
chr16	16039094	16039394
chr16	16041167	16041456
chr16	16039556	16040789
chr16	16042214	16044786
chr16	16045397	16046861
chr16	16046960	16047200
chr16	16049885	16050165
chr16	16051149	16051235
chr16	16056411	16056579
chr16	16057753	16058223

chr16	16059279	16059702
chr16	16059985	16060922
chr16	16061047	16061716
chr16	16062315	16062562
chr16	16062823	16063081
chr16	16064676	16064941
chr16	16068749	16069031
chr16	16069445	16070206
chr16	16070738	16071156
chr16	16071994	16072193
chr16	16080842	16081041
chr16	16081591	16081891
chr16	16082171	16083076
chr16	16083244	16083633
chr16	16083961	16084206
chr16	16084611	16084810
chr16	16086284	16086480
chr16	16090014	16090699
chr16	16090929	16092728
chr16	16099244	16099379
chr16	16101241	16101383
chr16	16102934	16102972
chr16	16105395	16105883
chr16	16106507	16106863
chr16	16111614	16111827
chr16	16112265	16112868
chr16	16113012	16113344
chr16	16113521	16114109
chr16	16115317	16115910
chr16	16116165	16116398
chr16	16116712	16116996
chr16	16117379	16117419
chr16	16117865	16118105
chr16	16120607	16120716
chr16	16123180	16123620
chr16	16124123	16124435
chr16	16122998	16123086
chr16	16125919	16126037
chr16	16127581	16127923
chr16	16129344	16129769
chr16	16130031	16130145
chr16	16134482	16134675
chr16	16135651	16135842
chr16	16135942	16135954
chr16	16137240	16137594
chr16	16138998	16139248

chr16	16140171	16140286
chr16	16143919	16143939
chr16	16145870	16146390
chr16	16150207	16150211
chr16	16156093	16156437
chr16	16159272	16159514
chr16	16162285	16163380
chr16	16164102	16164512
chr16	16166173	16166994
chr16	16167501	16167964
chr16	16168339	16169044
chr16	16169341	16169702
chr16	16186628	16186827
chr16	16188266	16188855
chr16	16189986	16190028
chr16	16199622	16199935
chr16	16202267	16202466
chr16	16203063	16203298
chr16	16208368	16208391
chr16	16217771	16217859
chr16	16221717	16221869
chr16	16223306	16223347
chr16	16223583	16223624
chr16	16228631	16228830
chr16	16231419	16231710
chr16	16237524	16237782
chr16	16238757	16239076
chr16	16245942	16246038
chr16	16287387	16287650
chr16	16289130	16289140
chr16	16290517	16290737
chr16	16304604	16304740
chr16	16306244	16306481
chr16	16307612	16307673
chr16	16311746	16312205
chr16	16314656	16314668
chr16	16317016	16317787
chr16	16321511	16321629
chr16	16326297	16327788
chr16	16329401	16329718
chr16	16330167	16330739
chr16	16332544	16332859
chr16	16335308	16335542
chr16	16337406	16337654
chr16	16344941	16345721
chr16	16346116	16346159

chr16	16348807	16348818
chr16	16353873	16354139
chr16	16358363	16358610
chr16	16362892	16363054
chr16	16384370	16384615
chr16	16384725	16384810
chr16	16393086	16393384
chr16	16393674	16394062
chr16	16394220	16394938
chr16	16402998	16403633
chr16	16403821	16404146
chr16	16404276	16405806
chr16	16408452	16408691
chr16	16434615	16435278
chr16	16439033	16439179
chr16	16448932	16449123
chr16	16476880	16477054
chr16	16478467	16478652
chr16	16478743	16479160
chr16	16480825	16481079
chr16	16489164	16489363
chr16	16490270	16490471
chr16	16543618	16543934
chr16	16699477	16699676
chr16	16728224	16728253
chr16	16729068	16729278
chr16	16734544	16734769
chr16	17564030	17564487
chr16	17564934	17565153
chr16	17609677	17609952
chr16	17645653	17646662
chr16	17648978	17649157
chr16	17650436	17650635
chr16	17650829	17651025
chr16	17659045	17659164
chr16	17660501	17661138
chr16	17661307	17661690
chr16	17665175	17665399
chr16	17665534	17665613
chr16	17665845	17666007
chr16	17726291	17726315
chr16	17726497	17726568
chr16	17726817	17726973
chr16	17727413	17727643
chr16	18301094	18301375
chr16	18375846	18375931

chr16	18381665	18381864
chr16	18394246	18394268
chr16	18394406	18394445
chr16	18410186	18410448
chr16	18421429	18421651
chr16	18447420	18447637
chr16	18457022	18457178
chr16	18458698	18458844
chr16	18460583	18460904
chr16	18461140	18461554
chr16	18461804	18461863
chr16	18462803	18463057
chr16	18465114	18465268
chr16	18491027	18491359
chr16	18491541	18491700
chr16	18491805	18492179
chr16	18492698	18493004
chr16	18494752	18495611
chr16	18495737	18496032
chr16	18496250	18497141
chr16	18497208	18497329
chr16	18504936	18505139
chr16	18505777	18506162
chr16	18509431	18509660
chr16	18515150	18515545
chr16	18519659	18519858
chr16	18519991	18519995
chr16	18538029	18538194
chr16	18547269	18548016
chr16	18554146	18554966
chr16	18555061	18555551
chr16	18556047	18556186
chr16	18563922	18564075
chr16	18567031	18567112
chr16	18570275	18570603
chr16	18571105	18571529
chr16	18572005	18573504
chr16	18576165	18576385
chr16	18576837	18577027
chr16	18582228	18582852
chr16	18587870	18588110
chr16	18593298	18593505
chr16	18640696	18640895
chr16	18770299	18770434
chr16	18771037	18771284
chr16	18774065	18774264

chr16	18785183	18785399
chr16	18788068	18788118
chr16	18790570	18790836
chr16	18794406	18794949
chr16	18795163	18795236
chr16	18795507	18795546
chr16	18797421	18798193
chr16	18798383	18799290
chr16	18799844	18800148
chr16	18800889	18802875
chr16	18804329	18805520
chr16	18812012	18813042
chr16	18813249	18813890
chr16	18815442	18816202
chr16	18818082	18818454
chr16	18831507	18831889
chr16	18850832	18851016
chr16	18851186	18851313
chr16	18862726	18862844
chr16	18878422	18878817
chr16	18899240	18899439
chr16	18904793	18904908
chr16	18905183	18905346
chr16	18913691	18913953
chr16	18914245	18914444
chr16	18916332	18916650
chr16	18917181	18917233
chr16	18918267	18918418
chr16	18920023	18920189
chr16	18920494	18920643
chr16	18921767	18922004
chr16	18928135	18928324
chr16	18929206	18929467
chr16	18930257	18931791
chr16	18934138	18934465
chr16	18934646	18934909
chr16	18936496	18939685
chr16	18939827	18940169
chr16	18942848	18943103
chr16	18950140	18950503
chr16	18950643	18951735
chr16	18953342	18953390
chr16	18953586	18953966
chr16	18954709	18954904
chr16	18955600	18955780
chr16	18956094	18956402

chr16	18957049	18957929
chr16	18958018	18958463
chr16	18958538	18958595
chr16	18958968	18959190
chr16	18959357	18959382
chr16	18961697	18962397
chr16	18967925	18968181
chr16	18968846	18968894
chr16	18969246	18970092
chr16	18970359	18970879
chr16	18971171	18971572
chr16	18972410	18972423
chr16	18972532	18972657
chr16	18973011	18973877
chr16	18974128	18974527
chr16	18976102	18976192
chr16	18976601	18976934
chr16	18977350	18977916
chr16	18980350	18980859
chr16	18980967	18981080
chr16	18981180	18981302
chr16	18984844	18985083
chr16	18985451	18985650
chr16	18989731	18989930
chr16	18990777	18990983
chr16	18993856	18994644
chr16	18994818	18995826
chr16	18997072	18997412
chr16	18997804	18997815
chr16	18999374	19000253
chr16	19000352	19001277
chr16	19008686	19009306
chr16	19009396	19010985
chr16	19011218	19011333
chr16	19013611	19013862
chr16	19016583	19017077
chr16	19017271	19018454
chr16	19018725	19018892
chr16	19019206	19019960
chr16	19023710	19023980
chr16	19034035	19034197
chr16	19053493	19053702
chr16	19055288	19055653
chr16	19060468	19060491
chr16	19063729	19063953
chr16	19064082	19064199

chr16	19064820	19065083
chr16	19072726	19072761
chr16	19078499	19080106
chr16	19080960	19081038
chr16	19083808	19084020
chr16	19084301	19084616
chr16	19087799	19088000
chr16	19093634	19093694
chr16	19097156	19097900
chr16	19098045	19098390
chr16	19099036	19099134
chr16	19103099	19103772
chr16	19103894	19104068
chr16	19106902	19107585
chr16	19108383	19108833
chr16	19117990	19118227
chr16	19118482	19120047
chr16	19121533	19121665
chr16	19121953	19122280
chr16	19123218	19123417
chr16	19124018	19127349
chr16	19127425	19127624
chr16	19128024	19128793
chr16	19130200	19130651
chr16	19130939	19131354
chr16	19131478	19131693
chr16	19131901	19132364
chr16	19132796	19134231
chr16	19134294	19134938
chr16	19135047	19135095
chr16	19136585	19136864
chr16	19139225	19139293
chr16	19141527	19141726
chr16	19142058	19142672
chr16	19142776	19143859
chr16	19144098	19144328
chr16	19145351	19145550
chr16	19146418	19148555
chr16	19148806	19149172
chr16	19149804	19150048
chr16	19150166	19150343
chr16	19154378	19154565
chr16	19154805	19155002
chr16	19176201	19176555
chr16	19177207	19177490
chr16	19177606	19178030

chr16	19178436	19180482
chr16	19185980	19186414
chr16	19186643	19187194
chr16	19191186	19191343
chr16	19193777	19194023
chr16	19195006	19196044
chr16	19196108	19196729
chr16	19197628	19197959
chr16	19198584	19198784
chr16	19201936	19202212
chr16	19203341	19203729
chr16	19219566	19219717
chr16	19221807	19221847
chr16	19232265	19232463
chr16	19238859	19238861
chr16	19293731	19293977
chr16	19328950	19329008
chr16	19382888	19382994
chr16	19395358	19395691
chr16	19400344	19400489
chr16	19404765	19405521
chr16	19408024	19408258
chr16	19408372	19408400
chr16	19421597	19421821
chr16	19421953	19422307
chr16	19424156	19425178
chr16	19425979	19426301
chr16	19427075	19427668
chr16	19428105	19428308
chr16	19430158	19430444
chr16	19430688	19431486
chr16	19443820	19444216
chr16	19456928	19457367
chr16	19474133	19474206
chr16	19475541	19475740
chr16	19484412	19484469
chr16	19484564	19484611
chr16	19494241	19494504
chr16	19501145	19501374
chr16	19510678	19510993
chr16	19513919	19514056
chr16	19518122	19518188
chr16	19519323	19519334
chr16	19528734	19529180
chr16	19530203	19530231
chr16	19531615	19533270

chr16	19534897	19534902
chr16	19535266	19536937
chr16	19549775	19549975
chr16	19565340	19565354
chr16	19565545	19565559
chr16	19566196	19567375
chr16	19567645	19567990
chr16	19568486	19568513
chr16	19568659	19568894
chr16	19571185	19571315
chr16	19582684	19582886
chr16	19594988	19595389
chr16	19606210	19606851
chr16	19607137	19607237
chr16	19607765	19608025
chr16	19612404	19612614
chr16	19624515	19624738
chr16	19630288	19630457
chr16	19649651	19649833
chr16	19650144	19650417
chr16	19656700	19656882
chr16	19699244	19699443
chr16	19700670	19700894
chr16	19701416	19701531
chr16	19707394	19707729
chr16	19715405	19715844
chr16	19722180	19722487
chr16	19724464	19724699
chr16	19726877	19727042
chr16	19727861	19728018
chr16	19728631	19730541
chr16	19732974	19733016
chr16	19734072	19734332
chr16	19769529	19769747
chr16	19809479	19809483
chr16	19812604	19812845
chr16	19822504	19822513
chr16	19895948	19896152
chr16	19896847	19897046
chr16	19897874	19898057
chr16	19907098	19907297
chr16	20686514	20687445
chr16	20719442	20719645
chr16	20751594	20751793
chr16	20752172	20752728
chr16	20752856	20753128

chr16	20775338	20775461
chr16	20817359	20818764
chr16	20832671	20832750
chr16	20861161	20861590
chr16	20862892	20863142
chr16	20863261	20863394
chr16	20876843	20878062
chr16	20879011	20879219
chr16	20879384	20879480
chr16	20880737	20880877
chr16	20885927	20886277
chr16	20886453	20886514
chr16	20890400	20890409
chr16	20891676	20891870
chr16	20910467	20910665
chr16	20911088	20913357
chr16	20922041	20922240
chr16	20922722	20922939
chr16	20922379	20922590
chr16	20925009	20925901
chr16	21168304	21168453
chr16	21169384	21171345
chr16	21178604	21178702
chr16	21179076	21179155
chr16	21258317	21258530
chr16	21311691	21311729
chr16	21312222	21312712
chr16	21312847	21313447
chr16	21313827	21314423
chr16	21314573	21314668
chr16	21374957	21375105
chr16	21375443	21375646
chr16	21376098	21376355
chr16	21386063	21386262
chr16	21386505	21386782
chr16	21386983	21386986
chr16	21400453	21400643
chr16	21409084	21409319
chr16	21419962	21420161
chr16	21422813	21423122
chr16	21423708	21423722
chr16	21425297	21425416
chr16	21425673	21425959
chr16	21429696	21429788
chr16	21431655	21431974
chr16	21432495	21432838

chr16	21435081	21435280
chr16	21435796	21436216
chr16	21436355	21436528
chr16	21437682	21438271
chr16	21438457	21438661
chr16	21439627	21439799
chr16	21445338	21445784
chr16	21445945	21446310
chr16	21451256	21451557
chr16	21452438	21452667
chr16	21452802	21453324
chr16	21455464	21455573
chr16	21455646	21455893
chr16	21460188	21460402
chr16	21479381	21479688
chr16	21488984	21489014
chr16	21490172	21490530
chr16	21494201	21494218
chr16	21494958	21495090
chr16	21504036	21504235
chr16	21507159	21507617
chr16	21507829	21507990
chr16	21508188	21508402
chr16	21509666	21510343
chr16	21512253	21513744
chr16	21513924	21515049
chr16	21515215	21516052
chr16	21516289	21516536
chr16	21516849	21517162
chr16	21517720	21518170
chr16	21518917	21519220
chr16	21519437	21519959
chr16	21520596	21520942
chr16	21521814	21525537
chr16	21526113	21526226
chr16	21526469	21526579
chr16	21534132	21534246
chr16	21526726	21534013
chr16	21536068	21536601
chr16	21536861	21537070
chr16	21537705	21537996
chr16	21539921	21540235
chr16	21541402	21541639
chr16	21543604	21543837
chr16	21545573	21545773
chr16	21551007	21551449

chr16	21555631	21555858
chr16	21557898	21558097
chr16	21559470	21559669
chr16	21564544	21564899
chr16	21565979	21566603
chr16	21573930	21574129
chr16	21579771	21580592
chr16	21582863	21582928
chr16	21584030	21584230
chr16	21585543	21585742
chr16	21588091	21588276
chr16	21595149	21595844
chr16	21602877	21603089
chr16	21609355	21609572
chr16	21614454	21614708
chr16	21609914	21612177
chr16	21616786	21617569
chr16	21619525	21619584
chr16	21622491	21623752
chr16	21627607	21627893
chr16	21630334	21630368
chr16	21630678	21630697
chr16	21632694	21632893
chr16	21637835	21638682
chr16	21640753	21640792
chr16	21642072	21642437
chr16	21643390	21643585
chr16	21643839	21644057
chr16	21644889	21645037
chr16	21649856	21650424
chr16	21650613	21650812
chr16	21651964	21652368
chr16	21661129	21661328
chr16	21662311	21662714
chr16	21664777	21664801
chr16	21665496	21665695
chr16	21668561	21668910
chr16	21672694	21672995
chr16	21708400	21708508
chr16	21713347	21713546
chr16	21720089	21720193
chr16	21740520	21740624
chr16	21745764	21745922
chr16	21747610	21747646
chr16	21760575	21760809
chr16	21761055	21761479

chr16	21762734	21762990
chr16	21766824	21766978
chr16	21768402	21768829
chr16	21768943	21769248
chr16	21770076	21770150
chr16	21770798	21770858
chr16	21777371	21777593
chr16	21779406	21779581
chr16	21779807	21782641
chr16	21783216	21783483
chr16	21783916	21784492
chr16	21786350	21786603
chr16	21787989	21787997
chr16	21790758	21790916
chr16	21791338	21791503
chr16	21792011	21792278
chr16	21792719	21792986
chr16	21795062	21795229
chr16	21795925	21796074
chr16	21796270	21796275
chr16	21801724	21802269
chr16	21803445	21803636
chr16	21804649	21804761
chr16	21805015	21805126
chr16	21812728	21813150
chr16	21813554	21813739
chr16	21814922	21815211
chr16	21815424	21815798
chr16	21816317	21816452
chr16	21831143	21832613
chr16	21833062	21833246
chr16	21836290	21836804
chr16	21837196	21837247
chr16	21839544	21839743
chr16	21841217	21841608
chr16	21843256	21843722
chr16	21857781	21857874
chr16	21858047	21858298
chr16	21859387	21859772
chr16	21861689	21861931
chr16	21868066	21868808
chr16	21869450	21869455
chr16	21869568	21869835
chr16	21870195	21870675
chr16	21870800	21870936
chr16	21871806	21872170

chr16	21873176	21873418
chr16	21875950	21876339
chr16	21877509	21877995
chr16	21878198	21878580
chr16	21879506	21879838
chr16	21883746	21883827
chr16	21884359	21884708
chr16	21885074	21885109
chr16	21887392	21887394
chr16	21887630	21887857
chr16	21888863	21889094
chr16	21892498	21892581
chr16	21894172	21894241
chr16	21897473	21897602
chr16	21898110	21898493
chr16	21903352	21903376
chr16	21903601	21903749
chr16	21919257	21919273
chr16	21921820	21922043
chr16	21923091	21923209
chr16	21924031	21924040
chr16	21926269	21926539
chr16	21930773	21931104
chr16	21932208	21932465
chr16	21933123	21933358
chr16	21934362	21934998
chr16	21935543	21935565
chr16	21935778	21936295
chr16	21937450	21937540
chr16	21938782	21939002
chr16	21939302	21939631
chr16	21941276	21941600
chr16	21945837	21946036
chr16	21946147	21946449
chr16	21948032	21948292
chr16	21950658	21950701
chr16	21958319	21958638
chr16	21960635	21960895
chr16	21961792	21962683
chr16	21963636	21965210
chr16	21971797	21971860
chr16	21990848	21991047
chr16	21992739	21992880
chr16	21996301	21996553
chr16	21997188	21997295
chr16	21999165	21999173

chr16	21999396	21999642
chr16	21999861	21999917
chr16	22006793	22007556
chr16	22009486	22009553
chr16	22009855	22010504
chr16	22011853	22012096
chr16	22012182	22012253
chr16	22012521	22012909
chr16	22013132	22013739
chr16	22018311	22021251
chr16	22029993	22030442
chr16	22033092	22033207
chr16	22037054	22037264
chr16	22037425	22038338
chr16	22039024	22039337
chr16	22043915	22044112
chr16	22044756	22044875
chr16	22056206	22056446
chr16	22057587	22057699
chr16	22058495	22058841
chr16	22062282	22062717
chr16	22073697	22073827
chr16	22076101	22077011
chr16	22081664	22083072
chr16	22086420	22086618
chr16	22089367	22089466
chr16	22108377	22108573
chr16	22109593	22109609
chr16	22109699	22109792
chr16	22110292	22110481
chr16	22111057	22111307
chr16	22179945	22180075
chr16	22181414	22181444
chr16	22193926	22194125
chr16	22196965	22197196
chr16	22198799	22198872
chr16	22199114	22199399
chr16	22199545	22199608
chr16	22200158	22200752
chr16	22201004	22201947
chr16	22203142	22203981
chr16	22204506	22204655
chr16	22202066	22203068
chr16	22205131	22205350
chr16	22206629	22207409
chr16	22207660	22208021

chr16	22208599	22208980
chr16	22209501	22209744
chr16	22209835	22211754
chr16	22212855	22213095
chr16	22213481	22213694
chr16	22216083	22216436
chr16	22216499	22219514
chr16	22220432	22220687
chr16	22220986	22221436
chr16	22222935	22223137
chr16	22224109	22224347
chr16	22224505	22224566
chr16	22225489	22225700
chr16	22228193	22228392
chr16	22229315	22229539
chr16	22231847	22232245
chr16	22235363	22235418
chr16	22235636	22235743
chr16	22237510	22237513
chr16	22240164	22240521
chr16	22241680	22242159
chr16	22242932	22243131
chr16	22243660	22244308
chr16	22246950	22247638
chr16	22247814	22248043
chr16	22248708	22248861
chr16	22250704	22250999
chr16	22252002	22252383
chr16	22252698	22253064
chr16	22253184	22253594
chr16	22253847	22254319
chr16	22254764	22255069
chr16	22258007	22258206
chr16	22260440	22260639
chr16	22260875	22260972
chr16	22267492	22267719
chr16	22281255	22281913
chr16	22299315	22299392
chr16	22306056	22306130
chr16	22306266	22306460
chr16	22307574	22308012
chr16	22308197	22308322
chr16	22308614	22310112
chr16	22310178	22310433
chr16	22311012	22311208
chr16	22311746	22311946

chr16	22312624	22312830
chr16	22313111	22313307
chr16	22317636	22317684
chr16	22321917	22321934
chr16	22323011	22323021
chr16	22323713	22323880
chr16	22325379	22327401
chr16	22330313	22330618
chr16	22332934	22333212
chr16	22341513	22341774
chr16	22342055	22342111
chr16	22342213	22342254
chr16	22357969	22357972
chr16	22359572	22359663
chr16	22359861	22360160
chr16	22363305	22363570
chr16	22365144	22365563
chr16	22365831	22365838
chr16	22369371	22369639
chr16	22377738	22377914
chr16	22378582	22378686
chr16	22379733	22380388
chr16	22380600	22381197
chr16	22381475	22382525
chr16	22382606	22382947
chr16	22383133	22383793
chr16	22384396	22384868
chr16	22384933	22386953
chr16	22388555	22388760
chr16	22389738	22389987
chr16	22394547	22394981
chr16	22395701	22395899
chr16	22396174	22396369
chr16	22398339	22398592
chr16	22408834	22409262
chr16	22411944	22412572
chr16	22414569	22415148
chr16	22413033	22413454
chr16	22419850	22419969
chr16	22424137	22424460
chr16	22425741	22425890
chr16	22426912	22427076
chr16	22428654	22429027
chr16	22429156	22429706
chr16	22443727	22443931
chr16	22447669	22449563

chr16	22450484	22450713
chr16	22451755	22451904
chr16	22453424	22454174
chr16	22454280	22454879
chr16	22457028	22457084
chr16	22458107	22458259
chr16	22459249	22459549
chr16	22459860	22460098
chr16	22460170	22460475
chr16	22461160	22461350
chr16	22473957	22474167
chr16	22483718	22483871
chr16	22495241	22495676
chr16	22498850	22499177
chr16	22501229	22501376
chr16	22505390	22505805
chr16	22506006	22506316
chr16	22507108	22507354
chr16	22507860	22508685
chr16	22508998	22509474
chr16	22515529	22515728
chr16	22515852	22516139
chr16	22517595	22517641
chr16	22517979	22518145
chr16	22522890	22523726
chr16	22523848	22524366
chr16	22525024	22525773
chr16	22526559	22526609
chr16	22529272	22529331
chr16	22532080	22532091
chr16	22532272	22532402
chr16	22532641	22532642
chr16	22534204	22534730
chr16	22535623	22535864
chr16	22536143	22536219
chr16	22538447	22538851
chr16	22540239	22540399
chr16	22547003	22547062
chr16	22549313	22549538
chr16	22550250	22550449
chr16	22551711	22551914
chr16	22553939	22554012
chr16	22554131	22554468
chr16	22555415	22555650
chr16	22561733	22562495
chr16	22563228	22563426

chr16	22564006	22564232
chr16	22586323	22586392
chr16	22586852	22587287
chr16	22590021	22590323
chr16	22593472	22593725
chr16	22596059	22597616
chr16	22597844	22598356
chr16	22598916	22599652
chr16	22601750	22602171
chr16	22605946	22605949
chr16	22606852	22607103
chr16	22607925	22608343
chr16	22608860	22608904
chr16	22609123	22609139
chr16	22609592	22609817
chr16	22612623	22612679
chr16	22616456	22616697
chr16	22617698	22618054
chr16	22618337	22618697
chr16	22691158	22691394
chr16	22691981	22692069
chr16	22692373	22692399
chr16	22696169	22696262
chr16	22732232	22732475
chr16	22801156	22801543
chr16	22960353	22960465
chr16	23082495	23083710
chr16	23084122	23084304
chr16	23084435	23084734
chr16	23091195	23091389
chr16	23093209	23093449
chr16	23121541	23122046
chr16	23130914	23131306
chr16	23135593	23136029
chr16	23136218	23136519
chr16	23153507	23154098
chr16	23156723	23157353
chr16	23158368	23159179
chr16	23159704	23160662
chr16	23160995	23161330
chr16	23164021	23164092
chr16	23182787	23182935
chr16	23183209	23183536
chr16	23269783	23269943
chr16	23270195	23270535
chr16	23291531	23291862

chr16	23294440	23295096
chr16	23297335	23297404
chr16	23373557	23373653
chr16	23434526	23434821
chr16	23434949	23435443
chr16	23435518	23436440
chr16	23463652	23464986
chr16	23481093	23481322
chr16	23481442	23481798
chr16	23502851	23503426
chr16	23503540	23504175
chr16	23504572	23504771
chr16	23508175	23508521
chr16	23508685	23508998
chr16	23509224	23509499
chr16	23509634	23509787
chr16	23510454	23510565
chr16	23512195	23512215
chr16	23512704	23512788
chr16	23513832	23514110
chr16	23515110	23515483
chr16	23515941	23516071
chr16	23520514	23521213
chr16	23521357	23522423
chr16	23549572	23549668
chr16	23549916	23550390
chr16	23551700	23552128
chr16	23558948	23559295
chr16	23559519	23559558
chr16	23563762	23563961
chr16	23567584	23567967
chr16	23568281	23569925
chr16	23600925	23601124
chr16	23606592	23606686
chr16	23606850	23608016
chr16	23608234	23608508
chr16	23608689	23608838
chr16	23610784	23610861
chr16	23613712	23613848
chr16	23619450	23619648
chr16	23619880	23619962
chr16	23622514	23622713
chr16	23640853	23641009
chr16	23652354	23652587
chr16	23652774	23653405
chr16	23675337	23675372

chr16	23689575	23691607
chr16	23692854	23693073
chr16	23735981	23736079
chr16	23739052	23739250
chr16	23746448	23746678
chr16	23765941	23766241
chr16	23779903	23780369
chr16	23781977	23782754
chr16	23782996	23783346
chr16	23792249	23792301
chr16	23792562	23792633
chr16	23792890	23792924
chr16	24515831	24516109
chr16	24519894	24520036
chr16	24520167	24520466
chr16	24528663	24528744
chr16	24535927	24536384
chr16	24537391	24537598
chr16	24537858	24538055
chr16	24538381	24539240
chr16	24547993	24548219
chr16	24550238	24551805
chr16	24588362	24588631
chr16	24588768	24588862
chr16	24592134	24592311
chr16	24596728	24596983
chr16	24601356	24601623
chr16	24621041	24621199
chr16	24621605	24622270
chr16	24631224	24631419
chr16	24637584	24637913
chr16	24647406	24647654
chr16	24648402	24648579
chr16	24657094	24657293
chr16	24657492	24657554
chr16	24657776	24658076
chr16	24679248	24679549
chr16	24680139	24680208
chr16	24680549	24681275
chr16	24684980	24685073
chr16	24690780	24691057
chr16	24691489	24691707
chr16	24692597	24693572
chr16	24694523	24695013
chr16	24695112	24695311
chr16	24697316	24697342

chr16	24697511	24697827
chr16	24712025	24713096
chr16	24721762	24721922
chr16	24732145	24732253
chr16	24733610	24733889
chr16	24734988	24735187
chr16	24735344	24735398
chr16	24740210	24741603
chr16	24746489	24746688
chr16	24748712	24748958
chr16	24759472	24759948
chr16	24783404	24783441
chr16	24845900	24846165
chr16	24849735	24850221
chr16	24850338	24850573
chr16	24855584	24855766
chr16	24860278	24860338
chr16	24862475	24863068
chr16	24906464	24906585
chr16	24906680	24906681
chr16	24917170	24917198
chr16	24917500	24917862
chr16	24977707	24977713
chr16	24986742	24987346
chr16	24998109	24998149
chr16	24999065	24999474
chr16	24999967	25000362
chr16	25005504	25005619
chr16	25005937	25006013
chr16	25008096	25008556
chr16	25023957	25023982
chr16	25024222	25024386
chr16	25025618	25027197
chr16	25037811	25038038
chr16	25038316	25038356
chr16	25043109	25043522
chr16	25044213	25044431
chr16	25059821	25059929
chr16	25060062	25060968
chr16	25061657	25061963
chr16	25062656	25062750
chr16	25062868	25063063
chr16	25063432	25063697
chr16	25063887	25064241
chr16	25067166	25067389
chr16	25073764	25073895

chr16	25077815	25077836
chr16	25077933	25078849
chr16	25117906	25118760
chr16	25121938	25122037
chr16	25122244	25122789
chr16	25123049	25123672
chr16	25123798	25123909
chr16	25137831	25138053
chr16	25236819	25237175
chr16	25245012	25245137
chr16	25267932	25268696
chr16	25268812	25269900
chr16	27150346	27150721
chr16	27187990	27188278
chr16	27207003	27207251
chr16	27207549	27207914
chr16	27214613	27215707
chr16	27215987	27216316
chr16	27216841	27216855
chr16	27247587	27247671
chr16	27257080	27257570
chr16	27257893	27258100
chr16	27259417	27259517
chr16	27265783	27266047
chr16	27266399	27266529
chr16	27269730	27270204
chr16	27275394	27275594
chr16	27275769	27275892
chr16	27279842	27280006
chr16	27280111	27280366
chr16	27295329	27295532
chr16	27297350	27298426
chr16	27314363	27314409
chr16	27324244	27324367
chr16	27324562	27326161
chr16	27329571	27330980
chr16	27331571	27332994
chr16	27334172	27334503
chr16	27326259	27329288
chr16	27334648	27336974
chr16	27337572	27338268
chr16	27338425	27339161
chr16	27343392	27343626
chr16	27348262	27348500
chr16	27349697	27349729
chr16	27350966	27351514

chr16	27371455	27371694
chr16	27371988	27372284
chr16	27372456	27372805
chr16	27375297	27375557
chr16	27375763	27376026
chr16	27376192	27376333
chr16	27377168	27377633
chr16	27378443	27379606
chr16	27387000	27387232
chr16	27443356	27443374
chr16	27443903	27444147
chr16	27466805	27466862
chr16	27466953	27467212
chr16	27469506	27469544
chr16	27472489	27472864
chr16	27521541	27521743
chr16	27524611	27524839
chr16	27532288	27532381
chr16	27552253	27552570
chr16	27558406	27558675
chr16	27558851	27558994
chr16	27560194	27561249
chr16	27561437	27561918
chr16	27562330	27562567
chr16	27564151	27564301
chr16	27582459	27582577
chr16	27589641	27590501
chr16	27590610	27591360
chr16	27615762	27615959
chr16	27670280	27670331
chr16	28094358	28094557
chr16	28100878	28101143
chr16	28103651	28104045
chr16	28104257	28104636
chr16	28127595	28127824
chr16	28152408	28152659
chr16	28152867	28152914
chr16	28153368	28153691
chr16	28153960	28154064
chr16	28167355	28167416
chr16	28178096	28178218
chr16	28178819	28178914
chr16	28184769	28184928
chr16	28192744	28193300
chr16	28193542	28193792
chr16	28197954	28198485

chr16	28199933	28200116
chr16	28200828	28201131
chr16	28202658	28203073
chr16	28203212	28203468
chr16	28203762	28203977
chr16	28204191	28204401
chr16	28205577	28206285
chr16	28212867	28213035
chr16	28221134	28221715
chr16	28221938	28224227
chr16	28224406	28224711
chr16	28275804	28276004
chr16	28289415	28289681
chr16	28301455	28301654
chr16	28302950	28303417
chr16	28303889	28304184
chr16	28328193	28328402
chr16	28340215	28340599
chr16	28340814	28341238
chr16	28348816	28349104
chr16	28353122	28353321
chr16	28359716	28359874
chr16	28363667	28363883
chr16	28364937	28365136
chr16	28366844	28367068
chr16	28373638	28373826
chr16	28374257	28374288
chr16	28375724	28376246
chr16	28382513	28383701
chr16	28384335	28384514
chr16	28384804	28385461
chr16	28385768	28386507
chr16	28386729	28386968
chr16	28393613	28393841
chr16	28394047	28394272
chr16	28414425	28415166
chr16	28415269	28415462
chr16	28415759	28416032
chr16	28416762	28416835
chr16	28429854	28430105
chr16	28430218	28430303
chr16	28431043	28431271
chr16	28432161	28433154
chr16	28433555	28434021
chr16	28434800	28434889
chr16	28436453	28436641

chr16	28436904	28437279
chr16	28437389	28437788
chr16	28438021	28438157
chr16	28449322	28449543
chr16	28455180	28455283
chr16	28456154	28456400
chr16	28470523	28470788
chr16	28477058	28477148
chr16	28500909	28501229
chr16	28501813	28503839
chr16	28504720	28504748
chr16	28511776	28511975
chr16	28515525	28515692
chr16	28515990	28516189
chr16	28516719	28517100
chr16	28538470	28538710
chr16	28544253	28544553
chr16	28544971	28545607
chr16	28545749	28546239
chr16	28546523	28546722
chr16	28547416	28547575
chr16	28547770	28547857
chr16	28548320	28549849
chr16	28550109	28550909
chr16	28551420	28551664
chr16	28551866	28552089
chr16	28554817	28554899
chr16	28560959	28561038
chr16	28563846	28564111
chr16	28564964	28565394
chr16	28565727	28566032
chr16	28566186	28566298
chr16	28568042	28568088
chr16	28598213	28598267
chr16	28598555	28598576
chr16	28599135	28599348
chr16	28607654	28607892
chr16	28608005	28608301
chr16	28608519	28608726
chr16	28610007	28610141
chr16	28610452	28610706
chr16	28611278	28611741
chr16	28623321	28623731
chr16	28624466	28624673
chr16	28627170	28627640
chr16	28628045	28628827

chr16	28629496	28629603
chr16	28633343	28633455
chr16	28633813	28635216
chr16	28641881	28642113
chr16	28642273	28642342
chr16	28646239	28646585
chr16	28647467	28648586
chr16	28648806	28649100
chr16	28649576	28650333
chr16	28651759	28651793
chr16	28660063	28660307
chr16	28660479	28660628
chr16	28662246	28662417
chr16	28666952	28667103
chr16	28672223	28672480
chr16	28673573	28673886
chr16	28677047	28677246
chr16	28678229	28678299
chr16	28680376	28680397
chr16	28688953	28689152
chr16	28699883	28700391
chr16	28700475	28700513
chr16	28700948	28701162
chr16	28702567	28703076
chr16	28705425	28706297
chr16	28704488	28705280
chr16	28721866	28722181
chr16	28722371	28723356
chr16	28721167	28721217
chr16	28740998	28741197
chr16	28741277	28741854
chr16	28741968	28741976
chr16	28742159	28742167
chr16	28742537	28743300
chr16	28743417	28743622
chr16	28743698	28744245
chr16	28747286	28747485
chr16	28750627	28750826
chr16	28751658	28751929
chr16	28752082	28752634
chr16	28753984	28754313
chr16	28754455	28755442
chr16	28757106	28757269
chr16	28757604	28757882
chr16	28761718	28762312
chr16	28764009	28764298

chr16	28774606	28774707
chr16	28775189	28775405
chr16	28805314	28805613
chr16	28812562	28812597
chr16	28813717	28813856
chr16	28814078	28814907
chr16	28815147	28815632
chr16	28817765	28818094
chr16	28820422	28820851
chr16	28818635	28819524
chr16	28821500	28821728
chr16	28822107	28822368
chr16	28826910	28827241
chr16	28833377	28836318
chr16	28837449	28837775
chr16	28838771	28839006
chr16	28840165	28840256
chr16	28848778	28848933
chr16	28850589	28850788
chr16	28856245	28856444
chr16	28856788	28857697
chr16	28857882	28858736
chr16	28859380	28859645
chr16	28860280	28860287
chr16	28866490	28866761
chr16	28869155	28870396
chr16	28870986	28871080
chr16	28873816	28876021
chr16	28881975	28882174
chr16	28890634	28892148
chr16	28910314	28910532
chr16	28912679	28912914
chr16	28921030	28921220
chr16	28921425	28921536
chr16	28921630	28922211
chr16	28923268	28923385
chr16	28927289	28927476
chr16	28930390	28930571
chr16	28932070	28932456
chr16	28935104	28936087
chr16	28936231	28937550
chr16	28938940	28939139
chr16	28956266	28956382
chr16	28956485	28957494
chr16	28959052	28959940
chr16	28960011	28960058

chr16	28961643	28961979
chr16	28962259	28963371
chr16	28964354	28964368
chr16	28965073	28965191
chr16	28985205	28985969
chr16	28986266	28986641
chr16	28987358	28987472
chr16	28990865	28991064
chr16	29034556	29034723
chr16	29048273	29048472
chr16	29054602	29054658
chr16	29079856	29079997
chr16	29086585	29086784
chr16	29093233	29093786
chr16	29106025	29106201
chr16	29106418	29106822
chr16	29156710	29157211
chr16	29167379	29168026
chr16	29168644	29168899
chr16	29169034	29169349
chr16	29262959	29263478
chr16	29272726	29273106
chr16	29273435	29273658
chr16	29275304	29275646
chr16	29301876	29302263
chr16	29302535	29303178
chr16	29303348	29303547
chr16	29311019	29311386
chr16	29364114	29364193
chr16	29365726	29366159
chr16	29379140	29379513
chr16	29388304	29388374
chr16	29404317	29404363
chr16	29414631	29415249
chr16	29415587	29415778
chr16	29415906	29416755
chr16	29417104	29417283
chr16	29424094	29424385
chr16	29425983	29426182
chr16	29429991	29430236
chr16	29431063	29431109
chr16	29431425	29431515
chr16	29433473	29433546
chr16	29451590	29451898
chr16	29459341	29459417
chr16	29459592	29459824

chr16	29461358	29462003
chr16	29462548	29463731
chr16	29463854	29464195
chr16	29464350	29466647
chr16	29466829	29467073
chr16	29467365	29468643
chr16	29469329	29472729
chr16	29472897	29473284
chr16	29473642	29474260
chr16	29477783	29477986
chr16	29478147	29478491
chr16	29478602	29480426
chr16	29480620	29482219
chr16	29482406	29482863
chr16	29482998	29483359
chr16	29483597	29483782
chr16	29484495	29484754
chr16	29492079	29492558
chr16	29493195	29493433
chr16	29505819	29505920
chr16	29513049	29513148
chr16	29516268	29517030
chr16	29517806	29518111
chr16	29518301	29519100
chr16	29520104	29520516
chr16	29524038	29524211
chr16	29525243	29525285
chr16	29525608	29526369
chr16	29527829	29527954
chr16	29530465	29530642
chr16	29533253	29533402
chr16	29533690	29534253
chr16	29535203	29535277
chr16	29535784	29536397
chr16	29541880	29542174
chr16	29547133	29547425
chr16	29547792	29548150
chr16	29558899	29559007
chr16	29588025	29588291
chr16	29589812	29590011
chr16	29599360	29599756
chr16	29603121	29603394
chr16	29603681	29603880
chr16	29607941	29608061
chr16	29605432	29607587
chr16	29608662	29608994

chr16	29609676	29609747
chr16	29609900	29610456
chr16	29611645	29612140
chr16	29614852	29615437
chr16	29615989	29616613
chr16	29616811	29617033
chr16	29619450	29619748
chr16	29620817	29621158
chr16	29621357	29626000
chr16	29626790	29627130
chr16	29690177	29690696
chr16	29691057	29691516
chr16	29691815	29691854
chr16	29706933	29706981
chr16	29707088	29707161
chr16	29712117	29712296
chr16	29721558	29721764
chr16	29762619	29762814
chr16	29781537	29781816
chr16	29787062	29787254
chr16	29791112	29791339
chr16	29795431	29795685
chr16	29797717	29797911
chr16	29800171	29800178
chr16	29800913	29801904
chr16	29802030	29802793
chr16	29807481	29807828
chr16	29815661	29817305
chr16	29817990	29818217
chr16	29818427	29820290
chr16	29821903	29822486
chr16	29822806	29823759
chr16	29826487	29826711
chr16	29826853	29830079
chr16	29830845	29831360
chr16	29833932	29834218
chr16	29831703	29833518
chr16	29834911	29836075
chr16	29836214	29836955
chr16	29839150	29839436
chr16	29840540	29840724
chr16	29844230	29844429
chr16	29854538	29854737
chr16	29863742	29863939
chr16	29873233	29874857
chr16	29875036	29875896

chr16	29880605	29880702
chr16	29886088	29886107
chr16	29893645	29893651
chr16	29909733	29909960
chr16	29910114	29910871
chr16	29911088	29912565
chr16	29921208	29921357
chr16	29929985	29930201
chr16	29936436	29938215
chr16	29955137	29955995
chr16	29956309	29956539
chr16	29972648	29974359
chr16	29975379	29975654
chr16	29976935	29977332
chr16	29980566	29980810
chr16	29984638	29984817
chr16	29985101	29985652
chr16	29985720	29986296
chr16	29987096	29987128
chr16	29988543	29988809
chr16	30005208	30006279
chr16	30006391	30008546
chr16	30008938	30009161
chr16	30022079	30022562
chr16	30022705	30022904
chr16	30025919	30026024
chr16	30026803	30027002
chr16	30032034	30033077
chr16	30033645	30034109
chr16	30050535	30050733
chr16	30051288	30051487
chr16	30056399	30056760
chr16	30057499	30057711
chr16	30063736	30064386
chr16	30064524	30066168
chr16	30066484	30066713
chr16	30066976	30067066
chr16	30067878	30068096
chr16	30068322	30068532
chr16	30072802	30073062
chr16	30074089	30074357
chr16	30074893	30076889
chr16	30077097	30078748
chr16	30079029	30079233
chr16	30079795	30079912
chr16	30082334	30082672

chr16	30083133	30083413
chr16	30083582	30083781
chr16	30084104	30084570
chr16	30084821	30084934
chr16	30085563	30085635
chr16	30086151	30088047
chr16	30092749	30092954
chr16	30099937	30100187
chr16	30100667	30101747
chr16	30101888	30104238
chr16	30104578	30104811
chr16	30106423	30106977
chr16	30107095	30108184
chr16	30115621	30116013
chr16	30116173	30116282
chr16	30124317	30124770
chr16	30133591	30135303
chr16	30135488	30135793
chr16	30135931	30136151
chr16	30136403	30136674
chr16	30145999	30146073
chr16	30152097	30152231
chr16	30154319	30154518
chr16	30169909	30170353
chr16	30172012	30172288
chr16	30185158	30185363
chr16	30186121	30186313
chr16	30188336	30188535
chr16	30189243	30189418
chr16	30194197	30194862
chr16	30195048	30196059
chr16	30200992	30201266
chr16	30202032	30202324
chr16	30202555	30205006
chr16	30205241	30207900
chr16	30208197	30208974
chr16	30209313	30212107
chr16	30212957	30213613
chr16	30215356	30215710
chr16	30217142	30222278
chr16	30222406	30223050
chr16	30223566	30223918
chr16	30224697	30224953
chr16	30228031	30228446
chr16	30228836	30229170
chr16	30231494	30231918

chr16	30239008	30239176
chr16	30241937	30242184
chr16	30245143	30245365
chr16	30245783	30245828
chr16	30245941	30246338
chr16	30247928	30248237
chr16	30255150	30255331
chr16	30256275	30257062
chr16	30257710	30258173
chr16	30258264	30259099
chr16	30259996	30260578
chr16	30263782	30263809
chr16	30263920	30264060
chr16	30265581	30266199
chr16	30266729	30266874
chr16	30267694	30268048
chr16	30272748	30272947
chr16	30273165	30273944
chr16	30274716	30274902
chr16	30275131	30276180
chr16	30278412	30278428
chr16	30282887	30283094
chr16	30287155	30287419
chr16	30287785	30288311
chr16	30301007	30301395
chr16	30320871	30320955
chr16	30345493	30347427
chr16	30365723	30366675
chr16	30366790	30368109
chr16	30373887	30374060
chr16	30378865	30379054
chr16	30379688	30380611
chr16	30380725	30381900
chr16	30382396	30383215
chr16	30383653	30383852
chr16	30384766	30384997
chr16	30388010	30390757
chr16	30391852	30392051
chr16	30394158	30394357
chr16	30405693	30407882
chr16	30408016	30408409
chr16	30410734	30410965
chr16	30411281	30412437
chr16	30415719	30415950
chr16	30418234	30418778
chr16	30418909	30419453

chr16	30420239	30420343
chr16	30420518	30420649
chr16	30421688	30421773
chr16	30424873	30425622
chr16	30426868	30427058
chr16	30428736	30429852
chr16	30430259	30430485
chr16	30437306	30437652
chr16	30437719	30438104
chr16	30440306	30441315
chr16	30441725	30442193
chr16	30445937	30446039
chr16	30448663	30448709
chr16	30456495	30457184
chr16	30457371	30457728
chr16	30461391	30461462
chr16	30476456	30476727
chr16	30479885	30480196
chr16	30480592	30481381
chr16	30483808	30484192
chr16	30497516	30497549
chr16	30499932	30500019
chr16	30501024	30501302
chr16	30501464	30502091
chr16	30502330	30502693
chr16	30503284	30504067
chr16	30508860	30508890
chr16	30508957	30509094
chr16	30519770	30520493
chr16	30540045	30540187
chr16	30537366	30539960
chr16	30541332	30541579
chr16	30542035	30542234
chr16	30543222	30543544
chr16	30544525	30546320
chr16	30546430	30546794
chr16	30546956	30547130
chr16	30547262	30547603
chr16	30560922	30561452
chr16	30565782	30566042
chr16	30568506	30569648
chr16	30570019	30570176
chr16	30578376	30580409
chr16	30582273	30584031
chr16	30590700	30590899
chr16	30595184	30595436

chr16	30595681	30596033
chr16	30596157	30597656
chr16	30599827	30600049
chr16	30603154	30603398
chr16	30609420	30609502
chr16	30609789	30609875
chr16	30610049	30610248
chr16	30617680	30617715
chr16	30622281	30622604
chr16	30620472	30622154
chr16	30638036	30638336
chr16	30644708	30644947
chr16	30645094	30645662
chr16	30645839	30646483
chr16	30646827	30648300
chr16	30648452	30648585
chr16	30648867	30649450
chr16	30656324	30656495
chr16	30661142	30663720
chr16	30664904	30664942
chr16	30665159	30665181
chr16	30667932	30669485
chr16	30669646	30670073
chr16	30672171	30672264
chr16	30670250	30672099
chr16	30674445	30674459
chr16	30675336	30675535
chr16	30683189	30683373
chr16	30690148	30690405
chr16	30694470	30694483
chr16	30698433	30698832
chr16	30702791	30702846
chr16	30705106	30705837
chr16	30708796	30709255
chr16	30709399	30711065
chr16	30719783	30720038
chr16	30724724	30725077
chr16	30725268	30725504
chr16	30726548	30727485
chr16	30729696	30729954
chr16	30730669	30731077
chr16	30742842	30743171
chr16	30743358	30743602
chr16	30750917	30753412
chr16	30757586	30757724
chr16	30758226	30758466

chr16	30758628	30759380
chr16	30759681	30760873
chr16	30772194	30772475
chr16	30772752	30773303
chr16	30773529	30774367
chr16	30774630	30774785
chr16	30782011	30782259
chr16	30786835	30788084
chr16	30797382	30799797
chr16	30801576	30801715
chr16	30807640	30807726
chr16	30808024	30808223
chr16	30812642	30812841
chr16	30816006	30816015
chr16	30816432	30816765
chr16	30816949	30817969
chr16	30818271	30819116
chr16	30825125	30825703
chr16	30825950	30826117
chr16	30833858	30833884
chr16	30834079	30834111
chr16	30835520	30836080
chr16	30836426	30836553
chr16	30856237	30856735
chr16	30861582	30861636
chr16	30864952	30865218
chr16	30865360	30865566
chr16	30865817	30866439
chr16	30866913	30867199
chr16	30868359	30868775
chr16	30874445	30874475
chr16	30884785	30885134
chr16	30885463	30887083
chr16	30887311	30888218
chr16	30888712	30889023
chr16	30889710	30889977
chr16	30891999	30892199
chr16	30892516	30892793
chr16	30895958	30896157
chr16	30903174	30903460
chr16	30904532	30906618
chr16	30906710	30908768
chr16	30909421	30909710
chr16	30912071	30912281
chr16	30912929	30914476
chr16	30914681	30914880

chr16	30915075	30915396
chr16	30919416	30919540
chr16	30924001	30924239
chr16	30927484	30927595
chr16	30932452	30933826
chr16	30934136	30934623
chr16	30934796	30936428
chr16	30937001	30937200
chr16	30944244	30944579
chr16	30945365	30945527
chr16	30945860	30946145
chr16	30948109	30948200
chr16	30949537	30949827
chr16	30950783	30951988
chr16	30952438	30952771
chr16	30953566	30953845
chr16	30959811	30961476
chr16	30961689	30962385
chr16	30962879	30963445
chr16	30965322	30965676
chr16	30967256	30967281
chr16	30967483	30967741
chr16	30968496	30970093
chr16	30972307	30972494
chr16	30996413	30997092
chr16	31005315	31005917
chr16	31007496	31010037
chr16	31006135	31007149
chr16	31011871	31012103
chr16	31012377	31013154
chr16	31013701	31013900
chr16	31022108	31022439
chr16	31042070	31042332
chr16	31042903	31043456
chr16	31043840	31044654
chr16	31044800	31046009
chr16	31063268	31063630
chr16	31067943	31068188
chr16	31075982	31076415
chr16	31081628	31081683
chr16	31082211	31082260
chr16	31082519	31082739
chr16	31082972	31083072
chr16	31083552	31086193
chr16	31101358	31101614
chr16	31104672	31106950

chr16	31116636	31116759
chr16	31117149	31117549
chr16	31117810	31118253
chr16	31119276	31120775
chr16	31125001	31125427
chr16	31126740	31127032
chr16	31127280	31127761
chr16	31128287	31130874
chr16	31132436	31132613
chr16	31133002	31133201
chr16	31133791	31133830
chr16	31136524	31136850
chr16	31136913	31136948
chr16	31139222	31139421
chr16	31139710	31139909
chr16	31141686	31143191
chr16	31143434	31143786
chr16	31145701	31147512
chr16	31147849	31148394
chr16	31149036	31149394
chr16	31149521	31152522
chr16	31152648	31154516
chr16	31148497	31148911
chr16	31155732	31155969
chr16	31159679	31160047
chr16	31171416	31171499
chr16	31178411	31178610
chr16	31190025	31190265
chr16	31190460	31192869
chr16	31195373	31195392
chr16	31195778	31195824
chr16	31202001	31202267
chr16	31204031	31204213
chr16	31211821	31212209
chr16	31212607	31213314
chr16	31213445	31214009
chr16	31226383	31226826
chr16	31233468	31233667
chr16	31264672	31264750
chr16	31265169	31265368
chr16	31276280	31276582
chr16	31277590	31277663
chr16	31279032	31279301
chr16	31281584	31281955
chr16	31286306	31286526
chr16	31397348	31397571

chr16	31415332	31415572
chr16	31416245	31416272
chr16	31438807	31439233
chr16	31439742	31439956
chr16	31446619	31446854
chr16	31453785	31454497
chr16	31454669	31454793
chr16	31455783	31455843
chr16	31462969	31463088
chr16	31463213	31463682
chr16	31463811	31465722
chr16	31466412	31466746
chr16	31467212	31467668
chr16	31467809	31467853
chr16	31468541	31469159
chr16	31469472	31471230
chr16	31471430	31471732
chr16	31471830	31472175
chr16	31482552	31482653
chr16	31482764	31482907
chr16	31482994	31484117
chr16	31484313	31484612
chr16	31498213	31498960
chr16	31499238	31499437
chr16	31499651	31500040
chr16	31517965	31518189
chr16	31518650	31518868
chr16	31518985	31520527
chr16	31711593	31711736
chr16	31711899	31712759
chr16	31713418	31713747
chr16	31724561	31725340
chr16	31884870	31884877
chr16	31885070	31885750
chr16	31885876	31886247
chr16	31886465	31886692
chr16	31888790	31889141
chr16	32358064	32358160
chr16	32468725	32468862
chr16	33237264	33237675
chr16	33237898	33238097
chr16	33239245	33239583
chr16	33294031	33294309
chr16	33382408	33382500
chr16	33409450	33409649
chr16	33414601	33414647

chr16	33865243	33865484
chr16	33866340	33866540
chr16	33873913	33874242
chr16	33887476	33887849
chr16	33887972	33887983
chr16	33888346	33888561
chr16	33894217	33894403
chr16	33895731	33896023
chr16	33896441	33896650
chr16	33899454	33900066
chr16	33902429	33902484
chr16	33934075	33934204
chr16	33934560	33934853
chr16	33945990	33946544
chr16	33952994	33953388
chr16	33955191	33955390
chr16	33955600	33955935
chr16	33957151	33957396
chr16	33958612	33958883
chr16	33959193	33959409
chr16	33960225	33960837
chr16	33963069	33963436
chr16	33963813	33964015
chr16	33967285	33967299
chr16	33972219	33972493
chr16	33972884	33973951
chr16	33974173	33976113
chr16	34021362	34021712
chr16	34022044	34022369
chr16	34022517	34022931
chr16	34188900	34189149
chr16	34368787	34368899
chr16	34369252	34369315
chr16	34530792	34531305
chr16	34532792	34532995
chr16	34533678	34533900
chr16	34539775	34540995
chr16	34541628	34541705
chr16	34543443	34543451
chr16	34597729	34597884
chr16	34598936	34599053
chr16	34599761	34599915
chr16	34600490	34600648
chr16	35239483	35239852
chr16	46410546	46411073
chr16	46411189	46414590

chr16	46385753	46410254
chr16	46414749	46415228
chr16	46415536	46415786
chr16	46416555	46418080
chr16	46418203	46418569
chr16	46418903	46430064
chr16	46431018	46431894
chr16	46431983	46436029
chr16	46449575	46449810
chr16	46455817	46456063
chr16	46499896	46500117
chr16	46607247	46607623
chr16	46654645	46655261
chr16	46655424	46655575
chr16	46657446	46657662
chr16	46707260	46707360
chr16	46709708	46710084
chr16	46722052	46724308
chr16	46724801	46725000
chr16	46738795	46738866
chr16	46809140	46809323
chr16	46823682	46823811
chr16	46824015	46824214
chr16	46864111	46865903
chr16	46898066	46898578
chr16	46917306	46918934
chr16	46919072	46919271
chr16	46919480	46919682
chr16	46921263	46921462
chr16	46925450	46925584
chr16	46929384	46929486
chr16	46983140	46983188
chr16	47006412	47008035
chr16	47008312	47008546
chr16	47037222	47037537
chr16	47041239	47041373
chr16	47071357	47072084
chr16	47077021	47077220
chr16	47170005	47170675
chr16	47171582	47171781
chr16	47171934	47172188
chr16	47173595	47173815
chr16	47175576	47175833
chr16	47175973	47176109
chr16	47176426	47178413
chr16	47481166	47481197

chr16	47490225	47490437
chr16	47494365	47495462
chr16	47495540	47496159
chr16	47509790	47509991
chr16	47538503	47538637
chr16	47538971	47538989
chr16	47599650	47599785
chr16	47607953	47608581
chr16	47608754	47608902
chr16	47766809	47767047
chr16	47881328	47881543
chr16	47882281	47882318
chr16	48049039	48049144
chr16	48190891	48191059
chr16	48198989	48199663
chr16	48199906	48199955
chr16	48215628	48215768
chr16	48228665	48229162
chr16	48229456	48229576
chr16	48277051	48279041
chr16	48325214	48325611
chr16	48329424	48329772
chr16	48351627	48351800
chr16	48380843	48381009
chr16	48386764	48386894
chr16	48387082	48387386
chr16	48387469	48387889
chr16	48391144	48391157
chr16	48392437	48392718
chr16	48399554	48400797
chr16	48403274	48403592
chr16	48403861	48404070
chr16	48404484	48404755
chr16	48404924	48405334
chr16	48417980	48420040
chr16	48424694	48424893
chr16	48427796	48427866
chr16	48429289	48429488
chr16	48429591	48429645
chr16	48430677	48431364
chr16	48431935	48431966
chr16	48431561	48431778
chr16	48432091	48432226
chr16	48443840	48444705
chr16	48445050	48445370
chr16	48447646	48447879

chr16	48472006	48472364
chr16	48472726	48472797
chr16	48478412	48479706
chr16	48492118	48492672
chr16	48495858	48496082
chr16	48496351	48496556
chr16	48513886	48514241
chr16	48526178	48526468
chr16	48532418	48532639
chr16	48532812	48532979
chr16	48533043	48533175
chr16	48533559	48533594
chr16	48533852	48534384
chr16	48536397	48536675
chr16	48539470	48539565
chr16	48546409	48546692
chr16	48544893	48546308
chr16	48560629	48560960
chr16	48569607	48571138
chr16	48571246	48572015
chr16	48586321	48586481
chr16	48590758	48591097
chr16	48592208	48592313
chr16	48619483	48619788
chr16	48640838	48641498
chr16	48641648	48641889
chr16	48642158	48642934
chr16	48644703	48644920
chr16	48645389	48645849
chr16	48643019	48644386
chr16	48645980	48646184
chr16	48646266	48646742
chr16	48647014	48647971
chr16	48656908	48657150
chr16	48657632	48657831
chr16	48658149	48658348
chr16	48664195	48664394
chr16	49057316	49057506
chr16	49151208	49151357
chr16	49888531	49888796
chr16	49889066	49889434
chr16	49889600	49890094
chr16	49891395	49891618
chr16	49914710	49914909
chr16	49994398	49994597
chr16	50058716	50058838

chr16	50059101	50059605
chr16	50059731	50059984
chr16	50097361	50097499
chr16	50098489	50098635
chr16	50099551	50099802
chr16	50099938	50100887
chr16	50101157	50101544
chr16	50102175	50102388
chr16	50122605	50122652
chr16	50130441	50130803
chr16	50130907	50131027
chr16	50133854	50134458
chr16	50136256	50136282
chr16	50136553	50136657
chr16	50137655	50138092
chr16	50147974	50148730
chr16	50185809	50186008
chr16	50186179	50186268
chr16	50186392	50187160
chr16	50187562	50188269
chr16	50188439	50188617
chr16	50188756	50189110
chr16	50200745	50201087
chr16	50202576	50202793
chr16	50204009	50204336
chr16	50205730	50205828
chr16	50215320	50215364
chr16	50215902	50216142
chr16	50227355	50227551
chr16	50227764	50227925
chr16	50229242	50229613
chr16	50230713	50231070
chr16	50235227	50236007
chr16	50236810	50237202
chr16	50242283	50242332
chr16	50243472	50243746
chr16	50244126	50244187
chr16	50244257	50244325
chr16	50268731	50269068
chr16	50277976	50278091
chr16	50278673	50280740
chr16	50300399	50300834
chr16	50335308	50335692
chr16	50394291	50394331
chr16	50401182	50401381
chr16	50402098	50402805

chr16	50403032	50403202
chr16	50409416	50409579
chr16	50429823	50431605
chr16	50434014	50434228
chr16	50500994	50501193
chr16	50501881	50502534
chr16	50537884	50538242
chr16	50538372	50538721
chr16	50564233	50564577
chr16	50566387	50566625
chr16	50571406	50571603
chr16	50571884	50571930
chr16	50572374	50572671
chr16	50581144	50581294
chr16	50581705	50581907
chr16	50582289	50583298
chr16	50690941	50691208
chr16	50698947	50699885
chr16	50720345	50720389
chr16	50721013	50721212
chr16	50721978	50722234
chr16	50722450	50722540
chr16	50722767	50723101
chr16	50726390	50728311
chr16	50729580	50729730
chr16	50729930	50732607
chr16	50733203	50734301
chr16	50752703	50752772
chr16	50752884	50752959
chr16	50753257	50754070
chr16	50775145	50775557
chr16	50775941	50776696
chr16	50776831	50776922
chr16	50778599	50779195
chr16	50781715	50781863
chr16	50790431	50790545
chr16	50797173	50797526
chr16	50840300	50840570
chr16	50959590	50959828
chr16	50960234	50960242
chr16	53056623	53056689
chr16	53070716	53071105
chr16	53075436	53076734
chr16	53086334	53087506
chr16	53088225	53090252
chr16	53092405	53092773

chr16	53094571	53094770
chr16	53113274	53113615
chr16	53113763	53114743
chr16	53114890	53115365
chr16	53117863	53118652
chr16	53119347	53119852
chr16	53120118	53120445
chr16	53116541	53117416
chr16	53122703	53122788
chr16	53124256	53124368
chr16	53125479	53125705
chr16	53127869	53128119
chr16	53128248	53129100
chr16	53132353	53134269
chr16	53141151	53141318
chr16	53145481	53145591
chr16	53163856	53166053
chr16	53176687	53176802
chr16	53176998	53177107
chr16	53241641	53242554
chr16	53291039	53291252
chr16	53406969	53407900
chr16	53412513	53412518
chr16	53449311	53449418
chr16	53450859	53450941
chr16	53452311	53452720
chr16	53468128	53469478
chr16	53469679	53469695
chr16	53516158	53516389
chr16	53536082	53536395
chr16	53536599	53537201
chr16	53537583	53537782
chr16	53537938	53538766
chr16	53584157	53584274
chr16	53737388	53737906
chr16	53738205	53738267
chr16	53778434	53778692
chr16	53779990	53780138
chr16	53798253	53798303
chr16	53802868	53802954
chr16	53815077	53815206
chr16	53818547	53818618
chr16	53851212	53851411
chr16	53922319	53922665
chr16	53926007	53926088
chr16	53926284	53926406

chr16	53928003	53928124
chr16	53928254	53928387
chr16	54111418	54111786
chr16	54114348	54114500
chr16	54114965	54115016
chr16	54300607	54300751
chr16	54318465	54318791
chr16	54319016	54321708
chr16	54376126	54376850
chr16	54377192	54377227
chr16	54880687	54880712
chr16	54880938	54881578
chr16	54962505	54962998
chr16	54963739	54964101
chr16	55543104	55543769
chr16	55586495	55586938
chr16	55589405	55589880
chr16	55591125	55591505
chr16	55601291	55601690
chr16	55602029	55602432
chr16	55689706	55689966
chr16	55697739	55697767
chr16	55698023	55698351
chr16	55698615	55699002
chr16	55701752	55702318
chr16	55702607	55703029
chr16	55786860	55787180
chr16	55787366	55787634
chr16	56225356	56225592
chr16	56359013	56359325
chr16	56364528	56365187
chr16	56367192	56367397
chr16	56368795	56369668
chr16	56371545	56371746
chr16	56372168	56372430
chr16	56376664	56377096
chr16	56377206	56377492
chr16	56379142	56379341
chr16	56390551	56391491
chr16	56443103	56443562
chr16	56446445	56446562
chr16	56447295	56447418
chr16	56453199	56453634
chr16	56458253	56459320
chr16	56459503	56459846
chr16	56477299	56477396

chr16	56484689	56485287
chr16	56485400	56486503
chr16	56486689	56486758
chr16	56510932	56511414
chr16	56548983	56549237
chr16	56553279	56554359
chr16	56554606	56554705
chr16	56580864	56581595
chr16	56581738	56581961
chr16	56582078	56582462
chr16	56610709	56610820
chr16	56611337	56611360
chr16	56634348	56634725
chr16	56637453	56637880
chr16	56638149	56638279
chr16	56639584	56644635
chr16	56645007	56645069
chr16	56645282	56645370
chr16	56645437	56645597
chr16	56647686	56648042
chr16	56648397	56648898
chr16	56649215	56649235
chr16	56649386	56649776
chr16	56658828	56661713
chr16	56662358	56662579
chr16	56677177	56677515
chr16	56691198	56691400
chr16	56691625	56691955
chr16	56706396	56706424
chr16	56707830	56707961
chr16	56712736	56712909
chr16	56715718	56716293
chr16	56716399	56717980
chr16	56718152	56718738
chr16	56719158	56719211
chr16	56719469	56719558
chr16	56725318	56726572
chr16	56735264	56735293
chr16	56735873	56736386
chr16	56763246	56763901
chr16	56764030	56764660
chr16	56769978	56770150
chr16	56776300	56776933
chr16	56777718	56777741
chr16	56798136	56798386
chr16	56806855	56806953

chr16	56827772	56827844
chr16	56846752	56847144
chr16	56847972	56848409
chr16	56848558	56849003
chr16	56867095	56867339
chr16	56882242	56882287
chr16	56882822	56882908
chr16	56883044	56883726
chr16	56884449	56884469
chr16	56885610	56885834
chr16	56884810	56885482
chr16	56902651	56902948
chr16	56903369	56903639
chr16	56903824	56904040
chr16	56904907	56905268
chr16	56944255	56944790
chr16	56944934	56945105
chr16	56945438	56945805
chr16	56946369	56947467
chr16	56950309	56950605
chr16	56951452	56951694
chr16	56952615	56952827
chr16	56953084	56953382
chr16	56953604	56953825
chr16	56962766	56963204
chr16	56963758	56963946
chr16	56964041	56964079
chr16	56964232	56964444
chr16	56965261	56965849
chr16	56966135	56967367
chr16	56972532	56972614
chr16	56974285	56974473
chr16	56979227	56979569
chr16	56993274	56993521
chr16	56994618	56994887
chr16	56995300	56995480
chr16	56995808	56996041
chr16	56997556	56997932
chr16	56998420	56999220
chr16	57000158	57000820
chr16	56999727	56999963
chr16	57000962	57001398
chr16	57001625	57001862
chr16	57018169	57018191
chr16	57019067	57019546
chr16	57019799	57020121

chr16	57022771	57024032
chr16	57031608	57031796
chr16	57033339	57033477
chr16	57034117	57034353
chr16	57036755	57036869
chr16	57048567	57049124
chr16	57072255	57072446
chr16	57073129	57073166
chr16	57073353	57073695
chr16	57082420	57082694
chr16	57084847	57085074
chr16	57087596	57087861
chr16	57093503	57093809
chr16	57094054	57095928
chr16	57103777	57104042
chr16	57109037	57109050
chr16	57109154	57109294
chr16	57118552	57118598
chr16	57125986	57127463
chr16	57133809	57134063
chr16	57156482	57156766
chr16	57163923	57164190
chr16	57169984	57170265
chr16	57173857	57174762
chr16	57174865	57175225
chr16	57182860	57182866
chr16	57182430	57182542
chr16	57218964	57219129
chr16	57219320	57219920
chr16	57220026	57221836
chr16	57223554	57223763
chr16	57225040	57225406
chr16	57226755	57227162
chr16	57227515	57227967
chr16	57228479	57228977
chr16	57240622	57240758
chr16	57242462	57242644
chr16	57247347	57247383
chr16	57248651	57248688
chr16	57277488	57277626
chr16	57278461	57278812
chr16	57278980	57279793
chr16	57287345	57287512
chr16	57294045	57295166
chr16	57296004	57296637
chr16	57296821	57297108

chr16	57299565	57299947
chr16	57305572	57305907
chr16	57308550	57308589
chr16	57309786	57310973
chr16	57311176	57312021
chr16	57313507	57313706
chr16	57317828	57319072
chr16	57332771	57333746
chr16	57333922	57334433
chr16	57334947	57335561
chr16	57338374	57338765
chr16	57346919	57346925
chr16	57348351	57348597
chr16	57350096	57350295
chr16	57350545	57350628
chr16	57350797	57350985
chr16	57351331	57351369
chr16	57351485	57351824
chr16	57373353	57373453
chr16	57373589	57373908
chr16	57375254	57375720
chr16	57375976	57376142
chr16	57379255	57379454
chr16	57388200	57388836
chr16	57407285	57407532
chr16	57410236	57410435
chr16	57411674	57412276
chr16	57412468	57412810
chr16	57414020	57414043
chr16	57421103	57421148
chr16	57422153	57422372
chr16	57426970	57427118
chr16	57427913	57428147
chr16	57450779	57451120
chr16	57451273	57452913
chr16	57455960	57455998
chr16	57456601	57456677
chr16	57458535	57458670
chr16	57478873	57478994
chr16	57480498	57481976
chr16	57496302	57497129
chr16	57519519	57521184
chr16	57566661	57566920
chr16	57568160	57568391
chr16	57569352	57571275
chr16	57574017	57574128

chr16	57575000	57575186
chr16	57624536	57624571
chr16	57625570	57625738
chr16	57640866	57641065
chr16	57646907	57646940
chr16	57649428	57649538
chr16	57651130	57651656
chr16	57651931	57656782
chr16	57656961	57658801
chr16	57659664	57660028
chr16	57662051	57663133
chr16	57676626	57676720
chr16	57676895	57677852
chr16	57679046	57679770
chr16	57679885	57680709
chr16	57681102	57681753
chr16	57681937	57682297
chr16	57687343	57688128
chr16	57688243	57688963
chr16	57701088	57701288
chr16	57701625	57702061
chr16	57705336	57705520
chr16	57709151	57709319
chr16	57709561	57709606
chr16	57710558	57710811
chr16	57728518	57728535
chr16	57767750	57768020
chr16	57768325	57769239
chr16	57769710	57771181
chr16	57793304	57793915
chr16	57794027	57794374
chr16	57797750	57798198
chr16	57798321	57798674
chr16	57798832	57799243
chr16	57801526	57802148
chr16	57802967	57803022
chr16	57802772	57802862
chr16	57813280	57813687
chr16	57814945	57814963
chr16	57816525	57816950
chr16	57826842	57826867
chr16	57829102	57829346
chr16	57829496	57829899
chr16	57830739	57831258
chr16	57831425	57831815
chr16	57832171	57833199

chr16	57834175	57835037
chr16	57835147	57837236
chr16	57838137	57838299
chr16	57839431	57839666
chr16	57839888	57840153
chr16	57840706	57841661
chr16	57841848	57842404
chr16	57843675	57844312
chr16	57844419	57845038
chr16	57845395	57845781
chr16	57846144	57847127
chr16	57848287	57848376
chr16	57849303	57850395
chr16	57850618	57850792
chr16	57850955	57851508
chr16	57852344	57852377
chr16	57852594	57852703
chr16	57853064	57853263
chr16	57854197	57854473
chr16	57855744	57856016
chr16	57857497	57857804
chr16	57858241	57858326
chr16	57869094	57869838
chr16	57871037	57871236
chr16	57893633	57893721
chr16	57897482	57897856
chr16	57900012	57900150
chr16	57900287	57900836
chr16	57902516	57902776
chr16	57917399	57917476
chr16	57918140	57918339
chr16	57919643	57919887
chr16	57924727	57924820
chr16	57926255	57926447
chr16	57926543	57926858
chr16	57927301	57927925
chr16	57928051	57928321
chr16	57929625	57930121
chr16	57930983	57931192
chr16	57931933	57932264
chr16	57932394	57932748
chr16	57932962	57933484
chr16	57933572	57933699
chr16	57945557	57945672
chr16	57951120	57951493
chr16	57955232	57955298

chr16	57956115	57956349
chr16	57972339	57973720
chr16	57973933	57974851
chr16	57974916	57975323
chr16	57977331	57977435
chr16	57991983	57992182
chr16	57993265	57993445
chr16	58000240	58000376
chr16	58017718	58018120
chr16	58018531	58018797
chr16	58020589	58020788
chr16	58021843	58022042
chr16	58022182	58022481
chr16	58023683	58023867
chr16	58026284	58026869
chr16	58027038	58027469
chr16	58027633	58028075
chr16	58032272	58032587
chr16	58032696	58035942
chr16	58036482	58036714
chr16	58036926	58037168
chr16	58054305	58054532
chr16	58056247	58056463
chr16	58057077	58057329
chr16	58058020	58058265
chr16	58058659	58059443
chr16	58059670	58061269
chr16	58095638	58095665
chr16	58096145	58096376
chr16	58122568	58122796
chr16	58131354	58131612
chr16	58142449	58142597
chr16	58155098	58155713
chr16	58157284	58157294
chr16	58158426	58158706
chr16	58160871	58161309
chr16	58162154	58162419
chr16	58162489	58162648
chr16	58162782	58163135
chr16	58163555	58163867
chr16	58187634	58187833
chr16	58231119	58231635
chr16	58231889	58232785
chr16	58270218	58270525
chr16	58283341	58283726
chr16	58283981	58284711

chr16	58298670	58299328
chr16	58313701	58315290
chr16	58366158	58366315
chr16	58373198	58373337
chr16	58418243	58418859
chr16	58426056	58426117
chr16	58426290	58427433
chr16	58431330	58431557
chr16	58432943	58433063
chr16	58441256	58441293
chr16	58445600	58445699
chr16	58498253	58498452
chr16	58534514	58534799
chr16	58547581	58547936
chr16	58548264	58550010
chr16	58551085	58551224
chr16	58551499	58551577
chr16	58597203	58597512
chr16	58597784	58597925
chr16	58607835	58608015
chr16	58633873	58633949
chr16	58646932	58647355
chr16	58647755	58648070
chr16	58648567	58648711
chr16	58649770	58649900
chr16	58654320	58654453
chr16	58655937	58655974
chr16	58658974	58659169
chr16	58661845	58662127
chr16	58662735	58664756
chr16	58671392	58671491
chr16	58672032	58672291
chr16	58672470	58672606
chr16	58677527	58677771
chr16	58684495	58685108
chr16	58685476	58685908
chr16	58695823	58696022
chr16	58707523	58707767
chr16	58718049	58718611
chr16	58720905	58721387
chr16	58766441	58766825
chr16	58767143	58767367
chr16	58767470	58768557
chr16	58770041	58770637
chr16	65692006	65692154
chr16	65692529	65692909

chr16	65693050	65693192
chr16	66303930	66304161
chr16	66304292	66304542
chr16	66311723	66311876
chr16	66312358	66312588
chr16	66362462	66362763
chr16	66391783	66391941
chr16	66404560	66404759
chr16	66415832	66416195
chr16	66416576	66417350
chr16	66417468	66418008
chr16	66440570	66440870
chr16	66441213	66441238
chr16	66442194	66443607
chr16	66460464	66460958
chr16	66461305	66461538
chr16	66512531	66512641
chr16	66513354	66513572
chr16	66519842	66520214
chr16	66536898	66537097
chr16	66541165	66541291
chr16	66541354	66541787
chr16	66541941	66542114
chr16	66542457	66543290
chr16	66544878	66544948
chr16	66545177	66545244
chr16	66545450	66545661
chr16	66545924	66546345
chr16	66546453	66546870
chr16	66550238	66550547
chr16	66550988	66551962
chr16	66556328	66556727
chr16	66557339	66557747
chr16	66558493	66558574
chr16	66583064	66584739
chr16	66585348	66585645
chr16	66585904	66586047
chr16	66586158	66587173
chr16	66614981	66615068
chr16	66622697	66622783
chr16	66629489	66629674
chr16	66637197	66637514
chr16	66637634	66640983
chr16	66641815	66642128
chr16	66642700	66643325
chr16	66644811	66644918

chr16	66652298	66652477
chr16	66652845	66653019
chr16	66660250	66660900
chr16	66673102	66673863
chr16	66673982	66674234
chr16	66679596	66679916
chr16	66684823	66684853
chr16	66687594	66687793
chr16	66694583	66695064
chr16	66695545	66695896
chr16	66725232	66725431
chr16	66726044	66726635
chr16	66728858	66729223
chr16	66729595	66731276
chr16	66734391	66734652
chr16	66735023	66735052
chr16	66735159	66735242
chr16	66735323	66735555
chr16	66745760	66745959
chr16	66747898	66748737
chr16	66752195	66752565
chr16	66760747	66761004
chr16	66770148	66770192
chr16	66771105	66771251
chr16	66773436	66773672
chr16	66774647	66774839
chr16	66775344	66775543
chr16	66775725	66775807
chr16	66781006	66781029
chr16	66782972	66783240
chr16	66784467	66785915
chr16	66796511	66796675
chr16	66832121	66832436
chr16	66861083	66861344
chr16	66862467	66862841
chr16	66863409	66863718
chr16	66863781	66863998
chr16	66864226	66864824
chr16	66865067	66865168
chr16	66866405	66867125
chr16	66867627	66867647
chr16	66868355	66868611
chr16	66869061	66869145
chr16	66879634	66879833
chr16	66899232	66899475
chr16	66899834	66900002

chr16	66902162	66902670
chr16	66904081	66904306
chr16	66906563	66908152
chr16	66909309	66909598
chr16	66910989	66911362
chr16	66911566	66911825
chr16	66913807	66915812
chr16	66916769	66916988
chr16	66922183	66922232
chr16	66922618	66922656
chr16	66923734	66923990
chr16	66929342	66929578
chr16	66931312	66931409
chr16	66930224	66930885
chr16	66931906	66932113
chr16	66932220	66932328
chr16	66933837	66934036
chr16	66934527	66934815
chr16	66958915	66959380
chr16	66962011	66962268
chr16	66964630	66964972
chr16	66967022	66967221
chr16	66967327	66967550
chr16	66967869	66969259
chr16	66969376	66970576
chr16	66970835	66971144
chr16	66978470	66978734
chr16	66979516	66980302
chr16	66981038	66982317
chr16	66982431	66983160
chr16	66986997	66988338
chr16	66991760	66991959
chr16	66995187	66995478
chr16	67002445	67002668
chr16	67003024	67003364
chr16	67003944	67004292
chr16	67004479	67004583
chr16	67005575	67005673
chr16	67005765	67006036
chr16	67009920	67010148
chr16	67021747	67022100
chr16	67032405	67032660
chr16	67033483	67034957
chr16	67035364	67035838
chr16	67041246	67041978
chr16	67044303	67044406

chr16	67047694	67048501
chr16	67048829	67049028
chr16	67049610	67049732
chr16	67054682	67054778
chr16	67061821	67062020
chr16	67062476	67062924
chr16	67063030	67064176
chr16	67064571	67064886
chr16	67076860	67076882
chr16	67089511	67089703
chr16	67089983	67090332
chr16	67123444	67123663
chr16	67138761	67138764
chr16	67140740	67141041
chr16	67143080	67144431
chr16	67144756	67145249
chr16	67145454	67145814
chr16	67146122	67146278
chr16	67167743	67167801
chr16	67181030	67181454
chr16	67181590	67181873
chr16	67182116	67182146
chr16	67183800	67185748
chr16	67189101	67189398
chr16	67192728	67192996
chr16	67193263	67195434
chr16	67196011	67196034
chr16	67196939	67198894
chr16	67202108	67202225
chr16	67203785	67205714
chr16	67206309	67206752
chr16	67206949	67209013
chr16	67211503	67211947
chr16	67212059	67213080
chr16	67215881	67216198
chr16	67216319	67216786
chr16	67216879	67217332
chr16	67217458	67219652
chr16	67225694	67226941
chr16	67227117	67227935
chr16	67228095	67228403
chr16	67232354	67234305
chr16	67239143	67239369
chr16	67240454	67240654
chr16	67240830	67241125
chr16	67241272	67241939

chr16	67242620	67242819
chr16	67246165	67247567
chr16	67252104	67252612
chr16	67252829	67253085
chr16	67260140	67261908
chr16	67267104	67267265
chr16	67274163	67276733
chr16	67277163	67280683
chr16	67280839	67281442
chr16	67281612	67282057
chr16	67282290	67283545
chr16	67309759	67310008
chr16	67310952	67314437
chr16	67315134	67315225
chr16	67315345	67315361
chr16	67345405	67345754
chr16	67345866	67346143
chr16	67358628	67358655
chr16	67360504	67360575
chr16	67360797	67360824
chr16	67363554	67363597
chr16	67429826	67430236
chr16	67437376	67438651
chr16	67438867	67439599
chr16	67439855	67440264
chr16	67440589	67441073
chr16	67441848	67442318
chr16	67444598	67444678
chr16	67447592	67447937
chr16	67448356	67448454
chr16	67449362	67450643
chr16	67450984	67451302
chr16	67452030	67452427
chr16	67464233	67465938
chr16	67482364	67482585
chr16	67496336	67496643
chr16	67502478	67502752
chr16	67501941	67502155
chr16	67502873	67503193
chr16	67503257	67503508
chr16	67511798	67511946
chr16	67512123	67512299
chr16	67513167	67513348
chr16	67513994	67515037
chr16	67515291	67516147
chr16	67518756	67519063

chr16	67518560	67518669
chr16	67522632	67522901
chr16	67523127	67523514
chr16	67524190	67524316
chr16	67530057	67530256
chr16	67535009	67535138
chr16	67536930	67537413
chr16	67538582	67538894
chr16	67539925	67540030
chr16	67542541	67542669
chr16	67542788	67542899
chr16	67547988	67548412
chr16	67548552	67548695
chr16	67550138	67550468
chr16	67550646	67550768
chr16	67554902	67555101
chr16	67555207	67555544
chr16	67559470	67559889
chr16	67560770	67561120
chr16	67561279	67561476
chr16	67561605	67561654
chr16	67562362	67562575
chr16	67562701	67564498
chr16	67567719	67568565
chr16	67570038	67570380
chr16	67570739	67572466
chr16	67576015	67576254
chr16	67577434	67577651
chr16	67585616	67585647
chr16	67585911	67585981
chr16	67587500	67587714
chr16	67589297	67590271
chr16	67590733	67591101
chr16	67591409	67591608
chr16	67592956	67593203
chr16	67593418	67593981
chr16	67595315	67595432
chr16	67595555	67598145
chr16	67600708	67601051
chr16	67602052	67602218
chr16	67619065	67619227
chr16	67628948	67629072
chr16	67631970	67632174
chr16	67637775	67638044
chr16	67648756	67648853
chr16	67669030	67669150

chr16	67669292	67669641
chr16	67678724	67679444
chr16	67693336	67694187
chr16	67694293	67695128
chr16	67698995	67699213
chr16	67699354	67701777
chr16	67705410	67705645
chr16	67706333	67706603
chr16	67707543	67707867
chr16	67710466	67710863
chr16	67713108	67713289
chr16	67727810	67728181
chr16	67736391	67736473
chr16	67752329	67752735
chr16	67752878	67753250
chr16	67753385	67753937
chr16	67755628	67755849
chr16	67756068	67756099
chr16	67757152	67757407
chr16	67779900	67780062
chr16	67782513	67782798
chr16	67783351	67783630
chr16	67790510	67790709
chr16	67801876	67801971
chr16	67802097	67802258
chr16	67813337	67813366
chr16	67813772	67814555
chr16	67818253	67818427
chr16	67830544	67830576
chr16	67839381	67841053
chr16	67841322	67841530
chr16	67849608	67849845
chr16	67850023	67850186
chr16	67850256	67850282
chr16	67850834	67850935
chr16	67853281	67853607
chr16	67866832	67866878
chr16	67866560	67866739
chr16	67867000	67867836
chr16	67871448	67871504
chr16	67875317	67876945
chr16	67877171	67877677
chr16	67878320	67878771
chr16	67879329	67882438
chr16	67884873	67885256
chr16	67891784	67892398

chr16	67894818	67895052
chr16	67897487	67897686
chr16	67900361	67900583
chr16	67901035	67901152
chr16	67901457	67901637
chr16	67903772	67903921
chr16	67904856	67904989
chr16	67906473	67907543
chr16	67907840	67908057
chr16	67921438	67921629
chr16	67926098	67926270
chr16	67926768	67926938
chr16	67927105	67928357
chr16	67929620	67929654
chr16	67931620	67931798
chr16	67939365	67939706
chr16	67940209	67940408
chr16	67940480	67940557
chr16	67943626	67943825
chr16	67944803	67945100
chr16	67944334	67944679
chr16	67945258	67945457
chr16	67954986	67955244
chr16	67958461	67958660
chr16	67960293	67960528
chr16	67967878	67968378
chr16	67968589	67968921
chr16	67969202	67969522
chr16	67969761	67971504
chr16	67971888	67972007
chr16	67972344	67972481
chr16	67972691	67972751
chr16	67973167	67973335
chr16	67974630	67974986
chr16	67975211	67975436
chr16	67989670	67989912
chr16	67990319	67990482
chr16	67992279	67992459
chr16	67992820	67993533
chr16	67993648	67994301
chr16	67995185	67995578
chr16	67994941	67995080
chr16	67995874	67996758
chr16	67998228	67998235
chr16	67998420	67998427
chr16	67999022	67999264

chr16	67999769	68003544
chr16	68004202	68004448
chr16	68004740	68005034
chr16	68006058	68006319
chr16	68007306	68007419
chr16	68007603	68007703
chr16	68017324	68017465
chr16	68017824	68018591
chr16	68018825	68018938
chr16	68024571	68024695
chr16	68026892	68027244
chr16	68027350	68027867
chr16	68029578	68029982
chr16	68038568	68039332
chr16	68039501	68040662
chr16	68044263	68044502
chr16	68047254	68047946
chr16	68050276	68050401
chr16	68050655	68050783
chr16	68056280	68057853
chr16	68064077	68064432
chr16	68064912	68065375
chr16	68065765	68066798
chr16	68070493	68070604
chr16	68073979	68074553
chr16	68074796	68074833
chr16	68080729	68080744
chr16	68097815	68098136
chr16	68098438	68099097
chr16	68099276	68099796
chr16	68101250	68101266
chr16	68105527	68105539
chr16	68106384	68106824
chr16	68117434	68117925
chr16	68118144	68119031
chr16	68119251	68120120
chr16	68120838	68121037
chr16	68125457	68126014
chr16	68148492	68148734
chr16	68149029	68149307
chr16	68176166	68176622
chr16	68181654	68181725
chr16	68241424	68241488
chr16	68266921	68267128
chr16	68267575	68271522
chr16	68271727	68272574

chr16	68273119	68273331
chr16	68274262	68274738
chr16	68276537	68276681
chr16	68277682	68277755
chr16	68278619	68279121
chr16	68279315	68279564
chr16	68279718	68280012
chr16	68286598	68286797
chr16	68290962	68291238
chr16	68292490	68292726
chr16	68295606	68295917
chr16	68297142	68297492
chr16	68297639	68299760
chr16	68300876	68301069
chr16	68303557	68303594
chr16	68303884	68303975
chr16	68305180	68306148
chr16	68307805	68308078
chr16	68309443	68309928
chr16	68316284	68316507
chr16	68320320	68320503
chr16	68321571	68321982
chr16	68331989	68332191
chr16	68332510	68332925
chr16	68333213	68334355
chr16	68334576	68334798
chr16	68344091	68344140
chr16	68344265	68345681
chr16	68362767	68362950
chr16	68367715	68368014
chr16	68368301	68368536
chr16	68451819	68452041
chr16	68526289	68526552
chr16	68526861	68527077
chr16	68529274	68529752
chr16	68529908	68530086
chr16	68542226	68542300
chr16	68542415	68542612
chr16	68544347	68544586
chr16	68554871	68555268
chr16	68562986	68563167
chr16	68564087	68564742
chr16	68565770	68565997
chr16	68572754	68572857
chr16	68572972	68573982
chr16	68609118	68609195

chr16	68624583	68625065
chr16	68630197	68630294
chr16	68633196	68633456
chr16	68639551	68639751
chr16	68640073	68640177
chr16	68640398	68640592
chr16	68662708	68662939
chr16	68669575	68670118
chr16	68670259	68670596
chr16	68670682	68670986
chr16	68674392	68675941
chr16	68676442	68676649
chr16	68677233	68677720
chr16	68678092	68680655
chr16	68680750	68681225
chr16	68681933	68681957
chr16	68682386	68682491
chr16	68683232	68683343
chr16	68683714	68684074
chr16	68686374	68687048
chr16	68687529	68687886
chr16	68688826	68689385
chr16	68689506	68690021
chr16	68690146	68690841
chr16	68690996	68691235
chr16	68691445	68691930
chr16	68692022	68692903
chr16	68693342	68693666
chr16	68694157	68694809
chr16	68698501	68699859
chr16	68700116	68700501
chr16	68701414	68701897
chr16	68701974	68702891
chr16	68703038	68703534
chr16	68713472	68713671
chr16	68714286	68714462
chr16	68719076	68719509
chr16	68719636	68719980
chr16	68724230	68724466
chr16	68725917	68726234
chr16	68733498	68733901
chr16	68736730	68736860
chr16	68737576	68737731
chr16	68738113	68738686
chr16	68739641	68740448
chr16	68740788	68742188

chr16	68742908	68743587
chr16	68744934	68745241
chr16	68749287	68749450
chr16	68749688	68749998
chr16	68751418	68751553
chr16	68752050	68752083
chr16	68759624	68760322
chr16	68762839	68762920
chr16	68763917	68763946
chr16	68765548	68765971
chr16	68766101	68766788
chr16	68769201	68769448
chr16	68770049	68772698
chr16	68772833	68772945
chr16	68774333	68774543
chr16	68775462	68776790
chr16	68777081	68777112
chr16	68777383	68777582
chr16	68778007	68778773
chr16	68778901	68779392
chr16	68779779	68779978
chr16	68780379	68780383
chr16	68780498	68780635
chr16	68780839	68781002
chr16	68783426	68783748
chr16	68785892	68786205
chr16	68786278	68786739
chr16	68787133	68787428
chr16	68787790	68790209
chr16	68790512	68791445
chr16	68791547	68791974
chr16	68794027	68794461
chr16	68794543	68794765
chr16	68795188	68795634
chr16	68795814	68796025
chr16	68798511	68798787
chr16	68801156	68801366
chr16	68806594	68806787
chr16	68810793	68811024
chr16	68811869	68812877
chr16	68813092	68813787
chr16	68817084	68817097
chr16	68820709	68820923
chr16	68821653	68821898
chr16	68822930	68823260
chr16	68823543	68824293

chr16	68824406	68824706
chr16	68825932	68826200
chr16	68827953	68828074
chr16	68829458	68829496
chr16	68829801	68830038
chr16	68832086	68833200
chr16	68834120	68834602
chr16	68834981	68835312
chr16	68836344	68836677
chr16	68837412	68837601
chr16	68839780	68839983
chr16	68840302	68840429
chr16	68853720	68853957
chr16	68857145	68857652
chr16	68857959	68858158
chr16	68877199	68877311
chr16	68877471	68878225
chr16	68879491	68879814
chr16	68880937	68881187
chr16	68881338	68881457
chr16	68901501	68901700
chr16	68905563	68905814
chr16	68935625	68935874
chr16	68936646	68936722
chr16	68937241	68937440
chr16	68946919	68947202
chr16	68949111	68949871
chr16	68954184	68954626
chr16	68955470	68955981
chr16	68957283	68957534
chr16	68970350	68971252
chr16	68986279	68986899
chr16	68992591	68993776
chr16	68999479	68999648
chr16	69002464	69002481
chr16	69002597	69002739
chr16	69006600	69006925
chr16	69007430	69007487
chr16	69009754	69009969
chr16	69017587	69017716
chr16	69019211	69019562
chr16	69019713	69020749
chr16	69021302	69021524
chr16	69024879	69025370
chr16	69026353	69026989
chr16	69027733	69028198

chr16	69029010	69029262
chr16	69029412	69029711
chr16	69051619	69051753
chr16	69065642	69065718
chr16	69066967	69067073
chr16	69070624	69070831
chr16	69072914	69073304
chr16	69074054	69074320
chr16	69074404	69074813
chr16	69082215	69082455
chr16	69083185	69083373
chr16	69084601	69084801
chr16	69085084	69085690
chr16	69091425	69092674
chr16	69096586	69096956
chr16	69098077	69098120
chr16	69103898	69104239
chr16	69105603	69106630
chr16	69106999	69107701
chr16	69107831	69108043
chr16	69109335	69109807
chr16	69110201	69110374
chr16	69111099	69111621
chr16	69123101	69123435
chr16	69124434	69124693
chr16	69130356	69130376
chr16	69134999	69135511
chr16	69136896	69137068
chr16	69137632	69138015
chr16	69138397	69138477
chr16	69138858	69138891
chr16	69139226	69139400
chr16	69139516	69142333
chr16	69142451	69142770
chr16	69145137	69145368
chr16	69157922	69158195
chr16	69160962	69161089
chr16	69161729	69161950
chr16	69165194	69165404
chr16	69165620	69167153
chr16	69206249	69206437
chr16	69207415	69207567
chr16	69208540	69208564
chr16	69214221	69214322
chr16	69216770	69216834
chr16	69218603	69218684

chr16	69220502	69220710
chr16	69220999	69221332
chr16	69221504	69222366
chr16	69222762	69222961
chr16	69224547	69224780
chr16	69225591	69225833
chr16	69233176	69233388
chr16	69233706	69233724
chr16	69235667	69236066
chr16	69244424	69244715
chr16	69251333	69251433
chr16	69255443	69255818
chr16	69256054	69256461
chr16	69263288	69263341
chr16	69271561	69271671
chr16	69325848	69325934
chr16	69327876	69328363
chr16	69331855	69332092
chr16	69332410	69332736
chr16	69344461	69344947
chr16	69345175	69346235
chr16	69346564	69346855
chr16	69349209	69349408
chr16	69356697	69356896
chr16	69361011	69361257
chr16	69361906	69362337
chr16	69362684	69362804
chr16	69363020	69365062
chr16	69365468	69365640
chr16	69372685	69372884
chr16	69373028	69373479
chr16	69373626	69374560
chr16	69393997	69394394
chr16	69397117	69397160
chr16	69397430	69397720
chr16	69399465	69399692
chr16	69411210	69411373
chr16	69419312	69420209
chr16	69429997	69430396
chr16	69431929	69432628
chr16	69433110	69433227
chr16	69435360	69435559
chr16	69436864	69437220
chr16	69438275	69438821
chr16	69439679	69440055
chr16	69440227	69440232

chr16	69442404	69442523
chr16	69443684	69443831
chr16	69451627	69451818
chr16	69458009	69458271
chr16	69458493	69458993
chr16	69473448	69473657
chr16	69477566	69477765
chr16	69478179	69478348
chr16	69498063	69498131
chr16	69504308	69504337
chr16	69513719	69514118
chr16	69535789	69536250
chr16	69547309	69547431
chr16	69547750	69547925
chr16	69549405	69549525
chr16	69561581	69561900
chr16	69563407	69565044
chr16	69568935	69569311
chr16	69569437	69569849
chr16	69573393	69573809
chr16	69578296	69578495
chr16	69580337	69580374
chr16	69584100	69584318
chr16	69597942	69598752
chr16	69599253	69599660
chr16	69599907	69601405
chr16	69605720	69605919
chr16	69632658	69633288
chr16	69644555	69644667
chr16	69646521	69646850
chr16	69647015	69647307
chr16	69647445	69647572
chr16	69692163	69692330
chr16	69694059	69694470
chr16	69715344	69715367
chr16	69715543	69715578
chr16	69739872	69740485
chr16	69743977	69744207
chr16	69745230	69745727
chr16	69746023	69746345
chr16	69754533	69754660
chr16	69754814	69755135
chr16	69757022	69757077
chr16	69758951	69759190
chr16	69759501	69759822
chr16	69760117	69762169

chr16	69762483	69762840
chr16	69763178	69763493
chr16	69768245	69768512
chr16	69771963	69772137
chr16	69773022	69773407
chr16	69773528	69773660
chr16	69773926	69774231
chr16	69775195	69775953
chr16	69776134	69776333
chr16	69780746	69780910
chr16	69781819	69781981
chr16	69784423	69784526
chr16	69786617	69786976
chr16	69788316	69789228
chr16	69793402	69793485
chr16	69796869	69797242
chr16	69795827	69796678
chr16	69821989	69822242
chr16	69860332	69860443
chr16	69880417	69880605
chr16	69893207	69893320
chr16	69904842	69905146
chr16	69951547	69951824
chr16	69951963	69952138
chr16	69979597	69979632
chr16	69998781	69999670
chr16	70003917	70004026
chr16	70008059	70008258
chr16	70021235	70021367
chr16	70028774	70028994
chr16	70029418	70029885
chr16	70030137	70030174
chr16	70030750	70031114
chr16	70031260	70031471
chr16	70033308	70033444
chr16	70050048	70050178
chr16	70059021	70059241
chr16	70092427	70092615
chr16	70098512	70100992
chr16	70132791	70133355
chr16	70143675	70144068
chr16	70146990	70149002
chr16	70150292	70150455
chr16	70156819	70157107
chr16	70175168	70175871
chr16	70179176	70179218

chr16	70198740	70199122
chr16	70204592	70204836
chr16	70221533	70222877
chr16	70223415	70223664
chr16	70229364	70229702
chr16	70247491	70247690
chr16	70248099	70248367
chr16	70250636	70251050
chr16	70264127	70264482
chr16	70269545	70269690
chr16	70282659	70282767
chr16	70284240	70286006
chr16	70313031	70313126
chr16	70315774	70315838
chr16	70317677	70317874
chr16	70319202	70319811
chr16	70321355	70323478
chr16	70323582	70324103
chr16	70333032	70333675
chr16	70339003	70339034
chr16	70366052	70366162
chr16	70366607	70366662
chr16	70376579	70376769
chr16	70379967	70380166
chr16	70380261	70381249
chr16	70403007	70403424
chr16	70403588	70403802
chr16	70407828	70408110
chr16	70408434	70408544
chr16	70411507	70411717
chr16	70413472	70413524
chr16	70413767	70415404
chr16	70415508	70417034
chr16	70417190	70417389
chr16	70418671	70418907
chr16	70419672	70419871
chr16	70420406	70420907
chr16	70421115	70421223
chr16	70423309	70423586
chr16	70423845	70424089
chr16	70425088	70425620
chr16	70436853	70437236
chr16	70437589	70438875
chr16	70439156	70439535
chr16	70443775	70443977
chr16	70446894	70447219

chr16	70449731	70449982
chr16	70457065	70458316
chr16	70461823	70462014
chr16	70462780	70463295
chr16	70468082	70468276
chr16	70469947	70470381
chr16	70471912	70472887
chr16	70473254	70473588
chr16	70474945	70475260
chr16	70481982	70482460
chr16	70487833	70488933
chr16	70489040	70489737
chr16	70489882	70490113
chr16	70499454	70499653
chr16	70505388	70505634
chr16	70505792	70506344
chr16	70509707	70510274
chr16	70510392	70510604
chr16	70510929	70511865
chr16	70512947	70513281
chr16	70522058	70523300
chr16	70526316	70526515
chr16	70527227	70527428
chr16	70540103	70540106
chr16	70546251	70546314
chr16	70546620	70546856
chr16	70552673	70552823
chr16	70554623	70554638
chr16	70554759	70554982
chr16	70556928	70558175
chr16	70558365	70559238
chr16	70559595	70559954
chr16	70560195	70560558
chr16	70582953	70583152
chr16	70592791	70592827
chr16	70595102	70595249
chr16	70595872	70595901
chr16	70597255	70597611
chr16	70597753	70597908
chr16	70600749	70600960
chr16	70602636	70602680
chr16	70603051	70603265
chr16	70603715	70604018
chr16	70604483	70604551
chr16	70606149	70606402
chr16	70607249	70607439

chr16	70608368	70608649
chr16	70608881	70610068
chr16	70610240	70611574
chr16	70611838	70612006
chr16	70612553	70613004
chr16	70613236	70613373
chr16	70614338	70614502
chr16	70617734	70617933
chr16	70620191	70620324
chr16	70623720	70623939
chr16	70670289	70670502
chr16	70680014	70680214
chr16	70686909	70687242
chr16	70687325	70688293
chr16	70706108	70706217
chr16	70706807	70706980
chr16	70708576	70708969
chr16	70709185	70709688
chr16	70709810	70710675
chr16	70710792	70711203
chr16	70715129	70715837
chr16	70716046	70716303
chr16	70716450	70716998
chr16	70717624	70717824
chr16	70717951	70719784
chr16	70720032	70721309
chr16	70721412	70722422
chr16	70724556	70724813
chr16	70726863	70729882
chr16	70729999	70732111
chr16	70732787	70737519
chr16	70738453	70738806
chr16	70739903	70741798
chr16	70742029	70742457
chr16	70742637	70743064
chr16	70743193	70743683
chr16	70743833	70744258
chr16	70744800	70745032
chr16	70745167	70745596
chr16	70746840	70750234
chr16	70750346	70750545
chr16	70751479	70751944
chr16	70752073	70752391
chr16	70765272	70765421
chr16	70766476	70766723
chr16	70771775	70772103

chr16	70774595	70775157
chr16	70775681	70776093
chr16	70779559	70779803
chr16	70780116	70780722
chr16	70780966	70781281
chr16	70781562	70783260
chr16	70783439	70783755
chr16	70793877	70794079
chr16	70794162	70794753
chr16	70794947	70795333
chr16	70799763	70799962
chr16	70801440	70802574
chr16	70804504	70805075
chr16	70805402	70805410
chr16	70805592	70805827
chr16	70810867	70811183
chr16	70812792	70812824
chr16	70834433	70835420
chr16	70838231	70838982
chr16	70887752	70887785
chr16	70910317	70910367
chr16	70910708	70910711
chr16	70915075	70915267
chr16	70953420	70953577
chr16	70962118	70962149
chr16	70970862	70971195
chr16	71104201	71104321
chr16	71202243	71202329
chr16	71322713	71323535
chr16	71323640	71323827
chr16	71324317	71324387
chr16	71371983	71372235
chr16	71392453	71392923
chr16	71459697	71460415
chr16	71460752	71460991
chr16	71471403	71471686
chr16	71494207	71494321
chr16	71494501	71494560
chr16	71495476	71496436
chr16	71508877	71509350
chr16	71517896	71518771
chr16	71598394	71598427
chr16	71598531	71598839
chr16	71599007	71599708
chr16	71659713	71661500
chr16	71663968	71664141

chr16	71665087	71665226
chr16	71673092	71673299
chr16	71741734	71742133
chr16	71743544	71743873
chr16	71756604	71756749
chr16	71756886	71757857
chr16	71758311	71758350
chr16	71769293	71769519
chr16	71782984	71783044
chr16	71824160	71824316
chr16	71827503	71827818
chr16	71841998	71843077
chr16	71843191	71843609
chr16	71844235	71844587
chr16	71845548	71845699
chr16	71849178	71849395
chr16	71850773	71850926
chr16	71870168	71870388
chr16	71872519	71872736
chr16	71873851	71874199
chr16	71875871	71875921
chr16	71876126	71876167
chr16	71879319	71880392
chr16	71880712	71880849
chr16	71916504	71916715
chr16	71916807	71917127
chr16	71917290	71918742
chr16	71927575	71927788
chr16	71927936	71930134
chr16	71931000	71931074
chr16	71931188	71931222
chr16	71938117	71938279
chr16	71945123	71945300
chr16	71954575	71954663
chr16	71961166	71961421
chr16	71962362	71962482
chr16	71990282	71990496
chr16	72041844	72042084
chr16	72042235	72043152
chr16	72045222	72045316
chr16	72047036	72047273
chr16	72047530	72047919
chr16	72126687	72127437
chr16	72127830	72128461
chr16	72223617	72223783
chr16	72325221	72325823

chr16	72613390	72614076
chr16	72661050	72661683
chr16	72671500	72671659
chr16	72698225	72698899
chr16	72699012	72699279
chr16	72821207	72821931
chr16	72882411	72882684
chr16	72908972	72909408
chr16	72909536	72909596
chr16	72909700	72909988
chr16	72911219	72911391
chr16	72911622	72911890
chr16	72912072	72912366
chr16	72961420	72961505
chr16	72995257	72995495
chr16	73015833	73015886
chr16	73021953	73021960
chr16	73022053	73022152
chr16	73025196	73025338
chr16	73023328	73024811
chr16	73030971	73031126
chr16	73031661	73032227
chr16	73049380	73049609
chr16	73054442	73054658
chr16	73055161	73055293
chr16	73069969	73069975
chr16	73070245	73070460
chr16	73081059	73081121
chr16	73081253	73083033
chr16	73083171	73083347
chr16	73087168	73087951
chr16	73089074	73089179
chr16	73089342	73090280
chr16	73090927	73091126
chr16	73091341	73092931
chr16	73098071	73098414
chr16	73098542	73098613
chr16	73098743	73099299
chr16	73102300	73102984
chr16	73103127	73103310
chr16	73104315	73104961
chr16	73105187	73105419
chr16	73116248	73116978
chr16	73147182	73147275
chr16	73160312	73160518
chr16	73208375	73208476

chr16	73210968	73210986
chr16	73685490	73685898
chr16	73768842	73769347
chr16	73769690	73769810
chr16	73983534	73983812
chr16	74325570	74325923
chr16	74326055	74326199
chr16	74330133	74331426
chr16	74356801	74357000
chr16	74357206	74357414
chr16	74365912	74366126
chr16	74373094	74373842
chr16	74374883	74374987
chr16	74401091	74401235
chr16	74401525	74402779
chr16	74408157	74408560
chr16	74415943	74416180
chr16	74416293	74416492
chr16	74427489	74427725
chr16	74440617	74441245
chr16	74449188	74449293
chr16	74459914	74460078
chr16	74460205	74460283
chr16	74461286	74461464
chr16	74480961	74481117
chr16	74482154	74482819
chr16	74482949	74484353
chr16	74484813	74485093
chr16	74488976	74489180
chr16	74489381	74490464
chr16	74493648	74493867
chr16	74496197	74496775
chr16	74498111	74498343
chr16	74502333	74502419
chr16	74513979	74514870
chr16	74592814	74593187
chr16	74597650	74598159
chr16	74611716	74611995
chr16	74612173	74612377
chr16	74620786	74620873
chr16	74635121	74635187
chr16	74640120	74641362
chr16	74643941	74644145
chr16	74658641	74658747
chr16	74666781	74667038
chr16	74667568	74667933

chr16	74673095	74673426
chr16	74673978	74674326
chr16	74686159	74686281
chr16	74686825	74687025
chr16	74700262	74700755
chr16	74701140	74701150
chr16	74701257	74701658
chr16	74729994	74730280
chr16	74732228	74732595
chr16	74733426	74735483
chr16	74741609	74741771
chr16	74763145	74763387
chr16	74763481	74763699
chr16	74763819	74764154
chr16	74764346	74764920
chr16	74765799	74766080
chr16	74771478	74771706
chr16	74771907	74772442
chr16	74773872	74774042
chr16	74774541	74775642
chr16	74775801	74775933
chr16	74776954	74778292
chr16	74778424	74778946
chr16	74776461	74776819
chr16	74779641	74780209
chr16	74807858	74808199
chr16	74808307	74808902
chr16	74876502	74876796
chr16	74878187	74878311
chr16	74896928	74897017
chr16	74899562	74899751
chr16	74901541	74901923
chr16	74902042	74902914
chr16	74915397	74915546
chr16	74919933	74920209
chr16	74921684	74921989
chr16	74962034	74962088
chr16	74971442	74971608
chr16	75009049	75009232
chr16	75013647	75013891
chr16	75017265	75017701
chr16	75018544	75019565
chr16	75029266	75029419
chr16	75031222	75031344
chr16	75031882	75032343
chr16	75032511	75033885

chr16	75045400	75046251
chr16	75079089	75079127
chr16	75124708	75125648
chr16	75142084	75142219
chr16	75142556	75142975
chr16	75143084	75145089
chr16	75145459	75145837
chr16	75150353	75150661
chr16	75160164	75160734
chr16	75182082	75182977
chr16	75233965	75234221
chr16	75250848	75251106
chr16	75253264	75253406
chr16	75254310	75254497
chr16	75266193	75266392
chr16	75266795	75267362
chr16	75267724	75268302
chr16	75271716	75271947
chr16	75277712	75277922
chr16	75278036	75280455
chr16	75280622	75281514
chr16	75282495	75282957
chr16	75283200	75285918
chr16	75286371	75286570
chr16	75286725	75287475
chr16	75287781	75288088
chr16	75289370	75289932
chr16	75290189	75290396
chr16	75293233	75293485
chr16	75293806	75294115
chr16	75297274	75297473
chr16	75298524	75300339
chr16	75300484	75301800
chr16	75302841	75302917
chr16	75302342	75302536
chr16	75303319	75303373
chr16	75304618	75304902
chr16	75305202	75305525
chr16	75307740	75307798
chr16	75307883	75308087
chr16	75310939	75311270
chr16	75312859	75312990
chr16	75313119	75313253
chr16	75313415	75313614
chr16	75313692	75314337
chr16	75317258	75317550

chr16	75338026	75338781
chr16	75339034	75340623
chr16	75356612	75357696
chr16	75385128	75385504
chr16	75410404	75410883
chr16	75411717	75412391
chr16	75414956	75415170
chr16	75416252	75416784
chr16	75419458	75419739
chr16	75426952	75427177
chr16	75427569	75428197
chr16	75432836	75432910
chr16	75436799	75437240
chr16	75439680	75439930
chr16	75443679	75443789
chr16	75445937	75446151
chr16	75454853	75454892
chr16	75455047	75455261
chr16	75460686	75460810
chr16	75463779	75463780
chr16	75463934	75464301
chr16	75465719	75466054
chr16	75466587	75468418
chr16	75488974	75489173
chr16	75497176	75498655
chr16	75498909	75499046
chr16	75505991	75506053
chr16	75507900	75508052
chr16	75511727	75512064
chr16	75514752	75514988
chr16	75516303	75516454
chr16	75516950	75517175
chr16	75517354	75518179
chr16	75518598	75519060
chr16	75519809	75519986
chr16	75520279	75520497
chr16	75521447	75521582
chr16	75522332	75522341
chr16	75522529	75522701
chr16	75528655	75529379
chr16	75536807	75537115
chr16	75540251	75540429
chr16	75540796	75540965
chr16	75550283	75551254
chr16	75551536	75551782
chr16	75553238	75554161

chr16	75554387	75554749
chr16	75555094	75555316
chr16	75555612	75555637
chr16	75588970	75589089
chr16	75589278	75590507
chr16	75598800	75601122
chr16	75605639	75605849
chr16	75612944	75613005
chr16	75621444	75621930
chr16	75627863	75628756
chr16	75655766	75656500
chr16	75656738	75657674
chr16	75658630	75658829
chr16	75659147	75659185
chr16	75661996	75662238
chr16	75665976	75666125
chr16	75681148	75681583
chr16	75681786	75682721
chr16	75683224	75683668
chr16	75685323	75685678
chr16	75854967	75855423
chr16	76093055	76093208
chr16	76093385	76093515
chr16	76159770	76159830
chr16	76228505	76228633
chr16	76422978	76422987
chr16	76710146	76710298
chr16	76710495	76710840
chr16	76814489	76814530
chr16	76814731	76814844
chr16	76821353	76821603
chr16	76838975	76839047
chr16	77224172	77224570
chr16	77224753	77225238
chr16	77245984	77246973
chr16	77247245	77247761
chr16	77249889	77249923
chr16	77332911	77333029
chr16	77755988	77756042
chr16	77756278	77756964
chr16	77867661	77867839
chr16	77868007	77868234
chr16	78133013	78134262
chr16	78138998	78139810
chr16	78156701	78156911
chr16	78244464	78244692

chr16	78245638	78245946
chr16	78717824	78717842
chr16	78718048	78718292
chr16	79021473	79021708
chr16	79020071	79020279
chr16	79021931	79022064
chr16	79022362	79022404
chr16	79022614	79022674
chr16	79031775	79032026
chr16	79042007	79042395
chr16	79042474	79042541
chr16	79083876	79083892
chr16	79084323	79084440
chr16	79084628	79084758
chr16	79632458	79632803
chr16	79633939	79634572
chr16	79634850	79635355
chr16	79635494	79635648
chr16	79636081	79636115
chr16	79673419	79673939
chr16	79674059	79674722
chr16	79692058	79692308
chr16	79692481	79692972
chr16	79785658	79785694
chr16	79786074	79786109
chr16	79786350	79786465
chr16	79786579	79786688
chr16	79792558	79792881
chr16	79793137	79793230
chr16	79804048	79804353
chr16	79804650	79804886
chr16	79844651	79845070
chr16	79845505	79845696
chr16	79881397	79881537
chr16	79882600	79883095
chr16	79909694	79909737
chr16	79919575	79919729
chr16	79933421	79933744
chr16	80031495	80031573
chr16	80060869	80061688
chr16	80137183	80137921
chr16	80139007	80139197
chr16	80139435	80139497
chr16	80208222	80208615
chr16	80231522	80232115
chr16	80237955	80237993

chr16	80307477	80307658
chr16	80307791	80308017
chr16	80308172	80308180
chr16	80308515	80308564
chr16	80308786	80308908
chr16	80334863	80335167
chr16	80574298	80574881
chr16	80578265	80578293
chr16	80648242	80648288
chr16	80648530	80648704
chr16	80838598	80838919
chr16	80965736	80966398
chr16	80966515	80966827
chr16	80966932	80967163
chr16	81000580	81000964
chr16	81012892	81012895
chr16	81021574	81021703
chr16	81026839	81027339
chr16	81038269	81039010
chr16	81039179	81039682
chr16	81039831	81042262
chr16	81043560	81043677
chr16	81069130	81069152
chr16	81069655	81070730
chr16	81105365	81105495
chr16	81110077	81111583
chr16	81126042	81126049
chr16	81129297	81129986
chr16	81130203	81130718
chr16	81136402	81136661
chr16	81175256	81175443
chr16	81175586	81175908
chr16	81177377	81177610
chr16	81177760	81178251
chr16	81178426	81178790
chr16	81179656	81180086
chr16	81180209	81180568
chr16	81214133	81214651
chr16	81215002	81215354
chr16	81236694	81236905
chr16	81271915	81272470
chr16	81277036	81277542
chr16	81294496	81294506
chr16	81300861	81300887
chr16	81305714	81305855
chr16	81316953	81317258

chr16	81348190	81348371
chr16	81348523	81349606
chr16	81361188	81361350
chr16	81361600	81361637
chr16	81410410	81410545
chr16	81435367	81435376
chr16	81437334	81437533
chr16	81439212	81439505
chr16	81439672	81440184
chr16	81440637	81441092
chr16	81441851	81442045
chr16	81458455	81458637
chr16	81458811	81458870
chr16	81459722	81459890
chr16	81477648	81478313
chr16	81481852	81482534
chr16	81478546	81481726
chr16	81498271	81498470
chr16	81499846	81500100
chr16	81515678	81515849
chr16	81526705	81527441
chr16	81527609	81528278
chr16	81528698	81528871
chr16	81529475	81529696
chr16	81532826	81533833
chr16	81535305	81535504
chr16	81536410	81536486
chr16	81536941	81538607
chr16	81538824	81539232
chr16	81544348	81545535
chr16	81546873	81547311
chr16	81547579	81547825
chr16	81551191	81551312
chr16	81551689	81552446
chr16	81552642	81553095
chr16	81554007	81554032
chr16	81555582	81555623
chr16	81556601	81557791
chr16	81558083	81558370
chr16	81559813	81561090
chr16	81561899	81562143
chr16	81562594	81563066
chr16	81563535	81565204
chr16	81565430	81565887
chr16	81566080	81566738
chr16	81567538	81567744

chr16	81569529	81569689
chr16	81574068	81574267
chr16	81574861	81574881
chr16	81581597	81581990
chr16	81582326	81582514
chr16	81583513	81583712
chr16	81584227	81585200
chr16	81585342	81585838
chr16	81585957	81586059
chr16	81586251	81586591
chr16	81588146	81588345
chr16	81593571	81593760
chr16	81594536	81594749
chr16	81601480	81601777
chr16	81601899	81602105
chr16	81602357	81602470
chr16	81606730	81606823
chr16	81609308	81609507
chr16	81609772	81609835
chr16	81614238	81614847
chr16	81614959	81615832
chr16	81615907	81616053
chr16	81616496	81617601
chr16	81617794	81618160
chr16	81618353	81618611
chr16	81619513	81619733
chr16	81620219	81620329
chr16	81632374	81632771
chr16	81632911	81633196
chr16	81633766	81635297
chr16	81635456	81635734
chr16	81636592	81636985
chr16	81637313	81637413
chr16	81638104	81638576
chr16	81638843	81639311
chr16	81639688	81639887
chr16	81644983	81645182
chr16	81647502	81647720
chr16	81649305	81649366
chr16	81654174	81655161
chr16	81655357	81655900
chr16	81656571	81656795
chr16	81657267	81657655
chr16	81658681	81659457
chr16	81659616	81660037
chr16	81660521	81660758

chr16	81669924	81670115
chr16	81679923	81680027
chr16	81680115	81680225
chr16	81680639	81680810
chr16	81681403	81681579
chr16	81692867	81692899
chr16	81693927	81694104
chr16	81717566	81717609
chr16	81729094	81729535
chr16	81732598	81732684
chr16	81745106	81745179
chr16	81745509	81745768
chr16	81749888	81750087
chr16	81751413	81751612
chr16	81751894	81751899
chr16	81752167	81752860
chr16	81753065	81753412
chr16	81756649	81756812
chr16	81760649	81760848
chr16	81761827	81762124
chr16	81763276	81764798
chr16	81771294	81772025
chr16	81774052	81774251
chr16	81779230	81779904
chr16	81789132	81789197
chr16	81789440	81789465
chr16	81794663	81794862
chr16	81811730	81811804
chr16	81812635	81813357
chr16	81861407	81861663
chr16	81861970	81862169
chr16	81922020	81922178
chr16	81923971	81924377
chr16	81928272	81928447
chr16	81939256	81939511
chr16	81941821	81942185
chr16	81942444	81942842
chr16	81943045	81943095
chr16	81943777	81943916
chr16	81945457	81945482
chr16	81962395	81962463
chr16	81962593	81963743
chr16	81964024	81964323
chr16	81971614	81971791
chr16	82030371	82031793
chr16	82044674	82045322

chr16	82050537	82050538
chr16	82050646	82050753
chr16	82065038	82065180
chr16	82067113	82067142
chr16	82068480	82068679
chr16	82089270	82089906
chr16	82090066	82090277
chr16	82095531	82095808
chr16	82096094	82096236
chr16	82096449	82096833
chr16	82097357	82097909
chr16	82104292	82105407
chr16	82145431	82145631
chr16	82145852	82146014
chr16	82166099	82166420
chr16	82172758	82173303
chr16	82203323	82204482
chr16	82226267	82226474
chr16	82226714	82226763
chr16	82230881	82231724
chr16	82263458	82263640
chr16	82660624	82662187
chr16	82662358	82662769
chr16	82662989	82663712
chr16	82663816	82664620
chr16	82665673	82665944
chr16	82666659	82666829
chr16	82667532	82668256
chr16	82671799	82672420
chr16	82672853	82672918
chr16	82673160	82673439
chr16	82673576	82673688
chr16	82674963	82674965
chr16	82686958	82687999
chr16	82733286	82733495
chr16	82794754	82794914
chr16	82795106	82795309
chr16	82806907	82807009
chr16	83029750	83029818
chr16	83129840	83130258
chr16	83134191	83134382
chr16	83248757	83248905
chr16	83436184	83436198
chr16	83436501	83437052
chr16	83437381	83437412
chr16	83437642	83437858

chr16	83567146	83567328
chr16	83840956	83841155
chr16	83841218	83841448
chr16	83841628	83842382
chr16	83842589	83842788
chr16	83930671	83930909
chr16	83931689	83931834
chr16	83932084	83933173
chr16	83933361	83933574
chr16	83959993	83960069
chr16	83960157	83960234
chr16	83960370	83961107
chr16	83961403	83962097
chr16	83962349	83962741
chr16	83978155	83978354
chr16	83985183	83986014
chr16	83986200	83988205
chr16	83988391	83988786
chr16	84001628	84001827
chr16	84027994	84028193
chr16	84057893	84058115
chr16	84058280	84058479
chr16	84060838	84060893
chr16	84114390	84114690
chr16	84133137	84133922
chr16	84139684	84139734
chr16	84140088	84140133
chr16	84145619	84145898
chr16	84149762	84151486
chr16	84151588	84151756
chr16	84168036	84168203
chr16	84175721	84175762
chr16	84177903	84178805
chr16	84201392	84201554
chr16	84208596	84208716
chr16	84208848	84208972
chr16	84209386	84209585
chr16	84210349	84211419
chr16	84219780	84220957
chr16	84230103	84231330
chr16	84232421	84232674
chr16	84246648	84246871
chr16	84247243	84247562
chr16	84247899	84248063
chr16	84248219	84248609
chr16	84249672	84249877

chr16	84261794	84262015
chr16	84262812	84262847
chr16	84385491	84385845
chr16	84389492	84390037
chr16	84391273	84391398
chr16	84395342	84395350
chr16	84401508	84401802
chr16	84402213	84402518
chr16	84403788	84404593
chr16	84405035	84405112
chr16	84405731	84405847
chr16	84412320	84412956
chr16	84424825	84424867
chr16	84428274	84428390
chr16	84429056	84429199
chr16	84429562	84430529
chr16	84430796	84430869
chr16	84437286	84437407
chr16	84533172	84533526
chr16	84534187	84535963
chr16	84536130	84539071
chr16	84539239	84539566
chr16	84539845	84540442
chr16	84540621	84540719
chr16	84544717	84544891
chr16	84545244	84545448
chr16	84565975	84566454
chr16	84566614	84566813
chr16	84585351	84585627
chr16	84630776	84630942
chr16	84633180	84633302
chr16	84633572	84633824
chr16	84635409	84635772
chr16	84636092	84636291
chr16	84636569	84637655
chr16	84645490	84645667
chr16	84646033	84646267
chr16	84646795	84646928
chr16	84647826	84648025
chr16	84649243	84649363
chr16	84650028	84650354
chr16	84650679	84651644
chr16	84651835	84652549
chr16	84652788	84652935
chr16	84654719	84654918
chr16	84655677	84655915

chr16	84674418	84674555
chr16	84674781	84675218
chr16	84676465	84676818
chr16	84676979	84677673
chr16	84677919	84677969
chr16	84679133	84679288
chr16	84681089	84681191
chr16	84681608	84683194
chr16	84684065	84684076
chr16	84684146	84684310
chr16	84705741	84705913
chr16	84706211	84706342
chr16	84706416	84706534
chr16	84707447	84707516
chr16	84707808	84707851
chr16	84723026	84723177
chr16	84726043	84726289
chr16	84732938	84734534
chr16	84752110	84752367
chr16	84759060	84760117
chr16	84764011	84764994
chr16	84768480	84768753
chr16	84769406	84769585
chr16	84769862	84769889
chr16	84771238	84771413
chr16	84772928	84773169
chr16	84773261	84773331
chr16	84773519	84773686
chr16	84774874	84775220
chr16	84775328	84775527
chr16	84787997	84788402
chr16	84788573	84789039
chr16	84789728	84790137
chr16	84805828	84805922
chr16	84819105	84819463
chr16	84820280	84820522
chr16	84829524	84829677
chr16	84839608	84839740
chr16	84840179	84840378
chr16	84869551	84869750
chr16	84871129	84871477
chr16	84875277	84876586
chr16	84888501	84888700
chr16	84920044	84920239
chr16	84931442	84932391
chr16	84933012	84933022

chr16	84933323	84933503
chr16	84964270	84964559
chr16	84973567	84973807
chr16	84975809	84975989
chr16	84976670	84976990
chr16	84996180	84996386
chr16	85004067	85004156
chr16	85005534	85005986
chr16	85006059	85006214
chr16	85017529	85018089
chr16	85018229	85018428
chr16	85018778	85019686
chr16	85019774	85020110
chr16	85020367	85020936
chr16	85021132	85021156
chr16	85021506	85021725
chr16	85025097	85025382
chr16	85026967	85027180
chr16	85027999	85029555
chr16	85033330	85034117
chr16	85034213	85034572
chr16	85032567	85033169
chr16	85037144	85037162
chr16	85037434	85038827
chr16	85039184	85039849
chr16	85044076	85045637
chr16	85049193	85049502
chr16	85052052	85052378
chr16	85052675	85052877
chr16	85060283	85060299
chr16	85060791	85062386
chr16	85062704	85062939
chr16	85063338	85063871
chr16	85064008	85064215
chr16	85066015	85066199
chr16	85069129	85069328
chr16	85069959	85070537
chr16	85071077	85071116
chr16	85088581	85088758
chr16	85089021	85089287
chr16	85120170	85120517
chr16	85122965	85123553
chr16	85138643	85138842
chr16	85148557	85149106
chr16	85149282	85149377
chr16	85156922	85157211

chr16	85157427	85157957
chr16	85170878	85171143
chr16	85171367	85171621
chr16	85171713	85171772
chr16	85184153	85184548
chr16	85184887	85185331
chr16	85186608	85187299
chr16	85199865	85200169
chr16	85200312	85200587
chr16	85202405	85202630
chr16	85203134	85204274
chr16	85205457	85206300
chr16	85206431	85206659
chr16	85207284	85207513
chr16	85207867	85208607
chr16	85232054	85233053
chr16	85234476	85234896
chr16	85237360	85237811
chr16	85238659	85238828
chr16	85253717	85254703
chr16	85269517	85269718
chr16	85339038	85339237
chr16	85347343	85347812
chr16	85364356	85364555
chr16	85383913	85384112
chr16	85384318	85384701
chr16	85387250	85387440
chr16	85387678	85388036
chr16	85393384	85393751
chr16	85394279	85394501
chr16	85398344	85398797
chr16	85403370	85403656
chr16	85418298	85418497
chr16	85418922	85419228
chr16	85428758	85429084
chr16	85429423	85429652
chr16	85430101	85430618
chr16	85450264	85450565
chr16	85525120	85525376
chr16	85526307	85526931
chr16	85528091	85528302
chr16	85547647	85548336
chr16	85548529	85549559
chr16	85549849	85550143
chr16	85550596	85550643
chr16	85554074	85554442

chr16	85554734	85554933
chr16	85556967	85559440
chr16	85565271	85565470
chr16	85586653	85588377
chr16	85588580	85589008
chr16	85589218	85590382
chr16	85643690	85644059
chr16	85644399	85645843
chr16	85646004	85648845
chr16	85648994	85649319
chr16	85694056	85694208
chr16	85694283	85694421
chr16	85709388	85709552
chr16	85715721	85715770
chr16	85721554	85722433
chr16	85724838	85724910
chr16	85725144	85725150
chr16	85725695	85725957
chr16	85727501	85727802
chr16	85731042	85731313
chr16	85739554	85739663
chr16	85744672	85744734
chr16	85750213	85750334
chr16	85754749	85755056
chr16	85753907	85754424
chr16	85756656	85756730
chr16	85761055	85761254
chr16	85765224	85765469
chr16	85765673	85765979
chr16	85766070	85767868
chr16	85771778	85772293
chr16	85778658	85781645
chr16	85781847	85785753
chr16	85785941	85787287
chr16	85787441	85787904
chr16	85789291	85789528
chr16	85790670	85791436
chr16	85793894	85794114
chr16	85801597	85802121
chr16	85805877	85805952
chr16	85806066	85806103
chr16	85806323	85806530
chr16	85811133	85811528
chr16	85818936	85818999
chr16	85832337	85834279
chr16	86543213	86543431

chr16	86588401	86589391
chr16	86597054	86597523
chr16	86600469	86600732
chr16	86609119	86609360
chr16	86609570	86610300
chr16	86610704	86610912
chr16	86612176	86613318
chr16	86697392	86697419
chr16	86697599	86697982
chr16	86698053	86698847
chr16	86699047	86699208
chr16	86751495	86751626
chr16	86752215	86752311
chr16	86752392	86752579
chr16	86795426	86795676
chr16	86806411	86806694
chr16	86848111	86848322
chr16	86857467	86857610
chr16	86858826	86858975
chr16	86870304	86870734
chr16	86871114	86871600
chr16	86949663	86949822
chr16	86964756	86965105
chr16	86965644	86965909
chr16	86980336	86980548
chr16	86985370	86985933
chr16	86986168	86986326
chr16	87051416	87051580
chr16	87078012	87078029
chr16	87096295	87096302
chr16	87211796	87212221
chr16	87240775	87240989
chr16	87271590	87271779
chr16	87271939	87272047
chr16	87273648	87273955
chr16	87274206	87274496
chr16	87350563	87351874
chr16	87410098	87410701
chr16	87408614	87409733
chr16	87416032	87418690
chr16	87421585	87422469
chr16	87422979	87423178
chr16	87424680	87425723
chr16	87427972	87428157
chr16	87425942	87427449
chr16	87435426	87435688

chr16	87467430	87467568
chr16	87469779	87470351
chr16	87484882	87484949
chr16	87491338	87491396
chr16	87491564	87491763
chr16	87494110	87494252
chr16	87495349	87495645
chr16	87497689	87497805
chr16	87498016	87498999
chr16	87502109	87502118
chr16	87511328	87511545
chr16	87518624	87519010
chr16	87519401	87519643
chr16	87520881	87521035
chr16	87521603	87521644
chr16	87521803	87522152
chr16	87522278	87522412
chr16	87524002	87524289
chr16	87524443	87525448
chr16	87526931	87527432
chr16	87551023	87552376
chr16	87552513	87552712
chr16	87772269	87772815
chr16	87799133	87800069
chr16	87802033	87802409
chr16	87802543	87802839
chr16	87809054	87809221
chr16	87810219	87810405
chr16	87810863	87811456
chr16	87811728	87811993
chr16	87812142	87813279
chr16	87813514	87813807
chr16	87814136	87814829
chr16	87815715	87816007
chr16	87816365	87816784
chr16	87817143	87817154
chr16	87819559	87819626
chr16	87820002	87820328
chr16	87822926	87825324
chr16	87825577	87826252
chr16	87826742	87827086
chr16	87827218	87828082
chr16	87828184	87828913
chr16	87829526	87829738
chr16	87830219	87830561
chr16	87830886	87831274

chr16	87831706	87832058
chr16	87833279	87833394
chr16	87834524	87834754
chr16	87837725	87838138
chr16	87838450	87839361
chr16	87839458	87840511
chr16	87840824	87841297
chr16	87841842	87841870
chr16	87842696	87842934
chr16	87843371	87843582
chr16	87845284	87845606
chr16	87847428	87847766
chr16	87854788	87855116
chr16	87855776	87855940
chr16	87856302	87856911
chr16	87857036	87857975
chr16	87858334	87858583
chr16	87859427	87859879
chr16	87860098	87860387
chr16	87860541	87862458
chr16	87863733	87864170
chr16	87864504	87864861
chr16	87867414	87867653
chr16	87868144	87868343
chr16	87869238	87869904
chr16	87870904	87872580
chr16	87872697	87873974
chr16	87874366	87876291
chr16	87876410	87877178
chr16	87879819	87880104
chr16	87880431	87880803
chr16	87881826	87882189
chr16	87882410	87882701
chr16	87883179	87883697
chr16	87884457	87887526
chr16	87887961	87888970
chr16	87889566	87889790
chr16	87890081	87891499
chr16	87892061	87892297
chr16	87892544	87894656
chr16	87894917	87896333
chr16	87896445	87896723
chr16	87896836	87897389
chr16	87897560	87905244
chr16	87905447	87905527
chr16	87906998	87907091

chr16	87936402	87936459
chr16	87943548	87943747
chr16	87945765	87945964
chr16	87953881	87954086
chr16	87958406	87958740
chr16	87964375	87964596
chr16	87979037	87979258
chr16	87979443	87979786
chr16	87979992	87980071
chr16	87980360	87980580
chr16	87980962	87981161
chr16	87981738	87981752
chr16	87982735	87982762
chr16	87983468	87983496
chr16	87984101	87986171
chr16	87986520	87986786
chr16	87987510	87987876
chr16	88157766	88157835
chr16	88268032	88268253
chr16	88268412	88268660
chr16	88269356	88269555
chr16	88270205	88270267
chr16	88271364	88271476
chr16	88298094	88298541
chr16	88298840	88299382
chr16	88299754	88299953
chr16	88332295	88332549
chr16	88349121	88349484
chr16	88349615	88349960
chr16	88365405	88365604
chr16	88366178	88366813
chr16	88367313	88367880
chr16	88491224	88491504
chr16	88497038	88497374
chr16	88519016	88519508
chr16	88519652	88519913
chr16	88520112	88520502
chr16	88521119	88521338
chr16	88521453	88521820
chr16	88521951	88522434
chr16	88522558	88522775
chr16	88575669	88576860
chr16	88599533	88600146
chr16	88600715	88601038
chr16	88601335	88601838
chr16	88629317	88629501

chr16	88635907	88637484
chr16	88666771	88666963
chr16	88674842	88675039
chr16	88700995	88701798
chr16	88703630	88704134
chr16	88726086	88726348
chr16	88726804	88726883
chr16	88728688	88730747
chr16	88731382	88731673
chr16	88734540	88734739
chr16	88742011	88742261
chr16	88746081	88746301
chr16	88746874	88747195
chr16	88750920	88751501
chr16	88751736	88752352
chr16	88752589	88753010
chr16	88767114	88767313
chr16	88767709	88767908
chr16	88771996	88773958
chr16	88811883	88812100
chr16	88821197	88821897
chr16	88822126	88822631
chr16	88826863	88827322
chr16	88830984	88831468
chr16	88836592	88837366
chr16	88837771	88838075
chr16	88838359	88838558
chr16	88846739	88847389
chr16	88847880	88848757
chr16	88848891	88849363
chr16	88849522	88849805
chr16	88850000	88852265
chr16	88852674	88852800
chr16	88852480	88852604
chr16	88856881	88857151
chr16	88862393	88862592
chr16	88867823	88868070
chr16	88868352	88870921
chr16	88871156	88871426
chr16	88877219	88877450
chr16	88877667	88879789
chr16	88892126	88892456
chr16	88892768	88893312
chr16	88922547	88922961
chr16	88923173	88923419
chr16	88923538	88924273

chr16	88946971	88947194
chr16	88947379	88948390
chr16	88984803	88985748
chr16	88986418	88986872
chr16	88987089	88988115
chr16	88988737	88988936
chr16	88993131	88993330
chr16	89118842	89119249
chr16	89119403	89119916
chr16	89159884	89161167
chr16	89161407	89161451
chr16	89181379	89181643
chr16	89189303	89189915
chr16	89237400	89237682
chr16	89259330	89259529
chr16	89266642	89268043
chr16	89282391	89282628
chr16	89283035	89283313
chr16	89283649	89284691
chr16	89306714	89307452
chr16	89307645	89308537
chr16	89310856	89311140
chr16	89313410	89314259
chr16	89322616	89322850
chr16	89322913	89323406
chr16	89323549	89324458
chr16	89336038	89336253
chr16	89368480	89368973
chr16	89381882	89382454
chr16	89386051	89386800
chr16	89387121	89387606
chr16	89388621	89388825
chr16	89389073	89389341
chr16	89392116	89392174
chr16	89392289	89393194
chr16	89396191	89396436
chr16	89397235	89397339
chr16	89400276	89400870
chr16	89401100	89401203
chr16	89406677	89406922
chr16	89409633	89409756
chr16	89412240	89412594
chr16	89418367	89418664
chr16	89418892	89419322
chr16	89419878	89420077
chr16	89423344	89423346

chr16	89427720	89427987
chr16	89428716	89429216
chr16	89429343	89429635
chr16	89429893	89430698
chr16	89431084	89431332
chr16	89431725	89431971
chr16	89432278	89432557
chr16	89432928	89432997
chr16	89434261	89434436
chr16	89436434	89437164
chr16	89449344	89449534
chr16	89450417	89450747
chr16	89451088	89451252
chr16	89451432	89451644
chr16	89457785	89458826
chr16	89458893	89459177
chr16	89459302	89459672
chr16	89460418	89460688
chr16	89461434	89461594
chr16	89462011	89462281
chr16	89470463	89470734
chr16	89471649	89471829
chr16	89476103	89476178
chr16	89476278	89476630
chr16	89482721	89482979
chr16	89506461	89506474
chr16	89506967	89507070
chr16	89508379	89509751
chr16	89510022	89511518
chr16	89512008	89512190
chr16	89512653	89512939
chr16	89515356	89515715
chr16	89516159	89516280
chr16	89516581	89516659
chr16	89522280	89522388
chr16	89524868	89525634
chr16	89526310	89526426
chr16	89526716	89527046
chr16	89527767	89527917
chr16	89532094	89532212
chr16	89536967	89537153
chr16	89537816	89538094
chr16	89538827	89539058
chr16	89540371	89540803
chr16	89541298	89541543
chr16	89542733	89542937

chr16	89543053	89544356
chr16	89544909	89545935
chr16	89546083	89546403
chr16	89548621	89548853
chr16	89555381	89557133
chr16	89557381	89558355
chr16	89563496	89563641
chr16	89564383	89564417
chr16	89565719	89565998
chr16	89566305	89566511
chr16	89568412	89569469
chr16	89570171	89570372
chr16	89571132	89572663
chr16	89573291	89576107
chr16	89577135	89577237
chr16	89594479	89594699
chr16	89602483	89602819
chr16	89624968	89626975
chr16	89627143	89628512
chr16	89628694	89628979
chr16	89629127	89629375
chr16	89629735	89630119
chr16	89630809	89631214
chr16	89631512	89631943
chr16	89632086	89632490
chr16	89640818	89641133
chr16	89641298	89643395
chr16	89647480	89647735
chr16	89647896	89648411
chr16	89693425	89693624
chr16	89693856	89694342
chr16	89699531	89699807
chr16	89700540	89700739
chr16	89701121	89701525
chr16	89713838	89714037
chr16	89719569	89719754
chr16	89722094	89722130
chr16	89723197	89725202
chr16	89725311	89725486
chr16	89737257	89737549
chr16	89753098	89754192
chr16	89754345	89754431
chr16	89767008	89768471
chr16	89771913	89772669
chr16	89778231	89779889
chr16	89780014	89780173

chr16	89785086	89789213
chr16	89789527	89789745
chr16	89790986	89791185
chr16	89792144	89792248
chr16	89794671	89794872
chr16	89808357	89808369
chr16	89808436	89808783
chr16	89830922	89831049
chr16	89831162	89831386
chr16	89831735	89831901
chr16	89873794	89874131
chr16	89879976	89880077
chr16	89882093	89883823
chr16	89894407	89894724
chr16	89895071	89895398
chr16	89904386	89904938
chr16	89923507	89923807
chr16	89930626	89930979
chr16	89938627	89940550
chr16	89940839	89941183
chr16	89945085	89945334
chr16	89946743	89947425
chr16	89947631	89948241
chr16	89982939	89984144
chr16	89984482	89985238
chr16	89987733	89990148
chr16	90014618	90015093
chr16	90015655	90016272
chr16	90038336	90039765
chr16	90049451	90049561
chr16	90059414	90059467
chr16	90060830	90061894
chr16	90062059	90062721
chr16	90066333	90066532
chr16	90077719	90077933
chr16	90085015	90086702
chr16	90087946	90088355
chr16	90088595	90089788
chr16	90090093	90090176
chr16	90090431	90090672
chr16	90110309	90110511
chr16	90111884	90112106
chr16	90112336	90112535
chr16	90113243	90114207
chr16	90143975	90144590
chr16	90148318	90148433

chr16	90148625	90148834
chr16	90165233	90165946
chr16	90166970	90167169
chr16	90168572	90168809
chr16	90168947	90169463
chr16	90182938	90183023
chr16	90183190	90183732
chr16	90184145	90184472
chr16	90189851	90190193
chr16	90224329	90224519
chr16	90237399	90237745
chr16	90276511	90276633
chr16	90286372	90286538
chr17	199190	199270
chr17	200432	200957
chr17	235450	235860
chr17	259425	259727
chr17	260158	260467
chr17	260614	260729
chr17	261163	261178
chr17	262038	262050
chr17	262208	262292
chr17	449924	450378
chr17	461726	461961
chr17	467658	467919
chr17	468396	468449
chr17	500884	500928
chr17	525605	526065
chr17	572571	573002
chr17	596080	596382
chr17	597798	598002
chr17	617445	618124
chr17	618252	619629
chr17	624763	625306
chr17	628978	629503
chr17	630583	631184
chr17	632125	632254
chr17	632424	632848
chr17	633038	633998
chr17	634919	636775
chr17	636998	637095
chr17	637254	637407
chr17	646975	647021
chr17	654275	654745
chr17	654862	656220
chr17	684564	684960

chr17	685086	686097
chr17	691763	691809
chr17	691913	692081
chr17	692557	693092
chr17	693340	693777
chr17	717067	717486
chr17	742734	742964
chr17	767730	768021
chr17	771344	771548
chr17	773158	774111
chr17	781003	781106
chr17	782748	783307
chr17	783398	783707
chr17	784079	784478
chr17	786934	786947
chr17	787230	787352
chr17	787482	787585
chr17	805898	806097
chr17	806906	807700
chr17	812675	813543
chr17	813979	814333
chr17	814490	814966
chr17	817658	817695
chr17	818572	819006
chr17	823285	823529
chr17	823902	824219
chr17	824336	825431
chr17	825823	826031
chr17	826148	826170
chr17	828007	829160
chr17	829735	830489
chr17	830565	831991
chr17	836110	836291
chr17	836779	837066
chr17	837964	838558
chr17	839010	839292
chr17	840023	840524
chr17	844376	844625
chr17	850938	851181
chr17	853823	854216
chr17	854457	854648
chr17	854919	855301
chr17	855836	856041
chr17	858277	858381
chr17	858614	858708
chr17	858852	859004

chr17	859801	860019
chr17	860188	860387
chr17	860540	860858
chr17	863785	863905
chr17	865386	865811
chr17	865983	866143
chr17	873695	873862
chr17	876193	876446
chr17	876616	876822
chr17	877300	877510
chr17	880197	880754
chr17	881370	882333
chr17	882738	883641
chr17	884009	884641
chr17	899625	899860
chr17	899980	900826
chr17	964039	964122
chr17	965522	965732
chr17	967124	967338
chr17	968614	968777
chr17	970398	970461
chr17	984152	984691
chr17	984839	985182
chr17	1001514	1001729
chr17	1002051	1002061
chr17	1002342	1002477
chr17	1011233	1013353
chr17	1014241	1014440
chr17	1014880	1015139
chr17	1017362	1017802
chr17	1018058	1018657
chr17	1018802	1019259
chr17	1026139	1026348
chr17	1082790	1083205
chr17	1106886	1107147
chr17	1125991	1126004
chr17	1132926	1133183
chr17	1148457	1148471
chr17	1162442	1162595
chr17	1162895	1163332
chr17	1170786	1172438
chr17	1259848	1260052
chr17	1263766	1263977
chr17	1268767	1268876
chr17	1268993	1269130
chr17	1269431	1269641

chr17	1269984	1270341
chr17	1270623	1270833
chr17	1271316	1271659
chr17	1276541	1276666
chr17	1284598	1284681
chr17	1286794	1286963
chr17	1289989	1290188
chr17	1291016	1291180
chr17	1292779	1293074
chr17	1295769	1295985
chr17	1302347	1303632
chr17	1303757	1304294
chr17	1321807	1322006
chr17	1326538	1326626
chr17	1326813	1327635
chr17	1330588	1330707
chr17	1342169	1342404
chr17	1347600	1347844
chr17	1358011	1358551
chr17	1358783	1359556
chr17	1360924	1360964
chr17	1359816	1360814
chr17	1363165	1363510
chr17	1363886	1364150
chr17	1364687	1364872
chr17	1365295	1365605
chr17	1366058	1366686
chr17	1367105	1367357
chr17	1388388	1389839
chr17	1389909	1392082
chr17	1392641	1393182
chr17	1393554	1395868
chr17	1398253	1398538
chr17	1398707	1398906
chr17	1402500	1402562
chr17	1414520	1414711
chr17	1415232	1415986
chr17	1418186	1419044
chr17	1419576	1419844
chr17	1420381	1420995
chr17	1421566	1421574
chr17	1439954	1440156
chr17	1448565	1448626
chr17	1448848	1450013
chr17	1450704	1450826
chr17	1464709	1465969

chr17	1466167	1466911
chr17	1477898	1479280
chr17	1479440	1479771
chr17	1480093	1481540
chr17	1482847	1483046
chr17	1485125	1485606
chr17	1488432	1488888
chr17	1492901	1493337
chr17	1493622	1494172
chr17	1500862	1501214
chr17	1510990	1511196
chr17	1520604	1521185
chr17	1521397	1521554
chr17	1525982	1525985
chr17	1526074	1526181
chr17	1530209	1530420
chr17	1531009	1531655
chr17	1531823	1533412
chr17	1546028	1546343
chr17	1546695	1548163
chr17	1548288	1548496
chr17	1551691	1552265
chr17	1552463	1553420
chr17	1572643	1572779
chr17	1573199	1573287
chr17	1587558	1588736
chr17	1589270	1589756
chr17	1589856	1590111
chr17	1612422	1612705
chr17	1613049	1614595
chr17	1616690	1617969
chr17	1618121	1619533
chr17	1619892	1621260
chr17	1622980	1623248
chr17	1623690	1624755
chr17	1624893	1625105
chr17	1625358	1625557
chr17	1625709	1625777
chr17	1625877	1626329
chr17	1627390	1628556
chr17	1632812	1633370
chr17	1633864	1634723
chr17	1634945	1635270
chr17	1639537	1640075
chr17	1649788	1650513
chr17	1650790	1651251

chr17	1655530	1656943
chr17	1659585	1659792
chr17	1662825	1662886
chr17	1667060	1667553
chr17	1667862	1668022
chr17	1671935	1672020
chr17	1672642	1674438
chr17	1681415	1681496
chr17	1683022	1683226
chr17	1683330	1683910
chr17	1691140	1691287
chr17	1693873	1694091
chr17	1696578	1697258
chr17	1715003	1715205
chr17	1718420	1718656
chr17	1718796	1718920
chr17	1732243	1733684
chr17	1733776	1733974
chr17	1751635	1752038
chr17	1768889	1768899
chr17	1884377	1884757
chr17	1885198	1885581
chr17	1885950	1885966
chr17	1886358	1886716
chr17	1891437	1892259
chr17	1892813	1892993
chr17	1901091	1901290
chr17	1901430	1902061
chr17	1902488	1903060
chr17	1913742	1914140
chr17	1914619	1914877
chr17	1915191	1915390
chr17	1916430	1916629
chr17	1927149	1927683
chr17	1927796	1928874
chr17	1929078	1929376
chr17	1933536	1934134
chr17	1934473	1934521
chr17	1944736	1946272
chr17	1947058	1947909
chr17	1948041	1948200
chr17	1952313	1953243
chr17	1953392	1954126
chr17	1957184	1957882
chr17	1958386	1959295
chr17	1994637	1994933

chr17	1996382	1996866
chr17	2004726	2004747
chr17	2004931	2005263
chr17	2016082	2016281
chr17	2021323	2021541
chr17	2040149	2040368
chr17	2061280	2061333
chr17	2061532	2061623
chr17	2080877	2081085
chr17	2093572	2093661
chr17	2095699	2096154
chr17	2096264	2096624
chr17	2121671	2121830
chr17	2130573	2130586
chr17	2130726	2130925
chr17	2143325	2143485
chr17	2148006	2148051
chr17	2155673	2155677
chr17	2161617	2161866
chr17	2186541	2186621
chr17	2206086	2207668
chr17	2215652	2215738
chr17	2220010	2220487
chr17	2235147	2235325
chr17	2239130	2241527
chr17	2242175	2242176
chr17	2242262	2242607
chr17	2256703	2256768
chr17	2256834	2257124
chr17	2261686	2261969
chr17	2262278	2264609
chr17	2264922	2265429
chr17	2278312	2278561
chr17	2286117	2286447
chr17	2295666	2297791
chr17	2299966	2301108
chr17	2302173	2304949
chr17	2305053	2305997
chr17	2306256	2306557
chr17	2306961	2311271
chr17	2315675	2315750
chr17	2315854	2315874
chr17	2317758	2317949
chr17	2319371	2319466
chr17	2325280	2325472
chr17	2326051	2326433

chr17	2326798	2327155
chr17	2362131	2362348
chr17	2362830	2363203
chr17	2388104	2388361
chr17	2388472	2388658
chr17	2389081	2389247
chr17	2390860	2390967
chr17	2391618	2391833
chr17	2396327	2396661
chr17	2400356	2400597
chr17	2403558	2403697
chr17	2414608	2415159
chr17	2415337	2415729
chr17	2431988	2432160
chr17	2450339	2450609
chr17	2450823	2450907
chr17	2451281	2451881
chr17	2453205	2453404
chr17	2453602	2453669
chr17	2456319	2456513
chr17	2463978	2463994
chr17	2466196	2466329
chr17	2466577	2467220
chr17	2467307	2467666
chr17	2468141	2468976
chr17	2475504	2476170
chr17	2495699	2495920
chr17	2496267	2498106
chr17	2520996	2521166
chr17	2537528	2537910
chr17	2538172	2538375
chr17	2553655	2554120
chr17	2562547	2562967
chr17	2591588	2591630
chr17	2612185	2612384
chr17	2612591	2613170
chr17	2613343	2615093
chr17	2615257	2616574
chr17	2617282	2617693
chr17	2620806	2621199
chr17	2621424	2621570
chr17	2621836	2622199
chr17	2626685	2627260
chr17	2627383	2628496
chr17	2639811	2640378
chr17	2640644	2640750

chr17	2641009	2641235
chr17	2658477	2658550
chr17	2658738	2659346
chr17	2679641	2679883
chr17	2689966	2690279
chr17	2715136	2715419
chr17	2715885	2716061
chr17	2736259	2736301
chr17	2751818	2752161
chr17	2756205	2757258
chr17	2757406	2757792
chr17	2758036	2758428
chr17	2758538	2758615
chr17	2785541	2785789
chr17	2785858	2785978
chr17	2790366	2790550
chr17	2796061	2796320
chr17	2796459	2796712
chr17	2796913	2797423
chr17	2797654	2797781
chr17	2829009	2829456
chr17	2829623	2830055
chr17	2853252	2853431
chr17	2857561	2857982
chr17	2858261	2858834
chr17	2859215	2859404
chr17	2860371	2860662
chr17	2862548	2862713
chr17	2863031	2863060
chr17	2863308	2864460
chr17	2869061	2869389
chr17	2878956	2879151
chr17	2907592	2908295
chr17	2952420	2952896
chr17	3460757	3461074
chr17	3461427	3461863
chr17	3462105	3462316
chr17	3462466	3462624
chr17	3466081	3466415
chr17	3479125	3479365
chr17	3535545	3535741
chr17	3538175	3538624
chr17	3538769	3540020
chr17	3540150	3540392
chr17	3570145	3571866
chr17	3572585	3572647

chr17	3572125	3572491
chr17	3599286	3599531
chr17	3599665	3599771
chr17	3623255	3623356
chr17	3623505	3623970
chr17	3624292	3624496
chr17	3626655	3627910
chr17	3637555	3637700
chr17	3641478	3641738
chr17	3641806	3641814
chr17	3655841	3656037
chr17	3657447	3657915
chr17	3658212	3658572
chr17	3658769	3659038
chr17	3659594	3659649
chr17	3712444	3712848
chr17	3713174	3713371
chr17	3746675	3746866
chr17	3748636	3749534
chr17	3766989	3767030
chr17	3790670	3790944
chr17	3791110	3794104
chr17	3795067	3797124
chr17	3798490	3798764
chr17	3836962	3836996
chr17	3837064	3837213
chr17	3848397	3848706
chr17	3852125	3852324
chr17	3984269	3984359
chr17	4045501	4046241
chr17	4046446	4047697
chr17	4051045	4051064
chr17	4051681	4051988
chr17	4052863	4053062
chr17	4053923	4054122
chr17	4063703	4065483
chr17	4066120	4066265
chr17	4067262	4067390
chr17	4090333	4090918
chr17	4118660	4118810
chr17	4133129	4133433
chr17	4133685	4133853
chr17	4135454	4135863
chr17	4150904	4151070
chr17	4151178	4152089
chr17	4166357	4167771

chr17	4167899	4167908
chr17	4230178	4230340
chr17	4230880	4231003
chr17	4231202	4231375
chr17	4233083	4233275
chr17	4233434	4233624
chr17	4236085	4236260
chr17	4247899	4248098
chr17	4258403	4258811
chr17	4268386	4268549
chr17	4268678	4269915
chr17	4270024	4270882
chr17	4277454	4277541
chr17	4295566	4296201
chr17	4296921	4298070
chr17	4299365	4299571
chr17	4300552	4300604
chr17	4310471	4310496
chr17	4310632	4310859
chr17	4311207	4311477
chr17	4311613	4311719
chr17	4312978	4313010
chr17	4313182	4313334
chr17	4313454	4313773
chr17	4315356	4315389
chr17	4316319	4316650
chr17	4332781	4332925
chr17	4333123	4333151
chr17	4333386	4333697
chr17	4347161	4347380
chr17	4379939	4380338
chr17	4385214	4385322
chr17	4385931	4386914
chr17	4390502	4390620
chr17	4391118	4391600
chr17	4392939	4393433
chr17	4393790	4394196
chr17	4394464	4394744
chr17	4416408	4416607
chr17	4417006	4417483
chr17	4417708	4418015
chr17	4419359	4419413
chr17	4419558	4419985
chr17	4420727	4420940
chr17	4422974	4423637
chr17	4423725	4423762

chr17	4424060	4424065
chr17	4424380	4424529
chr17	4424821	4425250
chr17	4425767	4425869
chr17	4432715	4432833
chr17	4439888	4440253
chr17	4440617	4440816
chr17	4451674	4451882
chr17	4452653	4453069
chr17	4453147	4453322
chr17	4456378	4456516
chr17	4458027	4458649
chr17	4458809	4459341
chr17	4463414	4463855
chr17	4464015	4464273
chr17	4465920	4466064
chr17	4468649	4468924
chr17	4469126	4469305
chr17	4507209	4507401
chr17	4507672	4507871
chr17	4509473	4509543
chr17	4509617	4509672
chr17	4543338	4543540
chr17	4543953	4544152
chr17	4581745	4582167
chr17	4603331	4603599
chr17	4606942	4608035
chr17	4613062	4614635
chr17	4627919	4627968
chr17	4631642	4631646
chr17	4632124	4632221
chr17	4633913	4635070
chr17	4635353	4635372
chr17	4638962	4638985
chr17	4639241	4639285
chr17	4639508	4639829
chr17	4641478	4643451
chr17	4690268	4690448
chr17	4690511	4691045
chr17	4692205	4694185
chr17	4698752	4699330
chr17	4699495	4699732
chr17	4699894	4700256
chr17	4700659	4700683
chr17	4709770	4709808
chr17	4709981	4711985

chr17	4712104	4712372
chr17	4713698	4713805
chr17	4714711	4714835
chr17	4715122	4715250
chr17	4715763	4715939
chr17	4716090	4716780
chr17	4725542	4725683
chr17	4735631	4738323
chr17	4735042	4735231
chr17	4749871	4750227
chr17	4750435	4750529
chr17	4752039	4753373
chr17	4753450	4754458
chr17	4750753	4751604
chr17	4754938	4757386
chr17	4757489	4758063
chr17	4758218	4758647
chr17	4759333	4759749
chr17	4760486	4762292
chr17	4762396	4762727
chr17	4764077	4764089
chr17	4764252	4764342
chr17	4765718	4766065
chr17	4766130	4766190
chr17	4767202	4767502
chr17	4767884	4767939
chr17	4769164	4769382
chr17	4769776	4770460
chr17	4773403	4773659
chr17	4774415	4774679
chr17	4777798	4777957
chr17	4783418	4783740
chr17	4784016	4784246
chr17	4784797	4785055
chr17	4786548	4786812
chr17	4787642	4787700
chr17	4807051	4807251
chr17	4811472	4812428
chr17	4812582	4813541
chr17	4813799	4814009
chr17	4815623	4815714
chr17	4823576	4823959
chr17	4824066	4824198
chr17	4837090	4837199
chr17	4837284	4837289
chr17	4838344	4838770

chr17	4842200	4844406
chr17	4844890	4845175
chr17	4846306	4846505
chr17	4846949	4847265
chr17	4847450	4848489
chr17	4848724	4849315
chr17	4849627	4849911
chr17	4850286	4854438
chr17	4866336	4866368
chr17	4867806	4867841
chr17	4868178	4868257
chr17	4868556	4868571
chr17	4869516	4870571
chr17	4870708	4873347
chr17	4879628	4879931
chr17	4888961	4891385
chr17	4891575	4891655
chr17	4900357	4902904
chr17	4903121	4903335
chr17	4903940	4904139
chr17	4919447	4919609
chr17	4930866	4931116
chr17	4934690	4935757
chr17	4938198	4938384
chr17	4938529	4939594
chr17	4945936	4945985
chr17	4965665	4965761
chr17	4965922	4966419
chr17	4967862	4967977
chr17	4980415	4980477
chr17	4981045	4982264
chr17	5014491	5016020
chr17	5025807	5026355
chr17	5026572	5026811
chr17	5094651	5095158
chr17	5095398	5095555
chr17	5100165	5100540
chr17	5115781	5115998
chr17	5144934	5145175
chr17	5145840	5146202
chr17	5153002	5153261
chr17	5185012	5185357
chr17	5185514	5186388
chr17	5187238	5187571
chr17	5267490	5267840
chr17	5268911	5269511

chr17	5270610	5270864
chr17	5317000	5317190
chr17	5317375	5317504
chr17	5320541	5320572
chr17	5321558	5321757
chr17	5322301	5323349
chr17	5323531	5324131
chr17	5326230	5326280
chr17	5335189	5335671
chr17	5341998	5342518
chr17	5341062	5341460
chr17	5371594	5372234
chr17	5372462	5372609
chr17	5389210	5389419
chr17	5389771	5390416
chr17	5419318	5419776
chr17	5487125	5487912
chr17	5489311	5489393
chr17	5488152	5488938
chr17	5489861	5490074
chr17	5552149	5552364
chr17	5732650	5732825
chr17	5737833	5738232
chr17	5738343	5738542
chr17	5740777	5740860
chr17	6064654	6064784
chr17	6064947	6065060
chr17	6135097	6135359
chr17	6135509	6135965
chr17	6143076	6143364
chr17	6154926	6155026
chr17	6155334	6155613
chr17	6159985	6160121
chr17	6160424	6160672
chr17	6161175	6161264
chr17	6330899	6330901
chr17	6346772	6348148
chr17	6377053	6377267
chr17	6379873	6380213
chr17	6409880	6410159
chr17	6410520	6410996
chr17	6426653	6426839
chr17	6427371	6427570
chr17	6454623	6456481
chr17	6457198	6457302
chr17	6457684	6459824

chr17	6460177	6460436
chr17	6463902	6463960
chr17	6470619	6470947
chr17	6472457	6472571
chr17	6478511	6478959
chr17	6494819	6494951
chr17	6495114	6495471
chr17	6511821	6512020
chr17	6542784	6545227
chr17	6546096	6546208
chr17	6547112	6547339
chr17	6554225	6554839
chr17	6554988	6555979
chr17	6558022	6558553
chr17	6558797	6559139
chr17	6559617	6559931
chr17	6560065	6561309
chr17	6569188	6569406
chr17	6569643	6569845
chr17	6569959	6570032
chr17	6572431	6573094
chr17	6573261	6573587
chr17	6573788	6574071
chr17	6574262	6575005
chr17	6585031	6585242
chr17	6602954	6603175
chr17	6614681	6614743
chr17	6615018	6615088
chr17	6616110	6616309
chr17	6655826	6656165
chr17	6658982	6659181
chr17	6659573	6659740
chr17	6757275	6757750
chr17	6771957	6772019
chr17	6772262	6772391
chr17	6775296	6775520
chr17	6775728	6775863
chr17	6810645	6810979
chr17	6826879	6827150
chr17	6898407	6900132
chr17	6900804	6900837
chr17	6914662	6915029
chr17	6915507	6919136
chr17	6919689	6919950
chr17	6923080	6923641
chr17	6924743	6925455

chr17	6925605	6925804
chr17	6926129	6927007
chr17	6938568	6939161
chr17	6939398	6940230
chr17	6940385	6940613
chr17	6940809	6941259
chr17	6945497	6947741
chr17	6948149	6948467
chr17	6948813	6950704
chr17	6963618	6963833
chr17	6964237	6964516
chr17	7038777	7038937
chr17	7039376	7039409
chr17	7078286	7078454
chr17	7077767	7078028
chr17	7080652	7080811
chr17	7080958	7081183
chr17	7086528	7086568
chr17	7117751	7118172
chr17	7118662	7119112
chr17	7120359	7121421
chr17	7122608	7123832
chr17	7123950	7124435
chr17	7124824	7125023
chr17	7131190	7131295
chr17	7131096	7131114
chr17	7134956	7135201
chr17	7136198	7137867
chr17	7137976	7138504
chr17	7138915	7139046
chr17	7140610	7141846
chr17	7142080	7142834
chr17	7144166	7144400
chr17	7144716	7144995
chr17	7145122	7147279
chr17	7151466	7151531
chr17	7152707	7153072
chr17	7154311	7156382
chr17	7157029	7157238
chr17	7158902	7158966
chr17	7160301	7160414
chr17	7160805	7160807
chr17	7161067	7161732
chr17	7162369	7162589
chr17	7162695	7163215
chr17	7164039	7167433

chr17	7167677	7168479
chr17	7173415	7173640
chr17	7174125	7174379
chr17	7175329	7175642
chr17	7175762	7175998
chr17	7183128	7183867
chr17	7184051	7184651
chr17	7197375	7198113
chr17	7198220	7199078
chr17	7199914	7200303
chr17	7207177	7207303
chr17	7209414	7209679
chr17	7209780	7211552
chr17	7211832	7212628
chr17	7208996	7209077
chr17	7214437	7214573
chr17	7218012	7219183
chr17	7219719	7219852
chr17	7231661	7232082
chr17	7232213	7232866
chr17	7233155	7233930
chr17	7248535	7248581
chr17	7248728	7248734
chr17	7253110	7253486
chr17	7253754	7256194
chr17	7257488	7257914
chr17	7258169	7258376
chr17	7259605	7259804
chr17	7264431	7264829
chr17	7265108	7265213
chr17	7269993	7270196
chr17	7283733	7284998
chr17	7285493	7285581
chr17	7296528	7298651
chr17	7303057	7304536
chr17	7305181	7305291
chr17	7305747	7305840
chr17	7306418	7306854
chr17	7307651	7309405
chr17	7338128	7338473
chr17	7338839	7339752
chr17	7339888	7344546
chr17	7346881	7348064
chr17	7348367	7349379
chr17	7349668	7349766
chr17	7352815	7353154

chr17	7353582	7355253
chr17	7355622	7355982
chr17	7356132	7356263
chr17	7357001	7357115
chr17	7361811	7361879
chr17	7362039	7362123
chr17	7362195	7362238
chr17	7364587	7364797
chr17	7365163	7365363
chr17	7367159	7367361
chr17	7367802	7368013
chr17	7368366	7368502
chr17	7373113	7373312
chr17	7377272	7377713
chr17	7378045	7378608
chr17	7378819	7380611
chr17	7380738	7381587
chr17	7381796	7384049
chr17	7386166	7388013
chr17	7388144	7388667
chr17	7391105	7391298
chr17	7391567	7391644
chr17	7410960	7411180
chr17	7413638	7413752
chr17	7418597	7418796
chr17	7424259	7424693
chr17	7451875	7452857
chr17	7460844	7461297
chr17	7461428	7463296
chr17	7464026	7466274
chr17	7469506	7469616
chr17	7471801	7471961
chr17	7472532	7472807
chr17	7475405	7475798
chr17	7475960	7478243
chr17	7478682	7479029
chr17	7479201	7479461
chr17	7482505	7482837
chr17	7482951	7484366
chr17	7484536	7485282
chr17	7488372	7489086
chr17	7485558	7488195
chr17	7489450	7489730
chr17	7489859	7490017
chr17	7490146	7491397
chr17	7491503	7492022

chr17	7492242	7493432
chr17	7493940	7494662
chr17	7516953	7518279
chr17	7518622	7518846
chr17	7530376	7531165
chr17	7531326	7531540
chr17	7531844	7532017
chr17	7535710	7535825
chr17	7553717	7553954
chr17	7583028	7583056
chr17	7588791	7589429
chr17	7589543	7590151
chr17	7590405	7591129
chr17	7591338	7591509
chr17	7591906	7592168
chr17	7592408	7592685
chr17	7608088	7608818
chr17	7619883	7620930
chr17	7646807	7646999
chr17	7647415	7647596
chr17	7649009	7649229
chr17	7649539	7649569
chr17	7653804	7653868
chr17	7654028	7654154
chr17	7658047	7658433
chr17	7658670	7659220
chr17	7707147	7707304
chr17	7707585	7707747
chr17	7712406	7712507
chr17	7713029	7713068
chr17	7713291	7713673
chr17	7735640	7735839
chr17	7737109	7741093
chr17	7741190	7741728
chr17	7742029	7742657
chr17	7744620	7745005
chr17	7745229	7748150
chr17	7748359	7748666
chr17	7759519	7760435
chr17	7760614	7762164
chr17	7762417	7762558
chr17	7763620	7763819
chr17	7767645	7768162
chr17	7770942	7771081
chr17	7771455	7771725
chr17	7788103	7788570

chr17	7788812	7790414
chr17	7790598	7792673
chr17	7794697	7794792
chr17	7796179	7796257
chr17	7816154	7816802
chr17	7817936	7817981
chr17	7818148	7819308
chr17	7819474	7820000
chr17	7820165	7820414
chr17	7820523	7820529
chr17	7821471	7821658
chr17	7823577	7824013
chr17	7825902	7825927
chr17	7826113	7826822
chr17	7833518	7833993
chr17	7834680	7835282
chr17	7835583	7836748
chr17	7892282	7892397
chr17	7893146	7893643
chr17	7893812	7894141
chr17	7895811	7896078
chr17	7908609	7908863
chr17	7919212	7921055
chr17	7921292	7921676
chr17	7922348	7922422
chr17	7922620	7923508
chr17	7924851	7925056
chr17	7927549	7928016
chr17	7928112	7928572
chr17	7928733	7928825
chr17	7942377	7942589
chr17	7942776	7943135
chr17	7943420	7943655
chr17	7943899	7945094
chr17	7961365	7961417
chr17	7976422	7977062
chr17	7977386	7977679
chr17	7982838	7983850
chr17	7983943	7984050
chr17	7985114	7985313
chr17	8006568	8007061
chr17	8008580	8008656
chr17	8009274	8009931
chr17	8010705	8010965
chr17	8013319	8013741
chr17	8020627	8020827

chr17	8021009	8022466
chr17	8022570	8022690
chr17	8022813	8023158
chr17	8023434	8023642
chr17	8023746	8028882
chr17	8029119	8029822
chr17	8029930	8030676
chr17	8030965	8031096
chr17	8030822	8030841
chr17	8035432	8035847
chr17	8037380	8037712
chr17	8040474	8040604
chr17	8041031	8042289
chr17	8042454	8042746
chr17	8043077	8043333
chr17	8051004	8051687
chr17	8052137	8053059
chr17	8054471	8054731
chr17	8055017	8055793
chr17	8056026	8056342
chr17	8056473	8056707
chr17	8056778	8057686
chr17	8057811	8059646
chr17	8060133	8060898
chr17	8060972	8061394
chr17	8061610	8062035
chr17	8064099	8064122
chr17	8065207	8067143
chr17	8067397	8067663
chr17	8067863	8068092
chr17	8069138	8069432
chr17	8070007	8070026
chr17	8076356	8076444
chr17	8077753	8077830
chr17	8076588	8077676
chr17	8078013	8078157
chr17	8078820	8079603
chr17	8079933	8080636
chr17	8085137	8085248
chr17	8089104	8090945
chr17	8091481	8091721
chr17	8092547	8093485
chr17	8094915	8095369
chr17	8095511	8096154
chr17	8096773	8097633
chr17	8098926	8099530

chr17	8099913	8100084
chr17	8103170	8104217
chr17	8105390	8105589
chr17	8112402	8112753
chr17	8113057	8114215
chr17	8118540	8118544
chr17	8123035	8123963
chr17	8124027	8124186
chr17	8124329	8125548
chr17	8125717	8127921
chr17	8128161	8128436
chr17	8129648	8130790
chr17	8150619	8152448
chr17	8152701	8153015
chr17	8154755	8155020
chr17	8155251	8155556
chr17	8191343	8192816
chr17	8197269	8197379
chr17	8198285	8199488
chr17	8230305	8230613
chr17	8244936	8244947
chr17	8269469	8269606
chr17	8270070	8270273
chr17	8279599	8280052
chr17	8280221	8280538
chr17	8282623	8282846
chr17	8284804	8285165
chr17	8285954	8286171
chr17	8286245	8286499
chr17	8286660	8287753
chr17	8287941	8288728
chr17	8291039	8291286
chr17	8294965	8295170
chr17	8296696	8297056
chr17	8311549	8312261
chr17	8313524	8313696
chr17	8314039	8314060
chr17	8316006	8316292
chr17	8316466	8316628
chr17	8321192	8321582
chr17	8338831	8338957
chr17	8339254	8340105
chr17	8446472	8446650
chr17	8446754	8446914
chr17	8456119	8456633
chr17	8484582	8484587

chr17	8484869	8484982
chr17	8532628	8534095
chr17	8534308	8535059
chr17	8629897	8631032
chr17	8648548	8648621
chr17	8648916	8649078
chr17	8649209	8649677
chr17	8710363	8710419
chr17	8761240	8761255
chr17	8819147	8819479
chr17	8825706	8825729
chr17	8826034	8826262
chr17	8924475	8925197
chr17	8925451	8925916
chr17	8953881	8953970
chr17	8954270	8954509
chr17	8954932	8955075
chr17	8959344	8959518
chr17	9143064	9143293
chr17	9143519	9143802
chr17	9193194	9193313
chr17	9253269	9253425
chr17	9253736	9254144
chr17	9292363	9292486
chr17	9303806	9303827
chr17	9304662	9304704
chr17	9415919	9416067
chr17	9479375	9479448
chr17	9480396	9480595
chr17	9548117	9549633
chr17	9549789	9550069
chr17	9551283	9551677
chr17	9552422	9552525
chr17	9571741	9571869
chr17	9572151	9572253
chr17	9573107	9573306
chr17	9580750	9580997
chr17	9588241	9589009
chr17	9633066	9633104
chr17	9633487	9633774
chr17	9705685	9706082
chr17	9739097	9739372
chr17	10525281	10525527
chr17	10525883	10526423
chr17	10600455	10601229
chr17	11847531	11847683

chr17	11847915	11847965
chr17	11848097	11848492
chr17	11900046	11900775
chr17	11900974	11901412
chr17	11923830	11923920
chr17	11924126	11924990
chr17	11927621	11927697
chr17	11927979	11927998
chr17	11993412	11993549
chr17	12692842	12693396
chr17	12920485	12921321
chr17	12921632	12922086
chr17	13049386	13049454
chr17	13210273	13210307
chr17	13249478	13249616
chr17	13250244	13250803
chr17	13251041	13251826
chr17	13296655	13296922
chr17	13299115	13299376
chr17	13314555	13314566
chr17	13324112	13324200
chr17	13324557	13324684
chr17	13498108	13498185
chr17	13498299	13498440
chr17	13501424	13501446
chr17	13503987	13504703
chr17	13505321	13505774
chr17	13631291	13631557
chr17	13647681	13647713
chr17	13678719	13678797
chr17	13679052	13679161
chr17	13679351	13679734
chr17	13679880	13680010
chr17	13972306	13972581
chr17	13972869	13973081
chr17	14200522	14200818
chr17	14202634	14203018
chr17	14203490	14203691
chr17	14204420	14206898
chr17	14207235	14207514
chr17	14210903	14211196
chr17	14212170	14212616
chr17	14213058	14213185
chr17	14641199	14641630
chr17	14641958	14642099
chr17	14644868	14645233

chr17	14648468	14648897
chr17	14718387	14718721
chr17	14736486	14736614
chr17	14736759	14736941
chr17	14748716	14748911
chr17	14749145	14749672
chr17	14814274	14814563
chr17	14829863	14829932
chr17	14845237	14845446
chr17	14941808	14942052
chr17	14942263	14942432
chr17	15163614	15164195
chr17	15366460	15366639
chr17	15456338	15456622
chr17	15465458	15465631
chr17	15466232	15466930
chr17	15467115	15467280
chr17	15541664	15541993
chr17	15545940	15546197
chr17	15546302	15546781
chr17	15548840	15550380
chr17	15551721	15552083
chr17	15553145	15553229
chr17	15548013	15548440
chr17	15553443	15553511
chr17	15554151	15555061
chr17	15555246	15555541
chr17	15555808	15556104
chr17	15556576	15556898
chr17	15558535	15558895
chr17	15559143	15559215
chr17	15561210	15561892
chr17	15562889	15563013
chr17	15563396	15563473
chr17	15586992	15587823
chr17	15602237	15602512
chr17	15602589	15602837
chr17	15603102	15603770
chr17	15689432	15689934
chr17	15823898	15824109
chr17	15824448	15824668
chr17	15842696	15842749
chr17	15847844	15852010
chr17	15852959	15853188
chr17	15857186	15857361
chr17	15857453	15857649

chr17	15857785	15858078
chr17	15859995	15860162
chr17	15862198	15863222
chr17	15866749	15866913
chr17	15867324	15867580
chr17	15885188	15885901
chr17	15900247	15900446
chr17	15901729	15901804
chr17	15901942	15902110
chr17	15902322	15903672
chr17	15913700	15913911
chr17	15917427	15917772
chr17	15931181	15931303
chr17	15931380	15931882
chr17	16025257	16025628
chr17	16026015	16026309
chr17	16026439	16026757
chr17	16053570	16053614
chr17	16053792	16053889
chr17	16093128	16093527
chr17	16094983	16095171
chr17	16109811	16109879
chr17	16110092	16110095
chr17	16111263	16111346
chr17	16118094	16119644
chr17	16120296	16120987
chr17	16132038	16132237
chr17	16133148	16133206
chr17	16177061	16177107
chr17	16188712	16188807
chr17	16188917	16188961
chr17	16189122	16190357
chr17	16198914	16199158
chr17	16199522	16199752
chr17	16222109	16222150
chr17	16255492	16255650
chr17	16255773	16256048
chr17	16256166	16257154
chr17	16275030	16275122
chr17	16275271	16275452
chr17	16277197	16277293
chr17	16281908	16281914
chr17	16282638	16282969
chr17	16283140	16283368
chr17	16283673	16284995
chr17	16285125	16285482

chr17	16287316	16287470
chr17	16289648	16290808
chr17	16310112	16310625
chr17	16311163	16311689
chr17	16317382	16317445
chr17	16322647	16322865
chr17	16336320	16336432
chr17	16340360	16340592
chr17	16341583	16341958
chr17	16342331	16343666
chr17	16344248	16344583
chr17	16346007	16346246
chr17	16362996	16363272
chr17	16389353	16389974
chr17	16390239	16390475
chr17	16394995	16395442
chr17	16395570	16395849
chr17	16424532	16424718
chr17	16428003	16428299
chr17	16438785	16438995
chr17	16439193	16439732
chr17	16440034	16440142
chr17	16472231	16472539
chr17	16487915	16488094
chr17	16492337	16492742
chr17	16492893	16492965
chr17	16556474	16557419
chr17	16570182	16570906
chr17	16589408	16589437
chr17	16676353	16676842
chr17	16742422	16742667
chr17	16763940	16764229
chr17	16764437	16764712
chr17	16765299	16765411
chr17	16767017	16767094
chr17	16767640	16768219
chr17	16768719	16769308
chr17	16912623	16912982
chr17	16913213	16913220
chr17	16913420	16915270
chr17	16915922	16916325
chr17	16917012	16917331
chr17	16917900	16918681
chr17	16918794	16918978
chr17	16919185	16919325
chr17	16921563	16922017

chr17	16922229	16922428
chr17	16922648	16923492
chr17	16923708	16923862
chr17	16924280	16924298
chr17	16929423	16929779
chr17	16930991	16931556
chr17	16931716	16932135
chr17	16932587	16932945
chr17	16935391	16935836
chr17	16940523	16941083
chr17	16942276	16942680
chr17	16946207	16946592
chr17	16946708	16947053
chr17	16948098	16948492
chr17	16965750	16965970
chr17	16970870	16970967
chr17	16975780	16976078
chr17	16978757	16978911
chr17	17011507	17011711
chr17	17045694	17045931
chr17	17053666	17053774
chr17	17054283	17054474
chr17	17058666	17058821
chr17	17060870	17061074
chr17	17073566	17073730
chr17	17074119	17074387
chr17	17075709	17075909
chr17	17088837	17088881
chr17	17089883	17090017
chr17	17090091	17090309
chr17	17108567	17108706
chr17	17108813	17110622
chr17	17138179	17138305
chr17	17139729	17142030
chr17	17166228	17166447
chr17	17183284	17183951
chr17	17184121	17184449
chr17	17184835	17184859
chr17	17188780	17188852
chr17	17199514	17199866
chr17	17203920	17204056
chr17	17205891	17206478
chr17	17206641	17207354
chr17	17216485	17216566
chr17	17291074	17291624
chr17	17315590	17315798

chr17	17319404	17319509
chr17	17319709	17320032
chr17	17380077	17380179
chr17	17380330	17380800
chr17	17384086	17384259
chr17	17391031	17391092
chr17	17399119	17399642
chr17	17399847	17400194
chr17	17494281	17494963
chr17	17495236	17496308
chr17	17498233	17498404
chr17	17498546	17498835
chr17	17526452	17526898
chr17	17531938	17531941
chr17	17532054	17532228
chr17	17532293	17532608
chr17	17536501	17537032
chr17	17545021	17545171
chr17	17567008	17567175
chr17	17569316	17569353
chr17	17578869	17579890
chr17	17580797	17580840
chr17	17584260	17584459
chr17	17584631	17586871
chr17	17587265	17587636
chr17	17588352	17590638
chr17	17591056	17591291
chr17	17593345	17593672
chr17	17594112	17594554
chr17	17594877	17595195
chr17	17595347	17595467
chr17	17595802	17595808
chr17	17599745	17599819
chr17	17596293	17599319
chr17	17600106	17600305
chr17	17613855	17614257
chr17	17614426	17615016
chr17	17615674	17615922
chr17	17616609	17616804
chr17	17629742	17629937
chr17	17630029	17630310
chr17	17630743	17631008
chr17	17632246	17632718
chr17	17642693	17643002
chr17	17653647	17653874
chr17	17654129	17654232

chr17	17654421	17654620
chr17	17655194	17655400
chr17	17655774	17656739
chr17	17657741	17657863
chr17	17667895	17668080
chr17	17677470	17677929
chr17	17679646	17679867
chr17	17682918	17683135
chr17	17683603	17683647
chr17	17683757	17683916
chr17	17684433	17687355
chr17	17706069	17706312
chr17	17722344	17723190
chr17	17723320	17723947
chr17	17724195	17724764
chr17	17724874	17725332
chr17	17725524	17726146
chr17	17726214	17726876
chr17	17727154	17730051
chr17	17730223	17730620
chr17	17731908	17732186
chr17	17737749	17737775
chr17	17737880	17738112
chr17	17738356	17738781
chr17	17738895	17740634
chr17	17740785	17743058
chr17	17743191	17745439
chr17	17746203	17746559
chr17	17745670	17746140
chr17	17758941	17759019
chr17	17759570	17759815
chr17	17762975	17763497
chr17	17777645	17777808
chr17	17778091	17778218
chr17	17803671	17804132
chr17	17804548	17804722
chr17	17817161	17817168
chr17	17817642	17817729
chr17	17817811	17818038
chr17	17822503	17822742
chr17	17824536	17824764
chr17	17825459	17825719
chr17	17826365	17826561
chr17	17839242	17839491
chr17	17841690	17841839
chr17	17842067	17842473

chr17	17847121	17847351
chr17	17851716	17851877
chr17	17854580	17854630
chr17	17854843	17854921
chr17	17855677	17855752
chr17	17861113	17861730
chr17	17861957	17862145
chr17	17874537	17874921
chr17	17875251	17875731
chr17	17876394	17876482
chr17	17893898	17894416
chr17	17894697	17894864
chr17	17936748	17936891
chr17	17937149	17937289
chr17	17939482	17939705
chr17	17941199	17941398
chr17	17941897	17944241
chr17	17974288	17974427
chr17	17990744	17991801
chr17	18046199	18046406
chr17	18056334	18057311
chr17	18061035	18061581
chr17	18061695	18062019
chr17	18085399	18086591
chr17	18086704	18088417
chr17	18088930	18089149
chr17	18089663	18089668
chr17	18090245	18090769
chr17	18123789	18124390
chr17	18128474	18128678
chr17	18128955	18130006
chr17	18130667	18130932
chr17	18138512	18139688
chr17	18139813	18140046
chr17	18146521	18146729
chr17	18153303	18153464
chr17	18160144	18160581
chr17	18160717	18162602
chr17	18162710	18165333
chr17	18169642	18169670
chr17	18171970	18172147
chr17	18172300	18172517
chr17	18217358	18219301
chr17	18265730	18266829
chr17	18267028	18267222
chr17	18272104	18272293

chr17	18274651	18274850
chr17	18276577	18276942
chr17	18280317	18280729
chr17	18280975	18281410
chr17	18281573	18282095
chr17	18282329	18282645
chr17	18313009	18313100
chr17	18313933	18315936
chr17	18343303	18343574
chr17	18350183	18350497
chr17	18351048	18352380
chr17	18353033	18353447
chr17	18354016	18354215
chr17	18355145	18355273
chr17	18362221	18362285
chr17	18362520	18362764
chr17	18370894	18371159
chr17	18371727	18371843
chr17	18372035	18372248
chr17	18378304	18378467
chr17	18380132	18380499
chr17	18380685	18380962
chr17	18383381	18383580
chr17	18445485	18445550
chr17	18445901	18445952
chr17	18467332	18467647
chr17	18529271	18529381
chr17	18538107	18538309
chr17	18584783	18585606
chr17	18585935	18585983
chr17	18601110	18601921
chr17	18615931	18615984
chr17	18620744	18621216
chr17	18621465	18623285
chr17	18625084	18625297
chr17	18625465	18625714
chr17	18626244	18626469
chr17	18627160	18627396
chr17	18629670	18629944
chr17	18684152	18684343
chr17	18684582	18685009
chr17	18685617	18685877
chr17	18733251	18733450
chr17	18752851	18753913
chr17	18754707	18754895
chr17	18759043	18759439

chr17	18761019	18761182
chr17	18759579	18759868
chr17	18761426	18762063
chr17	18828138	18828174
chr17	18870607	18871973
chr17	18872144	18873131
chr17	18875959	18876230
chr17	18876502	18877853
chr17	18878890	18879089
chr17	18885238	18885675
chr17	18885921	18886125
chr17	18901453	18901738
chr17	18902387	18903391
chr17	18903499	18904668
chr17	18904817	18906274
chr17	18906477	18908108
chr17	18908218	18908530
chr17	18914477	18915091
chr17	18917162	18917510
chr17	18919134	18919665
chr17	18928322	18928795
chr17	18933299	18933693
chr17	18934271	18934428
chr17	18934647	18934653
chr17	18935311	18936506
chr17	18937071	18937295
chr17	18940937	18941272
chr17	18941628	18942127
chr17	18954584	18954596
chr17	18955646	18955769
chr17	18958785	18958906
chr17	18961901	18961993
chr17	18962987	18963331
chr17	18963835	18965239
chr17	18965404	18967075
chr17	18967431	18969470
chr17	18970633	18971232
chr17	18972287	18972663
chr17	18973081	18973213
chr17	18973477	18974514
chr17	18976353	18976552
chr17	18976822	18977016
chr17	18977837	18977938
chr17	18978779	18978981
chr17	18979587	18980754
chr17	18982709	18983145

chr17	18984398	18984594
chr17	18984799	18985399
chr17	18985962	18986208
chr17	18989903	18990145
chr17	18991285	18992551
chr17	18992717	18993269
chr17	18993485	18993802
chr17	18994045	18994244
chr17	18995104	18995794
chr17	18996015	18996507
chr17	18996707	18997315
chr17	18999981	19000191
chr17	19000326	19000419
chr17	19001985	19002626
chr17	19003772	19003934
chr17	19004218	19004430
chr17	19004620	19004935
chr17	19006926	19007345
chr17	19007769	19007990
chr17	19008327	19009492
chr17	19009622	19010587
chr17	19010678	19010815
chr17	19011246	19011924
chr17	19012153	19012350
chr17	19012707	19012969
chr17	19013164	19013292
chr17	19013355	19015770
chr17	19015923	19016617
chr17	19016743	19017368
chr17	19021984	19022185
chr17	19039067	19039609
chr17	19039882	19040478
chr17	19040829	19041108
chr17	19053305	19053451
chr17	19053743	19054043
chr17	19054277	19054483
chr17	19054701	19054992
chr17	19055739	19056812
chr17	19057697	19057906
chr17	19061994	19062673
chr17	19062925	19063353
chr17	19064483	19064701
chr17	19065046	19065995
chr17	19066130	19067524
chr17	19072111	19072210
chr17	19073309	19073956

chr17	19077526	19077725
chr17	19078135	19078334
chr17	19078644	19078843
chr17	19079329	19079402
chr17	19079522	19079695
chr17	19083219	19083340
chr17	19084341	19085009
chr17	19085116	19085400
chr17	19086091	19086492
chr17	19087167	19087569
chr17	19088444	19088923
chr17	19089919	19093160
chr17	19093521	19095056
chr17	19095475	19095731
chr17	19096134	19096560
chr17	19096791	19096889
chr17	19097132	19097775
chr17	19098418	19098782
chr17	19099089	19099391
chr17	19099461	19099833
chr17	19100089	19100705
chr17	19101093	19101407
chr17	19105157	19106264
chr17	19106397	19106899
chr17	19108977	19109264
chr17	19111021	19111586
chr17	19113034	19113497
chr17	19114051	19114259
chr17	19114328	19114376
chr17	19117628	19118722
chr17	19118914	19119872
chr17	19121347	19121964
chr17	19122221	19122870
chr17	19124319	19124765
chr17	19127526	19127769
chr17	19128193	19128817
chr17	19128925	19129123
chr17	19130220	19130506
chr17	19130849	19131087
chr17	19133087	19133316
chr17	19133600	19134079
chr17	19134234	19136608
chr17	19137638	19138970
chr17	19139112	19141970
chr17	19145629	19146014
chr17	19149270	19149309

chr17	19162901	19163060
chr17	19197245	19197358
chr17	19229935	19230189
chr17	19230509	19231103
chr17	19243809	19243974
chr17	19249199	19249398
chr17	19250542	19251334
chr17	19252058	19252148
chr17	19251914	19251927
chr17	19256330	19256343
chr17	19257385	19257549
chr17	19264982	19264994
chr17	19265315	19266817
chr17	19280776	19281327
chr17	19281706	19282577
chr17	19289211	19290501
chr17	19290644	19291004
chr17	19302089	19302144
chr17	19350905	19351010
chr17	19351471	19351757
chr17	19351903	19352123
chr17	19372644	19372727
chr17	19409521	19409728
chr17	19409977	19410045
chr17	19410535	19411285
chr17	19411538	19412094
chr17	19413003	19413154
chr17	19417171	19417542
chr17	19436976	19437923
chr17	19482745	19482964
chr17	19483049	19483074
chr17	19483223	19483559
chr17	19511048	19511320
chr17	19549554	19549777
chr17	19550249	19550700
chr17	19550937	19552091
chr17	19552195	19552661
chr17	19553525	19553921
chr17	19608572	19608582
chr17	19608939	19608989
chr17	19609260	19609296
chr17	19609651	19610556
chr17	19610871	19611530
chr17	19611612	19611730
chr17	19612697	19612882
chr17	19614041	19614292

chr17	19620023	19621195
chr17	19621357	19622739
chr17	19622844	19624882
chr17	19626160	19626410
chr17	19626520	19626747
chr17	19630520	19630651
chr17	19627207	19629072
chr17	19630807	19632402
chr17	19632498	19632861
chr17	19633417	19633891
chr17	19639505	19639546
chr17	19639862	19640019
chr17	19647404	19647519
chr17	19647789	19648267
chr17	19648430	19648629
chr17	19649198	19649432
chr17	19649579	19649734
chr17	19650575	19650702
chr17	19652031	19652415
chr17	19653970	19654791
chr17	19655090	19657536
chr17	19659196	19659251
chr17	19769782	19771510
chr17	19800403	19800586
chr17	19830170	19830351
chr17	19841531	19841692
chr17	19861297	19861381
chr17	19880557	19881844
chr17	19890827	19890843
chr17	19890090	19890641
chr17	19894927	19895069
chr17	19896159	19896366
chr17	19912713	19913202
chr17	19913763	19913823
chr17	19962939	19963207
chr17	19966998	19967339
chr17	19969851	19970051
chr17	20058845	20060278
chr17	20110108	20110308
chr17	20127055	20127060
chr17	20127358	20127564
chr17	20167139	20167279
chr17	20223644	20224095
chr17	20233962	20234111
chr17	20304887	20304950
chr17	20305322	20305391

chr17	20377039	20377062
chr17	20379115	20379175
chr17	20379796	20379800
chr17	20388329	20388396
chr17	20388705	20388795
chr17	20396866	20397943
chr17	20399146	20399428
chr17	20407668	20407750
chr17	20434198	20434528
chr17	20434858	20435904
chr17	20436493	20436508
chr17	20610341	20610644
chr17	20621857	20621994
chr17	20704939	20705491
chr17	20811602	20811920
chr17	20812038	20812242
chr17	20864791	20864964
chr17	20865176	20865571
chr17	20932357	20932464
chr17	20943467	20943854
chr17	20944105	20944877
chr17	20944982	20946360
chr17	20946789	20947253
chr17	20998378	20998554
chr17	21002123	21002663
chr17	21002973	21003389
chr17	21029918	21031004
chr17	21116756	21118435
chr17	21118557	21118857
chr17	21119108	21119453
chr17	21155982	21156500
chr17	21156856	21157511
chr17	21160520	21160762
chr17	21166945	21167344
chr17	21173708	21173740
chr17	21174075	21174079
chr17	21176067	21176266
chr17	21178820	21179239
chr17	21179376	21179655
chr17	21180202	21180284
chr17	21181474	21181515
chr17	21187295	21189829
chr17	21189948	21191226
chr17	21191623	21192304
chr17	21192857	21193065
chr17	21194042	21194448

chr17	21194685	21194690
chr17	21208809	21209008
chr17	21209943	21210142
chr17	21216023	21216266
chr17	21220250	21221019
chr17	21226000	21226423
chr17	21226861	21227145
chr17	21256889	21257162
chr17	21271942	21272141
chr17	21279816	21281586
chr17	21286024	21286378
chr17	21305879	21306245
chr17	21313941	21314169
chr17	21314732	21314971
chr17	21330979	21331157
chr17	21331265	21331444
chr17	21333622	21334098
chr17	21354682	21355106
chr17	21355866	21356096
chr17	21356247	21356492
chr17	21414788	21414944
chr17	21415264	21415383
chr17	21454571	21454849
chr17	21906114	21906313
chr17	21906628	21906883
chr17	22244585	22252450
chr17	22252575	22262277
chr17	22262522	22262867
chr17	25263690	25264165
chr17	25264672	25265636
chr17	25266163	25266498
chr17	25266618	25267033
chr17	25267252	25268026
chr17	25553622	25553759
chr17	25568386	25569109
chr17	25569480	25570096
chr17	25570205	25570370
chr17	25571153	25571331
chr17	25571566	25572419
chr17	25572755	25572914
chr17	25574396	25574438
chr17	25620388	25620637
chr17	25620791	25622035
chr17	25622149	25622329
chr17	25623527	25623780
chr17	25624386	25624388

chr17	25646757	25646897
chr17	25650155	25650695
chr17	25658498	25659540
chr17	25659788	25660968
chr17	25675955	25676262
chr17	25676382	25676700
chr17	25680549	25681496
chr17	25783060	25784038
chr17	25784134	25784207
chr17	25784456	25784585
chr17	25798357	25798534
chr17	25798957	25799305
chr17	25799470	25799636
chr17	25815978	25816013
chr17	25816574	25816649
chr17	25821190	25821729
chr17	25821946	25822286
chr17	25824924	25825161
chr17	25834749	25834877
chr17	25856291	25856781
chr17	25856856	25856910
chr17	25867005	25867449
chr17	25879712	25879875
chr17	25880234	25880574
chr17	25886870	25887081
chr17	25887390	25887648
chr17	25907884	25908083
chr17	25912213	25912317
chr17	25926989	25927234
chr17	26120638	26120977
chr17	26132724	26133119
chr17	26219984	26220373
chr17	26220533	26220702
chr17	26307112	26307164
chr17	26307492	26307523
chr17	26316458	26316469
chr17	26361081	26361749
chr17	26367911	26368535
chr17	26368927	26369334
chr17	26384936	26385057
chr17	26547014	26547226
chr17	26554434	26556045
chr17	26560733	26561034
chr17	26578871	26579579
chr17	26605914	26606084
chr17	26606220	26606258

chr17	26609502	26610046
chr17	26645236	26645766
chr17	26646209	26646624
chr17	26661913	26662490
chr17	26662798	26663999
chr17	26665073	26665395
chr17	26665943	26666025
chr17	26673395	26673560
chr17	26673998	26674124
chr17	26674566	26674790
chr17	26683859	26684956
chr17	26699157	26699449
chr17	26707067	26707235
chr17	26707457	26707654
chr17	26708257	26708614
chr17	26710973	26711603
chr17	26711804	26712003
chr17	26732665	26733861
chr17	26734524	26734779
chr17	26734967	26735462
chr17	26743769	26743952
chr17	26744097	26744393
chr17	26763608	26764187
chr17	26773679	26774165
chr17	26817389	26817588
chr17	26819053	26819753
chr17	26832894	26833093
chr17	26833411	26833747
chr17	26834602	26834982
chr17	26835360	26835671
chr17	26852049	26852683
chr17	26854325	26854723
chr17	26879059	26880073
chr17	26897241	26897461
chr17	26897970	26898517
chr17	26898675	26898874
chr17	26899274	26899328
chr17	26903403	26903954
chr17	26904092	26904602
chr17	26925633	26926055
chr17	26926372	26926613
chr17	26926809	26927246
chr17	26971445	26971822
chr17	26971946	26972820
chr17	26984773	26984998
chr17	26988616	26989730

chr17	26999593	26999607
chr17	27037447	27037673
chr17	27037794	27039101
chr17	27043456	27043626
chr17	27043848	27046000
chr17	27046117	27048278
chr17	27048812	27049452
chr17	27049976	27050110
chr17	27050416	27050610
chr17	27051203	27053663
chr17	27053807	27054685
chr17	27054774	27056383
chr17	27056458	27056839
chr17	27069626	27073775
chr17	27073911	27074121
chr17	27074356	27074832
chr17	27075292	27075446
chr17	27079940	27080055
chr17	27081841	27082086
chr17	27088261	27088334
chr17	27104075	27104567
chr17	27104731	27105055
chr17	27134949	27135359
chr17	27135889	27136517
chr17	27138177	27138394
chr17	27138695	27138978
chr17	27139088	27139421
chr17	27139651	27140099
chr17	27140615	27140768
chr17	27153392	27153617
chr17	27169560	27170117
chr17	27170374	27170673
chr17	27173275	27173288
chr17	27181311	27182460
chr17	27193187	27193699
chr17	27205127	27205525
chr17	27205670	27206118
chr17	27206532	27206731
chr17	27207524	27207826
chr17	27219410	27219416
chr17	27219622	27220019
chr17	27220224	27220523
chr17	27223778	27223947
chr17	27224111	27225522
chr17	27229010	27230185
chr17	27232078	27232229

chr17	27235305	27235446
chr17	27273728	27273963
chr17	27275876	27276059
chr17	27276190	27277203
chr17	27277388	27277690
chr17	27277845	27278628
chr17	27278841	27279359
chr17	27279714	27279841
chr17	27428920	27428995
chr17	27429106	27429458
chr17	27429666	27430020
chr17	27430213	27430580
chr17	27434594	27434741
chr17	27434806	27435006
chr17	27461113	27461312
chr17	27472987	27473139
chr17	27473956	27474168
chr17	27474257	27474646
chr17	27475399	27475886
chr17	27477162	27477716
chr17	27476259	27476872
chr17	27481623	27481921
chr17	27482115	27483100
chr17	27483286	27483519
chr17	27483729	27483874
chr17	27484099	27484435
chr17	27488396	27488408
chr17	27494538	27494695
chr17	27502883	27503180
chr17	27503387	27504320
chr17	27504604	27505733
chr17	27505879	27508105
chr17	27542648	27542652
chr17	27542893	27543393
chr17	27544055	27544347
chr17	27546370	27546588
chr17	27565115	27565548
chr17	27567887	27568053
chr17	27568473	27568498
chr17	27618299	27618519
chr17	27619579	27621134
chr17	27621387	27621570
chr17	27690751	27690915
chr17	27712178	27712315
chr17	27716820	27717808
chr17	27718028	27718740

chr17	27740247	27740311
chr17	27822133	27822358
chr17	27841755	27841791
chr17	27853326	27853601
chr17	27892548	27893379
chr17	27893625	27894025
chr17	27894280	27895015
chr17	27895273	27895539
chr17	27895906	27896183
chr17	27906292	27906320
chr17	27907640	27908169
chr17	27908807	27909006
chr17	27909127	27909326
chr17	27910209	27910770
chr17	27911370	27911627
chr17	27911774	27912145
chr17	27912871	27913124
chr17	27915220	27915745
chr17	27915875	27916349
chr17	27916655	27917536
chr17	27918024	27918799
chr17	27919133	27919472
chr17	27940138	27941152
chr17	27942808	27943132
chr17	27944306	27945569
chr17	27949317	27949904
chr17	27950132	27950350
chr17	27950712	27950944
chr17	27966196	27966347
chr17	27966776	27966955
chr17	27967542	27967884
chr17	28019193	28019227
chr17	28019448	28019501
chr17	28038182	28038297
chr17	28059735	28059761
chr17	28060068	28060174
chr17	28071815	28072279
chr17	28076724	28076786
chr17	28087894	28088833
chr17	28113437	28113584
chr17	28135108	28135164
chr17	28246377	28246755
chr17	28255986	28256344
chr17	28256427	28257684
chr17	28258294	28258536
chr17	28367687	28367808

chr17	28443560	28444376
chr17	28444537	28444672
chr17	28497874	28497957
chr17	28556391	28556462
chr17	28618074	28619411
chr17	28623878	28624006
chr17	28667072	28667221
chr17	28672253	28672291
chr17	28690520	28690782
chr17	28694949	28695148
chr17	28705602	28705760
chr17	28705989	28706886
chr17	28799641	28799802
chr17	28803893	28804261
chr17	28804373	28804994
chr17	28927028	28927278
chr17	28927385	28928084
chr17	28928388	28928600
chr17	28965968	28966359
chr17	28978016	28978323
chr17	28991858	28992011
chr17	28993768	28993990
chr17	28994145	28995225
chr17	28995357	28995607
chr17	28995765	28995961
chr17	29020447	29020607
chr17	29024432	29024960
chr17	29025235	29025691
chr17	29035563	29037551
chr17	29057848	29058140
chr17	29058205	29059544
chr17	29126646	29126869
chr17	29142028	29142337
chr17	29150974	29151288
chr17	29151381	29151686
chr17	29151890	29152161
chr17	29152843	29153234
chr17	29153838	29154037
chr17	29158670	29158889
chr17	29159036	29159623
chr17	29185632	29186022
chr17	29233420	29234349
chr17	29234584	29234674
chr17	29248881	29249296
chr17	29297266	29298616
chr17	29298734	29299646

chr17	29307877	29308455
chr17	29308545	29308548
chr17	29322414	29322718
chr17	29335354	29335931
chr17	29379026	29379266
chr17	29379475	29379819
chr17	29394610	29395060
chr17	29399220	29399953
chr17	29421939	29423038
chr17	29463412	29463754
chr17	29465624	29465732
chr17	29601267	29601354
chr17	29601490	29601620
chr17	29641386	29641387
chr17	29654828	29655169
chr17	29815026	29815709
chr17	29821593	29821718
chr17	29861369	29861598
chr17	29875766	29875814
chr17	29876268	29877597
chr17	29885869	29887618
chr17	29887814	29887986
chr17	29888092	29888291
chr17	29888628	29888794
chr17	29891955	29892353
chr17	29906372	29906764
chr17	29922097	29922123
chr17	29922387	29922463
chr17	29922768	29922792
chr17	29922971	29923328
chr17	29923521	29923768
chr17	29924029	29924312
chr17	30001274	30001397
chr17	30001686	30001788
chr17	30053565	30053773
chr17	30115015	30115644
chr17	30115870	30115978
chr17	30117457	30117478
chr17	30153209	30153741
chr17	30182900	30183110
chr17	30185260	30186288
chr17	30196210	30196419
chr17	30211194	30211357
chr17	30227988	30228698
chr17	30228965	30229192
chr17	30263361	30263776

chr17	30263931	30264799
chr17	30283727	30284483
chr17	30294654	30294714
chr17	30294975	30295179
chr17	30301006	30301704
chr17	30333573	30333962
chr17	30334094	30335197
chr17	30410482	30411690
chr17	30442560	30442569
chr17	30454273	30455012
chr17	30455421	30455865
chr17	30465859	30465991
chr17	30466231	30466313
chr17	30468336	30468619
chr17	30468931	30469330
chr17	30469483	30470227
chr17	30470385	30470400
chr17	30663948	30664006
chr17	30668173	30668441
chr17	30668577	30669209
chr17	30669407	30669462
chr17	30676936	30677108
chr17	30677288	30678019
chr17	30683819	30684117
chr17	30684509	30684993
chr17	30771240	30772523
chr17	30810871	30811128
chr17	30813309	30813845
chr17	30814177	30814926
chr17	30823686	30824612
chr17	30825976	30826229
chr17	30846343	30846937
chr17	30878215	30878281
chr17	30878459	30878621
chr17	30896287	30896570
chr17	30925142	30925454
chr17	30948914	30949196
chr17	31011293	31011387
chr17	31074104	31074106
chr17	31093542	31093744
chr17	31096891	31096960
chr17	31104745	31104862
chr17	31140336	31140478
chr17	31149617	31150048
chr17	31150166	31150355
chr17	31155296	31155666

chr17	31203509	31204329
chr17	31254888	31255399
chr17	31264198	31264398
chr17	31339511	31339759
chr17	31611014	31611175
chr17	33288066	33288694
chr17	33307239	33308446
chr17	33350778	33350892
chr17	33390488	33390646
chr17	33390915	33391200
chr17	33412010	33412027
chr17	33414789	33415559
chr17	33415921	33416971
chr17	33421884	33421897
chr17	33422181	33422356
chr17	33445690	33447226
chr17	33468896	33468907
chr17	33469061	33469644
chr17	33545343	33545522
chr17	33566137	33566175
chr17	33569077	33569369
chr17	33571424	33572533
chr17	33569578	33571054
chr17	33581119	33581298
chr17	33581675	33581819
chr17	33639968	33640171
chr17	33642205	33642432
chr17	33673073	33673518
chr17	33673742	33673859
chr17	33699923	33700232
chr17	33700806	33701347
chr17	33759186	33761113
chr17	33775184	33775706
chr17	33775965	33776182
chr17	33787381	33787629
chr17	33787940	33787971
chr17	33814191	33814617
chr17	33814712	33814767
chr17	33814946	33815370
chr17	33866403	33866486
chr17	33867949	33868295
chr17	33894443	33895348
chr17	33895587	33895909
chr17	33905293	33905565
chr17	33905704	33906113
chr17	33915492	33915688

chr17	33914106	33915075
chr17	33915817	33915955
chr17	33916503	33916921
chr17	34058658	34058996
chr17	34068234	34068448
chr17	34078436	34078494
chr17	34078581	34078697
chr17	34091052	34091251
chr17	34091468	34091718
chr17	34103828	34104392
chr17	34104763	34105297
chr17	34105441	34105474
chr17	34105902	34106092
chr17	34109041	34109240
chr17	34110466	34110938
chr17	34111521	34111928
chr17	34112189	34112700
chr17	34115382	34115553
chr17	34115952	34116295
chr17	34117801	34118278
chr17	34120928	34121202
chr17	34121596	34122667
chr17	34122906	34123067
chr17	34127187	34127360
chr17	34135625	34135883
chr17	34136004	34136338
chr17	34136527	34137094
chr17	34164571	34164583
chr17	34193404	34193410
chr17	34216664	34216920
chr17	34257447	34257698
chr17	34301375	34301775
chr17	34493640	34493839
chr17	34612108	34612285
chr17	34780382	34780499
chr17	34838596	34838957
chr17	34839154	34839412
chr17	34841754	34842970
chr17	34889789	34890086
chr17	34890466	34890698
chr17	34890925	34891785
chr17	34895484	34895724
chr17	34899793	34901224
chr17	34901384	34901973
chr17	34904393	34904774
chr17	34948001	34949032

chr17	34949178	34949552
chr17	34957170	34957763
chr17	34958086	34958682
chr17	34958822	34959046
chr17	34965478	34965723
chr17	35206098	35206652
chr17	35207019	35207186
chr17	35207358	35208573
chr17	35213274	35213928
chr17	35215583	35216032
chr17	35277560	35277808
chr17	35278052	35278174
chr17	35278368	35279364
chr17	35279707	35279753
chr17	35305823	35307035
chr17	35448934	35449150
chr17	35515314	35515636
chr17	35536880	35537010
chr17	35633375	35633426
chr17	35662440	35662744
chr17	35713246	35713636
chr17	35715458	35716905
chr17	35717650	35718000
chr17	35718459	35718926
chr17	35725488	35725786
chr17	35730492	35730592
chr17	35749052	35749382
chr17	35766501	35766743
chr17	35767011	35767822
chr17	35783080	35783365
chr17	35790104	35790303
chr17	35806248	35806302
chr17	35806467	35806557
chr17	35807851	35808173
chr17	35810094	35810266
chr17	35810524	35810632
chr17	35811791	35811990
chr17	35836486	35837220
chr17	35838155	35838455
chr17	35838598	35838699
chr17	35841032	35841570
chr17	35843437	35843966
chr17	35848854	35849900
chr17	35848096	35848714
chr17	35850116	35851751
chr17	35853269	35853407

chr17	35853524	35853574
chr17	35855744	35855748
chr17	35911884	35911951
chr17	35912107	35912274
chr17	35949989	35950206
chr17	35968849	35969082
chr17	35969255	35969485
chr17	35969663	35970074
chr17	35974408	35974413
chr17	35989229	35989645
chr17	36003190	36003470
chr17	36003615	36003846
chr17	36031685	36032533
chr17	36126893	36126941
chr17	36173674	36173713
chr17	36176163	36176264
chr17	36180683	36180721
chr17	36184066	36184136
chr17	36184238	36184534
chr17	36185011	36185117
chr17	36329829	36329833
chr17	36348784	36348995
chr17	36350900	36351300
chr17	36353048	36353343
chr17	36354237	36354341
chr17	36355109	36355535
chr17	36356642	36356813
chr17	36357602	36357751
chr17	36359477	36359692
chr17	36360609	36361158
chr17	36362568	36362773
chr17	36366423	36366630
chr17	36370808	36371105
chr17	36371557	36371854
chr17	36372326	36372523
chr17	36374660	36374833
chr17	36375846	36376058
chr17	36376994	36377130
chr17	36377487	36378998
chr17	36379164	36379410
chr17	36380390	36380487
chr17	36380768	36381055
chr17	36381365	36381514
chr17	36381930	36381957
chr17	36382641	36383413
chr17	36383749	36384001

chr17	36384448	36384991
chr17	36385311	36385572
chr17	36385793	36386267
chr17	36386346	36386716
chr17	36387035	36387489
chr17	36388806	36389438
chr17	36391674	36391941
chr17	36405180	36406126
chr17	36411826	36413329
chr17	36432723	36432901
chr17	36442376	36442483
chr17	36442605	36443018
chr17	36451499	36451746
chr17	36452439	36452903
chr17	36453142	36453406
chr17	36453658	36453980
chr17	36476298	36476306
chr17	36507234	36507559
chr17	36507722	36508870
chr17	36570883	36571487
chr17	36583500	36583842
chr17	36584332	36585912
chr17	36596168	36596367
chr17	36600780	36601035
chr17	36603065	36603385
chr17	36603646	36606763
chr17	36607006	36608781
chr17	36608954	36610020
chr17	36610280	36612804
chr17	36615881	36616308
chr17	36618201	36618775
chr17	36619404	36619611
chr17	36620158	36621037
chr17	36621167	36622720
chr17	36626392	36627037
chr17	36627281	36627421
chr17	36628611	36628829
chr17	36628982	36629332
chr17	36713829	36714386
chr17	36714602	36715079
chr17	36715211	36715806
chr17	36758032	36758362
chr17	36762279	36762750
chr17	36817191	36817316
chr17	36829307	36829474
chr17	36829876	36831277

chr17	36835906	36836054
chr17	36843683	36843957
chr17	36844207	36844620
chr17	36844757	36844899
chr17	36855847	36856899
chr17	36858281	36858555
chr17	36858749	36859350
chr17	36859550	36859851
chr17	36859969	36861397
chr17	36861573	36862672
chr17	36886005	36887189
chr17	36891370	36891629
chr17	36903162	36904665
chr17	36904779	36904995
chr17	36905113	36906117
chr17	36906402	36906893
chr17	36907195	36907878
chr17	36908148	36908694
chr17	36908862	36909746
chr17	36913802	36913829
chr17	36951685	36951981
chr17	36954936	36956615
chr17	36980976	36981532
chr17	36981727	36982088
chr17	36990188	36990356
chr17	36992099	36992472
chr17	36996688	36996876
chr17	36997064	36997853
chr17	37000191	37001008
chr17	37009502	37010018
chr17	37010153	37010237
chr17	37013829	37013853
chr17	37019713	37020189
chr17	37020858	37021229
chr17	37021374	37021625
chr17	37024218	37025588
chr17	37025728	37026896
chr17	37027119	37027862
chr17	37027973	37028229
chr17	37028484	37028966
chr17	37030726	37030932
chr17	37031315	37031574
chr17	37035171	37035647
chr17	37036399	37037412
chr17	37037602	37037765
chr17	37038481	37038532

chr17	37052002	37052031
chr17	37053895	37054305
chr17	37055201	37055643
chr17	37056212	37056319
chr17	37056505	37057235
chr17	37062417	37062760
chr17	37064199	37064438
chr17	37064848	37065186
chr17	37065387	37065835
chr17	37155683	37155817
chr17	37212949	37213131
chr17	37213364	37213619
chr17	37307591	37307828
chr17	37308735	37308997
chr17	37309349	37309965
chr17	37310222	37310723
chr17	37310900	37310975
chr17	37314602	37314856
chr17	37319921	37319999
chr17	37320768	37321004
chr17	37334722	37335013
chr17	37352482	37354145
chr17	37355857	37357099
chr17	37365749	37366599
chr17	37368831	37369255
chr17	37369440	37369895
chr17	37370030	37370161
chr17	37391846	37391963
chr17	37395159	37395476
chr17	37402016	37402384
chr17	37402594	37402929
chr17	37477792	37477808
chr17	37519821	37520182
chr17	37533807	37534041
chr17	37534343	37534396
chr17	37557289	37558782
chr17	37560741	37560841
chr17	37560962	37561148
chr17	37561237	37561242
chr17	37607332	37608090
chr17	37614067	37614632
chr17	37617456	37617530
chr17	37617778	37618249
chr17	37619106	37619563
chr17	37618470	37618867
chr17	37635956	37636424

chr17	37641448	37642282
chr17	37701922	37702097
chr17	37709400	37709638
chr17	37729763	37730454
chr17	37773403	37773602
chr17	37773749	37775181
chr17	37778236	37778629
chr17	37778835	37779094
chr17	37779563	37779745
chr17	37781866	37782235
chr17	37782450	37783324
chr17	37783540	37783739
chr17	37787091	37787912
chr17	37791207	37791460
chr17	37792783	37793099
chr17	37793210	37794221
chr17	37823947	37824886
chr17	37843377	37843613
chr17	37843860	37844690
chr17	37855938	37856058
chr17	37856212	37856747
chr17	37856893	37857217
chr17	37860891	37860935
chr17	37862292	37862547
chr17	37886203	37887281
chr17	37888157	37888164
chr17	37891415	37891594
chr17	37894150	37897031
chr17	37897189	37898140
chr17	37905990	37906317
chr17	37909120	37909356
chr17	37909770	37911420
chr17	37911696	37911954
chr17	37926498	37926695
chr17	37938410	37938881
chr17	37938991	37939173
chr17	38020238	38020339
chr17	38083089	38083908
chr17	38085286	38085589
chr17	38085723	38086001
chr17	38086252	38086469
chr17	38086719	38086920
chr17	38108469	38109113
chr17	38109549	38109769
chr17	38115415	38115862
chr17	38118818	38119135

chr17	38119790	38120132
chr17	38135231	38135428
chr17	38135686	38135722
chr17	38136093	38136504
chr17	38137078	38137712
chr17	38145553	38145855
chr17	38169814	38170080
chr17	38170252	38173644
chr17	38173811	38178648
chr17	38178776	38179405
chr17	38179782	38180300
chr17	38182131	38183449
chr17	38180481	38181501
chr17	38210480	38211207
chr17	38217956	38218334
chr17	38218871	38220129
chr17	38221339	38221812
chr17	38224386	38224447
chr17	38224575	38226257
chr17	38226371	38226926
chr17	38227140	38228348
chr17	38228499	38228935
chr17	38231596	38231795
chr17	38231911	38232263
chr17	38234032	38234214
chr17	38234324	38234825
chr17	38237367	38237550
chr17	38241569	38241833
chr17	38241965	38242590
chr17	38243598	38243724
chr17	38253257	38253650
chr17	38254103	38255251
chr17	38255685	38258326
chr17	38258503	38258900
chr17	38259786	38260534
chr17	38261074	38261100
chr17	38261254	38261591
chr17	38263866	38264907
chr17	38265511	38266124
chr17	38267367	38268114
chr17	38268239	38268395
chr17	38269298	38269475
chr17	38269628	38270123
chr17	38270366	38270673
chr17	38271161	38271427
chr17	38276153	38276224

chr17	38277315	38280239
chr17	38280378	38281004
chr17	38295774	38296377
chr17	38296521	38297259
chr17	38313317	38313417
chr17	38321423	38321428
chr17	38324788	38325007
chr17	38328898	38329146
chr17	38331990	38332518
chr17	38332660	38333016
chr17	38333259	38334608
chr17	38335598	38335767
chr17	38341115	38341273
chr17	38341649	38342632
chr17	38344225	38344445
chr17	38350455	38350907
chr17	38357991	38358173
chr17	38374926	38375161
chr17	38375254	38375808
chr17	38375929	38376298
chr17	38439998	38440094
chr17	38440418	38441034
chr17	38444088	38444522
chr17	38444624	38444949
chr17	38447065	38447316
chr17	38459607	38459648
chr17	38465448	38466329
chr17	38466453	38466672
chr17	38467188	38467781
chr17	38468226	38469639
chr17	38470070	38470955
chr17	38471743	38472363
chr17	38472790	38473334
chr17	38473848	38474907
chr17	38475096	38475464
chr17	38478654	38479079
chr17	38481239	38481438
chr17	38491658	38492012
chr17	38497604	38498869
chr17	38498995	38499473
chr17	38501148	38502188
chr17	38505303	38506210
chr17	38508885	38509084
chr17	38514698	38515075
chr17	38515241	38515652
chr17	38519052	38519597

chr17	38519876	38520098
chr17	38529546	38529624
chr17	38542783	38543042
chr17	38571304	38571554
chr17	38573272	38574963
chr17	38598621	38600748
chr17	38601486	38601971
chr17	38600950	38601411
chr17	38627700	38628134
chr17	38630232	38630922
chr17	38631269	38631622
chr17	38632571	38633359
chr17	38636281	38636690
chr17	38638040	38638229
chr17	38637353	38637488
chr17	38638576	38639540
chr17	38639603	38641491
chr17	38641994	38644219
chr17	38644671	38644793
chr17	38645114	38645123
chr17	38645197	38645438
chr17	38645591	38645690
chr17	38645924	38648722
chr17	38651545	38651855
chr17	38652143	38652908
chr17	38653441	38654489
chr17	38654657	38655071
chr17	38655684	38656304
chr17	38656418	38657307
chr17	38657418	38658396
chr17	38649693	38650968
chr17	38658500	38658699
chr17	38662506	38662604
chr17	38665232	38665606
chr17	38665833	38666693
chr17	38667674	38668185
chr17	38668744	38669402
chr17	38666833	38667443
chr17	38670486	38670885
chr17	38672813	38673895
chr17	38674192	38676547
chr17	38676793	38677394
chr17	38677542	38678427
chr17	38679323	38679563
chr17	38685752	38685951
chr17	38687309	38687423

chr17	38689430	38690050
chr17	38691159	38691452
chr17	38691584	38691609
chr17	38692514	38693143
chr17	38693304	38693485
chr17	38693889	38695188
chr17	38697209	38698682
chr17	38698768	38700486
chr17	38700677	38701138
chr17	38701254	38701513
chr17	38703539	38703728
chr17	38704009	38704465
chr17	38705101	38705490
chr17	38705621	38705899
chr17	38708000	38708143
chr17	38710097	38710328
chr17	38716220	38716599
chr17	38738475	38738583
chr17	38738944	38738956
chr17	38762562	38762735
chr17	38769500	38769765
chr17	38804008	38805036
chr17	38852788	38853086
chr17	38865173	38865525
chr17	38883857	38884022
chr17	38970297	38970449
chr17	38975049	38975512
chr17	38975754	38975953
chr17	39010531	39010655
chr17	39010775	39010879
chr17	39059452	39059453
chr17	39059865	39059956
chr17	39059615	39059795
chr17	39065564	39065591
chr17	39065849	39066001
chr17	39066123	39066465
chr17	39070754	39070895
chr17	39071403	39071870
chr17	39072046	39072330
chr17	39108875	39109325
chr17	39110152	39110163
chr17	39113002	39113410
chr17	39114342	39114715
chr17	39159343	39159397
chr17	39215529	39215633
chr17	39215913	39215943

chr17	39216520	39216710
chr17	39238888	39239128
chr17	39255091	39255128
chr17	39261041	39261224
chr17	39288269	39288621
chr17	39457551	39457897
chr17	39458004	39458137
chr17	39495238	39495871
chr17	39504315	39504539
chr17	39504662	39504979
chr17	39508956	39509757
chr17	39523711	39524331
chr17	39536140	39536872
chr17	39551139	39551540
chr17	39558764	39558771
chr17	39560360	39560943
chr17	39574140	39574245
chr17	39575972	39576462
chr17	39577204	39577497
chr17	39595430	39595712
chr17	39620903	39621121
chr17	39650785	39651024
chr17	39651295	39651529
chr17	39653366	39653575
chr17	39654620	39654821
chr17	39655102	39655325
chr17	39656663	39657263
chr17	39662246	39662586
chr17	39662895	39663373
chr17	39674987	39675186
chr17	39675400	39676353
chr17	39677777	39677976
chr17	39678275	39679003
chr17	39683070	39684555
chr17	39685021	39685152
chr17	39685342	39685369
chr17	39694026	39694912
chr17	39696619	39696769
chr17	39703955	39705908
chr17	39706012	39706230
chr17	39706964	39707159
chr17	39711335	39711450
chr17	39711574	39711668
chr17	39735692	39735931
chr17	39736086	39737617
chr17	39737720	39738047

chr17	39738169	39738822
chr17	39740167	39741455
chr17	39741957	39744304
chr17	39744572	39745116
chr17	39745403	39745659
chr17	39768639	39769261
chr17	39769365	39769564
chr17	39771304	39771627
chr17	39771936	39772722
chr17	39773927	39774247
chr17	39774789	39775042
chr17	39775366	39776056
chr17	39776246	39777096
chr17	39777229	39777448
chr17	39777882	39780825
chr17	39781120	39782796
chr17	39783132	39783370
chr17	39788750	39788915
chr17	39789786	39790103
chr17	39790565	39790789
chr17	39793725	39794081
chr17	39797492	39797521
chr17	39798646	39798663
chr17	39801920	39802468
chr17	39803665	39805419
chr17	39805739	39806195
chr17	39806516	39808244
chr17	39814994	39815547
chr17	39808435	39814722
chr17	39815901	39816910
chr17	39816993	39817890
chr17	39818265	39818496
chr17	39818796	39818931
chr17	39819185	39819576
chr17	39819666	39819743
chr17	39819864	39820588
chr17	39820742	39821023
chr17	39821316	39823444
chr17	39834228	39834300
chr17	39834768	39834920
chr17	39835080	39835592
chr17	39835755	39835789
chr17	39843445	39843536
chr17	39844038	39844923
chr17	39845118	39845585
chr17	39845818	39846168

chr17	39847961	39848213
chr17	39849663	39849833
chr17	39856426	39856590
chr17	39873511	39873940
chr17	39874216	39874669
chr17	39881771	39881970
chr17	39882528	39883567
chr17	39882231	39882306
chr17	39885213	39885274
chr17	39890097	39890686
chr17	39892483	39893200
chr17	39893374	39893426
chr17	39893586	39893940
chr17	39894541	39896119
chr17	39896350	39896699
chr17	39916822	39916897
chr17	39917498	39917528
chr17	39927040	39927403
chr17	39927518	39927801
chr17	39927905	39928274
chr17	39928379	39929246
chr17	39929580	39929792
chr17	39930212	39930411
chr17	39931524	39931838
chr17	39933054	39933145
chr17	39933384	39933387
chr17	39934664	39935112
chr17	39935727	39936270
chr17	39937581	39937778
chr17	39939115	39939405
chr17	39939635	39939979
chr17	39940094	39941272
chr17	39941693	39942930
chr17	39943461	39943588
chr17	39944157	39944188
chr17	39944510	39944770
chr17	39945141	39946461
chr17	39947161	39948674
chr17	39948855	39949490
chr17	39949605	39950592
chr17	39951081	39951217
chr17	39951456	39951465
chr17	39952546	39952564
chr17	39952661	39952865
chr17	39954003	39954231
chr17	39954602	39954840

chr17	39954968	39956083
chr17	39956673	39957006
chr17	39957189	39957891
chr17	39961076	39961260
chr17	39965716	39966081
chr17	39966375	39969875
chr17	39991998	39992490
chr17	39992703	39993264
chr17	40002316	40003045
chr17	40020870	40021717
chr17	40021944	40022058
chr17	40048097	40048288
chr17	40068081	40068365
chr17	40071531	40071832
chr17	40072026	40072178
chr17	40073087	40073376
chr17	40073619	40073877
chr17	40076544	40077116
chr17	40077840	40077881
chr17	40073986	40076434
chr17	40078070	40078172
chr17	40082330	40082487
chr17	40086447	40086912
chr17	40087024	40087503
chr17	40117374	40117877
chr17	40118025	40119515
chr17	40119804	40119904
chr17	40119994	40120008
chr17	40162236	40162759
chr17	40164909	40165038
chr17	40165436	40165569
chr17	40168435	40168895
chr17	40169048	40170162
chr17	40170347	40170722
chr17	40171778	40172402
chr17	40172746	40173076
chr17	40176527	40176843
chr17	40188128	40188168
chr17	40200350	40200708
chr17	40200799	40200800
chr17	40202175	40202872
chr17	40227298	40227399
chr17	40249938	40250021
chr17	40250249	40250422
chr17	40263909	40264646
chr17	40264852	40265056

chr17	40265220	40265595
chr17	40268378	40268548
chr17	40272670	40273461
chr17	40287966	40288127
chr17	40304506	40304784
chr17	40305631	40305680
chr17	40306583	40306833
chr17	40307139	40307800
chr17	40311794	40312069
chr17	40317018	40317217
chr17	40345742	40345759
chr17	40346348	40346453
chr17	40346616	40346808
chr17	40350797	40350971
chr17	40419162	40419352
chr17	40427441	40427648
chr17	40428005	40429107
chr17	40439781	40439850
chr17	40440190	40440906
chr17	40472053	40472377
chr17	40477309	40477497
chr17	40477880	40477987
chr17	40478176	40478625
chr17	40481030	40481977
chr17	40482333	40482650
chr17	40519306	40519696
chr17	40539597	40539651
chr17	40539789	40540482
chr17	40540904	40541130
chr17	40550418	40550823
chr17	40552913	40553061
chr17	40553283	40553456
chr17	40558340	40558389
chr17	40559515	40560192
chr17	40561361	40561541
chr17	40561690	40562033
chr17	40562539	40563061
chr17	40565182	40565202
chr17	40565682	40565890
chr17	40566062	40566294
chr17	40566547	40566775
chr17	40571584	40572436
chr17	40572971	40573219
chr17	40573358	40575280
chr17	40575533	40575732
chr17	40575847	40576335

chr17	40580126	40580482
chr17	40580710	40581150
chr17	40581904	40582101
chr17	40584835	40585173
chr17	40588416	40588807
chr17	40591195	40591557
chr17	40608918	40609222
chr17	40610544	40610839
chr17	40611008	40611564
chr17	40611963	40612203
chr17	40625041	40625117
chr17	40625375	40625634
chr17	40670024	40671355
chr17	40671451	40672727
chr17	40678029	40678098
chr17	40678428	40678491
chr17	40678839	40679095
chr17	40679195	40679338
chr17	40679473	40679709
chr17	40681588	40682699
chr17	40682773	40683230
chr17	40683434	40684350
chr17	40687735	40688488
chr17	40688648	40688847
chr17	40692473	40692502
chr17	40700495	40701070
chr17	40705656	40707690
chr17	40708000	40708222
chr17	40711665	40711996
chr17	40713436	40713717
chr17	40713860	40714093
chr17	40714445	40715085
chr17	40715957	40716003
chr17	40715274	40715857
chr17	40718605	40719477
chr17	40719636	40720211
chr17	40723072	40723131
chr17	40723482	40723494
chr17	40729181	40729293
chr17	40729814	40730580
chr17	40738936	40739337
chr17	40740030	40740249
chr17	40740322	40740427
chr17	40760704	40761393
chr17	40761753	40762694
chr17	40771476	40771772

chr17	40776135	40776196
chr17	40776521	40776702
chr17	40781347	40781417
chr17	40781590	40781741
chr17	40783180	40783337
chr17	40811000	40811150
chr17	40811336	40812291
chr17	40812662	40812912
chr17	40819105	40819951
chr17	40820274	40820478
chr17	40820902	40821282
chr17	40824086	40824553
chr17	40826421	40826817
chr17	40827262	40829070
chr17	40830906	40831329
chr17	40829403	40830807
chr17	40831433	40832606
chr17	40832792	40833014
chr17	40833223	40833326
chr17	40833439	40833838
chr17	40834170	40835045
chr17	40896143	40897032
chr17	40897160	40897409
chr17	40912702	40913553
chr17	40915651	40915876
chr17	40918412	40918785
chr17	40925173	40925375
chr17	40925485	40926060
chr17	40935038	40935507
chr17	40950026	40950325
chr17	40950513	40950764
chr17	40950979	40950996
chr17	40955933	40956032
chr17	40961623	40961693
chr17	40975795	40976265
chr17	40976411	40976720
chr17	40984707	40985847
chr17	41116209	41116504
chr17	41116651	41117386
chr17	41121367	41121562
chr17	41131723	41133279
chr17	41133411	41133540
chr17	41133713	41133901
chr17	41134108	41134136
chr17	41149489	41149742
chr17	41150546	41151080

chr17	41153527	41154244
chr17	41156235	41156252
chr17	41158564	41159502
chr17	41172148	41172273
chr17	41172377	41172552
chr17	41172630	41174369
chr17	41174523	41174985
chr17	41176187	41176561
chr17	41176857	41177057
chr17	41177220	41177579
chr17	41276398	41276858
chr17	41277029	41277095
chr17	41277596	41278301
chr17	41278546	41278702
chr17	41279938	41280106
chr17	41280812	41281749
chr17	41299441	41299481
chr17	41320833	41321677
chr17	41321848	41322058
chr17	41322360	41322711
chr17	41322857	41323768
chr17	41323884	41324444
chr17	41374853	41375060
chr17	41381067	41382783
chr17	41385588	41385741
chr17	41385966	41385997
chr17	41387404	41387562
chr17	41392304	41393801
chr17	41399690	41401230
chr17	41416196	41416653
chr17	41434776	41434920
chr17	41439537	41439882
chr17	41445526	41446969
chr17	41462415	41462814
chr17	41462885	41463151
chr17	41463424	41463493
chr17	41464640	41465187
chr17	41465507	41467137
chr17	41470602	41470752
chr17	41471253	41471357
chr17	41471573	41471671
chr17	41471957	41472241
chr17	41476148	41476795
chr17	41476953	41476981
chr17	41477210	41477643
chr17	41477795	41478077

chr17	41478615	41479620
chr17	41487140	41487383
chr17	41487668	41487867
chr17	41494585	41495422
chr17	41495765	41495774
chr17	41496091	41496247
chr17	41496527	41497209
chr17	41506740	41506985
chr17	41507110	41507149
chr17	41520410	41520486
chr17	41520745	41520944
chr17	41521601	41522579
chr17	41526880	41527061
chr17	41527165	41527318
chr17	41529272	41529517
chr17	41532612	41532784
chr17	41534285	41534484
chr17	41534604	41534631
chr17	41535182	41536020
chr17	41536179	41536369
chr17	41536739	41537093
chr17	41545609	41546930
chr17	41551573	41551716
chr17	41554712	41555138
chr17	41560873	41561033
chr17	41561258	41561805
chr17	41561961	41562160
chr17	41566460	41566681
chr17	41607363	41608079
chr17	41614416	41614422
chr17	41615133	41615479
chr17	41621449	41623381
chr17	41624703	41624785
chr17	41624983	41625205
chr17	41625990	41626501
chr17	41655757	41656040
chr17	41656337	41657035
chr17	41657182	41657491
chr17	41663024	41663324
chr17	41669077	41669468
chr17	41669688	41669980
chr17	41677252	41677274
chr17	41683923	41684122
chr17	41684322	41684658
chr17	41700649	41700848
chr17	41704765	41704783

chr17	41711448	41711647
chr17	41723331	41723977
chr17	41733575	41733768
chr17	41747306	41747522
chr17	41777830	41778390
chr17	41791021	41791461
chr17	41791627	41791658
chr17	41797849	41798528
chr17	41827332	41828026
chr17	41841670	41841884
chr17	41855312	41855660
chr17	41855803	41856562
chr17	41856712	41856925
chr17	41892032	41892243
chr17	41892359	41892488
chr17	41909788	41911091
chr17	41917535	41917600
chr17	41918427	41918551
chr17	41949536	41950011
chr17	41975987	41976279
chr17	41977812	41978516
chr17	41985786	41986043
chr17	41987923	41988134
chr17	42005563	42005713
chr17	42006807	42007091
chr17	42015129	42015389
chr17	42015610	42016199
chr17	42016833	42017045
chr17	42021072	42021343
chr17	42021875	42021909
chr17	42060901	42061151
chr17	42061464	42061725
chr17	42072067	42072484
chr17	42072912	42073154
chr17	42080159	42080477
chr17	42080837	42081201
chr17	42081512	42084266
chr17	42084626	42084858
chr17	42085386	42085625
chr17	42091403	42092327
chr17	42092527	42092705
chr17	42092841	42092971
chr17	42110297	42110439
chr17	42117657	42117988
chr17	42118850	42118857
chr17	42119563	42119803

chr17	42119931	42120052
chr17	42122928	42123170
chr17	42134430	42134801
chr17	42140153	42140540
chr17	42141810	42142023
chr17	42143280	42144351
chr17	42146161	42146479
chr17	42147056	42147979
chr17	42148176	42148769
chr17	42149006	42149725
chr17	42150305	42150768
chr17	42151034	42151301
chr17	42158712	42158925
chr17	42159048	42159060
chr17	42159196	42159274
chr17	42161249	42161462
chr17	42163481	42164117
chr17	42164716	42165439
chr17	42165516	42165672
chr17	42172679	42173740
chr17	42173964	42174439
chr17	42176279	42176538
chr17	42178435	42178689
chr17	42178983	42179027
chr17	42180092	42180540
chr17	42180897	42181501
chr17	42183244	42183347
chr17	42183551	42183579
chr17	42183954	42184169
chr17	42184448	42184755
chr17	42188108	42188556
chr17	42188780	42189222
chr17	42197944	42198143
chr17	42200265	42200464
chr17	42200609	42202222
chr17	42204377	42204959
chr17	42209726	42210039
chr17	42210188	42210639
chr17	42212563	42212766
chr17	42213729	42214145
chr17	42214259	42214398
chr17	42214933	42217067
chr17	42217734	42219142
chr17	42219364	42220397
chr17	42221828	42222740
chr17	42223367	42223566

chr17	42224370	42224610
chr17	42224839	42224927
chr17	42226628	42226880
chr17	42227753	42227983
chr17	42228774	42228858
chr17	42229901	42231718
chr17	42235962	42236280
chr17	42240549	42240770
chr17	42243551	42243886
chr17	42246065	42246427
chr17	42252230	42252429
chr17	42256619	42256968
chr17	42261333	42261369
chr17	42262073	42262555
chr17	42262752	42263011
chr17	42263135	42264977
chr17	42265095	42265335
chr17	42270794	42271003
chr17	42275519	42277657
chr17	42277783	42278462
chr17	42279427	42279451
chr17	42279531	42279656
chr17	42281570	42281811
chr17	42281944	42282256
chr17	42285580	42286663
chr17	42294707	42298185
chr17	42298342	42299256
chr17	42299525	42299724
chr17	42299857	42300174
chr17	42312114	42312231
chr17	42312525	42313069
chr17	42313260	42313431
chr17	42342677	42342733
chr17	42342821	42342891
chr17	42400988	42404042
chr17	42404423	42404682
chr17	42408997	42409120
chr17	42409506	42410069
chr17	42410396	42410580
chr17	42410698	42410732
chr17	42411328	42411527
chr17	42416495	42416649
chr17	42421582	42422029
chr17	42422174	42422358
chr17	42422646	42423165
chr17	42423857	42423874

chr17	42424205	42426235
chr17	42426406	42426958
chr17	42430776	42432504
chr17	42437864	42438063
chr17	42440287	42441262
chr17	42450788	42450827
chr17	42451144	42451586
chr17	42461909	42463538
chr17	42494841	42495047
chr17	42522067	42522473
chr17	42522921	42523140
chr17	42523346	42523381
chr17	42528377	42528777
chr17	42580031	42581583
chr17	42589511	42589573
chr17	42595297	42595392
chr17	42595653	42595945
chr17	42611349	42611959
chr17	42612336	42612631
chr17	42613158	42613380
chr17	42619572	42621747
chr17	42622204	42622857
chr17	42623756	42625446
chr17	42628753	42628770
chr17	42628994	42629076
chr17	42629610	42630452
chr17	42631436	42631775
chr17	42633426	42633752
chr17	42633876	42634265
chr17	42634329	42635766
chr17	42644620	42644837
chr17	42645644	42646022
chr17	42646456	42646705
chr17	42654409	42654514
chr17	42664236	42664776
chr17	42665053	42665157
chr17	42665387	42665558
chr17	42683089	42683288
chr17	42688497	42688638
chr17	42704583	42704820
chr17	42711845	42712211
chr17	42733713	42734215
chr17	42734383	42734572
chr17	42766423	42767895
chr17	42767993	42768766
chr17	42785795	42786756

chr17	42801315	42801467
chr17	42822991	42823073
chr17	42823172	42823190
chr17	42836041	42836240
chr17	42836528	42836860
chr17	42852600	42853014
chr17	42853271	42853645
chr17	42856644	42856908
chr17	42869683	42869987
chr17	42872132	42873012
chr17	42888792	42889052
chr17	42890955	42891339
chr17	42897898	42898017
chr17	42898155	42898320
chr17	42898398	42898665
chr17	42900025	42900123
chr17	42901061	42901260
chr17	42901879	42901957
chr17	42902912	42903054
chr17	42905472	42905514
chr17	42906757	42908005
chr17	42924491	42924665
chr17	42933618	42933647
chr17	42956045	42956352
chr17	42967879	42968001
chr17	42968799	42968864
chr17	42974411	42974646
chr17	42976237	42977892
chr17	42978217	42978467
chr17	42982920	42984217
chr17	42985807	42986608
chr17	42986725	42986970
chr17	42993491	42993732
chr17	42994519	42995866
chr17	42996682	42997013
chr17	43015416	43015457
chr17	43017704	43018203
chr17	43018300	43018529
chr17	43018842	43019197
chr17	43019493	43019973
chr17	43020498	43020588
chr17	43024371	43025054
chr17	43025232	43025881
chr17	43027953	43028182
chr17	43028369	43028622
chr17	43045062	43046449

chr17	43048217	43051063
chr17	43051331	43051334
chr17	43053197	43053211
chr17	43057513	43057942
chr17	43058243	43059976
chr17	43077313	43077474
chr17	43090029	43090255
chr17	43092316	43092318
chr17	43096270	43096847
chr17	43098436	43099651
chr17	43105293	43105548
chr17	43106059	43106347
chr17	43120667	43120724
chr17	43120895	43122143
chr17	43124100	43125146
chr17	43125554	43126350
chr17	43126576	43126896
chr17	43127273	43127536
chr17	43127736	43128994
chr17	43129269	43130193
chr17	43133120	43133464
chr17	43137241	43137578
chr17	43137853	43139197
chr17	43153192	43153262
chr17	43153813	43153975
chr17	43155290	43155490
chr17	43166989	43167231
chr17	43168560	43168759
chr17	43169180	43169769
chr17	43170127	43170384
chr17	43170501	43170756
chr17	43170993	43171286
chr17	43172553	43172789
chr17	43172934	43172947
chr17	43178677	43178731
chr17	43178851	43179126
chr17	43198974	43199214
chr17	43199765	43199806
chr17	43199896	43199973
chr17	43200078	43200545
chr17	43200708	43202524
chr17	43204096	43205892
chr17	43206204	43206951
chr17	43207115	43207455
chr17	43207576	43207949
chr17	43209031	43210979

chr17	43212365	43212762
chr17	43212959	43213566
chr17	43219634	43219723
chr17	43220004	43220162
chr17	43223082	43223289
chr17	43221127	43222867
chr17	43223800	43223980
chr17	43224059	43225701
chr17	43225851	43227502
chr17	43227610	43227737
chr17	43228642	43228953
chr17	43229172	43229776
chr17	43232275	43232424
chr17	43232541	43232922
chr17	43233035	43233359
chr17	43233430	43233597
chr17	43237856	43239018
chr17	43239390	43239674
chr17	43241551	43241752
chr17	43247235	43247347
chr17	43247423	43247434
chr17	43247587	43250918
chr17	43254893	43255010
chr17	43252437	43254241
chr17	43256610	43256909
chr17	43260894	43261127
chr17	43261317	43262072
chr17	43270472	43271036
chr17	43274740	43274859
chr17	43275086	43275635
chr17	43277168	43277367
chr17	43278452	43278591
chr17	43278844	43279055
chr17	43279193	43279707
chr17	43280035	43280342
chr17	43288870	43288996
chr17	43290849	43290963
chr17	43296057	43296326
chr17	43296497	43296703
chr17	43297496	43297585
chr17	43298340	43300386
chr17	43303261	43305109
chr17	43305220	43306473
chr17	43306641	43307705
chr17	43307859	43308260
chr17	43308477	43308723

chr17	43310141	43310403
chr17	43314477	43314480
chr17	43317136	43317684
chr17	43317882	43319782
chr17	43324488	43326679
chr17	43327094	43327137
chr17	43337044	43337160
chr17	43337394	43337457
chr17	43349400	43349666
chr17	43354501	43354586
chr17	43356479	43356690
chr17	43357535	43357648
chr17	43358617	43358819
chr17	43358918	43359235
chr17	43359484	43359712
chr17	43360169	43360327
chr17	43360847	43360932
chr17	43361194	43362593
chr17	43365868	43366096
chr17	43366373	43366663
chr17	43369694	43369814
chr17	43370178	43370380
chr17	43370593	43371678
chr17	43373278	43373816
chr17	43374307	43374569
chr17	43376047	43376227
chr17	43376615	43376872
chr17	43377331	43377382
chr17	43378051	43378197
chr17	43378304	43378658
chr17	43383015	43383171
chr17	43384738	43384756
chr17	43385014	43385289
chr17	43388147	43388488
chr17	43389069	43389853
chr17	43390006	43391066
chr17	43391659	43391926
chr17	43392954	43393315
chr17	43393530	43394459
chr17	43394681	43395215
chr17	43396080	43397252
chr17	43397794	43398378
chr17	43415062	43415091
chr17	43443518	43443870
chr17	43445501	43445570
chr17	43445849	43446009

chr17	43447413	43448108
chr17	43448406	43450261
chr17	43451124	43451875
chr17	43452401	43452670
chr17	43450346	43450819
chr17	43453020	43453359
chr17	43454183	43454633
chr17	43458190	43458263
chr17	43458518	43458780
chr17	43458943	43459220
chr17	43459520	43459859
chr17	43459983	43460327
chr17	43461711	43461932
chr17	43462743	43463039
chr17	43463184	43463331
chr17	43463563	43463619
chr17	43469505	43469656
chr17	43474727	43474920
chr17	43484726	43484741
chr17	43488811	43488841
chr17	43488932	43489686
chr17	43494051	43494154
chr17	43501808	43503236
chr17	43505807	43506246
chr17	43508120	43509328
chr17	43509468	43510833
chr17	43511265	43511633
chr17	43515487	43515713
chr17	43516628	43517233
chr17	43517567	43518279
chr17	43522111	43522419
chr17	43522928	43523015
chr17	43523182	43523973
chr17	43524513	43526238
chr17	43534802	43535045
chr17	43536197	43536474
chr17	43537806	43538498
chr17	43538988	43539251
chr17	43539533	43539989
chr17	43540628	43541044
chr17	43546397	43546938
chr17	43556801	43557000
chr17	43560428	43560643
chr17	43561093	43561720
chr17	43561825	43562442
chr17	43562970	43562991

chr17	43564022	43564095
chr17	43564248	43564264
chr17	43564813	43565294
chr17	43565890	43566258
chr17	43566471	43566481
chr17	43565401	43565683
chr17	43566913	43569036
chr17	43575009	43575065
chr17	43579620	43579819
chr17	43583896	43584095
chr17	43593497	43593705
chr17	43594764	43594963
chr17	43651730	43652086
chr17	43654233	43654571
chr17	43656074	43656193
chr17	43656397	43656795
chr17	43657300	43657512
chr17	43658620	43658643
chr17	43658709	43659051
chr17	43659426	43659514
chr17	43660122	43660424
chr17	43661023	43661307
chr17	43661559	43664041
chr17	43664901	43665100
chr17	43669050	43669172
chr17	43697083	43697297
chr17	43697608	43698481
chr17	43699685	43699732
chr17	43717706	43718076
chr17	43861739	43862028
chr17	43862134	43862409
chr17	43971250	43971729
chr17	43971947	43972354
chr17	44026356	44026377
chr17	44069229	44069305
chr17	44080682	44080694
chr17	44140606	44140640
chr17	44140938	44141048
chr17	44150411	44150492
chr17	44150642	44150769
chr17	44193729	44193855
chr17	44211416	44211704
chr17	44231755	44232001
chr17	44240707	44241074
chr17	44256242	44256285
chr17	44264207	44264531

chr17	44269065	44269330
chr17	44269936	44270830
chr17	44270934	44271475
chr17	44271606	44271744
chr17	44290509	44290964
chr17	44291801	44291979
chr17	44300712	44300730
chr17	44302107	44303445
chr17	44337580	44338128
chr17	44339956	44340012
chr17	44340169	44340216
chr17	44341539	44342123
chr17	44343338	44345077
chr17	44438439	44439033
chr17	44441673	44441690
chr17	44449767	44450077
chr17	44450217	44450872
chr17	44467826	44468173
chr17	44487515	44487585
chr17	44523237	44523304
chr17	44523539	44523613
chr17	44636324	44636430
chr17	44653112	44653416
chr17	44656019	44656218
chr17	44656288	44656981
chr17	44657416	44657709
chr17	44667592	44668385
chr17	44668625	44668776
chr17	44688743	44688758
chr17	44741246	44741321
chr17	44769594	44769705
chr17	44769865	44769934
chr17	44805201	44805359
chr17	44811443	44811553
chr17	44848467	44848863
chr17	44849376	44849889
chr17	44896274	44896760
chr17	44928542	44928741
chr17	44928953	44929248
chr17	45000049	45001100
chr17	45006722	45006977
chr17	45056201	45056555
chr17	45129037	45129329
chr17	45144478	45144931
chr17	45145059	45145119
chr17	45146859	45146956

chr17	45147477	45148108
chr17	45148768	45148989
chr17	45155248	45155322
chr17	45175882	45176648
chr17	45176736	45178161
chr17	45178787	45178976
chr17	45212919	45213118
chr17	45213709	45214130
chr17	45219617	45219847
chr17	45234136	45234637
chr17	45258974	45259164
chr17	45266083	45266616
chr17	45266841	45267165
chr17	45303046	45303073
chr17	45307455	45307716
chr17	45317145	45317376
chr17	45331077	45331711
chr17	45355996	45356653
chr17	45357040	45357271
chr17	45371102	45371207
chr17	45382918	45383090
chr17	45384787	45384825
chr17	45390907	45391089
chr17	45391275	45391771
chr17	45392728	45392995
chr17	45393238	45393348
chr17	45393731	45393829
chr17	45400623	45400682
chr17	45400899	45401976
chr17	45402060	45402374
chr17	45500672	45501109
chr17	45534973	45535190
chr17	45556213	45556266
chr17	45569177	45569897
chr17	45570059	45570590
chr17	45571412	45571492
chr17	45577881	45577928
chr17	45581215	45581754
chr17	45579340	45581018
chr17	45587573	45587739
chr17	45590093	45590223
chr17	45590589	45590934
chr17	45591598	45591792
chr17	45592556	45592775
chr17	45594035	45594209
chr17	45608088	45608290

chr17	45608448	45610140
chr17	45634519	45634723
chr17	45642502	45642678
chr17	45644753	45645057
chr17	45651957	45652213
chr17	45666525	45666604
chr17	45695857	45696090
chr17	45697784	45698586
chr17	45698806	45699313
chr17	45699445	45699539
chr17	45704455	45704770
chr17	45705636	45705835
chr17	45709823	45709914
chr17	45720544	45720752
chr17	45725010	45725064
chr17	45726284	45727062
chr17	45727432	45728409
chr17	45733635	45733661
chr17	45733807	45733971
chr17	45743276	45743319
chr17	45748235	45748596
chr17	45763417	45763449
chr17	45770567	45772250
chr17	45772379	45772662
chr17	45887207	45887686
chr17	45887843	45888342
chr17	45898655	45899822
chr17	45908252	45908834
chr17	45907836	45908075
chr17	45909139	45909384
chr17	45910962	45911222
chr17	45917939	45918715
chr17	45918839	45919040
chr17	45919115	45919377
chr17	45919524	45919676
chr17	45920349	45920414
chr17	45920662	45921153
chr17	45927825	45929453
chr17	45938756	45939652
chr17	45941834	45943306
chr17	45940116	45940952
chr17	45944976	45945206
chr17	45948384	45948674
chr17	45949361	45950510
chr17	45950929	45951226
chr17	45963184	45963383

chr17	45963955	45964624
chr17	45965453	45965625
chr17	45966901	45967106
chr17	45971239	45971769
chr17	45972316	45973144
chr17	45973542	45974724
chr17	45982336	45982605
chr17	45982861	45983140
chr17	45988922	45988932
chr17	45990096	45990320
chr17	45995416	45995727
chr17	46004587	46004786
chr17	46012496	46012670
chr17	46013228	46013447
chr17	46013628	46013890
chr17	46014093	46014329
chr17	46015381	46015463
chr17	46017979	46018012
chr17	46018371	46018722
chr17	46018968	46020536
chr17	46020605	46020817
chr17	46021260	46021462
chr17	46024624	46024847
chr17	46026948	46027348
chr17	46043249	46043303
chr17	46043733	46043959
chr17	46044216	46044415
chr17	46044807	46045170
chr17	46045998	46046415
chr17	46047777	46048054
chr17	46048481	46049075
chr17	46049230	46049529
chr17	46054467	46054857
chr17	46063014	46063187
chr17	46063427	46063442
chr17	46080012	46080299
chr17	46083111	46083510
chr17	46088927	46089321
chr17	46089519	46089535
chr17	46095617	46096061
chr17	46096799	46097064
chr17	46099223	46099459
chr17	46100759	46101853
chr17	46101999	46102381
chr17	46102800	46103025
chr17	46104803	46104809

chr17	46103283	46104728
chr17	46106636	46106880
chr17	46107025	46107664
chr17	46114499	46115139
chr17	46115263	46115626
chr17	46121673	46122019
chr17	46122600	46123562
chr17	46123701	46123995
chr17	46124574	46127142
chr17	46127592	46127831
chr17	46128167	46128501
chr17	46128993	46129192
chr17	46129415	46129639
chr17	46130402	46130753
chr17	46131318	46131599
chr17	46132975	46133778
chr17	46137710	46137931
chr17	46139584	46139631
chr17	46155060	46155342
chr17	46156326	46156656
chr17	46158511	46158822
chr17	46171264	46171499
chr17	46173300	46173499
chr17	46173908	46174069
chr17	46177258	46177579
chr17	46177717	46179395
chr17	46184221	46186683
chr17	46192988	46193282
chr17	46195932	46195986
chr17	46199913	46200055
chr17	46204538	46204754
chr17	46209715	46209742
chr17	46876390	46876542
chr17	46908089	46908238
chr17	46908373	46908872
chr17	46909025	46909031
chr17	46916429	46916599
chr17	46919896	46920105
chr17	46922473	46922515
chr17	46968849	46969726
chr17	46969925	46970990
chr17	46983626	46983978
chr17	46984928	46986752
chr17	46987093	46987504
chr17	46988119	46988391
chr17	46989484	46989683

chr17	46992503	46993192
chr17	46993391	46993704
chr17	46997379	46997578
chr17	47015876	47016081
chr17	47018832	47018834
chr17	47023160	47023240
chr17	47021166	47022785
chr17	47035946	47035998
chr17	47049427	47049748
chr17	47050197	47050239
chr17	47093836	47094041
chr17	47099508	47099801
chr17	47100112	47100242
chr17	47110089	47110306
chr17	47125178	47125425
chr17	47178409	47178571
chr17	47179014	47179705
chr17	47179779	47179991
chr17	47187939	47187941
chr17	47189260	47189599
chr17	47194860	47195304
chr17	47203542	47203791
chr17	47203897	47204554
chr17	47209882	47210761
chr17	47223997	47224326
chr17	47224522	47224819
chr17	47268155	47268530
chr17	47269082	47271320
chr17	47271428	47271600
chr17	47271729	47271928
chr17	47275658	47275903
chr17	47307898	47308098
chr17	47325073	47325282
chr17	47329340	47329552
chr17	47332210	47332334
chr17	47334273	47334338
chr17	47337375	47338122
chr17	47338253	47338687
chr17	47343211	47344106
chr17	47354978	47355361
chr17	47365879	47366035
chr17	47393167	47393292
chr17	47419336	47419639
chr17	47437821	47439812
chr17	47439924	47440187
chr17	47440325	47440534

chr17	47440765	47440992
chr17	47491770	47492363
chr17	47526576	47526810
chr17	47526927	47527102
chr17	47527494	47527856
chr17	47528401	47528838
chr17	47573120	47574023
chr17	47574469	47575447
chr17	47580364	47580492
chr17	47580270	47580295
chr17	47584531	47585474
chr17	47585677	47585896
chr17	47586319	47586638
chr17	47586754	47586993
chr17	47587182	47587393
chr17	47592348	47593232
chr17	47594032	47594236
chr17	47599496	47599932
chr17	47600042	47600614
chr17	47600892	47601096
chr17	47601485	47602140
chr17	47602842	47603029
chr17	47604733	47605374
chr17	47620086	47620561
chr17	47630254	47630453
chr17	47633278	47633663
chr17	47643771	47644095
chr17	47645381	47645939
chr17	47646960	47647459
chr17	47652676	47655032
chr17	47657793	47657992
chr17	47660282	47660847
chr17	47695023	47695179
chr17	47723651	47725072
chr17	47725810	47726386
chr17	47745286	47745635
chr17	47754525	47754841
chr17	47754983	47755839
chr17	47765454	47765717
chr17	47784849	47786141
chr17	47840515	47841587
chr17	47841935	47842046
chr17	47865668	47865826
chr17	47865936	47866465
chr17	47928300	47928784
chr17	47938435	47938634

chr17	47962898	47963118
chr17	47963833	47963930
chr17	47964042	47964611
chr17	47964839	47965855
chr17	47966094	47966136
chr17	47966330	47966885
chr17	47972345	47972582
chr17	47979919	47980081
chr17	47987706	47988081
chr17	48032747	48032946
chr17	48038377	48038560
chr17	48045878	48046242
chr17	48055897	48056319
chr17	48057312	48057570
chr17	48071576	48071628
chr17	48070665	48071426
chr17	48072561	48073010
chr17	48074216	48074519
chr17	48075871	48076070
chr17	48104518	48105092
chr17	48105197	48106952
chr17	48112210	48112244
chr17	48112308	48112540
chr17	48112988	48113294
chr17	48113589	48115360
chr17	48115550	48116084
chr17	48118716	48119100
chr17	48122867	48123064
chr17	48123132	48123521
chr17	48124132	48125117
chr17	48125336	48125458
chr17	48125638	48125700
chr17	48125906	48126128
chr17	48126321	48126765
chr17	48126904	48129829
chr17	48129953	48130035
chr17	48130403	48131506
chr17	48131676	48138003
chr17	48143281	48143651
chr17	48144412	48144524
chr17	48138399	48143206
chr17	48144686	48144760
chr17	48145890	48146156
chr17	48146779	48146978
chr17	48147704	48148140
chr17	48149306	48149505

chr17	48150847	48151046
chr17	48161323	48161585
chr17	48162089	48162532
chr17	48163785	48164115
chr17	48164237	48164374
chr17	48165290	48165489
chr17	48169922	48170245
chr17	48170577	48171503
chr17	48171872	48172404
chr17	48172665	48173573
chr17	48177144	48177214
chr17	48183978	48184598
chr17	48184679	48186312
chr17	48194585	48195327
chr17	48204566	48204812
chr17	48205864	48206083
chr17	48206267	48206466
chr17	48206794	48207432
chr17	48207616	48208235
chr17	48208547	48208848
chr17	48225115	48225314
chr17	48225595	48226208
chr17	48226416	48229202
chr17	48229507	48230267
chr17	48234545	48234854
chr17	48234988	48235217
chr17	48238438	48239331
chr17	48246442	48246810
chr17	48252189	48252252
chr17	48252358	48252388
chr17	48255207	48255847
chr17	48256104	48256808
chr17	48261266	48261946
chr17	48262111	48262576
chr17	48262658	48262686
chr17	48276086	48276363
chr17	48276635	48277928
chr17	48278086	48278741
chr17	48282776	48282975
chr17	48287911	48288838
chr17	48290026	48290430
chr17	48296285	48296571
chr17	48297224	48297376
chr17	48300427	48300626
chr17	48298672	48300046
chr17	48349292	48349348

chr17	48349721	48352543
chr17	48353205	48353619
chr17	48354115	48354611
chr17	48355470	48355905
chr17	48356144	48356530
chr17	48356890	48357373
chr17	48364595	48364994
chr17	48365513	48365711
chr17	48366528	48366727
chr17	48367178	48368195
chr17	48365904	48365954
chr17	48368991	48368995
chr17	48369609	48369774
chr17	48394374	48394497
chr17	48399253	48399486
chr17	48399963	48400116
chr17	48422763	48423288
chr17	48423405	48424735
chr17	48439599	48439966
chr17	48448949	48449366
chr17	48449674	48451015
chr17	48468801	48469059
chr17	48469214	48469427
chr17	48470506	48470558
chr17	48473565	48474383
chr17	48474678	48475206
chr17	48501090	48501742
chr17	48501869	48502341
chr17	48502806	48505157
chr17	48505384	48506246
chr17	48506375	48506923
chr17	48509481	48509593
chr17	48513741	48514072
chr17	48514894	48514966
chr17	48517818	48518230
chr17	48518543	48518792
chr17	48519276	48519431
chr17	48520113	48521187
chr17	48522570	48522769
chr17	48529936	48530182
chr17	48529318	48529626
chr17	48531433	48531616
chr17	48535987	48536186
chr17	48536456	48537028
chr17	48544286	48544485
chr17	48555234	48556749

chr17	48556991	48557670
chr17	48558472	48558592
chr17	48558735	48558793
chr17	48559618	48560323
chr17	48560614	48560860
chr17	48564506	48565190
chr17	48565436	48565816
chr17	48567233	48567416
chr17	48568955	48569233
chr17	48573317	48573641
chr17	48575044	48575299
chr17	48578513	48578853
chr17	48581734	48582211
chr17	48584067	48584467
chr17	48585481	48586179
chr17	48587068	48587267
chr17	48588557	48588846
chr17	48588950	48589235
chr17	48595148	48595273
chr17	48595497	48595599
chr17	48595675	48595696
chr17	48595872	48596036
chr17	48596102	48596391
chr17	48596580	48596938
chr17	48597078	48597435
chr17	48598970	48599169
chr17	48600048	48600788
chr17	48600962	48601316
chr17	48609620	48609890
chr17	48610092	48613045
chr17	48616454	48616971
chr17	48617139	48617518
chr17	48617724	48617923
chr17	48618130	48618368
chr17	48618511	48618710
chr17	48618885	48619908
chr17	48620179	48620491
chr17	48622069	48622328
chr17	48624423	48625127
chr17	48636710	48637311
chr17	48637450	48637901
chr17	48638056	48638742
chr17	48642511	48642714
chr17	48654027	48654096
chr17	48687337	48687536
chr17	48707940	48708144

chr17	48711893	48713697
chr17	48714217	48714665
chr17	48714807	48714901
chr17	48715049	48716390
chr17	48716503	48716779
chr17	48716889	48718496
chr17	48718940	48719242
chr17	48719413	48719493
chr17	48719894	48720296
chr17	48737055	48737730
chr17	48737880	48740426
chr17	48740514	48740596
chr17	48745261	48745425
chr17	48745581	48746497
chr17	48747064	48747276
chr17	48747468	48747555
chr17	48748000	48749201
chr17	48749494	48749748
chr17	48749852	48750051
chr17	48751014	48751294
chr17	48754266	48754581
chr17	48761708	48762136
chr17	48762360	48762559
chr17	48763676	48763684
chr17	48763809	48764073
chr17	48766765	48766964
chr17	48772436	48772562
chr17	48780820	48781078
chr17	48782574	48782632
chr17	48782872	48783108
chr17	48784405	48785620
chr17	48785924	48786320
chr17	48791847	48791876
chr17	48792709	48794666
chr17	48795383	48795548
chr17	48796390	48797454
chr17	48799875	48799999
chr17	48811868	48811956
chr17	48812340	48812569
chr17	48839394	48839702
chr17	48857644	48857774
chr17	48860201	48860379
chr17	48860844	48860956
chr17	48861304	48861321
chr17	48862846	48863093
chr17	48863197	48863208

chr17	48863516	48864479
chr17	48864663	48865135
chr17	48865402	48865569
chr17	48865674	48866100
chr17	48866808	48867581
chr17	48867895	48868094
chr17	48873092	48873491
chr17	48875732	48876447
chr17	48876792	48876883
chr17	48877055	48877545
chr17	48880493	48880512
chr17	48882839	48882962
chr17	48894965	48895351
chr17	48895603	48896012
chr17	48896223	48896400
chr17	48924656	48924839
chr17	48929340	48929506
chr17	48929693	48930120
chr17	48931986	48932343
chr17	48942264	48944816
chr17	48945036	48945492
chr17	48946415	48946645
chr17	48960132	48960602
chr17	48961401	48961908
chr17	48962737	48962853
chr17	48970014	48970193
chr17	48970714	48971105
chr17	48973907	48974105
chr17	48974292	48974558
chr17	48979703	48979906
chr17	48981058	48983185
chr17	48983926	48984142
chr17	48984953	48984976
chr17	48985758	48986274
chr17	48991815	48992021
chr17	48992836	48992872
chr17	48993671	48993878
chr17	48996729	48997221
chr17	48997387	48997833
chr17	48998082	48998105
chr17	48998636	48998775
chr17	48998990	49000233
chr17	49000615	49000851
chr17	49001043	49001500
chr17	49002450	49003023
chr17	49003208	49003407

chr17	49006908	49007050
chr17	49008273	49008536
chr17	49008717	49009374
chr17	49009594	49009979
chr17	49010458	49010569
chr17	49010744	49011326
chr17	49012771	49012984
chr17	49013119	49013148
chr17	49018692	49018867
chr17	49025685	49026347
chr17	49026701	49027132
chr17	49027254	49029338
chr17	49029646	49030262
chr17	49030432	49031838
chr17	49032339	49032550
chr17	49078451	49078561
chr17	49134146	49134705
chr17	49137541	49137664
chr17	49151290	49151569
chr17	49153358	49153581
chr17	49159490	49159695
chr17	49163238	49163772
chr17	49164766	49165142
chr17	49189955	49190460
chr17	49193776	49194063
chr17	49196873	49196967
chr17	49197080	49198823
chr17	49198951	49199222
chr17	49199340	49200296
chr17	49200552	49200727
chr17	49201294	49201447
chr17	49203033	49203251
chr17	49217565	49217764
chr17	49218053	49218467
chr17	49225217	49225303
chr17	49229601	49230049
chr17	49230140	49230698
chr17	49230882	49231503
chr17	49231585	49231941
chr17	49235450	49235779
chr17	49239679	49239894
chr17	49240280	49240583
chr17	49243022	49244852
chr17	49246858	49247057
chr17	49247657	49247918
chr17	49252033	49252478

chr17	49263180	49263221
chr17	49303634	49303795
chr17	49303909	49303950
chr17	49304192	49304740
chr17	49305474	49305787
chr17	49324149	49324192
chr17	49336794	49337799
chr17	49337941	49338596
chr17	49339094	49339466
chr17	49340432	49340604
chr17	49342956	49342972
chr17	49376436	49376437
chr17	49449945	49450211
chr17	50222978	50223177
chr17	51648194	51648442
chr17	51694355	51694370
chr17	51703863	51704018
chr17	51705429	51705738
chr17	52977889	52979240
chr17	53025958	53026041
chr17	53044869	53044891
chr17	53045365	53045976
chr17	53046249	53046745
chr17	53249960	53250058
chr17	53311217	53311559
chr17	53315693	53315752
chr17	53341807	53341823
chr17	53342024	53342120
chr17	53342776	53343320
chr17	53351248	53351424
chr17	53362821	53363082
chr17	53364509	53364666
chr17	53365866	53366056
chr17	53366172	53366192
chr17	53398417	53398450
chr17	53497995	53498140
chr17	53498245	53499362
chr17	53500111	53500258
chr17	53510442	53510709
chr17	53510931	53511580
chr17	53518250	53518259
chr17	53532637	53532834
chr17	53820397	53820521
chr17	53828234	53828866
chr17	53828997	53829071
chr17	54671351	54671649

chr17	54672101	54672496
chr17	54793590	54793599
chr17	54794643	54794933
chr17	54795098	54795485
chr17	54857839	54858086
chr17	54863516	54863949
chr17	54864610	54864826
chr17	54885742	54886072
chr17	54886222	54886285
chr17	54886605	54886606
chr17	54886799	54886824
chr17	54887135	54887171
chr17	54910003	54910189
chr17	54910331	54912756
chr17	54945202	54945247
chr17	54948067	54948114
chr17	54974018	54974217
chr17	54977208	54977219
chr17	54978628	54978831
chr17	54989110	54990498
chr17	54990578	54993253
chr17	54993394	54993707
chr17	54996339	54996835
chr17	54997154	54997391
chr17	55017291	55017293
chr17	55017497	55017718
chr17	55021216	55021460
chr17	55037924	55038411
chr17	55038536	55038717
chr17	55044028	55044169
chr17	55050797	55050897
chr17	55054974	55055259
chr17	55055582	55055763
chr17	55056427	55056783
chr17	55057928	55058259
chr17	55059371	55059697
chr17	55060032	55060204
chr17	55060341	55060531
chr17	55129667	55129698
chr17	55131660	55131859
chr17	55139350	55139489
chr17	55145910	55146020
chr17	55157384	55157523
chr17	55157773	55158472
chr17	55162052	55162340
chr17	55162585	55163857

chr17	55164090	55164133
chr17	55165987	55166016
chr17	55184867	55185085
chr17	55208608	55208900
chr17	55210059	55210258
chr17	55287642	55287904
chr17	55332916	55333177
chr17	55333312	55333830
chr17	55333981	55334659
chr17	55334855	55335078
chr17	55335717	55336030
chr17	55341936	55342240
chr17	55342448	55342576
chr17	55369757	55369905
chr17	55370026	55370258
chr17	55370368	55370530
chr17	55370827	55371006
chr17	55386687	55386954
chr17	55387115	55387732
chr17	55388613	55388727
chr17	55388830	55388936
chr17	55389088	55389180
chr17	55444352	55444468
chr17	55446418	55446777
chr17	55447338	55447965
chr17	55449602	55449765
chr17	55452568	55453266
chr17	55453546	55453677
chr17	55454003	55454202
chr17	55454501	55454911
chr17	55455283	55455414
chr17	55499671	55499874
chr17	55517578	55517821
chr17	55601170	55601360
chr17	55601614	55601884
chr17	55607841	55608057
chr17	55607670	55607770
chr17	55655048	55655247
chr17	55675103	55675306
chr17	55685276	55685578
chr17	55713713	55715323
chr17	55715951	55715991
chr17	55739769	55739797
chr17	55740084	55740283
chr17	55752087	55752113
chr17	55925776	55925885

chr17	55926542	55926741
chr17	55927007	55928105
chr17	55928516	55928666
chr17	55935264	55935694
chr17	55938116	55938608
chr17	55938745	55939770
chr17	55941259	55941422
chr17	55942283	55942458
chr17	55944607	55946921
chr17	55947420	55947784
chr17	55948230	55948354
chr17	55951347	55952837
chr17	55953004	55954100
chr17	55954548	55954787
chr17	55955402	55955946
chr17	55954915	55955197
chr17	55956052	55956207
chr17	55956271	55957598
chr17	55966881	55967098
chr17	55967283	55967757
chr17	55968024	55968400
chr17	55968516	55968715
chr17	55969963	55971179
chr17	55971483	55971516
chr17	55976691	55977657
chr17	55978596	55978722
chr17	55979020	55979537
chr17	55979610	55979702
chr17	55980452	55980719
chr17	55980883	55981141
chr17	55981517	55981611
chr17	55990530	55990644
chr17	55995553	55995772
chr17	56005535	56005816
chr17	56012550	56013216
chr17	56013379	56013899
chr17	56015223	56015312
chr17	56019769	56019903
chr17	56020464	56020637
chr17	56023137	56024602
chr17	56025582	56028222
chr17	56029307	56029555
chr17	56029649	56029996
chr17	56031566	56032472
chr17	56032702	56033247
chr17	56034243	56034351

chr17	56062888	56063277
chr17	56064116	56066426
chr17	56066669	56066765
chr17	56072722	56072737
chr17	56073037	56073198
chr17	56082689	56083072
chr17	56083750	56085526
chr17	56085823	56086335
chr17	56100169	56100448
chr17	56100577	56100966
chr17	56159865	56160083
chr17	56160291	56160608
chr17	56160800	56162135
chr17	56296009	56296637
chr17	56296749	56297086
chr17	56297314	56297390
chr17	56326651	56327650
chr17	56395414	56395663
chr17	56401390	56402304
chr17	56429055	56430240
chr17	56430325	56430603
chr17	56439701	56439905
chr17	56441694	56442038
chr17	56479989	56480408
chr17	56480669	56481082
chr17	56486839	56487137
chr17	56488688	56489057
chr17	56492049	56492319
chr17	56492702	56493088
chr17	56493213	56493489
chr17	56494335	56494956
chr17	56495202	56495406
chr17	56569612	56569811
chr17	56583609	56584092
chr17	56584157	56584503
chr17	56584799	56585097
chr17	56586783	56586941
chr17	56587833	56587997
chr17	56589398	56589626
chr17	56590325	56590581
chr17	56590909	56591388
chr17	56591513	56592938
chr17	56594280	56595342
chr17	56595754	56597419
chr17	56597972	56598189
chr17	56600424	56600560

chr17	56600634	56600859
chr17	56602308	56602699
chr17	56602953	56603606
chr17	56603757	56604125
chr17	56609077	56609524
chr17	56652876	56653251
chr17	56660520	56660572
chr17	56660824	56660960
chr17	56661803	56662002
chr17	56702579	56702754
chr17	56708536	56708746
chr17	56708894	56708978
chr17	56709169	56709739
chr17	56718006	56718116
chr17	56720834	56720896
chr17	56735765	56736067
chr17	56736160	56737078
chr17	56742966	56743036
chr17	56743459	56744154
chr17	56744348	56744793
chr17	56754528	56754794
chr17	56756623	56756782
chr17	56757029	56757697
chr17	56769684	56769856
chr17	56770054	56770387
chr17	56770590	56770946
chr17	56775411	56775488
chr17	56780073	56780178
chr17	56780576	56780826
chr17	56784708	56784892
chr17	56818323	56818514
chr17	56818725	56819143
chr17	56819772	56820067
chr17	56833310	56833580
chr17	56833876	56834109
chr17	56863651	56863754
chr17	56864013	56864108
chr17	57082266	57082604
chr17	57151894	57152217
chr17	57170957	57171674
chr17	57172176	57172499
chr17	57183351	57184172
chr17	57184408	57184826
chr17	57186299	57186300
chr17	57190641	57190951
chr17	57199585	57199931

chr17	57212236	57212529
chr17	57215796	57216084
chr17	57217318	57217684
chr17	57219253	57219668
chr17	57226546	57226772
chr17	57227289	57227640
chr17	57227865	57228180
chr17	57231710	57232626
chr17	57233008	57233327
chr17	57233901	57234313
chr17	57236027	57236749
chr17	57240488	57240689
chr17	57243519	57243637
chr17	57255379	57255453
chr17	57286623	57287820
chr17	57287891	57288394
chr17	57293601	57294360
chr17	57297528	57297716
chr17	57297997	57298184
chr17	57298250	57298618
chr17	57364793	57364951
chr17	57407309	57407523
chr17	57408692	57410381
chr17	57473333	57473346
chr17	57474118	57474334
chr17	57512638	57512661
chr17	57518102	57518243
chr17	57529402	57529567
chr17	57529645	57529870
chr17	57531349	57531555
chr17	57598866	57598996
chr17	57642647	57643200
chr17	57695634	57695947
chr17	57696368	57697700
chr17	57697775	57698159
chr17	57698291	57698613
chr17	57718694	57718893
chr17	57733933	57734325
chr17	57739189	57739471
chr17	57771627	57771826
chr17	57773632	57773687
chr17	57782919	57783118
chr17	57784367	57784700
chr17	57785118	57785515
chr17	57806596	57807072
chr17	57830832	57831146

chr17	57831889	57832304
chr17	57833659	57833935
chr17	57839189	57839429
chr17	57846421	57846799
chr17	57860123	57861198
chr17	57862815	57863055
chr17	57863803	57864314
chr17	57867355	57867489
chr17	57877913	57878112
chr17	57898943	57899159
chr17	57906256	57907084
chr17	57908128	57908688
chr17	57909278	57909477
chr17	57912019	57912218
chr17	57914346	57915171
chr17	57915354	57915842
chr17	57916425	57916890
chr17	57917884	57918266
chr17	57918473	57918806
chr17	57919420	57922005
chr17	57922294	57922664
chr17	57922853	57924699
chr17	57917361	57917622
chr17	57925883	57927005
chr17	57928252	57928719
chr17	57928852	57929092
chr17	57929219	57929509
chr17	57929678	57929688
chr17	57930145	57931307
chr17	57931823	57932027
chr17	57932556	57932874
chr17	57933012	57933476
chr17	57953958	57954355
chr17	57969492	57970282
chr17	57970501	57971264
chr17	57972735	57972934
chr17	57976172	57976371
chr17	57976478	57976658
chr17	57976827	57976861
chr17	57982332	57982527
chr17	57983727	57983881
chr17	57992976	57993088
chr17	58041214	58042627
chr17	58102962	58104789
chr17	58154593	58154905
chr17	58155680	58157125

chr17	58157920	58158228
chr17	58159356	58160324
chr17	58164710	58164777
chr17	58165542	58165685
chr17	58179922	58180306
chr17	58196401	58196449
chr17	58196709	58198259
chr17	58211992	58212935
chr17	58219204	58219823
chr17	58241261	58241460
chr17	58258338	58258529
chr17	58290007	58290564
chr17	58306584	58306783
chr17	58308826	58308858
chr17	58350381	58350880
chr17	58351014	58351403
chr17	58365344	58365414
chr17	58434908	58435372
chr17	58449635	58449903
chr17	58450088	58450951
chr17	58467567	58467617
chr17	58467808	58467916
chr17	58468202	58469684
chr17	58469801	58470779
chr17	58471054	58471308
chr17	58498821	58499267
chr17	58513280	58513367
chr17	58516549	58517140
chr17	58547240	58547407
chr17	58555917	58556150
chr17	58583359	58583969
chr17	58602779	58603640
chr17	58603745	58604120
chr17	58606688	58606887
chr17	58606994	58607626
chr17	58616014	58616235
chr17	58631855	58632083
chr17	58647378	58647516
chr17	58676763	58677439
chr17	58677628	58678579
chr17	58678882	58678887
chr17	58679301	58679722
chr17	58682314	58682645
chr17	58686784	58686861
chr17	58708627	58708672
chr17	58754081	58754998

chr17	58755221	58755698
chr17	58760476	58760839
chr17	58773187	58773302
chr17	58776486	58776686
chr17	58858711	58858955
chr17	58964140	58964921
chr17	59433726	59433766
chr17	59434853	59434925
chr17	59437096	59437182
chr17	59438425	59438797
chr17	59476478	59476959
chr17	59481603	59481893
chr17	59486641	59486818
chr17	59487131	59487368
chr17	59488591	59490434
chr17	59529448	59529975
chr17	59539628	59539857
chr17	59551311	59551342
chr17	59551607	59552089
chr17	59552261	59552505
chr17	59553169	59553260
chr17	59572563	59572724
chr17	59573153	59573292
chr17	59613842	59614000
chr17	59614143	59614169
chr17	59645894	59645904
chr17	59883065	59883117
chr17	59910912	59911340
chr17	59914606	59914847
chr17	59925636	59925805
chr17	59939502	59939955
chr17	59940263	59941487
chr17	59970196	59970379
chr17	60004467	60005253
chr17	60005431	60005567
chr17	60014923	60014971
chr17	60064422	60064614
chr17	60141417	60141633
chr17	60141875	60142662
chr17	60142774	60143420
chr17	60143551	60143705
chr17	60220997	60221448
chr17	60246017	60246127
chr17	60246379	60246802
chr17	60248308	60249016
chr17	60256309	60256364

chr17	60256791	60257049
chr17	60257212	60257454
chr17	60266426	60266591
chr17	60498709	60498819
chr17	60499036	60499195
chr17	60500828	60501740
chr17	60538709	60538892
chr17	60547296	60547720
chr17	60547789	60548212
chr17	60550526	60551236
chr17	60551490	60551626
chr17	60553695	60553835
chr17	60554494	60554822
chr17	60555022	60555933
chr17	60556129	60557639
chr17	60559791	60560013
chr17	60562334	60562445
chr17	60564524	60564908
chr17	60602700	60603094
chr17	60630485	60631096
chr17	60640219	60640381
chr17	60641696	60641857
chr17	60641924	60642234
chr17	60642348	60642391
chr17	60643135	60643383
chr17	60668709	60668840
chr17	60669647	60670006
chr17	60675399	60675598
chr17	60703942	60706078
chr17	60710775	60711046
chr17	60714790	60714848
chr17	60715977	60716142
chr17	60729576	60730180
chr17	60745123	60745817
chr17	60746010	60747573
chr17	60747780	60749830
chr17	60749966	60750411
chr17	60771452	60771786
chr17	60772007	60772206
chr17	60772501	60772778
chr17	60780721	60782213
chr17	60782342	60783642
chr17	60793764	60793832
chr17	60794200	60794641
chr17	60801951	60802629
chr17	60809513	60809730

chr17	60809959	60809983
chr17	60815104	60815216
chr17	60830427	60830440
chr17	60830650	60830688
chr17	60837025	60837179
chr17	60839119	60839155
chr17	60855133	60855458
chr17	60856133	60856495
chr17	60856688	60857441
chr17	60858202	60858640
chr17	60858797	60859354
chr17	60859501	60859753
chr17	60867846	60868425
chr17	60869056	60869508
chr17	60869711	60870699
chr17	60882096	60882122
chr17	60882298	60882543
chr17	60885529	60886013
chr17	60897096	60897694
chr17	60898643	60898937
chr17	60900481	60901411
chr17	60901605	60902333
chr17	60902438	60902836
chr17	60908271	60908531
chr17	60911569	60911790
chr17	60921784	60921806
chr17	60921947	60922157
chr17	60927345	60927743
chr17	60950464	60950891
chr17	60951508	60951709
chr17	60952992	60953226
chr17	60953381	60954246
chr17	60970166	60970800
chr17	60971332	60971533
chr17	60986842	60987041
chr17	60993339	60993538
chr17	61027469	61027546
chr17	61043061	61044623
chr17	61045789	61046206
chr17	61090887	61091105
chr17	61096565	61096903
chr17	61107258	61107275
chr17	61117491	61117877
chr17	61119074	61119168
chr17	61119407	61119477
chr17	61173187	61173192

chr17	61352699	61353127
chr17	61446827	61446877
chr17	61484415	61484453
chr17	61510516	61510604
chr17	61511363	61511614
chr17	61512944	61513330
chr17	61522001	61525335
chr17	61525601	61525796
chr17	61537941	61538276
chr17	61538521	61538768
chr17	61539673	61539917
chr17	61554269	61554607
chr17	61555152	61555464
chr17	61613837	61613989
chr17	61626933	61627575
chr17	61627702	61628892
chr17	61629017	61629533
chr17	61647257	61647456
chr17	61677153	61677476
chr17	61677957	61679125
chr17	61689874	61690014
chr17	61698803	61699310
chr17	61699781	61700165
chr17	61700323	61700842
chr17	61774178	61774377
chr17	61776716	61779816
chr17	61817610	61817813
chr17	61818233	61819180
chr17	61819439	61820660
chr17	61838799	61838813
chr17	61847647	61847913
chr17	61850512	61850972
chr17	61851190	61852204
chr17	61857869	61858335
chr17	61862941	61863305
chr17	61864344	61865134
chr17	61869676	61870062
chr17	61903780	61905282
chr17	61912515	61913249
chr17	61914148	61914441
chr17	61917936	61918215
chr17	61915428	61917658
chr17	61918451	61918966
chr17	61919086	61920335
chr17	61920505	61921536
chr17	61924507	61924706

chr17	61925613	61925621
chr17	61926027	61928077
chr17	61929073	61929255
chr17	61934436	61934477
chr17	61942673	61942905
chr17	61965821	61966020
chr17	61966860	61967022
chr17	62051860	62052291
chr17	62075355	62075554
chr17	62075973	62076172
chr17	62092150	62092361
chr17	62103509	62103561
chr17	62106213	62106271
chr17	62106534	62106653
chr17	62139033	62139190
chr17	62151049	62151460
chr17	62152055	62152281
chr17	62152462	62152720
chr17	62154808	62154924
chr17	62159415	62160214
chr17	62172766	62172880
chr17	62173160	62173213
chr17	62181313	62181533
chr17	62183863	62184067
chr17	62190988	62191207
chr17	62192404	62192805
chr17	62193190	62193334
chr17	62193502	62193674
chr17	62206149	62206554
chr17	62206733	62207510
chr17	62207702	62208597
chr17	62210900	62210971
chr17	62211143	62211326
chr17	62222371	62222669
chr17	62222891	62224971
chr17	62227979	62228039
chr17	62233671	62233849
chr17	62238849	62239085
chr17	62240195	62240394
chr17	62248336	62248436
chr17	62248807	62249219
chr17	62261534	62261721
chr17	62267965	62268157
chr17	62288099	62288454
chr17	62307622	62308289
chr17	62308395	62308998

chr17	62309229	62309501
chr17	62310025	62310298
chr17	62317772	62317971
chr17	62327737	62328012
chr17	62334995	62335170
chr17	62337302	62337385
chr17	62339632	62340608
chr17	62340811	62341486
chr17	62355637	62355773
chr17	62355836	62356096
chr17	62356336	62356370
chr17	62358639	62358757
chr17	62363721	62363972
chr17	62379544	62379607
chr17	62379712	62379956
chr17	62380769	62380812
chr17	62383498	62383697
chr17	62384295	62384324
chr17	62398834	62398853
chr17	62403326	62403959
chr17	62404164	62404548
chr17	62407254	62407392
chr17	62408646	62408887
chr17	62409198	62409689
chr17	62409923	62410287
chr17	62410545	62410681
chr17	62492397	62493923
chr17	62495125	62495346
chr17	62501404	62501894
chr17	62502043	62502632
chr17	62502738	62503929
chr17	62520721	62521012
chr17	62530230	62530315
chr17	62563686	62563885
chr17	62598534	62598863
chr17	62610666	62610988
chr17	62611624	62611669
chr17	62619250	62619310
chr17	62619669	62619855
chr17	62627249	62627451
chr17	62631846	62632107
chr17	62637551	62637603
chr17	62637932	62638191
chr17	62645239	62645606
chr17	62649638	62649913
chr17	62654058	62654257

chr17	62656314	62656632
chr17	62657160	62658796
chr17	62658991	62659430
chr17	62660549	62661036
chr17	62664007	62664736
chr17	62667230	62668050
chr17	62675762	62677521
chr17	62678290	62678586
chr17	62678736	62678995
chr17	62679998	62680190
chr17	62682244	62682443
chr17	62684110	62684453
chr17	62690261	62690428
chr17	62696491	62696531
chr17	62696974	62697283
chr17	62701122	62701266
chr17	62702259	62703314
chr17	62703717	62704047
chr17	62710494	62710700
chr17	62723595	62723794
chr17	62724028	62724238
chr17	62726576	62726601
chr17	62727009	62727213
chr17	62733289	62733473
chr17	62733551	62733710
chr17	62735357	62735672
chr17	62737150	62737387
chr17	62747441	62747674
chr17	62769381	62770363
chr17	62772625	62772997
chr17	62773213	62773444
chr17	62773805	62774934
chr17	62775134	62778577
chr17	62779071	62779179
chr17	62781817	62781874
chr17	62781943	62782650
chr17	62782858	62783099
chr17	62783517	62783824
chr17	62787973	62788253
chr17	62788553	62791746
chr17	62799853	62799944
chr17	62801583	62801793
chr17	62802764	62802767
chr17	62802927	62803982
chr17	62804335	62804741
chr17	62804922	62805280

chr17	62805733	62805992
chr17	62807573	62807610
chr17	62807699	62807772
chr17	62810933	62811168
chr17	62811437	62811722
chr17	62811875	62812360
chr17	62816953	62817165
chr17	62826851	62826961
chr17	62827523	62827878
chr17	62828807	62829133
chr17	62829662	62829890
chr17	62830147	62830869
chr17	62831281	62831956
chr17	62832059	62834338
chr17	62837632	62837924
chr17	62840034	62840237
chr17	62841468	62841726
chr17	62844856	62844916
chr17	62895264	62895496
chr17	62914871	62916880
chr17	62923421	62923778
chr17	62928102	62928604
chr17	62956066	62956149
chr17	62957237	62957616
chr17	62957872	62958035
chr17	62958806	62958973
chr17	62961597	62961708
chr17	62961784	62962475
chr17	62970489	62970812
chr17	62971003	62971661
chr17	62971769	62972486
chr17	62972587	62972756
chr17	62976812	62977110
chr17	62984558	62984984
chr17	62985205	62985592
chr17	62987055	62987116
chr17	62987490	62987844
chr17	62988360	62988450
chr17	62988608	62989081
chr17	62989889	62989980
chr17	63009915	63010367
chr17	63016443	63016533
chr17	63016702	63016793
chr17	63019156	63019374
chr17	63019532	63019564
chr17	63023420	63023824

chr17	63024146	63024345
chr17	63035807	63035959
chr17	63036069	63036194
chr17	63050594	63050875
chr17	63051548	63054121
chr17	63054427	63054898
chr17	63055016	63055213
chr17	63055887	63056156
chr17	63058306	63058996
chr17	63060406	63060527
chr17	63060757	63061257
chr17	63072504	63072678
chr17	63085054	63085253
chr17	63096004	63097330
chr17	63099136	63099168
chr17	63102476	63102714
chr17	63103020	63104069
chr17	63118899	63120046
chr17	63133019	63133810
chr17	63169370	63169402
chr17	63179782	63179973
chr17	63187876	63187914
chr17	63188167	63188785
chr17	63456759	63457494
chr17	63459159	63459392
chr17	63556257	63556936
chr17	63557897	63558167
chr17	63779995	63780346
chr17	63781279	63781624
chr17	63851263	63851494
chr17	64187700	64188482
chr17	64188700	64188706
chr17	64242731	64242802
chr17	64264656	64264748
chr17	64270088	64270114
chr17	64271019	64271222
chr17	64271652	64273435
chr17	64297957	64298470
chr17	64298944	64299633
chr17	64299749	64299939
chr17	64300430	64300544
chr17	64301007	64301273
chr17	64428206	64428374
chr17	64436737	64437426
chr17	64455740	64456456
chr17	64459044	64459786

chr17	64463014	64463252
chr17	64463448	64463632
chr17	64464615	64464899
chr17	64465063	64465257
chr17	64502842	64503041
chr17	64503685	64504045
chr17	64555314	64555513
chr17	64561081	64561221
chr17	64563327	64563497
chr17	64563603	64563757
chr17	64649353	64649720
chr17	64650222	64650242
chr17	64650375	64650421
chr17	64651944	64652067
chr17	64657061	64657371
chr17	64669932	64670041
chr17	64671435	64672731
chr17	64672845	64673053
chr17	64673251	64673514
chr17	64673731	64674021
chr17	64674260	64674385
chr17	64698640	64699158
chr17	64794651	64794960
chr17	64900856	64901292
chr17	64902018	64902225
chr17	64902752	64902901
chr17	64909937	64909974
chr17	64911565	64911901
chr17	64911974	64912089
chr17	64944798	64944807
chr17	64946074	64946085
chr17	64947101	64948276
chr17	64948464	64949715
chr17	64960033	64960276
chr17	64960533	64960753
chr17	64961068	64961536
chr17	64976332	64976481
chr17	64976633	64977028
chr17	65091397	65091788
chr17	65136197	65136440
chr17	65136546	65136759
chr17	65147830	65147916
chr17	65154236	65154295
chr17	65166488	65166567
chr17	65196845	65196933
chr17	65209298	65209554

chr17	65207756	65208549
chr17	65211059	65211162
chr17	65211528	65211721
chr17	65217293	65217700
chr17	65224940	65225217
chr17	65240372	65242891
chr17	65244433	65244828
chr17	65245236	65245466
chr17	65249475	65249836
chr17	65250049	65250405
chr17	65243018	65243878
chr17	65253895	65254533
chr17	65256395	65257187
chr17	65267044	65267369
chr17	65272221	65272240
chr17	65272416	65272693
chr17	65276325	65276907
chr17	65277318	65277547
chr17	65277774	65278355
chr17	65279035	65279332
chr17	65281498	65281885
chr17	65282745	65283284
chr17	65288390	65288616
chr17	65290031	65290305
chr17	65301865	65302475
chr17	65304530	65304668
chr17	65331678	65331804
chr17	65335416	65335571
chr17	65342259	65342478
chr17	65357323	65357353
chr17	65361069	65361269
chr17	65361568	65362720
chr17	65362874	65363069
chr17	65369136	65369824
chr17	65370750	65371193
chr17	65372883	65375457
chr17	65386764	65387227
chr17	65409480	65409810
chr17	65417415	65417450
chr17	65417655	65418074
chr17	65418305	65419149
chr17	65420482	65420963
chr17	65421268	65421444
chr17	65423387	65423663
chr17	65424090	65424315
chr17	65426910	65427143

chr17	65427250	65428186
chr17	65429257	65430375
chr17	65430643	65430877
chr17	65431167	65431641
chr17	65436575	65437202
chr17	65471472	65471900
chr17	65473266	65473441
chr17	65473724	65474039
chr17	65487073	65487147
chr17	65604541	65604924
chr17	65605110	65605278
chr17	65633093	65633309
chr17	65633961	65634130
chr17	65634279	65634547
chr17	65636785	65637071
chr17	65713560	65713781
chr17	65714104	65714924
chr17	65725425	65725474
chr17	65725666	65725924
chr17	65751838	65752057
chr17	65793767	65793966
chr17	65821083	65821162
chr17	65821249	65822664
chr17	65823806	65824181
chr17	65829902	65830007
chr17	65830903	65831115
chr17	65831720	65831788
chr17	65832373	65832419
chr17	65832695	65832723
chr17	65854589	65855056
chr17	65855282	65855607
chr17	65887066	65887324
chr17	65887841	65888229
chr17	65904478	65904543
chr17	65959328	65959517
chr17	65959715	65959725
chr17	65974722	65974770
chr17	65977044	65977390
chr17	65988521	65988720
chr17	65989201	65989680
chr17	65989913	65990557
chr17	65990817	65991177
chr17	65991422	65992291
chr17	65992482	65992967
chr17	65995285	65995881
chr17	66014794	66014941

chr17	66015633	66015875
chr17	66016112	66016891
chr17	66017033	66017446
chr17	66024809	66025111
chr17	66031296	66031491
chr17	66031847	66032561
chr17	66036055	66036254
chr17	66037468	66037851
chr17	66039509	66039514
chr17	66082243	66082442
chr17	66093222	66093787
chr17	66096811	66097014
chr17	66097084	66098876
chr17	66099679	66099687
chr17	66168533	66168569
chr17	66169110	66169446
chr17	66179365	66179389
chr17	66192829	66193233
chr17	66195320	66195613
chr17	66201111	66202604
chr17	66227263	66227536
chr17	66231236	66231675
chr17	66243266	66244892
chr17	66254326	66254401
chr17	66255030	66255196
chr17	66255300	66256074
chr17	66259417	66259872
chr17	66286546	66287138
chr17	66287258	66288630
chr17	66308975	66309335
chr17	66334137	66334207
chr17	66350286	66350541
chr17	66351024	66351050
chr17	66373621	66373878
chr17	66374134	66374910
chr17	66375236	66375616
chr17	66379655	66379974
chr17	66392178	66392374
chr17	66410002	66410438
chr17	66432392	66432986
chr17	66452378	66453699
chr17	66454074	66454661
chr17	66457431	66458000
chr17	66458870	66459105
chr17	66461715	66462226
chr17	66462619	66462621

chr17	66470376	66470471
chr17	66470720	66470896
chr17	66506213	66506225
chr17	66506490	66507022
chr17	66507406	66507489
chr17	66507654	66509646
chr17	66511054	66511300
chr17	66693983	66694536
chr17	66755507	66756157
chr17	66790469	66790546
chr17	66905389	66905721
chr17	67043357	67043525
chr17	67171826	67172259
chr17	67176055	67176082
chr17	67265762	67265979
chr17	67322390	67323267
chr17	67323395	67324013
chr17	67410616	67410681
chr17	67426606	67426701
chr17	67519426	67519463
chr17	67535993	67536004
chr17	67536130	67536589
chr17	67542064	67542135
chr17	67547459	67547658
chr17	67547785	67548129
chr17	67565415	67565823
chr17	67603306	67603322
chr17	67603587	67603676
chr17	67604866	67604948
chr17	67729747	67730263
chr17	67754035	67754413
chr17	67757977	67758598
chr17	67805467	67805857
chr17	67808590	67809059
chr17	67866416	67866572
chr17	67866766	67866914
chr17	68133462	68133893
chr17	68133987	68134337
chr17	68164790	68165311
chr17	68268132	68268283
chr17	68401656	68401673
chr17	68401805	68402217
chr17	68618845	68618883
chr17	68851695	68851784
chr17	69068250	69068431
chr17	69204214	69204295

chr17	69204475	69204959
chr17	69398653	69398725
chr17	69399012	69399152
chr17	69401628	69401709
chr17	69405961	69406336
chr17	69408951	69409432
chr17	69420085	69420370
chr17	69420656	69420717
chr17	69423988	69424078
chr17	69434809	69434933
chr17	69435272	69435398
chr17	69437471	69437638
chr17	69469374	69469382
chr17	69469696	69469727
chr17	69544753	69544931
chr17	69628487	69628912
chr17	69629550	69629695
chr17	69630005	69630165
chr17	69630545	69630744
chr17	69787020	69787064
chr17	69896925	69897072
chr17	69897265	69897391
chr17	69918568	69918657
chr17	69918930	69919027
chr17	69971394	69971402
chr17	69971525	69972251
chr17	70000462	70000533
chr17	70004769	70004804
chr17	70114628	70114849
chr17	70115907	70116250
chr17	70116387	70116602
chr17	70117231	70118523
chr17	70240977	70240982
chr17	70269367	70269961
chr17	70293143	70293202
chr17	70293436	70293578
chr17	70325832	70325915
chr17	70334711	70334834
chr17	70334941	70335152
chr17	70354248	70355071
chr17	70355359	70355519
chr17	70361898	70362252
chr17	70366932	70367089
chr17	70382258	70382422
chr17	70382550	70382762
chr17	70383399	70383776

chr17	70384662	70384940
chr17	70385736	70386025
chr17	70386152	70386205
chr17	70389635	70390120
chr17	70402505	70402718
chr17	70402945	70403135
chr17	70403395	70403510
chr17	70404178	70404377
chr17	70406356	70406642
chr17	70407067	70407381
chr17	70407542	70407800
chr17	70407976	70408333
chr17	70411809	70411975
chr17	70412366	70412646
chr17	70412766	70413163
chr17	70417094	70417333
chr17	70417653	70418149
chr17	70418716	70419686
chr17	70454774	70455025
chr17	70457307	70457677
chr17	70461393	70461694
chr17	70461949	70462276
chr17	70462643	70462776
chr17	70463599	70464427
chr17	70464625	70465494
chr17	70465683	70465932
chr17	70474490	70474681
chr17	70498837	70499583
chr17	70512419	70512698
chr17	70536329	70536565
chr17	70553939	70554138
chr17	70569078	70569355
chr17	70582383	70582500
chr17	70583401	70583841
chr17	70584209	70585936
chr17	70586011	70586397
chr17	70586502	70588070
chr17	70588360	70588908
chr17	70590069	70590153
chr17	70598639	70598960
chr17	70599348	70599757
chr17	70605076	70605275
chr17	70609816	70610208
chr17	70636674	70636919
chr17	70657008	70657456
chr17	70658231	70658377

chr17	70677214	70677339
chr17	70679588	70679773
chr17	70679895	70680094
chr17	70680211	70680571
chr17	70680755	70681076
chr17	70683295	70683665
chr17	70706976	70707138
chr17	70707459	70707721
chr17	70711954	70713041
chr17	70713662	70713686
chr17	70714631	70714848
chr17	70852922	70853069
chr17	70875010	70875216
chr17	70875831	70876031
chr17	71088009	71088242
chr17	71088430	71089126
chr17	71162908	71163108
chr17	71187830	71187955
chr17	71188261	71188972
chr17	71189165	71189991
chr17	71191615	71191689
chr17	71217847	71217925
chr17	71218131	71218157
chr17	71220883	71221553
chr17	71224828	71225120
chr17	71227640	71228456
chr17	71228670	71229315
chr17	71229517	71229746
chr17	71267135	71267375
chr17	71267490	71267621
chr17	71268076	71268464
chr17	71270079	71270118
chr17	71286799	71287023
chr17	71287434	71288990
chr17	71289061	71289460
chr17	71290189	71290528
chr17	71291571	71291717
chr17	71296882	71297120
chr17	71299467	71299621
chr17	71299700	71299772
chr17	71300190	71300239
chr17	71301333	71301792
chr17	71302384	71303107
chr17	71303524	71304360
chr17	71304749	71305760
chr17	71305955	71308132

chr17	71308284	71308776
chr17	71310031	71310194
chr17	71312013	71312017
chr17	71312206	71312433
chr17	71312536	71312711
chr17	71312794	71312975
chr17	71314349	71314350
chr17	71317529	71317836
chr17	71318213	71318427
chr17	71319773	71319900
chr17	71319349	71319707
chr17	71320013	71320852
chr17	71322465	71322727
chr17	71322961	71323057
chr17	71326732	71326916
chr17	71328928	71328953
chr17	71329076	71330038
chr17	71330988	71331370
chr17	71335236	71335596
chr17	71336956	71337192
chr17	71335933	71336716
chr17	71337645	71338762
chr17	71339005	71339416
chr17	71351313	71351721
chr17	71355565	71355855
chr17	71356446	71356708
chr17	71359261	71359488
chr17	71366302	71366371
chr17	71370617	71370816
chr17	71416038	71416067
chr17	71416892	71417373
chr17	71421199	71421253
chr17	71592188	71592387
chr17	71610148	71610780
chr17	71621490	71621595
chr17	71621788	71622065
chr17	71796247	71796437
chr17	72199805	72200551
chr17	72201038	72201388
chr17	72201572	72201770
chr17	72202560	72202612
chr17	72202720	72203400
chr17	72203860	72204034
chr17	72205403	72205602
chr17	72206938	72207202
chr17	72208121	72208788

chr17	72209142	72209505
chr17	72209709	72210310
chr17	72426592	72426901
chr17	72427018	72427912
chr17	72428090	72428289
chr17	72428468	72428715
chr17	72449536	72449760
chr17	72452664	72452729
chr17	72452798	72452863
chr17	72460969	72461152
chr17	72536036	72536195
chr17	72565223	72566327
chr17	72566603	72566898
chr17	72596971	72597170
chr17	72601380	72601594
chr17	72602113	72602436
chr17	72743417	72744689
chr17	72744838	72746085
chr17	72753661	72754059
chr17	72754455	72755064
chr17	72755181	72756947
chr17	72771596	72771950
chr17	72772187	72773318
chr17	72778208	72778228
chr17	72778884	72779083
chr17	72838862	72839392
chr17	72868407	72869102
chr17	72867788	72868137
chr17	72869614	72869995
chr17	72870124	72870784
chr17	72873005	72873296
chr17	72873515	72873755
chr17	72894865	72894907
chr17	72895098	72895229
chr17	72918826	72919059
chr17	72919186	72920552
chr17	72938415	72939008
chr17	72939353	72940322
chr17	72940451	72940827
chr17	72941884	72942362
chr17	72942445	72942483
chr17	72946598	72946989
chr17	72953786	72954077
chr17	72957127	72957753
chr17	72962691	72962847
chr17	72967880	72969078

chr17	72977445	72979143
chr17	72981879	72982346
chr17	72983078	72983498
chr17	72983636	72984974
chr17	72985613	72985647
chr17	72986943	72987255
chr17	72987705	72987881
chr17	72987996	72988396
chr17	72988576	72989008
chr17	72989339	72990140
chr17	72990609	72990689
chr17	72990817	72991000
chr17	72994368	72994768
chr17	73007922	73009548
chr17	73009922	73010158
chr17	73028960	73029322
chr17	73029535	73029874
chr17	73030104	73032094
chr17	73032358	73032794
chr17	73042061	73043502
chr17	73043627	73044302
chr17	73044514	73044708
chr17	73044826	73044947
chr17	73046141	73046186
chr17	73049564	73049668
chr17	73051598	73051734
chr17	73065329	73065621
chr17	73065753	73065836
chr17	73066179	73066283
chr17	73072453	73072532
chr17	73074555	73075454
chr17	73075622	73076213
chr17	73077219	73077532
chr17	73079083	73079347
chr17	73079479	73080126
chr17	73081086	73081749
chr17	73081985	73082062
chr17	73082357	73085580
chr17	73085769	73087351
chr17	73087514	73087877
chr17	73088203	73090088
chr17	73092921	73093636
chr17	73090203	73092742
chr17	73094345	73094683
chr17	73098809	73099226
chr17	73105161	73105808

chr17	73106403	73107022
chr17	73109116	73109412
chr17	73110420	73110742
chr17	73111366	73111781
chr17	73116083	73116283
chr17	73118824	73119693
chr17	73121939	73122154
chr17	73119970	73121876
chr17	73122704	73123140
chr17	73123462	73124135
chr17	73126989	73128605
chr17	73128715	73129169
chr17	73129243	73129396
chr17	73129565	73130783
chr17	73130902	73131058
chr17	73131405	73131714
chr17	73133396	73134271
chr17	73136000	73136267
chr17	73138025	73138118
chr17	73138488	73138687
chr17	73140132	73140155
chr17	73141935	73142335
chr17	73142806	73143010
chr17	73143795	73143905
chr17	73144331	73144586
chr17	73148911	73149950
chr17	73150055	73150574
chr17	73150929	73151330
chr17	73151457	73152258
chr17	73152629	73153307
chr17	73155127	73156123
chr17	73154233	73154886
chr17	73157422	73157662
chr17	73157791	73157822
chr17	73167162	73167387
chr17	73168237	73168297
chr17	73169428	73169529
chr17	73169635	73169819
chr17	73177950	73179898
chr17	73185222	73186005
chr17	73187911	73187958
chr17	73191329	73191540
chr17	73194841	73195040
chr17	73197270	73197326
chr17	73198017	73198227
chr17	73200592	73202182

chr17	73202454	73202898
chr17	73213175	73213401
chr17	73229912	73230114
chr17	73231328	73231802
chr17	73246110	73246315
chr17	73246516	73246797
chr17	73246887	73247165
chr17	73248088	73248184
chr17	73255162	73255325
chr17	73255747	73255769
chr17	73255942	73256180
chr17	73257052	73258654
chr17	73258822	73259021
chr17	73261352	73261412
chr17	73265611	73265947
chr17	73266715	73267925
chr17	73268160	73268492
chr17	73269791	73269845
chr17	73276285	73276484
chr17	73283974	73284019
chr17	73284323	73284553
chr17	73284804	73285229
chr17	73285403	73286863
chr17	73288884	73289097
chr17	73290691	73290890
chr17	73305894	73306303
chr17	73313199	73313232
chr17	73313417	73313662
chr17	73319477	73319657
chr17	73320011	73320179
chr17	73337265	73337456
chr17	73363414	73363790
chr17	73364496	73364559
chr17	73369117	73369347
chr17	73389529	73389978
chr17	73390169	73391130
chr17	73391817	73392231
chr17	73400787	73400960
chr17	73400190	73400622
chr17	73401155	73401769
chr17	73402030	73402992
chr17	73406240	73406363
chr17	73418334	73418594
chr17	73418872	73419166
chr17	73422742	73422881
chr17	73435468	73435710

chr17	73438247	73438375
chr17	73438625	73438882
chr17	73452162	73453720
chr17	73507053	73507306
chr17	73508823	73509056
chr17	73510610	73512511
chr17	73512615	73513991
chr17	73521141	73522398
chr17	73522979	73523178
chr17	73525065	73525260
chr17	73526635	73526892
chr17	73527733	73527932
chr17	73528148	73528648
chr17	73544421	73544680
chr17	73545223	73546176
chr17	73548323	73548547
chr17	73549971	73550121
chr17	73572569	73572768
chr17	73583937	73586151
chr17	73589920	73590119
chr17	73619467	73619685
chr17	73639221	73639553
chr17	73639977	73640176
chr17	73641917	73642252
chr17	73642367	73642684
chr17	73649709	73649872
chr17	73650260	73650402
chr17	73660748	73660925
chr17	73662836	73663347
chr17	73663462	73663853
chr17	73670849	73671516
chr17	73675700	73676226
chr17	73676613	73677031
chr17	73677342	73677916
chr17	73679676	73682072
chr17	73682481	73682844
chr17	73683563	73683802
chr17	73684050	73686032
chr17	73686440	73686993
chr17	73687486	73687747
chr17	73688083	73688653
chr17	73688913	73690385
chr17	73690646	73690868
chr17	73691163	73692025
chr17	73692260	73692392
chr17	73692937	73693797

chr17	73693901	73694036
chr17	73694434	73694993
chr17	73695504	73695897
chr17	73695981	73698689
chr17	73698832	73699103
chr17	73702343	73702752
chr17	73702936	73703234
chr17	73703396	73703618
chr17	73706141	73707064
chr17	73707450	73707759
chr17	73708981	73709229
chr17	73709782	73710001
chr17	73715747	73716079
chr17	73716302	73721347
chr17	73721527	73722329
chr17	73722402	73722783
chr17	73722941	73723086
chr17	73724247	73725197
chr17	73728861	73729188
chr17	73730438	73730638
chr17	73731566	73732059
chr17	73733811	73734045
chr17	73734894	73735352
chr17	73739378	73739688
chr17	73740784	73740945
chr17	73742111	73742555
chr17	73742715	73742929
chr17	73743699	73744403
chr17	73745241	73745440
chr17	73751432	73751631
chr17	73751797	73752223
chr17	73754067	73754266
chr17	73755026	73755656
chr17	73755772	73757158
chr17	73757473	73757762
chr17	73758062	73758441
chr17	73760245	73761924
chr17	73766483	73766631
chr17	73771758	73772177
chr17	73772541	73772740
chr17	73773080	73773287
chr17	73774052	73774080
chr17	73774344	73776063
chr17	73776274	73776358
chr17	73776844	73777606
chr17	73779199	73779806

chr17	73780007	73780622
chr17	73780836	73781825
chr17	73783308	73783695
chr17	73792506	73792726
chr17	73801467	73801928
chr17	73806913	73807125
chr17	73810361	73811039
chr17	73811208	73811792
chr17	73823609	73823813
chr17	73828151	73828356
chr17	73828477	73828713
chr17	73835195	73835645
chr17	73839621	73839977
chr17	73840086	73842645
chr17	73846869	73847068
chr17	73848602	73848802
chr17	73850042	73851459
chr17	73851706	73852795
chr17	73854497	73854708
chr17	73855562	73855650
chr17	73858296	73858495
chr17	73860297	73860366
chr17	73861025	73861442
chr17	73864859	73865569
chr17	73865711	73865910
chr17	73866264	73866557
chr17	73867770	73868954
chr17	73869140	73869702
chr17	73870039	73870473
chr17	73871640	73875477
chr17	73878491	73878606
chr17	73880887	73880898
chr17	73884361	73884424
chr17	73890225	73890442
chr17	73891638	73893308
chr17	73898403	73898486
chr17	73898261	73898331
chr17	73899095	73899112
chr17	73899228	73899310
chr17	73899792	73900218
chr17	73900422	73900822
chr17	73900957	73902015
chr17	73902182	73902400
chr17	73902888	73903126
chr17	73904248	73904281
chr17	73935893	73936152

chr17	73936258	73937146
chr17	73974752	73976482
chr17	73976743	73976942
chr17	73977737	73978070
chr17	73989805	73989906
chr17	73992289	73992907
chr17	73994297	73994573
chr17	74002322	74002571
chr17	74002652	74002664
chr17	74009237	74009436
chr17	74012823	74013096
chr17	74024066	74024160
chr17	74021911	74023961
chr17	74026159	74026163
chr17	74027399	74028240
chr17	74047247	74047404
chr17	74047509	74047515
chr17	74048342	74048843
chr17	74049091	74049352
chr17	74049642	74050661
chr17	74050891	74051321
chr17	74051498	74051717
chr17	74052675	74052827
chr17	74060663	74060991
chr17	74066060	74066084
chr17	74066414	74066421
chr17	74067953	74069349
chr17	74098371	74098404
chr17	74098557	74098645
chr17	74098983	74100551
chr17	74100864	74100870
chr17	74117263	74118471
chr17	74129076	74129821
chr17	74137798	74137997
chr17	74176207	74176345
chr17	74177999	74178364
chr17	74236799	74237118
chr17	74237229	74237510
chr17	74244432	74244648
chr17	74247522	74247678
chr17	74248991	74249191
chr17	74256248	74256447
chr17	74258312	74258760
chr17	74259232	74259465
chr17	74259929	74260376
chr17	74260538	74260887

chr17	74261592	74262838
chr17	74263090	74263567
chr17	74263777	74264332
chr17	74264616	74265408
chr17	74279998	74280261
chr17	74295569	74295749
chr17	74301336	74301538
chr17	74346258	74346462
chr17	74346825	74347049
chr17	74349032	74350162
chr17	74350351	74351092
chr17	74352773	74353388
chr17	74363030	74363229
chr17	74366534	74366874
chr17	74367441	74367771
chr17	74367014	74367147
chr17	74368030	74368079
chr17	74368473	74368744
chr17	74378423	74382638
chr17	74384830	74385029
chr17	74390168	74390550
chr17	74390779	74390862
chr17	74439970	74440283
chr17	74442595	74443052
chr17	74443313	74443507
chr17	74443986	74444048
chr17	74448481	74449434
chr17	74456894	74457112
chr17	74457722	74457783
chr17	74461926	74462313
chr17	74479540	74479797
chr17	74479999	74480130
chr17	74480646	74481487
chr17	74481558	74481755
chr17	74482218	74482852
chr17	74485403	74485581
chr17	74488846	74489279
chr17	74489407	74489906
chr17	74490024	74490310
chr17	74493808	74495395
chr17	74495506	74498390
chr17	74501977	74502190
chr17	74505159	74505279
chr17	74512383	74512442
chr17	74512674	74512873
chr17	74516698	74516980

chr17	74518650	74518961
chr17	74519115	74519121
chr17	74519610	74520003
chr17	74520596	74520711
chr17	74520839	74521038
chr17	74521408	74521796
chr17	74522206	74527613
chr17	74527719	74529282
chr17	74529442	74529945
chr17	74530318	74531842
chr17	74532202	74534950
chr17	74535374	74535714
chr17	74536432	74536972
chr17	74537278	74537558
chr17	74541974	74542183
chr17	74542415	74542776
chr17	74543137	74543773
chr17	74544679	74545133
chr17	74546368	74546998
chr17	74547398	74547549
chr17	74549741	74550016
chr17	74551354	74551396
chr17	74551601	74552031
chr17	74552801	74554473
chr17	74554540	74554816
chr17	74556792	74557154
chr17	74558065	74558341
chr17	74561638	74561837
chr17	74573265	74573413
chr17	74573532	74573631
chr17	74574973	74575183
chr17	74575330	74575389
chr17	74575689	74576668
chr17	74580462	74582660
chr17	74576972	74580328
chr17	74582946	74583461
chr17	74589214	74589358
chr17	74589772	74589971
chr17	74590099	74590568
chr17	74593309	74594079
chr17	74594875	74595000
chr17	74596577	74596950
chr17	74597915	74598165
chr17	74598273	74598408
chr17	74599485	74599605
chr17	74607718	74607847

chr17	74608314	74608375
chr17	74617410	74617514
chr17	74619936	74620615
chr17	74622731	74623249
chr17	74625739	74625931
chr17	74631454	74631763
chr17	74631917	74632072
chr17	74637571	74637786
chr17	74637895	74638072
chr17	74639649	74639662
chr17	74655670	74655878
chr17	74656364	74657046
chr17	74662742	74663455
chr17	74663963	74664405
chr17	74667030	74667738
chr17	74667988	74668521
chr17	74680503	74680702
chr17	74685060	74685484
chr17	74685745	74686061
chr17	74691966	74692241
chr17	74694611	74694631
chr17	74696303	74697396
chr17	74705144	74706092
chr17	74706204	74706863
chr17	74707133	74707619
chr17	74710964	74711279
chr17	74712896	74712953
chr17	74713250	74713287
chr17	74720233	74720633
chr17	74721715	74722756
chr17	74722979	74723855
chr17	74725159	74725210
chr17	74727228	74727551
chr17	74728097	74728502
chr17	74729801	74729909
chr17	74730340	74730753
chr17	74731145	74731404
chr17	74732072	74733429
chr17	74733634	74734536
chr17	74734692	74734996
chr17	74747520	74747724
chr17	74749710	74750181
chr17	74768792	74769045
chr17	74787473	74787573
chr17	74835224	74835920
chr17	74836695	74836894

chr17	74864297	74864681
chr17	74868961	74869212
chr17	75042185	75042321
chr17	75082604	75082949
chr17	75084306	75085567
chr17	75086785	75086897
chr17	75092097	75092478
chr17	75095010	75095184
chr17	75100245	75100478
chr17	75102437	75102450
chr17	75113222	75113472
chr17	75114593	75115040
chr17	75115281	75115422
chr17	75118140	75118243
chr17	75118516	75119388
chr17	75119900	75120106
chr17	75121441	75122142
chr17	75120171	75120954
chr17	75122711	75122910
chr17	75127888	75128017
chr17	75128167	75128754
chr17	75128879	75129561
chr17	75129689	75129884
chr17	75130511	75130599
chr17	75134229	75134579
chr17	75135152	75135564
chr17	75135849	75135926
chr17	75138278	75138477
chr17	75136233	75137936
chr17	75142323	75142333
chr17	75150147	75150445
chr17	75183845	75184060
chr17	75202803	75202995
chr17	75206500	75206588
chr17	75229693	75229892
chr17	75230057	75230757
chr17	75243023	75243500
chr17	75248246	75248369
chr17	75249531	75252605
chr17	75257296	75257389
chr17	75260285	75260690
chr17	75265723	75265778
chr17	75267180	75267409
chr17	75274320	75274890
chr17	75275553	75277314
chr17	75277536	75278445

chr17	75278757	75279219
chr17	75286062	75286307
chr17	75286568	75286884
chr17	75287438	75288516
chr17	75288712	75289021
chr17	75289243	75289541
chr17	75289963	75290290
chr17	75279440	75285647
chr17	75298346	75298704
chr17	75298784	75298842
chr17	75299413	75299468
chr17	75299542	75299912
chr17	75302302	75302961
chr17	75305952	75306222
chr17	75310037	75310236
chr17	75313620	75313773
chr17	75314552	75314785
chr17	75315135	75316545
chr17	75316683	75317142
chr17	75317466	75317932
chr17	75318661	75319899
chr17	75320873	75321257
chr17	75320365	75320775
chr17	75321549	75321884
chr17	75323127	75323326
chr17	75324488	75324687
chr17	75326309	75326528
chr17	75327102	75327423
chr17	75327809	75327952
chr17	75328974	75329357
chr17	75337735	75337929
chr17	75341725	75341771
chr17	75346116	75346315
chr17	75352269	75352468
chr17	75352805	75353242
chr17	75353576	75353907
chr17	75355880	75355950
chr17	75358418	75358700
chr17	75359598	75359607
chr17	75361403	75362013
chr17	75387820	75388019
chr17	75400264	75400637
chr17	75401595	75401822
chr17	75403106	75403450
chr17	75407033	75408097
chr17	75408551	75408846

chr17	75410239	75410511
chr17	75416440	75420593
chr17	75420825	75423216
chr17	75423334	75424372
chr17	75427129	75427199
chr17	75427912	75427950
chr17	75444918	75445140
chr17	75445434	75445633
chr17	75446100	75448763
chr17	75449388	75451557
chr17	75455620	75455819
chr17	75457126	75457290
chr17	75458308	75458677
chr17	75460948	75461001
chr17	75461116	75461149
chr17	75461264	75461315
chr17	75461498	75461875
chr17	75462058	75462340
chr17	75462535	75462797
chr17	75463952	75464373
chr17	75466672	75467189
chr17	75467413	75469237
chr17	75469885	75470755
chr17	75470930	75471183
chr17	75471328	75472027
chr17	75472698	75473616
chr17	75473751	75474119
chr17	75474571	75474669
chr17	75480523	75481129
chr17	75489823	75490184
chr17	75561016	75561215
chr17	75597732	75598623
chr17	75597346	75597646
chr17	75598921	75599422
chr17	75599724	75599925
chr17	75612702	75613145
chr17	75832380	75832694
chr17	75833046	75833290
chr17	75953641	75954476
chr17	75954702	75954995
chr17	75955322	75955540
chr17	76030690	76030907
chr17	76036824	76037571
chr17	76122740	76122979
chr17	76123119	76125289
chr17	76127442	76128796

chr17	76135545	76135888
chr17	76135957	76136684
chr17	76136868	76138207
chr17	76138432	76139368
chr17	76140433	76140921
chr17	76144215	76144412
chr17	76145824	76145833
chr17	76147096	76147508
chr17	76148133	76148403
chr17	76150047	76150735
chr17	76151165	76151744
chr17	76152455	76152494
chr17	76152626	76152697
chr17	76153161	76153572
chr17	76154904	76156355
chr17	76156604	76157414
chr17	76157584	76159282
chr17	76161646	76161983
chr17	76162924	76163691
chr17	76163909	76165436
chr17	76166476	76166675
chr17	76174830	76174866
chr17	76175346	76175592
chr17	76175772	76176212
chr17	76177807	76178284
chr17	76181304	76181693
chr17	76182535	76184193
chr17	76184457	76184807
chr17	76185775	76185881
chr17	76191949	76192685
chr17	76193973	76194424
chr17	76194794	76195133
chr17	76195281	76195568
chr17	76195722	76196025
chr17	76209810	76210252
chr17	76210392	76210790
chr17	76211973	76212200
chr17	76223936	76224135
chr17	76247291	76247490
chr17	76249762	76249769
chr17	76249929	76250949
chr17	76268935	76269349
chr17	76278175	76278575
chr17	76285427	76286435
chr17	76289844	76290210
chr17	76291112	76291453

chr17	76293209	76293347
chr17	76294454	76294653
chr17	76294985	76295181
chr17	76295474	76297569
chr17	76299253	76299360
chr17	76304654	76305150
chr17	76307166	76307367
chr17	76308216	76308441
chr17	76309430	76313652
chr17	76313798	76314163
chr17	76317061	76317270
chr17	76319699	76319898
chr17	76321012	76321211
chr17	76321524	76322005
chr17	76322491	76322559
chr17	76322637	76322711
chr17	76322819	76323143
chr17	76323993	76324915
chr17	76325264	76326291
chr17	76326424	76326432
chr17	76327036	76327350
chr17	76327545	76328079
chr17	76328925	76329066
chr17	76330367	76331276
chr17	76331487	76331588
chr17	76332356	76332768
chr17	76333203	76334174
chr17	76334516	76335126
chr17	76335273	76335387
chr17	76335635	76336235
chr17	76337095	76338755
chr17	76338971	76339622
chr17	76339836	76340423
chr17	76343104	76343187
chr17	76343309	76343462
chr17	76345293	76345562
chr17	76345774	76345926
chr17	76346782	76347221
chr17	76347385	76347652
chr17	76347908	76349681
chr17	76349818	76350836
chr17	76351266	76351473
chr17	76351701	76351730
chr17	76352234	76352649
chr17	76352875	76352949
chr17	76353023	76353599

chr17	76353900	76354399
chr17	76354517	76357020
chr17	76367520	76367972
chr17	76370868	76372339
chr17	76372753	76375578
chr17	76380551	76380618
chr17	76380705	76381069
chr17	76381771	76381902
chr17	76382070	76382367
chr17	76385282	76385410
chr17	76386366	76386433
chr17	76386919	76387043
chr17	76387485	76388147
chr17	76388318	76388359
chr17	76389258	76389379
chr17	76395562	76395813
chr17	76396111	76396448
chr17	76397234	76397544
chr17	76403812	76404069
chr17	76406195	76406360
chr17	76408789	76409044
chr17	76409520	76409719
chr17	76418691	76419203
chr17	76473408	76473602
chr17	76473675	76473735
chr17	76479676	76479968
chr17	76480215	76481654
chr17	76493495	76493787
chr17	76496171	76496370
chr17	76501714	76501907
chr17	76528855	76529915
chr17	76531160	76531566
chr17	76532034	76532319
chr17	76532620	76533129
chr17	76533353	76533663
chr17	76533812	76533871
chr17	76534098	76534555
chr17	76535124	76535323
chr17	76537543	76537919
chr17	76538060	76538829
chr17	76539158	76539460
chr17	76564626	76565080
chr17	76586654	76587063
chr17	76588205	76588593
chr17	76588856	76589289
chr17	76594821	76594973

chr17	76602510	76602577
chr17	76627562	76628194
chr17	76628392	76628615
chr17	76656903	76657879
chr17	76658227	76658382
chr17	76669272	76669366
chr17	76676024	76676205
chr17	76676688	76676921
chr17	76677151	76677869
chr17	76696715	76696909
chr17	76700709	76701046
chr17	76702260	76702811
chr17	76703190	76703456
chr17	76704552	76704578
chr17	76713706	76714463
chr17	76719330	76719475
chr17	76731070	76731788
chr17	76731923	76732926
chr17	76733045	76733233
chr17	76733453	76733723
chr17	76738535	76738745
chr17	76741555	76741919
chr17	76744848	76744986
chr17	76745148	76745268
chr17	76768910	76768970
chr17	76769124	76769223
chr17	76770644	76770836
chr17	76771127	76771347
chr17	76772035	76772264
chr17	76773272	76773486
chr17	76776069	76776736
chr17	76776830	76777189
chr17	76777331	76778307
chr17	76778627	76779932
chr17	76781014	76781276
chr17	76783797	76784022
chr17	76784358	76784387
chr17	76786157	76786347
chr17	76786826	76786874
chr17	76787126	76787158
chr17	76787302	76787543
chr17	76787976	76788003
chr17	76788076	76788254
chr17	76794775	76794974
chr17	76813456	76813693
chr17	76823813	76824126

chr17	76833892	76834058
chr17	76835218	76835610
chr17	76835746	76836960
chr17	76837153	76837779
chr17	76838048	76838175
chr17	76838422	76838454
chr17	76855199	76855258
chr17	76855363	76855635
chr17	76856139	76856404
chr17	76858017	76858764
chr17	76861779	76861915
chr17	76862842	76863043
chr17	76863350	76863470
chr17	76864448	76864772
chr17	76865906	76865919
chr17	76869466	76869665
chr17	76870883	76871226
chr17	76872456	76872722
chr17	76873521	76873630
chr17	76876538	76876755
chr17	76878745	76880870
chr17	76882957	76882977
chr17	76883426	76883439
chr17	76894844	76895043
chr17	76896525	76896618
chr17	76907429	76907432
chr17	76907656	76908057
chr17	76910836	76911149
chr17	76911548	76911960
chr17	76912259	76912722
chr17	76913044	76913277
chr17	76914710	76915200
chr17	76919929	76921146
chr17	76921295	76922270
chr17	76922761	76922937
chr17	76928993	76931173
chr17	76931816	76932720
chr17	76934454	76934718
chr17	76954469	76954794
chr17	76956537	76956639
chr17	76958795	76958999
chr17	76967132	76967501
chr17	76967862	76968061
chr17	76969920	76970211
chr17	76970437	76970783
chr17	76971635	76971939

chr17	76973709	76973856
chr17	76974193	76974451
chr17	76974736	76976920
chr17	76981781	76982121
chr17	76982325	76982460
chr17	76982704	76983333
chr17	76983730	76984206
chr17	76984633	76984929
chr17	77000773	77001370
chr17	77004417	77006465
chr17	77006925	77007349
chr17	77015657	77016009
chr17	77020333	77021000
chr17	77022237	77023924
chr17	77024170	77024185
chr17	77027182	77027429
chr17	77031725	77031842
chr17	77032130	77033303
chr17	77033773	77034042
chr17	77034262	77035357
chr17	77036399	77037128
chr17	77037236	77037355
chr17	77037476	77037844
chr17	77056081	77056280
chr17	77067066	77067431
chr17	77070174	77070672
chr17	77070945	77071676
chr17	77072412	77072694
chr17	77073029	77073284
chr17	77086107	77086830
chr17	77221455	77221710
chr17	77223353	77224873
chr17	77225023	77225221
chr17	77225355	77225671
chr17	77227912	77228113
chr17	77236774	77236866
chr17	77268334	77268691
chr17	77269265	77269554
chr17	77355388	77355587
chr17	77638345	77638640
chr17	77638961	77639171
chr17	77639573	77639824
chr17	77702001	77702421
chr17	77719939	77720036
chr17	77733733	77733878
chr17	77751318	77751613

chr17	77751961	77752591
chr17	77770369	77770991
chr17	77771207	77771507
chr17	77771702	77772182
chr17	77772349	77772548
chr17	77774585	77774898
chr17	77775016	77775332
chr17	77776374	77776997
chr17	77777122	77777321
chr17	77777947	77778464
chr17	77786227	77786426
chr17	77786913	77787209
chr17	77787549	77787795
chr17	77788030	77789656
chr17	77801785	77801984
chr17	77810244	77810443
chr17	77810609	77811145
chr17	77811273	77811947
chr17	77812076	77813076
chr17	77813298	77814622
chr17	77816229	77816529
chr17	77817034	77817532
chr17	77817761	77818358
chr17	77823758	77824451
chr17	77826480	77827248
chr17	77827384	77827661
chr17	77824704	77825705
chr17	77827945	77828144
chr17	77830802	77831001
chr17	77838299	77838870
chr17	77840488	77840678
chr17	77863681	77863894
chr17	77876417	77876639
chr17	77889433	77889644
chr17	77893025	77893509
chr17	77921566	77921988
chr17	77943738	77943935
chr17	77961070	77961665
chr17	77961892	77962346
chr17	77965078	77965426
chr17	77965748	77967419
chr17	77967529	77968183
chr17	77971625	77971816
chr17	77972102	77972216
chr17	77979923	77980150
chr17	77993747	77993800

chr17	77996572	77996801
chr17	77998238	77998461
chr17	78008898	78009509
chr17	78009762	78010865
chr17	78033562	78033728
chr17	78074878	78076248
chr17	78082869	78083440
chr17	78083663	78084085
chr17	78119831	78120136
chr17	78120301	78120993
chr17	78121170	78122102
chr17	78122575	78122827
chr17	78122901	78122963
chr17	78129706	78129817
chr17	78149111	78149305
chr17	78149454	78149901
chr17	78160389	78161934
chr17	78162517	78162829
chr17	78166591	78166604
chr17	78166800	78166828
chr17	78192793	78192877
chr17	78193391	78194701
chr17	78218413	78218610
chr17	78218676	78218746
chr17	78226944	78227110
chr17	78228222	78229537
chr17	78229647	78230243
chr17	78232684	78232883
chr17	78233052	78234335
chr17	78235715	78236404
chr17	78234609	78235610
chr17	78236528	78236734
chr17	78240887	78241155
chr17	78248690	78248951
chr17	78257894	78258072
chr17	78388710	78389775
chr17	78427852	78430013
chr17	78448725	78448956
chr17	78449525	78450136
chr17	78450439	78450666
chr17	78451008	78451840
chr17	78451989	78452485
chr17	78474740	78475833
chr17	78476129	78477020
chr17	78489622	78489712
chr17	78500687	78500910

chr17	78501059	78501273
chr17	78503482	78504013
chr17	78518375	78518423
chr17	78518661	78519331
chr17	78522467	78522578
chr17	78549335	78549579
chr17	78573135	78573175
chr17	78573243	78573334
chr17	78593597	78594088
chr17	78622809	78623814
chr17	78625552	78625756
chr17	78651363	78651432
chr17	78652101	78652385
chr17	78652565	78653344
chr17	78653540	78653793
chr17	78667399	78667605
chr17	78667749	78668154
chr17	78668309	78668777
chr17	78669772	78669871
chr17	78678183	78678586
chr17	78679587	78679720
chr17	78694495	78695538
chr17	78696598	78696890
chr17	78698163	78699128
chr17	78699467	78699903
chr17	78723658	78723791
chr17	78733734	78734075
chr17	78734198	78734533
chr17	78734686	78734817
chr17	78735120	78735804
chr17	78736052	78736389
chr17	78763499	78763670
chr17	78764043	78764607
chr17	78765629	78765917
chr17	78766097	78766583
chr17	78798527	78798562
chr17	78799329	78799694
chr17	78799961	78801661
chr17	78806355	78807012
chr17	78807166	78807691
chr17	78823317	78823542
chr17	78824071	78825147
chr17	78826042	78826068
chr17	78826134	78826556
chr17	78827094	78827885
chr17	78828311	78828453

chr17	78829567	78829702
chr17	78830150	78831308
chr17	78832484	78832566
chr17	78832689	78834023
chr17	78834940	78836721
chr17	78840328	78840360
chr17	78864029	78864230
chr17	78864358	78864957
chr17	78865162	78865890
chr17	78866088	78867439
chr17	78874333	78875788
chr17	78932369	78932616
chr17	78941769	78941968
chr17	78954702	78955281
chr17	78958427	78958516
chr17	78962424	78962716
chr17	78964686	78966559
chr17	78966843	78967030
chr17	78992700	78992779
chr17	78992893	78993039
chr17	78997822	78998026
chr17	79004213	79004412
chr17	79007879	79008100
chr17	79008203	79008697
chr17	79009243	79010476
chr17	79011010	79011342
chr17	79013765	79015124
chr17	79015292	79015400
chr17	79015837	79016374
chr17	79016555	79018837
chr17	79018993	79019235
chr17	79019437	79019937
chr17	79021787	79022146
chr17	79022832	79024087
chr17	79024207	79024643
chr17	79024806	79025839
chr17	79030647	79031981
chr17	79033308	79033651
chr17	79035786	79035961
chr17	79037190	79037324
chr17	79050566	79050768
chr17	79060401	79061220
chr17	79061724	79061943
chr17	79063582	79064847
chr17	79065123	79065486
chr17	79066696	79067161

chr17	79067879	79069515
chr17	79070482	79071932
chr17	79072331	79072644
chr17	79092703	79093466
chr17	79158951	79159150
chr17	79192808	79193285
chr17	79194709	79194757
chr17	79196364	79196880
chr17	79197150	79197615
chr17	79198843	79198941
chr17	79199043	79199138
chr17	79209965	79210182
chr17	79212009	79212844
chr17	79213084	79214435
chr17	79226903	79226947
chr17	79228316	79228383
chr17	79228481	79228742
chr17	79229424	79229741
chr17	79245520	79245541
chr17	79267136	79267462
chr17	79268230	79270419
chr17	79275504	79276163
chr17	79279798	79280031
chr17	79282571	79283338
chr17	79283552	79284507
chr17	79295426	79295446
chr17	79297223	79297922
chr17	79299828	79300849
chr17	79301041	79301923
chr17	79302044	79303475
chr17	79303646	79304464
chr17	79314824	79316290
chr17	79316425	79320021
chr17	79320232	79320635
chr17	79323858	79324058
chr17	79324929	79325208
chr17	79336205	79336305
chr17	79339295	79339611
chr17	79339807	79340210
chr17	79343965	79344370
chr17	79348187	79348231
chr17	79360425	79361731
chr17	79366767	79367815
chr17	79368475	79368933
chr17	79369801	79371032
chr17	79371281	79371582

chr17	79371725	79374413
chr17	79386307	79387108
chr17	79393215	79394325
chr17	79395798	79396586
chr17	79396778	79397855
chr17	79398656	79399434
chr17	79448118	79448548
chr17	79451380	79452214
chr17	79452955	79453204
chr17	79454115	79455750
chr17	79464463	79464777
chr17	79476945	79477166
chr17	79477341	79478069
chr17	79478274	79478617
chr17	79478755	79482728
chr17	79482923	79483123
chr17	79486096	79486734
chr17	79486915	79487471
chr17	79488258	79488649
chr17	79518634	79519934
chr17	79520189	79521663
chr17	79527276	79527652
chr17	79527831	79528263
chr17	79528819	79528871
chr17	79529070	79529387
chr17	79529748	79529895
chr17	79530894	79531618
chr17	79531750	79532044
chr17	79533397	79533584
chr17	79533688	79534905
chr17	79535124	79535212
chr17	79543241	79543345
chr17	79544678	79544886
chr17	79545385	79545698
chr17	79545907	79546245
chr17	79558239	79558244
chr17	79559751	79559886
chr17	79568504	79568663
chr17	79583043	79583242
chr17	79586235	79586372
chr17	79602442	79602945
chr17	79603291	79604436
chr17	79607040	79607176
chr17	79608248	79608504
chr17	79608798	79608804
chr17	79608993	79609785

chr17	79610046	79610182
chr17	79609965	79609982
chr17	79625259	79625420
chr17	79626384	79626687
chr17	79627446	79627833
chr17	79626799	79627145
chr17	79630897	79631190
chr17	79631530	79631648
chr17	79632427	79635149
chr17	79635685	79635721
chr17	79635987	79636337
chr17	79637998	79638193
chr17	79650005	79650276
chr17	79650526	79652134
chr17	79652251	79652555
chr17	79652921	79653266
chr17	79667366	79667466
chr17	79668886	79671082
chr17	79671175	79671578
chr17	79678405	79680293
chr17	79685346	79685807
chr17	79688832	79690516
chr17	79690636	79690768
chr17	79691003	79691197
chr17	79694854	79695131
chr17	79698504	79698917
chr17	79702301	79702540
chr17	79702679	79702882
chr17	79703054	79703731
chr17	79773821	79774020
chr17	79788758	79788840
chr17	79790085	79790974
chr17	79791120	79792180
chr17	79797683	79797907
chr17	79798015	79800494
chr17	79805554	79805768
chr17	79806606	79806880
chr17	79808113	79808316
chr17	79813672	79813910
chr17	79815001	79815014
chr17	79815583	79815602
chr17	79815760	79816151
chr17	79817131	79818449
chr17	79818980	79819776
chr17	79821407	79822002
chr17	79822561	79823244

chr17	79823771	79823971
chr17	79824737	79825269
chr17	79825452	79827165
chr17	79827310	79827614
chr17	79830509	79831518
chr17	79831610	79831623
chr17	79827745	79830409
chr17	79832767	79833070
chr17	79843930	79844129
chr17	79844559	79844972
chr17	79847106	79847367
chr17	79848160	79849303
chr17	79849423	79850479
chr17	79851521	79851577
chr17	79853373	79853572
chr17	79856026	79856163
chr17	79855580	79855783
chr17	79856858	79857328
chr17	79859854	79860529
chr17	79868576	79869279
chr17	79869596	79869934
chr17	79870239	79870473
chr17	79871213	79871446
chr17	79874422	79875321
chr17	79874220	79874298
chr17	79875481	79876072
chr17	79876229	79876428
chr17	79880342	79881817
chr17	79882058	79882579
chr17	79882948	79883194
chr17	79883419	79883888
chr17	79884439	79885341
chr17	79885678	79886805
chr17	79893838	79896042
chr17	79917331	79918363
chr17	79923349	79923665
chr17	79922987	79923271
chr17	79925801	79926571
chr17	79934224	79934434
chr17	79934776	79936209
chr17	79936309	79936600
chr17	79937174	79937385
chr17	79937806	79940271
chr17	79940478	79940548
chr17	79940901	79940996
chr17	79945908	79945991

chr17	79946071	79946128
chr17	79960806	79961029
chr17	79961103	79961709
chr17	79961998	79962329
chr17	79979742	79980885
chr17	79981130	79981738
chr17	79988628	79989375
chr17	79989553	79990365
chr17	79994803	79996000
chr17	79996104	79996278
chr17	80008704	80009470
chr17	80009577	80010036
chr17	80010147	80010712
chr17	80022271	80022476
chr17	80022622	80023622
chr17	80024377	80024734
chr17	80023760	80023995
chr17	80027409	80027746
chr17	80028248	80028541
chr17	80030942	80031366
chr17	80031939	80032372
chr17	80032999	80034202
chr17	80034426	80034629
chr17	80051818	80052130
chr17	80052374	80052779
chr17	80053244	80058299
chr17	80060430	80060726
chr17	80061293	80061492
chr17	80061744	80062111
chr17	80062237	80065131
chr17	80067086	80067262
chr17	80068176	80068649
chr17	80069533	80070056
chr17	80078590	80079027
chr17	80087961	80088027
chr17	80088291	80088304
chr17	80089212	80089721
chr17	80091355	80092416
chr17	80103137	80103208
chr17	80110635	80111009
chr17	80111548	80111790
chr17	80116152	80116401
chr17	80117289	80117373
chr17	80117526	80117683
chr17	80118131	80118573
chr17	80120030	80120042

chr17	80125277	80125395
chr17	80160350	80160564
chr17	80161902	80162428
chr17	80162562	80162793
chr17	80163059	80164985
chr17	80166309	80166792
chr17	80167750	80167777
chr17	80167935	80167954
chr17	80168215	80168858
chr17	80169915	80173090
chr17	80173536	80177163
chr17	80178914	80178981
chr17	80180364	80180579
chr17	80181308	80181507
chr17	80183224	80183554
chr17	80185889	80189985
chr17	80190163	80190441
chr17	80191634	80191774
chr17	80191971	80192021
chr17	80192286	80192505
chr17	80192915	80193315
chr17	80193592	80194316
chr17	80194516	80194824
chr17	80195185	80195787
chr17	80196116	80196364
chr17	80196907	80197106
chr17	80198278	80200593
chr17	80201186	80201923
chr17	80202126	80202355
chr17	80204338	80204593
chr17	80204815	80205334
chr17	80207614	80208056
chr17	80209190	80209694
chr17	80218501	80218723
chr17	80224626	80225053
chr17	80225412	80225831
chr17	80226116	80226912
chr17	80228815	80228914
chr17	80229115	80232003
chr17	80235120	80235384
chr17	80238453	80238854
chr17	80239539	80239784
chr17	80239974	80242677
chr17	80242959	80243600
chr17	80244275	80245261
chr17	80245670	80246847

chr17	80247246	80247602
chr17	80247681	80247790
chr17	80248065	80248099
chr17	80248180	80248681
chr17	80248987	80249239
chr17	80249550	80251211
chr17	80251354	80252788
chr17	80252980	80253333
chr17	80253432	80253558
chr17	80253724	80256970
chr17	80257292	80257899
chr17	80258254	80258608
chr17	80258984	80259559
chr17	80261194	80261393
chr17	80266149	80266319
chr17	80267399	80267705
chr17	80278460	80279174
chr17	80279363	80279792
chr17	80288072	80288409
chr17	80289613	80290958
chr17	80291189	80291995
chr17	80292210	80294789
chr17	80296107	80297069
chr17	80297238	80297904
chr17	80313027	80313421
chr17	80313806	80313933
chr17	80314106	80315580
chr17	80328761	80329048
chr17	80339083	80339534
chr17	80342204	80342899
chr17	80344752	80345129
chr17	80375264	80375354
chr17	80376198	80377042
chr17	80378027	80378121
chr17	80407858	80408646
chr17	80408911	80409015
chr17	80409653	80409956
chr17	80413566	80413666
chr17	80415040	80415249
chr17	80415826	80417279
chr17	80417440	80417452
chr17	80418273	80418606
chr17	80429098	80429100
chr17	80443135	80443334
chr17	80450415	80450605
chr17	80450842	80451050

chr17	80451542	80452121
chr17	80452341	80453725
chr17	80453902	80454458
chr17	80454614	80455073
chr17	80455477	80456473
chr17	80456661	80457038
chr17	80458888	80459180
chr17	80459396	80460630
chr17	80462107	80462219
chr17	80463391	80463590
chr17	80463844	80464704
chr17	80467971	80468093
chr17	80473399	80473478
chr17	80473650	80473818
chr17	80475093	80475699
chr17	80476979	80477494
chr17	80478952	80479335
chr17	80479582	80480494
chr17	80480796	80481032
chr17	80477892	80478639
chr17	80482508	80482777
chr17	80485200	80485489
chr17	80485741	80485968
chr17	80486362	80486722
chr17	80486855	80487899
chr17	80488500	80488794
chr17	80489542	80489863
chr17	80491618	80492109
chr17	80492517	80492762
chr17	80493828	80494142
chr17	80495475	80495835
chr17	80497611	80497828
chr17	80497951	80498140
chr17	80498481	80498680
chr17	80507690	80508029
chr17	80508924	80509294
chr17	80509588	80509605
chr17	80510278	80510507
chr17	80511628	80511807
chr17	80512147	80512383
chr17	80512603	80512704
chr17	80512774	80512993
chr17	80518818	80519030
chr17	80520174	80520370
chr17	80539525	80539775
chr17	80542675	80542731

chr17	80543926	80544454
chr17	80549886	80550100
chr17	80551165	80552547
chr17	80585870	80586069
chr17	80590616	80590685
chr17	80604482	80606373
chr17	80606720	80607126
chr17	80607417	80607704
chr17	80655523	80656473
chr17	80656905	80657104
chr17	80659522	80660114
chr17	80666234	80666444
chr17	80666510	80666651
chr17	80673826	80674238
chr17	80674592	80675530
chr17	80692494	80694267
chr17	80699634	80699749
chr17	80707700	80707936
chr17	80708987	80709410
chr17	80709574	80710858
chr17	80711418	80711682
chr17	80711774	80712002
chr17	80733040	80733239
chr17	80744219	80744430
chr17	80744591	80744728
chr17	80745192	80745516
chr17	80745763	80745991
chr17	80776757	80776904
chr17	80784525	80784533
chr17	80792798	80793480
chr17	80793612	80794049
chr17	80794161	80794508
chr17	80794754	80794955
chr17	80795533	80795636
chr17	80796199	80796378
chr17	80797590	80798284
chr17	80798522	80799099
chr17	80799610	80799812
chr17	80804861	80805210
chr17	80805720	80807267
chr17	80807878	80808040
chr17	80811024	80811392
chr17	80813292	80813398
chr17	80814249	80814670
chr17	80816100	80816443
chr17	80816674	80818276

chr17	80820065	80820077
chr17	80831770	80831864
chr17	80831931	80833354
chr17	80833467	80833778
chr17	80833865	80834025
chr17	80834165	80834984
chr17	80881918	80882164
chr17	80921357	80921612
chr17	81006298	81006397
chr17	81006677	81006785
chr17	81006869	81007215
chr17	81008879	81009596
chr17	81009845	81010337
chr17	81011761	81012054
chr17	81022153	81022712
chr17	81022856	81023197
chr17	81031462	81031851
chr17	81036769	81037377
chr17	81037811	81039259
chr17	81041085	81041399
chr17	81042287	81042500
chr17	81061140	81061339
chr17	81067120	81067978
chr18	105405	105882
chr18	106163	112015
chr18	112203	112671
chr18	158063	158293
chr18	158468	159200
chr18	267578	268690
chr18	597319	597509
chr18	657222	658342
chr18	658677	658847
chr18	697822	697895
chr18	702855	702986
chr18	703143	704055
chr18	711437	711711
chr18	711895	712580
chr18	712769	712968
chr18	811559	813341
chr18	2302605	2303255
chr18	2303370	2303651
chr18	2303840	2304133
chr18	2304401	2304606
chr18	2304750	2304805
chr18	2410741	2411065
chr18	2411381	2411788

chr18	2443022	2443199
chr18	2515697	2515885
chr18	2516067	2516600
chr18	2536497	2536812
chr18	2570637	2571682
chr18	2592868	2593044
chr18	2646383	2647056
chr18	2655075	2656986
chr18	2755805	2755818
chr18	2873294	2873413
chr18	2905811	2907436
chr18	2948359	2948491
chr18	2977037	2977121
chr18	2988056	2988139
chr18	2988298	2988608
chr18	3012517	3013304
chr18	3013621	3013627
chr18	3013794	3014635
chr18	3015112	3015211
chr18	3036432	3036495
chr18	3037333	3037462
chr18	3037711	3037797
chr18	3054695	3055223
chr18	3217972	3218132
chr18	3245874	3246109
chr18	3246344	3247351
chr18	3247482	3248210
chr18	3248604	3248928
chr18	3250685	3251078
chr18	3260823	3261225
chr18	3261382	3262894
chr18	3264415	3264614
chr18	3266270	3266494
chr18	3297157	3297540
chr18	3305261	3305448
chr18	3305613	3306339
chr18	3306408	3306491
chr18	3306689	3306766
chr18	3321791	3321811
chr18	3329853	3329879
chr18	3345960	3346086
chr18	3346293	3346404
chr18	3350671	3351264
chr18	3351413	3351525
chr18	3380470	3380793
chr18	3409097	3409607

chr18	3411898	3412648
chr18	3412786	3412952
chr18	3441348	3441663
chr18	3446699	3447050
chr18	3447392	3447772
chr18	3447878	3448226
chr18	3448470	3450053
chr18	3450156	3450886
chr18	3451081	3452896
chr18	3453251	3454137
chr18	3459417	3459624
chr18	3459782	3459835
chr18	3465497	3465630
chr18	3466278	3466454
chr18	3499135	3499334
chr18	3512306	3512366
chr18	3541289	3541488
chr18	3593478	3594315
chr18	3594441	3594666
chr18	3594733	3595242
chr18	3595436	3596095
chr18	3598356	3598518
chr18	3598841	3599021
chr18	3602666	3603835
chr18	3604016	3604378
chr18	3604547	3605355
chr18	3605995	3606333
chr18	3610853	3610966
chr18	3618338	3618707
chr18	3622243	3622629
chr18	3622997	3623282
chr18	3623345	3624683
chr18	3624815	3625515
chr18	3649965	3650441
chr18	3652402	3652744
chr18	3653015	3653244
chr18	3665805	3666011
chr18	3666131	3666397
chr18	3675404	3675526
chr18	3675668	3675862
chr18	3678932	3679272
chr18	3679608	3679711
chr18	3703694	3703812
chr18	3758495	3758518
chr18	3758859	3758969
chr18	3771270	3771490

chr18	3771688	3771811
chr18	3787250	3787645
chr18	3794901	3795199
chr18	3816847	3817063
chr18	3817193	3817336
chr18	3995732	3995740
chr18	5237509	5238315
chr18	5238471	5238547
chr18	5295638	5296008
chr18	5296294	5296851
chr18	5297109	5297211
chr18	6729632	6730140
chr18	6994042	6994166
chr18	6994397	6994480
chr18	6994877	6994988
chr18	7038160	7038553
chr18	7038827	7039022
chr18	7084169	7084438
chr18	7117052	7118031
chr18	7272851	7273437
chr18	7301479	7301613
chr18	7414436	7414929
chr18	7415530	7415904
chr18	7417717	7418233
chr18	7566570	7567221
chr18	7567353	7568236
chr18	7568339	7568708
chr18	7627199	7627360
chr18	7871544	7871662
chr18	7871795	7872039
chr18	7872511	7872923
chr18	7877710	7877909
chr18	7921241	7921778
chr18	7922124	7922632
chr18	7941484	7941549
chr18	7941662	7941978
chr18	7946675	7947245
chr18	7946101	7946308
chr18	7950615	7951227
chr18	8038417	8038434
chr18	8038740	8038816
chr18	8116284	8116334
chr18	8116692	8116941
chr18	8423157	8423200
chr18	8517124	8517469
chr18	8553073	8553302

chr18	8553427	8553922
chr18	8608721	8609208
chr18	8609386	8609636
chr18	8610058	8610753
chr18	8623800	8623948
chr18	8655450	8655562
chr18	8658362	8658566
chr18	8665832	8665988
chr18	8685230	8685393
chr18	8685592	8686027
chr18	8702086	8702183
chr18	8704467	8705221
chr18	8705337	8707587
chr18	8707757	8707897
chr18	8722032	8722155
chr18	8724410	8724773
chr18	8730329	8730555
chr18	8747419	8747618
chr18	8749644	8749794
chr18	8750259	8750557
chr18	8750925	8751295
chr18	8786187	8786779
chr18	8786931	8787318
chr18	8790788	8791047
chr18	8800369	8801892
chr18	8811012	8811238
chr18	8830312	8830415
chr18	8842332	8842478
chr18	8859515	8859581
chr18	8890846	8891045
chr18	8946256	8946489
chr18	8949472	8949598
chr18	8961087	8961273
chr18	8961417	8961517
chr18	8982476	8983070
chr18	8983262	8983497
chr18	8987498	8987620
chr18	9016804	9017165
chr18	9017443	9018247
chr18	9038292	9038394
chr18	9055327	9055387
chr18	9055573	9055708
chr18	9074039	9074136
chr18	9101710	9102064
chr18	9102254	9102452
chr18	9102740	9103354

chr18	9104070	9104293
chr18	9117206	9117754
chr18	9135786	9135984
chr18	9136110	9136501
chr18	9136794	9137284
chr18	9137642	9138067
chr18	9148185	9148236
chr18	9333723	9333900
chr18	9334073	9334432
chr18	9334708	9335477
chr18	9364493	9364662
chr18	9366410	9366589
chr18	9422774	9422973
chr18	9423159	9423229
chr18	9474478	9475276
chr18	9475508	9476155
chr18	9526779	9526803
chr18	9539266	9539371
chr18	9557567	9558051
chr18	9559220	9559627
chr18	9561433	9561489
chr18	9575496	9575583
chr18	9575930	9575950
chr18	9595045	9595205
chr18	9612187	9612643
chr18	9613521	9613580
chr18	9613725	9614564
chr18	9614690	9615411
chr18	9616168	9616373
chr18	9617146	9617599
chr18	9625509	9625949
chr18	9627634	9627984
chr18	9631830	9632319
chr18	9642912	9643431
chr18	9643550	9643981
chr18	9647477	9647796
chr18	9647952	9648302
chr18	9648423	9648817
chr18	9648968	9649207
chr18	9658003	9658846
chr18	9671163	9671336
chr18	9671546	9671650
chr18	9673106	9674030
chr18	9683390	9683505
chr18	9688240	9688370
chr18	9688690	9688889

chr18	9701593	9701710
chr18	9707827	9708706
chr18	9709320	9709385
chr18	9718695	9718818
chr18	9719371	9720134
chr18	9720345	9720616
chr18	9721489	9721699
chr18	9731574	9731823
chr18	9733726	9733922
chr18	9734016	9734742
chr18	9740063	9740363
chr18	9742622	9743130
chr18	9743441	9743656
chr18	9743841	9743997
chr18	9753804	9753941
chr18	9754095	9754363
chr18	9771418	9771587
chr18	9779583	9779658
chr18	9779886	9780129
chr18	9782627	9782839
chr18	9824960	9825183
chr18	9825685	9826053
chr18	9827497	9828676
chr18	9828786	9829445
chr18	9835109	9835730
chr18	9851548	9851806
chr18	9868203	9868388
chr18	9874636	9874770
chr18	9874952	9874977
chr18	9895440	9895987
chr18	9913190	9915349
chr18	9917968	9918222
chr18	9928861	9929038
chr18	9933427	9933803
chr18	9947408	9947582
chr18	9952696	9952895
chr18	10015177	10015389
chr18	10015686	10015689
chr18	10035545	10035848
chr18	10036012	10036017
chr18	10036749	10036893
chr18	10058415	10058576
chr18	10131933	10132211
chr18	10413228	10413347
chr18	10417122	10417235
chr18	10455035	10455236

chr18	10525729	10526746
chr18	10560335	10560540
chr18	11635119	11635390
chr18	11638840	11638930
chr18	11639554	11639586
chr18	11689391	11689797
chr18	11733249	11733389
chr18	11768331	11768356
chr18	11849846	11850533
chr18	11850885	11851229
chr18	11851381	11852213
chr18	11857115	11858015
chr18	11859517	11860031
chr18	11860177	11860348
chr18	11871646	11871793
chr18	11872506	11872655
chr18	11892367	11892777
chr18	11894105	11894223
chr18	11907571	11907792
chr18	11908121	11909281
chr18	11909464	11909547
chr18	11942901	11943181
chr18	11946020	11946584
chr18	11946706	11948051
chr18	11951714	11952030
chr18	11952409	11952457
chr18	11954248	11954362
chr18	11954514	11955075
chr18	11955275	11955474
chr18	11955647	11956116
chr18	11976512	11976669
chr18	11977065	11977320
chr18	11978098	11978800
chr18	11980271	11980852
chr18	11981245	11982258
chr18	11982463	11982671
chr18	11993755	11993944
chr18	11994243	11994492
chr18	11994672	11995019
chr18	11995204	11995410
chr18	12015249	12015439
chr18	12026705	12027115
chr18	12038258	12038827
chr18	12255228	12255427
chr18	12271407	12272340
chr18	12278038	12278263

chr18	12278783	12278982
chr18	12279908	12280107
chr18	12281960	12282154
chr18	12282411	12282625
chr18	12287016	12287039
chr18	12287854	12288203
chr18	12288334	12288622
chr18	12294642	12294906
chr18	12295299	12295587
chr18	12307353	12309218
chr18	12309531	12310422
chr18	12333611	12333686
chr18	12375092	12375306
chr18	12376011	12377318
chr18	12377566	12378100
chr18	12391913	12392063
chr18	12403553	12403752
chr18	12403901	12404003
chr18	12402583	12403299
chr18	12404163	12404308
chr18	12407415	12407703
chr18	12407833	12408625
chr18	12408745	12408907
chr18	12409516	12409626
chr18	12419261	12420531
chr18	12470530	12471189
chr18	12518453	12518620
chr18	12531762	12531827
chr18	12539857	12539949
chr18	12554021	12554385
chr18	12569237	12569504
chr18	12596797	12597086
chr18	12597708	12597720
chr18	12598069	12598184
chr18	12599496	12600017
chr18	12606604	12607251
chr18	12607320	12608151
chr18	12608373	12608535
chr18	12608650	12608680
chr18	12629323	12629704
chr18	12630122	12630535
chr18	12637421	12637741
chr18	12637909	12638456
chr18	12640271	12641136
chr18	12643859	12643899
chr18	12644095	12644273

chr18	12645395	12645594
chr18	12645708	12645761
chr18	12646001	12646200
chr18	12653030	12653388
chr18	12656864	12658914
chr18	12667988	12668202
chr18	12671532	12671924
chr18	12679201	12679537
chr18	12698254	12698532
chr18	12698644	12698748
chr18	12702150	12703373
chr18	12741169	12741374
chr18	12741618	12742101
chr18	12742365	12742881
chr18	12744094	12744145
chr18	12747525	12747618
chr18	12748469	12748775
chr18	12770113	12770312
chr18	12811902	12812059
chr18	12838030	12838199
chr18	12838356	12838744
chr18	12839134	12839333
chr18	12841078	12841321
chr18	12853227	12853391
chr18	12862483	12862544
chr18	12872947	12873146
chr18	12883308	12884597
chr18	12889892	12890692
chr18	12897377	12897724
chr18	12911053	12911479
chr18	12946720	12946863
chr18	12947013	12947673
chr18	12947888	12948679
chr18	12990699	12991163
chr18	12991331	12991912
chr18	13003350	13003544
chr18	13019397	13019509
chr18	13224643	13224774
chr18	13567810	13567871
chr18	13568035	13568200
chr18	13641661	13641997
chr18	13710318	13710544
chr18	13710648	13710775
chr18	13726086	13727395
chr18	13791164	13791335
chr18	13797044	13797475

chr18	14132048	14132473
chr18	14132672	14132807
chr18	18510884	18512504
chr18	18512683	18513824
chr18	18515351	18520487
chr18	18513942	18515249
chr18	18633223	18633292
chr18	18633562	18633660
chr18	18654479	18654677
chr18	18691011	18691762
chr18	18692452	18692510
chr18	18692677	18692716
chr18	18743886	18743997
chr18	18744152	18744282
chr18	18810384	18810808
chr18	18822094	18822945
chr18	18823015	18823190
chr18	18970903	18971067
chr18	18971233	18971440
chr18	18971599	18971741
chr18	19019823	19019921
chr18	19137756	19138262
chr18	19178976	19179390
chr18	19179554	19180599
chr18	19180830	19181424
chr18	19192012	19192711
chr18	19199672	19200013
chr18	19218590	19218797
chr18	19247912	19248399
chr18	19253166	19253364
chr18	19255364	19255561
chr18	19283238	19284830
chr18	19284952	19285103
chr18	19319919	19320503
chr18	19320650	19322115
chr18	19406066	19406186
chr18	19406347	19406539
chr18	19476994	19477035
chr18	19611537	19611743
chr18	19612575	19612785
chr18	19749622	19749936
chr18	19750746	19750975
chr18	19751309	19752249
chr18	19762858	19763121
chr18	19793591	19793763
chr18	19793953	19794264

chr18	19794821	19795096
chr18	19862298	19862715
chr18	19880915	19881005
chr18	19928170	19928731
chr18	19929017	19929335
chr18	19964999	19965607
chr18	19965672	19965852
chr18	20012886	20012967
chr18	20045958	20046051
chr18	20046386	20047134
chr18	20047714	20047971
chr18	20048082	20048488
chr18	20050924	20051133
chr18	20131707	20131924
chr18	20132105	20132228
chr18	20139614	20140051
chr18	20140169	20140307
chr18	20160213	20160305
chr18	20160456	20160532
chr18	20274855	20274874
chr18	20291039	20291528
chr18	20297490	20297755
chr18	20393629	20393983
chr18	20394064	20394294
chr18	20406462	20406754
chr18	20406947	20407193
chr18	20408204	20408491
chr18	20411746	20411854
chr18	20421902	20422240
chr18	20424226	20424353
chr18	20424494	20424549
chr18	20428477	20429064
chr18	20436799	20437315
chr18	20492818	20493191
chr18	20493633	20494270
chr18	20510071	20510111
chr18	20512884	20513134
chr18	20513372	20514159
chr18	20558404	20558726
chr18	20611746	20612264
chr18	20612578	20612611
chr18	20618679	20618782
chr18	20675439	20675548
chr18	20686814	20686939
chr18	20703103	20703259
chr18	20711964	20712039

chr18	20714900	20715693
chr18	20715840	20716892
chr18	20811239	20811680
chr18	20830995	20831550
chr18	20839590	20840567
chr18	20861721	20862080
chr18	20863540	20864126
chr18	20882513	20882648
chr18	20883018	20883217
chr18	20883359	20883617
chr18	20893019	20893245
chr18	20911302	20911766
chr18	20957696	20958306
chr18	20958417	20959196
chr18	20959980	20960042
chr18	20966164	20966419
chr18	20967074	20967223
chr18	21002660	21002792
chr18	21017365	21017563
chr18	21017726	21018119
chr18	21032677	21033982
chr18	21034934	21035480
chr18	21082729	21083865
chr18	21090274	21090488
chr18	21095058	21095255
chr18	21095394	21095980
chr18	21109755	21109981
chr18	21143873	21144072
chr18	21145964	21145988
chr18	21146200	21146302
chr18	21152537	21152736
chr18	21156046	21156326
chr18	21165447	21165862
chr18	21166016	21166816
chr18	21167243	21167428
chr18	21168822	21169532
chr18	21173717	21173796
chr18	21186699	21186765
chr18	21199075	21199274
chr18	21206598	21207032
chr18	21207267	21207665
chr18	21209726	21209911
chr18	21211790	21212069
chr18	21216380	21216452
chr18	21216708	21216836
chr18	21231599	21231712

chr18	21242281	21242845
chr18	21242972	21243138
chr18	21269443	21270503
chr18	21274584	21274760
chr18	21275496	21275751
chr18	21296068	21297134
chr18	21297714	21297861
chr18	21297964	21298091
chr18	21298767	21299157
chr18	21299577	21299751
chr18	21299879	21300122
chr18	21365140	21365299
chr18	21407618	21407777
chr18	21407938	21408102
chr18	21408413	21408528
chr18	21408775	21408814
chr18	21417775	21417960
chr18	21418744	21419080
chr18	21424941	21424978
chr18	21425505	21425665
chr18	21429156	21429523
chr18	21437713	21437782
chr18	21438039	21438181
chr18	21439210	21439242
chr18	21451282	21451805
chr18	21452278	21452749
chr18	21452955	21453314
chr18	21454896	21455157
chr18	21455248	21455267
chr18	21455525	21455651
chr18	21457395	21457485
chr18	21457658	21457727
chr18	21463080	21463823
chr18	21464335	21465035
chr18	21504824	21504831
chr18	21572466	21572726
chr18	21574370	21574411
chr18	21574946	21574973
chr18	21594128	21595462
chr18	21596607	21596758
chr18	21595616	21595626
chr18	21596879	21596945
chr18	21606692	21606810
chr18	21607142	21607617
chr18	21644747	21644779
chr18	21643230	21643439

chr18	21644934	21645079
chr18	21656039	21656230
chr18	21718703	21718783
chr18	21718913	21719553
chr18	21785928	21786055
chr18	21789626	21789890
chr18	21792772	21792910
chr18	21793043	21793518
chr18	21808939	21809004
chr18	21851115	21851900
chr18	21852133	21852460
chr18	21853647	21853967
chr18	21922349	21922605
chr18	21939877	21940130
chr18	21976939	21977129
chr18	21977289	21977731
chr18	21977923	21978810
chr18	22006279	22007393
chr18	22007494	22007635
chr18	22931774	22932061
chr18	23619979	23619994
chr18	23636477	23636646
chr18	23669587	23671202
chr18	23671385	23671443
chr18	23677798	23677991
chr18	23805410	23805637
chr18	23805999	23806216
chr18	23806470	23807438
chr18	23807527	23808008
chr18	23848369	23848484
chr18	23848689	23848810
chr18	24009488	24009542
chr18	24009690	24009953
chr18	24016067	24016207
chr18	24016323	24016791
chr18	24017490	24017723
chr18	24020532	24020843
chr18	24021131	24021648
chr18	24036151	24036281
chr18	24036429	24036478
chr18	24040618	24040849
chr18	24040980	24041652
chr18	24057839	24058159
chr18	24060712	24061824
chr18	24063645	24063702
chr18	24063889	24063996

chr18	24067419	24067883
chr18	24112446	24112497
chr18	24115941	24116008
chr18	24120035	24120071
chr18	24120223	24120506
chr18	24125759	24126021
chr18	24126695	24126936
chr18	24127066	24128616
chr18	24128723	24130262
chr18	24130461	24130977
chr18	24155151	24155644
chr18	24201272	24201481
chr18	24236285	24237226
chr18	24326530	24326783
chr18	24716056	24716195
chr18	24765453	24765508
chr18	24795018	24795155
chr18	25439833	25439932
chr18	25440099	25440422
chr18	26348185	26348243
chr18	26614395	26614580
chr18	26614704	26614828
chr18	26727957	26728191
chr18	26734785	26735419
chr18	26736510	26737173
chr18	26744322	26744448
chr18	26745200	26745261
chr18	26745497	26745611
chr18	27081892	27081894
chr18	27231230	27231345
chr18	27420059	27420068
chr18	27986906	27987064
chr18	28567349	28567397
chr18	28567634	28567773
chr18	28572525	28572622
chr18	28591624	28591818
chr18	28619463	28619542
chr18	28619818	28619862
chr18	28620911	28621268
chr18	28621333	28623249
chr18	28626840	28626905
chr18	28657894	28658192
chr18	28680830	28682626
chr18	28682967	28683333
chr18	28683447	28683680
chr18	28726867	28726909

chr18	28737079	28737178
chr18	28737316	28737370
chr18	28742255	28742633
chr18	28783563	28783700
chr18	28786664	28786749
chr18	28787148	28787442
chr18	28810447	28810553
chr18	28810662	28810897
chr18	28821786	28821896
chr18	28822242	28822295
chr18	28835894	28836331
chr18	28854969	28855302
chr18	28855804	28856003
chr18	28999599	28999998
chr18	29009584	29009670
chr18	29009798	29009967
chr18	29027485	29027963
chr18	29028955	29029174
chr18	29030380	29030720
chr18	29031564	29031905
chr18	29034006	29034440
chr18	29066437	29066490
chr18	29067400	29067705
chr18	29067889	29068019
chr18	29068579	29068901
chr18	29069307	29069520
chr18	29069626	29069690
chr18	29070363	29070789
chr18	29077547	29078748
chr18	29088726	29089008
chr18	29090467	29090573
chr18	29092353	29092869
chr18	29093441	29093718
chr18	29093842	29093938
chr18	29199241	29199396
chr18	29223156	29223583
chr18	29228875	29228906
chr18	29259116	29259734
chr18	29264065	29264117
chr18	29264351	29265659
chr18	29304191	29304358
chr18	29365264	29365389
chr18	29365588	29365710
chr18	29499326	29499391
chr18	29512758	29513065
chr18	29522205	29523150

chr18	29523464	29523813
chr18	29523942	29523966
chr18	29536736	29537514
chr18	29597978	29598137
chr18	29598608	29599226
chr18	29600053	29600252
chr18	29665334	29665347
chr18	29671592	29672377
chr18	29672684	29673296
chr18	29708057	29708423
chr18	29881994	29882357
chr18	29888463	29889104
chr18	29897473	29897482
chr18	29929771	29930075
chr18	29942509	29942802
chr18	29952348	29952559
chr18	29952804	29952961
chr18	29958546	29958688
chr18	29958941	29959208
chr18	29963358	29963516
chr18	30005484	30005804
chr18	30006044	30006279
chr18	30050115	30050320
chr18	30050604	30050838
chr18	30051455	30051612
chr18	30111278	30111284
chr18	30111477	30111599
chr18	30122237	30123087
chr18	30501066	30501170
chr18	32173325	32173395
chr18	32227341	32227380
chr18	32241748	32241881
chr18	32288978	32289101
chr18	32440104	32440268
chr18	32556830	32556988
chr18	32557097	32557296
chr18	32559546	32559569
chr18	32573313	32573443
chr18	32621024	32621233
chr18	32621412	32621879
chr18	32811228	32811288
chr18	32820319	32820841
chr18	32821024	32821372
chr18	32846966	32847148
chr18	32869825	32870098
chr18	32870358	32870621

chr18	32923797	32924814
chr18	32956556	32956622
chr18	32956838	32957106
chr18	32992815	32993013
chr18	33032880	33033388
chr18	33036299	33036878
chr18	33037073	33037474
chr18	33038610	33039320
chr18	33040165	33040471
chr18	33041003	33041171
chr18	33058423	33058909
chr18	33076902	33077922
chr18	33078090	33078216
chr18	33081907	33082072
chr18	33082350	33082614
chr18	33093062	33093195
chr18	33106491	33106835
chr18	33160818	33161383
chr18	33161778	33162067
chr18	33196260	33196647
chr18	33196756	33196945
chr18	33197073	33197204
chr18	33333675	33333889
chr18	33551695	33552415
chr18	33552659	33553073
chr18	33565091	33565333
chr18	33597662	33597699
chr18	33647046	33647515
chr18	33652371	33652599
chr18	33655776	33656009
chr18	33668970	33669499
chr18	33704801	33705168
chr18	33708459	33708811
chr18	33708966	33709334
chr18	33709494	33709800
chr18	33709922	33710310
chr18	33756384	33756747
chr18	33757068	33757200
chr18	33766625	33769159
chr18	33877195	33877628
chr18	33877740	33877963
chr18	33888981	33889026
chr18	33889189	33889480
chr18	33889834	33890031
chr18	33890130	33890183
chr18	33890309	33890563

chr18	34161029	34161469
chr18	34160304	34160697
chr18	34169708	34169746
chr18	34169870	34170471
chr18	34170674	34171062
chr18	34172358	34172680
chr18	34207916	34207923
chr18	34208830	34209031
chr18	34214104	34214142
chr18	34252276	34252414
chr18	34252699	34252839
chr18	34325112	34325653
chr18	34336442	34336478
chr18	34337112	34337181
chr18	34366668	34366700
chr18	34366949	34367122
chr18	34408176	34410062
chr18	34430756	34430901
chr18	34528112	34528162
chr18	34724920	34724974
chr18	34834074	34834652
chr18	36520739	36520858
chr18	36994211	36994512
chr18	37184858	37184934
chr18	37228137	37228262
chr18	37368334	37368569
chr18	37378971	37379321
chr18	37379494	37379795
chr18	37380087	37380485
chr18	37450652	37450932
chr18	37526466	37526616
chr18	37526824	37527152
chr18	37539860	37540197
chr18	37540575	37540927
chr18	37795954	37796386
chr18	37952061	37952201
chr18	38430952	38431246
chr18	38446293	38446589
chr18	38447394	38447815
chr18	38448037	38448818
chr18	38457061	38457343
chr18	38491867	38491950
chr18	38492100	38492172
chr18	38724402	38724473
chr18	38724721	38724812
chr18	38725361	38725682

chr18	38725943	38725995
chr18	38971812	38971950
chr18	39324618	39324794
chr18	39341536	39342053
chr18	39364880	39365220
chr18	39425537	39425856
chr18	39450189	39450522
chr18	39450778	39451144
chr18	39451586	39451788
chr18	39451926	39452269
chr18	39455074	39455776
chr18	39535301	39535609
chr18	39628112	39628171
chr18	39628433	39628488
chr18	39891322	39891354
chr18	41241595	41241889
chr18	41242907	41243028
chr18	41245378	41245440
chr18	41361026	41361035
chr18	41729571	41729736
chr18	42207360	42207515
chr18	42259088	42259487
chr18	42260272	42260602
chr18	42305827	42305926
chr18	42311667	42312024
chr18	42312316	42312524
chr18	42313299	42313369
chr18	42919792	42919874
chr18	43145443	43145574
chr18	43145755	43145986
chr18	43355368	43355775
chr18	43370542	43370943
chr18	43373534	43373929
chr18	43381163	43381449
chr18	43381682	43382086
chr18	43382509	43383518
chr18	43386185	43386554
chr18	43417506	43417732
chr18	43451607	43451770
chr18	43500225	43500338
chr18	43531303	43531347
chr18	43531514	43531750
chr18	43542431	43542506
chr18	43542654	43542814
chr18	43542979	43543178
chr18	43546985	43547449

chr18	43547738	43548015
chr18	43554027	43554067
chr18	43607928	43608473
chr18	43639210	43639382
chr18	43642542	43643254
chr18	43643328	43643584
chr18	43643956	43644204
chr18	43644325	43644669
chr18	43648846	43649163
chr18	43649513	43650158
chr18	43651002	43651201
chr18	43651316	43651525
chr18	43651629	43652272
chr18	43673629	43673860
chr18	43677754	43678724
chr18	43684015	43684092
chr18	43684351	43684749
chr18	43733122	43733495
chr18	43735521	43735557
chr18	43735812	43735940
chr18	43736126	43736247
chr18	43736402	43736780
chr18	43739408	43739493
chr18	43753261	43753816
chr18	43753998	43754871
chr18	43772319	43772779
chr18	43772917	43773530
chr18	43829417	43829709
chr18	43830242	43830875
chr18	43839999	43840076
chr18	43901167	43901521
chr18	43901945	43902087
chr18	43902623	43902844
chr18	43904521	43904819
chr18	43913647	43914030
chr18	43914268	43914441
chr18	43967666	43968397
chr18	44496802	44498075
chr18	44526506	44526535
chr18	44552082	44552281
chr18	44553221	44553420
chr18	44675987	44676186
chr18	44676313	44677552
chr18	44689526	44689923
chr18	44701041	44701133
chr18	44701423	44701460

chr18	44701868	44702775
chr18	45408200	45408294
chr18	45408436	45408550
chr18	45456277	45457072
chr18	45457179	45458112
chr18	45458306	45458474
chr18	45471819	45472261
chr18	45529651	45529816
chr18	45534738	45534937
chr18	45550332	45550537
chr18	45679384	45679454
chr18	45687374	45688848
chr18	45851465	45851489
chr18	45851982	45852077
chr18	45856738	45856984
chr18	45919846	45919907
chr18	45936699	45937273
chr18	46065049	46065316
chr18	46065514	46066485
chr18	46066908	46067779
chr18	46067952	46068015
chr18	46072429	46072524
chr18	46072751	46072760
chr18	46073057	46073201
chr18	46074282	46075206
chr18	46076679	46076840
chr18	46079881	46080051
chr18	46089399	46089511
chr18	46089719	46089878
chr18	46098800	46098841
chr18	46107491	46107540
chr18	46147442	46147591
chr18	46147697	46148109
chr18	46148322	46149424
chr18	46149585	46150030
chr18	46150228	46150445
chr18	46150833	46151133
chr18	46152596	46152956
chr18	46153664	46154044
chr18	46154446	46154470
chr18	46156124	46156468
chr18	46157362	46157573
chr18	46159538	46159654
chr18	46188436	46189013
chr18	46201895	46202094
chr18	46271261	46271504

chr18	46358684	46359417
chr18	46359648	46359911
chr18	46360350	46360514
chr18	46365297	46365965
chr18	46374016	46374102
chr18	46374715	46374973
chr18	46375402	46375671
chr18	46378689	46378888
chr18	46400096	46400129
chr18	46428333	46428657
chr18	46461637	46461705
chr18	46466949	46466961
chr18	46473934	46474139
chr18	46474997	46475211
chr18	46475426	46475802
chr18	46475905	46478390
chr18	46478818	46479740
chr18	46480913	46481778
chr18	46483475	46483480
chr18	46481914	46482643
chr18	46483725	46483905
chr18	46484611	46484832
chr18	46486375	46486392
chr18	46486482	46486851
chr18	46486929	46487038
chr18	46487161	46487296
chr18	46499893	46500033
chr18	46500160	46500792
chr18	46501729	46502186
chr18	46502333	46503158
chr18	46517263	46517475
chr18	46517652	46518185
chr18	46518521	46518758
chr18	46523077	46523299
chr18	46523979	46524167
chr18	46524394	46524668
chr18	46524874	46525312
chr18	46532902	46533297
chr18	46533416	46533772
chr18	46542691	46542760
chr18	46543016	46543657
chr18	46543780	46544361
chr18	46545938	46546503
chr18	46546678	46547889
chr18	46572607	46572806
chr18	46581953	46582189

chr18	46582548	46582753
chr18	46733726	46734006
chr18	46734177	46734264
chr18	46763767	46763788
chr18	46768593	46768644
chr18	46775905	46776308
chr18	46804026	46804259
chr18	46839555	46839723
chr18	46841248	46841404
chr18	46925408	46925414
chr18	46942065	46942177
chr18	46986489	46987867
chr18	47001726	47002126
chr18	47003849	47004085
chr18	47013115	47013681
chr18	47014321	47014597
chr18	47015150	47015535
chr18	47018101	47018909
chr18	47019060	47019123
chr18	47047049	47047184
chr18	47047416	47047446
chr18	47074904	47075065
chr18	47087400	47087662
chr18	47088487	47088878
chr18	47089085	47089477
chr18	47216671	47216870
chr18	47217033	47217116
chr18	47255403	47255615
chr18	47255869	47256018
chr18	47258781	47258844
chr18	47305326	47305349
chr18	47305792	47305926
chr18	47310808	47310849
chr18	47314948	47315269
chr18	47339071	47339169
chr18	47339373	47340989
chr18	47361235	47361396
chr18	47361597	47361761
chr18	47363993	47364048
chr18	47364608	47364679
chr18	47365733	47365873
chr18	47375439	47375478
chr18	47375670	47376121
chr18	47460251	47460312
chr18	47687539	47687750
chr18	47721052	47721521

chr18	47766914	47767097
chr18	47792484	47793197
chr18	47794494	47794776
chr18	47804279	47804710
chr18	47806564	47806685
chr18	47807102	47808552
chr18	47813780	47814068
chr18	47814253	47814839
chr18	47834271	47834534
chr18	47834847	47834974
chr18	47883160	47883253
chr18	47883574	47883618
chr18	47901053	47901250
chr18	47901474	47901689
chr18	47979515	47979703
chr18	48158603	48158684
chr18	48158827	48159273
chr18	48159566	48159749
chr18	48393220	48393241
chr18	48394940	48395436
chr18	48396351	48396641
chr18	48396893	48397054
chr18	48404432	48406251
chr18	48494079	48495135
chr18	48554540	48554688
chr18	48555827	48557087
chr18	48596913	48597337
chr18	48661040	48662999
chr18	48660010	48660628
chr18	48664586	48664870
chr18	48721768	48721961
chr18	48722206	48722333
chr18	48722474	48723918
chr18	48724771	48725044
chr18	48792481	48792803
chr18	48793108	48793152
chr18	48847730	48847946
chr18	48848062	48848516
chr18	48856251	48856624
chr18	48866351	48866664
chr18	49039875	49040340
chr18	49049157	49049288
chr18	49049493	49049594
chr18	49057867	49058021
chr18	49058227	49058303
chr18	49686987	49687064

chr18	49687254	49687504
chr18	51549784	51549921
chr18	51635841	51635873
chr18	51646393	51646416
chr18	51660527	51660776
chr18	51675392	51676138
chr18	51676826	51676987
chr18	51717736	51718140
chr18	51749602	51750480
chr18	51750704	51751062
chr18	51751262	51751492
chr18	51759720	51759919
chr18	51772989	51773890
chr18	51780766	51781101
chr18	51784185	51784621
chr18	51787050	51787531
chr18	51795406	51796939
chr18	51811633	51811767
chr18	51883637	51884203
chr18	51884318	51885042
chr18	51915045	51915521
chr18	51992949	51993174
chr18	52275095	52275288
chr18	52275688	52275888
chr18	52379483	52379503
chr18	52407368	52407506
chr18	52407707	52407739
chr18	52442976	52443547
chr18	52443762	52444283
chr18	52490444	52490637
chr18	52495355	52496023
chr18	52496203	52496230
chr18	52496434	52496633
chr18	52613451	52613871
chr18	52626486	52626963
chr18	52635470	52635940
chr18	52650330	52650399
chr18	52768281	52768463
chr18	52988938	52988999
chr18	53255240	53255971
chr18	53256845	53257502
chr18	53302008	53302140
chr18	53446074	53446385
chr18	53446728	53447783
chr18	53448052	53448220
chr18	53457451	53457514

chr18	53501194	53501300
chr18	53523292	53523653
chr18	54070140	54070213
chr18	54212802	54213006
chr18	54305220	54305238
chr18	54305370	54305875
chr18	54306099	54306314
chr18	54318152	54318884
chr18	54376637	54376769
chr18	54671942	54672282
chr18	55253303	55254346
chr18	55254421	55254602
chr18	55288487	55289844
chr18	55297252	55298000
chr18	55299558	55299646
chr18	55317004	55317049
chr18	55334027	55334162
chr18	55366727	55366942
chr18	55383152	55383224
chr18	55395140	55395746
chr18	55396231	55396878
chr18	55416544	55416627
chr18	55426889	55427140
chr18	55438426	55438489
chr18	55438726	55438794
chr18	55442281	55442485
chr18	55443097	55443206
chr18	55455869	55455984
chr18	55456188	55456219
chr18	55469121	55469230
chr18	55469469	55471041
chr18	55471219	55471226
chr18	55474146	55474284
chr18	55474429	55474569
chr18	55480481	55480555
chr18	55480819	55480977
chr18	55483520	55483719
chr18	55483912	55484166
chr18	55486036	55486167
chr18	55487261	55487662
chr18	55507188	55507211
chr18	55507533	55507694
chr18	55513843	55513946
chr18	55566306	55566329
chr18	55567537	55567862
chr18	55568826	55569204

chr18	55569345	55569608
chr18	55574360	55574448
chr18	55576811	55577114
chr18	55588785	55589097
chr18	55589260	55589374
chr18	55609303	55609760
chr18	55614370	55614399
chr18	55614569	55614777
chr18	55672317	55672479
chr18	55673688	55673782
chr18	55710310	55710516
chr18	55710692	55713211
chr18	55713316	55713413
chr18	55721607	55721780
chr18	55743338	55743373
chr18	55743519	55743557
chr18	55746124	55746133
chr18	55750847	55751232
chr18	55754992	55755677
chr18	55758786	55758981
chr18	55789427	55789484
chr18	55844278	55844460
chr18	55852067	55852497
chr18	55853436	55854305
chr18	55858098	55858449
chr18	55862491	55863102
chr18	55863415	55863711
chr18	55869984	55869998
chr18	55875527	55875769
chr18	55876001	55876200
chr18	55876731	55876788
chr18	55883344	55883712
chr18	55888483	55888523
chr18	55888839	55889053
chr18	55889234	55889559
chr18	55889700	55889961
chr18	55890874	55891620
chr18	55897827	55898573
chr18	55899199	55899475
chr18	55903476	55903654
chr18	55909300	55909526
chr18	55930244	55930747
chr18	55935944	55936104
chr18	55936229	55936455
chr18	55937137	55937607
chr18	55939464	55939827

chr18	55944386	55944825
chr18	55948287	55948491
chr18	55953024	55953365
chr18	55956976	55957038
chr18	55968124	55968348
chr18	55975768	55975977
chr18	55977652	55977740
chr18	55978098	55978214
chr18	55980748	55981026
chr18	55981169	55981702
chr18	55981933	55981987
chr18	55983268	55983333
chr18	55988725	55988738
chr18	55989150	55989375
chr18	56029340	56029543
chr18	56029824	56029897
chr18	56133986	56134192
chr18	56134305	56134465
chr18	56135591	56135848
chr18	56161302	56161447
chr18	56187534	56187969
chr18	56188206	56188247
chr18	56208286	56208994
chr18	56215601	56215924
chr18	56234195	56234759
chr18	56234886	56235056
chr18	56235434	56235633
chr18	56267364	56267606
chr18	56306974	56307127
chr18	56307318	56307347
chr18	56334944	56335371
chr18	56336163	56336244
chr18	56337069	56337369
chr18	56337510	56337619
chr18	56338177	56338498
chr18	56338601	56339737
chr18	56349602	56349727
chr18	56350027	56350108
chr18	56431848	56431872
chr18	56435627	56435974
chr18	56442111	56442173
chr18	56449020	56449212
chr18	56449513	56449554
chr18	56454456	56454718
chr18	56460508	56460706
chr18	56460957	56461199

chr18	56467799	56468285
chr18	56516173	56516387
chr18	56516736	56516820
chr18	56517014	56517257
chr18	56517443	56517655
chr18	56530207	56530631
chr18	56530801	56531746
chr18	56531966	56532331
chr18	56672490	56672583
chr18	56675393	56676062
chr18	56700508	56700953
chr18	56806498	56806600
chr18	56806743	56807143
chr18	57025743	57026327
chr18	57299528	57299735
chr18	57329289	57329616
chr18	57329786	57330084
chr18	57330350	57330490
chr18	57330658	57330740
chr18	57363699	57364618
chr18	57364724	57365126
chr18	57367079	57368215
chr18	57371322	57371785
chr18	57383127	57383324
chr18	57383656	57383664
chr18	57385322	57385374
chr18	57385705	57385840
chr18	57386221	57386309
chr18	57411391	57411522
chr18	57411682	57411892
chr18	57447154	57447204
chr18	57482211	57482254
chr18	57482432	57482541
chr18	57504715	57504825
chr18	57505020	57505328
chr18	57511015	57511698
chr18	57514287	57514665
chr18	57531698	57531987
chr18	57552937	57553325
chr18	57566184	57567614
chr18	57567797	57568453
chr18	57571814	57572090
chr18	57572270	57572443
chr18	57576038	57576203
chr18	57576336	57576432
chr18	57577512	57577878

chr18	57584536	57585073
chr18	57585602	57586091
chr18	57594372	57594906
chr18	57620249	57620914
chr18	57628633	57628648
chr18	57652438	57652545
chr18	57652772	57652819
chr18	57686673	57686769
chr18	57721361	57721450
chr18	58045310	58045644
chr18	58559270	58559469
chr18	58559616	58559856
chr18	58560089	58560508
chr18	58561444	58562200
chr18	59249314	59249519
chr18	59269061	59269260
chr18	59273324	59273966
chr18	59274336	59274424
chr18	59519908	59520144
chr18	59528545	59528676
chr18	59528885	59528919
chr18	59558520	59558593
chr18	59560075	59562335
chr18	59562489	59562756
chr18	59563927	59564214
chr18	59564357	59564518
chr18	59617073	59617240
chr18	59617423	59617647
chr18	59618581	59618836
chr18	59619466	59619600
chr18	59619805	59620131
chr18	59659543	59659904
chr18	59662801	59663125
chr18	59666371	59666387
chr18	59694168	59694253
chr18	59798566	59798639
chr18	59853331	59853562
chr18	59853825	59854250
chr18	59854660	59855417
chr18	60001279	60001480
chr18	60104925	60105293
chr18	60105367	60105627
chr18	60118052	60118762
chr18	60119361	60119511
chr18	60152795	60152908
chr18	60153150	60153367

chr18	60153478	60153635
chr18	60153873	60154026
chr18	60189406	60190102
chr18	60191097	60192201
chr18	60192334	60192432
chr18	60193364	60194348
chr18	60205177	60205571
chr18	60222766	60222773
chr18	60302133	60302173
chr18	60381778	60382133
chr18	60382584	60383025
chr18	60383222	60385012
chr18	60408453	60408854
chr18	60430228	60430543
chr18	60430652	60430869
chr18	60433686	60433820
chr18	60434621	60435158
chr18	60448569	60448850
chr18	60449024	60449218
chr18	60449520	60449659
chr18	60467876	60467926
chr18	60489754	60489793
chr18	60549083	60549250
chr18	60604340	60604529
chr18	60683436	60683550
chr18	60742152	60742615
chr18	60750602	60750979
chr18	60905315	60905921
chr18	60968990	60969031
chr18	60986827	60987287
chr18	60987683	60987889
chr18	60988074	60988277
chr18	61003916	61004228
chr18	61011729	61011835
chr18	61013227	61014116
chr18	61033400	61033505
chr18	61033738	61034496
chr18	61034693	61035485
chr18	61076852	61076986
chr18	61078074	61078407
chr18	61087943	61088206
chr18	61089047	61089259
chr18	61089379	61090437
chr18	61094620	61094790
chr18	61094957	61095212
chr18	61106735	61106903

chr18	61107616	61107784
chr18	61111105	61111520
chr18	61111738	61111821
chr18	61112080	61112228
chr18	61113071	61113270
chr18	61116190	61116237
chr18	61118987	61119507
chr18	61120102	61120317
chr18	61121740	61122288
chr18	61137834	61138505
chr18	61139578	61140118
chr18	61143797	61143884
chr18	61144157	61144701
chr18	61145192	61145592
chr18	61146650	61146900
chr18	61185708	61185736
chr18	61220306	61220763
chr18	61252036	61252436
chr18	61253652	61254012
chr18	61254158	61254579
chr18	61256444	61256553
chr18	61271085	61271424
chr18	61271951	61272242
chr18	61308501	61308657
chr18	61308897	61308942
chr18	61331855	61332129
chr18	61344067	61344134
chr18	61344830	61345174
chr18	61350370	61350513
chr18	61350700	61350835
chr18	61352067	61352135
chr18	61442392	61442854
chr18	61444897	61445186
chr18	61455677	61455818
chr18	61455929	61456096
chr18	61515386	61515518
chr18	61515655	61515785
chr18	61517189	61517476
chr18	61517594	61517633
chr18	61527064	61527097
chr18	61534658	61534938
chr18	61536589	61537229
chr18	61549167	61549612
chr18	61549750	61550520
chr18	61550871	61551223
chr18	61555844	61556043

chr18	61557683	61557811
chr18	61559664	61559921
chr18	61560094	61560096
chr18	61577136	61577489
chr18	61603329	61603351
chr18	61603536	61604060
chr18	61616417	61617020
chr18	61617543	61617758
chr18	61629362	61629515
chr18	61637034	61637768
chr18	61637941	61638986
chr18	61801536	61801737
chr18	62072393	62072770
chr18	62903214	62903278
chr18	63236361	63236498
chr18	64179055	64179305
chr18	64227408	64227426
chr18	65027328	65027760
chr18	65085448	65085594
chr18	65183091	65183169
chr18	65183377	65184024
chr18	65184380	65184644
chr18	65192464	65192497
chr18	65192896	65192998
chr18	65256755	65256925
chr18	65295434	65295480
chr18	65295616	65295888
chr18	65398166	65398292
chr18	65438683	65438801
chr18	65930232	65930443
chr18	65930713	65931071
chr18	66095359	66095700
chr18	66097105	66097369
chr18	66103801	66103827
chr18	66346994	66347103
chr18	66382065	66382861
chr18	66508072	66508176
chr18	66584359	66584409
chr18	66925300	66925491
chr18	66925613	66925704
chr18	67711781	67712278
chr18	67713645	67713771
chr18	67715156	67715799
chr18	67848305	67848637
chr18	67872743	67873215
chr18	67873413	67873561

chr18	67953541	67953809
chr18	67955568	67956018
chr18	67956141	67957304
chr18	67999979	68000041
chr18	68000243	68000323
chr18	68000935	68001182
chr18	68002215	68002224
chr18	68003576	68003877
chr18	68004322	68004454
chr18	68004634	68004750
chr18	68024657	68025685
chr18	68031122	68031479
chr18	68038886	68038948
chr18	68040728	68040833
chr18	68049353	68049668
chr18	68051288	68051612
chr18	68051790	68052141
chr18	68087381	68087617
chr18	68087911	68087921
chr18	68089280	68089421
chr18	68091251	68091334
chr18	68098331	68098886
chr18	68101069	68101183
chr18	70500662	70500833
chr18	70500982	70501108
chr18	70514492	70514589
chr18	70515960	70516175
chr18	70518646	70518796
chr18	70533899	70535148
chr18	70535318	70536519
chr18	70537089	70537243
chr18	70538134	70538337
chr18	70538633	70538679
chr18	70662812	70662884
chr18	70663051	70663177
chr18	70683632	70683737
chr18	70983785	70983812
chr18	70984040	70984091
chr18	70997429	70997563
chr18	71007633	71007937
chr18	71231418	71231673
chr18	71281157	71281350
chr18	71281522	71281547
chr18	71281955	71282091
chr18	71282412	71282514
chr18	71314100	71314399

chr18	71337788	71337922
chr18	71356158	71356352
chr18	71416564	71416914
chr18	71510075	71510142
chr18	71680095	71680149
chr18	71809587	71809646
chr18	71814654	71815020
chr18	71815305	71815665
chr18	71815819	71816008
chr18	71830798	71830866
chr18	71863459	71863859
chr18	71886309	71886368
chr18	71901621	71901706
chr18	71949246	71949377
chr18	71956614	71956813
chr18	71958540	71959078
chr18	71959481	71959727
chr18	71977655	71978059
chr18	72162784	72163243
chr18	72163596	72164165
chr18	72165993	72166273
chr18	72166387	72166518
chr18	72166666	72166779
chr18	72166857	72167338
chr18	72168301	72169009
chr18	72264378	72265743
chr18	72265856	72266251
chr18	72436457	72436503
chr18	72517978	72518285
chr18	72518592	72518719
chr18	72544202	72544289
chr18	72609948	72610060
chr18	72706455	72707588
chr18	72708106	72708130
chr18	72708258	72708305
chr18	72780650	72781025
chr18	72782395	72782780
chr18	72782890	72783534
chr18	72895378	72895506
chr18	72916851	72917120
chr18	72919512	72919591
chr18	72919713	72920200
chr18	72920334	72922164
chr18	72922519	72922700
chr18	72923225	72923399
chr18	72923775	72924349

chr18	72960202	72960401
chr18	73843750	73843851
chr18	74061569	74061661
chr18	74104665	74104896
chr18	74106654	74107446
chr18	74162923	74163382
chr18	74171418	74171626
chr18	74188987	74189522
chr18	74202549	74203393
chr18	74203619	74204161
chr18	74205774	74207199
chr18	74204360	74205467
chr18	74207502	74207897
chr18	74208129	74208163
chr18	74237082	74237281
chr18	74240334	74240821
chr18	74265878	74266329
chr18	74270741	74271218
chr18	74271392	74271766
chr18	74275134	74275547
chr18	74275758	74276542
chr18	74277089	74278199
chr18	74278651	74279015
chr18	74279264	74279612
chr18	74279786	74280049
chr18	74280644	74281226
chr18	74284435	74284476
chr18	74285342	74285968
chr18	74316262	74316310
chr18	74316696	74316795
chr18	74323410	74323875
chr18	74331570	74332747
chr18	74398631	74398801
chr18	74452299	74452469
chr18	74514178	74514583
chr18	74518056	74518213
chr18	74518502	74518856
chr18	74531396	74531836
chr18	74532175	74532382
chr18	74532845	74533153
chr18	74533268	74534273
chr18	74534461	74534687
chr18	74534825	74535331
chr18	74535572	74535821
chr18	74539379	74539532
chr18	74573871	74574070

chr18	74724519	74724754
chr18	74773356	74773567
chr18	74773722	74774006
chr18	74777025	74777264
chr18	74777675	74778041
chr18	74780532	74780967
chr18	74783056	74783396
chr18	74783535	74784374
chr18	74797903	74798026
chr18	74798264	74798402
chr18	74798951	74798996
chr18	74799107	74799738
chr18	74799900	74800099
chr18	74800866	74801090
chr18	74801234	74801647
chr18	74812633	74814334
chr18	74814560	74814964
chr18	74826293	74826419
chr18	74826691	74827398
chr18	74839709	74840633
chr18	74841046	74841516
chr18	74843146	74845280
chr18	74873885	74874027
chr18	76822733	76822926
chr18	76823190	76823300
chr18	76828831	76829902
chr18	76916687	76916743
chr18	76916921	76917113
chr18	77005735	77006082
chr18	77108448	77108779
chr18	77153720	77153947
chr18	77154277	77154645
chr18	77155907	77156483
chr18	77159129	77160135
chr18	77160299	77160681
chr18	77176850	77177284
chr18	77326824	77327412
chr18	77327558	77328220
chr18	77349288	77349695
chr18	77370907	77371149
chr18	77385546	77385670
chr18	77402763	77403000
chr18	77437620	77437884
chr18	77438678	77439271
chr18	77439478	77440616
chr18	77448208	77448314

chr18	77548014	77548356
chr18	77550613	77551044
chr18	77551246	77551571
chr18	77553224	77553423
chr18	77555890	77555947
chr18	77706837	77706887
chr18	77709754	77709961
chr18	77710229	77710389
chr18	77710072	77710121
chr18	77710501	77712750
chr18	77714796	77714964
chr18	77715428	77715460
chr18	77720086	77720634
chr18	77721080	77721336
chr18	77721621	77722487
chr18	77724129	77724407
chr18	77726272	77726473
chr18	77724511	77725802
chr18	77728569	77728589
chr18	77746762	77746832
chr18	77747040	77747175
chr18	77748081	77749509
chr18	77793169	77794922
chr18	77795198	77795442
chr18	77866882	77867096
chr18	77867257	77868051
chr18	77882168	77882390
chr18	77890416	77890468
chr18	77904958	77905178
chr18	77905370	77905650
chr18	77933203	77933611
chr18	77937749	77938681
chr18	78003519	78005377
chr19	69908	71632
chr19	72235	72304
chr19	267261	267533
chr19	267699	267723
chr19	288916	291451
chr19	291643	291966
chr19	315215	315362
chr19	342856	343897
chr19	344587	345395
chr19	343987	344510
chr19	349544	349638
chr19	408857	409203
chr19	462025	462244

chr19	488340	489046
chr19	489183	489759
chr19	506270	506833
chr19	506999	508221
chr19	511663	512054
chr19	527344	527543
chr19	527809	528583
chr19	529303	529484
chr19	530667	531693
chr19	531819	533277
chr19	551999	552111
chr19	552197	552198
chr19	570831	571819
chr19	571942	574714
chr19	575199	575398
chr19	576095	577138
chr19	578652	578851
chr19	587882	588081
chr19	609607	609806
chr19	610421	610867
chr19	610988	612072
chr19	626561	626855
chr19	627108	627376
chr19	631397	631716
chr19	631918	632199
chr19	632417	632655
chr19	632763	633589
chr19	633698	634028
chr19	634189	634508
chr19	639962	640232
chr19	659814	660045
chr19	660273	660915
chr19	661072	661419
chr19	661638	663883
chr19	664727	665008
chr19	665118	665631
chr19	672302	672748
chr19	675850	679289
chr19	679580	680474
chr19	680670	680879
chr19	750197	750501
chr19	751042	752076
chr19	752250	753466
chr19	753568	754018
chr19	765816	766015
chr19	768116	768146

chr19	770125	770348
chr19	770936	771660
chr19	773579	773652
chr19	774591	775052
chr19	776843	777019
chr19	777801	779656
chr19	781261	781514
chr19	781969	783959
chr19	784083	784423
chr19	786672	786964
chr19	788024	788407
chr19	788700	789671
chr19	789783	792873
chr19	793079	793464
chr19	796171	796999
chr19	797336	799278
chr19	799796	799817
chr19	800261	800732
chr19	803250	803449
chr19	821497	821897
chr19	829836	830168
chr19	842913	843508
chr19	843693	846056
chr19	846923	847273
chr19	848789	849140
chr19	850384	851052
chr19	856709	857096
chr19	860017	860047
chr19	860253	860510
chr19	860727	861090
chr19	868543	869436
chr19	869546	869825
chr19	870315	870615
chr19	871930	873236
chr19	873448	873695
chr19	873988	875307
chr19	892319	893945
chr19	902636	902835
chr19	906809	907099
chr19	908316	908994
chr19	909059	909412
chr19	909575	910175
chr19	911658	911896
chr19	912271	914081
chr19	915097	915516
chr19	916181	916340

chr19	920297	920792
chr19	924421	924698
chr19	924844	926587
chr19	926690	927496
chr19	940723	941148
chr19	941508	941814
chr19	951932	952358
chr19	952493	953196
chr19	953458	954108
chr19	959268	959467
chr19	982749	983187
chr19	983686	984755
chr19	984828	985277
chr19	995895	996560
chr19	996757	996970
chr19	1016654	1017998
chr19	1018652	1019211
chr19	1019324	1019750
chr19	1020130	1021116
chr19	1021290	1021538
chr19	1021699	1022135
chr19	1025957	1028122
chr19	1028299	1029090
chr19	1029169	1029690
chr19	1035756	1035960
chr19	1038569	1038895
chr19	1039400	1039837
chr19	1040268	1040838
chr19	1064658	1065410
chr19	1065524	1067706
chr19	1070085	1072317
chr19	1074617	1074816
chr19	1075022	1075613
chr19	1076645	1076851
chr19	1085319	1085528
chr19	1092481	1092593
chr19	1093737	1093936
chr19	1094349	1095394
chr19	1095664	1095863
chr19	1096090	1096271
chr19	1097660	1097859
chr19	1098456	1098816
chr19	1099029	1100356
chr19	1100632	1100720
chr19	1100816	1101757
chr19	1102007	1102603

chr19	1102769	1103722
chr19	1103993	1104956
chr19	1105062	1105440
chr19	1120464	1121175
chr19	1125134	1125427
chr19	1127912	1128111
chr19	1128580	1128909
chr19	1130022	1130244
chr19	1130527	1130852
chr19	1131115	1132314
chr19	1132538	1132934
chr19	1134284	1135023
chr19	1144925	1145124
chr19	1153463	1153662
chr19	1153840	1153859
chr19	1154048	1155184
chr19	1155352	1155597
chr19	1155739	1155966
chr19	1161352	1161828
chr19	1165686	1165993
chr19	1167559	1170333
chr19	1170521	1171214
chr19	1171738	1172298
chr19	1172524	1174611
chr19	1175220	1175533
chr19	1175751	1178348
chr19	1180235	1181446
chr19	1181559	1182493
chr19	1182755	1183023
chr19	1183281	1184304
chr19	1184434	1185444
chr19	1185514	1185700
chr19	1178598	1179952
chr19	1188133	1188364
chr19	1189197	1189475
chr19	1189587	1189885
chr19	1191626	1191825
chr19	1193294	1193479
chr19	1193806	1194022
chr19	1194369	1195049
chr19	1200871	1201018
chr19	1205250	1206035
chr19	1206173	1206668
chr19	1207032	1207306
chr19	1208156	1208622
chr19	1208685	1209057

chr19	1209211	1209468
chr19	1209704	1210967
chr19	1211219	1211232
chr19	1212237	1212804
chr19	1213109	1213809
chr19	1214215	1216945
chr19	1219637	1219836
chr19	1225264	1225549
chr19	1236889	1237954
chr19	1238060	1238462
chr19	1239163	1239390
chr19	1240227	1242357
chr19	1242537	1243562
chr19	1244487	1244909
chr19	1246883	1247751
chr19	1248143	1250149
chr19	1250458	1251352
chr19	1251508	1252956
chr19	1253170	1254494
chr19	1256197	1257197
chr19	1258211	1259995
chr19	1260219	1263335
chr19	1263599	1264279
chr19	1265714	1267178
chr19	1269152	1271154
chr19	1273274	1273528
chr19	1273758	1275865
chr19	1276071	1276733
chr19	1312067	1312525
chr19	1315296	1315313
chr19	1314644	1315116
chr19	1318614	1318813
chr19	1320455	1320880
chr19	1324576	1325228
chr19	1326291	1326648
chr19	1333130	1333867
chr19	1354402	1354745
chr19	1355013	1355410
chr19	1383580	1384496
chr19	1384912	1384930
chr19	1385099	1385424
chr19	1394737	1394973
chr19	1401269	1401658
chr19	1402819	1403296
chr19	1403512	1403727
chr19	1404168	1404183

chr19	1404550	1404823
chr19	1405402	1405709
chr19	1405943	1406665
chr19	1406796	1407381
chr19	1407627	1409269
chr19	1409639	1410069
chr19	1411816	1412015
chr19	1414094	1414305
chr19	1414500	1414632
chr19	1414794	1415579
chr19	1415752	1416222
chr19	1436402	1436439
chr19	1437262	1437993
chr19	1438435	1439229
chr19	1439535	1439812
chr19	1441672	1441971
chr19	1442782	1443406
chr19	1443743	1444080
chr19	1445782	1446183
chr19	1467135	1469410
chr19	1469554	1470772
chr19	1471646	1471866
chr19	1474335	1474534
chr19	1477062	1477224
chr19	1478151	1480268
chr19	1484598	1484780
chr19	1484847	1484987
chr19	1490011	1490459
chr19	1491141	1491688
chr19	1492170	1492552
chr19	1498331	1498561
chr19	1503327	1503857
chr19	1507481	1507680
chr19	1507832	1508287
chr19	1508742	1508769
chr19	1508423	1508669
chr19	1511010	1511209
chr19	1512434	1513988
chr19	1545774	1546139
chr19	1546543	1546771
chr19	1565073	1565409
chr19	1566769	1567692
chr19	1568702	1568983
chr19	1572743	1573596
chr19	1586761	1587262
chr19	1587328	1587654

chr19	1587869	1588788
chr19	1589377	1590564
chr19	1590664	1590791
chr19	1590899	1592705
chr19	1593099	1593523
chr19	1603436	1603635
chr19	1603980	1605442
chr19	1605697	1606032
chr19	1606155	1606413
chr19	1606647	1606806
chr19	1607795	1608160
chr19	1651040	1651065
chr19	1651177	1651477
chr19	1651649	1652357
chr19	1653353	1654046
chr19	1672094	1672951
chr19	1725834	1726044
chr19	1746442	1747014
chr19	1747635	1747990
chr19	1749610	1749846
chr19	1754825	1755073
chr19	1773230	1773366
chr19	1774148	1774399
chr19	1811754	1812787
chr19	1813036	1813540
chr19	1825498	1825654
chr19	1847214	1847413
chr19	1847850	1848779
chr19	1848908	1849493
chr19	1849566	1849722
chr19	1850302	1850502
chr19	1850624	1850967
chr19	1853258	1853458
chr19	1853645	1855423
chr19	1855591	1857351
chr19	1857458	1858282
chr19	1858927	1859310
chr19	1859952	1860283
chr19	1860532	1861950
chr19	1862183	1862839
chr19	1862988	1863279
chr19	1863399	1863598
chr19	1864015	1864707
chr19	1864828	1865307
chr19	1865421	1865746
chr19	1874172	1874192

chr19	1875118	1875317
chr19	1875567	1877208
chr19	1884810	1885400
chr19	1885721	1886519
chr19	1897459	1898508
chr19	1898663	1898792
chr19	1904400	1905162
chr19	1905453	1906392
chr19	1928957	1929031
chr19	1929191	1929315
chr19	1929581	1930262
chr19	1930857	1931084
chr19	1939045	1939311
chr19	1939526	1941158
chr19	1941360	1942167
chr19	1942373	1942641
chr19	1943005	1943396
chr19	2014788	2015477
chr19	2015983	2016395
chr19	2017027	2017121
chr19	2016571	2016782
chr19	2026374	2026756
chr19	2027613	2028254
chr19	2029123	2029264
chr19	2031289	2031318
chr19	2031422	2032450
chr19	2032651	2032962
chr19	2041456	2042041
chr19	2042540	2042739
chr19	2042857	2043106
chr19	2047708	2047955
chr19	2048653	2048852
chr19	2049423	2049622
chr19	2050222	2051209
chr19	2051345	2052014
chr19	2053188	2053259
chr19	2053478	2053716
chr19	2054611	2054672
chr19	2055073	2055289
chr19	2055775	2057336
chr19	2058299	2060359
chr19	2060437	2062428
chr19	2069354	2069832
chr19	2075863	2076108
chr19	2080522	2082085
chr19	2083961	2084250

chr19	2085477	2085762
chr19	2086449	2087211
chr19	2087344	2088112
chr19	2088500	2091121
chr19	2091337	2091622
chr19	2092503	2096659
chr19	2124778	2125060
chr19	2147602	2147819
chr19	2150033	2151159
chr19	2152195	2152563
chr19	2153139	2153600
chr19	2154331	2155521
chr19	2155907	2156289
chr19	2160103	2160436
chr19	2161522	2161617
chr19	2163123	2163666
chr19	2162162	2163024
chr19	2163854	2164533
chr19	2164680	2164916
chr19	2165063	2165330
chr19	2165626	2166029
chr19	2167823	2168852
chr19	2169380	2169452
chr19	2234935	2234981
chr19	2234456	2234812
chr19	2235347	2238268
chr19	2250724	2251061
chr19	2251317	2251682
chr19	2252987	2253218
chr19	2253588	2254101
chr19	2259623	2259797
chr19	2260296	2260909
chr19	2266565	2266765
chr19	2268096	2268100
chr19	2269060	2269377
chr19	2269491	2270768
chr19	2271057	2271386
chr19	2272206	2272459
chr19	2282257	2282456
chr19	2297603	2297759
chr19	2320371	2320921
chr19	2327100	2329414
chr19	2373563	2373701
chr19	2385382	2386002
chr19	2386087	2386260
chr19	2391029	2391278

chr19	2425184	2425720
chr19	2426303	2426593
chr19	2426894	2427973
chr19	2454026	2454338
chr19	2455231	2456881
chr19	2457121	2457523
chr19	2461072	2461224
chr19	2461475	2462189
chr19	2462376	2463049
chr19	2465285	2465548
chr19	2468937	2469121
chr19	2469263	2469627
chr19	2469784	2470220
chr19	2471758	2472070
chr19	2474469	2474602
chr19	2474820	2475082
chr19	2475656	2476010
chr19	2476221	2476705
chr19	2478067	2480052
chr19	2488987	2489433
chr19	2490518	2491230
chr19	2492380	2492767
chr19	2491759	2491937
chr19	2494601	2495715
chr19	2498884	2498902
chr19	2499168	2499420
chr19	2503422	2503515
chr19	2507015	2507313
chr19	2507423	2507652
chr19	2512571	2512847
chr19	2515854	2516016
chr19	2519854	2520075
chr19	2525166	2526036
chr19	2527028	2527183
chr19	2527630	2527709
chr19	2528662	2528911
chr19	2529098	2529254
chr19	2529342	2529606
chr19	2529848	2530102
chr19	2532719	2532895
chr19	2540517	2540803
chr19	2541023	2541332
chr19	2541656	2542258
chr19	2542338	2542661
chr19	2542849	2543607
chr19	2543979	2544194

chr19	2561736	2562328
chr19	2578202	2579455
chr19	2599324	2599395
chr19	2599626	2599825
chr19	2610743	2610959
chr19	2611072	2611273
chr19	2617775	2617974
chr19	2618293	2618978
chr19	2619877	2620141
chr19	2620649	2622086
chr19	2624436	2624646
chr19	2624862	2625088
chr19	2625415	2625640
chr19	2625777	2625939
chr19	2626698	2627482
chr19	2627928	2628047
chr19	2654107	2654148
chr19	2671838	2672235
chr19	2702459	2702711
chr19	2739730	2740014
chr19	2749498	2749581
chr19	2776541	2776915
chr19	2782784	2783276
chr19	2783497	2783786
chr19	2785052	2785162
chr19	2785516	2786597
chr19	2818435	2818634
chr19	2819268	2819672
chr19	2819858	2820476
chr19	2820703	2820806
chr19	2840796	2842395
chr19	2887587	2887793
chr19	2900035	2900621
chr19	2901628	2902243
chr19	2900900	2901376
chr19	2907764	2907963
chr19	2908350	2909272
chr19	2910735	2911043
chr19	2922780	2923059
chr19	2923307	2923506
chr19	2923784	2924075
chr19	2944344	2944934
chr19	2945148	2945897
chr19	2947664	2947755
chr19	2950177	2950795
chr19	2951338	2951364

chr19	2952883	2953625
chr19	2954441	2954552
chr19	2955638	2955730
chr19	2959601	2959800
chr19	2967349	2967602
chr19	2977146	2977378
chr19	2982532	2982979
chr19	2996682	2997316
chr19	3035000	3035656
chr19	3035900	3036176
chr19	3036330	3037164
chr19	3038498	3038653
chr19	3043777	3043945
chr19	3047129	3047526
chr19	3048426	3048532
chr19	3047939	3048129
chr19	3048639	3048910
chr19	3049033	3049200
chr19	3053604	3053990
chr19	3054506	3055289
chr19	3055482	3056045
chr19	3056317	3056742
chr19	3059467	3059568
chr19	3061107	3061551
chr19	3061659	3063169
chr19	3064808	3064962
chr19	3070938	3071050
chr19	3073865	3073995
chr19	3078804	3078816
chr19	3078954	3079355
chr19	3079564	3079924
chr19	3081366	3081486
chr19	3086727	3087208
chr19	3093498	3094210
chr19	3094591	3095062
chr19	3095870	3096106
chr19	3115743	3116439
chr19	3116562	3117074
chr19	3132201	3132395
chr19	3135616	3135845
chr19	3136010	3137159
chr19	3137297	3137562
chr19	3141486	3142093
chr19	3142505	3142989
chr19	3147289	3147413
chr19	3148281	3148321

chr19	3154473	3155084
chr19	3155388	3155569
chr19	3156000	3156362
chr19	3156799	3157185
chr19	3158163	3158192
chr19	3169778	3169941
chr19	3175770	3177268
chr19	3178381	3179032
chr19	3184444	3184657
chr19	3185251	3186734
chr19	3186927	3187177
chr19	3275558	3275830
chr19	3284568	3284767
chr19	3290682	3290952
chr19	3291048	3291122
chr19	3327320	3327330
chr19	3336560	3336849
chr19	3337683	3337861
chr19	3338298	3338449
chr19	3340762	3340961
chr19	3342847	3343648
chr19	3343883	3344283
chr19	3345166	3345457
chr19	3346647	3347161
chr19	3347384	3347506
chr19	3347569	3347768
chr19	3358521	3359415
chr19	3359588	3360065
chr19	3360312	3360727
chr19	3363446	3363800
chr19	3365502	3366486
chr19	3366643	3368102
chr19	3368335	3369278
chr19	3369391	3369590
chr19	3370094	3370301
chr19	3377659	3378028
chr19	3378861	3379175
chr19	3379298	3380553
chr19	3380902	3381437
chr19	3387941	3388141
chr19	3388803	3389024
chr19	3395311	3395625
chr19	3396653	3396854
chr19	3402596	3402795
chr19	3428308	3428701
chr19	3428919	3429670

chr19	3429900	3430499
chr19	3430639	3431176
chr19	3431272	3431435
chr19	3434618	3434817
chr19	3434945	3435737
chr19	3436403	3436798
chr19	3440483	3440682
chr19	3440800	3441084
chr19	3447058	3447277
chr19	3450177	3450456
chr19	3450654	3450868
chr19	3451112	3451983
chr19	3470443	3470642
chr19	3472541	3472832
chr19	3485372	3485571
chr19	3486787	3487228
chr19	3489895	3490096
chr19	3500062	3500386
chr19	3500454	3500955
chr19	3501067	3501327
chr19	3501572	3501736
chr19	3503604	3503803
chr19	3505402	3505978
chr19	3506227	3507429
chr19	3508164	3508495
chr19	3509926	3510125
chr19	3518106	3518306
chr19	3521195	3521526
chr19	3545273	3545485
chr19	3552259	3552810
chr19	3553791	3553997
chr19	3555818	3556180
chr19	3556367	3557636
chr19	3557786	3558642
chr19	3558776	3559322
chr19	3560232	3560690
chr19	3560894	3563097
chr19	3563220	3564861
chr19	3564943	3565890
chr19	3566471	3566888
chr19	3567095	3567236
chr19	3567610	3567904
chr19	3568505	3569537
chr19	3571194	3571347
chr19	3571844	3572456
chr19	3572908	3573878

chr19	3579907	3580214
chr19	3605751	3605963
chr19	3606454	3607885
chr19	3622609	3622792
chr19	3625770	3625856
chr19	3626104	3626303
chr19	3626421	3627615
chr19	3628038	3628255
chr19	3629389	3629588
chr19	3635589	3635611
chr19	3654567	3654653
chr19	3655410	3655819
chr19	3656020	3656744
chr19	3681198	3681318
chr19	3699313	3699787
chr19	3699995	3700473
chr19	3700872	3701232
chr19	3716679	3716937
chr19	3717672	3717705
chr19	3717892	3719178
chr19	3724301	3724465
chr19	3727695	3727949
chr19	3760926	3762085
chr19	3762244	3762252
chr19	3762391	3763328
chr19	3764171	3764360
chr19	3765772	3765981
chr19	3828173	3828352
chr19	3828812	3828923
chr19	3868095	3869125
chr19	3955157	3955444
chr19	3965654	3965680
chr19	3965476	3965570
chr19	3967188	3967739
chr19	3968172	3968642
chr19	3968938	3971150
chr19	3971614	3972128
chr19	3975488	3975687
chr19	3983436	3983804
chr19	3984323	3985678
chr19	3986110	3987176
chr19	3987272	3988140
chr19	3988754	3989097
chr19	3989209	3990477
chr19	4000270	4000308
chr19	4006418	4006723

chr19	4006985	4007449
chr19	4007578	4008234
chr19	4040467	4040559
chr19	4050480	4051855
chr19	4052031	4052522
chr19	4052674	4053391
chr19	4056440	4056742
chr19	4057447	4061309
chr19	4061419	4061902
chr19	4062179	4062398
chr19	4062603	4062802
chr19	4063666	4065870
chr19	4066307	4067657
chr19	4068641	4068886
chr19	4078373	4078572
chr19	4083657	4083856
chr19	4084099	4084320
chr19	4084575	4084774
chr19	4085365	4085659
chr19	4103906	4104105
chr19	4123207	4124575
chr19	4127483	4127555
chr19	4127794	4127863
chr19	4129617	4129917
chr19	4130257	4130392
chr19	4130683	4130897
chr19	4130971	4132264
chr19	4135387	4135443
chr19	4137681	4137717
chr19	4138240	4138358
chr19	4138609	4138639
chr19	4139301	4139551
chr19	4139648	4139840
chr19	4140088	4140170
chr19	4140356	4140514
chr19	4144526	4144972
chr19	4145286	4145837
chr19	4181436	4182566
chr19	4182781	4183103
chr19	4183868	4184105
chr19	4188413	4188485
chr19	4203573	4203772
chr19	4208209	4209089
chr19	4215192	4215391
chr19	4220631	4220837
chr19	4220978	4221145

chr19	4227818	4227909
chr19	4228999	4229272
chr19	4229940	4230559
chr19	4246774	4247808
chr19	4304562	4304822
chr19	4328771	4329339
chr19	4330845	4331302
chr19	4338008	4339092
chr19	4342178	4342746
chr19	4341424	4341823
chr19	4343071	4343938
chr19	4368149	4370052
chr19	4370439	4370960
chr19	4374141	4375269
chr19	4376188	4376580
chr19	4377939	4378138
chr19	4378422	4378689
chr19	4378923	4379416
chr19	4379737	4380558
chr19	4380807	4381556
chr19	4382034	4382235
chr19	4387892	4388139
chr19	4389848	4390777
chr19	4392713	4392800
chr19	4391394	4392289
chr19	4392969	4393382
chr19	4395617	4395758
chr19	4396209	4396367
chr19	4398372	4398462
chr19	4398540	4398647
chr19	4399191	4399584
chr19	4399665	4400483
chr19	4400835	4400990
chr19	4401128	4401327
chr19	4402162	4402237
chr19	4402540	4403037
chr19	4403211	4403854
chr19	4407723	4407893
chr19	4414106	4414521
chr19	4417134	4417146
chr19	4419150	4419349
chr19	4420194	4421217
chr19	4454603	4455146
chr19	4455464	4455808
chr19	4456044	4457741
chr19	4458618	4458699

chr19	4458097	4458551
chr19	4470411	4470673
chr19	4470864	4471562
chr19	4472235	4473148
chr19	4473290	4473568
chr19	4478985	4479070
chr19	4479601	4479800
chr19	4519719	4520077
chr19	4520252	4520457
chr19	4521951	4522304
chr19	4536316	4536518
chr19	4539589	4539976
chr19	4544873	4545013
chr19	4564438	4564458
chr19	4564529	4564660
chr19	4565377	4565817
chr19	4566015	4566200
chr19	4574511	4574731
chr19	4575078	4575447
chr19	4576271	4576505
chr19	4584351	4584569
chr19	4593772	4594083
chr19	4594334	4594566
chr19	4595040	4595165
chr19	4596930	4597234
chr19	4609761	4610179
chr19	4616627	4616690
chr19	4616976	4617197
chr19	4624579	4624925
chr19	4634937	4635180
chr19	4636094	4636927
chr19	4638676	4638743
chr19	4639098	4639229
chr19	4639671	4640182
chr19	4642031	4642514
chr19	4665512	4665513
chr19	4669563	4670398
chr19	4670732	4671873
chr19	4680063	4680584
chr19	4682075	4682116
chr19	4693346	4694524
chr19	4694627	4695726
chr19	4695834	4697016
chr19	4706587	4706747
chr19	4708664	4708689
chr19	4711563	4711690

chr19	4712306	4712528
chr19	4712978	4713444
chr19	4714148	4714737
chr19	4716761	4717074
chr19	4717250	4717588
chr19	4718048	4718444
chr19	4723169	4723878
chr19	4724136	4724935
chr19	4726160	4726931
chr19	4732889	4733047
chr19	4740486	4740685
chr19	4789779	4790230
chr19	4791093	4791222
chr19	4791749	4792677
chr19	4795203	4795865
chr19	4796251	4796467
chr19	4800477	4800801
chr19	4801411	4801555
chr19	4801151	4801226
chr19	4809555	4809703
chr19	4809938	4810200
chr19	4810318	4810389
chr19	4811452	4811758
chr19	4811931	4812926
chr19	4813057	4813506
chr19	4814467	4814939
chr19	4815435	4815836
chr19	4819591	4819697
chr19	4830345	4832386
chr19	4860710	4860844
chr19	4861469	4861745
chr19	4862024	4862183
chr19	4866113	4867249
chr19	4867474	4868710
chr19	4868833	4869268
chr19	4869490	4871597
chr19	4872310	4872767
chr19	4873025	4873158
chr19	4873244	4873484
chr19	4887403	4887602
chr19	4889379	4889897
chr19	4890089	4890248
chr19	4901943	4902146
chr19	4904448	4904615
chr19	4908330	4908567
chr19	4909408	4911511

chr19	4911583	4911955
chr19	4912198	4912899
chr19	4913769	4913774
chr19	4913877	4914352
chr19	4914591	4915254
chr19	4915844	4916193
chr19	4916416	4916673
chr19	4915381	4915590
chr19	4918471	4918671
chr19	4919972	4920296
chr19	4927006	4927270
chr19	4966952	4966971
chr19	4967610	4968119
chr19	4968261	4968521
chr19	4968984	4969210
chr19	4969348	4969571
chr19	4969927	4970329
chr19	4970558	4970945
chr19	4971244	4971455
chr19	4972494	4972923
chr19	4973206	4973549
chr19	4976241	4976691
chr19	4977373	4977572
chr19	4978149	4978730
chr19	4979892	4980091
chr19	4993761	4993959
chr19	5002085	5002359
chr19	5039076	5039301
chr19	5048357	5048789
chr19	5049063	5049350
chr19	5138752	5138951
chr19	5170659	5170782
chr19	5172125	5172380
chr19	5172783	5173236
chr19	5173405	5173796
chr19	5176503	5177278
chr19	5178369	5178412
chr19	5183581	5183674
chr19	5183813	5184042
chr19	5292969	5293272
chr19	5340126	5340836
chr19	5344099	5344381
chr19	5345707	5345978
chr19	5466738	5466876
chr19	5467121	5467356
chr19	5470161	5470749

chr19	5472303	5472383
chr19	5511659	5511867
chr19	5536476	5537916
chr19	5548129	5548283
chr19	5556430	5556684
chr19	5557219	5558311
chr19	5558421	5558652
chr19	5561474	5561578
chr19	5562769	5563257
chr19	5563547	5564011
chr19	5564292	5564558
chr19	5567560	5567964
chr19	5568413	5568492
chr19	5578069	5578403
chr19	5621744	5622822
chr19	5623129	5623709
chr19	5623978	5624279
chr19	5658087	5658229
chr19	5679936	5680421
chr19	5680776	5681555
chr19	5686775	5687185
chr19	5687392	5687987
chr19	5689444	5689667
chr19	5689923	5690147
chr19	5690262	5691460
chr19	5718558	5719052
chr19	5719822	5721288
chr19	5736463	5736698
chr19	5738457	5738927
chr19	5773028	5773371
chr19	5773626	5773802
chr19	5775399	5775498
chr19	5788622	5788812
chr19	5789886	5790623
chr19	5790775	5791501
chr19	5791628	5791907
chr19	5792988	5793119
chr19	5794483	5794682
chr19	5794852	5794913
chr19	5803652	5804038
chr19	5808861	5809467
chr19	5819337	5819536
chr19	5827346	5829946
chr19	5840322	5840614
chr19	5840905	5841164
chr19	5850079	5850097

chr19	5850419	5850815
chr19	5851148	5851522
chr19	5876917	5877116
chr19	5881474	5881708
chr19	5901895	5902092
chr19	5902421	5904024
chr19	5904186	5904400
chr19	5904888	5905515
chr19	5909556	5909632
chr19	5909885	5910172
chr19	5910336	5910670
chr19	5911654	5911812
chr19	5961456	5961528
chr19	5961639	5961664
chr19	5967949	5968128
chr19	5968558	5969003
chr19	5977251	5978102
chr19	5978292	5978665
chr19	5984042	5984306
chr19	5987650	5987781
chr19	6010859	6011084
chr19	6048769	6048870
chr19	6049041	6049229
chr19	6056543	6056746
chr19	6056857	6058072
chr19	6058155	6058354
chr19	6060269	6060540
chr19	6060869	6061780
chr19	6062177	6062411
chr19	6063404	6063874
chr19	6066694	6066721
chr19	6070648	6070849
chr19	6076680	6076938
chr19	6077065	6077105
chr19	6086333	6086523
chr19	6087809	6087857
chr19	6088081	6088320
chr19	6092266	6092475
chr19	6092648	6092944
chr19	6097081	6097225
chr19	6103191	6103792
chr19	6104638	6105043
chr19	6107060	6107335
chr19	6108747	6109259
chr19	6110105	6110514
chr19	6111395	6111451

chr19	6110879	6111309
chr19	6126361	6126443
chr19	6198999	6199577
chr19	6199928	6200153
chr19	6231636	6231790
chr19	6237449	6237559
chr19	6239576	6239785
chr19	6273595	6273808
chr19	6273940	6274707
chr19	6278902	6279715
chr19	6280294	6280584
chr19	6361249	6361418
chr19	6361577	6362626
chr19	6362779	6362798
chr19	6370515	6370557
chr19	6372310	6372637
chr19	6372785	6373355
chr19	6373589	6374015
chr19	6374140	6374380
chr19	6392313	6392538
chr19	6392646	6392650
chr19	6392986	6393966
chr19	6394166	6394427
chr19	6410291	6410521
chr19	6410857	6410871
chr19	6423024	6423344
chr19	6422622	6422794
chr19	6423830	6424107
chr19	6424242	6424926
chr19	6425629	6426753
chr19	6429384	6429978
chr19	6432739	6432894
chr19	6459177	6460257
chr19	6460399	6460982
chr19	6461466	6461711
chr19	6462690	6463289
chr19	6464246	6464611
chr19	6476830	6477065
chr19	6480675	6480908
chr19	6483572	6484001
chr19	6484539	6484702
chr19	6484861	6484884
chr19	6504622	6504860
chr19	6516336	6516982
chr19	6530553	6531543
chr19	6532240	6532495

chr19	6538155	6538672
chr19	6538957	6539102
chr19	6577372	6577424
chr19	6591370	6591428
chr19	6666647	6666855
chr19	6667729	6667991
chr19	6675050	6675249
chr19	6719391	6719744
chr19	6720258	6720306
chr19	6720372	6720687
chr19	6723024	6724259
chr19	6728218	6728332
chr19	6734971	6735192
chr19	6735571	6737101
chr19	6737338	6737608
chr19	6738022	6738550
chr19	6738668	6739523
chr19	6739670	6741534
chr19	6743916	6745877
chr19	6748615	6748830
chr19	6750174	6750373
chr19	6757651	6757840
chr19	6762272	6762794
chr19	6764115	6764318
chr19	6764424	6764495
chr19	6764600	6764802
chr19	6765497	6768060
chr19	6768271	6768302
chr19	6781498	6781875
chr19	6800847	6801122
chr19	6801248	6802172
chr19	6802495	6802815
chr19	6862706	6862773
chr19	6863429	6863636
chr19	7069054	7069058
chr19	7069419	7070228
chr19	7098664	7099764
chr19	7197386	7198126
chr19	7294056	7294357
chr19	7294579	7294807
chr19	7318489	7318733
chr19	7320051	7320386
chr19	7434033	7434226
chr19	7445324	7445644
chr19	7450842	7451203
chr19	7451440	7451677

chr19	7454868	7455165
chr19	7455645	7456906
chr19	7457824	7457881
chr19	7459687	7460814
chr19	7461115	7461786
chr19	7462637	7463025
chr19	7463583	7464039
chr19	7475047	7475048
chr19	7475176	7475246
chr19	7476022	7476201
chr19	7476539	7476749
chr19	7482332	7482743
chr19	7482885	7483165
chr19	7483361	7483560
chr19	7485882	7485999
chr19	7487049	7487180
chr19	7487275	7487368
chr19	7489626	7490431
chr19	7490804	7491629
chr19	7494015	7494349
chr19	7515497	7516011
chr19	7521821	7522564
chr19	7524140	7524572
chr19	7524696	7526242
chr19	7526547	7526960
chr19	7530262	7530622
chr19	7537155	7537323
chr19	7539732	7540232
chr19	7540460	7540666
chr19	7540868	7541165
chr19	7543344	7543584
chr19	7544017	7544441
chr19	7544597	7545454
chr19	7546322	7546353
chr19	7548124	7548464
chr19	7549130	7549323
chr19	7552339	7552384
chr19	7553462	7554237
chr19	7554774	7554851
chr19	7557011	7557908
chr19	7558423	7558841
chr19	7559191	7559269
chr19	7559636	7560070
chr19	7560147	7560272
chr19	7563811	7563985
chr19	7564679	7565114

chr19	7565472	7566409
chr19	7566633	7566806
chr19	7570024	7570132
chr19	7570664	7570980
chr19	7571221	7571497
chr19	7580603	7580993
chr19	7581182	7581564
chr19	7581865	7581910
chr19	7583549	7583691
chr19	7583847	7583932
chr19	7586792	7587338
chr19	7587527	7587864
chr19	7598109	7599517
chr19	7599709	7601641
chr19	7633207	7633452
chr19	7633592	7633673
chr19	7638356	7638373
chr19	7650232	7650624
chr19	7660533	7660746
chr19	7660976	7661522
chr19	7667964	7668163
chr19	7668303	7669025
chr19	7693793	7695105
chr19	7695773	7696037
chr19	7701452	7701798
chr19	7702045	7702330
chr19	7702503	7702733
chr19	7717391	7717420
chr19	7719337	7719433
chr19	7739957	7739999
chr19	7745020	7745086
chr19	7745160	7745399
chr19	7745653	7746321
chr19	7863749	7864118
chr19	7872043	7872120
chr19	7893990	7894616
chr19	7895098	7895619
chr19	7895910	7896129
chr19	7897843	7899297
chr19	7902476	7903492
chr19	7904687	7904928
chr19	7927056	7927375
chr19	7930408	7930614
chr19	7930841	7931796
chr19	7933088	7933729
chr19	7932684	7932756

chr19	7933877	7935005
chr19	7935961	7936193
chr19	7936409	7936645
chr19	7949016	7949117
chr19	7952803	7954542
chr19	7954843	7954996
chr19	7957914	7958616
chr19	7958961	7959199
chr19	7959338	7959481
chr19	7959706	7960657
chr19	7961730	7961746
chr19	7962207	7962343
chr19	7962498	7963524
chr19	7965539	7965811
chr19	7966526	7966733
chr19	7967014	7970078
chr19	7980897	7983461
chr19	7984122	7985481
chr19	7985818	7986175
chr19	7989810	7990902
chr19	7991082	7991345
chr19	7993520	7993719
chr19	7994511	7994718
chr19	7994948	7995555
chr19	8004634	8004731
chr19	8006126	8006148
chr19	8006424	8006670
chr19	8006893	8007192
chr19	8007562	8007793
chr19	8008113	8008626
chr19	8009272	8009591
chr19	8015998	8016325
chr19	8017393	8018712
chr19	8035124	8035323
chr19	8050619	8051011
chr19	8051337	8051523
chr19	8051746	8051797
chr19	8053218	8053682
chr19	8053908	8055027
chr19	8055606	8055676
chr19	8064005	8064219
chr19	8067114	8067339
chr19	8067712	8067731
chr19	8069563	8070274
chr19	8070694	8071119
chr19	8071573	8071723

chr19	8144573	8144727
chr19	8146972	8147123
chr19	8194892	8195104
chr19	8203682	8203860
chr19	8204180	8204396
chr19	8205039	8205385
chr19	8210449	8210555
chr19	8211485	8211529
chr19	8211742	8211818
chr19	8213954	8214521
chr19	8223788	8223794
chr19	8229164	8229412
chr19	8229896	8229991
chr19	8232885	8233136
chr19	8235285	8235590
chr19	8239460	8239550
chr19	8239852	8240174
chr19	8241137	8241342
chr19	8245688	8245905
chr19	8246251	8246540
chr19	8251705	8251752
chr19	8258672	8258702
chr19	8258931	8259833
chr19	8259967	8260035
chr19	8260160	8260196
chr19	8268650	8268849
chr19	8269064	8269263
chr19	8269398	8269618
chr19	8269700	8269845
chr19	8271887	8272372
chr19	8272733	8275257
chr19	8275517	8275716
chr19	8287761	8288002
chr19	8288252	8288568
chr19	8288991	8289034
chr19	8289143	8289835
chr19	8289992	8290149
chr19	8290266	8290298
chr19	8291564	8291791
chr19	8292624	8293002
chr19	8293635	8293866
chr19	8302673	8302756
chr19	8302909	8303235
chr19	8303821	8304045
chr19	8304713	8304960
chr19	8305142	8305677

chr19	8305918	8306177
chr19	8333529	8333546
chr19	8334290	8334519
chr19	8371887	8372086
chr19	8372972	8373257
chr19	8373383	8373781
chr19	8383609	8383837
chr19	8385340	8387288
chr19	8387419	8387569
chr19	8388411	8388649
chr19	8399475	8400764
chr19	8420149	8420310
chr19	8424016	8424320
chr19	8424808	8424998
chr19	8426454	8426920
chr19	8427134	8428065
chr19	8429761	8430046
chr19	8454610	8454884
chr19	8454999	8455810
chr19	8457168	8457480
chr19	8459023	8459117
chr19	8459297	8460475
chr19	8461408	8461679
chr19	8462119	8462457
chr19	8477479	8477848
chr19	8478222	8478448
chr19	8488006	8488205
chr19	8488486	8489242
chr19	8489435	8489738
chr19	8509412	8510494
chr19	8516560	8516756
chr19	8554189	8554411
chr19	8554756	8554957
chr19	8559474	8559751
chr19	8559878	8560008
chr19	8567782	8567936
chr19	8570279	8570615
chr19	8570920	8571275
chr19	8576351	8577154
chr19	8577420	8579381
chr19	8579458	8579860
chr19	8590030	8590479
chr19	8590825	8592019
chr19	8592810	8592970
chr19	8605260	8605699
chr19	8605818	8605902

chr19	8644137	8644359
chr19	8647028	8647681
chr19	8942597	8943287
chr19	9165338	9165530
chr19	9165832	9166478
chr19	9167571	9167605
chr19	9167744	9167837
chr19	9170286	9170520
chr19	9185918	9186104
chr19	9186672	9186793
chr19	9190443	9190478
chr19	9192864	9192897
chr19	9238779	9238938
chr19	9239191	9239290
chr19	9246967	9247281
chr19	9250832	9250944
chr19	9251085	9251402
chr19	9419807	9421095
chr19	9434170	9434188
chr19	9434430	9435258
chr19	9517501	9517745
chr19	9517907	9518106
chr19	9545941	9546510
chr19	9546651	9546975
chr19	9649199	9649776
chr19	9694791	9694850
chr19	9694958	9695471
chr19	9697401	9697529
chr19	9707479	9708117
chr19	9710459	9710658
chr19	9733168	9733614
chr19	9731681	9732816
chr19	9736509	9736525
chr19	9785382	9786250
chr19	9813269	9813286
chr19	9878828	9879876
chr19	9893006	9893165
chr19	9895005	9895064
chr19	9895327	9896245
chr19	9896428	9897362
chr19	9901376	9901607
chr19	9901716	9901899
chr19	9902084	9902511
chr19	9902679	9903262
chr19	9903471	9904031
chr19	9907065	9907287

chr19	9929180	9929775
chr19	9933443	9933463
chr19	9933705	9933886
chr19	9937912	9938079
chr19	9938208	9938816
chr19	9945472	9945745
chr19	9946000	9946580
chr19	9946691	9946920
chr19	9957533	9957809
chr19	9963702	9963984
chr19	9988433	9988831
chr19	9988949	9989807
chr19	9992567	9992823
chr19	9993606	9993697
chr19	9993783	9993944
chr19	10024003	10024006
chr19	10024090	10024605
chr19	10024765	10025580
chr19	10025729	10025998
chr19	10027908	10028013
chr19	10046664	10047015
chr19	10048051	10048269
chr19	10056519	10056641
chr19	10121578	10121831
chr19	10196632	10197899
chr19	10206336	10206470
chr19	10206949	10207912
chr19	10209931	10210102
chr19	10217020	10217941
chr19	10218021	10218039
chr19	10222559	10222763
chr19	10223004	10223257
chr19	10223393	10223801
chr19	10229173	10229290
chr19	10229664	10230992
chr19	10231416	10231918
chr19	10232260	10232502
chr19	10233722	10234154
chr19	10246852	10247260
chr19	10269939	10270098
chr19	10272697	10272911
chr19	10273592	10273791
chr19	10274410	10274633
chr19	10284086	10284618
chr19	10302230	10302407
chr19	10303937	10304272

chr19	10304906	10305725
chr19	10305873	10306035
chr19	10309387	10309619
chr19	10310839	10311237
chr19	10311450	10311725
chr19	10312045	10312147
chr19	10316066	10316364
chr19	10316595	10316742
chr19	10316826	10316911
chr19	10327336	10327550
chr19	10329216	10329690
chr19	10339999	10340258
chr19	10340890	10341087
chr19	10341214	10342604
chr19	10349622	10349942
chr19	10350902	10351064
chr19	10359220	10359329
chr19	10359476	10359577
chr19	10360720	10360786
chr19	10361030	10361186
chr19	10362266	10362593
chr19	10362733	10363656
chr19	10370125	10370326
chr19	10371700	10371844
chr19	10371509	10371584
chr19	10372733	10373049
chr19	10373375	10373388
chr19	10379909	10380644
chr19	10380757	10382838
chr19	10383260	10384637
chr19	10386325	10386557
chr19	10387470	10387669
chr19	10387773	10388253
chr19	10388592	10388665
chr19	10389460	10389668
chr19	10390426	10390459
chr19	10391103	10391270
chr19	10392588	10392715
chr19	10393032	10393507
chr19	10393764	10393930
chr19	10394275	10394521
chr19	10396022	10396746
chr19	10396963	10398261
chr19	10398851	10399421
chr19	10399551	10402526
chr19	10414752	10414996

chr19	10418233	10418420
chr19	10425511	10425732
chr19	10425952	10427053
chr19	10442829	10442994
chr19	10443151	10443302
chr19	10443390	10444160
chr19	10444452	10445034
chr19	10445219	10445587
chr19	10447174	10447405
chr19	10447842	10447941
chr19	10457133	10457322
chr19	10457489	10457965
chr19	10482768	10482909
chr19	10489642	10489947
chr19	10490615	10491230
chr19	10491469	10492107
chr19	10492832	10492940
chr19	10499480	10499614
chr19	10501181	10501463
chr19	10510441	10510640
chr19	10511093	10511186
chr19	10511516	10511535
chr19	10512259	10512354
chr19	10512874	10513133
chr19	10513260	10513635
chr19	10513858	10514654
chr19	10514789	10515695
chr19	10515813	10515891
chr19	10520284	10520443
chr19	10526239	10526528
chr19	10526695	10527299
chr19	10528318	10528677
chr19	10530049	10530334
chr19	10542424	10542696
chr19	10572062	10572786
chr19	10582539	10582635
chr19	10594633	10594996
chr19	10601145	10601207
chr19	10601563	10601747
chr19	10604633	10604695
chr19	10607515	10607714
chr19	10609469	10609648
chr19	10611006	10611150
chr19	10611281	10611474
chr19	10612333	10614629
chr19	10616191	10616385

chr19	10619096	10619632
chr19	10620011	10620363
chr19	10621072	10621676
chr19	10626209	10626979
chr19	10627183	10629521
chr19	10630198	10630416
chr19	10630690	10631088
chr19	10631301	10632042
chr19	10633441	10633682
chr19	10635134	10635338
chr19	10636251	10636450
chr19	10644243	10645059
chr19	10646154	10647105
chr19	10647189	10647709
chr19	10648333	10648722
chr19	10648923	10649228
chr19	10650119	10650120
chr19	10651244	10651528
chr19	10651976	10652160
chr19	10654234	10655131
chr19	10655327	10655771
chr19	10662269	10662416
chr19	10664217	10668368
chr19	10668662	10668937
chr19	10673068	10673278
chr19	10673895	10674018
chr19	10674450	10674940
chr19	10675174	10675404
chr19	10675681	10676739
chr19	10677130	10677474
chr19	10678660	10678808
chr19	10679172	10679721
chr19	10680056	10680980
chr19	10682868	10683135
chr19	10688842	10689068
chr19	10689182	10689208
chr19	10697290	10698265
chr19	10701798	10702115
chr19	10702441	10702988
chr19	10704219	10704280
chr19	10704666	10704691
chr19	10705717	10705726
chr19	10705805	10705916
chr19	10706142	10706438
chr19	10712805	10712977
chr19	10713154	10713353

chr19	10713503	10713655
chr19	10725426	10725580
chr19	10727025	10727338
chr19	10728035	10729124
chr19	10729259	10729993
chr19	10730379	10731020
chr19	10732594	10732602
chr19	10731448	10731867
chr19	10734282	10734474
chr19	10735059	10736073
chr19	10736224	10736839
chr19	10737313	10737723
chr19	10737810	10737941
chr19	10740963	10741318
chr19	10752636	10752772
chr19	10755251	10755572
chr19	10755706	10756002
chr19	10756261	10756460
chr19	10762297	10762550
chr19	10762720	10762772
chr19	10762932	10763309
chr19	10763372	10763958
chr19	10764162	10764471
chr19	10764916	10765586
chr19	10766441	10766922
chr19	10769980	10770045
chr19	10771183	10771376
chr19	10773744	10773989
chr19	10784407	10784573
chr19	10810732	10810956
chr19	10811557	10811975
chr19	10812331	10812530
chr19	10812737	10813198
chr19	10813494	10813667
chr19	10827801	10828109
chr19	10828629	10829606
chr19	10840712	10840765
chr19	10854556	10854825
chr19	10857507	10857686
chr19	10857935	10857957
chr19	10859761	10859960
chr19	10865309	10865370
chr19	10868890	10869089
chr19	10871189	10871345
chr19	10872857	10873298
chr19	10873743	10873858

chr19	10874097	10874191
chr19	10884420	10884485
chr19	10891110	10891310
chr19	10920855	10921073
chr19	10923568	10923600
chr19	10924765	10924992
chr19	10938734	10938942
chr19	10946060	10947257
chr19	10947375	10947942
chr19	10964106	10964241
chr19	10974392	10974567
chr19	10974634	10975084
chr19	10975478	10975695
chr19	10977088	10977155
chr19	10977232	10977372
chr19	10980769	10981567
chr19	10982432	10983359
chr19	10983547	10983872
chr19	10983998	10984382
chr19	10985003	10985338
chr19	10986006	10986177
chr19	10986841	10987040
chr19	10989683	10989718
chr19	10990417	10990585
chr19	10996775	10996969
chr19	10997385	10997554
chr19	11021382	11021489
chr19	11036878	11037090
chr19	11037700	11037845
chr19	11037989	11038948
chr19	11039024	11039319
chr19	11039495	11040025
chr19	11040149	11040348
chr19	11041894	11042212
chr19	11043693	11043788
chr19	11047266	11047537
chr19	11047722	11047748
chr19	11049812	11051330
chr19	11052654	11052703
chr19	11052973	11053132
chr19	11054577	11054649
chr19	11056778	11056950
chr19	11058331	11058625
chr19	11060705	11061148
chr19	11061634	11061857
chr19	11063145	11063863

chr19	11064577	11065178
chr19	11059010	11059304
chr19	11069521	11069748
chr19	11070518	11071413
chr19	11071547	11072875
chr19	11073148	11073351
chr19	11073478	11073742
chr19	11075055	11075199
chr19	11077160	11077175
chr19	11092958	11093092
chr19	11146191	11146441
chr19	11146892	11147177
chr19	11152732	11152931
chr19	11184971	11185338
chr19	11188005	11188204
chr19	11194351	11195436
chr19	11195732	11197341
chr19	11198351	11198784
chr19	11199842	11201045
chr19	11201253	11201723
chr19	11202134	11202705
chr19	11202808	11204265
chr19	11204385	11204467
chr19	11204588	11206178
chr19	11206711	11207073
chr19	11207281	11207325
chr19	11207647	11207877
chr19	11209072	11209309
chr19	11210165	11210373
chr19	11211340	11211696
chr19	11211798	11212081
chr19	11212190	11212438
chr19	11212934	11213022
chr19	11215122	11215355
chr19	11218371	11218431
chr19	11218947	11218984
chr19	11219168	11219452
chr19	11220848	11221088
chr19	11224636	11224835
chr19	11228546	11228913
chr19	11232324	11232402
chr19	11233503	11233839
chr19	11234548	11234803
chr19	11235296	11235709
chr19	11235982	11235992
chr19	11239488	11239938

chr19	11240921	11241308
chr19	11243764	11243969
chr19	11247997	11248930
chr19	11249340	11249679
chr19	11249927	11250935
chr19	11251071	11251402
chr19	11252213	11252287
chr19	11252860	11253166
chr19	11253426	11254012
chr19	11254126	11255116
chr19	11255210	11256066
chr19	11262671	11263000
chr19	11263505	11263918
chr19	11264150	11264575
chr19	11264794	11265170
chr19	11265269	11265523
chr19	11266200	11266477
chr19	11266854	11266974
chr19	11267128	11267430
chr19	11274078	11274367
chr19	11277743	11277880
chr19	11278362	11279311
chr19	11279501	11279860
chr19	11288299	11288445
chr19	11299942	11300460
chr19	11300583	11301137
chr19	11301345	11301499
chr19	11301608	11301985
chr19	11307672	11309359
chr19	11309962	11310540
chr19	11357709	11357908
chr19	11368539	11368568
chr19	11372718	11373105
chr19	11373247	11374161
chr19	11374644	11374714
chr19	11374954	11375213
chr19	11375432	11376001
chr19	11376257	11376924
chr19	11377336	11377884
chr19	11379220	11379571
chr19	11379849	11380272
chr19	11380583	11380993
chr19	11404255	11404490
chr19	11404784	11404969
chr19	11406324	11406434
chr19	11407082	11407780

chr19	11417929	11417968
chr19	11428286	11428642
chr19	11429862	11430369
chr19	11432697	11432792
chr19	11434281	11435078
chr19	11435390	11435988
chr19	11449852	11450708
chr19	11451042	11451074
chr19	11452659	11453008
chr19	11454991	11455152
chr19	11455388	11455805
chr19	11456128	11457004
chr19	11457340	11457947
chr19	11458306	11458346
chr19	11463811	11463997
chr19	11466066	11466798
chr19	11483663	11484112
chr19	11484504	11484692
chr19	11484761	11485120
chr19	11485257	11485784
chr19	11486855	11487140
chr19	11485909	11486726
chr19	11488098	11488297
chr19	11489157	11489445
chr19	11489954	11490551
chr19	11491348	11491923
chr19	11492161	11494051
chr19	11494254	11494544
chr19	11494755	11495081
chr19	11514843	11515148
chr19	11516722	11517018
chr19	11518447	11518486
chr19	11529094	11529458
chr19	11533065	11533264
chr19	11543150	11543223
chr19	11545489	11545715
chr19	11546310	11547037
chr19	11547449	11547694
chr19	11553587	11553826
chr19	11553978	11554220
chr19	11554638	11555963
chr19	11581910	11582020
chr19	11593116	11593514
chr19	11609940	11610152
chr19	11614914	11615023
chr19	11615692	11615927

chr19	11616139	11616687
chr19	11616841	11617922
chr19	11639512	11639936
chr19	11640190	11640315
chr19	11643117	11643250
chr19	11646471	11646941
chr19	11668087	11668219
chr19	11669438	11669941
chr19	11670125	11670467
chr19	11671092	11671497
chr19	11671922	11671977
chr19	11672179	11672443
chr19	11689220	11689986
chr19	11690115	11690327
chr19	11703587	11703595
chr19	11706143	11706311
chr19	11707917	11708798
chr19	11735813	11735814
chr19	11750046	11750124
chr19	11750665	11751052
chr19	11804646	11805092
chr19	11805226	11805675
chr19	11806177	11806401
chr19	11811178	11811286
chr19	11849026	11849700
chr19	11849967	11850332
chr19	11877338	11878280
chr19	11878564	11878989
chr19	11908243	11908348
chr19	11909568	11909767
chr19	11925072	11925553
chr19	11925728	11926192
chr19	11926373	11926423
chr19	12034828	12036309
chr19	12099233	12099595
chr19	12250582	12251106
chr19	12251349	12251552
chr19	12254398	12254451
chr19	12264165	12264292
chr19	12266854	12267522
chr19	12267725	12267871
chr19	12273875	12274331
chr19	12400057	12400149
chr19	12401108	12401111
chr19	12404530	12405710
chr19	12661647	12662340

chr19	12662596	12662937
chr19	12663009	12663208
chr19	12711809	12711829
chr19	12721716	12722466
chr19	12777154	12777853
chr19	12778184	12778553
chr19	12779416	12779431
chr19	12779618	12781256
chr19	12781733	12781758
chr19	12784279	12784478
chr19	12789251	12789348
chr19	12791281	12791383
chr19	12791479	12792996
chr19	12793523	12793772
chr19	12794611	12794612
chr19	12802813	12802926
chr19	12806955	12807220
chr19	12807522	12808100
chr19	12808371	12808423
chr19	12829374	12829503
chr19	12830280	12830342
chr19	12830881	12834176
chr19	12834564	12835020
chr19	12836513	12836673
chr19	12843303	12843434
chr19	12843548	12843749
chr19	12844118	12844120
chr19	12844480	12844506
chr19	12845229	12846280
chr19	12847252	12847682
chr19	12848034	12848807
chr19	12859895	12860807
chr19	12861180	12861379
chr19	12861595	12861794
chr19	12868642	12868893
chr19	12869581	12869640
chr19	12871694	12871788
chr19	12884268	12884467
chr19	12885072	12885269
chr19	12885374	12886702
chr19	12887855	12888379
chr19	12888513	12889456
chr19	12889905	12890672
chr19	12892303	12897137
chr19	12898457	12898744
chr19	12899103	12901091

chr19	12897308	12898196
chr19	12901238	12902231
chr19	12902336	12904298
chr19	12904502	12905917
chr19	12906116	12906377
chr19	12906455	12907408
chr19	12911709	12913081
chr19	12917129	12917941
chr19	12918475	12918693
chr19	12981010	12981132
chr19	12991864	12992764
chr19	12996494	12996866
chr19	12997147	12997537
chr19	12999415	12999693
chr19	13001035	13001572
chr19	13002017	13002247
chr19	13002353	13002552
chr19	13029485	13029537
chr19	13029804	13030127
chr19	13030309	13030684
chr19	13043957	13044833
chr19	13048162	13049121
chr19	13049433	13050424
chr19	13051158	13051621
chr19	13052491	13054160
chr19	13054426	13054561
chr19	13055285	13056412
chr19	13056563	13058196
chr19	13061679	13061899
chr19	13063128	13063362
chr19	13064450	13064662
chr19	13065576	13065834
chr19	13066943	13067089
chr19	13067463	13068671
chr19	13070395	13070646
chr19	13076066	13077094
chr19	13093812	13094001
chr19	13105912	13106222
chr19	13106842	13107214
chr19	13123852	13124370
chr19	13134062	13134352
chr19	13135260	13135312
chr19	13144937	13145136
chr19	13147847	13148577
chr19	13172048	13172259
chr19	13210110	13210355

chr19	13213357	13213995
chr19	13227184	13227521
chr19	13227628	13228400
chr19	13228480	13228998
chr19	13229244	13230356
chr19	13230448	13230504
chr19	13242802	13243143
chr19	13258353	13258486
chr19	13258896	13258992
chr19	13259915	13261045
chr19	13261226	13265746
chr19	13265902	13266566
chr19	13271218	13271538
chr19	13271698	13271725
chr19	13272478	13273783
chr19	13273889	13276166
chr19	13276329	13276856
chr19	13278055	13278399
chr19	13278596	13279113
chr19	13279398	13279761
chr19	13280355	13281521
chr19	13282717	13283235
chr19	13283392	13283782
chr19	13294262	13294460
chr19	13305220	13305435
chr19	13315384	13316251
chr19	13317956	13319251
chr19	13320571	13320874
chr19	13326668	13327035
chr19	13510113	13510533
chr19	13832329	13832455
chr19	13841568	13841773
chr19	13841837	13842753
chr19	13858268	13858507
chr19	13858789	13859739
chr19	13861243	13861380
chr19	13874837	13874897
chr19	13875002	13876748
chr19	13884502	13884805
chr19	13885299	13886226
chr19	13892240	13892286
chr19	13893481	13893926
chr19	13895718	13895771
chr19	13896321	13896387
chr19	13898391	13899144
chr19	13899428	13900312

chr19	13902662	13902692
chr19	13904278	13904353
chr19	13904714	13906059
chr19	13906235	13908028
chr19	13908549	13909404
chr19	13914265	13914612
chr19	13914808	13915186
chr19	13930588	13930887
chr19	13931106	13931291
chr19	13943160	13943421
chr19	13943530	13944355
chr19	13944716	13945495
chr19	13945611	13953676
chr19	13953845	13955148
chr19	13955326	13956401
chr19	13956663	13958554
chr19	13958736	13958935
chr19	13959132	13959595
chr19	13962992	13964005
chr19	13964070	13964772
chr19	13965125	13966976
chr19	13968825	13970183
chr19	13970476	13970485
chr19	13960505	13962745
chr19	13970709	13971165
chr19	13971313	13972782
chr19	13973219	13973781
chr19	13975768	13976202
chr19	13976455	13977118
chr19	13983006	13983302
chr19	13990684	13990929
chr19	13991248	13991506
chr19	14014140	14014340
chr19	14016343	14017766
chr19	14017896	14018211
chr19	14019455	14019759
chr19	14039902	14040101
chr19	14042821	14043091
chr19	14043203	14043472
chr19	14048661	14049190
chr19	14049354	14049968
chr19	14050035	14050376
chr19	14057232	14057299
chr19	14057434	14057947
chr19	14063159	14064503
chr19	14084361	14084986

chr19	14085587	14085605
chr19	14089566	14089834
chr19	14096944	14097880
chr19	14098108	14098307
chr19	14113789	14114164
chr19	14114711	14115017
chr19	14116066	14116112
chr19	14116561	14117251
chr19	14117485	14118140
chr19	14129428	14129544
chr19	14134318	14134415
chr19	14135505	14135878
chr19	14136340	14137274
chr19	14142192	14143691
chr19	14153682	14154025
chr19	14168645	14168847
chr19	14179092	14179345
chr19	14179636	14179753
chr19	14182510	14182550
chr19	14184391	14184863
chr19	14185369	14185667
chr19	14185771	14185970
chr19	14186264	14186311
chr19	14191600	14192115
chr19	14192466	14192819
chr19	14193167	14193197
chr19	14200468	14201565
chr19	14201966	14203144
chr19	14220335	14220605
chr19	14228138	14228390
chr19	14229032	14229731
chr19	14229851	14230140
chr19	14240819	14241077
chr19	14247018	14247424
chr19	14247644	14247843
chr19	14248139	14248663
chr19	14249406	14250196
chr19	14250680	14251243
chr19	14252073	14252370
chr19	14252477	14252624
chr19	14254608	14254809
chr19	14256776	14257045
chr19	14306784	14306983
chr19	14315556	14316029
chr19	14316282	14317286
chr19	14327777	14328215

chr19	14358561	14360562
chr19	14362181	14362375
chr19	14366894	14367371
chr19	14444495	14444832
chr19	14445033	14445121
chr19	14445256	14445492
chr19	14455410	14455431
chr19	14474210	14474413
chr19	14474904	14474958
chr19	14491541	14491765
chr19	14492115	14492554
chr19	14493606	14493936
chr19	14495401	14495557
chr19	14495972	14496044
chr19	14512722	14512736
chr19	14512817	14513236
chr19	14526420	14526423
chr19	14526565	14526831
chr19	14527566	14528306
chr19	14528895	14529319
chr19	14529749	14530267
chr19	14530406	14530646
chr19	14543915	14544808
chr19	14553204	14553285
chr19	14553512	14553602
chr19	14576492	14576806
chr19	14582625	14582906
chr19	14586199	14586501
chr19	14603117	14603385
chr19	14605376	14606985
chr19	14607335	14607894
chr19	14607957	14608060
chr19	14608431	14609071
chr19	14609554	14609729
chr19	14609903	14610169
chr19	14610271	14610558
chr19	14610642	14610851
chr19	14611841	14611934
chr19	14612299	14612317
chr19	14612796	14612995
chr19	14613248	14613949
chr19	14614509	14614906
chr19	14615744	14617510
chr19	14618175	14619212
chr19	14619973	14620161
chr19	14620764	14622223

chr19	14622336	14622472
chr19	14623344	14623345
chr19	14624423	14624622
chr19	14627759	14629210
chr19	14629470	14630007
chr19	14633454	14634033
chr19	14636515	14636699
chr19	14636875	14637235
chr19	14638234	14638863
chr19	14639167	14639425
chr19	14639908	14640148
chr19	14640485	14640892
chr19	14647067	14647075
chr19	14648013	14648231
chr19	14648393	14648587
chr19	14653393	14653824
chr19	14655482	14655694
chr19	14660177	14660383
chr19	14660648	14661066
chr19	14661360	14662262
chr19	14662418	14662723
chr19	14665977	14666000
chr19	14665141	14665909
chr19	14666619	14666822
chr19	14667114	14667599
chr19	14667737	14668071
chr19	14668188	14668194
chr19	14668453	14668858
chr19	14670048	14670595
chr19	14670865	14671934
chr19	14672209	14672419
chr19	14673094	14673496
chr19	14680817	14680989
chr19	14681410	14681538
chr19	14681701	14682266
chr19	14682431	14682997
chr19	14683151	14683249
chr19	14683332	14683556
chr19	14684706	14685151
chr19	14800293	14801368
chr19	14869741	14869744
chr19	14886308	14886360
chr19	14886573	14886956
chr19	14887257	14887765
chr19	14887963	14888362
chr19	14895773	14896081

chr19	14896393	14896452
chr19	14896748	14896826
chr19	15208805	15208885
chr19	15209553	15209824
chr19	15217773	15218039
chr19	15218301	15219308
chr19	15235254	15236570
chr19	15236959	15236999
chr19	15288481	15288910
chr19	15301416	15301426
chr19	15305388	15305675
chr19	15305748	15306035
chr19	15306111	15306195
chr19	15306406	15306898
chr19	15310560	15312012
chr19	15320251	15320840
chr19	15321443	15321742
chr19	15322120	15322154
chr19	15326845	15326856
chr19	15334490	15334633
chr19	15334702	15334802
chr19	15340179	15340353
chr19	15340432	15340569
chr19	15344525	15345884
chr19	15360154	15360594
chr19	15360980	15361239
chr19	15361413	15362060
chr19	15387419	15387904
chr19	15391575	15391684
chr19	15392215	15392406
chr19	15393397	15393723
chr19	15394852	15395018
chr19	15410745	15410844
chr19	15411389	15411484
chr19	15411625	15412052
chr19	15412409	15412824
chr19	15421275	15421372
chr19	15425838	15425934
chr19	15426317	15426608
chr19	15429093	15429295
chr19	15441715	15442658
chr19	15442885	15443185
chr19	15443450	15443981
chr19	15444497	15444736
chr19	15446561	15446690
chr19	15451702	15451811

chr19	15488414	15488713
chr19	15488897	15490695
chr19	15490845	15491654
chr19	15522304	15522452
chr19	15528709	15528804
chr19	15529131	15529877
chr19	15542365	15542752
chr19	15542861	15543243
chr19	15560544	15560815
chr19	15564371	15564565
chr19	15666341	15666608
chr19	15917437	15917507
chr19	15917669	15917906
chr19	15938992	15939230
chr19	15939595	15939967
chr19	15940204	15940279
chr19	15946510	15946881
chr19	15947157	15948597
chr19	15979847	15979859
chr19	15980358	15980567
chr19	16112777	16112957
chr19	16152276	16152388
chr19	16157768	16157967
chr19	16174682	16174776
chr19	16177462	16179419
chr19	16179608	16180436
chr19	16180515	16181373
chr19	16181930	16182278
chr19	16182651	16182850
chr19	16183328	16183375
chr19	16183750	16184056
chr19	16185640	16185954
chr19	16191032	16191074
chr19	16186452	16190601
chr19	16191290	16191506
chr19	16191881	16191937
chr19	16192137	16192154
chr19	16195851	16196118
chr19	16208986	16209131
chr19	16210149	16210243
chr19	16213082	16213369
chr19	16221411	16222149
chr19	16222697	16223469
chr19	16224261	16224460
chr19	16231708	16232029
chr19	16253947	16254047

chr19	16254480	16254735
chr19	16254833	16255257
chr19	16256213	16256330
chr19	16261186	16261429
chr19	16270034	16270335
chr19	16271904	16273176
chr19	16273888	16274203
chr19	16290983	16291182
chr19	16291408	16291537
chr19	16291654	16291831
chr19	16293457	16293976
chr19	16295795	16296021
chr19	16296201	16296417
chr19	16296506	16296878
chr19	16306196	16306611
chr19	16307534	16307733
chr19	16308078	16309446
chr19	16309693	16309833
chr19	16364952	16365019
chr19	16390430	16390786
chr19	16403686	16403698
chr19	16403957	16404861
chr19	16405399	16405406
chr19	16407918	16408124
chr19	16423477	16423639
chr19	16424149	16424244
chr19	16435279	16436167
chr19	16439478	16439564
chr19	16558024	16558238
chr19	16567507	16567628
chr19	16568067	16568482
chr19	16568865	16569064
chr19	16580773	16580982
chr19	16581647	16582836
chr19	16583100	16583432
chr19	16606739	16606883
chr19	16607263	16608117
chr19	16650857	16651124
chr19	16652596	16653224
chr19	16653364	16653924
chr19	16654010	16654209
chr19	16654563	16654765
chr19	16660096	16660148
chr19	16679606	16680307
chr19	16680555	16680861
chr19	16681936	16681987

chr19	16682101	16683155
chr19	16683426	16684270
chr19	16684514	16684791
chr19	16691357	16691556
chr19	16694849	16694865
chr19	16694954	16695760
chr19	16723164	16723283
chr19	16738048	16740150
chr19	16769877	16771047
chr19	16771151	16772483
chr19	16829224	16829971
chr19	16830205	16830818
chr19	16836862	16837330
chr19	16843714	16843755
chr19	16844522	16845440
chr19	16875609	16876428
chr19	16932120	16932274
chr19	16933868	16933967
chr19	16934208	16934410
chr19	16935458	16935792
chr19	16935973	16936290
chr19	16940129	16940838
chr19	16944783	16944885
chr19	16945830	16946076
chr19	16985085	16985277
chr19	16994327	16995192
chr19	16995952	16996069
chr19	16997054	16998113
chr19	16998494	16998785
chr19	17006981	17007766
chr19	17008047	17009102
chr19	17031453	17031633
chr19	17031759	17032046
chr19	17133292	17133623
chr19	17137508	17137877
chr19	17183472	17183671
chr19	17184169	17184393
chr19	17184894	17185135
chr19	17185450	17185689
chr19	17185762	17185836
chr19	17185940	17186312
chr19	17186432	17187128
chr19	17187239	17187464
chr19	17187687	17187949
chr19	17189507	17189706
chr19	17190473	17190841

chr19	17190954	17191328
chr19	17192360	17192496
chr19	17207671	17207982
chr19	17219415	17219420
chr19	17219575	17220171
chr19	17222529	17222625
chr19	17230418	17230681
chr19	17239980	17240347
chr19	17240481	17240741
chr19	17242668	17243052
chr19	17245723	17245981
chr19	17246188	17247107
chr19	17324809	17325130
chr19	17325347	17326484
chr19	17326629	17326771
chr19	17326986	17327119
chr19	17330812	17331440
chr19	17332191	17332346
chr19	17336405	17337316
chr19	17337422	17337795
chr19	17337979	17338181
chr19	17343792	17343991
chr19	17344635	17345271
chr19	17345598	17346899
chr19	17347129	17347420
chr19	17352658	17353140
chr19	17354576	17359248
chr19	17359360	17359748
chr19	17360077	17360724
chr19	17360936	17361385
chr19	17361816	17362223
chr19	17362742	17362874
chr19	17363200	17363664
chr19	17363989	17364052
chr19	17364609	17364964
chr19	17365500	17365568
chr19	17365716	17365759
chr19	17365839	17366412
chr19	17376257	17376385
chr19	17377428	17377752
chr19	17378223	17378509
chr19	17378938	17379335
chr19	17391819	17391852
chr19	17391943	17393463
chr19	17398530	17398907
chr19	17399588	17399641

chr19	17403329	17403695
chr19	17403807	17404379
chr19	17406901	17406937
chr19	17407030	17407100
chr19	17407430	17407567
chr19	17407743	17408139
chr19	17411221	17411402
chr19	17413300	17415097
chr19	17417896	17417934
chr19	17416682	17417506
chr19	17418534	17418544
chr19	17419583	17420199
chr19	17420319	17420936
chr19	17421207	17421273
chr19	17421618	17421764
chr19	17421894	17422806
chr19	17422947	17423872
chr19	17424086	17424285
chr19	17424491	17424615
chr19	17427250	17427635
chr19	17427712	17429132
chr19	17429559	17429733
chr19	17429883	17430351
chr19	17431817	17432627
chr19	17433013	17433325
chr19	17436307	17436538
chr19	17436763	17438817
chr19	17439013	17440612
chr19	17444762	17446018
chr19	17446134	17446288
chr19	17447032	17448189
chr19	17448512	17449368
chr19	17451435	17451634
chr19	17453156	17453179
chr19	17456537	17456739
chr19	17458943	17459146
chr19	17459571	17460298
chr19	17461889	17462011
chr19	17466349	17466548
chr19	17466874	17466995
chr19	17467870	17468144
chr19	17493888	17494005
chr19	17495019	17495226
chr19	17501269	17501803
chr19	17501965	17502180
chr19	17504654	17504980

chr19	17505690	17505889
chr19	17515039	17519220
chr19	17519677	17520013
chr19	17514764	17514842
chr19	17527050	17527154
chr19	17530379	17530620
chr19	17530860	17531503
chr19	17536186	17536411
chr19	17543796	17543924
chr19	17545807	17545822
chr19	17558649	17559506
chr19	17559712	17559810
chr19	17561080	17561197
chr19	17561301	17562199
chr19	17562645	17562745
chr19	17576902	17577119
chr19	17579985	17581850
chr19	17583509	17583746
chr19	17583844	17584344
chr19	17585867	17587286
chr19	17588104	17588253
chr19	17588448	17588460
chr19	17588648	17588766
chr19	17589044	17589556
chr19	17589798	17590155
chr19	17590302	17590332
chr19	17592342	17592624
chr19	17596075	17596078
chr19	17601794	17601937
chr19	17602013	17602063
chr19	17610231	17610981
chr19	17613465	17613774
chr19	17614808	17614813
chr19	17618501	17619545
chr19	17619974	17620197
chr19	17620505	17620716
chr19	17621162	17621745
chr19	17621821	17623401
chr19	17650329	17650670
chr19	17664263	17664660
chr19	17664728	17665031
chr19	17666022	17667496
chr19	17668445	17668707
chr19	17670387	17670683
chr19	17670771	17671216
chr19	17671320	17671520

chr19	17672190	17672418
chr19	17675996	17676085
chr19	17685128	17685144
chr19	17716599	17717755
chr19	17727162	17727858
chr19	17815740	17815948
chr19	17822420	17822619
chr19	17828570	17828659
chr19	17829991	17830924
chr19	17857942	17858141
chr19	17858552	17858935
chr19	17861649	17861796
chr19	17861911	17862567
chr19	17862653	17862685
chr19	17865544	17865756
chr19	17867811	17868086
chr19	17868474	17868686
chr19	17873339	17873527
chr19	17875525	17877290
chr19	17877957	17877992
chr19	17877453	17877862
chr19	17879349	17879563
chr19	17889266	17890405
chr19	17901464	17901922
chr19	17901987	17902056
chr19	17902712	17902896
chr19	17905207	17907647
chr19	17909459	17909612
chr19	17907808	17908972
chr19	17929792	17929968
chr19	17930413	17930567
chr19	17930910	17931149
chr19	17940308	17940382
chr19	17940952	17941499
chr19	17942037	17942608
chr19	17942717	17942873
chr19	17951949	17953898
chr19	17955480	17955686
chr19	17956060	17956115
chr19	17958326	17958968
chr19	17959524	17960190
chr19	17960264	17960538
chr19	17960772	17961439
chr19	17961630	17961813
chr19	17963325	17963516
chr19	17966817	17966917

chr19	17970743	17971096
chr19	17971164	17972023
chr19	17972310	17972806
chr19	17973078	17973149
chr19	17974814	17975295
chr19	17975514	17975573
chr19	17975706	17975838
chr19	17976090	17976635
chr19	17981263	17981447
chr19	17996377	17996538
chr19	18013488	18014023
chr19	18016382	18016828
chr19	18016915	18017211
chr19	18025814	18026013
chr19	18026637	18027129
chr19	18032373	18032582
chr19	18033268	18033596
chr19	18033804	18034422
chr19	18042297	18044683
chr19	18044788	18044974
chr19	18045815	18046022
chr19	18048471	18048569
chr19	18048905	18049110
chr19	18054827	18055026
chr19	18057313	18057530
chr19	18057630	18058622
chr19	18058833	18059584
chr19	18061085	18061284
chr19	18062746	18063076
chr19	18091755	18091890
chr19	18104881	18105810
chr19	18104480	18104675
chr19	18107536	18107713
chr19	18107939	18109544
chr19	18110317	18110333
chr19	18111078	18111378
chr19	18111459	18112930
chr19	18118519	18118838
chr19	18119024	18119492
chr19	18130384	18130787
chr19	18131017	18131278
chr19	18131788	18132239
chr19	18132425	18133744
chr19	18134119	18134580
chr19	18135578	18135773
chr19	18145609	18145997

chr19	18147822	18148176
chr19	18148542	18149015
chr19	18155662	18155779
chr19	18156732	18156918
chr19	18168835	18169288
chr19	18197646	18197997
chr19	18199478	18200452
chr19	18200673	18201103
chr19	18205057	18205210
chr19	18205568	18205645
chr19	18208363	18208716
chr19	18214552	18215318
chr19	18215520	18215719
chr19	18216983	18217182
chr19	18218676	18218875
chr19	18220493	18221191
chr19	18223218	18223548
chr19	18223691	18223768
chr19	18228387	18229486
chr19	18230339	18230538
chr19	18238768	18238979
chr19	18247307	18248570
chr19	18249188	18249230
chr19	18254005	18254240
chr19	18262528	18263736
chr19	18263960	18264858
chr19	18265355	18265597
chr19	18265843	18266037
chr19	18269211	18269429
chr19	18268798	18268919
chr19	18270180	18270359
chr19	18271237	18272239
chr19	18283743	18284463
chr19	18285772	18285845
chr19	18284628	18285554
chr19	18286178	18286744
chr19	18302362	18303156
chr19	18303228	18303610
chr19	18304047	18304482
chr19	18304751	18305037
chr19	18313691	18314045
chr19	18314352	18314832
chr19	18315291	18316600
chr19	18334841	18335373
chr19	18335521	18336029
chr19	18385561	18386548

chr19	18387197	18387269
chr19	18388306	18388505
chr19	18389881	18390150
chr19	18390559	18390696
chr19	18390773	18391210
chr19	18391326	18391724
chr19	18391933	18392302
chr19	18392869	18394158
chr19	18394288	18394495
chr19	18394799	18395005
chr19	18402316	18403927
chr19	18409699	18409898
chr19	18411320	18411321
chr19	18411526	18411787
chr19	18413797	18414054
chr19	18414639	18414874
chr19	18415021	18415957
chr19	18416072	18416707
chr19	18427897	18427956
chr19	18429809	18429849
chr19	18429938	18430082
chr19	18433100	18433489
chr19	18433660	18433880
chr19	18434189	18434344
chr19	18434475	18435122
chr19	18439438	18440248
chr19	18442876	18442926
chr19	18443451	18443610
chr19	18443890	18444014
chr19	18444181	18444291
chr19	18444442	18444859
chr19	18446670	18446775
chr19	18451074	18452606
chr19	18454432	18455123
chr19	18456713	18456831
chr19	18460344	18460390
chr19	18460723	18460726
chr19	18462201	18462358
chr19	18467113	18467504
chr19	18467701	18467726
chr19	18471796	18472047
chr19	18473894	18474096
chr19	18475162	18475380
chr19	18475632	18476139
chr19	18476987	18477217
chr19	18481006	18481195

chr19	18481753	18482015
chr19	18483120	18483211
chr19	18483858	18484111
chr19	18484541	18485404
chr19	18485540	18485721
chr19	18485971	18486190
chr19	18486395	18486682
chr19	18488411	18488552
chr19	18488680	18489082
chr19	18489145	18489507
chr19	18489609	18489797
chr19	18491075	18491910
chr19	18493385	18493618
chr19	18494337	18494777
chr19	18495227	18495279
chr19	18496019	18496594
chr19	18496955	18497205
chr19	18497389	18497631
chr19	18497933	18498320
chr19	18498851	18498857
chr19	18499101	18499329
chr19	18505762	18505961
chr19	18509449	18509891
chr19	18510103	18510269
chr19	18510351	18510706
chr19	18511070	18511344
chr19	18511489	18511600
chr19	18516393	18516595
chr19	18519448	18520083
chr19	18520628	18520804
chr19	18521032	18521461
chr19	18522233	18522363
chr19	18526741	18527299
chr19	18527421	18528436
chr19	18528612	18529009
chr19	18529125	18529391
chr19	18529673	18530884
chr19	18531305	18531486
chr19	18531756	18531777
chr19	18543697	18545054
chr19	18547758	18548037
chr19	18548174	18549579
chr19	18549767	18550510
chr19	18550770	18551015
chr19	18564718	18564917
chr19	18565084	18565639

chr19	18570342	18571164
chr19	18571469	18571793
chr19	18572922	18573121
chr19	18578369	18578435
chr19	18581775	18581848
chr19	18583791	18584064
chr19	18585835	18585989
chr19	18589331	18589865
chr19	18590581	18590897
chr19	18591079	18591830
chr19	18594704	18594903
chr19	18596561	18597391
chr19	18605234	18605268
chr19	18605859	18607785
chr19	18607934	18608564
chr19	18609326	18609477
chr19	18610752	18611020
chr19	18622638	18622703
chr19	18625531	18625697
chr19	18628923	18629220
chr19	18630774	18632939
chr19	18633461	18633711
chr19	18634198	18634371
chr19	18634660	18634937
chr19	18636060	18636148
chr19	18636417	18636559
chr19	18636807	18637039
chr19	18637363	18637951
chr19	18641629	18641828
chr19	18645708	18645913
chr19	18651327	18651590
chr19	18653512	18654465
chr19	18654630	18655321
chr19	18655528	18655789
chr19	18667841	18668200
chr19	18668302	18668426
chr19	18668554	18668940
chr19	18669209	18669466
chr19	18680849	18681186
chr19	18682019	18682286
chr19	18682544	18683398
chr19	18683644	18683749
chr19	18683913	18684490
chr19	18699069	18701336
chr19	18705346	18705874
chr19	18709887	18709994

chr19	18710066	18710120
chr19	18713473	18713672
chr19	18746533	18746794
chr19	18747039	18747634
chr19	18747780	18748320
chr19	18788574	18788952
chr19	18789194	18789719
chr19	18790227	18790496
chr19	18792068	18792406
chr19	18792525	18792558
chr19	18793327	18794634
chr19	18867339	18867931
chr19	18868087	18868491
chr19	18873701	18874684
chr19	18875153	18875440
chr19	18876533	18876732
chr19	18883493	18883769
chr19	18942065	18943121
chr19	18943236	18943591
chr19	18944348	18944352
chr19	18949856	18950021
chr19	18972821	18973043
chr19	18973240	18974172
chr19	19027663	19027931
chr19	19029395	19029602
chr19	19029708	19030238
chr19	19030865	19031009
chr19	19031131	19031151
chr19	19045507	19046309
chr19	19047942	19047948
chr19	19049873	19050029
chr19	19050169	19052000
chr19	19052246	19052568
chr19	19059454	19059825
chr19	19061117	19061307
chr19	19061733	19061752
chr19	19062036	19062485
chr19	19062646	19064891
chr19	19078073	19078573
chr19	19078926	19079060
chr19	19080213	19080485
chr19	19080671	19081291
chr19	19081394	19081660
chr19	19083314	19083637
chr19	19134156	19134637
chr19	19138555	19138573

chr19	19143860	19145508
chr19	19173799	19173909
chr19	19174103	19174231
chr19	19174707	19175311
chr19	19213941	19214330
chr19	19216218	19216883
chr19	19248711	19249261
chr19	19250398	19250562
chr19	19285188	19285958
chr19	19301885	19302256
chr19	19302476	19303415
chr19	19303693	19304065
chr19	19304290	19304609
chr19	19304790	19305379
chr19	19311811	19311988
chr19	19313820	19314762
chr19	19335741	19336012
chr19	19430063	19430095
chr19	19430367	19430389
chr19	19430749	19430816
chr19	19430921	19432510
chr19	19432874	19433150
chr19	19441341	19441412
chr19	19450533	19450779
chr19	19471211	19471344
chr19	19471767	19472023
chr19	19475677	19476237
chr19	19477174	19477738
chr19	19478192	19478605
chr19	19479872	19479893
chr19	19480086	19480136
chr19	19481505	19482312
chr19	19483400	19483800
chr19	19484029	19484307
chr19	19484575	19484820
chr19	19488110	19489350
chr19	19489923	19490419
chr19	19495264	19496139
chr19	19497215	19497833
chr19	19496735	19497125
chr19	19498186	19498501
chr19	19500171	19500436
chr19	19505467	19505771
chr19	19505932	19506070
chr19	19513867	19514740
chr19	19515238	19515485

chr19	19515913	19516401
chr19	19516772	19517540
chr19	19517681	19517760
chr19	19535319	19535540
chr19	19549098	19549190
chr19	19555493	19555615
chr19	19561795	19561999
chr19	19594052	19594360
chr19	19596447	19596878
chr19	19599002	19599406
chr19	19599882	19600244
chr19	19600384	19600564
chr19	19601115	19601645
chr19	19626386	19626809
chr19	19627039	19627556
chr19	19627633	19627862
chr19	19628373	19628491
chr19	19649109	19649308
chr19	19650502	19651403
chr19	19651657	19652088
chr19	19683871	19684109
chr19	19701577	19701720
chr19	19702471	19702784
chr19	19712783	19713662
chr19	19713936	19714372
chr19	19724193	19724394
chr19	19724798	19724990
chr19	19725253	19725600
chr19	19725911	19726114
chr19	19728757	19729555
chr19	19730443	19730642
chr19	19738215	19740157
chr19	19753992	19754794
chr19	19773657	19775555
chr19	19779645	19780804
chr19	19780911	19780933
chr19	19781161	19781254
chr19	19843320	19843834
chr19	19844032	19844421
chr19	19886731	19887168
chr19	19887585	19887824
chr19	19932052	19933087
chr19	19976775	19977245
chr19	19977380	19977641
chr19	20011393	20011548
chr19	20011811	20012818

chr19	20149760	20150329
chr19	20150600	20150615
chr19	20156658	20156756
chr19	20162508	20162888
chr19	20163145	20163909
chr19	20188940	20189936
chr19	20256013	20256379
chr19	20278080	20278568
chr19	20282402	20282462
chr19	20348525	20349245
chr19	20349478	20349582
chr19	20606334	20606548
chr19	20606775	20607115
chr19	20607552	20607729
chr19	20748119	20748670
chr19	20843928	20844134
chr19	20959096	20959907
chr19	21105745	21105788
chr19	21106025	21106786
chr19	21182378	21182465
chr19	21182956	21183261
chr19	21203161	21203180
chr19	21203381	21204083
chr19	21205926	21206093
chr19	21264634	21264817
chr19	21264970	21265349
chr19	21265520	21265550
chr19	21267173	21267203
chr19	21324485	21324539
chr19	21324827	21325389
chr19	21326663	21326809
chr19	21326912	21327030
chr19	21419806	21419988
chr19	21511526	21512220
chr19	21512441	21512499
chr19	21541689	21542569
chr19	21579563	21579806
chr19	21580073	21580366
chr19	21688112	21689035
chr19	21787083	21787281
chr19	21787722	21788141
chr19	21788264	21788380
chr19	21903163	21903383
chr19	21949908	21950434
chr19	23431997	23432328
chr19	23432569	23433106

chr19	23433450	23433590
chr19	23456262	23456927
chr19	23457144	23457325
chr19	23492678	23492822
chr19	23492978	23493133
chr19	23493355	23493592
chr19	23494112	23494272
chr19	23495258	23495356
chr19	23495500	23495832
chr19	23577295	23577549
chr19	23577741	23578271
chr19	23578483	23578647
chr19	23597898	23598143
chr19	23598254	23598590
chr19	23598919	23599147
chr19	23653597	23653761
chr19	23663870	23663971
chr19	23703616	23703620
chr19	23703789	23704364
chr19	23704563	23704672
chr19	23704969	23705672
chr19	23714269	23714439
chr19	23719990	23720064
chr19	23730373	23731217
chr19	23731842	23732688
chr19	23732751	23733410
chr19	23737268	23737279
chr19	23737665	23737948
chr19	23744094	23744426
chr19	23744574	23744667
chr19	23869348	23870059
chr19	23940948	23941735
chr19	23945528	23945651
chr19	23945818	23946621
chr19	23952450	23952458
chr19	23960328	23960538
chr19	23970062	23970364
chr19	23971213	23971446
chr19	24084521	24084564
chr19	24097312	24097549
chr19	24097715	24098225
chr19	24140793	24140936
chr19	24215925	24216124
chr19	24216277	24217239
chr19	24234254	24234298
chr19	24235633	24235692

chr19	24237436	24237806
chr19	24242300	24242357
chr19	24243298	24243497
chr19	24256568	24256885
chr19	24257021	24257171
chr19	24269951	24270473
chr19	24614246	24614562
chr19	24628595	24629067
chr19	27731790	27734969
chr19	27735125	27740427
chr19	27756000	27756358
chr19	27760378	27760648
chr19	27764477	27764669
chr19	27769118	27769338
chr19	27789658	27789798
chr19	27890014	27890372
chr19	28283885	28284203
chr19	28284527	28284726
chr19	28284890	28285550
chr19	29703860	29704112
chr19	29704321	29704456
chr19	30096156	30096325
chr19	30096591	30097451
chr19	30137020	30137227
chr19	30155130	30156929
chr19	30158634	30158699
chr19	30158762	30158935
chr19	30159105	30159813
chr19	30160906	30160999
chr19	30177782	30177811
chr19	30178025	30178241
chr19	30178683	30179092
chr19	30182210	30182272
chr19	30204802	30204813
chr19	30205034	30205066
chr19	30205437	30205478
chr19	30205728	30207502
chr19	30301497	30301786
chr19	30301864	30302527
chr19	30303042	30303780
chr19	30331724	30331925
chr19	30331995	30332118
chr19	30335332	30335656
chr19	30336072	30337028
chr19	30364157	30364548
chr19	30431870	30432069

chr19	30432276	30432287
chr19	30432645	30433819
chr19	31210300	31210916
chr19	31618354	31618553
chr19	31643896	31644054
chr19	31705416	31705501
chr19	31840069	31840949
chr19	31841106	31841210
chr19	31841404	31841692
chr19	31899644	31899775
chr19	32834716	32835130
chr19	32836237	32837237
chr19	32845612	32845638
chr19	32854273	32854411
chr19	32864136	32864345
chr19	32864540	32864797
chr19	32864958	32865189
chr19	32895113	32895429
chr19	32895572	32897419
chr19	33071290	33072579
chr19	33072685	33072932
chr19	33090634	33090748
chr19	33096006	33096049
chr19	33111954	33112360
chr19	33145662	33146140
chr19	33146410	33146465
chr19	33164928	33166505
chr19	33167066	33167967
chr19	33168699	33168898
chr19	33181334	33181385
chr19	33181560	33181759
chr19	33182284	33182482
chr19	33182730	33183844
chr19	33344994	33345193
chr19	33358656	33358971
chr19	33456699	33456922
chr19	33461436	33461556
chr19	33462245	33462861
chr19	33463199	33463909
chr19	33466905	33466915
chr19	33496047	33496117
chr19	33496344	33496432
chr19	33501792	33501893
chr19	33502156	33502482
chr19	33515115	33515197
chr19	33523014	33523169

chr19	33531201	33531836
chr19	33532094	33533472
chr19	33538170	33538352
chr19	33538518	33538908
chr19	33539995	33540047
chr19	33541832	33542122
chr19	33543155	33543278
chr19	33543600	33543722
chr19	33544843	33545042
chr19	33545383	33545387
chr19	33546018	33546317
chr19	33548288	33548548
chr19	33548792	33548855
chr19	33549148	33549191
chr19	33550068	33550164
chr19	33554374	33554464
chr19	33554803	33555664
chr19	33555827	33556368
chr19	33556875	33556980
chr19	33560449	33560510
chr19	33562757	33563428
chr19	33565853	33566289
chr19	33567933	33568198
chr19	33568609	33569123
chr19	33569336	33569457
chr19	33569741	33569956
chr19	33571766	33572542
chr19	33627267	33627466
chr19	33667080	33667770
chr19	33668241	33668482
chr19	33669322	33669585
chr19	33674793	33674929
chr19	33685421	33685720
chr19	33685839	33686343
chr19	33740394	33740930
chr19	33741046	33741243
chr19	33764412	33765303
chr19	33783391	33784291
chr19	33784657	33784952
chr19	33785483	33785771
chr19	33787851	33788065
chr19	33788438	33789785
chr19	33789924	33790271
chr19	33793017	33793461
chr19	33793605	33794033
chr19	33794146	33794900

chr19	33817950	33818189
chr19	33821561	33821724
chr19	33830615	33831002
chr19	33841234	33841936
chr19	33849284	33849668
chr19	33857963	33857993
chr19	33859212	33859467
chr19	33860166	33860372
chr19	33861506	33862824
chr19	33862909	33863190
chr19	33863960	33865480
chr19	33867387	33868114
chr19	33876106	33876305
chr19	33884449	33885392
chr19	33964420	33964733
chr19	34012270	34012642
chr19	34013162	34013370
chr19	34209015	34209085
chr19	34285726	34285963
chr19	34286196	34288709
chr19	34307093	34307286
chr19	34310557	34310921
chr19	34311105	34312449
chr19	34312809	34312882
chr19	34313220	34313365
chr19	34395588	34395838
chr19	34396031	34396067
chr19	34396163	34397045
chr19	34397190	34397799
chr19	34625048	34625226
chr19	34625333	34626061
chr19	34626126	34626590
chr19	34649895	34650163
chr19	34662429	34663278
chr19	34663398	34664692
chr19	34668336	34669174
chr19	34679496	34679588
chr19	34723116	34723138
chr19	34723412	34723434
chr19	34724191	34724421
chr19	34725460	34725481
chr19	34744563	34744971
chr19	34745151	34745438
chr19	34745632	34746205
chr19	34752994	34753651
chr19	34756221	34756429

chr19	34758751	34758950
chr19	34760917	34761495
chr19	34783476	34783623
chr19	34783744	34783901
chr19	34848978	34849003
chr19	34849903	34851305
chr19	34851399	34851930
chr19	34852007	34852102
chr19	34853740	34853798
chr19	34853936	34854211
chr19	34855357	34857356
chr19	34858807	34859060
chr19	34864137	34864262
chr19	34894080	34894279
chr19	34894817	34895906
chr19	34899224	34899429
chr19	34907043	34907193
chr19	34917239	34917471
chr19	34918005	34918377
chr19	34918494	34918760
chr19	34918991	34920145
chr19	34921904	34921979
chr19	34966185	34966356
chr19	34972053	34972649
chr19	34972841	34974138
chr19	34989373	34989637
chr19	34989829	34990196
chr19	34992318	34992595
chr19	35158281	35158465
chr19	35158585	35158705
chr19	35167928	35169066
chr19	35224202	35225457
chr19	35225584	35225969
chr19	35263335	35264382
chr19	35350953	35351405
chr19	35411490	35411566
chr19	35417582	35417656
chr19	35417848	35418350
chr19	35454344	35454910
chr19	35487813	35488012
chr19	35489107	35489114
chr19	35489904	35492698
chr19	35492854	35492977
chr19	35493337	35493546
chr19	35494808	35495010
chr19	35496970	35497295

chr19	35497540	35498088
chr19	35520676	35520875
chr19	35521607	35522351
chr19	35526149	35526225
chr19	35531920	35532451
chr19	35542292	35542298
chr19	35559011	35559284
chr19	35579832	35580047
chr19	35580995	35581778
chr19	35586845	35587567
chr19	35587630	35587737
chr19	35589166	35590316
chr19	35598657	35598868
chr19	35600155	35600603
chr19	35600793	35601071
chr19	35601827	35602098
chr19	35603760	35603959
chr19	35604484	35604642
chr19	35605435	35608383
chr19	35608541	35610511
chr19	35610634	35612379
chr19	35612990	35617028
chr19	35617302	35617643
chr19	35618063	35618945
chr19	35619193	35619614
chr19	35626262	35626461
chr19	35633725	35633954
chr19	35644837	35646537
chr19	35646661	35646860
chr19	35654230	35654264
chr19	35659322	35660621
chr19	35704514	35704574
chr19	35705153	35705365
chr19	35738405	35740708
chr19	35749108	35749361
chr19	35754853	35755099
chr19	35757819	35759035
chr19	35759159	35759727
chr19	35760235	35761045
chr19	35809059	35809203
chr19	35809307	35809913
chr19	35810338	35810745
chr19	35895852	35896013
chr19	35927435	35927463
chr19	35927735	35928046
chr19	35931259	35931465

chr19	35939362	35939933
chr19	35958037	35958323
chr19	35984550	35984593
chr19	35986129	35986328
chr19	35998954	35999153
chr19	35999981	36001926
chr19	36003107	36003240
chr19	36003908	36005741
chr19	36008059	36008217
chr19	36008676	36010961
chr19	36021164	36021389
chr19	36035800	36037341
chr19	36103179	36104325
chr19	36119016	36120306
chr19	36126008	36126255
chr19	36133686	36135367
chr19	36135474	36135997
chr19	36136374	36136480
chr19	36137740	36137969
chr19	36138813	36139981
chr19	36140238	36140459
chr19	36144016	36144349
chr19	36144475	36144588
chr19	36164792	36164862
chr19	36171271	36171470
chr19	36191409	36191582
chr19	36192792	36193294
chr19	36193994	36194247
chr19	36195930	36196129
chr19	36197362	36197445
chr19	36200959	36201086
chr19	36202031	36202337
chr19	36206776	36210246
chr19	36210461	36210660
chr19	36212734	36213178
chr19	36231028	36231355
chr19	36231690	36232138
chr19	36232433	36232872
chr19	36235384	36235584
chr19	36235876	36236366
chr19	36236555	36237123
chr19	36237213	36237214
chr19	36237415	36237659
chr19	36238677	36238835
chr19	36239053	36239162
chr19	36239495	36240261

chr19	36240368	36240530
chr19	36246116	36246544
chr19	36246720	36248099
chr19	36248296	36250121
chr19	36266306	36267099
chr19	36328722	36328786
chr19	36373988	36373994
chr19	36380130	36380400
chr19	36389041	36391425
chr19	36391575	36392151
chr19	36392284	36392806
chr19	36393848	36394367
chr19	36402672	36402814
chr19	36422148	36422256
chr19	36425044	36425213
chr19	36426294	36427749
chr19	36427827	36427834
chr19	36484311	36484352
chr19	36485453	36486892
chr19	36494961	36495184
chr19	36498838	36499630
chr19	36499841	36500911
chr19	36502803	36503078
chr19	36504503	36505127
chr19	36505357	36506275
chr19	36513727	36514134
chr19	36520141	36520327
chr19	36527534	36527695
chr19	36531666	36532337
chr19	36532634	36533194
chr19	36533312	36533657
chr19	36537679	36537715
chr19	36540103	36540256
chr19	36540547	36540725
chr19	36544193	36546588
chr19	36546838	36547114
chr19	36547249	36547461
chr19	36547605	36547990
chr19	36561194	36561406
chr19	36561605	36561906
chr19	36589999	36590112
chr19	36592961	36593316
chr19	36596468	36596797
chr19	36603298	36603537
chr19	36604672	36607306
chr19	36607621	36607720

chr19	36607934	36607937
chr19	36610902	36611101
chr19	36617698	36618629
chr19	36618964	36619366
chr19	36621794	36622097
chr19	36622193	36622573
chr19	36622921	36623502
chr19	36623675	36623865
chr19	36624134	36624295
chr19	36625029	36625130
chr19	36626642	36626713
chr19	36626993	36627867
chr19	36628130	36628375
chr19	36629760	36630764
chr19	36631017	36632210
chr19	36632680	36633231
chr19	36639034	36639306
chr19	36640033	36640475
chr19	36657278	36657551
chr19	36661555	36661833
chr19	36661944	36661971
chr19	36672509	36672702
chr19	36673383	36673551
chr19	36681418	36681814
chr19	36704530	36706253
chr19	36822364	36822539
chr19	36822919	36823131
chr19	36823345	36823353
chr19	36869382	36870669
chr19	36908703	36910043
chr19	36979684	36981112
chr19	36981360	36981596
chr19	37018992	37019567
chr19	37019712	37019920
chr19	37020211	37020248
chr19	37020480	37020500
chr19	37063803	37064184
chr19	37064292	37065225
chr19	37068723	37068815
chr19	37095446	37096957
chr19	37157268	37158111
chr19	37177766	37178022
chr19	37178482	37178889
chr19	37236772	37236921
chr19	37237126	37237196
chr19	37262288	37263015

chr19	37263114	37263658
chr19	37264043	37264485
chr19	37287981	37288895
chr19	37328823	37330055
chr19	37339770	37340287
chr19	37340587	37341145
chr19	37341349	37341704
chr19	37341869	37342134
chr19	37406599	37407649
chr19	37408027	37408260
chr19	37463473	37463972
chr19	37464134	37464622
chr19	37568687	37569520
chr19	37663331	37663440
chr19	37663959	37664024
chr19	37701260	37701818
chr19	37708554	37709445
chr19	37712041	37712457
chr19	37742138	37742615
chr19	37742842	37743058
chr19	37784554	37784869
chr19	37803357	37803690
chr19	37804266	37804413
chr19	37807719	37808574
chr19	37808786	37809197
chr19	37812257	37812343
chr19	37825474	37826040
chr19	37861252	37861852
chr19	37862033	37862401
chr19	37957430	37958644
chr19	37959781	37960860
chr19	37996863	37998206
chr19	37998614	37998649
chr19	38042056	38042322
chr19	38084829	38085696
chr19	38085921	38086301
chr19	38145654	38147425
chr19	38209890	38210645
chr19	38210991	38211027
chr19	38221161	38221414
chr19	38251528	38251903
chr19	38252275	38252560
chr19	38253961	38254638
chr19	38269272	38269631
chr19	38269785	38270762
chr19	38345378	38345473

chr19	38345580	38345774
chr19	38346041	38346211
chr19	38356655	38356826
chr19	38365412	38365472
chr19	38389671	38389693
chr19	38394186	38394498
chr19	38394623	38394787
chr19	38396522	38398571
chr19	38399383	38399506
chr19	38406055	38406070
chr19	38417910	38418158
chr19	38421011	38421265
chr19	38420670	38420758
chr19	38421853	38423033
chr19	38423171	38423441
chr19	38423572	38424057
chr19	38457053	38457623
chr19	38464747	38464778
chr19	38467209	38467290
chr19	38467814	38468027
chr19	38468643	38469112
chr19	38469959	38471056
chr19	38471392	38471435
chr19	38469366	38469706
chr19	38471713	38471860
chr19	38476247	38476329
chr19	38477051	38477557
chr19	38478077	38478123
chr19	38478801	38479099
chr19	38479470	38480684
chr19	38480792	38481626
chr19	38481941	38481946
chr19	38482221	38482528
chr19	38486739	38487001
chr19	38488361	38488625
chr19	38489006	38489293
chr19	38489678	38490623
chr19	38491979	38492359
chr19	38493728	38496031
chr19	38498900	38499296
chr19	38502700	38502920
chr19	38505817	38505994
chr19	38506157	38506333
chr19	38514025	38514247
chr19	38517741	38518021
chr19	38523581	38523695

chr19	38530508	38530811
chr19	38539792	38539796
chr19	38540153	38540164
chr19	38550453	38550851
chr19	38551944	38552021
chr19	38552226	38552365
chr19	38561496	38561606
chr19	38585202	38585209
chr19	38586672	38586741
chr19	38599386	38599616
chr19	38607022	38607087
chr19	38624766	38624965
chr19	38625249	38625427
chr19	38626194	38626417
chr19	38626560	38626651
chr19	38635796	38635810
chr19	38636513	38636712
chr19	38645414	38645649
chr19	38650887	38651191
chr19	38652650	38652716
chr19	38658365	38658564
chr19	38663455	38663964
chr19	38664251	38665093
chr19	38666816	38667122
chr19	38700315	38700738
chr19	38710078	38710347
chr19	38714283	38715397
chr19	38717162	38717364
chr19	38719254	38719691
chr19	38725701	38725982
chr19	38729609	38729965
chr19	38731384	38731915
chr19	38734114	38734953
chr19	38735184	38735394
chr19	38735886	38736831
chr19	38737017	38737336
chr19	38739368	38739396
chr19	38739596	38739670
chr19	38741907	38743127
chr19	38743243	38743372
chr19	38743727	38744188
chr19	38746462	38747844
chr19	38748899	38749249
chr19	38749613	38749812
chr19	38750512	38750734
chr19	38751634	38751750

chr19	38753128	38753270
chr19	38753376	38753607
chr19	38753858	38754010
chr19	38754378	38758402
chr19	38758661	38758910
chr19	38759365	38759516
chr19	38759662	38760005
chr19	38760074	38760503
chr19	38761309	38761431
chr19	38767924	38768163
chr19	38768340	38768453
chr19	38768922	38768998
chr19	38769197	38769334
chr19	38773802	38774001
chr19	38774533	38774742
chr19	38776076	38776100
chr19	38780224	38780317
chr19	38780844	38780848
chr19	38781105	38781755
chr19	38785485	38786150
chr19	38786238	38787694
chr19	38787901	38787951
chr19	38788059	38788105
chr19	38788191	38788782
chr19	38790163	38791366
chr19	38791511	38792033
chr19	38792439	38792697
chr19	38792979	38793403
chr19	38793633	38795736
chr19	38795895	38796099
chr19	38796661	38797064
chr19	38805350	38806611
chr19	38806805	38807966
chr19	38809172	38811495
chr19	38812072	38812369
chr19	38812776	38813038
chr19	38814328	38814569
chr19	38825816	38825931
chr19	38826073	38826200
chr19	38826423	38826965
chr19	38827130	38828209
chr19	38828537	38828736
chr19	38830080	38830291
chr19	38833199	38833324
chr19	38833624	38833823
chr19	38852830	38853238

chr19	38853359	38854028
chr19	38864826	38865141
chr19	38865351	38866753
chr19	38866979	38867483
chr19	38868334	38868584
chr19	38868936	38869140
chr19	38878208	38880344
chr19	38880769	38881084
chr19	38892882	38893005
chr19	38893161	38893470
chr19	38893783	38894389
chr19	38894505	38894817
chr19	38908134	38908480
chr19	38908607	38908941
chr19	39035232	39035353
chr19	39035732	39035915
chr19	39036179	39036377
chr19	39041169	39041333
chr19	39045717	39045915
chr19	39047427	39047554
chr19	39047738	39047794
chr19	39047878	39047954
chr19	39049169	39049523
chr19	39049819	39050412
chr19	39050616	39051479
chr19	39051743	39051758
chr19	39054491	39054571
chr19	39055459	39055824
chr19	39055952	39056915
chr19	39064589	39064876
chr19	39066031	39066308
chr19	39067068	39067377
chr19	39067655	39067753
chr19	39068227	39068446
chr19	39069074	39069288
chr19	39069475	39070816
chr19	39071185	39071279
chr19	39071415	39071787
chr19	39072136	39072470
chr19	39076332	39076501
chr19	39086985	39087394
chr19	39099852	39100090
chr19	39109083	39109649
chr19	39110523	39110939
chr19	39109757	39110066
chr19	39111722	39111876

chr19	39121430	39121460
chr19	39122123	39122213
chr19	39122751	39122915
chr19	39124025	39124400
chr19	39124491	39125147
chr19	39126959	39127584
chr19	39127861	39128500
chr19	39128815	39129098
chr19	39129255	39129579
chr19	39129645	39130154
chr19	39130963	39131830
chr19	39136323	39136542
chr19	39137376	39137601
chr19	39137815	39137884
chr19	39138051	39139013
chr19	39139386	39142370
chr19	39143186	39144042
chr19	39144989	39145476
chr19	39146022	39146046
chr19	39146787	39147645
chr19	39147841	39148059
chr19	39148240	39148948
chr19	39149167	39149705
chr19	39149849	39150482
chr19	39151287	39151628
chr19	39151771	39151917
chr19	39152548	39153092
chr19	39153222	39153514
chr19	39154129	39154333
chr19	39154479	39154871
chr19	39155078	39155948
chr19	39156181	39157458
chr19	39158462	39158585
chr19	39157679	39158110
chr19	39158771	39159411
chr19	39159601	39159717
chr19	39160126	39160191
chr19	39160365	39160564
chr19	39160717	39161532
chr19	39162943	39163317
chr19	39165048	39166647
chr19	39166983	39167168
chr19	39167285	39167617
chr19	39168288	39169038
chr19	39169208	39170602
chr19	39170757	39170968

chr19	39171034	39171239
chr19	39171348	39171868
chr19	39172263	39172452
chr19	39172913	39174277
chr19	39175707	39177461
chr19	39177950	39178209
chr19	39178831	39180369
chr19	39180845	39181276
chr19	39181469	39184992
chr19	39174461	39175489
chr19	39185108	39186114
chr19	39187196	39187471
chr19	39188184	39190623
chr19	39194685	39194980
chr19	39196988	39197323
chr19	39198631	39198830
chr19	39199931	39200145
chr19	39203335	39203565
chr19	39205693	39206108
chr19	39206600	39206842
chr19	39216593	39216792
chr19	39222812	39223171
chr19	39223345	39223622
chr19	39224178	39224377
chr19	39224953	39226260
chr19	39226634	39227619
chr19	39227723	39228469
chr19	39228619	39228840
chr19	39232093	39232292
chr19	39236590	39236833
chr19	39260031	39261933
chr19	39262036	39262432
chr19	39263256	39263402
chr19	39263470	39264158
chr19	39264361	39264934
chr19	39265060	39266952
chr19	39267928	39268189
chr19	39272191	39272418
chr19	39273282	39273434
chr19	39277242	39278080
chr19	39278332	39278656
chr19	39278809	39284613
chr19	39285004	39285140
chr19	39286001	39286347
chr19	39288372	39288677
chr19	39290123	39290812

chr19	39293392	39293525
chr19	39293748	39293776
chr19	39294875	39295036
chr19	39296075	39296415
chr19	39297411	39297610
chr19	39297844	39297957
chr19	39298371	39298704
chr19	39299007	39299219
chr19	39301202	39301371
chr19	39304809	39305008
chr19	39312911	39313172
chr19	39319299	39320079
chr19	39321497	39322462
chr19	39322611	39322853
chr19	39324103	39324264
chr19	39325987	39326144
chr19	39326302	39327007
chr19	39327457	39327839
chr19	39328378	39328728
chr19	39328935	39329209
chr19	39329335	39329534
chr19	39331714	39332005
chr19	39334478	39334679
chr19	39335362	39335584
chr19	39337230	39337400
chr19	39337508	39337732
chr19	39338277	39338512
chr19	39339028	39339359
chr19	39339743	39340032
chr19	39340170	39340665
chr19	39341289	39343909
chr19	39344115	39344427
chr19	39344649	39344667
chr19	39353118	39353501
chr19	39354169	39354260
chr19	39354701	39354902
chr19	39355461	39355872
chr19	39357555	39358064
chr19	39359163	39359303
chr19	39359485	39361124
chr19	39368334	39369444
chr19	39388546	39388609
chr19	39389418	39390248
chr19	39390606	39391085
chr19	39411508	39411660
chr19	39412487	39412656

chr19	39417878	39418072
chr19	39420651	39420858
chr19	39421026	39421434
chr19	39421658	39421962
chr19	39422061	39422319
chr19	39438292	39438491
chr19	39452801	39452816
chr19	39458822	39458954
chr19	39461420	39461516
chr19	39464817	39465110
chr19	39465322	39465647
chr19	39465726	39467626
chr19	39468608	39468638
chr19	39482478	39482620
chr19	39502484	39502572
chr19	39521462	39524560
chr19	39542837	39542962
chr19	39554838	39555048
chr19	39563184	39563500
chr19	39564110	39564330
chr19	39564578	39565081
chr19	39567029	39567188
chr19	39569097	39570212
chr19	39573367	39573809
chr19	39574426	39574762
chr19	39576576	39576591
chr19	39578858	39579072
chr19	39584667	39585290
chr19	39588597	39588793
chr19	39597184	39597447
chr19	39598770	39598947
chr19	39601416	39601615
chr19	39601772	39603025
chr19	39603277	39603400
chr19	39603725	39603924
chr19	39605566	39605753
chr19	39610398	39610528
chr19	39612391	39612795
chr19	39612950	39613338
chr19	39615377	39616139
chr19	39617330	39617412
chr19	39616406	39617101
chr19	39644468	39644580
chr19	39656855	39656939
chr19	39657080	39657261
chr19	39657531	39658661

chr19	39673677	39673890
chr19	39683773	39683972
chr19	39684525	39684726
chr19	39686164	39686224
chr19	39686320	39686832
chr19	39687648	39687996
chr19	39737828	39738997
chr19	39755198	39756538
chr19	39790022	39790221
chr19	39797890	39798113
chr19	39798645	39798981
chr19	39803287	39803486
chr19	39810808	39811417
chr19	39824855	39825093
chr19	39828066	39828283
chr19	39828450	39828649
chr19	39830692	39831040
chr19	39831439	39832572
chr19	39833058	39833900
chr19	39873046	39873245
chr19	39880760	39881054
chr19	39881128	39881877
chr19	39882075	39882518
chr19	39882892	39883240
chr19	39883664	39884020
chr19	39887131	39887465
chr19	39888237	39889966
chr19	39890360	39890538
chr19	39890735	39890971
chr19	39891506	39891665
chr19	39891887	39894370
chr19	39894580	39896026
chr19	39896264	39900127
chr19	39900276	39902831
chr19	39903152	39904512
chr19	39919279	39919478
chr19	39919816	39919924
chr19	39920136	39920500
chr19	39920721	39920830
chr19	39921071	39922530
chr19	39923230	39923427
chr19	39924471	39924693
chr19	39925137	39925588
chr19	39925662	39927159
chr19	39928939	39930071
chr19	39930864	39931775

chr19	39934161	39934321
chr19	39935248	39935695
chr19	39935902	39936727
chr19	39937567	39937761
chr19	39940990	39941093
chr19	39970819	39972448
chr19	39972781	39972946
chr19	39983208	39983472
chr19	39987155	39987197
chr19	39988627	39988896
chr19	39989838	39990124
chr19	39990858	39991057
chr19	39993303	39994119
chr19	39996997	39998238
chr19	39998365	39998660
chr19	40005069	40005268
chr19	40006241	40006554
chr19	40006976	40007205
chr19	40011417	40011504
chr19	40022861	40022866
chr19	40023088	40023486
chr19	40023649	40023849
chr19	40030319	40030853
chr19	40034053	40034680
chr19	40034850	40035587
chr19	40048313	40048316
chr19	40048398	40048776
chr19	40168478	40168521
chr19	40314506	40314515
chr19	40314804	40315221
chr19	40323295	40324246
chr19	40331245	40331322
chr19	40334730	40334753
chr19	40334981	40335062
chr19	40335858	40335950
chr19	40336249	40337354
chr19	40369474	40369723
chr19	40415287	40415577
chr19	40415720	40415980
chr19	40439501	40439564
chr19	40452445	40452556
chr19	40474729	40474956
chr19	40475283	40476855
chr19	40476980	40477728
chr19	40501395	40501541
chr19	40502469	40503303

chr19	40561652	40561680
chr19	40561845	40562329
chr19	40596121	40597104
chr19	40646061	40646109
chr19	40680030	40680133
chr19	40684561	40684583
chr19	40696123	40698608
chr19	40725676	40726043
chr19	40730052	40730256
chr19	40730452	40730708
chr19	40731354	40731386
chr19	40730955	40731204
chr19	40732125	40732334
chr19	40732964	40733217
chr19	40752138	40752311
chr19	40781749	40781948
chr19	40783097	40783204
chr19	40790024	40791254
chr19	40791564	40791823
chr19	40792848	40793025
chr19	40798212	40798309
chr19	40801899	40802141
chr19	40808083	40808455
chr19	40808972	40809241
chr19	40810742	40811070
chr19	40811186	40811193
chr19	40814390	40814497
chr19	40836366	40836753
chr19	40837218	40837359
chr19	40853677	40854409
chr19	40854760	40855809
chr19	40871754	40872167
chr19	40882176	40882421
chr19	40899150	40899499
chr19	40906398	40906436
chr19	40907743	40907942
chr19	40908546	40909002
chr19	40909075	40909227
chr19	40909777	40910153
chr19	40910417	40910810
chr19	40916567	40917207
chr19	40917354	40917926
chr19	40918038	40918129
chr19	40922789	40922967
chr19	40927920	40928211
chr19	40928351	40928615

chr19	40926465	40927625
chr19	40929033	40929321
chr19	40930463	40931732
chr19	40932068	40932625
chr19	40936155	40936334
chr19	40939106	40941544
chr19	40942480	40942481
chr19	40943264	40943478
chr19	40944732	40944750
chr19	40945091	40945251
chr19	40948571	40948635
chr19	40948708	40949093
chr19	40949356	40951152
chr19	40951519	40952523
chr19	40952598	40952864
chr19	40953047	40953289
chr19	40957651	40958574
chr19	40960434	40960743
chr19	40959736	40960263
chr19	40966233	40966433
chr19	40967393	40967811
chr19	40969061	40971810
chr19	40972246	40972513
chr19	40975170	40975470
chr19	41018601	41019211
chr19	41025469	41025704
chr19	41034754	41035064
chr19	41035417	41035616
chr19	41042256	41042392
chr19	41047655	41048030
chr19	41073573	41073772
chr19	41075591	41076684
chr19	41082022	41083723
chr19	41089820	41090033
chr19	41091341	41092381
chr19	41092661	41092985
chr19	41093837	41094074
chr19	41099269	41099469
chr19	41101104	41101304
chr19	41101722	41101786
chr19	41101989	41102022
chr19	41103148	41103532
chr19	41104147	41104587
chr19	41105509	41105708
chr19	41106436	41107038
chr19	41107329	41107664

chr19	41107875	41108778
chr19	41108884	41109861
chr19	41112651	41113255
chr19	41110283	41112581
chr19	41113373	41113677
chr19	41114655	41114759
chr19	41115443	41118021
chr19	41118880	41120522
chr19	41121047	41121463
chr19	41121907	41122106
chr19	41122236	41122611
chr19	41137268	41137467
chr19	41140057	41140462
chr19	41151988	41152171
chr19	41155327	41155542
chr19	41168468	41169279
chr19	41169401	41169871
chr19	41170035	41170268
chr19	41170422	41170671
chr19	41170782	41170938
chr19	41171278	41171667
chr19	41172037	41172391
chr19	41172738	41172962
chr19	41185294	41185382
chr19	41192165	41192326
chr19	41195197	41195625
chr19	41195881	41197556
chr19	41216825	41217395
chr19	41219352	41219770
chr19	41220240	41222694
chr19	41226921	41227227
chr19	41227468	41228614
chr19	41222836	41226772
chr19	41229396	41229648
chr19	41238584	41238600
chr19	41240898	41241820
chr19	41241901	41242570
chr19	41249125	41249339
chr19	41251262	41251851
chr19	41252235	41252244
chr19	41252356	41252557
chr19	41253699	41253757
chr19	41255634	41257930
chr19	41260041	41260275
chr19	41261370	41261603
chr19	41254803	41255570

chr19	41261851	41262143
chr19	41262534	41262572
chr19	41266589	41266753
chr19	41275656	41275873
chr19	41279206	41279291
chr19	41280136	41280480
chr19	41282163	41282362
chr19	41283070	41285059
chr19	41298602	41298645
chr19	41299542	41299643
chr19	41303890	41304772
chr19	41305092	41306490
chr19	41308093	41308438
chr19	41316145	41316424
chr19	41316713	41317945
chr19	41319568	41319884
chr19	41321735	41321934
chr19	41322923	41323243
chr19	41324262	41324563
chr19	41337670	41338308
chr19	41641160	41641443
chr19	41646282	41646409
chr19	41681520	41681719
chr19	41682498	41682701
chr19	41699076	41699731
chr19	41702334	41702583
chr19	41710493	41710692
chr19	41718633	41718660
chr19	41719432	41720082
chr19	41722481	41722954
chr19	41723337	41723563
chr19	41723682	41727565
chr19	41727878	41728604
chr19	41728713	41729524
chr19	41729636	41733128
chr19	41734292	41734348
chr19	41735098	41735948
chr19	41736017	41736657
chr19	41738984	41739065
chr19	41756696	41756737
chr19	41761850	41761940
chr19	41767781	41768295
chr19	41768492	41769759
chr19	41770181	41771441
chr19	41771521	41772140
chr19	41772717	41773060

chr19	41773498	41773707
chr19	41775571	41775583
chr19	41782150	41782457
chr19	41782560	41782624
chr19	41813358	41813557
chr19	41814251	41815075
chr19	41815230	41817399
chr19	41818700	41818729
chr19	41828676	41829970
chr19	41830298	41830580
chr19	41830742	41832207
chr19	41832477	41833931
chr19	41834087	41834419
chr19	41835149	41835399
chr19	41836167	41836506
chr19	41841024	41841220
chr19	41841979	41842186
chr19	41853656	41853950
chr19	41856702	41856958
chr19	41857065	41860058
chr19	41862321	41862520
chr19	41868633	41868700
chr19	41869518	41870112
chr19	41870272	41870540
chr19	41871466	41871711
chr19	41872477	41872632
chr19	41872764	41872963
chr19	41873567	41874713
chr19	41876623	41876984
chr19	41879668	41879976
chr19	41880313	41880791
chr19	41880953	41881765
chr19	41882074	41882986
chr19	41883124	41883395
chr19	41883563	41883914
chr19	41884683	41884870
chr19	41885651	41886624
chr19	41890980	41891182
chr19	41892231	41892461
chr19	41900198	41900354
chr19	41902970	41903249
chr19	41903401	41904299
chr19	41904877	41905076
chr19	41905843	41906055
chr19	41914811	41915458
chr19	41915744	41916060

chr19	41918162	41918317
chr19	41921953	41922152
chr19	41922498	41922758
chr19	41932910	41934564
chr19	41936569	41936726
chr19	41944827	41945084
chr19	41945293	41946235
chr19	41955089	41955414
chr19	42278825	42279068
chr19	42363446	42363597
chr19	42363806	42365615
chr19	42367174	42367614
chr19	42368192	42368624
chr19	42370193	42370294
chr19	42370558	42370802
chr19	42385869	42388238
chr19	42388455	42389117
chr19	42389905	42391552
chr19	42391696	42392553
chr19	42432426	42432835
chr19	42443984	42445263
chr19	42462503	42463538
chr19	42463929	42464071
chr19	42464205	42464297
chr19	42539096	42539599
chr19	42553077	42553145
chr19	42555656	42556297
chr19	42576315	42576621
chr19	42577685	42578257
chr19	42578973	42580920
chr19	42586372	42586926
chr19	42592552	42593065
chr19	42593200	42593626
chr19	42601547	42601778
chr19	42602257	42602373
chr19	42603872	42604543
chr19	42604669	42605372
chr19	42605744	42606019
chr19	42606501	42606641
chr19	42612436	42612773
chr19	42612894	42613204
chr19	42613475	42613562
chr19	42614179	42614393
chr19	42614567	42614940
chr19	42615277	42617268
chr19	42617590	42617827

chr19	42617998	42618759
chr19	42618907	42618969
chr19	42619080	42619421
chr19	42619567	42619950
chr19	42623786	42623866
chr19	42625200	42627055
chr19	42628210	42628329
chr19	42630478	42630633
chr19	42630725	42630756
chr19	42633131	42634069
chr19	42634210	42634843
chr19	42635535	42637350
chr19	42637618	42637817
chr19	42679884	42679960
chr19	42680756	42681045
chr19	42681411	42681610
chr19	42695557	42696516
chr19	42698453	42698665
chr19	42699705	42699871
chr19	42700817	42701051
chr19	42701184	42701579
chr19	42701871	42702452
chr19	42702559	42704577
chr19	42705271	42705530
chr19	42709967	42710251
chr19	42710442	42710538
chr19	42710610	42710861
chr19	42711081	42711808
chr19	42712273	42712752
chr19	42713014	42713395
chr19	42716437	42716520
chr19	42720552	42724907
chr19	42725089	42725434
chr19	42739178	42739377
chr19	42742986	42743213
chr19	42745589	42745634
chr19	42746111	42746889
chr19	42747521	42749004
chr19	42749499	42749902
chr19	42750018	42750402
chr19	42750686	42751014
chr19	42751165	42751446
chr19	42752039	42752378
chr19	42753030	42753277
chr19	42753947	42755518
chr19	42755641	42755861

chr19	42756242	42756463
chr19	42756642	42760269
chr19	42763343	42763462
chr19	42762231	42763159
chr19	42763759	42764119
chr19	42767125	42767371
chr19	42771808	42771828
chr19	42771979	42772321
chr19	42772462	42772840
chr19	42773371	42775089
chr19	42775378	42775724
chr19	42777548	42777793
chr19	42779190	42779567
chr19	42780156	42785185
chr19	42785310	42788504
chr19	42788733	42789160
chr19	42800050	42800312
chr19	42800793	42801018
chr19	42804827	42804961
chr19	42806076	42806845
chr19	42806980	42807798
chr19	42827937	42829351
chr19	42829672	42830543
chr19	42830682	42830828
chr19	42849328	42849441
chr19	42851503	42851834
chr19	42883097	42883296
chr19	42891675	42891834
chr19	42891919	42892168
chr19	42900230	42900275
chr19	42900432	42902003
chr19	42902214	42902517
chr19	42902673	42902706
chr19	42902824	42902957
chr19	42924945	42924989
chr19	42925245	42925394
chr19	42926946	42927690
chr19	42927803	42928547
chr19	42946938	42946968
chr19	42947314	42947641
chr19	42960567	42960714
chr19	43030281	43030480
chr19	43032392	43032514
chr19	43032662	43033484
chr19	43035024	43035192
chr19	43035913	43036014

chr19	43215938	43216207
chr19	43239850	43240443
chr19	43240613	43240950
chr19	43241154	43241161
chr19	43242072	43242285
chr19	43246152	43246452
chr19	43262818	43263381
chr19	43265341	43265407
chr19	43265487	43265963
chr19	43266051	43266876
chr19	43303973	43304341
chr19	43323560	43323670
chr19	43324114	43324860
chr19	43324975	43325322
chr19	43328312	43328481
chr19	43335374	43335567
chr19	43336350	43336406
chr19	43335753	43335874
chr19	43337021	43337546
chr19	43355029	43356674
chr19	43357352	43357643
chr19	43358319	43358637
chr19	43359743	43359984
chr19	43361705	43361889
chr19	43375436	43375712
chr19	43379048	43379924
chr19	43380768	43380967
chr19	43383543	43383752
chr19	43383944	43384106
chr19	43384180	43384279
chr19	43384429	43384631
chr19	43385091	43385839
chr19	43388971	43388985
chr19	43389259	43389462
chr19	43392125	43392775
chr19	43393193	43393462
chr19	43393536	43394277
chr19	43401877	43402296
chr19	43402833	43402948
chr19	43403105	43403258
chr19	43403469	43403920
chr19	43412081	43412276
chr19	43424501	43424728
chr19	43427221	43427653
chr19	43433868	43433898
chr19	43434287	43434763

chr19	43434109	43434124
chr19	43436546	43437123
chr19	43437370	43437404
chr19	43437582	43438255
chr19	43440755	43440954
chr19	43441033	43441124
chr19	43459629	43459879
chr19	43492431	43493101
chr19	43493361	43493720
chr19	43494087	43494400
chr19	43494526	43494592
chr19	43495641	43495857
chr19	43507798	43508389
chr19	43522833	43522894
chr19	43522970	43523032
chr19	43523968	43524089
chr19	43525788	43527298
chr19	43528265	43528571
chr19	43564263	43564376
chr19	43564636	43564725
chr19	43582188	43583280
chr19	43585801	43585929
chr19	43588193	43588392
chr19	43588641	43588899
chr19	43589944	43590259
chr19	43591720	43592010
chr19	43614733	43615105
chr19	43624606	43624808
chr19	43624959	43625067
chr19	43631398	43631815
chr19	43633259	43634045
chr19	43652339	43653014
chr19	43653908	43654432
chr19	43656559	43656613
chr19	43666177	43666357
chr19	43667417	43667487
chr19	43667765	43668222
chr19	43670766	43670970
chr19	43682810	43683019
chr19	43685504	43687631
chr19	43688357	43688799
chr19	43690556	43690795
chr19	43691998	43692283
chr19	43692469	43692861
chr19	43696084	43696196
chr19	43705046	43705504

chr19	43705789	43705967
chr19	43768387	43768845
chr19	43768961	43771625
chr19	43772604	43772616
chr19	43773540	43774165
chr19	43775051	43775467
chr19	43775573	43775713
chr19	43779118	43779249
chr19	43845577	43845713
chr19	43847236	43847301
chr19	43849923	43850044
chr19	43860543	43861067
chr19	43860379	43860474
chr19	43881702	43881901
chr19	43882063	43882677
chr19	43885816	43885819
chr19	43926619	43926764
chr19	43928766	43928873
chr19	43929823	43930222
chr19	43930669	43930868
chr19	43942717	43943011
chr19	43943284	43943376
chr19	43952331	43952518
chr19	43954022	43954479
chr19	43956596	43956724
chr19	43958774	43958857
chr19	43959505	43960174
chr19	43961611	43961863
chr19	43966477	43966683
chr19	43966765	43967529
chr19	43967668	43970417
chr19	43970606	43970734
chr19	43971512	43972276
chr19	43972603	43972957
chr19	43973622	43973775
chr19	43974351	43975000
chr19	43975439	43975638
chr19	43975862	43976011
chr19	43976604	43976616
chr19	43976896	43977095
chr19	43978901	43979401
chr19	43979703	43979846
chr19	43979928	43980213
chr19	43980478	43980840
chr19	43981163	43981437
chr19	44003320	44003560

chr19	44006834	44007203
chr19	44007394	44009200
chr19	44010074	44010079
chr19	44009506	44009997
chr19	44011961	44012160
chr19	44020357	44020556
chr19	44021808	44022090
chr19	44022326	44022614
chr19	44023472	44023586
chr19	44024568	44024851
chr19	44023727	44024125
chr19	44027060	44027404
chr19	44027706	44028256
chr19	44028959	44029665
chr19	44029778	44032391
chr19	44033003	44033209
chr19	44033631	44033887
chr19	44035075	44035094
chr19	44035259	44035656
chr19	44036137	44038153
chr19	44038457	44038678
chr19	44060757	44060953
chr19	44061821	44062012
chr19	44066620	44066985
chr19	44067280	44067843
chr19	44069104	44069283
chr19	44074248	44074416
chr19	44074588	44074849
chr19	44077165	44077182
chr19	44078452	44078615
chr19	44078858	44081469
chr19	44085002	44085372
chr19	44094558	44094567
chr19	44095679	44095903
chr19	44098310	44098968
chr19	44099125	44100251
chr19	44100577	44101006
chr19	44101154	44101682
chr19	44110390	44110603
chr19	44110740	44110753
chr19	44110921	44111150
chr19	44111451	44111521
chr19	44111623	44111731
chr19	44112655	44112887
chr19	44121984	44122273
chr19	44122608	44124421

chr19	44124879	44124905
chr19	44122402	44122527
chr19	44138537	44139287
chr19	44139813	44140012
chr19	44143502	44143829
chr19	44144068	44145157
chr19	44155276	44155475
chr19	44163225	44163305
chr19	44172171	44172476
chr19	44173147	44173809
chr19	44173886	44174353
chr19	44174476	44174725
chr19	44176446	44176681
chr19	44177418	44177489
chr19	44178261	44180042
chr19	44180346	44180455
chr19	44183800	44184096
chr19	44191365	44191659
chr19	44204417	44206359
chr19	44207211	44207538
chr19	44216163	44216496
chr19	44219630	44219679
chr19	44224729	44225155
chr19	44228027	44228237
chr19	44242836	44243061
chr19	44243576	44244086
chr19	44244231	44244747
chr19	44245292	44245497
chr19	44247730	44247882
chr19	44249407	44249478
chr19	44249704	44249862
chr19	44257127	44259752
chr19	44259869	44260016
chr19	44260522	44260860
chr19	44261361	44262580
chr19	44263050	44263871
chr19	44264112	44264158
chr19	44265985	44266293
chr19	44266681	44266868
chr19	44267511	44267692
chr19	44267935	44268021
chr19	44268225	44268324
chr19	44268435	44268462
chr19	44268684	44269428
chr19	44269738	44270078
chr19	44277227	44277365

chr19	44281064	44281223
chr19	44281349	44281560
chr19	44282163	44282405
chr19	44282955	44285732
chr19	44285875	44286170
chr19	44288420	44288512
chr19	44287395	44287594
chr19	44288706	44289831
chr19	44302130	44302812
chr19	44303022	44303692
chr19	44319102	44319105
chr19	44319405	44319578
chr19	44324641	44324867
chr19	44331204	44331218
chr19	44331411	44331971
chr19	44376769	44377235
chr19	44405569	44405933
chr19	44406054	44406294
chr19	44438625	44438858
chr19	44438985	44439007
chr19	44439123	44439920
chr19	44455010	44455214
chr19	44455357	44455531
chr19	44487938	44488222
chr19	44488344	44489166
chr19	44506747	44507018
chr19	44507247	44507790
chr19	44507877	44508340
chr19	44529256	44530022
chr19	44555199	44556005
chr19	44556245	44556578
chr19	44575897	44576123
chr19	44576352	44576674
chr19	44598187	44598401
chr19	44598671	44598827
chr19	44599249	44599670
chr19	44616992	44617314
chr19	44617609	44617892
chr19	44618006	44618296
chr19	44645365	44646360
chr19	44668790	44669088
chr19	44669239	44669548
chr19	44711698	44711958
chr19	44716293	44716953
chr19	44717104	44717145
chr19	44717363	44717427

chr19	44763775	44764014
chr19	44764171	44764808
chr19	44781535	44781837
chr19	44808544	44809150
chr19	44860492	44860769
chr19	44861089	44861193
chr19	44905381	44905724
chr19	44905864	44906058
chr19	44915528	44915755
chr19	44951986	44952085
chr19	44952906	44952969
chr19	45003782	45005084
chr19	45030769	45030915
chr19	45035260	45035266
chr19	45056610	45056919
chr19	45057029	45057509
chr19	45058803	45058855
chr19	45074386	45074743
chr19	45091561	45091856
chr19	45092059	45092416
chr19	45110729	45110735
chr19	45111707	45112040
chr19	45127610	45127720
chr19	45128815	45128871
chr19	45129074	45129373
chr19	45129619	45130584
chr19	45133183	45133190
chr19	45140633	45140758
chr19	45142021	45142102
chr19	45143616	45143844
chr19	45146727	45147040
chr19	45147214	45148284
chr19	45148799	45149138
chr19	45174694	45174790
chr19	45177623	45177904
chr19	45180097	45180261
chr19	45180408	45181238
chr19	45181317	45181585
chr19	45182527	45182787
chr19	45184936	45185151
chr19	45185237	45185548
chr19	45186236	45186488
chr19	45187690	45187933
chr19	45188168	45189109
chr19	45189174	45189234
chr19	45190504	45190785

chr19	45191936	45192233
chr19	45192383	45192831
chr19	45194811	45195906
chr19	45196337	45196580
chr19	45197506	45198151
chr19	45198329	45198734
chr19	45199315	45199362
chr19	45201596	45202013
chr19	45219956	45219987
chr19	45220246	45220306
chr19	45223672	45223879
chr19	45224729	45224975
chr19	45227417	45228458
chr19	45229428	45229661
chr19	45229808	45229847
chr19	45230040	45230171
chr19	45230301	45230348
chr19	45231313	45231438
chr19	45231571	45231859
chr19	45233063	45233263
chr19	45237322	45237529
chr19	45237945	45238301
chr19	45239954	45240295
chr19	45240561	45240607
chr19	45241094	45241310
chr19	45241972	45242171
chr19	45242303	45242811
chr19	45243940	45245184
chr19	45245341	45246357
chr19	45246605	45247417
chr19	45247839	45253275
chr19	45253832	45254286
chr19	45253502	45253768
chr19	45254676	45255127
chr19	45255487	45259822
chr19	45264472	45264760
chr19	45265968	45266328
chr19	45266584	45266911
chr19	45267124	45267334
chr19	45267915	45268001
chr19	45268136	45269234
chr19	45270233	45270521
chr19	45270624	45270895
chr19	45271047	45271246
chr19	45271998	45272352
chr19	45273637	45273879

chr19	45273985	45274458
chr19	45276745	45278026
chr19	45278426	45280664
chr19	45280758	45281705
chr19	45282012	45282353
chr19	45282527	45283076
chr19	45283467	45283910
chr19	45295218	45295431
chr19	45301633	45301961
chr19	45305251	45305546
chr19	45309576	45310407
chr19	45310632	45311343
chr19	45311520	45314269
chr19	45314946	45315199
chr19	45336036	45336294
chr19	45344784	45344804
chr19	45345039	45345097
chr19	45346565	45346784
chr19	45347565	45349197
chr19	45349489	45351319
chr19	45352075	45352311
chr19	45353934	45354133
chr19	45355516	45355715
chr19	45366442	45366609
chr19	45371562	45371654
chr19	45378552	45378751
chr19	45386666	45386882
chr19	45388454	45389058
chr19	45393295	45393669
chr19	45393795	45394075
chr19	45394414	45395596
chr19	45396063	45396074
chr19	45396406	45396605
chr19	45407560	45407780
chr19	45408371	45409741
chr19	45410113	45410415
chr19	45413308	45413527
chr19	45413965	45414622
chr19	45414775	45415115
chr19	45415374	45415390
chr19	45416564	45417056
chr19	45417433	45419181
chr19	45427945	45427946
chr19	45428312	45428565
chr19	45429089	45429369
chr19	45430114	45430519

chr19	45430748	45430775
chr19	45430843	45430993
chr19	45433504	45433703
chr19	45436733	45437271
chr19	45437466	45437487
chr19	45437675	45437730
chr19	45442534	45442675
chr19	45450909	45451010
chr19	45453692	45453776
chr19	45454727	45455295
chr19	45456759	45456938
chr19	45457461	45459241
chr19	45460076	45460118
chr19	45461041	45461257
chr19	45465815	45466030
chr19	45475221	45475265
chr19	45503422	45503510
chr19	45503826	45504091
chr19	45504324	45504508
chr19	45504725	45505181
chr19	45505544	45505910
chr19	45506058	45506069
chr19	45507414	45507773
chr19	45508979	45508980
chr19	45509597	45509767
chr19	45509946	45510054
chr19	45513207	45513479
chr19	45516579	45516765
chr19	45522321	45522495
chr19	45533626	45533878
chr19	45541607	45541758
chr19	45542016	45542212
chr19	45542335	45542940
chr19	45552260	45552362
chr19	45554819	45555018
chr19	45557693	45557726
chr19	45564723	45564922
chr19	45566866	45567065
chr19	45578138	45578694
chr19	45578844	45580292
chr19	45582063	45583903
chr19	45585631	45585677
chr19	45585820	45585943
chr19	45590303	45590502
chr19	45594046	45594785
chr19	45595267	45596975

chr19	45598529	45598825
chr19	45600988	45601821
chr19	45602111	45602605
chr19	45610154	45610244
chr19	45615235	45615488
chr19	45622379	45622535
chr19	45622630	45622654
chr19	45641261	45641460
chr19	45655198	45658451
chr19	45664114	45665212
chr19	45665826	45666047
chr19	45666276	45666622
chr19	45667600	45668062
chr19	45668214	45669640
chr19	45671452	45671476
chr19	45670646	45670752
chr19	45673001	45673203
chr19	45674231	45674960
chr19	45675640	45675877
chr19	45676564	45676763
chr19	45680194	45680611
chr19	45680897	45681586
chr19	45677922	45679816
chr19	45682047	45683774
chr19	45683852	45684322
chr19	45685980	45686187
chr19	45694069	45695256
chr19	45702948	45703422
chr19	45712292	45712549
chr19	45718518	45718687
chr19	45718939	45719182
chr19	45733901	45734159
chr19	45734647	45735279
chr19	45735489	45735869
chr19	45738461	45738661
chr19	45751859	45752051
chr19	45752288	45752426
chr19	45752502	45752960
chr19	45753845	45756202
chr19	45759470	45759768
chr19	45762899	45763043
chr19	45827196	45827395
chr19	45828645	45828665
chr19	45828399	45828441
chr19	45828909	45829428
chr19	45829626	45829864

chr19	45830034	45830494
chr19	45838465	45839299
chr19	45840551	45840574
chr19	45840637	45840812
chr19	45842123	45842509
chr19	45843547	45844625
chr19	45844847	45845188
chr19	45847607	45847919
chr19	45848278	45848677
chr19	45864077	45864358
chr19	45868561	45868852
chr19	45870761	45871254
chr19	45872538	45872714
chr19	45872966	45873862
chr19	45874031	45874393
chr19	45874668	45875010
chr19	45875114	45875139
chr19	45876181	45876562
chr19	45877252	45877596
chr19	45877787	45878360
chr19	45878822	45880950
chr19	45881036	45881576
chr19	45881784	45882078
chr19	45882329	45882712
chr19	45886508	45886749
chr19	45887382	45887426
chr19	45887522	45887867
chr19	45891564	45891763
chr19	45892191	45892340
chr19	45892780	45892884
chr19	45896117	45896415
chr19	45896743	45897081
chr19	45897716	45898071
chr19	45899239	45899499
chr19	45900830	45901115
chr19	45901821	45902020
chr19	45902824	45903292
chr19	45903713	45903992
chr19	45904327	45905336
chr19	45905413	45906440
chr19	45906527	45909070
chr19	45909241	45910634
chr19	45913829	45914046
chr19	45914939	45915189
chr19	45919599	45919995
chr19	45921096	45921197

chr19	45924367	45924588
chr19	45924750	45924808
chr19	45924948	45925903
chr19	45926324	45926782
chr19	45926900	45928489
chr19	45928578	45929139
chr19	45929573	45930688
chr19	45930865	45933414
chr19	45934235	45934853
chr19	45938860	45939442
chr19	45939762	45940375
chr19	45942713	45944208
chr19	45945766	45946664
chr19	45946893	45947874
chr19	45948130	45949089
chr19	45949470	45954673
chr19	45955987	45956299
chr19	45956407	45956694
chr19	45957614	45958085
chr19	45958400	45959929
chr19	45961696	45962165
chr19	45963572	45963771
chr19	45967262	45967500
chr19	45970370	45971207
chr19	45971423	45972530
chr19	45972715	45973927
chr19	45975100	45975507
chr19	45979999	45980309
chr19	45980433	45980708
chr19	45981150	45982439
chr19	45982565	45982665
chr19	45982925	45983396
chr19	45987293	45987682
chr19	45988158	45988594
chr19	45988753	45989164
chr19	45989524	45989772
chr19	45990016	45990060
chr19	45993354	45993553
chr19	45994044	45994579
chr19	45994847	45994874
chr19	45999988	46000332
chr19	46000484	46000827
chr19	46001009	46002730
chr19	46002972	46003180
chr19	46006792	46007013
chr19	46007838	46007898

chr19	46008010	46008368
chr19	46008846	46013174
chr19	46013948	46014150
chr19	46015736	46016000
chr19	46016664	46017277
chr19	46017498	46018293
chr19	46018933	46019161
chr19	46019959	46020217
chr19	46021864	46022184
chr19	46030819	46031368
chr19	46031724	46032034
chr19	46032183	46032593
chr19	46045517	46046177
chr19	46046666	46046876
chr19	46047789	46047942
chr19	46048976	46049035
chr19	46049816	46049942
chr19	46054784	46055045
chr19	46055455	46055569
chr19	46061370	46061720
chr19	46065895	46065992
chr19	46066373	46066623
chr19	46071574	46071768
chr19	46072358	46072805
chr19	46073585	46073728
chr19	46078295	46078306
chr19	46080292	46081186
chr19	46081708	46082046
chr19	46083340	46083521
chr19	46084255	46084261
chr19	46085556	46085755
chr19	46086182	46086262
chr19	46086967	46088738
chr19	46088860	46088966
chr19	46090445	46090650
chr19	46092700	46092715
chr19	46096075	46096245
chr19	46108641	46108763
chr19	46108893	46109003
chr19	46109466	46109828
chr19	46110244	46110364
chr19	46110753	46111049
chr19	46111235	46111292
chr19	46111411	46111763
chr19	46114826	46114996
chr19	46115634	46115903

chr19	46117126	46117583
chr19	46117798	46118782
chr19	46119717	46120445
chr19	46130429	46130649
chr19	46131493	46131845
chr19	46132663	46133477
chr19	46133579	46134425
chr19	46135200	46135524
chr19	46135632	46135890
chr19	46136697	46136794
chr19	46136880	46137139
chr19	46137720	46137763
chr19	46138053	46139110
chr19	46139283	46139552
chr19	46140814	46141401
chr19	46141468	46141971
chr19	46142038	46142827
chr19	46142957	46143391
chr19	46143526	46144016
chr19	46144130	46146472
chr19	46146582	46146994
chr19	46162922	46163162
chr19	46168495	46168755
chr19	46171458	46172124
chr19	46172909	46174012
chr19	46179621	46180794
chr19	46180966	46181165
chr19	46182996	46183615
chr19	46184157	46184366
chr19	46184478	46185283
chr19	46185892	46186102
chr19	46194323	46195518
chr19	46195969	46196976
chr19	46200348	46200542
chr19	46200931	46201430
chr19	46202664	46202821
chr19	46204004	46204121
chr19	46210476	46210756
chr19	46219548	46220554
chr19	46220678	46221780
chr19	46223207	46223495
chr19	46224504	46224928
chr19	46225161	46225697
chr19	46225876	46226732
chr19	46227698	46227886
chr19	46228101	46228507

chr19	46229992	46230303
chr19	46231281	46231542
chr19	46231776	46232319
chr19	46233281	46234206
chr19	46234429	46235189
chr19	46239239	46239472
chr19	46240006	46240993
chr19	46249876	46249989
chr19	46252636	46253515
chr19	46253743	46254036
chr19	46255230	46256026
chr19	46256229	46257160
chr19	46257343	46257575
chr19	46258737	46258945
chr19	46260810	46261574
chr19	46263658	46264134
chr19	46264461	46264657
chr19	46269198	46269456
chr19	46269804	46270752
chr19	46270858	46272675
chr19	46272907	46273414
chr19	46273611	46274473
chr19	46276958	46277360
chr19	46282013	46282095
chr19	46282263	46282291
chr19	46282647	46283328
chr19	46285456	46286420
chr19	46286651	46286963
chr19	46287090	46287435
chr19	46291270	46291278
chr19	46291044	46291131
chr19	46295113	46296121
chr19	46301137	46301439
chr19	46301912	46302052
chr19	46302117	46302823
chr19	46304089	46304351
chr19	46304751	46305181
chr19	46306256	46306584
chr19	46306792	46307181
chr19	46309433	46310016
chr19	46310615	46310954
chr19	46312811	46314328
chr19	46314743	46314966
chr19	46315390	46315763
chr19	46315905	46316294
chr19	46316631	46316937

chr19	46317234	46317484
chr19	46335919	46336200
chr19	46339896	46340139
chr19	46354585	46355127
chr19	46361764	46361963
chr19	46364045	46364115
chr19	46364720	46365163
chr19	46365635	46366533
chr19	46366674	46367258
chr19	46367476	46367830
chr19	46386270	46386574
chr19	46388513	46390584
chr19	46396574	46396754
chr19	46403299	46403312
chr19	46404199	46405863
chr19	46406265	46406784
chr19	46418989	46419151
chr19	46431164	46431468
chr19	46472332	46472347
chr19	46492004	46492241
chr19	46497334	46497705
chr19	46497939	46498343
chr19	46498651	46499516
chr19	46518260	46518712
chr19	46518940	46520000
chr19	46523368	46523771
chr19	46532193	46532240
chr19	46559801	46559953
chr19	46568755	46569186
chr19	46572095	46572331
chr19	46573108	46573128
chr19	46573267	46573735
chr19	46581773	46581867
chr19	46582442	46582832
chr19	46583967	46584233
chr19	46585576	46586105
chr19	46618379	46618447
chr19	46619058	46619263
chr19	46636308	46637017
chr19	46637883	46637922
chr19	46684836	46685067
chr19	46693401	46693600
chr19	46696197	46696796
chr19	46697854	46697940
chr19	46699419	46699787
chr19	46700871	46701225

chr19	46704451	46704573
chr19	46704685	46704917
chr19	46709301	46709529
chr19	46709819	46710503
chr19	46711604	46711871
chr19	46712017	46712473
chr19	46712776	46712877
chr19	46714177	46714360
chr19	46714636	46715115
chr19	46726353	46726392
chr19	46762390	46762892
chr19	46764759	46764958
chr19	46766069	46766252
chr19	46768737	46768974
chr19	46785899	46786100
chr19	46792085	46792389
chr19	46792790	46792957
chr19	46798386	46798539
chr19	46799803	46801346
chr19	46801660	46802289
chr19	46802493	46803212
chr19	46803448	46804893
chr19	46805543	46806365
chr19	46806890	46807116
chr19	46812997	46813196
chr19	46820421	46820583
chr19	46849443	46851007
chr19	46852177	46852657
chr19	46853044	46855777
chr19	46855948	46856197
chr19	46868456	46868508
chr19	46872962	46873188
chr19	46874084	46874612
chr19	46874898	46876605
chr19	46876948	46877681
chr19	46888599	46889143
chr19	46892437	46892844
chr19	46897874	46898023
chr19	46898237	46899120
chr19	46903682	46904056
chr19	46905479	46905702
chr19	46906815	46908393
chr19	46909957	46910471
chr19	46910880	46911339
chr19	46912319	46912558
chr19	46912870	46913152

chr19	46914877	46916864
chr19	46917095	46917634
chr19	46918782	46918932
chr19	46919103	46920137
chr19	46929816	46930065
chr19	46945975	46946299
chr19	46946527	46946772
chr19	46954512	46954981
chr19	46956904	46957313
chr19	46957655	46957874
chr19	46964244	46965280
chr19	46966301	46966500
chr19	46969716	46970427
chr19	46974307	46974826
chr19	46977138	46977893
chr19	46986121	46986240
chr19	46990833	46991166
chr19	46991400	46991747
chr19	46996030	46997243
chr19	47015207	47015432
chr19	47026887	47027104
chr19	47032720	47033323
chr19	47049751	47049846
chr19	47050054	47050253
chr19	47075041	47075157
chr19	47103361	47103560
chr19	47103676	47105998
chr19	47121039	47121058
chr19	47122004	47122203
chr19	47122461	47122690
chr19	47122866	47123100
chr19	47123368	47123492
chr19	47127341	47128923
chr19	47130150	47130771
chr19	47133586	47133984
chr19	47134320	47134726
chr19	47135345	47135585
chr19	47136767	47137318
chr19	47137886	47138113
chr19	47139156	47139273
chr19	47141966	47142234
chr19	47142648	47143073
chr19	47143280	47143479
chr19	47147064	47147165
chr19	47153097	47153308
chr19	47163089	47163613

chr19	47164064	47164488
chr19	47172075	47172349
chr19	47174347	47174520
chr19	47196338	47196627
chr19	47210991	47211248
chr19	47213194	47213393
chr19	47215162	47215229
chr19	47215422	47215631
chr19	47216929	47217574
chr19	47217731	47218339
chr19	47218696	47221423
chr19	47221613	47221829
chr19	47245868	47246245
chr19	47246807	47246991
chr19	47247591	47247737
chr19	47248273	47250255
chr19	47253025	47253314
chr19	47257303	47257450
chr19	47257528	47257900
chr19	47260303	47260453
chr19	47260843	47261225
chr19	47262035	47262084
chr19	47262754	47263716
chr19	47263904	47264266
chr19	47265383	47266382
chr19	47267982	47268093
chr19	47268337	47269032
chr19	47272801	47273153
chr19	47274434	47274635
chr19	47275928	47276269
chr19	47277071	47277295
chr19	47277433	47277728
chr19	47279371	47279648
chr19	47282354	47282866
chr19	47283329	47283528
chr19	47285384	47291821
chr19	47283803	47285003
chr19	47292156	47292642
chr19	47292884	47293215
chr19	47293976	47294210
chr19	47294794	47294958
chr19	47299114	47299325
chr19	47299932	47300281
chr19	47304418	47304819
chr19	47306584	47306796
chr19	47323958	47324062

chr19	47324508	47324536
chr19	47324690	47324862
chr19	47328467	47329612
chr19	47332389	47332861
chr19	47333017	47333570
chr19	47335669	47335961
chr19	47337654	47337696
chr19	47340689	47341000
chr19	47341724	47342104
chr19	47342669	47342923
chr19	47342992	47344908
chr19	47345055	47345565
chr19	47346756	47346956
chr19	47347236	47347563
chr19	47347677	47347886
chr19	47349688	47350175
chr19	47350834	47350919
chr19	47351478	47351580
chr19	47351908	47352158
chr19	47352357	47354108
chr19	47359027	47359372
chr19	47359625	47360474
chr19	47363073	47363325
chr19	47363628	47364100
chr19	47364236	47365672
chr19	47365768	47366103
chr19	47367081	47367295
chr19	47369763	47370173
chr19	47374902	47375101
chr19	47381321	47381526
chr19	47401912	47402221
chr19	47403537	47403782
chr19	47404988	47406086
chr19	47412705	47413043
chr19	47415612	47415903
chr19	47428102	47429225
chr19	47430258	47430820
chr19	47435582	47435600
chr19	47438268	47438499
chr19	47440127	47440351
chr19	47447388	47447613
chr19	47485500	47485699
chr19	47487307	47487316
chr19	47487475	47487883
chr19	47488037	47488291
chr19	47488905	47489029

chr19	47490567	47490778
chr19	47494656	47494829
chr19	47515091	47516443
chr19	47516798	47517072
chr19	47517409	47517716
chr19	47518094	47518175
chr19	47523037	47523391
chr19	47524008	47524602
chr19	47538014	47538271
chr19	47538354	47538960
chr19	47539236	47540141
chr19	47546883	47547090
chr19	47546577	47546818
chr19	47550892	47551091
chr19	47551328	47551947
chr19	47552137	47552756
chr19	47552866	47552959
chr19	47559904	47560136
chr19	47560593	47560671
chr19	47565303	47565420
chr19	47565696	47565852
chr19	47566469	47566751
chr19	47581667	47581806
chr19	47582084	47582131
chr19	47582555	47582616
chr19	47582859	47583436
chr19	47583860	47584200
chr19	47584272	47584468
chr19	47595327	47595391
chr19	47598734	47598863
chr19	47599183	47599269
chr19	47599447	47599652
chr19	47599865	47600269
chr19	47601621	47601634
chr19	47604374	47604631
chr19	47606810	47606826
chr19	47609518	47609886
chr19	47613843	47614777
chr19	47614899	47617351
chr19	47617481	47618058
chr19	47618124	47618360
chr19	47618480	47618586
chr19	47620886	47621026
chr19	47622748	47622982
chr19	47624600	47626286
chr19	47627651	47627875

chr19	47629240	47629463
chr19	47628082	47628798
chr19	47632590	47632866
chr19	47633397	47633848
chr19	47634083	47634394
chr19	47634507	47635560
chr19	47635727	47636108
chr19	47640313	47640512
chr19	47642998	47643197
chr19	47644649	47644848
chr19	47655862	47656005
chr19	47662389	47662685
chr19	47679322	47679398
chr19	47679506	47679724
chr19	47679896	47680076
chr19	47682663	47682862
chr19	47690306	47690309
chr19	47704227	47704420
chr19	47711519	47711840
chr19	47712513	47712526
chr19	47713893	47714321
chr19	47717893	47718008
chr19	47721275	47721543
chr19	47728367	47728759
chr19	47729034	47736913
chr19	47738864	47739038
chr19	47742553	47743255
chr19	47744537	47745056
chr19	47745869	47746187
chr19	47746465	47748031
chr19	47748112	47748663
chr19	47749339	47749590
chr19	47749715	47750037
chr19	47750333	47750674
chr19	47753375	47753671
chr19	47754312	47754705
chr19	47755075	47755526
chr19	47756061	47757485
chr19	47757965	47758301
chr19	47758379	47761720
chr19	47761934	47762300
chr19	47762768	47763028
chr19	47776012	47778694
chr19	47780417	47780517
chr19	47782366	47782787
chr19	47783236	47783767

chr19	47784006	47784035
chr19	47784356	47784662
chr19	47784906	47785181
chr19	47785329	47785376
chr19	47787376	47787601
chr19	47787791	47788023
chr19	47789191	47789239
chr19	47789761	47789800
chr19	47790805	47790934
chr19	47791580	47791900
chr19	47794187	47794366
chr19	47792064	47793610
chr19	47794658	47795125
chr19	47795219	47795397
chr19	47799310	47799419
chr19	47799614	47799929
chr19	47800423	47800581
chr19	47808784	47809461
chr19	47809593	47809806
chr19	47810576	47810775
chr19	47811374	47811464
chr19	47811871	47812605
chr19	47813821	47814119
chr19	47814372	47814535
chr19	47816407	47816897
chr19	47818013	47818269
chr19	47818340	47818671
chr19	47818849	47819237
chr19	47820799	47821040
chr19	47821985	47822072
chr19	47833526	47833818
chr19	47834040	47834244
chr19	47835080	47835323
chr19	47835956	47836084
chr19	47837069	47837189
chr19	47838366	47838951
chr19	47839919	47840222
chr19	47840495	47840623
chr19	47842606	47842664
chr19	47851578	47851790
chr19	47852147	47852329
chr19	47852518	47852824
chr19	47855853	47855868
chr19	47858855	47859124
chr19	47859742	47860412
chr19	47861946	47862140

chr19	47863961	47864263
chr19	47864736	47864956
chr19	47866693	47868019
chr19	47868132	47868264
chr19	47869882	47870038
chr19	47870123	47870150
chr19	47870956	47871057
chr19	47871585	47871784
chr19	47873606	47874232
chr19	47885960	47886179
chr19	47889868	47890300
chr19	47890572	47891218
chr19	47892122	47892272
chr19	47892865	47893202
chr19	47893373	47893666
chr19	47894914	47895113
chr19	47895299	47895782
chr19	47905074	47905335
chr19	47905423	47905469
chr19	47921492	47922761
chr19	47928224	47928423
chr19	47930277	47930549
chr19	47930748	47931513
chr19	47931868	47932067
chr19	47933132	47933621
chr19	47933923	47934178
chr19	47940261	47940476
chr19	47948462	47948661
chr19	47949676	47950044
chr19	47954429	47954566
chr19	47960306	47960549
chr19	47986792	47987885
chr19	47987965	47988285
chr19	47988485	47988628
chr19	48011511	48011868
chr19	48011931	48011947
chr19	48016179	48016331
chr19	48016511	48016829
chr19	48017159	48018334
chr19	48018739	48019199
chr19	48019320	48019601
chr19	48025778	48025807
chr19	48026232	48026448
chr19	48037626	48037830
chr19	48060772	48060979
chr19	48074995	48075011

chr19	48076214	48076790
chr19	48081753	48082115
chr19	48082960	48083159
chr19	48083361	48083430
chr19	48083921	48084259
chr19	48084951	48085219
chr19	48086138	48086955
chr19	48088627	48088794
chr19	48094372	48094433
chr19	48098017	48098212
chr19	48101462	48103507
chr19	48104365	48104729
chr19	48103729	48104221
chr19	48106581	48107034
chr19	48108269	48108579
chr19	48108754	48109055
chr19	48111026	48112152
chr19	48112420	48113886
chr19	48114073	48114108
chr19	48115208	48115679
chr19	48119675	48119882
chr19	48124412	48124577
chr19	48127017	48127473
chr19	48127582	48127750
chr19	48128031	48128344
chr19	48131335	48131659
chr19	48133484	48133540
chr19	48134364	48134717
chr19	48136679	48136930
chr19	48137159	48137537
chr19	48139716	48139962
chr19	48140425	48140723
chr19	48147244	48147525
chr19	48149403	48149671
chr19	48151329	48152504
chr19	48153330	48153699
chr19	48155504	48155785
chr19	48165334	48168271
chr19	48169243	48170112
chr19	48170334	48171450
chr19	48171768	48172135
chr19	48168633	48169117
chr19	48173181	48173373
chr19	48173953	48174023
chr19	48200841	48201447
chr19	48209770	48209957

chr19	48210906	48210998
chr19	48211098	48211355
chr19	48215620	48217443
chr19	48217642	48218353
chr19	48219171	48219458
chr19	48221043	48221331
chr19	48221580	48223255
chr19	48223322	48223711
chr19	48224106	48224334
chr19	48220350	48220969
chr19	48224851	48225464
chr19	48225673	48227220
chr19	48227366	48227577
chr19	48227880	48228272
chr19	48228873	48229425
chr19	48229543	48230166
chr19	48230485	48230672
chr19	48230891	48233739
chr19	48234683	48234849
chr19	48235081	48235765
chr19	48235916	48235933
chr19	48236362	48236719
chr19	48237841	48237984
chr19	48238454	48238465
chr19	48243359	48243612
chr19	48245511	48246486
chr19	48246601	48247857
chr19	48248243	48249277
chr19	48250673	48251161
chr19	48278384	48278583
chr19	48279583	48279792
chr19	48279988	48280285
chr19	48280422	48283285
chr19	48292316	48292646
chr19	48292979	48293460
chr19	48293540	48293647
chr19	48296880	48296952
chr19	48313298	48313590
chr19	48352594	48352980
chr19	48353456	48353476
chr19	48353555	48353749
chr19	48361405	48361653
chr19	48398151	48398478
chr19	48407446	48407645
chr19	48408382	48408583
chr19	48423521	48423750

chr19	48450203	48450319
chr19	48455630	48455763
chr19	48459198	48459489
chr19	48613194	48613415
chr19	48613556	48613746
chr19	48614272	48614300
chr19	48614745	48614963
chr19	48624986	48625212
chr19	48626827	48627174
chr19	48629681	48629755
chr19	48638537	48638665
chr19	48656107	48656471
chr19	48672674	48674584
chr19	48706886	48707016
chr19	48707344	48708067
chr19	48747646	48747845
chr19	48758773	48759428
chr19	48770132	48770443
chr19	48772876	48773075
chr19	48773289	48773521
chr19	48775699	48777262
chr19	48774008	48775557
chr19	48788091	48788209
chr19	48791541	48792064
chr19	48792545	48792686
chr19	48793290	48793692
chr19	48794181	48794946
chr19	48821172	48821387
chr19	48822741	48822759
chr19	48822838	48822940
chr19	48823192	48823391
chr19	48823658	48824026
chr19	48824257	48825203
chr19	48825405	48826152
chr19	48826401	48826752
chr19	48827991	48829845
chr19	48829999	48830053
chr19	48830375	48830381
chr19	48830479	48830574
chr19	48835029	48835038
chr19	48835208	48835282
chr19	48835431	48835518
chr19	48835585	48835983
chr19	48836337	48837743
chr19	48837835	48838103
chr19	48843368	48843737

chr19	48849393	48849520
chr19	48865897	48865901
chr19	48866105	48866304
chr19	48866556	48868527
chr19	48869363	48869629
chr19	48869696	48869765
chr19	48877426	48877625
chr19	48889691	48889692
chr19	48892296	48892395
chr19	48892960	48894842
chr19	48897250	48897593
chr19	48897911	48898536
chr19	48899115	48899353
chr19	48900400	48902385
chr19	48902512	48903007
chr19	48904044	48904243
chr19	48905063	48905170
chr19	48906816	48906932
chr19	48907124	48907274
chr19	48917016	48917780
chr19	48917891	48919195
chr19	48927022	48927652
chr19	48928002	48928506
chr19	48929557	48929675
chr19	48931372	48931390
chr19	48943239	48943438
chr19	48943694	48944038
chr19	48946515	48947222
chr19	48947392	48947693
chr19	48947981	48948572
chr19	48948683	48950064
chr19	48950285	48951119
chr19	48971638	48972150
chr19	48972440	48973684
chr19	48974241	48974258
chr19	48974861	48975243
chr19	48979746	48979861
chr19	48983617	48983917
chr19	48984059	48985223
chr19	48985808	48986113
chr19	48987602	48987716
chr19	48989185	48989261
chr19	48989436	48991353
chr19	48991478	48992302
chr19	48992587	48992863
chr19	48993699	48993898

chr19	48995555	48995789
chr19	48995893	48996462
chr19	48996820	48997105
chr19	48997253	48997937
chr19	48999306	48999505
chr19	49003534	49004098
chr19	49004298	49006122
chr19	49007801	49008115
chr19	49009324	49009479
chr19	49014156	49014752
chr19	49016219	49017199
chr19	49023092	49023253
chr19	49023858	49024198
chr19	49025888	49025920
chr19	49042608	49042750
chr19	49043154	49043251
chr19	49043384	49043425
chr19	49046261	49047434
chr19	49049437	49049658
chr19	49054876	49055075
chr19	49056619	49056923
chr19	49057437	49057765
chr19	49058049	49058268
chr19	49058791	49059341
chr19	49059709	49060284
chr19	49061906	49062132
chr19	49066482	49067260
chr19	49068136	49068457
chr19	49068579	49068919
chr19	49069360	49069523
chr19	49071115	49071148
chr19	49071367	49071691
chr19	49071955	49072304
chr19	49075854	49076053
chr19	49077122	49077452
chr19	49083156	49083306
chr19	49083406	49083583
chr19	49087294	49087539
chr19	49089840	49090287
chr19	49090479	49090678
chr19	49091415	49091712
chr19	49092011	49092499
chr19	49092685	49093937
chr19	49094084	49094378
chr19	49104943	49105483
chr19	49108633	49108815

chr19	49112241	49112722
chr19	49117526	49119945
chr19	49120287	49121011
chr19	49121693	49122735
chr19	49121198	49121254
chr19	49122870	49123539
chr19	49125682	49126019
chr19	49126102	49126528
chr19	49126991	49127391
chr19	49127561	49127998
chr19	49135260	49135961
chr19	49137432	49141598
chr19	49136152	49137253
chr19	49141749	49141967
chr19	49146118	49146301
chr19	49146382	49146435
chr19	49146576	49146996
chr19	49148314	49150947
chr19	49151490	49151745
chr19	49152185	49153418
chr19	49154045	49154363
chr19	49160106	49160516
chr19	49160752	49161286
chr19	49162418	49162669
chr19	49163273	49164796
chr19	49178870	49179050
chr19	49188554	49188662
chr19	49188741	49188781
chr19	49197847	49198540
chr19	49199103	49200405
chr19	49200932	49201049
chr19	49201308	49201347
chr19	49201715	49202027
chr19	49209504	49209611
chr19	49209962	49209994
chr19	49216563	49217386
chr19	49217606	49219043
chr19	49219300	49220471
chr19	49224000	49224300
chr19	49227159	49227345
chr19	49228063	49229841
chr19	49231894	49233556
chr19	49233811	49233815
chr19	49238399	49239019
chr19	49239104	49239240
chr19	49241319	49241707

chr19	49241817	49243521
chr19	49246677	49246876
chr19	49251150	49251367
chr19	49254640	49254698
chr19	49255099	49256241
chr19	49256364	49256661
chr19	49257027	49257416
chr19	49258223	49259344
chr19	49261938	49262206
chr19	49262333	49262358
chr19	49263234	49263248
chr19	49263466	49263741
chr19	49268876	49269052
chr19	49269146	49269311
chr19	49273427	49274367
chr19	49277563	49277877
chr19	49278865	49279069
chr19	49283508	49283647
chr19	49286030	49286063
chr19	49286286	49286485
chr19	49287222	49287285
chr19	49287936	49288323
chr19	49290640	49291137
chr19	49291318	49291540
chr19	49291853	49292052
chr19	49294142	49294164
chr19	49297966	49298557
chr19	49299014	49299213
chr19	49302049	49302202
chr19	49302462	49303031
chr19	49305121	49305277
chr19	49306061	49306101
chr19	49307176	49307382
chr19	49307452	49307621
chr19	49313739	49315598
chr19	49315929	49316251
chr19	49319154	49319336
chr19	49320131	49320141
chr19	49320983	49321073
chr19	49321254	49321319
chr19	49321817	49321949
chr19	49322445	49322680
chr19	49322936	49323265
chr19	49323567	49324455
chr19	49334069	49334322
chr19	49334996	49335165

chr19	49335525	49335744
chr19	49337406	49337622
chr19	49338060	49339594
chr19	49342969	49343226
chr19	49346975	49347270
chr19	49348141	49348340
chr19	49352714	49352913
chr19	49353190	49353444
chr19	49353529	49353585
chr19	49370740	49372390
chr19	49372695	49372901
chr19	49374365	49374705
chr19	49374875	49375493
chr19	49375667	49379782
chr19	49380512	49381053
chr19	49381503	49381890
chr19	49382471	49383141
chr19	49382136	49382403
chr19	49384807	49385056
chr19	49386214	49386440
chr19	49402496	49402824
chr19	49402999	49404996
chr19	49405445	49405646
chr19	49405843	49406391
chr19	49420738	49421032
chr19	49427056	49427461
chr19	49428118	49429639
chr19	49431791	49432065
chr19	49429822	49431715
chr19	49432187	49432207
chr19	49432306	49432506
chr19	49435777	49436718
chr19	49436856	49437413
chr19	49454438	49454544
chr19	49456654	49456910
chr19	49457370	49457913
chr19	49458028	49458842
chr19	49465471	49468261
chr19	49468602	49470250
chr19	49470396	49470754
chr19	49475082	49475544
chr19	49480406	49480816
chr19	49480943	49481077
chr19	49479709	49480152
chr19	49481293	49481571
chr19	49481731	49482217

chr19	49482390	49482722
chr19	49482807	49483141
chr19	49491176	49491375
chr19	49492465	49492594
chr19	49493338	49493491
chr19	49493726	49493968
chr19	49494101	49494348
chr19	49494819	49497503
chr19	49497767	49498152
chr19	49504308	49504385
chr19	49521086	49523550
chr19	49524442	49524649
chr19	49525152	49525343
chr19	49542466	49542737
chr19	49545055	49545563
chr19	49557045	49557181
chr19	49559594	49561594
chr19	49566113	49569010
chr19	49570345	49570624
chr19	49575091	49576687
chr19	49576840	49577900
chr19	49587060	49587414
chr19	49588173	49588475
chr19	49588682	49589187
chr19	49591243	49591687
chr19	49591891	49592160
chr19	49592501	49592726
chr19	49596814	49597072
chr19	49599416	49599615
chr19	49600307	49600554
chr19	49600788	49600918
chr19	49602823	49603101
chr19	49603851	49604084
chr19	49608778	49609051
chr19	49613445	49613764
chr19	49616650	49616883
chr19	49617017	49618188
chr19	49621397	49623559
chr19	49630635	49631624
chr19	49631789	49632293
chr19	49645928	49646824
chr19	49652203	49654893
chr19	49660534	49660931
chr19	49661066	49662053
chr19	49662314	49662570
chr19	49703107	49703203

chr19	49705722	49705855
chr19	49707365	49707425
chr19	49707589	49707805
chr19	49711506	49711959
chr19	49715743	49715835
chr19	49716100	49716215
chr19	49716357	49716449
chr19	49719280	49719363
chr19	49719793	49719995
chr19	49723436	49723515
chr19	49726238	49726319
chr19	49728418	49728564
chr19	49728649	49728663
chr19	49729031	49729391
chr19	49729587	49729641
chr19	49730040	49730236
chr19	49775362	49775628
chr19	49775793	49775882
chr19	49776964	49777346
chr19	49833259	49833458
chr19	49838221	49838270
chr19	49841879	49842081
chr19	49842370	49842681
chr19	49844088	49844132
chr19	49842984	49843875
chr19	49860017	49860235
chr19	49864960	49865076
chr19	49865151	49866495
chr19	49867091	49867589
chr19	49895607	49895851
chr19	49899276	49899475
chr19	49911893	49911991
chr19	49945973	49947045
chr19	49947219	49947408
chr19	49947736	49947935
chr19	49948773	49949187
chr19	49953621	49954079
chr19	49954307	49955025
chr19	49955783	49957350
chr19	49957719	49957727
chr19	49955267	49955565
chr19	49960831	49961030
chr19	49973219	49973248
chr19	49972966	49973046
chr19	49975494	49976062
chr19	49976680	49977090

chr19	49977325	49978515
chr19	49981714	49981996
chr19	49982337	49982531
chr19	49982647	49983080
chr19	49986866	49987086
chr19	49990157	49991652
chr19	49991850	49992381
chr19	49992802	49993619
chr19	49995052	49995251
chr19	49995902	49996101
chr19	49996535	49997205
chr19	49998339	49998612
chr19	49998941	49999316
chr19	49999629	50000055
chr19	50000386	50001159
chr19	50001398	50001518
chr19	50004000	50004199
chr19	50004779	50004997
chr19	50005397	50005612
chr19	50006227	50006800
chr19	50009348	50009810
chr19	50010111	50010379
chr19	50013509	50013722
chr19	50014350	50015000
chr19	50015336	50017074
chr19	50020578	50020825
chr19	50030569	50030972
chr19	50031116	50031758
chr19	50036228	50036309
chr19	50037435	50037919
chr19	50040020	50040224
chr19	50049192	50049993
chr19	50050192	50050356
chr19	50056372	50056489
chr19	50056795	50056980
chr19	50057149	50058849
chr19	50064936	50065094
chr19	50065539	50065712
chr19	50068261	50068605
chr19	50068862	50069061
chr19	50073418	50073568
chr19	50073770	50073833
chr19	50074519	50074630
chr19	50074837	50075032
chr19	50075581	50075815
chr19	50077639	50078311

chr19	50079085	50079523
chr19	50081910	50082163
chr19	50083170	50083413
chr19	50083517	50083884
chr19	50083992	50084152
chr19	50084367	50085279
chr19	50087743	50087828
chr19	50093215	50097196
chr19	50097314	50097513
chr19	50101325	50101524
chr19	50102012	50102211
chr19	50108475	50108500
chr19	50114196	50114476
chr19	50115230	50115637
chr19	50115996	50116218
chr19	50116392	50117736
chr19	50117839	50118019
chr19	50122913	50123109
chr19	50123208	50123380
chr19	50135837	50136069
chr19	50136221	50136420
chr19	50136789	50137132
chr19	50140551	50142113
chr19	50142270	50143461
chr19	50143671	50145113
chr19	50145344	50146430
chr19	50146979	50147472
chr19	50147764	50147936
chr19	50164783	50165048
chr19	50169761	50170044
chr19	50166991	50169693
chr19	50175373	50175744
chr19	50177890	50178026
chr19	50178748	50179995
chr19	50180445	50182664
chr19	50182786	50182985
chr19	50183376	50183665
chr19	50184477	50184785
chr19	50184212	50184303
chr19	50186979	50187019
chr19	50192709	50194247
chr19	50194414	50194686
chr19	50194937	50195505
chr19	50211411	50211655
chr19	50220669	50220737
chr19	50221357	50221407

chr19	50227048	50227959
chr19	50242085	50242285
chr19	50262806	50262811
chr19	50264818	50265110
chr19	50265367	50265809
chr19	50267074	50267798
chr19	50267878	50269221
chr19	50269329	50269720
chr19	50270261	50271867
chr19	50272913	50273214
chr19	50274365	50274482
chr19	50274893	50274940
chr19	50275186	50275773
chr19	50276129	50276576
chr19	50278187	50278386
chr19	50278665	50278873
chr19	50289110	50289358
chr19	50312181	50312852
chr19	50313094	50313293
chr19	50314579	50314778
chr19	50316080	50317153
chr19	50317652	50317943
chr19	50318554	50318616
chr19	50319963	50322702
chr19	50351279	50351840
chr19	50353216	50355493
chr19	50356735	50356934
chr19	50361926	50362993
chr19	50368937	50372104
chr19	50372249	50372733
chr19	50372876	50373613
chr19	50378670	50380609
chr19	50380774	50381957
chr19	50389295	50389494
chr19	50392595	50392794
chr19	50393093	50393773
chr19	50400934	50401133
chr19	50401263	50401696
chr19	50428171	50428329
chr19	50431272	50431541
chr19	50431637	50433289
chr19	50457631	50457856
chr19	50507537	50508120
chr19	50528031	50528166
chr19	50528294	50528574
chr19	50528940	50529747

chr19	50531645	50531683
chr19	50595294	50595456
chr19	50596270	50596810
chr19	50597044	50597275
chr19	50601660	50601859
chr19	50602844	50603165
chr19	50605815	50605893
chr19	50608315	50608563
chr19	50614322	50614629
chr19	50616200	50616345
chr19	50620562	50620843
chr19	50626958	50627252
chr19	50632136	50632353
chr19	50635738	50635942
chr19	50637659	50638018
chr19	50638177	50638561
chr19	50638725	50638968
chr19	50639248	50640627
chr19	50641121	50641602
chr19	50650855	50651114
chr19	50708647	50709136
chr19	50719035	50719317
chr19	50738398	50738597
chr19	50741700	50741899
chr19	50763526	50763599
chr19	50804083	50804210
chr19	50836526	50837750
chr19	50841853	50841977
chr19	50853507	50853662
chr19	50872963	50873199
chr19	50872004	50872884
chr19	50879282	50880529
chr19	50886697	50887390
chr19	50887564	50888004
chr19	50888722	50889090
chr19	50890475	50890912
chr19	50891375	50891524
chr19	50892317	50892437
chr19	50932921	50933168
chr19	50934556	50934760
chr19	50935040	50935781
chr19	50979759	50980767
chr19	51012346	51012485
chr19	51013656	51013939
chr19	51014043	51014535
chr19	51014802	51015519

chr19	51161887	51162192
chr19	51227935	51228134
chr19	51228344	51229022
chr19	51250454	51250653
chr19	51287209	51287266
chr19	51303774	51303810
chr19	51304776	51304887
chr19	51304992	51305518
chr19	51306223	51306292
chr19	51306421	51306757
chr19	51307349	51308141
chr19	51308262	51308742
chr19	51322088	51322287
chr19	51324347	51324706
chr19	51338647	51339736
chr19	51341156	51341164
chr19	51399620	51399819
chr19	51433108	51433141
chr19	51434523	51434722
chr19	51435096	51435181
chr19	51436899	51437330
chr19	51482216	51482251
chr19	51504441	51504773
chr19	51514355	51514497
chr19	51522057	51522100
chr19	51532076	51532269
chr19	51540693	51540851
chr19	51610600	51610814
chr19	51610906	51611597
chr19	51612029	51612499
chr19	51841800	51844053
chr19	51852750	51853106
chr19	51853633	51853991
chr19	51854161	51854576
chr19	51854653	51854964
chr19	51855948	51856000
chr19	51860665	51860962
chr19	51861797	51862195
chr19	51862527	51862560
chr19	51862780	51863209
chr19	51868019	51868187
chr19	51868367	51870811
chr19	51870970	51871616
chr19	51872678	51872877
chr19	51896927	51897436
chr19	51897852	51898076

chr19	51898547	51898791
chr19	52074200	52074314
chr19	52074548	52074662
chr19	52097030	52097486
chr19	52097804	52098410
chr19	52190799	52194723
chr19	52194794	52195015
chr19	52203942	52203944
chr19	52205292	52205415
chr19	52205827	52207941
chr19	52208028	52208297
chr19	52210010	52210052
chr19	52227808	52228068
chr19	52228994	52229299
chr19	52229779	52230146
chr19	52230387	52230586
chr19	52280919	52281099
chr19	52310572	52310835
chr19	52313036	52313275
chr19	52313520	52313706
chr19	52390796	52391196
chr19	52407597	52408315
chr19	52429834	52429976
chr19	52430137	52430857
chr19	52489896	52490412
chr19	52510835	52511039
chr19	52511159	52511694
chr19	52531299	52532166
chr19	52551707	52552816
chr19	52590043	52590186
chr19	52598054	52598176
chr19	52598332	52599028
chr19	52599341	52599356
chr19	52641898	52642121
chr19	52642241	52642331
chr19	52642402	52643160
chr19	52645640	52645728
chr19	52673876	52673906
chr19	52674167	52675062
chr19	52691427	52691543
chr19	52692165	52692899
chr19	52693290	52694594
chr19	52703189	52703435
chr19	52772289	52772497
chr19	52772777	52773423
chr19	52799615	52800139

chr19	52800394	52801108
chr19	52872846	52873672
chr19	52900810	52900983
chr19	52901281	52901691
chr19	53030009	53030744
chr19	53030917	53031989
chr19	53032398	53032550
chr19	53033850	53033916
chr19	53036936	53037558
chr19	53038529	53038831
chr19	53039651	53039946
chr19	53040053	53040111
chr19	53069437	53069742
chr19	53069821	53070318
chr19	53072866	53074208
chr19	53103799	53104243
chr19	53104574	53105510
chr19	53140854	53141562
chr19	53141777	53142006
chr19	53160818	53160987
chr19	53168279	53168605
chr19	53168733	53169223
chr19	53165870	53166648
chr19	53173203	53173275
chr19	53173732	53173841
chr19	53176370	53176397
chr19	53176500	53176630
chr19	53187947	53188178
chr19	53192591	53193737
chr19	53194001	53194412
chr19	53200571	53200664
chr19	53224583	53224623
chr19	53225423	53225674
chr19	53225924	53226267
chr19	53237173	53237372
chr19	53237444	53238428
chr19	53238573	53238634
chr19	53240072	53240145
chr19	53240684	53240794
chr19	53289213	53289872
chr19	53290312	53291053
chr19	53321560	53321759
chr19	53324093	53324385
chr19	53325051	53325210
chr19	53359906	53360200
chr19	53360341	53360853

chr19	53399420	53399634
chr19	53399877	53400953
chr19	53425114	53425248
chr19	53425477	53426716
chr19	53427724	53427791
chr19	53427870	53428296
chr19	53442953	53443144
chr19	53445155	53445845
chr19	53446428	53446588
chr19	53447572	53447754
chr19	53465655	53466256
chr19	53466713	53466849
chr19	53493996	53494936
chr19	53495077	53495162
chr19	53495414	53495423
chr19	53520010	53520227
chr19	53605040	53605055
chr19	53605911	53606568
chr19	53606889	53607173
chr19	53661328	53661461
chr19	53661685	53662259
chr19	53696730	53696876
chr19	53700726	53700952
chr19	53836355	53836485
chr19	53837098	53837721
chr19	53868673	53868702
chr19	53869085	53869564
chr19	53869656	53870029
chr19	53898090	53898289
chr19	53898775	53899481
chr19	53934582	53934952
chr19	53935269	53935920
chr19	53970991	53971601
chr19	54023651	54024178
chr19	54024346	54024862
chr19	54040452	54042194
chr19	54044454	54044653
chr19	54048369	54049353
chr19	54052593	54052962
chr19	54053268	54053611
chr19	54056941	54057240
chr19	54057848	54058524
chr19	54092483	54093441
chr19	54095728	54095972
chr19	54096281	54096420
chr19	54096555	54097049

chr19	54097138	54097484
chr19	54097806	54098202
chr19	54099005	54099268
chr19	54099495	54099695
chr19	54100106	54101000
chr19	54104810	54104854
chr19	54125552	54126017
chr19	54127916	54128206
chr19	54128429	54128437
chr19	54129603	54129720
chr19	54133635	54133749
chr19	54140768	54140932
chr19	54250887	54251126
chr19	54295907	54295913
chr19	54344187	54344591
chr19	54346087	54346237
chr19	54345839	54345850
chr19	54355982	54356181
chr19	54362113	54362245
chr19	54368318	54368761
chr19	54369537	54370398
chr19	54370508	54370883
chr19	54371071	54372906
chr19	54373357	54373701
chr19	54382279	54382649
chr19	54382902	54383333
chr19	54385312	54385537
chr19	54459536	54459557
chr19	54463470	54464288
chr19	54465277	54465617
chr19	54482024	54482051
chr19	54482128	54482466
chr19	54482765	54483470
chr19	54483586	54484172
chr19	54493752	54493951
chr19	54494978	54495240
chr19	54523223	54523449
chr19	54523689	54523985
chr19	54526277	54526471
chr19	54539202	54539433
chr19	54598669	54600445
chr19	54604188	54604292
chr19	54605486	54605705
chr19	54606171	54607146
chr19	54608682	54608886
chr19	54617282	54617318

chr19	54617436	54617644
chr19	54618129	54619340
chr19	54619913	54620436
chr19	54636064	54636086
chr19	54636174	54636388
chr19	54637485	54637785
chr19	54638073	54638519
chr19	54638846	54638960
chr19	54639406	54640404
chr19	54641413	54642747
chr19	54643157	54643356
chr19	54661586	54661802
chr19	54662468	54663508
chr19	54663859	54664386
chr19	54664494	54664697
chr19	54665049	54665248
chr19	54675539	54676932
chr19	54681196	54681455
chr19	54691642	54692075
chr19	54692339	54693532
chr19	54693816	54694627
chr19	54694806	54696642
chr19	54700214	54700261
chr19	54700337	54700733
chr19	54701164	54701396
chr19	54704104	54705303
chr19	54707233	54707432
chr19	54707867	54708228
chr19	54708295	54708480
chr19	54714462	54714661
chr19	54941536	54941735
chr19	54943036	54943462
chr19	54950939	54951138
chr19	54957593	54957888
chr19	54958685	54958979
chr19	54959314	54961333
chr19	54962184	54962363
chr19	54973045	54975022
chr19	54975217	54975468
chr19	54975585	54977180
chr19	54979863	54980163
chr19	54980539	54980666
chr19	54980931	54981130
chr19	54981316	54981680
chr19	54981835	54983870
chr19	54984032	54984970

chr19	55561582	55561817
chr19	55567147	55567346
chr19	55572415	55572974
chr19	55573194	55574589
chr19	55574973	55575083
chr19	55575544	55575701
chr19	55580477	55581418
chr19	55581546	55582389
chr19	55582604	55583241
chr19	55586342	55586638
chr19	55587034	55587941
chr19	55588466	55588726
chr19	55591245	55591517
chr19	55591776	55593044
chr19	55598089	55598393
chr19	55615798	55616154
chr19	55625276	55625475
chr19	55627832	55628703
chr19	55629287	55629813
chr19	55629947	55630184
chr19	55652913	55653168
chr19	55657684	55659312
chr19	55670924	55671854
chr19	55677632	55677980
chr19	55678090	55679178
chr19	55680802	55680908
chr19	55683823	55685459
chr19	55689868	55689960
chr19	55690033	55690067
chr19	55690294	55690493
chr19	55696731	55696930
chr19	55698251	55698351
chr19	55699520	55701688
chr19	55704260	55704449
chr19	55710360	55710594
chr19	55711311	55711416
chr19	55714305	55714362
chr19	55715628	55715799
chr19	55718772	55719806
chr19	55719936	55720823
chr19	55721021	55721447
chr19	55721686	55722573
chr19	55723187	55723242
chr19	55727499	55727583
chr19	55727656	55727860
chr19	55728053	55728587

chr19	55769312	55770440
chr19	55772064	55772148
chr19	55770853	55771510
chr19	55789360	55789626
chr19	55790745	55792160
chr19	55792332	55792523
chr19	55794723	55795204
chr19	55795442	55795780
chr19	55813081	55813288
chr19	55813413	55813848
chr19	55827418	55827457
chr19	55827918	55828404
chr19	55834415	55834428
chr19	55834566	55834614
chr19	55834890	55835056
chr19	55839124	55839389
chr19	55840276	55840772
chr19	55841410	55841686
chr19	55843157	55843665
chr19	55844409	55844576
chr19	55845752	55845925
chr19	55846867	55848183
chr19	55848492	55848733
chr19	55848866	55848879
chr19	55849096	55850936
chr19	55851222	55852629
chr19	55855659	55856233
chr19	55864613	55866188
chr19	55866704	55867288
chr19	55867747	55867889
chr19	55878006	55878205
chr19	55880555	55880874
chr19	55881165	55881388
chr19	55882073	55882338
chr19	55885007	55885262
chr19	55885431	55885699
chr19	55887110	55887603
chr19	55887991	55888629
chr19	55894235	55895093
chr19	55895328	55897083
chr19	55897244	55899192
chr19	55903624	55903826
chr19	55905111	55905779
chr19	55906997	55907216
chr19	55914754	55914877
chr19	55915879	55916357

chr19	55917389	55919497
chr19	55920221	55920420
chr19	55920571	55920906
chr19	55919658	55919996
chr19	55952940	55953139
chr19	55956984	55957120
chr19	55957203	55958653
chr19	55959267	55959736
chr19	55959912	55960635
chr19	55962802	55963020
chr19	55963265	55963524
chr19	55972484	55972979
chr19	55973251	55974004
chr19	55974077	55974257
chr19	55984124	55984323
chr19	55985963	55986381
chr19	55986817	55989236
chr19	55996166	55996867
chr19	55998091	55998317
chr19	55998578	55999180
chr19	55999901	56000111
chr19	56003421	56003709
chr19	56005521	56006151
chr19	56014593	56014742
chr19	56014855	56015092
chr19	56028098	56028869
chr19	56058177	56058708
chr19	56059035	56059592
chr19	56060556	56061522
chr19	56063825	56064204
chr19	56064402	56064601
chr19	56090877	56092232
chr19	56092348	56092677
chr19	56096913	56097165
chr19	56097407	56098747
chr19	56099543	56099672
chr19	56099082	56099091
chr19	56102314	56102658
chr19	56110167	56113343
chr19	56115848	56117567
chr19	56117747	56118127
chr19	56118580	56118779
chr19	56119117	56119346
chr19	56130214	56130446
chr19	56135322	56136752
chr19	56143217	56143428

chr19	56143686	56143803
chr19	56144630	56144752
chr19	56145774	56147170
chr19	56148826	56148985
chr19	56150327	56150380
chr19	56150782	56150884
chr19	56151124	56153833
chr19	56154010	56156154
chr19	56157216	56157441
chr19	56157733	56158258
chr19	56158514	56160363
chr19	56164759	56167270
chr19	56167648	56167908
chr19	56168187	56168705
chr19	56164261	56164482
chr19	56169463	56169601
chr19	56170416	56170677
chr19	56183229	56183416
chr19	56183954	56183985
chr19	56185817	56186333
chr19	56186545	56187539
chr19	56595744	56595943
chr19	56601934	56602380
chr19	56603677	56603917
chr19	56625342	56625431
chr19	56625179	56625225
chr19	56628211	56628417
chr19	56630291	56630668
chr19	56630822	56632790
chr19	56633104	56633252
chr19	56651394	56651574
chr19	56651794	56653147
chr19	56683659	56683675
chr19	56683826	56683943
chr19	56728444	56729381
chr19	56805903	56806558
chr19	56806631	56807735
chr19	56813481	56813625
chr19	56825334	56826620
chr19	56826728	56827073
chr19	56879268	56880071
chr19	56904473	56904512
chr19	56904645	56905304
chr19	56915036	56915948
chr19	56987821	56988029
chr19	56988182	56988853

chr19	56988980	56989534
chr19	56989798	56990274
chr19	57019055	57019887
chr19	57049735	57050063
chr19	57050303	57051083
chr19	57078226	57079490
chr19	57106343	57107198
chr19	57256324	57256373
chr19	57683767	57684174
chr19	57702293	57703829
chr19	57751504	57752544
chr19	57752951	57753216
chr19	57790084	57790437
chr19	57791003	57792295
chr19	57793563	57793565
chr19	57793948	57794050
chr19	57794176	57794433
chr19	57794618	57794997
chr19	57813630	57814722
chr19	57816284	57816824
chr19	57831541	57832662
chr19	57845549	57846021
chr19	57862264	57862456
chr19	57862668	57862861
chr19	57863209	57863462
chr19	57873298	57873492
chr19	57874357	57874626
chr19	57874930	57875491
chr19	57900880	57901934
chr19	57922534	57922975
chr19	57946141	57946487
chr19	57946681	57947364
chr19	57988445	57988895
chr19	57988999	57989291
chr19	57999061	57999933
chr19	58011310	58012116
chr19	58038618	58039293
chr19	58070954	58071145
chr19	58089887	58090361
chr19	58090466	58090730
chr19	58095012	58095268
chr19	58095749	58095914
chr19	58111271	58111869
chr19	58125593	58126059
chr19	58144517	58144977
chr19	58162567	58163280

chr19	58175503	58175954
chr19	58186422	58186621
chr19	58192942	58193079
chr19	58193321	58193741
chr19	58238229	58238464
chr19	58238555	58238925
chr19	58239030	58239276
chr19	58258157	58258899
chr19	58259003	58259165
chr19	58280711	58280875
chr19	58281128	58281816
chr19	58286439	58286596
chr19	58286849	58286956
chr19	58304326	58304757
chr19	58310133	58311051
chr19	58313042	58313114
chr19	58313393	58313444
chr19	58314793	58315694
chr19	58315919	58316600
chr19	58323179	58323395
chr19	58325232	58325482
chr19	58325703	58326261
chr19	58326503	58326561
chr19	58329599	58329963
chr19	58330099	58331554
chr19	58332151	58332496
chr19	58334533	58334732
chr19	58341095	58342125
chr19	58342810	58343015
chr19	58360654	58360987
chr19	58361231	58361671
chr19	58366414	58366432
chr19	58377818	58377944
chr19	58395144	58395597
chr19	58396596	58396814
chr19	58399549	58400975
chr19	58405956	58406088
chr19	58427454	58428375
chr19	58445598	58445792
chr19	58446291	58446740
chr19	58458392	58459577
chr19	58513599	58515261
chr19	58519982	58521834
chr19	58570290	58570461
chr19	58570676	58571253
chr19	58609168	58609659

chr19	58661508	58662451
chr19	58665364	58667209
chr19	58667300	58667315
chr19	58693295	58693366
chr19	58693625	58695600
chr19	58695759	58696074
chr19	58696531	58696759
chr19	58739236	58741038
chr19	58751878	58752036
chr19	58789543	58789980
chr19	58790238	58791328
chr19	58791741	58791940
chr19	58794237	58794363
chr19	58816091	58816469
chr19	58816622	58817130
chr19	58817280	58817672
chr19	58837394	58837568
chr19	58837779	58839466
chr19	58841806	58842019
chr19	58854633	58854879
chr19	58857131	58857258
chr19	58858442	58859795
chr19	58860452	58860593
chr19	58868058	58868846
chr19	58872080	58872450
chr19	58872579	58874156
chr19	58874347	58874795
chr19	58875047	58875322
chr19	58882111	58882201
chr19	58891080	58891420
chr19	58891631	58892655
chr19	58892826	58893043
chr19	58893293	58893352
chr19	58893637	58894039
chr19	58894192	58894196
chr19	58895827	58896084
chr19	58896813	58897012
chr19	58897306	58898502
chr19	58898705	58899626
chr19	58900007	58900257
chr19	58902652	58903690
chr19	58904149	58904394
chr19	58905394	58905686
chr19	58906427	58908373
chr19	58910386	58910598
chr19	58912225	58912609

chr19	58912903	58913805
chr19	58914182	58914286
chr19	58918838	58921152
chr19	58950881	58951598
chr19	58951814	58952240
chr19	58963018	58963935
chr19	58969592	58969779
chr19	58975565	58975810
chr19	58977643	58978140
chr19	58978448	58978647
chr19	58979013	58979229
chr19	58984643	58984924
chr19	58986847	58987725
chr19	58987845	58988401
chr19	58988809	58989008
chr19	58989731	58989942
chr19	58995661	58995827
chr19	58999192	58999327
chr19	58999440	58999736
chr19	58999844	58999883
chr19	59004520	59004719
chr19	59006449	59006563
chr19	59009537	59009650
chr19	59009747	59010595
chr19	59011031	59011655
chr19	59016109	59016235
chr19	59024989	59026851
chr19	59029728	59033040
chr19	59033672	59034023
chr19	59033240	59033606
chr19	59041700	59042036
chr19	59049144	59050804
chr19	59051544	59051866
chr19	59053985	59054196
chr19	59054409	59054434
chr19	59054829	59057834
chr19	59058158	59058421
chr19	59064426	59064472
chr19	59065363	59067089
chr19	59068274	59071774
chr19	59073586	59074396
chr19	59076040	59076264
chr19	59077765	59077857
chr19	59083752	59084087
chr19	59086482	59087035
chr19	59084257	59086169

chr19	59087152	59087839
chr19	59088861	59089202
chr19	59092266	59092598
chr19	59101335	59101582
chr19	59108400	59108493
chr2	37951	38268
chr2	45339	45584
chr2	45654	47195
chr2	263362	265368
chr2	287571	287832
chr2	425002	425244
chr2	675924	675940
chr2	677141	677404
chr2	677890	677973
chr2	740271	740864
chr2	1628812	1628992
chr2	1629514	1629720
chr2	1632680	1632769
chr2	1658626	1658817
chr2	1679322	1679356
chr2	1708432	1708848
chr2	1711711	1712166
chr2	1712827	1713074
chr2	1724230	1724473
chr2	1724732	1726270
chr2	1733720	1734542
chr2	1747163	1748736
chr2	3381173	3381546
chr2	3381667	3382270
chr2	3383149	3383280
chr2	3383474	3384018
chr2	3384170	3384379
chr2	3469505	3469670
chr2	3469873	3470034
chr2	3470167	3470189
chr2	3522111	3522718
chr2	3522863	3523844
chr2	3582629	3584730
chr2	3605480	3605974
chr2	3606245	3606489
chr2	3622482	3622681
chr2	3622947	3623858
chr2	3624804	3625009
chr2	3652581	3652754
chr2	3652894	3653566
chr2	3653895	3654142

chr2	3668343	3668767
chr2	3698770	3699138
chr2	3759817	3760064
chr2	5305251	5305381
chr2	5765235	5765389
chr2	5768386	5768735
chr2	5774707	5774883
chr2	7005276	7006230
chr2	7017626	7017785
chr2	8738762	8738869
chr2	8819231	8819503
chr2	8825835	8826035
chr2	8833743	8833900
chr2	8944466	8944582
chr2	8962694	8962835
chr2	8977328	8977714
chr2	9142414	9142773
chr2	9143074	9144766
chr2	9144867	9145066
chr2	9145181	9145356
chr2	9198185	9198349
chr2	9211086	9211334
chr2	9211491	9211699
chr2	9211821	9212203
chr2	9247869	9248331
chr2	9252047	9252554
chr2	9252841	9253034
chr2	9282684	9282904
chr2	9283251	9283450
chr2	9298901	9299037
chr2	9303156	9303355
chr2	9310910	9310986
chr2	9311196	9311314
chr2	9318422	9318621
chr2	9318758	9319107
chr2	9319529	9320635
chr2	9346218	9346219
chr2	9346473	9346751
chr2	9347141	9348211
chr2	9374321	9374527
chr2	9374715	9376062
chr2	9376139	9376455
chr2	9386518	9386657
chr2	9386908	9387217
chr2	9389345	9390217
chr2	9390500	9391110

chr2	9391351	9392449
chr2	9394060	9394325
chr2	9426093	9426431
chr2	9426772	9427972
chr2	9428159	9428664
chr2	9440124	9440392
chr2	9440604	9440803
chr2	9450213	9450278
chr2	9450489	9451440
chr2	9451542	9451739
chr2	9451912	9452157
chr2	9455109	9455820
chr2	9468042	9468077
chr2	9473602	9473855
chr2	9476897	9477221
chr2	9520901	9521136
chr2	9521707	9521957
chr2	9526398	9527697
chr2	9527975	9528238
chr2	9528565	9528626
chr2	9556644	9556683
chr2	9557009	9557152
chr2	9562627	9564478
chr2	9614265	9615565
chr2	9622853	9623052
chr2	9623414	9623969
chr2	9629435	9629544
chr2	9689878	9690077
chr2	9690268	9690305
chr2	9690513	9690675
chr2	9692585	9693009
chr2	9695005	9695915
chr2	9696152	9696730
chr2	9697983	9698314
chr2	9698974	9699219
chr2	9719160	9719369
chr2	9747987	9748397
chr2	9769386	9771315
chr2	9771544	9771688
chr2	9784587	9785050
chr2	9796831	9796893
chr2	9799372	9799561
chr2	9815875	9816072
chr2	9893066	9894661
chr2	9910757	9911256
chr2	9921208	9921502

chr2	9921699	9922913
chr2	9940046	9940320
chr2	9940637	9940711
chr2	9940787	9944035
chr2	9944129	9944751
chr2	9948464	9948900
chr2	9949055	9949521
chr2	9952907	9953349
chr2	9953746	9954336
chr2	9954557	9954708
chr2	9957645	9957764
chr2	9959941	9960598
chr2	9970344	9970524
chr2	9970914	9970967
chr2	9977015	9977459
chr2	9983065	9984961
chr2	9986624	9987023
chr2	9987336	9987740
chr2	10035389	10035613
chr2	10035839	10036218
chr2	10037369	10038032
chr2	10045562	10045718
chr2	10045848	10045877
chr2	10054704	10055185
chr2	10090674	10091533
chr2	10091651	10091953
chr2	10092106	10092541
chr2	10116055	10116148
chr2	10124938	10125137
chr2	10152702	10152901
chr2	10153009	10153531
chr2	10155613	10155809
chr2	10159192	10159297
chr2	10158046	10158496
chr2	10159587	10160049
chr2	10160573	10160727
chr2	10170437	10170734
chr2	10176249	10176461
chr2	10176683	10176918
chr2	10180332	10180559
chr2	10182638	10183218
chr2	10183735	10184078
chr2	10184364	10184666
chr2	10190339	10190452
chr2	10196734	10196970
chr2	10197235	10197924

chr2	10219847	10220074
chr2	10220271	10220863
chr2	10236059	10236123
chr2	10237329	10237355
chr2	10237449	10237528
chr2	10252825	10253062
chr2	10256183	10256322
chr2	10258411	10258655
chr2	10260205	10261192
chr2	10261381	10263243
chr2	10263833	10264082
chr2	10264185	10264494
chr2	10264676	10265135
chr2	10278083	10278221
chr2	10410770	10411014
chr2	10411334	10411373
chr2	10423005	10423441
chr2	10425251	10425450
chr2	10438217	10438554
chr2	10442245	10443544
chr2	10443711	10444414
chr2	10479822	10479837
chr2	10481302	10481341
chr2	10523407	10523785
chr2	10529952	10530557
chr2	10533959	10534313
chr2	10542057	10542550
chr2	10545289	10545582
chr2	10547442	10547613
chr2	10547841	10548505
chr2	10548751	10549049
chr2	10549153	10549317
chr2	10572562	10572877
chr2	10574948	10575149
chr2	10587637	10588699
chr2	10588901	10590161
chr2	10590343	10590657
chr2	10590963	10591221
chr2	10592777	10593136
chr2	10593684	10594394
chr2	10601567	10601626
chr2	10605091	10605290
chr2	10630283	10630329
chr2	10633189	10633488
chr2	10634100	10634729
chr2	10638153	10638675

chr2	10654749	10654996
chr2	10678290	10678511
chr2	10715699	10715994
chr2	10716371	10716483
chr2	10724920	10724943
chr2	10794145	10794247
chr2	10800418	10800596
chr2	10829861	10830830
chr2	10859967	10860135
chr2	10914307	10914790
chr2	10950397	10950596
chr2	10952069	10953061
chr2	10953164	10953581
chr2	11017062	11017199
chr2	11018112	11018295
chr2	11051767	11052190
chr2	11093747	11094137
chr2	11261743	11261942
chr2	11270389	11270652
chr2	11294785	11295037
chr2	11295712	11296131
chr2	11335593	11335913
chr2	11356329	11356786
chr2	11470609	11470876
chr2	11482836	11482928
chr2	11483038	11483423
chr2	11483603	11483961
chr2	11484126	11485795
chr2	11487688	11488020
chr2	11510671	11510820
chr2	11511154	11511246
chr2	11514466	11514829
chr2	11514973	11515245
chr2	11519730	11520027
chr2	11526345	11526548
chr2	11526732	11526761
chr2	11529915	11530007
chr2	11530337	11530904
chr2	11531521	11531772
chr2	11532221	11532301
chr2	11534471	11534855
chr2	11585476	11585638
chr2	11605555	11607044
chr2	11622845	11623334
chr2	11646074	11646097
chr2	11646382	11646546

chr2	11686730	11687081
chr2	11687214	11687360
chr2	11751620	11751767
chr2	11752089	11752554
chr2	11753023	11753253
chr2	11828398	11828682
chr2	11828789	11828904
chr2	11833054	11833148
chr2	11885029	11885308
chr2	11886107	11886583
chr2	11886720	11887309
chr2	11887473	11887626
chr2	11888013	11888215
chr2	11888343	11888747
chr2	11890133	11890366
chr2	11890623	11890803
chr2	11890995	11891194
chr2	11893578	11893799
chr2	11964902	11965109
chr2	11969521	11970788
chr2	12003662	12003911
chr2	12004229	12004397
chr2	12008468	12008483
chr2	12054513	12054586
chr2	12078168	12078394
chr2	12078615	12078778
chr2	12091683	12092016
chr2	12187147	12187198
chr2	12245954	12246883
chr2	12295831	12295884
chr2	12440434	12440497
chr2	12458124	12458729
chr2	12465964	12466390
chr2	12487069	12487316
chr2	12487667	12487723
chr2	12596606	12597162
chr2	12627255	12627259
chr2	12633428	12633671
chr2	12647347	12647546
chr2	12647763	12647913
chr2	12674674	12674933
chr2	12709588	12709934
chr2	12728337	12728807
chr2	12824424	12824524
chr2	12824957	12825107
chr2	12842526	12842919

chr2	12854582	12855267
chr2	12856016	12856910
chr2	12857067	12858535
chr2	12858848	12859642
chr2	12859915	12860238
chr2	12899241	12899260
chr2	12899554	12899899
chr2	12959153	12959371
chr2	12959475	12959657
chr2	13119532	13119630
chr2	13146993	13147192
chr2	14330104	14330164
chr2	14428067	14428201
chr2	14428590	14428716
chr2	14437434	14437579
chr2	14437752	14437862
chr2	14633021	14633066
chr2	14633447	14633510
chr2	14656978	14656987
chr2	14657407	14657418
chr2	14657894	14657966
chr2	14675702	14675726
chr2	14697250	14697365
chr2	14727209	14727675
chr2	14769028	14769137
chr2	14772285	14772724
chr2	14772880	14773423
chr2	14774303	14775610
chr2	14776021	14776036
chr2	15299198	15299355
chr2	15309686	15309784
chr2	15310136	15310486
chr2	15364040	15364438
chr2	15475146	15475223
chr2	15499795	15499858
chr2	15500067	15500780
chr2	15593745	15594135
chr2	15700410	15700629
chr2	15701147	15701683
chr2	15731470	15732667
chr2	15773582	15773724
chr2	15813819	15814003
chr2	16956819	16956831
chr2	17042752	17042802
chr2	17043102	17043159
chr2	17090156	17090248

chr2	17097011	17097020
chr2	17484163	17484317
chr2	17484549	17485123
chr2	17598755	17598766
chr2	17656897	17656948
chr2	17699435	17699535
chr2	17719809	17720309
chr2	17720622	17721752
chr2	17722179	17722242
chr2	17769325	17769427
chr2	17769638	17769664
chr2	17770742	17771069
chr2	17771735	17771902
chr2	17772228	17772239
chr2	17774279	17774678
chr2	17801926	17801961
chr2	17802146	17802568
chr2	17803019	17803243
chr2	17803397	17803439
chr2	17824267	17824588
chr2	17824827	17824905
chr2	17826979	17827294
chr2	17934258	17934390
chr2	17934509	17936068
chr2	17938635	17939021
chr2	17994229	17994702
chr2	17996759	17997308
chr2	18059491	18059827
chr2	18060115	18060523
chr2	18228054	18228058
chr2	18229083	18229243
chr2	18229474	18229904
chr2	18465580	18465784
chr2	18480689	18481520
chr2	18740833	18742303
chr2	18805096	18805215
chr2	18805426	18805449
chr2	18811421	18811542
chr2	19092055	19092241
chr2	19092400	19092550
chr2	19141601	19141676
chr2	19173179	19173201
chr2	19238428	19238756
chr2	19258958	19259206
chr2	19273206	19273333
chr2	19273513	19273791

chr2	19427956	19428155
chr2	19428361	19428443
chr2	19434930	19435214
chr2	19435477	19435681
chr2	19455552	19455591
chr2	19547223	19548348
chr2	19550155	19550648
chr2	19558167	19558413
chr2	19561719	19561765
chr2	19562573	19562588
chr2	19562966	19563165
chr2	19688004	19688212
chr2	19688402	19688428
chr2	19736000	19736258
chr2	19738055	19738137
chr2	19738412	19738820
chr2	19740635	19740996
chr2	19741165	19741297
chr2	19776187	19776233
chr2	19776519	19776624
chr2	19847998	19848361
chr2	19848759	19848764
chr2	19849521	19849996
chr2	19854433	19854626
chr2	19985087	19985157
chr2	19985266	19985418
chr2	19986293	19986372
chr2	19986477	19986777
chr2	19995885	19996223
chr2	19996401	19996498
chr2	20063201	20063857
chr2	20068760	20069018
chr2	20100290	20100542
chr2	20100747	20102131
chr2	20102909	20103303
chr2	20111466	20111652
chr2	20111886	20112037
chr2	20133036	20133174
chr2	20133288	20133449
chr2	20189256	20189628
chr2	20189910	20190374
chr2	20211836	20212822
chr2	20249873	20250225
chr2	20251039	20251350
chr2	20250395	20250787
chr2	20251472	20252123

chr2	20252584	20252815
chr2	20265819	20266144
chr2	20266296	20266515
chr2	20267636	20267864
chr2	20267979	20268125
chr2	20269649	20269874
chr2	20271019	20272044
chr2	20273317	20273450
chr2	20274031	20274257
chr2	20291045	20291122
chr2	20291246	20291889
chr2	20306284	20306963
chr2	20308368	20308740
chr2	20309206	20309790
chr2	20311374	20311724
chr2	20312041	20312188
chr2	20338926	20339178
chr2	20341423	20341823
chr2	20349257	20349391
chr2	20353147	20353270
chr2	20353441	20353662
chr2	20364498	20364537
chr2	20366054	20366071
chr2	20366633	20366769
chr2	20372088	20372596
chr2	20374978	20375184
chr2	20378565	20378752
chr2	20378885	20379220
chr2	20379310	20380186
chr2	20381005	20381237
chr2	20382506	20382705
chr2	20382815	20383038
chr2	20383767	20385174
chr2	20385285	20385922
chr2	20389342	20389541
chr2	20405768	20406434
chr2	20406541	20406923
chr2	20407036	20407430
chr2	20407977	20408308
chr2	20408413	20408680
chr2	20408800	20408999
chr2	20409124	20409648
chr2	20409801	20409907
chr2	20410156	20410516
chr2	20414790	20416549
chr2	20410731	20414581

chr2	20416734	20418756
chr2	20419260	20419459
chr2	20419875	20420812
chr2	20421256	20425899
chr2	20426009	20426208
chr2	20426462	20426535
chr2	20430702	20431448
chr2	20431700	20432726
chr2	20433652	20433751
chr2	20432923	20433107
chr2	20435451	20435898
chr2	20436018	20436721
chr2	20488736	20489172
chr2	20513571	20513832
chr2	20542912	20542920
chr2	20549822	20550612
chr2	20550862	20551644
chr2	20551784	20552032
chr2	20553676	20553875
chr2	20555851	20556548
chr2	20562507	20562576
chr2	20562682	20562863
chr2	20565338	20565574
chr2	20569872	20570455
chr2	20579204	20579239
chr2	20583221	20583453
chr2	20601114	20601665
chr2	20621297	20621543
chr2	20621663	20621709
chr2	20624445	20624894
chr2	20638105	20638408
chr2	20638591	20638711
chr2	20638886	20639289
chr2	20646357	20646658
chr2	20646834	20648321
chr2	20648461	20648548
chr2	20690219	20690418
chr2	20700635	20701058
chr2	20701773	20702240
chr2	20704561	20704712
chr2	20709920	20710595
chr2	20712856	20713103
chr2	20714088	20714736
chr2	20714875	20715441
chr2	20760594	20760794
chr2	20761724	20762186

chr2	20767953	20768765
chr2	20768939	20769385
chr2	20777835	20779864
chr2	20783912	20784258
chr2	20784635	20784982
chr2	20791448	20791676
chr2	20792575	20793240
chr2	20793399	20793713
chr2	20795918	20796281
chr2	20814048	20814391
chr2	20817396	20817595
chr2	20817845	20818323
chr2	20833376	20833686
chr2	20841613	20842201
chr2	20850138	20850928
chr2	20851069	20851450
chr2	20861143	20861369
chr2	20996594	20996630
chr2	21021819	21023123
chr2	21261461	21261563
chr2	23509653	23509662
chr2	23509792	23509991
chr2	23589854	23590000
chr2	23600620	23601034
chr2	23601490	23601754
chr2	23603545	23604097
chr2	23604355	23604486
chr2	23607299	23608042
chr2	23608297	23610167
chr2	23635052	23635240
chr2	23703599	23703749
chr2	23704298	23704592
chr2	23786802	23787126
chr2	23873571	23873812
chr2	23873998	23874125
chr2	23892550	23892751
chr2	23893150	23893372
chr2	24026421	24026571
chr2	24027086	24027122
chr2	24049687	24050019
chr2	24123533	24124141
chr2	24124875	24125164
chr2	24125254	24125384
chr2	24125558	24125902
chr2	24134951	24135322
chr2	24148622	24148714

chr2	24148850	24149977
chr2	24150132	24150617
chr2	24162639	24162924
chr2	24162999	24163982
chr2	24232757	24233246
chr2	24235167	24235366
chr2	24236344	24236423
chr2	24237772	24238113
chr2	24238272	24238439
chr2	24238817	24239396
chr2	24243636	24244002
chr2	24263676	24263860
chr2	24270019	24270285
chr2	24270737	24270936
chr2	24271788	24272394
chr2	24272580	24273312
chr2	24298758	24299261
chr2	24299856	24300278
chr2	24300411	24300775
chr2	24301607	24301811
chr2	24303661	24303952
chr2	24304200	24305105
chr2	24305642	24305973
chr2	24306592	24308281
chr2	24331495	24331755
chr2	24331865	24331901
chr2	24334612	24334726
chr2	24345969	24347063
chr2	24363158	24363209
chr2	24398534	24398701
chr2	24492189	24492376
chr2	24492481	24492514
chr2	24499232	24499365
chr2	24499488	24499710
chr2	24535947	24536182
chr2	24537122	24537240
chr2	24582771	24583832
chr2	24590677	24590770
chr2	24594279	24594640
chr2	24605289	24605301
chr2	24608316	24608515
chr2	24713137	24713864
chr2	24714473	24714969
chr2	25015539	25017079
chr2	25049152	25049351
chr2	25058659	25058735

chr2	25096471	25096902
chr2	25142157	25142945
chr2	25143255	25143656
chr2	25194171	25195242
chr2	25195399	25195440
chr2	25198716	25198749
chr2	25322027	25322043
chr2	25354778	25354995
chr2	25438571	25438605
chr2	25438702	25439259
chr2	25439710	25439955
chr2	25474176	25475754
chr2	25486875	25487578
chr2	25488437	25488658
chr2	25501692	25501901
chr2	25502039	25502666
chr2	25513940	25514258
chr2	25563189	25563674
chr2	25563785	25564024
chr2	25564357	25564571
chr2	25565089	25565604
chr2	25568306	25568505
chr2	25572188	25572223
chr2	25585209	25585305
chr2	25596505	25596721
chr2	25599506	25600349
chr2	25600480	25600679
chr2	25841505	25841755
chr2	25857164	25857405
chr2	25878294	25878599
chr2	25887495	25887673
chr2	25887903	25888150
chr2	25895450	25896476
chr2	25896720	25896930
chr2	26074732	26074946
chr2	26100184	26100245
chr2	26100513	26101240
chr2	26101714	26101833
chr2	26101924	26102310
chr2	26183938	26184159
chr2	26186780	26186917
chr2	26204602	26205896
chr2	26214469	26214644
chr2	26214872	26215146
chr2	26220512	26221075
chr2	26221520	26221719

chr2	26222317	26222730
chr2	26222989	26223192
chr2	26226714	26226881
chr2	26227281	26227508
chr2	26231548	26231840
chr2	26232160	26232497
chr2	26233086	26233279
chr2	26234408	26234696
chr2	26234833	26235197
chr2	26235487	26235709
chr2	26237517	26237529
chr2	26240923	26241073
chr2	26241364	26241420
chr2	26243584	26243605
chr2	26250004	26250240
chr2	26255490	26257694
chr2	26258936	26259381
chr2	26270441	26270746
chr2	26291480	26291600
chr2	26367953	26368069
chr2	26395357	26395672
chr2	26396051	26396293
chr2	26396648	26396876
chr2	26401585	26402439
chr2	26407393	26408563
chr2	26465713	26466114
chr2	26466836	26469111
chr2	26469683	26470124
chr2	26491078	26491413
chr2	26491566	26491974
chr2	26492467	26492755
chr2	26496864	26497012
chr2	26497153	26497595
chr2	26507016	26507170
chr2	26507277	26507373
chr2	26515815	26515978
chr2	26520913	26521163
chr2	26521423	26523601
chr2	26552884	26552906
chr2	26567502	26567574
chr2	26567822	26567836
chr2	26568294	26568678
chr2	26568977	26569268
chr2	26569410	26569817
chr2	26572582	26572942
chr2	26578258	26578763

chr2	26623854	26623994
chr2	26726529	26726891
chr2	26972819	26972914
chr2	26980778	26980907
chr2	26981208	26981525
chr2	26983117	26983153
chr2	26986310	26986442
chr2	26986666	26988385
chr2	26991392	26991996
chr2	26992178	26992695
chr2	26993203	26994173
chr2	26994373	26994535
chr2	26994665	26995126
chr2	27007644	27007898
chr2	27008257	27008623
chr2	27008959	27009238
chr2	27009475	27010116
chr2	27027583	27028212
chr2	27028407	27028700
chr2	27193416	27194254
chr2	27234043	27234154
chr2	27238061	27238345
chr2	27238713	27238725
chr2	27238918	27239072
chr2	27252034	27252233
chr2	27255303	27256680
chr2	27256749	27256959
chr2	27272735	27274959
chr2	27293448	27293823
chr2	27293991	27295759
chr2	27300636	27300835
chr2	27303904	27304163
chr2	27304371	27304549
chr2	27304717	27304870
chr2	27306752	27306975
chr2	27308790	27309397
chr2	27318755	27319319
chr2	27341036	27342484
chr2	27346368	27346787
chr2	27356804	27357459
chr2	27357657	27358126
chr2	27392983	27393071
chr2	27434098	27436208
chr2	27440215	27441028
chr2	27484964	27485189
chr2	27485376	27485787

chr2	27486097	27486344
chr2	27487159	27487469
chr2	27487675	27487920
chr2	27516550	27516907
chr2	27525926	27526406
chr2	27543772	27544007
chr2	27544258	27544763
chr2	27545083	27545598
chr2	27546002	27546580
chr2	27546799	27547227
chr2	27570703	27570844
chr2	27576927	27577250
chr2	27578913	27579764
chr2	27580072	27580642
chr2	27586575	27586632
chr2	27592583	27593197
chr2	27593440	27593929
chr2	27593995	27594138
chr2	27594250	27594571
chr2	27602648	27604477
chr2	27621573	27621867
chr2	27631712	27632453
chr2	27632675	27633482
chr2	27636937	27637050
chr2	27637291	27637513
chr2	27650995	27652395
chr2	27652803	27653142
chr2	27664918	27665663
chr2	27665801	27666379
chr2	27667494	27667523
chr2	27668664	27669043
chr2	27669205	27669363
chr2	27711958	27712307
chr2	27712747	27713294
chr2	27717683	27718307
chr2	27781300	27781507
chr2	27781779	27781828
chr2	27805525	27805791
chr2	27805940	27806255
chr2	27850738	27850877
chr2	27851102	27852088
chr2	27870736	27870975
chr2	27886118	27887374
chr2	27905161	27905424
chr2	27906288	27906696
chr2	27910173	27910306

chr2	27910422	27910572
chr2	27912206	27912374
chr2	27912542	27912638
chr2	27926854	27926966
chr2	27928703	27929798
chr2	27933858	27933962
chr2	27934076	27934255
chr2	27936182	27936751
chr2	27936980	27939192
chr2	27939660	27940045
chr2	27942598	27943116
chr2	27954074	27954565
chr2	27958093	27958531
chr2	27960131	27960295
chr2	27960465	27960510
chr2	27974886	27975131
chr2	27975411	27976472
chr2	27976980	27977139
chr2	27984517	27984632
chr2	27985384	27985652
chr2	27986982	27987181
chr2	27994266	27995191
chr2	27998978	27999406
chr2	28020065	28020129
chr2	28021392	28021600
chr2	28021772	28022081
chr2	28022246	28022884
chr2	28025393	28025411
chr2	28030370	28030684
chr2	28066851	28067244
chr2	28067595	28067970
chr2	28078372	28078532
chr2	28080339	28080383
chr2	28083240	28083362
chr2	28112760	28114327
chr2	28137643	28137982
chr2	28155237	28155427
chr2	28155588	28155740
chr2	28177070	28177476
chr2	28177575	28177980
chr2	28178104	28178288
chr2	28179749	28180084
chr2	28182392	28182654
chr2	28184876	28185214
chr2	28186051	28186445
chr2	28200702	28201175

chr2	28215885	28216056
chr2	28216167	28216289
chr2	28216369	28216387
chr2	28216563	28216568
chr2	28227045	28227337
chr2	28291023	28291353
chr2	28389870	28390198
chr2	28395225	28395646
chr2	28408018	28408055
chr2	28472871	28473072
chr2	28491243	28491447
chr2	28491949	28492410
chr2	28497614	28498486
chr2	28498902	28499439
chr2	28516579	28516713
chr2	28519811	28520101
chr2	28593219	28593408
chr2	28593570	28593598
chr2	28598901	28599462
chr2	28599846	28601813
chr2	28604470	28604647
chr2	28610625	28610690
chr2	28612402	28613355
chr2	28613478	28616857
chr2	28617009	28619901
chr2	28625275	28625488
chr2	28627087	28627128
chr2	28627718	28627917
chr2	28629435	28629523
chr2	28630050	28630326
chr2	28630840	28631039
chr2	28631373	28632231
chr2	28632356	28632682
chr2	28632833	28633087
chr2	28658823	28659124
chr2	28659268	28660406
chr2	28660608	28661331
chr2	28668370	28668716
chr2	28668928	28669189
chr2	28670807	28671660
chr2	28672094	28672484
chr2	28673257	28673456
chr2	28672612	28673057
chr2	28693586	28694457
chr2	28730957	28730961
chr2	28768617	28768805

chr2	28769539	28769540
chr2	28769797	28770022
chr2	28785698	28785848
chr2	28785960	28786071
chr2	28788391	28788816
chr2	28789004	28789741
chr2	28789881	28790451
chr2	28806388	28806780
chr2	28807138	28807757
chr2	28816970	28817266
chr2	28817398	28817710
chr2	28819547	28819995
chr2	28822555	28822574
chr2	28831675	28832124
chr2	28833254	28833397
chr2	28833810	28833913
chr2	28834550	28834697
chr2	28836761	28837563
chr2	28838146	28838368
chr2	28844045	28844994
chr2	28847917	28848175
chr2	28856961	28857109
chr2	28866034	28866330
chr2	28866526	28866655
chr2	28866806	28866893
chr2	28867115	28867489
chr2	28871081	28871280
chr2	28872286	28872419
chr2	28872799	28873072
chr2	28877830	28878174
chr2	28878526	28878598
chr2	28881285	28881313
chr2	28881568	28881643
chr2	28904070	28904492
chr2	28915145	28915308
chr2	28934768	28934995
chr2	28936420	28936750
chr2	28939185	28939775
chr2	28940194	28940764
chr2	28941166	28941365
chr2	28973634	28975443
chr2	28976214	28976603
chr2	29024651	29025004
chr2	29032710	29033522
chr2	29033723	29034169
chr2	29034570	29034780

chr2	29044300	29044388
chr2	29092599	29093112
chr2	29093366	29093677
chr2	29117065	29117376
chr2	29117537	29118819
chr2	29119906	29120080
chr2	29126820	29127089
chr2	29127321	29127549
chr2	29128470	29128766
chr2	29150682	29150700
chr2	29151019	29151059
chr2	29175223	29175526
chr2	29175595	29175625
chr2	29175769	29175971
chr2	29195020	29195143
chr2	29199309	29199454
chr2	29276843	29277044
chr2	29288529	29288792
chr2	29289078	29289343
chr2	29289477	29289899
chr2	29290360	29290687
chr2	29291354	29291460
chr2	29291653	29291852
chr2	29293523	29293721
chr2	29331026	29331199
chr2	29331311	29331489
chr2	29333822	29334163
chr2	29337476	29339614
chr2	29340751	29341130
chr2	29346365	29346715
chr2	29346861	29346936
chr2	29353292	29353298
chr2	29353416	29353506
chr2	29388463	29388640
chr2	29415304	29415465
chr2	29483575	29483669
chr2	29591361	29591519
chr2	30294879	30295015
chr2	30359565	30360003
chr2	30362298	30362665
chr2	30369120	30369444
chr2	30369566	30370832
chr2	30453944	30454284
chr2	30454431	30454829
chr2	30495898	30496097
chr2	30497516	30497605

chr2	30497870	30498043
chr2	30669501	30670652
chr2	30734262	30734353
chr2	30888267	30888272
chr2	31203692	31203882
chr2	31359793	31361749
chr2	31456560	31456605
chr2	31456887	31457648
chr2	31550582	31550822
chr2	31560650	31560912
chr2	31561090	31561244
chr2	31601886	31602330
chr2	31603560	31603665
chr2	31607219	31608630
chr2	31609545	31609652
chr2	31610441	31610852
chr2	31613132	31613324
chr2	31613520	31613736
chr2	31624739	31624986
chr2	31625302	31625797
chr2	31634675	31635020
chr2	31636709	31637368
chr2	31637454	31637708
chr2	31637967	31638171
chr2	31652663	31652856
chr2	31653074	31653431
chr2	32062691	32062859
chr2	32147647	32147852
chr2	32165733	32165932
chr2	32199539	32199760
chr2	32212691	32212870
chr2	32228838	32229332
chr2	32230434	32230692
chr2	32233422	32233763
chr2	32234508	32235081
chr2	32235194	32236109
chr2	32236239	32236751
chr2	32243880	32244079
chr2	32244339	32244495
chr2	32257808	32257995
chr2	32259788	32259937
chr2	32264388	32265267
chr2	32286854	32287411
chr2	32287476	32287877
chr2	32288036	32288552
chr2	32288720	32289716

chr2	32357994	32358152
chr2	32360267	32360867
chr2	32390283	32390739
chr2	32391024	32391358
chr2	32459228	32459448
chr2	32460696	32460781
chr2	32502715	32502846
chr2	32503000	32503696
chr2	32509439	32509875
chr2	32513598	32514016
chr2	32514234	32514363
chr2	32514548	32514573
chr2	32559069	32559225
chr2	32578836	32579078
chr2	32581469	32581961
chr2	32582213	32583083
chr2	32583267	32583583
chr2	32613226	32613574
chr2	32645649	32645848
chr2	32650952	32651151
chr2	32709835	32709981
chr2	32710288	32710362
chr2	32785954	32786171
chr2	32852642	32852877
chr2	32853248	32853457
chr2	32903959	32904140
chr2	32940649	32940862
chr2	32941086	32941148
chr2	32941338	32941427
chr2	33141137	33141708
chr2	33172087	33172755
chr2	33172874	33173079
chr2	33295245	33295444
chr2	33295612	33295782
chr2	33701073	33701286
chr2	33701757	33701900
chr2	33823529	33824294
chr2	33824481	33824916
chr2	34774701	34775051
chr2	36580009	36580247
chr2	36581813	36584669
chr2	36584935	36585423
chr2	36585616	36585888
chr2	36587342	36587550
chr2	36588748	36589031
chr2	36589364	36589514

chr2	36589651	36589768
chr2	36590273	36590591
chr2	36592718	36592980
chr2	36594668	36595187
chr2	36595315	36595634
chr2	36598335	36598697
chr2	36599074	36599260
chr2	36600215	36600342
chr2	36600488	36600670
chr2	36603419	36603848
chr2	36601629	36602150
chr2	36604610	36604630
chr2	36608091	36608414
chr2	36608768	36608820
chr2	36660412	36660860
chr2	36666101	36666148
chr2	36676502	36676541
chr2	36683322	36683338
chr2	36683442	36683521
chr2	36683613	36683825
chr2	36686909	36687025
chr2	36687194	36687365
chr2	36688269	36688864
chr2	36705328	36705883
chr2	36713015	36713054
chr2	36713751	36713980
chr2	36734500	36734584
chr2	36788668	36789040
chr2	36790118	36790127
chr2	36790263	36790663
chr2	36795690	36796037
chr2	36824605	36825192
chr2	36825522	36825594
chr2	36877521	36877643
chr2	36928636	36928683
chr2	36928943	36929102
chr2	36934561	36934575
chr2	37116861	37117429
chr2	37153527	37153726
chr2	37157872	37158492
chr2	37169972	37170085
chr2	37190273	37190289
chr2	37192779	37193797
chr2	37193982	37194509
chr2	37219096	37219527
chr2	37279722	37280438

chr2	37286518	37286848
chr2	37310835	37311482
chr2	37311756	37311916
chr2	37330874	37331232
chr2	37338404	37338425
chr2	37339393	37339429
chr2	37383087	37384481
chr2	37395540	37395868
chr2	37407177	37407200
chr2	37423466	37423535
chr2	37423731	37424512
chr2	37438391	37438478
chr2	37458308	37459054
chr2	37459270	37459745
chr2	37536377	37536450
chr2	37535469	37535897
chr2	37536584	37537066
chr2	37550848	37551173
chr2	37551458	37551885
chr2	37552269	37552739
chr2	37578735	37578945
chr2	37614884	37614929
chr2	37615190	37615261
chr2	37615684	37615706
chr2	37617176	37617278
chr2	37617410	37617689
chr2	37617921	37617982
chr2	37750986	37751338
chr2	37763077	37763602
chr2	37788761	37788841
chr2	37789183	37789184
chr2	37789430	37789542
chr2	37813320	37813526
chr2	37815614	37815951
chr2	37816453	37816666
chr2	37851626	37852022
chr2	37852271	37852641
chr2	37856920	37857058
chr2	37862935	37863121
chr2	37879216	37879739
chr2	37881070	37881676
chr2	37882683	37882795
chr2	37898040	37899767
chr2	37900021	37900073
chr2	37903374	37903585
chr2	37903767	37903854

chr2	37916166	37916254
chr2	37916690	37916732
chr2	37936024	37936170
chr2	37938443	37938953
chr2	37939284	37939295
chr2	37940441	37940772
chr2	37980396	37980700
chr2	37983306	37983452
chr2	37995867	37995969
chr2	38002307	38003379
chr2	38003620	38003717
chr2	38008205	38008541
chr2	38013864	38014097
chr2	38014344	38014440
chr2	38029533	38029680
chr2	38029798	38030392
chr2	38097982	38098058
chr2	38098944	38098979
chr2	38136457	38136549
chr2	38144246	38144265
chr2	38152019	38153075
chr2	38240359	38240558
chr2	38292309	38292373
chr2	38300503	38301196
chr2	38301373	38304668
chr2	38309252	38309253
chr2	38319083	38320164
chr2	38322886	38324108
chr2	38325847	38326120
chr2	38333419	38333983
chr2	38336604	38337252
chr2	38342636	38342740
chr2	38343964	38344272
chr2	38373950	38374409
chr2	38375561	38375742
chr2	38376005	38376329
chr2	38443195	38443248
chr2	38466486	38466813
chr2	38501456	38501618
chr2	38501787	38501799
chr2	38553841	38554014
chr2	38596366	38596389
chr2	38603263	38604408
chr2	38604725	38605013
chr2	38622435	38622472
chr2	38623174	38623215

chr2	38624084	38624126
chr2	38625778	38626315
chr2	38626431	38626470
chr2	38741928	38742279
chr2	38743057	38743304
chr2	38755194	38755503
chr2	38763050	38763293
chr2	38763627	38763684
chr2	38827628	38827969
chr2	38829164	38830166
chr2	38830514	38830805
chr2	38875258	38875625
chr2	38917675	38917886
chr2	38977432	38978464
chr2	38978770	38979151
chr2	38992589	38992628
chr2	39005056	39005258
chr2	39005391	39005843
chr2	39054788	39055189
chr2	39083348	39083425
chr2	39093907	39094427
chr2	39102483	39102997
chr2	39103314	39103594
chr2	39103697	39103726
chr2	39162865	39162966
chr2	39186987	39187520
chr2	39187858	39188205
chr2	39252348	39252364
chr2	39261419	39261511
chr2	39295462	39295480
chr2	39295767	39296650
chr2	39324592	39325124
chr2	39327234	39327458
chr2	39341958	39342087
chr2	39346862	39347158
chr2	39347407	39348683
chr2	39351034	39351474
chr2	39351590	39352075
chr2	39456979	39457099
chr2	39457215	39457343
chr2	39462153	39462298
chr2	39470839	39471117
chr2	39606926	39607130
chr2	39608939	39609077
chr2	39632371	39633033
chr2	39638672	39639172

chr2	39663313	39663377
chr2	39663593	39664510
chr2	39664863	39665429
chr2	39666989	39667122
chr2	39715814	39716003
chr2	39719591	39719701
chr2	39731788	39732175
chr2	39732291	39732436
chr2	39740991	39741147
chr2	39750476	39751209
chr2	39773738	39773873
chr2	39774042	39774432
chr2	39774913	39775786
chr2	39795638	39796005
chr2	39970456	39970900
chr2	39999540	39999635
chr2	40005922	40006573
chr2	40072749	40073040
chr2	40330280	40330630
chr2	40611944	40612037
chr2	40629020	40629372
chr2	40630635	40630653
chr2	40661961	40662122
chr2	40662361	40662468
chr2	40678769	40679246
chr2	40782061	40782182
chr2	40944036	40944065
chr2	41937357	41937395
chr2	41937518	41937757
chr2	41945056	41945394
chr2	41949902	41950269
chr2	42068299	42068506
chr2	42274751	42274985
chr2	42328183	42328844
chr2	42328928	42329150
chr2	42353986	42354453
chr2	42396140	42396214
chr2	42396537	42397265
chr2	42542479	42542543
chr2	42569498	42569699
chr2	42570041	42570280
chr2	42570362	42570372
chr2	42587002	42587273
chr2	42587979	42588748
chr2	42588881	42588996
chr2	42598696	42598802

chr2	42598995	42599221
chr2	42599360	42599516
chr2	42647645	42647987
chr2	42720720	42721857
chr2	42746576	42746640
chr2	42795444	42796337
chr2	42796575	42796889
chr2	42799414	42799845
chr2	42813153	42813403
chr2	42826485	42826797
chr2	43019600	43020042
chr2	43036809	43038693
chr2	43053317	43053603
chr2	43056800	43056999
chr2	43063463	43063705
chr2	43077276	43077523
chr2	43081051	43081225
chr2	43081328	43081392
chr2	43123828	43123989
chr2	43133972	43134171
chr2	43150592	43151121
chr2	43151911	43151969
chr2	43152273	43152596
chr2	43163055	43163561
chr2	43163738	43164329
chr2	43164736	43164935
chr2	43177731	43177984
chr2	43201252	43201620
chr2	43202372	43202962
chr2	43204994	43205108
chr2	43213159	43213359
chr2	43214237	43214765
chr2	43237834	43237936
chr2	43238126	43238220
chr2	43241910	43242433
chr2	43245676	43245862
chr2	43246037	43246707
chr2	43248432	43248553
chr2	43248935	43249255
chr2	43251484	43251540
chr2	43251607	43251755
chr2	43252575	43252813
chr2	43253170	43253241
chr2	43257068	43257237
chr2	43265373	43265524
chr2	43265931	43266162

chr2	43266500	43267300
chr2	43267388	43267660
chr2	43267818	43268125
chr2	43270596	43270873
chr2	43268243	43270468
chr2	43271560	43271966
chr2	43273479	43273483
chr2	43273699	43274167
chr2	43274393	43274505
chr2	43277479	43278955
chr2	43282693	43282892
chr2	43283193	43283395
chr2	43285177	43285275
chr2	43285403	43285429
chr2	43286575	43286619
chr2	43286967	43287141
chr2	43287538	43287641
chr2	43296083	43296282
chr2	43296717	43296916
chr2	43297207	43297470
chr2	43300460	43301023
chr2	43301200	43302783
chr2	43303679	43303982
chr2	43302901	43303306
chr2	43307093	43307495
chr2	43308442	43309006
chr2	43309640	43311877
chr2	43312349	43312730
chr2	43313267	43314423
chr2	43329845	43330137
chr2	43332488	43332606
chr2	43332946	43333412
chr2	43334089	43334344
chr2	43335121	43335225
chr2	43336069	43336136
chr2	43355829	43355907
chr2	43356573	43356584
chr2	43356712	43356985
chr2	43357367	43357946
chr2	43358012	43360111
chr2	43360550	43360749
chr2	43362513	43362758
chr2	43371439	43371750
chr2	43372728	43372848
chr2	43372988	43373161
chr2	43373237	43373370

chr2	43379522	43379996
chr2	43380296	43380829
chr2	43382425	43382653
chr2	43383045	43383325
chr2	43388416	43388480
chr2	43383937	43388163
chr2	43394970	43397056
chr2	43398996	43399162
chr2	43405569	43406507
chr2	43406781	43407087
chr2	43425432	43425707
chr2	43425962	43426063
chr2	43426649	43427806
chr2	43432865	43433022
chr2	43435242	43435560
chr2	43436894	43436923
chr2	43436988	43437521
chr2	43445652	43447689
chr2	43447924	43448247
chr2	43448385	43448752
chr2	43448942	43449643
chr2	43449837	43450107
chr2	43450634	43450980
chr2	43451211	43456084
chr2	43456147	43456544
chr2	43456799	43457193
chr2	43464953	43464977
chr2	43521255	43521405
chr2	43538527	43538557
chr2	43560684	43560984
chr2	43568175	43568374
chr2	43653571	43653622
chr2	43653871	43653995
chr2	43656513	43656845
chr2	43685260	43685271
chr2	43705859	43705934
chr2	43722829	43722862
chr2	43723007	43723214
chr2	43745360	43745559
chr2	43747093	43747422
chr2	43767099	43767204
chr2	43767337	43767588
chr2	43768230	43768793
chr2	43822746	43823164
chr2	43823313	43823712
chr2	43863881	43863943

chr2	43864391	43864816
chr2	43903724	43903892
chr2	44001215	44001478
chr2	44109077	44109217
chr2	44222620	44223340
chr2	44223556	44223736
chr2	44224109	44224230
chr2	44351311	44351585
chr2	44363478	44363737
chr2	44394714	44394904
chr2	44395086	44395591
chr2	44396027	44396923
chr2	44397572	44397774
chr2	44398203	44398405
chr2	44415694	44415849
chr2	44588180	44589364
chr2	44589599	44589936
chr2	44805531	44805608
chr2	44805859	44805925
chr2	44942762	44942843
chr2	45185880	45185909
chr2	45187983	45188034
chr2	45191859	45191873
chr2	45235239	45235666
chr2	45235841	45236598
chr2	45236975	45237901
chr2	45240620	45241271
chr2	45396016	45396577
chr2	45396684	45396883
chr2	45405270	45405523
chr2	45696863	45697076
chr2	45796190	45796592
chr2	45805697	45805896
chr2	45837608	45838346
chr2	45838540	45838694
chr2	45839379	45839809
chr2	45870484	45870663
chr2	45870739	45870928
chr2	45877160	45877503
chr2	45877733	45879100
chr2	45879345	45879742
chr2	45902394	45902967
chr2	45909126	45909244
chr2	45909632	45910180
chr2	45910388	45910603
chr2	46049441	46049452

chr2	46076590	46076668
chr2	46077332	46077396
chr2	46163061	46163089
chr2	46165634	46165928
chr2	46166295	46166581
chr2	46189964	46190023
chr2	46190931	46191538
chr2	46219196	46219498
chr2	46220559	46220560
chr2	46219782	46220079
chr2	46220821	46220913
chr2	46256836	46257057
chr2	46294857	46294898
chr2	46295141	46295338
chr2	46295835	46296034
chr2	46300670	46300848
chr2	46301073	46301203
chr2	46310007	46310572
chr2	46324831	46324838
chr2	46325162	46325316
chr2	46325617	46326244
chr2	46361520	46361983
chr2	46389033	46389110
chr2	46390634	46390647
chr2	46390946	46392481
chr2	46393081	46393311
chr2	46393982	46394217
chr2	46398063	46399974
chr2	46419528	46419573
chr2	46424804	46425046
chr2	46425399	46425491
chr2	46425687	46425915
chr2	46451847	46451919
chr2	46452421	46452510
chr2	46523685	46524395
chr2	46524512	46526738
chr2	46527015	46527308
chr2	46530730	46530948
chr2	46532428	46532562
chr2	46533381	46533539
chr2	46533733	46533909
chr2	46536631	46536697
chr2	46536979	46537148
chr2	46538225	46538662
chr2	46541516	46541923
chr2	46542065	46542207

chr2	46542445	46543033
chr2	46543625	46544292
chr2	46546164	46546329
chr2	46546489	46546580
chr2	46550686	46551472
chr2	46552461	46552844
chr2	46553309	46553830
chr2	46556102	46556361
chr2	46556850	46557377
chr2	46557626	46557857
chr2	46558176	46558519
chr2	46558751	46559386
chr2	46559488	46560001
chr2	46560233	46560541
chr2	46561139	46562259
chr2	46562890	46563062
chr2	46563844	46564053
chr2	46592278	46592288
chr2	46610558	46610617
chr2	46613009	46613754
chr2	46635628	46637053
chr2	46662306	46662546
chr2	46688225	46688637
chr2	46708771	46708859
chr2	46720917	46721269
chr2	46726696	46727315
chr2	46727630	46727772
chr2	46768843	46769657
chr2	46770188	46770932
chr2	46843130	46844585
chr2	46872162	46872514
chr2	46872739	46873327
chr2	46875160	46875320
chr2	46875475	46875863
chr2	46877901	46878017
chr2	46884073	46884187
chr2	46925421	46926015
chr2	46926318	46927037
chr2	46976423	46976429
chr2	47004052	47004231
chr2	47006475	47006734
chr2	47007223	47007296
chr2	47031184	47031488
chr2	47031617	47031623
chr2	47073558	47074347
chr2	47074551	47075943

chr2	47077849	47077974
chr2	47079414	47079663
chr2	47080014	47080034
chr2	47081482	47081876
chr2	47082272	47084077
chr2	47093920	47094264
chr2	47094604	47095145
chr2	47103775	47103939
chr2	47104289	47104647
chr2	47116027	47116782
chr2	47140861	47141587
chr2	47142304	47143068
chr2	47143252	47144045
chr2	47146312	47146343
chr2	47152908	47152975
chr2	47167031	47168275
chr2	47168378	47169698
chr2	47181990	47182044
chr2	47182923	47182927
chr2	47191313	47191674
chr2	47192271	47192953
chr2	47193682	47194871
chr2	47194982	47195617
chr2	47196589	47197240
chr2	47197458	47197873
chr2	47198071	47198504
chr2	47211874	47212107
chr2	47212392	47213064
chr2	47213367	47214332
chr2	47243219	47243300
chr2	47243381	47243430
chr2	47243939	47244100
chr2	47258096	47258102
chr2	47260637	47261568
chr2	47261822	47262063
chr2	47269385	47269752
chr2	47269975	47270119
chr2	47270352	47270879
chr2	47271058	47271555
chr2	47296067	47296796
chr2	47303243	47303761
chr2	47303923	47304131
chr2	47304277	47304719
chr2	47401948	47402061
chr2	47402192	47402462
chr2	47402712	47402823

chr2	47402978	47404340
chr2	47418719	47419394
chr2	47419911	47419944
chr2	47420137	47420327
chr2	47477564	47477725
chr2	47478210	47478390
chr2	47483481	47483677
chr2	47489914	47489948
chr2	47496479	47496884
chr2	47497354	47497371
chr2	47499309	47499858
chr2	47516039	47516077
chr2	47516407	47516433
chr2	47526685	47527061
chr2	47550195	47550363
chr2	47561514	47562071
chr2	47582983	47583315
chr2	47587908	47588173
chr2	47596018	47596103
chr2	47595579	47595668
chr2	47596362	47597416
chr2	47629923	47631011
chr2	47647970	47648197
chr2	47649290	47649568
chr2	47715924	47716018
chr2	47716377	47716707
chr2	47725511	47725611
chr2	47728455	47728806
chr2	47728995	47729346
chr2	47748594	47748815
chr2	47979693	47980165
chr2	47980331	47980425
chr2	47995057	47995228
chr2	48009409	48010185
chr2	48010353	48011196
chr2	48011462	48011567
chr2	48107990	48108329
chr2	48120020	48120424
chr2	48131494	48131842
chr2	48132202	48132365
chr2	48132543	48133469
chr2	48133978	48134218
chr2	48134354	48134363
chr2	48333623	48335187
chr2	48338255	48338396
chr2	48338588	48339272

chr2	48340586	48340667
chr2	48341391	48341538
chr2	48341745	48341806
chr2	48475664	48475781
chr2	48475954	48476095
chr2	48478188	48478493
chr2	48533844	48534416
chr2	48539209	48539479
chr2	48539593	48540090
chr2	48540277	48540572
chr2	48541159	48541477
chr2	48541765	48542652
chr2	48542815	48543447
chr2	48580323	48580715
chr2	48647504	48647920
chr2	48648228	48648301
chr2	48667401	48667723
chr2	48667850	48668354
chr2	48675840	48676039
chr2	48731805	48731918
chr2	48735719	48735857
chr2	48737410	48737648
chr2	48756919	48757127
chr2	48757349	48757877
chr2	50774729	50774790
chr2	50773891	50774302
chr2	50814221	50814298
chr2	50877310	50877709
chr2	51179741	51180265
chr2	51180386	51180604
chr2	52010338	52010601
chr2	53357052	53357434
chr2	53358584	53358897
chr2	53539122	53539236
chr2	53540991	53541125
chr2	53580715	53580966
chr2	53624308	53624309
chr2	53631155	53631277
chr2	53786676	53786860
chr2	53787057	53787094
chr2	53994596	53995578
chr2	53997399	53997715
chr2	54012697	54012947
chr2	54013664	54014044
chr2	54014166	54014700
chr2	54014778	54014979

chr2	54043273	54043472
chr2	54070099	54070136
chr2	54092660	54092764
chr2	54124395	54124534
chr2	54140880	54141040
chr2	54188992	54189270
chr2	54196396	54196548
chr2	54196709	54196844
chr2	54199125	54199472
chr2	54197081	54198442
chr2	54268218	54268383
chr2	54271330	54271514
chr2	54300971	54301378
chr2	54328590	54328773
chr2	54334342	54334435
chr2	54335552	54335754
chr2	54342385	54343297
chr2	54350010	54350497
chr2	54351741	54352176
chr2	54557372	54557879
chr2	54682432	54682652
chr2	54682938	54683243
chr2	54683475	54684272
chr2	54749786	54749820
chr2	54751450	54751965
chr2	54752081	54752099
chr2	54758629	54759111
chr2	54760067	54760139
chr2	54760269	54760509
chr2	54784689	54785487
chr2	54785708	54786368
chr2	54786613	54787824
chr2	54798072	54798591
chr2	54799294	54799805
chr2	54801403	54801968
chr2	54802572	54803292
chr2	54804253	54804427
chr2	54817026	54817045
chr2	54817226	54817338
chr2	54827185	54827441
chr2	54857293	54857468
chr2	54879708	54879908
chr2	54896997	54897125
chr2	54897230	54897318
chr2	54900469	54901229
chr2	54950279	54950420

chr2	54950789	54951106
chr2	54951703	54952203
chr2	54982736	54983030
chr2	54994847	54995111
chr2	54998260	54998658
chr2	54999276	54999640
chr2	55036847	55036970
chr2	55037247	55037396
chr2	55037983	55038144
chr2	55040756	55040955
chr2	55041429	55041820
chr2	55077394	55077760
chr2	55131076	55131310
chr2	55131458	55131685
chr2	55132077	55132406
chr2	55155886	55156083
chr2	55156201	55156284
chr2	55211265	55211397
chr2	55226296	55226652
chr2	55238106	55238408
chr2	55242144	55242496
chr2	55242720	55243032
chr2	55274934	55275549
chr2	55276031	55277552
chr2	55277904	55278093
chr2	55278166	55278968
chr2	55283490	55283567
chr2	55319033	55319292
chr2	55319585	55319915
chr2	55320481	55320490
chr2	55320847	55320944
chr2	55336635	55336864
chr2	55337005	55337031
chr2	55338814	55338969
chr2	55339191	55339338
chr2	55343004	55343242
chr2	55375709	55375901
chr2	55380704	55381455
chr2	55450382	55451200
chr2	55458900	55460510
chr2	55495963	55496703
chr2	55496994	55497235
chr2	55509084	55509941
chr2	55536649	55536867
chr2	55646360	55646481
chr2	55646613	55646920

chr2	55647052	55647746
chr2	55652452	55652695
chr2	55652849	55653056
chr2	55727489	55727576
chr2	55738654	55738785
chr2	55745932	55746629
chr2	55746787	55747698
chr2	55783073	55783155
chr2	55783433	55783457
chr2	55812550	55813186
chr2	55817911	55818380
chr2	55842572	55843114
chr2	55843592	55844414
chr2	55844544	55844903
chr2	55845096	55845243
chr2	55845482	55846183
chr2	55847652	55847909
chr2	55856109	55856398
chr2	55900667	55900840
chr2	55920153	55920429
chr2	55920770	55921256
chr2	55921830	55922478
chr2	56006821	56007297
chr2	56065830	56066511
chr2	56066617	56066995
chr2	56070203	56070688
chr2	56084196	56084479
chr2	56089376	56090276
chr2	56112815	56113173
chr2	56115383	56115702
chr2	56128470	56128634
chr2	56150286	56150425
chr2	56150685	56151201
chr2	56170925	56170944
chr2	56185161	56185669
chr2	56185781	56185891
chr2	56188471	56188540
chr2	57302649	57302690
chr2	57408506	57408602
chr2	57600677	57600736
chr2	57718089	57718183
chr2	58269926	58270022
chr2	58273501	58274752
chr2	58325404	58325987
chr2	58334873	58335476
chr2	58339929	58340549

chr2	58340616	58340800
chr2	58340964	58341080
chr2	58346266	58346303
chr2	58467928	58468764
chr2	58861405	58861467
chr2	59883271	59883322
chr2	59972180	59972334
chr2	60367583	60367793
chr2	60440850	60440997
chr2	60441327	60441397
chr2	60487760	60488239
chr2	60511406	60511605
chr2	60512792	60513013
chr2	60513252	60513383
chr2	60516523	60516982
chr2	60524676	60525085
chr2	60581764	60582039
chr2	60582195	60582307
chr2	60583459	60583557
chr2	60694201	60694306
chr2	60694895	60695158
chr2	60695964	60696215
chr2	60706321	60706511
chr2	60706708	60706908
chr2	60707143	60707482
chr2	60726828	60726840
[truncated: 355,695 more chars]
